# Characterization of a toxin-antitoxin system in *Mycobacterium tuberculosis* suggests neutralization by phosphorylation as the antitoxicity mechanism

**DOI:** 10.1038/s42003-020-0941-1

**Published:** 2020-05-07

**Authors:** Xia Yu, Xiaopan Gao, Kaixiang Zhu, Han Yin, Xujian Mao, Justyna Aleksandra Wojdyla, Bo Qin, Hairong Huang, Meitian Wang, Yi-Cheng Sun, Sheng Cui

**Affiliations:** 1 NHC Key Laboratory of Systems Biology of Pathogens, Institute of Pathogen Biology, and Center for Tuberculosis Research, Chinese Academy of Medical Sciences and Peking Union Medical College, Beijing, 100730 China; 2 Swiss Light Source at the Paul Scherrer Institute, CH-5232 Villigen, Switzerland; 3 National Clinical Laboratory on Tuberculosis, Beijing Key Laboratory for Drug-Resistant Tuberculosis Research Beijing Chest Hospital, Capital Medical University, Beijing Tuberculosis and Thoracic Tumor Institute, 101149 Beijing, China; 4 Sanming Project of Medicine in Shenzhen on construction of novel systematic network against Tuberculosis, National Clinical Research Center for Infectious Diseases, Shenzhen Third People’s Hospital, Southern University of Science and Technology, Guangdong, China

**Keywords:** Transferases, Structural biology, Kinases

## Abstract

*Mycobacterium tuberculosis* (Mtb) encodes an exceptionally large number of toxin-antitoxin (TA) systems, supporting the hypothesis that TA systems are involved in pathogenesis. We characterized the putative Mtb *Rv1044-Rv1045* TA locus structurally and functionally, demonstrating that it constitutes a bona fide TA system but adopts a previously unobserved antitoxicity mechanism involving phosphorylation of the toxin. While *Rv1045* encodes the guanylyltransferase TglT functioning as a toxin, *Rv1044* encodes the novel atypical serine protein kinase TakA, which specifically phosphorylates the cognate toxin at residue S78, thereby neutralizing its toxicity. In contrast to previous predictions, we found that *Rv1044-Rv1045* does not belong to the type IV TA family because TglT and TakA interact with each other as substrate and kinase, suggesting an unusual type of TA system. Protein homology analysis suggests that other COG5340-DUF1814 protein pairs, two highly associated but uncharacterized protein families widespread in prokaryotes, might share this unusual antitoxicity mechanism.

## Abstract (web summary)

Xia Yu et al. report the characterization of a toxin-antitoxin system with an unusual mechanism in *Mycobacterium tuberculosis*. They find that the antitoxin locus Rv1044 encodes an atypical serine protein kinase that phosphorylates the toxin to neutralize toxicity.

## Introduction

*Mycobacterium tuberculosis* (Mtb) is one of the most lethal bacterial pathogens threatening humanity in the 21^st^ century. One striking feature of Mtb is that it has an exceptionally large number of toxin-antitoxin (TA) loci. At least 88 TAs were identified in Mtb^1^. In stark contrast, a harmless relative of Mtb, *M. smegmatis*, encodes only 5 TA systems^2^. This gave rise to the assumption that the TA system may play an important role in pathogenicity. Deep and colleagues reported that the VapBC11 TA system is essential for establishing Mtb infection in vivo, providing an example of the TA system contributing to Mtb virulence^3^. Whether TA systems contribute to the formation of bacterial persister cells is currently under debate. While some studies contradict the involvement of TA systems in persister cells formation in *E. coli*^4–8^, *S. aureus*^9^ *and S. enterica*^10^, there is evidence supporting the link between TA systems and persister cell formation in other bacteria^11–15^. 10 TA modules were found to be overexpressed in the Mtb persister cells^16^, hinting a possible role of TA systems in Mtb persister formation. Mtb TAs are considered promising anti-TB drug targets^17,18^. Molecules that disrupt the type II or III toxin-antitoxin complex may act as novel antimicrobial agents^19^. For example, based on the structure of the VapBC26 TA complex, toxin-mimicking peptides were designed. The peptides can activate the toxic function of the toxin by disrupting TA complex formation^20^, thus providing a strategy for developing novel antibiotics.

Six TA families have been identified^21^. Type I system contains a toxin protein and a small antisense RNA that specifically targets the mRNA of the toxin, thus inhibiting its expression. Type II system, in which both toxin and antitoxin are proteins, is the most common and abundant TA systems. The unstable antitoxin deactivates the toxin via forming a stable protein complex with the toxin. In type III system, the toxin protein is directly bound by an RNA molecule acting as the antitoxin. Type IV system also encodes two protein components; however, in contrast with the type II TA, they do not interact with each other. Instead, both toxin and antitoxin act on the same target; however they prompt opposing outcomes. Type V system includes an endoribonuclease as the antitoxin, which degrades the mRNA of the toxin, effectively down-regulating its expression^22^. Type VI TA system encompasses an atypical SocAB TA system identified in *C. crescentus*. The labile toxin ScoB is neutralized by ClpXP proteinase degradation that is assisted by the antitoxin SocA acting as the proteolytic adaptor^23^.

Among numerous TA systems encoded by the Mtb H37Rv strain, most are type II members, including the VapBC, MazEF, YefM/YoeB, RelBE, HigBA and ParDE families, as well as the tripartite type II TAC (Toxin-Antitoxin-Chaperone) system. Among the remaining 11 uncharacterized TAs, *Rv0836c-0837c, Rv1045-1044 and Rv2827c-2826c* were predicted to be type IV TAs^24^. One well-characterized type IV TA is the YeeU/CbtA module from *E. coli*^25^. The antitoxin YeeU acts against the toxic activity of toxin CbtA like a canonical TA system, however the toxin and the antitoxin do not interact with each other. Instead, CbtA and YeeU both act on the assembly of FtsZ and MreB filaments. While YeeU promotes filament formation, CbtA inhibits it. Thus, the lack of toxin-antitoxin interaction becomes a hallmark of the type IV family. Recently, a type IV TA family member AbiE from bacterial abortive infection systems was identified^26^. However, the interaction between AbiEii and AbiEi is undetectable, which classifies AbiE as a type IV TA. AbiEii is a putative nucleotidyltransferase (NTase) belonging to the DNA polymerase β superfamily, which preferentially binds GTP; thus, it is a guanylyltransferase. Four conserved motifs (I–IV) were identified in AbiEii, the mutations of which impaired or abolished GTP binding activity and toxicity. AbiEi is comprised of an N-terminal winged-helix-turn-helix (wHTH) domain and a C-terminal uncharacterized domain (CTD). The N-terminal wHTH recognizes a DNA cassette of the *abiE* promoter repressing its own transcriptional level, whereas the CTD is an uncharacterized domain responsible for neutralization of the toxicity of AbiEii.

AbiE TA and the putative Mtb type IV TAs share extensive homology. They all belong to a widely spread and highly associated gene pairs COG5340-COG2253^26^. In which, a gene encoding an NTase acting as the toxin is always followed by a gene encoding a transcriptional regulator acting as the antitoxin. It is worth noting that COG2253 belongs to the large protein superfamily DUF1814, which is ubiquitous not only in bacteria but also in archaea and fungi. However, these abundant protein pairs are largely uncharacterized and the interplay between the toxin and antitoxin remains elusive.

Here, we characterized structurally and functionally *Rv1044-Rv1045* system, a putative type IV TA system from Mtb. We found that while *Rv1045* encodes the guanylyltransferase TglT (unusual type guanylyltransferase-like toxin), which arrests bacterial growth, *Rv1044* encodes the atypical protein kinase TakA (unusual type of atypical kinase antitoxin), which neutralizes the activity of TglT via phosphorylation. TglT and TakA interact with each other directly; thus, they do not belong to type IV TA family. Instead, it is an unusual type of TA system, because the antitoxicity mechanism involving the phosphorylation of the toxin identified in this study has not been observed previously.

## Results

### *Rv1044* and *Rv1045* constitute a TA system

**Fig. 1:**
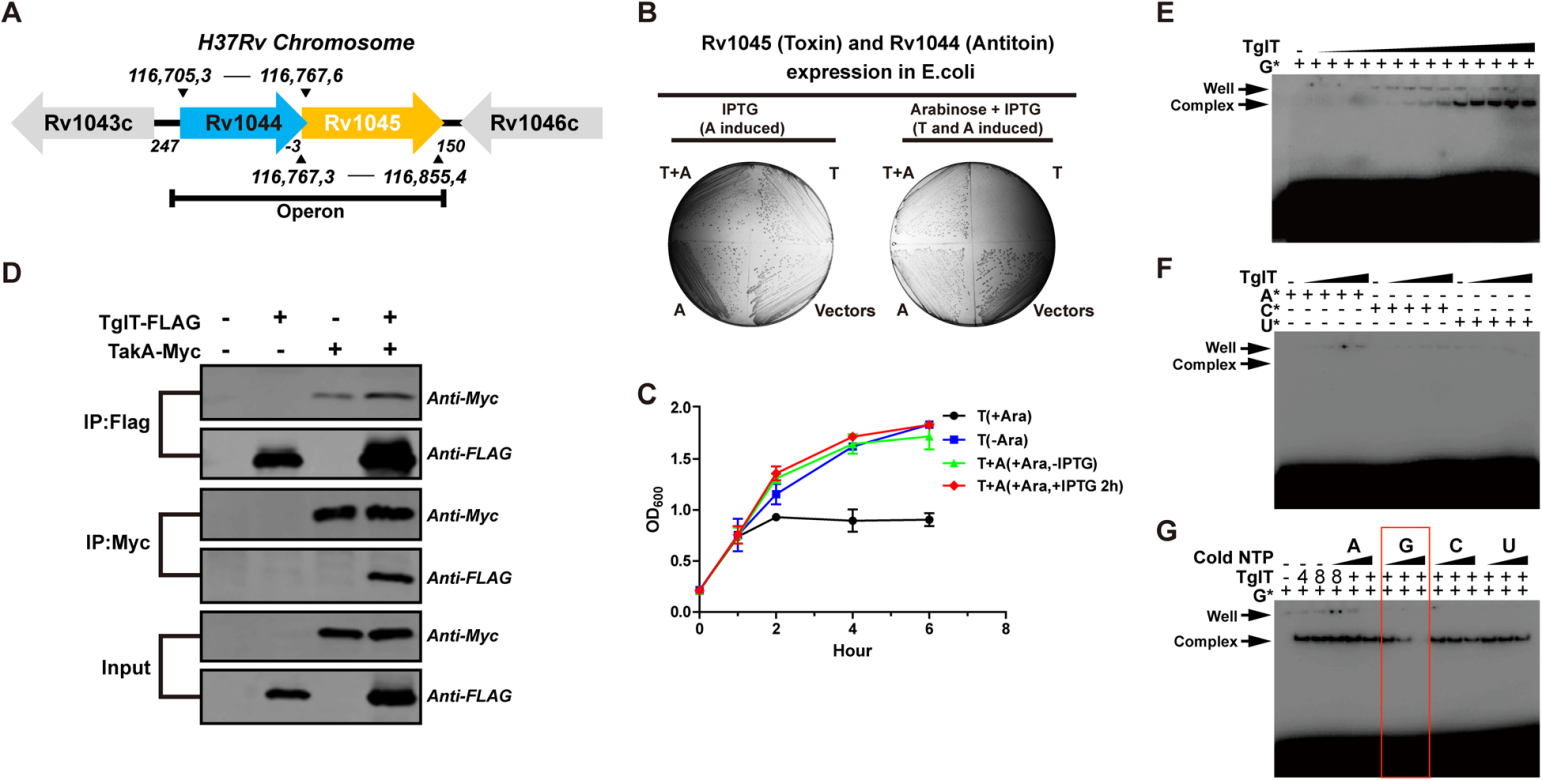
Rv1044-Rv1045 of H37Rv constitutes a bona fide TA system. **a** Diagram of the genetic organization of the Rv1044-Rv1045 operon in the H37Rv genome (not to scale). The location of the genes is indicated. **b** Toxicity and antitoxicity assay of Rv1044-Rv1045 pair in *E. coli*. The expression of the pBAD33-c-6His-Rv1045 plasmid encoding TglT-His resulted in cell growth arrest; the toxicity was neutralized when the pET28-n-6His-Rv1044 plasmid encoding His-TakA was co-expressed. See plasmid details in Supplementary Table 2. **c** Growth curves of BL21 cells containing plasmids encoding the toxin (pBAD33-c-6His-Rv1045) and the antitoxin (pET28-n-6His-Rv1044), or the toxin with the empty vector. For all experiments, the bacteria were induced when OD_600_ reached 0.2, which was set as hour 0. The OD_600_ was then measured at the indicated time points. Red curve, the toxin was induced (+Ara) first and the antitoxin was induced (+IPTG) 2 h later; green curve, the toxin was induced (+Ara) but the antitoxin was not induced (−IPTG); blue curve, BL21 cells containing pBAD33-c-6His-Rv1045 and the empty pET28a but without induction (−Ara); black curve, BL21 cells containing pBAD33-c-6His-Rv1045 and the empty pET28a with induction (+Ara). Data shown are mean OD_600_ value ± SD (*n* = 3). **d** The interaction between TglT and TakA was detected by a Co-IP experiment. **e**–**g** NTP binding assays demonstrate that toxin TglT preferentially binds GTP. In the upper two native-PAGEs, TglT forms a complex with radioactively labeled GTP, but not with ATP, CTP or UTP. The concentration of all NTPs in the assays was constant (3.3 nM). The concentration of TglT in the GTP binding assay started from the highest 16 μM (right side) to the lowest 2 nM (left side) by two-fold serial dilutions; the concentrations of TglT used in other NTP binding assays were 16 μM, 8 μM, 4 μM and 2 μM. The bottom native-PAGE shows competition binding. The concentrations of TglT and [α-^32^P] labeled GTP were 8 μM and 3.3 nM, whereas the concentrations of the cold competitors were 0.5 μM, 4 μM and 16 μM, respectively. Source data are provided as a Supplementary Data 2.

*Rv1044* (antitoxin) and *Rv1045* (toxin) are placed under the same operon in the Mtb H37Rv genome (Fig. 1a). The 3′ end of *Rv1044* overlaps with the 5′ end of *Rv1045* by 3 nucleotides, an arrangement resembling the bicistronic abiEi/abiEii operon^26^. *Rv1044* was predicted to be essential in Mtb based on the Tn library screening^27^, whereas comprehensive essentiality analysis of the Mtb genome suggested that *Rv1045* might be toxic in cells^28^.

To verify *Rv1045* toxicity and *Rv1044* antitoxicity, we inserted the toxin into the L-arabinose inducible expression vector pBAD33 (TglT-His) and the antitoxin into the IPTG inducible expression vector pET28a (His-TakA). In toxicity and antitoxicity assay, the bacterial growth was examined (Fig. 1b). The expression of the His-TakA alone did not lead to growth arrest. On the contrary, colonies could not form when TglT-His was expressed. When the expression of both TglT-His and His-TakA was induced, the toxicity of TglT was neutralized (Fig. 1b), implying that TakA counteracted the toxicity of TglT. This result indicated that *Rv1044-Rv1045* constitutes a TA system. To obtain more details, we recorded the time course of bacterial growth (Fig. 1c). When the toxin was induced first, bacteria growth could be rescued by the induction of the antitoxin later. It is worth noting that the toxicity of the toxin could still be neutralized when the TakA-expressing vector was available but was not induced (Fig. 1c green curve). This was likely due to the “leaking phenomenon” of the T7 system^29^, in which the antitoxin was expressing at a low level despite the absence of IPTG. In the following sections, we will demonstrate that the antitoxin TakA is a kinase that neutralizes the toxin via direct phosphorylation of the toxin, which offers a plausible explanation for this phenomenon: as an enzyme, the antitoxin could be very effective even when expressed at a low level.

To investigate whether *Rv1044-Rv1045* belongs to the type IV TA, we studied the direct interaction between TglT and TakA. We engineered a FLAG-tag at the C-terminus of TglT (TglT-FLAG) and a Myc-tag to the C-terminus of TakA (TakA-Myc). TglT- FLAG and TakA-Myc were co-expressed in BL21 cells and the expression of both proteins was detectable by western blotting (Fig. 1d). In co-immunoprecipitation (Co-IP) experiment, when we used anti-FLAG magnetic beads to pull-down TglT-FLAG as bait, we observed the presence of TakA-Myc (prey). However, we found that TakA-Myc bound to anti-FLAG beads nonspecifically because TakA-Myc was also pulled down in the absence of TglT-FLAG. Therefore, we chose anti-Myc magnetic beads for Co-IP. Anti-Myc beads did not exhibit nonspecific binding to TglT-FLAG (Fig. 1d). When we used anti-Myc beads to pull down TakA-Myc as bait, we observed the presence of TglT-FLAG (prey). The Co-IP experiment demonstrated that TglT and TakA interact directly with each other. This behavior is atypical for type IV TA family members.

### TglT preferentially binds GTPn

Like AbiEii, TglT belongs to the DUF1814 superfamily and contains similar conserved motifs (Supplementary Fig. 1), suggesting that it may bind NTP. We mixed purified TglT with [α-^32^P] labeled GTP, and resolved the mixtures by native-PAGE. A radioautograph of the native-PAGE (Fig. 1e) revealed a predominant band corresponding to the molecular mass of TglT. The intensity of the band increased as the protein concentration increased suggesting that TglT and GTP form a stable complex. The same experiment was performed using [α-^32^P] labeled ATP, UTP and CTP, respectively, however we did not observe the ATP-TglT, UTP-TglT or CTP-TglT complexes (Fig. 1f). To confirm GTP specificity, we performed a competition assay. After the formation of the TglT- [α-^32^P] GTP complex, we added a large excess of unlabeled ATP, GTP, CTP or UTP. The TglT-[α-^32^P] GTP complex was broken down only by unlabeled GTP (Fig. 1g). We conclude that TglT specifically binds GTP.

### Structural features of TglT

**Fig. 2:**
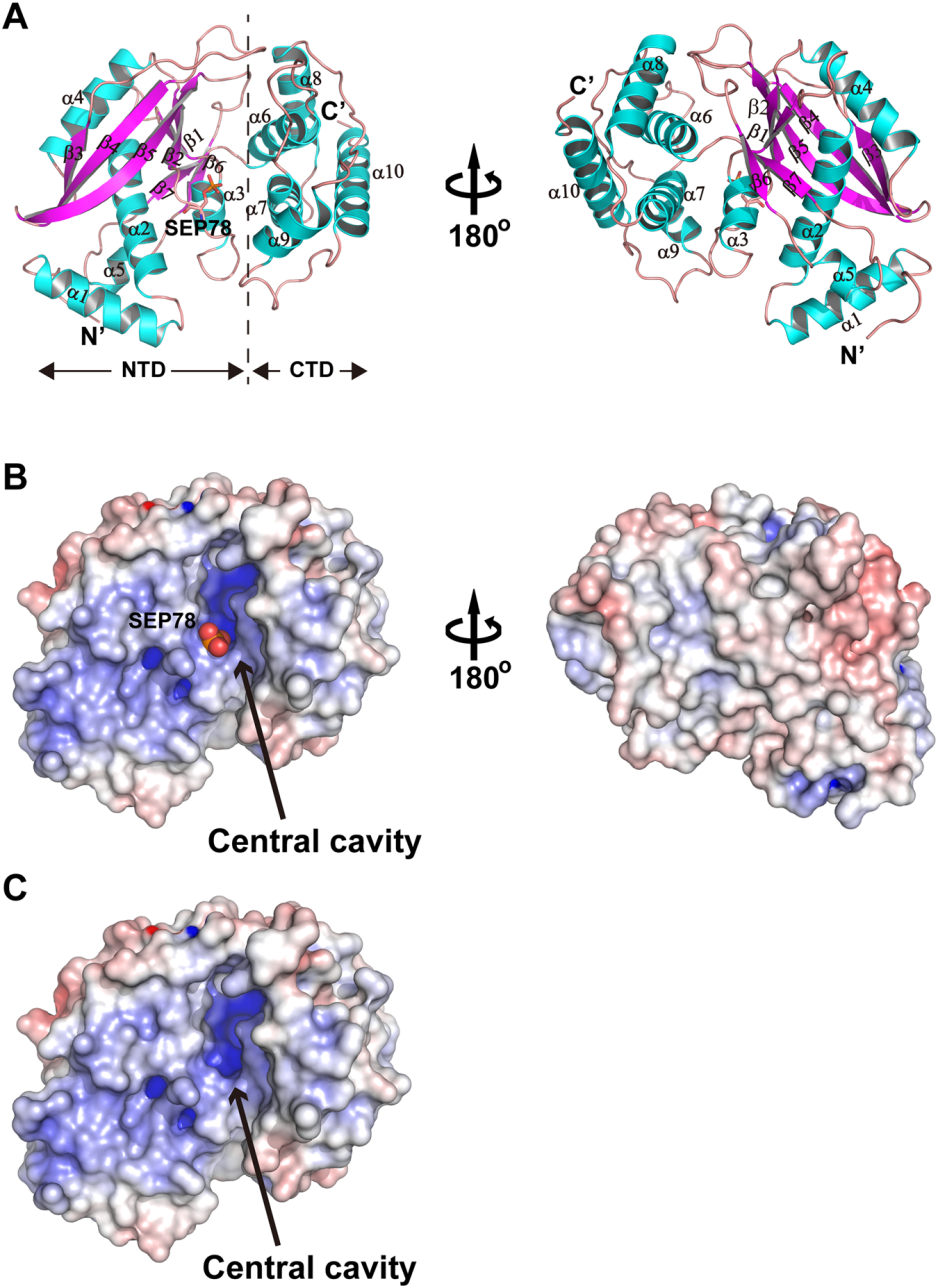
The crystal structure of TlgT reveals a putative catalytic cavity. **a** Ribbon model of the TglT crystal structure with annotated secondary structure elements. The phosphorylated S78 between NTD and CTD is highlighted with a stick model. Left, standard view; right, 180° rotation around the vertical axis. **b** Solvent accessible molecular surface of TglT colored by potential from −15 kT/e (red) to 9 kT/e (blue). The phosphate group (spheres) of SEP78 protrudes out of the left wall of the central cavity. Left, standard view; right, 180° rotation around the vertical axis. **c** Molecular surface of non-phosphorylated TglT with the same view and coloring scheme as panel **b**.

TglT has a compact fold with dimensions of 59.2 Å × 47.7 Å × 43.1 Å. It encompasses an N-terminal domain (NTD) and a C-terminal domain (CTD) (Fig. 2a). The NTD (1-181aa) exhibits an α/β fold that commonly found in nucleotidyltransferase-like proteins. The CTD is a twisted helical bundle formed by five helices (α6-α10). A large central cavity is formed between NTD and CTD. By plotting the solvent accessible surface potential of TglT (Fig. 2b, c), we revealed the positively charged cavity, suggesting it functioned as an NTP binding pocket and a NTase active site.

**Fig. 3:**
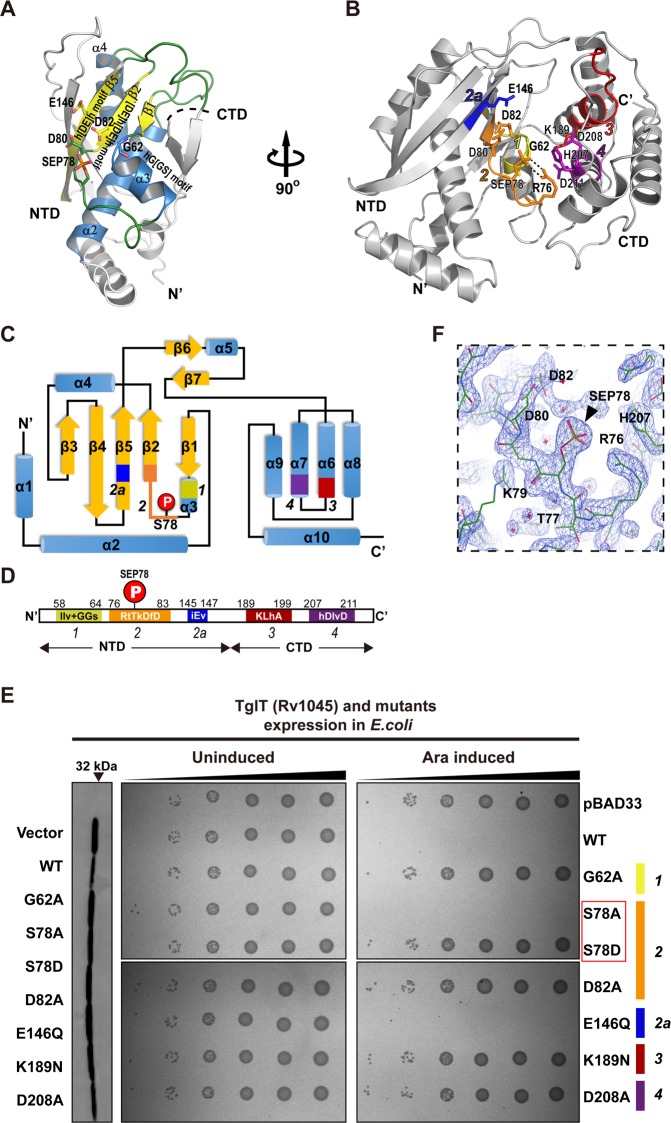
Key structural features and the conserved motifs of TglT. **a** Ribbon model of TglT NTD with the common NTase core highlighted in color and annotated secondary structures. The conserved motifs 1, 2 and 2a, and the conserved Asp/Glu residues are labeled. **b** Stander view of TglT structure with highlighted and color-coded conserved motifs 1 (yellow), 2 (brown), 2a (blue), 3 (red) and 4 (magenta). Catalytically important residues from these conserved domains are shown in stick model, and annotated. Two hydrogen bonds stabilizing the phosphate of SEP78 are shown with dashed lines. **c** Secondary structure diagram, and **d** 1-D diagram of TglT with detailed structural features; the color code is the same as panel **a**. **e** Mutations in conserved motifs affect the toxicity of TglT. Bacteria expressing the annotated proteins were spotted on M9 plates (without and with l-arabinose) in 10-fold serial dilutions, from the right to the left: 10^−1^ 10^−2^ 10^−3^, 10^−4^, 10^−5^ and 10^−6^. Cell growth was examined after overnight incubation. The expression of TglT and its variants is verified three hours post induction by western blotting, left. **f** A magnified view of the phosphorylation site at the active site of TglT. The composite omit map calculated with simulated annealing is superimposed with the atomic model. Residues around SEP78 are annotated. The phosphate group fit the map, indicated with a black triangle. Source data are provided as a Supplementary Data 2.

The NTD has a common NTase core, comprising a three-stranded mixed β-sheet (Fig. 3a). We identified three NTase motifs in the core, constituting the left-side wall of the central cavity. The hG[GS] motif is located at the beginning of helix α3 (motif 1), the loop between α3 and β2 harbors a [DE]h[DE]h motif (motif 2), and the adjacent β5-strand harbors an h[DE]h motif (motif 2a) (Fig. 3a). Three conserved Asp/Glu residues D80 and D82 from motif 2 and E146 from motif 2a were identified inside the central cavity (Fig. 3b), suggesting their role in coordinating divalent ions and the activation of the acceptor OH group of the substrate. Helices α6 and α7 of CTD harbor the conserved motif 3 and motif 4. They constitute the right-side wall of the central cavity (Fig. 3b). K189 in motif 3, as well as H207 and D208 in motif 4 are located inside the central cavity, suggesting their involvement in catalytic activity. Despite extensive efforts, neither the crystallization of TglT-GTP complex nor the soaking of concentrated GTP with TglT crystals was successful. Therefore, the exact function of these conserved residues in catalysis requires further investigation.

The TglT structure shares limited similarity with known proteins. We compared the TglT structure against all entries in PDB using the Dali server^30^. The best hit was JHP933, a putative nucleotidyltransferase from *H. pylori*, PDB id: 4OK0^31^ (Supplementary Table 1). The alignment gave a Dali *Z*-score = 14.4 with the rmsd = 4.1 Å and 199 Cα aligned. JHP933 is another DUF1814 family member, which shares 16% sequence identity with TglT.

### The NTase motifs of TglT are important to toxicity

We performed a mutagenesis study to investigate whether the conserved motifs of TglT are important to toxicity. We introduced mutation to each of the five conserved motifs identified in the catalytic center, including motifs 1, 2, 2a in the NTD and motifs 3 and 4 in the CTD (Fig.  3c, d, Supplementary Fig.  1). Whatever possible we targeted invariant and charged residues, because they are more likely playing critical roles in catalysis. We chose G62 because it is the only invariant residue in the motif 1. Motif 2a does not contain invariant residue, however residue 146 is highly conserved and negatively charged. Additionally, we investigated position of selected residues in the structure of TglT to ensure that their side chains have access to the central cavity (Fig.  3b).

While the expression of wildtype TglT inhibited bacterial growth, mutants G62A (motif 1), D82A (motif 2), K189N (motif 3) and D208A (motif 4) were non-toxic (Fig. 3e). Surprisingly, mutant E146Q (motif 2a) exhibited toxicity similar to the wildtype protein. We next compared the initial growth rates of E146Q, D82A and wildtype TglT, which revealed a clear difference 4 hours after induction (Supplementary Fig. 2). While the growth rate of D82A exhibited negligible differences from the plasmid control, the growth rate of E146Q was significantly lower. On the contrary, the density of *E. coli* expressing wildtype TglT started to decline rapidly. The difference in initial growth rates was probably below the detection limit of the growth inhibition assay (Fig. 3e). Thus, E146Q mutation on motif 2a led to attenuated toxicity rather than complete loss of function. Collectively, our findings suggest that all conserved motifs constituting the central cavity are important to the toxicity of TglT.

### Crystallographic study revealed the phosphorylation of TglT

We observed a strong positive difference map peak connected to the hydroxyl side chain of residue S78 of wildtype TglT, which was absent in D82A (Supplementary Fig. 3). The additional electron density could accommodate a phosphate, thus we modeled a phosphoserine (SEP) at this site instead of a serine. SEP78 fitted the electron density of a composite omit map calculated with simulated annealing (Fig. 3f). The prediction of the phosphorylation sites within TglT using the NetPhos 3.1 server^32^ indicated that the most likely phosphorylation site is indeed residue S78 (Supplementary Fig.  4).

The molecular surface analysis of the TglT structure (Fig. 2b, c) demonstrated that the phosphorylation of S78 may not only influence the local electrostatic charge in the central cavity, but also introduce a protrusion on the right-side wall of the cavity which can potentially block access to the cavity. Specifically, the phosphate of SEP78 is virtually located at the very center of the cavity, where it stays in proximity to all residues inside the cavity. The distances measured from the phosphate group to residues G62 (motif 1), D80 and D82 (motif 2), K189 (motif 3) and D208 (motif 4) range from 3.9 Å to 6.4 Å. R76 from motif 2 specifically interacts with the phosphate via two hydrogen bonds (Fig. 3b, f). Collectively, our crystallographic data suggest that the phosphate group may inhibit the activity of TglT and in turn the toxicity. It is worth noting that in the DUF1814 protein family (Supplementary Fig.  1), while R76 is invariant, S78 may be replaced by with threonine in some family members but both residues are phosphorylation targets of Ser/Thr protein kinases.

To shed lights on the origin of TglT phosphorylation, we determined the crystal structures of a selection of mutants, S78A, D82A and E146Q. We expressed each mutant alone and co-expressed with the antitoxin, respectively. The crystal structures of both variants were determined and compared (Supplementary Fig. 3). The mutant S78A served as the negative control as it cannot be phosphorylated. Crystal structures of TglT mutant S78A expressed alone or co-expressed with TakA showed no sign of the phosphate group adjacent to residue 78. Similarly, the crystal structures of TglT mutant D82A expressed alone and co-expressed with TakA did not have electron density for the phosphate group next to S78, suggesting that the D82A remained non-phosphorylated in the presence of TakA. On the contrary, while no electron density was observed for S78 phosphorylation in the crystal structure of TglT mutant E146Q expressed alone, we observed clear electron density for a phosphate group connected with residue S78 side chain in the TglT mutant E146Q structure co-expressed with TakA, resembling the structure of the wildtype TglT (Supplementary Fig.  3). These results suggest that while the TglT mutant D82A prevented phosphorylation of residue S78, the TglT mutant E146Q permitted phosphorylation and more importantly the phosphorylation of TglT was attributed to TakA expression.

### TglT phosphorylation was attributed to TakA

**Fig. 4:**
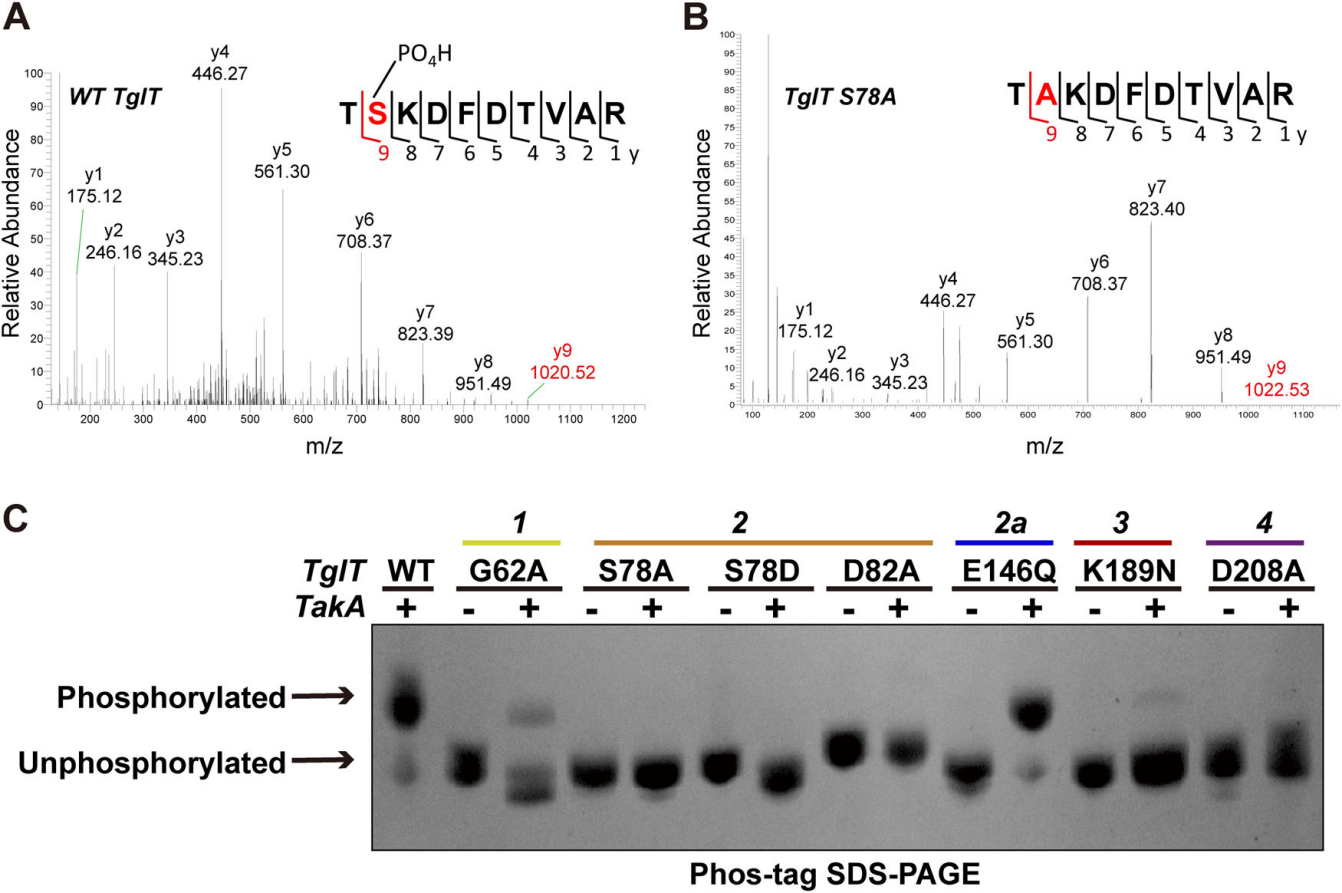
Phos-tag SDS-PAGE and LC-MS/MS and analyses confirmed the phosphorylation at residue S78. **a** LC-MS/MS analysis of the upper band of wildtype TglT sample by Phos-tag SDS-PAGE, confirming that it was phosphorylated at S78. See the detailed results in Supplementary Fig.  5. **b** LC-MS/MS analysis of the band in sample S78A mutant co-expressed with TakA by Phos-tag SDS-PAGE, confirming that the S78A mutation prevented the phosphorylation. See the detailed results in Supplementary Fig.  6. **c** TglT and its variants expressed in the presence and absence of TakA were analyzed by Phos-tag SDS-PAGE providing maximal separation between the phosphorylated and non-phosphorylated species. The mutations from the five conserved motifs are annotated; conserved motifs are color-coded as in Fig. 3. Source data are provided as a Supplementary Data 2.

To confirm S78 phosphorylation, we employed mass spectrometry. wildtype TglT and the mutant S78A co-expressed with TakA were analyzed with LC-MS/MS. A fragment _71_GIPDSRTSKDFDTVAR_86_ from wildtype TglT was observed; the calculated molecular mass for S78 was 184.9984 Da, matching the theoretical molecular mass of phosphoserine, 185.07 Da, confirming that S78 was phosphorylated (Fig. 4a, Supplementary Fig.  5). The fragment _77_TAKDFDTVAR_86_ from S78A mutant was observed; the calculated molecular mass of A78 was 89.0371 Da, matching the theoretical molecular mass of alanine, 89.09 Da, confirming that the mutated residue was an alanine (Fig. 4b, Supplementary Fig.  6).

Next, we examined the phosphorylation of a selection of TglT mutants in *E. coli* using Phos-tag SDS-PAGE^33^. wildtype TglT co-expressed with TakA showed two bands in the Phos-tag SDS-PAGE, a major upper band and a minor lower band (Fig. 4c). We analyzed the two species directly from the PAGE by LC-MS/MS, confirming that the upper band was the phosphorylated TglT (Supplementary Fig.  7), whereas the lower band was the non-phosphorylated TglT (Supplementary Fig.  8). Mutants S78A and S78D do not contain phosphorylation target, and we did not observe the upper band corresponding to the phosphorylated species. G62A (motif 1) accounted for the major loss of phosphorylation, however a minor phosphorylated portion (upper band) was faintly visible. D82A (motif 2), either expressed alone or co-expressed with TakA, exhibited a single band in Phos-tag PAGE. The band was slightly higher than the non-phosphorylated species observed for wildtype protein, but was lower than the phosphorylated band. Our crystallographic study showed that the D82A structure was non-phosphorylated. Collectively, D82A remained non-phosphorylated in the presence of TakA. K189N and D208A from motifs 3 and 4 impaired phosphorylation. The majority of these two mutants remained non-phosphorylated when co-expressed with TakA (Fig. 4c). E146Q (motif 2a) was the only exception as it was highly phosphorylated when co-expressed with TakA. We analyzed the lower and upper bands of E146Q by LC-MS/MS, confirming that while the upper major band was the phosphorylated E146Q, the lower minor band was the non-phosphorylated E146Q (Supplementary Figs.  9 and 10). In summary, with the exception of E146Q, all of the examined mutations within the conserved motifs of TglT impaired the phosphorylation of TglT.

### TakA phosphorylates TglT directly

**Fig. 5:**
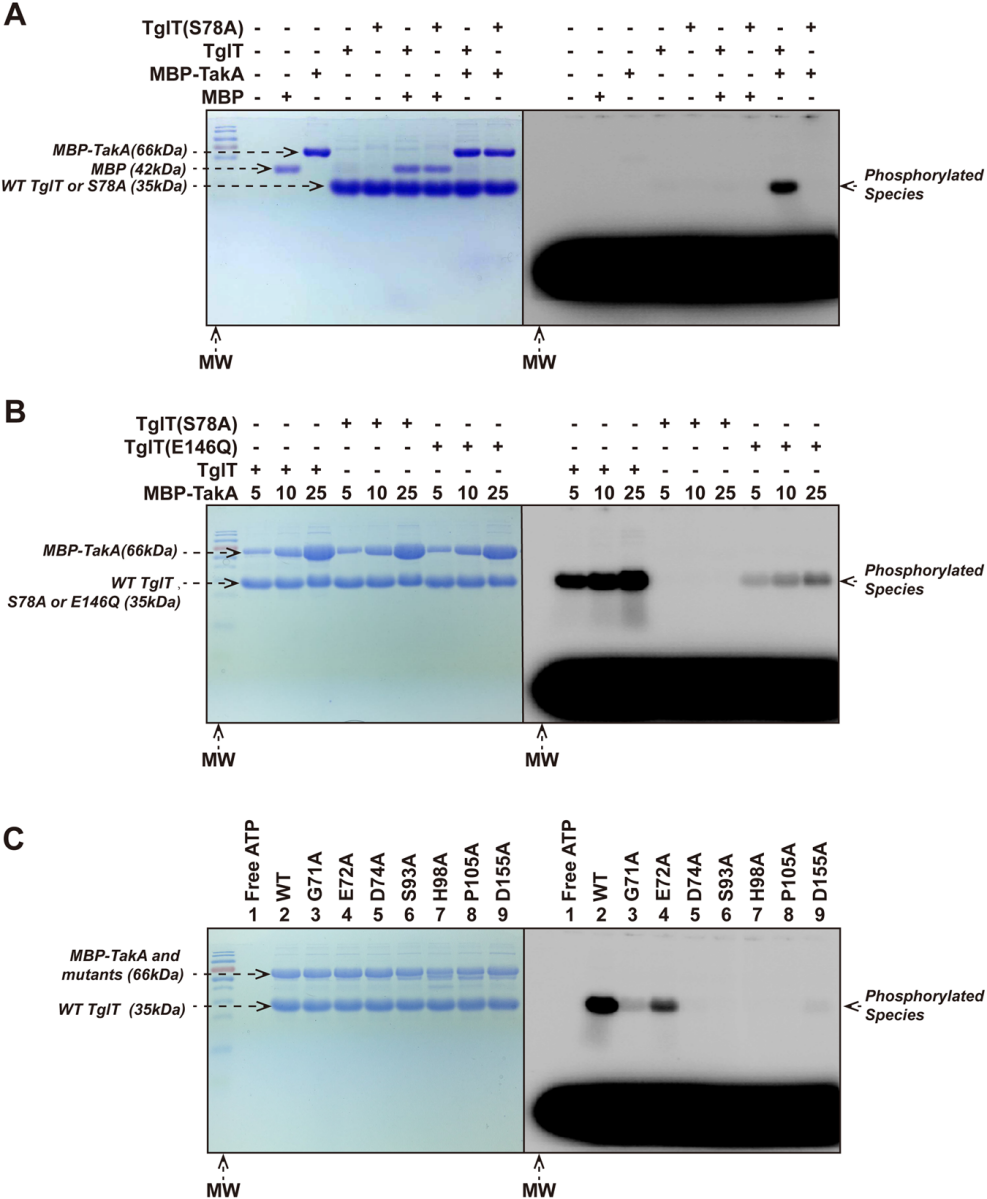
In vitro kinase assay TakA directly phosphorylates TglT in vitro. In each kinase reaction, radioactive probe [γ-^32^P] ATP was included to visualize the phosphorylated protein species. An excess of unlabeled ATP (6×10^4^ folds over the labeled ATP) was added to remove the non-covalently bound probe. The indicated kinase reactions were resolved by SDS-PAGE. The SDS-PAGE was first exposed to phosphoscreen and visualized with Typhoon imager (right); the same SDS-PAGE was then stained with Coomassie brilliant blue (left). **a** Purified, non-phosphorylated TglT and the S78A mutant were incubated with MBP-TakA or MBP (as nonspecific control). In the presence of MBP-TakA, phosphorylated TglT was detected, whereas TglT S78A was not phosphorylated. **b** Mutation S78A of TglT prevented TakA catalyzed phosphorylation, whereas E146Q impaired the phosphorylation. As the concentration of TakA increased, more phosphorylated TglT E146Q was observed, but the phosphorylation efficiency was lower than wildtype TglT. **c** Conserved residues of TakA were critical to its kinase activity. wildtype TglT was incubated with MBP-TakA (lane 2) and annotated TakA mutants including: G71A (lane 3), E72A (lane 4), D74A (lane 5), S93A (lane 6), H98A (lane 7), P105A (lane 8) and D155A (lane 9); lane 1: a free ATP control. While E72A retained similar activity as the wildtype protein, the other mutants showed loss of the activity to different extents. Especially, D74A, S93A, H98A, P105A and D155A showed complete loss of kinase activity. The conserved residues of TakA were identified by structure-based multiple sequence alignment, see Supplementary Fig.  13. Source data are provided as a Supplementary Data 2.

Our results demonstrated that TglT phosphorylation was always coupled with TakA co-expression, which strongly hinted the direct role of TakA in phosphorylation. To confirm this postulation, we devised an in vitro kinase assay using purified TakA (Supplementary Fig. 11A, B) and TglT. When incubating MBP-TakA with TglT (Fig. 5a), we detected radioactively labeled TglT species in SDS-PAGE. The signal vanished when the TglT S78A mutant was used in the reaction (MBP did not affect the kinase assay, Fig. 5a lane 3&7). The result provided the evidence that TakA is directly responsible for the phosphorylation of TglT at the residue S78 in vitro, which demonstrated that the antitoxin TakA is a novel serine protein kinase.

We found it difficult to understand why conserved motifs 1–4 were required for TglT phosphorylation, while E146Q in motif 2a did not cause loss of phosphorylation activity in *E. coli* (Fig. 4c). Therefore, we used the kinase assay to compare the phosphorylation efficiency of TglT mutant E146Q and wildtype TglT (Fig. 5b). Our results showed that TakA phosphorylated wildtype TglT with higher efficiency than E146Q, whereas TakA did not phosphorylate S78A even at the highest kinase concentration. Thus, motif 2a is also important in TakA catalyzed phosphorylation, although its role is not as critical as those of the other motifs. The discrepancy between the in vivo and the in vitro results was likely due to various reasons. The phosphorylation of TglT mutant E146Q by TakA in *E. coli* might benefit from the higher local concentration of TakA, longer incubation time and better physiological conditions (overnight *E. coli* culture at 37 °C vs 45 min in vitro incubation at 25^o^C), which eventually compensated phosphorylation impairment caused by E146Q.

### Phosphorylation of TglT leads to toxicity inhibition

**Fig. 6:**
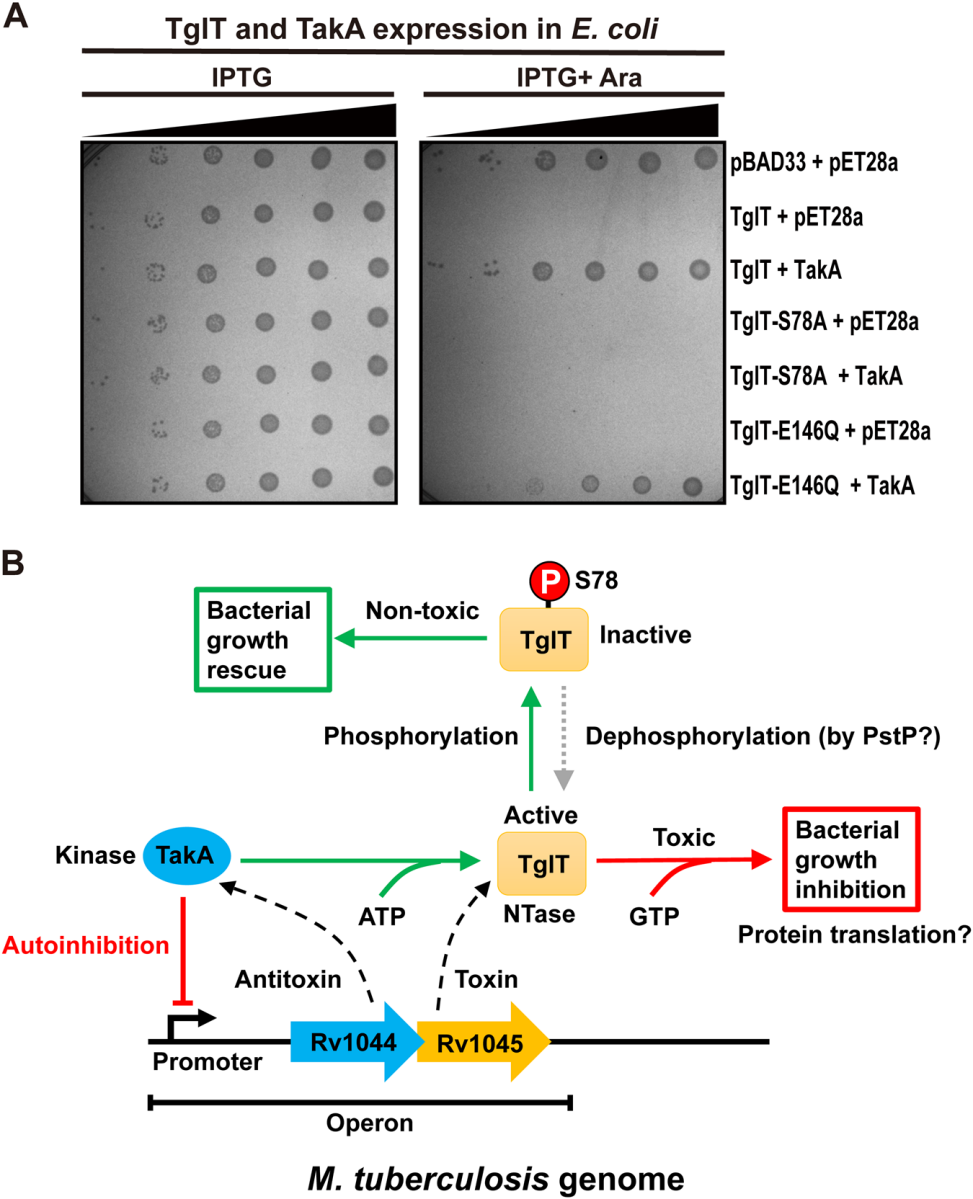
Phosphorylation of TglT results in toxicity neutralization and a proposed model of the Rv1044-Rv1045 TA system. **a** The antitoxicity mechanism of TakA involves phosphorylation of TglT at S78 site. BL21 cells expressing annotated proteins were spotted on M9 plates with 10-fold serial dilutions from the right to the left: 10^−1^ 10^−2^ 10^−3^, 10^−4^, 10^−5^ and 10^−6^, respectively. The plate on the left contained IPTG inducing TakA expression, whereas the plate on the right contained both IPTG and L-arabinose inducing toxin and antitoxin expressions. Bacterial growth was examined after overnight incubation. The toxicity of S78A was not rescued by TakA because the mutant cannot be phosphorylated. The neutralization of TglT E146Q toxicity was about 10-fold less efficient that that of wildtype TglT, because the phosphorylation of TglT E146Q by TakA was also less efficient (Fig. 5b). **b** The Rv1044 gene (blue arrow) encodes the atypical serine protein kinase TakA (blue ellipse), acting as the antitoxin, while the Rv1045 gene (orange arrow) encodes the guanylyltransferase TglT (orange rectangle), acting as the toxin. TakA negatively regulates its own promoter. TglT binds GTP and targets a vital cellular process, which leads to bacterial growth arrest. A possible cellular process targeted by TglT is protein translation. TakA recognizes TglT and phosphorylates the S78 residue, thus inhibiting the catalytic activity of TglT resulting in the neutralization of toxicity. The gray dashed arrow indicates the reversal of antitoxicity, which required an unknown phosphatase. The PstP phosphatase encoded by Mtb is a possible candidate. Source data are provided as a Supplementary Data 2.

Our crystallographic studies demonstrated that the presence of SEP78 not only introduced steric hindrance at the catalytic center but also altered the local electrostatic charging, both of which might hinder the substrate entry into the catalytic cavity (Fig. 2b, c). Thus, the phosphorylation at S78 may inhibit the activity of TglT, and in turn the toxic function. To test our hypothesis, we first compared the GTP binding affinity of the phosphorylated and non-phosphorylated TglT species (Supplementary Fig.  12). It was evident that the phosphorylated TglT exhibited lower GTP binding affinity than the non-phosphorylated TglT, which supports the theory that SEP78 impairs GTP binding. Next, we assessed the toxicity of TglT mutant S78D (functioning as a mimic of the phosphoserine). As expected, the S78D was non-toxic (Fig. 3e). Finally, we performed the kill-and-rescue experiment. TglT and variants were cloned into L-arabinose inducible expression vector pBAD33, whereas TakA was cloned to an IPTG inducible vector pET28a. Upon induction, the toxic effect of wildtype TglT was rescued by TakA efficiently (Fig. 6a). The S78A prevented phosphorylation, thus S78A was toxic and the toxicity was not rescuable. The E146Q mutant exhibited toxicity similar to the wildtype TglT, which was rescued by TakA. However, the rescuing of E146Q toxicity was much less efficient (~10 fold less) than the rescuing the toxicity of the wildtype TglT (Fig. 6a). This observation is consistent with our in vitro kinase assay, in which the TakA catalyzed phosphorylation of the mutant E146Q was less efficient than phosphorylation of the wildtype TglT (Fig. 5b). The phosphorylation of TglT is directly coupled with toxicity neutralization. Collectively, our results support an unusual antitoxicity mechanism, in which the antitoxin TakA is a novel protein kinase that phosphorylates the cognate toxin TglT, thereby inhibiting its toxicity.

### TakA is a novel atypical kinase

TakA shares limited homology with the structurally characterized proteins, thus, we employed the HHpred server^34^ to detect remote homology and to predict its structure. The best hit is *Rv2827c* (probability score = 99.59, PDB ID: 1ZEL) from Mtb. *Rv2827c* was predicted as the antitoxin of *R2827c-Rv2826c* TA^24^. *Rv2827c* contains an N-terminal wHTH domain and a C-terminal uncharacterized domain^35^. Based on our biochemical characterization and HHpred prediction, we postulated that TakA and *Rv2827c* belong to the same kinase family which encompasses an N-terminal wHTH domain and a C-terminal kinase domain. Thus, the conserved residues identified by sequence alignment might reveal catalytically important residues (Supplementary Fig.  13). To support our theory, we performed site-directed mutagenesis studies. As shown in Fig. 5c, G71, E72 and D74 are non-conserved residues located between wHTH and C-terminal kinase domain. While G71A and D74A led to major loss of kinase activity, E72A retained most of its activity. In contrast, nearly all mutations of the conserved residues S93, H98, P105 and D155 located within C-terminal domain resulted in the abolition of activity.

Mtb encodes 11 eukaryotic-like serine/threonine protein kinases (STPKs), from PknA to PknL^36^. Some STPKs are essential for Mtb growth, while others play an important role in virulence, adaption and survival in animal models or macrophages in vitro; however, none of these STPKs is similar to TakA.

In the search for structural homolog of *Rv2827c* using Dali server, we found a rather remote homolog, Rio2 (Z-score = 5.5). Rio2 belongs to an atypical serine/threonine kinase family^37,38^. Rio2 contains an N-terminal wHTH domain followed by a C-terminal protein kinase domain, a domain organization shared by *Rv2827c* and TakA. See Supplementary Data 3 for the Dali search full results.

### TakA negatively autoregulates *Rv1044-Rv1045* promoter

**Fig. 7:**
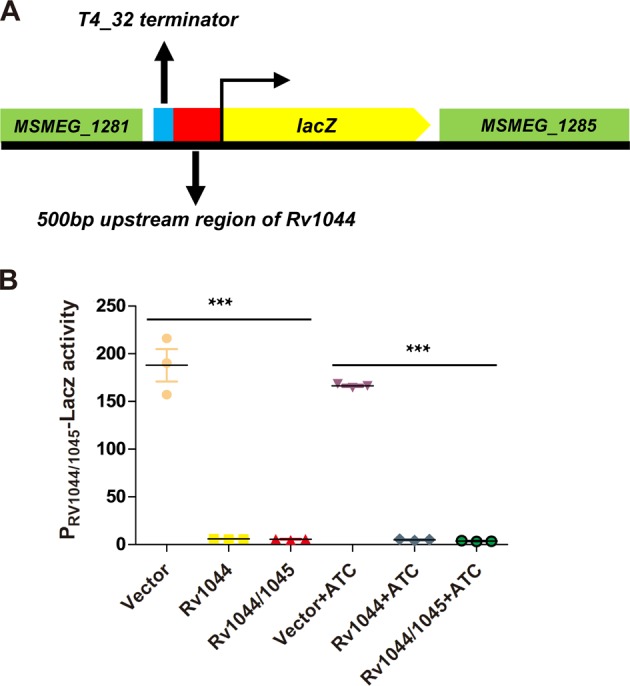
TakA (Rv1044) autoregulates the promoter of Rv1044-Rv1045 TA. **a** Schematic diagram of the P_*Rv1044*_*-lacZ* fusion reporter in *M. smegmatis*. A P_*Rv1044*_*-lacZ* reporter containing a 460 bp homology sequence of MSMEG_1282 gene, a T4_32 terminator (32 bp), a 500 bp upstream of Rv1044 gene and a 501 bp homology sequence of lacZ gene was inserted into the chromosome of *M. smegmatis*. **b** The promoter activity of Rv1044 was assessed with empty vector (vector), the plasmid expressing Rv1044 (the antitoxin) or the plasmid expressing Rv1044 and Rv1045 (antitoxin-toxin). The experiments were conducted in the absence and presence of anhydrotetracycline (ATC), respectively. Data are presented as a scatterplot showing the mean and standard error of three independent experiments (each dot represents a biological replicate). Asterisks represent statistically significant differences using the one way ANOVA analyses. ***p < 0.0001. Source data are provided as a Supplementary Data 2.

Given that TakA contains a putative N-terminal wHTH domain (Supplementary Fig.  13), it may play a role in promoter regulation. To investigate whether TakA autoregulates its own promoter, we constructed a P_*Rv1044*_*-lacZ* fusion reporter in *M. smegmatis* mc^2^-155 (Fig.  7a). Expression of *Rv1044* either alone or together with *Rv1045* strongly repressed transcription of the P_*Rv1044*_*-lacZ* fusion (Fig. 7b), indicating that TakA negatively autoregulates the *Rv1044-Rv1045* promoter.

## Discussion

Combining of our mutagenesis and functional studies, we concluded that the active site plays at least two distinct roles. First, it is the catalytic center of the putative guanylyltransferase, whose catalytic activity is responsible for the toxicity. We demonstrated that mutations in these conserved motifs led to the loss of toxicity (Fig. 3e, Supplementary Fig.  2), thus linking the active site to the toxic function. Second, the active site of TglT is also the substrate of the kinase TakA. In Phos-tag SDS-PAGE and in vitro kinase assays (Fig.  4c, Fig. 5a, b), we showed that mutations in the conserved motif impaired the kinase reaction catalyzed by TakA. Although phosphorylation of TglT mutant E146Q by TakA exhibited a similar-to-wildtype level in *E. coli*, the phosphorylation efficiency of purified TglT E146Q in vitro was much lower than wildtype level (Fig. 5b). These results suggest that in order to phosphorylate S78 of TglT, its entire active site is required by kinase TakA. This finding is consistent with our Co-IP experiment, which confirmed the direct interaction between TglT and TakA.

The known antitoxicity mechanisms are fundamentally different from what we report here, which involves phosphorylation of the toxin. It is intriguing that although the phosphorylation target S78 is located at the very center of the catalytic cavity and is highly conserved, this residue is nonessential for toxin function (Fig. 3e). Instead, S78 is more important for the regulation of toxicity. In non-phosphorylated form (S78 or S78A) the toxin is active and toxic, while in phosphorylated form (SEP78 or a phosphorylation mimic S78D), the toxin is inactive and the toxicity is inhibited. Collectively, the relation between antitoxin and toxin is that of kinase and substrate, and the antitoxicity mechanism is not based on physical interaction but on the phosphorylation of the toxin.

In response to stressful conditions, the inhibition of the toxin is terminated, typically via proteolytic degradation of the antitoxin, releasing the toxin to arrest cell growth, which may eventually lead to non-replicating state. Obviously, similar mechanism cannot be applied here because degrading the kinase TakA cannot dephosphorylate TglT. An Mtb phosphatase is necessary for TglT activation.

To investigate how TglT is dephosphorylated, we conducted protein phosphatase assays. We incubated the phosphorylated TglT (5 μM) with increasing amounts of the TakA kinase (0.4–2 μM); the reactions were then analyzed by Phos-tag SDS-PAGE. We did not observe the dephosphorylation of TglT (Supplementary Fig.  14A), suggesting that TakA kinase cannot reverse TglT phosphorylation. Under the same conditions, we also incubated TglT alone overnight (12 h), and no sign of dephosphorylation was visible (Supplementary Fig.  14A, lane 12); thus, TglT is most likely not be dephosphorylated by itself.

The phosphatases encoded by Mtb include 2 tyrosine phosphatases^39^ (*Rv2234* and *Rv0153c*) and 1 Ser/Thr phosphatase (*Rv0018c*)^40^. *Rv0018c* was initially referred to as ppp during Mtb genomic sequence analysis and later renamed PstP by Biotel and colleagues. They demonstrated that PstP can specifically dephosphorylate the PknB kinase (*Rv0014c*)^41^. Given that PstP is by far the only Ser/Thr phosphatase identified in Mtb, it is possible that PstP can dephosphorylate TglT. We therefore expressed PstP and tested its ability to dephosphorylate TglT. As shown in Supplementary Fig.  14A, PstP (0.4–2 μM) could completely dephosphorylate PknB (5 μM) in 30 minutes, indicating a phosphatase activity similar to that reported by Biotel et al.^41^. By contrast, we observed only slight increase in the dephosphorylated TglT species in the gel. To confirm the above observations, we extended the incubation time of the phosphatase assays to 2 h, 4 h and 12 h, respectively. While most of TglT still remained unchanged in the presence of MBP-TakA (Supplementary Fig.  14B), it became evident that PstP could indeed dephosphorylate TglT (Supplementary Fig.  14C), although in a rather unspecific manner. PstP required 12 h to fully dephosphorylate TglT, which was a slower rate than was observed for the dephosphorylation of its own substrate PknB. Taken together, these results suggest a possible role of PstP phosphatase in TglT dephosphorylation. Nevertheless, whether PstP phosphatase is able to reverse the TglT phosphorylation and finally leads to the activation of the TA system remains to be verified in vivo. Another challenging study is the existence of other unknown Ser/Thr phosphatases in Mtb that can specifically and efficiently dephosphorylate TglT. The solution of the above scientific questions is the focus of our ongoing studies, and the exact mechanism of TglT activation will be finally clarified.

To understand the potential cellular processes targeted by TglT, we employed RNA sequencing (RNA-seq) to study the transcriptomic profiles of bacteria expressing wildtype TglT and the nontoxic TglT mutant D82A. Sequencing libraries were constructed and sequenced, which resulted in the identification of 2604 differentially expressed genes (DEGs) between the bacteria expressing wildtype TglT and the D82A mutant (Supplementary Fig.  15A). Among these DEGs, there are 2078 up and 526 downregulated genes (Supplementary Fig.  15B, C and Supplementary dataset 1). Gene Ontology (GO) enrichment analysis of the DEGs showed that the toxin TglT induced gene expression changes were preferentially correlated with ribosome-related genes (Supplementary Fig.  15D). KEGG pathway enrichment analysis implied that TglT mostly alters ribosome and metabolic pathways (Supplementary Fig.  15E). Together, these analyses suggest that TglT most likely targets genes involved in protein translation. Notwithstanding, the RNA-seq is an indirect method to investigate the cellular process targeted by TglT. Assessing the protein translation level in bacteria overexpressing the toxin is an important experiment to understand whether the TglT inhibits translation in vivo. To identify the substrate of TglT via in vitro translation assay and mass spectrometry is our current goal, which will eventually reveal the enzymatic characteristics of this novel NTase.

In summary, our results suggest a model for the TA system *Rv1044-Rv1045* from Mtb (Fig. 6b). *Rv1044* encodes the atypical serine protein kinase TakA acting as the antitoxin, and *Rv1045* encodes the guanylyltransferase TglT acting as the toxin. The promoter of the *Rv1044-Rv0145* TA is negatively autoregulated by TakA. The catalytic activity of TglT requires GTP, which leads to toxicity and eventually bacterial growth arrest. Protein translation is a possible cellular process targeted by TglT. The toxic function of TglT is inhibited by TakA via phosphorylation of the toxin. TakA recognizes the active site of TglT and specifically phosphorylates residue S78. The phosphorylation of S78 results in steric hindrance and change in charging at the active site, thus inhibiting the catalytic activity of TglT. Consequently, the toxicity of TglT is neutralized. We postulate that a currently unidentified phosphatase is required to remove the phosphorylation of S78 and obliterate antitoxicity. The PstP phosphatase might participate in dephosphorylation of the toxin. Our findings not only reveal an unusual antitoxicity mechanism involving phosphorylation of the toxin, but also shed light on the function of two large uncharacterized protein families COG5340 and DUF1814, which are widespread in prokaryotes.

## Methods

### Reagents

All chemicals used in this study were purchased from Sigma-Aldrich unless otherwise specified.

### Bacterial strains and culture

Bacteria were grown at 37 °C in LB medium or M9 minimal media supplemented with 0.2% casamino acids. Antibiotics used were 100 μg/ml ampicillin, 50 μg/ml kanamycin and 25 μg/ml chloramphenicol, as indicated. Expression of the recombinant proteins was induced by l-arabinose and/or IPTG.

### Bioinformatic analysesn

Structural-based multi-sequence alignment was performed using programs MUSCLE^42^ and HHpred^34^. Program ESPript^43^ was used for generating multi-sequence alignment figures. Phosphorylation site prediction was conducted using NetPhos 3.1 server^32^.

### Plasmid construction

All plasmids used in this study are listed in Supplementary Table 2. The genes of toxin and antitoxin *Rv1045* and *Rv1044* were amplified from MTB H37Rv strain genomic DNA by PCR. The genomic DNA was a generous gift from Hairong Huang’s lab, Beijing chest hospital, Capital Medical University. Plasmids used for expression in this study include pET28a (+) (Novagene), pBAD/Myc-His-A (Thermo Fisher Scientific), pBAD33, pMAL-c5x (NEW ENGLAND BioLabs) and pETDuet-1 (Novagene).

Constructing plasmids for *E. coli* toxicity assays, *Rv1045* gene was amplified by PCR, treated with restriction enzymes KpnI and HindIII, and was subsequently inserted into pBAD33, encoding wildtype TglT with a C-terminal 6xHis-tag.

Constructing plasmids for toxin neutralization, *Rv1044* gene was amplified by PCR, treated with NdeI and XhoI enzymes and was inserted to pET28a, encoding antitoxin TakA with a N-terminal 6xHis-tag.

Constructing plasmid for Co-IPs, *Rv1045* gene was amplified by PCR; the coding sequence for and a FLAG-tag, DYKDDDDK was introduced to the C-terminus of the protein via 3′ primer. The DNA fragment was cloned into pET28a between NcoI and XholI restriction sites. *Rv1044* gene was amplified by PCR and cloned between NcoI and HindIII sites of pBAD/Myc-His-A, yielding the C-Myc-tag plus 6xHis-tag protein.

For MBP tagged TakA expression, *Rv1044* gene was amplified by PCR; the fragment was cloned between NcoI and HindIII restriction sites of pMAL-c5x, yielding N-terminal MBP tagged TakA.

For crystallization of TglT and variants, *Rv1045* gene was cloned between NdeI and XhoI restriction sites of pET28a (+), yielding N-terminal His-tagged protein. *Rv1044* gene was cloned between restriction sites NcoI and HindIII of MCS-1 of pET-Duet-1, yielding non-tagged protein. TglT and variants were expressed alone or co-expressed with TakA depending on the purpose of the experiments, reducing the toxicity of obtaining phosphorylated proteins.

For expressing non-phosphorylated wildtype TglT protein, pET28-n-6his-*Rv1045* was co-transformed with pMAL-c5x empty plasmid, yielding minimal quantity sufficient for biochemical characterizations.

All plasmids encoding protein mutants were constructed using site-directed mutagenesis (QuickChange). All plasmids were verified by DNA sequencing.

### Protein expression and purification

The plasmids encoding TglT and variants were transformed to *E. coli* BL21(DE3) competent cells. The bacteria culture was grown in LB medium at 37 °C. The induction was initiated by adding isopropyl β-D-1-thiogalactopyranoside (IPTG) to 0.5 mM when the culture reached a density of OD_600_ = 1.0. The culture was cooled to 18°C and the shaking continued overnight. The bacteria were then harvested by centrifugation at 5000 rpm for 20 minutes. To purify the proteins, the cell pellets were re-suspended in a lysis buffer containing 50 mM Tris pH = 8.0, 150 mM NaCl, 10 mM imidazole, 1 mM PMSF and 1 mM 2-mercaptoethanol. The cells were disrupted by ultrasonication on ice. The cell debris was removed by centrifugation and the clarified supernatant was loaded to Ni-NTA resin (GE healthcare) pre-equilibrated with the lysis buffer, and the bound protein was eluted with an elution buffer containing 300 mM imidazole. The eluate was then loaded to HiTrap Q HP column (GE Healthcare) and the protein was eluted with a linear gradient of 75–1000 mM NaCl. The proteins were finally purified using Superdex 75 HR 10/30 column (GE Healthcare) pre-equilibrated with a buffer containing 20 mM Tris-HCl pH = 8.0, 100 mM NaCl and 2 mM DTT. To prepare selenomethionine derivative, the plasmid was transformed to B834 (DE3) competent cells grown in LeMASTER medium (Molecular Dimensions) containing l-selenomethionine. The purification followed the same protocols described above.

To prepare the non-phosphorylated wildtype TglT for substrate in kinase assay, we co-transformed pMAL-c5x empty plasmid (encoding only MBP protein) and pET28a-n-6His-*Rv1045* (His-TglT) into *E. coli* BL21 cells. The purification followed the same protocols described above. We found that simultaneous induction of both plasmids reduced TglT toxicity, which eventually allowed us obtaining a minimal but sufficient quantity of non-phosphorylated TglT for biochemical characterizations. We verified the non-phosphorylated TglT protein by LC-MS/MS (Supplementary Fig.  16) prior to the kinase assay. The reduced toxicity was probably attributed to the overexpression of MBP. Although MBP was irrelevant to antitoxicity, its overexpression might simply counterbalance the overall production of TglT in bacteria and had kept the concentration of TglT below lethal level.

To express MBP-tagged TakA, plasmid pMAL-*Rv1044* was transformed to BL21 (DE3) competent cells. The induction was initiated by adding IPTG to 0.5 mM when the bacterial culture reached OD_600_ = 1.0. The culturing continued at 18 °C overnight. Bacteria were harvested and re-suspended in the lysis buffer and disrupted by ultrasonication. The supernatant was clarified by centrifugation and passed over amylose resin (NEW ENGLAND BioLabs) at 4 °C. The resin was washed with a washing buffer containing 50 mM Tris pH = 8.0, 150 mM NaCl. The protein was eluted using an elution buffer containing 50 mM Tris, pH = 8.0, 150 mM NaCl and 10 mM maltose. The eluate was loaded to HiTrap S HP column (GE Healthcare) and eluted with the linear gradient of 75–1000 mM NaCl. The eluted protein was finally purified with Superdex 200 HR 10/30 column (GE Healthcare) equilibrated with 20 mM Tris-HCl pH = 8.0, 100 mM NaCl.

### Co-immunoprecipitation (Co-IP)

*Rv1045* and *Rv1044* genes were amplified by PCR and cloned to pET28a and pBAD/Myc-His-A vectors, between the restriction sites NcoI/XhoI and NcoI/HindIII, respectively. Plasmid pET28-c-FLAG-*Rv1045* encodes TglT with C-terminal FLAG-tag (termed TglT-FLAG), whereas plasmid pBAD/Myc-H-*Rv1044* encodes a C-terminal Myc tagged and C-terminal His-tagged TakA (termed TakA-Myc-His). The plasmids were transformed to BL21 DE3 competent cells. A single colony was picked to inoculate 2 ml of LB medium and incubated overnight at 37 °C. Then, the cells were diluted by 10^2^ folds in 20 ml fresh LB medium. The cultures were grown at 37 °C till OD_600_ = 0.4. The expression of TglT-FLAG and TakA-Myc-His was induced by the addition of 0.5 mM IPTG and 0.2% (w/v) l-arabinose. The culturing was continued for at 37 °C for 3 h. The bacteria cells were collected by centrifugation (3000 rpm, 30 min) and re-suspended in an IP-lysis buffer containing 50 mM Tris, pH = 8.0, 150 mM NaCl and protease inhibitor cocktail (Roche). The cells were disrupted by ultrasonication and the cell debris were removed by centrifugation (4 °C, 15,000 rpm for 20 min). The supernatants were passed through a 0.45 µm filter before use. 300 μl of the filtrated supernatant were added to 20 μl of ANTI-FLAG M2 magnetic beads or Anti-Myc magnetic beads. The incubation was overnight at 4 °C. The beads were washed with 500 μl IP-lysis buffer for 5 times using spin column kit (Sigma-Aldrich) before heat denaturing and loading to SDS-PAGE. The gels were then transferred to PVDF membranes (Bio-Rad) using Criterion blotter (Bio-Rad). The membrane was finally analyzed by western blot.

### Growth curve

Plasmids encoding TglT and variants were transformed to BL21(DE3) competent cells. The bacteria were grown in liquid LB medium containing chloramphenicol (25 μg/ml) at 37 °C to OD_600_ = 0.6. The cultures were diluted to OD_600_ = 0.2 before induction by adding 0.2% l-arabinose. To generate growth curve, the bacteria cultures were incubated at 37 °C, and OD_600_ was measured every 2 h.

### Growth inhibition assay

Plasmids encoding TglT or variants were transformed to BL21 DE3 competent cells. The overnight culture was used to inoculate (1:100) 20 ml fresh liquid M9 medium supplemented with 25 μg/ml chloramphenicol and was grown to OD_600_ = 0.8. The bacteria were then diluted by 10-folds serial dilution, from 10^−1^ to 10^−6^. Each dilution was finally spotted (1 μl) onto M9 agar plates containing chloramphenicol, with and without inducer 0.2% l-arabinose. The plates were incubated overnight at 37 °C before taking pictures.

### Western blot

Tagged proteins (His, Myc or FLAG epitope), separated by SDS-PAGE, were transferred to a PVDF membrane (Bio-Rad) using a Criterion blotter (Bio-Rad). The membrane was washed with Western wash buffer (TBS + 0.1% Tween 20) and blocked for 1 h with 5% skim milk powder at room temperature. The membrane was washed 3 × 5 min with wash buffer before incubating with primary antibody for 2 h. The membrane was again washed 3 × 5 min before incubating with the secondary antibody for 1 h at room temperature and finally washed 3 × 5 min. Primary antibodies used were rabbit monoclonal antibodies, anti-FLAG (Sigma-Aldrich), anti-Myc (Sigma-Aldrich) and anti-His antibodies (Cell Signaling Technology) at a dilution 1:1000; and the secondary antibody was goat anti-rabbit IgGHRP (Li-Cor) at a dilution of 1:10,000. Membranes were finally visualized by odyssey (Li-Cor).

### Protein phosphatase assay

The protein phosphatase assay was performed as previously described with minor modifications. Recombinant PstP_1-240aa_ and PknB_1-279aa_ proteins were expressed and purified following the methods published previously. The reaction mixture contained 20 mM Tris-HCl pH = 8.0, 100 mM NaCl, 1 mM DTT, 2 mM MnCl_2_ and 5 μM protein substrate (TglT or PknB_1-279aa_). The reaction was initiated by adding MBP-TakA or PstP phosphatase (concentrations, 0.4 μM, 1 μM or 2 μM). The mixtures were incubated at 35 °C. The reaction products were finally loaded onto Phos-tag™ SDS-PAGE (Wako Pure Chemical Industries) and stained with Coomassie R250.

### Crystallization and structure determination

To gain structural insights into *Rv1044-Rv1045* TA we carried out crystallographic studies. Due to the intrinsic toxicity, the overexpression of the wildtype TglT completely arrested *E. coli* growth and eventually led to cell lysis after induction overnight. We therefore devised two strategies: (a) Introduction of mutations to the conserved motifs, which abolished or attenuated the toxicity, resulting in mutants that were expressible. These conserved motifs include motifs I & II from the catalytic motifs of the DNA polβ family and motifs III & IV exclusive to the DUF1814 family^26^. (b) Co-expression with the antitoxin TakA, which neutralizes the toxicity of TglT, and allows the expression of the wildtype toxin.

The toxin TglT was crystallized in a hanging-drop vapor-diffusion system at 22 °C. The protein was concentrated to 8 mg/ml. The crystallization was carried out by mixing 1 μl of protein sample with 1 μl of reservoir buffer containing 0.1 M Bis-Tris pH = 6.5, 0.2 M magnesium chloride hexahydrate and 25% polyethylene glycol 3350 (v/v). The crystals of TglT appeared after 5 days; they exhibited a rod-like shape with an average size of 0.3–0.5 millimeters. The cryocooling of the crystals was performed by soaking the crystals in the reservoir buffer containing 10% ethylene glycol for 30–60 s before flash freezing in liquid nitrogen.

There are no known TglT homologous structures for molecular replacement, however protein sequence contains four internal methionine residues, which allowed exploiting the single-wavelength anomalous diffraction method for phasing. Redundant X ray diffraction data for TglT mutant D82A crystal containing selenomethionine were collected at beamline BL19U of Shanghai synchrotron radiation facility (SSRF). The X-ray source had a wavelength of 0.979 Å, at the peak adoption edge of the Se atoms. D82A crystals belonged to the space group P3_2_21 and diffracted the X-ray to 1.9 Å. The data was processed using the XDS package^44^. Single copy of TglT was found in an asymmetric unit, and significant anomalous differences correlation was observed up to 2.8 Å. Software AUTOSHARP/SHARP^45^ was used to locate 4 Se atoms and to calculate the initial phases, yielding an interpretable electron density map. A preliminary atomic model of D82A was built automatically by ARP/wARP^46^. The model was then completed by manual building using the Coot^47^, and it was finally refined using the PHENIX software^48^. In the final model, we located 290 out of total 293 residues of TglT, including 4 selenomethionine. The X ray diffraction datasets for the wildtype TglT crystals and other mutants reported in this paper were collected in SSRF and at PX III beamline at the Swiss Light Source, Paul Scherrer Institute (Villigen Switzerland). Wavelength used for data collection were 0.98 Å. All structures were solved by molecular replacement with Phaser program^49^ using D82A as the search model.

In the finally refined the structures, the Ramachandran statistics are: TglT D82A (alone expressed, Se-Met crystal), 96.88% favored, 2.77% allowed and 0.35% outliers; TglT wildtype (co-expressed with TakA), 96.83% favored, 3.17% allowed and zero outliers; TglT S78A (alone expressed), 97.22% favored, 2.43% allowed and 0.35% outliers; TglT E146Q (alone expressed), 96.18% favored, 3.82% allowed and zero outliers; TglT S78A (co-expressed with TakA), 96.86%, 2.79% and 0.35% outliers; TglT D82A (co-expressed with TakA), 96.53% favored, 3.47% allowed and zero outliers; TglT E146Q (co-expressed with TakA), 96.10% favored, 3.90% allowed and zero outliers.

The statistics of data collection, reduction and structure refinement are summarized in Tables 1 and 2. The final 2Fo-Fc map of the crystal structure of wildtype TglT is shown in Supplementary Fig.  17.

**Table 1:** Data collection and refinement statistics 1.

	TglT D82A alone expressed Se-Met crystal	TglT WT co-expressed with TakA	TglT S78A alone expressed	TglT E146Q alone expressed
	(PDB ID: 6J7T)	(PDB ID: 6J7S)	(PDB ID: 6J7Q)	(PDB ID: 6J7O)
Data collection				
Space group	P3_2_21	P3_2_21	P3_2_21	P3_2_21
Cell dimensions				
a, b, c (Å)	94.69, 94.69, 61.84	95.89, 95.89, 68.89	95.41, 95.41, 68.13	95.44, 95.44, 69.21
α, β, γ (°)	90.00, 90.00, 120.00	90.00, 90.00, 120.00	90.00, 90.00, 120.00	90.00, 90.00, 120.00
Resolution (Å)	49.37 (1.86)	28.56 (2.10)	39.07 (1.85)	39.28 (1.90)
*R*_sym_	0.176 (1.33)	0.081 (0.746)	0.056 (0.961)	0.078 (1.12)
*I*/σ*I*	14.94 (1.65)	23.47 (3.18)	22.42 (2.45)	17.42 (2.01)
Completeness (%)	97.3 (83.4)	99.9 (99.9)	99.9 (99.5)	99.8 (99.4)
Redundancy	18.67 (12.46)	11.04 (11.23)	9.98 (10.12)	9.97 (9.98)
Refinement				
Resolution (Å)	49.37 (1.90)	28.56 (2.10)	39.08 (1.85)	39.28 (1.90)
No. reflections	48568	21589	30867	28961
*R*_work_/*R*_free_	0.1883/0.2057 (0.4065/0.4211)	0.1994/0.2311 (0.2463/0.2872)	0.1939/0.2198 (0.3017/0.3345)	0.1953/0.2162 (0.3109/0.3843)
No. atoms				
Protein	2251	2214	2233	2228
Ligand/ion	0	10	0	0
Water	196	204	217	196
*B*-factors				
Protein	33.03	41.92	48.07	49.07
Ligand/ion	0	40.87	0	0
Water	38.18	40.94	48.36	48.04
R.m.s. deviations				
Bond lengths (Å)	0.003	0.004	0.009	0.007
Bond angles (°)	0.706	0.731	0.843	0.924

Values in parentheses are for highest-resolution shell.

**Table 2:** Data collection and refinement statistics 2.

	TglT S78A co-expressed with TakA	TglT D82A co-expressed with TakA	TglT E146Q co-expressed with TakA
	(PDB ID: 6J7R)	(PDB ID: 6J7N)	(PDB ID: 6J7P)
Data collection			
Space group	P3_2_21	P3_2_21	P3_2_21
Cell dimensions			
a, b, c (Å)	95.65, 95.65, 68.65	95.50,95.50,68.18	94.69,94.69,67.25
α, β, γ (°)	90.00, 90.00, 120.00	90.00, 90.00, 120.00	90.00, 90.00, 120.00
Resolution (Å)	47.82 (2.30)	47.75 (2.29)	47.34 (2.63)
*R*_sym_	0.075 (0.520)	0.078 (0.906)	0.149 (0.744)
*I*/σ*I*	11.69 (2.52)	7.25 (0.87)	4.45 (0.94)
Completeness (%)	96.3 (98.3)	95.6 (94.7)	89.5 (75.6)
Redundancy	4.55 (4.66)	2.80 (2.74)	2.47 (1.95)
Refinement			
Resolution (Å)	47.82 (2.30)	47.75 (2.29)	47.34 (2.63)
No. reflections	15851	29769	9935
*R*_work_/*R*_free_	0.2156/0.2517 (0.2562/0.2904)	0.2112/0.2672 (0.3235/0.3552)	0.2719/0.2945 (0.3852/0.3991)
No. atoms			
Protein	2213	2232	2198
Ligand/ion	0	0	10
Water	53	21	10
*B*-factors			
Protein	57.81	77.97	67.04
Ligand/ion	0	0	66.42
Water	57.32	73.36	53.24
R.m.s. deviations			
Bond lengths (Å)	0.010	0.007	0.004
Bond angles (°)	0.993	0.832	0.598

*Values in parentheses are for highest-resolution shell.

### Toxicity and antitoxicity assay

*Rv1045* and *Rv1044* genes were amplified and cloned into pBAD33 and pET28a vectors respectively, encoding TglT-His and His-TakA (Supplementary Table 2). The plasmids were transformed to BL21 (DE3) competent cells. Single colonies were isolated and inoculated in 2 ml LB medium overnight. The culture was used to inoculate (1:100) 20 ml of M9 medium; the cultures were grown to OD_600_ = 0.8. The bacteria were then streaked on M9 plate supplemented with inducer IPTG (16 μM) or l-arabinose (0.2% w/v); the plates were included overnight incubation at 37 °C before the examination of cell growth.

### Kill-and-rescue assay

Compatible plasmid pairs (pBAD33 and pET28a with and without *Rv1044* and *Rv1045* genes) were co-transformed to BL21(DE3) competent cells and grown overnight. The overnight cultures were diluted 100 folds in 20 ml fresh M9 minimal medium containing 25 μg/ml chloramphenicol and 50 μg/ml kanamycin, and the diluted cultures were grown to a density OD_600_ = 0.8 at 37 °C. The cultures were then further diluted by 10-fold serial dilution, from 10^−1^ to 10^−6^. Each dilution was finally spotted (1 μl) onto M9 agar plates containing chloramphenicol, kanamycin, with and without inducers 0.2% l-arabinose and 16 μM IPTG. The plates were incubated overnight at 37 °C before bacterial growth was examined.

### Phos-tag SDS-PAGE

TglT and its variants either expressed alone or co-expressed with TakA were purified as described in the Protein expression and purification section. To analyzed the proteins, 2 µl samples were loaded to a precast SuperSep Phos-tag™ SDS-PAGE (Wako Pure Chemical Industries). The Phos-tag molecules in the gel specifically bind protein phosphorylation site, thus maximizing the separation of the phosphorylated TglT from the non-phosphorylated species. The electrophoresis was conducted in Tris-glycine SDS running buffer at room temperature; the voltage was constant at 180 V for 70 min. The gel was then stained with Coomassie brilliant blue R250 and photographed.

### NTP binding assay

The purified His-TglT protein at various concentration was incubated with 3.3 nM (final concentration) of [α-^32^P] NTPs in a binding buffer containing 20 mM Tris-HCl, 1.5 mM MgCl_2_, 100 mM NaCl, pH = 8.0 at 37 °C for 1 h. The resulting mixtures were then resolved by 10% Tris-Glycine native-PAGE running on ice for 70 min at 100 volts (constant). The gels were exposed overnight to a phosphor screen and analyzed using GE Typhoon^TM^ FLA 7000 biomolecular imager. To investigate GTP binding capacity of TglT, a concentration series of TglT were prepared. The highest concentration was 16 μM, followed by 13 steps of two-fold serial dilutions, resulting the following concentrations: 8 μM, 4 μM, 2 μM, 1 μM, 0.5 μM, 0.25 μM, 0.125 μM, 0.0625 μM, 0.0313 μM, 0.0156 μM, 0.0078 μM, 0.0039 μM and 0.002 μM. Only the top four concentrations (16 μM, 8 μM, 4 μM and 2 μM) were used for studying binding with the other NTPs. In the competition binding experiments, 8 μM TglT protein was pre-incubated with 3.3 nM of radioactively labeled GTP and the unlabeled NTPs were then added to the mixtures as competitors, reaching the final concentrations of 0.5 μM, 4 μM and 16 μM, respectively.

### In vitro kinase assay

The kinase reaction mixture (20 μl) contained 10 mM Tris-HCl pH = 8.0, 75 mM NaCl, 10 mM MgCl_2_ and 10 mM MnCl_2_, 2.5 nM [γ-^32^P] ATP, 150 μM unlabeled ATP and 50 μM of protein substrate (TglT or variants). The reaction was initiated by adding 5 μM MBP-TakA kinase and the incubation time was for 45 min at 25 °C. The samples were then analyzed by 12% SDS-PAGE. The electrophoresis started with a constant voltage of 180 V for 40 min. To analyze the results, the SDS-PAGE was first exposed to a phosphor screen and visualized by GE Typhoon^TM^ FLA 7000 biomolecular imager. The same SDS-PAGE was then stained with Coomassie Brilliant Blue R250 to visualize all proteins.

### Promoter activity assay

The lacZ reporter gene was first amplified using plasmid pMV261-null-lacZ^50^ as PCR template, and then inserted into the chromosome of *M. smegmatis* mc^2^-155 to replace MSMEG_1283_1284 genes using the method described previously^51^. Next, a DNA fragment containing a 460 bp homology sequence of MSMEG_1282 gene, a T4_32 terminator (32 bp), a 500 bp upstream of *Rv1044* gene (Supplementary Table 3) and a 501 bp homology sequence of lacZ gene were synthesized and inserted into the above strain using the methods described previously^52^, resulting in the *M. smegmatis* P_*Rv1044*_*-lacZ* reporter strain (Fig.  7a). To construct the plasmids for expression of *Rv1044* and *Rv1044-Rv1045*, their encoding genes were amplified from Mtb genomic DNA by PCR and were subcloned into a shuttle vector pYC601 containing anhydrotetracycline (ATC)-inducible promoter using Seamless Cloning and Assembly Kit. The resulting plasmids and the empty vector were further transformed into the *M. smegmatis* P_*Rv1044*_*-lacZ* reporter strain and then used for *Rv1044* promoter activity analysis. 100 ng/ml ATC was added to induce the expression of the antitoxin and the toxin-antitoxin complex. β-galactosidase promoter activity assays were performed according to the standard Miller method^53^.

### RNA sequencing (RNA-seq)

Plasmids encoding wildtype TglT and mutant D82A were transformed to BL21 (DE3) competent cells. The bacteria were grown in liquid LB medium containing chloramphenicol (25 μg/ml) at 37 °C to OD_600_ = 0.6. The cultures were diluted to OD_600_ = 0.2 before induction by adding 0.2% L-arabinose. 0.5 ml of bacteria cultures was removed after 2 hours induction at 37 °C and quickly frozen with liquid nitrogen for subsequent RNA-Seq (each with three replicates). Our bacteria growth curve showed that toxicity of TglT manifested about 2 h after induction. Total RNA was extracted from each sample and RNA-seq was performed using BGISEQ-500 sequencing system^54^. The sequence reads were mapped to the reference genome *E.coli* BL21 using HISAT and Bowtie2 software^55,56^. Gene expression level for each sample was quantified and normalized utilizing the software package RSEM tool^56,57^. Differentially expressed genes (DEGs) between bacteria expressing wildtype TglT and mutant D82A were analyzed using DEGseq method. DEGs were defined by a fold change ≥ 2 and adjusted *P*-value ≤ 0.001^58^. Gene Ontology (GO) enrichment analysis^59^ and KEGG pathway enrichment analysis^60^ of DEGs were conducted based on Gene Ontology Consortium(http://geneontology.org/) and KEGG database (https://www.kegg.jp/kegg/pathway.html).

### Statistics and reproducibility

All experiments were performed in independent biological triplicate and the results of replicates were consistent.

One-way ANOVA analyses was performed using GraphPad Prism 7 (GraphPad, CA, USA). Details of the number of biological replicates are described in the figure legends and Methods. Error bars represent standard deviation. *P* value of <0.0001 was considered as extremely significant, which is indicated with ***.

### Reporting summary

Further information on research design is available in the Nature Research Reporting Summary linked to this article.

## Supplementary information

Supplementary Information

Supplementary Data 1

Supplementary Data 2

Description of Additional Supplementary Files

Reporting Summary

Peer Review File

Supplementary Data 3

## Data Availability

All data relevant to this study are supplied in the manuscript and supplementary files or are available from the corresponding author upon request. Coordinates and structure factors are deposited in the Protein Data Bank with the PDB entries: 6J7T, 6J7S, 6J7Q, 6J7O, 6J7R, 6J7N and 6J7P. RNA-Seq data are deposited in NCBI Sequence Read Archive with accession No. PRJNA560278. The original data underlying Figs. 1b–g, 3e, 4c, 5a–c, 6a, 7b and Supplementary Figs.  2, 11, 12, 14 is provided in the Supplementary Data 2. The RNA-seq data underlying Supplementary Fig. 15 is provided in the Supplementary Data 1.
