# Supplementary material for: Characterization of a toxin-antitoxin system in Mycobacterium tuberculosis suggests neutralization by phosphorylation as the antitoxicity mechanism
Source: Commun Biol. 2020 May 7;3:216. doi: 10.1038/s42003-020-0941-1 (PMC7205606; doi:10.1038/s42003-020-0941-1)
Supplement: Supplementary file 1 — Supplementary Information [file 42003_2020_941_MOESM1_ESM.pdf]

Supplementary Figures

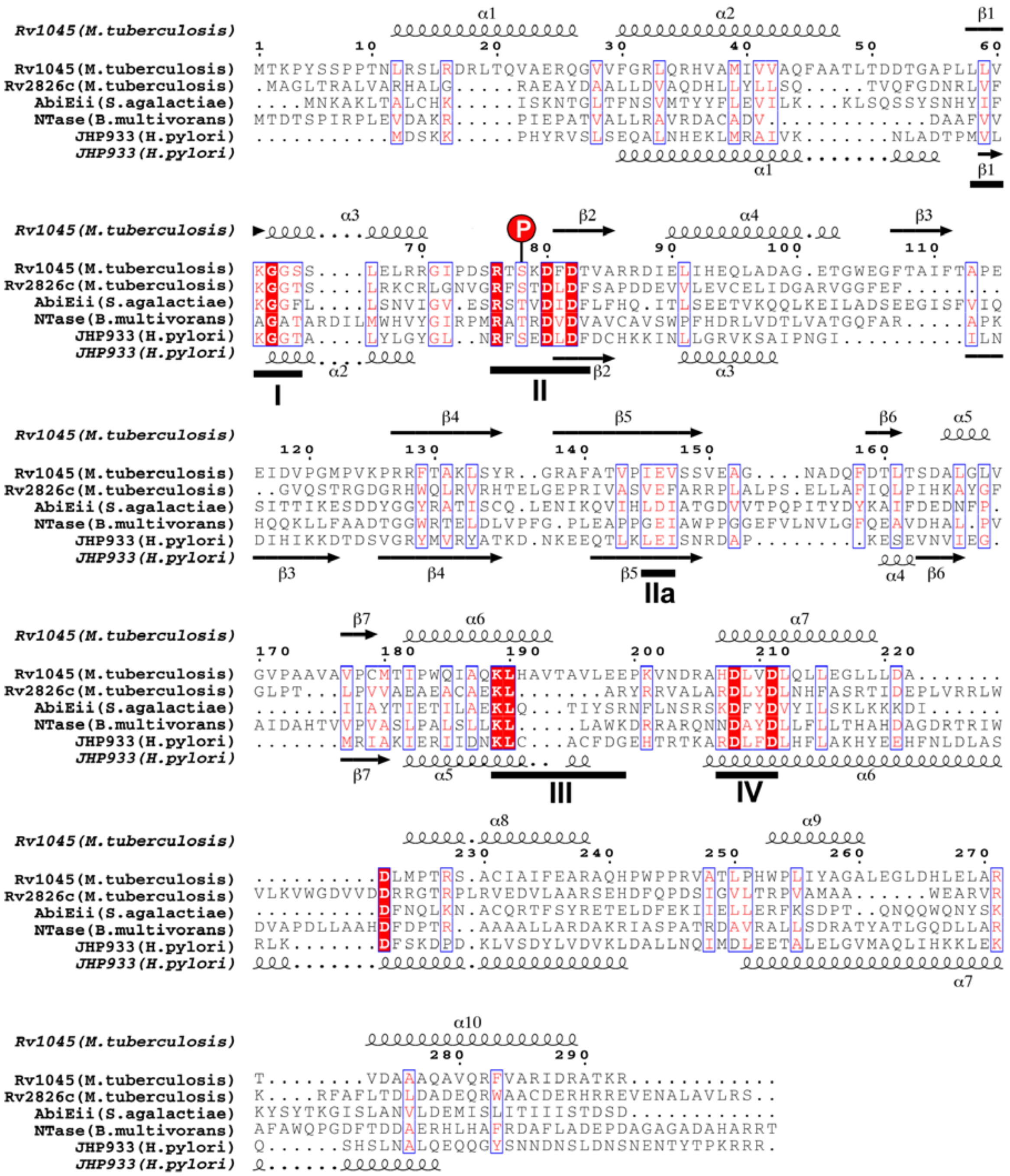

Supplementary Figure 1 Structure-based multiple sequence alignment of DUF1814 family

Sequences of DUF1814 family members Rv1045, Rv2826c from *M. tuberculosis*, AbiEii from *S. agalactiae*, NTase from *B. multivarans*, and JHP933 form *H, pylori* were aligned by software MUSCLE; and annotated secondary structure elements of

TglT (Rv1045) and JHP933 were aligned to the top and the bottom of the sequences using ESPript server. Residues in red indicate the conserved reisdues; residues with red backgroud indicated the invariant residues. Conserved motifs are indicted at the bottom of the sequences. The phsophoryation target S78 of TglT is highlighted by a phosphate symbol with red background.

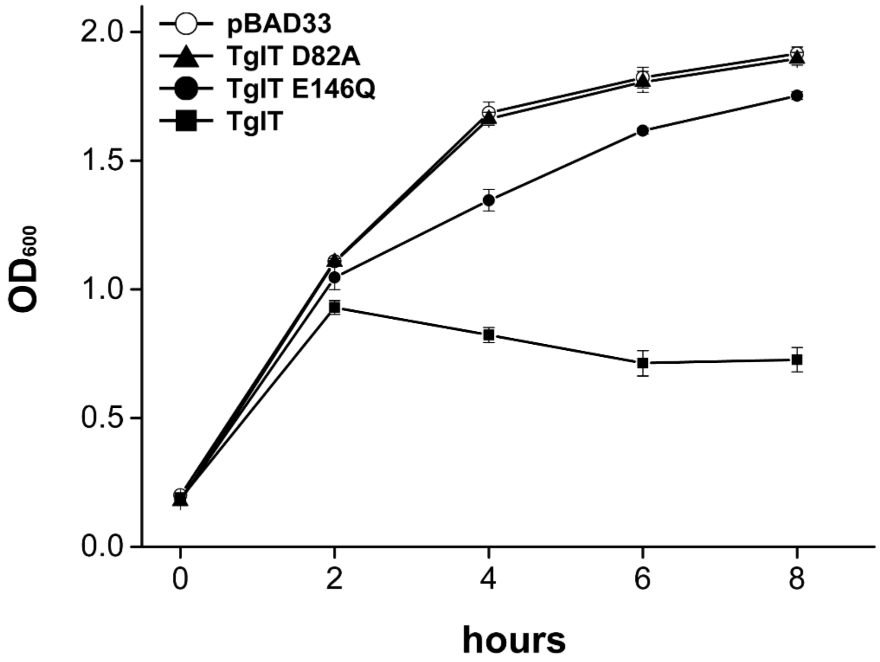

Supplementary Figure 2 Initial growth rates of BL21 cells after the induction of expression of TglT and variants.

BL21 competent cells were transformed with the indicated expression plasmids. The bacteria cultures were grown to OD_{600}=0.6 and then diluted to OD_{600}=0.2 before adding L-arabinose for induction. The growth of the bacteria culture was monitored every two hours after induction. Bacteria densities (OD_{600}) was plotted as the function of time. At least three independent measurements were taken for each time point. Bacteria transformed with TglT expression plasmid exhibited growth inhibition 2 hours post induction. E146Q mutant showed attenuated toxicity comparing to wildtype protein, whereas D82A was completely non-toxic. Data shown are mean OD_{600} value ± SD (n = 3).

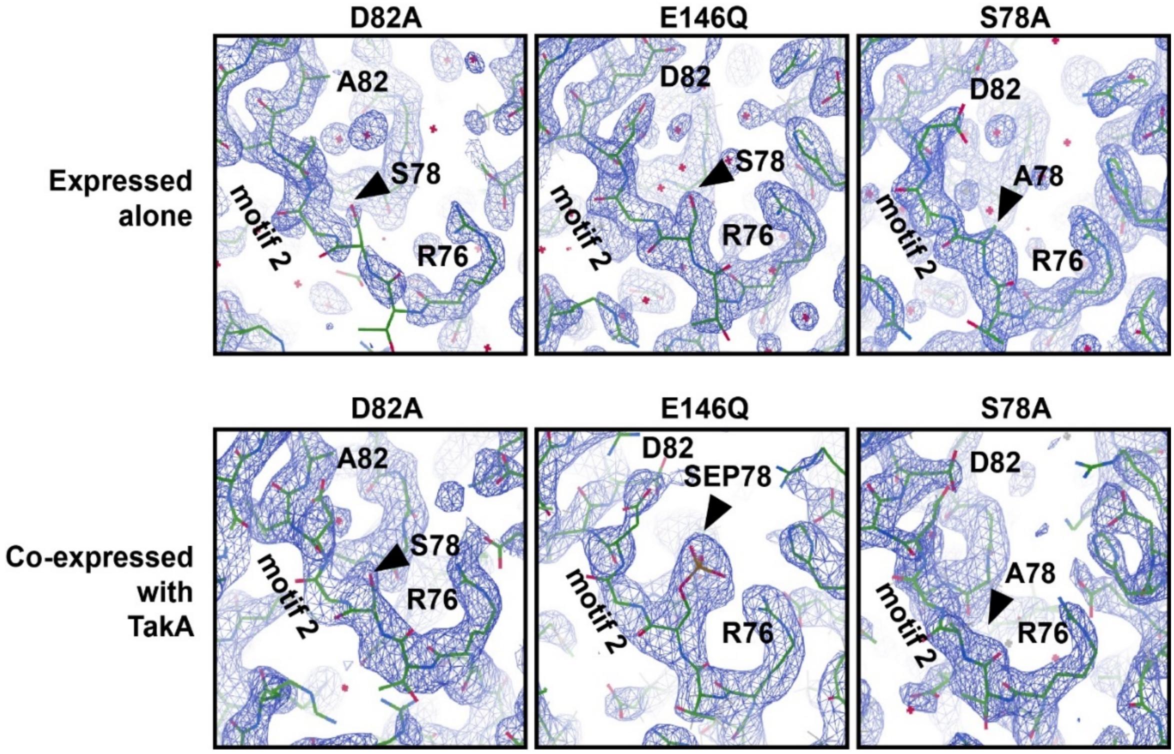

Supplementary Figure 3 Crystallographic study of TglT variants revealed that residue S78 was phosphorylated when the protein was co-expressed with the antitoxin TakA.

Composite omit maps (with annealing) were calculated for crystal structures of TglT mutants D82A, E146Q and S78A. The black boxes show the magnified view of the phosphorylation at the active site of TglT variants. The map (contour 1.5 s) is shown with blue mesh, which is superimposed with the stick model of the protein. Key residues at the active site are labeled. The phosphorylation target residue 78 is indicated with black triangles.

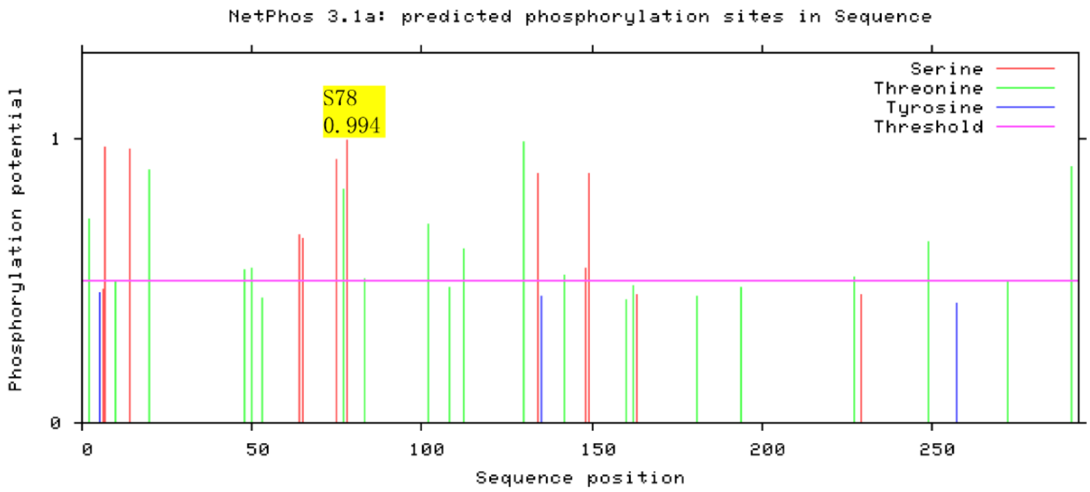

# Sequence	78	S	DSRTSKDFD	0. 994	unsp	YES
# Sequence	78	S	DSRTSKDFD	0. 496	cdc2	.
# Sequence	78	S	DSRTSKDFD	0. 479	RSK	.
# Sequence	78	S	DSRTSKDFD	0. 443	CaM-II	.
# Sequence	78	S	DSRTSKDFD	0. 439	PKA	.
# Sequence	78	S	DSRTSKDFD	0. 435	GSK3	.
# Sequence	78	S	DSRTSKDFD	0. 397	CKII	.
# Sequence	78	S	DSRTSKDFD	0. 387	CKI	.
# Sequence	78	S	DSRTSKDFD	0. 358	DNAPK	.
# Sequence	78	S	DSRTSKDFD	0. 335	p38MAPK	.
# Sequence	78	S	DSRTSKDFD	0. 299	PKG	.
# Sequence	78	S	DSRTSKDFD	0. 294	ATM	.
# Sequence	78	S	DSRTSKDFD	0. 251	PKC	.
# Sequence	78	S	DSRTSKDFD	0. 177	cdk5	.
# Sequence	78	S	DSRTSKDFD	0. 087	PKB	.

Supplementary Figure 4 NetPhos 3.1 prediction

Prediction of phosphorylation site in TglT sequence generated from NetPhos 3.1 server. The score of the prediction ranges from 0-1; score higher than threshold 0.5 indicates a significant prediction. S78 from motif 2 in context $_{74}$-DSRTSKDFD-$_{82}$ received the highest score 0.994 among all significant hits. The software found that S78 is the phosphorylation site for unspecified kinase, rather than any of 14 characterized kinases whose scores were all below significant threshold.

{MATRIX SCIENCE} **Mascot Search Results**

Peptide View

MS/MS Fragmentation of **GIPDSRTSKDFDTVAR**
Found in **tr|A0A045JBD4|A0A045JBD4_MYCTX**, Nucleotidyl transferase of uncharacterised function (DUF1814) OS=Mycobacterium tuberculosis GN=B7R63_05680 PE=4 SV=1

Match to Query 8929: 1843.838096 from(461.966800,4+) intensity(19859188.0000)
Title: File43610 Spectrum1939 scans: 4176
Data file F:\DATA\2017\201712\20171214\BPI_14819_RV1054.raw.mgf

Click mouse within plot area to zoom in by factor of two about that point
Or, Plot from 100 to 1900 Da Full range
Label all possible matches ○ Label matches used for scoring ◉

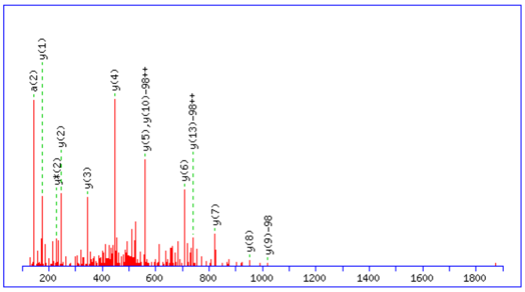

Monoisotopic mass of neutral peptide Mr(calc): 1843.8466
Fixed modifications: Carbamidomethyl (C) (apply to specified residues or termini only)
Variable modifications:
S8 : Phospho (ST), with neutral losses 97.9769(shown in table), 0.0000
Ions Score: 71 **Expect:** 5.1e-007
Matches : 13/252 fragment ions using 18 most intense peaks (help)

#	a	a^{++}	a^{*}	a^{*++}	b	b^{++}	b^{*}	b^{*++}	Seq.	y	y^{++}	y^{*}	y^{*++}	#
1	30.0338	15.5206			58.0287	29.5180			G					16
2	143.1179	72.0626			171.1128	86.0600			I	1689.8555	845.4314	1672.8289	836.9181	15
3	240.1707	120.5890			268.1656	134.5864			P	1576.7714	788.8893	1559.7449	780.3761	14
4	355.1976	178.1024			383.1925	192.0999			D	1479.7186	740.3630	1462.6921	731.8497	13
5	442.2296	221.6185			470.2245	235.6159			S	1364.6917	682.8495	1347.6652	674.3362	12
6	598.3307	299.6690	581.3042	291.1557	626.3257	313.6665	609.2991	305.1532	R	1277.6597	639.3335	1260.6331	630.8202	11
7	699.3784	350.1928	682.3519	341.6796	727.3733	364.1903	710.3468	355.6770	T	1121.5586	561.2829	1104.5320	552.7696	10
8	768.3999	384.7036	751.3733	376.1903	796.3948	398.7010	779.3682	390.1878	S	***1020.5109***	510.7591	1003.4843	502.2458	9
9	896.4948	448.7511	879.4683	440.2378	924.4898	462.7485	907.4632	454.2352	K	***951.4894***	476.2483	934.4629	467.7351	8
10	1011.5218	506.2645	994.4952	497.7513	1039.5167	520.2620	1022.4901	511.7487	D	***823.3945***	412.2009	806.3679	403.6876	7
11	1158.5902	579.7987	1141.5636	571.2855	1186.5851	593.7962	1169.5586	585.2829	F	***708.3675***	354.6874	691.3410	346.1741	6
12	1273.6171	637.3122	1256.5906	628.7989	1301.6121	651.3097	1284.5855	642.7964	D	***561.2991***	281.1532	544.2726	272.6399	5
13	1374.6648	687.8360	1357.6383	679.3228	1402.6597	701.8335	1385.6332	693.3202	T	***446.2722***	223.6397	429.2456	215.1264	4
14	1473.7332	737.3703	1456.7067	728.8570	1501.7281	751.3677	1484.7016	742.8544	V	***345.2245***	173.1159	328.1979	164.6026	3
15	1544.7703	772.8888	1527.7438	764.3755	1572.7653	786.8863	1555.7387	778.3730	A	***246.1561***	123.5817	229.1295	115.0684	2
16									R	***175.1190***	88.0631	158.0924	79.5498	1

Supplementary Figure 5. LC-MS/MS analysis of purified wildtype TglT.

A fragment$_{71}$GIPDSRTSKDFDTVAR$_{86}$ belonging to TglT was observed. In which, the molecular mass of residue S78 (underlined) calculates: (1020.5109-951.4894) * 1 + 97.9769 (neutral losses, orange underline) + 18 (1 water) = 184.9984Da (orange box indicated that data used for calculation). The theoretical molecular mass of phosphoserine is 185.07Da. Thus, S78 was phosphorylated.

Mascot Search Results

Peptide View

MS/MS Fragmentation of **TAKDFDTVAR**
Found in **S78A**

Match to Query 1886: 1122.565288 from(562.289920,2+) intensity(10497550.0000)
Title: File43612 Spectrum2637 scans: 3948
Data file F:\DATA\2017\201712\20171214\bpi_14819\BPI_14819_S78A.raw.mgf

Click mouse within plot area to zoom in by factor of two about that point
Or, Plot from 50 to 1050 Da Full range
Label all possible matches ○ Label matches used for scoring ◉

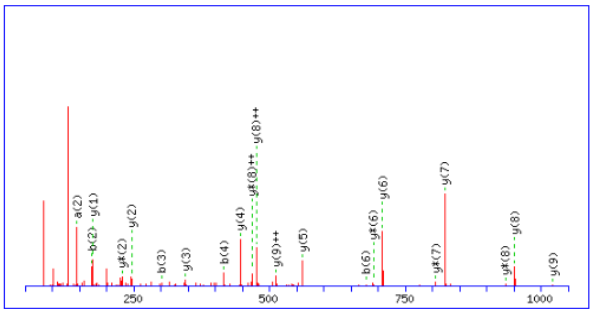

Monoisotopic mass of neutral peptide Mr(calc): 1122.5669
Fixed modifications: Carbamidomethyl (C) (apply to specified residues or termini only)
Ions Score: 59 Expect: 1.4e-006
Matches : 21/100 fragment ions using 42 most intense peaks (help)

#	a	a^{++}	a*	a^{*++}	b	b^{++}	b*	b^{*++}	Seq.	y	y^{++}	y*	y^{*++}	#
1	74.0600	37.5337			102.0550	51.5311			T					10
2	**145.0972**	73.0522			**173.0921**	87.0497			A	***1022.5265***	511.7669	1005.5000	503.2536	9
3	273.1921	137.0997	256.1656	128.5864	**301.1870**	151.0972	284.1605	142.5839	K	***951.4894***	476.2483	934.4629	467.7351	8
4	388.2191	194.6132	371.1925	186.0999	**416.2140**	208.6106	399.1874	200.0974	D	***823.3945***	412.2009	806.3679	403.6876	7
5	535.2875	268.1474	518.2609	259.6341	563.2824	282.1448	546.2558	273.6316	F	***708.3675***	354.6874	691.3410	346.1741	6
6	650.3144	325.6608	633.2879	317.1476	**678.3093**	339.6583	661.2828	331.1450	D	***561.2991***	281.1532	544.2726	272.6399	5
7	751.3621	376.1847	734.3355	367.6714	779.3570	390.1821	762.3305	381.6689	T	***446.2722***	223.6397	429.2456	215.1264	4
8	850.4305	425.7189	833.4040	417.2056	878.4254	439.7164	861.3989	431.2031	V	***345.2245***	173.1159	328.1979	164.6026	3
9	921.4676	461.2375	904.4411	452.7242	949.4625	475.2349	932.4360	466.7216	A	***246.1561***	123.5817	229.1295	115.0684	2
10									R	***175.1190***	88.0631	158.0924	79.5498	1

Supplementary Figure 6. LC-MS/MS analysis of purified TglT mutant S78A. A fragment $_{77}$TAKDFDTVAR$_{86}$ belonging to TglT S78A mutant was observed. In which, the molecular mass of residue A78 (underlined) calculates: (1022.5265-951.4894) * 1 + 18 (1 water) = 89.0371Da (orange box indicated that data used for calculation). The theoretical molecular mass of phosphoserine is 185.07Da. Thus, residue 78 was alanine, non-phosphorylated.

Mascot Search Results

Peptide View

MS/MS Fragmentation of TSKDFDTVARR
Found in tr|A0A045JBD4|A0A045JBD4_MYCTX, Nucleotidyl transferase of uncharacterised function (DUF1814) OS=Mycobacterium tuberculosis GN=B7R63_05680 PE=4 SV=1

Match to Query 7048: 1374.630132 from(459.217320,3+) intensity(771675.4375)
Title: File4302 Spectrum905 scans: 2846
Data file F:\LCMS\7yue\20180708\16444\BPI_16444_2_WT_POS.mgf

Click mouse within plot area to zoom in by factor of two about that point
Or, Plot from 50 to 850 Da Full range
Label all possible matches Label matches used for scoring

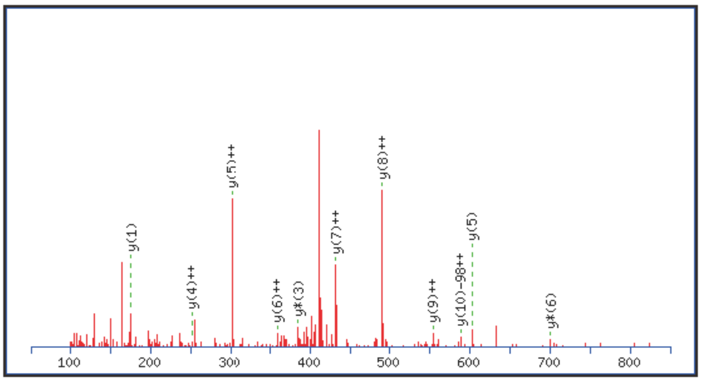

Monoisotopic mass of neutral peptide Mr(calc): 1374.6293
Fixed modifications: Carbamidomethyl (C) (apply to specified residues or termini only)
Variable modifications:
S2 : Phospho (ST), with neutral losses 97.9769(shown in table), 0.0000
Ions Score: 13 **Expect:** 1
Matches : 11/184 fragment ions using 56 most intense peaks (help)

#	a	a++	a*	a*++	b	b++	b*	b*++	Seq.	y	y++	y*	y*++	#
1	74.0600	37.5337			102.0550	51.5311			T					11
2	143.0815	72.0444			171.0764	86.0418			S	1176.6120	588.8096	1159.5854	580.2964	10
3	271.1765	136.0919	254.1499	127.5786	299.1714	150.0893	282.1448	141.5761	K	1107.5905	554.2989	1090.5640	545.7856	9
4	386.2034	193.6053	369.1769	185.0921	414.1983	207.6028	397.1718	199.0895	D	979.4956	490.2514	962.4690	481.7381	8
5	533.2718	267.1395	516.2453	258.6263	561.2667	281.1370	544.2402	272.6237	F	864.4686	432.7380	847.4421	424.2247	7
6	648.2988	324.6530	631.2722	316.1397	676.2937	338.6505	659.2671	330.1372	D	717.4002	359.2037	700.3737	350.6905	6
7	749.3464	375.1769	732.3199	366.6636	777.3414	389.1743	760.3148	380.6610	T	**602.3733**	301.6903	585.3467	293.1770	5
8	848.4149	424.7111	831.3883	416.1978	876.4098	438.7085	859.3832	430.1953	V	501.3256	251.1664	484.2990	242.6532	4
9	919.4520	460.2296	902.4254	451.7163	947.4469	474.2271	930.4203	465.7138	A	402.2572	201.6322	385.2306	193.1190	3
10	1075.5531	538.2802	1058.5265	529.7669	1103.5480	552.2776	1086.5215	543.7644	R	331.2201	166.1137	314.1935	157.6004	2
11									R	**175.1190**	88.0631	158.0924	79.5498	1

Supplementary Figure 7. LC-MS/MS analysis of the upper band of wildtype TglT sample in Phos-tag SDS-PAGE

A fragment $_{77}$TSKDFDTVARR$_{87}$ belonging to TglT was identified. In which, the molecular mass of residue S78 (underlined) calculates: (588.8096-554.2989) * 2 + 97.9769 (neutral losses, orange underline) + 18(1 water) = 184.9983Da (orange box indicated that data used for calculation). The theoretical molecular mass of phosphoserine is 185.07Da. Thus, S78 was phosphorylated.

{MATRIX SCIENCE} Mascot Search Results

Peptide View

MS/MS Fragmentation of TSKDFDTVARR
Found in tr|A0A045JBD4|A0A045JBD4_MYCTX, Nucleotidyl transferase of uncharacterised function (DUF1814) OS=Mycobacterium tuberculosis GN=B7R63_05680 PE=4 SV=1

Match to Query 6229: 1294.664742 from(432.562190,3+) intensity(176276.6094)
Title: File4318 Spectrum726 scans: 2640
Data file F:\LCMS\7yue\20180708\16444\BPI_16444_1_WT.mgf

Click mouse within plot area to zoom in by factor of two about that point
Or, Plot from 50 to 1000 Da Full range
Label all possible matches Label matches used for scoring

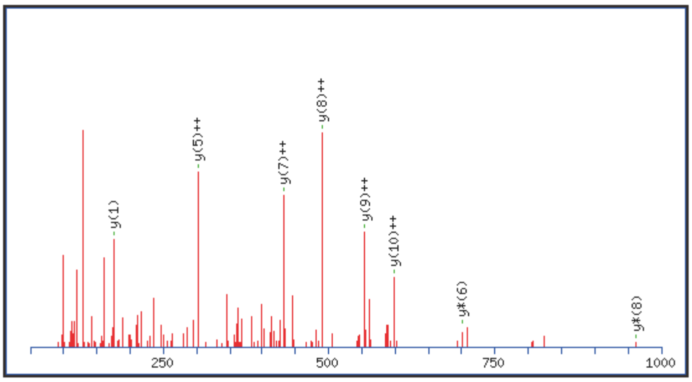

Monoisotopic mass of neutral peptide Mr(calc): 1294.6630
Fixed modifications: Carbamidomethyl (C) (apply to specified residues or termini only)
Ions Score: 24 **Expect:** 0.009
Matches : 8/112 fragment ions using 17 most intense peaks (help)

#	a	a++	a*	a*++	b	b++	b*	b*++	Seq.	y	y++	y*	y*++	#
1	74.0600	37.5337			102.0550	51.5311			T					11
2	161.0921	81.0497			189.0870	95.0471			S	1194.6226	597.8149	1177.5960	589.3016	10
3	289.1870	145.0972	272.1605	136.5839	317.1819	159.0946	300.1554	150.5813	K	1107.5905	554.2989	1090.5640	545.7856	9
4	404.2140	202.6106	387.1874	194.0974	432.2089	216.6081	415.1823	208.0948	D	979.4956	490.2514	962.4690	481.7381	8
5	551.2824	276.1448	534.2558	267.6316	579.2773	290.1423	562.2508	281.6290	F	864.4686	432.7380	847.4421	424.2247	7
6	666.3093	333.6583	649.2828	325.1450	694.3042	347.6558	677.2777	339.1425	D	717.4002	359.2037	700.3737	350.6905	6
7	767.3570	384.1821	750.3305	375.6689	795.3519	398.1796	778.3254	389.6663	T	602.3733	301.6903	585.3467	293.1770	5
8	866.4254	433.7164	849.3989	425.2031	894.4203	447.7138	877.3938	439.2005	V	501.3256	251.1664	484.2990	242.6532	4
9	937.4625	469.2349	920.4360	460.7216	965.4575	483.2324	948.4309	474.7191	A	402.2572	201.6322	385.2306	193.1190	3
10	1093.5636	547.2855	1076.5371	538.7722	1121.5586	561.2829	1104.5320	552.7696	R	331.2201	166.1137	314.1935	157.6004	2
11									R	175.1190	88.0631	158.0924	79.5498	1

Supplementary Figure 8. LC-MS/MS analysis of the lower band of wildtype TglT sample in Phos-tag SDS-PAGE

A fragment $_{77}$TSKDFDTVARR$_{87}$ belonging to TglT was identified. In which, the molecular mass of residue S78 (underlined) calculates: (597.8149-554.2989) * 2 + 18(1 water) = 105.032Da (orange box indicated that data used for calculation). The theoretical molecular mass of serine is 105.09Da. Thus, S78 was non-phosphorylated.

Mascot Search Results

Peptide View

MS/MS Fragmentation of **TSKDFDTVARR**
Found in **tr|A0A045JBD4|A0A045JBD4_MYCTX**, Nucleotidyl transferase of uncharacterised function (DUF1814) OS=Mycobacterium tuberculosis GN=B7R63_05680 PE=4 SV=1

Match to Query 6508: 1374.630132 from(459.217320,3+) intensity(881110.2500)
Title: File4306 Spectrum688 scans: 2721
Data file F:\LCMS\7yue\20180708\16444\BPI_16444_3_E146Q_PHOS.mgf

Click mouse within plot area to zoom in by factor of two about that point
Or, Plot from 50 to 750 Da Full range
Label all possible matches ○ Label matches used for scoring ◉

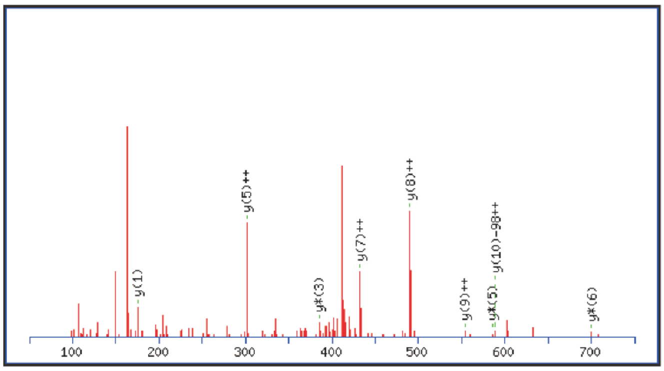

Monoisotopic mass of neutral peptide Mr(calc): 1374.6293
Fixed modifications: Carbamidomethyl (C) (apply to specified residues or termini only)
Variable modifications:
S2 : Phospho (ST), with neutral losses 97.9769(shown in table), 0.0000
Ions Score: 12 **Expect:** 1
Matches : 9/184 fragment ions using 30 most intense peaks (help)

#	a	a++	a*	a*++	b	b++	b*	b*++	Seq.	y	y++	y*	y*++	#
1	74.0600	37.5337			102.0550	51.5311			T					11
2	143.0815	72.0444			171.0764	86.0418			S	1176.6120	588.8096	1159.5854	580.2964	10
3	271.1765	136.0919	254.1499	127.5786	299.1714	150.0893	282.1448	141.5761	K	1107.5905	554.2989	1090.5640	545.7856	9
4	386.2034	193.6053	369.1769	185.0921	414.1983	207.6028	397.1718	199.0895	D	979.4956	490.2514	962.4690	481.7381	8
5	533.2718	267.1395	516.2453	258.6263	561.2667	281.1370	544.2402	272.6237	F	864.4686	432.7380	847.4421	424.2247	7
6	648.2988	324.6530	631.2722	316.1397	676.2937	338.6505	659.2671	330.1372	D	717.4002	359.2037	700.3737	350.6905	6
7	749.3464	375.1769	732.3199	366.6636	777.3414	389.1743	760.3148	380.6610	T	602.3733	301.6903	585.3467	293.1770	5
8	848.4149	424.7111	831.3883	416.1978	876.4098	438.7085	859.3832	430.1953	V	501.3256	251.1664	484.2990	242.6532	4
9	919.4520	460.2296	902.4254	451.7163	947.4469	474.2271	930.4203	465.7138	A	402.2572	201.6322	385.2306	193.1190	3
10	1075.5531	538.2802	1058.5265	529.7669	1103.5480	552.2776	1086.5215	543.7644	R	331.2201	166.1137	314.1935	157.6004	2
11									R	175.1190	88.0631	158.0924	79.5498	1

Supplementary Figure 9. LC-MS/MS analysis of the upper band of TglT E146Q sample in Phos-tag SDS-PAGE

A fragment $_{77}$TSKDFDTVARR$_{87}$ belonging to TglT was identified. In which, the molecular mass of residue S78 (underlined) calculates: (588.8096-554.2989) * 2 + 97.9769 (neutral losses, orange underline) + 18(1 water) = 184.9983Da (orange box indicated that data used for calculation). The theoretical molecular mass of phosphoserine is 185.07Da. Thus, S78 was phosphorylated.

Mascot Search Results

Peptide View

MS/MS Fragmentation of TSKDFDTVARR
Found in tr|A0A045JBD4|A0A045JBD4_MYCTX, Nucleotidyl transferase of uncharacterised function (DUF1814) OS=Mycobacterium tuberculosis GN=B7R63_05680 PE=4 SV=1

Match to Query 5410: 1294.666782 from(432.562870,3+) intensity(582001.4375)
Title: File4304 Spectrum623 scans: 2574
Data file F:\LCMS\7yue\20180708\16444\BPI_16444_3_E146Q.mgf

Click mouse within plot area to zoom in by factor of two about that point
Or, Plot from 50 to 1000 Da Full range
Label all possible matches ○ Label matches used for scoring ◉

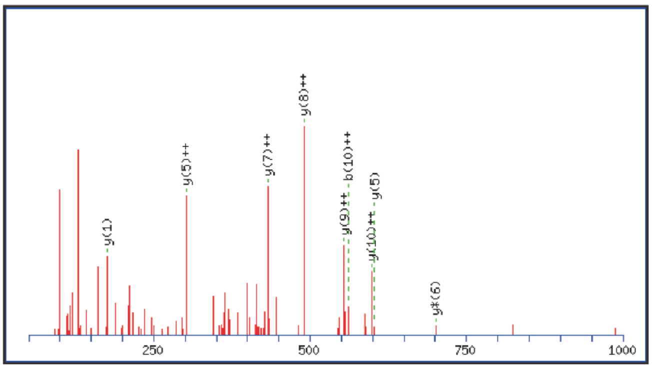

Monoisotopic mass of neutral peptide Mr(calc): 1294.6630
Fixed modifications: Carbamidomethyl (C) (apply to specified residues or termini only)
Ions Score: 21 **Expect:** 0.86
Matches : 9/112 fragment ions using 21 most intense peaks (help)

#	a	a++	a*	a*++	b	b++	b*	b*++	Seq.	y	y++	y*	y*++	#
1	74.0600	37.5337			102.0550	51.5311			T					11
2	161.0921	81.0497			189.0870	95.0471			S	1194.6226	597.8149	1177.5960	589.3016	10
3	289.1870	145.0972	272.1605	136.5839	317.1819	159.0946	300.1554	150.5813	K	1107.5905	554.2989	1090.5640	545.7856	9
4	404.2140	202.6106	387.1874	194.0974	432.2089	216.6081	415.1823	208.0948	D	979.4956	490.2514	962.4690	481.7381	8
5	551.2824	276.1448	534.2558	267.6316	579.2773	290.1423	562.2508	281.6290	F	864.4686	432.7380	847.4421	424.2247	7
6	666.3093	333.6583	649.2828	325.1450	694.3042	347.6558	677.2777	339.1425	D	717.4002	359.2037	700.3737	350.6905	6
7	767.3570	384.1821	750.3305	375.6689	795.3519	398.1796	778.3254	389.6663	T	**602.3733**	301.6903	585.3467	293.1770	5
8	866.4254	433.7164	849.3989	425.2031	894.4203	447.7138	877.3938	439.2005	V	501.3256	251.1664	484.2990	242.6532	4
9	937.4625	469.2349	920.4360	460.7216	965.4575	483.2324	948.4309	474.7191	A	402.2572	201.6322	385.2306	193.1190	3
10	1093.5636	547.2855	1076.5371	538.7722	1121.5586	**561.2829**	1104.5320	552.7696	R	331.2201	166.1137	314.1935	157.6004	2
11									R	**175.1190**	88.0631	158.0924	79.5498	1

Supplementary Figure 10. LC-MS/MS analysis of the lower band of TglT E146Q sample in Phos-tag SDS-PAGE

A fragment $_{77}$TSKDFDTVARR$_{87}$ belonging to TglT was identified. In which, the molecular mass of residue S78 (underlined) calculates: (597.8149-554.2989) * 2 + 18(1 water) = 105.032Da (orange box indicated that data used for calculation). The theoretical molecular mass of serine is 105.09Da. Thus, S78 was non-phosphorylated.

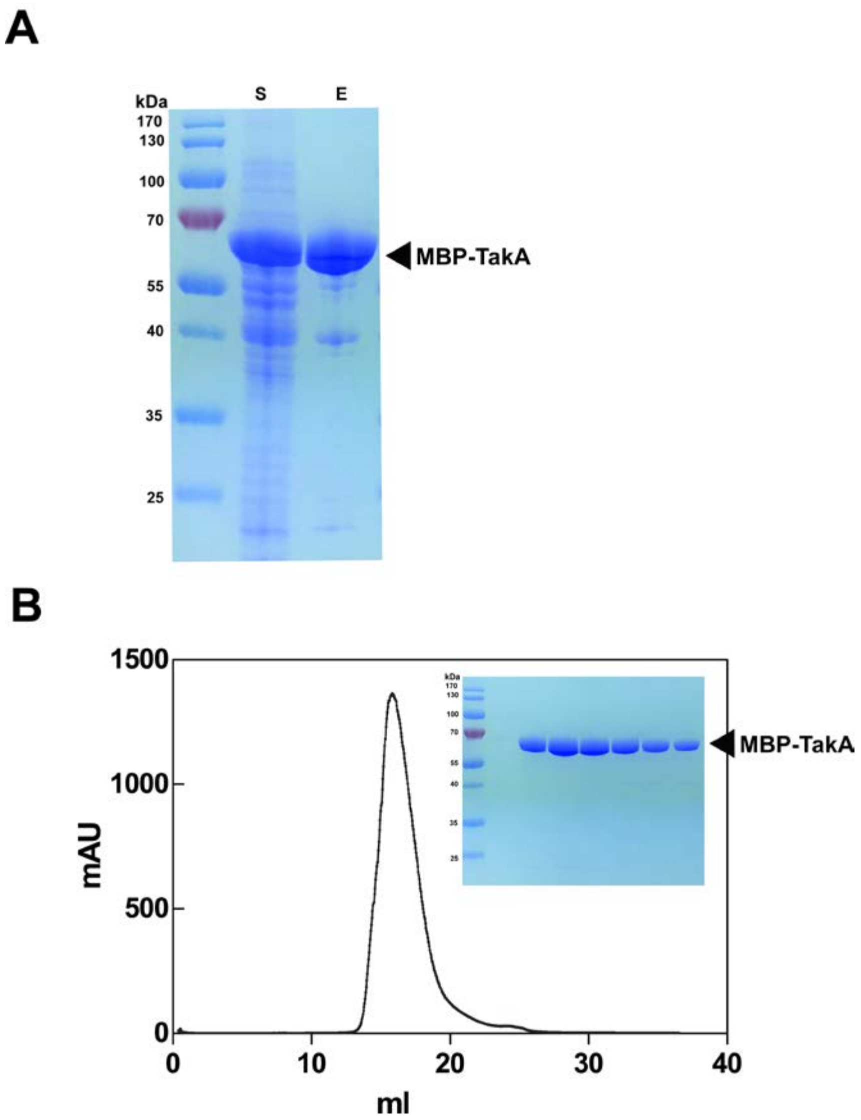

Supplementary Figure 11 Purification of MBP-TakA.

A. SDS-PAGE analysis of MBP-TakA purification using amylose resin (NEW ENGLAND BioLabs). Left lane, molecular weight standards, the molecular mass of the standard proteins are indicated. Middle lane, S stands for supernatant. Cells lysate was clarified by centrifugation and the supernatant was loaded to SDS-PAGE. Right lane, E stands for elution. MBP-TakA was eluted from amylose resin using an elution buffer containing 50mM Tris, pH=8.0, 150mM NaCl and 10mM maltose.

B. Size-exclusion chromatography was used as the final step of the purification. Average fraction absorbance (mAu) is plot as the function of elution volume (ml). Right insert: SDS-PAGE analysis of the fractions corresponding to the elution peak.

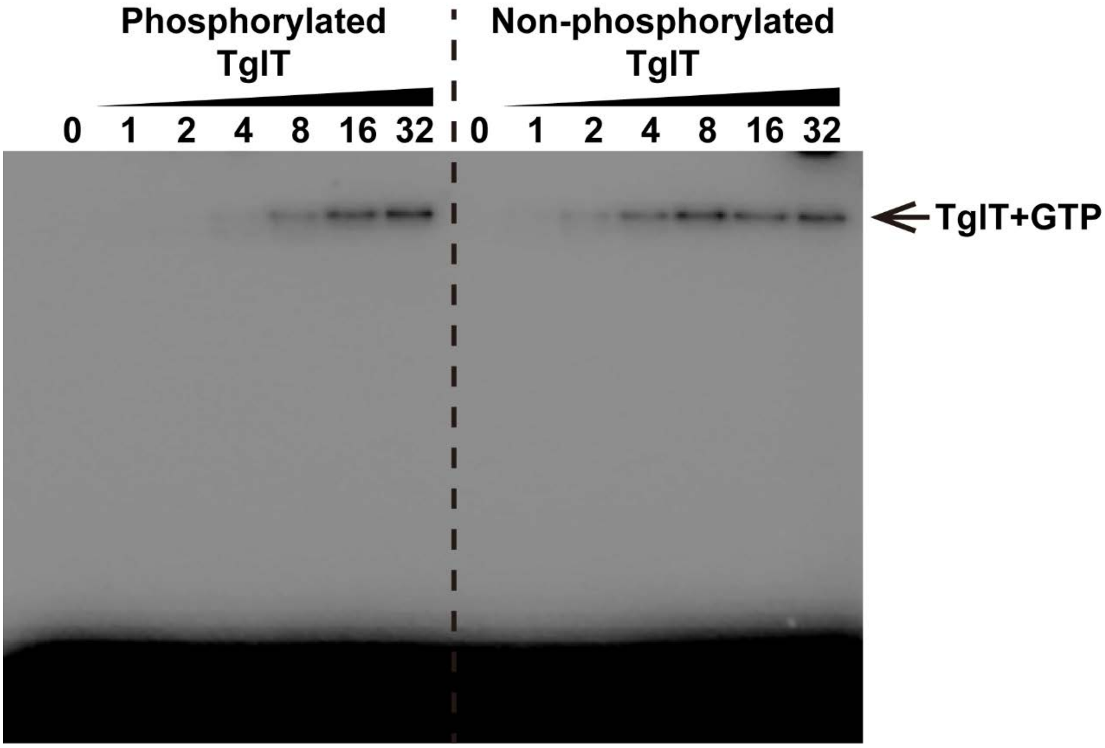

Supplementary Figure 12 Phosphorylation at residue S78 hindered GTP binding. GTP binding assay showed non-phosphorylated TglT exhibited higher binding affinity comparing to phosphorylated TglT. Non-phosphorylated TglT and phosphorylated TglT proteins were verified by LC-MS/MS (Supplementary Fig.5 & Fig.16) prior to GTP binding assay. [α-^{32}P] GTP (3.3nM) was inoculated with increasing amount of phosphorylated TglT and non-phosphorylated TglT. Protein concentration series were 1μM, 2μM, 4μM, 8μM, 16μM and 32μM. The mixtures were analyzed by Native-PAGE and visualized by autoradiography.

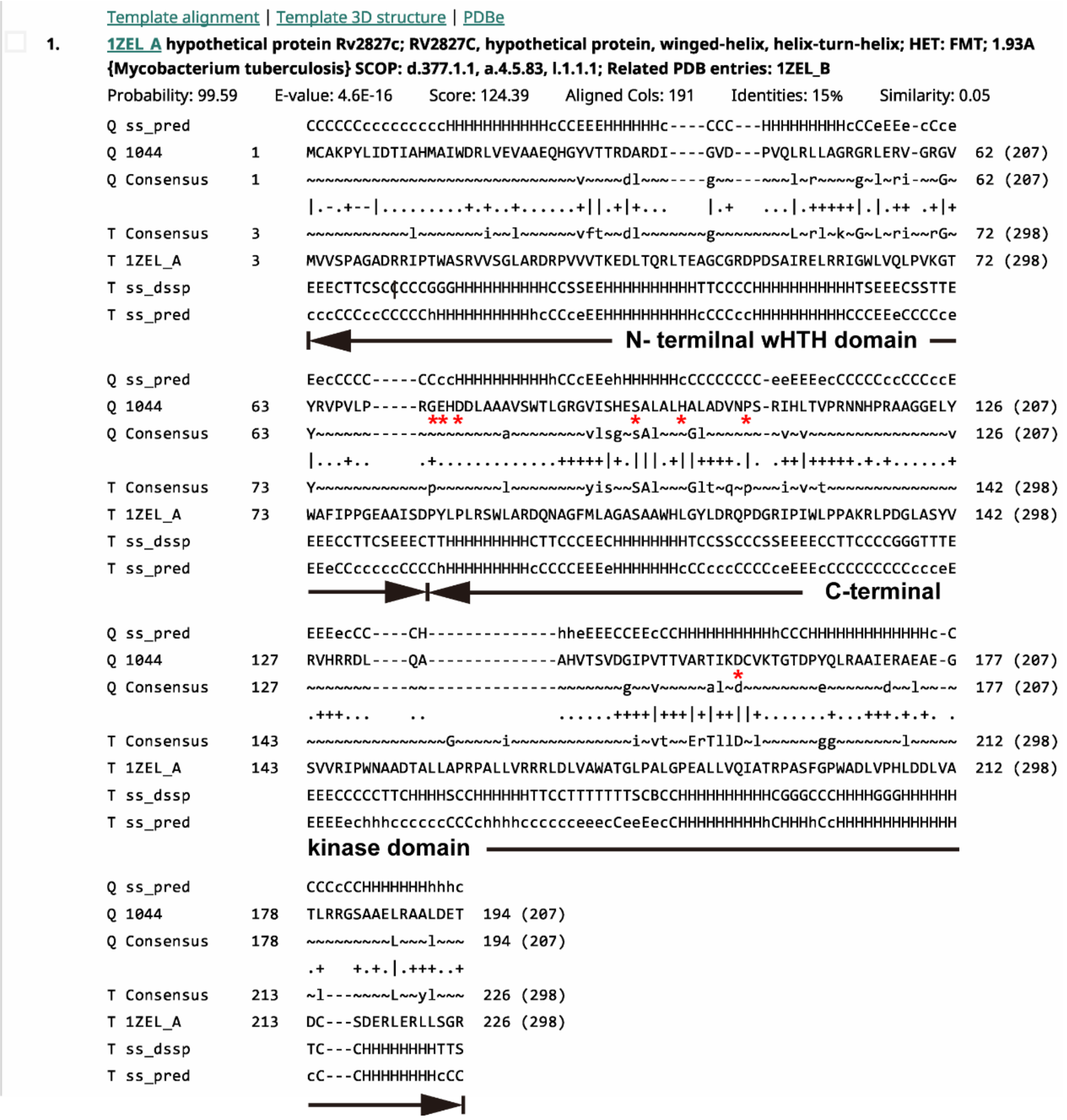

Supplementary Figure 13 Homology detection and structure prediction of TakA (Rv1044) using HHpred server.

Amino acids sequence of TakA (Rv1044) was analyzed by HHpred server http://protevo.eb.tuebingen.mpg.de/hhpred. The best hit was a hypothetic protein Rv2827c from MTB, the antitoxin in the putative Rv2826c-Rv2827c TA pair. The structure of Rv2827c is available, PDB ID: 1ZEL. The HHpred analysis gave a Probability: 99.59 for this hit, suggesting Rv1044 and Rv2827c share significant structure homology. However, the sequence identity between the two proteins is remote, 15%. HHpred predicted the secondary structures of Rv1044 and aligned the structures. N-terminal wHTH domain and C-terminal kinase domain are indicated. We selected a set of residues (marked with red stars) according to the alignment. G71, E72 and D74 are not conserved and located between the N-terminal HTH domain; S93, H98 and P105 and D155 are conserved and located within the C-terminal kinase domain. Mutagenesis study was performed on these residues, see Figure 5.

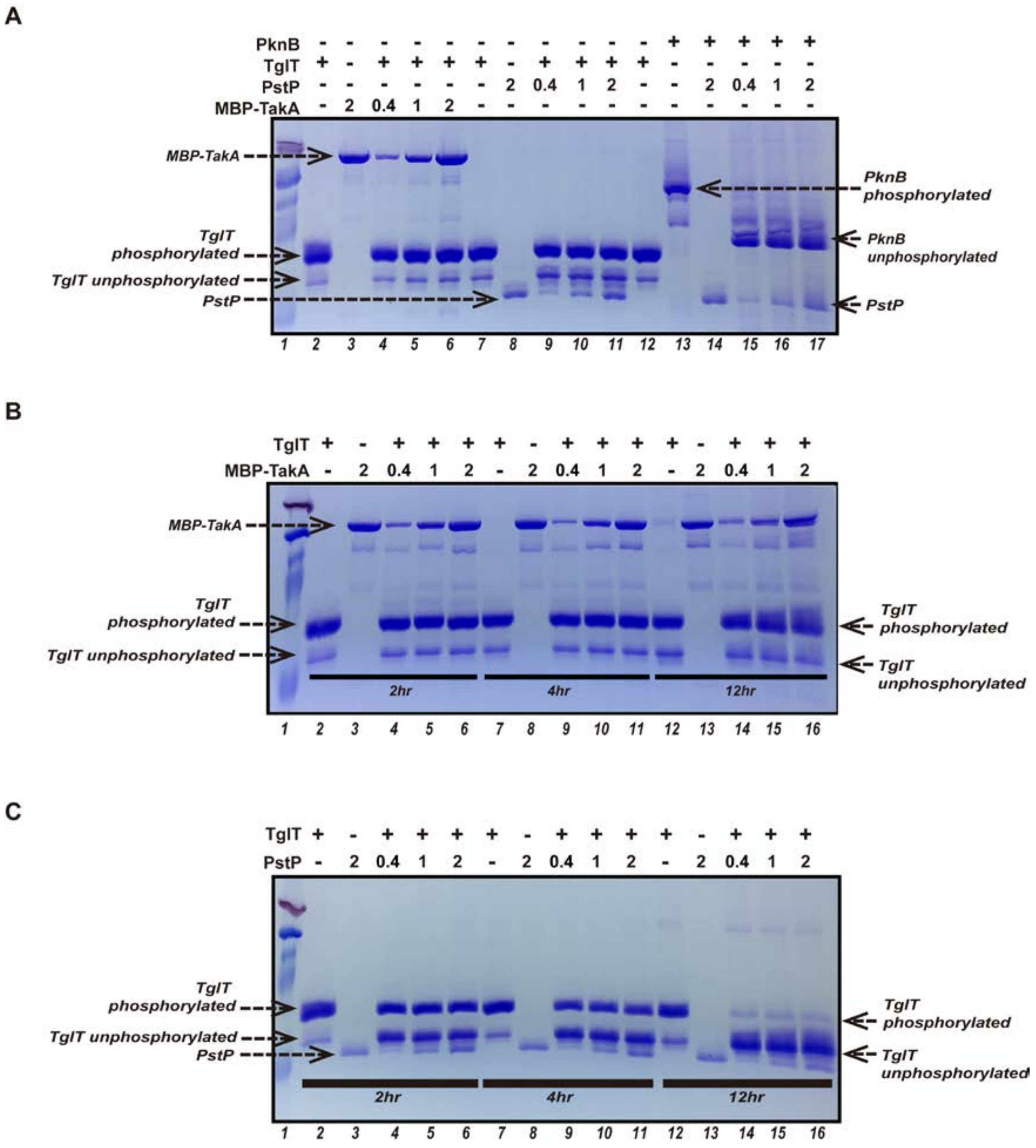

Supplementary Figure 14 Protein phosphatase assays suggest how TglT is dephosphorylated.

To investigate the mechanism of TglT dephosphorylation, we conducted protein phosphatase assay as previously described[1]. We incubated the phosphorylated TglT alone, with TakA kinase and with Mtb Ser-Thr phosphatase PstP (Rv0018c), respectively. The reactions were then analyzed by using Phos-tag SDS-PAGE.

A. The phosphorylated TglT (lane 2), the full-length MBP-TakA (lane 3), the catalytic domain of $PstP_{1-240aa}$ (lane 8) and the autophosphorylated catalytic domain of $PknB_{1-279aa}$ (lane 13) were expressed and purified. Increasing amount of MBP-TakA (0.4-2μM) were incubated with TglT (5μM), lanes 4-6; increasing amount of $PstP_{1-240aa}$ (0.4-2μM) were incubated with TglT(5μM), lanes 9-11. TglT was incubated alone for 12 hours, lane 12. $PstP_{1-240aa}$ (0.4-2μM) catalyzed dephosphorylation of $PknB_{1-279aa}$ (5μM) served as the positive control, lanes 14-17

B. Phosphatase assays with extended incubation time. The phosphorylated TglT (5μM)

was incubated with increasing amount of MBP-TakA (0.4-2μM). The incubation time of the reactions were 2 hours (lanes 2-6), 4 hours (lanes 7-11) and 12 hours (lanes 12-16) as indicated.

C. Phosphatase assays with extended incubation time. The phosphorylated TglT (5μM) was incubated with increasing amount of $PstP_{1-240aa}$ (0.4-2μM). The incubation time of the reactions were 2 hours (lanes 2-6), 4 hours (lanes 7-11) and 12 hours (lanes 12-16) as indicated.

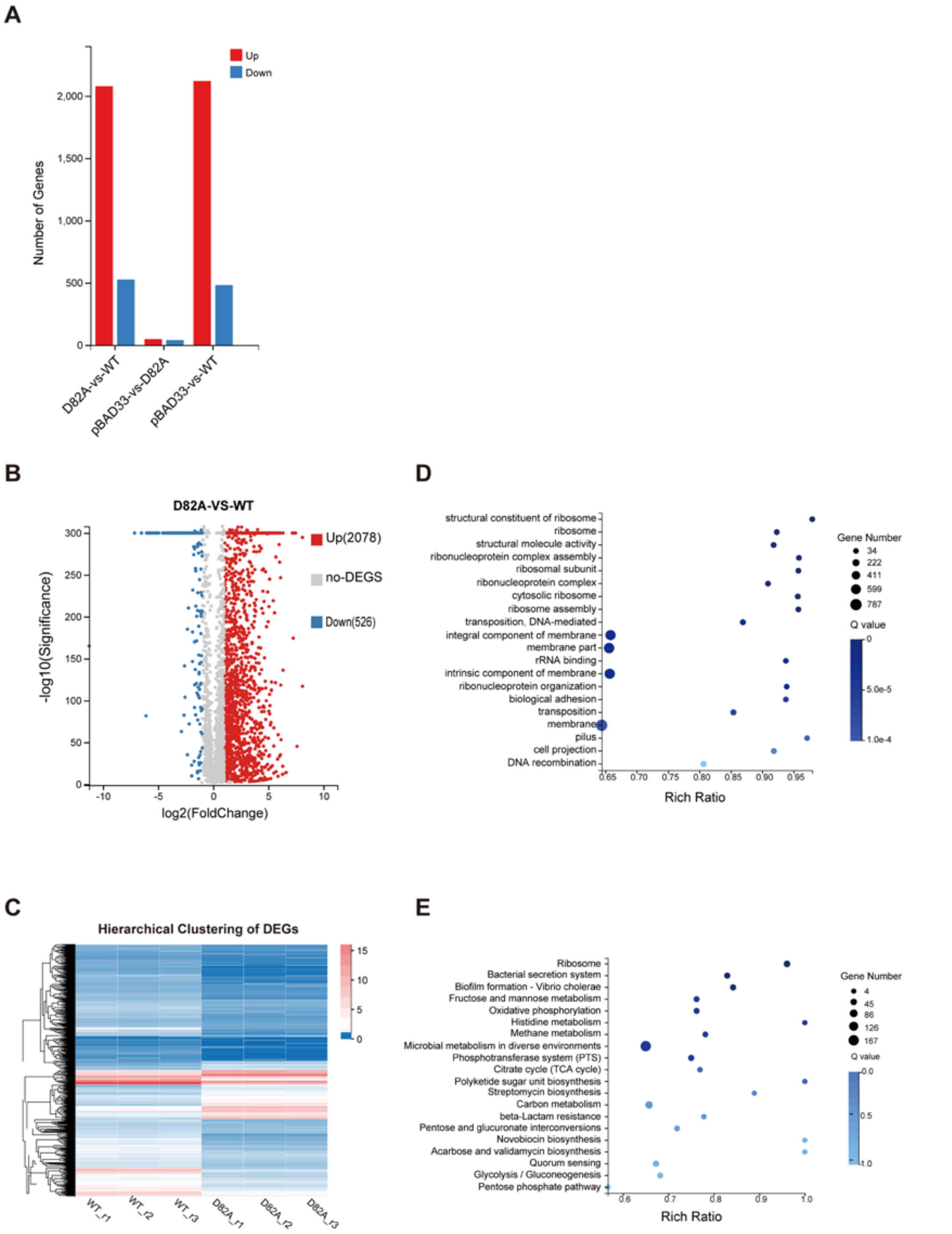

Supplementary Figure 15 RNA-seq analysis of differentially expressed genes (DEGs) of bacteria expressing wildtype TglT and mutant D82A.

A. DEGs analyses of bacteria expressing wildtype TglT vs D82A mutant, empty vector pBAD33 vs D82A mutant and pBAD33 vs wildtype TglT. The x axis represents pairwise between each group. The y axis means DEG numbers. Red color represents up-regulated DEGs, and blue color represents down-regulated DEGs. Compared with the wildtype TglT, the transcriptional profiles of the blank

control (emptor vector) and D82A mutant show only minor variations that were not statistically significant.

B. Scatter plot of DEGs between bacteria expressing wildtype TglT and mutant D82A. The x axis represents log2 transformed fold change, whereas the y axis represents -log10 transformed significance. Red points are up-regulated DEGs; blue points are down-regulated DEGs; and gray points are non-DEGs.

C. Hierarchical clustering of DEGs. Gradient color barcode at the right top indicates log2(FC) value. FC is Fold-Change of expression in TglT and D82A, from high (red) to low (blue). The x axis represents samples, whereas the y axis represents DEGs.

D. GO functional enrichment of DEGs. 20 most enriched terms are displayed. The x axis represents the Rich Ratio, whereas the y axis represents GO term. Lower the Q value, more significant enrichment. Point size indicates DEGs number. Rich Ratio is the quotient of the number of DEGs over total gene number, larger the value, the more significant.

E. KEGG-database-based pathway enrichment analysis. The top 20 enriched pathways are plotted. X axis represents Rich Ration. The y axis represents pathways, whereas the x axis represents Rich Ratio (the quotient of DEGs numbers over all gene numbers annotated in the pathway). The color indicates the Q value, lower the Q value, more significant. Point size indicates DEG number (bigger dot indicates larger number).

Mascot Search Results

Peptide View

MS/MS Fragmentation of **TSKDFDTVAR**
Found in **tr|A0A045JBD4|A0A045JBD4_MYCTX**, Nucleotidyl transferase of uncharacterised function (DUF1814)
OS=Mycobacterium tuberculosis GN=B7R63_05680 PE=4 SV=1

Match to Query 2782: 1138.562968 from(570.288760,2+) intensity(20893570.0000)
Title: File8725 Spectrum1329 scans: 3198
Data file F:\DATA\2019\1yue\20190114\17872\BPI_17872_1045MBP.mgf

Click mouse within plot area to zoom in by factor of two about that point
Or, Plot from 50 to 1050 Da Full range
Label all possible matches ○ Label matches used for scoring ◉

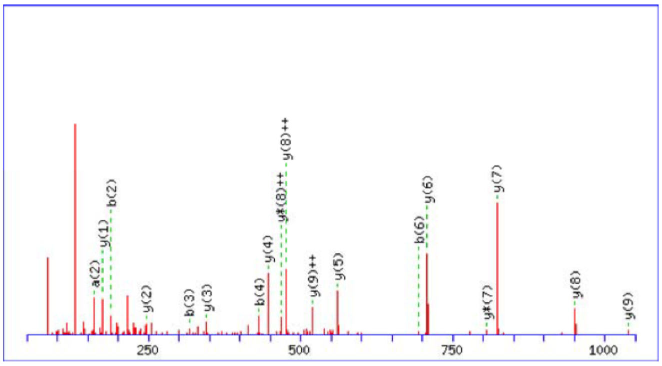

Monoisotopic mass of neutral peptide Mr(calc): 1138.5619
Fixed modifications: Carbamidomethyl (C) (apply to specified residues or termini only)
Ions Score: 49 **Expect:** 0.00012
Matches : 18/100 fragment ions using 49 most intense peaks (help)

#	a	a^{++}	a*	a^{*++}	b	b^{++}	b*	b^{*++}	Seq.	y	y^{++}	y*	y^{*++}	#
1	74.0600	37.5337			102.0550	51.5311			T					10
2	161.0921	81.0497			189.0870	95.0471			S	1038.5215	519.7644	1021.4949	511.2511	9
3	289.1870	145.0972	272.1605	136.5839	317.1819	159.0946	300.1554	150.5813	K	951.4894	476.2483	934.4629	467.7351	8
4	404.2140	202.6106	387.1874	194.0974	432.2089	216.6081	415.1823	208.0948	D	823.3945	412.2009	806.3679	403.6876	7
5	551.2824	276.1448	534.2558	267.6316	579.2773	290.1423	562.2508	281.6290	F	708.3675	354.6874	691.3410	346.1741	6
6	666.3093	333.6583	649.2828	325.1450	694.3042	347.6558	677.2777	339.1425	D	561.2991	281.1532	544.2726	272.6399	5
7	767.3570	384.1821	750.3305	375.6689	795.3519	398.1796	778.3254	389.6663	T	446.2722	223.6397	429.2456	215.1264	4
8	866.4254	433.7164	849.3989	425.2031	894.4203	447.7138	877.3938	439.2005	V	345.2245	173.1159	328.1979	164.6026	3
9	937.4625	469.2349	920.4360	460.7216	965.4575	483.2324	948.4309	474.7191	A	246.1561	123.5817	229.1295	115.0684	2
10									R	175.1190	88.0631	158.0924	79.5498	1

Supplementary Figure 16. LC-MS/MS analysis of the non-phosphorylated TglT, co-expressed with MBP.
A fragment $_{77}$TSKDFDTVARR$_{87}$ belonging to TglT was identified. In which, the molecular mass of residue S78 (underlined) calculates: 1038.5215-951.4894 + 18(1 water) = 105.0321Da (orange box indicated that data used for calculation). The theoretical molecular mass of serine is 105.09Da. Thus, S78 was non-phosphorylated.

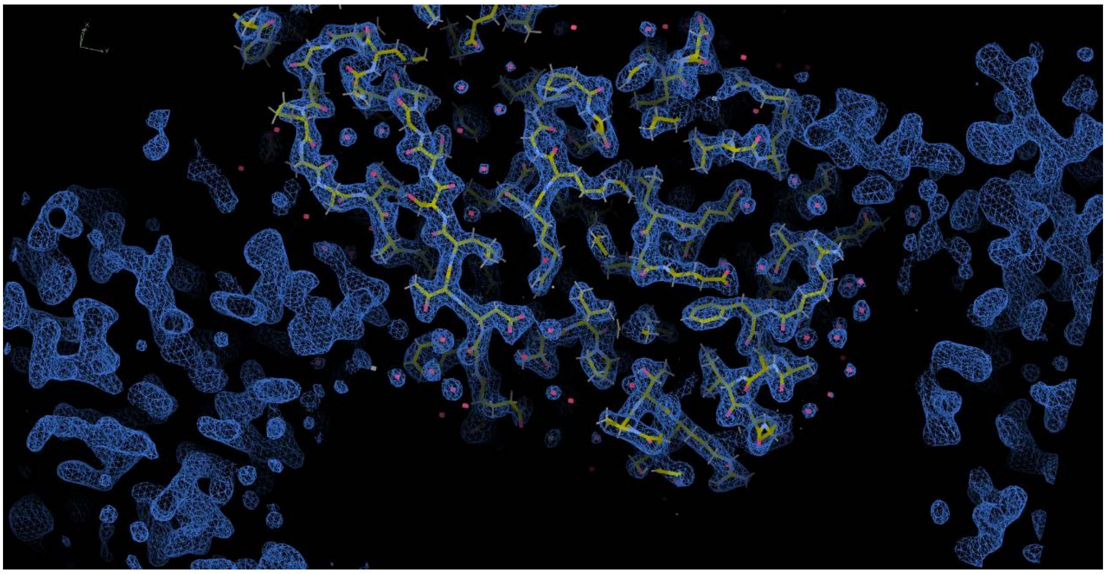

Supplementary Figure 17 Final 2Fo-Fc map of the crystal structure of wildtype TglT.

An overview of final 2Fo-Fc map (contour 1.5s, blue mesh) of the crystal structure of wildtype TglT is superimposed with final stick model of wildtype TglT, colored by atom type. There is one molecular in asymmetric unit; the models of the symmetry related molecules are not shown.

Supplementary Tables

Supplementary Table 1 Dali search of TglT structure agaist PDB90 database[a] (top 30 Z-score hits).

No.	Chain[b]	Z[c]	rmsd	lali[d]	nres[e]	%id[f]	Description
**1:**	4ok0-A	14.4	4.1	199	225	16	MOLECULE: PUTATIVE;
**2:**	4ok0-B	14.4	4.2	204	227	16	MOLECULE: PUTATIVE;
**3:**	4o8s-A	14.1	4.1	204	232	15	MOLECULE: PUTATIVE;
**4:**	5cfu-A	11.3	3.6	153	174	10	MOLECULE: AMINOGLYCOSIDE NUCLEOTIDYLTRANSFERASE (2")-IA;
**5:**	5cft-A	11.2	3.4	151	175	10	MOLECULE: AMINOGLYCOSIDE NUCLEOTIDYLTRANSFERASE (2")-IA;
**6:**	5cfs-A	11.1	3.5	152	176	10	MOLECULE: AAD(2"),GENTAMICIN 2"-NUCLEOTIDYLTRANSFERASE,GE
**7:**	4xje-A	11.1	3.8	153	175	11	MOLECULE: AADB;
**8:**	4wql-A	11.1	3.7	153	175	10	MOLECULE: 2"-AMINOGLYCOSIDE NUCLEOTIDYLTRANSFERASE;
**9:**	4wqk-A	10.6	3.7	156	175	12	MOLECULE: 2"-AMINOGLYCOSIDE NUCLEOTIDYLTRANSFERASE;
**10:**	4e8j-B	9.6	3.0	133	158	11	MOLECULE: LINCOSAMIDE RESISTANCE PROTEIN;
**11:**	2ewr-A	9.5	3.3	139	156	9	MOLECULE: HYPOTHETICAL PROTEIN TM1012;
**12:**	2fcl-A	9.4	3.3	140	157	9	MOLECULE: HYPOTHETICAL PROTEIN TM1012;
**13:**	4e8i-B	9.2	3.0	134	158	11	MOLECULE: LINCOSAMIDE RESISTANCE PROTEIN;
**14:**	4fo1-B	9.2	3.1	134	158	11	MOLECULE:

							LINCOSAMIDE RESISTANCE PROTEIN;
**15:**	4wh5-B	9.1	3.0	133	158	11	MOLECULE: LINCOSAMIDE RESISTANCE PROTEIN;
**16:**	4fo1-A	9.1	3.1	134	158	11	MOLECULE: LINCOSAMIDE RESISTANCE PROTEIN;
**17:**	4wh5-A	9.1	2.9	132	158	11	MOLECULE: LINCOSAMIDE RESISTANCE PROTEIN;
**18:**	4e8j-A	9.1	2.9	132	158	11	MOLECULE: LINCOSAMIDE RESISTANCE PROTEIN;
**19:**	4e8i-A	9.1	3.1	135	158	11	MOLECULE: LINCOSAMIDE RESISTANCE PROTEIN;
**20:**	4wse-B	8.9	4.8	176	495	9	MOLECULE: PUTATIVE POLY(A) POLYMERASE CATALYTIC SUBUNIT;
**21:**	4hx0-A	8.5	2.9	132	161	8	MOLECULE: PUTATIVE NUCLEOTIDYLTRANSFERA SE TM1012;
**22:**	4tur-A	7.4	5.2	116	327	10	MOLECULE:DNA POLYMERASE BETA;
**23:**	4p37-A	7.3	4.8	165	498	10	MOLECULE:PUTATIVE POLY(A) POLYMERASE CATALYTIC SUBUNIT;
**24:**	5j29-A	7.3	5.1	117	326	10	MOLECULE:DNA POLYMERASE BETA;
**25:**	4p2h-A	7.3	4.1	112	324	10	MOLECULE:DNA POLYMERASE BETA;
**26:**	4yn4-A	7.3	4.9	117	327	10	MOLECULE: DNA POLYMERASE BETA;
**27:**	4pgq-A	7.3	4.2	114	324	10	MOLECULE: DNA POLYMERASE BETA;
**28:**	5j0v-A	7.3	4.1	113	326	10	MOLECULE: DNA POLYMERASE BETA;

**29:**	5j0u-A	7.3	4.1	113	326	10	MOLECULE: DNA POLYMERASE BETA;
**30:**	4mf8-A	7.3	4.7	114	318	11	MOLECULE: DNA POLYMERASE BETA;

[a] PDB90 is a non-redundant subset of Protein Data Bank structures with less than 90% sequence identity to each other.

[b] PDB code and chain ID

[c] Dali Z-score

[d] The number of aligned C-alpha atoms

[e] The number of C-alpha atoms in the database structure

[f] Amino acids sequence identity

Supplementary Table 2 Plasmids used in this study.

Vector name	*Descriptions*	*Name of the protein expressed*	*Expression inducer*	*Antibiotic resistance*	*Oligo sequences*
pET28a	Expression plasmid	None	IPTG	kanamycin	
pET28a-n-6His-Rv1044	Expressing antitoxin TakA with N-terminal 6×Histag	His-TakA	IPTG	kanamycin	Forward:<br>GTCGCATATGTGT<br>GCAAAACCGTAT<br>CT<br>Reverse:<br>TCTGCTCGAGTC<br>ACGCCGATGCTC<br>GCTTC
pETDuet-1	Expression plasmid	None	IPTG	ampicillin	
pETDuet-1-Rv1044 no tag	Expressing antitoxin TakA for neutralizing toxicity	TakA	IPTG	ampicillin	Forward:<br>GTCGCCATGGGC<br>TTGTGTGCAAAA<br>CCGTATC<br>Reverse:TCTGAAG<br>CTTTCACGCCGA<br>TGCTCGCTTCGG<br>CCG
pET28a-n-6His-Rv1045	Expressing TglT protein with N-terminal-6×His	His-TglT	IPTG	kanamycin	Forward:<br>GTCGCATATGAC<br>CAAGCCCTATTC<br>Reverse:<br>TCTGCTCGAGTC

					ATCTTTTCGTCGC CCGAT
pET28a-n-6His-Rv1045-G62A	Expressing TglT G62A mutant with N-terminal-6×His	His-TglT-G62A	IPTG	kanamycin	Forward: CTGTTGGTCAAAGCCGGATCGTCGCTG Reverse: CAGCGACGATCCGGCTTTGACCAACAG
pET28a-n-6His-Rv1045-S78A	Expressing TglT S78A mutant with N-terminal-6×His	His-TglT-S78A	IPTG	kanamycin	Forward: GATTCGCGGACCGCCAAAGACTTCG Reverse: CGAAGTCTTTGGCGGTCCGCGAATC
pET28a-n-6His-Rv1045-S78D	Expressing TglT S78D mutant with N-terminal-6×His	His-TglT-S78D	IPTG	kanamycin	Forward: CCGATTCGCGGACCGACAAAGACTTCGAC Reverse: GTCGAAGTCTTTGTCGGTCCGCGAATCGG
pET28a-n-6His-Rv1045-D82A	Expressing TglT D82A mutant with N-terminal-6×His	His-TglT-D82A	IPTG	kanamycin	Forward: CTCCAAAGACTTCGCAACGGTCGCACGTC Reverse: GACGTGCGACCGTTGCGAAGTCTTTGGAG
pET28a-n-6His-Rv1045-E146Q	Expressing TglT E146Q mutant with N-terminal-6×His	His-TglT-E146Q	IPTG	kanamycin	Forward: CAACTGTTCCGATCCAGGTCTCCTCCGTC Reverse: GACGGAGGAGACCTGGATCGGAACAGTTG

pET28a-n-6His-Rv1045-K189N	Expressing TglT K189N mutant with N-terminal-6×His	His-TglT-K189N	IPTG	kanamycin	Forward: CAAATCGCGCAG AACCTGCACGCA GTAAC Reverse: GTTACTGCGTGC AGGTTCTGCGCG ATTTG
pET28a-n-6His-Rv1045-D208A	Expressing TglT D208A mutant with N-terminal-6×His	His-TglT-D208A	IPTG	kanamycin	Forward: ACCGCGCTCACG CACTGGTGGACT TGCAGCTTCTT Reverse: AAGAAGCTGCA AGTCCACCAGTG CGTGAGCGCGGT
pBAD33	Arabinose-inducible expression plasmid	None	L-arabinose	chloramphenicol	
pBAD33-c-6His-Rv1045	Expressing TglT toxin with C-terminal-6×His	TglT-His	L-arabinose	chloramphenicol	Forward: GTCG GGTACCAAGAAG GAGATATACATAT G ATGACCAAGCCC TATTCGTC Reverse: TCTGAAGCTTTC A GTGGTGGTGGTG GTGGTGTCTTTT CGTCGCCCGATC
pBAD33-c-6His-Rv1045-G62A	Expressing TglT G62A mutant with C-terminal-6×His	TglT-G62A-His	L-arabinose	chloramphenicol	Forward: CTGTTGGTCAAA GCCGGATCGTCG CTG

					Reverse: CAGCGACGATCCGGCTTTGACCAACAG
pBAD33-c-6His-Rv1045-S78A	Expressing TglT S78A mutant with C-terminal-6×His	TglT-S78A-His	L-arabinose	chloramphenicol	Forward: GATTCGCGGACCGCCAAAGACTTCG Reverse: CGAAGTCTTTGGCGGTCCGCGAATC
pBAD33-c-6His-Rv1045-S78D	Expressing TglT S78D mutant with C-terminal-6×His	TglT-S78D-His	L-arabinose	chloramphenicol	Forward: CCGATTCGCGGACCGACAAAGACTTCGAC Reverse: GTCGAAGTCTTTGTCGGTCCGCGAATCGG
pBAD33-c-6His-Rv1045-D82A	Expressing TglT D82A mutant with C-terminal-6×His	TglT-D82A-His	L-arabinose	chloramphenicol	Forward: CTCCAAAGACTTCGCAACGGTCGCACGTC Reverse: GACGTGCGACCGTTGCGAAGTCTTTGGAG
pBAD33-c-6His-Rv1045-E146Q	Expressing TglT E146Q mutant with C-terminal-6×His	TglT-E146Q-His	L-arabinose	chloramphenicol	Forward: CAACTGTTCCGATCCAGGTCTCCTCCGTC Reverse: GACGGAGGAGACCTGGATCGGAACAGTTG
pBAD33-c-6His-Rv1045-K189N	Expressing TglT K189N mutant with C-terminal-6×His	TglT-K189N-His	L-arabinose	chloramphenicol	Forward: CAAATCGCGCAGAACCTGCACGCAGTAAC Reverse: GTTACTGCGTGC

					AGGTTCTGCGCG ATTTG
pBAD33-c-6His-Rv1045-D208A	Expressing TglT D208A mutant with C-terminal-6×His	TglT-D208A-His	L-arabinose	chloramphenicol	Forward: ACCGCGCTCACG CACTGGTGGACT TGCAGCTTCTT Reverse: AAGAAGCTGCA AGTCCACCAGTG CGTGAGCGCGGT
pET28a-c-FLAG-Rv1045	Expressing TglT with C-terminal FLAG tag	TglT-FLAG	IPTG	kanamycin	Forward: GTCGCCATGGGA GTGACCAAGCCC TATTC Reverse: TCTGCTCGAGTC ACTTGTCGTCAT CGTCTTTGTAGT CTCTTTTCGTCG CCCG
pBAD/Myc-His A	Expression plasmid	None	L-arabinose	ampicillin	
pBAD/Myc-His-Rv1044	Expressing C-terminal Myc and 6xHis tagged TakA	TakA-Myc	L-arabinose	ampicillin	Forward: GTCGCCATGGGA TTGTGTGCAAAA CCGTATCT Reverse: TCTGAAGCTTCG CCGATGCTCGCT TC

pMAL-c5X	Expression vector for producing MBP fusion protein	MBP	IPTG	ampicillin	
pMAL-Rv1044	Expressing N-terminal MBP-tagged TakA	MBP-TakA	IPTG	ampicillin	Forward: GTCGCATATGTTG TGTGCAAAACCG Reverse: TCTGGAATTCTC ACGCCGATGCTC GCTTCGG
pMAL-Rv1044-G71A	Expressing N-terminal MBP-tagged TakA with G71A mutant	MBP-TakA G71A	IPTG	ampicillin	Forward: GCTGCCGCGTGC AGAGCACGACG AT Reverse: ATCGTCGTGCTC TGCACGCGGCAG C
pMAL-Rv1044-E72A	Expressing N-terminal MBP-tagged TakA with E72A mutant	MBP-TakA E72A	IPTG	ampicillin	Forward: CTGCCGCGTGGT GCGCACGACGAT C Reverse: GATCGTCGTGCG CACCACGCGGCA G
pMAL-Rv1044-D74A	Expressing N-terminal MBP-tagged TakA with D74A mutant	MBP-TakA D74A	IPTG	ampicillin	Forward: CGTGGTGAGCAC GCCGATCTCGCA G Reverse: CTGCGAGATCGG CGTGCTCACCAC G
pMAL-Rv1044-S93A	Expressing N-terminal MBP-tagged TakA with	MBP-TakA S93A	IPTG	ampicillin	Forward: GTTATCTCGCATG AGGCAGCCTTGG CGCTTCATG

	S93A mutant				Reverse: CATGAAGCGCCAAGGCTGCCTCATGCGAGATAAC
pMAL-Rv1044-H98A	Expressing N-terminal MBP-tagged TakA with H98A mutant	MBP-TakA H98A	IPTG	ampicillin	Forward: CCTTGGCGCTTGCAGCCCTCGCTGA Reverse: TCAGCGAGGGCTGCAAGCGCCAAGG
pMAL-Rv1044-D105A	Expressing N-terminal MBP-tagged TakA withD105A mutant	MBP-TakA D105A	IPTG	ampicillin	Forward: CTGACGTGAACGCGTCGCGCATCCATCTCA Reverse: TGAGATGGATGCGCGACGCGTTCACGTCAG
pMAL-Rv1044D155A	Expressing N-terminal MBP-tagged TakA with D155A mutant	MBP-TakA D155A	IPTG	ampicillin	Forward: CACCATCAAAGCCTGCGTGAAGACGG Reverse: CCGTCTTCACGCAGGCTTTGATGGTG
pYC601	Shuttle vector containing anhydrotetracycline (ATC)-inducible promoter	None	Anhydrotetracycline (ATC)	hygromycin	
pYC601-Rv1044/1045	Expressing TakA and TglT	TakA-TglT	Anhydrotetracycline (ATC)	hygromycin	Forward: TCCGCATGCGGAGGAATCAGTTGTGTGCAAAACCGTATCT Reverse: GTCCCCAATTAATTAGCTAATCATCT

					TTTCGTCGCCCG AT
pYC601-Rv1044	Expressing TakA alone	TakA	Anhydrotetra cycline (ATC)	hygromycin	Forward: TCCGCATGCGGA GGAATCAGTTGT GTGCAAAACCGT ATCT Reverse: GTCCCCAATTAAT TAGCTAATCACG CCGATGCTCGCT TCG

Supplementary Table 3 The sequence upstream of predicted *Rv1044* gene used in this study

The promoter sequence of predicted *Rv1044*	GGGTCCCAACCGAGCGGCAGCAGAAGTCC CGCGCGTTCCATCGAGCTGAGGATGCGGTG GAGGGTCACCGCGTCGCCCGC GGCGGGCAGGCCGAGGGTGCTCAGGTATC GGGAGAAATCTGCGACCGACCACGGTTCG AAGGGCACCGTCGTCGGCAGAC CGATATCCGAGTCAACCGGTGGTGGTTCGG GTTTGCCGATCGCCGCCGCAACCACCGGGT TGTGGACCAGCCCGAAGAAT TGATGGGCGCACATCGCCACGTTCACACGC CACGCAGGAGTCCCGGGCTTCAGGTCGGC CGCCGTGAGCTGTCGCGGTCA GGTGCTTTCCGCGCCATCCGCCGTCACCTC TGCCATGGTCCATCTACGGTATCTGCGACA AGGGCAGCGTCGATGCCTCG ACATGCAGAGTCGGTGTTCGCTTCACGCGA ACTAGGCGCGCCTAGCCTGGACGAGTCCCC GGGCCGACATTCGCCCGAGG CCTTGGCCTCCATCACCTAATTGTGTGCAA AACCGTATCTAATTGATACGATTGCGCAC

References

1 Boitel, B. *et al.* PknB kinase activity is regulated by phosphorylation in two Thr residues and dephosphorylation by PstP, the cognate phospho-Ser/Thr phosphatase, in Mycobacterium tuberculosis. *Molecular microbiology* **49**, 1493-1508 (2003).
