# Supplementary material for: Characterization of a toxin-antitoxin system in Mycobacterium tuberculosis suggests neutralization by phosphorylation as the antitoxicity mechanism
Source: Commun Biol. 2020 May 7;3:216. doi: 10.1038/s42003-020-0941-1 (PMC7205606; doi:10.1038/s42003-020-0941-1)
Supplement: Supplementary file 4 — Description of Additional Supplementary Files [file 42003_2020_941_MOESM4_ESM.pdf]

Description of Additional Supplementary Files

File Name: **Supplementary Data 1**
Description: contains the original RNA-seq data underlying Supplementary Fig.15

File Name: **Supplementary Data 2**
Description: source data file contains the original data underlying Figs.1B-G,3E,4C,5A-C,6A,7B and Supplementary Fig.2,11,12,14

File Name: Supplementary Data 3
Description: Full results of the Dali search
