# Supplementary material for: Characterization of a toxin-antitoxin system in Mycobacterium tuberculosis suggests neutralization by phosphorylation as the antitoxicity mechanism
Source: Commun Biol. 2020 May 7;3:216. doi: 10.1038/s42003-020-0941-1 (PMC7205606; doi:10.1038/s42003-020-0941-1)
Supplement: Supplementary file 7 — Supplementary Data 3 [file 42003_2020_941_MOESM7_ESM.pdf]

Results

Parseable data
Matches to PDB90

- mol1A

Query: mol1A

Select neighbours (check boxes) for viewing as multiple structural alignment or 3D superimposition. The list of neighbours is sorted by Z-score. Similarities with a Z-score lower than 2 are spurious. Each neighbour has links to pairwise structural alignment with the query structure, to pre-computed structural neighbours in the Dali Database, and to the PDB format coordinate file where the neighbour is superimposed onto the query structure.

Structural Alignment | Expand gaps | 3D Superimposition (Jmol Applet) | Sequence Similarity Search | Reset Selection

Summary

No:	Chain	Z	rmsd	lali	nres	%id	PDB	Description
1:	4ok0-A	14.4	4.1	199	225	16	PDB	MOLECULE: PUTATIVE;
2:	4ok0-B	14.4	4.2	204	227	16	PDB	MOLECULE: PUTATIVE;
3:	4o8s-A	14.1	4.1	204	232	15	PDB	MOLECULE: PUTATIVE;
4:	5cfu-A	11.3	3.6	153	174	10	PDB	MOLECULE: AMINOGLYCOSIDE NUCLEOTIDYLTRANSFERASE (2")-IA;
5:	5cft-A	11.2	3.4	151	175	10	PDB	MOLECULE: AMINOGLYCOSIDE NUCLEOTIDYLTRANSFERASE (2")-IA;
6:	5cfs-A	11.1	3.5	152	176	10	PDB	MOLECULE: AAD(2''),GENTAMICIN 2''-NUCLEOTIDYLTRANSFERASE,GE
7:	4xje-A	11.1	3.8	153	175	11	PDB	MOLECULE: AADB;
8:	4wql-A	11.1	3.7	153	175	10	PDB	MOLECULE: 2''-AMINOGLYCOSIDE NUCLEOTIDYLTRANSFERASE;
9:	4wqk-A	10.6	3.7	156	175	12	PDB	MOLECULE: 2''-AMINOGLYCOSIDE NUCLEOTIDYLTRANSFERASE;
10:	4e8j-B	9.6	3.0	133	158	11	PDB	MOLECULE: LINCOSAMIDE RESISTANCE PROTEIN;
11:	2ewr-A	9.5	3.3	139	156	9	PDB	MOLECULE: HYPOTHETICAL PROTEIN TM1012;
12:	2fcl-A	9.4	3.3	140	157	9	PDB	MOLECULE: HYPOTHETICAL PROTEIN TM1012;
13:	4e8i-B	9.2	3.0	134	158	11	PDB	MOLECULE: LINCOSAMIDE RESISTANCE PROTEIN;
14:	4fo1-B	9.2	3.1	134	158	11	PDB	MOLECULE: LINCOSAMIDE RESISTANCE PROTEIN;
15:	4wh5-B	9.1	3.0	133	158	11	PDB	MOLECULE: LINCOSAMIDE RESISTANCE PROTEIN;
16:	4fo1-A	9.1	3.1	134	158	11	PDB	MOLECULE: LINCOSAMIDE RESISTANCE PROTEIN;
17:	4wh5-A	9.1	2.9	132	158	11	PDB	MOLECULE: LINCOSAMIDE RESISTANCE PROTEIN;
18:	4e8j-A	9.1	2.9	132	158	11	PDB	MOLECULE: LINCOSAMIDE RESISTANCE PROTEIN;
19:	4e8i-A	9.1	3.1	135	158	11	PDB	MOLECULE: LINCOSAMIDE RESISTANCE PROTEIN;
20:	4wse-B	8.9	4.8	176	495	9	PDB	MOLECULE: PUTATIVE POLY(A) POLYMERASE CATALYTIC SUBUNIT;
21:	4hx0-A	8.5	2.9	132	161	8	PDB	MOLECULE: PUTATIVE NUCLEOTIDYLTRANSFERASE TM1012;
22:	4tur-A	7.4	5.2	116	327	10	PDB	MOLECULE: DNA POLYMERASE BETA;
23:	4p37-A	7.3	4.8	165	498	10	PDB	MOLECULE: PUTATIVE POLY(A) POLYMERASE CATALYTIC SUBUNIT;
24:	5j29-A	7.3	5.1	117	326	10	PDB	MOLECULE: DNA POLYMERASE BETA;
25:	4p2h-A	7.3	4.1	112	324	10	PDB	MOLECULE: DNA POLYMERASE BETA;
26:	4yn4-A	7.3	4.9	117	327	10	PDB	MOLECULE: DNA POLYMERASE BETA;
27:	4pgq-A	7.3	4.2	114	324	10	PDB	MOLECULE: DNA POLYMERASE BETA;
28:	5j0v-A	7.3	4.1	113	326	10	PDB	MOLECULE: DNA POLYMERASE BETA;
29:	5j0u-A	7.3	4.1	113	326	10	PDB	MOLECULE: DNA POLYMERASE BETA;
30:	4mf8-A	7.3	4.7	114	318	11	PDB	MOLECULE: DNA POLYMERASE BETA;
31:	4f5q-A	7.3	5.4	121	320	10	PDB	MOLECULE: DNA POLYMERASE BETA;
32:	4f5n-A	7.3	4.7	115	323	10	PDB	MOLECULE: DNA POLYMERASE BETA;
33:	2bpg-B	7.3	5.0	122	324	10	PDB	MOLECULE: DNA POLYMERASE BETA;
34:	1zjm-A	7.3	4.7	115	323	10	PDB	MOLECULE: DNA POLYMERASE BETA;
35:	1zqz-A	7.3	4.7	114	242	10	PDB	MOLECULE: DNA POLYMERASE BETA;
36:	4r66-A	7.3	4.9	116	326	10	PDB	MOLECULE: DNA POLYMERASE BETA;
37:	4ny8-A	7.3	5.0	121	323	8	PDB	MOLECULE: DNA POLYMERASE BETA;
38:	4ppx-A	7.3	4.7	115	314	10	PDB	MOLECULE: DNA POLYMERASE BETA;
39:	4tup-A	7.2	4.2	112	326	10	PDB	MOLECULE: DNA POLYMERASE BETA;

40:	5tzv-A	7.2	4.9	116	328	10	PDB	MOLECULE: DNA POLYMERASE BETA;
41:	5j0t-A	7.2	4.9	116	327	10	PDB	MOLECULE: DNA POLYMERASE BETA;
42:	4pgy-A	7.2	4.8	117	327	10	PDB	MOLECULE: DNA POLYMERASE BETA;
43:	5dbb-A	7.2	4.9	116	327	10	PDB	MOLECULE: DNA POLYMERASE BETA;
44:	5j0o-A	7.2	4.9	116	327	10	PDB	MOLECULE: DNA POLYMERASE BETA;
45:	3uxn-A	7.2	4.6	115	325	9	PDB	MOLECULE: DNA POLYMERASE BETA;
46:	3uxo-A	7.2	5.2	119	325	9	PDB	MOLECULE: DNA POLYMERASE BETA;
47:	1tv9-A	7.2	5.3	121	331	10	PDB	MOLECULE: DNA POLYMERASE BETA;
48:	3isc-A	7.2	4.6	114	327	10	PDB	MOLECULE: DNA POLYMERASE BETA;
49:	3isb-A	7.2	4.6	116	327	9	PDB	MOLECULE: DNA POLYMERASE BETA;
50:	2van-A	7.2	4.7	112	240	11	PDB	MOLECULE: DNA POLYMERSE BETA;
51:	1jn3-A	7.2	4.7	113	242	10	PDB	MOLECULE: DNA POLYMERASE BETA;
52:	2i9g-A	7.2	4.7	115	325	10	PDB	MOLECULE: DNA POLYMERASE BETA;
53:	4o5c-A	7.2	4.3	113	327	10	PDB	MOLECULE: DNA POLYMERASE BETA;
54:	5j0y-A	7.2	4.9	117	327	10	PDB	MOLECULE: DNA POLYMERASE BETA;
55:	5db6-A	7.2	4.2	114	327	10	PDB	MOLECULE: DNA POLYMERASE BETA;
56:	4nln-A	7.2	4.7	119	326	8	PDB	MOLECULE: DNA POLYMERASE BETA;
57:	4o9m-A	7.2	5.0	120	326	9	PDB	MOLECULE: DNA (5'-D(*CP*CP*GP*AP*CP*AP*GP*CP*GP*CP*AP*TP*CP
58:	4m9l-A	7.2	5.2	124	313	10	PDB	MOLECULE: DNA POLYMERASE BETA;
59:	5j0r-A	7.2	4.9	116	327	10	PDB	MOLECULE: DNA POLYMERASE BETA;
60:	4r63-A	7.2	4.2	112	327	10	PDB	MOLECULE: DNA POLYMERASE BETA;
61:	5dbc-A	7.2	4.2	113	327	10	PDB	MOLECULE: DNA POLYMERASE BETA;
62:	5dba-A	7.2	4.9	116	327	10	PDB	MOLECULE: DNA POLYMERASE BETA;
63:	3lqc-B	7.2	4.6	116	193	9	PDB	MOLECULE: DNA REPAIR PROTEIN XRCC1;
64:	5j0q-A	7.1	4.9	116	330	10	PDB	MOLECULE: DNA POLYMERASE BETA;
65:	5j0w-A	7.1	4.3	113	329	10	PDB	MOLECULE: DNA POLYMERASE BETA;
66:	5j0p-A	7.1	4.3	112	330	10	PDB	MOLECULE: DNA POLYMERASE BETA;
67:	4ub2-A	7.1	4.9	116	319	10	PDB	MOLECULE: DNA POLYMERASE BETA;
68:	5db8-A	7.1	4.2	112	327	10	PDB	MOLECULE: DNA POLYMERASE BETA;
69:	5db9-A	7.1	4.3	113	327	10	PDB	MOLECULE: DNA POLYMERASE BETA;
70:	4kli-A	7.1	5.0	120	326	10	PDB	MOLECULE: 5'-D(*CP*CP*GP*AP*CP*GP*GP*CP*GP*CP*AP*TP*CP*AP*G
71:	4klo-A	7.1	5.0	120	326	10	PDB	MOLECULE: 5'-D(*CP*CP*GP*AP*CP*GP*GP*CP*GP*CP*AP*TP*CP*AP*G
72:	4klf-A	7.1	5.1	121	326	10	PDB	MOLECULE: 5'-D(*CP*CP*GP*AP*CP*GP*GP*CP*GP*CP*AP*TP*CP*AP*G
73:	4klj-A	7.1	5.0	120	326	10	PDB	MOLECULE: 5'-D(*CP*CP*GP*AP*CP*GP*GP*CP*GP*CP*AP*TP*CP*AP*G
74:	4gxk-A	7.1	5.1	120	316	10	PDB	MOLECULE: DNA POLYMERASE BETA;
75:	4klm-A	7.1	5.0	120	326	10	PDB	MOLECULE: 5'-D(*CP*CP*GP*AP*CP*GP*GP*CP*GP*CP*AP*TP*CP*AP*G
76:	4klg-A	7.1	5.0	120	326	10	PDB	MOLECULE: 5'-D(*CP*CP*GP*AP*CP*GP*GP*CP*GP*CP*AP*TP*CP*AP*G
77:	3v7j-A	7.1	4.8	118	327	9	PDB	MOLECULE: DNA POLYMERASE BETA;
78:	3v7k-A	7.1	4.9	118	331	9	PDB	MOLECULE: DNA POLYMERASE BETA;
79:	3ogu-A	7.1	4.7	114	326	10	PDB	MOLECULE: DNA POLYMERASE BETA;
80:	3rje-A	7.1	4.9	116	328	9	PDB	MOLECULE: DNA POLYMERASE BETA;
81:	4f5o-A	7.1	4.7	115	321	10	PDB	MOLECULE: DNA POLYMERASE BETA;
82:	1tva-A	7.1	4.7	114	331	10	PDB	MOLECULE: DNA POLYMERASE BETA;
83:	4f5r-A	7.1	4.7	114	325	10	PDB	MOLECULE: DNA POLYMERASE BETA;
84:	4gxi-A	7.1	4.7	115	328	10	PDB	MOLECULE: DNA POLYMERASE BETA;
85:	4klu-A	7.1	4.9	115	322	10	PDB	MOLECULE: DNA POLYMERASE BETA;
86:	1nom-A	7.1	4.4	112	242	10	PDB	MOLECULE: DNA POLYMERASE BETA;
87:	2fmp-A	7.1	5.2	121	326	10	PDB	MOLECULE: 5'-
88:	1huo-A	7.1	5.3	125	325	10	PDB	MOLECULE: DNA POLYMERASE BETA;
89:	3rjk-A	7.1	5.0	120	326	10	PDB	MOLECULE: DNA POLYMERASE BETA;
90:	5j2d-A	7.1	5.1	117	326	10	PDB	MOLECULE: DNA POLYMERASE BETA;
91:	4m2y-A	7.1	5.2	122	319	8	PDB	MOLECULE: DNA POLYMERASE BETA;
92:	1zqy-A	7.1	4.7	115	242	10	PDB	MOLECULE: DNA POLYMERASE BETA;
93:	1zqx-A	7.1	5.1	116	242	9	PDB	MOLECULE: DNA POLYMERASE BETA;
94:	1bpb-A	7.1	4.7	115	242	10	PDB	MOLECULE: DNA POLYMERASE BETA;
95:	2bpc-A	7.1	4.7	116	242	9	PDB	MOLECULE: DNA POLYMERASE BETA;
96:	5j0s-A	7.1	4.9	118	327	10	PDB	MOLECULE: DNA POLYMERASE BETA;
97:	4m47-A	7.1	5.4	125	314	10	PDB	MOLECULE: DNA POLYMERASE BETA;
98:	4nlz-A	7.1	5.4	125	323	10	PDB	MOLECULE: DNA POLYMERASE BETA;
99:	4nm2-A	7.1	5.0	122	321	9	PDB	MOLECULE: DNA POLYMERASE BETA;
100:	4phd-A	7.1	4.6	116	326	10	PDB	MOLECULE: DNA POLYMERASE BETA;
101:	4m9h-A	7.1	4.8	117	314	9	PDB	MOLECULE: DNA POLYMERASE BETA;
102:	4mfa-A	7.1	4.8	118	321	10	PDB	MOLECULE: DNA POLYMERASE BETA;

103:	4p37-B	7.0	5.1	171	498	9	PDB	MOLECULE: PUTATIVE POLY(A) POLYMERASE CATALYTIC SUBUNIT;
104:	4tus-A	7.0	5.2	117	325	10	PDB	MOLECULE: DNA POLYMERASE BETA;
105:	4ub4-A	7.0	5.1	117	326	10	PDB	MOLECULE: DNA POLYMERASE BETA;
106:	5bom-A	7.0	4.9	116	320	10	PDB	MOLECULE: DNA POLYMERASE BETA;
107:	3uxp-A	7.0	5.3	123	326	11	PDB	MOLECULE: DNA POLYMERASE BETA;
108:	4f5r-B	7.0	5.3	121	321	10	PDB	MOLECULE: DNA POLYMERASE BETA;
109:	4kll-A	7.0	5.1	121	326	10	PDB	MOLECULE: 5'-D(*CP*CP*GP*AP*CP*GP*GP*CP*GP*CP*AP*TP*CP*AP*G
110:	4doc-A	7.0	5.0	120	326	10	PDB	MOLECULE: DNA POLYMERASE BETA;
111:	1bpy-A	7.0	5.1	121	326	10	PDB	MOLECULE: PROTEIN (DNA POLYMERASE BETA);
112:	4klp-A	7.0	5.0	120	324	10	PDB	MOLECULE: DNA POLYMERASE BETA;
113:	3uxo-B	7.0	5.0	120	325	10	PDB	MOLECULE: DNA POLYMERASE BETA;
114:	3k75-E	7.0	4.6	114	243	10	PDB	MOLECULE: DNA REPAIR PROTEIN XRCC1;
115:	2p66-A	7.0	4.8	115	326	10	PDB	MOLECULE: DNA POLYMERASE BETA;
116:	9ics-A	7.0	4.8	116	327	9	PDB	MOLECULE: PROTEIN (DNA POLYMERASE BETA (E.C.2.7.7.7));
117:	3gdx-A	7.0	5.3	126	326	10	PDB	MOLECULE: DNA POLYMERASE BETA;
118:	3c2l-A	7.0	5.9	122	326	8	PDB	MOLECULE: DNA POLYMERASE BETA;
119:	1rpl-A	7.0	4.7	114	241	10	PDB	MOLECULE: DNA POLYMERASE BETA;
120:	1bpd-A	7.0	4.8	117	324	9	PDB	MOLECULE: DNA POLYMERASE BETA;
121:	4rq7-A	7.0	4.9	115	325	10	PDB	MOLECULE: DNA POLYMERASE BETA;
122:	4m9j-A	7.0	4.6	111	307	9	PDB	MOLECULE: DNA POLYMERASE BETA;
123:	4z6d-A	7.0	5.1	118	327	10	PDB	MOLECULE: DNA POLYMERASE BETA;
124:	4ymn-A	7.0	4.8	117	327	10	PDB	MOLECULE: DNA POLYMERASE BETA;
125:	4ub5-A	7.0	4.6	113	310	11	PDB	MOLECULE: 5'-D(*CP*CP*GP*AP*CP*CP*GP*CP*GP*CP*AP*TP*CP*AP*G
126:	4kle-A	7.0	5.3	125	326	10	PDB	MOLECULE: 5'-D(*CP*CP*GP*AP*CP*GP*GP*CP*GP*CP*AP*TP*CP*AP*G
127:	5j2f-A	6.9	5.1	117	326	10	PDB	MOLECULE: DNA POLYMERASE BETA;
128:	5j2c-A	6.9	5.1	117	326	10	PDB	MOLECULE: DNA POLYMERASE BETA;
129:	4phe-A	6.9	4.9	116	325	10	PDB	MOLECULE: DNA POLYMERASE BETA;
130:	4o5e-A	6.9	4.9	117	327	10	PDB	MOLECULE: DNA POLYMERASE BETA;
131:	5db7-A	6.9	4.2	114	327	10	PDB	MOLECULE: DNA POLYMERASE BETA;
132:	3rh4-A	6.9	5.0	120	326	10	PDB	MOLECULE: 5'-D(*CP*CP*GP*AP*CP*GP*CP*CP*GP*CP*AP*TP*CP*AP*G
133:	4kls-A	6.9	5.3	120	324	10	PDB	MOLECULE: DNA POLYMERASE BETA;
134:	4klt-A	6.9	5.2	119	314	10	PDB	MOLECULE: DNA POLYMERASE BETA;
135:	3uql-A	6.9	5.1	121	315	8	PDB	MOLECULE: DNA POLYMERASE LAMBDA;
136:	4fo6-A	6.9	5.4	122	315	8	PDB	MOLECULE: DNA POLYMERASE LAMBDA;
137:	1bpz-A	6.9	4.9	119	331	8	PDB	MOLECULE: PROTEIN (DNA POLYMERASE BETA);
138:	2fmq-A	6.9	5.2	121	326	10	PDB	MOLECULE: DNA POLYMERASE BETA;
139:	2bpf-A	6.9	5.2	120	324	10	PDB	MOLECULE: PROTEIN (DNA POLYMERASE BETA (E.C.2.7.7.7));
140:	7icq-A	6.9	4.7	116	327	9	PDB	MOLECULE: PROTEIN (DNA POLYMERASE BETA (E.C.2.7.7.7));
141:	9ict-A	6.9	4.7	115	327	9	PDB	MOLECULE: PROTEIN (DNA POLYMERASE BETA (E.C.2.7.7.7));
142:	9icv-A	6.9	4.8	116	327	9	PDB	MOLECULE: PROTEIN (DNA POLYMERASE BETA (E.C.2.7.7.7));
143:	9ick-A	6.9	4.7	114	327	10	PDB	MOLECULE: PROTEIN (DNA POLYMERASE BETA (E.C.2.7.7.7));
144:	7ich-A	6.9	4.8	116	327	9	PDB	MOLECULE: PROTEIN (DNA POLYMERASE BETA (E.C.2.7.7.7));
145:	7icv-A	6.9	4.7	114	327	10	PDB	MOLECULE: PROTEIN (DNA POLYMERASE BETA (E.C.2.7.7.7));
146:	7icj-A	6.9	4.8	117	327	9	PDB	MOLECULE: PROTEIN (DNA POLYMERASE BETA (E.C.2.7.7.7));
147:	4gxj-A	6.9	5.0	120	315	10	PDB	MOLECULE: DNA POLYMERASE BETA;
148:	5eoz-A	6.9	5.1	117	326	10	PDB	MOLECULE: DNA POLYMERASE BETA;
149:	4mfc-A	6.9	5.5	125	318	9	PDB	MOLECULE: DNA POLYMERASE BETA;
150:	5ddy-C	6.9	5.0	123	327	9	PDB	MOLECULE: DNA POLYMERASE LAMBDA;
151:	3rjg-A	6.9	4.8	115	326	10	PDB	MOLECULE: DNA POLYMERASE BETA;
152:	1zqw-A	6.9	5.1	117	242	9	PDB	MOLECULE: DNA POLYMERASE BETA;
153:	5j0x-A	6.9	4.2	112	328	10	PDB	MOLECULE: DNA POLYMERASE BETA;
154:	4ubl-A	6.9	4.9	116	317	10	PDB	MOLECULE: DNA POLYMERASE BETA;
155:	4mff-A	6.9	5.5	125	321	10	PDB	MOLECULE: DNA POLYMERASE BETA;
156:	4m9g-A	6.9	4.7	115	314	10	PDB	MOLECULE: DNA POLYMERASE BETA;
157:	5j2j-A	6.9	5.1	117	326	10	PDB	MOLECULE: DNA POLYMERASE BETA;
158:	5bol-A	6.9	4.7	123	325	9	PDB	MOLECULE: DNA (5'-D(*CP*CP*GP*AP*CP*(CDO)
159:	4pha-A	6.9	5.1	118	327	10	PDB	MOLECULE: DNA POLYMERASE BETA;
160:	4klq-A	6.9	5.4	125	324	10	PDB	MOLECULE: DNA POLYMERASE BETA;
161:	4f5p-A	6.9	5.4	126	326	10	PDB	MOLECULE: DNA POLYMERASE BETA;
162:	5j2h-A	6.9	5.0	120	326	10	PDB	MOLECULE: DNA POLYMERASE BETA;
163:	4wse-A	6.8	4.0	147	495	11	PDB	MOLECULE: PUTATIVE POLY(A) POLYMERASE CATALYTIC SUBUNIT;
164:	4tuq-A	6.8	5.1	117	326	10	PDB	MOLECULE: DNA POLYMERASE BETA;
165:	4o5k-A	6.8	5.1	117	326	10	PDB	MOLECULE: DNA (5'-D(*CP*CP*GP*AP*CP*(FMG)

166:	5j2e-A	6.8	5.1	117	326	10	PDB	MOLECULE: DNA POLYMERASE BETA;
167:	5j2g-A	6.8	5.1	117	326	10	PDB	MOLECULE: DNA POLYMERASE BETA;
168:	5j2k-A	6.8	5.1	117	326	10	PDB	MOLECULE: DNA POLYMERASE BETA;
169:	4klh-A	6.8	5.0	120	326	10	PDB	MOLECULE: 5'-D(*CP*CP*GP*AP*CP*GP*GP*CP*GP*CP*AP*TP*CP*AP*G
170:	4jwm-A	6.8	5.0	120	326	11	PDB	MOLECULE: DNA POLYMERASE BETA;
171:	3rji-A	6.8	5.0	120	326	10	PDB	MOLECULE: DNA POLYMERASE BETA;
172:	4jwn-A	6.8	5.0	120	326	10	PDB	MOLECULE: DNA POLYMERASE BETA;
173:	3rjh-A	6.8	5.0	120	326	10	PDB	MOLECULE: DNA POLYMERASE BETA;
174:	3uxp-B	6.8	5.0	120	326	10	PDB	MOLECULE: DNA POLYMERASE BETA;
175:	3jpq-A	6.8	5.0	120	326	10	PDB	MOLECULE: DNA POLYMERASE BETA;
176:	3rjj-A	6.8	4.6	114	326	10	PDB	MOLECULE: DNA POLYMERASE BETA;
177:	9icp-A	6.8	4.8	115	327	9	PDB	MOLECULE: PROTEIN (DNA POLYMERASE BETA (E.C.2.7.7.7));
178:	8ics-A	6.8	5.0	116	327	9	PDB	MOLECULE: DNA (5'-D(*CP*AP*TP*TP*GP*AP*A)-3');
179:	9ico-A	6.8	5.0	116	327	9	PDB	MOLECULE: PROTEIN (DNA POLYMERASE BETA (E.C.2.7.7.7));
180:	1zqa-A	6.8	5.0	116	327	9	PDB	MOLECULE: PROTEIN (DNA POLYMERASE BETA (E.C.2.7.7.7));
181:	1zqn-A	6.8	4.7	116	327	9	PDB	MOLECULE: PROTEIN (DNA POLYMERASE BETA (E.C.2.7.7.7));
182:	9icy-A	6.8	4.8	116	327	9	PDB	MOLECULE: PROTEIN (DNA POLYMERASE BETA (E.C.2.7.7.7));
183:	9icx-A	6.8	4.8	116	327	9	PDB	MOLECULE: PROTEIN (DNA POLYMERASE BETA (E.C.2.7.7.7));
184:	1zqo-A	6.8	4.7	114	327	10	PDB	MOLECULE: PROTEIN (DNA POLYMERASE BETA (E.C.2.7.7.7));
185:	8icg-A	6.8	4.7	114	327	10	PDB	MOLECULE: PROTEIN (DNA POLYMERASE BETA (E.C.2.7.7.7));
186:	1zqe-A	6.8	4.7	114	327	10	PDB	MOLECULE: PROTEIN (DNA POLYMERASE BETA (E.C.2.7.7.7));
187:	1zqg-A	6.8	4.8	115	327	10	PDB	MOLECULE: PROTEIN (DNA POLYMERASE BETA (E.C.2.7.7.7));
188:	1zqr-A	6.8	4.7	114	327	10	PDB	MOLECULE: PROTEIN (DNA POLYMERASE BETA (E.C.2.7.7.7));
189:	1zqq-A	6.8	4.7	115	327	9	PDB	MOLECULE: PROTEIN (DNA POLYMERASE BETA (E.C.2.7.7.7));
190:	7icp-A	6.8	4.8	116	327	9	PDB	MOLECULE: PROTEIN (DNA POLYMERASE BETA (E.C.2.7.7.7));
191:	1zqi-A	6.8	4.7	114	327	10	PDB	MOLECULE: PROTEIN (DNA POLYMERASE BETA (E.C.2.7.7.7));
192:	1zqf-A	6.8	4.7	115	327	9	PDB	MOLECULE: PROTEIN (DNA POLYMERASE BETA (E.C.2.7.7.7));
193:	1zqp-A	6.8	5.0	115	327	9	PDB	MOLECULE: PROTEIN (DNA POLYMERASE BETA (E.C.2.7.7.7));
194:	9ice-A	6.8	4.7	115	327	9	PDB	MOLECULE: PROTEIN (DNA POLYMERASE BETA (E.C.2.7.7.7));
195:	9ica-A	6.8	4.8	116	327	9	PDB	MOLECULE: PROTEIN (DNA POLYMERASE BETA (E.C.2.7.7.7));
196:	3c2m-A	6.8	6.0	124	326	8	PDB	MOLECULE: DNA POLYMERASE BETA;
197:	1huz-A	6.8	5.3	122	325	10	PDB	MOLECULE: DNA POLYMERASE BETA;
198:	4pgx-A	6.8	5.1	117	326	10	PDB	MOLECULE: DNA (5'-D(*CP*CP*GP*AP*CP*GP*TP*CP*GP*CP*AP*TP*CP
199:	4ubb-A	6.8	5.1	117	326	10	PDB	MOLECULE: 5'-D(*CP*CP*GP*AP*CP*CP*GP*CP*GP*CP*AP*TP*CP*AP*G
200:	4ymo-A	6.8	5.1	117	326	10	PDB	MOLECULE: DNA POLYMERASE BETA;
201:	7ice-A	6.8	4.7	116	327	9	PDB	MOLECULE: PROTEIN (DNA POLYMERASE BETA);
202:	4r64-A	6.8	4.9	116	326	10	PDB	MOLECULE: DNA POLYMERASE BETA;
203:	4ymm-A	6.8	4.9	116	327	10	PDB	MOLECULE: DNA POLYMERASE BETA;
204:	1bpe-A	6.8	5.1	119	284	8	PDB	MOLECULE: DNA POLYMERASE BETA;
205:	4ubc-A	6.8	5.1	117	326	10	PDB	MOLECULE: 5'-D(*CP*CP*GP*AP*CP*CP*GP*CP*GP*CP*AP*TP*CP*AP*G
206:	5j2b-A	6.8	5.1	117	326	10	PDB	MOLECULE: DNA POLYMERASE BETA;
207:	4mf2-A	6.8	4.9	120	321	9	PDB	MOLECULE: DNA POLYMERASE BETA;
208:	5cr0-A	6.8	4.5	125	246	7	PDB	MOLECULE: DNA POLYMERASE LAMBDA;
209:	5cp2-B	6.8	5.5	129	246	8	PDB	MOLECULE: DNA POLYMERASE LAMBDA;
210:	5j2i-A	6.7	4.7	123	326	9	PDB	MOLECULE: DNA POLYMERASE BETA;
211:	4ub3-A	6.7	5.1	117	323	10	PDB	MOLECULE: 5'-D(*CP*CP*GP*AP*CP*CP*GP*CP*GP*CP*AP*TP*CP*AP*G
212:	4rq5-A	6.7	5.0	116	326	10	PDB	MOLECULE: DNA POLYMERASE BETA;
213:	4rpy-A	6.7	5.1	117	326	10	PDB	MOLECULE: DNA POLYMERASE BETA;
214:	4rq0-A	6.7	5.1	117	327	10	PDB	MOLECULE: DNA POLYMERASE BETA;
215:	3k75-D	6.7	4.7	119	248	9	PDB	MOLECULE: DNA REPAIR PROTEIN XRCC1;
216:	3jpt-A	6.7	5.0	120	326	10	PDB	MOLECULE: DNA POLYMERASE BETA;
217:	3isd-A	6.7	5.0	120	326	10	PDB	MOLECULE: DNA POLYMERASE BETA;
218:	4doa-A	6.7	5.0	120	326	10	PDB	MOLECULE: DNA POLYMERASE BETA;
219:	3rh6-A	6.7	5.0	120	326	10	PDB	MOLECULE: DNA POLYMERASE BETA;
220:	3jps-A	6.7	5.0	120	326	10	PDB	MOLECULE: DNA POLYMERASE BETA;
221:	3uxn-B	6.7	5.1	120	325	10	PDB	MOLECULE: DNA POLYMERASE BETA;
222:	9icn-A	6.7	4.8	116	327	9	PDB	MOLECULE: PROTEIN (DNA POLYMERASE BETA (E.C.2.7.7.7));
223:	2fms-A	6.7	5.2	123	326	10	PDB	MOLECULE: DNA POLYMERASE BETA;
224:	1huo-B	6.7	5.2	121	325	10	PDB	MOLECULE: DNA POLYMERASE BETA;
225:	2pxi-A	6.7	5.2	121	326	10	PDB	MOLECULE: DNA POLYMERASE BETA;
226:	9icm-A	6.7	4.8	116	327	9	PDB	MOLECULE: PROTEIN (DNA POLYMERASE BETA (E.C.2.7.7.7));
227:	8ico-A	6.7	4.8	116	327	9	PDB	MOLECULE: PROTEIN (DNA POLYMERASE BETA (E.C.2.7.7.7));
228:	8ict-A	6.7	5.0	116	327	9	PDB	MOLECULE: PROTEIN (DNA POLYMERASE BETA (E.C.2.7.7.7));

229:	9icl-A	6.7	4.7	114	327	10	PDB	MOLECULE: PROTEIN (DNA POLYMERASE BETA (E.C.2.7.7.7));
230:	8icc-A	6.7	4.7	114	327	10	PDB	MOLECULE: PROTEIN (DNA POLYMERASE BETA (E.C.2.7.7.7));
231:	9icw-A	6.7	4.7	115	327	9	PDB	MOLECULE: PROTEIN (DNA POLYMERASE BETA (E.C.2.7.7.7));
232:	7ici-A	6.7	4.8	116	327	9	PDB	MOLECULE: PROTEIN (DNA POLYMERASE BETA (E.C.2.7.7.7));
233:	7icr-A	6.7	4.8	116	327	9	PDB	MOLECULE: PROTEIN (DNA POLYMERASE BETA (E.C.2.7.7.7));
234:	8icb-A	6.7	4.8	115	327	10	PDB	MOLECULE: PROTEIN (DNA POLYMERASE BETA (E.C.2.7.7.7));
235:	8icv-A	6.7	4.7	114	327	10	PDB	MOLECULE: PROTEIN (DNA POLYMERASE BETA (E.C.2.7.7.7));
236:	9icu-A	6.7	4.8	117	327	9	PDB	MOLECULE: PROTEIN (DNA POLYMERASE BETA (E.C.2.7.7.7));
237:	1zqk-A	6.7	5.1	116	327	9	PDB	MOLECULE: PROTEIN (DNA POLYMERASE BETA (E.C.2.7.7.7));
238:	7ics-A	6.7	4.8	116	327	9	PDB	MOLECULE: PROTEIN (DNA POLYMERASE BETA (E.C.2.7.7.7));
239:	9icg-A	6.7	4.8	116	327	9	PDB	MOLECULE: PROTEIN (DNA POLYMERASE BETA (E.C.2.7.7.7));
240:	3c2k-A	6.7	5.0	118	326	8	PDB	MOLECULE: DNA POLYMERASE BETA;
241:	4r65-A	6.7	5.2	120	326	10	PDB	MOLECULE: DNA POLYMERASE BETA;
242:	2bpg-A	6.7	5.1	123	324	10	PDB	MOLECULE: DNA POLYMERASE BETA;
243:	4rpx-A	6.7	5.1	116	326	10	PDB	MOLECULE: DNA POLYMERASE BETA;
244:	8icq-A	6.7	4.7	115	327	9	PDB	MOLECULE: PROTEIN (DNA POLYMERASE BETA (E.C.2.7.7.7));
245:	1zqv-A	6.7	4.8	115	242	10	PDB	MOLECULE: DNA POLYMERASE BETA;
246:	8icr-A	6.7	4.7	114	327	10	PDB	MOLECULE: PROTEIN (DNA POLYMERASE BETA (E.C.2.7.7.7));
247:	8ick-A	6.7	4.8	116	327	9	PDB	MOLECULE: PROTEIN (DNA POLYMERASE BETA (E.C.2.7.7.7));
248:	4nlk-A	6.7	5.3	125	326	10	PDB	MOLECULE: 5'-D(*CP*CP*GP*AP*CP*(BGM)P*TP*CP*GP*CP*AP*TP*CP*
249:	3v72-A	6.7	4.9	120	327	9	PDB	MOLECULE: DNA POLYMERASE BETA;
250:	4nm1-A	6.7	5.3	127	326	9	PDB	MOLECULE: 5'-D(*CP*CP*GP*AP*CP*GP*(BGM)P*CP*GP*CP*AP*TP*CP*
251:	4nxz-A	6.7	5.4	126	326	10	PDB	MOLECULE: 5'-D(*CP*CP*GP*AP*CP*(6OG)P*TP*CP*GP*CP*AP*TP*CP*
252:	4uay-A	6.7	5.1	117	326	10	PDB	MOLECULE: 5'-D(*CP*CP*GP*AP*CP*AP*GP*CP*GP*CP*AP*TP*CP*AP*G
253:	4ph5-A	6.7	5.1	118	326	10	PDB	MOLECULE: DNA (5'-D(*CP*CP*GP*AP*CP*GP*AP*CP*GP*CP*AP*TP*CP
254:	4k4h-I	6.7	4.6	123	332	8	PDB	MOLECULE: DNA POLYMERASE LAMBDA;
255:	3rh5-A	6.7	5.3	125	326	10	PDB	MOLECULE: DNA POLYMERASE BETA;
256:	4ycx-A	6.7	5.5	126	331	9	PDB	MOLECULE: DNA-DIRECTED DNA/RNA POLYMERASE MU;
257:	5ca7-B	6.7	5.5	129	247	8	PDB	MOLECULE: DNA POLYMERASE LAMBDA;
258:	4xq8-B	6.7	5.5	129	247	8	PDB	MOLECULE: DNA POLYMERASE LAMBDA;
259:	5chg-A	6.7	4.5	125	246	7	PDB	MOLECULE: DNA POLYMERASE LAMBDA;
260:	4z6f-A	6.6	4.6	123	326	10	PDB	MOLECULE: DNA POLYMERASE BETA;
261:	1zjn-A	6.6	5.0	118	325	10	PDB	MOLECULE: D(*CP*CP*GP*AP*CP*CP*AP*CP*GP*CP*AP*TP*CP*AP*GP*C
262:	4rpz-A	6.6	5.1	116	326	10	PDB	MOLECULE: DNA POLYMERASE BETA;
263:	3lk9-A	6.6	5.0	119	326	10	PDB	MOLECULE: DNA POLYMERASE BETA;
264:	9icr-A	6.6	4.8	116	327	9	PDB	MOLECULE: PROTEIN (DNA POLYMERASE BETA (E.C.2.7.7.7));
265:	9icq-A	6.6	4.8	116	327	9	PDB	MOLECULE: PROTEIN (DNA POLYMERASE BETA (E.C.2.7.7.7));
266:	1huz-B	6.6	5.3	123	325	10	PDB	MOLECULE: DNA POLYMERASE BETA;
267:	8ice-A	6.6	4.7	115	327	9	PDB	MOLECULE: PROTEIN (DNA POLYMERASE BETA (E.C.2.7.7.7));
268:	9ici-A	6.6	4.7	114	327	10	PDB	MOLECULE: PROTEIN (DNA POLYMERASE BETA (E.C.2.7.7.7));
269:	8icy-A	6.6	4.8	116	327	9	PDB	MOLECULE: PROTEIN (DNA POLYMERASE BETA (E.C.2.7.7.7));
270:	9icj-A	6.6	4.8	116	327	9	PDB	MOLECULE: PROTEIN (DNA POLYMERASE BETA (E.C.2.7.7.7));
271:	8icm-A	6.6	4.7	114	327	10	PDB	MOLECULE: PROTEIN (DNA POLYMERASE BETA (E.C.2.7.7.7));
272:	7ict-A	6.6	4.7	114	327	10	PDB	MOLECULE: PROTEIN (DNA POLYMERASE BETA (E.C.2.7.7.7));
273:	7icu-A	6.6	4.7	116	327	9	PDB	MOLECULE: PROTEIN (DNA POLYMERASE BETA (E.C.2.7.7.7));
274:	8ich-A	6.6	4.8	116	327	9	PDB	MOLECULE: PROTEIN (DNA POLYMERASE BETA (E.C.2.7.7.7));
275:	8ici-A	6.6	4.7	114	327	10	PDB	MOLECULE: PROTEIN (DNA POLYMERASE BETA (E.C.2.7.7.7));
276:	8icp-A	6.6	4.8	116	327	9	PDB	MOLECULE: PROTEIN (DNA POLYMERASE BETA (E.C.2.7.7.7));
277:	7icm-A	6.6	4.8	116	327	9	PDB	MOLECULE: PROTEIN (DNA POLYMERASE BETA (E.C.2.7.7.7));
278:	1zqt-A	6.6	4.7	114	327	10	PDB	MOLECULE: PROTEIN (DNA POLYMERASE BETA (E.C.2.7.7.7));
279:	8icx-A	6.6	4.7	115	327	9	PDB	MOLECULE: PROTEIN (DNA POLYMERASE BETA (E.C.2.7.7.7));
280:	8ica-A	6.6	4.7	115	327	9	PDB	MOLECULE: PROTEIN (DNA POLYMERASE BETA (E.C.2.7.7.7));
281:	8icn-A	6.6	4.7	114	327	10	PDB	MOLECULE: PROTEIN (DNA POLYMERASE BETA (E.C.2.7.7.7));
282:	8icz-A	6.6	4.7	114	327	10	PDB	MOLECULE: PROTEIN (DNA POLYMERASE BETA (E.C.2.7.7.7));
283:	9icb-A	6.6	4.7	114	327	10	PDB	MOLECULE: PROTEIN (DNA POLYMERASE BETA (E.C.2.7.7.7));
284:	9ich-A	6.6	5.1	116	327	9	PDB	MOLECULE: PROTEIN (DNA POLYMERASE BETA (E.C.2.7.7.7));
285:	9icf-A	6.6	4.8	116	327	9	PDB	MOLECULE: PROTEIN (DNA POLYMERASE BETA (E.C.2.7.7.7));
286:	4php-A	6.6	5.1	117	326	10	PDB	MOLECULE: DNA (5'-D(*CP*CP*GP*AP*CP*TP*GP*CP*GP*CP*AP*TP*CP
287:	1zqu-A	6.6	4.7	115	242	10	PDB	MOLECULE: DNA POLYMERASE BETA;
288:	1xsl-I	6.6	5.0	127	326	8	PDB	MOLECULE: 5'-D(*CP*GP*GP*CP*AP*GP*CP*GP*CP*AP*C)-3';
289:	2gws-M	6.6	5.3	128	326	8	PDB	MOLECULE: 5'-D(*CP*GP*GP*CP*AP*GP*CP*GP*CP*AP*C)-3';
290:	8icu-A	6.6	4.8	116	327	9	PDB	MOLECULE: PROTEIN (DNA POLYMERASE BETA (E.C.2.7.7.7));
291:	7ick-A	6.6	4.8	116	327	9	PDB	MOLECULE: PROTEIN (DNA POLYMERASE BETA (E.C.2.7.7.7));

☐	292:	8icw-A	6.6	4.7	117	327	9 PDB	MOLECULE: PROTEIN (DNA POLYMERASE BETA (E.C.2.7.7.7));
☐	293:	1xsn-A	6.6	5.1	122	324	9 PDB	MOLECULE: DNA POLYMERASE LAMBDA;
☐	294:	3pnc-A	6.6	5.4	125	316	8 PDB	MOLECULE: DNA POLYMERASE LAMBDA;
☐	295:	4uaw-A	6.6	5.1	119	326	10 PDB	MOLECULE: 5'-D(*CP*CP*GP*AP*CP*AP*GP*CP*GP*CP*AP*TP*CP*AP*G
☐	296:	4k4g-I	6.6	4.7	123	327	8 PDB	MOLECULE: DNA POLYMERASE LAMBDA;
☐	297:	4rq1-A	6.6	5.0	120	324	11 PDB	MOLECULE: DNA POLYMERASE BETA;
☐	298:	3pmn-A	6.6	5.4	125	312	8 PDB	MOLECULE: DNA POLYMERASE LAMBDA;
☐	299:	4z6e-A	6.6	5.1	126	326	10 PDB	MOLECULE: DNA (5'-D(*CP*CP*GP*AP*CP*GP*TP*CP*GP*CP*AP*TP*CP
☐	300:	4rq2-A	6.5	4.1	119	333	10 PDB	MOLECULE: DNA POLYMERASE BETA;
☐	301:	5j2a-A	6.5	5.1	120	326	10 PDB	MOLECULE: DNA POLYMERASE BETA;
☐	302:	3mby-A	6.5	5.0	120	326	10 PDB	MOLECULE: DNA POLYMERASE BETA;
☐	303:	3jpr-A	6.5	5.0	120	326	10 PDB	MOLECULE: DNA POLYMERASE BETA;
☐	304:	3rjf-A	6.5	5.3	121	326	10 PDB	MOLECULE: DNA POLYMERASE BETA;
☐	305:	3jpn-A	6.5	5.0	120	326	10 PDB	MOLECULE: DNA POLYMERASE BETA;
☐	306:	4do9-A	6.5	5.0	120	326	10 PDB	MOLECULE: DNA POLYMERASE BETA;
☐	307:	3jpo-A	6.5	5.0	120	326	10 PDB	MOLECULE: DNA POLYMERASE BETA;
☐	308:	4dob-A	6.5	5.1	121	326	10 PDB	MOLECULE: DNA POLYMERASE BETA;
☐	309:	3c5g-B	6.5	5.4	128	329	9 PDB	MOLECULE: DNA POLYMERASE LAMBDA;
☐	310:	3mda-A	6.5	5.1	122	325	8 PDB	MOLECULE: DNA POLYMERASE LAMBDA;
☐	311:	3uq0-A	6.5	5.1	120	310	8 PDB	MOLECULE: DNA POLYMERASE LAMBDA;
☐	312:	3mgh-C	6.5	5.3	124	321	8 PDB	MOLECULE: DNA POLYMERASE LAMBDA;
☐	313:	2isp-A	6.5	5.0	120	326	10 PDB	MOLECULE: POLYMERASE (DNA DIRECTED), BETA;
☐	314:	7icg-A	6.5	4.8	118	327	8 PDB	MOLECULE: PROTEIN (DNA POLYMERASE BETA (E.C.2.7.7.7));
☐	315:	8icl-A	6.5	4.8	117	327	9 PDB	MOLECULE: PROTEIN (DNA POLYMERASE BETA (E.C.2.7.7.7));
☐	316:	1zqb-A	6.5	4.7	114	327	10 PDB	MOLECULE: PROTEIN (DNA POLYMERASE BETA (E.C.2.7.7.7));
☐	317:	7icn-A	6.5	4.7	115	327	9 PDB	MOLECULE: PROTEIN (DNA POLYMERASE BETA (E.C.2.7.7.7));
☐	318:	1zqd-A	6.5	4.7	115	327	9 PDB	MOLECULE: PROTEIN (DNA POLYMERASE BETA (E.C.2.7.7.7));
☐	319:	7icf-A	6.5	4.8	116	327	9 PDB	MOLECULE: PROTEIN (DNA POLYMERASE BETA (E.C.2.7.7.7));
☐	320:	9icc-A	6.5	4.7	115	327	9 PDB	MOLECULE: PROTEIN (DNA POLYMERASE BETA (E.C.2.7.7.7));
☐	321:	8icj-A	6.5	4.7	114	327	10 PDB	MOLECULE: PROTEIN (DNA POLYMERASE BETA (E.C.2.7.7.7));
☐	322:	3jpp-A	6.5	5.0	120	326	10 PDB	MOLECULE: DNA POLYMERASE BETA;
☐	323:	4kld-A	6.5	5.0	120	326	10 PDB	MOLECULE: 5'-D(*CP*CP*GP*AP*CP*GP*GP*CP*GP*CP*AP*TP*CP*AP*G
☐	324:	4rq6-A	6.5	5.1	117	326	10 PDB	MOLECULE: DNA POLYMERASE BETA;
☐	325:	1xsl-M	6.5	5.3	128	327	9 PDB	MOLECULE: 5'-D(*CP*GP*GP*CP*AP*GP*CP*GP*CP*AP*C)-3';
☐	326:	2bcr-A	6.5	5.1	122	325	9 PDB	MOLECULE: DNA POLYMERASE LAMBDA;
☐	327:	2pfn-A	6.5	5.1	120	325	9 PDB	MOLECULE: DNA POLYMERASE LAMBDA;
☐	328:	1rzt-A	6.5	4.3	124	327	8 PDB	MOLECULE: DNA POLYMERASE LAMBDA;
☐	329:	7ico-A	6.5	5.1	118	327	8 PDB	MOLECULE: PROTEIN (DNA POLYMERASE BETA (E.C.2.7.7.7));
☐	330:	3hw8-A	6.5	5.2	124	327	9 PDB	MOLECULE: DNA POLYMERASE LAMBDA;
☐	331:	4uaz-A	6.5	5.1	117	325	10 PDB	MOLECULE: 5'-D(*CP*CP*GP*AP*CP*AP*GP*CP*GP*CP*AP*TP*CP*AP*G
☐	332:	4rq3-A	6.5	5.1	116	326	10 PDB	MOLECULE: DNA POLYMERASE BETA;
☐	333:	5cr0-B	6.5	5.2	128	243	8 PDB	MOLECULE: DNA POLYMERASE LAMBDA;
☐	334:	5iim-A	6.5	5.2	125	326	9 PDB	MOLECULE: DNA POLYMERASE LAMBDA;
☐	335:	5iin-A	6.5	5.2	125	326	9 PDB	MOLECULE: DNA POLYMERASE LAMBDA;
☐	336:	4rq8-A	6.4	4.5	125	333	10 PDB	MOLECULE: DNA POLYMERASE BETA;
☐	337:	4fhx-A	6.4	8.9	132	323	7 PDB	MOLECULE: POLY(A) RNA POLYMERASE PROTEIN CID1;
☐	338:	4rq4-A	6.4	5.1	117	326	10 PDB	MOLECULE: DNA POLYMERASE BETA;
☐	339:	3pml-A	6.4	5.2	124	322	8 PDB	MOLECULE: DNA POLYMERASE LAMBDA;
☐	340:	3mgi-A	6.4	5.1	121	318	8 PDB	MOLECULE: DNA POLYMERASE LAMBDA;
☐	341:	3uq2-A	6.4	5.1	119	318	8 PDB	MOLECULE: DNA POLYMERASE LAMBDA;
☐	342:	2iso-A	6.4	5.2	123	326	10 PDB	MOLECULE: POLYMERASE (DNA DIRECTED), BETA;
☐	343:	1mq3-A	6.4	5.2	121	326	10 PDB	MOLECULE: DNA POLYMERASE BETA;
☐	344:	1zqs-A	6.4	4.7	114	327	10 PDB	MOLECULE: PROTEIN (DNA POLYMERASE BETA (E.C.2.7.7.7));
☐	345:	1zqh-A	6.4	4.7	114	327	10 PDB	MOLECULE: PROTEIN (DNA POLYMERASE BETA (E.C.2.7.7.7));
☐	346:	1zqj-A	6.4	5.1	116	327	9 PDB	MOLECULE: PROTEIN (DNA POLYMERASE BETA (E.C.2.7.7.7));
☐	347:	3hwt-A	6.4	5.4	123	319	8 PDB	MOLECULE: DNA POLYMERASE LAMBDA;
☐	348:	2pfp-A	6.4	5.2	124	326	9 PDB	MOLECULE: DNA POLYMERASE LAMBDA;
☐	349:	2pfq-A	6.4	5.1	121	324	9 PDB	MOLECULE: DNA POLYMERASE LAMBDA;
☐	350:	5ddy-G	6.4	4.4	123	244	8 PDB	MOLECULE: DNA POLYMERASE LAMBDA;
☐	351:	2bcv-A	6.4	5.2	124	323	9 PDB	MOLECULE: DNA POLYMERASE LAMBDA;
☐	352:	2bcq-A	6.4	5.2	124	324	9 PDB	MOLECULE: DNA POLYMERASE LAMBDA;
☐	353:	3hx0-F	6.4	5.5	126	329	8 PDB	MOLECULE: DNA POLYMERASE LAMBDA;
☐	354:	3hx0-A	6.4	5.2	125	330	8 PDB	MOLECULE: DNA POLYMERASE LAMBDA;

☐	355:	5cbl-A	6.4	4.9	128	326	9 PDB	MOLECULE: DNA POLYMERASE LAMBDA;
☐	356:	4k4i-E	6.4	4.3	124	329	8 PDB	MOLECULE: DNA POLYMERASE LAMBDA;
☐	357:	5dkw-A	6.4	5.6	129	319	9 PDB	MOLECULE: DNA POLYMERASE LAMBDA;
☐	358:	5iil-A	6.4	5.4	126	326	9 PDB	MOLECULE: DNA POLYMERASE LAMBDA;
☐	359:	5iik-A	6.4	5.4	126	325	9 PDB	MOLECULE: DNA POLYMERASE LAMBDA;
☐	360:	4fh3-A	6.3	8.9	132	323	6 PDB	MOLECULE: POLY(A) RNA POLYMERASE PROTEIN CID1;
☐	361:	4fh5-A	6.3	9.0	131	323	6 PDB	MOLECULE: POLY(A) RNA POLYMERASE PROTEIN CID1;
☐	362:	4fhw-A	6.3	9.0	131	323	6 PDB	MOLECULE: POLY(A) RNA POLYMERASE PROTEIN CID1;
☐	363:	4fhv-A	6.3	8.9	131	323	6 PDB	MOLECULE: POLY(A) RNA POLYMERASE PROTEIN CID1;
☐	364:	4p4o-A	6.3	4.7	119	306	8 PDB	MOLECULE: DNA POLYMERASE BETA;
☐	365:	1bpx-A	6.3	5.0	116	331	10 PDB	MOLECULE: PROTEIN (DNA POLYMERASE BETA);
☐	366:	7icl-A	6.3	5.1	116	327	9 PDB	MOLECULE: PROTEIN (DNA POLYMERASE BETA (E.C.2.7.7.7));
☐	367:	1zql-A	6.3	4.7	115	327	9 PDB	MOLECULE: PROTEIN (DNA POLYMERASE BETA (E.C.2.7.7.7));
☐	368:	1zqc-A	6.3	4.7	114	327	10 PDB	MOLECULE: PROTEIN (DNA POLYMERASE BETA (E.C.2.7.7.7));
☐	369:	1mq2-A	6.3	4.8	118	326	8 PDB	MOLECULE: DNA POLYMERASE BETA;
☐	370:	1zqm-A	6.3	4.7	115	327	9 PDB	MOLECULE: PROTEIN (DNA POLYMERASE BETA (E.C.2.7.7.7));
☐	371:	8icf-A	6.3	4.7	114	327	10 PDB	MOLECULE: PROTEIN (DNA POLYMERASE BETA (E.C.2.7.7.7));
☐	372:	2bcu-A	6.3	5.2	122	326	9 PDB	MOLECULE: DNA POLYMERASE LAMBDA;
☐	373:	2ihm-A	6.3	3.7	118	332	8 PDB	MOLECULE: DNA POLYMERASE MU;
☐	374:	1xsp-A	6.3	5.2	124	327	9 PDB	MOLECULE: DNA POLYMERASE LAMBDA;
☐	375:	2bcs-A	6.3	5.2	124	324	9 PDB	MOLECULE: DNA POLYMERASE LAMBDA;
☐	376:	4k4h-A	6.3	5.6	131	327	9 PDB	MOLECULE: DNA POLYMERASE LAMBDA;
☐	377:	5ddm-A	6.3	5.2	128	242	9 PDB	MOLECULE: DNA POLYMERASE LAMBDA;
☐	378:	5cbl-B	6.3	4.3	125	245	8 PDB	MOLECULE: DNA POLYMERASE LAMBDA;
☐	379:	4xus-A	6.3	5.2	121	323	9 PDB	MOLECULE: DNA POLYMERASE LAMBDA;
☐	380:	4x5v-A	6.3	5.2	125	317	9 PDB	MOLECULE: DNA POLYMERASE LAMBDA;
☐	381:	5ddy-E	6.3	4.5	125	244	8 PDB	MOLECULE: DNA POLYMERASE LAMBDA;
☐	382:	1xsl-A	6.2	5.5	128	327	8 PDB	MOLECULE: 5'-D(*CP*GP*GP*CP*AP*GP*CP*GP*CP*AP*C)-3';
☐	383:	3pml-B	6.2	5.5	119	324	8 PDB	MOLECULE: DNA POLYMERASE LAMBDA;
☐	384:	4p4p-A	6.2	4.5	116	282	7 PDB	MOLECULE: DNA POLYMERASE BETA;
☐	385:	4yd2-A	6.2	4.6	112	323	10 PDB	MOLECULE: DNA-DIRECTED DNA/RNA POLYMERASE MU;
☐	386:	4k4i-A	6.2	5.6	131	333	9 PDB	MOLECULE: DNA POLYMERASE LAMBDA;
☐	387:	5ddy-A	6.2	6.1	132	327	8 PDB	MOLECULE: DNA POLYMERASE LAMBDA;
☐	388:	4xrh-B	6.2	5.5	129	247	8 PDB	MOLECULE: DNA POLYMERASE LAMBDA;
☐	389:	5ddm-B	6.2	5.3	129	247	8 PDB	MOLECULE: DNA POLYMERASE LAMBDA;
☐	390:	4qzf-A	6.2	4.5	119	336	8 PDB	MOLECULE: DNA NUCLEOTIDYLEXOTRANSFERASE;
☐	391:	1kej-A	6.1	4.5	125	357	8 PDB	MOLECULE: TERMINAL DEOXYNUCLEOTIDYLTRANSFERASE SHORT
☐	392:	1rzt-M	6.1	4.7	127	327	7 PDB	MOLECULE: DNA POLYMERASE LAMBDA;
☐	393:	4p4m-A	6.1	4.5	116	270	7 PDB	MOLECULE: DNA POLYMERASE BETA;
☐	394:	4m0a-A	6.1	5.5	123	325	9 PDB	MOLECULE: DNA-DIRECTED DNA/RNA POLYMERASE MU;
☐	395:	4fhy-A	6.0	9.0	129	318	6 PDB	MOLECULE: POLY(A) RNA POLYMERASE PROTEIN CID1;
☐	396:	4k4i-I	6.0	4.7	123	325	8 PDB	MOLECULE: DNA POLYMERASE LAMBDA;
☐	397:	3c5g-A	6.0	5.5	126	324	9 PDB	MOLECULE: DNA POLYMERASE LAMBDA;
☐	398:	2be3-B	6.0	4.5	123	189	6 PDB	MOLECULE: GTP PYROPHOSPHOKINASE;
☐	399:	4qze-A	6.0	4.4	114	335	8 PDB	MOLECULE: DNA NUCLEOTIDYLEXOTRANSFERASE;
☐	400:	4e80-A	5.9	8.4	126	317	7 PDB	MOLECULE: POLY(A) RNA POLYMERASE PROTEIN CID1;
☐	401:	1jms-A	5.9	4.3	117	360	8 PDB	MOLECULE: TERMINAL DEOXYNUCLEOTIDYLTRANSFERASE;
☐	402:	3mgh-A	5.9	5.3	124	319	8 PDB	MOLECULE: DNA POLYMERASE LAMBDA;
☐	403:	4k4g-A	5.9	5.4	131	322	8 PDB	MOLECULE: DNA POLYMERASE LAMBDA;
☐	404:	4qzh-A	5.9	4.4	116	337	8 PDB	MOLECULE: DNA NUCLEOTIDYLEXOTRANSFERASE;
☐	405:	5ded-G	5.9	4.5	123	190	8 PDB	MOLECULE: GTP PYROPHOSPHOKINASE YJBM;
☐	406:	5ded-A	5.9	4.2	123	196	8 PDB	MOLECULE: GTP PYROPHOSPHOKINASE YJBM;
☐	407:	4ig8-A	5.8	5.7	131	338	11 PDB	MOLECULE: 2'-5'-OLIGOADENYLATE SYNTHASE 1;
☐	408:	3nyb-A	5.8	8.5	131	323	6 PDB	MOLECULE: POLY(A) RNA POLYMERASE PROTEIN 2;
☐	409:	5dec-A	5.8	4.9	124	188	8 PDB	MOLECULE: GTP PYROPHOSPHOKINASE YJBM;
☐	410:	4ud5-A	5.8	8.4	130	338	8 PDB	MOLECULE: POLY(A) RNA POLYMERASE PROTEIN CID1;
☐	411:	4ud5-B	5.8	8.0	127	342	7 PDB	MOLECULE: POLY(A) RNA POLYMERASE PROTEIN CID1;
☐	412:	4k4g-E	5.8	5.2	116	323	9 PDB	MOLECULE: DNA POLYMERASE LAMBDA;
☐	413:	4m04-A	5.8	5.4	118	327	9 PDB	MOLECULE: DNA-DIRECTED DNA/RNA POLYMERASE MU;
☐	414:	5ded-C	5.8	5.0	127	194	8 PDB	MOLECULE: GTP PYROPHOSPHOKINASE YJBM;
☐	415:	4fhp-A	5.7	8.9	135	323	6 PDB	MOLECULE: POLY(A) RNA POLYMERASE PROTEIN CID1;
☐	416:	5bpc-A	5.7	5.2	116	313	10 PDB	MOLECULE: DNA POLYMERASE BETA;
☐	417:	4m9n-A	5.7	5.7	124	300	10 PDB	MOLECULE: DNA POLYMERASE BETA;

418:	5ded-B	5.7	4.2	122	191	8	PDB	MOLECULE: GTP PYROPHOSPHOKINASE YJBM;
419:	4qzi-A	5.7	4.6	122	336	7	PDB	MOLECULE: DNA NUCLEOTIDYLEXOTRANSFERASE;
420:	3hiy-B	5.6	8.4	128	382	5	PDB	MOLECULE: MINOR EDITOSOME-ASSOCIATED TUTASE;
421:	4nkt-A	5.6	8.9	123	319	7	PDB	MOLECULE: POLY(A) RNA POLYMERASE PROTEIN CID1;
422:	4nku-A	5.6	9.0	130	320	8	PDB	MOLECULE: POLY(A) RNA POLYMERASE PROTEIN CID1;
423:	3wfp-H	5.6	4.4	127	411	12	PDB	MOLECULE: POLY A POLYMERASE;
424:	4e7x-D	5.6	8.3	127	320	6	PDB	MOLECULE: POLY(A) RNA POLYMERASE PROTEIN CID1;
425:	5ded-H	5.5	4.9	124	194	7	PDB	MOLECULE: GTP PYROPHOSPHOKINASE YJBM;
426:	4e8f-A	5.5	8.9	130	327	6	PDB	MOLECULE: POLY(A) RNA POLYMERASE PROTEIN CID1;
427:	4e7x-A	5.5	8.8	126	319	6	PDB	MOLECULE: POLY(A) RNA POLYMERASE PROTEIN CID1;
428:	4e80-B	5.5	8.8	121	311	7	PDB	MOLECULE: POLY(A) RNA POLYMERASE PROTEIN CID1;
429:	4lzd-A	5.5	4.8	123	335	11	PDB	MOLECULE: DNA-DIRECTED DNA/RNA POLYMERASE MU;
430:	3wfp-B	5.5	4.4	127	450	12	PDB	MOLECULE: POLY A POLYMERASE;
431:	4rwo-A	5.4	5.7	133	347	14	PDB	MOLECULE: RNA (5'-
432:	5f2v-P	5.4	5.0	118	183	8	PDB	MOLECULE: GTP PYROPHOSPHOKINASE YJBM;
433:	4ud4-B	5.4	7.6	124	317	6	PDB	MOLECULE: POLY(A) RNA POLYMERASE PROTEIN CID1;
434:	4ep7-A	5.4	8.8	121	319	8	PDB	MOLECULE: POLY(A) RNA POLYMERASE PROTEIN CID1;
435:	2gws-A	5.4	4.3	124	327	7	PDB	MOLECULE: 5'-D(*CP*GP*GP*CP*AP*GP*CP*GP*CP*AP*C)-3';
436:	2gws-E	5.4	4.3	124	326	7	PDB	MOLECULE: 5'-D(*CP*GP*GP*CP*AP*GP*CP*GP*CP*AP*C)-3';
437:	1rzt-I	5.4	4.3	123	326	9	PDB	MOLECULE: DNA POLYMERASE LAMBDA;
438:	3erc-C	5.4	5.0	163	445	7	PDB	MOLECULE: CAP-SPECIFIC MRNA (NUCLEOSIDE-2'-O-)-
439:	3erc-D	5.4	5.0	162	445	7	PDB	MOLECULE: CAP-SPECIFIC MRNA (NUCLEOSIDE-2'-O-)-
440:	4xq8-A	5.4	5.2	128	244	9	PDB	MOLECULE: DNA POLYMERASE LAMBDA;
441:	5cj7-B	5.4	5.2	128	243	8	PDB	MOLECULE: DNA POLYMERASE LAMBDA;
442:	5chg-B	5.4	4.8	127	243	8	PDB	MOLECULE: DNA POLYMERASE LAMBDA;
443:	4xrh-A	5.4	5.3	128	243	8	PDB	MOLECULE: DNA POLYMERASE LAMBDA;
444:	5dee-T	5.4	5.0	126	195	10	PDB	MOLECULE: GTP PYROPHOSPHOKINASE YJBM;
445:	5f2v-T	5.4	5.1	127	195	9	PDB	MOLECULE: GTP PYROPHOSPHOKINASE YJBM;
446:	5dec-C	5.4	4.9	118	183	8	PDB	MOLECULE: GTP PYROPHOSPHOKINASE YJBM;
447:	3wfp-F	5.4	4.4	126	411	13	PDB	MOLECULE: POLY A POLYMERASE;
448:	4xq7-A	5.3	5.0	134	334	10	PDB	MOLECULE: 2'-5'-OLIGOADENYLATE SYNTHASE-LIKE PROTEIN;
449:	5dec-D	5.3	4.4	123	189	9	PDB	MOLECULE: GTP PYROPHOSPHOKINASE YJBM;
450:	4at9-A	5.3	9.1	139	332	13	PDB	MOLECULE: INTERLEUKIN ENHANCER-BINDING FACTOR 2;
451:	5a2z-B	5.3	9.1	140	462	11	PDB	MOLECULE: MITOCHONDRIAL PROTEIN;
452:	5dee-V	5.3	4.9	116	180	8	PDB	MOLECULE: GTP PYROPHOSPHOKINASE YJBM;
453:	5f2v-Q	5.3	4.8	114	179	8	PDB	MOLECULE: GTP PYROPHOSPHOKINASE YJBM;
454:	4ep7-B	5.3	8.8	123	315	6	PDB	MOLECULE: POLY(A) RNA POLYMERASE PROTEIN CID1;
455:	4z6c-A	5.3	4.6	123	325	10	PDB	MOLECULE: DNA (5'-D(*CP*CP*GP*AP*CP*GP*TP*CP*GP*CP*AP*TP*CP
456:	2ga9-D	5.3	5.0	162	445	7	PDB	MOLECULE: CAP-SPECIFIC MRNA (NUCLEOSIDE-2'-O-)-METHYLTRANSF
457:	5dee-R	5.3	5.1	119	184	8	PDB	MOLECULE: GTP PYROPHOSPHOKINASE YJBM;
458:	5f2v-R	5.3	5.1	119	184	8	PDB	MOLECULE: GTP PYROPHOSPHOKINASE YJBM;
459:	5f2v-Y	5.3	5.4	119	182	8	PDB	MOLECULE: GTP PYROPHOSPHOKINASE YJBM;
460:	5dee-P	5.3	5.4	120	183	8	PDB	MOLECULE: GTP PYROPHOSPHOKINASE YJBM;
461:	5f2v-V	5.3	5.1	117	180	8	PDB	MOLECULE: GTP PYROPHOSPHOKINASE YJBM;
462:	4yd1-A	5.3	4.2	117	333	9	PDB	MOLECULE: DNA-DIRECTED DNA/RNA POLYMERASE MU;
463:	5cj7-A	5.3	5.5	129	246	8	PDB	MOLECULE: DNA POLYMERASE LAMBDA;
464:	5cp2-A	5.3	5.2	128	240	8	PDB	MOLECULE: DNA POLYMERASE LAMBDA;
465:	5ca7-A	5.3	5.2	128	243	8	PDB	MOLECULE: DNA POLYMERASE LAMBDA;
466:	4k4i-M	5.3	5.3	127	329	9	PDB	MOLECULE: DNA POLYMERASE LAMBDA;
467:	5dec-B	5.3	5.0	126	191	9	PDB	MOLECULE: GTP PYROPHOSPHOKINASE YJBM;
468:	5dee-S	5.3	5.1	118	182	8	PDB	MOLECULE: GTP PYROPHOSPHOKINASE YJBM;
469:	5dee-Q	5.3	4.9	115	179	9	PDB	MOLECULE: GTP PYROPHOSPHOKINASE YJBM;
470:	5f2v-Z	5.3	4.9	115	179	9	PDB	MOLECULE: GTP PYROPHOSPHOKINASE YJBM;
471:	3er9-B	5.3	5.0	162	445	7	PDB	MOLECULE: CAP-SPECIFIC MRNA (NUCLEOSIDE-2'-O-)-METHYLTRANSF
472:	3wfp-C	5.3	4.4	131	419	12	PDB	MOLECULE: POLY A POLYMERASE;
473:	5hc9-B	5.3	3.9	127	410	11	PDB	MOLECULE: TRNA NUCLEOTIDYL TRANSFERASE-RELATED PROTEIN;
474:	4rwn-A	5.2	5.7	132	349	14	PDB	MOLECULE: 2'-5'-OLIGOADENYLATE SYNTHASE 1;
475:	4at8-A	5.2	7.0	134	333	10	PDB	MOLECULE: INTERLEUKIN ENHANCER-BINDING FACTOR 2;
476:	4at7-A	5.2	9.2	138	334	13	PDB	MOLECULE: INTERLEUKIN ENHANCER-BINDING FACTOR 2;
477:	5dee-O	5.2	4.9	119	184	8	PDB	MOLECULE: GTP PYROPHOSPHOKINASE YJBM;
478:	5a30-B	5.2	9.2	139	464	11	PDB	MOLECULE: MITOCHONDRIAL PROTEIN;
479:	5f2v-S	5.2	4.9	117	182	9	PDB	MOLECULE: GTP PYROPHOSPHOKINASE YJBM;
480:	5dee-X	5.2	4.8	114	181	8	PDB	MOLECULE: GTP PYROPHOSPHOKINASE YJBM;

481:	4e7x-C	5.2	8.3	119	316	6 PDB	MOLECULE: POLY(A) RNA POLYMERASE PROTEIN CID1;
482:	4e80-C	5.2	8.4	120	330	7 PDB	MOLECULE: POLY(A) RNA POLYMERASE PROTEIN CID1;
483:	3mdc-A	5.2	5.4	123	312	8 PDB	MOLECULE: DNA POLYMERASE LAMBDA;
484:	3upq-A	5.2	5.1	119	308	8 PDB	MOLECULE: DNA POLYMERASE LAMBDA;
485:	1xsl-E	5.2	4.5	124	325	7 PDB	MOLECULE: 5'-D(*CP*GP*GP*CP*AP*GP*CP*GP*CP*AP*C)-3';
486:	5dee-Y	5.2	5.3	119	182	8 PDB	MOLECULE: GTP PYROPHOSPHOKINASE YJBM;
487:	2gaf-D	5.2	5.1	162	447	7 PDB	MOLECULE: CAP-SPECIFIC MRNA (NUCLEOSIDE-2'-O-)-
488:	3owg-A	5.2	4.9	161	457	7 PDB	MOLECULE: POLY(A) POLYMERASE CATALYTIC SUBUNIT;
489:	3er8-C	5.2	4.9	161	445	7 PDB	MOLECULE: CAP-SPECIFIC MRNA (NUCLEOSIDE-2'-O-)-METHYLTRANSF
490:	5iio-M	5.2	5.3	128	326	8 PDB	MOLECULE: DNA (5'-D(*CP*GP*GP*CP*(8OG)P*GP*TP*AP*CP*TP*G)-3
491:	5iio-I	5.2	4.2	124	326	7 PDB	MOLECULE: DNA (5'-D(*CP*GP*GP*CP*(8OG)P*GP*TP*AP*CP*TP*G)-3
492:	5iij-A	5.2	5.5	126	314	8 PDB	MOLECULE: DNA POLYMERASE LAMBDA;
493:	5cwr-B	5.2	5.5	127	323	8 PDB	MOLECULE: DNA POLYMERASE LAMBDA;
494:	5f2v-U	5.2	5.1	122	186	8 PDB	MOLECULE: GTP PYROPHOSPHOKINASE YJBM;
495:	5dee-U	5.2	5.1	122	186	7 PDB	MOLECULE: GTP PYROPHOSPHOKINASE YJBM;
496:	5f2v-X	5.2	5.1	117	181	8 PDB	MOLECULE: GTP PYROPHOSPHOKINASE YJBM;
497:	3oux-A	5.2	4.8	116	503	9 PDB	MOLECULE: CATENIN BETA-1;
498:	5ded-D	5.1	4.5	125	188	8 PDB	MOLECULE: GTP PYROPHOSPHOKINASE YJBM;
499:	2be3-A	5.1	5.0	126	203	5 PDB	MOLECULE: GTP PYROPHOSPHOKINASE;
500:	5ded-F	5.1	4.3	123	192	8 PDB	MOLECULE: GTP PYROPHOSPHOKINASE YJBM;
501:	4atb-A	5.1	9.5	138	334	13 PDB	MOLECULE: INTERLEUKIN ENHANCER-BINDING FACTOR 2;
502:	4atb-C	5.1	9.1	139	331	13 PDB	MOLECULE: INTERLEUKIN ENHANCER-BINDING FACTOR 2;
503:	5a2v-A	5.1	8.6	141	470	12 PDB	MOLECULE: MITOCHONDRIAL PROTEIN;
504:	2m2u-A	5.1	4.3	104	174	8 PDB	MOLECULE: REPAIR DNA POLYMERASE X;
505:	5cwr-A	5.1	5.0	124	325	7 PDB	MOLECULE: DNA POLYMERASE LAMBDA;
506:	3hx0-K	5.1	5.5	129	327	8 PDB	MOLECULE: DNA POLYMERASE LAMBDA;
507:	2gws-I	5.1	4.3	124	326	9 PDB	MOLECULE: 5'-D(*CP*GP*GP*CP*AP*GP*CP*GP*CP*AP*C)-3';
508:	3er8-D	5.1	4.9	162	445	7 PDB	MOLECULE: CAP-SPECIFIC MRNA (NUCLEOSIDE-2'-O-)-METHYLTRANSF
509:	3hx0-P	5.1	5.4	128	327	8 PDB	MOLECULE: DNA POLYMERASE LAMBDA;
510:	3owg-B	5.1	5.0	161	457	7 PDB	MOLECULE: POLY(A) POLYMERASE CATALYTIC SUBUNIT;
511:	4qzg-A	5.1	4.7	124	340	7 PDB	MOLECULE: DNA NUCLEOTIDYLEXOTRANSFERASE;
512:	4k4g-M	5.1	4.4	128	328	8 PDB	MOLECULE: DNA POLYMERASE LAMBDA;
513:	4k4h-M	5.1	5.3	127	325	9 PDB	MOLECULE: DNA POLYMERASE LAMBDA;
514:	5iio-E	5.1	4.3	123	325	8 PDB	MOLECULE: DNA (5'-D(*CP*GP*GP*CP*(8OG)P*GP*TP*AP*CP*TP*G)-3
515:	4k4h-E	5.1	5.2	126	326	8 PDB	MOLECULE: DNA POLYMERASE LAMBDA;
516:	5iii-A	5.1	5.2	125	314	9 PDB	MOLECULE: DNA POLYMERASE LAMBDA;
517:	5dkw-B	5.1	5.5	129	319	9 PDB	MOLECULE: DNA POLYMERASE LAMBDA;
518:	4xa5-A	5.1	5.2	125	314	9 PDB	MOLECULE: DNA POLYMERASE LAMBDA;
519:	5ded-E	5.1	4.3	122	190	8 PDB	MOLECULE: GTP PYROPHOSPHOKINASE YJBM;
520:	3l9d-B	5.1	5.0	121	193	7 PDB	MOLECULE: PUTATIVE GTP PYROPHOSPHOKINASE;
521:	1g3j-A	5.1	4.8	117	522	9 PDB	MOLECULE: BETA-CATENIN ARMADILLO REPEAT REGION;
522:	1v18-A	5.1	4.8	117	507	9 PDB	MOLECULE: BETA-CATENIN;
523:	1t08-A	5.1	4.8	116	510	9 PDB	MOLECULE: BETA-CATENIN;
524:	4rwp-A	5.0	5.3	136	346	13 PDB	MOLECULE: 2'-5'-OLIGOADENYLATE SYNTHASE 1;
525:	3l9d-A	5.0	5.0	124	198	6 PDB	MOLECULE: PUTATIVE GTP PYROPHOSPHOKINASE;
526:	5f2v-W	5.0	5.1	120	187	8 PDB	MOLECULE: GTP PYROPHOSPHOKINASE YJBM;
527:	3hiy-A	5.0	8.5	123	382	6 PDB	MOLECULE: MINOR EDITOSOME-ASSOCIATED TUTASE;
528:	4e7x-B	5.0	8.9	121	312	6 PDB	MOLECULE: POLY(A) RNA POLYMERASE PROTEIN CID1;
529:	4nkt-B	5.0	9.0	117	308	6 PDB	MOLECULE: POLY(A) RNA POLYMERASE PROTEIN CID1;
530:	4k96-A	5.0	8.5	133	355	6 PDB	MOLECULE: CYCLIC GMP-AMP SYNTHASE;
531:	2pfo-A	5.0	5.1	120	323	9 PDB	MOLECULE: DNA POLYMERASE LAMBDA;
532:	1rzt-E	5.0	4.3	124	326	9 PDB	MOLECULE: DNA POLYMERASE LAMBDA;
533:	5f2v-O	5.0	5.1	120	184	8 PDB	MOLECULE: GTP PYROPHOSPHOKINASE YJBM;
534:	5d46-A	5.0	4.6	122	346	7 PDB	MOLECULE: TERMINAL DEOXYNUCLEOTIDYLTRANSFERASE;
535:	5iio-A	5.0	5.3	128	327	9 PDB	MOLECULE: DNA (5'-D(*CP*GP*GP*CP*(8OG)P*GP*TP*AP*CP*TP*G)-3
536:	4x4t-C	5.0	3.7	124	436	9 PDB	MOLECULE: CCA-ADDING ENZYME;
537:	3ouy-A	5.0	3.5	124	441	8 PDB	MOLECULE: CCA-ADDING ENZYME;
538:	4wc1-A	5.0	3.9	116	364	11 PDB	MOLECULE: POLY A POLYMERASE;
539:	3ifq-A	5.0	4.8	117	546	9 PDB	MOLECULE: PLAKOGLOBIN;
540:	4at8-C	4.9	7.1	134	332	13 PDB	MOLECULE: INTERLEUKIN ENHANCER-BINDING FACTOR 2;
541:	5dee-Z	4.9	4.8	114	179	8 PDB	MOLECULE: GTP PYROPHOSPHOKINASE YJBM;
542:	3hj1-A	4.9	8.5	123	384	6 PDB	MOLECULE: MINOR EDITOSOME-ASSOCIATED TUTASE;
543:	4k99-A	4.9	9.1	134	354	5 PDB	MOLECULE: CYCLIC GMP-AMP SYNTHASE;

544:	5dee-W	4.9	5.4	122	187	7	PDB	MOLECULE: GTP PYROPHOSPHOKINASE YJBM;
545:	4i27-A	4.9	4.6	121	361	8	PDB	MOLECULE: DNA NUCLEOTIDYLEXOTRANSFERASE;
546:	3wfr-F	4.9	4.3	128	439	13	PDB	MOLECULE: RNA (74-MER);
547:	1qz7-A	4.9	4.7	116	524	9	PDB	MOLECULE: BETA-CATENIN;
548:	1th1-B	4.9	4.8	118	513	8	PDB	MOLECULE: BETA-CATENIN;
549:	2ii2-A	4.8	7.7	91	304	9	PDB	MOLECULE: ALPHA-11 GIARDIN;
550:	5a2w-B	4.8	9.2	139	467	11	PDB	MOLECULE: MITOCHONDRIAL PROTEIN;
551:	4at8-B	4.8	9.2	144	316	8	PDB	MOLECULE: INTERLEUKIN ENHANCER-BINDING FACTOR 2;
552:	4k97-A	4.8	8.7	136	351	4	PDB	MOLECULE: CYCLIC GMP-AMP SYNTHASE;
553:	4k98-A	4.8	7.0	134	354	4	PDB	MOLECULE: CYCLIC GMP-AMP SYNTHASE;
554:	3au2-A	4.8	4.0	110	575	7	PDB	MOLECULE: DNA POLYMERASE BETA FAMILY (X FAMILY);
555:	4lzg-A	4.8	5.4	117	325	9	PDB	MOLECULE: DNA-DIRECTED DNA/RNA POLYMERASE MU;
556:	3b0x-A	4.8	4.2	109	575	7	PDB	MOLECULE: DNA POLYMERASE BETA FAMILY (X FAMILY);
557:	3b0y-A	4.8	4.2	109	575	8	PDB	MOLECULE: DNA POLYMERASE BETA FAMILY (X FAMILY);
558:	1jqr-A	4.8	5.9	108	174	8	PDB	MOLECULE: DNA POLYMERASE BETA-LIKE;
559:	3wfr-E	4.8	4.3	127	439	12	PDB	MOLECULE: RNA (74-MER);
560:	3wfq-G	4.8	4.3	127	439	11	PDB	MOLECULE: RNA (73-MER);
561:	1luj-A	4.8	4.8	116	501	9	PDB	MOLECULE: CATENIN BETA-1;
562:	3wfr-H	4.7	4.1	129	463	11	PDB	MOLECULE: RNA (74-MER);
563:	1jaj-A	4.7	4.6	110	174	7	PDB	MOLECULE: DNA POLYMERASE BETA-LIKE PROTEIN;
564:	2z6g-A	4.7	4.6	116	550	7	PDB	MOLECULE: B-CATENIN;
565:	5a2y-B	4.7	9.3	141	464	11	PDB	MOLECULE: MITOCHONDRIAL PROTEIN;
566:	5a2x-A	4.7	9.0	141	468	11	PDB	MOLECULE: MITOCHONDRIAL PROTEIN;
567:	5a2x-B	4.7	9.0	139	467	11	PDB	MOLECULE: MITOCHONDRIAL PROTEIN;
568:	4e80-D	4.7	9.1	120	324	6	PDB	MOLECULE: POLY(A) RNA POLYMERASE PROTEIN CID1;
569:	4nku-B	4.7	9.0	117	310	6	PDB	MOLECULE: POLY(A) RNA POLYMERASE PROTEIN CID1;
570:	5a2v-B	4.7	8.3	148	472	11	PDB	MOLECULE: MITOCHONDRIAL PROTEIN;
571:	4i2i-A	4.7	5.2	123	338	8	PDB	MOLECULE: DNA NUCLEOTIDYLEXOTRANSFERASE;
572:	2m2v-A	4.7	5.6	114	174	8	PDB	MOLECULE: REPAIR DNA POLYMERASE X;
573:	3wfq-E	4.7	4.2	126	439	12	PDB	MOLECULE: RNA (73-MER);
574:	1q78-A	4.6	6.1	136	470	10	PDB	MOLECULE: POLY(A) POLYMERASE ALPHA;
575:	5a2y-A	4.6	9.2	141	468	11	PDB	MOLECULE: MITOCHONDRIAL PROTEIN;
576:	3hj4-A	4.6	8.7	126	381	6	PDB	MOLECULE: MINOR EDITOSOME-ASSOCIATED TUTASE;
577:	3hj4-B	4.6	8.4	123	382	7	PDB	MOLECULE: MINOR EDITOSOME-ASSOCIATED TUTASE;
578:	4e8f-B	4.6	8.8	125	319	6	PDB	MOLECULE: POLY(A) RNA POLYMERASE PROTEIN CID1;
579:	4atb-B	4.6	9.4	144	319	8	PDB	MOLECULE: INTERLEUKIN ENHANCER-BINDING FACTOR 2;
580:	3v7l-A	4.6	5.1	122	325	10	PDB	MOLECULE: DNA POLYMERASE BETA;
581:	3auo-A	4.6	4.3	107	572	8	PDB	MOLECULE: DNA POLYMERASE BETA FAMILY (X FAMILY);
582:	2ihm-B	4.6	4.3	115	336	8	PDB	MOLECULE: DNA POLYMERASE MU;
583:	4k8v-A	4.6	7.5	141	362	6	PDB	MOLECULE: CYCLIC GMP-AMP SYNTHASE;
584:	1jpw-A	4.6	4.7	118	502	8	PDB	MOLECULE: BETA-CATENIN;
585:	4at8-D	4.5	7.2	142	316	8	PDB	MOLECULE: INTERLEUKIN ENHANCER-BINDING FACTOR 2;
586:	2w9m-A	4.5	7.0	120	541	13	PDB	MOLECULE: POLYMERASE X;
587:	3auo-B	4.5	4.2	104	572	9	PDB	MOLECULE: DNA POLYMERASE BETA FAMILY (X FAMILY);
588:	2m2t-A	4.5	4.2	102	174	8	PDB	MOLECULE: REPAIR DNA POLYMERASE X;
589:	2m2w-A	4.5	5.5	111	174	7	PDB	MOLECULE: REPAIR DNA POLYMERASE X;
590:	4wc7-A	4.5	4.0	115	367	10	PDB	MOLECULE: POLY A POLYMERASE;
591:	1vj7-A	4.4	5.2	112	326	12	PDB	MOLECULE: BIFUNCTIONAL RELA/SPOT;
592:	1zel-A	4.4	4.2	136	293	10	PDB	MOLECULE: HYPOTHETICAL PROTEIN RV2827C;
593:	5cwu-A	4.3	4.6	98	298	11	PDB	MOLECULE: NUCLEOPORIN NUP188;
594:	1dk5-A	4.3	12.2	92	316	7	PDB	MOLECULE: ANNEXIN 24(CA32);
595:	4k96-B	4.3	7.0	128	358	8	PDB	MOLECULE: CYCLIC GMP-AMP SYNTHASE;
596:	1r8b-A	4.3	4.2	114	437	9	PDB	MOLECULE: TRNA NUCLEOTIDYLTRANSFERASE;
597:	3au6-A	4.3	7.8	132	562	8	PDB	MOLECULE: DNA POLYMERASE BETA FAMILY (X FAMILY);
598:	4wc6-A	4.3	4.1	115	367	9	PDB	MOLECULE: POLY A POLYMERASE;
599:	3c2g-A	4.2	4.5	129	618	9	PDB	MOLECULE: SYS-1 PROTEIN;
600:	4x4p-G	4.2	4.1	128	428	7	PDB	MOLECULE: CCA-ADDING ENZYME;
601:	4wbz-B	4.2	4.0	112	350	10	PDB	MOLECULE: POLY A POLYMERASE;
602:	4wc0-B	4.2	4.0	112	350	9	PDB	MOLECULE: POLY A POLYMERASE;
603:	4woy-A	4.1	5.4	133	329	13	PDB	MOLECULE: MITOCHONDRIAL DYNAMICS PROTEIN MID49;
604:	3hj1-B	4.1	8.7	126	387	6	PDB	MOLECULE: MINOR EDITOSOME-ASSOCIATED TUTASE;
605:	4x4p-C	4.1	7.1	119	434	8	PDB	MOLECULE: CCA-ADDING ENZYME;
606:	3ups-A	4.1	4.1	87	108	10	PDB	MOLECULE: IOJAP-LIKE PROTEIN;

607:	5hzd-A	4.1	7.3	132	476	8	PDB	MOLECULE: 3' TERMINAL URIDYLYL TRANSFERASE;
608:	4r10-A	4.1	4.9	118	537	9	PDB	MOLECULE: PROTEIN HUMPBACK-2;
609:	4lt6-A	4.0	6.2	137	467	7	PDB	MOLECULE: POLY(A) POLYMERASE GAMMA;
610:	4r0z-A	4.0	5.0	94	557	9	PDB	MOLECULE: PROTEIN HUMPBACK-2;
611:	4txz-A	4.0	8.7	137	370	9	PDB	MOLECULE: CYCLIC AMP-GMP SYNTHASE;
612:	5a2z-A	3.9	9.0	142	466	11	PDB	MOLECULE: MITOCHONDRIAL PROTEIN;
613:	4r11-E	3.9	4.8	120	508	9	PDB	MOLECULE: PROTEIN HUMPBACK-2;
614:	4k9b-A	3.8	7.5	131	355	8	PDB	MOLECULE: CYCLIC GMP-AMP SYNTHASE;
615:	4txy-B	3.8	8.6	137	364	8	PDB	MOLECULE: CYCLIC AMP-GMP SYNTHASE;
616:	4x4n-C	3.8	6.8	124	436	8	PDB	MOLECULE: CCA-ADDING ENZYME;
617:	1n44-A	3.8	9.6	94	318	12	PDB	MOLECULE: ANNEXIN V;
618:	4evp-A	3.8	4.8	116	489	9	PDB	MOLECULE: CATENIN BETA-1;
619:	4ev9-A	3.8	4.8	116	492	9	PDB	MOLECULE: CATENIN BETA-1;
620:	4evt-A	3.8	4.9	116	490	9	PDB	MOLECULE: CATENIN BETA-1;
621:	4eva-A	3.8	4.8	116	491	9	PDB	MOLECULE: CATENIN BETA-1;
622:	4ev8-A	3.8	4.9	116	492	9	PDB	MOLECULE: CATENIN BETA-1;
623:	4eva-C	3.8	4.8	116	488	9	PDB	MOLECULE: CATENIN BETA-1;
624:	1g3j-C	3.8	5.3	104	439	10	PDB	MOLECULE: BETA-CATENIN ARMADILLO REPEAT REGION;
625:	4amq-A	3.7	6.8	115	341	7	PDB	MOLECULE: L544;
626:	3ovs-B	3.7	6.4	120	437	8	PDB	MOLECULE: CCA-ADDING ENZYME;
627:	4x4u-F	3.7	6.7	119	436	8	PDB	MOLECULE: CCA-ADDING ENZYME;
628:	4x4u-A	3.7	6.8	123	435	8	PDB	MOLECULE: CCA-ADDING ENZYME;
629:	1kdh-A	3.7	4.5	130	356	8	PDB	MOLECULE: TERMINAL DEOXYNUCLEOTIDYLTRANSFERASE SHORT
630:	5jnb-A	3.6	4.3	129	329	8	PDB	MOLECULE: POLY(A) RNA POLYMERASE GLD-2;
631:	4xj3-A	3.6	7.1	129	369	5	PDB	MOLECULE: CYCLIC AMP-GMP SYNTHASE;
632:	4wcw-C	3.6	3.8	91	117	9	PDB	MOLECULE: RIBOSOMAL SILENCING FACTOR RSFS;
633:	1px5-B	3.6	9.0	131	341	11	PDB	MOLECULE: 2'-5'-OLIGOADENYLATE SYNTHETASE 1;
634:	4x4n-F	3.6	6.7	118	437	8	PDB	MOLECULE: CCA-ADDING ENZYME;
635:	4x4s-C	3.6	6.8	123	437	7	PDB	MOLECULE: CCA-ADDING ENZYME;
636:	4yjl-C	3.6	3.7	87	338	3	PDB	MOLECULE: ADENOMATOUS POLYPOSIS COLI PROTEIN;
637:	5eog-F	3.5	5.6	115	330	7	PDB	MOLECULE: PROTEIN MAB-21-LIKE 1;
638:	1miv-A	3.5	4.0	117	395	6	PDB	MOLECULE: TRNA CCA-ADDING ENZYME;
639:	2q0c-B	3.5	8.0	117	311	10	PDB	MOLECULE: RNA URIDYLYL TRANSFERASE;
640:	2pbe-A	3.5	8.8	118	263	8	PDB	MOLECULE: AMINOGLYCOSIDE 6-ADENYLYLTRANSFERASE;
641:	4s3n-A	3.5	5.3	126	343	9	PDB	MOLECULE: 2'-5'-OLIGOADENYLATE SYNTHASE 3;
642:	3nmx-B	3.5	5.2	128	325	5	PDB	MOLECULE: APC VARIANT PROTEIN;
643:	3nmz-A	3.5	4.9	122	406	7	PDB	MOLECULE: APC VARIANT PROTEIN;
644:	4atb-D	3.4	7.3	137	306	9	PDB	MOLECULE: INTERLEUKIN ENHANCER-BINDING FACTOR 2;
645:	4x4u-E	3.4	6.8	124	437	8	PDB	MOLECULE: CCA-ADDING ENZYME;
646:	4x4t-E	3.4	6.9	123	437	7	PDB	MOLECULE: CCA-ADDING ENZYME;
647:	4x4n-A	3.4	6.8	117	436	8	PDB	MOLECULE: CCA-ADDING ENZYME;
648:	4x4o-C	3.4	6.9	118	442	8	PDB	MOLECULE: CCA-ADDING ENZYME;
649:	4x4p-A	3.4	7.1	119	437	8	PDB	MOLECULE: CCA-ADDING ENZYME;
650:	4evf-A	3.3	4.2	106	294	4	PDB	MOLECULE: GIARDIN SUBUNIT ALPHA-1;
651:	4txa-A	3.3	5.2	76	367	12	PDB	MOLECULE: ROQUIN-1;
652:	4at9-B	3.3	7.9	129	308	10	PDB	MOLECULE: INTERLEUKIN ENHANCER-BINDING FACTOR 2;
653:	4r11-C	3.3	4.9	118	536	9	PDB	MOLECULE: PROTEIN HUMPBACK-2;
654:	5jnb-C	3.2	8.5	120	289	9	PDB	MOLECULE: POLY(A) RNA POLYMERASE GLD-2;
655:	4ams-A	3.2	5.3	114	367	5	PDB	MOLECULE: MG662;
656:	1axn-A	3.2	5.0	91	323	5	PDB	MOLECULE: ANNEXIN III;
657:	2nrk-A	3.2	6.3	93	165	14	PDB	MOLECULE: HYPOTHETICAL PROTEIN GRPB;
658:	4ud4-A	3.2	9.0	129	324	6	PDB	MOLECULE: POLY(A) RNA POLYMERASE PROTEIN CID1;
659:	4xj6-A	3.1	7.3	136	370	7	PDB	MOLECULE: VC0179-LIKE PROTEIN;
660:	2zoc-A	3.1	5.4	88	319	11	PDB	MOLECULE: ANNEXIN A4;
661:	4z31-B	3.1	3.7	67	295	10	PDB	MOLECULE: ROQUIN-2;
662:	4rwq-B	3.1	5.1	126	346	9	PDB	MOLECULE: 2'-5'-OLIGOADENYLATE SYNTHASE 1;
663:	4x4v-C	3.1	6.9	118	431	8	PDB	MOLECULE: CCA-ADDING ENZYME;
664:	3wfq-H	3.0	3.6	113	441	10	PDB	MOLECULE: RNA (73-MER);
665:	3m62-A	3.0	4.6	146	955	7	PDB	MOLECULE: UBIQUITIN CONJUGATION FACTOR E4;
666:	2h0k-A	3.0	4.5	100	318	11	PDB	MOLECULE: ANNEXIN A5;
667:	1ycn-A	3.0	12.2	93	312	11	PDB	MOLECULE: PUTATIVE CA2+-DEPENDENT MEMBRANE-BINDING PROTEIN
668:	3jyy-B	3.0	4.7	114	266	4	PDB	MOLECULE: LINCOSAMIDE NUCLEOTIDYLTRANSFERASE;
669:	4rwq-A	3.0	5.1	125	346	10	PDB	MOLECULE: 2'-5'-OLIGOADENYLATE SYNTHASE 1;

670:	3zym-C	3.0	10.9	104	280	9	PDB	MOLECULE: PHOSPHATIDYLINOSITOL-BINDING CLATHRIN ASSEMBLY PR
671:	1y1u-A	3.0	5.9	85	544	14	PDB	MOLECULE: SIGNAL TRANSDUCER AND ACTIVATOR OF TRANSCRIPTION
672:	5tgq-A	2.9	4.5	99	226	2	PDB	MOLECULE: R.SWAI PROTEIN;
673:	4x4w-A	2.9	5.0	106	398	11	PDB	MOLECULE: CCA TRNA NUCLEOTIDYLTRANSFERASE 1, MITOCHONDRIAL;
674:	4ebj-A	2.9	4.1	98	258	7	PDB	MOLECULE: AMINOGLYCOSIDE NUCLEOTIDYLTRANSFERASE;
675:	1aei-A	2.9	4.5	101	315	13	PDB	MOLECULE: ANNEXIN XII;
676:	2zw6-A	2.9	3.4	69	297	13	PDB	MOLECULE: BLEOMYCIN ACETYLTRANSFERASE;
677:	3tjz-B	2.9	3.2	68	264	9	PDB	MOLECULE: ADP-RIBOSYLATION FACTOR 1;
678:	3ov7-B	2.9	8.2	119	437	8	PDB	MOLECULE: CCA-ADDING ENZYME;
679:	2ia7-A	2.9	4.1	85	111	12	PDB	MOLECULE: TAIL LYSOZYME, PUTATIVE;
680:	3hpv-A	2.9	5.3	93	297	3	PDB	MOLECULE: CATECHOL 2,3-DIOXYGENASE;
681:	2iic-A	2.9	9.3	95	302	7	PDB	MOLECULE: ALPHA-11 GIARDIN;
682:	2iic-B	2.9	9.3	95	302	7	PDB	MOLECULE: ALPHA-11 GIARDIN;
683:	2iic-C	2.9	9.3	96	302	7	PDB	MOLECULE: ALPHA-11 GIARDIN;
684:	2w3c-A	2.9	4.1	114	551	7	PDB	MOLECULE: GENERAL VESICULAR TRANSPORT FACTOR P115;
685:	2iic-D	2.9	9.3	96	302	7	PDB	MOLECULE: ALPHA-11 GIARDIN;
686:	4u03-A	2.8	6.8	131	390	8	PDB	MOLECULE: CYCLIC AMP-GMP SYNTHASE;
687:	4kf8-B	2.8	4.6	101	258	11	PDB	MOLECULE: NUP188;
688:	1n00-A	2.8	12.1	87	318	11	PDB	MOLECULE: ANNEXIN GH1;
689:	3qml-D	2.8	4.4	85	273	7	PDB	MOLECULE: 78 KDA GLUCOSE-REGULATED PROTEIN HOMOLOG;
690:	3ovb-B	2.8	6.5	116	437	9	PDB	MOLECULE: CCA-ADDING ENZYME;
691:	2b51-A	2.8	8.5	127	444	9	PDB	MOLECULE: RNA EDITING COMPLEX PROTEIN MP57;
692:	4dvg-B	2.7	3.9	82	305	10	PDB	MOLECULE: RHO-LIKE SMALL GTPASE;
693:	3ovb-A	2.7	6.5	116	441	9	PDB	MOLECULE: CCA-ADDING ENZYME;
694:	3evh-A	2.7	7.0	97	353	10	PDB	MOLECULE: ALGINATE LYASE;
695:	5hc9-A	2.6	5.2	118	425	8	PDB	MOLECULE: TRNA NUCLEOTIDYL TRANSFERASE-RELATED PROTEIN;
696:	3gq2-A	2.6	5.9	106	614	9	PDB	MOLECULE: GENERAL VESICULAR TRANSPORT FACTOR P115;
697:	3hm0-A	2.6	4.3	72	126	6	PDB	MOLECULE: PROBABLE THIOESTERASE;
698:	3bg3-A	2.6	9.2	83	680	6	PDB	MOLECULE: PYRUVATE CARBOXYLASE, MITOCHONDRIAL;
699:	2pbi-A	2.6	5.9	84	415	5	PDB	MOLECULE: REGULATOR OF G-PROTEIN SIGNALING 9;
700:	4mdu-B	2.6	8.3	73	360	7	PDB	MOLECULE: ANNEXIN;
701:	3n0f-A	2.6	14.5	80	531	5	PDB	MOLECULE: ISOPRENE SYNTHASE;
702:	3p3q-B	2.6	4.4	58	253	9	PDB	MOLECULE: MMOQ;
703:	3rtr-C	2.6	3.3	58	317	7	PDB	MOLECULE: CULLIN-1;
704:	1bg1-A	2.6	6.0	83	559	7	PDB	MOLECULE: PROTEIN (TRANSCRIPTION FACTOR STAT3B);
705:	2o5a-A	2.6	4.2	82	108	5	PDB	MOLECULE: BH1328 PROTEIN;
706:	3inn-C	2.6	5.1	71	284	8	PDB	MOLECULE: PANTOTHENATE SYNTHETASE;
707:	3ebb-C	2.6	5.3	110	265	8	PDB	MOLECULE: PHOSPHOLIPASE A2-ACTIVATING PROTEIN;
708:	3pq1-A	2.5	6.9	113	355	10	PDB	MOLECULE: POLY(A) RNA POLYMERASE;
709:	1hm6-A	2.5	3.8	90	343	7	PDB	MOLECULE: ANNEXIN 1;
710:	4bwr-A	2.5	10.7	99	467	8	PDB	MOLECULE: PROTEIN CORRESPONDING TO LOCUS C5321 FROM CFT073
711:	3jui-A	2.5	4.4	72	174	3	PDB	MOLECULE: TRANSLATION INITIATION FACTOR EIF-2B SUBUNIT EPSI
712:	4l7m-A	2.5	4.0	85	261	9	PDB	MOLECULE: PUTATIVE UNCHARACTERIZED PROTEIN;
713:	5byu-A	2.5	3.7	70	128	6	PDB	MOLECULE: THIOESTERASE;
714:	1yvl-B	2.5	11.3	89	653	8	PDB	MOLECULE: SIGNAL TRANSDUCER AND ACTIVATOR OF TRANSCRIPTION
715:	2a4w-A	2.5	3.9	79	128	10	PDB	MOLECULE: MITOMYCIN-BINDING PROTEIN;
716:	5fu7-A	2.4	3.8	96	519	13	PDB	MOLECULE: CCR4-NOT TRANSCRIPTION COMPLEX SUBUNIT 1;
717:	5i6e-A	2.4	4.3	98	687	7	PDB	MOLECULE: ACETYL-COA CARBOXYLASE;
718:	3oms-A	2.4	4.0	75	129	5	PDB	MOLECULE: PHNB PROTEIN;
719:	2i52-F	2.4	4.1	75	119	9	PDB	MOLECULE: HYPOTHETICAL PROTEIN;
720:	1kw2-A	2.4	13.0	77	455	8	PDB	MOLECULE: VITAMIN D-BINDING PROTEIN;
721:	3ct8-A	2.4	4.0	72	133	7	PDB	MOLECULE: PUTATIVE GLYOXALASE;
722:	5dhh-B	2.4	3.9	57	387	7	PDB	MOLECULE: GPCR-BRIL CHIMERA;
723:	4wzs-C	2.4	3.5	59	722	7	PDB	MOLECULE: ECU11_1470 PROTEIN;
724:	3cwg-B	2.4	6.0	81	507	7	PDB	MOLECULE: SIGNAL TRANSDUCER AND ACTIVATOR OF TRANSCRIPTION
725:	3ebb-A	2.4	5.2	110	265	9	PDB	MOLECULE: PHOSPHOLIPASE A2-ACTIVATING PROTEIN;
726:	3nmw-B	2.4	5.2	99	331	6	PDB	MOLECULE: APC VARIANT PROTEIN;
727:	2q0e-A	2.3	5.1	124	331	10	PDB	MOLECULE: RNA URIDYLYL TRANSFERASE;
728:	2i4g-A	2.3	4.5	113	289	4	PDB	MOLECULE: RECEPTOR-TYPE TYROSINE-PROTEIN PHOSPHATASE BETA;
729:	2p25-A	2.3	4.1	72	126	10	PDB	MOLECULE: GLYOXALASE FAMILY PROTEIN;
730:	4o10-B	2.3	3.9	75	902	4	PDB	MOLECULE: GTP-BINDING NUCLEAR PROTEIN RAN;
731:	2kjz-A	2.3	3.5	69	122	4	PDB	MOLECULE: ATC0852;
732:	2j5c-B	2.3	4.7	69	491	3	PDB	MOLECULE: 1,8-CINEOLE SYNTHASE;

☐	733:	3a6p-A	2.3	3.3	57	1072	9	PDB	MOLECULE: EXPORTIN-5;
☐	734:	1xm9-A	2.3	3.0	62	420	11	PDB	MOLECULE: PLAKOPHILIN 1;
☐	735:	4yc7-B	2.2	6.0	117	291	5	PDB	MOLECULE: FORMIN-LIKE PROTEIN 2;
☐	736:	1z1w-A	2.2	4.8	114	780	8	PDB	MOLECULE: TRICORN PROTEASE INTERACTING FACTOR F3;
☐	737:	3j2m-A	2.2	4.4	93	211	4	PDB	MOLECULE: TAIL CONNECTOR PROTEIN GP15;
☐	738:	1s5u-E	2.2	3.8	71	136	6	PDB	MOLECULE: PROTEIN YBGC;
☐	739:	3zhp-A	2.2	4.7	91	315	12	PDB	MOLECULE: CALCIUM-BINDING PROTEIN 39-LIKE;
☐	740:	2mxz-A	2.2	3.4	74	137	7	PDB	MOLECULE: L-ALANYL-D-GLUTAMATE PEPTIDASE;
☐	741:	1gxb-D	2.2	3.8	78	345	9	PDB	MOLECULE: ANTHRANILATE PHOSPHORIBOSYLTRANSFERASE;
☐	742:	4c00-A	2.2	11.9	76	544	8	PDB	MOLECULE: TRANSLOCATION AND ASSEMBLY MODULE TAMA;
☐	743:	3jbr-A	2.2	15.5	95	1224	4	PDB	MOLECULE: VOLTAGE-DEPENDENT L-TYPE CALCIUM CHANNEL SUBUNIT
☐	744:	5i6x-A	2.2	8.8	78	544	8	PDB	MOLECULE: SODIUM-DEPENDENT SEROTONIN TRANSPORTER;
☐	745:	2wzk-A	2.2	3.2	66	369	6	PDB	MOLECULE: CULLIN-5;
☐	746:	4yay-A	2.2	3.6	56	393	7	PDB	MOLECULE: SOLUBLE CYTOCHROME B562,TYPE-1 ANGIOTENSIN II REC
☐	747:	2wm9-A	2.2	4.2	90	407	7	PDB	MOLECULE: DEDICATOR OF CYTOKINESIS PROTEIN 9;
☐	748:	4oww-A	2.2	4.9	81	463	5	PDB	MOLECULE: DNA (5'-D(P*TP*TP*TP*TP*TP*T)-3');
☐	749:	5i7n-A	2.1	4.4	100	325	9	PDB	MOLECULE: MAOC-LIKE DEHYDRATASE;
☐	750:	1wa5-B	2.1	4.2	107	463	9	PDB	MOLECULE: GTP-BINDING NUCLEAR PROTEIN RAN;
☐	751:	4hbd-A	2.1	3.5	89	245	7	PDB	MOLECULE: KN MOTIF AND ANKYRIN REPEAT DOMAIN-CONTAINING PRO
☐	752:	4i2w-A	2.1	9.5	98	904	8	PDB	MOLECULE: PROTEIN UNC-45;
☐	753:	3q0q-A	2.1	6.0	94	343	9	PDB	MOLECULE: PUMILIO HOMOLOG 2;
☐	754:	1f7u-A	2.1	9.0	92	607	10	PDB	MOLECULE: TRNA(ARG);
☐	755:	5emw-A	2.1	3.7	82	208	4	PDB	MOLECULE: TRANSCRIPTIONAL ENHANCER FACTOR TEF-5;
☐	756:	3hbz-A	2.1	4.6	102	341	11	PDB	MOLECULE: PUTATIVE GLYCOSIDE HYDROLASE;
☐	757:	2h1j-A	2.1	6.8	75	154	5	PDB	MOLECULE: HYPOTHETICAL PROTEIN;
☐	758:	3b18-A	2.1	3.6	69	167	4	PDB	MOLECULE: UNCHARACTERIZED PROTEIN RV0098/MT0107;
☐	759:	1kgz-B	2.1	3.8	77	330	6	PDB	MOLECULE: ANTHRANILATE PHOSPHORIBOSYLTRANSFERASE;
☐	760:	3l7t-A	2.1	4.0	68	133	9	PDB	MOLECULE: PUTATIVE UNCHARACTERIZED PROTEIN;
☐	761:	4tnm-A	2.1	4.9	86	407	5	PDB	MOLECULE: IMPORTIN SUBUNIT ALPHA;
☐	762:	3s4k-A	2.1	4.6	69	124	10	PDB	MOLECULE: PUTATIVE ESTERASE RV1847/MT1895;
☐	763:	5dqq-A	2.1	3.4	58	649	7	PDB	MOLECULE: TWO PORE CALCIUM CHANNEL PROTEIN 1;
☐	764:	4n6h-A	2.1	3.1	53	408	4	PDB	MOLECULE: SOLUBLE CYTOCHROME B562, DELTA-TYPE OPIOID RECEPT
☐	765:	4fml-A	2.1	3.1	53	204	9	PDB	MOLECULE: VSDC;
☐	766:	1g87-B	2.1	3.8	86	613	5	PDB	MOLECULE: ENDOCELLULASE 9G;
☐	767:	3chl-A	2.0	5.0	116	315	7	PDB	MOLECULE: ALPHA-14 GIARDIN;
☐	768:	3now-A	2.0	5.0	102	786	8	PDB	MOLECULE: UNC-45 PROTEIN, SD10334P;
☐	769:	3tb5-A	2.0	5.8	97	254	12	PDB	MOLECULE: METHIONINE AMINOPEPTIDASE;
☐	770:	2opd-A	2.0	3.9	69	121	6	PDB	MOLECULE: PILX;
☐	771:	2i7r-A	2.0	4.1	69	115	7	PDB	MOLECULE: CONSERVED DOMAIN PROTEIN;
☐	772:	3jcm-I	2.0	3.3	60	416	7	PDB	MOLECULE: PRE-MRNA-SPLICING FACTOR 8;
☐	773:	3nmz-B	2.0	6.7	122	400	7	PDB	MOLECULE: APC VARIANT PROTEIN;

Pairwise Structural Alignments

Notation: three-state secondary structure definitions by DSSP (reduced to H=helix, E=sheet, L=coil) are shown above the amino acid sequence. Structurally equivalent residues are in uppercase, structurally non-equivalent residues (e.g. in loops) are in lowercase. Amino acid identities are marked by vertical bars.

No 1: Query=mol1A Sbjct=4ok0A Z-score=14.4

back to top

```
DSSP  llllllllllhhhhhhhhhhhhhhllLLHHHHHHHHHHHHHHHHHHLLlllllllLEEEE
Query aapysspptnlrslrdrltqvaerqgVVFGRLQRHVAXIVVAQFAATLtddtgapLLLVK   60
ident                                |             |             |
Sbjct --------------------------LSEQALNHEKLMRAIVKNLADT-------PMVLK   27
DSSP  --------------------------LLHHHHHHHHHHHHHHHHHHHL-------LLEEL


DSSP  HHHHHHHHHLllLLLLLLEEEEEELLLHhHHHHHHHHHHhhlLLLE-eeEELLLEELlll
Query GGSSLELRRGipDSRTSKDFATVARRDIeLIHEQLADAGetgWEGF-taIFTAPEEIaap  119
ident |   | |  |    | | |        |         |      |
```

```
Sbjct GETALYLGYG--LNRFSEDLDFDCHKKI-NLLGRVKSAI---PNGIilnDIHIKKDT---   78
DSSP  HHHHHHHHLL--LLLLLLLEEEELLLLL-LHHHHHHHHL---LLLEeeeEEEEEEEL---


DSSP  lllLLLEEEEEEEEEL-lEEEEEEEEEEEELLllLLLL--LEEEEelhhhhllllleEE
Query gxpVKPRRFTAKLSYR-gRAFATVPIEVSSVEagNADQ--FDTLTsdalglvgvpaavAV  176
ident        |              |   | |
Sbjct ---DSVGRYMVRYATKdnKEEQTLKLEISYRD--APKEseVNVIE-------------GM  120
DSSP  ---LLEEEEEEEEEELllLLLLEEEEEEEEELLL--LLLHhhEEEEL-------------LE


DSSP  EELLHHHHHHHHHHHHHLLLlllllLLLLHHHHHHHHHH-HHLLhhhHHHHHhhhhhhh
Query PCXTIPWQIAQKLHAVTAVLeepkaNDRAHDLVDLQLLE-GLLLdadLXPTRsaciaife  235
ident     |   |  || |             | || ||  |
Sbjct RIAKIERIIDNKLCACFDGE---htRTKARDLFDLHFLAkHYEE-hfNLDLA--------  168
DSSP  EEELHHHHHHHHHHHHHLLLL---llLLLHHHHHHHHHHHhHLHH-hlLHHHH--------


DSSP  hhlllllLLLL----lLLLLHHHHHHHH--HLLLllllllLLHHHHHHHHHHHHHHHHH-
Query araqhpwPPRV----aTLPHWPLIYAGA--LEGLdhlelaRTVDAAAQAVQRFVARIDR-  288
ident                               |               |         |
Sbjct ----srlKDFSkdpdkLVSDYLVDVKLDalLNQI------MDLEETALELGVMAQLIHKk  218
DSSP  ----hhhHHHLllhhhHHHHHHHHHHHHLllLLLL------LLHHHHHHHHHHHHHHHHHh


DSSP  LLLL---
Query ATKR---  292
ident   |
Sbjct LEKLehh  225
DSSP  HHLLlll
```

No 2: Query=mol1A Sbjct=4ok0B Z-score=14.4

back to top

```
DSSP  lllllllllhhhhhhhhhhhhhllLLHHHHHHHHHHHHHHHHHHHLLllllllLEEEE
Query aapysspptnlrslrdrltqvaerqgVVFGRLQRHVAXIVVAQFAATLtddtgapLLLVK   60
ident                                |             |             |
Sbjct --------------------------LSEQALNHEKLMRAIVKNLADT-------PMVLK   27
DSSP  --------------------------LLHHHHHHHHHHHHHHHHHHHL-------LLEEL


DSSP  HHHHHHHHHLllLLLLLLLLEEEEELLLHhHHHHHHHHHHhhlLLLLEEE-EELLLLEELlll
Query GGSSLELRRGipDSRTSKDFATVARRDIeLIHEQLADAGetgWEGFTA-IFTAPEEIaap  119
ident |   | |  |    | | |        |         |      |
Sbjct GETALYLGYG--LNRFSEDLDFDCHKKI-NLLGRVKSAI---PNGIILnDIHIKKDT---   78
DSSP  HHHHHHHHLL--LLLLLLLEEEELLLLL-LHHHHHHHHL---LLLEEEeEEEEEEEL---


DSSP  lllLLLEEEEEEEEEL-lEEEEEEEEEEEELLllLLLL--LEEEEelhhhhllllleEE
Query gxpVKPRRFTAKLSYR-gRAFATVPIEVSSVEagNADQ--FDTLTsdalglvgvpaavAV  176
ident        |              |   | |
Sbjct ---DSVGRYMVRYATKdnKEEQTLKLEISYRD--APKEseVNVIE-------------GM  120
DSSP  ---LLEEEEEEEEEELleEEEEEEEEEEEELLL--LLLHhhEEEEL-------------LE


DSSP  EELLHHHHHHHHHHHHHLLLlllllLLLLHHHHHHHHHH-HHLLHhHHHHHHhhhhhhh
Query PCXTIPWQIAQKLHAVTAVLeepkaNDRAHDLVDLQLLE-GLLLDaDLXPTRsaciaife  235
ident     |   |  || |             | || ||  |
Sbjct RIAKIERIIDNKLCACFDGE---htRTKARDLFDLHFLAkHYEEH-FNLDLA--------  168
DSSP  EEELHHHHHHHHHHHHHLLLL---llLLLHHHHHHHHHHHhHLHHH-LLHHHH--------


DSSP  hhlllllLLLL----LLLLLHHHHHHHH--HLLLllllllLLHHHHHHHHHHHHHHHHH-
Query araqhpwPPRV----ATLPHWPLIYAGA--LEGLdhlelaRTVDAAAQAVQRFVARIDR-  288
ident                               |               |         |
Sbjct ----srlKDFSkdpdKLVSDYLVDVKLDalLNQI------MDLEETALELGVMAQLIHKk  218
DSSP  ----hhhHHHHllhhHHHHHHHHHHHHHLllLLLL------LLHHHHHHHHHHHHHHHHHh


DSSP  LLLL-----
Query ATKR-----  292
ident   |
Sbjct LEKLehhhh  227
DSSP  HHHHhhlll
```

No 3: Query=mol1A Sbjct=4o8sA Z-score=14.1

back to top

```
DSSP  lllllllllhhhhhhhhhhhhhhlLLLHHHHHHHHHHHHHHHHHHHHLLlllllllLEEEE
Query aapysspptnlrslrdrltqvaerqGVVFGRLQRHVAXIVVAQFAATLtddtgapLLLVK   60
ident                                |     |       |             |
Sbjct -------------------------SLSEQALNHEKLXRAIVKNLADT-------PXVLK   28
DSSP  -------------------------LLLHHHHHHHHHHHHHHHHHHHHL-------LLEEL


DSSP  HHHHHHHHHLllLLLLLLLEEEEEELLLhhHHHHHHHHHHHhhLLLLE-eeEELLLEELlll
Query GGSSLELRRGipDSRTSKDFATVARRDieLIHEQLADAGetGWEGF-taIFTAPEEIaap  119
ident |   | |  |    | | |                         |
Sbjct GETALYLGYG--LNRFSEDLDFDCHKK--INLLGRVKSA--IPNGIilnDIHIKKDT---   79
DSSP  HHHHHHHHLL--LLLLLLLEEEEELLL--LLHHHHHHHH--LLLLEeeeEEEEEEEL---


DSSP  lllLLLEEEEEEEEEL-lEEEEEEEEEEEELLllLLLL--LEEEEelhhhhllllleEE
Query gxpVKPRRFTAKLSYR-gRAFATVPIEVSSVEagNADQ--FDTLTsdalglvgvpaavAV  176
ident        |              |   | |
Sbjct ---DSVGRYXVRYATKdnKEEQTLKLEISYRD--APKEseVNVIE-------------GX  121
DSSP  ---LLEEEEEEEEEELllLLLEEEEEEEEELL--LLLHhhEEEEL-------------LE


DSSP  EELLHHHHHHHHHHHHHLLLLllllLLLLHHHHHHHHHH-HHLLHhHHHHHHhhhhhhh
Query PCXTIPWQIAQKLHAVTAVLEepkaNDRAHDLVDLQLLE-GLLLDaDLXPTRsaciaife  235
ident     |   |  || |             | || ||  |
Sbjct RIAKIERIIDNKLCACFDGEH---tRTKARDLFDLHFLAkHYEEH-FNLDLA--------  169
DSSP  EEELHHHHHHHHHHHHHLLLLL---lLLLHHHHHHHHHHHHhHLHHH-LLHHHH--------


DSSP  hhllllllLLLL----LLLLLHHHHHHHHH--HLLLllllllLLHHHHHHHHHHHHHHHHH-
Query araqhpwPPRV----ATLPHWPLIYAGA--LEGLdhlelaRTVDAAAQAVQRFVARIDR-  288
ident                               |               |         |
Sbjct ----srlKDFSkdpdKLVSDYLVDVKLDalLNQI------XDLEETALELGVXAQLIHKk  219
DSSP  ----hhhHHHHllhhHHHHHHHHHHHHHLllLLLL------LLHHHHHHHHHHHHHHHHHh


DSSP  LLLL---------
Query ATKR---------  292
ident   |
Sbjct LEKQshslnalqe  232
DSSP  HHHHhhhhhhhhl
```

No 4: Query=mol1A Sbjct=5cfuA Z-score=11.3

back to top

```
DSSP  lllllllllhhhhhhhhhhhhhhllllhhhhhhhHHHHHHHHHHH-LLLLllLLLLLEEE
Query aapysspptnlrslrdrltqvaerqgvvfgrlqrhVAXIVVAQFAA-TLTDdtGAPLLLV   59
ident                                                          |
Sbjct -----------------------------------TTQVTLIHKILaAADE--RNLPLWI   23
DSSP  -----------------------------------LHHHHHHHHHHhHHHH--LLLLLEE


DSSP  EHHHHHHH-HHLLllLLLLLLEEEEEELL-LHHHHHHHHHHHhhhlllleeeEELLLEELL
Query KGGSSLEL-RRGIpdSRTSKDFATVARR-DIELIHEQLADAgetgwegftaIFTAPEEIA  117
ident  ||             |   |                                   ||
Sbjct GGGWAIDArLGRV--TRKHDDIDLTFPGeRRGELEAIVEML----------GGRVMEELD   71
DSSP  EHHHHHHHhHLLL--LLLLLLEEEEEEHhHHHHHHHHHHHL----------LLEEEEELL


DSSP  llllllLLEEEEEEeeelleeeeEEEEEEEEL------------lLLLLLL-LEEEEELh
Query apgxpvKPRRFTAKlsyrgrafaTVPIEVSSV------------eAGNADQ-FDTLTSDa  164
ident                                               |
Sbjct ------YGFLAEIG---------DELLDCEPAwwadeayeiaeapQGSCPEaAEGVIAG-  115
DSSP  ------LLEEEEEL---------LEEEEEEEEeellllleeellllLLLLLLlLLEEELL-


DSSP  hhhllllllEEEEELLHHHHHHHHHHHHHHLLLllllLLLLLHHHHHHHHHHHHHLLHHHHH
Query lglvgvpaaVAVPCXTIPWQIAQKLHAVTAVLeepkANDRAHDLVDLQLLEGLLLDADLX  224
ident            | |      |         |                 |    |
Sbjct ---------RPVRCNSWEAIIWDYFYYADEVP---pVDWPTKHIESYRLACTSLGAEKVE  163
DSSP  ---------EEELLLLHHHHHHHHHHHHHHLL---hHHLLHHHHHHHHHHHHHHHLHHHHH
```

```
DSSP  HHHHhhhhhhhhhlllLLLLllllllllhhhhhhhhhllllllllllllhhhhhhhhhhhh
Query PTRSaciaifearaqhPWPPrvatlphwpliyagalegldhlelartvdaaaqavqrfva  284
ident   |
Sbjct VLRA----------afRSRY----------------------------------------  173
DSSP  HHHH----------hhHHHL----------------------------------------


DSSP  hhhhllll
Query ridratkr  292
ident
Sbjct -------a  174
DSSP  -------l
```

No 5: Query=mol1A Sbjct=5cftA Z-score=11.2

back to top

```
DSSP  llllllllllhhhhhhhhhhhhhhllllhhhhhhHHHHHHHHHHHH-LLLLllLLLLLEEE
Query aapysspptnlrslrdrltqvaerqgvvfgrlqrHVAXIVVAQFAA-TLTDdtGAPLLLV   59
ident                                                          |
Sbjct ----------------------------------DTTQVTLIHKILaAADE--RNLPLWI   24
DSSP  ----------------------------------LHHHHHHHHHHHhHHHH--LLLLLEE


DSSP  EHHHHHHH-HHLLlLLLLLLLEEEEELL-LHHHHHHHHHHHhhhllllleeeEELLLEELL
Query KGGSSLEL-RRGIpdSRTSKDFATVARR-DIELIHEQLADAgetgwegftaIFTAPEEIA  117
ident  ||             |   |                                   ||
Sbjct GGGWAIDArLGRV--TRKHDDIDLTFPGeRRGELEAIVEML----------GGRVMEELD   72
DSSP  EHHHHHHHhHLLL--LLLLLLEEEEEEHhHHHHHHHHHHHL----------LLEEEEELL


DSSP  llllllLLEEEEEEeeelleeeeEEEEEEEEL------------lLLLLLL-LEEEEELh
Query apgxpvKPRRFTAKlsyrgrafaTVPIEVSSV------------eAGNADQ-FDTLTSDa  164
ident                                               |
Sbjct ------YGFLAEIG---------DELLDCEPAwwadeayeiaeapQGSCPEaAEGVIAG-  116
DSSP  ------LLEEEEEL---------LEEEEEEEEeeelleeelllllLLLLLLlLLEEELL-


DSSP  hhhllllllEEEEEELLHHHHHHHHHHHHHHLLllllllLLLLHHHHHHHHHHHHHLLHHHHH
Query lglvgvpaaVAVPCXTIPWQIAQKLHAVTAVleepkaNDRAHDLVDLQLLEGLLLDADLX  224
ident            | |      |         |                 |    |
Sbjct ---------RPVRCNSWEAIIWDYFYYADEV---ppvDWPTKHIESYRLACTSLGAEKVE  164
DSSP  ---------EEELLLLHHHHHHHHHHHHHHL---lhhHLLHHHHHHHHHHHHHHLHHHHH


DSSP  HHHhhhhhhhhhhllLLLLlllllllhhhhhhhhhllllllllllllhhhhhhhhhhhh
Query PTRsaciaifearaqHPWPprvatlphwpliyagalegldhlelartvdaaaqavqrfva  284
ident   |
Sbjct VLR---------aafRSRY-----------------------------------------  174
DSSP  HHH---------hhhHHHH-----------------------------------------


DSSP  hhhhllll
Query ridratkr  292
ident
Sbjct -------a  175
DSSP  -------l
```

No 6: Query=mol1A Sbjct=5cfsA Z-score=11.1

back to top

```
DSSP  llllllllllhhhhhhhhhhhhhhllllhhhhhhHHHHHHHHHHHH-LLLLllLLLLLEEE
Query aapysspptnlrslrdrltqvaerqgvvfgrlqrHVAXIVVAQFAA-TLTDdtGAPLLLV   59
ident                                                          |
Sbjct ----------------------------------DTTQVTLIHKILaAADE--RNLPLWI   24
DSSP  ----------------------------------LHHHHHHHHHHHhHHHH--LLLLLEE


DSSP  EHHHHHHH-HHLLlLLLLLLLEEEEELL-LHHHHHHHHHHHhhhllllleeeEELLLEELL
Query KGGSSLEL-RRGIpdSRTSKDFATVARR-DIELIHEQLADAgetgwegftaIFTAPEEIA  117
ident  ||             |   |                                   ||
Sbjct GGGWAIDArLGRV--TRKHDDIDLTFPGeRRGELEAIVEML----------GGRVMEELD   72
```

```
DSSP  EHHHHHHHhHLLL--LLLLLLEEEEEEHhHHHHHHHHHHHL----------LLEEEEELL
```

```
DSSP  llllllLLEEEEEEeeelleeeeEEEEEEEEL------------lLLLLLL-LEEEEELh
Query apgxpvKPRRFTAKlsyrgrafaTVPIEVSSV------------eAGNADQ-FDTLTSDa   164
ident                                               |
Sbjct ------YGFLAEIG---------DELLDCEPAwwadeayeiaeapQGSCPEaAEGVIAG-   116
DSSP  ------LLEEEEEL---------LEEEEEEEEeeelleeeellllLLLLLLlLLEEELL-
```

```
DSSP  hhhllllllEEEEELLHHHHHHHHHHHHHHLLllllllLLLLHHHHHHHHHHHHHLLHHHHH
Query lglvgvpaaVAVPCXTIPWQIAQKLHAVTAVleepkaNDRAHDLVDLQLLEGLLLDADLX   224
ident            | |      |         |                 |    |
Sbjct ---------RPVRCNSWEAIIWDYFYYADEV---ppvDWPTKHIESYRLACTSLGAEKVE   164
DSSP  ---------EEELLLLHHHHHHHHHHHHHHHL---lhhHLLHHHHHHHHHHHHHHHLHHHHH
```

```
DSSP  HHHHhhhhhhhhhllLLLLlllllllllhhhhhhhhllllllllllhhhhhhhhhhhh
Query PTRSaciaifearaqHPWPprvatlphwpliyagalegldhlelartvdaaaqavqrfva   284
ident   |
Sbjct VLRA---------afRSRY-----------------------------------------   174
DSSP  HHHH---------hhHHHH-----------------------------------------
```

```
DSSP  hhhhllll
Query ridratkr  292
ident
Sbjct ------aa  176
DSSP  ------ll
```

No 7: Query=mol1A Sbjct=4xjeA Z-score=11.1

back to top

```
DSSP  llllllllllhhhhhhhhhhhhhhllllhhhhhhHHHHHHHHHHHH-LLLLllLLLLEEE
Query aapyssppptnlrslrdrltqvaerqgvvfgrlqrHVAXIVVAQFAA-TLTDdtGAPLLLV    59
ident                                                          |
Sbjct -----------------------------------DTTQVTLIHKILaAADE--RNLPLWI    24
DSSP  -----------------------------------LHHHHHHHHHHHhHHHH--LLLLLEE
```

```
DSSP  EHHHHHHHHHLLllLLLLLLEEEEEELL-LHHHHHHHHHHHhhhllllleeeEELLLEELLl
Query KGGSSLELRRGIpdSRTSKDFATVARR-DIELIHEQLADAgetgwegftaIFTAPEEIAa   118
ident  ||     | |    |   |                                   ||
Sbjct GGGWAIDARLGR-vTRKHDDIDLTFPGeRRGELEAIVEML----------GGRVMEELD-    72
DSSP  EHHHHHHHHHLL-lLLLLLLEEEEEEHhHHHHHHHHHHHHL----------LLEEEEELL-
```

```
DSSP  lllllLLEEEEEEeeelleeeeEEEEEEEE--------LLLL----LLLL-lEEEEELhh
Query pgxpvKPRRFTAKlsyrgrafaTVPIEVSS--------VEAG----NADQ-fDTLTSDal   165
ident                                         |
Sbjct -----YGFLAEIG---------DELLDCEPawwadeayEIAEapqgSCPEaaEGVIAG--   116
DSSP  -----LLEEEEEL---------LEEEEEEEeeellllleEELLllllLLLLllLEEELL--
```

```
DSSP  hhllllllEEEEELLHHHHHHHHHHHHHHLLlllllLLLLLHHHHHHHHHHHHHLLHHHHHH
Query glvgvpaaVAVPCXTIPWQIAQKLHAVTAVleepkANDRAHDLVDLQLLEGLLLDADLXP   225
ident           | |      |         |                 |    |
Sbjct --------RPVRCNSWEAIIWDYFYYADEV---ppVDWPTKHIESYRLACTSLGAEKVEV   165
DSSP  --------EEELLLLHHHHHHHHHHHHHHHL---lhHHLLHHHHHHHHHHHHHHHLHHHHHH
```

```
DSSP  HHHhhhhhhhhhllLLLLlllllllllhhhhhhhhllllllllllhhhhhhhhhhhhh
Query TRSaciaifearaqHPWPprvatlphwpliyagalegldhlelartvdaaaqavqrfvar   285
ident  |
Sbjct LRA---------afRSRY------------------------------------------   174
DSSP  HHH---------hhHHHH------------------------------------------
```

```
DSSP  hhhllll
Query idratkr  292
ident
Sbjct ------a  175
DSSP  ------l
```

No 8: Query=mol1A Sbjct=4wqlA Z-score=11.1

back to top

```
DSSP  lllllllllhhhhhhhhhhhhhhllllhhhhhhhHHHHHHHHHHH-LLLLllLLLLEEE
Query aapysspptnlrslrdrltqvaerqgvvfgrlqrhVAXIVVAQFAA-TLTDdtGAPLLLV   59
ident                                                          |
Sbjct -----------------------------------TTQVTLIHKILaAADE--RNLPLWI   23
DSSP  -----------------------------------LHHHHHHHHHHhHHHH--LLLLLEE


DSSP  EHHHHHHH-HHLLlLLLLLLLEEEEELL-LHHHHHHHHHHhhhhlllleeEEELLLEELL
Query KGGSSLEL-RRGIpdSRTSKDFATVARR-DIELIHEQLADagetgwegftAIFTAPEEIA  117
ident  ||             |   |                                   ||
Sbjct GGGWAIDArLGRV--TRKHDDIDLTFPGeRRGELEAIVEM----------LGGRVMEELD   71
DSSP  EHHHHHHHhHLLL--LLLLLLEEEEEEHhHHHHHHHHHHH----------LLLEEEEELL


DSSP  llllllLLEEEEEEeeelleeeeEEEEEEEE--------LLLL----LLLL-lEEEEELh
Query apgxpvKPRRFTAKlsyrgrafaTVPIEVSS--------VEAG----NADQ-fDTLTSDa  164
ident                                          |
Sbjct ------YGFLAEIG---------DELLDCEPawwadeayEIAEapqgSCPEaaEGVIAG-  115
DSSP  ------LLEEEEEL---------LEEEEEEEeeellllleELLLllllLLLLllLEEELL-


DSSP  hhhllllllEEEEELLHHHHHHHHHHHHHHLLLllllLLLLLHHHHHHHHHHHHHLLHHHHH
Query lglvgvpaaVAVPCXTIPWQIAQKLHAVTAVLeepkANDRAHDLVDLQLLEGLLLDADLX  224
ident            | |      |         |                 |    |
Sbjct ---------RPVRCNSWEAIIWDYFYYADEVP---pVDWPTKHIESYRLACTSLGAEKVE  163
DSSP  ---------EEELLLLHHHHHHHHHHHHHHHHLL---hHHLLHHHHHHHHHHHHHHHLHHHHH


DSSP  HHHHhhhhhhhhhlllLLLLlllllllhhhhhhhhllllllllllhhhhhhhhhhhh
Query PTRSaciaifearaqhPWPPrvatlphwpliyagalegldhlelartvdaaaqavqrfva  284
ident   |
Sbjct VLRA----------afRSRY----------------------------------------  173
DSSP  HHHH----------hhHHHH----------------------------------------


DSSP  hhhhllll
Query ridratkr  292
ident
Sbjct ------aa  175
DSSP  ------ll
```

No 9: Query=mol1A Sbjct=4wqkA Z-score=10.6

back to top

```
DSSP  lllllllllhhhhhhhhhhhhhhllllhhhHHHHHHHHHHHHHHHHLLLlllllLLEEEE
Query aapysspptnlrslrdrltqvaerqgvvfgrLQRHVAXIVVAQFAATLTddtgaPLLLVK   60
ident                                             |           |
Sbjct -------------------------------TTQVTLIHKILAAADERN-----LPLWIG   24
DSSP  -------------------------------LHHHHHHHHHHHHHHHHLL-----LLLEEE


DSSP  HHHHHHHHHLLlLLLLLLLEEEEELL-LHHHHHHHHHHhhhhlllleeEEELLLEELLll
Query GGSSLELRRGIpDSRTSKDFATVARR-DIELIHEQLADagetgwegftAIFTAPEEIAap  119
ident ||     | |    |   |                                   ||
Sbjct GGWAIDARLGR-VTRKHDDIDLTFPGeRRGELEAIVEM----------LGGRVMEELD--   71
DSSP  HHHHHHHHHLL-LLLLLLLEEEEEEHhHHHHHHHHHHH----------LLLEEEEEEL--


DSSP  llllLLEEEEEEeeelleeeeEEEEEEEEL--------LLLL-lLLLE---EEEELHhhh
Query gxpvKPRRFTAKlsyrgrafaTVPIEVSSV--------EAGN-aDQFD---TLTSDAlgl  167
ident                                        |
Sbjct ----YGFLAEIG---------DELLDCEPAwwadeayeIAEApqGSCPeaaEGVIAG---  115
DSSP  ----LEEEEEEL---------LEEEEEEEEeellllleeLLLLllLLLLlllLEEELL---


DSSP  llllllEEEEELLHHHHHHHHHHHHHHLLLllllLLLLLHHHHHHHHHHHHHLLHHHHHHHH
Query vgvpaaVAVPCXTIPWQIAQKLHAVTAVLeepkANDRAHDLVDLQLLEGLLLDADLXPTR  227
ident         | |      |         |                 |    |        |
Sbjct ------RPVRCNSWEAIIWDYFYYADEVP---pVDWPTKHIESYRLACTSLGAEKVEVLR  166
DSSP  ------EEELLLLHHHHHHHHHHHHHHHHLL---hHHLLHHHHHHHHHHHHHHHLHHHHHHHH
```

```
DSSP  HhhhhhhhhhlllLLLLlllllllhhhhhhhhhllllllllllllhhhhhhhhhhhhhhhh
Query SaciaifearaqhPWPPrvatlphwpliyagalegldhlelartvdaaaqavqrfvarid  287
ident
Sbjct A---------afrSRYA-------------------------------------------  174
DSSP  H---------hhhHHHL-------------------------------------------


DSSP  hllll
Query ratkr  292
ident
Sbjct ----a  175
DSSP  ----l
```

No 10: Query=mol1A Sbjct=4e8jB Z-score=9.6

back to top

```
DSSP  llllllllllhhhhhhhhhhhhhhllllhhhhhHHHHHHHHHHHHHLLLlllllLLEEEE
Query aapyssppptnlrslrdrltqvaerqgvvfgrlqRHVAXIVVAQFAATLTddtgaPLLLVK   60
ident
Sbjct ------------------------------nnvTEKELFYILDLFEHXK-----VTYWLD   25
DSSP  ------------------------------lllLHHHHHHHHHHHHHLL-----LLEEEE


DSSP  HHHHHHHHHLLlLLLLLLLEEEEELL-LHHHHHHHHHHhhhhlllleeEEELLLEELlll
Query GGSSLELRRGIpDSRTSKDFATVARR-DIELIHEQLADagetgwegftAIFTAPEEIaap  119
ident ||       |    |   |                ||
Sbjct GGWGVDVLTGK-QQREHRDIDIDFDAqHTQKVIQKLED----------IGYKIEVHW---   71
DSSP  HHHHHHHHHLL-LLLLLLEEEEEEEHhHHHHHHHHHHH----------LLLEEEEEE---


DSSP  lllLLLEEEEEEeeelleeeEEEEEEEEEL-----------------LLLLlLLLEEEEE
Query gxpVKPRRFTAKlsyrgrafATVPIEVSSV-----------------EAGNaDQFDTLTS  162
ident                                                     | |
Sbjct --xPSRXELKHE--------EYGYLDIHPInlnddgsitqanpeggnYVFQnDWFSETNY  121
DSSP  --lLLEEEEEEL--------LLEEEEEEEEeellllleeeelllllleEELLhHHEEEEEE


DSSP  LhhhhlllllLEEEEELLHHHHHHHHhhHHHLlllllllllLLHHHHHHHHHHHHllhhh
Query DalglvgvpaAVAVPCXTIPWQIAQKlhAVTAvleepkandRAHDLVDLQLLEGLlldad  222
ident               ||     |                      |  |   |
Sbjct K---------DRKIPCISKEAQLLFH--SGYD--------lTETDHFDIKNLKSI-----  157
DSSP  L---------LEEEEEELHHHHHHHL--LLLL--------lLHHHHHHHHHHHLL-----


DSSP  hhhhhhhhhhhhhhhllllllllllllllhhhhhhhhhllllllllllllhhhhhhhhhhh
Query lxptrsaciaifearaqhpwpprvatlphwpliyagalegldhlelartvdaaaqavqrf  282
ident
Sbjct ------------------------------------------------------------  157
DSSP  ------------------------------------------------------------


DSSP  hhhhhhllll
Query varidratkr  292
ident
Sbjct ---------t  158
DSSP  ---------l
```

No 11: Query=mol1A Sbjct=2ewrA Z-score=9.5

back to top

```
DSSP  llllllllllhhhhhhhhhhhhhhllllhhHHHHHHHHHHHHHHHHLLLlllllLLEEEE
Query aapyssppptnlrslrdrltqvaerqgvvfgRLQRHVAXIVVAQFAATLTddtgaPLLLVK   60
ident                               |                           |
Sbjct -----------------------------iRPEYLRVLRKIYDRLKNEK-----VNWVVT   26
DSSP  -----------------------------lLHHHHHHHHHHHHHHLLLL-----LLEEEE


DSSP  HHHHHHHHHllllLLLLLLEEEEELLL-HHHHHHHHHhhhhhlllleeeEELLLE-ELLL
Query GGSSLELRRgipdSRTSKDFATVARRD-IELIHEQLAdagetgwegftaIFTAPE-EIAA  118
ident |  |  |           |            |                  |
Sbjct GSLSFALQG---vPVEVHDIDIQTDEEgAYEIERIFS------------EFVSKKvRFSS   71
```

```
DSSP  HHHHHHHLL---lLLLLLLEEEEELHHhHHHHHHHLH-----------HHEEEEeEEEE


DSSP  L-LLLLLLEEEEEeeeelleeeEEEEEEEEELL-----------LLLL-LLLEEEEELhh
Query P-GXPVKPRRFTAklsyrgrafATVPIEVSSVE-----------AGNA-DQFDTLTSDal   165
ident                            |
Sbjct TeKICSHFGELII---------DGIKVEIXGDIrkrledgtwedPVDLnKYKRFVETH--   120
DSSP  LlLEEEEEEEEEE---------LLEEEEEEEEEeellllllllLLLHhHHEEEEEEL--


DSSP  hhllllLEEEEELLHHHHHHHHHHHHhllllllllllhHHHHHHHHHHHLLHHHhhh
Query glvgvpaAVAVPCXTIPWQIAQKLHAVtavleepkandrahDLVDLQLLEGLLLDADlxp   225
ident            |           |                        |   |
Sbjct -------GXKIPVLSLEYEYQAYLKLG--------------RVEKAETLRKWLNERK---   156
DSSP  -------LEEEEEELHHHHHHHHHHHL--------------LHHHHHHHHHHHHHLL---


DSSP  hhhhhhhhhhhllllllllllllhhhhhhhhhllllllllllhhhhhhhhhhhhh
Query trsaciaifearaqhpwpprvatlphwpliyagalegldhlelartvdaaaqavqrfvar   285
ident
Sbjct ------------------------------------------------------------   156
DSSP  ------------------------------------------------------------


DSSP  hhhllll
Query idratkr  292
ident
Sbjct -------  156
DSSP  -------
```

No 12: Query=mol1A Sbjct=2fclA Z-score=9.4

back to top

```
DSSP  llllllllllhhhhhhhhhhhhhhllllhhHHHHHHHHHHHHHHHHHLLLlllllLLEEEE
Query aapysspptnlrslrdrltqvaerqgvvfgRLQRHVAXIVVAQFAATLTddtgaPLLLVK    60
ident                               |                           |
Sbjct ----------------------------miRPEYLRVLRXIYDRLXNEX-----VNWVVT    27
DSSP  ----------------------------llLHHHHHHHHHHHHHHHLLLL-----LLEEEE


DSSP  HHHHHHHHHllllLLLLLLEEEEELLL-HHHHHHHHHhhhhhllllleeEEELL-LEELLL
Query GGSSLELRRgipdSRTSKDFATVARRD-IELIHEQLAdagetgwegftAIFTA-PEEIAA   118
ident |  |  |           |            |
Sbjct GSLSFALQG---vPVEVHDIDIQTDEEgAYEIERIFS-----------EFVSXxVRFSST    73
DSSP  HHHHHHHLL---lLLLLLLEEEEELHHhHHHHHHHLH-----------HHEEEeEEEEEL


DSSP  LLLlLLLEEEEEEeeelleeeEEEEEEEEEL------------LLLLLLLLEEEEELhhh
Query PGXpVKPRRFTAKlsyrgrafATVPIEVSSV------------EAGNADQFDTLTSDalg   166
ident                           |
Sbjct EXI-CSHFGELII--------DGIXVEIMGDirxrledgtwedPVDLNXYXRFVETH---   121
DSSP  LLE-EEEEEEEEE--------LLEEEEEEEEeeellllllllLLLHHHHEEEEEEL---


DSSP  hlllllLEEEEELLHHHHHHHHHHHHhllllllllllhHHHHHHHHHHHLLHHHhhhh
Query lvgvpaAVAVPCXTIPWQIAQKLHAVtavleepkandrahDLVDLQLLEGLLLDADlxpt   226
ident           |           |                        |   |
Sbjct ------GMXIPVLSLEYEYQAYLXLG--------------RVEXAETLRXWLNERX----   157
DSSP  ------LEEEEEELHHHHHHHHHHHL--------------LHHHHHHHHHHHHHHL----


DSSP  hhhhhhhhhhllllllllllllhhhhhhhhhllllllllllhhhhhhhhhhhhhh
Query rsaciaifearaqhpwpprvatlphwpliyagalegldhlelartvdaaaqavqrfvari   286
ident
Sbjct ------------------------------------------------------------   157
DSSP  ------------------------------------------------------------


DSSP  hhllll
Query dratkr  292
ident
Sbjct ------  157
DSSP  ------
```

No 13: Query=mol1A Sbjct=4e8iB Z-score=9.2

back to top

```
DSSP  llllllllllhhhhhhhhhhhhhhllllhhhhhHHHHHHHHHHHHHHLLLlllllLLEEEE
Query aapysspptnlrslrdrltqvaerqgvvfgrlqRHVAXIVVAQFAATLTddtgaPLLLVK   60
ident
Sbjct ------------------------------nnvTEKELFYILDLFEHXK-----VTYWLD   25
DSSP  ------------------------------lllLHHHHHHHHHHHHHLL-----LLEEEE


DSSP  HHHHHHHHHLLlLLLLLLLEEEEELL-LHHHHHHHHHHhhhhllllleeEEELLLEELlll
Query GGSSLELRRGIpDSRTSKDFATVARR-DIELIHEQLADagetgwegftAIFTAPEEIaap  119
ident ||       |    |   |                |  |
Sbjct GGWGVDVLTGK-QQREHRDIDIDFDAqHTQKVIQKLED----------IGYKIEVHW---   71
DSSP  HHHHHHHHHLL-LLLLLLEEEEEEEHhHHHHHHHHHHH----------LLLEEEEEE---


DSSP  lllLLLEEEEEEEeeelleeeEEEEEEEEEL-----------------LLLLlLLLEEEEE
Query gxpVKPRRFTAKlsyrgrafATVPIEVSSV-----------------EAGNaDQFDTLTS  162
ident                                                     | |
Sbjct --xPSRXELKHE--------EYGYLDIHPInlnddgsitqanpeggnYVFQnDWFSETNY  121
DSSP  --lLLEEEEEEL--------LLEEEEEEEEeelllllleeeeelleeeEEELhHHEEEEEE


DSSP  LhhhhllllLEEEEEELLHHHHHHHHhhHHHLllllllllLLHHHHHHHHHHHHLlhhh
Query DalglvgvpaAVAVPCXTIPWQIAQKlhAVTAvleepkandRAHDLVDLQLLEGLLldad  222
ident               ||     |                      |  |   |
Sbjct K---------DRKIPCISKEAQLLFH--SGYD--------lTETDHFDIKNLKSIT----  158
DSSP  L---------LEEEEEEELHHHHHHHL--LLLL--------lLHHHHHHHHHHHHHLL----


DSSP  hhhhhhhhhhhhhhhllllllllllllllhhhhhhhhhlllllllllllhhhhhhhhhhh
Query lxptrsaciaifearaqhpwpprvatlphwpliyagalegldhlelartvdaaaqavqrf  282
ident
Sbjct ------------------------------------------------------------  158
DSSP  ------------------------------------------------------------


DSSP  hhhhhhllll
Query varidratkr  292
ident
Sbjct ----------  158
DSSP  ----------
```

No 14: Query=mol1A Sbjct=4fo1B Z-score=9.2

back to top

```
DSSP  llllllllllhhhhhhhhhhhhhhllllhhhhhHHHHHHHHHHHHHHLLLlllllLLEEEE
Query aapysspptnlrslrdrltqvaerqgvvfgrlqRHVAXIVVAQFAATLTddtgaPLLLVK   60
ident
Sbjct ------------------------------nnvTEKELFYILDLFEHXK-----VTYWLD   25
DSSP  ------------------------------lllLHHHHHHHHHHHHHLL-----LLEEEE


DSSP  HHHHHHHHHLLlLLLLLLLEEEEELL-LHHHHHHHHHHhhhhllllleeEEELLLEELlll
Query GGSSLELRRGIpDSRTSKDFATVARR-DIELIHEQLADagetgwegftAIFTAPEEIaap  119
ident ||       |    |   |                |  |
Sbjct GGWGVDVLTGK-QQREHRDIDIDFDAqHTQKVIQKLED----------IGYKIEVHW---   71
DSSP  HHHHHHHHHLL-LLLLLLEEEEEEEHhHHHHHHHHHHH----------LLLEEEEEL---


DSSP  lllLLLEEEEEEEeeelleeeEEEEEEEEEL-----------------LLLLlLLLEEEEE
Query gxpVKPRRFTAKlsyrgrafATVPIEVSSV-----------------EAGNaDQFDTLTS  162
ident                                                     | |
Sbjct --xPSRXELKHE--------EYGYLDIHPInlnddgsitqanpeggnYVFQnDWFSETNY  121
DSSP  --lLLLEEEEEL--------LLEEEEEEEEeelllllleeeeelleeeEEELhHHEEEEEL


DSSP  LhhhhllllLEEEEEELLHHHHHHHHhhHHHLllllllllLLHHHHHHHHHHHHLlhhh
Query DalglvgvpaAVAVPCXTIPWQIAQKlhAVTAvleepkandRAHDLVDLQLLEGLLldad  222
ident               ||     |                      |  |   |
Sbjct K---------DRKIPCISKEAQLLFH--SGYD--------lTETDHFDIKNLKSIT----  158
DSSP  L---------LLEEEEEELHHHHHHHL--LLLL--------lLHHHHHHHHHHHHHL----
```

```
DSSP  hhhhhhhhhhhhhhhlllllllllllllhhhhhhhhhllllllllllhhhhhhhhhh
Query lxptrsaciaifearaqhpwpprvatlphwpliyagalegldhlelartvdaaaqavqrf  282
ident
Sbjct ------------------------------------------------------------  158
DSSP  ------------------------------------------------------------


DSSP  hhhhhhllll
Query varidratkr  292
ident
Sbjct ----------  158
DSSP  ----------
```

No 15: Query=mol1A Sbjct=4wh5B Z-score=9.1

back to top

```
DSSP  lllllllllhhhhhhhhhhhhhhllllhhhhhHHHHHHHHHHHHHHLLLlllllLLEEEE
Query aapyssppptnlrslrdrltqvaerqgvvfgrlqRHVAXIVVAQFAATLTddtgaPLLLVK   60
ident
Sbjct ------------------------------nnvTEKELFYILDLFEHXK-----VTYWLD   25
DSSP  ------------------------------lllLHHHHHHHHHHHHHLL-----LLEEEE


DSSP  HHHHHHHHHLLlLLLLLLLEEEEELL-LHHHHHHHHHHhhhhllllleeEEELLLEELlll
Query GGSSLELRRGIpDSRTSKDFATVARR-DIELIHEQLADagetgwegftAIFTAPEEIaap  119
ident ||       |    |   |                 | |
Sbjct GGWGVDVLTGK-QQREHRDIDIDFDAqHTQKVIQKLED----------IGYKIEVHW---   71
DSSP  HHHHHHHHHLL-LLLLLLEEEEEEEHhHHHHHHHHHHH----------LLLEEEEEEE---


DSSP  lllLLLEEEEEEeeelleeeEEEEEEEEEL-----------------LLLLlLLLEEEEE
Query gxpVKPRRFTAKlsyrgrafATVPIEVSSV-----------------EAGNaDQFDTLTS  162
ident                                                       | |
Sbjct --xPSRXELKHE--------EYGYLDIHPInlnddgsitqanpeggnYVFQnDWFSETNY  121
DSSP  --lLLEEEEEEL--------LLEEEEEEEEeellllleeeelllllleEELLhHHEEEEEE


DSSP  LhhhhlllllLEEEEELLHHHHHHHHHhHHHLlllllllllLLHHHHHHHHHHHHHllhhh
Query DalglvgvpaAVAVPCXTIPWQIAQKlhAVTAvleepkandRAHDLVDLQLLEGLlldad  222
ident               ||     |                      |  |   |
Sbjct K---------DRKIPCISKEAQLLFH--SGYD--------lTETDHFDIKNLKSI-----  157
DSSP  L---------LEEEEEELHHHHHHHL--LLLL--------lLHHHHHHHHHHHLL-----


DSSP  hhhhhhhhhhhhhhhlllllllllllllhhhhhhhhhllllllllllhhhhhhhhhh
Query lxptrsaciaifearaqhpwpprvatlphwpliyagalegldhlelartvdaaaqavqrf  282
ident
Sbjct ------------------------------------------------------------  157
DSSP  ------------------------------------------------------------


DSSP  hhhhhhllll
Query varidratkr  292
ident
Sbjct ---------t  158
DSSP  ---------l
```

No 16: Query=mol1A Sbjct=4fo1A Z-score=9.1

back to top

```
DSSP  lllllllllhhhhhhhhhhhhhhllllhhhhhHHHHHHHHHHHHHHLLLlllllLLEEEE
Query aapyssppptnlrslrdrltqvaerqgvvfgrlqRHVAXIVVAQFAATLTddtgaPLLLVK   60
ident
Sbjct ------------------------------nnvTEKELFYILDLFEHXK-----VTYWLD   25
DSSP  ------------------------------lllLHHHHHHHHHHHHHLL-----LLEEEE


DSSP  HHHHHHHHHLLlLLLLLLLEEEEELL-LHHHHHHHHHHhhhhllllleeEEELLLEELlll
Query GGSSLELRRGIpDSRTSKDFATVARR-DIELIHEQLADagetgwegftAIFTAPEEIaap  119
ident ||       |    |   |                 | |
Sbjct GGWGVDVLTGK-QQREHRDIDIDFDAqHTQKVIQKLED----------IGYKIEVHW---   71
```

```
DSSP  HHHHHHHHHLL-LLLLLLEEEEEEEHhHHHHHHHHHHH----------LLLEEEEEL---


DSSP  lllLLLEEEEEEeeelleeeEEEEEEEEEL-----------------LLLLlLLLEEEEE
Query gxpVKPRRFTAKlsyrgrafATVPIEVSSV-----------------EAGNaDQFDTLTS  162
ident                                                     | |
Sbjct --xPSRXELKHE--------EYGYLDIHPInlnddgsitqanpeggnYVFQnDWFSETNY  121
DSSP  --lLLLEEEEEL--------LLEEEEEEEEeellllleeeellllleEEELhHHEEEEEE


DSSP  LhhhhlllllLEEEEELLHHHHHHHHhhHHHLlllllllllLLHHHHHHHHHHHHHLlhhh
Query DalglvgvpaAVAVPCXTIPWQIAQKlhAVTAvleepkandRAHDLVDLQLLEGLLldad  222
ident               ||     |                      |  |   |
Sbjct K---------DRKIPCISKEAQLLFH--SGYD--------lTETDHFDIKNLKSIT----  158
DSSP  L---------LEEEEEELHHHHHHHL--LLLL--------lLHHHHHHHHHHHHLL----


DSSP  hhhhhhhhhhhhhhhlllllllllllllhhhhhhhhhllllllllllhhhhhhhhhh
Query lxptrsaciaifearaqhpwpprvatlphwpliyagalegldhlelartvdaaaqavqrf  282
ident
Sbjct ------------------------------------------------------------  158
DSSP  ------------------------------------------------------------


DSSP  hhhhhhllll
Query varidratkr  292
ident
Sbjct ----------  158
DSSP  ----------
```

No 17: Query=mol1A Sbjct=4wh5A Z-score=9.1

[back to top]

```
DSSP  llllllllllhhhhhhhhhhhhhhllllhhhhhHHHHHHHHHHHHHHLLLlllllLLEEEE
Query aapysspptnlrslrdrltqvaerqgvvfgrlqRHVAXIVVAQFAATLTddtgaPLLLVK   60
ident
Sbjct ------------------------------nnvTEKELFYILDLFEHXK-----VTYWLD   25
DSSP  ------------------------------lllLHHHHHHHHHHHHHHL-----LLEEEE


DSSP  HHHHHHH-HHLLllLLLLLLEEEEELL-LHHHHHHHHHHhhhhlllleeEEELLLEELll
Query GGSSLEL-RRGIpdSRTSKDFATVARR-DIELIHEQLADagetgwegftAIFTAPEEIaa  118
ident ||             |   |                | |
Sbjct GGWGVDVlTGKQ--QREHRDIDIDFDAqHTQKVIQKLED----------IGYKIEVHW--   71
DSSP  HHHHHHHhHLLL--LLLLLEEEEEEEEHhHHHHHHHHHH----------LLLEEEEEE--


DSSP  llllLLLEEEEEEeeelleeeEEEEEEEEEL-----------------LLLLlLLLEEEE
Query pgxpVKPRRFTAKlsyrgrafATVPIEVSSV-----------------EAGNaDQFDTLT  161
ident                                                      | |
Sbjct ---xPSRXELKHE--------EYGYLDIHPInlnddgsitqanpeggnYVFQnDWFSETN  120
DSSP  ---lLLLEEEEEL--------LLEEEEEEEEeellllleeeellllleEEELhHHEEEEE


DSSP  ELhhhhlllllLEEEEELLHHHHHHHHhhHHHLlllllllllLLHHHHHHHHHHHHHllhh
Query SDalglvgvpaAVAVPCXTIPWQIAQKlhAVTAvleepkandRAHDLVDLQLLEGLLlda  221
ident                ||     |                      |  |   |
Sbjct YK---------DRKIPCISKEAQLLFH--SGYD--------lTETDHFDIKNLKSI----  157
DSSP  EL---------LEEEEEELHHHHHHHL--LHHH--------lLHHHHHHHHHHHHLL----


DSSP  hhhhhhhhhhhhhhhhlllllllllllllhhhhhhhhhllllllllllhhhhhhhhh
Query dlxptrsaciaifearaqhpwpprvatlphwpliyagalegldhlelartvdaaaqavqr  281
ident
Sbjct ------------------------------------------------------------  157
DSSP  ------------------------------------------------------------


DSSP  hhhhhhhllll
Query fvaridratkr  292
ident
Sbjct ----------t  158
DSSP  ----------l
```

No 18: Query=mol1A Sbjct=4e8jA Z-score=9.1

[back to top](#)

```
DSSP  lllllllllhhhhhhhhhhhhhhllllhhhhhHHHHHHHHHHHHHHLLLlllllLLEEEE
Query aapysspptnlrslrdrltqvaerqgvvfgrlqRHVAXIVVAQFAATLTddtgaPLLLVK   60
ident                                                               
Sbjct ------------------------------nnvTEKELFYILDLFEHXK-----VTYWLD   25
DSSP  ------------------------------lllLHHHHHHHHHHHHHHL-----LLEEEE


DSSP  HHHHHHH-HHLLllLLLLLLLEEEEEELL-LHHHHHHHHHHhhhhlllleeEEELLLEELll
Query GGSSLEL-RRGIpdSRTSKDFATVARR-DIELIHEQLADagetgwegftAIFTAPEEIaa  118
ident ||             |   |                | |                     
Sbjct GGWGVDVlTGKQ--QREHRDIDIDFDAqHTQKVIQKLED----------IGYKIEVHW--   71
DSSP  HHHHHHHhHLLL--LLLLLEEEEEEEEHhHHHHHHHHHHH----------LLLEEEEEE--


DSSP  llllLLLEEEEEEeeelleeeEEEEEEEEEL-----------------LLLLlLLLEEEE
Query pgxpVKPRRFTAKlsyrgrafATVPIEVSSV-----------------EAGNaDQFDTLT  161
ident                                                      | |    
Sbjct ---xPSRXELKHE--------EYGYLDIHPInlnddgsitqanpeggnYVFQnDWFSETN  120
DSSP  ---lLLEEEEEEL--------LLEEEEEEEEeellllleeeellllleEEELhHHEEEEE


DSSP  ELhhhhllllLEEEEEELLHHHHHHHHhhHHHLlllllllllLLHHHHHHHHHHHHllhh
Query SDalglvgvpaAVAVPCXTIPWQIAQKlhAVTAvleepkandRAHDLVDLQLLEGLllda  221
ident                ||     |                      |  |   |       
Sbjct YK---------DRKIPCISKEAQLLFH--SGYD--------lTETDHFDIKNLKSI----  157
DSSP  EL---------LEEEEEELHHHHHHHL--LHHH--------lLHHHHHHHHHHHHLL----


DSSP  hhhhhhhhhhhhhhhhlllllllllllllhhhhhhhhhllllllllllhhhhhhhhh
Query dlxptrsaciaifearaqhpwpprvatlphwpliyagalegldhlelartvdaaaqavqr  281
ident                                                               
Sbjct ------------------------------------------------------------  157
DSSP  ------------------------------------------------------------


DSSP  hhhhhhhllll
Query fvaridratkr  292
ident            
Sbjct ----------t  158
DSSP  ----------l
```

No 19: Query=mol1A Sbjct=4e8iA Z-score=9.1

[back to top](#)

```
DSSP  lllllllllhhhhhhhhhhhhhhllllhhhhhHHHHHHHHHHHHHHLLLlllllLLEEEE
Query aapysspptnlrslrdrltqvaerqgvvfgrlqRHVAXIVVAQFAATLTddtgaPLLLVK   60
ident                                                               
Sbjct ------------------------------nnvTEKELFYILDLFEHXK-----VTYWLD   25
DSSP  ------------------------------lllLHHHHHHHHHHHHHHLL-----LLEEE


DSSP  HHHHHHHHHLLlLLLLLLLEEEEEELL-LHHHHHHHHHHhhhhlllleeEEELLLEELlll
Query GGSSLELRRGIpDSRTSKDFATVARR-DIELIHEQLADagetgwegftAIFTAPEEIaap  119
ident ||       |    |   |                | |                      
Sbjct GGWGVDVLTGK-QQREHRDIDIDFDAqHTQKVIQKLED----------IGYKIEVHW---   71
DSSP  HHHHHHHHHLL-LLLLLLEEEEEEEEHhHHHHHHHHHHH----------LLLEEEEEE---


DSSP  lllLLLEEEEEEeeelleeeEEEEEEEEEL-----------------LLLLLLLLEEEEE
Query gxpVKPRRFTAKlsyrgrafATVPIEVSSV-----------------EAGNADQFDTLTS  162
ident                                                     | |     
Sbjct --xPSRXELKHE--------EYGYLDIHPInlnddgsitqanpeggnYVFQNDWFSETNY  121
DSSP  --lLLEEEEEEL--------LLEEEEEEEEeellllleeeellllleEEELHHHEEEEEE


DSSP  LhhhhllllLEEEEEELLHHHHHHHHhhHHHLlllllllllLLHHHHHHHHHHHHHLlhhh
Query DalglvgvpaAVAVPCXTIPWQIAQKlhAVTAvleepkandRAHDLVDLQLLEGLLldad  222
ident               ||     |                      |  |   |       
Sbjct K---------DRKIPCISKEAQLLFH--SGYD--------lTETDHFDIKNLKSIT----  158
DSSP  L---------LEEEEEELHHHHHHHL--LLLL--------lLHHHHHHHHHHHHHLL----
```

```
DSSP  hhhhhhhhhhhhhhhlllllllllllllhhhhhhhhhlllllllllllhhhhhhhhhhhh
Query lxptrsaciaifearaqhpwpprvatlphwpliyagalegldhlelartvdaaaqavqrf  282
ident
Sbjct ------------------------------------------------------------  158
DSSP  ------------------------------------------------------------
```

```
DSSP  hhhhhhllll
Query varidratkr  292
ident
Sbjct ----------  158
DSSP  ----------
```

No 20: Query=mol1A Sbjct=4wseB Z-score=8.9

back to top

```
DSSP  -------------llllllllllhHHHHHHHHHhhhhllllhHHHHHHHHHHHHHHHHHL
Query -------------aapysspptnlRSLRDRLTQvaerqgvvfGRLQRHVAXIVVAQFAAT   47
ident                                                     |   |
Sbjct ekyqtyyttneyqivkeklpdiirDAEIKASEV------lepTIYEKRAIMEVIKDFIRD   54
DSSP  llllllllllhhhhhhhhhhhhhhHHHHHHHHH------lllLHHHHHHHHHHHHHHHHH
```

```
DSSP  LlllllllLEEEEHHHHHHHHHL---LLLLLL----lLLEEEEELLlHHHHHHHHHHHHH
Query LtddtgapLLLVKGGSSLELRRG---IPDSRT----sKDFATVARRdIELIHEQLADAGE  100
ident            | ||  |          |         |               |
Sbjct H-------QRKVYGGTALNEALKqvnPKDAIYdnysfSDIEFYSPT-PVQDLVDLCNILY  106
DSSP  H-------LLEELHHHHHHHHHHhhlLLLLLLlllllLLEEEEELL-HHHHHHHHHHHHH
```

```
DSSP  HLllleeeEELLLEELlllllllLLEEEEEEEeelleeeeEEEEEEEELL-LLLLL-LLE
Query TGwegftaIFTAPEEIaapgxpvKPRRFTAKLsyrgrafaTVPIEVSSVE-AGNAD-QFD  158
ident
Sbjct RK--gykfVQGKDAQH-------EETYSIFVN-------fQLYCDITYSPtRVFYGiKTI  150
DSSP  HL--llllEEEEELLL-------LLEEEEEEL-------lLEEEEEEELLhHHHHHlLEE
```

```
DSSP  EEEelhhhhlllllleEEEELLHHHHHHHHHHHHHHLLLlllLLLL-LHHHHHHHHHHH-
Query TLTsdalglvgvpaavAVPCXTIPWQIAQKLHAVTAVLEepkANDR-AHDLVDLQLLEG-  216
ident                               |  |   |    |  |          |
Sbjct EID-------------GINYTDPHFMLIDYLRMVNQPLT--aAGQRwEKAFERMYRLLKd  195
DSSP  EEL-------------LEEEELHHHHHHHHHHHHLLHHH--hHHHHhHHHHHHHHHHHHh
```

```
DSSP  HLLHH-------------------------------------------------------
Query LLLDA-------------------------------------------------------  221
ident
Sbjct YPIEDfdkrldipeppeeiqsyisrikteflsdnklnesflisgieaynfyirhaassln  255
DSSP  LLEELlllllllllllhhhhhhhhhhhhhllllllllllleeehhhhhhhhhhhhhlllll
```

```
DSSP  -----------------------------------hhhhhhhhhhhHHHH----------
Query -----------------------------------dlxptrsaciaIFEA----------  236
ident                                                |
Sbjct nfianvpfselisvnyredvkntynflrmivedkekisvdeyfplfQFTGystvikyddh  315
DSSP  llllllleeeeellhhhhhhhhhhhhhhhlllhhheeeeeeellllLLLLleeeeeelle
```

```
DSSP  --------hLLLL-------------lLLLLlllllhhhhhhhhhlllllllllllhhhh
Query --------rAQHP-------------wPPRVatlphwpliyagalegldhlelartvdaa  275
ident
Sbjct piiriyegdGYCIpnvktvktkyeykyVSFQ-----------------------------  346
DSSP  eeeeeeellLLLEeeeeeelllleeeeELHH-----------------------------
```

```
DSSP  hhhhhhHHHHH----------------hHLLL----------------------------
Query aqavqrFVARI----------------dRATK----------------------------  291
ident
Sbjct ----yvLMILYinkfrahldknkpmyfnYGIAisnlvkarniyldqtgksvldntvfkef  402
DSSP  ----hhHHHHHhhhhhhhlllhhhhhhhHHHHhhhhhhhhhhhhhhhhllllllllllll
```

```
DSSP  ------------------------------------------------------------
Query ------------------------------------------------------------  291
ident
```

```
Sbjct rtnctgntisftrmnrlrllekrkqgkqtsfvytpedffkkdletqakldpskarfknts  462
DSSP  llleellllhhhhhhhhhhhhhhllllllleelhhhhllllhhhhhhllllllleeelll
```

```
DSSP  --------------------------------l
Query --------------------------------r  292
ident
Sbjct gnkimvpkyllfkidnngniednihseeaeise  495
DSSP  llllllhhhlleeellllleeellllllleeell
```

No 21: Query=mol1A Sbjct=4hx0A Z-score=8.5

back to top

```
DSSP  lllllllllhhhhhhhhhhhhhhllllhhHHHHHHHHHHHHHHHHLLLlllllLLEEEE
Query aapysspptnlrslrdrltqvaerqgvvfgRLQRHVAXIVVAQFAATLTddtgaPLLLVK   60
ident                               |                           |
Sbjct -----------------------hhhhhmiRPEYLRVLRKIYDRLKNEK-----VNWVVT   32
DSSP  -----------------------lhhhlllLHHHHHHHHHHHHHHHLLLL-----LLEEEE
```

```
DSSP  HHHHHHHHHlllLLLLLLLEEEEELLL-HHHHHHHHHhhhhhllleeeEELL-LEELLL
Query GGSSLELRRgipDSRTSKDFATVARRD-IELIHEQLAdagetgwegftaIFTA-PEEIAA  118
ident |  |  |           |            |
Sbjct GSLSFALQG---VPVEVHDIDIQTDEEgAYEIERIFS-----------eFVSKkVRFSST   78
DSSP  HHHHHHHLL---LLLLLLLEEEEELHHhHHHHHHHHLH-----------hHEEEeEEEEEL
```

```
DSSP  LLLlLLLEEEEEEeeelleeeEEEEEEEEEL------------LLLLLLLLEEEEELhhh
Query PGXpVKPRRFTAKlsyrgrafATVPIEVSSV------------EAGNADQFDTLTSDalg  166
ident                           |
Sbjct EKI-CSHFGELII--------DGIKVEIMGDirkrledgtwedPVDLNKYKRFVETH---  126
DSSP  LLE-EEEEEEEEE--------LLEEEEEEEEeeellllllllLLLHHHHEEEEEEL---
```

```
DSSP  hlllllLEEEEELLHHHHHHHHHHHHHLL--LLLLLlllllhhhhhhhhhhhllhhhhh
Query lvgvpaAVAVPCXTIPWQIAQKLHAVTAV--LEEPKandrahdlvdlqllegllldadlx  224
ident           |           |            |
Sbjct ------GMKIPVLSLEYEYQAYLKLGRVEkaETLRK------------------------  156
DSSP  ------LEEEEEELHHHHHHHHHHHHHHHHhhHHHHH------------------------
```

```
DSSP  hhhhhhhhhhhhlllllllllllllhhhhhhhhhllllllllllhhhhhhhhhhhh
Query ptrsaciaifearaqhpwpprvatlphwpliyagalegldhlelartvdaaaqavqrfva  284
ident
Sbjct ------------------------------------------------------------  156
DSSP  ------------------------------------------------------------
```

```
DSSP  hhhhllll
Query ridratkr  292
ident
Sbjct ---wlner  161
DSSP  ---hhhll
```

No 22: Query=mol1A Sbjct=4turA Z-score=7.4

back to top

```
DSSP  ------------------------------------------------------------
Query ------------------------------------------------------------    0
ident
Sbjct pqetlnggitdmltelanfeknvsqaihkynayrkaasviakyphkiksgaeakklpgvg   60
DSSP  llllllhhhhhhhhhhhhhhhhlllllhhhhhhhhhhhhhhhlllllllllhhhhhllllll
```

```
DSSP  ---------------------------------------------------lllllllll
Query ---------------------------------------------------aapysspppt    9
ident
Sbjct tkiaekideflatgklrklekirqddtsssinfltrvsgigpsaarkfvdegiktledlr  120
DSSP  hhhhhhhhhhhhhlllhhhhhhhhlhhhhhhhhhlllllllhhhhhhhhhlllllhhhhh
```

```
DSSP  lhhhhhhhhhhhhhhllllhhhhhHHHHHHHHHHHHHH-LLLLllLLLLEEEEHHHHHHHh
```

```
Query nlrslrdrltqvaerqgvvfgrlqRHVAXIVVAQFAA-TLTDdtGAPLLLVKGGSSLELr   68
ident                                                   | |
Sbjct knedklnhhqriglkyfgdfekriPREEMLQMQDIVLnEVKKvdSEYIATVCGSFRRGA-  179
DSSP  hlhhhllhhhhhhhhlhhhhllleEHHHHHHHHHHHHhHHHHhlLLLEEEELHHHHLLL-


DSSP  hllllllLLLLEEEEEL----------lLHHHHHHHHHHHHhhlllleeEEELLLEElll
Query rgipdsrTSKDFATVAR----------rDIELIHEQLADAGetgwegftAIFTAPEEiaa  118
ident         | |                       |||             |
Sbjct ------eSSGDMDVLLThpsftsesqpkLLHQVVEQLQKVH--------FITDTLSK---  222
DSSP  ------lEELLEEEEEEllllllllllhHHHHHHHHHHHLL--------LEEEEEEE---


DSSP  llllLLLEEEEEEEEEL--LEEEEEEEEEEEEL-LLLLlllleeeeelhhhhllllllee
Query pgxpVKPRRFTAKLSYR--GRAFATVPIEVSSV-EAGNadqfdtltsdalglvgvpaava  175
ident              |             |
Sbjct ---gETKFMGVCQLPSKndEKEYPHRRIDIRLIpKDQY----------------------  257
DSSP  ---lLLEEEEEEELLLLllLLLLLLEEEEEEELlHHHH----------------------


DSSP  eeellhHHHHhhhhhhhhllllllllllllHHHH-------------------hhhhhhh
Query vpcxtiPWQIaqklhavtavleepkandraHDLV-------------------dlqlleg  216
ident                                |
Sbjct -----yCGVL----------------yftgSDIFnknmrahalekgftineytirplgvt  296
DSSP  -----hHHHH----------------hhhlLHHHhhhhhhhhhhlleeelllleeellll


DSSP  hllhHHHHHhhHHHHhhhhhhlllLLLLLlllllhhhhhhhhhllllllllllllhhhhh
Query llldADLXPtrSACIaifearaqhPWPPRvatlphwpliyagalegldhlelartvdaaa  276
ident       |
Sbjct gvagEPLPV--DSEK-----difdYIQWK-------------------------------  318
DSSP  llllLLLLL--LLHH-----hhhhHLLLL-------------------------------


DSSP  hhhhhhhhhhhhllll
Query qavqrfvaridratkr  292
ident
Sbjct -------yrepkdrse  327
DSSP  -------lllhhhlll
```

No 23: Query=mol1A Sbjct=4p37A Z-score=7.3

back to top

```
DSSP  ------------------llllllllllhhHHHHHHHHHhhhllllhhhhhhHHHHHHHH
Query ------------------aapyssppptnlrSLRDRLTQVaerqgvvfgrlqrHVAXIVVA   42
ident                                                      | |
Sbjct nrnrklsyqeyyvdgdyeevrkklpeiikqARIKASQVX------eptiyekRVVXEIIK   54
DSSP  llllllllllllllhhhhhhhhhhhhhhhhHHHHHHHHL------lllhhhhHHHHHHHH


DSSP  HHHHLLlllllllLEEEEHHHHHHHHHL---LLLLLL----lLLEEEEELLlHHHHHHHH
Query QFAATLtddtgapLLLVKGGSSLELRRG---IPDSRT----sKDFATVARRdIELIHEQL   95
ident  |              | ||  |          |         |               |
Sbjct DFIRDK-------GRKVYGGTALNETIKkknPEDAIYdsylfSDIEFYSPT-PVPDLKEL  106
DSSP  HHHHHH-------LLEEEHHHHHHHHHHhhlHHHLLLlllllLLEEEEELL-HHHHHHHH


DSSP  HHHHHHLllleeeEELLLEELlllllllLLEEEEEEeeelleeeeEEEEEEEEL--llll
Query ADAGETGwegftaIFTAPEEIaapgxpvKPRRFTAKlsyrgrafaTVPIEVSSV--eagn  153
ident  |                                                   |
Sbjct CDILYHK--gydpVQGKEAQH-------EETYSIFV-------nlQLYCDITYVptkvyh  150
DSSP  HHHHHHL--llllEEEEELLL-------LLEEEEEE-------llEEEEEEEELlhhhhh


DSSP  lLLLEEEEelhhhhlllllleEEEELLHHHHHHHHHHHHHLLLLlllLLLLLHHHHHHHH
Query aDQFDTLTsdalglvgvpaavAVPCXTIPWQIAQKLHAVTAVLEepkANDRAHDLVDLQL  213
ident                                    |      |
Sbjct gIKTIEID-------------GINYTHPHFXLIDYLRXINQPLT-aaEQRWEKAFDRXYV  196
DSSP  hLLEEEEL-------------LEEEELHHHHHHHHHHHHLLHHH-hhHHHHHHHHHHHHH


DSSP  HHH-HLLHH---------------------------------------------------
Query LEG-LLLDA---------------------------------------------------  221
ident |
Sbjct LLKnYPXEKydnsxritsprddiqxyigkvksefxkipeiqesclisgfdaynffirhax  256
DSSP  HHHhLLEELllllllllllllhhhhhhhhhhhhlhhhlhhhhlleeelhhhhhhhhhhhh
```

```
DSSP  -----------------------------------------hhhhhhhhhhhHHHH----
Query -----------------------------------------dlxptrsaciaIFEA----  236
ident                                                      |
Sbjct gdrslknfitvlpfxelisvkykdtveklynflrekvvnpdlitideyfplfQFTGysvs  316
DSSP  lllllhhhlllllleeeeellhhhhhhhhhhhhhhhlllhhheeeeeeellllLLLLleee


DSSP  ---------------hLLLL-----------LLLLllllllhhhhhhhhhllllllllll
Query ---------------rAQHP-----------WPPRvatlphwpliyagalegldhlelar  270
ident                    |
Sbjct inydgipivkvyeadgYCVPdikttsgyryvSYQY-------------------------  351
DSSP  eeelleeeeeeeellllLLEEeeellllleeeLHHH-------------------------


DSSP  lhhhhhhhhhhHHHHH----------------hHLLL-----------------------
Query tvdaaaqavqrFVARI----------------dRATK-----------------------  291
ident
Sbjct ----------iLXIXYiskfkahldknkexyfnYGIAisnlvqarnsylnqknigvindt  401
DSSP  ----------hHHHHHhhhhhhhhhlllhhhhhhHHHHhhhhhhhhhhhhhhhhllllllll


DSSP  ------------------------------------------------------------
Query ------------------------------------------------------------  291
ident
Sbjct vfsefrigcigttvsytrxsrlrxlekkkqgkviqfvytpkqyfsqtpeqqnnfdesxkk  461
DSSP  lllllllleellllllhhhhhhhhhhhhhhhllllllleelhhhhhlllhhhhhhhhhhhh


DSSP  ------------------------------------l
Query ------------------------------------r  292
ident
Sbjct yrfkntsgnkitipknllfkidergniseeisteeay  498
DSSP  lllllllllllllhhhlleeellllleeellllllll
```

No 24: Query=mol1A Sbjct=5j29A Z-score=7.3

back to top

```
DSSP  ------------------------------------------------------------
Query ------------------------------------------------------------    0
ident
Sbjct tlnggitdmltelanfeknvsqaihkynayrkaasviakyphkiksgaeakklpgvgtki   60
DSSP  lllhhhhhhhhhhhhhhhhllllhhhhhhhhhhhhhhhlllllllllhhhhllllllllhhh


DSSP  ------------------------------------------------llllllllllhh
Query ------------------------------------------------aapysspptnlr   12
ident
Sbjct aekideflatgklrklekirqddtsssinfltrvsgigpsaarkfvdegiktledlrkne  120
DSSP  hhhhhhhhhhlllhhhhhhhhlhhhhhhhhhlllllllhhhhhhhhhlllllhhhhhhlh


DSSP  hhhhhhhhhhhhllllhhhhhHHHHHHHHHHHHH-LLLLllLLLLEEEEHHHHHHHhhll
Query slrdrltqvaerqgvvfgrlqRHVAXIVVAQFAA-TLTDdtGAPLLLVKGGSSLELrrgi   71
ident                                                |  |
Sbjct dklnhhqriglkyfgdfekriPREEMLQMQDIVLnEVKKvdSEYIATVCGSFRRGA----  176
DSSP  hhllhhhhhhhhlllhhhlleEHHHHHHHHHHHHhHHHHhlLLLEEEELHHHHLLL----


DSSP  llllLLLLEEEEEL------------lLHHHHHHHHHHHHhhlllleeEEELLLEEllll
Query pdsrTSKDFATVAR------------rDIELIHEQLADAGetgwegftAIFTAPEEiaap  119
ident      | |                         |||             |
Sbjct ---eSSGDMDVLLThpsftsestkqpkLLHQVVEQLQKVH--------FITDTLSK----  221
DSSP  ---lEELLEEEEEElllee lleellllHHHHHHHHHHHHLL--------LEEEEEEE----


DSSP  lllLLLEEEEEEEEEL--LEEEEEEEEEEEEL-LLLLlllleeeeelhhhhllllllleee
Query gxpVKPRRFTAKLSYR--GRAFATVPIEVSSV-EAGNadqfdtltsdalglvgvpaavav  176
ident             |             |
Sbjct --gETKFMGVCQLPSKndEKEYPHRRIDIRLIpKDQY-----------------------  256
DSSP  --lLLEEEEEELLLLLllLLLLLLEEEEEEEELlHHHH-----------------------


DSSP  eellhHHHHhhhhhhhhlllllllllllHHHH-------------------hhhhhhhh
Query pcxtiPWQIaqklhavtavleepkandraHDLV-------------------dlqllegl  217
```

```
ident                               |
Sbjct ----yCGVL----------------yftgSDIFnknmrahalekgftineytirplgvtg  296
DSSP  ----hHHHH----------------hhhlLHHHhhhhhhhhhhhleeellllleeeeelll


DSSP  llhHHHHHhhHHHHhhhhhhlllLLLLLLlllllhhhhhhhhhlllllllllllhhhhhh
Query lldADLXPtrSACIaifearaqhPWPPRVatlphwpliyagalegldhlelartvdaaaq  277
ident      |
Sbjct vagEPLPV--DSEK-----difdYIQWKY-------------------------------  318
DSSP  eelLLLLL--LLHH-----hhhhHLLLLL-------------------------------


DSSP  hhhhhhhhhhhllll
Query avqrfvaridratkr  292
ident
Sbjct -------repkdrse  326
DSSP  -------llhhhlll
```

No 25: Query=mol1A Sbjct=4p2hA Z-score=7.3

back to top

```
DSSP  ------------------------------------------------------------
Query ------------------------------------------------------------    0
ident
Sbjct tlnggitdmltelanfeknvsqaihkynayrkaasviakyphkiksgaeakklpgvgtki   60
DSSP  lllhhhhhhhhhhhhhhhhhllllhhhhhhhhhhhhhhhhhllllllllhhhhllllllllhhh


DSSP  ------------------------------------------------llllllllllhh
Query ------------------------------------------------aapysspptnlr   12
ident
Sbjct aekideflatgklrklekirqddtsssinfltrvsgigpsaarkfvdegiktledlrkne  120
DSSP  hhhhhhhhhhlllhhhhhhhhlhhhhhhhhhlllllllhhhhhhhhhlllllhhhhhhllh


DSSP  hhhhhhhhhhhhllllhhhhhHHHHHHHHHHHHHH-LLLLllLLLLLEEEEHHHHHHHhhll
Query slrdrltqvaerqgvvfgrlqRHVAXIVVAQFAA-TLTDdtGAPLLLVKGGSSLELrrgi   71
ident                                                |  |
Sbjct dklnhhqriglkyfgdfekriPREEMLQMQDIVLnEVKKvdSEYIATVCGSFRRGA----  176
DSSP  hhllhhhhhhhhlllhhhlleEHHHHHHHHHHHHHhHHHHhlLLLLEEEELHHHHLLL----


DSSP  llllLLLLEEEEEL----------lLHHHHHHHHHHHHHhhllllleeEEELLLEEllllll
Query pdsrTSKDFATVAR----------rDIELIHEQLADAGetgwegftAIFTAPEEiaapgx  121
ident      | |                       |||             |
Sbjct ---eSSGDMDVLLThpsftsesqpkLLHQVVEQLQKVH--------FITDTLSK------  219
DSSP  ---lEELLEEEEEElllllllllllHHHHHHHHHHHHLL--------LEEEEEEE------


DSSP  lLLLEEEEEEEEEL--LEEEEEEEEEEEEL-LLLLllllleeeeelhhhhlllllleeeee
Query pVKPRRFTAKLSYR--GRAFATVPIEVSSV-EAGNadqfdtltsdalglvgvpaavavpc  178
ident           |             |
Sbjct gETKFMGVCQLPSKndEKEYPHRRIDIRLIpKDQY-------------------------  254
DSSP  lLLEEEEEEELLLLLllLLLLLLLEEEEEEEELlHHHH-------------------------


DSSP  llhHHHHhhhhhhhhlllllllllllHHHH----------------hhhhhhhhllhh
Query xtiPWQIaqklhavtavleepkandraHDLV----------------dlqlleglllda  221
ident                             |
Sbjct --yCGVL----------------yftgSDIFnknmrahalekgftineytirplgvtgva  296
DSSP  --hHHHH----------------hhhlLHHHhhhhhhhhhhhlleeellllleeellllll


DSSP  hhhhhhHHHHHhhhhhlllLLLLLLlllllhhhhhhhhhllllllllllhhhhhhhhh
Query dlxptrSACIAifearaqhPWPPRvatlphwpliyagalegldhlelartvdaaaqavqr  281
ident
Sbjct geplpvDSEKD-----ifdYIQWK------------------------------------  315
DSSP  llllllLLHHH-----hhhHLLLL------------------------------------


DSSP  hhhhhhhllll
Query fvaridratkr  292
ident
Sbjct --yrepkdrse  324
DSSP  --lllhhhlll
```

No 26: Query=mol1A Sbjct=4yn4A Z-score=7.3

back to top

```
DSSP  ------------------------------------------------------------
Query ------------------------------------------------------------     0
ident 
Sbjct pqetlnggitdmltelanfeknvsqaihkynayrkaasviakyphkiksgaeakklpgvg    60
DSSP  llhhhlhhhhhhhhhhhhhhhhllllhhhhhhhhhhhhhhhllllllllhhhhlllllll


DSSP  ---------------------------------------------------lllllllll
Query ---------------------------------------------------aapysppt     9
ident 
Sbjct tkiaekideflatgklrklekirqddtsssinfltrvsgigpsaarkfvdegiktledlr   120
DSSP  hhhhhhhhhhhhhlllhhhhhhhhlhhhhhhhhhlllllllhhhhhhhhhlllllhhhhh


DSSP  lhhhhhhhhhhhhhhllllhhhhhHHHHHHHHHHHHH-LLLLllLLLLEEEEHHHHHHHh
Query nlrslrdrltqvaerqgvvfgrlqRHVAXIVVAQFAA-TLTDdtGAPLLLVKGGSSLELr    68
ident                                                   | |
Sbjct knedklnhhqriglkyfgdfekriPREEMLQMQDIVLnEVKKvdSEYIATVCGSFRRGA-   179
DSSP  hlhhhllhhhhhhhhlhhhhhlleEHHHHHHHHHHHHhHHHHhlLLLEEEELHHHHLLL-


DSSP  hllllllLLLLEEEEEL----------lLHHHHHHHHHHHHhhlllleeEEELLLEElll
Query rgipdsrTSKDFATVAR----------rDIELIHEQLADAGetgwegftAIFTAPEEiaa   118
ident         | |                       |||             |
Sbjct ------eSSGDMDVLLThpsftsesqpkLLHQVVEQLQKVH--------FITDTLSK---   222
DSSP  ------lEELLEEEEEElllllllllllHHHHHHHHHHHLL--------LEEEEEEE---


DSSP  llllLLLEEEEEEEEELL--EEEEEEEEEEEELLLLLLllleeeeelhhhhlllllleee
Query pgxpVKPRRFTAKLSYRG--RAFATVPIEVSSVEAGNAdqfdtltsdalglvgvpaavav   176
ident              |             |
Sbjct ---gETKFMGVCQLPSKNdeKEYPHRRIDIRLIPKDQY----------------------   257
DSSP  ---lLLEEEEEELLLLLLllLLLLLEEEEEEELLHHHH----------------------


DSSP  eellhHHHHhhhhhhhhllllllllllllHHHH-------------------hhhhhhhh
Query pcxtiPWQIaqklhavtavleepkandraHDLV-------------------dlqllegl   217
ident                               |
Sbjct ----yCGVL----------------yftgSDIFnknmrahalekgftineytirplgvtg   297
DSSP  ----hHHHH----------------hlllLHHHhhhhhhllllleeellllleeelllll


DSSP  llhhHHHHhhHHHHHhhhhhlllLLLLLllllllhhhhhhhhhllllllllllllhhhhhh
Query lldaDLXPtrSACIAifearaqhPWPPRvatlphwpliyagalegldhlelartvdaaaq   277
ident      |
Sbjct vagePLPV--DSEKD-----ifdYIQWK--------------------------------   318
DSSP  llllLLLL--LLHHH-----hhhHLLLL--------------------------------


DSSP  hhhhhhhhhhhllll
Query avqrfvaridratkr  292
ident 
Sbjct ------yrepkdrse  327
DSSP  ------lllhhhlll
```

No 27: Query=mol1A Sbjct=4pgqA Z-score=7.3

back to top

```
DSSP  ------------------------------------------------------------
Query ------------------------------------------------------------     0
ident 
Sbjct etlnggitdmltelanfeknvsqaihkynayrkaasviakyphkiksgaeakklpgvgtk    60
DSSP  lllhhhhhhhhhhhhhhhhhhllllhhhhhhhhhhhhhhllllllllhhhhhllllllhh


DSSP  -------------------------------------------------llllllllllh
Query -------------------------------------------------aapyssspptnl    11
ident 
Sbjct iaekideflatgklrklekirqddtsssinfltrvsgigpsaarkfvdegiktledlrkn   120
```

```
DSSP  hhhhhhhhhhhlllhhhhhhhhlhhhhhhhhhllllllhhhhhhhhhlllllhhhhhll


DSSP  hhhhhhhhhhhhhllllhhhhhHHHHHHHHHHHHH-LLLLllLLLLEEEEHHHHHHhhhl
Query rslrdrltqvaerqgvvfgrlqRHVAXIVVAQFAA-TLTDdtGAPLLLVKGGSSLElrrg   70
ident                                                 | |
Sbjct edklnhhqriglkyfgdfekriPREEMLQMQDIVLnEVKKvdSEYIATVCGSFRRG----  176
DSSP  hhhllhhhhhhhhlllhhhlleEHHHHHHHHHHHHhHHHHhlLLLEEEELHHHHLL----


DSSP  lllLLLLLLEEEEEL----------lLHHHHHHHHHHHHhhllllleeEEELLLEEllll
Query ipdSRTSKDFATVAR----------rDIELIHEQLADAGetgwegftAIFTAPEEiaapg  120
ident       | |                       |||             |
Sbjct ---AESSGDMDVLLThpsftsesqpkLLHQVVEQLQKVH--------FITDTLSK-----  220
DSSP  ---LLEELLEEEEEEllllllllllhHHHHHHHHHHHHLL--------LEEEEEEE-----


DSSP  llLLLEEEEEEEEEEL-LEEEEEEEEEEEELLLLLLllleeeeelhhhhllllllleeeeel
Query xpVKPRRFTAKLSYR-GRAFATVPIEVSSVEAGNAdqfdtltsdalglvgvpaavavpcx  179
ident            |            |
Sbjct -gETKFMGVCQLPSKdEKEYPHRRIDIRLIPKDQY-------------------------  254
DSSP  -lLLEEEEEELLLLlLLLLLLEEEEEEEELLHHHH-------------------------


DSSP  lhHHHHhhhhhhhhlllllllllllHHHH-----------------hhhhhhhhllhhh
Query tiPWQIaqklhavtavleepkandraHDLV-----------------dlqllegllldad  222
ident                            |
Sbjct -yCGVL----------------yftgSDIFnknmrahalekgftineytirplgvtgvag  297
DSSP  -hHHHH----------------hlllLHHHhhhhhhhhhllleeellllleeellllllll


DSSP  hhhhhHHHHHhhhhhlllLLLLLllllllhhhhhhhhhllllllllllhhhhhhhhhh
Query lxptrSACIAifearaqhPWPPRvatlphwpliyagalegldhlelartvdaaaqavqrf  282
ident
Sbjct eplpvDSEKD-----ifdYIQWK-------------------------------------  315
DSSP  lllllLLHHH-----hhhHLLLL-------------------------------------


DSSP  hhhhhhllll
Query varidratkr  292
ident
Sbjct -yrepkdrse  324
DSSP  -lllhhhlll
```

No 28: Query=mol1A Sbjct=5j0vA Z-score=7.3

back to top

```
DSSP  ------------------------------------------------------------
Query ------------------------------------------------------------    0
ident
Sbjct apqetlnggitdmltelanfeknvsqaihkynayrkaasviakyphkiksgaeakklpgv   60
DSSP  lllllllhhhhhhhhhhhhhhhhhllllhhhhhhhhhhhhhhhhhlllllllhhhhhlllll


DSSP  ----------------------------------------------------llllllll
Query ----------------------------------------------------aapysspp    8
ident
Sbjct gtkiaekideflatgklrklekirqddtsssinfltrvsgigpsaarkfvdegiktledl  120
DSSP  lhhhhhhhhhhhhhlllhhhhhhhhlhhhhhhhhhlllllllhhhhhhhhhlllllhhhh


DSSP  llhhhhhhhhhhhhhhllllhhhhhHHHHHHHHHHHHH-LLLLllLLLLEEEEHHHHHHh
Query tnlrslrdrltqvaerqgvvfgrlqRHVAXIVVAQFAA-TLTDdtGAPLLLVKGGSSLEl   67
ident                                                    | |
Sbjct rknedklnhhqriglkyfgdfekriPREEMLQMQDIVLnEVKKvdSEYIATVCGSFRRG-  179
DSSP  hllhhhllhhhhhhhhlhhhhhlleEHHHHHHHHHHHHhHHHHhlLLLEEEELLHHHHL-


DSSP  hhllllLLLLLLEEEEEL--------lLHHHHHHHHHHHHhhllllleeEEELLLEEllll
Query rrgipdSRTSKDFATVAR--------rDIELIHEQLADAGetgwegftAIFTAPEEiaap  119
ident          | |                       |||             |
Sbjct ------AESSGDMDVLLThpsftseskLLHQVVEQLQKVH--------FITDTLSK----  221
DSSP  ------LLEELLEEEEEEllllllllllHHHHHHHHHHHHLL--------LEEEEEEE----


DSSP  lllLLLEEEEEEEEEELL--EEEEEEEEEEEEL-LLLLllllleeeeelhhhhllllllleee
```

```
Query gxpVKPRRFTAKLSYRG--RAFATVPIEVSSV-EAGNadqfdtltsdalglvgvpaavav  176
ident             |             |
Sbjct --gETKFMGVCQLPSKNdeKEYPHRRIDIRLIpKDQY-----------------------  256
DSSP  --lLLEEEEEEELLLLLLllLLLLLLEEEEEEEELlHHHH-----------------------


DSSP  eellhHHHHhhhhhhhhlllllllllllHHHH-----------------hhhhhhhhll
Query pcxtiPWQIaqklhavtavleepkandraHDLV-----------------dlqlleglll  219
ident                               |
Sbjct ----yCGVL----------------yftgSDIFnknmrahalekgftineytirplgvtg  296
DSSP  ----hHHHH----------------hhhlLHHHhhhhhhhhhhlleeellllleeellll


DSSP  hhhhhhhhHHHHHhhhhhlllLLLLLllllllhhhhhhhhhllllllllllhhhhhhhh
Query dadlxptrSACIAifearaqhPWPPRvatlphwpliyagalegldhlelartvdaaaqav  279
ident
Sbjct vageplpvDSEKD-----ifdYIQWK----------------------------------  317
DSSP  llllllllLLHHH-----hhhHLLLL----------------------------------


DSSP  hhhhhhhhhllll
Query qrfvaridratkr  292
ident
Sbjct ----yrepkdrse  326
DSSP  ----lllhhhlll
```

No 29: Query=mol1A Sbjct=5j0uA Z-score=7.3

back to top

```
DSSP  ------------------------------------------------------------
Query ------------------------------------------------------------    0
ident
Sbjct apqetlnggitdmltelanfeknvsqaihkynayrkaasviakyphkiksgaeakklpgv   60
DSSP  llllllhhhhhhhhhhhhhhhhllllhhhhhhhhhhhhhhhhhlllllllhhhhhlllll


DSSP  ----------------------------------------------------llllllll
Query ----------------------------------------------------aapysspp    8
ident
Sbjct gtkiaekideflatgklrklekirqddtsssinfltrvsgigpsaarkfvdegiktledl  120
DSSP  lhhhhhhhhhhhhhllllhhhhhhhhlhhhhhhhhhlllllllhhhhhhhhhlllllhhhh


DSSP  llhhhhhhhhhhhhhhllllhhhhhHHHHHHHHHHHHH-LLLLllLLLLEEEEHHHHHHh
Query tnlrslrdrltqvaerqgvvfgrlqRHVAXIVVAQFAA-TLTDdtGAPLLLVKGGSSLEl   67
ident                                                    | |
Sbjct rknedklnhhqriglkyfgdfekriPREEMLQMQDIVLnEVKKvdSEYIATVCGSFRRG-  179
DSSP  hllhhhllhhhhhhhhlhhhhhlleEHHHHHHHHHHHHhHHHHhlLLLEEEEELLHHHHL-


DSSP  hhllllLLLLLLEEEEEL--------lLHHHHHHHHHHHHhhllllleeEEELLLEElll l
Query rrgipdSRTSKDFATVAR--------rDIELIHEQLADAGetgwegftAIFTAPEEiaap  119
ident          | |                     |||             |
Sbjct ------AESSGDMDVLLThpsftseskLLHQVVEQLQKVH--------FITDTLSK----  221
DSSP  ------LLEELLEEEEEEllllllllHHHHHHHHHHHLL--------LEEEEEEE----


DSSP  lllLLLEEEEEEEEEELL--EEEEEEEEEEEEL-LLLLlllleeeeelhhhhllllllleee
Query gxpVKPRRFTAKLSYRG--RAFATVPIEVSSV-EAGNadqfdtltsdalglvgvpaavav  176
ident             |             |
Sbjct --gETKFMGVCQLPSKNdeKEYPHRRIDIRLIpKDQY-----------------------  256
DSSP  --lLLEEEEEEELLLLLLllLLLLLLEEEEEEEELlHHHH-----------------------


DSSP  eellhHHHHhhhhhhhhlllllllllllHHHH-----------------hhhhhhhhll
Query pcxtiPWQIaqklhavtavleepkandraHDLV-----------------dlqlleglll  219
ident                               |
Sbjct ----yCGVL----------------yftgSDIFnknmrahalekgftineytirplgvtg  296
DSSP  ----hHHHH----------------hhhlLHHHhhhhhhhhhhlleeellllleeellll


DSSP  hhhhhhhhHHHHHhhhhhlllLLLLLllllllhhhhhhhhhllllllllllhhhhhhhh
Query dadlxptrSACIAifearaqhPWPPRvatlphwpliyagalegldhlelartvdaaaqav  279
ident
Sbjct vageplpvDSEKD-----ifdYIQWK----------------------------------  317
DSSP  llllllllLLHHH-----hhhHLLLL----------------------------------
```

```
DSSP  hhhhhhhhhllll
Query qrfvaridratkr  292
ident
Sbjct ----yrepkdrse  326
DSSP  ----lllhhhlll
```

No 30: Query=mol1A Sbjct=4mf8A Z-score=7.3

back to top

```
DSSP  ------------------------------------------------------------
Query ------------------------------------------------------------    0
ident
Sbjct pqetlnggitdmltelanfeknvsqaihkynayrkaasviakyphkiksgaeakklpgvg   60
DSSP  llhhhhhhhhhhhhhhhhhhhhhllllhhhhhhhhhhhhhhhhlllllllhhhhlllllll


DSSP  ---------------------------------------------------lllllllll
Query ---------------------------------------------------aapyssppt    9
ident
Sbjct tkiaekideflatgklrklekirqddtsssinfltrvsgigpsaarkfvdegiktledlr  120
DSSP  hhhhhhhhhhhhhlllhhhhhhhhlhhhhhhhhhlllllllhhhhhhhhhlllllhhhhh


DSSP  lhhhhhhhhhhhhhhllllhhhhhHHHHHHHHHHHHHH-LLLLllLLLLEEEEHHHHHHhh
Query nlrslrdrltqvaerqgvvfgrlqRHVAXIVVAQFAA-TLTDdtGAPLLLVKGGSSLElr   68
ident                                                   | |
Sbjct knedklnhhqriglkyfgdfekriPREEMLQMQDIVLnEVKKvdSEYIATVCGSFRRG--  178
DSSP  llhhhllhhhhhhhhlhhhhhlleEHHHHHHHHHHHHHhHHHHhlLLLEEEELHHHHLL--


DSSP  hllllLLLLLLEEEEEL-------lLHHHHHHHHHHHHhhllllleeEEELLLEElllll
Query rgipdSRTSKDFATVAR-------rDIELIHEQLADAGetgwegftAIFTAPEEiaapgx  121
ident         | |                    |||             |
Sbjct -----AESSGDMDVLLThpsftspkLLHQVVEQLQKVH--------FITDTLSK------  219
DSSP  -----LLEELLEEEEEEllllllhHHHHHHHHHHHHLL--------LEEEEEEE------


DSSP  lLLLEEEEEEEEEElleEEEEEEEEEEEEL-LLLLllllleeeeelhhhhlllllleeeeell
Query pVKPRRFTAKLSYrgrAFATVPIEVSSV-EAGNadqfdtltsdalglvgvpaavavpcxt  180
ident           |           |
Sbjct gETKFMGVCQLPS---EYPHRRIDIRLIpKDQY---------------------------  249
DSSP  lLLEEEEEELLLL---LLLLEEEEEEEELlHHHH---------------------------


DSSP  hHHHHhhhhhhhhllllllllllllHHHH------------------hhhhhhhhllhhH
Query iPWQIaqklhavtavleepkandraHDLV------------------dlqlleglllldaD  222
ident                           |
Sbjct yCGVL-----------------yftgSDIFnknmrahalekgftineytirplgtgvageP  293
DSSP  hHHHH-----------------hhhlLHHHhhhhhhhhhhhlleeellllleeellllllllL


DSSP  HHHhhHHHHHhhhhhlllLLLLLllllllhhhhhhhhhlllllllllllhhhhhhhhhhh
Query LXPtrSACIAifearaqhPWPPRvatlphwpliyagalegldhlelartvdaaaqavqrf  282
ident |
Sbjct LPV--DSEKD-----ifdYIQWK-------------------------------------  309
DSSP  LLL--LLHHH-----hhhHLLLL-------------------------------------


DSSP  hhhhhhllll
Query varidratkr  292
ident
Sbjct -yrepkdrse  318
DSSP  -lllhhhlll
```

No 31: Query=mol1A Sbjct=4f5qA Z-score=7.3

back to top

```
DSSP  ------------------------------------------------------------
Query ------------------------------------------------------------    0
ident
```

```
Sbjct tlnggitdmltelanfeknvsqaihkynayrkaasviakyphkiksgaeakklpgvgtki   60
DSSP  lllhhhhhhhhhhhhhhhllllhhhhhhhhhhhhhhhlllllllhhhhhhlllllhhh


DSSP  -----------------------------------------------llllllllllhh
Query -----------------------------------------------aapyssspptnlr   12
ident
Sbjct aekideflatgklrklekirqddtsssinfltrvsgigpsaarkfvdegiktledlrkne  120
DSSP  hhhhhhhhhhlllhhhhhhhhlhhhhhhhhhllllllhhhhhhhhhlllllhhhhhhlh


DSSP  hhhhhhhhhhhhllllhhhhhHHHHHHHHHHHHH-LLLLllLLLLEEEEHHHHHHHHhhll
Query slrdrltqvaerqgvvfgrlqRHVAXIVVAQFAA-TLTDdtGAPLLLVKGGSSLELrrgi   71
ident                                                |  |
Sbjct dklnhhqriglkyfgdfekriPREEMLQMQDIVLnEVKKvdSEYIATVCGSFRRGA----  176
DSSP  hhllhhhhhhhhlhhhhhlleEHHHHHHHHHHHHhHHHLllLLLEEEEEHHHLLLL----


DSSP  llllLLLLEEEEEL--------lLHHHHHHHHHHHHhhlllleeEEELLLEELllllllL
Query pdsrTSKDFATVAR--------rDIELIHEQLADAGetgwegftAIFTAPEEIaapgxpV  123
ident      | |                     |||           |
Sbjct ---eSSGDMDVLLThpsftseskLLHQVVEQLQKVH--------FITDTLSKG------E  219
DSSP  ---lEELLEEEEEEllllllllHHHHHHHHHHHHLL--------LEEEEEEL------L


DSSP  LLEEEEEEEEELLEEEEEEEEEEEEL-LLLLllllleeeeelhhhhllllllleeeeellhH
Query KPRRFTAKLSYRGRAFATVPIEVSSV-EAGNadqfdtltsdalglvgvpaavavpcxtiP  182
ident         |           |
Sbjct TKFMGVCQLPSKEKEYPHRRIDIRLIpKDQY---------------------------yC  252
DSSP  LEEEEEELLLLLLLLLLLEEEEEEELlHHHH---------------------------hH


DSSP  HHHhhhhhhhhlllllllllllHHHHHHH-------------------hhhhhllhHHH
Query WQIaqklhavtavleepkandraHDLVDLQ-------------------llegllldADL  223
ident                         |                                  |
Sbjct GVL----------------yftgSDIFNKNmkahalekgftineytirplgvtgvagEPL  296
DSSP  HHH----------------hhhlLHHHHHHhhhhhhllleeellllleeellllllllLLL


DSSP  HHhhHHHHhhhhhhlllLLLLLLlllllhhhhhhhhhlllllllllllhhhhhhhhhhh
Query XPtrSACIaifearaqhPWPPRVatlphwpliyagalegldhlelartvdaaaqavqrfv  283
ident
Sbjct PV--DSEK-----difdYIQWKY-------------------------------------  312
DSSP  LL--LLHH-----hhhhHHLLLLL------------------------------------


DSSP  hhhhhllll
Query aridratkr  292
ident
Sbjct -repkdrse  320
DSSP  -llhhhlll
```

No 32: Query=mol1A Sbjct=4f5nA Z-score=7.3

back to top

```
DSSP  ------------------------------------------------------------
Query ------------------------------------------------------------    0
ident
Sbjct qetlnggitdmltelanfeknvsqaihkynayrkaasviakyphkiksgaeakklpgvgt   60
DSSP  lhhhhhhhhhhhhhhhhhhhhllllhhhhhhhhhhhhhhhllllllllhhhhhhllllllh


DSSP  -------------------------------------------------lllllllllll
Query -------------------------------------------------aapyssspptn   10
ident
Sbjct kiaekideflatgklrklekirqddtsssinfltrvsgigpsaarkfvdegiktledlrk  120
DSSP  hhhhhhhhhhhhlllhhhhhhhhllhhhhhhhhhhllllllhhhhhhhhhlllllhhhhhl


DSSP  hhhhhhhhhhhhhhllllhhhhhHHHHHHHHHHHHH-LLLLllLLLLEEEEHHHHHHHHhh
Query lrslrdrltqvaerqgvvfgrlqRHVAXIVVAQFAA-TLTDdtGAPLLLVKGGSSLELrr   69
ident                                                  |  |
Sbjct nedklnhhqriglkyfgdfekriPREEMLQMQDIVLnEVKKvdSEYIATVCGSFRRGA--  178
DSSP  lhhhllhhhhhhhhlhhhhhlleEHHHHHHHHHHHHhHHHHhlLLLEEEEEHHHLLLL--
```

```
DSSP  llllllLLLLEEEEEL---------lLHHHHHHHHHHHHhhlllleeEEELLLEELllll
Query gipdsrTSKDFATVAR---------rDIELIHEQLADAGetgwegftAIFTAPEEIaapg  120
ident        | |                     |||             |
Sbjct -----eSSGDMDVLLThpsftsespkLLHQVVEQLQKVH--------FITDTLSKG----  221
DSSP  -----lEELLEEEEEElllllllllHHHHHHHHHHHLL--------LEEEEEEEL----


DSSP  llLLLEEEEEEEEEELLEEEEEEEEEEEELLLLLLllleeeeelhhhhlllllleeeeell
Query xpVKPRRFTAKLSYRGRAFATVPIEVSSVEAGNAdqfdtltsdalglvgvpaavavpcxt  180
ident            |           |
Sbjct --ETKFMGVCQLPSDEKEYPHRRIDIRLIPKDQY--------------------------  253
DSSP  --LLEEEEEEELLLLLLLLLLLEEEEEEEELLHHHH--------------------------


DSSP  hHHHhhhhhhhhlllllllllllhhhhHHHHHH---------------------hhl
Query iPWQIaqklhavtavleepkandrahdlvDLQLLE---------------------gll  218
ident
Sbjct yCGVL--------------------yftgSDIFNKnmkahalekgftineytirplgvtg  293
DSSP  hHHHH--------------------hhhlLHHHHHhhhhhhhhlleeellllleeelllll


DSSP  lhhhhhhhhHHHhhhhhhllllLLLLLlllllhhhhhhhhhlllllllllllhhhhhhh
Query ldadlxptrSACIaifearaqhpWPPRVatlphwpliyagalegldhlelartvdaaaqa  278
ident          |
Sbjct vageplpvdSEKD-------ifdYIQWK---------------------------------  314
DSSP  lllllllllLHHH-------hhhHLLLL---------------------------------


DSSP  hhhhhhhhhhllll
Query vqrfvaridratkr  292
ident
Sbjct -----yrepkdrse  323
DSSP  -----lllhhhlll
```

No 33: Query=mol1A Sbjct=2bpgB Z-score=7.3

back to top

```
DSSP  ------------------------------------------------------------
Query ------------------------------------------------------------    0
ident
Sbjct etlnggitdmlvelanfeknvsqaihkynayrkaasviakyphkiksgaeakklpgvgtk   60
DSSP  llllhhhhhhhhhhhhhhhhllllhhhhhhhhhhhhhhhllllllllhhhhhllllllhh


DSSP  -------------------------------------------------llllllllllh
Query -------------------------------------------------aapysspptnl   11
ident
Sbjct iaekideflatgklrklekirqddtsssinfltrvtgigpsaarklvdegiktledlrkn  120
DSSP  hhhhhhhhhhllllhhhhhhhhlhhhhhhhhhhlllllhhhhhhhhhlllllhhhhhhl


DSSP  hhhhhhhhhhhhhllllhhhhhHHHHHHHHHHHHH-LLLLLLLLLLEEEEHHHHHHHhhhl
Query rslrdrltqvaerqgvvfgrlqRHVAXIVVAQFAA-TLTDDTGAPLLLVKGGSSLElrrg   70
ident                                                 | |
Sbjct edklnhhqriglkyfedfekriPREEMLQMQDIVLnEVKKLDPEYIATVCGSFRRG----  176
DSSP  hhhllhhhhhhhhhhhhhhhlleEHHHHHHHHHHHHhHHHHHLLLLEEEELHHHHLL----


DSSP  lllLLLLLLLEEEEEL------------lLHHHHHHHHHHHHhhlllleeEEELLLEELll
Query ipdSRTSKDFATVAR------------rDIELIHEQLADAGetgwegftAIFTAPEEIaa  118
ident       | |                         |||             |
Sbjct ---AESSGDMDVLLThpnftsesskqpkLLHRVVEQLQKVR--------FITDTLSKG--  223
DSSP  ---LLEELLEEEEEElllllllllllhHHHHHHHHHHHLLL--------LLLLEEEEL--


DSSP  llllLLLEEEEEEEEEELLeEEEEEEEEEEEL-LLLLlllleeeeelhhhhllllllleeee
Query pgxpVKPRRFTAKLSYRGrAFATVPIEVSSV-EAGNadqfdtltsdalglvgvpaavavp  177
ident              |           |
Sbjct ----ETKFMGVCQLPSEN-EYPHRRIDIRLIpKDQY------------------------  254
DSSP  ----LLEEEEELLLLLLL-LLLLLEEEEEELlHHHH------------------------


DSSP  ellhHHHHhhhhhhhhlllllllllllHHHHHHH-------------------hhhhhl
Query cxtiPWQIaqklhavtavleepkandraHDLVDLQ-------------------llegll  218
ident                              |
Sbjct ---yCGVL----------------yftgSDIFNKNmrahalekgftineytirplgvtgv  295
```

```
DSSP  ---hHHHH----------------hhhlLLLHHHHhhhhhhhlleellllllleellllll


DSSP  lhHHHHhhHHHHHhhhhhhllllLLLLLllllhhhhhhhhhllllllllllhhhhhhh
Query ldADLXptRSACIaifearaqhpWPPRVatlphwpliyagalegldhlelartvdaaaqa  278
ident     |
Sbjct agEPLP--VDSEQ-------difDYIQW--------------------------------  314
DSSP  llLLLL--LLLHH-------hhhHHHLL--------------------------------


DSSP  hhhhhhhhhhllll
Query vqrfvaridratkr  292
ident
Sbjct ----ryrepkdrse  324
DSSP  ----llllhhhhhl
```

No 34: Query=mol1A Sbjct=1zjmA Z-score=7.3

back to top

```
DSSP  ------------------------------------------------------------
Query ------------------------------------------------------------    0
ident
Sbjct tlnggitdmltelanfeknvsqaihkynayrkaasviakyphkiksgaeakklpgvgtki   60
DSSP  lllhhhhhhhhhhhhhhhhllllhhhhhhhhhhhhhhhhlllllllhhhhhlllllhhh


DSSP  ------------------------------------------------llllllllllhh
Query ------------------------------------------------aapysspptnlr   12
ident
Sbjct aekideflatgklrklekirqddtsssinfltrvsgigpsaarkfvdegiktledlrkne  120
DSSP  hhhhhhhhhhlllhhhhhhhhlhhhhhhhhhlllllllhhhhhhhhhlllllhhhhhllh


DSSP  hhhhhhhhhhhllllhhhhhHHHHHHHHHHHHH-LLLLllLLLLEEEEHHHHHHHHhhll
Query slrdrltqvaerqgvvfgrlqRHVAXIVVAQFAA-TLTDdtGAPLLLVKGGSSLELrrgi   71
ident                                                | |
Sbjct dklnhhqriglkyfgdfekriPREEMLQMQDIVLnEVKKvdSEYIATVCGSFRRGA----  176
DSSP  hhllhhhhhhhhlhhhhhlleEHHHHHHHHHHHHHhHHHHhlLLLEEEELHHHHLLL----


DSSP  llllLLLLEEEEEL---------lLHHHHHHHHHHHHhhllllleeEEELLLEELllllll
Query pdsrTSKDFATVAR---------rDIELIHEQLADAGetgwegftAIFTAPEEIaapgxp  122
ident      | |                      |||             |
Sbjct ---eSSGDMDVLLThpsftsestkLLHQVVEQLQKVH--------FITDTLSKG------  219
DSSP  ---lEELLEEEEEElllllllllHHHHHHHHHHHHLL--------LEEEEEEEL------


DSSP  LLLEEEEEEEEEEL--LEEEEEEEEEEEEELLLLLLllleeeeeelhhhhllllleeeeell
Query VKPRRFTAKLSYR--GRAFATVPIEVSSVEAGNAdqfdtltsdalglvgvpaavavpcxt  180
ident          |             |
Sbjct ETKFMGVCQLPSKndEKEYPHRRIDIRLIPKDQY--------------------------  253
DSSP  LLEEEEEEELLLLllLLLLLEEEEEEEELLHHHH--------------------------


DSSP  hHHHHhhhhhhhhlllllllllllhhhhHHHHHH----------------------hhl
Query iPWQIaqklhavtavleepkandrahdlvDLQLLE----------------------gll  218
ident
Sbjct yCGVL--------------------yftgSDIFNKnmrahalekgftineytirplgvtg  293
DSSP  hHHHH--------------------hhhlLHHHHHhhhhhhhlleeellllleeellllll


DSSP  lhhhhhhhhHHHHhhhhhhllllLLLLLllllhhhhhhhhhllllllllllhhhhhhh
Query ldadlxptrSACIaifearaqhpWPPRVatlphwpliyagalegldhlelartvdaaaqa  278
ident          |
Sbjct vageplpvdSEKD-------ifdYIQWK--------------------------------  314
DSSP  lllllllllLHHH-------hhhHLLLL--------------------------------


DSSP  hhhhhhhhhhllll
Query vqrfvaridratkr  292
ident
Sbjct -----yrepkdrse  323
DSSP  -----lllhhhlll
```

No 35: Query=mol1A Sbjct=1zqzA Z-score=7.3

back to top

```
DSSP  ---------------------------llllllllllhhhhhhhhhhhhhhllllhhhhh
Query ---------------------------aapysspptnlrslrdrltqvaerqgvvfgrlq   33
ident 
Sbjct ddtsssinfltrvtgigpsaarklvdegiktledlrknedklnhhqriglkyfedfekri   60
DSSP  lllhhhhhhhlllllllhhhhhhhhhlllllhhhhhllhhhllhhhhhhhhlllhhhlle


DSSP  HHHHHHHHHHHHH-LLLLllLLLLEEEEHHHHHHHhhllllllLLLLEEEEEL-------
Query RHVAXIVVAQFAA-TLTDdtGAPLLLVKGGSSLELrrgipdsrTSKDFATVAR-------   85
ident                           | |               | |
Sbjct PREEMLQMQDIVLnEVKKldPEYIATVCGSFRRGA-------eSSGDMDVLLThpnftse  113
DSSP  EHHHHHHHHHHHHhHHHHhlLLLEEEELLLLLLLL-------lEELLEEEEEElllllll


DSSP  -----lLHHHHHHHHHHHHhhlllleeEEELLLEELllllllLLLEEEEEEEEELLeEEE
Query -----rDIELIHEQLADAGetgwegftAIFTAPEEIaapgxpVKPRRFTAKLSYRGrAFA  140
ident             |||             |                      |
Sbjct sskqpkLLHRVVEQLQKVR--------FITDTLSKG------ETKFMGVCQLPSEN-EYP  158
DSSP  llllhhHHHHHHHHHHHHL--------LEEEEEEEL------LLEEEEEELLLLLL-LLL


DSSP  EEEEEEEELLLLLLllleeeeelhhhhllllllleeeeellhHHHHhhhhhhhhlllllll
Query TVPIEVSSVEAGNAdqfdtltsdalglvgvpaavavpcxtiPWQIaqklhavtavleepk  200
ident    |
Sbjct HRRIDIRLIPKDQY--------------------------yCGVL---------------  177
DSSP  LEEEEEEELLHHHH--------------------------hHHHH---------------


DSSP  lllllhhhhHHHHHH----------------------hhllhhhhhhhhHHHHhhhhhhl
Query andrahdlvDLQLLE----------------------gllldadlxptrSACIaifeara  238
ident                                                  |
Sbjct -----yftgSDIFNKnmrahalekgftineytirplgvtgvageplpvdSEQD-------  225
DSSP  -----hlllLHHHHHhhhhhhhhlleeellllleeellllllllllllllLHHH-------


DSSP  lllLLLLLlllllhhhhhhhhhllllllllllhhhhhhhhhhhhhhhhhllll
Query qhpWPPRVatlphwpliyagalegldhlelartvdaaaqavqrfvaridratkr  292
ident 
Sbjct ifdYIQWR-------------------------------------yrepkdrse  242
DSSP  hhhHHLLL-------------------------------------lllhhhhll
```

No 36: Query=mol1A Sbjct=4r66A Z-score=7.3

back to top

```
DSSP  ------------------------------------------------------------
Query ------------------------------------------------------------    0
ident 
Sbjct tlnggitdmltelanfeknvsqaihkynayrkaasviakyphkiksgaeakklpgvgtki   60
DSSP  lllhhhhhhhhhhhhhhhhhllllhhhhhhhhhhhhhhhlllllllhhhhhhllllllhhh


DSSP  ------------------------------------------------llllllllllhh
Query ------------------------------------------------aapysspptnlr   12
ident 
Sbjct aekideflatgklrklekirqddtsssinfltrvsgigpsaarkfvdegiktledlrkne  120
DSSP  hhhhhhhhhhlllhhhhhhhhlhhhhhhhhhllllllhhhhhhhhhlllllhhhhhhllh


DSSP  hhhhhhhhhhhllllhhhhhhHHHHHHHHHHHHH-LLLLllLLLLEEEEHHHHHHHhhll
Query slrdrltqvaerqgvvfgrlqRHVAXIVVAQFAA-TLTDdtGAPLLLVKGGSSLELrrgi   71
ident                                                | |
Sbjct dklnhhqriglkyfgdfekriPREEMLQMQDIVLnEVKKvdSEYIATVCGSFRRGA----  176
DSSP  hhllhhhhhhhlhhhhhhlleEHHHHHHHHHHHHhHHHHhlLLLEEEELHHHHLLL----


DSSP  llllLLLLEEEEEL------------lLHHHHHHHHHHHHhhlllleeEEELLLEEllll
Query pdsrTSKDFATVAR------------rDIELIHEQLADAGetgwegftAIFTAPEEiaap  119
ident      | |                         |||             |
Sbjct ---eSSGDMDVLLThpsftsestkqpkLLHQVVEQLQKVH--------FITDTLSK----  221
DSSP  ---lEELLEEEEEElllllllllllllHHHHHHHHHHHLL--------LEEEEEEE----
```

```
DSSP  lllLLLEEEEEEEEEL--LEEEEEEEEEEEEL-LLLLllllleeeeelhhhhllllllleee
Query gxpVKPRRFTAKLSYR--GRAFATVPIEVSSV-EAGNadqfdtltsdalglvgvpaavav  176
ident             |             |
Sbjct --gETKFMGVCQLPSKndEKEYPHRRIDIRLIpKDQY-----------------------  256
DSSP  --lLLEEEEEEELLLLLllLLLLLLEEEEEEEELlHHHH-----------------------
```

```
DSSP  eellhHHHHhhhhhhhhllllllllllHHHH-------------------hhhhhhhh
Query pcxtiPWQIaqklhavtavleepkandraHDLV-------------------dlqllegl  217
ident                               |
Sbjct ----yCGVL----------------yftgSDIFnknmrahalekgftinkytirplgvtg  296
DSSP  ----hHHHH----------------hhhlLHHHhhhhhhhhllllleeellllleeellll
```

```
DSSP  llhhHHHHhhHHHHHhhhhhlllLLLLLlllllhhhhhhhhhlllllllllhhhhhh
Query lldaDLXPtrSACIAifearaqhPWPPRvatlphwpliyagalegldhlelartvdaaaq  277
ident      |
Sbjct vagePLPV--DSEKD-----ifdYIQWK--------------------------------  317
DSSP  llllLLLL--LLHHH-----hhhHLLLL--------------------------------
```

```
DSSP  hhhhhhhhhhhllll
Query avqrfvaridratkr  292
ident
Sbjct ------yrepkdrse  326
DSSP  ------lllhhhlll
```

No 37: Query=mol1A Sbjct=4ny8A Z-score=7.3

back to top

```
DSSP  ------------------------------------------------------------
Query ------------------------------------------------------------    0
ident
Sbjct lnggitdmltelanfeknvsqaihkynayrkaasviakyphkiksgaeakklpgvgtkia   60
DSSP  llhhhhhhhhhhhhhhhhllllhhhhhhhhhhhhhhhllllllllhhhhllllllllhhhh
```

```
DSSP  -----------------------------------------------lllllllllhhh
Query -----------------------------------------------aapyssspptnlrs   13
ident
Sbjct ekideflatgklrklekirqddtsssinfltrvsgigpsaarkfvdegiktledlrkned  120
DSSP  hhhhhhhhhlllhhhhhhhhlhhhhhhhhhlllllllhhhhhhhhhllllhhhhhhlhh
```

```
DSSP  hhhhhhhhhhhllllhHHHHHHHHHHHHHHHHHH-LLLLllLLLLEEEEHHHHHHHhhlll
Query lrdrltqvaerqgvvfGRLQRHVAXIVVAQFAA-TLTDdtGAPLLLVKGGSSLELrrgip   72
ident                                               | |
Sbjct klnhhqriglkyfgdfEKRIPREEMLQMQDIVLnEVKKvdSEYIATVCGSFRRGA-----  175
DSSP  hllhhhhhhhlhhhhLLLEEHHHHHHHHHHHHHhHHHHhlLLLEEEELHHHHLLL-----
```

```
DSSP  lllLLLLEEEEEL----------lLHHHHHHHHHHHHhhlllleeEEELLLEELllllll
Query dsrTSKDFATVAR----------rDIELIHEQLADAGetgwegftAIFTAPEEIaapgxp  122
ident     | |                       |||             |
Sbjct --eSSGDMDVLLThpsftsesqpkLLHQVVEQLQKVH--------FITDTLSKG------  219
DSSP  --lEELLEEEEEEllllllllllHHHHHHHHHHHHLL--------LEEEEEEEL------
```

```
DSSP  LLLEEEEEEEEEL--LEEEEEEEEEEEELLLLLLllleeeeelhhhhllllllleeeeell
Query VKPRRFTAKLSYR--GRAFATVPIEVSSVEAGNAdqfdtltsdalglvgvpaavavpcxt  180
ident          |             |
Sbjct ETKFMGVCQLPSKndEKEYPHRRIDIRLIPKDQY--------------------------  253
DSSP  LLEEEEEEELLLLLllLLLLLLEEEEEEEELLHHHH--------------------------
```

```
DSSP  hHHHHhhhhhhhhlllllllllllhhhhHHHHHH---------------------hhll
Query iPWQIaqklhavtavleepkandrahdlvDLQLLE---------------------glll  219
ident
Sbjct yCGVL---------------------yftgSDIFNKnmrahalekgftineytirplgvtg  293
DSSP  hHHHH---------------------hhhlLHHHHHhhhhhhhhlleeellllleeellll
```

```
DSSP  hhhhhhhHHHHHhhhhhhllllLLLLLLllllhhhhhhhhhlllllllllllhhhhhhhh
Query dadlxptRSACIaifearaqhpWPPRVAtlphwpliyagalegldhlelartvdaaaqav  279
ident
```

```
Sbjct vageplpVDSEK-------difDYIQWK--------------------------------  314
DSSP  lllllllLLLHH-------hhhHHLLLL--------------------------------
```

```
DSSP  hhhhhhhhhllll
Query qrfvaridratkr  292
ident
Sbjct ----yrepkdrse  323
DSSP  ----lllhhhlll
```

No 38: Query=mol1A Sbjct=4ppxA Z-score=7.3

back to top

```
DSSP  ------------------------------------------------------------
Query ------------------------------------------------------------    0
ident
Sbjct etlnggitdmltelanfeknvsqaihkynayrkaasviakyphkiksgaeakklpgvgtk   60
DSSP  llllhhhhhhhhhhhhhhhhhllllhhhhhhhhhhhhhhhlllllllhhhhhllllllhh
```

```
DSSP  -------------------------------------------------llllllllllh
Query -------------------------------------------------aapysspptnl   11
ident
Sbjct iaekideflatgklrklekirqddtsssinfltrvsgigpsaarkfvdegiktledlrkn  120
DSSP  hhhhhhhhhhhlllhhhhhhhhlhhhhhhhhhllllllhhhhhhhhhllllhhhhhll
```

```
DSSP  hhhhhhhhhhhhllllhhHHHHHHHHHHHHHHHH-LLLLllLLLLEEEEHHHHHHHHhhl
Query rslrdrltqvaerqgvvfgRLQRHVAXIVVAQFAA-TLTDdtGAPLLLVKGGSSLELrrg   70
ident                                                 | |
Sbjct edklnhhqriglkyfgdfeKRIPREEMLQMQDIVLnEVKKvdSEYIATVCGSFRRGA---  177
DSSP  hhhllhhhhhhhhlhhhhlLLEEHHHHHHHHHHHHHhHHHHhlLLLEEEELHHHHLLL---
```

```
DSSP  lllllLLLLEEEEEL--------lLHHHHHHHHHHHHhhlllleeEEELLLEELllllll
Query ipdsrTSKDFATVAR--------rDIELIHEQLADAGetgwegftAIFTAPEEIaapgxp  122
ident       | |                     |||             |
Sbjct ----eSSGDMDVLLThpsftseskLLHQVVEQLQKVH--------FITDTLSKG------  219
DSSP  ----lEELLEEEEEEllllllllHHHHHHHHHHHHLL--------LEEEEEEEL------
```

```
DSSP  LLLEEEEEEEEELleEEEEEEEEEEEL-LLLLllleeeeelhhhhlllllleeeeellh
Query VKPRRFTAKLSYRgrAFATVPIEVSSV-EAGNadqfdtltsdalglvgvpaavavpcxti  181
ident          |           |
Sbjct ETKFMGVCQLPSK--EYPHRRIDIRLIpKDQY---------------------------y  250
DSSP  LLEEEEEELLLLL--LLLLEEEEEEELlHHHH---------------------------h
```

```
DSSP  HHHHhhhhhhhlllllllllllhhhhHHHHHHHH-----------------hhllhhhhh
Query PWQIaqklhavtavleepkandrahdlvDLQLLE-----------------gllldadlx  224
ident
Sbjct CGVL--------------------yftgSDIFNKnmrahalekgftinkytirplagepl  290
DSSP  HHHH--------------------hhhlLHHHHHhhhhhhllleeellllleeellllll
```

```
DSSP  hhhHHHHhhhhhhllllLLLLLlllllhhhhhhhhhllllllllllhhhhhhhhhhhh
Query ptrSACIaifearaqhpWPPRVatlphwpliyagalegldhlelartvdaaaqavqrfva  284
ident    |
Sbjct pvdSEKD-------ifdYIQWK-------------------------------------y  306
DSSP  lllLHHH-------hhhHLLLL-------------------------------------l
```

```
DSSP  hhhhllll
Query ridratkr  292
ident
Sbjct repkdrse  314
DSSP  llhhhlll
```

No 39: Query=mol1A Sbjct=4tupA Z-score=7.2

back to top

```
DSSP  ------------------------------------------------------------
```

```
Query ------------------------------------------------------------    0
ident
Sbjct pqetlnggitdmltelanfeknvsqaihkynayrkaasviakyphkiksgaeakklpgvg   60
DSSP  llhhhhhhhhhhhhhhhhhhhhllllhhhhhhhhhhhhhhhllllllllhhhhhllllll


DSSP  ---------------------------------------------------lllllllll
Query ---------------------------------------------------aapyssppt    9
ident
Sbjct tkiaekideflatgklrklekirqddtsssinfltrvsgigpsaarkfvdegiktledlr  120
DSSP  hhhhhhhhhhhhhlllhhhhhhllhhhhhhhhhhllllllhhhhhhhhhhlllllhhhhh


DSSP  lhhhhhhhhhhhhhhllllhhhhhHHHHHHHHHHHHH-LLLLllLLLLEEEEHHHHHHHh
Query nlrslrdrltqvaerqgvvfgrlqRHVAXIVVAQFAA-TLTDdtGAPLLLVKGGSSLELr   68
ident                                                   | |
Sbjct knedklnhhqriglkyfgdfekriPREEMLQMQDIVLnEVKKvdSEYIATVCGSFRRGA-  179
DSSP  llhhhllhhhhhhhhlhhhhhlleEHHHHHHHHHHHHhHHHHhlLLLEEEELHHHHLLL-


DSSP  hllllllLLLLEEEEEL----------lLHHHHHHHHHHHHhhlllleeEEELLLEElll
Query rgipdsrTSKDFATVAR----------rDIELIHEQLADAGetgwegftAIFTAPEEiaa  118
ident         | |                       |||             |
Sbjct ------eSSGDMDVLLThpsftsesqpkLLHQVVEQLQKVH--------FITDTLSK---  222
DSSP  ------lEELLEEEEEElllllllllllHHHHHHHHHHHLL--------LEEEEEEE---


DSSP  llllLLLEEEEEEEEEL-LEEEEEEEEEEEEL-LLLLllllleeeeelhhhhlllllleee
Query pgxpVKPRRFTAKLSYR-GRAFATVPIEVSSV-EAGNadqfdtltsdalglvgvpaavav  176
ident              |            |
Sbjct ---gETKFMGVCQLPSKdEKEYPHRRIDIRLIpKDQY-----------------------  256
DSSP  ---lLLEEEEEEELLLLlLLLLLLEEEEEEELlHHHH-----------------------


DSSP  eellhHHHHhhhhhhhhhllllllllllHHHH------------------hhhhhhhhll
Query pcxtiPWQIaqklhavtavleepkandraHDLV------------------dlqlleglll  219
ident                               |
Sbjct ----yCGVL----------------yftgSDIFnknmrahalekgftineytirplgvtg  296
DSSP  ----hHHHH----------------hhhlLHHHhhhhhhhhhhlleeellllleeelllll


DSSP  hhhhhhhhHHHHHhhhhhlllLLLLLllllllhhhhhhhhhllllllllllhhhhhhhh
Query dadlxptrSACIAifearaqhPWPPRvatlphwpliyagalegldhlelartvdaaaqav  279
ident
Sbjct vageplpvDSEKD-----ifdYIQWK----------------------------------  317
DSSP  llllllllLLHHH-----hhhHLLLL----------------------------------


DSSP  hhhhhhhhhllll
Query qrfvaridratkr  292
ident
Sbjct ----yrepkdrse  326
DSSP  ----lllhhhlll
```

No 40: Query=mol1A Sbjct=5tzvA Z-score=7.2

back to top

```
DSSP  ------------------------------------------------------------
Query ------------------------------------------------------------    0
ident
Sbjct apqetlnggitdmltelanfeknvsqaihkynayrkaasviakyphkiksgaeakklpgv   60
DSSP  lllllllhhhhhhhhhhhhhhhhlllhhhhhhhhhhhhhhhhhlllllllhhhhhlllll


DSSP  ----------------------------------------------------llllllll
Query ----------------------------------------------------aapysspp    8
ident
Sbjct gtkiaekideflatgklrklekirqddtsssinfltrvsgigpsaarkfvdegiktledl  120
DSSP  lhhhhhhhhhhhhhlllhhhhhhhlhhhhhhhhhhllllllhhhhhhhhhhlllllhhhh


DSSP  llhhhhhhhhhhhhhhllllhhhhhHHHHHHHHHHHHH-LLLLllLLLLEEEEHHHHHHH
Query tnlrslrdrltqvaerqgvvfgrlqRHVAXIVVAQFAA-TLTDdtGAPLLLVKGGSSLEL   67
ident                                                    | |
Sbjct rknedklnhhqriglkyfgdfekriPREEMLQMQDIVLnEVKKvdSEYIATVCGSFRRGA  180
DSSP  hllhhhllhhhhhhhhlhhhhhlleEHHHHHHHHHHHHhHHHHhlLLLEEEELHHHHLLL
```

```
DSSP  hhllllllLLLLEEEEEL----------lLHHHHHHHHHHHHHhhllllleeEEELLLLEEll
Query rrgipdsrTSKDFATVAR----------rDIELIHEQLADAGetgwegftAIFTAPEEia  117
ident          | |                       |||             |
Sbjct -------eSSGDMDVLLThpsftsestpkLLHQVVEQLQKVH--------FITDTLSK--  223
DSSP  -------lEELLEEEEEElllllllllllHHHHHHHHHHHHLL--------LEEEEEEE--


DSSP  lllllLLLEEEEEEEEEEL--LEEEEEEEEEEEEL-LLLLllllleeeeelhhhhllllllle
Query apgxpVKPRRFTAKLSYR--GRAFATVPIEVSSV-EAGNadqfdtltsdalglvgvpaav  174
ident               |             |
Sbjct ----gETKFMGVCQLPSKndEKEYPHRRIDIRLIpKDQY---------------------  258
DSSP  ----lLLEEEEEEELLLLLllLLLLLLLEEEEEEEELlHHHH---------------------


DSSP  eeeellhHHHHhhhhhhhhllllllllllHHHH-------------------hhhhhh
Query avpcxtiPWQIaqklhavtavleepkandraHDLV-------------------dlqlle  215
ident                                 |
Sbjct ------yCGVL----------------yftgSDIFnknmrahalekgftineytirplgv  296
DSSP  ------hHHHH----------------hhhlLHHHhhhhhhhhhhlleeellllleeelll


DSSP  hhllhhHHHHhhHHHHHhhhhhlllLLLLLllllllhhhhhhhhhllllllllllhhhh
Query glllldaDLXPtrSACIAifearaqhPWPPRvatlphwpliyagalegldhlelartvdaa  275
ident        |
Sbjct tgvagePLPV--DSEKD-----ifdYIQWK------------------------------  319
DSSP  llllllLLLL--LLHHH-----hhhHLLLL------------------------------


DSSP  hhhhhhhhhhhhhllll
Query aqavqrfvaridratkr  292
ident
Sbjct --------yrepkdrse  328
DSSP  --------lllhhhlll
```

No 41: Query=mol1A Sbjct=5j0tA Z-score=7.2

back to top

```
DSSP  ------------------------------------------------------------
Query ------------------------------------------------------------    0
ident
Sbjct apqetlnggitdmltelanfeknvsqaihkynayrkaasviakyphkiksgaeakklpgv   60
DSSP  lllhhhhhhhhhhhhhhhhhhhhhhllllhhhhhhhhhhhhhhhlllllllllhhhhhlllll


DSSP  ----------------------------------------------------llllllll
Query ----------------------------------------------------aapysspp    8
ident
Sbjct gtkiaekideflatgklrklekirqddtsssinfltrvsgigpsaarkfvdegiktledl  120
DSSP  lhhhhhhhhhhhhhlllhhhhhhhhlhhhhhhhhhlllllllhhhhhhhhhlllllhhhh


DSSP  llhhhhhhhhhhhhhhllllhhhhhHHHHHHHHHHHHH-LLLLllLLLLEEEEHHHHHHH
Query tnlrslrdrltqvaerqgvvfgrlqRHVAXIVVAQFAA-TLTDdtGAPLLLVKGGSSLEL   67
ident                                                    | |
Sbjct rknedklnhhqriglkyfgdfekriPREEMLQMQDIVLnEVKKvdSEYIATVCGSFRRGA  180
DSSP  hhlhhhllhhhhhhhhlhhhhhlleEHHHHHHHHHHHHHhHHHHhlLLLLEEEELHHHHLLL


DSSP  hhllllllLLLLEEEEEL---------lLHHHHHHHHHHHHHhhllllleeEEELLLLEElll
Query rrgipdsrTSKDFATVAR---------rDIELIHEQLADAGetgwegftAIFTAPEEiaa  118
ident          | |                      |||             |
Sbjct -------eSSGDMDVLLThpsftsespkLLHQVVEQLQKVH--------FITDTLSK---  222
DSSP  -------lEELLEEEEEElllllllllllHHHHHHHHHHHHLL--------LEEEEEEE---


DSSP  llllLLLEEEEEEEEEEL--LEEEEEEEEEEEEL-LLLLllllleeeeelhhhhllllllee
Query pgxpVKPRRFTAKLSYR--GRAFATVPIEVSSV-EAGNadqfdtltsdalglvgvpaava  175
ident              |             |
Sbjct ---gETKFMGVCQLPSKndEKEYPHRRIDIRLIpKDQY----------------------  257
DSSP  ---lLLEEEEEEELLLLLllLLLLLLLEEEEEEEELlHHHH----------------------


DSSP  eeellhHHHHhhhhhhhhllllllllllHHHH-------------------hhhhhhh
Query vpcxtiPWQIaqklhavtavleepkandraHDLV-------------------dlqlleg  216
```

```
ident                               |
Sbjct -----yCGVL----------------yftgSDIFnknmrahalekgftineytirplgvt  296
DSSP  -----hHHHH----------------hhhlLHHHhhhhhhhhhhlleeelllleeellll


DSSP  hllhhHHHHhhHHHHHhhhhhlllLLLLLllllllhhhhhhhhhllllllllllhhhhh
Query llldaDLXPtrSACIAifearaqhPWPPRvatlphwpliyagalegldhlelartvdaaa  276
ident       |
Sbjct gvagePLPV--DSEKD-----ifdYIQWK-------------------------------  318
DSSP  lllllLLLL--LLHHH-----hhhHLLLL-------------------------------


DSSP  hhhhhhhhhhhllll
Query qavqrfvaridratkr  292
ident
Sbjct -------yrepkdrse  327
DSSP  -------lllhhhlll
```

No 42: Query=mol1A Sbjct=4pgyA Z-score=7.2

back to top

```
DSSP  ------------------------------------------------------------
Query ------------------------------------------------------------    0
ident
Sbjct pqetlnggitdmltelanfeknvsqaihkynayrkaasviakyphkiksgaeakklpgvg   60
DSSP  llllllhhhhhhhhhhhhhhhhllllhhhhhhhhhhhhhhhllllllllhhhhllllllll


DSSP  ---------------------------------------------------lllllllll
Query ---------------------------------------------------aapysssppt    9
ident
Sbjct tkiaekideflatgklrklekirqddtsssinfltrvsgigpsaarkfvdegiktledlr  120
DSSP  hhhhhhhhhhhhhlllhhhhhhhhlhhhhhhhhhllllllhhhhhhhhhlllllhhhhh


DSSP  lhhhhhhhhhhhhhllllhhhhhHHHHHHHHHHHHH-LLLLllLLLLEEEEHHHHHHHh
Query nlrslrdrltqvaerqgvvfgrlqRHVAXIVVAQFAA-TLTDdtGAPLLLVKGGSSLELr   68
ident                                                   | |
Sbjct knedklnhhqriglkyfgdfekriPREEMLQMQDIVLnEVKKvdSEYIATVCGSFRRGA-  179
DSSP  hlhhhllhhhhhhhhlhhhhhlleEHHHHHHHHHHHHhHHHHhlLLLEEEELHHHHLLL-


DSSP  hllllllLLLLEEEEEL----------lLHHHHHHHHHHHHhhllleeEEELLLEElll
Query rgipdsrTSKDFATVAR----------rDIELIHEQLADAGetgwegftAIFTAPEEiaa  118
ident         | |                       |||             |
Sbjct ------eSSGDMDVLLThpsftsesqpkLLHQVVEQLQKVH--------FITDTLSK---  222
DSSP  ------lEELLEEEEEllllllllllHHHHHHHHHHHHLL--------LEEEEEEE---


DSSP  llllLLLEEEEEEEEEL--LEEEEEEEEEEEELLLLLLllleeeeelhhhhllllllleee
Query pgxpVKPRRFTAKLSYR--GRAFATVPIEVSSVEAGNAdqfdtltsdalglvgvpaavav  176
ident              |             |
Sbjct ---gETKFMGVCQLPSKndEKEYPHRRIDIRLIPKDQY----------------------  257
DSSP  ---lLLEEEEEELLLLllLLLLLLLEEEEEEEELLHHHH----------------------


DSSP  eellhHHHHhhhhhhhhlllllllllllHHHH-------------------hhhhhhhh
Query pcxtiPWQIaqklhavtavleepkandraHDLV-------------------dlqllegl  217
ident                               |
Sbjct ----yCGVL----------------yftgSDIFnknmrahalekgftineytirplgvtg  297
DSSP  ----hHHHH----------------hhhlLHHHhhhhhhhhhhlleeelllleeelllll


DSSP  llhhHHHHhhHHHHHhhhhhlllLLLLLllllllhhhhhhhhhllllllllllhhhhhh
Query lldaDLXPtrSACIAifearaqhPWPPRvatlphwpliyagalegldhlelartvdaaaq  277
ident      |
Sbjct vagePLPV--DSEKD-----ifdYIQWK--------------------------------  318
DSSP  llllLLLL--LLHHH-----hhhHLLLL--------------------------------


DSSP  hhhhhhhhhhllll
Query avqrfvaridratkr  292
ident
Sbjct ------yrepkdrse  327
DSSP  ------lllhhhlll
```

No 43: Query=mol1A Sbjct=5dbbA Z-score=7.2

back to top

```
DSSP  ------------------------------------------------------------
Query ------------------------------------------------------------   0
ident 
Sbjct pqetlnggitdmltelanfeknvsqaihkynayrkaasviakyphkiksgaeakklpgvg  60
DSSP  llhhhhhhhhhhhhhhhhhhhhllllhhhhhhhhhhhhhhhhlllllllhhhhlllllll


DSSP  ---------------------------------------------------lllllllll
Query ---------------------------------------------------aapyssppt   9
ident 
Sbjct tkiaekideflatgklrklekirqddtsssinfltrvsgigpsaarkfvdegiktledlr 120
DSSP  hhhhhhhhhhhhhlllhhhhhhhhlhhhhhhhhhllllllhhhhhhhhhlllllhhhhhh


DSSP  lhhhhhhhhhhhhhhhhllllhhhHHHHHHHHHHHHHH-LLLLllLLLLEEEEHHHHHHh
Query nlrslrdrltqvaerqgvvfgrlqRHVAXIVVAQFAA-TLTDdtGAPLLLVKGGSSLELr  68
ident                                                   | |
Sbjct knedklnhhqriglkyfgdfekriPREEMLQMQDIVLnEVKKvdSEYIATVCGSFRRGA- 179
DSSP  hlhhhllhhhhhhhhlhhhhllleEHHHHHHHHHHHHhHHHHhlLLLEEEELHHHHLLL-


DSSP  hllllllLLLLEEEEEL----------lLHHHHHHHHHHHHhhlllleeEEELLLEElll
Query rgipdsrTSKDFATVAR----------rDIELIHEQLADAGetgwegftAIFTAPEEiaa 118
ident         | |                       |||             |
Sbjct ------eSSGDMDVLLThpsftsesqpkLLHQVVEQLQKVH--------FITDTLSK--- 222
DSSP  ------lEELLEEEEEElllllllllllHHHHHHHHHHHLL--------LEEEEEEE---


DSSP  llllLLLEEEEEEEEELL--EEEEEEEEEEEEL-LLLLlllleeeeelhhhhllllllee
Query pgxpVKPRRFTAKLSYRG--RAFATVPIEVSSV-EAGNadqfdtltsdalglvgvpaava 175
ident              |             |
Sbjct ---gETKFMGVCQLPSKNdeKEYPHRRIDIRLIpKDQY---------------------- 257
DSSP  ---lLLEEEEEEELLLLLllLLLLLEEEEEEELlHHHH----------------------


DSSP  eeellhHHHHhhhhhhhhhlllllllllllHHHH-------------------hhhhhhh
Query vpcxtiPWQIaqklhavtavleepkandraHDLV-------------------dlqlleg 216
ident                                |
Sbjct -----yCGVL----------------yftgSDIFnknmrahalekgftineytirplgvt 296
DSSP  -----hHHHH----------------hhhlLHHHhhhhhhhhhhlleeellllleeellll


DSSP  hllhhHHHHhhHHHHHhhhhhlllLLLLLllllllhhhhhhhhhlllllllllllhhhhh
Query llldaDLXPtrSACIAifearaqhPWPPRvatlphwpliyagalegldhlelartvdaaa 276
ident       |
Sbjct gvagePLPV--DSEKD-----ifdYIQWK------------------------------- 318
DSSP  lllllLLLL--LLHHH-----hhhHLLLL-------------------------------


DSSP  hhhhhhhhhhhhllll
Query qavqrfvaridratkr  292
ident 
Sbjct -------yrepkdrse  327
DSSP  -------lllhhhlll
```

No 44: Query=mol1A Sbjct=5j0oA Z-score=7.2

back to top

```
DSSP  ------------------------------------------------------------
Query ------------------------------------------------------------   0
ident 
Sbjct pqetlnggitdmltelanfeknvsqaihkynayrkaasviakyphkiksgaeakklpgvg  60
DSSP  llllllhhhhhhhhhhhhhhhhllllhhhhhhhhhhhhhhhhlllllllhhhhhllllll


DSSP  ---------------------------------------------------lllllllll
Query ---------------------------------------------------aapyssppt   9
ident 
Sbjct tkiaekideflatgklrklekirqddtsssinfltrvsgigpsaarkfvdegiktledlr 120
```

```
DSSP  hhhhhhhhhhhhhlllhhhhhhhhlhhhhhhhhhllllllhhhhhhhhhlllllhhhhh
```

```
DSSP  lhhhhhhhhhhhhhhllllhhhhhHHHHHHHHHHHHH-LLLLllLLLLEEEEHHHHHHHh
Query nlrslrdrltqvaerqgvvfgrlqRHVAXIVVAQFAA-TLTDdtGAPLLLVKGGSSLELr   68
ident                                                   | |
Sbjct knedklnhhqriglkyfgdfekriPREEMLQMQDIVLnEVKKvdSEYIATVCGSFRRGA-  179
DSSP  llhhhllhhhhhhhhlhhhhllleEHHHHHHHHHHHHhHHHHhlLLLEEEELLLLLLLL-
```

```
DSSP  hllllllLLLLEEEEEL----------lLHHHHHHHHHHHHhhlllleeEEELLLEElll
Query rgipdsrTSKDFATVAR----------rDIELIHEQLADAGetgwegftAIFTAPEEiaa  118
ident         | |                       |||             |
Sbjct ------eSSGDMDVLLThpsftsesqpkLLHQVVEQLQKVH--------FITDTLSK---  222
DSSP  ------lEELLEEEEEEllllllllllHHHHHHHHHHHLL--------LEEEEEEE---
```

```
DSSP  llllLLLEEEEEEEEEEL--LEEEEEEEEEEEEL-LLLLllllleeeeelhhhhllllllee
Query pgxpVKPRRFTAKLSYR--GRAFATVPIEVSSV-EAGNadqfdtltsdalglvgvpaava  175
ident              |             |
Sbjct ---gETKFMGVCQLPSKndEKEYPHRRIDIRLIpKDQY----------------------  257
DSSP  ---lLLEEEEEEELLLLLllLLLLLLEEEEEEELlHHHH----------------------
```

```
DSSP  eeellhHHHHhhhhhhhhlllllllllllHHHH-------------------hhhhhhh
Query vpcxtiPWQIaqklhavtavleepkandraHDLV-------------------dlqlleg  216
ident                                |
Sbjct -----yCGVL----------------yftgSDIFnknmrahalekgftineytirplgvt  296
DSSP  -----hHHHH----------------hlllLHHHhhhhhhhhhhhleeellllleeellll
```

```
DSSP  hllhhHHHHhhHHHHHhhhhhlllLLLLLllllllhhhhhhhhhllllllllllhhhhh
Query llldaDLXPtrSACIAifearaqhPWPPRvatlphwpliyagalegldhlelartvdaaa  276
ident       |
Sbjct gvagePLPV--DSEKD-----ifdYIQWK-------------------------------  318
DSSP  lllllLLLL--LLHHH-----hhhHLLLL-------------------------------
```

```
DSSP  hhhhhhhhhhhhllll
Query qavqrfvaridratkr  292
ident
Sbjct -------yrepkdrse  327
DSSP  -------lllhhhlll
```

No 45: Query=mol1A Sbjct=3uxnA Z-score=7.2

back to top

```
DSSP  ------------------------------------------------------------
Query ------------------------------------------------------------    0
ident
Sbjct tlnggitdmlvelanfeknvsqaihkynayrkaasviakyphkiksgaeakklpgvgtki   60
DSSP  llllhhhhhhhhhhhhhhhllllhhhhhhhhhhhhhhhhlllllllllllllllllhhh
```

```
DSSP  ------------------------------------------------lllllllllhh
Query ------------------------------------------------aapysspptnlr   12
ident
Sbjct aekideflatgklrklekirqddtsssinfltrvtgigpsaarklvdegiktledlrkne  120
DSSP  hhhhhhhhhlllhhhhhhhlllhhhhhhhhlllllllhhhhhhhhlllllllhhhhlllh
```

```
DSSP  hhhhhhhhhhhhllllhhhhhHHHHHHHHHHHHH-LLLLllLLLLEEEEHHHHHHHHhhll
Query slrdrltqvaerqgvvfgrlqRHVAXIVVAQFAA-TLTDdtGAPLLLVKGGSSLELrrgi   71
ident                                                |  |
Sbjct dklnhhqriglkyfedfekriPREEMLQMQDIVLnEVKKldPEYIATVCGSFRRGA----  176
DSSP  hhllhhhhhhhhlhhhhllleEHHHHHHHHHHHHhHHHHhlLLLEEEELHHHHLLL----
```

```
DSSP  llllLLLLEEEEEL------------lLHHHHHHHHHHHHhhlllleeEEELLLEELlll
Query pdsrTSKDFATVAR------------rDIELIHEQLADAGetgwegftAIFTAPEEIaap  119
ident      | |                         |||             |
Sbjct ---eSSGDMDVLLThpnftsesskqpkLLHRVVEQLQKVR--------FITDTLSKG---  222
DSSP  ---lEELLEEEEEEllllllllllllhHHHHHHHHHHHHLL--------LEEEEEEEL---
```

```
DSSP  lllLLLEEEEEEEEEELLE--EEEEEEEEEEEL-LLLLlllleeeeelhhhhllllllleee
```

```
Query gxpVKPRRFTAKLSYRGR--AFATVPIEVSSV-EAGNadqfdtltsdalglvgvpaavav  176
ident             |             |
Sbjct ---ETKFMGVCQLPSENDenEYPHRRIDIRLIpKDQY-----------------------  256
DSSP  ---LLEEEEEELLLLLLLllLLLLEEEEEEELlHHHH-----------------------


DSSP  eellhHHHHhhhhhhhlllllllllllllhhhhHHHHHH---------------------
Query pcxtiPWQIaqklhavtavleepkandrahdlvDLQLLE---------------------  215
ident
Sbjct ----yCGVL--------------------yftgSDIFNKnmrahalekgftineytirpl  292
DSSP  ----hHHHH--------------------hhhlLHHHHHhhhhhhlllleeellllleeel


DSSP  hhllhhhhhhhHHHHHhhhhhhllllLLLLLlllllhhhhhhhhhlllllllllllhhhh
Query gllldadlxptRSACIaifearaqhpWPPRVatlphwpliyagalegldhlelartvdaa  275
ident
Sbjct gvtgvageplpVDSEQ-------difDYIQW-----------------------------  316
DSSP  lllllllllllLLLHH-------hhhHHLLL-----------------------------


DSSP  hhhhhhhhhhhhhllll
Query aqavqrfvaridratkr  292
ident
Sbjct --------ryrepkdrs  325
DSSP  --------llllhhhll
```

No 46: Query=mol1A Sbjct=3uxoA Z-score=7.2

back to top

```
DSSP  ------------------------------------------------------------
Query ------------------------------------------------------------    0
ident
Sbjct tlnggitdmlvelanfeknvsqaihkynayrkaasviakyphkiksgaeakklpgvgtki   60
DSSP  lllhhhhhhhhhhhhhhhhllllhhhhhhhhhhhhhhhhllllllhhhhhhllllllhhh


DSSP  ------------------------------------------------llllllllllhh
Query ------------------------------------------------aapysspptnlr   12
ident
Sbjct aekideflatgklrklekirqddtsssinfltrvtgigpsaarklvdegiktledlrkne  120
DSSP  hhhhhhlllllllhhhhhhhhllhhhhhhhhlllllllhhhhhhhhllllllhhhhlllh


DSSP  hhhhhhhhhhhhllllhhhhhHHHHHHHHHHHHHH-LLLLllLLLLEEEHHHHHHHHhhll
Query slrdrltqvaerqgvvfgrlqRHVAXIVVAQFAA-TLTDdtGAPLLLVKGGSSLELrrgi   71
ident                                                | |
Sbjct dklnhhqriglkyfedfekriPREEMLQMQDIVLnEVKKldPEYIATVCGSFRRGA----  176
DSSP  hhllhhhhhhhhlhhhhhlleEHHHHHHHHHHHHhHHHHhlLLLEEEELHHHHLLL----


DSSP  llllLLLLEEEEEL------------lLHHHHHHHHHHHHhhlllleeEEELLLEELlll
Query pdsrTSKDFATVAR------------rDIELIHEQLADAGetgwegftAIFTAPEEIaap  119
ident      | |                         |||             |
Sbjct ---eSSGDMDVLLThpnftsesskqpkLLHRVVEQLQKVR--------FITDTLSKG---  222
DSSP  ---lEELLEEEEEElllllllllllhhHHHHHHHHHHHLL--------LEEEEEEEL---


DSSP  lllLLLEEEEEEEEELLE--EEEEEEEEEEEL-LLLLlllleeeeelhhhhlllllleee
Query gxpVKPRRFTAKLSYRGR--AFATVPIEVSSV-EAGNadqfdtltsdalglvgvpaavav  176
ident             |             |
Sbjct ---ETKFMGVCQLPSENDenEYPHRRIDIRLQpKDQY-----------------------  256
DSSP  ---LLEEEEEELLLLLLLllLLLLEEEEEEELlHHHH-----------------------


DSSP  eellhHHHHhhhhhhhlllllllllllllhHHHHHH--------------------hhhh
Query pcxtiPWQIaqklhavtavleepkandrahDLVDLQ--------------------lleg  216
ident
Sbjct ----yCGVL-----------------yftgSDIFNKnmrahalekgftineytirplgvt  295
DSSP  ----hHHHH-----------------hhhlLHHHHHhhhhhhhhlleeellllleeellll


DSSP  hllhHHHHhhHHHHHhhhhhhllllLLLLLlllllhhhhhhhhhlllllllllllhhhhh
Query llldADLXptRSACIaifearaqhpWPPRVatlphwpliyagalegldhlelartvdaaa  276
ident       |
Sbjct gvagEPLP--VDSEQ-------difDYIQW------------------------------  316
DSSP  llllLLLL--LLLHH-------hhhHHLLL------------------------------
```

```
DSSP  hhhhhhhhhhhhllll
Query qavqrfvaridratkr  292
ident 
Sbjct -------ryrepkdrs  325
DSSP  -------llllhhhll
```

No 47: Query=mol1A Sbjct=1tv9A Z-score=7.2

back to top

```
DSSP  ------------------------------------------------------------
Query ------------------------------------------------------------    0
ident 
Sbjct kapqetlnggitdmltelanfeknvsqaihkynayrkaasviakyphkiksgaeakklpg   60
DSSP  llllhhhhhhhhhhhhhhhhhhhhhllllhhhhhhhhhhhhhhhhllllllllhhhhhllll


DSSP  -----------------------------------------------------lllllll
Query -----------------------------------------------------aapyssp    7
ident 
Sbjct vgtkiaekideflatgklrklekirqddtsssinfltrvsgigpsaarkfvdegiktled  120
DSSP  llhhhhhhhhhhhhhlllhhhhhhhhlhhhhhhhhhlllllllhhhhhhhhhlllllhhh


DSSP  lllhhhhhhhhhhhhhhllllhhhhhHHHHHHHHHHHHH-LLLLllLLLLEEEEHHHHHH
Query ptnlrslrdrltqvaerqgvvfgrlqRHVAXIVVAQFAA-TLTDdtGAPLLLVKGGSSLE   66
ident                                                     | |
Sbjct lrknedklnhhqriglkyfgdfekriPREEMLQMQDIVLnEVKKvdSEYIATVCGSFRRG  180
DSSP  hhhlhhhllhhhhhhhhlhhhhhlleEHHHHHHHHHHHHHhHHHHhlLLLEEEELHHHHLL


DSSP  HhhlllllllLLLLEEEEEL------------lLHHHHHHHHHHHHhhlllleeEEELLLE
Query LrrgipdsrTSKDFATVAR------------rDIELIHEQLADAGetgwegftAIFTAPE  114
ident           | |                         |||             |
Sbjct A-------eSSGDMDVLLThpsftsestkqpkLLHQVVEQLQKVH--------FITDTLS  225
DSSP  L-------lEELLEEEEEElllllllllllhhHHHHHHHHHHHHLL--------LEEEEEE


DSSP  ELllllllLLLEEEEEEEEEEL--LEEEEEEEEEEEEL-LLLLllllleeeeelhhhhllll
Query EIaapgxpVKPRRFTAKLSYR--GRAFATVPIEVSSV-EAGNadqfdtltsdalglvgvp  171
ident                  |             |
Sbjct KG------ETKFMGVCQLPSKndEKEYPHRRIDIRLIpKDQY------------------  261
DSSP  EL------LLEEEEEELLLLLllLLLLLLEEEEEEELlHHHH------------------


DSSP  lleeeeellhHHHHhhhhhhhhllllllllllllHHHHHHHH------------------
Query aavavpcxtiPWQIaqklhavtavleepkandraHDLVDLQL------------------  213
ident                                    |
Sbjct ---------yCGVL----------------yftgSDIFNKNMrahalekgftineytirp  296
DSSP  ---------hHHHH----------------hhhlLHHHHHHHhhhhhhlleeellllleee


DSSP  -hhhhllhHHHHhhHHHHHhhhhhhllllLLLLLllllllhhhhhhhhhlllllllllllh
Query -leglllldADLXptRSACIaifearaqhpWPPRVatlphwpliyagalegldhlelartv  272
ident           |
Sbjct lgvtgvagEPLP--VDSEK-------difDYIQW--------------------------  321
DSSP  lllllllllLLLL--LLLHH-------hhhHHLLL--------------------------


DSSP  hhhhhhhhhhhhhhhhllll
Query daaaqavqrfvaridratkr  292
ident 
Sbjct ----------kyrepkdrse  331
DSSP  ----------llllhhhlll
```

No 48: Query=mol1A Sbjct=3iscA Z-score=7.2

back to top

```
DSSP  ------------------------------------------------------------
Query ------------------------------------------------------------    0
ident 
```

```
Sbjct pqetlnggitdmltelanfeknvsqaihkynayrkaasviakyphkiksgaeakklpgvg   60
DSSP  llhhhhhhhhhhhhhhhhhhhhhllllhhhhhhhhhhhhhhhhlllllllhhhhhlllll


DSSP  ---------------------------------------------------lllllllll
Query ---------------------------------------------------aapyssppt    9
ident
Sbjct tkiaekideflatgklrklekirqddtsssinfltrvsgigpsaarkfvdegiktledlr  120
DSSP  hhhhhhhhhhhhhlllhhhhhhhhlhhhhhhhhhllllllhhhhhhhhhllllhhhhh


DSSP  lhhhhhhhhhhhhhhllllhhhhhHHHHHHHHHHHHH-LLLLllLLLLEEEEHHHHHHHh
Query nlrslrdrltqvaerqgvvfgrlqRHVAXIVVAQFAA-TLTDdtGAPLLLVKGGSSLELr   68
ident                                                   | |
Sbjct knedklnhhqriglkyfgdfekriPREEMLQMQDIVLnEVKKvdSEYIATVCGSFRRGA-  179
DSSP  llhhhllhhhhhhhhlhhhhllleEHHHHHHHHHHHHHhHHHHhlLLLEEEELHHHHLLL-


DSSP  hllllllLLLLEEEEEL----------lLHHHHHHHHHHHHhhlllleeEEELLLEELll
Query rgipdsrTSKDFATVAR----------rDIELIHEQLADAGetgwegftAIFTAPEEIaa  118
ident         | |                       |||             |
Sbjct ------eSSGDMDVLLThpsftsesqpkLLHQVVEQLQKVH--------FITDTLSKG--  223
DSSP  ------lEELLEEEEEllllllllllHHHHHHHHHHHLL--------LEEEEEEL--


DSSP  llllLLLEEEEEEEEELL--EEEEEEEEEEEEL-LLLLlllleeeeelhhhhlllllllee
Query pgxpVKPRRFTAKLSYRG--RAFATVPIEVSSV-EAGNadqfdtltsdalglvgvpaava  175
ident              |             |
Sbjct ----ETKFMGVCQLPSKNdeKEYPHRRIDIRLIpKDQY----------------------  257
DSSP  ----LLEEEEEELLLLLLllLLLLLEEEEEEELlHHHH----------------------


DSSP  eeellhHHHHhhhhhhhhlllllllllllhhhhHHHHHH--------------------
Query vpcxtiPWQIaqklhavtavleepkandrahdlvDLQLLE--------------------  215
ident
Sbjct -----yCGVL--------------------yftgSDIFNKnmrahalekgftineytirp  292
DSSP  -----hHHHH--------------------hhhlLHHHHHhhhhhhhhhlleeellllleee


DSSP  --hhllhhhhhhhhHHHHhhhhhhllllLLLLLllllllhhhhhhhhhllllllllllhh
Query --gllldadlxptrSACIaifearaqhpWPPRVatlphwpliyagalegldhlelartvd  273
ident               |
Sbjct lgvtgvageplpvdSEKD-------ifdYIQWK---------------------------  318
DSSP  llllllllllllllLHHH-------hhhHLLLL---------------------------


DSSP  hhhhhhhhhhhhhhhllll
Query aaaqavqrfvaridratkr  292
ident
Sbjct ----------yrepkdrse  327
DSSP  ----------lllhhhlll
```

No 49: Query=mol1A Sbjct=3isbA Z-score=7.2

back to top

```
DSSP  ------------------------------------------------------------
Query ------------------------------------------------------------    0
ident
Sbjct pqetlnggitdmltelanfeknvsqaihkynayrkaasviakyphkiksgaeakklpgvg   60
DSSP  llhhhhhhhhhhhhhhhhhhhhhllllhhhhhhhhhhhhhhhhlllllllhhhhhlllll


DSSP  ---------------------------------------------------lllllllll
Query ---------------------------------------------------aapyssppt    9
ident
Sbjct tkiaekideflatgklrklekirqddtsssinfltrvsgigpsaarkfvdegiktledlr  120
DSSP  hhhhhhhhhhhhhlllhhhhhhhhllhhhhhhhhhllllllhhhhhhhhhllllhhhhh


DSSP  lhhhhhhhhhhhhhhllllhhhhhHHHHHHHHHHHHH-LLLLllLLLLEEEEHHHHHHHh
Query nlrslrdrltqvaerqgvvfgrlqRHVAXIVVAQFAA-TLTDdtGAPLLLVKGGSSLELr   68
ident                                                   | |
Sbjct knedklnhhqriglkyfgdfekriPREEMLQMQDIVLnEVKKvdSEYIATVCGSFRRGA-  179
DSSP  hlhhhllhhhhhhhhlhhhhllleEHHHHHHHHHHHHHhHHHHhlLLLEEEELHHHHHLL-
```

```
DSSP  hllllllLLLLEEEEEL----------lLHHHHHHHHHHHHhhllllleeEEELLLEELll
Query rgipdsrTSKDFATVAR----------rDIELIHEQLADAGetgwegftAIFTAPEEIaa  118
ident         | |                       |||             |
Sbjct ------eSSGDMDVLLThpsftsesqpkLLHQVVEQLQKVH--------FITDTLSKG--  223
DSSP  ------lEELLEEEEEEllllllllllHHHHHHHHHHHLL--------LEEEEEEEL--


DSSP  llllLLLEEEEEEEEELL--EEEEEEEEEEEEL-LLLLlllleeeeelhhhhllllllee
Query pgxpVKPRRFTAKLSYRG--RAFATVPIEVSSV-EAGNadqfdtltsdalglvgvpaava  175
ident              |             |
Sbjct ----ETKFMGVCQLPSKNdeKEYPHRRIDIRLIpKDQY----------------------  257
DSSP  ----LLEEEEEELLLLLLllLLLLLEEEEEEELlHHHH----------------------


DSSP  eeellhHHHhhhhhhhhhllllllllllllhhhhHHHHHH--------------------
Query vpcxtiPWQIaqklhavtavleepkandrahdlvDLQLLE--------------------  215
ident
Sbjct -----yCGVL--------------------yftgSDIFNKnmrahalekgftineytirp  292
DSSP  -----hHHHH--------------------hlllLHHHHHhhhhhhhllleeelllleee


DSSP  -hhllhhhhhhhHHHHHHhhhhhllllLLLLLllllhhhhhhhhhllllllllllllhhh
Query -gllldadlxptRSACIAifearaqhpWPPRVatlphwpliyagalegldhlelartvda  274
ident
Sbjct lgvtgvageplpVDSEKD-------ifDYIQW----------------------------  317
DSSP  llllllllllllLLLHHH-------hhHHLLL----------------------------


DSSP  hhhhhhhhhhhhhhllll
Query aaqavqrfvaridratkr  292
ident
Sbjct --------kyrepkdrse  327
DSSP  --------llllhhhlll
```

No 50: Query=mol1A Sbjct=2vanA Z-score=7.2

back to top

```
DSSP  ---------------------------llllllllllhhhhhhhhhhhhhhllllhhhhh
Query ---------------------------aapyssppptnlrslrdrltqvaerqgvvfgrlq   33
ident
Sbjct ddtsssinfltrvtgigpsaarklvdegiktledlrknedklnhhqriglkyfedfekri   60
DSSP  lhhhhhhhhhlllllllhhhhhhhhllllllhhhhhllhhhllhhhhhhhhlllhhhlle


DSSP  HHHHHHHHHHHHH-LLLLllLLLLEEEEHHHHHHHhhllllllLLLLEEEEEL-------
Query RHVAXIVVAQFAA-TLTDdtGAPLLLVKGGSSLELrrgipdsrTSKDFATVAR-------   85
ident                           | |               | |
Sbjct PREEMLQMQDIVLnEVKKldPEYIATVCGSFRRGA-------eSSGDMDVLLThpnftse  113
DSSP  EHHHHHHHHHHHHhHHHHhlLLLEEEELHHHHLLL-------lEELLEEEEEElllllll


DSSP  -----lLHHHHHHHHHHHHhhlllleeEEELLLEELllllllLLLEEEEEEEEElleEEE
Query -----rDIELIHEQLADAGetgwegftAIFTAPEEIaapgxpVKPRRFTAKLSYrgrAFA  140
ident             |||             |                      |
Sbjct sskqpkLLHRVVEQLQKVR--------FITDTLSKG------ETKFMGVCQLPS---EYP  156
DSSP  llllhhHHHHHHHHHHHLL--------LEEEEEEEL------LLEEEEEELLLL---LLL


DSSP  EEEEEEEELLLLLLllleeeeelhhhhlllllleeeeellhHHHhhhhhhhhhlllllll
Query TVPIEVSSVEAGNAdqfdtltsdalglvgvpaavavpcxtiPWQIaqklhavtavleepk  200
ident    |
Sbjct HRRIDIRLQPKDQY--------------------------yCGVL---------------  175
DSSP  LEEEEEEELLHHHH--------------------------hHHHH---------------


DSSP  lllllhhhhHHHHHH----------------------hhllhhhhhhhhHHHHhhhhhhl
Query andrahdlvDLQLLE----------------------gllldadlxptrSACIaifeara  238
ident                                                  |
Sbjct -----yftgSDIFNKnmrahalekgftineytirplgvtgvageplpvdSEQD------i  224
DSSP  -----hhhlLHHHHHhhhhhhhhlleeellllleeellllllllllllllLHHH------h


DSSP  llLLLLLlllllhhhhhhhhhllllllllllllhhhhhhhhhhhhhhhhhllll
Query qhPWPPRvatlphwpliyagalegldhlelartvdaaaqavqrfvaridratkr  292
ident       |
Sbjct fdYIQWR--------------------------------------yrepkdrse  240
```

```
DSSP  hhHLLLL---------------------------------------lllhhhlll
```

No 51: Query=mol1A Sbjct=1jn3A Z-score=7.2

back to top

```
DSSP  ---------------------------llllllllllhhhhhhhhhhhhhhllllhhhhh
Query ---------------------------aapyssspptnlrslrdrltqvaerqgvvfgrlq   33
ident 
Sbjct ddtsssinfltrvtgigpsaarklvdegiktledlrknedklnhhqriglkyfedfekri   60
DSSP  lhhhhhhhhhllllllllhhhhhhhhhlllllhhhhhllhhhllhhhhhhhhhlllhhhlle


DSSP  HHHHHHHHHHHHH-LLLLllLLLLEEEEHHHHHHHHhhllllllLLLLEEEEEL-------
Query RHVAXIVVAQFAA-TLTDdtGAPLLLVKGGSSLELrrgipdsrTSKDFATVAR-------   85
ident                           | |               | |
Sbjct PREEMLQMQDIVLnEVKKldPEYIATVCGSFRRGA-------eSSGDMDVLLThpnftse  113
DSSP  EHHHHHHHHHHHHHhHHHHhlLLLEEEELHHHHLLL-------lEELLEEEEEElllllll


DSSP  -----lLHHHHHHHHHHHHHhhlllleeEEELLLEELllllllLLLEEEEEEEEEELLeEEE
Query -----rDIELIHEQLADAGetgwegftAIFTAPEEIaapgxpVKPRRFTAKLSYRGrAFA  140
ident             |||             |                      |
Sbjct sskqpkLLHRVVEQLQKVR--------FITDTLSKG------ETKFMGVCQLPSEN-EYP  158
DSSP  llllhhHHHHHHHHHHHHHLL--------LEEEEEEEL------LLEEEEEEELLLLLL-LLL


DSSP  EEEEEEEEL-LLLLlllleeeeelhhhhllllllleeeeellhHHHHhhhhhhhhllllll
Query TVPIEVSSV-EAGNadqfdtltsdalglvgvpaavavpcxtiPWQIaqklhavtavleep  199
ident    |
Sbjct HRRIDIRLIpKDQY---------------------------yCGVL--------------  177
DSSP  LEEEEEEELlHHHH---------------------------hHHHH--------------


DSSP  llllllhhhhHHHHHH----------------------hhllhhhhhhhhHHHHhhhhhh
Query kandrahdlvDLQLLE----------------------glllldadlxptrSACIaifear  237
ident                                                   |
Sbjct ------yftgSDIFNKnlrahalekgftineytirplgvtgvageplpvdSEQD------  225
DSSP  ------hlllLHHHHHhhhhhhhhlleeellllleeellllllllllllllLHHH------


DSSP  llllLLLLLlllllhhhhhhhhhlllllllllllhhhhhhhhhhhhhhhhhllll
Query aqhpWPPRVatlphwpliyagalegldhlelartvdaaaqavqrfvaridratkr  292
ident 
Sbjct -ifdYIQWR-------------------------------------yrepkdrse  242
DSSP  -hhhHLLLL-------------------------------------lllhhhlll
```

No 52: Query=mol1A Sbjct=2i9gA Z-score=7.2

back to top

```
DSSP  ------------------------------------------------------------
Query ------------------------------------------------------------    0
ident 
Sbjct etlnggitdmltelanfeknvsqaihkynayrkaasviakyphkiksgaeakklpgvgtk   60
DSSP  llllhhhhhhhhhhhhhhhhllllhhhhhhhhhhhhhhhhlllllllhhhhhlllllllhh


DSSP  --------------------------------------------------llllllllllh
Query --------------------------------------------------aapyssspptnl   11
ident 
Sbjct iaekideflatgklrklekirqddtsssinfltrvsgigpsaarkfvdegiktledlrkn  120
DSSP  hhhhhhhhhhhlllhhhhhhhllhhhhhhhhhlllllllhhhhhhhhhlllllhhhhhll


DSSP  hhhhhhhhhhhhhllllhhhhhHHHHHHHHHHHHH-LLLLllLLLLEEEEHHHHHHHHhhl
Query rslrdrltqvaerqgvvfgrlqRHVAXIVVAQFAA-TLTDdtGAPLLLVKGGSSLELrrg   70
ident                                                 | |
Sbjct edklnhhqriglkyfgdfekriPREEMLQMQDIVLnEVKKvdSEYIATVCGSFRRGA---  177
DSSP  hhhllhhhhhhhhlhhhhhlleEHHHHHHHHHHHHHhHHHHhlLLLEEEELHHHHLLL---


DSSP  lllllLLLLEEEEEL----------lLHHHHHHHHHHHHHhhlllleeEEELLLEELllll
Query ipdsrTSKDFATVAR----------rDIELIHEQLADAGetgwegftAIFTAPEEIaapg  120
```

```
ident       | |                       |||             |
Sbjct ----eSSGDMDVLLThpsftsesqpkLLHQVVEQLQKVH--------FITDTLSKG----  221
DSSP  ----lEELLEEEEEEllllllllllhHHHHHHHHHHHHLL--------LEEEEEEEL----


DSSP  llLLLEEEEEEEEEEL--LEEEEEEEEEEEEELLLLLLllleeeeelhhhhllllllleeeee
Query xpVKPRRFTAKLSYR--GRAFATVPIEVSSVEAGNAdqfdtltsdalglvgvpaavavpc  178
ident            |             |
Sbjct --ETKFMGVCQLPSKndEKEYPHRRIDIRLIPKDQY------------------------  255
DSSP  --LLEEEEEEELLLLLllLLLLLLEEEEEEEELLHHHH------------------------


DSSP  llhHHHHhhhhhhhhllllllllllllhhhhHHHHHH----------------------h
Query xtiPWQIaqklhavtavleepkandrahdlvDLQLLE----------------------g  216
ident
Sbjct --yCGVL--------------------yftgSDIFNKnmrahalekgftineytirplgv  293
DSSP  --hHHHH--------------------hhhlLHHHHHhhhhhhhhlleeellllleeelll


DSSP  hllhhhhhhhhHHHHhhhhhhllllLLLLLllllllhhhhhhhhhllllllllllllhhhhh
Query llldadlxptrSACIaifearaqhpWPPRVatlphwpliyagalegldhlelartvdaaa  276
ident            |
Sbjct tgvageplpvdSEKD-------ifdYIQWK------------------------------  316
DSSP  lllllllllllLHHH-------hhhHLLLL------------------------------


DSSP  hhhhhhhhhhhhllll
Query qavqrfvaridratkr  292
ident
Sbjct -------yrepkdrse  325
DSSP  -------lllhhhlll
```

No 53: Query=mol1A Sbjct=4o5cA Z-score=7.2

back to top

```
DSSP  ------------------------------------------------------------
Query ------------------------------------------------------------    0
ident
Sbjct pqetlnggitdmltelanfeknvsqaihkynayrkaasviakyphkiksgaeakklpgvg   60
DSSP  llllllhhhhhhhhhhhhhhhhllllhhhhhhhhhhhhhhhhlllllllllhhhhhllllll


DSSP  ---------------------------------------------------lllllllll
Query ---------------------------------------------------aapyssppt    9
ident
Sbjct tkiaekideflatgklrklekirqddtsssinfltrvsgigpsaarkfvdegiktledlr  120
DSSP  hhhhhhhhhhhhhllllhhhhhhhhllhhhhhhhhhhlllllllhhhhhhhhhhllllllhhhhh


DSSP  lhhhhhhhhhhhhhhllllhhhhhHHHHHHHHHHHHHH-LLLLllLLLLEEEEHHHHHHHh
Query nlrslrdrltqvaerqgvvfgrlqRHVAXIVVAQFAA-TLTDdtGAPLLLVKGGSSLELr   68
ident                                                   | |
Sbjct knedklnhhqriglkyfgdfekriPREEMLQMQDIVLnEVKKvdSEYIATVCGSFRRGA-  179
DSSP  llhhhllhhhhhhhhlhhhhllleEHHHHHHHHHHHHHhHHHHhlLLLEEEELHHHHLLL-


DSSP  hllllllLLLLEEEEEEL----------lLHHHHHHHHHHHHhhlllleeEEELLLEElll
Query rgipdsrTSKDFATVAR----------rDIELIHEQLADAGetgwegftAIFTAPEEiaa  118
ident         | |                       |||             |
Sbjct ------eSSGDMDVLLThpsftsesqpkLLHQVVEQLQKVH--------FITDTLSK---  222
DSSP  ------lEELLEEEEEEllllllllllHHHHHHHHHHHHLL--------LEEEEEEE---


DSSP  llllLLLEEEEEEEEEEL--LEEEEEEEEEEEEELLLLLLllleeeeelhhhhllllllleee
Query pgxpVKPRRFTAKLSYR--GRAFATVPIEVSSVEAGNAdqfdtltsdalglvgvpaavav  176
ident              |             |
Sbjct ---gETKFMGVCQLPSKndEKEYPHRRIDIRLIPKDQY----------------------  257
DSSP  ---lLLEEEEEEELLLLLllLLLLLLEEEEEEEELLHHHH----------------------


DSSP  eellhHHHHhhhhhhhhllllllllllllHHHH-----------------hhhhhhhhll
Query pcxtiPWQIaqklhavtavleepkandraHDLV-----------------dlqlleglll  219
ident                               |
Sbjct ----yCGVL----------------yftgSDIFnknmrahalekgftineytirplgvtg  297
DSSP  ----hHHHH----------------hhhlLHHHhhhhhhhhhhlleeellllleeellll
```

```
DSSP  hhhhhhhhHHHHHhhhhhlllLLLLLllllllhhhhhhhhhllllllllllhhhhhhhh
Query dadlxptrSACIAifearaqhPWPPRvatlphwpliyagalegldhlelartvdaaaqav  279
ident
Sbjct vageplpvDSEKD-----ifdYIQWK----------------------------------  318
DSSP  llllllllLLHHH-----hhhHLLLL----------------------------------
```

```
DSSP  hhhhhhhhhllll
Query qrfvaridratkr  292
ident
Sbjct ----yrepkdrse  327
DSSP  ----lllhhhlll
```

No 54: Query=mol1A Sbjct=5j0yA Z-score=7.2

back to top

```
DSSP  ------------------------------------------------------------
Query ------------------------------------------------------------    0
ident
Sbjct apqetlnggitdmltelanfeknvsqaihkynayrkaasviakyphkiksgaeakklpgv   60
DSSP  lllhhhhhhhhhhhhhhhhhhhhhllllhhhhhhhhhhhhhhhhllllllllhhhhhlllll
```

```
DSSP  ----------------------------------------------------llllllll
Query ----------------------------------------------------aapysspp    8
ident
Sbjct gtkiaekideflatgklrklekirqddtsssinfltrvsgigpsaarkfvdegiktledl  120
DSSP  lhhhhhhhhhhhhhlllhhhhhhhhlhhhhhhhhhllllllllhhhhhhhhhlllllhhhh
```

```
DSSP  llhhhhhhhhhhhhhhllllhhhhhHHHHHHHHHHHHH-LLLLllLLLLEEEEHHHHHHH
Query tnlrslrdrltqvaerqgvvfgrlqRHVAXIVVAQFAA-TLTDdtGAPLLLVKGGSSLEL   67
ident                                                    | |
Sbjct rknedklnhhqriglkyfgdfekriPREEMLQMQDIVLnEVKKvdSEYIATVCGSFRRGA  180
DSSP  hhlhhhllhhhhhhhhlhhhhhlleEHHHHHHHHHHHHHhHHHHhlLLLEEEELHHHHLLL
```

```
DSSP  hhllllllLLLLEEEEEL---------lLHHHHHHHHHHHHhhllllleeEEELLLEElll
Query rrgipdsrTSKDFATVAR---------rDIELIHEQLADAGetgwegftAIFTAPEEiaa  118
ident          | |                      |||             |
Sbjct -------eSSGDMDVLLThpsftsespkLLHQVVEQLQKVH--------FITDTLSK---  222
DSSP  -------lEELLEEEEEEllllllllllHHHHHHHHHHHHLL--------LEEEEEEE---
```

```
DSSP  llllLLLEEEEEEEEEEL--LEEEEEEEEEEEEELLLLLLllleeeeelhhhhllllllleee
Query pgxpVKPRRFTAKLSYR--GRAFATVPIEVSSVEAGNAdqfdtltsdalglvgvpaavav  176
ident              |             |
Sbjct ---gETKFMGVCQLPSKndEKEYPHRRIDIRLIPKDQY----------------------  257
DSSP  ---lLLEEEEEEELLLLLllLLLLLLLEEEEEEEELLHHHH----------------------
```

```
DSSP  eellhHHHHhhhhhhhhllllllllllllHHHH-------------------hhhhhhhh
Query pcxtiPWQIaqklhavtavleepkandraHDLV-------------------dlqllegl  217
ident                               |
Sbjct ----yCGVL----------------yftgSDIFnknmrahalekgftineytirplgvtg  297
DSSP  ----hHHHH----------------hlllLHHHhhhhhhhhhllleeellllleeeeelll
```

```
DSSP  llhhHHHHhhHHHHHhhhhhlllLLLLLllllllhhhhhhhhhllllllllllhhhhhh
Query lldaDLXPtrSACIAifearaqhPWPPRvatlphwpliyagalegldhlelartvdaaaq  277
ident      |
Sbjct vagePLPV--DSEKD-----ifdYIQWK--------------------------------  318
DSSP  eellLLLL--LLHHH-----hhhHLLLL--------------------------------
```

```
DSSP  hhhhhhhhhhhllll
Query avqrfvaridratkr  292
ident
Sbjct ------yrepkdrse  327
DSSP  ------lllhhhlll
```

No 55: Query=mol1A Sbjct=5db6A Z-score=7.2

back to top

```
DSSP  ------------------------------------------------------------
Query ------------------------------------------------------------     0
ident 
Sbjct pqetlnggitdmltelanfeknvsqaihkynayrkaasviakyphkiksgaeakklpgvg    60
DSSP  llhhhhhhhhhhhhhhhhhhhhhllllhhhhhhhhhhhhhhhlllllllhhhhlllllll


DSSP  ---------------------------------------------------lllllllll
Query ---------------------------------------------------aapyssppt     9
ident 
Sbjct tkiaekideflatgklrklekirqddtsssinfltrvsgigpsaarkfvdegiktledlr   120
DSSP  hhhhhhhhhhhhhlllhhhhhhhllhhhhhhhhhlllllllhhhhhhhhhlllllhhhhh


DSSP  lhhhhhhhhhhhhhhllllhhhhhHHHHHHHHHHHHH-LLLLllLLLLEEEEHHHHHHhh
Query nlrslrdrltqvaerqgvvfgrlqRHVAXIVVAQFAA-TLTDdtGAPLLLVKGGSSLElr    68
ident                                                   | |
Sbjct knedklnhhqriglkyfgdfekriPREEMLQMQDIVLnEVKKvdSEYIATVCGSFRRG--   178
DSSP  hlhhhllhhhhhhhhlhhhhllleEHHHHHHHHHHHHHhHHHHhlLLLEEEEELHHHHLL--


DSSP  hllllLLLLLLEEEEEL----------lLHHHHHHHHHHHHhhllllleeEEELLLEElll
Query rgipdSRTSKDFATVAR----------rDIELIHEQLADAGetgwegftAIFTAPEEiaa   118
ident         | |                       |||             |
Sbjct -----AESSGDMDVLLThpsftsesqpkLLHQVVEQLQKVH--------FITDTLSK---   222
DSSP  -----LLEELLEEEEEllllllllllHHHHHHHHHHHLL--------LEEEEEEE---


DSSP  llllLLLEEEEEEEEEELL--EEEEEEEEEEEELLLLLLllleeeeelhhhhllllllleee
Query pgxpVKPRRFTAKLSYRG--RAFATVPIEVSSVEAGNAdqfdtltsdalglvgvpaavav   176
ident              |             |
Sbjct ---gETKFMGVCQLPSKNdeKEYPHRRIDIRLIPKDQY----------------------   257
DSSP  ---lLLEEEEEEELLLLLLllLLLLLLEEEEEEEELLHHHH----------------------


DSSP  eellhHHHHhhhhhhhhlllllllllllHHHH-----------------hhhhhhhhll
Query pcxtiPWQIaqklhavtavleepkandraHDLV-----------------dlqlleglll   219
ident                               |
Sbjct ----yCGVL----------------yftgSDIFnknmrahalekgftineytirplgvtg   297
DSSP  ----hHHHH----------------hhhlLHHHhhhhhhllllllleeellllleeelllll


DSSP  hhhhhhhhHHHHHhhhhhlllLLLLLLllllllhhhhhhhhhllllllllllhhhhhhhh
Query dadlxptrSACIAifearaqhPWPPRvatlphwpliyagalegldhlelartvdaaaqav   279
ident 
Sbjct vageplpvDSEKD-----ifdYIQWK----------------------------------   318
DSSP  llllllllLLHHH-----hhhHLLLL----------------------------------


DSSP  hhhhhhhhhllll
Query qrfvaridratkr  292
ident 
Sbjct ----yrepkdrse  327
DSSP  ----lllhhhlll
```

No 56: Query=mol1A Sbjct=4nlnA Z-score=7.2

back to top

```
DSSP  ------------------------------------------------------------
Query ------------------------------------------------------------     0
ident 
Sbjct pqetlnggitdmltelanfeknvsqaihkynayrkaasviakyphkiksgaeakklpgvg    60
DSSP  llllllhhhhhhhhhhhhhhhhhllllhhhhhhhhhhhhhhhhlllllllhhhhhllllll


DSSP  ---------------------------------------------------lllllllll
Query ---------------------------------------------------aapyssppt     9
ident 
Sbjct tkiaekideflatgklrklekirqddtsssinfltrvsgigpsaarkfvdegiktledlr   120
DSSP  hhhhhhhhhhhhhlllhhhhhhhllhhhhhhhhhlllllllhhhhhhhhhlllllhhhhh


DSSP  lhhhhhhhhhhhhhhllllhHHHHHHHHHHHHHHHHH-LLLLllLLLLEEEEHHHHHHHh
Query nlrslrdrltqvaerqgvvfGRLQRHVAXIVVAQFAA-TLTDdtGAPLLLVKGGSSLELr    68
```

```
ident                                                   | |
Sbjct knedklnhhqriglkyfgdfEKRIPREEMLQMQDIVLnEVKKvdSEYIATVCGSFRRGA-  179
DSSP  llhhhllhhhhhhhhlhhhhHLLEEHHHHHHHHHHHHhHHHHhlLLLEEEELHHHHLLL-


DSSP  hllllllLLLLEEEEEL---------lLHHHHHHHHHHHHhhlllleeEEELLLEELlll
Query rgipdsrTSKDFATVAR---------rDIELIHEQLADAGetgwegftAIFTAPEEIaap  119
ident         | |                      |||             |
Sbjct ------eSSGDMDVLLThpsftsespkLLHQVVEQLQKVH--------FITDTLSKG---  222
DSSP  ------lEELLEEEEEElllllllllhHHHHHHHHHHHLL--------LEEEEEEEL---


DSSP  lllLLLEEEEEEEEEL--LEEEEEEEEEEEEL-LLLLlllleeeeelhhhhlllllleee
Query gxpVKPRRFTAKLSYR--GRAFATVPIEVSSV-EAGNadqfdtltsdalglvgvpaavav  176
ident             |             |
Sbjct ---ETKFMGVCQLPSKndEKEYPHRRIDIRLIpKDQY-----------------------  256
DSSP  ---LLEEEEEEELLLLllLLLLLLEEEEEEELlHHHH-----------------------


DSSP  eellhHHHHhhhhhhhhhlllllllllllhhhhHHHHHH---------------------
Query pcxtiPWQIaqklhavtavleepkandrahdlvDLQLLE---------------------  215
ident
Sbjct ----yCGVL--------------------yftgSDIFNKnmrahalekgftineytirpl  292
DSSP  ----hHHHH--------------------hhhlLHHHHhhhhhhhhlleeellllleeel


DSSP  hhllhhhhhhhHHHHHhhhhhhllllLLLLLllllllhhhhhhhhhllllllllllhhhh
Query gllldadlxptRSACIaifearaqhpWPPRVatlphwpliyagalegldhlelartvdaa  275
ident
Sbjct gvtgvageplpVDSEK-------difDYIQW-----------------------------  316
DSSP  lllllllllllLLLHH-------hhhHHLLL-----------------------------


DSSP  hhhhhhhhhhhhhllll
Query aqavqrfvaridratkr  292
ident
Sbjct -------kyrepkdrse  326
DSSP  -------llllhhhlll
```

No 57: Query=mol1A Sbjct=4o9mA Z-score=7.2

back to top

```
DSSP  ------------------------------------------------------------
Query ------------------------------------------------------------    0
ident
Sbjct pqetlnggitdmltelanfeknvsqaihkynayrkaasviakyphkiksgaeakklpgvg   60
DSSP  llhhhhhhhhhhhhhhhhhhhhllllhhhhhhhhhhhhhhhhlllllllhhhhhllllll


DSSP  ---------------------------------------------------lllllllll
Query ---------------------------------------------------aapyssppt    9
ident
Sbjct tkiaekideflatgklrklekirqddtsssinfltrvsgigpsaarkfvdegiktledlr  120
DSSP  hhhhhhhhhhhhhhlllhhhhhhhhlhhhhhhhhhllllllhhhhhhhhhlllllhhhhh


DSSP  lhhhhhhhhhhhhhhllllhHHHHHHHHHHHHHHHHHL-LLLLlLLLLEEEEHHHHHHHh
Query nlrslrdrltqvaerqgvvfGRLQRHVAXIVVAQFAAT-LTDDtGAPLLLVKGGSSLELr   68
ident                                                   | |
Sbjct knedklnhhqriglkyfgdfEKRIPREEMLQMQDIVLNeVKKVdSEYIATVCGSFRRGA-  179
DSSP  llhhhllhhhhhhhhlhhhhHLLEEHHHHHHHHHHHHHhHHHHlLLLEEEELHHHHLLL-


DSSP  hllllllLLLLEEEEEL---------lLHHHHHHHHHHHHhhlllleeEEELLLEELlll
Query rgipdsrTSKDFATVAR---------rDIELIHEQLADAGetgwegftAIFTAPEEIaap  119
ident         | |                      |||             |
Sbjct ------eSSGDMDVLLThpsftsespkLLHQVVEQLQKVH--------FITDTLSKG---  222
DSSP  ------lEELLEEEEEEllllllllllHHHHHHHHHHHLL--------LEEEEEEEL---


DSSP  lllLLLEEEEEEEEEL--LEEEEEEEEEEEEL-LLLLlllleeeeelhhhhlllllleee
Query gxpVKPRRFTAKLSYR--GRAFATVPIEVSSV-EAGNadqfdtltsdalglvgvpaavav  176
ident             |             |
Sbjct ---ETKFMGVCQLPSKndEKEYPHRRIDIRLIpKDQY-----------------------  256
DSSP  ---LLEEEEEEELLLLllLLLLLLEEEEEEELlHHHH-----------------------
```

```
DSSP  eellhHHHHhhhhhhhhlllllllllllhhhhHHHHHH---------------------
Query pcxtiPWQIaqklhavtavleepkandrahdlvDLQLLE---------------------  215
ident
Sbjct ----yCGVL--------------------yftgSDIFNKnmrahalekgftineytirpl  292
DSSP  ----hHHHH--------------------hhhlLHHHHHhhhhhhhhlleeellllleeel
```

```
DSSP  -hhllhhhhhhhhHHHHhhhhhhlllLLLLLLlllllhhhhhhhhhlllllllllllhhh
Query -gllldadlxptrSACIaifearaqhPWPPRVatlphwpliyagalegldhlelartvda  274
ident              |
Sbjct gvtgvageplpvdSEKD-------ifDYIQWK----------------------------  317
DSSP  lllllllllllllLHHH-------hhLLLLLL----------------------------
```

```
DSSP  hhhhhhhhhhhhhhllll
Query aaqavqrfvaridratkr  292
ident
Sbjct ---------yrepkdrse  326
DSSP  ---------lllhhhlll
```

No 58: Query=mol1A Sbjct=4m91A Z-score=7.2

back to top

```
DSSP  ------------------------------------------------------------
Query ------------------------------------------------------------    0
ident
Sbjct tlnggitdmltelanfeknvsqaihkynayrkaasviakyphkiksgaeakklpgvgtki   60
DSSP  lllhhhhhhhhhhhhhhhhllllhhhhhhhhhhhhhhhhlllllllhhhhhlllllhhh
```

```
DSSP  ------------------------------------------------llllllllllhh
Query ------------------------------------------------aapysspptnlr   12
ident
Sbjct aekideflatgklrklekirqddtsssinfltrvsgigpsaarkfvdegiktledlrkne  120
DSSP  hhhhhhhhhhlllhhhhhhhllhhhhhhhhhlllllllhhhhhhhhhlllllhhhhhllh
```

```
DSSP  hhhhhhhhhhhhllllhHHHHHHHHHHHHHHHHHL-LLLLLLLLLEEEEHHHHHHHhhll
Query slrdrltqvaerqgvvfGRLQRHVAXIVVAQFAAT-LTDDTGAPLLLVKGGSSLELrrgi   71
ident                                                |  |
Sbjct dklnhhqriglkyfgdfEKRIPREEMLQMQDIVLNeVKKVDSEYIATVCGSFRRGA----  176
DSSP  hhllhhhhhhhhlhhhhLLLEEHHHHHHHHHHHHHHhHHHHLLLLEEEELHHHHLLL----
```

```
DSSP  llllLLLLEEEEEL--------lLHHHHHHHHHHHHhhlllleeEEELLLEELllllllL
Query pdsrTSKDFATVAR--------rDIELIHEQLADAGetgwegftAIFTAPEEIaapgxpV  123
ident      | |                     |||             |
Sbjct ---eSSGDMDVLLThpsftseskLLHQVVEQLQKVH--------FITDTLSKG------E  219
DSSP  ---lEELLEEEEEEllllllllHHHHHHHHHHHHLL--------LEEEEEEEL------L
```

```
DSSP  LLEEEEEEEEElLEEEEEEEEEEEEL-LLLLllleeeeelhhhhllllllleeeeellhH
Query KPRRFTAKLSYrGRAFATVPIEVSSV-EAGNadqfdtltsdalglvgvpaavavpcxtiP  182
ident         |           |
Sbjct TKFMGVCQLPS-EKEYPHRRIDIRLIpKDQY---------------------------yC  251
DSSP  LEEEEEELLLL-LLLLLLEEEEEEELlHHHH---------------------------hH
```

```
DSSP  HHHhhhhhhhhllllllllllllHHHHHHH--------------hhhhhllhHHHHhhhH
Query WQIaqklhavtavleepkandraHDLVDLQ--------------llegllldADLXptrS  228
ident                         |                             |
Sbjct GVL----------------yftgSDIFNKNmrahalekgftinkytirpvagEPLP---V  292
DSSP  HHH----------------hhhlLHHHHHHhhhhhhhlllleellllleellllLLLL---L
```

```
DSSP  HHHhhhhhhllllLLLLLlllllhhhhhhhhhllllllllllhhhhhhhhhhhhhhhhh
Query ACIaifearaqhpWPPRVatlphwpliyagalegldhlelartvdaaaqavqrfvaridr  288
ident
Sbjct SEK-------difDYIQW------------------------------------kyrepk  309
DSSP  LHH-------hhhHHLLL------------------------------------llllhh
```

```
DSSP  llll
Query atkr  292
ident
```

```
Sbjct drse  313
DSSP  hlll
```

No 59: Query=mol1A Sbjct=5j0rA Z-score=7.2

back to top

```
DSSP  ------------------------------------------------------------
Query ------------------------------------------------------------    0
ident 
Sbjct apqetlnggitdmltelanfeknvsqaihkynayrkaasviakyphkiksgaeakklpgv   60
DSSP  lllllllhhhhhhhhhhhhhhhhllllhhhhhhhhhhhhhhhhlllllllhhhhhlllll


DSSP  ----------------------------------------------------llllllll
Query ----------------------------------------------------aapysspp    8
ident 
Sbjct gtkiaekideflatgklrklekirqddtsssinfltrvsgigpsaarkfvdegiktledl  120
DSSP  lhhhhhhhhhhhhhlllhhhhhhhhlhhhhhhhhhlllllllhhhhhhhhhlllllhhhh


DSSP  llhhhhhhhhhhhhhhllllhhhhhHHHHHHHHHHHHHH-LLLLllLLLLEEEEHHHHHHH
Query tnlrslrdrltqvaerqgvvfgrlqRHVAXIVVAQFAA-TLTDdtGAPLLLVKGGSSLEL   67
ident                                                    | |
Sbjct rknedklnhhqriglkyfgdfekriPREEMLQMQDIVLnEVKKvdSEYIATVCGSFRRGA  180
DSSP  hhlhhhllhhhhhhhhlhhhhhlleEHHHHHHHHHHHHhHHHHhlLLLEEEELHHHHLLL


DSSP  hhllllllLLLLEEEEEL---------lLHHHHHHHHHHHhhllllleeEEELLLEElll
Query rrgipdsrTSKDFATVAR---------rDIELIHEQLADAGetgwegftAIFTAPEEiaa  118
ident          | |                      |||             |
Sbjct -------eSSGDMDVLLThpsftsespkLLHQVVEQLQKVH--------FITDTLSK---  222
DSSP  -------lEELLEEEEEEllllllllllHHHHHHHHHHHLL--------LEEEEEEE---


DSSP  llllLLLEEEEEEEEEL--LEEEEEEEEEEEEL-LLLLlllleeeeelhhhhllllllee
Query pgxpVKPRRFTAKLSYR--GRAFATVPIEVSSV-EAGNadqfdtltsdalglvgvpaava  175
ident              |             |
Sbjct ---gETKFMGVCQLPSKndEKEYPHRRIDIRLIpKDQY----------------------  257
DSSP  ---lLLEEEEEELLLLLllLLLLLLEEEEEEELlHHHH----------------------


DSSP  eeellhHHHHhhhhhhhhllllllllllllHHHH-------------------hhhhhhh
Query vpcxtiPWQIaqklhavtavleepkandraHDLV-------------------dlqlleg  216
ident                                |
Sbjct -----yCGVL----------------yftgSDIFnknmrahalekgftineytirplgvt  296
DSSP  -----hHHHH----------------hhhlLHHHhhhhhhhhhhlleeellllleeellll


DSSP  hllhhHHHHhhHHHHHhhhhhlllLLLLLlllllhhhhhhhhhllllllllllllhhhhh
Query llldaDLXPtrSACIAifearaqhPWPPRvatlphwpliyagalegldhlelartvdaaa  276
ident       |
Sbjct gvagePLPV--DSEKD-----ifdYIQWK-------------------------------  318
DSSP  lllllLLLL--LLHHH-----hhhHLLLL-------------------------------


DSSP  hhhhhhhhhhhhllll
Query qavqrfvaridratkr  292
ident 
Sbjct -------yrepkdrse  327
DSSP  -------lllhhhlll
```

No 60: Query=mol1A Sbjct=4r63A Z-score=7.2

back to top

```
DSSP  ------------------------------------------------------------
Query ------------------------------------------------------------    0
ident 
Sbjct pqetlnggitdmltelanfeknvsqaihkynayrkaasviakyphkiksgaeakklpgvg   60
DSSP  llhhhhhhhhhhhhhhhhhhhhllllhhhhhhhhhhhhhhhllllllllhhhhlllllll


DSSP  ---------------------------------------------------lllllllll
```

```
Query ---------------------------------------------------aapyssppt    9
ident
Sbjct tkiaekideflatgklrklekirqddtsssinfltrvsgigpsaarkfvdegiktledlr  120
DSSP  hhhhhhhhhhhhhlllhhhhhhhhlhhhhhhhhhlllllllhhhhhhhhhlllllhhhhh


DSSP  lhhhhhhhhhhhhhhllllhhhhhHHHHHHHHHHHHH-LLLLllLLLLEEEEHHHHHHHh
Query nlrslrdrltqvaerqgvvfgrlqRHVAXIVVAQFAA-TLTDdtGAPLLLVKGGSSLELr   68
ident                                                   | |
Sbjct knedklnhhqriglkyfgdfekriPREEMLQMQDIVLnEVKKvdSEYIATVCGSFRRGA-  179
DSSP  hlhhhllhhhhhhhhlhhhhhlleEHHHHHHHHHHHHHhHHHHhlLLLEEEELHHHHLLL-


DSSP  hllllllLLLLEEEEEL----------lLHHHHHHHHHHHHhhlllleeEEELLLEElll
Query rgipdsrTSKDFATVAR----------rDIELIHEQLADAGetgwegftAIFTAPEEiaa  118
ident         | |                       |||             |
Sbjct ------eSSGDMDVLLThpsftsesqpkLLHQVVEQLQKVH--------FITDTLSK---  222
DSSP  ------lEELLEEEEEEllllllllllHHHHHHHHHHHLL--------LEEEEEEE---


DSSP  llllLLLEEEEEEEEEELL--EEEEEEEEEEEEL-LLLLllllleeeeelhhhhlllllllee
Query pgxpVKPRRFTAKLSYRG--RAFATVPIEVSSV-EAGNadqfdtltsdalglvgvpaava  175
ident              |             |
Sbjct ---gETKFMGVCQLPSKNdeKEYPHRRIDIALIpKDQY----------------------  257
DSSP  ---lLLEEEEEEELLLLLLllLLLLLEEEEEEEELlHHHH----------------------


DSSP  eeellhHHHHhhhhhhhhllllllllllllHHHH-----------------hhhhhhhhl
Query vpcxtiPWQIaqklhavtavleepkandraHDLV-----------------dlqlleglll  218
ident                                |
Sbjct -----yCGVL----------------yftgSDIFnknmrahalekgftineytirplgvt  296
DSSP  -----hHHHH----------------hhhlLHHHhhhhhhhhhhlleeellllleeellll


DSSP  lhhhhhhhhHHHHHhhhhhlllLLLLLllllllhhhhhhhhhllllllllllhhhhhhh
Query ldadlxptrSACIAifearaqhPWPPRvatlphwpliyagalegldhlelartvdaaaqa  278
ident
Sbjct gvageplpvDSEKD-----ifdYIQWK---------------------------------  318
DSSP  lllllllllLLHHH-----hhhHLLLL---------------------------------


DSSP  hhhhhhhhhhllll
Query vqrfvaridratkr  292
ident
Sbjct -----yrepkdrse  327
DSSP  -----lllhhhlll
```

No 61: Query=mol1A Sbjct=5dbcA Z-score=7.2

back to top

```
DSSP  ------------------------------------------------------------
Query ------------------------------------------------------------    0
ident
Sbjct pqetlnggitdmltelanfeknvsqaihkynayrkaasviakyphkiksgaeakklpgvg   60
DSSP  llllllhhhhhhhhhhhhhhhhhllllhhhhhhhhhhhhhhhlllllllllhhhhhllllll


DSSP  ---------------------------------------------------lllllllll
Query ---------------------------------------------------aapyssppt    9
ident
Sbjct tkiaekideflatgklrklekirqddtsssinfltrvsgigpsaarkfvdegiktledlr  120
DSSP  hhhhhhhhhhhhhlllhhhhhhhhlhhhhhhhhhlllllllhhhhhhhhhlllllhhhhh


DSSP  lhhhhhhhhhhhhhhllllhhhhhHHHHHHHHHHHHH-LLLLllLLLLEEEEHHHHHHHh
Query nlrslrdrltqvaerqgvvfgrlqRHVAXIVVAQFAA-TLTDdtGAPLLLVKGGSSLELr   68
ident                                                   | |
Sbjct knedklnhhqriglkyfgdfekriPREEMLQMQDIVLnEVKKvdSEYIATVCGSFRRGA-  179
DSSP  llhhhllhhhhhhhhlhhhhhlleEHHHHHHHHHHHHHhHHHHhlLLLEEEELHHHLLLL-


DSSP  hllllllLLLLEEEEEL----------lLHHHHHHHHHHHHhhlllleeEEELLLEElll
Query rgipdsrTSKDFATVAR----------rDIELIHEQLADAGetgwegftAIFTAPEEiaa  118
ident         | |                       |||             |
Sbjct ------eSSGDMDVLLThpsftsesqpkLLHQVVEQLQKVH--------FITDTLSK---  222
DSSP  ------lEELLEEEEEEllllllllllLLHHHHHHHHHLL--------LEEEEEEE---
```

```
DSSP  llllLLLEEEEEEEEEELL--EEEEEEEEEEEEELLLLLllleeeeelhhhhlllllleee
Query pgxpVKPRRFTAKLSYRG--RAFATVPIEVSSVEAGNAdqfdtltsdalglvgvpaavav  176
ident              |             |
Sbjct ---gETKFMGVCQLPSKNdeKEYPHRRIDIRLIPKDQY----------------------  257
DSSP  ---lLLEEEEEEELLLLLllLLLLLEEEEEEELLHHHH----------------------


DSSP  eellhHHHhhhhhhhhlllllllllllllHHHH-----------------hhhhhhhhll
Query pcxtiPWQIaqklhavtavleepkandraHDLV-----------------dlqlleglll  219
ident                               |
Sbjct ----yCGVL----------------yftgSDIFnknmrahalekgftineytirplgvtg  297
DSSP  ----hHHHH----------------hhhlLHHHhhhhhhhhhllleeellllleeellll


DSSP  hhhhhhhhHHHHHhhhhhlllLLLLLlllllhhhhhhhhhllllllllllhhhhhhhhh
Query dadlxptrSACIAifearaqhPWPPRvatlphwpliyagalegldhlelartvdaaaqav  279
ident
Sbjct vageplpvDSEKD-----ifdYIQWK----------------------------------  318
DSSP  llllllllLLHHH-----hhhHLLLL----------------------------------


DSSP  hhhhhhhhhllll
Query qrfvaridratkr  292
ident
Sbjct ----yrepkdrse  327
DSSP  ----lllhhhlll
```

No 62: Query=mol1A Sbjct=5dbaA Z-score=7.2

back to top

```
DSSP  ------------------------------------------------------------
Query ------------------------------------------------------------    0
ident
Sbjct pqetlnggitdmltelanfeknvsqaihkynayrkaasviakyphkiksgaeakklpgvg   60
DSSP  llhhhhhhhhhhhhhhhhhhhhllllhhhhhhhhhhhhhhhhllllllllhhhhhlllll


DSSP  ---------------------------------------------------lllllllll
Query ---------------------------------------------------aapyssppt    9
ident
Sbjct tkiaekideflatgklrklekirqddtsssinfltrvsgigpsaarkfvdegiktledlr  120
DSSP  hhhhhhhhhhhhhlllhhhhhhhhlhhhhhhhhhllllllhhhhhhhhhhllllllhhhhh


DSSP  lhhhhhhhhhhhhhhhllllhhhhHHHHHHHHHHHHH-LLLLllLLLLEEEEHHHHHHHh
Query nlrslrdrltqvaerqgvvfgrlqRHVAXIVVAQFAA-TLTDdtGAPLLLVKGGSSLELr   68
ident                                                   | |
Sbjct knedklnhhqriglkyfgdfekriPREEMLQMQDIVLnEVKKvdSEYIATVCGSFRRGA-  179
DSSP  hlhhhllhhhhhhhhlhhhhhlleEHHHHHHHHHHHHhHHHHhlLLLEEEELHHHHLLL-


DSSP  hllllllLLLLEEEEEL----------lLHHHHHHHHHHHHhhlllleeEEELLLEElll
Query rgipdsrTSKDFATVAR----------rDIELIHEQLADAGetgwegftAIFTAPEEiaa  118
ident         | |                       |||             |
Sbjct ------eSSGDMDVLLThpsftsesqpkLLHQVVEQLQKVH--------FITDTLSK---  222
DSSP  ------lEELLEEEEEEllllllllllllHHHHHHHHHHHLL--------LEEEEEEE---


DSSP  llllLLLEEEEEEEEEL--LEEEEEEEEEEEEL-LLLLlllleeeeelhhhhllllllee
Query pgxpVKPRRFTAKLSYR--GRAFATVPIEVSSV-EAGNadqfdtltsdalglvgvpaava  175
ident              |             |
Sbjct ---gETKFMGVCQLPSKndEKEYPHRRIDIRLIpKDQY----------------------  257
DSSP  ---lLLEEEEEEELLLLLllLLLLLEEEEEEEElHHHH----------------------


DSSP  eeellhHHHhhhhhhhhlllllllllllllHHHH-------------------hhhhhhh
Query vpcxtiPWQIaqklhavtavleepkandraHDLV-------------------dlqlleg  216
ident                                |
Sbjct -----yCGVL----------------yftgSDIFnknmrahalekgftineytirplgvt  296
DSSP  -----hHHHH----------------hhhlLHHHhhhhhhhhhllleeelllleeellll


DSSP  hllhhHHHHhhHHHHHhhhhhlllLLLLLlllllhhhhhhhhhllllllllllhhhhhhh
Query llldaDLXPtrSACIAifearaqhPWPPRvatlphwpliyagalegldhlelartvdaaa  276
```

```
ident       |
Sbjct gvagePLPV--DSEKD-----ifdYIQWK-------------------------------  318
DSSP  lllllLLLL--LLHHH-----hhhHLLLL-------------------------------


DSSP  hhhhhhhhhhhhllll
Query qavqrfvaridratkr  292
ident
Sbjct -------yrepkdrse  327
DSSP  -------lllhhhlll
```

No 63: Query=mol1A Sbjct=3lqcB Z-score=7.2

back to top

```
DSSP  lllllllllhhhhhhhhhhhhhhllllhhhhhHHHHHHHHHHHHH-LLLLllLLLLLEEE
Query aapyssspptnlrslrdrltqvaerqgvvfgrlqRHVAXIVVAQFAA-TLTDdtGAPLLLV   59
ident                                                            |
Sbjct ------------------------yfedfekriPREEMLQMQDIVLnEVKKldPEYIATV   36
DSSP  ------------------------lllllllleEHHHHHHHHHHHHHhHHHHhlLLLEEEE


DSSP  EHHHHHHHhhllllllLLLLEEEEEL------------lLHHHHHHHHHHHHhhlllllee
Query KGGSSLELrrgipdsrTSKDFATVAR------------rDIELIHEQLADAGetgwegft  107
ident  |               | |                         |||
Sbjct CGSFRRGA-------eSSGDMDVLLThpnftsesskqpkLLHRVVEQLQKVR--------   81
DSSP  LHHHHLLL-------lEELLEEEEEEllllllllllllhHHHHHHHHHHHHLL--------


DSSP  EEELLLEELllllllLLLEEEEEEEEEL--LEEEEEEEEEEEEELLLLLLllleeeeelhh
Query AIFTAPEEIaapgxpVKPRRFTAKLSYR--GRAFATVPIEVSSVEAGNAdqfdtltsdal  165
ident  |                      |             |
Sbjct FITDTLSKG------ETKFMGVCQLPSEndENEYPHRRIDIRLIPKDQY-----------  124
DSSP  LEEEEEEEL------LLEEEEEEELLLLLllLLLLLLEEEEEEEELLHHHH-----------


DSSP  hhllllllleeeeellhHHHHhhhhhhhhllllllllllllhhhhHHHHHH----------
Query glvgvpaavavpcxtiPWQIaqklhavtavleepkandrahdlvDLQLLE----------  215
ident
Sbjct ---------------yCGVL--------------------yftgSDIFNKnmrahalekg  149
DSSP  ---------------hHHHH--------------------hhhlLHHHHHhhhhhhhhll


DSSP  -----------hhllhhhhhhhHHHHHhhhhhhllllLLLLLllllllhhhhhhhhhllll
Query -----------gllldadlxptRSACIaifearaqhpWPPRVatlphwpliyagalegld  264
ident
Sbjct ftineytirplgvtgvageplpVDSEQ-------difDYIQW------------------  184
DSSP  eeellllleeelllllllllllLLLHH-------hhhHHLLL------------------


DSSP  lllllllhhhhhhhhhhhhhhhhhhllll
Query hlelartvdaaaqavqrfvaridratkr  292
ident
Sbjct -------------------ryrepkdrs  193
DSSP  -------------------llllhhhll
```

No 64: Query=mol1A Sbjct=5j0qA Z-score=7.1

back to top

```
DSSP  ------------------------------------------------------------
Query ------------------------------------------------------------    0
ident
Sbjct apqetlnggitdmltelanfeknvsqaihkynayrkaasviakyphkiksgaeakklpgv   60
DSSP  lllhhhhhhhhhhhhhhhhhhhhllllhhhhhhhhhhhhhhhhllllllllhhhhhlllll


DSSP  ----------------------------------------------------llllllll
Query ----------------------------------------------------aapysspp    8
ident
Sbjct gtkiaekideflatgklrklekirqddtsssinfltrvsgigpsaarkfvdegiktledl  120
DSSP  lhhhhhhhhhhhhhlllhhhhhhhhlhhhhhhhhhlllllllhhhhhhhhhlllllhhhh
```

```
DSSP  llhhhhhhhhhhhhhhllllhhhhhHHHHHHHHHHHHHH-LLLLllLLLLEEEEHHHHHHH
Query tnlrslrdrltqvaerqgvvfgrlqRHVAXIVVAQFAA-TLTDdtGAPLLLVKGGSSLEL   67
ident                                                    | |
Sbjct rknedklnhhqriglkyfgdfekriPREEMLQMQDIVLnEVKKvdSEYIATVCGSFRRGA  180
DSSP  hhlhhhllhhhhhhhhlhhhhhlleEHHHHHHHHHHHHhHHHHhlLLLEEEELLHHHHLL


DSSP  hhllllllLLLLEEEEEL------------lLHHHHHHHHHHHHhhllllleeEEELLLEE
Query rrgipdsrTSKDFATVAR------------rDIELIHEQLADAGetgwegftAIFTAPEE  115
ident          | |                         |||             |
Sbjct -------eSSGDMDVLLThpsftsestkqpkLLHQVVEQLQKVH--------FITDTLSK  225
DSSP  -------lEELLEEEEEEllllllllllllHHHHHHHHHHHLL--------LEEEEEEE


DSSP  llllllLLLEEEEEEEEEEL--LEEEEEEEEEEEEL-LLLLllllleeeeelhhhhlllll
Query iaapgxpVKPRRFTAKLSYR--GRAFATVPIEVSSV-EAGNadqfdtltsdalglvgvpa  172
ident                 |             |
Sbjct ------gETKFMGVCQLPSKndEKEYPHRRIDIRLIpKDQY-------------------  260
DSSP  ------lLLEEEEEELLLLLlLLLLLLEEEEEEELlHHHH-------------------


DSSP  leeeeellhHHHHhhhhhhhhllllllllllllHHHH-------------------hhhh
Query avavpcxtiPWQIaqklhavtavleepkandraHDLV-------------------dlql  213
ident                                   |
Sbjct --------yCGVL----------------yftgSDIFnknmrahalekgftineytirpl  296
DSSP  --------hHHHH----------------hhhlLHHHhhhhhhhhhllllleellllleell


DSSP  hhhhllhhHHHHhhHHHHHhhhhhlllLLLLLllllllhhhhhhhhhlllllllllllhh
Query legllldaDLXPtrSACIAifearaqhPWPPRvatlphwpliyagalegldhlelartvd  273
ident          |
Sbjct gvtgvagePLPV--DSEKD-----ifdYIQWK----------------------------  321
DSSP  lllllllLLLL--LLHHH-----hhhHLLLL----------------------------


DSSP  hhhhhhhhhhhhhhhlllll
Query aaaqavqrfvaridratkr  292
ident
Sbjct ----------yrepkdrse  330
DSSP  ----------lllhhhlll
```

No 65: Query=mol1A Sbjct=5j0wA Z-score=7.1

back to top

```
DSSP  ------------------------------------------------------------
Query ------------------------------------------------------------    0
ident
Sbjct pqetlnggitdmltelanfeknvsqaihkynayrkaasviakyphkiksgaeakklpgvg   60
DSSP  llllllhhhhhhhhhhhhhhhhllllhhhhhhhhhhhhhhhhhlllllllhhhhhlllll


DSSP  ---------------------------------------------------lllllllll
Query ---------------------------------------------------aapyssppt    9
ident
Sbjct tkiaekideflatgklrklekirqddtsssinfltrvsgigpsaarkfvdegiktledlr  120
DSSP  hhhhhhhhhhhhhlllhhhhhhhllhhhhhhhhhllllllhhhhhhhhhlllllhhhhh


DSSP  lhhhhhhhhhhhhhhllllhhhhhHHHHHHHHHHHHHH-LLLLllLLLLEEEEHHHHHHHh
Query nlrslrdrltqvaerqgvvfgrlqRHVAXIVVAQFAA-TLTDdtGAPLLLVKGGSSLELr   68
ident                                                   | |
Sbjct knedklnhhqriglkyfgdfekriPREEMLQMQDIVLnEVKKvdSEYIATVCGSFRRGA-  179
DSSP  hlhhhllhhhhhhhhlllhhhlleEHHHHHHHHHHHHhHHHHhlLLLEEEELHHHHLLL-


DSSP  hllllllLLLLEEEEEL------------lLHHHHHHHHHHHHhhllllleeEEELLLEEl
Query rgipdsrTSKDFATVAR------------rDIELIHEQLADAGetgwegftAIFTAPEEi  116
ident         | |                         |||             |
Sbjct ------eSSGDMDVLLThpsftsestkqpkLLHQVVEQLQKVH--------FITDTLSK-  224
DSSP  ------lEELLEEEEEEllllllllllhhhHHHHHHHHHHHLL--------LEEEEEEE-


DSSP  lllllLLLEEEEEEEEEELL--EEEEEEEEEEEELLLLLLllleeeeelhhhhllllllle
Query aapgxpVKPRRFTAKLSYRG--RAFATVPIEVSSVEAGNAdqfdtltsdalglvgvpaav  174
ident                |             |
Sbjct -----gETKFMGVCQLPSKNdeKEYPHRRIDIRLIPKDQY--------------------  259
```

```
DSSP  -----lLLEEEEEELLLLLlllLLLLLEEEEEEELLLLLH--------------------


DSSP  eeeellhHHHHhhhhhhhhhlllllllllllHHHH-----------------hhhhhhhh
Query avpcxtiPWQIaqklhavtavleepkandraHDLV-----------------dlqllegl  217
ident                                 |
Sbjct ------yCGVL----------------yftgSDIFnknmrahalekgftineytirplgv  297
DSSP  ------hHHHH----------------hhhlLHHHhhhhhhhhhhlleeellllleeelll


DSSP  llhhhhhhhhHHHHHhhhhhlllLLLLLllllllhhhhhhhhhlllllllllllhhhhhh
Query lldadlxptrSACIAifearaqhPWPPRvatlphwpliyagalegldhlelartvdaaaq  277
ident
Sbjct tgvageplpvDSEKD-----ifdYIQWK--------------------------------  320
DSSP  llllllllllLLHHH-----hhhHLLLL--------------------------------


DSSP  hhhhhhhhhhhllll
Query avqrfvaridratkr  292
ident
Sbjct ------yrepkdrse  329
DSSP  ------lllhhhlll
```

No 66: Query=mol1A Sbjct=5j0pA Z-score=7.1

back to top

```
DSSP  ------------------------------------------------------------
Query ------------------------------------------------------------    0
ident
Sbjct apqetlnggitdmltelanfeknvsqaihkynayrkaasviakyphkiksgaeakklpgv   60
DSSP  lllhhhhhhhhhhhhhhhhhhhhllllhhhhhhhhhhhhhhhllllllllhhhhhlllll


DSSP  ----------------------------------------------------llllllll
Query ----------------------------------------------------aapysspp    8
ident
Sbjct gtkiaekideflatgklrklekirqddtsssinfltrvsgigpsaarkfvdegiktledl  120
DSSP  lhhhhhhhhhhhhhlllhhhhhhhhlhhhhhhhhhlllllllhhhhhhhhhlllllhhhh


DSSP  llhhhhhhhhhhhhhhllllhhhhhHHHHHHHHHHHHH-LLLLllLLLLEEEEHHHHHHH
Query tnlrslrdrltqvaerqgvvfgrlqRHVAXIVVAQFAA-TLTDdtGAPLLLVKGGSSLEL   67
ident                                                    | |
Sbjct rknedklnhhqriglkyfgdfekriPREEMLQMQDIVLnEVKKvdSEYIATVCGSFRRGA  180
DSSP  hhlhhhllhhhhhhhhlhhhhllleEHHHHHHHHHHHHhHHHHhlLLLEEEELHHHHLLL


DSSP  hhllllllLLLLEEEEEL------------lLHHHHHHHHHHHHhhllllleeEEELLLEE
Query rrgipdsrTSKDFATVAR------------rDIELIHEQLADAGetgwegftAIFTAPEE  115
ident          | |                         |||             |
Sbjct -------eSSGDMDVLLThpsftsestkqpkLLHQVVEQLQKVH--------FITDTLSK  225
DSSP  -------lEELLEEEEEElllllllllllllHHHHHHHHHHHLL--------LEEEEEEE


DSSP  lllllllLLLEEEEEEEEEL--lEEEEEEEEEEEELLLLLLllleeeeelhhhhllllll
Query iaapgxpVKPRRFTAKLSYR--gRAFATVPIEVSSVEAGNAdqfdtltsdalglvgvpaa  173
ident                 |             |
Sbjct ------gETKFMGVCQLPSKndeKEYPHRRIDIRLIPKDQY-------------------  260
DSSP  ------lLLEEEEEELLLLLlllLLLLLEEEEEEELLLLLH-------------------


DSSP  eeeeellhHHHHhhhhhhhhhlllllllllllHHHH-----------------hhhhhhh
Query vavpcxtiPWQIaqklhavtavleepkandraHDLV-----------------dlqlleg  216
ident                                  |
Sbjct -------yCGVL----------------yftgSDIFnknmrahalekgftineytirplg  297
DSSP  -------hHHHH----------------hlllLHHHhhhhhhhhhhhleeellllleeell


DSSP  hllhhhhhhhhHHHHHhhhhhlllLLLLLllllllhhhhhhhhhllllllllllllhhhhh
Query llldadlxptrSACIAifearaqhPWPPRvatlphwpliyagalegldhlelartvdaaa  276
ident
Sbjct vtgvageplpvDSEKD-----ifdYIQWK-------------------------------  321
DSSP  lllllllllllLLHHH-----hhhHLLLL-------------------------------


DSSP  hhhhhhhhhhhhllll
```

```
Query qavqrfvaridratkr  292
ident
Sbjct -------yrepkdrse  330
DSSP  -------lllhhhlll
```

No 67: Query=mol1A Sbjct=4ub2A Z-score=7.1

back to top

```
DSSP  ------------------------------------------------------------
Query ------------------------------------------------------------    0
ident
Sbjct lnggitdmltelanfeknvsqaihkynayrkaasviakyphkiksgaeakklpgvgtkia   60
DSSP  llhhhhhhhhhhhhhhhhllllhhhhhhhhhhhhhhhhllllllllhhhhhllllllhhhh


DSSP  -----------------------------------------------llllllllllhhh
Query -----------------------------------------------aapyssspptnlrs   13
ident
Sbjct ekideflatgklrklekirqddtsssinfltrvsgigpsaarkfvdegiktledlrkned  120
DSSP  hhhhhhhhhlllhhhhhhhllhhhhhhhhhlllllllhhhhhhhhhlllllhhhhhhlhh


DSSP  hhhhhhhhhhhllllhhhhhHHHHHHHHHHHHHH-LLLLllLLLLLEEEEHHHHHHHhlll
Query lrdrltqvaerqgvvfgrlqRHVAXIVVAQFAA-TLTDdtGAPLLLVKGGSSLELrrgip   72
ident                                               | |
Sbjct klnhhqriglkyfgdfekriPREEMLQMQDIVLnEVKKvdSEYIATVCGSFRRGA-----  175
DSSP  hllhhhhhhhhlhhhhhlleEHHHHHHHHHHHHHhHHLLllLLLEEEELLHHHHLL-----


DSSP  lllLLLLEEEEEL----------lLHHHHHHHHHHHHhhllllleeEEELLLEElllllll
Query dsrTSKDFATVAR----------rDIELIHEQLADAGetgwegftAIFTAPEEiaapgxp  122
ident     | |                       |||             |
Sbjct --eSSGDMDVLLThpsftsesqpkLLHQVVEQLQKVH--------FITDTLSK------g  219
DSSP  --lEELLEEEEEElllllllllllHHHHHHHHHHHHLL--------LEEEEEEE------l


DSSP  LLLEEEEEEEEELLEEEEEEEEEEEEL-LLLLllllleeeeelhhhhllllllleeeeellh
Query VKPRRFTAKLSYRGRAFATVPIEVSSV-EAGNadqfdtltsdalglvgvpaavavpcxti  181
ident          |           |
Sbjct ETKFMGVCQLPSKNKEYPHRRIDIRLIpKDQY---------------------------y  252
DSSP  LLEEEEEELLLLLLLLLLLEEEEEEEELlHHHH---------------------------h


DSSP  HHHHhhhhhhhhhllllllllllHHHH-----------------hhhhhhhhllhhHHH
Query PWQIaqklhavtavleepkandraHDLV-----------------dlqlleglllldaDLX  224
ident                          |                                |
Sbjct CGVL----------------yftgSDIFnknmrahalekgftineytirplggvagePLP  296
DSSP  HHHH----------------hlllLHHHhhhhhllhhhhleeellllleeellllllLLL


DSSP  HhhHHHHHhhhhhlllLLLLLlllllhhhhhhhhhllllllllllhhhhhhhhhhhh
Query PtrSACIAifearaqhPWPPRvatlphwpliyagalegldhlelartvdaaaqavqrfva  284
ident
Sbjct V--DSEKD-----ifdYIQWK--------------------------------------y  311
DSSP  L--LLHHH-----hhhHLLLL--------------------------------------l


DSSP  hhhhllll
Query ridratkr  292
ident
Sbjct repkdrse  319
DSSP  llhhhlll
```

No 68: Query=mol1A Sbjct=5db8A Z-score=7.1

back to top

```
DSSP  ------------------------------------------------------------
Query ------------------------------------------------------------    0
ident
Sbjct pqetlnggitdmltelanfeknvsqaihkynayrkaasviakyphkiksgaeakklpgvg   60
DSSP  llhhhlhhhhhhhhhhhhhhhhhllllhhhhhhhhhhhhhhhhllllllllhhhhhllllll
```

```
DSSP  ---------------------------------------------------lllllllll
Query ---------------------------------------------------aapyssppt    9
ident
Sbjct tkiaekideflatgklrklekirqddtsssinfltrvsgigpsaarkfvdegiktledlr  120
DSSP  hhhhhhhhhhhhhlllhhhhhhhllhhhhhhhhhlllllllhhhhhhhhhlllllhhhhh


DSSP  lhhhhhhhhhhhhhllllhhhhhHHHHHHHHHHHHH-LLLLllLLLLEEEEHHHHHHHh
Query nlrslrdrltqvaerqgvvfgrlqRHVAXIVVAQFAA-TLTDdtGAPLLLVKGGSSLELr   68
ident                                                   | |
Sbjct knedklnhhqriglkyfgdfekriPREEMLQMQDIVLnEVKKvdSEYIATVCGSFRRGA-  179
DSSP  llhhhllhhhhhhhhlhhhhllleEHHHHHHHHHHHHhHHHHhlLLLEEEELHHHLLLL-


DSSP  hllllllLLLLEEEEEL----------lLHHHHHHHHHHHHHhhllllleeEEELLLEElll
Query rgipdsrTSKDFATVAR----------rDIELIHEQLADAGetgwegftAIFTAPEEiaa  118
ident         | |                       |||             |
Sbjct ------eSSGDMDVLLThpsftsesqpkLLHQVVEQLQKVH--------FITDTLSK---  222
DSSP  ------lEELLEEEEEEllllllllllHHHHHHHHHHHLL--------LEEEEEEE---


DSSP  llllLLLEEEEEEEEEL--LEEEEEEEEEEEEL-LLLLllllleeeeelhhhhllllllee
Query pgxpVKPRRFTAKLSYR--GRAFATVPIEVSSV-EAGNadqfdtltsdalglvgvpaava  175
ident              |             |
Sbjct ---gETKFMGVCQLPSKndEKEYPHRRIDIRLIpKDQY----------------------  257
DSSP  ---lLLEEEEEEELLLLLlLLLLLLLEEEEEEEELlHHHH----------------------


DSSP  eeellhHHHHhhhhhhhhllllllllllHHHH-----------------hhhhhhhhl
Query vpcxtiPWQIaqklhavtavleepkandraHDLV-----------------dlqllegll  218
ident                                |
Sbjct -----yCGVL----------------yftgSDIFnknmrahalekgftineytirplgvt  296
DSSP  -----hHHHH----------------hlllLHHHhhhhhhhhhllleeellllleeellll


DSSP  lhhhhhhhhHHHHHhhhhhlllLLLLLllllllhhhhhhhhhlllllllllllhhhhhhh
Query ldadlxptrSACIAifearaqhPWPPRvatlphwpliyagalegldhlelartvdaaaqa  278
ident
Sbjct gvagepplpvDSEKD-----ifdYIQWK---------------------------------  318
DSSP  lllllllllLLHHH-----hhhHLLLL---------------------------------


DSSP  hhhhhhhhhhllll
Query vqrfvaridratkr  292
ident
Sbjct -----yrepkdrse  327
DSSP  -----lllhhhlll
```

No 69: Query=mol1A Sbjct=5db9A Z-score=7.1

back to top

```
DSSP  ------------------------------------------------------------
Query ------------------------------------------------------------    0
ident
Sbjct pqetlnggitdmltelanfeknvsqaihkynayrkaasviakyphkiksgaeakklpgvg   60
DSSP  llhhhhhhhhhhhhhhhhhhhhhllllhhhhhhhhhhhhhhhhhllllllhhhhhllllll


DSSP  ---------------------------------------------------lllllllll
Query ---------------------------------------------------aapyssppt    9
ident
Sbjct tkiaekideflatgklrklekirqddtsssinfltrvsgigpsaarkfvdegiktledlr  120
DSSP  hhhhhhhhhhhhhlllhhhhhhhhlhhhhhhhhhllllllhhhhhhhhhlllllhhhhh


DSSP  lhhhhhhhhhhhhhllllhhhhhHHHHHHHHHHHHH-LLLLllLLLLEEEEHHHHHHHh
Query nlrslrdrltqvaerqgvvfgrlqRHVAXIVVAQFAA-TLTDdtGAPLLLVKGGSSLELr   68
ident                                                   | |
Sbjct knedklnhhqriglkyfgdfekriPREEMLQMQDIVLnEVKKvdSEYIATVCGSFRRGA-  179
DSSP  llhhhllhhhhhhhhlhhhhllleEHHHHHHHHHHHHhHHHHhlLLLEEEELHHHHHLL-


DSSP  hllllllLLLLEEEEEL----------lLHHHHHHHHHHHHHhhllllleeEEELLLEElll
Query rgipdsrTSKDFATVAR----------rDIELIHEQLADAGetgwegftAIFTAPEEiaa  118
ident         | |                       |||             |
```

```
Sbjct ------eSSGDMDVLLThpsftsesqpkLLHQVVEQLQKVH--------FITDTLSK---  222
DSSP  ------lEELLEEEEEEllllllllllllHHHHHHHHHHHLL--------LEEEEEEE---


DSSP  llllLLLEEEEEEEEELL--EEEEEEEEEEEELLLLLLllleeeeelhhhhllllllleee
Query pgxpVKPRRFTAKLSYRG--RAFATVPIEVSSVEAGNAdqfdtltsdalglvgvpaavav  176
ident              |             |
Sbjct ---gETKFMGVCQLPSKNdeKEYPHRRIDIRLIPKDQY----------------------  257
DSSP  ---lLLEEEEEEELLLLLllLLLLLEEEEEEELLHHHH----------------------


DSSP  eellhHHHHhhhhhhhhllllllllllllHHHH-----------------hhhhhhhhll
Query pcxtiPWQIaqklhavtavleepkandraHDLV-----------------dlqlleglll  219
ident                               |
Sbjct ----yCGVL----------------yftgSDIFnknmrahalekgftineytirplgvtg  297
DSSP  ----hHHHH----------------hhhlLHHHhhhhhhhhhllleeellllleeelllll


DSSP  hhhhhhhhHHHHHhhhhhlllLLLLLllllllhhhhhhhhhlllllllllllhhhhhhhh
Query dadlxptrSACIAifearaqhPWPPRvatlphwpliyagalegldhlelartvdaaaqav  279
ident
Sbjct vageplpvDSEKD-----ifdYIQWK----------------------------------  318
DSSP  llllllllLLHHH-----hhhHLLLL----------------------------------


DSSP  hhhhhhhhhllll
Query qrfvaridratkr  292
ident
Sbjct ----yrepkdrse  327
DSSP  ----lllhhhlll
```

No 70: Query=mol1A Sbjct=4kliA Z-score=7.1

back to top

```
DSSP  ------------------------------------------------------------
Query ------------------------------------------------------------    0
ident
Sbjct tlnggitdmltelanfeknvsqaihkynayrkaasviakyphkiksgaeakklpgvgtki   60
DSSP  lllhhhhhhhhhhhhhhhhllllhhhhhhhhhhhhhhhhllllllllhhhhhhlllllhhh


DSSP  ------------------------------------------------llllllllllhh
Query ------------------------------------------------aapysspptnlr   12
ident
Sbjct aekideflatgklrklekirqddtsssinfltrvsgigpsaarkfvdegiktledlrkne  120
DSSP  hhhhhhhhhlllhhhhhhhhlhhhhhhhhhllllllhhhhhhhhhlllllhhhhhhlh


DSSP  hhhhhhhhhhhhllllhhhhhHHHHHHHHHHHHH-LLLLllLLLLEEEEHHHHHHHhhll
Query slrdrltqvaerqgvvfgrlqRHVAXIVVAQFAA-TLTDdtGAPLLLVKGGSSLELrrgi   71
ident                                                | |
Sbjct dklnhhqriglkyfgdfekriPREEMLQMQDIVLnEVKKvdSEYIATVCGSFRRGA----  176
DSSP  hhllhhhhhhhhlhhhhllleEHHHHHHHHHHHHhHHHHhlLLLEEEELHHHHLLL----


DSSP  llllLLLLEEEEEL------------lLHHHHHHHHHHHHhhllllleeEEELLLEELlll
Query pdsrTSKDFATVAR------------rDIELIHEQLADAGetgwegftAIFTAPEEIaap  119
ident      | |                         |||             |
Sbjct ---eSSGDMDVLLThpsftsestkqpkLLHQVVEQLQKVH--------FITDTLSKG---  222
DSSP  ---lEELLEEEEEElllllllllllllHHHHHHHHHHHLL--------LEEEEEEEL---


DSSP  lllLLLEEEEEEEEEL--LEEEEEEEEEEEEL-LLLLllllleeeeelhhhhllllllleee
Query gxpVKPRRFTAKLSYR--GRAFATVPIEVSSV-EAGNadqfdtltsdalglvgvpaavav  176
ident             |             |
Sbjct ---ETKFMGVCQLPSKndEKEYPHRRIDIRLIpKDQY-----------------------  256
DSSP  ---LLEEEEEEELLLLllLLLLLLEEEEEEELlHHHH-----------------------


DSSP  eellhHHHHhhhhhhhhllllllllllllHHHHHHH-------------------hhhhh
Query pcxtiPWQIaqklhavtavleepkandraHDLVDLQ-------------------llegl  217
ident                               |
Sbjct ----yCGVL----------------yftgSDIFNKNmrahalekgftineytirplgvtg  296
DSSP  ----hHHHH----------------hhhlLHHHHHHhhhhhhhhleeellllleeelllll
```

```
DSSP  llhHHHHhhHHHHHhhhhhhllllLLLLLlllllhhhhhhhhhllllllllllhhhhhh
Query lldADLXptRSACIaifearaqhpWPPRVatlphwpliyagalegldhlelartvdaaaq  277
ident      |
Sbjct vagEPLP--VDSEK-------difDYIQW-------------------------------  316
DSSP  lllLLLL--LLLHH-------hhhHHLLL-------------------------------


DSSP  hhhhhhhhhhhllll
Query avqrfvaridratkr  292
ident
Sbjct -----kyrepkdrse  326
DSSP  -----llllhhhlll
```

No 71: Query=mol1A Sbjct=4kloA Z-score=7.1

back to top

```
DSSP  ------------------------------------------------------------
Query ------------------------------------------------------------    0
ident
Sbjct tlnggitdmltelanfeknvsqaihkynayrkaasviakyphkiksgaeakklpgvgtki   60
DSSP  lllhhhhhhhhhhhhhhhhllllhhhhhhhhhhhhhhhhlllllllhhhhhlllllhhhh


DSSP  ------------------------------------------------llllllllllhh
Query ------------------------------------------------aapysspptnlr   12
ident
Sbjct aekideflatgklrklekirqddtsssinfltrvsgigpsaarkfvdegiktledlrkne  120
DSSP  hhhhhhhhhhlllhhhhhhhhlhhhhhhhhhlllllllhhhhhhhhhlllllhhhhhhlh


DSSP  hhhhhhhhhhhhllllhhhhhHHHHHHHHHHHHHH-LLLLllLLLLEEEEHHHHHHHhhll
Query slrdrltqvaerqgvvfgrlqRHVAXIVVAQFAA-TLTDdtGAPLLLVKGGSSLELrrgi   71
ident                                                |   |
Sbjct dklnhhqriglkyfgdfekriPREEMLQMQDIVLnEVKKvdSEYIATVCGSFRRGA----  176
DSSP  hhllhhhhhhhhlhhhhllleEHHHHHHHHHHHHHhHHHHhlLLLEEEELHHHHLLL----


DSSP  llllLLLLEEEEEL------------lLHHHHHHHHHHHHHhhllllleeEEELLLEELlll
Query pdsrTSKDFATVAR------------rDIELIHEQLADAGetgwegftAIFTAPEEIaap  119
ident      | |                         |||             |
Sbjct ---eSSGDMDVLLThpsftsestkqpkLLHQVVEQLQKVH--------FITDTLSKG---  222
DSSP  ---lEELLEEEEEEllllllllllllHHHHHHHHHHHLL--------LEEEEEEEL---


DSSP  lllLLLEEEEEEEEEL--LEEEEEEEEEEEEL-LLLLlllleeeeelhhhhllllllleee
Query gxpVKPRRFTAKLSYR--GRAFATVPIEVSSV-EAGNadqfdtltsdalglvgvpaavav  176
ident             |             |
Sbjct ---ETKFMGVCQLPSKndEKEYPHRRIDIRLIpKDQY-----------------------  256
DSSP  ---LLEEEEEEELLLLLllLLLLLLLEEEEEEEELlHHHH-----------------------


DSSP  eellhHHHHhhhhhhhhlllllllllllHHHHHHH-------------------hhhhh
Query pcxtiPWQIaqklhavtavleepkandraHDLVDLQ-------------------llegl  217
ident                               |
Sbjct ----yCGVL----------------yftgSDIFNKNmrahalekgftineytirplgvtg  296
DSSP  ----hHHHH----------------hhhlLHHHHHHhhhhhhhlleeellllleeellll


DSSP  llhHHHHhhHHHHHhhhhhhllllLLLLLlllllhhhhhhhhhllllllllllhhhhhh
Query lldADLXptRSACIaifearaqhpWPPRVatlphwpliyagalegldhlelartvdaaaq  277
ident      |
Sbjct vagEPLP--VDSEK-------difDYIQW-------------------------------  316
DSSP  lllLLLL--LLLHH-------hhhHHLLL-------------------------------


DSSP  hhhhhhhhhhhllll
Query avqrfvaridratkr  292
ident
Sbjct -----kyrepkdrse  326
DSSP  -----llllhhhlll
```

No 72: Query=mol1A Sbjct=4klfA Z-score=7.1

back to top

```
DSSP  ------------------------------------------------------------
Query ------------------------------------------------------------   0
ident 
Sbjct tlnggitdmltelanfeknvsqaihkynayrkaasviakyphkiksgaeakklpgvgtki  60
DSSP  lllhhhhhhhhhhhhhhhhhllllhhhhhhhhhhhhhhhhlllllllhhhhhlllllhhh


DSSP  ------------------------------------------------llllllllllhh
Query ------------------------------------------------aapysspptnlr  12
ident 
Sbjct aekideflatgklrklekirqddtsssinfltrvsgigpsaarkfvdegiktledlrkne 120
DSSP  hhhhhhhhhhlllhhhhhhhhhlhhhhhhhhhllllllhhhhhhhhhlllllhhhhhhlh


DSSP  hhhhhhhhhhhhllllhhhhhHHHHHHHHHHHHHH-LLLLllLLLLEEEEHHHHHHHhhll
Query slrdrltqvaerqgvvfgrlqRHVAXIVVAQFAA-TLTDdtGAPLLLVKGGSSLELrrgi  71
ident                                                | |
Sbjct dklnhhqriglkyfgdfekriPREEMLQMQDIVLnEVKKvdSEYIATVCGSFRRGA---- 176
DSSP  hhllhhhhhhhhlhhhhhlleEHHHHHHHHHHHHhHHHHhlLLLEEEELHHHLLLL----


DSSP  llllLLLLEEEEEL------------lLHHHHHHHHHHHHhhlllleeEEELLLEELlll
Query pdsrTSKDFATVAR------------rDIELIHEQLADAGetgwegftAIFTAPEEIaap 119
ident      | |                         |||             |
Sbjct ---eSSGDMDVLLThpsftsestkqpkLLHQVVEQLQKVH--------FITDTLSKG--- 222
DSSP  ---lEELLEEEEEEllllllllllllHHHHHHHHHHHLL--------LEEEEEEEL---


DSSP  lllLLLEEEEEEEEEL--LEEEEEEEEEEEEL-LLLLllllleeeeelhhhhlllllleee
Query gxpVKPRRFTAKLSYR--GRAFATVPIEVSSV-EAGNadqfdtltsdalglvgvpaavav 176
ident             |             |
Sbjct ---ETKFMGVCQLPSKndEKEYPHRRIDIRLIpKDQY----------------------- 256
DSSP  ---LLEEEEEEELLLLllLLLLLLEEEEEEELlHHHH-----------------------


DSSP  eellhHHHHhhhhhhhhllllllllllllHHHHHHHH-------------------hhhh
Query pcxtiPWQIaqklhavtavleepkandraHDLVDLQL-------------------legl 217
ident                               |
Sbjct ----yCGVL----------------yftgSDIFNKNMrahalekgftineytirplgvtg 296
DSSP  ----hHHHH----------------hhhlLHHHHHHHhhhhhhhleeellllleeellll


DSSP  llhHHHHhhHHHHHhhhhhhllllLLLLLlllllhhhhhhhhhlllllllllllhhhhhh
Query lldADLXptRSACIaifearaqhpWPPRVatlphwpliyagalegldhlelartvdaaaq 277
ident      |
Sbjct vagEPLP--VDSEK-------difDYIQW------------------------------- 316
DSSP  lllLLLL--LLLHH-------hhhHHLLL-------------------------------


DSSP  hhhhhhhhhhhllll
Query avqrfvaridratkr  292
ident 
Sbjct -----kyrepkdrse  326
DSSP  -----llllhhhlll
```

No 73: Query=mol1A Sbjct=4kljA Z-score=7.1

back to top

```
DSSP  ------------------------------------------------------------
Query ------------------------------------------------------------   0
ident 
Sbjct tlnggitdmltelanfeknvsqaihkynayrkaasviakyphkiksgaeakklpgvgtki  60
DSSP  lllhhhhhhhhhhhhhhhhhllllhhhhhhhhhhhhhhhhlllllllhhhhhlllllhhh


DSSP  ------------------------------------------------llllllllllhh
Query ------------------------------------------------aapysspptnlr  12
ident 
Sbjct aekideflatgklrklekirqddtsssinfltrvsgigpsaarkfvdegiktledlrkne 120
DSSP  hhhhhhhhhhlllhhhhhhhhhlhhhhhhhhhllllllhhhhhhhhhlllllhhhhhhlh


DSSP  hhhhhhhhhhhhllllhhhhhHHHHHHHHHHHHHH-LLLLllLLLLEEEEHHHHHHHhhll
Query slrdrltqvaerqgvvfgrlqRHVAXIVVAQFAA-TLTDdtGAPLLLVKGGSSLELrrgi  71
```

```
ident                                                  | |
Sbjct dklnhhqriglkyfgdfekriPREEMLQMQDIVLnEVKKvdSEYIATVCGSFRRGA----  176
DSSP  hhllhhhhhhhhlhhhhllleEHHHHHHHHHHHHhHHHHhlLLLEEEEEHHHLLLL----


DSSP  llllLLLLEEEEEL------------lLHHHHHHHHHHHHhhllllee EEELLLEELlll
Query pdsrTSKDFATVAR------------rDIELIHEQLADAGetgwegftAIFTAPEEIaap  119
ident      | |                        |||             |
Sbjct ---eSSGDMDVLLThpsftsestkqpkLLHQVVEQLQKVH--------FITDTLSKG---  222
DSSP  ---lEELLEEEEEEllllllllllllHHHHHHHHHHHHLL--------LEEEEEEEL---


DSSP  lllLLLEEEEEEEEEL--LEEEEEEEEEEEEL-LLLLlllleeeeelhhhhllllllleee
Query gxpVKPRRFTAKLSYR--GRAFATVPIEVSSV-EAGNadqfdtltsdalglvgvpaavav  176
ident             |             |
Sbjct ---ETKFMGVCQLPSKndEKEYPHRRIDIRLIpKDQY-----------------------  256
DSSP  ---LLEEEEEEELLLLLllLLLLLLLEEEEEEEELlHHHH-----------------------


DSSP  eellhHHHHhhhhhhhhllllllllllllHHHHHHH-------------------hhhhh
Query pcxtiPWQIaqklhavtavleepkandraHDLVDLQ-------------------llegl  217
ident                               |
Sbjct ----yCGVL----------------yftgSDIFNKNmrahalekgftineytirplgvtg  296
DSSP  ----hHHHH----------------hhhlLHHHHHHhhhhhhhlleeellllleeelllll


DSSP  llhHHHHhhHHHHHhhhhhhllllLLLLLlllllhhhhhhhhhllllllllllhhhhhh
Query lldADLXptRSACIaifearaqhpWPPRVatlphwpliyagalegldhlelartvdaaaq  277
ident      |
Sbjct vagEPLP--VDSEK-------difDYIQW-------------------------------  316
DSSP  lllLLLL--LLLHH-------hhhHHLLL-------------------------------


DSSP  hhhhhhhhhhhllll
Query avqrfvaridratkr  292
ident
Sbjct -----kyrepkdrse  326
DSSP  -----llllhhhlll
```

No 74: Query=mol1A Sbjct=4gxkA Z-score=7.1

back to top

```
DSSP  ------------------------------------------------------------
Query ------------------------------------------------------------    0
ident
Sbjct tlnggitdmltelanfeknvsqaihkynayrkaasviakyphkiksgaeakklpgvgtki   60
DSSP  lllhhhhhhhhhhhhhhhhllllhhhhhhhhhhhhhhhllllllllhhhhhlllllhhh


DSSP  ------------------------------------------------llllllllllhh
Query ------------------------------------------------aapysspptnlr   12
ident
Sbjct aekideflatgklrklekirqddtsssinfltrvsgigpsaarkfvdegiktledlrkne  120
DSSP  hhhhhhhhhhlllhhhhhhhhlhhhhhhhhhlllllllhhhhhhhhhlllllhhhhhhlh


DSSP  hhhhhhhhhhhllllhhhhhHHHHHHHHHHHHHH-LLLLllLLLLEEEEHHHHHHHhhll
Query slrdrltqvaerqgvvfgrlqRHVAXIVVAQFAA-TLTDdtGAPLLLVKGGSSLELrrgi   71
ident                                                  | |
Sbjct dklnhhqriglkyfgdfekriPREEMLQMQDIVLnEVKKvdSEYIATVCGSFRRGA----  176
DSSP  hhllhhhhhhhhlhhhhhlleEHHHHHHHHHHHHhHHHHhlLLLEEEEEELLLLLL----


DSSP  llllLLLLEEEEEL----------lLHHHHHHHHHHHHhhllllee EEELLLEELlllll
Query pdsrTSKDFATVAR----------rDIELIHEQLADAGetgwegftAIFTAPEEIaapgx  121
ident      | |                      |||             |
Sbjct ---eSSGDMDVLLThpsftsesqpkLLHQVVEQLQKVH--------FITDTLSKG-----  220
DSSP  ---lEELLEEEEEElllllllllllHHHHHHHHHHHHLL--------LEEEEEEEL-----


DSSP  lLLLEEEEEEEEEL-LEEEEEEEEEEEEL-LLLLlllleeeeelhhhhlllllleeeeel
Query pVKPRRFTAKLSYR-GRAFATVPIEVSSV-EAGNadqfdtltsdalglvgvpaavavpcx  179
ident           |             |
Sbjct -ETKFMGVCQLPSKdEKEYPHRRIDIRLIpKDQY--------------------------  253
DSSP  -LLEEEEEEELLLLLlLLLLLLLEEEEEEEELlHHHH--------------------------
```

```
DSSP  lhHHHHhhhhhhhhllllllllllllHHHHHHH------------hhhhhllhHHHHhhH
Query tiPWQIaqklhavtavleepkandraHDLVDLQ------------llegllldADLXptR  227
ident                            |                           |
Sbjct -yCGVL----------------yftgSDIFNKNmkahalekgftineytirpgEPLP--V  294
DSSP  -hHHHH----------------hhhlLHHHHHHhhhhhhlllleellllleellLLLL--L


DSSP  HHHHhhhhhhllllLLLLLlllllhhhhhhhhhlllllllllllhhhhhhhhhhhhhhh
Query SACIaifearaqhpWPPRVatlphwpliyagalegldhlelartvdaaaqavqrfvarid  287
ident
Sbjct DSEK-------difDYIQW------------------------------------kyrep  311
DSSP  LLHH-------hhhHHLLL------------------------------------llllh


DSSP  hllll
Query ratkr  292
ident
Sbjct kdrse  316
DSSP  hhhll
```

No 75: Query=mol1A Sbjct=4klmA Z-score=7.1

back to top

```
DSSP  ------------------------------------------------------------
Query ------------------------------------------------------------    0
ident
Sbjct tlnggitdmltelanfeknvsqaihkynayrkaasviakyphkiksgaeakklpgvgtki   60
DSSP  lllhhhhhhhhhhhhhhhhlllllhhhhhhhhhhhhhhhhlllllllhhhhhlllllhhh


DSSP  ------------------------------------------------llllllllllhh
Query ------------------------------------------------aapysspptnlr   12
ident
Sbjct aekideflatgklrklekirqddtsssinfltrvsgigpsaarkfvdegiktledlrkne  120
DSSP  hhhhhhhhhhlllhhhhhhhhlhhhhhhhhhllllllhhhhhhhhhhllllhhhhhhlh


DSSP  hhhhhhhhhhhhllllhhhhhHHHHHHHHHHHHH-LLLLllLLLLEEEEHHHHHHHhhll
Query slrdrltqvaerqgvvfgrlqRHVAXIVVAQFAA-TLTDdtGAPLLLVKGGSSLELrrgi   71
ident                                                  | |
Sbjct dklnhhqriglkyfgdfekriPREEMLQMQDIVLnEVKKvdSEYIATVCGSFRRGA----  176
DSSP  hhllhhhhhhhhlhhhhllleEHHHHHHHHHHHHhHHHHhlLLLEEEEEHHHLLLL----


DSSP  llllLLLLEEEEEL------------lLHHHHHHHHHHHHhhlllleeEEELLLEELlll
Query pdsrTSKDFATVAR------------rDIELIHEQLADAGetgwegftAIFTAPEEIaap  119
ident      | |                         |||             |
Sbjct ---eSSGDMDVLLThpsftsestkqpkLLHQVVEQLQKVH--------FITDTLSKG---  222
DSSP  ---lEELLEEEEEEllllllllllllHHHHHHHHHHHHLL--------LEEEEEEEL---


DSSP  lllLLLEEEEEEEEEL--LEEEEEEEEEEEL-LLLLlllleeeeelhhhhllllllleee
Query gxpVKPRRFTAKLSYR--GRAFATVPIEVSSV-EAGNadqfdtltsdalglvgvpaavav  176
ident             |             |
Sbjct ---ETKFMGVCQLPSKndEKEYPHRRIDIRLIpKDQY-----------------------  256
DSSP  ---LLEEEEEELLLLLllLLLLLLEEEEEEEELlHHHH-----------------------


DSSP  eellhHHHHhhhhhhhhllllllllllllHHHHHHH-------------------hhhhh
Query pcxtiPWQIaqklhavtavleepkandraHDLVDLQ-------------------llegl  217
ident                               |
Sbjct ----yCGVL----------------yftgSDIFNKNmrahalekgftineytirplgvtg  296
DSSP  ----hHHHH----------------hhhlLHHHHHHhhhhhhhlleeellllleeelllll


DSSP  llhHHHHhhHHHHHhhhhhhllllLLLLLlllllhhhhhhhhhlllllllllllhhhhhh
Query lldADLXptRSACIaifearaqhpWPPRVatlphwpliyagalegldhlelartvdaaaq  277
ident      |
Sbjct vagEPLP--VDSEK-------difDYIQW-------------------------------  316
DSSP  lllLLLL--LLLHH-------hhhHHLLL-------------------------------


DSSP  hhhhhhhhhhhllll
Query avqrfvaridratkr  292
ident
```

```
Sbjct -----kyrepkdrse  326
DSSP  -----llllhhhlll
```

No 76: Query=mol1A Sbjct=4klgA Z-score=7.1

back to top

```
DSSP  ------------------------------------------------------------
Query ------------------------------------------------------------    0
ident
Sbjct tlnggitdmltelanfeknvsqaihkynayrkaasviakyphkiksgaeakklpgvgtki   60
DSSP  lllhhhhhhhhhhhhhhhhllllhhhhhhhhhhhhhhhhlllllllhhhhhlllllllhhh


DSSP  ------------------------------------------------llllllllllhh
Query ------------------------------------------------aapysspptnlr   12
ident
Sbjct aekideflatgklrklekirqddtsssinfltrvsgigpsaarkfvdegiktledlrkne  120
DSSP  hhhhhhhhhlllhhhhhhhhlhhhhhhhhhllllllhhhhhhhhhhllllhhhhhhlh


DSSP  hhhhhhhhhhhllllhhhhhHHHHHHHHHHHHH-LLLLllLLLLEEEEHHHHHHHhhll
Query slrdrltqvaerqgvvfgrlqRHVAXIVVAQFAA-TLTDdtGAPLLLVKGGSSLELrrgi   71
ident                                                | |
Sbjct dklnhhqriglkyfgdfekriPREEMLQMQDIVLnEVKKvdSEYIATVCGSFRRGA----  176
DSSP  hhllhhhhhhhhlhhhhllleEHHHHHHHHHHHHhHHHHhlLLLEEEEEHHHLLLL----


DSSP  llllLLLLEEEEEL------------lLHHHHHHHHHHHHhhlllleeEEELLLEELlll
Query pdsrTSKDFATVAR------------rDIELIHEQLADAGetgwegftAIFTAPEEIaap  119
ident      | |                         |||             |
Sbjct ---eSSGDMDVLLThpsftsestkqpkLLHQVVEQLQKVH--------FITDTLSKG---  222
DSSP  ---lEELLEEEEEEllllllllllllHHHHHHHHHHHLL--------LEEEEEEEL---


DSSP  lllLLLEEEEEEEEEL--LEEEEEEEEEEEEL-LLLLlllleeeeelhhhhllllllleee
Query gxpVKPRRFTAKLSYR--GRAFATVPIEVSSV-EAGNadqfdtltsdalglvgvpaavav  176
ident             |             |
Sbjct ---ETKFMGVCQLPSKndEKEYPHRRIDIRLIpKDQY-----------------------  256
DSSP  ---LLEEEEEELLLLLllLLLLLLEEEEEEELlHHHH-----------------------


DSSP  eellhHHHHhhhhhhhhllllllllllllHHHHHHH-------------------hhhhh
Query pcxtiPWQIaqklhavtavleepkandraHDLVDLQ-------------------llegl  217
ident                               |
Sbjct ----yCGVL----------------yftgSDIFNKNmrahalekgftineytirplgvtg  296
DSSP  ----hHHHH----------------hhhlLHHHHHHhhhhhhhlleeellllleeellll


DSSP  llhHHHHhhHHHHHhhhhhhllllLLLLLlllllhhhhhhhhhllllllllllllhhhhhh
Query lldADLXptRSACIaifearaqhpWPPRVatlphwpliyagalegldhlelartvdaaaq  277
ident      |
Sbjct vagEPLP--VDSEK-------difDYIQW-------------------------------  316
DSSP  lllLLLL--LLLHH-------hhhHHLLL-------------------------------


DSSP  hhhhhhhhhhhllll
Query avqrfvaridratkr  292
ident
Sbjct -----kyrepkdrse  326
DSSP  -----llllhhhlll
```

No 77: Query=mol1A Sbjct=3v7jA Z-score=7.1

back to top

```
DSSP  ------------------------------------------------------------
Query ------------------------------------------------------------    0
ident
Sbjct etlnggitdmlvelanfeknvsqaihkynayrkaasviakyphkiksgaeakklpgvgtk   60
DSSP  llllhhhhhhhhhhhhhhhhllllhhhhhhhhhhhhhhhhllllllllhhhhhllllllhh


DSSP  -------------------------------------------------llllllllllh
```

```
Query -------------------------------------------------aapysspptnl   11
ident
Sbjct iaekideflatgklrklekirqddtsssinfltrvtgigpsaarklvdegiktledlrkn  120
DSSP  hhhhhhhhhhllllhhhhhhhhlhhhhhhhhhhllllllhhhhhhhhhlllllhhhhhll


DSSP  hhhhhhhhhhhhhllllhHHHHHHHHHHHHHHHHH-LLLLllLLLLEEEEHHHHHHHhhl
Query rslrdrltqvaerqgvvfGRLQRHVAXIVVAQFAA-TLTDdtGAPLLLVKGGSSLELrrg   70
ident                                                 | |
Sbjct edklnhhqriglkyfedfEKRIPREEMLQMQDIVLnEVKKldPEYIATVCGSFRRGA---  177
DSSP  hhhllhhhhhhhhlhhhhHLLEEHHHHHHHHHHHHhHHHHhlLLLEEEELHHHHLLL---


DSSP  lllllLLLLEEEEEL------------lLHHHHHHHHHHHHhhlllleeEEELLLEELll
Query ipdsrTSKDFATVAR------------rDIELIHEQLADAGetgwegftAIFTAPEEIaa  118
ident       | |                         |||             |
Sbjct ----eSSGDMDVLLThpnftsesskqpkLLHRVVEQLQKVR--------FITDTLSKG--  223
DSSP  ----lEELLEEEEEElllllllllllllhHHHHHHHHHHLL--------LEEEEEEEL--


DSSP  llllLLLEEEEEEEEELL--EEEEEEEEEEEEL-LLLLlllleeeeelhhhhllllllee
Query pgxpVKPRRFTAKLSYRG--RAFATVPIEVSSV-EAGNadqfdtltsdalglvgvpaava  175
ident              |             |
Sbjct ----ETKFMGVCQLPSENdeNEYPHRRIDIRLIpKDQY----------------------  257
DSSP  ----LLEEEEEELLLLLLllLLLLLEEEEEEELlHHHH----------------------


DSSP  eeellhHHHhhhhhhhhhhlllllllllllhhhhHHHHHH--------------------
Query vpcxtiPWQIaqklhavtavleepkandrahdlvDLQLLE--------------------  215
ident
Sbjct -----yCGVL--------------------yftgSDIFNKnmrahalekgftineytirp  292
DSSP  -----hHHHH--------------------hlllLHHHHHhhhhhhhhhleeellllleee


DSSP  --hhllhhhhhhhhHHHHhhhhhhllllLLLLLlllllhhhhhhhhhlllllllllllhh
Query --gllldadlxptrSACIaifearaqhpWPPRVatlphwpliyagalegldhlelartvd  273
ident               |
Sbjct lgvtgvageplpvdSEQD-------ifdYIQWR---------------------------  318
DSSP  llllllllllllllLHHH-------hhhHLLLL---------------------------


DSSP  hhhhhhhhhhhhhhhllll
Query aaaqavqrfvaridratkr  292
ident
Sbjct ----------yrepkdrse  327
DSSP  ----------lllhhhlll
```

No 78: Query=mol1A Sbjct=3v7kA Z-score=7.1

back to top

```
DSSP  ------------------------------------------------------------
Query ------------------------------------------------------------    0
ident
Sbjct kapqetlnggitdmlvelanfeknvsqaihkynayrkaasviakyphkiksgaeakklpg   60
DSSP  llllllllhhhhhhhhhhhhhhhllllhhhhhhhhhhhhhhhhhlllllllhhhhhllll


DSSP  -----------------------------------------------------lllllll
Query -----------------------------------------------------aapyssp    7
ident
Sbjct vgtkiaeeideflatgklrklekirqddtsssinfltrvtgigpsaarklvdegiktled  120
DSSP  llhhhhhhhhhhhhllllhhhhhhhhlhhhhhhhhhhllllllhhhhhhhhhlllllhhh


DSSP  lllhhhhhhhhhhhhhhllllhHHHHHHHHHHHHHHHHH-LLLLllLLLLEEEEHHHHHH
Query ptnlrslrdrltqvaerqgvvfGRLQRHVAXIVVAQFAA-TLTDdtGAPLLLVKGGSSLE   66
ident                                                     | |
Sbjct lrknedklnhhqriglkyfedfEKRIPREEMLQMQDIVLnEVKKldPEYIATVCGSFRRG  180
DSSP  hhllhhhllhhhhhhhhlllhhHLLEEHHHHHHHHHHHHhHHHHhlLLLEEEELHHHHLL


DSSP  HhhllllllLLLLEEEEEL------------lLHHHHHHHHHHHHhhlllleeEEELLLE
Query LrrgipdsrTSKDFATVAR------------rDIELIHEQLADAGetgwegftAIFTAPE  114
ident           | |                         |||             |
Sbjct A-------eSSGDMDVLLThpnftsesskqpkLLHRVVEQLQKVR--------FITDTLS  225
DSSP  L-------lEELLEEEEEElllllllllllllhHHHHHHHHHHLL--------LEEEEEE
```

```
DSSP  ELllllllLLLEEEEEEEEEELL--EEEEEEEEEEEEL-LLLLlllleeeeelhhhhllll
Query EIaapgxpVKPRRFTAKLSYRG--RAFATVPIEVSSV-EAGNadqfdtltsdalglvgvp  171
ident                  |             |
Sbjct KG------ETKFMGVCQLPSENdeNEYPHRRIDIRLIpKDQY------------------  261
DSSP  EL------LLEEEEEELLLLLLlLLLLLLEEEEEEELlHHHH------------------


DSSP  lleeeeellhHHHHhhhhhhhhllllllllllhhhhHHHHHH----------------
Query aavavpcxtiPWQIaqklhavtavleepkandrahdlvDLQLLE----------------  215
ident
Sbjct ---------yCGVL--------------------yftgSDIFNKnmrahalekgftiney  292
DSSP  ---------hHHHH--------------------hhhlLHHHHHhhhhhhhhhleeelll


DSSP  ------hhllhhhhhhhhHHHHhhhhhhllllLLLLLlllllhhhhhhhhhllllllll
Query ------glllddadlxptrSACIaifearaqhpWPPRVatlphwpliyagalegldhlela  269
ident                   |
Sbjct tirplgvtgvageplpvdSEQD-------ifdYIQWR-----------------------  322
DSSP  leeellllllllllllLHHH-------hhhHLLLL-----------------------


DSSP  llhhhhhhhhhhhhhhhhllll
Query rtvdaaaqavqrfvaridratkr  292
ident
Sbjct --------------yrepkdrse  331
DSSP  --------------lllhhhlll
```

No 79: Query=mol1A Sbjct=3oguA Z-score=7.1

back to top

```
DSSP  ------------------------------------------------------------
Query ------------------------------------------------------------    0
ident
Sbjct tlnggitdmltelanfeknvsqaihkynayrkaasviakyphkiksgaeakklpgvgtki   60
DSSP  lllhhhhhhhhhhhhhhhhllllhhhhhhhhhhhhhhhhlllllllhhhhhlllllhhh


DSSP  ------------------------------------------------llllllllllhh
Query ------------------------------------------------aapysspptnlr   12
ident
Sbjct aekideflatgklrklekirqddtsssinlltrvsgigpsaarkfvdegiktledlrkne  120
DSSP  hhhhhhhhhhlllhhhhhhhhlhhhhhhhhhlllllllhhhhhhhhhlllllhhhhhhlh


DSSP  hhhhhhhhhhhhllllhhhhhHHHHHHHHHHHHH-LLLLllLLLLEEEEHHHHHHHHhhll
Query slrdrltqvaerqgvvfgrlqRHVAXIVVAQFAA-TLTDdtGAPLLLVKGGSSLELrrgi   71
ident                                                 | |
Sbjct dklnhhqriglkyfgdfekriPREEMLQMQDIVLnEVKKvdSEYIATVCGSFRRGA----  176
DSSP  hhllhhhhhhhhlhhhhhlleEHHHHHHHHHHHHhHHHHHhlLLLEEEEELLHHHHLL----


DSSP  llllLLLLEEEEEEL------------lLHHHHHHHHHHHHHhhlllleeEEELLLEELlll
Query pdsrTSKDFATVAR------------rDIELIHEQLADAGetgwegftAIFTAPEEIaap  119
ident       | |                         |||             |
Sbjct ---eSSGDMDVLLThpsftsestkqpkLLHQVVEQLQKVH--------FITDTLSKG---  222
DSSP  ---lEELLEEEEEEllllllllllhhHHHHHHHHHHHHLL--------LEEEEEEEL---


DSSP  lllLLLEEEEEEEEEL--LEEEEEEEEEEEEL-LLLLlllleeeeelhhhhllllllleee
Query gxpVKPRRFTAKLSYR--GRAFATVPIEVSSV-EAGNadqfdtltsdalglvgvpaavav  176
ident             |             |
Sbjct ---KTKFMGVCQLPSKndEKEYPHRRIDIRLIpKDQY-----------------------  256
DSSP  ---LLEEEEEEELLLLLllLLLLLLLEEEEEEELlHHHH-----------------------


DSSP  eellhHHHHhhhhhhhhlllllllllllhhhhHHHHHH---------------------
Query pcxtiPWQIaqklhavtavleepkandrahdlvDLQLLE---------------------  215
ident
Sbjct ----yCGML--------------------yftgSDIFNKnmrahalekgftineytirpl  292
DSSP  ----hHHHH--------------------hhhlLHHHHHhhhhhhhhhlleeellllleeel


DSSP  -hhllhhhhhhhhHHHHhhhhhhllllLLLLLlllllhhhhhhhhhlllllllllllhhh
Query -glllddadlxptrSACIaifearaqhpWPPRVatlphwpliyagalegldhlelartvda  274
```

```
ident              |
Sbjct gvtgvageplpvdSEKD-------ifdYIQWK----------------------------  317
DSSP  lllllllllllllLHHH-------hhhHLLLL----------------------------
```

```
DSSP  hhhhhhhhhhhhhhllll
Query aaqavqrfvaridratkr  292
ident
Sbjct ---------yrepkdrse  326
DSSP  ---------lllhhhlll
```

No 80: Query=mol1A Sbjct=3rjeA Z-score=7.1

back to top

```
DSSP  ------------------------------------------------------------
Query ------------------------------------------------------------    0
ident
Sbjct pqtlnggitdmltelanfeknvsqaihkynayrkaasviakyphkiksgaeakklpgvgt   60
DSSP  llllhhhhhhhhhhhhhhhhllllhhhhhhhhhhhhhhhhllllllllhhhhhllllllh
```

```
DSSP  --------------------------------------------------llllllllll
Query --------------------------------------------------aapysspptn   10
ident
Sbjct kiaekideflatgklrklekirqddtsssinfltrvsgigpsaarkfvdegiktledlrk  120
DSSP  hhhhhhhhhhhhlllhhhhhhhhlhhhhhhhhhllllllhhhhhhhhhhlllllhhhhhl
```

```
DSSP  hhhhhhhhhhhhhhllllhhhhhHHHHHHHHHHHHH-LLLLllLLLLEEEEHHHHHHHhh
Query lrslrdrltqvaerqgvvfgrlqRHVAXIVVAQFAA-TLTDdtGAPLLLVKGGSSLELrr   69
ident                                                  | |
Sbjct nedklnhhqriglkyfgdfekriPREEMLQMQDIVLnEVKKvdSEYIATVCGSFRRGA--  178
DSSP  lhhhllhhhhhhhhlhhhhllleEHHHHHHHHHHHHhHHHHhlLLLEEEELLHHHHLL--
```

```
DSSP  llllllLLLLEEEEEL------------lLHHHHHHHHHHHHhhlllleeEEELLLEELl
Query gipdsrTSKDFATVAR------------rDIELIHEQLADAGetgwegftAIFTAPEEIa  117
ident        | |                         |||             |
Sbjct -----eSSGDMDVLLThpsftsestkqpkLLHQVVEQLQKVH--------FITDTLSKG-  224
DSSP  -----lEELLEEEEEEllllllllllllHHHHHHHHHHHLL--------LEEEEEEEL-
```

```
DSSP  lllllLLLEEEEEEEEEL--LEEEEEEEEEEEELLLLLLllleeeeelhhhhllllllee
Query apgxpVKPRRFTAKLSYR--GRAFATVPIEVSSVEAGNAdqfdtltsdalglvgvpaava  175
ident               |             |
Sbjct -----ETKFMGVCQLPSKndEKEYPHRRIDIRLIPKDQY---------------------  258
DSSP  -----LLEEEEEEELLLLllLLLLLLEEEEEEELLHHHH---------------------
```

```
DSSP  eeellhHHHHhhhhhhhhllllllllllllhhhhHHHHHH--------------------
Query vpcxtiPWQIaqklhavtavleepkandrahdlvDLQLLE--------------------  215
ident
Sbjct -----yCGVL--------------------yftgSDIFNKnmrahalekgftineytirp  293
DSSP  -----hHHHH--------------------hhhlLHHHHHhhhhhhhhlleeellllleee
```

```
DSSP  --hhllhhhhhhhhHHHHhhhhhhlllLLLLLLllllhhhhhhhhhllllllllllllhh
Query --gllldadlxptrSACIaifearaqhPWPPRVatlphwpliyagalegldhlelartvd  273
ident               |
Sbjct lgvtgvageplpvdSEKD-------ifDYIQWK---------------------------  319
DSSP  llllllllllllllLHHH-------hhHHLLLL---------------------------
```

```
DSSP  hhhhhhhhhhhhhhhllll
Query aaaqavqrfvaridratkr  292
ident
Sbjct ----------yrepkdrse  328
DSSP  ----------lllhhhlll
```

No 81: Query=mol1A Sbjct=4f5oA Z-score=7.1

back to top

```
DSSP  ------------------------------------------------------------
Query ------------------------------------------------------------    0
ident 
Sbjct tlnggitdmltelanfeknvsqaihkynayrkaasviakyphkiksgaeakklpgvgtki   60
DSSP  lllhhhhhhhhhhhhhhhhllllhhhhhhhhhhhhhhhllllllllhhhhhllllllhhh


DSSP  ------------------------------------------------llllllllllhh
Query ------------------------------------------------aapysspptnlr   12
ident 
Sbjct aekideflatgklrklekirqddtsssinfltrvsgigpsaarkfvdegiktledlrkne  120
DSSP  hhhhhhhhhhlllhhhhhhhhhlhhhhhhhhhllllllhhhhhhhhhllllllhhhhhhlh


DSSP  hhhhhhhhhhhhllllhhhhhHHHHHHHHHHHHH-LLLLllLLLLEEEEHHHHHHHhhll
Query slrdrltqvaerqgvvfgrlqRHVAXIVVAQFAA-TLTDdtGAPLLLVKGGSSLELrrgi   71
ident                                                |  |
Sbjct dklnhhqriglkyfgdfekriPREEMLQMQDIVLnEVKKvdSEYIATVCGSFRRGA----  176
DSSP  hhllhhhhhhhhlhhhhhlleEHHHHHHHHHHHHhHHHHhlLLLEEEEELHHHHLL----


DSSP  llllLLLLEEEEEL--------lLHHHHHHHHHHHHhhlllleeEEELLLEELllllllL
Query pdsrTSKDFATVAR--------rDIELIHEQLADAGetgwegftAIFTAPEEIaapgxpV  123
ident      | |                     |||             |
Sbjct ---eSSGDMDVLLThpsftseskLLHQVVEQLQKVH--------FITDTLSKG------E  219
DSSP  ---lEELLEEEEEElllllllllHHHHHHHHHHHLL--------LEEEEEEEL------L


DSSP  LLEEEEEEEEEL-LEEEEEEEEEEEELLLLLLllleeeeelhhhhlllllleeeeellhH
Query KPRRFTAKLSYR-GRAFATVPIEVSSVEAGNAdqfdtltsdalglvgvpaavavpcxtiP  182
ident         |            |
Sbjct TKFMGVCQLPSKdEKEYPHRRIDIRLIPKDQY--------------------------yC  253
DSSP  LEEEEEELLLLLlLLLLLLEEEEEEELLHHHH--------------------------hH


DSSP  HHHhhhhhhhhhlllllllllllhhhhHHHHHH----------------------hhllh
Query WQIaqklhavtavleepkandrahdlvDLQLLE----------------------gllld  220
ident 
Sbjct GVL--------------------yftgSDIFNKnmkahalekgftineytirplgvtgva  293
DSSP  HHH--------------------hhhlLHHHHHhhhhhhhhlleeellleeellllllll


DSSP  hhhhhhhHHHHhhhhhhllllLLLLLlllllhhhhhhhhhllllllllllllhhhhhhhhh
Query adlxptrSACIaifearaqhpWPPRVatlphwpliyagalegldhlelartvdaaaqavq  280
ident        |
Sbjct geplpvdSEKD-------ifdYIQWK----------------------------------  312
DSSP  lllllllLHHH-------hhhHLLLL----------------------------------


DSSP  hhhhhhhhllll
Query rfvaridratkr  292
ident 
Sbjct ---yrepkdrse  321
DSSP  ---lllhhhlll
```

No 82: Query=mol1A Sbjct=1tvaA Z-score=7.1

back to top

```
DSSP  ------------------------------------------------------------
Query ------------------------------------------------------------    0
ident 
Sbjct kapqetlnggitdmltelanfeknvsqaihkynayrkaasviakyphkiksgaeakklpg   60
DSSP  llllhhhhhhhhhhhhhhhhhhhhllllhhhhhhhhhhhhhhhhlllllllhhhhhllll


DSSP  -----------------------------------------------------lllllll
Query -----------------------------------------------------aapyssp    7
ident 
Sbjct vgtkiaekideflatgklrklekirqddtsssinfltrvsgigpsaarkfvdegiktled  120
DSSP  llhhhhhhhhhhhhhlllhhhhhhhhhlhhhhhhhhhllllllhhhhhhhhhlllllhhh


DSSP  lllhhhhhhhhhhhhhhllllhhhhhHHHHHHHHHHHHH-LLLLllLLLLEEEEHHHHHH
Query ptnlrslrdrltqvaerqgvvfgrlqRHVAXIVVAQFAA-TLTDdtGAPLLLVKGGSSLE   66
ident                                                     |  |
Sbjct lrknedklnhhqriglkyfgdfekriPREEMLQMQDIVLnEVKKvdSEYIATVCGSFRRG  180
```

```
DSSP  hhllhhhllhhhhhhhhhlllhhhlleEHHHHHHHHHHhHHHHhlLLLEEEEELHHHHLL


DSSP  HhhllllllLLLLEEEEEL------------lLHHHHHHHHHHHHhhlllleeEEELLLE
Query LrrgipdsrTSKDFATVAR------------rDIELIHEQLADAGetgwegftAIFTAPE  114
ident           | |                         |||             |
Sbjct A-------eSSGDMDVLLThpsftsestkqpkLLHQVVEQLQKVH--------FITDTLS  225
DSSP  L-------lEELLEEEEEEllllllllllllhHHHHHHHHHHHHLL--------LEEEEEE


DSSP  ELllllllLLLEEEEEEEEEEL--LEEEEEEEEEEEEL-LLLLlllleeeeelhhhhllll
Query EIaapgxpVKPRRFTAKLSYR--GRAFATVPIEVSSV-EAGNadqfdtltsdalglvgvp  171
ident                  |             |
Sbjct KG------ETKFMGVCQLPSKndEKEYPHRRIDIRLIpKDQY------------------  261
DSSP  EL------LLEEEEEEELLLLLllLLLLLLEEEEEEEELlHHHH------------------


DSSP  lleeeeellhHHHHhhhhhhhhllllllllllllhhhhHHHHHH----------------
Query aavavpcxtiPWQIaqklhavtavleepkandrahdlvDLQLLE----------------  215
ident
Sbjct ---------yCGVL--------------------yftgSDIFNKnmrahalekgftiney  292
DSSP  ---------hHHHH--------------------hhhlLHHHHHhhhhhhhhlleeelll


DSSP  ------hhllhhhhhhhhHHHHhhhhhhllllLLLLLlllllhhhhhhhhhllllllll
Query ------glllddadlxptrSACIaifearaqhpWPPRVatlphwpliyagalegldhlela  269
ident                   |
Sbjct tirplgvtgvageplpvdSEKD-------ifdYIQWK-----------------------  322
DSSP  leeellllllllllllllLHHH-------hhhHLLLL-----------------------


DSSP  llhhhhhhhhhhhhhhhhhllll
Query rtvdaaaqavqrfvaridratkr  292
ident
Sbjct --------------yrepkdrse  331
DSSP  --------------lllhhhlll
```

No 83: Query=mol1A Sbjct=4f5rA Z-score=7.1

back to top

```
DSSP  ------------------------------------------------------------
Query ------------------------------------------------------------    0
ident
Sbjct tlnggitdmltelanfeknvsqaihkynayrkaasviakyphkiksgaeakklpgvgtki   60
DSSP  lllhhhhhhhhhhhhhhhhhlllllhhhhhhhhhhhhhhhhhllllllllhhhhhlllllllhhh


DSSP  ------------------------------------------------llllllllllhh
Query ------------------------------------------------aapysspptnlr   12
ident
Sbjct aekideflatgklrklekirqddtsssinfltrvsgigpsaarkfvdegiktledlrkne  120
DSSP  hhhhhhhhhhlllhhhhhhhhllhhhhhhhhhhllllllllhhhhhhhhhlllllhhhhhhlh


DSSP  hhhhhhhhhhhhllllhhhhhHHHHHHHHHHHHHHH-LLLLllLLLLEEEEHHHHHHHHhhll
Query slrdrltqvaerqgvvfgrlqRHVAXIVVAQFAA-TLTDdtGAPLLLVKGGSSLELrrgi   71
ident                                                |
Sbjct dklnhhqriglkyfgdfekriPREEMLQMQDIVLnEVKKvdSEYIATVCGSFRRGA----  176
DSSP  hhllhhhhhhhhhlhhhhllleEHHHHHHHHHHHHHhHHHHhlLLLEEEEEELLLLLLL----


DSSP  llllLLLLEEEEEEL-----------lLHHHHHHHHHHHHHhhlllleeEEELLLEELllll
Query pdsrTSKDFATVAR-----------rDIELIHEQLADAGetgwegftAIFTAPEEIaapg  120
ident      | |                        |||             |
Sbjct ---eSSGDMDVLLThpsftsestqpkLLHQVVEQLQKVH--------FITDTLSKG----  221
DSSP  ---lEELLEEEEEEllllllllllllHHHHHHHHHHHHLL--------LEEEEEEEL----


DSSP  llLLLEEEEEEEEEEELL--EEEEEEEEEEEEEL-LLLLllllleeeeelhhhhllllllleeee
Query xpVKPRRFTAKLSYRG--RAFATVPIEVSSV-EAGNadqfdtltsdalglvgvpaavavp  177
ident            |             |
Sbjct --ETKFMGVCQLPSKNdeKEYPHRRIDIRLIpKDQY------------------------  255
DSSP  --LLEEEEEEELLLLLLllLLLLLLEEEEEEEELlHHHH------------------------


DSSP  ellhHHHHhhhhhhhhllllllllllllhhhhHHHHHHH----------------------
```

```
Query cxtiPWQIaqklhavtavleepkandrahdlvDLQLLE----------------------  215
ident
Sbjct ---yCGVL--------------------yftgSDIFNKnmkahalekgftineytirplg  292
DSSP  ---hHHHH--------------------hhhlLHHHHHhhhhhhhhlleeelllleeell


DSSP  hhllhhhhhhhhhHHHHhhhhhhllllLLLLLlllllhhhhhhhhlllllllllllhhhh
Query glllldadlxptrSACIaifearaqhpWPPRVatlphwpliyagalegldhlelartvdaa  275
ident             |
Sbjct vtgvageplpvdSEKD-------ifdYIQWK-----------------------------  316
DSSP  llllllllllllLHHH-------hhhHLLLL-----------------------------


DSSP  hhhhhhhhhhhhhllll
Query aqavqrfvaridratkr  292
ident
Sbjct --------yrepkdrse  325
DSSP  --------lllhhhlll
```

No 84: Query=mol1A Sbjct=4gxiA Z-score=7.1

back to top

```
DSSP  ------------------------------------------------------------
Query ------------------------------------------------------------    0
ident
Sbjct pqtlnggitdmltelanfeknvsqaihkynayrkaasviakyphkiksgaeakklpgvgt   60
DSSP  llllhhhhhhhhhhhhhhhhhllllhhhhhhhhhhhhhhhhhllllllhhhhhllllllh


DSSP  --------------------------------------------------llllllllll
Query --------------------------------------------------aapysspptn   10
ident
Sbjct kiaekideflatgklrklekirqddtsssinfltrvsgigpsaarkfvdegiktledlrk  120
DSSP  hhhhhhhhhhhhlllhhhhhhhhlhhhhhhhhhllllllhhhhhhhhhlllllhhhhhhh


DSSP  hhhhhhhhhhhhhhllllhhhhhHHHHHHHHHHHHH-LLLLllLLLLEEEEHHHHHHHhh
Query lrslrdrltqvaerqgvvfgrlqRHVAXIVVAQFAA-TLTDdtGAPLLLVKGGSSLELrr   69
ident                                                  | |
Sbjct nedklnhhqriglkyfgdfekriPREEMLQMQDIVLnEVKKvdSEYIATVCGSFRRGA--  178
DSSP  lhhhllhhhhhhhhlhhhhllleEHHHHHHHHHHHHhHHHHhlLLLEEEEELLLLLLL--


DSSP  llllllLLLLEEEEEL------------lLHHHHHHHHHHHHhhlllleeEEELLLEELl
Query gipdsrTSKDFATVAR------------rDIELIHEQLADAGetgwegftAIFTAPEEIa  117
ident        | |                         |||             |
Sbjct -----eSSGDMDVLLThpsftsestkqpkLLHQVVEQLQKVH--------FITDTLSKG-  224
DSSP  -----lEELLEEEEEElllllllllllllHHHHHHHHHHHLL--------LEEEEEEEL-


DSSP  lllllLLLEEEEEEEEELL--EEEEEEEEEEEELLLLLLllleeeeelhhhhllllllee
Query apgxpVKPRRFTAKLSYRG--RAFATVPIEVSSVEAGNAdqfdtltsdalglvgvpaava  175
ident               |             |
Sbjct -----ETKFMGVCQLPSKNdeKEYPHRRIDIRLIPKDQY---------------------  258
DSSP  -----LLEEEEEELLLLLLllLLLLEEEEEEEELLHHHH---------------------


DSSP  eeellhHHHHhhhhhhhhllllllllllllhhhhHHHHHH--------------------
Query vpcxtiPWQIaqklhavtavleepkandrahdlvDLQLLE--------------------  215
ident
Sbjct -----yCGVL--------------------yftgSDIFNKnmkahalekgftineytirp  293
DSSP  -----hHHHH--------------------hhhlLHHHHHhhhhhhhhlleeelllleee


DSSP  --hhllhhhhhhhhhHHHHhhhhhhllllLLLLLlllllhhhhhhhhlllllllllllhh
Query --glllldadlxptrSACIaifearaqhPWPPRvatlphwpliyagalegldhlelartvd  273
ident               |
Sbjct lgvtgvageplpvdSEKD------ifdYIQWK----------------------------  319
DSSP  llllllllllllllLHHH------hhhHLLLL----------------------------


DSSP  hhhhhhhhhhhhhhhllll
Query aaaqavqrfvaridratkr  292
ident
Sbjct ----------yrepkdrse  328
DSSP  ----------lllhhhlll
```

No 85: Query=mol1A Sbjct=4kluA Z-score=7.1

back to top

```
DSSP  ------------------------------------------------------------
Query ------------------------------------------------------------     0
ident 
Sbjct lnggitdmltelanfeknvsqaihkynayrkaasviakyphkiksgaeakklpgvgtkia    60
DSSP  llhhhhhhhhhhhhhhhhllllhhhhhhhhhhhhhhhlllllllhhhhhlllllhhhh


DSSP  -----------------------------------------------llllllllllhhh
Query -----------------------------------------------aapyssppptnlrs    13
ident 
Sbjct ekideflatgklrklekirqddtsssinfltrvsgigpsaarkfvdegiktledlrkned   120
DSSP  hhhhhhhhhlllhhhhhhhllhhhhhhhhhlllllllhhhhhhhhhlllllhhhhhhlhh


DSSP  hhhhhhhhhhllllhhhhhHHHHHHHHHHHHH-LLLLllLLLLEEEEHHHHHHHHhhlll
Query lrdrltqvaerqgvvfgrlqRHVAXIVVAQFAA-TLTDdtGAPLLLVKGGSSLELrrgip    72
ident                                               | |
Sbjct klnhhqriglkyfgdfekriPREEMLQMQDIVLnEVKKvdSEYIATVCGSFRRGA-----   175
DSSP  hllhhhhhhhlhhhhllleEHHHHHHHHHHHHHhHHHHhlLLLEEEEELLHHHHLL-----


DSSP  lllLLLLEEEEEL----------lLHHHHHHHHHHHHhhlllleeEEELLLEELllllll
Query dsrTSKDFATVAR----------rDIELIHEQLADAGetgwegftAIFTAPEEIaapgxp   122
ident     | |                       |||             |
Sbjct --eSSGDMDVLLThpsftsesqpkLLHQVVEQLQKVH--------FITDTLSKG------   219
DSSP  --lEELLEEEEEEllllllllllHHHHHHHHHHHLL--------LEEEEEEEL------


DSSP  LLLEEEEEEEEEEL-LEEEEEEEEEEEEL-LLLLllllleeeeelhhhhllllllleeeeell
Query VKPRRFTAKLSYR-GRAFATVPIEVSSV-EAGNadqfdtltsdalglvgvpaavavpcxt   180
ident          |            |
Sbjct ETKFMGVCQLPSKdEKEYPHRRIDIRLIpKDQY---------------------------   252
DSSP  LLEEEEEELLLLLlLLLLLLEEEEEEEELlHHHH---------------------------


DSSP  hHHHHhhhhhhhhlllllllllllhhhhHHHHHH----------------------hhl
Query iPWQIaqklhavtavleepkandrahdlvDLQLLE----------------------gll   218
ident 
Sbjct yCGVL--------------------yftgSDIFNKnmrahalekgftineytirplgvtg   292
DSSP  hHHHH--------------------hhhlLHHHHHhhhhhhhhlleeellllleeelllll


DSSP  lhhhhhhhhHHHHhhhhhhlllLLLLLLlllllhhhhhhhhhllllllllllhhhhhhh
Query ldadlxptrSACIaifearaqhPWPPRVatlphwpliyagalegldhlelartvdaaaqa   278
ident          |
Sbjct vageplpvdSEKD-------ifDYIQWK--------------------------------   313
DSSP  lllllllllLHHH-------hhHHLLLL--------------------------------


DSSP  hhhhhhhhhhllll
Query vqrfvaridratkr  292
ident 
Sbjct -----yrepkdrse  322
DSSP  -----lllhhhlll
```

No 86: Query=mol1A Sbjct=1nomA Z-score=7.1

back to top

```
DSSP  ---------------------------llllllllllhhhhhhhhhhhhhhllllhhhhh
Query ---------------------------aapyssppptnlrslrdrltqvaerqgvvfgrlq    33
ident 
Sbjct ddtsssinfltrvtgigpsaarklvdegiktledlrknedklnhhqriglkyfedfekri    60
DSSP  lllhhhhhhhlllllllhhhhhhhhhlllllhhhhhllhhhllhhhhhhhhhhhhhlllle


DSSP  HHHHHHHHHHHHH-LLLLllLLLLEEEEHHHHHHHHhhllllllLLLLEEEEEL-------
Query RHVAXIVVAQFAA-TLTDdtGAPLLLVKGGSSLELrrgipdsrTSKDFATVAR-------    85
ident                           | |               | |
```

```
Sbjct PREEMLQMQDIVLnEVKKvdPEYIATVCGSFRRGA-------eSSGDMDVLLThpsftse  113
DSSP  EHHHHHHHHHHHHHhHHHHhlLLLEEEELHHHHLLL-------lEELLEEEEEEllllll


DSSP  -----lLHHHHHHHHHHHHHhhllllleeEEELLLEELllllllLLLEEEEEEEEEELLeEEE
Query -----rDIELIHEQLADAGetgwegftAIFTAPEEIaapgxpVKPRRFTAKLSYRGrAFA  140
ident             |||             |                      |
Sbjct sskqpkLLHRVVEQLQKVR--------FITDTLSKG------ETKFMGVCQLPSEN-EYP  158
DSSP  llllhhHHHHHHHHHHHHHL--------LEEEEEEEL------LLEEEEEEELLLLLL-LLL


DSSP  EEEEEEEEL-LLLLllllleeeeelhhhhllllllleeeeellhHHHHhhhhhhhhllllll
Query TVPIEVSSV-EAGNadqfdtltsdalglvgvpaavavpcxtiPWQIaqklhavtavleep  199
ident    |
Sbjct HRRIDIRLIpKDQY---------------------------yCGVL--------------  177
DSSP  LEEEEEEELlLLLH---------------------------hHHHH--------------


DSSP  llllllhhhhHHHHHH----------------------hhllhhhhhhhhHHHHhhhhhh
Query kandrahdlvDLQLLE----------------------gllldadlxptrSACIaifear  237
ident                                                   |
Sbjct ------yftgSDIFNKnmrahalekgftineytirplgvtgvageplpvdSEQD------  225
DSSP  ------hlllLHHHHHhhhhhhhhlleeellllleeellllllllllllllLLHH------


DSSP  llllLLLLllllllhhhhhhhhhllllllllllhhhhhhhhhhhhhhhhhllll
Query aqhpWPPRvatlphwpliyagalegldhlelartvdaaaqavqrfvaridratkr  292
ident
Sbjct -ifdYIQW-------------------------------------ryrepkdrse  242
DSSP  -hhhHLLL-------------------------------------llllllhhhll
```

No 87: Query=mol1A Sbjct=2fmpA Z-score=7.1

back to top

```
DSSP  ------------------------------------------------------------
Query ------------------------------------------------------------    0
ident
Sbjct tlnggitdmltelanfeknvsqaihkynayrkaasviakyphkiksgaeakklpgvgtki   60
DSSP  lllhhhhhhhhhhhhhhhhhllllhhhhhhhhhhhhhhhhllllllllhhhhhlllllhhh


DSSP  ------------------------------------------------llllllllllhh
Query ------------------------------------------------aapysspptnlr   12
ident
Sbjct aekideflatgklrklekirqddtsssinfltrvsgigpsaarkfvdegiktledlrkne  120
DSSP  hhhhhhhhhhllllhhhhhhhhlhhhhhhhhhhllllllllhhhhhhhhhllllllhhhhhllh


DSSP  hhhhhhhhhhhhllllhhhhhHHHHHHHHHHHHH-LLLLllLLLLEEEEHHHHHHHhhll
Query slrdrltqvaerqgvvfgrlqRHVAXIVVAQFAA-TLTDdtGAPLLLVKGGSSLELrrgi   71
ident                                                |  |
Sbjct dklnhhqriglkyfgdfekriPREEMLQMQDIVLnEVKKvdSEYIATVCGSFRRGA----  176
DSSP  hhllhhhhhhhhhlhhhhllleEHHHHHHHHHHHHHhHHHHhlLLLEEEELHHHHLLL----


DSSP  llllLLLLEEEEEL------------lLHHHHHHHHHHHHHhhllllleeEEELLLEELlll
Query pdsrTSKDFATVAR------------rDIELIHEQLADAGetgwegftAIFTAPEEIaap  119
ident      | |                         |||             |
Sbjct ---eSSGDMDVLLThpsftsestkqpkLLHQVVEQLQKVH--------FITDTLSKG---  222
DSSP  ---lEELLEEEEEEllllllllllllllHHHHHHHHHHHHHLL--------LEEEEEEEL---


DSSP  lllLLLEEEEEEEEEEL--LEEEEEEEEEEEEL-LLLLllllleeeeelhhhhlllllleee
Query gxpVKPRRFTAKLSYR--GRAFATVPIEVSSV-EAGNadqfdtltsdalglvgvpaavav  176
ident             |             |
Sbjct ---ETKFMGVCQLPSKndEKEYPHRRIDIRLIpKDQY-----------------------  256
DSSP  ---LLEEEEEEELLLLLllLLLLLLLEEEEEEEELlHHHH-----------------------


DSSP  eellhHHHHhhhhhhhhlllllllllllHHHHHHH------------------hhhhh
Query pcxtiPWQIaqklhavtavleepkandraHDLVDLQ------------------llegl  217
ident                               |
Sbjct ----yCGVL----------------yftgSDIFNKNmrahalekgftineytirplgvtg  296
DSSP  ----hHHHH----------------hhhlLHHHHHHhhhhhhhlleeellllleeelllll
```

```
DSSP  llhHHHHhhHHHHHhhhhhhllllLLLLLLllllhhhhhhhhhlllllllllllhhhhhh
Query lldADLXptRSACIaifearaqhpWPPRVAtlphwpliyagalegldhlelartvdaaaq  277
ident      |
Sbjct vagEPLP--VDSEK-------difDYIQWK------------------------------  317
DSSP  lllLLLL--LLLHH-------hhhHHLLLL------------------------------
```

```
DSSP  hhhhhhhhhhllll
Query avqrfvaridratkr  292
ident
Sbjct ------yrepkdrse  326
DSSP  ------lllhhhlll
```

No 88: Query=mol1A Sbjct=1huoA Z-score=7.1

back to top

```
DSSP  ------------------------------------------------------------
Query ------------------------------------------------------------    0
ident
Sbjct tlnggitdmlvelanfeknvsqaihkynayrkaasviakyphkiksgaeakklpgvgtki   60
DSSP  lllhhhhhhhhhhhhhhhhlllllhhhhhhhhhhhhhhhlllllllhhhhhllllllhhh
```

```
DSSP  ------------------------------------------------llllllllllhh
Query ------------------------------------------------aapysspptnlr   12
ident
Sbjct aekideflatgklrklekirqddtsssinfltrvtgigpsaarklvdegiktledlrkne  120
DSSP  hhhhhhhhllllhhhhhhhhlhhhhhhhhhhlllllhhhhhhhhhlllllhhhhllll
```

```
DSSP  hhhhhhhhhhhhllllhHHHHHHHHHHHHHHHHH-LLLLllLLLLEEEEHHHHHHHhhll
Query slrdrltqvaerqgvvfGRLQRHVAXIVVAQFAA-TLTDdtGAPLLLVKGGSSLELrrgi   71
ident                                                | |
Sbjct dklnhhqriglkyfedfEKRIPREEMLQMQDIVLnEVKKldPEYIATVCGSFRRGA----  176
DSSP  llllhhhhhhhhlllllLLLEEHHHHHHHHHHHHhHHHHhlLLLEEELLHHHHLLL----
```

```
DSSP  llllLLLLEEEEEL------------lLHHHHHHHHHHHHhhllllleeEEELLLEELlll
Query pdsrTSKDFATVAR------------rDIELIHEQLADAGetgwegftAIFTAPEEIaap  119
ident      | |                         |||             |
Sbjct ---eSSGDMDVLLThpnftsesskqpkLLHRVVEQLQKVR--------FITDTLSKG---  222
DSSP  ---lEELEEEEEEEllllllllllllhHHHHHHHHHHHLL--------LEEEEEEEL---
```

```
DSSP  lllLLLEEEEEEEEEL--LEEEEEEEEEEEEL-LLLLllllleeeeelhhhhllllllleee
Query gxpVKPRRFTAKLSYR--GRAFATVPIEVSSV-EAGNadqfdtltsdalglvgvpaavav  176
ident             |             |
Sbjct ---ETKFMGVCQLPSEndENEYPHRRIDIRLIpKDQY------------------------  256
DSSP  ---LLEEEEEELLLLLllLLLLLLEEEEEEELlHHHH------------------------
```

```
DSSP  eellhHHHHhhhhhhhhllllllllllllHHHHHHH-------------------hhhhh
Query pcxtiPWQIaqklhavtavleepkandraHDLVDLQ-------------------llegl  217
ident                               |
Sbjct ----yCGVL----------------yftgSDIFNKNmrahalekgftineytirplgvtg  296
DSSP  ----hHHHH----------------hhhlLHHHHHHhhhhhhhlleeellllleeellll
```

```
DSSP  llhHHHHhhHHHHHhhhhhhllllLLLLLLllllhhhhhhhhhlllllllllllhhhhhh
Query lldADLXptRSACIaifearaqhpWPPRVAtlphwpliyagalegldhlelartvdaaaq  277
ident      |
Sbjct vagEPLP--VDSEQ-------difDYIQWR------------------------------  317
DSSP  lllLLLL--LLLHH-------hhhHHLLLL------------------------------
```

```
DSSP  hhhhhhhhhhllll
Query avqrfvaridratkr  292
ident
Sbjct -------yrepkdrs  325
DSSP  -------llllllll
```

No 89: Query=mol1A Sbjct=3rjkA Z-score=7.1

back to top

```
DSSP  ------------------------------------------------------------
Query ------------------------------------------------------------    0
ident 
Sbjct tlnggitdmltelanfeknvsqaihkynayrkaasviakyphkiksgaeakklpgvgtki   60
DSSP  lllhhhhhhhhhhhhhhhhllllhhhhhhhhhhhhhhhlllllllhhhhhllllllhhh


DSSP  ------------------------------------------------llllllllllhh
Query ------------------------------------------------aapyssspptnlr   12
ident 
Sbjct aekideflatgklrklekirqddtsssinfltrvsgigpsaarkfvdegiktledlrkne  120
DSSP  hhhhhhhhhhlllhhhhhhhhlhhhhhhhhhllllllhhhhhhhhhlllllhhhhhhlh


DSSP  hhhhhhhhhhhhllllhhhhhHHHHHHHHHHHHH-LLLLllLLLLEEEEHHHHHHHHhhll
Query slrdrltqvaerqgvvfgrlqRHVAXIVVAQFAA-TLTDdtGAPLLLVKGGSSLELrrgi   71
ident                                                |  |
Sbjct dklnhhqriglkyfgdfekriPREEMLQMQDIVLnEVKKvdSEYIATVCGSFRRGA----  176
DSSP  hhllhhhhhhhhlllhhhlleEHHHHHHHHHHHHHhHHHHhlLLLEEEEELHHHHLLL----


DSSP  llllLLLLEEEEEL------------lLHHHHHHHHHHHHhhlllleeEEELLLEELlll
Query pdsrTSKDFATVAR------------rDIELIHEQLADAGetgwegftAIFTAPEEIaap  119
ident      | |                         |||             |
Sbjct ---eSSGDMDVLLThpsftsestkqpkLLHQVVEQLQKVH--------FITDTLSKG---  222
DSSP  ---lEELLEEEEEElllllllllllllHHHHHHHHHHHLL--------LEEEEEEEL---


DSSP  lllLLLEEEEEEEEEL--LEEEEEEEEEEEEL-LLLLllllleeeeelhhhhllllllleee
Query gxpVKPRRFTAKLSYR--GRAFATVPIEVSSV-EAGNadqfdtltsdalglvgvpaavav  176
ident             |             |
Sbjct ---ETKFMGVCQLPSKndEKEYPHRRIDIRLIpKDQY-----------------------  256
DSSP  ---LLEEEEEEELLLLLllLLLLLLEEEEEEEELlHHHH-----------------------


DSSP  eellhHHHHhhhhhhhhlllllllllllHHHHHHH------------------hhhhh
Query pcxtiPWQIaqklhavtavleepkandraHDLVDLQ------------------llegl  217
ident                               |
Sbjct ----yCGVL----------------yftgSDIFNKNmrahalekgftineytirplgvtg  296
DSSP  ----hHHHH----------------hhhlLHHHHHHhhhhhhhlleeellllleeelllll


DSSP  llhHHHHhhHHHHHhhhhhhllllLLLLLllllhhhhhhhhhlllllllllllhhhhhh
Query lldADLXptRSACIaifearaqhpWPPRVatlphwpliyagalegldhlelartvdaaaq  277
ident      |
Sbjct vagEPLP--VDSEK-------difDYIQW--------------------------------  316
DSSP  lllLLLL--LLLHH-------hhhHHLLL--------------------------------


DSSP  hhhhhhhhhhhllll
Query avqrfvaridratkr  292
ident 
Sbjct -----kyrepkdrse  326
DSSP  -----llllhhhlll
```

No 90: Query=mol1A Sbjct=5j2dA Z-score=7.1

back to top

```
DSSP  ------------------------------------------------------------
Query ------------------------------------------------------------    0
ident 
Sbjct tlnggitdmltelanfeknvsqaihkynayrkaasviakyphkiksgaeakklpgvgtki   60
DSSP  lllhhhhhhhhhhhhhhhhllllhhhhhhhhhhhhhhhlllllllhhhhhllllllhhh


DSSP  ------------------------------------------------llllllllllhh
Query ------------------------------------------------aapyssspptnlr   12
ident 
Sbjct aekideflatgklrklekirqddtsssinfltrvsgigpsaarkfvdegiktledlrkne  120
DSSP  hhhhhhhhhhlllhhhhhhhhlhhhhhhhhhllllllhhhhhhhhhlllllhhhhhhlh


DSSP  hhhhhhhhhhhhllllhhhhhHHHHHHHHHHHHH-LLLLllLLLLEEEEHHHHHHHHhhll
Query slrdrltqvaerqgvvfgrlqRHVAXIVVAQFAA-TLTDdtGAPLLLVKGGSSLELrrgi   71
```

```
ident                                                  | |
Sbjct dklnhhqriglkyfgdfekriPREEMLQMQDIVLnEVKKvdSEYIATVCGSFRRGA----  176
DSSP  hhllhhhhhhhhlhhhhhlleEHHHHHHHHHHHHhHHHHhlLLLEEEELHHHHLLL----


DSSP  llllLLLLEEEEEL------------lLHHHHHHHHHHHHhhlllleeEEELLLEEllll
Query pdsrTSKDFATVAR------------rDIELIHEQLADAGetgwegftAIFTAPEEiaap  119
ident      | |                         |||             |
Sbjct ---eSSGDMDVLLThpsftsestkqpkLLHQVVEQLQKVH--------FITDTLSK----  221
DSSP  ---lEELLEEEEEElllllllllllllHHHHHHHHHHHLL--------LEEEEEEE----


DSSP  lllLLLEEEEEEEEEL--LEEEEEEEEEEEEL-LLLLlllleeeeelhhhhlllllleee
Query gxpVKPRRFTAKLSYR--GRAFATVPIEVSSV-EAGNadqfdtltsdalglvgvpaavav  176
ident             |             |
Sbjct --gETKFMGVCQLPSKndEKEYPHRRIDIRLIpKDQY-----------------------  256
DSSP  --lLLEEEEEELLLLLllLLLLLLEEEEEEELlHHHH-----------------------


DSSP  eellhHHHHhhhhhhhhllllllllllllHHHH-------------------hhhhhhhh
Query pcxtiPWQIaqklhavtavleepkandraHDLV-------------------dlqllegl  217
ident                               |
Sbjct ----yCGVL----------------yftgSDIFnknmrahalekgftineytirplgvtg  296
DSSP  ----hHHHH----------------hhhlLHHHhhhhhhhhhhlleeelllleeelllll


DSSP  llhHHHHHhhHHHHhhhhhhlllLLLLLLlllllhhhhhhhhhlllllllllllhhhhhh
Query lldADLXPtrSACIaifearaqhPWPPRVatlphwpliyagalegldhlelartvdaaaq  277
ident      |
Sbjct vagEPLPV--DSEK-----difdYIQWKY-------------------------------  318
DSSP  lllLLLLL--LLHH-----hhhhHLLLLL-------------------------------


DSSP  hhhhhhhhhhhllll
Query avqrfvaridratkr  292
ident
Sbjct -------repkdrse  326
DSSP  -------llhhhlll
```

No 91: Query=mol1A Sbjct=4m2yA Z-score=7.1

back to top

```
DSSP  ------------------------------------------------------------
Query ------------------------------------------------------------    0
ident
Sbjct lnggitdmltelanfeknvsqaihkynayrkaasviakyphkiksgaeakklpgvgtkia   60
DSSP  llhhhhhhhhhhhhhhhhllllhhhhhhhhhhhhhhhllllllllhhhhhllllllhhhh


DSSP  -----------------------------------------------llllllllllhhh
Query -----------------------------------------------aapysspptnlrs   13
ident
Sbjct ekideflatgklrklekirqddtsssinfltrvsgigpsaarkfvdegiktledlrkned  120
DSSP  hhhhhhhhhlllhhhhhhhhlhhhhhhhhhlllllllhhhhhhhhhlllllhhhhhllhh


DSSP  hhhhhhhhhhhllllhhhhhHHHHHHHHHHHHHHL-LLLLlLLLEEEEHHHHHHHHhhlll
Query lrdrltqvaerqgvvfgrlqRHVAXIVVAQFAAT-LTDDtGAPLLLVKGGSSLELrrgip   72
ident                                              | |
Sbjct klnhhqriglkyfgdfekriPREEMLQMQDIVLNeVKKVdSEYIATVCGSFRRGA-----  175
DSSP  hllhhhhhhhhlhhhhllleEHHHHHHHHHHHHHhHHHHlLLLEEEELHHHHLLL-----


DSSP  lllLLLLEEEEEL--------lLHHHHHHHHHHHHhhlllleeEEELLLEELllllllLL
Query dsrTSKDFATVAR--------rDIELIHEQLADAGetgwegftAIFTAPEEIaapgxpVK  124
ident     | |                     |||             |
Sbjct --eSSGDMDVLLThpsftseskLLHQVVEQLQKVH--------FITDTLSKG------ET  219
DSSP  --lEELLEEEEEElllllllllHHHHHHHHHHHLL--------LEEEEEEEL------LL


DSSP  LEEEEEEEEELLEEEEEEEEEEEEL-LLLLlllleeeeelhhhhlllllleeeeellhHH
Query PRRFTAKLSYRGRAFATVPIEVSSV-EAGNadqfdtltsdalglvgvpaavavpcxtiPW  183
ident        |           |
Sbjct KFMGVCQLPSDEKEYPHRRIDIRLIpKDQY---------------------------yCG  252
DSSP  EEEEEELLLLLLLLLLLEEEEEEELlHHHH---------------------------hHH
```

```
DSSP  HHhhhhhhhhllllllllllhhhhHHHHHHH-hllhhhhhhhHHHHHH----------
Query QIaqklhavtavleepkandrahdlvDLQLLEG-llldadlxptRSACIA----------  232
ident
Sbjct VL--------------------yftgSDIFNKNmrahalekgftINEYTIrplgvtgvag  292
DSSP  HH--------------------hhhlLHHHHHHhhhhhhhlleeELLLLEeellllllll


DSSP  hHHHH----llllLLLLLlllllhhhhhhhhhllllllllllhhhhhhhhhhhhhhhh
Query iFEAR----aqhpWPPRVatlphwpliyagalegldhlelartvdaaaqavqrfvaridr  288
ident
Sbjct ePLPVdsekdifdYIQWK------------------------------------yrepk  315
DSSP  lLLLLllhhhhhhHLLLL------------------------------------lllhh


DSSP  llll
Query atkr  292
ident
Sbjct drse  319
DSSP  hlll
```

No 92: Query=mol1A Sbjct=1zqyA Z-score=7.1

back to top

```
DSSP  ---------------------------lllllllllhhhhhhhhhhhhhhllllhhhhh
Query ---------------------------aapysspptnlrslrdrltqvaerqgvvfgrlq   33
ident
Sbjct ddtsssinfltrvtgigpsaarklvdegiktledlrknedklnhhqriglkyfedfekri   60
DSSP  lllhhhhhhhlllllllhhhhhhhhhlllllhhhhhllhhhllhhhhhhhhlllhhhlle


DSSP  HHHHHHHHHHHHHL-LLLLLLLLLEEEEHHHHHHHhhllllllLLLLEEEEEL-------
Query RHVAXIVVAQFAAT-LTDDTGAPLLLVKGGSSLELrrgipdsrTSKDFATVAR-------   85
ident                           | |               | |
Sbjct PREEMLQMQDIVLNeVKKLDPEYIATVCGSFRRGA-------eSSGDMDVLLThpnftse  113
DSSP  EHHHHHHHHHHHHHhHHHHLLLLEEEELHHHHLLL-------lEELLEEEEEElllllll


DSSP  -----lLHHHHHHHHHHHHhhlllleeEEELLLEELllllllLLLEEEEEEEEELLeEEE
Query -----rDIELIHEQLADAGetgwegftAIFTAPEEIaapgxpVKPRRFTAKLSYRGrAFA  140
ident             |||               |                      |
Sbjct sskqpkLLHRVVEQLQKVR--------FITDTLSKG------ETKFMGVCQLPSEN-EYP  158
DSSP  llllhhHHHHHHHHHHHHHL--------LEEEEEEEL------LLEEEEEELLLLLL-LLL


DSSP  EEEEEEEEL-LLLLllllleeeeelhhhhllllllleeeeellhHHHHhhhhhhhhllllll
Query TVPIEVSSV-EAGNadqfdtltsdalglvgvpaavavpcxtiPWQIaqklhavtavleep  199
ident    |
Sbjct HRRIDIRLIpKDQY----------------------------yCGVL-------------  177
DSSP  LEEEEEEELlHHHH----------------------------hHHHH-------------


DSSP  llllllhhhhHHHHHH----------------------hhllhhhhhhhhHHHHhhhhhh
Query kandrahdlvDLQLLE----------------------glllldadlxptrSACIaifear  237
ident                                                   |
Sbjct ------yftgSDIFNKnmrahalekgftineytirplgvtgvageplpvdSEQD------  225
DSSP  ------hlllLHHHHHhhhhhhhhlleeellllleeellllllllllllllLHHH------


DSSP  llllLLLLLllllllhhhhhhhhhllllllllllhhhhhhhhhhhhhhhhhllll
Query aqhpWPPRVatlphwpliyagalegldhlelartvdaaaqavqrfvaridratkr  292
ident
Sbjct -ifdYIQWR-------------------------------------yrepkdrse  242
DSSP  -hhhHLLLL-------------------------------------lllhhhlll
```

No 93: Query=mol1A Sbjct=1zqxA Z-score=7.1

back to top

```
DSSP  ---------------------------lllllllllhhhhhhhhhhhhhhllllhhhhh
Query ---------------------------aapysspptnlrslrdrltqvaerqgvvfgrlq   33
ident
Sbjct ddtsssinfltrvtgigpsaarklvdegiktledlrknedklnhhqriglkyfedfekri   60
```

```
DSSP  lllhhhhhhhhllllllhhhhhhhlllllllhhhhhhlhhhllhhhhhhhhhlllhhhlle


DSSP  HHHHHHHHHHHHHL-LLLLlLLLLEEEEHHHHHHHhhllllllLLLLEEEEEL-------
Query RHVAXIVVAQFAAT-LTDDtGAPLLLVKGGSSLELrrgipdsrTSKDFATVAR-------   85
ident                           | |                 | |
Sbjct PREEMLQMQDIVLNeVKKLdPEYIATVCGSFRRGA-------eSSGDMDVLLThpnftse  113
DSSP  EHHHHHHHHHHHHHHhHHHHlLLLEEEELHHHHLLL-------lEELLEEEEEElllllll


DSSP  -----lLHHHHHHHHHHHHHhhllllleeEEELLLEELllllllLLLEEEEEEEEELLeEEE
Query -----rDIELIHEQLADAGetgwegftAIFTAPEEIaapgxpVKPRRFTAKLSYRGrAFA  140
ident             |||              |                        |
Sbjct sskqpkLLHRVVEQLQKVR--------FITDTLSKG------ETKFMGVCQLPSEN-EYP  158
DSSP  llllhhHHHHHHHHHHHHLL--------LEEEEEEEL------LLEEEEEEELLLLLL-LLL


DSSP  EEEEEEEEL-LLLLllllleeeeelhhhhllllllleeeeellhHHHHhhhhhhhhllllll
Query TVPIEVSSV-EAGNadqfdtltsdalglvgvpaavavpcxtiPWQIaqklhavtavleep  199
ident    |
Sbjct HRRIDIRLIpKDQY---------------------------yCGVL--------------  177
DSSP  LEEEEEEELlLLLH---------------------------hHHHH--------------


DSSP  llllllhhhhHHHHHH---------------------hhllhhhhhhhHHHHHhhhhhhl
Query kandrahdlvDLQLLE---------------------glllldadlxptRSACIaifeara  238
ident
Sbjct ------yftgSDIFNKnmrahalekgftineytirplgvtgvageplpVDSEQ-------  224
DSSP  ------hhhlLHHHHHhhhhhhhhlleeellllleeellllllllllllLLLHH-------


DSSP  lllLLLLLLllllhhhhhhhhhllllllllllhhhhhhhhhhhhhhhhhhllll
Query qhpWPPRVAtlphwpliyagalegldhlelartvdaaaqavqrfvaridratkr  292
ident
Sbjct difDYIQWR------------------------------------yrepkdrse  242
DSSP  hhhHHLLLL------------------------------------lllhhhlll
```

No 94: Query=mol1A Sbjct=1bpbA Z-score=7.1

back to top

```
DSSP  ---------------------------llllllllllhhhhhhhhhhhhhhhllllhhhhh
Query ---------------------------aapysspptnlrslrdrltqvaerqgvvfgrlq   33
ident
Sbjct ddtsssinfltrvtgigpsaarklvdegiktledlrknedklnhhqriglkyfedfekri   60
DSSP  lllhhhhhhhhllllllhhhhhhhlllllllhhhhhhlhhhllhhhhhhhhhlllhhhlle


DSSP  HHHHHHHHHHHHHHLL-LLLLLLLLEEEEHHHHHHHhhllllllLLLLEEEEEL-------
Query RHVAXIVVAQFAATL-TDDTGAPLLLVKGGSSLELrrgipdsrTSKDFATVAR-------   85
ident                           | |                 | |
Sbjct PREEMLQMQDIVLNEvKKLDPEYIATVCGSFRRGA-------eSSGDMDVLLThpnftse  113
DSSP  EHHHHHHHHHHHHHHHhHHHLLLLEEEELHHHHLLL-------lEELLEEEEEElllllll


DSSP  -----lLHHHHHHHHHHHHHhhllllleeEEELLLEELllllllLLLEEEEEEEEELLeEEE
Query -----rDIELIHEQLADAGetgwegftAIFTAPEEIaapgxpVKPRRFTAKLSYRGrAFA  140
ident             |||              |                        |
Sbjct sskqpkLLHRVVEQLQKVR--------FITDTLSKG------ETKFMGVCQLPSEN-EYP  158
DSSP  llllhhHHHHHHHHHHHHHL--------LEEEEEEEL------LLEEEEEEELLLLLL-LLL


DSSP  EEEEEEEEL-LLLLllllleeeeelhhhhllllllleeeeellhHHHHhhhhhhhhllllll
Query TVPIEVSSV-EAGNadqfdtltsdalglvgvpaavavpcxtiPWQIaqklhavtavleep  199
ident    |
Sbjct HRRIDIRLIpKDQY---------------------------yCGVL--------------  177
DSSP  LEEEEEEELlHHHH---------------------------hHHHH--------------


DSSP  llllllhhhhHHHHHH----------------------hhllhhhhhhhhHHHHhhhhhh
Query kandrahdlvDLQLLE----------------------glllldadlxptrSACIaifear  237
ident                                                    |
Sbjct ------yftgSDIFNKnmrahalekgftineytirplgvtgvageplpvdSEQD------  225
DSSP  ------hhhlLHHHHHhhhhhhhhlleeellllleeellllllllllllllLHHH------


DSSP  llllLLLLLllllllhhhhhhhhhllllllllllhhhhhhhhhhhhhhhhhhllll
```

```
Query aqhpWPPRVatlphwpliyagalegldhlelartvdaaaqavqrfvaridratkr  292
ident
Sbjct -ifdYIQWR-------------------------------------yrepkdrse  242
DSSP  -hhhHLLLL-------------------------------------lllhhhlll
```

No 95: Query=mol1A Sbjct=2bpcA Z-score=7.1

back to top

```
DSSP  ---------------------------lllllllllhhhhhhhhhhhhhhllllhhhhh
Query ---------------------------aapyssspptnlrslrdrltqvaerqgvvfgrlq   33
ident
Sbjct ddtsssinfltrvtgigpsaarklvdegiktledlrknedklnhhqriglkyfedfekri   60
DSSP  lllhhhhhhhhllllllhhhhhhhhlllllllhhhhllhhhllhhhhhhhhlhhhhhlle


DSSP  HHHHHHHHHHHHHHLL-LLLLLLLLEEEEHHHHHHHhhllllllLLLLEEEEEL-------
Query RHVAXIVVAQFAATL-TDDTGAPLLLVKGGSSLELrrgipdsrTSKDFATVAR-------   85
ident                           | |               | |
Sbjct PREEMLQMQDIVLNEvKKLDPEYIATVCGSFRRGA-------eSSGDMDVLLThpnftse  113
DSSP  EHHHHHHHHHHHHHHhHHHLLLLEEEELLLLLLL-------lEELLEEEEEElllllll


DSSP  -----lLHHHHHHHHHHHHhhlllleeEEELLLEELllllllLLLEEEEEEEEEELLeEEE
Query -----rDIELIHEQLADAGetgwegftAIFTAPEEIaapgxpVKPRRFTAKLSYRGrAFA  140
ident             |||             |                      |
Sbjct sskqpkLLHRVVEQLQKVR--------FITDTLSKG------ETKFMGVCQLPSEN-EYP  158
DSSP  llllhhHHHHHHHHHHHHHL--------LEEEEEEEL------LLEEEEEEELLLLLL-LLL


DSSP  EEEEEEEELLLLLLllleeeeelhhhhllllllleeeeellhHHHHhhhhhhhhlllllll
Query TVPIEVSSVEAGNAdqfdtltsdalglvgvpaavavpcxtiPWQIaqklhavtavleepk  200
ident    |
Sbjct HRRIDIRLIPKDQY--------------------------yCGVL---------------  177
DSSP  LEEEEEEELLLLLH--------------------------hHHHH---------------


DSSP  lllllhhhhHHHHHH----------------------hhllhhhhhhhhHHHHhhhhhhl
Query andrahdlvDLQLLE----------------------glllldadlxptrSACIaifeara  238
ident                                                  |
Sbjct -----yftgSDIFNKnmrahalekgftineytirplgvtgvageplpvdSEQD-------  225
DSSP  -----hlllLHHHHHhhhhhhhhhleeellllleeellllllllllllllLLHH-------


DSSP  lllLLLLLllllhhhhhhhhhlllllllllllhhhhhhhhhhhhhhhhhllll
Query qhpWPPRVatlphwpliyagalegldhlelartvdaaaqavqrfvaridratkr  292
ident
Sbjct ifdYIQWR-------------------------------------yrepkdrse  242
DSSP  hhhHHLLL-------------------------------------lllhhhlll
```

No 96: Query=mol1A Sbjct=5j0sA Z-score=7.1

back to top

```
DSSP  ------------------------------------------------------------
Query ------------------------------------------------------------    0
ident
Sbjct apqetlnggitdmltelanfeknvsqaihkynayrkaasviakyphkiksgaeakklpgv   60
DSSP  llllllhhhhhhhhhhhhhhhhhllllhhhhhhhhhhhhhhhhhlllllllhhhhhlllll


DSSP  ----------------------------------------------------llllllll
Query ----------------------------------------------------aapysspp    8
ident
Sbjct gtkiaekideflatgklrklekirqddtsssinfltrvsgigpsaarkfvdegiktledl  120
DSSP  lhhhhhhhhhhhhhlllhhhhhhhhhlhhhhhhhhhlllllllhhhhhhhhhllllllhhhh


DSSP  llhhhhhhhhhhhhhhhllllhhhhhHHHHHHHHHHHHHH-LLLLLLLLLLEEEEHHHHHHHH
Query tnlrslrdrltqvaerqgvvfgrlqRHVAXIVVAQFAA-TLTDDTGAPLLLVKGGSSLEL   67
ident                                                    | |
Sbjct rknedklnhhqriglkyfgdfekriPREEMLQMQDIVLnEVKKVDSEYIATVCGSFRRGA  180
DSSP  hhlhhhllhhhhhhhhlhhhhhlleEHHHHHHHHHHHHHhHHHHHLLLLEEEELHHHHLLL
```

```
DSSP  hhllllllLLLLEEEEEL---------lLHHHHHHHHHHHHhhlllleeEEELLLEElll
Query rrgipdsrTSKDFATVAR---------rDIELIHEQLADAGetgwegftAIFTAPEEiaa  118
ident          | |                      |||             |
Sbjct -------eSSGDMDVLLThpsftsestkLLHQVVEQLQKVH--------FITDTLSK---  222
DSSP  -------lEELLEEEEEEllllllllllHHHHHHHHHHHLL--------LEEEEEEE---


DSSP  llllLLLEEEEEEEEEL--LEEEEEEEEEEEEL-LLLLlllleeeeelhhhhllllllee
Query pgxpVKPRRFTAKLSYR--GRAFATVPIEVSSV-EAGNadqfdtltsdalglvgvpaava  175
ident              |             |
Sbjct ---gETKFMGVCQLPSKndEKEYPHRRIDIRLIpKDQY----------------------  257
DSSP  ---lLLEEEEEELLLLLllLLLLLLEEEEEEELlHHHH----------------------


DSSP  eeellhHHHHhhhhhhhhllllllllllllHHHH-------------------hhhhhhh
Query vpcxtiPWQIaqklhavtavleepkandraHDLV-------------------dlqlleg  216
ident                                |
Sbjct -----yCGVL----------------yftgSDIFnknmrahalekgftineytirplgvt  296
DSSP  -----hHHHH----------------hhhlLHHHhhhhhhhhhhlleeellllleeellll


DSSP  hllhhHHHHhhHHHHHhhhhhlllLLLLLllllllhhhhhhhhhllllllllllllhhhhh
Query llldaDLXPtrSACIAifearaqhPWPPRvatlphwpliyagalegldhlelartvdaaa  276
ident       |
Sbjct gvagePLPV--DSEKD-----ifdYIQWK-------------------------------  318
DSSP  lllllLLLL--LLHHH-----hhhHLLLL-------------------------------


DSSP  hhhhhhhhhhhhllll
Query qavqrfvaridratkr  292
ident
Sbjct -------yrepkdrse  327
DSSP  -------lllhhhlll
```

No 97: Query=mol1A Sbjct=4m47A Z-score=7.1

back to top

```
DSSP  ------------------------------------------------------------
Query ------------------------------------------------------------    0
ident
Sbjct nggitdmltelanfeknvsqaihkynayrkaasviakyphkiksgaeakklpgvgtkiae   60
DSSP  lhhhhhhhhhhhhhhhhllllhhhhhhhhhhhhhhhllllllllhhhhhllllllhhhhh


DSSP  ---------------------------------------------llllllllllllhhhh
Query ---------------------------------------------aapyssppptnlrsl   14
ident
Sbjct kideflatgklrklekirqddtsssinfltrvsgigpsaarkfvdegiktledlrknedk  120
DSSP  hhhhhhhhlllhhhhhhhhllhhhhhhhhllllllhhhhhhhhhhlllllhhhhhllhhh


DSSP  hhhhhhhhhhllllhHHHHHHHHHHHHHHHHHL-LLLLLLLLEEEEHHHHHHHHhhllll
Query rdrltqvaerqgvvfGRLQRHVAXIVVAQFAAT-LTDDTGAPLLLVKGGSSLELrrgipd   73
ident                                              | |
Sbjct lnhhqriglkyfgdfEKRIPREEMLQMQDIVLNeVKKVDSEYIATVCGSFRRGA------  174
DSSP  llhhhhhhhhlllhhHLLEEHHHHHHHHHHHHHhHHHHLLLLEEEELHHHHLLL------


DSSP  llLLLLEEEEEL--------lLHHHHHHHHHHHHhhlllleeEEELLLEELllllllLLL
Query srTSKDFATVAR--------rDIELIHEQLADAGetgwegftAIFTAPEEIaapgxpVKP  125
ident    | |                     |||             |
Sbjct -eSSGDMDVLLThpsftseskLLHQVVEQLQKVH--------FITDTLSKG------ETK  219
DSSP  -lEELLEEEEEElllllllllHHHHHHHHHHHLL--------LEEEEEEEL------LLE


DSSP  EEEEEEEEEllEEEEEEEEEEEEL-LLLLlllleeeeelhhhhlllllleeeeellhHHH
Query RRFTAKLSYrgRAFATVPIEVSSV-EAGNadqfdtltsdalglvgvpaavavpcxtiPWQ  184
ident       |           |
Sbjct FMGVCQLPS--KEYPHRRIDIRLIpKDQY---------------------------yCGV  250
DSSP  EEEEELLLL--LLLLLEEEEEEELlHHHH---------------------------hHHH


DSSP  HhhhhhhhhllllllllllllHHHHHHH-----------------hhhhhllhHHHHhhH
Query IaqklhavtavleepkandraHDLVDLQ-----------------llegllldADLXptR  227
ident                       |                                |
```

```
Sbjct [illegible]
DSSP  H----------------hhhlLHHHHHHhhhhhhhlleeellllleeelllllLLLL--L


DSSP  HHHHhhhhhhllllLLLLLLllllhhhhhhhhhllllllllllhhhhhhhhhhhhhhhhh
Query SACIaifearaqhpWPPRVAtlphwpliyagalegldhlelartvdaaaqavqrfvarid  287
ident
Sbjct DSEK-------difDYIQWK------------------------------------yrep  309
DSSP  LLHH-------hhhHHHLLL------------------------------------lllh


DSSP  hllll
Query ratkr  292
ident
Sbjct kdrse  314
DSSP  hhlll
```

No 98: Query=mol1A Sbjct=4nlzA Z-score=7.1

back to top

```
DSSP  ------------------------------------------------------------
Query ------------------------------------------------------------    0
ident
Sbjct etlnggitdmltelanfeknvsqaihkynayrkaasviakyphkiksgaeakklpgvgtk   60
DSSP  llllhhhhhhhhhhhhhhhhhllllhhhhhhhhhhhhhhhhllllllllhhhhhllllllhh


DSSP  -------------------------------------------------llllllllllh
Query -------------------------------------------------aapyssspptnl   11
ident
Sbjct iaekideflatgklrklekirqddtsssinfltrvsgigpsaarkfvdegiktledlrkn  120
DSSP  hhhhhhhhhhhlllhhhhhhhhlhhhhhhhhhlllllllhhhhhhhhhllllhhhhhll


DSSP  hhhhhhhhhhhhhllllhHHHHHHHHHHHHHHHHHHL-LLLLLLLLLEEEEHHHHHHHhhl
Query rslrdrltqvaerqgvvfGRLQRHVAXIVVAQFAAT-LTDDTGAPLLLVKGGSSLELrrg   70
ident                                                 | |
Sbjct edklnhhqriglkyfgdfEKRIPREEMLQMQDIVLNeVKKVDSEYIATVCGSFRRGA---  177
DSSP  hhhllhhhhhhhhlhhhhLLLEEHHHHHHHHHHHHHHhHHHHLLLLEEEELHHHHLLL---


DSSP  lllllLLLLEEEEEEL---------lLHHHHHHHHHHHHHhhllllleeeEELL-LEELllll
Query ipdsrTSKDFATVAR---------rDIELIHEQLADAGetgwegftaIFTA-PEEIaapg  120
ident       | |                      |||               |
Sbjct ----eSSGDMDVLLThpsftsesqkLLHQVVEQLQKVH---------FITDtLSKG----  220
DSSP  ----lEELLEEEEEEllllllllllHHHHHHHHHHHHLL---------LEEEeEEEL----


DSSP  llLLLLEEEEEEEEEL-LEEEEEEEEEEEEL-LLLLllllleeeeelhhhhllllllleeeee
Query xpVKPRRFTAKLSYR-GRAFATVPIEVSSV-EAGNadqfdtltsdalglvgvpaavavpc  178
ident            |            |
Sbjct --ETKFMGVCQLPSKdEKEYPHRRIDIRLIpKDQY-------------------------  253
DSSP  --LLEEEEEELLLLLlLLLLLLLEEEEEEEELlHHHH-------------------------


DSSP  llhHHHHhhhhhhhhlllllllllllHHHHHHH------------------hhhhhll
Query xtiPWQIaqklhavtavleepkandraHDLVDLQ------------------lleglll  219
ident                             |
Sbjct --yCGVL----------------yftgSDIFNKNmrahalekgftineytirplgvtgva  295
DSSP  --hHHHH----------------hhhlLHHHHHHhhhhhhhlleeellllleeelllllll


DSSP  hHHHHhhHHHHHhhhhhhllllLLLLLlllllhhhhhhhhhllllllllllhhhhhhhh
Query dADLXptRSACIaifearaqhpWPPRVatlphwpliyagalegldhlelartvdaaaqav  279
ident    |
Sbjct gEPLP--VDSEK-------difDYIQW---------------------------------  313
DSSP  lLLLL--LLLHH-------hhhHHLLL---------------------------------


DSSP  hhhhhhhhhllll
Query qrfvaridratkr  292
ident
Sbjct ---kyrepkdrse  323
DSSP  ---llllhhhlll
```

No 99: Query=mol1A Sbjct=4nm2A Z-score=7.1

back to top

```
DSSP  ------------------------------------------------------------
Query ------------------------------------------------------------     0
ident 
Sbjct nggitdmltelanfeknvsqaihkynayrkaasviakyphkiksgaeakklpgvgtkiae    60
DSSP  lhhhhhhhhhhhhhhhhhllllhhhhhhhhhhhhhhhhlllllllhhhhhlllllhhhhh


DSSP  ----------------------------------------------llllllllllhhhh
Query ----------------------------------------------aapyssppptnlrsl    14
ident 
Sbjct kideflatgklrklekirqddtsssinfltrvsgigpsaarkfvdegiktledlrknedk   120
DSSP  hhhhhhhhlllhhhhhhhllhhhhhhhhhlllllllhhhhhhhhhlllllhhhhhllhhh


DSSP  hhhhhhhhhhllllhHHHHHHHHHHHHHHHHHHL-LLLLLLLLLEEEEHHHHHHHHhhllll
Query rdrltqvaerqgvvfGRLQRHVAXIVVAQFAAT-LTDDTGAPLLLVKGGSSLELrrgipd    73
ident                                              | |
Sbjct lnhhqriglkyfgdfEKRIPREEMLQMQDIVLNeVKKVDSEYIATVCGSFRRGA------   174
DSSP  llhhhhhhhhlhhhhLLLEEHHHHHHHHHHHHHHHhHHHHLLLLEEEELLLHHHLL------


DSSP  llLLLLEEEEEL----------lLHHHHHHHHHHHHhhlllleeEEELLLEELllllllL
Query srTSKDFATVAR----------rDIELIHEQLADAGetgwegftAIFTAPEEIaapgxpV   123
ident    | |                       |||             |
Sbjct -eSSGDMDVLLThpsftsesqpkLLHQVVEQLQKVH--------FITDTLSKG------E   219
DSSP  -lEELLEEEEEEllllllllllHHHHHHHHHHHHLL--------LEEEEEEEL------L


DSSP  LLEEEEEEEEEL-LEEEEEEEEEEEELLLLLLllleeeeelhhhhlllllleeeeellhH
Query KPRRFTAKLSYR-GRAFATVPIEVSSVEAGNAdqfdtltsdalglvgvpaavavpcxtiP   182
ident         |            |
Sbjct TKFMGVCQLPSKdEKEYPHRRIDIRLIPKDQY--------------------------yC   253
DSSP  LEEEEEELLLLlLLLLLLEEEEEEEELLLLLH--------------------------hH


DSSP  HHHhhhhhhhhllllllllllllhhhhHHHHHH----------------------hhllh
Query WQIaqklhavtavleepkandrahdlvDLQLLE----------------------gllld   220
ident 
Sbjct GVL--------------------yftgSDIFNKnmrahalekgftineytirplgvtgva   293
DSSP  HHH--------------------hhhlLHHHHHhhhhhhhhlleeelllleeelllllll


DSSP  hhhhhhhHHHHhhhhhhlllLLLLLLlllllhhhhhhhhhllllllllllhhhhhhhhhh
Query adlxptrSACIaifearaqhPWPPRVatlphwpliyagalegldhlelartvdaaaqavq   280
ident        |
Sbjct geplpvdSEKD-------ifDYIQWK----------------------------------   312
DSSP  lllllllLHHH-------hhHHHLLL----------------------------------


DSSP  hhhhhhhhllll
Query rfvaridratkr  292
ident 
Sbjct ---yrepkdrse  321
DSSP  ---lllhhhlll
```

No 100: Query=mol1A Sbjct=4phdA Z-score=7.1

back to top

```
DSSP  ------------------------------------------------------------
Query ------------------------------------------------------------     0
ident 
Sbjct pqetlnggitdmltelanfeknvsqaihkynayrkaasviakyphkiksgaeakklpgvg    60
DSSP  llllllhhhhhhhhhhhhhhhhhllllhhhhhhhhhhhhhhhhlllllllhhhhhlllll


DSSP  ---------------------------------------------------lllllllll
Query ---------------------------------------------------aapyssppt     9
ident 
Sbjct tkiaekideflatgklrklekirqddtsssinfltrvsgigpsaarkfvdegiktledlr   120
DSSP  hhhhhhhhhhhhhlllhhhhhhhllhhhhhhhhhlllllllhhhhhhhhhlllllhhhhh
```

```
DSSP  lhhhhhhhhhhhhhhllllhhhhhHHHHHHHHHHHHH-LLLLllLLLLEEEEHHHHHHHh
Query nlrslrdrltqvaerqgvvfgrlqRHVAXIVVAQFAA-TLTDdtGAPLLLVKGGSSLELr   68
ident                                                   | |
Sbjct knedklnhhqriglkyfgdfekriPREEMLQMQDIVLnEVKKvdSEYIATVCGSFRRGA-  179
DSSP  llhhhllhhhhhhhhlhhhhllleEHHHHHHHHHHHHhHHHHhlLLLEEEELHHHHLLL-


DSSP  hlllllLLLLLEEEEEL----------lLHHHHHHHHHHHHhhlllleeEEELLLEElll
Query rgipdsRTSKDFATVAR----------rDIELIHEQLADAGetgwegftAIFTAPEEiaa  118
ident         | |                       |||             |
Sbjct ------ESSGDMDVLLThpsftsesqpkLLHQVVEQLQKVH--------FITDTLSK---  222
DSSP  ------LEELLEEEEEEllllllllllHHHHHHHHHHHHLL--------LEEEEEEE---


DSSP  llllLLLEEEEEEEEEL-lEEEEEEEEEEEEL-LLLLlllleeeeelhhhhlllllleee
Query pgxpVKPRRFTAKLSYR-gRAFATVPIEVSSV-EAGNadqfdtltsdalglvgvpaavav  176
ident              |            |
Sbjct ---gETKFMGVCQLPSKdeKEYPHRRIDIRLIpKDQY-----------------------  256
DSSP  ---lLLEEEEEEELLLLLllLLLLLEEEEEEELlHHHH-----------------------


DSSP  eellhHHHHhhhhhhhhllllllllllHHHH-------------------hhhhhhhh
Query pcxtiPWQIaqklhavtavleepkandraHDLV-------------------dlqllegl  217
ident                               |
Sbjct ----yCGVL----------------yftgSDIFnknmrahalekgftineytirplgvtg  296
DSSP  ----hHHHH----------------hhhlLHHHhhhhhhhhhhllleellllleellllll


DSSP  llhhHHHHhhHHHHHhhhhhlllLLLLLllllllhhhhhhhhllllllllllhhhhhh
Query lldaDLXPtrSACIAifearaqhPWPPRvatlphwpliyagalegldhlelartvdaaaq  277
ident      |
Sbjct vagePLPV--DSEKD-----ifdYIQWK--------------------------------  317
DSSP  llllLLLL--LLHHH-----hhhHLLLL--------------------------------


DSSP  hhhhhhhhhhhllll
Query avqrfvaridratkr  292
ident
Sbjct ------yrepkdrse  326
DSSP  ------lllhhhlll
```

No 101: Query=mol1A Sbjct=4m9hA Z-score=7.1

back to top

```
DSSP  ------------------------------------------------------------
Query ------------------------------------------------------------    0
ident
Sbjct lnggitdmltelanfeknvsqaihkynayrkaasviakyphkiksgaeakklpgvgtkia   60
DSSP  llhhhhhhhhhhhhhhhhllllhhhhhhhhhhhhhhhhllllllllhhhhhllllllhhhh


DSSP  -----------------------------------------------lllllllllhhh
Query -----------------------------------------------aapyssppptnlrs   13
ident
Sbjct ekideflatgklrklekirqddtsssinfltrvsgigpsaarkfvdegiktledlrkned  120
DSSP  hhhhhhhhhlllhhhhhhhhlhhhhhhhhhlllllllhhhhhhhhhlllllhhhhhllhh


DSSP  hhhhhhhhhhhllllhHHHHHHHHHHHHHHHHHHH-LLLLllLLLLEEEEHHHHHHHHhlll
Query lrdrltqvaerqgvvfGRLQRHVAXIVVAQFAA-TLTDdtGAPLLLVKGGSSLELrrgip   72
ident                                               | |
Sbjct klnhhqriglkyfgdfEKRIPREEMLQMQDIVLnEVKKvdSEYIATVCGSFRRGA-----  175
DSSP  hllhhhhhhhhlhhhhLLLEEHHHHHHHHHHHHHhHHHHhlLLLEEEELHHHHLLL-----


DSSP  lllLLLLEEEEEL------lLHHHHHHHHHHHHhhlllleeEEELLLEELllllllLLLE
Query dsrTSKDFATVAR------rDIELIHEQLADAGetgwegftAIFTAPEEIaapgxpVKPR  126
ident     | |                   |||             |
Sbjct --eSSGDMDVLLThpsftskLLHQVVEQLQKVH--------FITDTLSKG------ETKF  219
DSSP  --lEELLEEEEEElllllllHHHHHHHHHHHHLL--------LEEEEEEEL------LLEE


DSSP  EEEEEEEEllEEEEEEEEEEEEELLLLLLllleeeeelhhhhllllllleeeeellhHHHHh
Query RFTAKLSYrgRAFATVPIEVSSVEAGNAdqfdtltsdalglvgvpaavavpcxtiPWQIa  186
ident      |           |
```

```
Sbjct MGVCQLPS--KEYPHRRIDIRLIPKDQY--------------------------yCGVL-  250
DSSP  EEEELLLL--LLLLLEEEEEEEELLHHHH--------------------------hHHHH-


DSSP  hhhhhhhlllllllllllhhhhHHHHHH----------------------hhllhhhhh
Query qklhavtavleepkandrahdlvDLQLLE----------------------glllldadlx  224
ident 
Sbjct -------------------yftgSDIFNKnmrahalekgftinkytirplgvtgvagepl  291
DSSP  -------------------hhhlLHHHHhhhhhhhhhlleeellllleeelllllllllll


DSSP  hhhHHHHhhhhhhllllLLLLLlllllhhhhhhhhhllllllllllhhhhhhhhhhhh
Query ptrSACIaifearaqhpWPPRVatlphwpliyagalegldhlelartvdaaaqavqrfva  284
ident    |
Sbjct pvdSEKD-------ifdYIQWK---------------------------------------  306
DSSP  lllLHHH-------hhhHLLLL---------------------------------------


DSSP  hhhhllll
Query ridratkr  292
ident 
Sbjct yrepkdrs  314
DSSP  lllhhhll
```

No 102: Query=mol1A Sbjct=4mfaA Z-score=7.1

back to top

```
DSSP  ------------------------------------------------------------
Query ------------------------------------------------------------    0
ident 
Sbjct lnggitdmltelanfeknvsqaihkynayrkaasviakyphkiksgaeakklpgvgtkia   60
DSSP  llhhhhhhhhhhhhhhhhllllhhhhhhhhhhhhhhhlllllllhhhhhlllllllhhhh


DSSP  -----------------------------------------------lllllllllhhh
Query -----------------------------------------------aapyssspptnlrs   13
ident 
Sbjct ekideflatgklrklekirqddtsssinfltrvsgigpsaarkfvdegiktledlrkned  120
DSSP  hhhhhhhhhlllhhhhhhhhlhhhhhhhhhlllllllhhhhhhhhhlllllhhhhhllhh


DSSP  hhhhhhhhhhhllllhhhhhHHHHHHHHHHHHH-LLLLllLLLLEEEEHHHHHHhhhlll
Query lrdrltqvaerqgvvfgrlqRHVAXIVVAQFAA-TLTDdtGAPLLLVKGGSSLElrrgip   72
ident                                               | |
Sbjct klnhhqriglkyfgdfekriPREEMLQMQDIVLnEVKKvdSEYIATVCGSFRRG------  174
DSSP  hllhhhhhhhhlhhhhllleEHHHHHHHHHHHHHhHHHHhlLLLEEEELHHHHLL------


DSSP  lLLLLLLLEEEEEL----------lLHHHHHHHHHHHHHhhllllleeEEELLLEElllllll
Query dSRTSKDFATVAR----------rDIELIHEQLADAGetgwegftAIFTAPEEiaapgxp  122
ident     | |                       |||           |
Sbjct -AESSGDMDVLLThpsftsesqpkLLHQVVEQLQKVH--------FITDTLSK------g  219
DSSP  -LLEELLEEEEEElllllllllllHHHHHHHHHHHHLL--------LEEEEEEE------l


DSSP  LLLEEEEEEEEEELLEEEEEEEEEEEEELLLLLLllleeeeelhhhhllllllleeeeellhH
Query VKPRRFTAKLSYRGRAFATVPIEVSSVEAGNAdqfdtltsdalglvgvpaavavpcxtiP  182
ident          |           |
Sbjct ETKFMGVCQLPSDEKEYPHRRIDIRLIPKDQY--------------------------yC  253
DSSP  LLEEEEEELLLLLLLLLLLEEEEEEEELLHHHH--------------------------hH


DSSP  HHHhhhhhhhhlllllllllllHHHH------------------hhhhhhhhllhhHH
Query WQIaqklhavtavleepkandraHDLV------------------dlqlleglllldaDL  223
ident                         |                                  |
Sbjct GVL----------------yftgSDIFnknmrahalekgftineytirplgvtgvagePL  297
DSSP  HHH----------------hlllLHHHhhhhhhhhhhhlleeellllleeelllllllllLL


DSSP  HHhhHHHHHhhhhhlllLLLLLlllllllhhhhhhhhhlllllllllllhhhhhhhhhhhh
Query XPtrSACIAifearaqhPWPPRvatlphwpliyagalegldhlelartvdaaaqavqrfv  283
ident 
Sbjct PV--DSEKD-----ifdYIQWK--------------------------------------  312
DSSP  LL--LLHHH-----hhhHLLLL--------------------------------------
```

```
DSSP  hhhhhllll
Query aridratkr  292
ident
Sbjct yrepkdrse  321
DSSP  lllhhhlll
```

No 103: Query=mol1A Sbjct=4p37B Z-score=7.0

back to top

```
DSSP  ------------------llllllllllhHHHHHHHHHHhhhllllhHHHHHHHHHHHHH
Query ------------------aapysspptnlRSLRDRLTQVaerqgvvfGRLQRHVAXIVVA   42
ident                                                      | |
Sbjct nrnrklsyqeyyvdgdyeevrkklpeiikQARIKASQVX------epTIYEKRVVXEIIK   54
DSSP  lllllleellllllllhhhhhhhhhhhhhhHHHHHHHHHL------llLHHHHHHHHHHHH


DSSP  HHHHLLlllllllLEEEEHHHHHHHHHL---lLLLLL----lLLEEEEELLlHHHHHHHH
Query QFAATLtddtgapLLLVKGGSSLELRRG---iPDSRT----sKDFATVARRdIELIHEQL   95
ident  |              | ||  |          |         |               |
Sbjct DFIRDK-------GRKVYGGTALNETIKkxnpEDAIYdsylfSDIEFYSPT-PVPDLKEL  106
DSSP  HHHHHH-------LLEEEHHHHHHHHHHhhlhHHLLLlllllLLEEEEELL-HHHHHHHH


DSSP  HHHHHHLllleeeEELLLEELlllllllLLEEEEEEEeelleeeeEEEEEEEEL-lLLLL
Query ADAGETGwegftaIFTAPEEIaapgxpvKPRRFTAKLsyrgrafaTVPIEVSSV-eAGNA  154
ident  |                                                   |
Sbjct CDILYHK--gydpVQGKEAQH-------EETYSIFVN-------lQLYCDITYVptKVYH  150
DSSP  HHHHHHL--llllEEEEELLL-------LLEEEEEEL-------lEEEEEEEELlhHHHH


DSSP  L-LLEEEEelhhhhlllllleEEEELLHHHHHHHHHHHHHLLLLlllLLLLLHHHHHHHH
Query D-QFDTLTsdalglvgvpaavAVPCXTIPWQIAQKLHAVTAVLEepkANDRAHDLVDLQL  213
ident                                    |      |
Sbjct GiKTIEID-------------GINYTHPHFXLIDYLRXINQPLT-aaEQRWEKAFDRXYV  196
DSSP  HlLEEEEL-------------LEEEELHHHHHHHHHHHHLLHHH-hhHHHHHHHHHHHHH


DSSP  HHH-HLLHH---------------------------------------------------
Query LEG-LLLDA---------------------------------------------------  221
ident |
Sbjct LLKnYPXEKydnsxritsprddiqxyigkvksefxkipeiqesclisgfdaynffirhax  256
DSSP  HHHhLLLLLllllllllllhhhhhhhhhhhhhlhhhlhhhhlleeelhhhhhhhhhhhhh


DSSP  ---------------------------------------hhhhhhhhhhhHHHH------
Query ---------------------------------------dlxptrsaciaIFEA------  236
ident                                                    |
Sbjct gdrknfitvlpfxelisvkykdtveklynflrekvvnpdlitideyfplfQFTGysvsin  316
DSSP  lllllllllllleeeeellhhhhhhhhhhhhhlllllhhheeeeeellllLLLLleeeee


DSSP  ------------------------hllllLLLLllllllhhhhhhhhhllllllllllllh
Query ------------------------raqhpWPPRvatlphwpliyagalegldhlelartv  272
ident
Sbjct ydgipivkvyeadgycvpdikttsgyryvSYQY---------------------------  349
DSSP  elleeeeeeeelllllleeeeelllllleeeLHHH---------------------------


DSSP  hhhhhhhhhHHHHH----------------hHLLL-------------------------
Query daaaqavqrFVARI----------------dRATK-------------------------  291
ident
Sbjct --------iLXIXYiskfkahldknkexyfnYGIAisnlvqarnsylnqknigvindtvf  401
DSSP  --------hHHHHHhhhhhhhhhlllhhhhhHHHHhhhhhhhhhhhhhhhhlllllllll


DSSP  ------------------------------------------------------------
Query ------------------------------------------------------------  291
ident
Sbjct sefrigcigttvsytrxsrlrxlekkkqgkviqfvytpkqyfsqtpeqqnnfdesxkkyr  461
DSSP  llllleellllllhhhhhhhhhhhhhhlllllleelhhhhhhllhhhhhhhhhhhhhlll


DSSP  ------------------------------------l
Query ------------------------------------r  292
ident
Sbjct fkntsgnkitipknllfkidergniseeisteeayit  498
```

```
DSSP  eelllllllllhhhlleeellllleeellllllleel
```

No 104: Query=mol1A Sbjct=4tusA Z-score=7.0

back to top

```
DSSP  ------------------------------------------------------------
Query ------------------------------------------------------------    0
ident 
Sbjct tlnggitdmltelanfeknvsqaihkynayrkaasviakyphkiksgaeakklpgvgtki   60
DSSP  lllhhhhhhhhhhhhhhhhhllllhhhhhhhhhhhhhhhhlllllllhhhhlllllllhhh


DSSP  ------------------------------------------------llllllllllhh
Query ------------------------------------------------aapyssppptnlr   12
ident 
Sbjct aekideflatgklrklekirqddtsssinfltrvsgigpsaarkfvdegiktledlrkne  120
DSSP  hhhhhhhhhhlllhhhhhhhhlhhhhhhhhhllllllhhhhhhhhhlllllhhhhhhlh


DSSP  hhhhhhhhhhhhllllhhhhhHHHHHHHHHHHHHH-LLLLllLLLLEEEEHHHHHHHhhll
Query slrdrltqvaerqgvvfgrlqRHVAXIVVAQFAA-TLTDdtGAPLLLVKGGSSLELrrgi   71
ident                                                 | |
Sbjct dklnhhqriglkyfgdfekriPREEMLQMQDIVLnEVKKvdSEYIATVCGSFRRGA----  176
DSSP  hhllhhhhhhhhlhhhhllleEHHHHHHHHHHHHHhHHHHhlLLLEEEEELHHHHLLL----


DSSP  llllLLLLEEEEEL------------lLHHHHHHHHHHHHhhlllleeEEELLLEEllll
Query pdsrTSKDFATVAR------------rDIELIHEQLADAGetgwegftAIFTAPEEiaap  119
ident        | |                          |||             |
Sbjct ---eSSGDMDVLLThpsftsestkqpkLLHQVVEQLQKVH--------FITDTLSK----  221
DSSP  ---lEELLEEEEEEllllllllllllHHHHHHHHHHHLL--------LEEEEEEE----


DSSP  lllLLLEEEEEEEEEL-LEEEEEEEEEEEEL-LLLLllllleeeeelhhhhlllllleeee
Query gxpVKPRRFTAKLSYR-GRAFATVPIEVSSV-EAGNadqfdtltsdalglvgvpaavavp  177
ident             |            |
Sbjct --gETKFMGVCQLPSKnEKEYPHRRIDIRLIpKDQY------------------------  255
DSSP  --lLLEEEEEEELLLLlLLLLLLLEEEEEEEELlHHHH------------------------


DSSP  ellhHHHHhhhhhhhhllllllllllllHHHH------------------hhhhhhhhl
Query cxtiPWQIaqklhavtavleepkandraHDLV------------------dlqllegll  218
ident                              |
Sbjct ---yCGVL----------------yftgSDIFnknmrahalekgftineytirplgvtgv  296
DSSP  ---hHHHH----------------hhhlLHHHhhhhhhhhhhhlleeellllleeellllll


DSSP  lhHHHHHhhHHHHhhhhhhlllLLLLLLlllllhhhhhhhhhlllllllllllhhhhhhh
Query ldADLXPtrSACIaifearaqhPWPPRVatlphwpliyagalegldhlelartvdaaaqa  278
ident     |
Sbjct agEPLPV--DSEK-----difdYIQWKY--------------------------------  317
DSSP  llLLLLL--LLHH-----hhhhHLLLLL--------------------------------


DSSP  hhhhhhhhhhllll
Query vqrfvaridratkr  292
ident 
Sbjct ------repkdrse  325
DSSP  ------llhhhlll
```

No 105: Query=mol1A Sbjct=4ub4A Z-score=7.0

back to top

```
DSSP  ------------------------------------------------------------
Query ------------------------------------------------------------    0
ident 
Sbjct tlnggitdmltelanfeknvsqaihkynayrkaasviakyphkiksgaeakklpgvgtki   60
DSSP  lllhhhhhhhhhhhhhhhhhllllhhhhhhhhhhhhhhhhlllllllhhhhhlllllllhhh


DSSP  ------------------------------------------------llllllllllhh
Query ------------------------------------------------aapyssppptnlr   12
```

```
ident
Sbjct aekideflatgklrklekirqddtsssinfltrvsgigpsaarkfvdegiktledlrkne  120
DSSP  hhhhhhhhhhlllhhhhhhhhlhhhhhhhhhllllllhhhhhhhhhlllllhhhhhllh


DSSP  hhhhhhhhhhhhllllhhhhhHHHHHHHHHHHHHH-LLLLllLLLLEEEEHHHHHHHHhhll
Query slrdrltqvaerqgvvfgrlqRHVAXIVVAQFAA-TLTDdtGAPLLLVKGGSSLELrrgi   71
ident                                                |  |
Sbjct dklnhhqriglkyfgdfekriPREEMLQMQDIVLnEVKKvdSEYIATVCGSFRRGA----  176
DSSP  hhllhhhhhhhhlhhhhhlleEHHHHHHHHHHHHHhHHHHhlLLLEEEELHHHHLLL----


DSSP  llllLLLLEEEEEL------------lLHHHHHHHHHHHHHhhlllleeEEELLLEEllll
Query pdsrTSKDFATVAR------------rDIELIHEQLADAGetgwegftAIFTAPEEiaap  119
ident      | |                         |||             |
Sbjct ---eSSGDMDVLLThpsftsestkqpkLLHQVVEQLQKVH--------FITDTLSK----  221
DSSP  ---lEELLEEEEEEllllllllllllHHHHHHHHHHHHLL--------LEEEEEEE----


DSSP  lllLLLEEEEEEEEEL--LEEEEEEEEEEEEL-LLLLlllleeeeelhhhhllllllleee
Query gxpVKPRRFTAKLSYR--GRAFATVPIEVSSV-EAGNadqfdtltsdalglvgvpaavav  176
ident             |             |
Sbjct --gETKFMGVCQLPSKndEKEYPHRRIDIRLIpKDQY-----------------------  256
DSSP  --lLLEEEEEELLLLLllLLLLLLLEEEEEEELlHHHH-----------------------


DSSP  eellhHHHHhhhhhhhhlllllllllllHHHH-------------------hhhhhhhh
Query pcxtiPWQIaqklhavtavleepkandraHDLV-------------------dlqllegl  217
ident                               |
Sbjct ----yCGVL----------------yftgSDIFnknmrahalekgftineytirplgvtg  296
DSSP  ----hHHHH----------------hhhlLHHHhhhhhhhhhhlleeellllleeellll


DSSP  llhHHHHHhhHHHHhhhhhhlllLLLLLLlllllhhhhhhhhhllllllllllhhhhhh
Query lldADLXPtrSACIaifearaqhPWPPRVatlphwpliyagalegldhlelartvdaaaq  277
ident      |
Sbjct vagEPLPV--DSEK-----difdYIQWKY-------------------------------  318
DSSP  lllLLLLL--LLHH-----hhhhHLLLLL-------------------------------


DSSP  hhhhhhhhhhhllll
Query avqrfvaridratkr  292
ident
Sbjct -------repkdrse  326
DSSP  -------llhhhlll
```

No 106: Query=mol1A Sbjct=5bomA Z-score=7.0

back to top

```
DSSP  ------------------------------------------------------------
Query ------------------------------------------------------------    0
ident
Sbjct lnggitdmltelanfeknvsqaihkynayrkaasviakyphkiksgaeakklpgvgtkia   60
DSSP  llhhhhhhhhhhhhhhhhllllhhhhhhhhhhhhhhhhllllllllhhhhhllllllhhhh


DSSP  -----------------------------------------------llllllllllhhh
Query -----------------------------------------------aapyssppptnlrs   13
ident
Sbjct ekideflatgklrklekirqddtsssinfltrvsgigpsaarkfvdegiktledlrkned  120
DSSP  hhhhhhhhlllhhhhhhhllhhhhhhhhhlllllllhhhhhhhhhllllllhhhhhhlhh


DSSP  hhhhhhhhhhhllllhhhhhHHHHHHHHHHHHHH-LLLLllLLLLEEEEHHHHHHHHhhlll
Query lrdrltqvaerqgvvfgrlqRHVAXIVVAQFAA-TLTDdtGAPLLLVKGGSSLELrrgip   72
ident                                               |  |
Sbjct klnhhqriglkyfgdfekriPREEMLQMQDIVLnEVKKvdSEYIATVCGSFRRGA-----  175
DSSP  hllhhhhhhhhlhhhhllleEHHHHHHHHHHHHHhHHHHhlLLLEEEELLHHHHLL-----


DSSP  lllLLLLEEEEEL---------lLHHHHHHHHHHHHHhhlllleeEEELLLEEllllllL
Query dsrTSKDFATVAR---------rDIELIHEQLADAGetgwegftAIFTAPEEiaapgxpV  123
ident     | |                      |||             |
Sbjct --eSSGDMDVLLThpsftsespkLLHQVVEQLQKVH--------FITDTLSK------gE  219
DSSP  --lEELLEEEEEEllllllllllHHHHHHHHHHHHLL--------LEEEEEEE------lL
```

```
DSSP  LLEEEEEEEEELLEEEEEEEEEEEEL-LLLLllleeeeelhhhhllllllleeeeellhH
Query KPRRFTAKLSYRGRAFATVPIEVSSV-EAGNadqfdtltsdalglvgvpaavavpcxtiP  182
ident         |           |
Sbjct TKFMGVCQLPSDEKEYPHRRIDIRLIpKDQY---------------------------yC  252
DSSP  LEEEEEELLLLLLLLLLLEEEEEEELlHHHH---------------------------hH


DSSP  HHHhhhhhhhhllllllllllllHHHH-------------------hhhhhhhhllhhHH
Query WQIaqklhavtavleepkandraHDLV-------------------dlqllegllldaDL  223
ident                         |                                  |
Sbjct GVL----------------yftgSDIFnknmrahalekgftineytirplgvtgvagePL  296
DSSP  HHH----------------hhhlLHHHhhhhhhhhhhlleeellllleeellllllllLL


DSSP  HHhhHHHHHhhhhhlllLLLLLllllllhhhhhhhhhlllllllllllhhhhhhhhhhhh
Query XPtrSACIAifearaqhPWPPRvatlphwpliyagalegldhlelartvdaaaqavqrfv  283
ident
Sbjct PV--DSEKD-----ifdYIQWK--------------------------------------  311
DSSP  LL--LLHHH-----hhhHLLLL--------------------------------------


DSSP  hhhhhllll
Query aridratkr  292
ident
Sbjct yrepkdrse  320
DSSP  lllhhhlll
```

No 107: Query=mol1A Sbjct=3uxpA Z-score=7.0

back to top

```
DSSP  ------------------------------------------------------------
Query ------------------------------------------------------------    0
ident
Sbjct etlnggitdmlvelanfeknvsqaihkynayrkaasviakyphkiksgaeakklpgvgtk   60
DSSP  llllhhhhhhhhhhhhhhhhhlllllhhhhhhhhhhhhhhhllllllhhhhhllllllhh


DSSP  -------------------------------------------------llllllllllh
Query -------------------------------------------------aapysspptnl   11
ident
Sbjct iaekideflatgklrklekirqddtsssinfltrvtgigpsaarklvdegiktledlrkn  120
DSSP  hhhhhhhhhhlllllhhhhhhllhhhhhhhhhllllllhhhhhhhhhhlllllhhhhhll


DSSP  hhhhhhhhhhhhhllllhhhhhHHHHHHHHHHHHH-LLLLLLLLLLEEEEHHHHHHHhhl
Query rslrdrltqvaerqgvvfgrlqRHVAXIVVAQFAA-TLTDDTGAPLLLVKGGSSLELrrg   70
ident                                                 | |
Sbjct edklnhhqriglkyfedfekriPREEMLQMQDIVLnEVKKLDPEYIATVCGSFRRGA---  177
DSSP  hhhllhhhhhhhhlhhhhhlleEHHHHHHHHHHHHhHHHHHLLLLEEEELHHHHLLL---


DSSP  lllllLLLLEEEEEL------------lLHHHHHHHHHHHHhhlllleeEEELLLEELll
Query ipdsrTSKDFATVAR------------rDIELIHEQLADAGetgwegftAIFTAPEEIaa  118
ident       | |                         |||             |
Sbjct ----eSSGDMDVLLThpnftsesskqpkLLHRVVEQLQKVR--------FITDTLSKG--  223
DSSP  ----lEELLEEEEEElllllllllllhhHHHHHHHHHHHLL--------LEEEEEEEL--


DSSP  llllLLLEEEEEEEEELL--EEEEEEEEEEEEL-LLLLllleeeeelhhhhllllllleee
Query pgxpVKPRRFTAKLSYRG--RAFATVPIEVSSV-EAGNadqfdtltsdalglvgvpaava  175
ident              |             |
Sbjct ----ETKFMGVCQLPSENdeNEYPHRRIDIRLQpKDQY----------------------  257
DSSP  ----LLEEEEEELLLLLLllLLLLLEEEEEEEElHHHH----------------------


DSSP  eeellhHHHHhhhhhhhhllllllllllllHHHHHHH-------------------hhhh
Query vpcxtiPWQIaqklhavtavleepkandraHDLVDLQ-------------------lleg  216
ident                                |
Sbjct -----yCGVL----------------yftgSDIFNKNmrahalekgftineytirplgvt  296
DSSP  -----hHHHH----------------hhllLHHHHHHhhhllllllllllllllllllll


DSSP  hllhHHHHHhhHHHHhhhhhhlllLLLLLLlllllhhhhhhhhhlllllllllllhhhhh
Query llldADLXPtrSACIaifearaqhPWPPRVatlphwpliyagalegldhlelartvdaaa  276
ident       |                     |
```

```
Sbjct gvagEPLPV--DSEQ-----diidYIQWKY------------------------------  319
DSSP  llllLLLLL--LLHH-----hhhhHLLLLL------------------------------


DSSP  hhhhhhhhhhhhllll
Query qavqrfvaridratkr  292
ident 
Sbjct ---------repkdrs  326
DSSP  ---------lllllll
```

No 108: Query=mol1A Sbjct=4f5rB Z-score=7.0

back to top

```
DSSP  ------------------------------------------------------------
Query ------------------------------------------------------------    0
ident 
Sbjct tlnggitdmltelanfeknvsqaihkynayrkaasviakyphkiksgaeakklpgvgtki   60
DSSP  lllhhhhhhhhhhhhhhhhllllhhhhhhhhhhhhhhhlllllllhhhhhllllllhhh


DSSP  ------------------------------------------------llllllllllhh
Query ------------------------------------------------aapysspptnlr   12
ident 
Sbjct aekideflatgklrklekirqddtsssinfltrvsgigpsaarkfvdegiktledlrkne  120
DSSP  hhhhhhhhhllllhhhhhhhhlhhhhhhhhhlllllllhhhhhhhhhlllllhhhhhhlh


DSSP  hhhhhhhhhhhllllhhhhhHHHHHHHHHHHHHH-LLLLllLLLLEEEEHHHHHHHhhll
Query slrdrltqvaerqgvvfgrlqRHVAXIVVAQFAA-TLTDdtGAPLLLVKGGSSLELrrgi   71
ident                                                | |
Sbjct dklnhhqriglkyfgdfekriPREEMLQMQDIVLnEVKKvdSEYIATVCGSFRRGA----  176
DSSP  hhllhhhhhhhhlhhhhllleEHHHHHHHHHHHHHhHHHHhlLLLEEEEEHHHLLLL----


DSSP  llllLLLLEEEEEL------------lLHHHHHHHHHHHHhhlllleeEEELLLEELlll
Query pdsrTSKDFATVAR------------rDIELIHEQLADAGetgwegftAIFTAPEEIaap  119
ident      | |                         |||             |
Sbjct ---eSSGDMDVLLThpsftsestkqpkLLHQVVEQLQKVH--------FITDTLSKG---  222
DSSP  ---lEELLEEEEEEllllllllllllHHHHHHHHHHHHLL--------LEEEEEEEL---


DSSP  lllLLLEEEEEEEEEL--LEEEEEEEEEEEEL-LLLLllllleeeeelhhhhlllllleee
Query gxpVKPRRFTAKLSYR--GRAFATVPIEVSSV-EAGNadqfdtltsdalglvgvpaavav  176
ident             |             |
Sbjct ---ETKFMGVCQLPSKndEKEYPHRRIDIRLIpKDQY-----------------------  256
DSSP  ---LLEEEEEELLLLLllLLLLLLEEEEEEELlHHHH-----------------------


DSSP  eellhHHHHhhhhhhhhllllllllllllHHHHHHH--------------hhhhhllhHH
Query pcxtiPWQIaqklhavtavleepkandraHDLVDLQ--------------lleglllldAD  222
ident                               |
Sbjct ----yCGVL----------------yftgSDIFNKNmkahalekgftineytirplagEP  296
DSSP  ----hHHHH----------------hhhlLHHHHHHhhhhhhllleeellllleeellLL


DSSP  HHhhHHHHHhhhhhhllLLLLLLLlllllhhhhhhhhhllllllllllhhhhhhhhhh
Query LXptRSACIaifearaqhPWPPRVatlphwpliyagalegldhlelartvdaaaqavqrf  282
ident |
Sbjct LP--VDSEK------difDYIQWK------------------------------------  312
DSSP  LL--LLLHH------hhhHHHLLL------------------------------------


DSSP  hhhhhhllll
Query varidratkr  292
ident 
Sbjct -yrepkdrse  321
DSSP  -lllhhhhll
```

No 109: Query=mol1A Sbjct=4kllA Z-score=7.0

back to top

```
DSSP  ------------------------------------------------------------
```

```
Query                                                                   0
ident
Sbjct tlnggitdmltelanfeknvsqaihkynayrkaasviakyphkiksgaeakklpgvgtki   60
DSSP  lllhhhhhhhhhhhhhhhhllllhhhhhhhhhhhhhhhhlllllllhhhhhlllllllhhh


DSSP  ------------------------------------------------lllllllllhh
Query ------------------------------------------------aapysspptnlr   12
ident
Sbjct aekideflatgklrklekirqddtsssinfltrvsgigpsaarkfvdegiktledlrkne  120
DSSP  hhhhhhhhhhlllhhhhhhhhlhhhhhhhhhllllllhhhhhhhhhlllllhhhhhhlh


DSSP  hhhhhhhhhhhhllllhhhhhHHHHHHHHHHHHH-LLLLllLLLLEEEEHHHHHHHHhll
Query slrdrltqvaerqgvvfgrlqRHVAXIVVAQFAA-TLTDdtGAPLLLVKGGSSLELrrgi   71
ident                                                | |
Sbjct dklnhhqriglkyfgdfekriPREEMLQMQDIVLnEVKKvdSEYIATVCGSFRRGA----  176
DSSP  hhllhhhhhhhhlhhhhllleEHHHHHHHHHHHHHhHHHHhlLLLEEEEEEHHHLLLL----


DSSP  llllLLLLEEEEEL------------lLHHHHHHHHHHHHhhllllleeEEELLLEELlll
Query pdsrTSKDFATVAR------------rDIELIHEQLADAGetgwegftAIFTAPEEIaap  119
ident      | |                         |||             |
Sbjct ---eSSGDMDVLLThpsftsestkqpkLLHQVVEQLQKVH--------FITDTLSKG---  222
DSSP  ---lEELLEEEEEEllllllllllllHHHHHHHHHHHHLL--------LEEEEEEEL---


DSSP  lllLLLEEEEEEEEEL--LEEEEEEEEEEEEL-LLLLllllleeeeelhhhhllllllleee
Query gxpVKPRRFTAKLSYR--GRAFATVPIEVSSV-EAGNadqfdtltsdalglvgvpaavav  176
ident             |             |
Sbjct ---ETKFMGVCQLPSKndEKEYPHRRIDIRLIpKDQY-----------------------  256
DSSP  ---LLEEEEEEELLLLLllLLLLLLEEEEEEEELlHHHH-----------------------


DSSP  eellhHHHHhhhhhhhhllllllllllllHHHHHHHH-------------------hhhh
Query pcxtiPWQIaqklhavtavleepkandraHDLVDLQL-------------------legl  217
ident                               |
Sbjct ----yCGVL----------------yftgSDIFNKNMrahalekgftineytirplgvtg  296
DSSP  ----hHHHH----------------hhhlLHHHHHHHHhhhhhhlleeellllleeellll


DSSP  llhHHHHhhHHHHHhhhhhhllllLLLLLlllllhhhhhhhhhllllllllllhhhhhh
Query lldADLXptRSACIaifearaqhpWPPRVatlphwpliyagalegldhlelartvdaaaq  277
ident      |
Sbjct vagEPLP--VDSEK-------difDYIQW-------------------------------  316
DSSP  lllLLLL--LLLHH-------hhhHHLLL-------------------------------


DSSP  hhhhhhhhhhhllll
Query avqrfvaridratkr  292
ident
Sbjct -----kyrepkdrse  326
DSSP  -----llllhhhlll
```

No 110: Query=mol1A Sbjct=4docA Z-score=7.0

back to top

```
DSSP  ------------------------------------------------------------
Query ------------------------------------------------------------    0
ident
Sbjct tlnggitdmltelanfeknvsqaihkynayrkaasviakyphkiksgaeakklpgvgtki   60
DSSP  lllhhhhhhhhhhhhhhhhllllhhhhhhhhhhhhhhhhlllllllhhhhhlllllllhhh


DSSP  ------------------------------------------------lllllllllhh
Query ------------------------------------------------aapysspptnlr   12
ident
Sbjct aekideflatgklrklekirqddtsssinfltrvsgigpsaarkfvdegiktledlrkne  120
DSSP  hhhhhhhhhhlllhhhhhhhhlhhhhhhhhhllllllhhhhhhhhhlllllhhhhhhlh


DSSP  hhhhhhhhhhhhllllhhhhhHHHHHHHHHHHHH-LLLLllLLLLEEEEHHHHHHHHhll
Query slrdrltqvaerqgvvfgrlqRHVAXIVVAQFAA-TLTDdtGAPLLLVKGGSSLELrrgi   71
ident                                                | |
Sbjct dklnhhqriglkyfgdfekriPREEMLQMQDIVLnEVKKvdSEYIATVCGSFRRGA----  176
DSSP  hhllhhhhhhhhlhhhhllleEHHHHHHHHHHHHHhHHHHhlLLLEEEELHHHHLLL----
```

```
DSSP  llllLLLLEEEEEL------------lLHHHHHHHHHHHHhhllllleeEEELLLLEELlll
Query pdsrTSKDFATVAR------------rDIELIHEQLADAGetgwegftAIFTAPEEIaap  119
ident      | |                         |||             |
Sbjct ---eSSGDMDVLLThpsftsestkqpkLLHQVVEQLQKVH--------FITDTLSKG---  222
DSSP  ---lEELLEEEEEEllllllllllllHHHHHHHHHHHHLL--------LEEEEEEEL---


DSSP  lllLLLEEEEEEEEEL--LEEEEEEEEEEEEL-LLLLlllleeeeelhhhhlllllleee
Query gxpVKPRRFTAKLSYR--GRAFATVPIEVSSV-EAGNadqfdtltsdalglvgvpaavav  176
ident             |             |
Sbjct ---ETKFMGVCQLPSKndEKEYPHRRIDIRLIpKDQY-----------------------  256
DSSP  ---LLEEEEEEELLLLLllLLLLLLLEEEEEEEELlHHHH-----------------------


DSSP  eellhHHHHhhhhhhhhllllllllllllHHHHHHH-------------------hhhhh
Query pcxtiPWQIaqklhavtavleepkandraHDLVDLQ-------------------llegl  217
ident                               |
Sbjct ----yCGVL----------------yftgSDIFNKNmrahalekgftineytirplgvtg  296
DSSP  ----hHHHH----------------hhhlLHHHHHHhhhhhhhlleeellllleeellll


DSSP  llhHHHHhhHHHHHhhhhhhllllLLLLLlllllhhhhhhhhhllllllllllhhhhhh
Query lldADLXptRSACIaifearaqhpWPPRVatlphwpliyagalegldhlelartvdaaaq  277
ident      |
Sbjct vagEPLP--VDSEK-------difDYIQW-------------------------------  316
DSSP  lllLLLL--LLLHH-------hhhHHLLL-------------------------------


DSSP  hhhhhhhhhhhllll
Query avqrfvaridratkr  292
ident
Sbjct -----kyrepkdrse  326
DSSP  -----llllhhhlll
```

No 111: Query=mol1A Sbjct=1bpyA Z-score=7.0

back to top

```
DSSP  ------------------------------------------------------------
Query ------------------------------------------------------------    0
ident
Sbjct tlnggitdmltelanfeknvsqaihkynayrkaasviakyphkiksgaeakklpgvgtki   60
DSSP  lllhhhhhhhhhhhhhhhhllllhhhhhhhhhhhhhhhhlllllllhhhhhlllllhhh


DSSP  ------------------------------------------------llllllllllhh
Query ------------------------------------------------aapysspptnlr   12
ident
Sbjct aekideflatgklrklekirqddtsssinfltrvsgigpsaarkfvdegiktledlrkne  120
DSSP  hhhhhhhhhhlllhhhhhhhhlhhhhhhhhhlllllllhhhhhhhhhlllllhhhhhllh


DSSP  hhhhhhhhhhhllllhhhhhHHHHHHHHHHHHH-LLLLllLLLLEEEEHHHHHHHHhhll
Query slrdrltqvaerqgvvfgrlqRHVAXIVVAQFAA-TLTDdtGAPLLLVKGGSSLELrrgi   71
ident                                                | |
Sbjct dklnhhqriglkyfgdfekriPREEMLQMQDIVLnEVKKvdSEYIATVCGSFRRGA----  176
DSSP  hhllhhhhhhhlllllllleEHHHHHHHHHHHHHhHHHHhlLLLEEEEELHHHHLLL----


DSSP  llllLLLLEEEEEL------------lLHHHHHHHHHHHHhhllllleeEEELLLLEELlll
Query pdsrTSKDFATVAR------------rDIELIHEQLADAGetgwegftAIFTAPEEIaap  119
ident      | |                         |||             |
Sbjct ---eSSGDMDVLLThpsftsestkqpkLLHQVVEQLQKVH--------FITDTLSKG---  222
DSSP  ---lEELLEEEEEEllllllllllhhHHHHHHHHHHHLLL--------LEEEEEEEL---


DSSP  lllLLLEEEEEEEEEL--LEEEEEEEEEEEEL-LLLLlllleeeeelhhhhlllllleee
Query gxpVKPRRFTAKLSYR--GRAFATVPIEVSSV-EAGNadqfdtltsdalglvgvpaavav  176
ident             |             |
Sbjct ---ETKFMGVCQLPSKndEKEYPHRRIDIRLIpKDQY-----------------------  256
DSSP  ---LLEEEEEEELLLLLllLLLLLLLEEEEEEEELlHHHH-----------------------


DSSP  eellhHHHHhhhhhhhhllllllllllllHHHHHHHH-------------------hhhh
Query pcxtiPWQIaqklhavtavleepkandraHDLVDLQL-------------------legl  217
```

```
ident                                |
Sbjct ----yCGVL----------------yftgSDIFNKNMrahalekgftineytirplgvtg  296
DSSP  ----hHHHH----------------hhhlLHHHHHHHhhhhhhlleeellllleeelllll


DSSP  llhHHHHhhHHHHHhhhhhhllllLLLLLlllllhhhhhhhhhlllllllllllhhhhhh
Query lldADLXptRSACIaifearaqhpWPPRVatlphwpliyagalegldhlelartvdaaaq  277
ident      |
Sbjct vagEPLP--VDSEK-------difDYIQW-------------------------------  316
DSSP  lllLLLL--LLLLH-------hhhHHHLL-------------------------------


DSSP  hhhhhhhhhhhllll
Query avqrfvaridratkr  292
ident
Sbjct -----kyrepkdrse  326
DSSP  -----llllhhhlll
```

No 112: Query=mol1A Sbjct=4klpA Z-score=7.0

back to top

```
DSSP  ------------------------------------------------------------
Query ------------------------------------------------------------    0
ident
Sbjct tlnggitdmltelanfeknvsqaihkynayrkaasviakyphkiksgaeakklpgvgtki   60
DSSP  lllhhhhhhhhhhhhhhhhllllhhhhhhhhhhhhhhhhlllllllhhhhhhllllllhhh


DSSP  ------------------------------------------------llllllllllhh
Query ------------------------------------------------aapysspptnlr   12
ident
Sbjct aekideflatgklrklekirqddtsssinfltrvsgigpsaarkfvdegiktledlrkne  120
DSSP  hhhhhhhhhhlllhhhhhhhhlhhhhhhhhhllllllhhhhhhhhhlllllhhhhhhhlh


DSSP  hhhhhhhhhhhhllllhhhhhHHHHHHHHHHHHHH-LLLLllLLLLEEEEHHHHHHHhhll
Query slrdrltqvaerqgvvfgrlqRHVAXIVVAQFAA-TLTDdtGAPLLLVKGGSSLELrrgi   71
ident                                                | |
Sbjct dklnhhqriglkyfgdfekriPREEMLQMQDIVLnEVKKvdSEYIATVCGSFRRGA----  176
DSSP  hhllhhhhhhhhlhhhhhlleEHHHHHHHHHHHHhHHHHhlLLLEEEEEHHHLLLL----


DSSP  llllLLLLEEEEEL------------lLHHHHHHHHHHHHhhlllleeEEELLLEELlll
Query pdsrTSKDFATVAR------------rDIELIHEQLADAGetgwegftAIFTAPEEIaap  119
ident      | |                         |||             |
Sbjct ---eSSGDMDVLLThpsftsestkqpkLLHQVVEQLQKVH--------FITDTLSKG---  222
DSSP  ---lEELLEEEEEElllllllllllllHHHHHHHHHHHLL--------LEEEEEEEL---


DSSP  lllLLLEEEEEEEEEELLEEEEEEEEEEEL-LLLLlllleeeeelhhhhlllllleeeee
Query gxpVKPRRFTAKLSYRGRAFATVPIEVSSV-EAGNadqfdtltsdalglvgvpaavavpc  178
ident             |           |
Sbjct ---ETKFMGVCQLPSKEKEYPHRRIDIRLIpKDQY-------------------------  254
DSSP  ---LLEEEEEEELLLLLLLLLLEEEEEEELlHHHH-------------------------


DSSP  llhHHHHhhhhhhhhllllllllllllHHHHHHH-------------------hhhhhll
Query xtiPWQIaqklhavtavleepkandraHDLVDLQ-------------------lleglll  219
ident                             |
Sbjct --yCGVL----------------yftgSDIFNKNmrahalekgftineytirplgvtgva  296
DSSP  --hHHHH----------------hhhlLHHHHHHHhhhhhhlleeellllleeellllllll


DSSP  hHHHHhhHHHHHhhhhhhllllLLLLLlllllhhhhhhhhhlllllllllllhhhhhhhh
Query dADLXptRSACIaifearaqhpWPPRVatlphwpliyagalegldhlelartvdaaaqav  279
ident    |
Sbjct gEPLP--VDSEK-------difDYIQW---------------------------------  314
DSSP  lLLLL--LLLHH-------hhhHHHLL---------------------------------


DSSP  hhhhhhhhhllll
Query qrfvaridratkr  292
ident
Sbjct ---kyrepkdrse  324
DSSP  ---llllhhhlll
```

No 113: Query=mol1A Sbjct=3uxoB Z-score=7.0

back to top

```
DSSP  ------------------------------------------------------------
Query ------------------------------------------------------------     0
ident 
Sbjct tlnggitdmlvelanfeknvsqaihkynayrkaasviakyphkiksgaeakklpgvgtki    60
DSSP  lllhhhhhhhhhhhhhhhhllllhhhhhhhhhhhhhhhllllllllhhhhhlllllllhhh


DSSP  ------------------------------------------------llllllllllhh
Query ------------------------------------------------aapysspptnlr    12
ident 
Sbjct aekideflatgklrklekirqddtsssinfltrvtgigpsaarklvdegiktledlrkne   120
DSSP  hhhhhhhhhhllllllllhhhhllllllllllllllllllllhhhhllllllllllllll


DSSP  hhhhhhhhhhhhllllhhhhhHHHHHHHHHHHHH-LLLLllLLLLEEEEHHHHHHHhhll
Query slrdrltqvaerqgvvfgrlqRHVAXIVVAQFAA-TLTDdtGAPLLLVKGGSSLELrrgi    71
ident                                                |  |
Sbjct dklnhhqriglkyfedfekriPREEMLQMQDIVLnEVKKldPEYIATVCGSFRRGA----   176
DSSP  lllllllhhhhhlhhhhllleEHHHHHHHHHHHHhHHHHhlLLLEEEELHHHHLLL----


DSSP  llllLLLLEEEEEL------------lLHHHHHHHHHHHHhhlllleeEEELLLEELlll
Query pdsrTSKDFATVAR------------rDIELIHEQLADAGetgwegftAIFTAPEEIaap   119
ident      | |                         |||              |
Sbjct ---eSSGDMDVLLThpnftsesskqpkLLHRVVEQLQKVR--------FITDTLSKG---   222
DSSP  ---lEELLEEEEEEllllllllllllhHHHHHHHHHHHLL--------LEEEEEEEL---


DSSP  lllLLLEEEEEEEEELLE--EEEEEEEEEEEL-LLLLllllleeeeelhhhhllllllleee
Query gxpVKPRRFTAKLSYRGR--AFATVPIEVSSV-EAGNadqfdtltsdalglvgvpaavav   176
ident             |             |
Sbjct ---ETKFMGVCQLPSENDenEYPHRRIDIRLQpKDQY-----------------------   256
DSSP  ---LLEEEEEEELLLLLLllLLLLEEEEEEELlHHHH-----------------------


DSSP  eellhHHHHhhhhhhhhllllllllllllHHHHHHH-------------------hhhhh
Query pcxtiPWQIaqklhavtavleepkandraHDLVDLQ-------------------llegl   217
ident                               |
Sbjct ----yCGVL----------------yftgSDIFNKNmrahalekgftineytirplgvtg   296
DSSP  ----hHHHH----------------hhhlLHHHHHHhhhhhhhlleeellllleeelllll


DSSP  llhHHHHhhHHHHHhhhhhhllllLLLLLlllllhhhhhhhhhlllllllllllhhhhhh
Query lldADLXptRSACIaifearaqhpWPPRVatlphwpliyagalegldhlelartvdaaaq   277
ident      |
Sbjct vagEPLP--VDSEQ-------difDYIQW-------------------------------   316
DSSP  lllLLLL--LLLHH-------hhhHHLLL-------------------------------


DSSP  hhhhhhhhhhhllll
Query avqrfvaridratkr  292
ident 
Sbjct ------ryrepkdrs  325
DSSP  ------llllhhhll
```

No 114: Query=mol1A Sbjct=3k75E Z-score=7.0

back to top

```
DSSP  -------------------------llllllllllhhhhhhhhhhhhhhllllhhhhhHH
Query -------------------------aapysspptnlrslrdrltqvaerqgvvfgrlqRH    35
ident 
Sbjct tsssinfltrvtgigpsaarklvdegiktledlrknedklnhhqriglkyfedfekriPR    60
DSSP  lhhhhhhhlllllllhhhhhhhhhllllllhhhhhhlhhhllhhhhhhhhllllllleEH


DSSP  HHHHHHHHHHH-LLLLllLLLLEEEEHHHHHHHhhllllllLLLLEEEEEL---------
Query VAXIVVAQFAA-TLTDdtGAPLLLVKGGSSLELrrgipdsrTSKDFATVAR---------    85
ident                         | |               | |
Sbjct EEMLQMQDIVLnEVKKldPEYIATVCGSFRRGA-------eSSGDMDVLLThpnftsess   113
```

```
DSSP  hhhhhhhhhhhhhhhhlLLLEEEEELLhhhhLL-------lEELLEEEEEEEllllllll

DSSP  ---lLHHHHHHHHHHHHhhlllleeEEELLLEELllllllLLLEEEEEEEEEL--LEEEE
Query ---rDIELIHEQLADAGetgwegftAIFTAPEEIaapgxpVKPRRFTAKLSYR--GRAFA  140
ident           |||             |                      |
Sbjct kqpkLLHRVVEQLQKVR--------FITDTLSKG------ETKFMGVCQLPSEndENEYP  159
DSSP  llhhHHHHHHHHHHHLL--------LEEEEEEEL------LLEEEEEEEELLLLlLLLLL


DSSP  EEEEEEEEL-LLLLlllleeeeelhhhhlllllleeeeellhHHHHhhhhhhhhllllll
Query TVPIEVSSV-EAGNadqfdtltsdalglvgvpaavavpcxtiPWQIaqklhavtavleep  199
ident    |
Sbjct HRRIDIRLIpKDQY---------------------------yCGVL--------------  178
DSSP  EEEEEEEELlHHHH---------------------------hHHHH--------------


DSSP  llllllhhhhHHHHHH----------------------hhllhhhhhhhhHHHHhhhhhh
Query kandrahdlvDLQLLE----------------------glllldadlxptrSACIaifear  237
ident                                                   |
Sbjct ------yftgSDIFNKnmrahalekgftineytirplgvtgvageplpvdSEQD------  226
DSSP  ------hhhlLHHHHhhhhhhhhhlleeeelleeeellllllllllllllLHHH------


DSSP  llllLLLLLlllllhhhhhhhhhhllllllllllhhhhhhhhhhhhhhhhhllll
Query aqhpWPPRVatlphwpliyagalegldhlelartvdaaaqavqrfvaridratkr  292
ident
Sbjct -ifdYIQWR-------------------------------------yrepkdrse  243
DSSP  -hhhHLLLL-------------------------------------lllhhhlll
```

No 115: Query=mol1A Sbjct=2p66A Z-score=7.0

back to top

```
DSSP  ------------------------------------------------------------
Query ------------------------------------------------------------    0
ident
Sbjct pqetlnggitdmltelanfeknvsqaihkynayrkaasviakyphkiksgaeakklpgvg   60
DSSP  llhhhlhhhhhhhhhhhhhhhhllllhhhhhhhhhhhhhhhhhlllllllhhhhhlllll


DSSP  ---------------------------------------------------lllllllll
Query ---------------------------------------------------aapyssppt    9
ident
Sbjct tkiaekideflatgklrklekirqddtsssinfltrvsgigpsaarkfvdegiktledlr  120
DSSP  hhhhhhhhhhhhlllhhhhhhhhhlllhhhhhhhlllllllhhhhhhhhhlllllhhhhh


DSSP  lhhhhhhhhhhhhhhllllhhhhhHHHHHHHHHHHHH-LLLLllLLLLEEEEHHHHHHHh
Query nlrslrdrltqvaerqggvvfgrlqRHVAXIVVAQFAA-TLTDdtGAPLLLVKGGSSLELr   68
ident                                                   | |
Sbjct knedklnhhqriglkyfgdfekriPREEMLQMQDIVLnEVKKvdSEYIATVCGSFRRGA-  179
DSSP  hlhhhllhhhhhhhhlhhhhhlleEHHHHHHHHHHHHhHHHHhlLLLEEEELLLLLLLL-


DSSP  hllllllLLLLEEEEEL---------lLHHHHHHHHHHHHhhlllleeEEELLLEELlll
Query rgipdsrTSKDFATVAR---------rDIELIHEQLADAGetgwegftAIFTAPEEIaap  119
ident         | |                      |||             |
Sbjct ------eSSGDMDVLLThpsftsespkLLHQVVEQLQKVH--------FITDTLSKG---  222
DSSP  ------lEELLEEEEEElllllllllhHHHHHHHHHHHLL--------LEEEEEEEL---


DSSP  lllLLLEEEEEEEEEL--LEEEEEEEEEEEEL-LLLLlllleeeeelhhhhlllllleee
Query gxpVKPRRFTAKLSYR--GRAFATVPIEVSSV-EAGNadqfdtltsdalglvgvpaavav  176
ident             |             |
Sbjct ---ETKFMGVCQLPSKndEKEYPHRRIDIRLIpKDQY-----------------------  256
DSSP  ---LLEEEEEEELLLLlLLLLLLEEEEEEEELlLLLH-----------------------


DSSP  eellhHHHHhhhhhhhhllllllllllllhhhhHHHHHH---------------------
Query pcxtiPWQIaqklhavtavleepkandrahdlvDLQLLE---------------------  215
ident
Sbjct ----yCGVL--------------------yftgSDIFNKnmrahalekgftineytirpl  292
DSSP  ----hHHHH--------------------hlllLHHHHhhhhhhhllleeellllleeel


DSSP  -hhllhhhhhhhhHHHHhhhhhhlllLLLLLLlllllhhhhhhhhhlllllllllllhhh
```

```
Query [illegible]
ident              |
Sbjct gvtgvageplpvdSEKD-------ifDYIQWK----------------------------  317
DSSP  lllllllllllllLHHH-------hhHHLLLL----------------------------


DSSP  hhhhhhhhhhhhhhllll
Query aaqavqrfvaridratkr  292
ident
Sbjct ---------yrepkdrse  326
DSSP  ---------lllhhhlll
```

No 116: Query=mol1A Sbjct=9icsA Z-score=7.0

back to top

```
DSSP  ------------------------------------------------------------
Query ------------------------------------------------------------    0
ident
Sbjct etlnggitdmltelanfeknvsqaihkynayrkaasviakyphkiksgaeakklpgvgtk   60
DSSP  llllhhhhhhhhhhhhhhhhllllhhhhhhhhhhhhhhhhllllllhhhhhllllllhh


DSSP  -------------------------------------------------llllllllllh
Query -------------------------------------------------aapysspptnl   11
ident
Sbjct iaekideflatgklrklekirqddtsssinfltrvsgigpsaarkfvdegiktledlrkn  120
DSSP  hhhhhhhhhhllllhhhhhhhhlhhhhhhhhhllllllhhhhhhhhlllllhhhhhhl


DSSP  hhhhhhhhhhhhhllllhhhhhHHHHHHHHHHHHH-LLLLllLLLLLEEEEHHHHHHHHhhl
Query rslrdrltqvaerqgvvfgrlqRHVAXIVVAQFAA-TLTDdtGAPLLLVKGGSSLELrrg   70
ident                                                 | |
Sbjct edklnhhqriglkyfgdfekriPREEMLQMQDIVLnEVKKvdSEYIATVCGSFRRGA---  177
DSSP  hhhllhhhhhhhhlhhhhhlleEHHHHHHHHHHHHhHHHHhlLLLEEEELLLLLLL---


DSSP  lllllLLLLEEEEEL------------lLHHHHHHHHHHHHhhlllleeEEELLLEELll
Query ipdsrTSKDFATVAR------------rDIELIHEQLADAGetgwegftAIFTAPEEIaa  118
ident       | |                         |||             |
Sbjct ----eSSGDMDVLLThpsftsestkqpkLLHQVVEQLQKVH--------FITDTLSKG--  223
DSSP  ----lEELLEEEEEEellllllllllhhHHHHHHHHHHHHLL--------LEEEEEEEL--


DSSP  llllLLLEEEEEEEEELL--EEEEEEEEEEEEL-LLLLlllleeeeelhhhhllllllee
Query pgxpVKPRRFTAKLSYRG--RAFATVPIEVSSV-EAGNadqfdtltsdalglvgvpaava  175
ident              |             |
Sbjct ----ETKFMGVCQLPSKNdeKEYPHRRIDIRLIpKDQY----------------------  257
DSSP  ----LLEEEEEELLLLLllLLLLLLEEEEEEEEeHHHH----------------------


DSSP  eeellhHHHHhhhhhhhhlllllllllllhhhhHHHHHH--------------------
Query vpcxtiPWQIaqklhavtavleepkandrahdlvDLQLLE--------------------  215
ident
Sbjct -----yCGVL--------------------yftgSDIFNKnmrahalekgftineytirp  292
DSSP  -----hHHHH--------------------hhllLHHHHHhhhhhhhhllllllllll


DSSP  -hhllhhhhhhhHHHHHHhhhhhllllLLLLLlllllhhhhhhhhhllllllllllhhh
Query -glllldadlxptRSACIAifearaqhpWPPRVatlphwpliyagalegldhlelartvda  274
ident
Sbjct lgvtgvageplpVDSEKD------ifdYIQWK----------------------------  318
DSSP  lllllllllllLLLHHH------hhhHLLLL----------------------------


DSSP  hhhhhhhhhhhhhhllll
Query aaqavqrfvaridratkr  292
ident
Sbjct ---------yrepkdrse  327
DSSP  ---------llllhhhll
```

No 117: Query=mol1A Sbjct=3gdxA Z-score=7.0

back to top

```
DSSP  ------------------------------------------------------------
Query ------------------------------------------------------------    0
ident 
Sbjct tlnggitdmltelanfeknvsqaihkynayrkaasviakyphkiksgaeakklpgvgtki   60
DSSP  lllhhhhhhhhhhhhhhhhllllhhhhhhhhhhhhhhhllllllllhhhhhlllllllhhh


DSSP  ------------------------------------------------llllllllllhh
Query ------------------------------------------------aapysspptnlr   12
ident 
Sbjct aekideflatgklrklekirqddtsssinfltrvsgigpsaarkfvdegiktledlrkne  120
DSSP  hhhhhhhhhhlllhhhhhhhhlhhhhhhhhhlllllllhhhhhhhhllllllhhhhhhlh


DSSP  hhhhhhhhhhhhllllhHHHHHHHHHHHHHHHHHL-LLLLlLLLLEEEEHHHHHHHhhll
Query slrdrltqvaerqgvvfGRLQRHVAXIVVAQFAAT-LTDDtGAPLLLVKGGSSLELrrgi   71
ident                                                |  |
Sbjct dklnhhqriglkyfgdfEKRIPREEMLQMQDIVLNeVKKVdSEYIATVCGSFRRGA----  176
DSSP  hhllhhhhhhhhlhhhhHLLEEHHHHHHHHHHHHHhHHHHlLLLEEEELHHHHLLL----


DSSP  llllLLLLEEEEEL------------lLHHHHHHHHHHHHhhlllleeEEELLLEELlll
Query pdsrTSKDFATVAR------------rDIELIHEQLADAGetgwegftAIFTAPEEIaap  119
ident      | |                         |||              |
Sbjct ---eSSGDMDVLLThpsftsestkqpkLLHQVVEQLQKVH--------FITDTLSKG---  222
DSSP  ---lEELLEEEEEEllleelleellllHHHHHHHHHHHLL--------LEEEEEEEL---


DSSP  lllLLLEEEEEEEEEL--LEEEEEEEEEEEEL-LLLLlllleeeeelhhhhlllllleee
Query gxpVKPRRFTAKLSYR--GRAFATVPIEVSSV-EAGNadqfdtltsdalglvgvpaavav  176
ident             |             |
Sbjct ---ETKFMGVCQLPSKndEKEYPHRRIDIRLIpKDQY-----------------------  256
DSSP  ---LLEEEEEELLLLLllLLLLLLEEEEEEELlHHHH-----------------------


DSSP  eellhHHHHhhhhhhhhhlllllllllllHHHHHHH-------------------hhhhh
Query pcxtiPWQIaqklhavtavleepkandraHDLVDLQ-------------------llegl  217
ident                               |
Sbjct ----yCGVL----------------yftgSDIFNKNmrahalekgftineytirplgvtg  296
DSSP  ----hHHHH----------------hhhlLHHHHHHhhhhhhhlleeelllleeelllll


DSSP  llhHHHHhhHHHHHhhhhhhllllLLLLLLllllhhhhhhhhhlllllllllllhhhhhh
Query lldADLXptRSACIaifearaqhpWPPRVAtlphwpliyagalegldhlelartvdaaaq  277
ident      |
Sbjct vagEPLP--VDSEK-------difDYIQWK------------------------------  317
DSSP  lllLLLL--LLLHH-------hhhHHLLLL------------------------------


DSSP  hhhhhhhhhhhllll
Query avqrfvaridratkr  292
ident 
Sbjct ------yrepkdrse  326
DSSP  ------lllhhhlll
```

No 118: Query=mol1A Sbjct=3c2lA Z-score=7.0

back to top

```
DSSP  ------------------------------------------------------------
Query ------------------------------------------------------------    0
ident 
Sbjct tlnggitdmltelanfeknvsqaihkynayrkaasviakyphkiksgaeakklpgvgtki   60
DSSP  lllhhhhhhhhhhhhhhhhllllhhhhhhhhhhhhhhlllllllllhhhhhlllllllhhh


DSSP  ------------------------------------------------llllllllllhh
Query ------------------------------------------------aapysspptnlr   12
ident 
Sbjct aekideflatgklrklekirqddtsssinfltrvsgigpsaarkfvdegiktledlrkne  120
DSSP  hhhhhhhhhllllhhhhhhhhlhhhhhhhhhlllllllhhhhhhhhhlllllhhhhhhlh


DSSP  hhhhhhhhhhhhllllhhhhhHHHHHHHHHHHHH-LLLLllLLLLEEEEHHHHHHHhhll
Query slrdrltqvaerqgvvfgrlqRHVAXIVVAQFAA-TLTDdtGAPLLLVKGGSSLELrrgi   71
```

```
ident                                                  | |
Sbjct dklnhhqriglkyfgdfekriPREEMLQMQDIVLnEVKKvdSEYIATVCGSFRRGA----  176
DSSP  hhllhhhhhhhhlhhhhhlleEHHHHHHHHHHHHhHHHLllLLLLLEEEHHHLLLL----


DSSP  llllLLLLEEEEEL------------lLHHHHHHHHHHHHhhlllleeEEELLLEELlll
Query pdsrTSKDFATVAR------------rDIELIHEQLADAGetgwegftAIFTAPEEIaap  119
ident      | |                         |||             |
Sbjct ---eSSGDMDVLLThpsftsestkqpkLLHQVVEQLQKVH--------FITDTLSKG---  222
DSSP  ---lEELLEEEEELlllllllllllllHHHHHHHHHHHLL--------LEEEEEEEL---


DSSP  lllLLLEEEEEEEEEL--LEEEEEEEEEEEEL-LLLLlllleeeeelhhhhlllllleee
Query gxpVKPRRFTAKLSYR--GRAFATVPIEVSSV-EAGNadqfdtltsdalglvgvpaavav  176
ident             |             |
Sbjct ---ETKFMGVCQLPSKndEKEYPHRRIDIRLIpKDQY-----------------------  256
DSSP  ---LLEEEEEEELLLLllLLLLLLEEEEEEELlHHHH-----------------------


DSSP  eellhHHHHhhhhhhhhllllllllllllhhhhHHHHHHHHL------lHHHH-------
Query pcxtiPWQIaqklhavtavleepkandrahdlvDLQLLEGLL------lDADL-------  223
ident
Sbjct ----yCGVL--------------------yftgSDIFNKNMRahalekgFTINeytirpl  292
DSSP  ----hHHHH--------------------hlllLHHHHHHHHhhhhhllEEELllleeel


DSSP  --------hhhHHHHHhhhhhhllllLLLLLlllllhhhhhhhhhllllllllllllhhhh
Query --------xptRSACIaifearaqhpWPPRVatlphwpliyagalegldhlelartvdaa  275
ident
Sbjct gvtgvageplpVDSEK-------difDYIQW-----------------------------  316
DSSP  lllllllllllLLLHH-------hhhHHLLL-----------------------------


DSSP  hhhhhhhhhhhhhllll
Query aqavqrfvaridratkr  292
ident
Sbjct -------kyrepkdrse  326
DSSP  -------llllhhhlll
```

No 119: Query=mol1A Sbjct=1rplA Z-score=7.0

back to top

```
DSSP  -----------------------llllllllllhhhhhhhhhhhhhhllllhhhhhHHHH
Query -----------------------aapysspptnlrslrdrltqvaerqgvvfgrlqRHVA   37
ident
Sbjct ssinfltrvtgigpsaarklvdegiktledlrknedklnhhqriglkyfedfekriPREE   60
DSSP  lhhhhhhllllllhhhhhhhhhlllllhhhhlllhhhllhhhhhhhhlllhhhlleEHHH


DSSP  HHHHHHHHH-LLLLllLLLLEEEEHHHHHHHhhllllllLLLLEEEEEL-----------
Query XIVVAQFAA-TLTDdtGAPLLLVKGGSSLELrrgipdsrTSKDFATVAR-----------   85
ident                       | |               | |
Sbjct MLQMQDIVLnEVKKldPEYIATVCGSFRRGA-------eSSGDMDVLLThpnftsesskq  113
DSSP  HHHHHHHHhHHHHhlLLLEEEELLLLLLLLL-------lEELLEEEEEllllllllllll


DSSP  -lLHHHHHHHHHHHHhhlllleeEEELLLEELllllllLLLEEEEEEEEEL--LEEEEEE
Query -rDIELIHEQLADAGetgwegftAIFTAPEEIaapgxpVKPRRFTAKLSYR--GRAFATV  142
ident         |||             |                      |
Sbjct pkLLHRVVEQLQKVR--------FITDTLSKG------ETKFMGVCQLPSEndENEYPHR  159
DSSP  hhHHHHHHHHHHHLL--------LEEEEEEEL------LLEEEEEEELLLLllLLLLLLE


DSSP  EEEEEEL-LLLLlllleeeeelhhhhlllllleeeeellhHHHHhhhhhhhhllllllll
Query PIEVSSV-EAGNadqfdtltsdalglvgvpaavavpcxtiPWQIaqklhavtavleepka  201
ident  |
Sbjct RIDIRLIpKDQY---------------------------yCGVL----------------  176
DSSP  EEEEEELlHHHH---------------------------hHHHH----------------


DSSP  llllhhhhHHHHHH----------------------hhllhhhhhhhhHHHHhhhhhhll
Query ndrahdlvDLQLLE----------------------gllldadlxptrSACIaifearaq  239
ident                                                 |
Sbjct ----yftgSDIFNKnmrahalekgftineytirplgvtgvageplpvdSEQD-------i  225
DSSP  ----hhhlLHHHHHhhhhhhhhlleeelllleeellllllllllllllLHHH-------h
```

```
DSSP  llLLLLLlllllhhhhhhhhhllllllllllhhhhhhhhhhhhhhhhhllll
Query hpWPPRVatlphwpliyagalegldhlelartvdaaaqavqrfvaridratkr  292
ident
Sbjct fdYIQWR------------------------------------yrepkdrse  241
DSSP  hhHLLLL------------------------------------lllhhhlll
```

No 120: Query=mol1A Sbjct=1bpdA Z-score=7.0

back to top

```
DSSP  ------------------------------------------------------------
Query ------------------------------------------------------------    0
ident
Sbjct etlnggitdmlvelanfeknvsqaihkynayrkaasviakyphkiksgaeakklpgvgtk   60
DSSP  llllhhhhhhhhhhhhhhhhhlllhhhhhhhhhhhhhhhhlllllllhhhhhhlllllhh


DSSP  -------------------------------------------------llllllllllh
Query -------------------------------------------------aapysspptnl   11
ident
Sbjct iaekideflatgklrklekirqddtsssinfltrvtgigpsaarklvdegiktledlrkn  120
DSSP  hhhhhhhhhhllllhhhhhhhhhllhhhhhhhhhhllllllhhhhhhhhhllllllhhhhlll


DSSP  hhhhhhhhhhhhhllllhHHHHHHHHHHHHHHHHH-LLLLllLLLLLEEEEHHHHHHHhhl
Query rslrdrltqvaerqgvvfGRLQRHVAXIVVAQFAA-TLTDdtGAPLLLVKGGSSLELrrg   70
ident                                                  | |
Sbjct edklnhhqriglkyfedfEKRIPREEMLQMQDIVLnEVKKldPEYIATVCGSFRRGA---  177
DSSP  hhhllhhhhhhhhhhhhhLLLLLHHHHHHHHHHHHhHHHHhlLLLEEEELLLLLLL---


DSSP  lllllLLLLEEEEEL------------lLHHHHHHHHHHHHHhhlllleeEEELLLEELll
Query ipdsrTSKDFATVAR------------rDIELIHEQLADAGetgwegftAIFTAPEEIaa  118
ident       | |                         |||             |
Sbjct ----eSSGDMDVLLThpnftsesskqpkLLHRVVEQLQKVR--------FITDTLSKG--  223
DSSP  ----lLLLLEEEEEElllllllllllhHHHHHHHHHHHHLL--------LEEEEEEEL--


DSSP  llllLLLEEEEEEEEEELLeEEEEEEEEEEEL-LLLLlllleeeeelhhhhllllllleeee
Query pgxpVKPRRFTAKLSYRGrAFATVPIEVSSV-EAGNadqfdtltsdalglvgvpaavavp  177
ident              |           |
Sbjct ----ETKFMGVCQLPSEN-EYPHRRIDIRLIpKDQY------------------------  254
DSSP  ----LLEEEEEEEELLLLL-LLLEEEEEEEEELlLLLH------------------------


DSSP  ellhHHHHhhhhhhhhllllllllllllhhhhHHHHHH----------------------
Query cxtiPWQIaqklhavtavleepkandrahdlvDLQLLE----------------------  215
ident
Sbjct ---yCGVL--------------------yftgSDIFNKnmrahalekgftineytirplg  291
DSSP  ---hHHHH--------------------hhhlLHHHHHhhhhhhhhlleeeelleeeell


DSSP  hhllhhhhhhhhHHHHhhhhhhllllLLLLLlllllhhhhhhhhhllllllllllllhhhh
Query glllddadlxptrSACIaifearaqhpWPPRVatlphwpliyagalegldhlelartvdaa  275
ident             |
Sbjct vtgvageplpvdSEQD-------ifdYIQWR-----------------------------  315
DSSP  lllllllllllLHHH-------hhhHLLLL-----------------------------


DSSP  hhhhhhhhhhhhhllll
Query aqavqrfvaridratkr  292
ident
Sbjct --------yrepkdrse  324
DSSP  --------lllllllll
```

No 121: Query=mol1A Sbjct=4rq7A Z-score=7.0

back to top

```
DSSP  ------------------------------------------------------------
Query ------------------------------------------------------------    0
ident
Sbjct tlnggitdmltelanfeknvsqaihkynayrkaasviakyphkiksgaeakklpgvgtki   60
```

```
DSSP  [illegible]


DSSP  ------------------------------------------------llllllllllhh
Query ------------------------------------------------aapyssspptnlr   12
ident 
Sbjct aekideflatgklrklekirqddtsssinfltrvsgigpsaarkfvdegiktledlrkne  120
DSSP  hhhhhhhhhhlllhhhhhhhhlhhhhhhhhhhllllllhhhhhhhhhlllllhhhhhhlh


DSSP  hhhhhhhhhhhhllllhhhhhHHHHHHHHHHHHH-LLLLllLLLLEEEEHHHHHHHHhhll
Query slrdrltqvaerqgvvfgrlqRHVAXIVVAQFAA-TLTDdtGAPLLLVKGGSSLELrrgi   71
ident                                                 | |
Sbjct dklnhhqriglkyfgdfekriPREEMLQMQDIVLnEVKKvdSEYIATVCGSFRRGA----  176
DSSP  hhllhhhhhhhhlhhhhllleEHHHHHHHHHHHHhHHHHhlLLLEEEEELLLLLLLL----


DSSP  llllLLLLEEEEEL------------lLHHHHHHHHHHHHhhllllleeEEELLLEEllll
Query pdsrTSKDFATVAR------------rDIELIHEQLADAGetgwegftAIFTAPEEiaap  119
ident      | |                         |||             |
Sbjct ---eSSGDMDVLLThpsftsestkqpkLLHQVVEQLQKVH--------FITDTLSK----  221
DSSP  ---lEELLEEEEEElllllllllllllHHHHHHHHHHHLL--------LEEEEEEE----


DSSP  lllLLLEEEEEEEEEL--LEEEEEEEEEEEEL-LLLLllllleeeeelhhhhlllllleee
Query gxpVKPRRFTAKLSYR--GRAFATVPIEVSSV-EAGNadqfdtltsdalglvgvpaavav  176
ident             |             |
Sbjct --gETKFMGVCQLPSKndEKEYPHRRIDIRLIpKDQY-----------------------  256
DSSP  --lLLEEEEEEELLLLllLLLLLLEEEEEEEELlHHHH-----------------------


DSSP  eellhHHHHhhhhhhhhllllllllllHHHH-------------------hhhhhhhh
Query pcxtiPWQIaqklhavtavleepkandraHDLV-------------------dlqllegl  217
ident                               |
Sbjct ----yCGVL-----------------yftgSDIFnknmrahalekgftineytirplgvtg  296
DSSP  ----hHHHH-----------------hhhlLHHHhhhhhhhhhllleeellllleeellll


DSSP  llhhHHHHhhHHHHhhhhhhlllLLLLLllllllhhhhhhhhhllllllllllhhhhhh
Query lldaDLXPtrSACIaifearaqhPWPPRvatlphwpliyagalegldhlelartvdaaaq  277
ident      |
Sbjct vagePLPV--DSEK-----difdYIQWK--------------------------------  317
DSSP  llllLLLL--LLHH-----hhhhHLLLL--------------------------------


DSSP  hhhhhhhhhhhllll
Query avqrfvaridratkr  292
ident 
Sbjct -------yrepkdrs  325
DSSP  -------lllhhhll
```

No 122: Query=mol1A Sbjct=4m9jA Z-score=7.0

[back to top]

```
DSSP  ------------------------------------------------------------
Query ------------------------------------------------------------    0
ident 
Sbjct tlnggitdmltelanfeknvsqaihkynayrkaasviakyphkiksgaeakklpgvgtki   60
DSSP  lllhhhhhhhhhhhhhhhhhllllhhhhhhhhhhhhhhhhlllllllllhhhhhlllllhhh


DSSP  ------------------------------------------------llllllllllhh
Query ------------------------------------------------aapyssspptnlr   12
ident 
Sbjct aekideflatgklrklekirqddtsssinfltrvsgigpsaarkfvdegiktledlrkne  120
DSSP  hhhhhhhhhhlllhhhhhhhhlhhhhhhhhhhllllllhhhhhhhhhlllllhhhhhllh


DSSP  hhhhhhhhhhhhllllhhhhhHHHHHHHHHHHHH-LLLLllLLLLEEEEHHHHHHHHhhll
Query slrdrltqvaerqgvvfgrlqRHVAXIVVAQFAA-TLTDdtGAPLLLVKGGSSLELrrgi   71
ident                                                 | |
Sbjct dklnhhqriglkyfgdfekriPREEMLQMQDIVLnEVKKvdSEYIATVCGSFRRGA----  176
DSSP  hhllhhhhhhhhlhhhhllleEHHHHHHHHHHHHhHHHHhlLLLEEEEELHHHHLLL----


DSSP  llllLLLLEEEEEL-------lLHHHHHHHHHHHHhhllllleeEEELLLEELllllllLL
```

```
Query pdSRTSKDFATVAR-------rDIELIHEQLADAGetgwegftAIFTAPEEiaapgxpVK  124
ident      | |                    |||             |
Sbjct ---eSSGDMDVLLThpsftspkLLHQVVEQLQKVH--------FITDTLSKG------ET  219
DSSP  ---lEELLEEEEEElllllllHHHHHHHHHHHLL--------LEEEEEEL------LL


DSSP  LEEEEEEEEElleEEEEEEEEEEEL-LLLLllllleeeeelhhhhllllllleeeeellhHH
Query PRRFTAKLSYrgrAFATVPIEVSSV-EAGNadqfdtltsdalglvgvpaavavpcxtiPW  183
ident        |           |
Sbjct KFMGVCQLPS---EYPHRRIDIRLIpKDQY---------------------------yCG  249
DSSP  EEEEEELLLL---LLLLEEEEEEEELlHHHH---------------------------hHH


DSSP  HHhhhhhhhhlllllllllllhhhhHHHHHH----------------hhllhhhhhhhH
Query QIaqklhavtavleepkandrahdlvDLQLLE----------------glllldadlxptR  227
ident
Sbjct VL--------------------yftgSDIFNKnmrahaftinkytirplvtgvageplpV  289
DSSP  HH--------------------hhhlLHHHHhhhhllleellllleelllllllllllL


DSSP  HHHHhhhhhhlllLLLLllllllhhhhhhhhhllllllllllhhhhhhhhhhhhhhh
Query SACIaifearaqhPWPPrvatlphwpliyagalegldhlelartvdaaaqavqrfvarid  287
ident
Sbjct DSEK-------diFDYY----------------------------------------rep  302
DSSP  LLHH-------hhHLLL----------------------------------------llh


DSSP  hllll
Query ratkr  292
ident
Sbjct kdrse  307
DSSP  hhlll
```

No 123: Query=mol1A Sbjct=4z6dA Z-score=7.0

back to top

```
DSSP  ------------------------------------------------------------
Query ------------------------------------------------------------    0
ident
Sbjct pqetlnggitdmltelanfeknvsqaihkynayrkaasviakyphkiksgaeakklpgvg   60
DSSP  lllllhhhhhhhhhhhhhhhhllllhhhhhhhhhhhhhhhhllllllllhhhhhllllll


DSSP  ---------------------------------------------------lllllllll
Query ---------------------------------------------------aapyssppt    9
ident
Sbjct tkiaekideflatgklrklekirqddtsssinfltrvsgigpsaarkfvdegiktledlr  120
DSSP  hhhhhhhhhhhhhlllhhhhhhhhlhhhhhhhhhlllllllhhhhhhhhhlllllhhhhh


DSSP  lhhhhhhhhhhhhhhllllhhhhhHHHHHHHHHHHHH-LLLLllLLLLEEEEHHHHHHhh
Query nlrslrdrltqvaerqgvvfgrlqRHVAXIVVAQFAA-TLTDdtGAPLLLVKGGSSLElr   68
ident                                                   | |
Sbjct knedklnhhqriglkyfgdfekriPREEMLQMQDIVLnEVKKvdSEYIATVCGSFRRG--  178
DSSP  llhhhllhhhhhhhhlllhhhlleEHHHHHHHHHHHHHhHHHHhlLLLEEEELHHHHLL--


DSSP  hllllLLLLLLEEEEEL----------lLHHHHHHHHHHHHhhlllleeEEELLLEElll
Query rgipdSRTSKDFATVAR----------rDIELIHEQLADAGetgwegftAIFTAPEEiaa  118
ident         | |                       |||             |
Sbjct -----AESSGDMDVLLThpsftsesqpkLLHQVVEQLQKVH--------FITDTLSK---  222
DSSP  -----LLEELLEEEEEEllllllllllHHHHHHHHHHHLL--------LEEEEEEE---


DSSP  llllLLLEEEEEEEEEELL--EEEEEEEEEEEEL-LLLLlllleeeeelhhhhllllllee
Query pgxpVKPRRFTAKLSYRG--RAFATVPIEVSSV-EAGNadqfdtltsdalglvgvpaava  175
ident              |             |
Sbjct ---gETKFMGVCQLPSKNdeKEYPHRRIDIRLIpKDQY----------------------  257
DSSP  ---lLLEEEEEEELLLLLllLLLLLEEEEEEEELlHHHH----------------------


DSSP  eeellhHHHHhhhhhhhhlllllllllllHHHH-------------------hhhhhhh
Query vpcxtiPWQIaqklhavtavleepkandraHDLV-------------------dlqlleg  216
ident                                |
Sbjct -----yCGVL----------------yftgSDIFaknmrahalekgftineytirplgvt  296
DSSP  -----hHHHH----------------hhhlLHHHhhhhhhhhhllleeellllleeellll
```

```
DSSP  hllhHHHHHhhHHHHhhhhhhlllLLLLLLlllllhhhhhhhhhlllllllllllhhhhh
Query llldADLXPtrSACIaifearaqhPWPPRVatlphwpliyagalegldhlelartvdaaa  276
ident       |
Sbjct gvagEPLPV--DSEK-----difdYIQWKY------------------------------  319
DSSP  llllLLLLL--LLHH-----hhhhHLLLLL------------------------------


DSSP  hhhhhhhhhhhhllll
Query qavqrfvaridratkr  292
ident
Sbjct --------repkdrse  327
DSSP  --------llhhhlll
```

No 124: Query=mol1A Sbjct=4ymnA Z-score=7.0

[back to top]

```
DSSP  ------------------------------------------------------------
Query ------------------------------------------------------------    0
ident
Sbjct pqetlnggitdmltelanfeknvsqaihkynayrkaasviakyphkiksgaeakklpgvg   60
DSSP  llhhhhhhhhhhhhhhhhhhhhhllllhhhhhhhhhhhhhhhlllllllllhhhhhllllll


DSSP  --------------------------------------------------llllllllll
Query --------------------------------------------------aapysssppt    9
ident
Sbjct tkiaekideflatgklrklekirqddtsssinfltrvsgigpsaarkfvdegiktledlr  120
DSSP  hhhhhhhhhhhhhlllhhhhhhhhlhhhhhhhhhllllllhhhhhhhhhlllllhhhhh


DSSP  lhhhhhhhhhhhhhhhllllhhhhhHHHHHHHHHHHHHH-LLLLllLLLLEEEEHHHHHHHh
Query nlrslrdrltqvaerqgvvfgrlqRHVAXIVVAQFAA-TLTDdtGAPLLLVKGGSSLELr   68
ident                                                   | |
Sbjct knedklnhhqriglkyfgdfekriPREEMLQMQDIVLnEVKKvdSEYIATVCGSFRRGA-  179
DSSP  llhhhllhhhhhhhhlhhhhhlleEHHHHHHHHHHHHhHHHHhlLLLEEEELHHHHLLL-


DSSP  hlllllLLLLLEEEEEL----------lLHHHHHHHHHHHHhhlllleeEEELLLEElll
Query rgipdsRTSKDFATVAR----------rDIELIHEQLADAGetgwegftAIFTAPEEiaa  118
ident         | |                       |||             |
Sbjct ------ESSGDMDVLLThpsftsesqpkLLHQVVEQLQKVH--------FITDTLSK---  222
DSSP  ------LEELLEEEEEEllllllllllhHHHHHHHHHHHHLL--------LEEEEEEE---


DSSP  llllLLLEEEEEEEEELL--EEEEEEEEEEEEL-LLLLlllleeeeelhhhhlllllee
Query pgxpVKPRRFTAKLSYRG--RAFATVPIEVSSV-EAGNadqfdtltsdalglvgvpaava  175
ident              |             |
Sbjct ---gETKFMGVCQLPSKNdeKEYPHRRIDIRLIpKDQY----------------------  257
DSSP  ---lLLEEEEEEELLLLLllLLLLLLEEEEEEEELlHHHH----------------------


DSSP  eeellhHHHHhhhhhhhhlllllllllllHHHH-------------------hhhhhhh
Query vpcxtiPWQIaqklhavtavleepkandraHDLV------------------dlqlleg  216
ident                                |
Sbjct -----yCGVL----------------yftgSDIFnknmrahalekgftineytirplgvt  296
DSSP  -----hHHHH----------------hhhlLHHHhhhhhhlhhhhlleellllleellll


DSSP  hllhhHHHHhhHHHHHhhhhhlllLLLLLlllllhhhhhhhhhlllllllllllhhhhh
Query llldaDLXPtrSACIAifearaqhPWPPRvatlphwpliyagalegldhlelartvdaaa  276
ident       |
Sbjct gvagePLPV--DSEKD-----ifdYIQWK-------------------------------  318
DSSP  lllllLLLL--LLHHH-----hhlLLLLL-------------------------------


DSSP  hhhhhhhhhhhhllll
Query qavqrfvaridratkr  292
ident
Sbjct -------yrepkdrse  327
DSSP  -------lllhhhlll
```

No 125: Query=mol1A Sbjct=4ub5A Z-score=7.0

back to top

```
DSSP  ------------------------------------------------------------
Query ------------------------------------------------------------   0
ident 
Sbjct tlnggitdmltelanfeknvsqaihkynayrkaasviakyphkiksgaeakklpgvgtki  60
DSSP  lllhhhhhhhhhhhhhhhhllllhhhhhhhhhhhhhhhhlllllllhhhhhlllllhhh


DSSP  ------------------------------------------------llllllllllhh
Query ------------------------------------------------aapysspptnlr  12
ident 
Sbjct aekideflatgklrklekirqddtsssinfltrvsgigpsaarkfvdegiktledlrkne 120
DSSP  hhhhhhhhhhlllhhhhhhhhlhhhhhhhhhlllllllhhhhhhhhhlllllhhhhhllh


DSSP  hhhhhhhhhhhhllllhhhhhHHHHHHHHHHHHH-LLLLllLLLLEEEEHHHHHHHhhll
Query slrdrltqvaerqgvvfgrlqRHVAXIVVAQFAA-TLTDdtGAPLLLVKGGSSLELrrgi  71
ident                                                | |
Sbjct dklnhhqriglkyfgdfekriPREEMLQMQDIVLnEVKKvdSEYIATVCGSFRRGA---- 176
DSSP  hhllhhhhhhhhhlhhhhhlleEHHHHHHHHHHHHHhHHHLllLLLEEEEELHHHHLLL----


DSSP  llllLLLLEEEEEL--------lLHHHHHHHHHHHHhhlllleeEEELLLEEllllllL
Query pdsrTSKDFATVAR--------rDIELIHEQLADAGetgwegftAIFTAPEEiaapgxpV 123
ident      | |                     |||             |
Sbjct ---eSSGDMDVLLThpsftseskLLHQVVEQLQKVH--------FITDTLSK------gE 219
DSSP  ---lEELLEEEEEEllllllllHHHHHHHHHHHHLL--------LEEEEEEE------lL


DSSP  LLEEEEEEEEEEllеEEEEEEEEEEEEL-LLLLlllleeeeelhhhhllllllleeeeellhH
Query KPRRFTAKLSYrgrAFATVPIEVSSV-EAGNadqfdtltsdalglvgvpaavavpcxtiP 182
ident         |           |
Sbjct TKFMGVCQLPS---EYPHRRIDIRLIpKDQY---------------------------yC 249
DSSP  LEEEEEELLLL---LLLLEEEEEEELlHHHH---------------------------hH


DSSP  HHHhhhhhhhhhllllllllllllHHHH------------hhhhhhhhllhhHHHHhhHHH
Query WQIaqklhavtavleepkandraHDLV------------dlqllegllldaDLXPtrSAC 230
ident                         |                           |
Sbjct GVL----------------yftgSDIFnknmrahalekgftineytirpgePLPV--DSE 291
DSSP  HHH----------------hhhlLHHHhhhhhhhhhhhllleellllleelllLLLL--LLH


DSSP  HhhhhhhlllLLLLLLlllllhhhhhhhhhlllllllllllhhhhhhhhhhhhhhhhhll
Query IaifearaqhPWPPRVatlphwpliyagalegldhlelartvdaaaqavqrfvaridrat 290
ident 
Sbjct K-----difdYIQWKY--------------------------------------repkdr 308
DSSP  H-----hhhhHLLLLL--------------------------------------llhhhl


DSSP  ll
Query kr  292
ident 
Sbjct se  310
DSSP  ll
```

No 126: Query=mol1A Sbjct=4kleA Z-score=7.0

back to top

```
DSSP  ------------------------------------------------------------
Query ------------------------------------------------------------   0
ident 
Sbjct tlnggitdmltelanfeknvsqaihkynayrkaasviakyphkiksgaeakklpgvgtki  60
DSSP  lllhhhhhhhhhhhhhhhhllllhhhhhhhhhhhhhhhhlllllllhhhhhlllllhhh


DSSP  ------------------------------------------------llllllllllhh
Query ------------------------------------------------aapysspptnlr  12
ident 
Sbjct aekideflatgklrklekirqddtsssinfltrvsgigpsaarkfvdegiktledlrkne 120
DSSP  hhhhhhhhhhlllhhhhhhhhlhhhhhhhhhlllllllhhhhhhhhhhllllhhhhhhhlh
```

```
DSSP  hhhhhhhhhhhhllllhHHHHHHHHHHHHHHHHHH-LLLLllLLLLEEEEHHHHHHHHhhll
Query slrdrltqvaerqgvvfGRLQRHVAXIVVAQFAA-TLTDdtGAPLLLVKGGSSLELrrgi   71
ident                                                | |
Sbjct dklnhhqriglkyfgdfEKRIPREEMLQMQDIVLnEVKKvdSEYIATVCGSFRRGA----  176
DSSP  hhllhhhhhhhhlhhhhLLLEEHHHHHHHHHHHHHhHHHHhlLLLEEEEEELLLLLLL----


DSSP  llllLLLLEEEEEL------------lLHHHHHHHHHHHHhhlllleeEEELLLEELlll
Query pdsrTSKDFATVAR------------rDIELIHEQLADAGetgwegftAIFTAPEEIaap  119
ident      | |                         |||             |
Sbjct ---eSSGDMDVLLThpsftsestkqpkLLHQVVEQLQKVH--------FITDTLSKG---  222
DSSP  ---lEELLEEEEEElllllllllllllHHHHHHHHHHHHLL--------LEEEEEEEL---


DSSP  lllLLLEEEEEEEEEL--LEEEEEEEEEEEEL-LLLLlllleeeeelhhhhllllllleee
Query gxpVKPRRFTAKLSYR--GRAFATVPIEVSSV-EAGNadqfdtltsdalglvgvpaavav  176
ident             |             |
Sbjct ---ETKFMGVCQLPSKndEKEYPHRRIDIRLIpKDQY-----------------------  256
DSSP  ---LLEEEEEEELLLLLllLLLLLLEEEEEEEELlHHHH-----------------------


DSSP  eellhHHHHhhhhhhhhllllllllllllHHHHHHH-------------------hhhhh
Query pcxtiPWQIaqklhavtavleepkandraHDLVDLQ-------------------llegl  217
ident                               |
Sbjct ----yCGVL-----------------yftgSDIFNKNmrahalekgftineytirplgvtg  296
DSSP  ----hHHHH-----------------hhhlLHHHHHHhhhhhhhlleeellllleeelllll


DSSP  llhHHHHhhHHHHHhhhhhhllllLLLLLLllllhhhhhhhhhlllllllllllhhhhhh
Query lldADLXptRSACIaifearaqhpWPPRVAtlphwpliyagalegldhlelartvdaaaq  277
ident      |
Sbjct vagEPLP--VDSEK-------difDYIQWK------------------------------  317
DSSP  lllLLLL--LLLHH-------hhhHHLLLL------------------------------


DSSP  hhhhhhhhhhhllll
Query avqrfvaridratkr  292
ident
Sbjct ------yrepkdrse  326
DSSP  ------lllhhhlll
```

No 127: Query=mol1A Sbjct=5j2fA Z-score=6.9

back to top

```
DSSP  ------------------------------------------------------------
Query ------------------------------------------------------------    0
ident
Sbjct tlnggitdmltelanfeknvsqaihkynayrkaasviakyphkiksgaeakklpgvgtki   60
DSSP  lllhhhhhhhhhhhhhhhhhllllhhhhhhhhhhhhhhhhllllllllhhhhhllllllhhh


DSSP  ------------------------------------------------llllllllllhh
Query ------------------------------------------------aapysspptnlr   12
ident
Sbjct aekideflatgklrklekirqddtsssinfltrvsgigpsaarkfvdegiktledlrkne  120
DSSP  hhhhhhhhhhlllhhhhhhhhlhhhhhhhhhlllllllhhhhhhhhhlllllhhhhhhllh


DSSP  hhhhhhhhhhhhllllhhhhhHHHHHHHHHHHHHH-LLLLllLLLLEEEEHHHHHHHHhhll
Query slrdrltqvaerqgvvfgrlqRHVAXIVVAQFAA-TLTDdtGAPLLLVKGGSSLELrrgi   71
ident                                                | |
Sbjct dklnhhqriglkyfgdfekriPREEMLQMQDIVLnEVKKvdSEYIATVCGSFRRGA----  176
DSSP  hhllhhhhhhhhlllhhhlleEHHHHHHHHHHHHHhHHHHhlLLLEEEEELHHHHLLL----


DSSP  llllLLLLEEEEEL------------lLHHHHHHHHHHHHhhlllleeEEELLLEEllll
Query pdsrTSKDFATVAR------------rDIELIHEQLADAGetgwegftAIFTAPEEiaap  119
ident      | |                         |||             |
Sbjct ---eSSGDMDVLLThpsftsestkqpkLLHQVVEQLQKVH--------FITDTLSK----  221
DSSP  ---lEELLEEEEEEllleelleellllHHHHHHHHHHHHLL--------LEEEEEEE----


DSSP  lllLLLEEEEEEEEEL--LEEEEEEEEEEEEL-LLLLlllleeeeelhhhhllllllleee
Query gxpVKPRRFTAKLSYR--GRAFATVPIEVSSV-EAGNadqfdtltsdalglvgvpaavav  176
ident             |             |
```

```
Sbjct --gETKFMGVCQLPSKndEKEYPHRRIDIRLIpKDQY-----------------------  256
DSSP  --lLLEEEEEEELLLLLllLLLLLLLEEEEEEEELlHHHH----------------------- 


DSSP  eellhHHHHhhhhhhhhlllllllllllHHHH------------------hhhhhhhh
Query pcxtiPWQIaqklhavtavleepkandraHDLV------------------dlqllegl  217
ident                               |
Sbjct ----yCGVL----------------yftgSDIFnknmrahalekgftineytirplgvtg  296
DSSP  ----hHHHH----------------hhhlLHHHhhhhhhhhhhhhleeellllleeellllll


DSSP  llhHHHHHhhHHHHhhhhhhlllLLLLLLllllllhhhhhhhhhlllllllllllhhhhhh
Query lldADLXPtrSACIaifearaqhPWPPRVatlphwpliyagalegldhlelartvdaaaq  277
ident      |
Sbjct vagEPLPV--DSEK-----difdYIQWKY-------------------------------  318
DSSP  lllLLLLL--LLHH-----hhhhHLLLLL-------------------------------


DSSP  hhhhhhhhhhhllll
Query avqrfvaridratkr  292
ident 
Sbjct -------repkdrse  326
DSSP  -------llhhhlll
```

No 128: Query=mol1A Sbjct=5j2cA Z-score=6.9

back to top

```
DSSP  ------------------------------------------------------------
Query ------------------------------------------------------------    0
ident 
Sbjct tlnggitdmltelanfeknvsqaihkynayrkaasviakyphkiksgaeakklpgvgtki   60
DSSP  lllhhhhhhhhhhhhhhhhllllhhhhhhhhhhhhhhhhlllllllhhhhhlllllllhhh


DSSP  ------------------------------------------------llllllllllhh
Query ------------------------------------------------aapysspptnlr   12
ident 
Sbjct aekideflatgklrklekirqddtsssinfltrvsgigpsaarkfvdegiktledlrkne  120
DSSP  hhhhhhhhhhlllhhhhhhhhlhhhhhhhhhllllllllhhhhhhhhhlllllhhhhhhlh


DSSP  hhhhhhhhhhhllllhhhhhHHHHHHHHHHHHHH-LLLLllLLLLEEEEHHHHHHHHhhll
Query slrdrltqvaerqgvvfgrlqRHVAXIVVAQFAA-TLTDdtGAPLLLVKGGSSLELrrgi   71
ident                                                | |
Sbjct dklnhhqriglkyfgdfekriPREEMLQMQDIVLnEVKKvdSEYIATVCGSFRRGA----  176
DSSP  hhllhhhhhhhhlhhhhhlleEHHHHHHHHHHHHHhHHHHhlLLLEEEELHHHHLLL----


DSSP  llllLLLLEEEEEL------------lLHHHHHHHHHHHHHhhllllleeEEELLLEEllll
Query pdsrTSKDFATVAR------------rDIELIHEQLADAGetgwegftAIFTAPEEiaap  119
ident      | |                         |||             |
Sbjct ---eSSGDMDVLLThpsftsestkqpkLLHQVVEQLQKVH--------FITDTLSK----  221
DSSP  ---lEELLEEEEEEllllllllllllllHHHHHHHHHHHHHLL--------LEEEEEEEE----


DSSP  lllLLLEEEEEEEEEEL--LEEEEEEEEEEEEL-LLLLllllleeeeelhhhhllllllleee
Query gxpVKPRRFTAKLSYR--GRAFATVPIEVSSV-EAGNadqfdtltsdalglvgvpaavav  176
ident             |             |
Sbjct --gETKFMGVCQLPSKndEKEYPHRRIDIRLIpKDQY-----------------------  256
DSSP  --lLLEEEEEEELLLLLllLLLLLLLEEEEEEEELlHHHH-----------------------


DSSP  eellhHHHHhhhhhhhhlllllllllllHHHH------------------hhhhhhhh
Query pcxtiPWQIaqklhavtavleepkandraHDLV------------------dlqllegl  217
ident                               |
Sbjct ----yCGVL----------------yftgSDIFnknmrahalekgftineytirplgvtg  296
DSSP  ----hHHHH----------------hhhlLHHHhhhhhhhhhhhhleeellllleeellllll


DSSP  llhHHHHHhhHHHHhhhhhhlllLLLLLLllllllhhhhhhhhhlllllllllllhhhhhh
Query lldADLXPtrSACIaifearaqhPWPPRVatlphwpliyagalegldhlelartvdaaaq  277
ident      |
Sbjct vagEPLPV--DSEK-----difdYIQWKY-------------------------------  318
DSSP  lllLLLLL--LLHH-----hhhhHLLLLL-------------------------------
```

```
DSSP  [illegible]
Query avqrfvaridratkr  292
ident
Sbjct -------repkdrse  326
DSSP  -------llhhhlll
```

No 129: Query=mol1A Sbjct=4pheA Z-score=6.9

back to top

```
DSSP  ------------------------------------------------------------
Query ------------------------------------------------------------    0
ident
Sbjct pqetlnggitdmltelanfeknvsqaihkynayrkaasviakyphkiksgaeakklpgvg   60
DSSP  llhhhhhhhhhhhhhhhhhhhhllllhhhhhhhhhhhhhhhhlllllllhhhhhllllll


DSSP  ---------------------------------------------------lllllllll
Query ---------------------------------------------------aapyssppt    9
ident
Sbjct tkiaekideflatgklrklekirqddtsssinfltrvsgigpsaarkfvdegiktledlr  120
DSSP  hhhhhhhhhhhhhlllhhhhhhhhhlhhhhhhhhhllllllhhhhhhhhhlllllhhhhh


DSSP  lhhhhhhhhhhhhhhllllhhhhhHHHHHHHHHHHHH-LLLLllLLLLEEEEHHHHHHHh
Query nlrslrdrltqvaerqgvvfgrlqRHVAXIVVAQFAA-TLTDdtGAPLLLVKGGSSLELr   68
ident                                                   | |
Sbjct knedklnhhqriglkyfgdfekriPREEMLQMQDIVLnEVKKvdSEYIATVCGSFRRGA-  179
DSSP  hlhhhllhhhhhhhhlhhhhllleEHHHHHHHHHHHHhHHHHhlLLLEEEELHHHHLLL-


DSSP  hllllllLLLLEEEEEL----------lLHHHHHHHHHHHHhhlllleeEEELLLEElll
Query rgipdsrTSKDFATVAR----------rDIELIHEQLADAGetgwegftAIFTAPEEiaa  118
ident         | |                       |||             |
Sbjct ------eSSGDMDVLLThpsftsesqpkLLHQVVEQLQKVH--------FITDTLSK---  222
DSSP  ------lEELLEEEEEllllllllllllHHHHHHHHHHHLL--------LEEEEEEE---


DSSP  llllLLLEEEEEEEEEELLEEEEEEEEEEEL-LLLLlllleeeeelhhhhlllllleeee
Query pgxpVKPRRFTAKLSYRGRAFATVPIEVSSV-EAGNadqfdtltsdalglvgvpaavavp  177
ident              |           |
Sbjct ---gETKFMGVCQLPSDEKEYPHRRIDIRLIpKDQY------------------------  255
DSSP  ---lLLEEEEEELLLLLLLLLLEEEEEEEELlHHHH------------------------


DSSP  ellhHHHHhhhhhhhhhlllllllllllHHHH-------------------hhhhhhhhl
Query cxtiPWQIaqklhavtavleepkandraHDLV-------------------dlqllegll  218
ident                              |
Sbjct ---yCGVL----------------yftgSDIFnknmrahalekgftineytirplgvtgv  296
DSSP  ---hHHHH----------------hlllLHHHhhhhhhhhhlleeellllleeellllll


DSSP  lhhHHHHhhHHHHHhhhhhlllLLLLLllllllhhhhhhhhhllllllllllllhhhhhhh
Query ldaDLXPtrSACIAifearaqhPWPPRvatlphwpliyagalegldhlelartvdaaaqa  278
ident     |
Sbjct agePLPV--DSEKD-----ifdYIQWK----------------------------------  316
DSSP  lllLLLL--LLHHH-----hhhHLLLL----------------------------------


DSSP  hhhhhhhhhhllll
Query vqrfvaridratkr  292
ident
Sbjct -----yrepkdrse  325
DSSP  -----lllhhhlll
```

No 130: Query=mol1A Sbjct=4o5eA Z-score=6.9

back to top

```
DSSP  ------------------------------------------------------------
Query ------------------------------------------------------------    0
ident
Sbjct pqetlnggitdmltelanfeknvsqaihkynayrkaasviakyphkiksgaeakklpgvg   60
DSSP  llhhhhhhhhhhhhhhhhhhhhllllhhhhhhhhhhhhhhhhllllllllhhhhhlllll
```

```
DSSP  ---------------------------------------------------lllllllll
Query ---------------------------------------------------aapyssppt    9
ident 
Sbjct tkiaekideflatgklrklekirqddtsssinfltrvsgigpsaarkfvdegiktledlr  120
DSSP  hhhhhhhhhhhhhhlllhhhhhhhhllhhhhhhhhhllllllllhhhhhhhhhlllllhhhhh


DSSP  lhhhhhhhhhhhhhhhllllhhhhhHHHHHHHHHHHHH-LLLLllLLLLEEEEHHHHHHHh
Query nlrslrdrltqvaerqgvvfgrlqRHVAXIVVAQFAA-TLTDdtGAPLLLVKGGSSLELr   68
ident                                                     | |
Sbjct knedklnhhqriglkyfgdfekriPREEMLQMQDIVLnEVKKvdSEYIATVCGSFRRGA-  179
DSSP  llhhhllhhhhhhhhlhhhhllleEHHHHHHHHHHHHHhHHHHhlLLLEEEELHHHHLLL-


DSSP  hllllllLLLLEEEEEL----------lLHHHHHHHHHHHHHhhllllleeEEELLLEElll
Query rgipdsrTSKDFATVAR----------rDIELIHEQLADAGetgwegftAIFTAPEEiaa  118
ident         | |                       |||             |
Sbjct ------eSSGDMDVLLThpsftsesqpkLLHQVVEQLQKVH--------FITDTLSK---  222
DSSP  ------lEELLEEEEEEllllllllllHHHHHHHHHHHHLL--------LEEEEEEE---


DSSP  llllLLLEEEEEEEEEELL--EEEEEEEEEEEELLLLLLlllleeeeelhhhhlllllleee
Query pgxpVKPRRFTAKLSYRG--RAFATVPIEVSSVEAGNAdqfdtltsdalglvgvpaavav  176
ident              |             |
Sbjct ---gETKFMGVCQLPSKNdeKEYPHRRIDIRLIPKDQY----------------------  257
DSSP  ---lLLEEEEEEELLLLLLllLLLLLLEEEEEEEELLHHHH----------------------


DSSP  eellhHHHHhhhhhhhhlllllllllllHHHH-------------------hhhhhhhh
Query pcxtiPWQIaqklhavtavleepkandraHDLV-------------------dlqllegl  217
ident                               |
Sbjct ----yCGVL----------------yftgSDIFnknmrahalekgftineytirplgvtg  297
DSSP  ----hHHHH----------------hhhlLHHHhhhhhhhhhllleeellllleeelllll


DSSP  llhhHHHHhhHHHHHhhhhhlllLLLLLllllllhhhhhhhhhlllllllllllhhhhhh
Query lldaDLXPtrSACIAifearaqhPWPPRvatlphwpliyagalegldhlelartvdaaaq  277
ident      |
Sbjct vagePLPV--DSEKD-----ifdYIQWK--------------------------------  318
DSSP  llllLLLL--LLHHH-----hhhHLLLL--------------------------------


DSSP  hhhhhhhhhhhllll
Query avqrfvaridratkr  292
ident 
Sbjct ------yrepkdrse  327
DSSP  ------lllhhhlll
```

No 131: Query=mol1A Sbjct=5db7A Z-score=6.9

back to top

```
DSSP  ------------------------------------------------------------
Query ------------------------------------------------------------    0
ident 
Sbjct pqetlnggitdmltelanfeknvsqaihkynayrkaasviakyphkiksgaeakklpgvg   60
DSSP  llhhhhhhhhhhhhhhhhhhhhhllllhhhhhhhhhhhhhhhllllllllhhhhhllllll


DSSP  ---------------------------------------------------lllllllll
Query ---------------------------------------------------aapyssppt    9
ident 
Sbjct tkiaekideflatgklrklekirqddtsssinfltrvsgigpsaarkfvdegiktledlr  120
DSSP  hhhhhhhhhhhhhhlllhhhhhhhhlhhhhhhhhhllllllllhhhhhhhhhlllllhhhhh


DSSP  lhhhhhhhhhhhhhhhllllhhhhhHHHHHHHHHHHHH-LLLLllLLLLEEEEHHHHHHHhh
Query nlrslrdrltqvaerqgvvfgrlqRHVAXIVVAQFAA-TLTDdtGAPLLLVKGGSSLElr   68
ident                                                     | |
Sbjct knedklnhhqriglkyfgdfekriPREEMLQMQDIVLnEVKKvdSEYIATVCGSFRRG--  178
DSSP  hlhhhllhhhhhhhhlhhhhllleEHHHHHHHHHHHHHhHHHHhlLLLEEEELHHHHLL--


DSSP  hllllLLLLLLEEEEEL----------lLHHHHHHHHHHHHHhhllllleeEEELLLEElll
Query rgipdSRTSKDFATVAR----------rDIELIHEQLADAGetgwegftAIFTAPEEiaa  118
```

```
ident        | |                        |||             |
Sbjct -----AESSGDMDVLLThpsftsesqpkLLHQVVEQLQKVH--------FITDTLSK---  222
DSSP  -----LLEELLEEEEEEllllllllllHHHHHHHHHHHHLL--------LEEEEEEE---


DSSP  llllLLLEEEEEEEEELL--EEEEEEEEEEEELLLLLLllleeeeelhhhhllllllleee
Query pgxpVKPRRFTAKLSYRG--RAFATVPIEVSSVEAGNAdqfdtltsdalglvgvpaavav  176
ident              |             |
Sbjct ---gETKFMGVCQLPSKNdeKEYPHRRIDIRLIPKDQY----------------------  257
DSSP  ---lLLEEEEEEELLLLLLllLLLLLLEEEEEEEELLLLLH----------------------


DSSP  eellhHHHHhhhhhhhhlllllllllllHHHH-----------------hhhhhhhll
Query pcxtiPWQIaqklhavtavleepkandraHDLV-----------------dlqlleglll  219
ident                               |
Sbjct ----yCGVL----------------yftgSDIFnknmrahalekgftineytirplgvtg  297
DSSP  ----hHHHH----------------hlllLHHHhhhhhhllllllleeellllleeelllll


DSSP  hhhhhhhhHHHHHhhhhhlllLLLLLllllllhhhhhhhhhllllllllllhhhhhhhh
Query dadlxptrSACIAifearaqhPWPPRvatlphwpliyagalegldhlelartvdaaaqav  279
ident
Sbjct vageplpvDSEKD-----ifdYIQWK----------------------------------  318
DSSP  llllllllLLHHH-----hhhHLLLL----------------------------------


DSSP  hhhhhhhhhllll
Query qrfvaridratkr  292
ident
Sbjct ----yrepkdrse  327
DSSP  ----lllhhhlll
```

No 132: Query=mol1A Sbjct=3rh4A Z-score=6.9

back to top

```
DSSP  ------------------------------------------------------------
Query ------------------------------------------------------------    0
ident
Sbjct tlnggitdmltelanfeknvsqaihkynayrkaasviakyphkiksgaeakklpgvgtki   60
DSSP  lllhhhhhhhhhhhhhhhhhllllhhhhhhhhhhhhhhhhhllllllhhhhhllllllhhh


DSSP  ------------------------------------------------lllllllllhh
Query ------------------------------------------------aapysspptnlr   12
ident
Sbjct aekideflatgklrklekirqddtsssinfltrvsgigpsaarkfvdegiktledlrkne  120
DSSP  hhhhhhhhhhlllhhhhhhhhhlhhhhhhhhhhllllllhhhhhhhhhhlllllhhhhhhllh


DSSP  hhhhhhhhhhhhllllhhhhhHHHHHHHHHHHHH-LLLLllLLLLEEEEHHHHHHHHhhll
Query slrdrltqvaerqgvvfgrlqRHVAXIVVAQFAA-TLTDdtGAPLLLVKGGSSLELrrgi   71
ident                                                | |
Sbjct dklnhhqriglkyfgdfekriPREEMLQMQDIVLnEVKKvdSEYIATVCGSFRRGA----  176
DSSP  hhllhhhhhhhhlhhhhllleEHHHHHHHHHHHHHhHHHHhlLLLEEEELHHHHLLL----


DSSP  llllLLLLEEEEEEL------------lLHHHHHHHHHHHHhhlllleeEEELLLEELlll
Query pdsrTSKDFATVAR------------rDIELIHEQLADAGetgwegftAIFTAPEEIaap  119
ident      | |                         |||             |
Sbjct ---eSSGDMDVLLThpsftestkqpkLLHQVVEQLQKVH--------FITDTLSKG---  222
DSSP  ---lEELLEEEEEEllllllllllllHHHHHHHHHHHHLL--------LEEEEEEEL---


DSSP  lllLLLEEEEEEEEEEL--LEEEEEEEEEEEEL-LLLLlllleeeeelhhhhllllllleee
Query gxpVKPRRFTAKLSYR--GRAFATVPIEVSSV-EAGNadqfdtltsdalglvgvpaavav  176
ident             |             |
Sbjct ---ETKFMGVCQLPSKndEKEYPHRRIDIRLIpKDQY-----------------------  256
DSSP  ---LLEEEEEEELLLLLllLLLLLLLEEEEEEEELlHHHH-----------------------


DSSP  eellhHHHHhhhhhhhhllllllllllllHHHHHHH-------------------hhhhh
Query pcxtiPWQIaqklhavtavleepkandraHDLVDLQ-------------------llegl  217
ident                               |
Sbjct ----yCGVL----------------yftgSDIFNKNmrahalekgftineytirplgvtg  296
DSSP  ----hHHHH----------------hhhlLHHHHHHhhhhhhhlleeellllleeelllll
```

```
DSSP  llhHHHHhhHHHHHhhhhhhllllLLLLLlllllhhhhhhhhhllllllllllhhhhhh
Query lldADLXptRSACIaifearaqhpWPPRVatlphwpliyagalegldhlelartvdaaaq  277
ident      |
Sbjct vagEPLP--VDSEK-------difDYIQW-------------------------------  316
DSSP  lllLLLL--LLLHH-------hhhHHLLL-------------------------------


DSSP  hhhhhhhhhhhllll
Query avqrfvaridratkr  292
ident
Sbjct -----kyrepkdrse  326
DSSP  -----llllhhhlll
```

No 133: Query=mol1A Sbjct=4klsA Z-score=6.9

back to top

```
DSSP  ------------------------------------------------------------
Query ------------------------------------------------------------    0
ident
Sbjct tlnggitdmltelanfeknvsqaihkynayrkaasviakyphkiksgaeakklpgvgtki   60
DSSP  lllhhhhhhhhhhhhhhhhllllhhhhhhhhhhhhhhhhlllllllhhhhhllllllhhh


DSSP  ------------------------------------------------llllllllllhh
Query ------------------------------------------------aapysspptnlr   12
ident
Sbjct aekideflatgklrklekirqddtsssinfltrvsgigpsaarkfvdegiktledlrkne  120
DSSP  hhhhhhhhhhlllhhhhhhhhlhhhhhhhhhlllllllhhhhhhhhhlllllhhhhhhlh


DSSP  hhhhhhhhhhhllllhhhhhHHHHHHHHHHHHHH-LLLLllLLLLEEEEHHHHHHHHhhll
Query slrdrltqvaerqgvvfgrlqRHVAXIVVAQFAA-TLTDdtGAPLLLVKGGSSLELrrgi   71
ident                                                | |
Sbjct dklnhhqriglkyfgdfekriPREEMLQMQDIVLnEVKKvdSEYIATVCGSFRRGA----  176
DSSP  hhllhhhhhhhhlhhhhhlleEHHHHHHHHHHHHhHHHHhlLLLEEEEELHHHHLL----


DSSP  llllLLLLEEEEEL------------lLHHHHHHHHHHHHHhhllllleeEEELLLEELlll
Query pdsrTSKDFATVAR------------rDIELIHEQLADAGetgwegftAIFTAPEEIaap  119
ident      | |                         |||             |
Sbjct ---eSSGDMDVLLThpsftsestkqpkLLHQVVEQLQKVH--------FITDTLSKG---  222
DSSP  ---lEELLEEEEEEllllllllllllHHHHHHHHHHHHLL--------LEEEEEEEL---


DSSP  lllLLLEEEEEEEEElLEEEEEEEEEEEEL-LLLLllllleeeeelhhhhlllllleeeee
Query gxpVKPRRFTAKLSYrGRAFATVPIEVSSV-EAGNadqfdtltsdalglvgvpaavavpc  178
ident             |           |
Sbjct ---ETKFMGVCQLPSdEKEYPHRRIDIRLIpKDQY-------------------------  254
DSSP  ---LLEEEEEEELLLLlLLLLLLEEEEEEEELlHHHH-------------------------


DSSP  llhHHHHhhhhhhhhllllllllllllHHHHHHH-------------------hhhhhll
Query xtiPWQIaqklhavtavleepkandraHDLVDLQ-------------------lleglll  219
ident                             |
Sbjct --yCGVL----------------yftgSDIFNKNmrahalekgftineytirplgvtgva  296
DSSP  --hHHHH----------------hhhlLHHHHHHhhhhhhllleeellllleeelllllll


DSSP  hHHHHHhhHHHHhhhhhhlllLLLLLLlllllhhhhhhhhhllllllllllhhhhhhhh
Query dADLXPtrSACIaifearaqhPWPPRVatlphwpliyagalegldhlelartvdaaaqav  279
ident    |
Sbjct gEPLPV--DSEK------difDYIQWK---------------------------------  315
DSSP  lLLLLL--LLHH------hhhHHHLLL---------------------------------


DSSP  hhhhhhhhhllll
Query qrfvaridratkr  292
ident
Sbjct ----yrepkdrse  324
DSSP  ----lllhhhhll
```

No 134: Query=mol1A Sbjct=4kltA Z-score=6.9

back to top

```
DSSP  ------------------------------------------------------------
Query ------------------------------------------------------------     0
ident 
Sbjct tlnggitdmltelanfeknvsqaihkynayrkaasviakyphkiksgaeakklpgvgtki    60
DSSP  lllhhhhhhhhhhhhhhhhllllhhhhhhhhhhhhhhhlllllllhhhhhlllllhhh


DSSP  ------------------------------------------------llllllllllhh
Query ------------------------------------------------aapyssppptnlr    12
ident 
Sbjct aekideflatgklrklekirqddtsssinfltrvsgigpsaarkfvdegiktledlrkne   120
DSSP  hhhhhhhhhllllhhhhhhhhlhhhhhhhhhllllllhhhhhhhhhlllllhhhhhllh


DSSP  hhhhhhhhhhhllllhhhhhHHHHHHHHHHHHHH-LLLLllLLLLEEEEHHHHHHHHhhll
Query slrdrltqvaerqgvvfgrlqRHVAXIVVAQFAA-TLTDdtGAPLLLVKGGSSLELrrgi    71
ident                                                | |
Sbjct dklnhhqriglkyfgdfekriPREEMLQMQDIVLnEVKKvdSEYIATVCGSFRRGA----   176
DSSP  hhllhhhhhhhhlhhhhhlleEHHHHHHHHHHHHHhHHHHhlLLLEEEEEHHHHHLL----


DSSP  llllLLLLEEEEEL---------lLHHHHHHHHHHHHhhlllleeEEELLLEELllllll
Query pdsrTSKDFATVAR---------rDIELIHEQLADAGetgwegftAIFTAPEEIaapgxp   122
ident      | |                      |||             |
Sbjct ---eSSGDMDVLLThpsfttkqpkLLHQVVEQLQKVH--------FITDTLSKG------   219
DSSP  ---lEELLEEEEEEllllllllllHHHHHHHHHHHHLL--------LEEEEEEEL------


DSSP  LLLEEEEEEEEElLEEEEEEEEEEEEL-LLLLllllleeeeelhhhhlllllleeeeellh
Query VKPRRFTAKLSYrGRAFATVPIEVSSV-EAGNadqfdtltsdalglvgvpaavavpcxti   181
ident          |           |
Sbjct ETKFMGVCQLPS-EKEYPHRRIDIRLIpKDQY---------------------------y   251
DSSP  LLEEEEEELLLL-LLLLLLEEEEEEELlHHHH---------------------------h


DSSP  HHHHhhhhhhhhlllllllllllHHHHHHH-------------hhhhhllhHHHHHhhH
Query PWQIaqklhavtavleepkandraHDLVDLQ-------------lleglllldADLXPtrS   228
ident                          |                            |
Sbjct CGVL----------------yftgSDIFNKNmrahalekgftineytirpagEPLPV--D   293
DSSP  HHHH----------------hhllLHHHHHHhhhhhllllleellllleelllLLLLL--L


DSSP  HHHhhhhhhllllLLLLLlllllhhhhhhhhhlllllllllllhhhhhhhhhhhhhhhh
Query ACIaifearaqhpWPPRVatlphwpliyagalegldhlelartvdaaaqavqrfvaridr   288
ident 
Sbjct SEK------difdYIQWK-------------------------------------yrepk   310
DSSP  LHH------hhhhHLLLL-------------------------------------lllhh


DSSP  llll
Query atkr  292
ident 
Sbjct drse  314
DSSP  hlll
```

No 135: Query=mol1A Sbjct=3uq1A Z-score=6.9

back to top

```
DSSP  ------------------------------------------------------------
Query ------------------------------------------------------------     0
ident 
Sbjct hnlhiteklevlakaysvqgdkwralgyakainalksfhkpvtsyqeacsipgigkrmae    60
DSSP  lllllhhhhhhhhhhhhhlllhhhhhhhhhhhhhhhlllllllllhhhhllllllllhhhhh


DSSP  -------------------------------------------llllllllllhhhhhhh
Query -------------------------------------------aapyssppptnlrslrdr    17
ident 
Sbjct kiieilesghlrkldhisesvpvlelfsniwgagtktaqmwyqqgfrsledirsqasltt   120
DSSP  hhhhhhhhlllhhhhlllllhhhhhhhhllllllhhhhhhhhhllllllhhhhhhhhllllh


DSSP  hhhhhhhllllhhhhHHHHHHHHHHHHHHHHLL-LLLLLLLLEEEEHHHHHHHHhhllllllL
Query ltqvaerqgvvfgrlQRHVAXIVVAQFAATL-TDDTGAPLLLVKGGSSLELrrgipdsrT    76
```

```
ident                            |             |    |              |
Sbjct qqaiglkhysdflerMPREEATEIEQTVQKAaQAFNSGLLCVACGSYRRGK-------aT  173
DSSP  hhhhhhhlllhhhllEEHHHHHHHHHHHHHHHhHLLLLLLEEEELHHHHLLL-------lE


DSSP  LLLEEEEEL----lLHHHHHHHHHHHHhhlllleeEEELLLEELllllllLLLEEEEEEE
Query SKDFATVAR----rDIELIHEQLADAGetgwegftAIFTAPEEIaapgxpVKPRRFTAKL  132
ident   |                   |   |                                |
Sbjct CGDVDVLIThpdggIFSRLLDSLRQEG--------FLTDDLVKG------ETKYLGVCRL  219
DSSP  ELLEEEEEEellllHHHHHHHHHHHLL--------LEEEEEEEL------LLEEEEEELL


DSSP  EEllEEEEEEEEEEEEL-LLLLllllleeeeelhhhhllllllleeeeellhHHHHhhhhhh
Query SYrgRAFATVPIEVSSV-EAGNadqfdtltsdalglvgvpaavavpcxtiPWQIaqklha  191
ident                 |
Sbjct PG--PGRRHRRLDIIVVpYSEF---------------------------aCALL------  244
DSSP  LL--LLLLLEEEEEEEEeHHHH---------------------------hHHHH------


DSSP  hhllllllllllllHHHHHHH-------------------------hhhhhllhHHHHhh
Query vtavleepkandraHDLVDLQ-------------------------llegllldADLXpt  226
ident                                                         |
Sbjct ----------yftgSAHFNRSmralaktkgmslsehalstavvrnthgakvgpgRVLP--  292
DSSP  ----------hhhlLHHHHHHhhhhhhhllleellllleeelleellllleeellEELL--


DSSP  HHHHHhhhhhhllllLLLLLlllllhhhhhhhhhlllllllllllhhhhhhhhhhhhhh
Query RSACIaifearaqhpWPPRVatlphwpliyagalegldhlelartvdaaaqavqrfvari  286
ident
Sbjct TPTEK-------dvfRLLGL------------------------------------pyre  309
DSSP  LLLHH-------hhhHHLLL------------------------------------llll


DSSP  hhllll
Query dratkr  292
ident
Sbjct paerdw  315
DSSP  hhhhll
```

No 136: Query=mol1A Sbjct=4fo6A Z-score=6.9

back to top

```
DSSP  ------------------------------------------------------------
Query ------------------------------------------------------------    0
ident
Sbjct hnlhiteklevlakaysvqgdkwralgyakainalksfhkpvtsyqeacsipgigkrmae   60
DSSP  lllllhhhhhhhhhhhhhlllhhhhhhhhhhhhhhhllllllllhhhhlllllllhhhhh


DSSP  -------------------------------------------lllllllllhhhhhhh
Query -------------------------------------------aapyssppptnlrslrdr   17
ident
Sbjct kiieilesghlrkldhisesvpvlelfsniwgagtktaqmwyqqgfrsledirsqasltt  120
DSSP  hhhhhhhhlllhhhhlllllhhhhhhhhlllllhhhhhhhhhlllllhhhhhhhllllh


DSSP  hhhhhhhllllhhhhHHHHHHHHHHHHHHHHLL-LLLLLLLLEEEEHHHHHHHhhllllllL
Query ltqvaerqgvvfgrlQRHVAXIVVAQFAATL-TDDTGAPLLLVKGGSSLELrrgipdsrT   76
ident                          |             |    |              |
Sbjct qqaiglkhysdflerMPREEATEIEQTVQKAaQAFNSGLLCVACGSYRRGK-------aT  173
DSSP  hhhhhhhlllhhhllEEHHHHHHHHHHHHHHHhHLLLLLLEEEELHHHHLLL-------lE


DSSP  LLLEEEEEL----lLHHHHHHHHHHHHhhlllleeEEELLLEELllllllLLLEEEEEEE
Query SKDFATVAR----rDIELIHEQLADAGetgwegftAIFTAPEEIaapgxpVKPRRFTAKL  132
ident   |                   |   |                                |
Sbjct CGDVDVLIThpdggIFSRLLDSLRQEG--------FLTDDLVKG------ETKYLGVCRL  219
DSSP  ELLEEEEEEellllHHHHHHHHHHHLL--------LEEEEEEEL------LLEEEEEELL


DSSP  EEllEEEEEEEEEEEEL-LLLLllllleeeeelhhhhllllllleeeeellhHHHHhhhhhh
Query SYrgRAFATVPIEVSSV-EAGNadqfdtltsdalglvgvpaavavpcxtiPWQIaqklha  191
ident                 |
Sbjct PG--PGRRHRRLDIIVVpYSEF---------------------------aCALL------  244
DSSP  LL--LLLLLEEEEEEEEeHHHH---------------------------hHHHH------
```

```
DSSP  hhllllllllllllHHHHHHH-------------------------hhhhhllhHHHHhh
Query vtavleepkandraHDLVDLQ-------------------------lleglllDADLXpt  226
ident                                                         |
Sbjct ----------yftgSAHFNRSmralaktkgmslsehalstavvrnthgakvgpgRVLP--  292
DSSP  ----------hhhlLHHHHHHhhhhhhllleellllleeelleellllleeellEELL--


DSSP  HHHHHhhhhhhlllLLLLLLllllhhhhhhhhhllllllllllhhhhhhhhhhhhhhh
Query RSACIaifearaqhPWPPRVatlphwpliyagalegldhlelartvdaaaqavqrfvari  286
ident
Sbjct TPTEK-----dvfrLLGLPY--------------------------------------re  309
DSSP  LLLHH-----hhhhHLLLLL--------------------------------------ll


DSSP  hhllll
Query dratkr  292
ident
Sbjct paerdw  315
DSSP  hhhhll
```

No 137: Query=mol1A Sbjct=1bpzA Z-score=6.9

back to top

```
DSSP  ------------------------------------------------------------
Query ------------------------------------------------------------    0
ident
Sbjct kapqetlnggitdmltelanfeknvsqaihkynayrkaasviakyphkiksgaeakklpg   60
DSSP  llllhhhhhhhhhhhhhhhhhhhhhhllhhhhhhhhhhhhhhhhlllllllhhhhhllll


DSSP  -----------------------------------------------------lllllll
Query -----------------------------------------------------aapyssp    7
ident
Sbjct vgtkiaekideflatgklrklekirqddtsssinfltrvsgigpsaarkfvdegiktled  120
DSSP  llhhhhhhhhhhhhhlllhhhhhhhhlhhhhhhhhhlllllllhhhhhhhhhlllllhhh


DSSP  lllhhhhhhhhhhhhhhhllllhhhhhHHHHHHHHHHHHH-LLLLllLLLLEEEEHHHHHH
Query ptnlrslrdrltqvaerqgvvfgrlqRHVAXIVVAQFAA-TLTDdtGAPLLLVKGGSSLE   66
ident                                                     | |
Sbjct lrknedklnhhqriglkyfgdfekriPREEMLQMQDIVLnEVKKvdSEYIATVCGSFRRG  180
DSSP  hhllhhhllhhhhhhhhlhhhhllllLHHHHHHHHHHHHhHHHHhlLLLEEEEELLHHHL


DSSP  HhhllllllLLLLEEEEEL------------lLHHHHHHHHHHHHhhlllleeEEELLLE
Query LrrgipdsrTSKDFATVAR------------rDIELIHEQLADAGetgwegftAIFTAPE  114
ident           | |                         |||             |
Sbjct A-------eSSGDMDVLLThpsftsestkqpkLLHQVVEQLQKVH--------FITDTLS  225
DSSP  L-------lLLLLEEEEEEelllllllllllhHHHHHHHHHHHLL--------LLLLEEE


DSSP  ELllllllLLLEEEEEEEEEL--LEEEEEEEEEEEEL-LLLLlllleeeeelhhhhllll
Query EIaapgxpVKPRRFTAKLSYR--GRAFATVPIEVSSV-EAGNadqfdtltsdalglvgvp  171
ident                  |             |
Sbjct KG------ETKFMGVCQLPSKndEKEYPHRRIDIRLIpKDQY------------------  261
DSSP  LL------LLEEEEEELLLLLllLLLLLLEEEEEEEEeHHHH------------------


DSSP  lleeeeellhHHHHHHHhhhhhlllllllllllhhhhHHHHHHH----------------
Query aavavpcxtiPWQIAQKlhavtavleepkandrahdlvDLQLLE----------------  215
ident
Sbjct ---------yCGVLYFT--------------------gSDIFNKnmrahalekgftiney  292
DSSP  ---------hHHHHHHH--------------------lLHHHHHhhhhhhhhlleeelll


DSSP  -----hhllhhhhhhhHHHHHhhhhhhlllLLLLLLlllllhhhhhhhhhllllllllll
Query -----gllldadlxptRSACIaifearaqhPWPPRVatlphwpliyagalegldhlelar  270
ident
Sbjct tirplgvtgvageplpVDSEK------difDYIQWK------------------------  322
DSSP  leeellllllllllllLLLHH------hhhHHLLLL------------------------


DSSP  lhhhhhhhhhhhhhhhhhllll
Query tvdaaaqavqrfvaridratkr  292
ident
```

```
Sbjct              yrepkdrse  331
DSSP  -------------lllhhhlll
```

No 138: Query=mol1A Sbjct=2fmqA Z-score=6.9

back to top

```
DSSP  ------------------------------------------------------------
Query ------------------------------------------------------------    0
ident
Sbjct tlnggitdmltelanfeknvsqaihkynayrkaasviakyphkiksgaeakklpgvgtki   60
DSSP  lllhhhhhhhhhhhhhhhhhllllhhhhhhhhhhhhhhhhlllllllhhhhllllllhhh


DSSP  ------------------------------------------------llllllllllhh
Query ------------------------------------------------aapysspptnlr   12
ident
Sbjct aekideflatgklrklekirqddtsssinfltrvsgigpsaarkfvdegiktledlrkne  120
DSSP  hhhhhhhhhhlllhhhhhhlllhhhhhhhhhlllllllhhhhhhhhhlllllhhhhhhlh


DSSP  hhhhhhhhhhhhllllhhhhhHHHHHHHHHHHHH-LLLLllLLLLEEEEHHHHHHHhhll
Query slrdrltqvaerqgvvfgrlqRHVAXIVVAQFAA-TLTDdtGAPLLLVKGGSSLELrrgi   71
ident                                                | |
Sbjct dklnhhqriglkyfgdfekriPREEMLQMQDIVLnEVKKvdSEYIATVCGSFRRGA----  176
DSSP  hhllhhhhhhhhlllhhhlleEHHHHHHHHHHHHhHHHHhlLLLEEEELHHHHLLL----


DSSP  llllLLLLEEEEEL------------lLHHHHHHHHHHHHhhlllleeEEELLLEELlll
Query pdsrTSKDFATVAR------------rDIELIHEQLADAGetgwegftAIFTAPEEIaap  119
ident      | |                         |||             |
Sbjct ---eSSGDMDVLLThpsftsestkqpkLLHQVVEQLQKVH--------FITDTLSKG---  222
DSSP  ---lEELLEEEEEElllllllllllllHHHHHHHHHHHLL--------LEEEEEEEL---


DSSP  lllLLLEEEEEEEEEL--LEEEEEEEEEEEEL-LLLLlllleeeeelhhhhlllllleee
Query gxpVKPRRFTAKLSYR--GRAFATVPIEVSSV-EAGNadqfdtltsdalglvgvpaavav  176
ident             |             |
Sbjct ---ETKFMGVCQLPSKndEKEYPHRRIDIRLIpKDQY-----------------------  256
DSSP  ---LLEEEEEELLLLLllLLLLLLEEEEEEELlHHHH-----------------------


DSSP  eellhHHHHhhhhhhhhllllllllllllHHHHHHH-------------------hhhhh
Query pcxtiPWQIaqklhavtavleepkandraHDLVDLQ-------------------llegl  217
ident                               |
Sbjct ----yCGVL----------------yftgSDIFNKNmrahalekgftineytirplgvtg  296
DSSP  ----hHHHH----------------hhhlLHHHHHHhhhhhhhlleeellllleeelllll


DSSP  llhHHHHhhHHHHHhhhhhhllllLLLLLLllllhhhhhhhhhlllllllllllhhhhhh
Query lldADLXptRSACIaifearaqhpWPPRVAtlphwpliyagalegldhlelartvdaaaq  277
ident      |
Sbjct vagEPLP--VDSEK-------difDYIQWK------------------------------  317
DSSP  lllLLLL--LLLHH-------hhhHHLLLL------------------------------


DSSP  hhhhhhhhhhhllll
Query avqrfvaridratkr  292
ident
Sbjct ------yrepkdrse  326
DSSP  ------lllhhhlll
```

No 139: Query=mol1A Sbjct=2bpfA Z-score=6.9

back to top

```
DSSP  ------------------------------------------------------------
Query ------------------------------------------------------------    0
ident
Sbjct etlnggitdmlvelanfeknvsqaihkynayrkaasviakyphkiksgaeakklpgvgtk   60
DSSP  llllhhhhhhhhhhhhhhhhllllhhhhhhhhhhhhhhhhlllllllhhhhhhlllllhh


DSSP  -------------------------------------------------llllllllllh
```

```
Query [illegible]aapysspptnl   11
ident
Sbjct iaekideflatgklrklekirqddtsssinfltrvtgigpsaarklvdegiktledlrkn  120
DSSP  hhhhhhhhhllllhhhhhhhhllhhhhhhhhhlllllhhhhhhhhhllllllhhhhhhl


DSSP  hhhhhhhhhhhhllllhhhhhHHHHHHHHHHHHHHH-LLLLllLLLLEEEEHHHHHHHhhl
Query rslrdrltqvaerqgvvfgrlqRHVAXIVVAQFAA-TLTDdtGAPLLLVKGGSSLELrrg   70
ident                                                   | |
Sbjct edklnhhqriglkyfedfekriPREEMLQMQDIVLnEVKKldPEYIATVCGSFRRGA---  177
DSSP  hhhllhhhhhhhhlhhhhllleEHHHHHHHHHHHHhHHHHhlLLLEEEELHHHHLLL---


DSSP  lllllLLLLEEEEEL------------lLHHHHHHHHHHHHhhlllleeEEELLLEELll
Query ipdsrTSKDFATVAR------------rDIELIHEQLADAGetgwegftAIFTAPEEIaa  118
ident       | |                         |||             |
Sbjct ----eSSGDMDVLLThpnftsesskqpkLLHRVVEQLQKVR--------FITDTLSKG--  223
DSSP  ----lEELLEEEEEEllllllllllllhHHHHHHHHHHLLL--------LEEEEEEEL--


DSSP  llllLLLEEEEEEEEEELLeEEEEEEEEEEL-LLLLlllleeeeelhhhhlllllleeee
Query pgxpVKPRRFTAKLSYRGrAFATVPIEVSSV-EAGNadqfdtltsdalglvgvpaavavp  177
ident              |           |
Sbjct ----ETKFMGVCQLPSEN-EYPHRRIDIRLIpKDQY------------------------  254
DSSP  ----LLEEEEEELLLLLL-LLLLEEEEEEELlHHHH------------------------


DSSP  ellhHHHHhhhhhhhhllllllllllllHHHHHHH-------------------hhhhhl
Query cxtiPWQIaqklhavtavleepkandraHDLVDLQ-------------------llegll  218
ident                              |
Sbjct ---yCGVL----------------yftgSDIFNKNmrahalekgftineytirplgvtgv  295
DSSP  ---hHHHH----------------hhhlLHHHHHHhhhhhhhlleeellllleeellllll


DSSP  lhHHHHhhHHHHHhhhhhhllllLLLLLLllllhhhhhhhhhllllllllllhhhhhhhh
Query ldADLXptRSACIaifearaqhpWPPRVAtlphwpliyagalegldhlelartvdaaaqa  278
ident     |
Sbjct agEPLP--VDSEQ-------difDYIQWR--------------------------------  315
DSSP  llLLLL--LLLHH-------hhhHHLLLL--------------------------------


DSSP  hhhhhhhhhhllll
Query vqrfvaridratkr  292
ident
Sbjct -----yrepkdrse  324
DSSP  -----lllhhhlll
```

No 140: Query=mol1A Sbjct=7icqA Z-score=6.9

back to top

```
DSSP  ------------------------------------------------------------
Query ------------------------------------------------------------    0
ident
Sbjct etlnggitdmltelanfeknvsqaihkynayrkaasviakyphkiksgaeakklpgvgtk   60
DSSP  llllhhhhhhhhhhhhhhhhllllhhhhhhhhhhhhhhhhllllllhhhhhlllllllhh


DSSP  -------------------------------------------------llllllllllh
Query -------------------------------------------------aapysspptnl   11
ident
Sbjct iaekideflatgklrklekirqddtsssinfltrvsgigpsaarkfvdegiktledlrkn  120
DSSP  hhhhhhhhhhllllhhhhhhhhlhhhhhhhhhllllllhhhhhhhhlllllllhhhhhhl


DSSP  hhhhhhhhhhhhllllhhhhhHHHHHHHHHHHHHHH-LLLLllLLLLEEEEHHHHHHHhhl
Query rslrdrltqvaerqgvvfgrlqRHVAXIVVAQFAA-TLTDdtGAPLLLVKGGSSLELrrg   70
ident                                                   | |
Sbjct edklnhhqriglkyfgdfekriPREEMLQMQDIVLnEVKKvdSEYIATVCGSFRRGA---  177
DSSP  hhhllhhhhhhhhlhhhhhlleEHHHHHHHHHHHHhHHHHhlLLLEEEELLLLLLLL---


DSSP  lllllLLLLEEEEEL------------lLHHHHHHHHHHHHhhlllleeEEELLLEELll
Query ipdsrTSKDFATVAR------------rDIELIHEQLADAGetgwegftAIFTAPEEIaa  118
ident       | |                         |||             |
Sbjct ----eSSGDMDVLLThpsftsestkqpkLLHQVVEQLQKVH--------FITDTLSKG--  223
DSSP  ----lEELLEEEEEEellllllllllllhHHHHHHHHHHHLL--------LEEEEEEEL--
```

```
DSSP  llllLLLEEEEEEEEEELL--EEEEEEEEEEEEL-LLLLllllleeeeelhhhhllllllee
Query pgxpVKPRRFTAKLSYRG--RAFATVPIEVSSV-EAGNadqfdtltsdalglvgvpaava  175
ident              |             |
Sbjct ----ETKFMGVCQLPSKNdeKEYPHRRIDIRLIpKDQY----------------------  257
DSSP  ----LLEEEEEELLLLLLllLLLLLLEEEEEEEEeHHHH----------------------


DSSP  eeellhHHHHHHhhhhhhllllllllllllhhhhHHHHHH--------------------
Query vpcxtiPWQIAQklhavtavleepkandrahdlvDLQLLE--------------------  215
ident
Sbjct -----yCGVLYF--------------------tgSDIFNKnmrahalekgftineytirp  292
DSSP  -----hHHHHHH--------------------hlLHHHHHhhhhhhhhlleeellllleee


DSSP  --hhllhhhhhhhhHHHHhhhhhhllllLLLLLlllllhhhhhhhhhllllllllllhh
Query --glldadlxptrSACIaifearaqhpWPPRVatlphwpliyagalegldhlelartvd  273
ident               |
Sbjct lgvtgvageplpvdSEKD-------ifdYIQWK---------------------------  318
DSSP  lllllllllllllLHHH-------hhhHLLLL---------------------------


DSSP  hhhhhhhhhhhhhhhllll
Query aaaqavqrfvaridratkr  292
ident
Sbjct ----------yrepkdrse  327
DSSP  ----------lllhhhlll
```

No 141: Query=mol1A Sbjct=9ictA Z-score=6.9

back to top

```
DSSP  ------------------------------------------------------------
Query ------------------------------------------------------------    0
ident
Sbjct etlnggitdmltelanfeknvsqaihkynayrkaasviakyphkiksgaeakklpgvgtk   60
DSSP  llllhhhhhhhhhhhhhhhhhlllhhhhhhhhhhhhhhhhlllllllhhhhhlllllllhh


DSSP  -------------------------------------------------llllllllllh
Query -------------------------------------------------aapysspptnl   11
ident
Sbjct iaekideflatgklrklekirqddtsssinfltrvsgigpsaarkfvdegiktledlrkn  120
DSSP  hhhhhhhhhhllllhhhhhhlllhhhhhhhhhlllllllhhhhhhhhlllllllhhhhhhl


DSSP  hhhhhhhhhhhhllllhhhhhHHHHHHHHHHHHH-LLLLllLLLLEEEEHHHHHHHHhhl
Query rslrdrltqvaerqgvvfgrlqRHVAXIVVAQFAA-TLTDdtGAPLLLVKGGSSLELrrg   70
ident                                                 | |
Sbjct edklnhhqriglkyfgdfekriPREEMLQMQDIVLnEVKKvdSEYIATVCGSFRRGA---  177
DSSP  lllllhhhhhhhhlhhhhhlleEHHHHHHHHHHHHHhHHHHhlLLLEEEELLHHHHLL---


DSSP  lllllLLLLEEEEEL------------lLHHHHHHHHHHHHHhhllllleeEEELLLEELll
Query ipdsrTSKDFATVAR------------rDIELIHEQLADAGetgwegftAIFTAPEEIaa  118
ident       | |                         |||             |
Sbjct ----eSSGDMDVLLThpsftsestkqpkLLHQVVEQLQKVH--------FITDTLSKG--  223
DSSP  ----lEELLEEEEEEllllllllllllhHHHHHHHHHHHHHLL--------LEEEEEEEL--


DSSP  llllLLLEEEEEEEEEELL--EEEEEEEEEEEEL-LLLLllllleeeeelhhhhllllllee
Query pgxpVKPRRFTAKLSYRG--RAFATVPIEVSSV-EAGNadqfdtltsdalglvgvpaava  175
ident              |             |
Sbjct ----ETKFMGVCQLPSKNdeKEYPHRRIDIRLIpKDQY----------------------  257
DSSP  ----LLEEEEEELLLLLLllLLLLLLEEEEEEEELlHHHH----------------------


DSSP  eeellhHHHHhhhhhhhhllllllllllllhhhhHHHHHH--------------------
Query vpcxtiPWQIaqklhavtavleepkandrahdlvDLQLLE--------------------  215
ident
Sbjct -----yCGVL--------------------yftgSDIFNKnmrahalekgftineytirp  292
DSSP  -----hHHHH--------------------hhhlLHHHHHhhhhhhhhlleeellllleee


DSSP  -hhllhhhhhhhHHHHHhhhhhhllllLLLLLlllllhhhhhhhhhllllllllllhhh
Query -glldadlxptRSACIaifearaqhpWPPRVatlphwpliyagalegldhlelartvda  274
```

```
ident
Sbjct lgvtgvageplpVDSEK-------difDYIQW----------------------------  317
DSSP  llllllllllllLLLHH-------hhhHHLLL----------------------------


DSSP  hhhhhhhhhhhhhhllll
Query aaqavqrfvaridratkr  292
ident
Sbjct --------kyrepkdrse  327
DSSP  --------lllllhhhhl
```

No 142: Query=mol1A Sbjct=9icvA Z-score=6.9

back to top

```
DSSP  ------------------------------------------------------------
Query ------------------------------------------------------------    0
ident
Sbjct etlnggitdmltelanfeknvsqaihkynayrkaasviakyphkiksgaeakklpgvgtk   60
DSSP  llllhhhhhhhhhhhhhhhhhlllhhhhhhhhhhhhhhhhhllllllllhhhhhllllllhh


DSSP  ------------------------------------------------llllllllllh
Query ------------------------------------------------aapyssppptnl   11
ident
Sbjct iaekideflatgklrklekirqddtsssinfltrvsgigpsaarkfvdegiktledlrkn  120
DSSP  hhhhhhhhhhllllhhhhhhhhlhhhhhhhhhllllllhhhhhhhhhhllllhhhhhhl


DSSP  hhhhhhhhhhhhhllllhhhhhHHHHHHHHHHHHH-LLLLllLLLLEEEEHHHHHHHHhhl
Query rslrdrltqvaerqgvvfgrlqRHVAXIVVAQFAA-TLTDdtGAPLLLVKGGSSLELrrg   70
ident                                                 | |
Sbjct edklnhhqriglkyfgdfekriPREEMLQMQDIVLnEVKKvdSEYIATVCGSFRRGA---  177
DSSP  hhhllhhhhhhhhlhhhhhlleEHHHHHHHHHHHHHhHHHHhlLLLEEEELLLLLLL---


DSSP  lllllLLLLEEEEEL------------lLHHHHHHHHHHHHhhlllleeEEELLLEELll
Query ipdsrTSKDFATVAR------------rDIELIHEQLADAGetgwegftAIFTAPEEIaa  118
ident       | |                         |||             |
Sbjct ----eSSGDMDVLLThpsftsestkqpkLLHQVVEQLQKVH--------FITDTLSKG--  223
DSSP  ----lEELLEEEEEEellllllllllhhHHHHHHHHHHHHLL--------LEEEEEEEL--


DSSP  llllLLLEEEEEEEEEELL--EEEEEEEEEEEEL-LLLLlllleeeeelhhhhllllllee
Query pgxpVKPRRFTAKLSYRG--RAFATVPIEVSSV-EAGNadqfdtltsdalglvgvpaava  175
ident              |             |
Sbjct ----ETKFMGVCQLPSKNdeKEYPHRRIDIRLIpKDQY----------------------  257
DSSP  ----LLEEEEEEELLLLLLllLLLLLEEEEEEEEeHHHH----------------------


DSSP  eeellhHHHHhhhhhhhhlllllllllllhhhhHHHHHHH--------------------
Query vpcxtiPWQIaqklhavtavleepkandrahdlvDLQLLE--------------------  215
ident
Sbjct -----yCGVL--------------------yftgSDIFNKnmrahalekgftineytirp  292
DSSP  -----hHHHH--------------------hhhlLHHHHHhhhhhhhhlleeellllleee


DSSP  -hhllhhhhhhhHHHHHHhhhhhllllLLLLLlllllhhhhhhhhhllllllllllhhh
Query -gllldadlxptRSACIAifearaqhpWPPRVatlphwpliyagalegldhlelartvda  274
ident
Sbjct lgvtgvageplpVDSEKD------ifdYIQWK----------------------------  318
DSSP  llllllllllllLLLHHH------hhhHHLLL----------------------------


DSSP  hhhhhhhhhhhhhhllll
Query aaqavqrfvaridratkr  292
ident
Sbjct ---------yrepkdrse  327
DSSP  ---------lllhhhlll
```

No 143: Query=mol1A Sbjct=9ickA Z-score=6.9

back to top

```
DSSP
Query ------------------------------------------------------------    0
ident
Sbjct etlnggitdmltelanfeknvsqaihkynayrkaasviakyphkiksgaeakklpgvgtk   60
DSSP  llllhhhhhhhhhhhhhhhhllllhhhhhhhhhhhhhhhhlllllllhhhhhllllllhh


DSSP  -------------------------------------------------llllllllllh
Query -------------------------------------------------aapyssspptnl   11
ident
Sbjct iaekideflatgklrklekirqddtsssinfltrvsgigpsaarkfvdegiktledlrkn  120
DSSP  hhhhhhhhhhllllhhhhhhhhhlhhhhhhhhhllllllhhhhhhhhhlllllhhhhhhl


DSSP  hhhhhhhhhhhhhllllhhhhhHHHHHHHHHHHHH-LLLLllLLLEEEEHHHHHHHHhhl
Query rslrdrltqvaerqgvvfgrlqRHVAXIVVAQFAA-TLTDdtGAPLLLVKGGSSLELrrg   70
ident                                                 | |
Sbjct edklnhhqriglkyfgdfekriPREEMLQMQDIVLnEVKKvdSEYIATVCGSFRRGA---  177
DSSP  hhhllhhhhhhhhlhhhhhlleEHHHHHHHHHHHHhHHHHhlLLLEEEELLHHHHLL---


DSSP  lllllLLLLEEEEEL------------lLHHHHHHHHHHHHhhlllleeEEELLLEELll
Query ipdsrTSKDFATVAR------------rDIELIHEQLADAGetgwegftAIFTAPEEIaa  118
ident       | |                         |||             |
Sbjct ----eSSGDMDVLLThpsftsestkqpkLLHQVVEQLQKVH--------FITDTLSKG--  223
DSSP  ----lEELLEEEEEEellllllllllllhHHHHHHHHHHHLL--------LEEEEEEEL--


DSSP  llllLLLEEEEEEEEELL--EEEEEEEEEEEEL-LLLLlllleeeeelhhhhllllllee
Query pgxpVKPRRFTAKLSYRG--RAFATVPIEVSSV-EAGNadqfdtltsdalglvgvpaava  175
ident              |             |
Sbjct ----ETKFMGVCQLPSKNdeKEYPHRRIDIRLIpKDQY----------------------  257
DSSP  ----LLEEEEEEELLLLLllLLLLLLEEEEEEEeHHHH----------------------


DSSP  eeellhHHHHhhhhhhhhhlllllllllllhhhhHHHHHH--------------------
Query vpcxtiPWQIaqklhavtavleepkandrahdlvDLQLLE--------------------  215
ident
Sbjct -----yCGVL--------------------yftgSDIFNKnmrahalekgftineytirp  292
DSSP  -----hHHHH--------------------hhhlLHHHHHhhhhhhhlleeellllleee


DSSP  --hhllhhhhhhhhHHHHhhhhhhllllLLLLLlllllhhhhhhhhhlllllllllllhh
Query --glllldadlxptrSACIaifearaqhpWPPRVatlphwpliyagalegldhlelartvd  273
ident               |
Sbjct lgvtgvageplpvdSEKD-------ifdYIQWK----------------------------  318
DSSP  llllllllllllllLHHH-------hhhHLLLL----------------------------


DSSP  hhhhhhhhhhhhhhhllll
Query aaaqavqrfvaridratkr  292
ident
Sbjct ----------yrepkdrse  327
DSSP  ----------llllhhhll
```

No 144: Query=mol1A Sbjct=7ichA Z-score=6.9

back to top

```
DSSP  ------------------------------------------------------------
Query ------------------------------------------------------------    0
ident
Sbjct etlnggitdmltelanfeknvsqaihkynayrkaasviakyphkiksgaeakklpgvgtk   60
DSSP  llllhhhhhhhhhhhhhhhhllllhhhhhhhhhhhhhhhhlllllllhhhhhllllllhh


DSSP  -------------------------------------------------llllllllllh
Query -------------------------------------------------aapyssspptnl   11
ident
Sbjct iaekideflatgklrklekirqddtsssinfltrvsgigpsaarkfvdegiktledlrkn  120
DSSP  hhhhhhhhhhllllhhhhhhhhhlhhhhhhhhhllllllhhhhhhhhhlllllhhhhhhl


DSSP  hhhhhhhhhhhhhllllhhhhhHHHHHHHHHHHHH-LLLLllLLLEEEEHHHHHHHHhhl
Query rslrdrltqvaerqgvvfgrlqRHVAXIVVAQFAA-TLTDdtGAPLLLVKGGSSLELrrg   70
ident                                                 | |
Sbjct edklnhhqriglkyfgdfekriPREEMLQMQDIVLnEVKKvdSEYIATVCGSFRRGA---  177
```

```
DSSP  [illegible]


DSSP  lllllLLLLEEEEEEL------------lLHHHHHHHHHHHHhhlllleeEEELLLEELll
Query ipdsrTSKDFATVAR------------rDIELIHEQLADAGetgwegftAIFTAPEEIaa  118
ident       | |                       |||             |
Sbjct ----eSSGDMDVLLThpsftsestkqpkLLHQVVEQLQKVH--------FITDTLSKG--  223
DSSP  ----lEELLEEEEEElllllllllllhHHHHHHHHHHHHLL--------LEEEEEEEL--


DSSP  llllLLLEEEEEEEEELL--EEEEEEEEEEEEL-LLLLlllleeeeelhhhhllllllee
Query pgxpVKPRRFTAKLSYRG--RAFATVPIEVSSV-EAGNadqfdtltsdalglvgvpaava  175
ident              |             |
Sbjct ----ETKFMGVCQLPSKNdeKEYPHRRIDIRLIpKDQY----------------------  257
DSSP  ----LLEEEEEEELLLLLllLLLLLLEEEEEEEELlHHHH----------------------


DSSP  eeellhHHHHhhhhhhhhlllllllllllhhhhHHHHHH--------------------
Query vpcxtiPWQIaqklhavtavleepkandrahdlvDLQLLE--------------------  215
ident
Sbjct -----yCGVL--------------------yftgSDIFNKnmrahalekgftineytirp  292
DSSP  -----hHHHH--------------------hhhlLHHHHHhhhhhhhhlleeeelleeee


DSSP  -hhllhhhhhhhHHHHHHhhhhhllllLLLLLllllllhhhhhhhhhlllllllllllhhh
Query -gllldadlxptRSACIAifearaqhpWPPRVatlphwpliyagalegldhlelartvda  274
ident
Sbjct lgvtgvageplpVDSEKD------ifdYIQWK----------------------------  318
DSSP  llllllllllllLLLHHH------hhhHLLLL----------------------------


DSSP  hhhhhhhhhhhhhhllll
Query aaqavqrfvaridratkr  292
ident
Sbjct ---------yrepkdrse  327
DSSP  ---------lllhhhlll
```

No 145: Query=mol1A Sbjct=7icvA Z-score=6.9

back to top

```
DSSP  ------------------------------------------------------------
Query ------------------------------------------------------------    0
ident
Sbjct etlnggitdmltelanfeknvsqaihkynayrkaasviakyphkiksgaeakklpgvgtk   60
DSSP  llllhhhhhhhhhhhhhhhhhllllhhhhhhhhhhhhhhhhhllllllllhhhhhllllllhh


DSSP  --------------------------------------------------llllllllllh
Query --------------------------------------------------aapysspptnl   11
ident
Sbjct iaekideflatgklrklekirqddtsssinfltrvsgigpsaarkfvdegiktledlrkn  120
DSSP  hhhhhhhhhhllllhhhhhhhhlhhhhhhhhhllllllllhhhhhhhhllllllhhhhhhl


DSSP  hhhhhhhhhhhhhllllhhhhhHHHHHHHHHHHHH-LLLLllLLLLLEEEEHHHHHHHHhl
Query rslrdrltqvaerqggvvfgrlqRHVAXIVVAQFAA-TLTDdtGAPLLLVKGGSSLELrrg   70
ident                                                 | |
Sbjct edklnhhqriglkyfgdfekriPREEMLQMQDIVLnEVKKvdSEYIATVCGSFRRGA---  177
DSSP  hhhllhhhhhhhhlhhhhhlleEHHHHHHHHHHHHHhHHHHhlLLLEEEEELLLLLLLL---


DSSP  lllllLLLLEEEEEEL------------lLHHHHHHHHHHHHhhlllleeEEELLLEELll
Query ipdsrTSKDFATVAR------------rDIELIHEQLADAGetgwegftAIFTAPEEIaa  118
ident       | |                       |||             |
Sbjct ----eSSGDMDVLLThpsftsestkqpkLLHQVVEQLQKVH--------FITDTLSKG--  223
DSSP  ----lEELLEEEEEEelllllllllllhHHHHHHHHHHHHLL--------LEEEEEEEL--


DSSP  llllLLLEEEEEEEEELL--EEEEEEEEEEEEL-LLLLlllleeeeelhhhhllllllee
Query pgxpVKPRRFTAKLSYRG--RAFATVPIEVSSV-EAGNadqfdtltsdalglvgvpaava  175
ident              |             |
Sbjct ----ETKFMGVCQLPSKNdeKEYPHRRIDIRLIpKDQY----------------------  257
DSSP  ----LLEEEEEEELLLLLllLLLLLLEEEEEEEEeHHHH----------------------


DSSP  eeellhHHHHhhhhhhhhlllllllllllhhhhHHHHHH--------------------
```

```
Query [illegible]
ident
Sbjct -----yCGVL--------------------yftgSDIFNKnmrahalekgftineytirp  292
DSSP  -----hHHHH--------------------hhhlLHHHHHhhhhhhhhlleeelllleee


DSSP  --hhllhhhhhhhhHHHHhhhhhhllllLLLLLlllllhhhhhhhhhllllllllllhh
Query --gllldadlxptrSACIaifearaqhpWPPRVatlphwpliyagalegldhlelartvd  273
ident               |
Sbjct lgvtgvageplpvdSEKD-------ifdYIQWK---------------------------  318
DSSP  llllllllllllllLHHH-------hhhHLLLL---------------------------


DSSP  hhhhhhhhhhhhhhhllll
Query aaaqavqrfvaridratkr  292
ident
Sbjct ----------yrepkdrse  327
DSSP  ----------lllhhhhll
```

No 146: Query=mol1A Sbjct=7icjA Z-score=6.9

back to top

```
DSSP  ------------------------------------------------------------
Query ------------------------------------------------------------    0
ident
Sbjct etlnggitdmltelanfeknvsqaihkynayrkaasviakyphkiksgaeakklpgvgtk   60
DSSP  llllhhhhhhhhhhhhhhhhllllhhhhhhhhhhhhhhhhlllllllhhhhhllllllhh


DSSP  -------------------------------------------------llllllllllh
Query -------------------------------------------------aapysspptnl   11
ident
Sbjct iaekideflatgklrklekirqddtsssinfltrvsgigpsaarkfvdegiktledlrkn  120
DSSP  hhhhhhhhhhllllhhhhhhlllhhhhhhhhhhlllllhhhhhhhhhhllllhhhhhhl


DSSP  hhhhhhhhhhhhhllllhhhhhHHHHHHHHHHHHHH-LLLLllLLLLEEEEHHHHHHHHhhl
Query rslrdrltqvaerqgvvfgrlqRHVAXIVVAQFAA-TLTDdtGAPLLLVKGGSSLELrrg   70
ident                                                 | |
Sbjct edklnhhqriglkyfgdfekriPREEMLQMQDIVLnEVKKvdSEYIATVCGSFRRGA---  177
DSSP  hhhllhhhhhhhhlllhhhlleEHHHHHHHHHHHHHhHHHHhlLLLLLLLLLLLLLLL---


DSSP  lllllLLLLEEEEEEL------------lLHHHHHHHHHHHHhhlllleeEEELLLEELll
Query ipdsrTSKDFATVAR------------rDIELIHEQLADAGetgwegftAIFTAPEEIaa  118
ident       | |                         |||             |
Sbjct ----eSSGDMDVLLThpsftsestkqpkLLHQVVEQLQKVH--------FITDTLSKG--  223
DSSP  ----lEELLEEEEELllllllllllllhHHHHHHHHHHHLL--------LEEEEEEEL--


DSSP  llllLLLEEEEEEEEEELL--EEEEEEEEEEEELLLLLLllleeeeelhhhhllllllleee
Query pgxpVKPRRFTAKLSYRG--RAFATVPIEVSSVEAGNAdqfdtltsdalglvgvpaavav  176
ident              |             |
Sbjct ----ETKFMGVCQLPSKNdeKEYPHRRIDIRLIPKDQY----------------------  257
DSSP  ----LLEEEEEEELLLLLLllLLLLLEEEEEEEELLHHHH----------------------


DSSP  eellhHHHHHHhhhhhhlllllllllllhhhhHHHHHH---------------------
Query pcxtiPWQIAQklhavtavleepkandrahdlvDLQLLE---------------------  215
ident
Sbjct ----yCGVLYF--------------------tgSDIFNKnmrahalekgftineytirpl  293
DSSP  ----hHHHHHH--------------------hlLHHHHHhhhhhhhhlleellllleel


DSSP  -hhllhhhhhhhhHHHHhhhhhhllllLLLLLlllllhhhhhhhhhllllllllllhhh
Query -gllldadlxptrSACIaifearaqhpWPPRVatlphwpliyagalegldhlelartvda  274
ident              |
Sbjct gvtgvageplpvdSEKD-------ifdYIQWK----------------------------  318
DSSP  lllllllllllllLLLH-------hhhHLLLL----------------------------


DSSP  hhhhhhhhhhhhhhllll
Query aaqavqrfvaridratkr  292
ident
Sbjct ---------yrepkdrse  327
DSSP  ---------lllhhhlll
```

No 147: Query=mol1A Sbjct=4gxjA Z-score=6.9

back to top

```
DSSP  ------------------------------------------------------------
Query ------------------------------------------------------------    0
ident 
Sbjct tlnggitdmltelanfeknvsqaihkynayrkaasviakyphkiksgaeakklpgvgtki   60
DSSP  lllhhhhhhhhhhhhhhhhllllhhhhhhhhhhhhhhhllllllllhhhhhllllllhhh


DSSP  ------------------------------------------------llllllllllhh
Query ------------------------------------------------aapysspptnlr   12
ident 
Sbjct aekideflatgklrklekirqddtsssinfltrvsgigpsaarkfvdegiktledlrkne  120
DSSP  hhhhhhhhhhlllhhhhhhhhlhhhhhhhhhhlllllhhhhhhhhhhlllllhhhhhhlh


DSSP  hhhhhhhhhhhhllllhhhhhHHHHHHHHHHHHH-LLLLllLLLLEEEEHHHHHHHhhll
Query slrdrltqvaerqgvvfgrlqRHVAXIVVAQFAA-TLTDdtGAPLLLVKGGSSLELrrgi   71
ident                                                | |
Sbjct dklnhhqriglkyfgdfekriPREEMLQMQDIVLnEVKKvdSEYIATVCGSFRRGA----  176
DSSP  hhllhhhhhhhhlhhhhhlleEHHHHHHHHHHHHhHHHHhlLLLEEEEEEHHHLLL----


DSSP  llllLLLLEEEEEL--------lLHHHHHHHHHHHHhhlllleeEEELLLEELllllllL
Query pdsrTSKDFATVAR--------rDIELIHEQLADAGetgwegftAIFTAPEEIaapgxpV  123
ident      | |                     |||             |
Sbjct ---eSSGDMDVLLThpsftseskLLHQVVEQLQKVH--------FITDTLSKG------E  219
DSSP  ---lEELLEEEEEElllllllllHHHHHHHHHHHLL--------LEEEEEEEL------L


DSSP  LLEEEEEEEEELLEEEEEEEEEEEEL-LLLLlllleeeeelhhhhlllllleeeeellhH
Query KPRRFTAKLSYRGRAFATVPIEVSSV-EAGNadqfdtltsdalglvgvpaavavpcxtiP  182
ident         |           |
Sbjct TKFMGVCQLPSKNDEYPHRRIDIRLIpKDQY---------------------------yC  252
DSSP  LEEEEEELLLLLLLLLLLEEEEEEELlHHHH---------------------------hH


DSSP  HHHhhhhhhhhllllllllllllHHHHHHH--------------hhhhhllhHHHHhhHH
Query WQIaqklhavtavleepkandraHDLVDLQ--------------llegllldADLXptRS  228
ident                         |                             |
Sbjct GVL----------------yftgSDIFNKNmkahalekgftineytirplagEPLP--VD  294
DSSP  HHH----------------hhhlLHHHHHHhhhhhhllleeelllleeelllLLLL--LL


DSSP  HHHhhhhhhllllLLLLLlllllhhhhhhhhhlllllllllllhhhhhhhhhhhhhhhhh
Query ACIaifearaqhpWPPRVatlphwpliyagalegldhlelartvdaaaqavqrfvaridr  288
ident 
Sbjct SEK-------difDYIQW------------------------------------kyrepk  311
DSSP  LHH-------hhhHHHLL------------------------------------llllhh


DSSP  llll
Query atkr  292
ident 
Sbjct drse  315
DSSP  hlll
```

No 148: Query=mol1A Sbjct=5eozA Z-score=6.9

back to top

```
DSSP  ------------------------------------------------------------
Query ------------------------------------------------------------    0
ident 
Sbjct tlnggitdmltelanfeknvsqaihkynayrkaasviakyphkiksgaeakklpgvgtki   60
DSSP  lllhhhhhhhhhhhhhhhhllllhhhhhhhhhhhhhhhllllllllhhhhhllllllhhh


DSSP  ------------------------------------------------llllllllllhh
Query ------------------------------------------------aapysspptnlr   12
ident 
```

```
[illegible]
DSSP  hhhhhhhhhhlllhhhhhhhhlhhhhhhhhhllllllhhhhhhhhhlllllhhhhhhlh


DSSP  hhhhhhhhhhhhllllhhhhhHHHHHHHHHHHHH-LLLLllLLLLEEEEHHHHHHHHhhll
Query slrdrltqvaerqgvvfgrlqRHVAXIVVAQFAA-TLTDdtGAPLLLVKGGSSLELrrgi   71
ident                                                |  |
Sbjct dklnhhqriglkyfgdfekriPREEMLQMQDIVLnEVKKvdSEYIATVCGSFRRGA----  176
DSSP  hhllhhhhhhhhlhhhhhlleEHHHHHHHHHHHHHhHHHHhlLLLEEEELHHHHLLL----


DSSP  llllLLLLEEEEEL------------lLHHHHHHHHHHHHhhlllleeEEELLLEEllll
Query pdsrTSKDFATVAR------------rDIELIHEQLADAGetgwegftAIFTAPEEiaap  119
ident      | |                         |||             |
Sbjct ---eSSGDMDVLLThpsftsestkqpkLLHQVVEQLQKVH--------FITDTLSK----  221
DSSP  ---lEELLEEEEEElllllllllllllHHHHHHHHHHHLL--------LEEEEEEE----


DSSP  lllLLLEEEEEEEEEL--LEEEEEEEEEEEEL-LLLLllllleeeeelhhhhllllllleee
Query gxpVKPRRFTAKLSYR--GRAFATVPIEVSSV-EAGNadqfdtltsdalglvgvpaavav  176
ident             |             |
Sbjct --gETKFMGVCQLPSKndEKEYPHRRIDIRLIpKDQY-----------------------  256
DSSP  --lLLEEEEEELLLLLllLLLLLLLEEEEEEELlHHHH-----------------------


DSSP  eellhHHHHhhhhhhhhllllllllllllHHHH-------------------hhhhhhhh
Query pcxtiPWQIaqklhavtavleepkandraHDLV-------------------dlqllegl  217
ident                               |
Sbjct ----yCGVL----------------yftgSDIFnknmrahalekgftineytirplgvtg  296
DSSP  ----hHHHH----------------hhhlLHHHhhhhhhhhhhlleeellllleeelllll


DSSP  llhHHHHHhhHHHHhhhhhhlllLLLLLLlllllhhhhhhhhhlllllllllllhhhhhh
Query lldADLXPtrSACIaifearaqhPWPPRVatlphwpliyagalegldhlelartvdaaaq  277
ident      |
Sbjct vagEPLPV--DSEK-----difdYIQWKY-------------------------------  318
DSSP  lllLLLLL--LLHH-----hhhhHLLLLL-------------------------------


DSSP  hhhhhhhhhhhllll
Query avqrfvaridratkr  292
ident
Sbjct -------repkdrse  326
DSSP  -------llhhhlll
```

No 149: Query=mol1A Sbjct=4mfcA Z-score=6.9

back to top

```
DSSP  ------------------------------------------------------------
Query ------------------------------------------------------------    0
ident
Sbjct lnggitdmltelanfeknvsqaihkynayrkaasviakyphkiksgaeakklpgvgtkia   60
DSSP  llhhhhhhhhhhhhhhhhhllllhhhhhhhhhhhhhhhhlllllllllhhhhhlllllllhhhh


DSSP  -----------------------------------------------llllllllllhhh
Query -----------------------------------------------aapysspptnlrs   13
ident
Sbjct ekideflatgklrklekirqddtsssinfltrvsgigpsaarkfvdegiktledlrkned  120
DSSP  hhhhhhhhhlllhhhhhhhhlhhhhhhhhhllllllhhhhhhhhhlllllhhhhhllhh


DSSP  hhhhhhhhhhhllllhhhhhHHHHHHHHHHHHHHL-LLLLLLLLLEEEEHHHHHHHHhhlll
Query lrdrltqvaerqgvvfgrlqRHVAXIVVAQFAAT-LTDDTGAPLLLVKGGSSLELrrgip   72
ident                                               |  |
Sbjct klnhhqriglkyfgdfekriPREEMLQMQDIVLNeVKKVDSEYIATVCGSFRRGA-----  175
DSSP  hllhhhhhhhhlhhhhllleEHHHHHHHHHHHHHHhHHHHLLLLEEEELHHHHLLL-----


DSSP  lllLLLLEEEEEL-------lLHHHHHHHHHHHHhhlllleeEEELLLEELlllllLLL
Query dsrTSKDFATVAR-------rDIELIHEQLADAGetgwegftAIFTAPEEIaapgxpVKP  125
ident     | |                    |||             |
Sbjct --eSSGDMDVLLThpsftsekLLHQVVEQLQKVH--------FITDTLSKG------ETK  219
DSSP  --lEELLEEEEEElllllllHHHHHHHHHHHHLL--------LEEEEEEEL------LLE
```

```
DSSP  [illegible]
Query RRFTAKLSYRGRAFATVPIEVSSV-EAGNadqfdtltsdalglvgvpaavavpcxtiPWQ  184
ident       |           |
Sbjct FMGVCQLPSDEKEYPHRRIDIRLIpKDQY---------------------------yCGV  252
DSSP  EEEEELLLLLLLLLLLEEEEEEELlHHHH---------------------------hHHH


DSSP  HhhhhhhhhlllllllllllhhhhHHHHHHH---hllhhhHHHHhhHHHH---------
Query IaqklhavtavleepkandrahdlvDLQLLEG---llldadLXPTrsACIA---------  232
ident                                                  |
Sbjct L--------------------yftgSDIFNKNmrahalekgFTIN-eYTIRplgvtgvag  291
DSSP  H--------------------hhhlLHHHHHHhhhhhhlllEEEL-lLLEEellllllll


DSSP  hHHHH----llllLLLLLlllllhhhhhhhhhllllllllllhhhhhhhhhhhhhhhhhh
Query iFEAR----aqhpWPPRVatlphwpliyagalegldhlelartvdaaaqavqrfvaridr  288
ident
Sbjct ePLPVdsekdifdYIQWK-------------------------------------yrepk  314
DSSP  lLLLLllhhhhhhHLLLL-------------------------------------lllhh


DSSP  llll
Query atkr  292
ident
Sbjct drse  318
DSSP  hlll
```

No 150: Query=mol1A Sbjct=5ddyC Z-score=6.9

back to top

```
DSSP  -llllllllllhhhhhhhhhhhhHHLL---------------------------------
Query -aapysspptnlrslrdrltqvaERQG---------------------------------   26
ident
Sbjct atnhnlhiteklevlakaysvqgDKWRalgyakainalksfhkpvtsyqeacsipgigkr   60
DSSP  llllhhhhhhhhhhhhhhhhhhlLHHHhhhhhhhhhhhhhlllllllhhhhhhlllllll


DSSP  ------------------------------------------------------------
Query ------------------------------------------------------------   26
ident
Sbjct maekiieilesghlrkldhisesvpvlelfsniwgagtktaqmwyqqgfrsledirsqas  120
DSSP  hhhhhhhhhhhlllllllllllhhhhhhhhlllllhhhhhhhhhhlllllhhhhhhll


DSSP  ------------llhhhhhHHHHHHHHHHHHHL-LLLLLLLLEEEEHHHHHHHhhllll
Query ------------vvfgrlqRHVAXIVVAQFAAT-LTDDTGAPLLLVKGGSSLELrrgipd   73
ident                             |             |    |
Sbjct lttqqaiglkhysdflermPREEATEIEQTVQKaAQAFNSGLLCVACGSYRRGK------  174
DSSP  llhhhhhllllhhhhllleEHHHHHHHHHHHHHhHHHHLLLLEEEELLHHHHLL------


DSSP  llLLLLEEEEEL--------lLHHHHHHHHHHHHhhlllleeeEELL-LEELLLLLlLLL
Query srTSKDFATVAR--------rDIELIHEQLADAGetgwegftaIFTA-PEEIAAPGxPVK  124
ident   |  |                       |   |           |         |
Sbjct -aTCGDVDVLIThpdgrshrgIFSRLLDSLRQEG---------FLTDdLVSQEENG-QQQ  223
DSSP  -lEELEEEEEEEllllllllHHHHHHHHHHHHLL---------LLLLlLLLLLEEL-LEE


DSSP  LEEEEEEEEEllEEEEEEEEEEEEL-LLLLlllleeeeelhhhhllllllleeeeellhHH
Query PRRFTAKLSYrgRAFATVPIEVSSV-EAGNadqfdtltsdalglvgvpaavavpcxtiPW  183
ident        |                |
Sbjct KYLGVCRLPG--PGRRHRRLDIIVVpYSEF----------------------------aCA  254
DSSP  EELLEEELLL--LLLLEEEEEEEEELlHHHH----------------------------hHH


DSSP  HHhhhhhhhhlllllllllllHHHHHHHhhhhhllhhhhhhhhhhhhHHHH--------
Query QIaqklhavtavleepkandraHDLVDLQllegllldadlxptrsaciAIFE--------  235
ident
Sbjct LL----------------yftgSAHFNRS-----mralaktkgmslseHALStavvrnth  293
DSSP  HH----------------hhhlLHHHHHH-----hhhhhhhlleeellLLEEellllllll


DSSP  -------------hhlllllLLLLLllllhhhhhhhhhllllllllllhhhhhhhhhhh
Query -------------araqhpwPPRVAtlphwpliyagalegldhlelartvdaaaqavqrf  282
ident
Sbjct gckvgpgrvlptptekdvfrLLGLP-----------------------------------  318
```

```
[illegible]
```

```
DSSP  hhhhhhllll
Query varidratkr  292
ident
Sbjct -yrepaerdw  327
DSSP  -lllhhhlll
```

No 151: Query=mol1A Sbjct=3rjgA Z-score=6.9

back to top

```
DSSP  ------------------------------------------------------------
Query ------------------------------------------------------------    0
ident
Sbjct tlnggitdmltelanfeknvsqaihkynayrkaasviakyphkiksgaeakklpgvgtki   60
DSSP  lllhhhhhhhhhhhhhhhhhllllhhhhhhhhhhhhhhhhllllllhhhhhllllllhhh


DSSP  ------------------------------------------------llllllllllhh
Query ------------------------------------------------aapysspptnlr   12
ident
Sbjct aekideflatgklrklekirqddtsssinfltrvsgigpsaarkfvdegiktledlrkne  120
DSSP  hhhhhhhhhhlllhhhhhhhhlhhhhhhhhhllllllhhhhhhhhhhlllllhhhhhllh


DSSP  hhhhhhhhhhhhllllhhhhhHHHHHHHHHHHHH-LLLLllLLLLEEEEHHHHHHHhhll
Query slrdrltqvaerqgvvfgrlqRHVAXIVVAQFAA-TLTDdtGAPLLLVKGGSSLELrrgi   71
ident                                                | |
Sbjct dklnhhqriglkyfgdfekriPREEMLQMQDIVLnEVKKvdSEYIATVCGSFRRGA----  176
DSSP  hhllhhhhhhhhlhhhhllleEHHHHHHHHHHHHhHHHHhlLLLEEEELHHHHLLL----


DSSP  llllLLLLEEEEEL------------lLHHHHHHHHHHHHhhlllleeEEELLLEELlll
Query pdsrTSKDFATVAR------------rDIELIHEQLADAGetgwegftAIFTAPEEIaap  119
ident      | |                         |||             |
Sbjct ---eSSGDMDVLLThpsftsestkqpkLLHQVVEQLQKVH--------FITDTLSKG---  222
DSSP  ---lEELLEEEEEElllllllllllllHHHHHHHHHHHLL--------LEEEEEEEL---


DSSP  lllLLLEEEEEEEEEL--LEEEEEEEEEEEEL-LLLLlllleeeeelhhhhlllllleee
Query gxpVKPRRFTAKLSYR--GRAFATVPIEVSSV-EAGNadqfdtltsdalglvgvpaavav  176
ident             |             |
Sbjct ---ETKFMGVCQLPSKndEKEYPHRRIDIRLIpKDQY-----------------------  256
DSSP  ---LLEEEEEELLLLLllLLLLLLEEEEEEELlHHHH-----------------------


DSSP  eellhHHHHhhhhhhhhllllllllllllhhhhHHHHHH---------------------
Query pcxtiPWQIaqklhavtavleepkandrahdlvDLQLLE---------------------  215
ident
Sbjct ----yCGVL--------------------yftgSDIFNKnmrahalekgftineytirpl  292
DSSP  ----hHHHH--------------------hhhlLHHHHHhhhhhhhllleeellllleeel


DSSP  -hhllhhhhhhhhHHHHhhhhhhlllLLLLLLllllhhhhhhhhhllllllllllllhhh
Query -glllldadlxptrSACIaifearaqhPWPPRVatlphwpliyagalegldhlelartvda  274
ident              |
Sbjct gvtgvageplpvdSEKD-------ifDYIQWK----------------------------  317
DSSP  lllllllllllllLHHH-------hhHHLLLL----------------------------


DSSP  hhhhhhhhhhhhhhllll
Query aaqavqrfvaridratkr  292
ident
Sbjct ---------yrepkdrse  326
DSSP  ---------lllhhhlll
```

No 152: Query=mol1A Sbjct=1zqwA Z-score=6.9

back to top

```
DSSP  ---------------------------llllllllllhhhhhhhhhhhhhhllllhhhhh
Query ---------------------------aapysspptnlrslrdrltqvaerqgvvfgrlq   33
```

```
ident
Sbjct ddtsssinfltrvtgigpsaarklvdegiktledlrknedklnhhqriglkyfedfekri    60
DSSP  lllhhhhhhhllllllhhhhhhhhhlllllhhhhhhlhhhllhhhhhhhhhlllhhhlle


DSSP  HHHHHHHHHHHHHLL-LLLLLLLEEEEHHHHHHHhhllllllLLLLEEEEEL-------
Query RHVAXIVVAQFAATL-TDDTGAPLLLVKGGSSLELrrgipdsrTSKDFATVAR-------    85
ident                           | |               | |
Sbjct PREEMLQMQDIVLNEvKKLDPEYIATVCGSFRRGA-------eSSGDMDVLLThpnftse   113
DSSP  EHHHHHHHHHHHHHHhHHHLLLLEEEELHHHHLLL-------lEELLEEEEEEllllll


DSSP  -----lLHHHHHHHHHHHHhhlllleeEEELLLEELllllllLLLEEEEEEEEEELLeEEE
Query -----rDIELIHEQLADAGetgwegftAIFTAPEEIaapgxpVKPRRFTAKLSYRGrAFA   140
ident             |||             |                      |
Sbjct sskqpkLLHRVVEQLQKVR--------FITDTLSKG------ETKFMGVCQLPSEN-EYP   158
DSSP  llllhhHHHHHHHHHHHLL--------LEEEEEEL------LLEEEEEELLLLLL-LLL


DSSP  EEEEEEEEL-LLLLlllleeeeelhhhhllllllleeeeellhHHHHhhhhhhhhllllll
Query TVPIEVSSV-EAGNadqfdtltsdalglvgvpaavavpcxtiPWQIaqklhavtavleep   199
ident    |
Sbjct HRRIDIRLIpKDQY---------------------------yCGVL--------------   177
DSSP  LEEEEEEELlHHHH---------------------------hHHHH--------------


DSSP  lllllllhhhhHHHHHH---------------------hhllhhhhhhhHHHHHhhhhhhl
Query kandrahdlvDLQLLE---------------------glllldadlxptRSACIaifeara   238
ident
Sbjct ------yftgSDIFNKnmrahalekgftineytirplgvtgvageplpVDSEQ-------   224
DSSP  ------hhhlLHHHHHhhhhhhhhlleeellllleeellllllllllllLLLHH-------


DSSP  lllLLLLLLllllhhhhhhhhhllllllllllhhhhhhhhhhhhhhhhllll
Query qhpWPPRVAtlphwpliyagalegldhlelartvdaaaqavqrfvaridratkr   292
ident
Sbjct difDYIQWR------------------------------------yrepkdrse   242
DSSP  hhhHHLLLL------------------------------------lllhhhlll
```

No 153: Query=mol1A Sbjct=5j0xA Z-score=6.9

back to top

```
DSSP  ------------------------------------------------------------
Query ------------------------------------------------------------     0
ident
Sbjct apqetlnggitdmltelanfeknvsqaihkynayrkaasviakyphkiksgaeakklpgv    60
DSSP  lllllllhhhhhhhhhhhhhhhhhllllhhhhhhhhhhhhhhhhlllllllllhhhhhlllll


DSSP  ----------------------------------------------------llllllll
Query ----------------------------------------------------aapysspp     8
ident
Sbjct gtkiaekideflatgklrklekirqddtsssinfltrvsgigpsaarkfvdegiktledl   120
DSSP  lhhhhhhhhhhhhhlllhhhhhhhhhlhhhhhhhhhhlllllllhhhhhhhhhllllllhhhh


DSSP  llhhhhhhhhhhhhhhhllllhhhhhHHHHHHHHHHHHHH-LLLLllLLLLEEEEHHHHHHH
Query tnlrslrdrltqvaerqgvvfgrlqRHVAXIVVAQFAA-TLTDdtGAPLLLVKGGSSLEL    67
ident                                                    | |
Sbjct rknedklnhhqriglkyfgdfekriPREEMLQMQDIVLnEVKKvdSEYIATVCGSFRRGA   180
DSSP  hllhhhllhhhhhhhhlhhhhllleEHHHHHHHHHHHHHHhHHHHhlLLLEEEELHHHHLLL


DSSP  hhlllllllLLLLEEEEEL----------lLHHHHHHHHHHHHhhlllleeEEELLLEEll
Query rrgipdsrTSKDFATVAR----------rDIELIHEQLADAGetgwegftAIFTAPEEia   117
ident          | |                       |||             |
Sbjct -------eSSGDMDVLLThpsftsestpkLLHQVVEQLQKVH--------FITDTLSK--   223
DSSP  -------lEELLEEEEEEllllllllllHHHHHHHHHHHLL--------LEEEEEEE--


DSSP  lllllLLLEEEEEEEEEEL--LEEEEEEEEEEEEEL-LLLLlllleeeeelhhhhllllle
Query apgxpVKPRRFTAKLSYR--GRAFATVPIEVSSV-EAGNadqfdtltsdalglvgvpaav   174
ident               |             |
Sbjct ----gETKFMGVCQLPSKndEKEYPHRRIDIRLIpKDQY---------------------   258
DSSP  ----lLLEEEEEELLLLLllLLLLLLEEEEEEELlHHHH---------------------
```

```
DSSP  eeeellhHHHHhhhhhhhhlllllllllllHHHH-----------------hhhhhhhh
Query avpcxtiPWQIaqklhavtavleepkandraHDLV-----------------dlqllegl  217
ident                               |
Sbjct ------yCGVL----------------yftgSDIFnknmrahalekgftineytirplgv  296
DSSP  ------hHHHH----------------hhhlLHHHhhhhhhhhhhlleeellllleeelll


DSSP  llhhhhhhhhHHHHHhhhhhlllLLLLLllllllhhhhhhhhhlllllllllllhhhhhh
Query lldadlxptrSACIAifearaqhPWPPRvatlphwpliyagalegldhlelartvdaaaq  277
ident
Sbjct tgvageplpvDSEKD-----ifdYIQWK--------------------------------  319
DSSP  lllllllllLLHHH-----hhhHLLLL--------------------------------


DSSP  hhhhhhhhhhhllll
Query avqrfvaridratkr  292
ident
Sbjct ------yrepkdrse  328
DSSP  ------lllhhhlll
```

No 154: Query=mol1A Sbjct=4ub1A Z-score=6.9

back to top

```
DSSP  ------------------------------------------------------------
Query ------------------------------------------------------------    0
ident
Sbjct nggitdmltelanfeknvsqaihkynayrkaasviakyphkiksgaeakklpgvgtkiae   60
DSSP  lhhhhhhhhhhhhhhhhhllllhhhhhhhhhhhhhhhhllllllllhhhhhhllllllhhhhh


DSSP  ----------------------------------------------llllllllllhhhh
Query ----------------------------------------------aapyssspptnlrsl   14
ident
Sbjct kideflatgklrklekirqddtsssinfltrvsgigpsaarkfvdegiktledlrknedk  120
DSSP  hhhhhhhhllllhhhhhhhhlhhhhhhhhhhllllllhhhhhhhhhlllllhhhhhhlhhh


DSSP  hhhhhhhhhhllllhhhhhHHHHHHHHHHHHH-LLLLllLLLLEEEEHHHHHHHhhllll
Query rdrltqvaerqgvvfgrlqRHVAXIVVAQFAA-TLTDdtGAPLLLVKGGSSLELrrgipd   73
ident                                             | |
Sbjct lnhhqriglkyfgdfekriPREEMLQMQDIVLnEVKKvdSEYIATVCGSFRRGA------  174
DSSP  llhhhhhhhhlhhhhllleEHHHHHHHHHHHHHhHHHHhlLLLEEEEELHHHLLLL------


DSSP  llLLLLLEEEEEL----------lLHHHHHHHHHHHHHhhllllleeEEELLLEEllllllL
Query srTSKDFATVAR----------rDIELIHEQLADAGetgwegftAIFTAPEEiaapgxpV  123
ident    | |                        |||             |
Sbjct -eSSGDMDVLLThpsftsesqpkLLHQVVEQLQKVH--------FITDTLSK------gE  219
DSSP  -lEELLEEEEEEllllllllllHHHHHHHHHHHHLL--------LEEEEEEE------lL


DSSP  LLEEEEEEEEELLEEEEEEEEEEEEL-LLLLllleeeeelhhhhlllllleeeeellhH
Query KPRRFTAKLSYRGRAFATVPIEVSSV-EAGNadqfdtltsdalglvgvpaavavpcxtiP  182
ident          |            |
Sbjct TKFMGVCQLPSKEKEYPHRRIDIRLIpKDQY---------------------------yC  252
DSSP  LEEEEEELLLLLLLLLLLEEEEEEELlHHHH---------------------------hH


DSSP  HHHhhhhhhhhlllllllllllHHHH-------------------hhhhhhhhllhhHH
Query WQIaqklhavtavleepkandraHDLV-------------------dlqlleglllldaDL  223
ident                        |                                   |
Sbjct GVL----------------yftgSDIFnknmrahalekgftineytirplgvtgvagePL  296
DSSP  HHH----------------hhhlLHHHhhhhhhhhhhlleeellllleeellllllllLL


DSSP  HHhhHHHHHhhhhhlllLLLLLllllllhhhhhhhhhlllllllllllhhhhhhhhhhh
Query XPtrSACIAifearaqhPWPPRvatlphwpliyagalegldhlelartvdaaaqavqrfv  283
ident
Sbjct PV--DSEKD-----ifdYIQWK--------------------------------------  311
DSSP  LL--LLHHH-----hhhHLLLL--------------------------------------


DSSP  hhhhhllll
Query aridratkr  292
ident
```

```
[illegible]
DSSP  ---llllll
```

No 155: Query=mol1A Sbjct=4mffA Z-score=6.9

[back to top](#)

```
DSSP  ------------------------------------------------------------
Query ------------------------------------------------------------    0
ident
Sbjct lnggitdmltelanfeknvsqaihkynayrkaasviakyphkiksgaeakklpgvgtkia   60
DSSP  llhhhhhhhhhhhhhhhhllllhhhhhhhhhhhhhhhlllllllhhhhlllllllhhhh


DSSP  ----------------------------------------------lllllllllllhhh
Query ----------------------------------------------aapyssppptnlrs   13
ident
Sbjct ekideflatgklrklekirqddtsssinfltrvsgigpsaarkfvdegiktledlrkned  120
DSSP  hhhhhhhhhlllhhhhhhhllhhhhhhhhhllllllllhhhhhhhhhlllllhhhhhllhh


DSSP  hhhhhhhhhhllllhHHHHHHHHHHHHHHHHH-LLLLllLLLLEEEEHHHHHHHHhhlll
Query lrdrltqvaerqgvvfGRLQRHVAXIVVAQFAA-TLTDdtGAPLLLVKGGSSLELrrgip   72
ident                                               | |
Sbjct klnhhqriglkyfgdfEKRIPREEMLQMQDIVLnEVKKvdSEYIATVCGSFRRGA-----  175
DSSP  hllhhhhhhhhlllhhHLLEEHHHHHHHHHHHHHhHHHHhlLLLEEEELHHHHHLL-----


DSSP  lllLLLLEEEEEL---------lLHHHHHHHHHHHHHhhllllleeEEELLLEELllllllL
Query dsrTSKDFATVAR---------rDIELIHEQLADAGetgwegftAIFTAPEEIaapgxpV  123
ident     | |                      |||             |
Sbjct --eSSGDMDVLLThpsftsespkLLHQVVEQLQKVH--------FITDTLSKG------E  219
DSSP  --lEELLEEEEEEllllllllllLLHHHHHHHHHLL--------LEEEEEEEL------L


DSSP  LLEEEEEEEEEL-LEEEEEEEEEEEEL-LLLLllllleeeeelhhhhllllllleeeeellh
Query KPRRFTAKLSYR-GRAFATVPIEVSSV-EAGNadqfdtltsdalglvgvpaavavpcxti  181
ident         |            |
Sbjct TKFMGVCQLPSKdEKEYPHRRIDIRLIpKDQY---------------------------y  252
DSSP  LEEEEEELLLLLlLLLLLLEEEEEEEELlHHHH---------------------------h


DSSP  HHHHhhhhhhhhllllllllllllHHHHHHH-------------------hhhhhllhHH
Query PWQIaqklhavtavleepkandraHDLVDLQ-------------------llegllldAD  222
ident                          |
Sbjct CGVL----------------yftgSDIFNKNmrahalekgftineytirplgvtgvagEP  296
DSSP  HHHH----------------hlllLHHHHHHhhhhhhhlleeellllleeellllllllLL


DSSP  HHhhHHHHHHhhhhhllllLLLLLLlllllhhhhhhhhhllllllllllhhhhhhhhhh
Query LXptRSACIAifearaqhpWPPRVatlphwpliyagalegldhlelartvdaaaqavqrf  282
ident |
Sbjct LP--VDSEKD------ifdYIQWK------------------------------------  312
DSSP  LL--LLLHHH------hhhHLLLL------------------------------------


DSSP  hhhhhhllll
Query varidratkr  292
ident
Sbjct -yrepkdrse  321
DSSP  -lllhhhlll
```

No 156: Query=mol1A Sbjct=4m9gA Z-score=6.9

[back to top](#)

```
DSSP  ------------------------------------------------------------
Query ------------------------------------------------------------    0
ident
Sbjct lnggitdmltelanfeknvsqaihkynayrkaasviakyphkiksgaeakklpgvgtkia   60
DSSP  llhhhhhhhhhhhhhhhhllllhhhhhhhhhhhhhhhlllllllhhhhhllllllhhhh


DSSP  ----------------------------------------------lllllllllllhhh
```

```
Query [illegible]
ident
Sbjct ekideflatgklrklekirqddtsssinfltrvsgigpsaarkfvdegiktledlrkned  120
DSSP  hhhhhhhhlllhhhhhhhhlhhhhhhhhhllllllhhhhhhhhhlllllhhhhhllhh


DSSP  hhhhhhhhhhhllllhhHHHHHHHHHHHHHHHHL-LLLLlLLLLEEEEHHHHHHHhhlll
Query lrdrltqvaerqgvvfgRLQRHVAXIVVAQFAAT-LTDDtGAPLLLVKGGSSLELrrgip   72
ident                                               | |
Sbjct klnhhqriglkyfgdfeKRIPREEMLQMQDIVLNeVKKVdSEYIATVCGSFRRGA-----  175
DSSP  hllhhhhhhhhlhhhhlLLEEHHHHHHHHHHHHHHHhHHHHlLLLEEEELHHHHLLL-----


DSSP  lllLLLLEEEEEL------lLHHHHHHHHHHHHHhhlllleeEEELLLEELllllllLLLE
Query dsrTSKDFATVAR------rDIELIHEQLADAGetgwegftAIFTAPEEIaapgxpVKPR  126
ident     | |                   |||             |
Sbjct --eSSGDMDVLLThpsftskLLHQVVEQLQKVH--------FITDTLSKG------ETKF  219
DSSP  --lEELLEEEEEEllllllHHHHHHHHHHHHLL--------LEEEEEEEL------LLEE


DSSP  EEEEEEEElleEEEEEEEEEEEL-LLLLllllleeeeelhhhhllllllleeeeellhHHHH
Query RFTAKLSYrgrAFATVPIEVSSV-EAGNadqfdtltsdalglvgvpaavavpcxtiPWQI  185
ident      |           |
Sbjct MGVCQLPS---EYPHRRIDIRLIpKDQY---------------------------yCGVL  249
DSSP  EEEELLLL---LLLLEEEEEEELlHHHH---------------------------hHHHH


DSSP  hhhhhhhhlllllllllllhhhhHHHHHH----------------------hhllhhhh
Query aqklhavtavleepkandrahdlvDLQLLE----------------------glllldadl  223
ident
Sbjct --------------------yftgSDIFNKnmrahalekgftinkytirplgvtgvagep  289
DSSP  --------------------hhhlLHHHHHhhhhhhhhlleeellllleeellllllllll


DSSP  hhhhHHHHhhhhhhllllLLLLLlllllhhhhhhhhhllllllllllhhhhhhhhhhhh
Query xptrSACIaifearaqhpWPPRVatlphwpliyagalegldhlelartvdaaaqavqrfv  283
ident     |
Sbjct lpvdSEKD-------ifdYIQWK-------------------------------------  305
DSSP  llllLHHH-------hhhHLLLL-------------------------------------


DSSP  hhhhhllll
Query aridratkr  292
ident
Sbjct yrepkdrse  314
DSSP  lllhhhlll
```

No 157: Query=mol1A Sbjct=5j2jA Z-score=6.9

back to top

```
DSSP  ------------------------------------------------------------
Query ------------------------------------------------------------    0
ident
Sbjct tlnggitdmltelanfeknvsqaihkynayrkaasviakyphkiksgaeakklpgvgtki   60
DSSP  lllhhhhhhhhhhhhhhhhhllllhhhhhhhhhhhhhhhhlllllllhhhhhlllllllhhh


DSSP  ------------------------------------------------llllllllllhh
Query ------------------------------------------------aapyssspptnlr   12
ident
Sbjct aekideflatgklrklekirqddtsssinfltrvsgigpsaarkfvdegiktledlrkne  120
DSSP  hhhhhhhhhhlllhhhhhhhhllhhhhhhhhhhllllllhhhhhhhhhhlllllhhhhhllh


DSSP  hhhhhhhhhhhhllllhhhhhHHHHHHHHHHHHHH-LLLLllLLLLEEEEHHHHHHHhhll
Query slrdrltqvaerqgvvfgrlqRHVAXIVVAQFAA-TLTDdtGAPLLLVKGGSSLELrrgi   71
ident                                                | |
Sbjct dklnhhqriglkyfgdfekriPREEMLQMQDIVLnEVKKvdSEYIATVCGSFRRGA----  176
DSSP  hhllhhhhhhhhlllhhhlleEHHHHHHHHHHHHHhHHHHhlLLLEEEELHHHHLLL----


DSSP  llllLLLLEEEEEL------------lLHHHHHHHHHHHHHhhlllleeEEELLLEEllll
Query pdsrTSKDFATVAR------------rDIELIHEQLADAGetgwegftAIFTAPEEiaap  119
ident      | |                         |||             |
Sbjct ---eSSGDMDVLLThpsftsestkqpkLLHQVVEQLQKVH--------FITDTLSK----  221
DSSP  ---lEELLEEEEEEllllllllllllHHHHHHHHHHHHLL--------LEEEEEEE----
```

```
DSSP  lllLLLEEEEEEEEEEL--LEEEEEEEEEEEEL-LLLLllllleeeeelhhhhllllllleee
Query gxpVKPRRFTAKLSYR--GRAFATVPIEVSSV-EAGNadqfdtltsdalglvgvpaavav  176
ident             |             |
Sbjct --gETKFMGVCQLPSKndEKEYPHRRIDIRLIpKDQY-----------------------  256
DSSP  --lLLEEEEEEELLLLLllLLLLLLEEEEEEEELlHHHH-----------------------


DSSP  eellhHHHHhhhhhhhhlllllllllllHHHH-------------------hhhhhhhh
Query pcxtiPWQIaqklhavtavleepkandraHDLV-------------------dlqllegl  217
ident                               |
Sbjct ----yCGVL----------------yftgSDIFnknmrahalekgftineytirplgvtg  296
DSSP  ----hHHHH----------------hhhlLHHHhhhhhhhhhhhleeellllleeellll


DSSP  llhHHHHHhhHHHHhhhhhhlllLLLLLLlllllhhhhhhhhhllllllllllhhhhhh
Query lldADLXPtrSACIaifearaqhPWPPRVatlphwpliyagalegldhlelartvdaaaq  277
ident      |
Sbjct vagEPLPV--DSEK-----difdYIQWKY-------------------------------  318
DSSP  lllLLLLL--LLHH-----hhhhHLLLLL-------------------------------


DSSP  hhhhhhhhhhhllll
Query avqrfvaridratkr  292
ident
Sbjct -------repkdrse  326
DSSP  -------llhhhlll
```

No 158: Query=mol1A Sbjct=5bolA Z-score=6.9

back to top

```
DSSP  ------------------------------------------------------------
Query ------------------------------------------------------------    0
ident
Sbjct tlnggitdmltelanfeknvsqaihkynayrkaasviakyphkiksgaeakklpgvgtki   60
DSSP  lllhhhhhhhhhhhhhhhhllllhhhhhhhhhhhhhhhhlllllllhhhhhhllllllhhh


DSSP  -----------------------------------------------llllllllllhh
Query -----------------------------------------------aapysspptnlr   12
ident
Sbjct aekideflatgklrklekirqddtsssinfltrvsgigpsaarkfvdegiktledlrkne  120
DSSP  hhhhhhhhhhlllhhhhhhhhlhhhhhhhhhlllllllhhhhhhhhhlllllhhhhhhlh


DSSP  hhhhhhhhhhhhllllhhhhhHHHHHHHHHHHHH-LLLLLLLLLLEEEEHHHHHHHHhhll
Query slrdrltqvaerqgvvfgrlqRHVAXIVVAQFAA-TLTDDTGAPLLLVKGGSSLELrrgi   71
ident                                                |  |
Sbjct dklnhhqriglkyfgdfekriPREEMLQMQDIVLnEVKKVDSEYIATVCGSFRRGA----  176
DSSP  hhllhhhhhhhhlhhhhllleEHHHHHHHHHHHHHhHHHHHLLLLEEEEELHHHHLLL----


DSSP  lllLLLLLLEEEEEL------------lLHHHHHHHHHHHHhhlllleeEEELLLEEllll
Query pdsRTSKDFATVAR------------rDIELIHEQLADAGetgwegftAIFTAPEEiaap  119
ident      | |                         |||             |
Sbjct ---ESSGDMDVLLThpsftsestkqpkLLHQVVEQLQKVH--------FITDTLSK----  221
DSSP  ---LEELLEEEEEEllllllllllllHHHHHHHHHHHHLL--------LEEEEEEE----


DSSP  lllLLLEEEEEEEEEEL--LEEEEEEEEEEEEL-LLLLllllleeeeelhhhhllllllleee
Query gxpVKPRRFTAKLSYR--GRAFATVPIEVSSV-EAGNadqfdtltsdalglvgvpaavav  176
ident             |             |
Sbjct --gETKFMGVCQLPSKndEKEYPHRRIDIRLIpKDQY-----------------------  256
DSSP  --lLLEEEEEEELLLLLllLLLLLLEEEEEEEELlHHHH-----------------------


DSSP  eellhHHHH---hhHHHHH-HLLLlllllllllhhhhhhhhhhhllhhHHHHHhhHHHHh
Query pcxtiPWQI---aqKLHAV-TAVLeepkandrahdlvdlqllegllldaDLXPtrSACIa  232
ident                                                   |
Sbjct ----yCGVLyftgSDIFNKnMRAH--alekgftineytirplgvtvagePLPV--DSEK-  307
DSSP  ----hHHHHhhhllHHHHHhHHHH--hhhlleeellllleeelllllllLLLL--LLHH-


DSSP  hhhhhlllLLLLLlllllhhhhhhhhhllllllllllhhhhhhhhhhhhhhhhhllll
Query ifearaqhPWPPRvatlphwpliyagalegldhlelartvdaaaqavqrfvaridratkr  292
```

```
ident
Sbjct ----difdYIQWK--------------------------------------yrepkdrse  325
DSSP  ----hhhhHLLLL--------------------------------------lllhhhlll
```

No 159: Query=mol1A Sbjct=4phaA Z-score=6.9

back to top

```
DSSP  ------------------------------------------------------------
Query ------------------------------------------------------------    0
ident
Sbjct pqetlnggitdmltelanfeknvsqaihkynayrkaasviakyphkiksgaeakklpgvg   60
DSSP  llllllhhhhhhhhhhhhhhhhllllhhhhhhhhhhhhhhhllllllllhhhhhllllll


DSSP  ---------------------------------------------------lllllllll
Query ---------------------------------------------------aapyssppt    9
ident
Sbjct tkiaekideflatgklrklekirqddtsssinfltrvsgigpsaarkfvdegiktledlr  120
DSSP  hhhhhhhhhhhhhlllhhhhhhhllhhhhhhhhhllllllhhhhhhhhhhlllllhhhhl


DSSP  lhhhhhhhhhhhhhhllllhhhhhHHHHHHHHHHHHH-LLLLllLLLLEEEEHHHHHHhh
Query nlrslrdrltqvaerqgvvfgrlqRHVAXIVVAQFAA-TLTDdtGAPLLLVKGGSSLElr   68
ident                                                   | |
Sbjct knedklnhhqriglkyfgdfekriPREEMLQMQDIVLnEVKKvdSEYIATVCGSFRRG--  178
DSSP  llhhhllhhhhhhhhlhhhhhlleEHHHHHHHHHHHHhHHHHhlLLLEEEELHHHHLL--


DSSP  hllllLLLLLLEEEEEL----------lLHHHHHHHHHHHHhhlllleeEEELLLEElll
Query rgipdSRTSKDFATVAR----------rDIELIHEQLADAGetgwegftAIFTAPEEiaa  118
ident         | |                       |||             |
Sbjct -----AESSGDMDVLLThpsftsesqpkLLHQVVEQLQKVH--------FITDTLSK---  222
DSSP  -----LLEELLEEEEEElllllllllllHHHHHHHHHHHLL--------LEEEEEEE---


DSSP  llllLLLEEEEEEEEEL--LEEEEEEEEEEEEL-LLLLlllleeeeelhhhhllllllee
Query pgxpVKPRRFTAKLSYR--GRAFATVPIEVSSV-EAGNadqfdtltsdalglvgvpaava  175
ident              |             |
Sbjct ---gETKFMGVCQLPSKndEKEYPHRRIDIRLIpKDQY----------------------  257
DSSP  ---lLLEEEEEELLLLLllLLLLLEEEEEEEELlHHHH----------------------


DSSP  eeellhHHHHhhhhhhhhllllllllllllHHHH-------------------hhhhhhh
Query vpcxtiPWQIaqklhavtavleepkandraHDLV-------------------dlqlleg  216
ident                                |
Sbjct -----yCGVL----------------yftgSDIFnknmrahalekgftineytirplgvt  296
DSSP  -----hHHHH----------------hhhlLHHHhhhhhhhhlllleellllllleellll


DSSP  hllhHHHHHhhHHHHhhhhhhlllLLLLLLlllllhhhhhhhhhllllllllllllhhhhh
Query llldADLXPtrSACIaifearaqhPWPPRVatlphwpliyagalegldhlelartvdaaa  276
ident       |
Sbjct gvagEPLPV--DSEK-----difdYIQWKY------------------------------  319
DSSP  llllLLLLL--LLHH-----hhhhHLLLLL------------------------------


DSSP  hhhhhhhhhhhhllll
Query qavqrfvaridratkr  292
ident
Sbjct --------repkdrse  327
DSSP  --------llhhhlll
```

No 160: Query=mol1A Sbjct=4klqA Z-score=6.9

back to top

```
DSSP  ------------------------------------------------------------
Query ------------------------------------------------------------    0
ident
Sbjct nggitdmltelanfeknvsqaihkynayrkaasviakyphkiksgaeakklpgvgtkiae   60
DSSP  lhhhhhhhhhhhhhhhhllllhhhhhhhhhhhhhhhllllllllhhhhhllllllhhhhh
```

```
DSSP  [illegible]
Query -----------------------------------------------aapyssspptnlrsl   14
ident
Sbjct kideflatgklrklekirqddtsssinfltrvsgigpsaarkfvdegiktledlrknedk  120
DSSP  hhhhhhhhlllhhhhhhhhhlhhhhhhhhhllllllhhhhhhhhhlllllhhhhhhlhhh


DSSP  hhhhhhhhhhllllhHHHHHHHHHHHHHHHHHH-LLLLllLLLLEEEEHHHHHHHHhhllll
Query rdrltqvaerqgvvfGRLQRHVAXIVVAQFAA-TLTDdtGAPLLLVKGGSSLELrrgipd   73
ident                                              | |
Sbjct lnhhqriglkyfgdfEKRIPREEMLQMQDIVLnEVKKvdSEYIATVCGSFRRGA------  174
DSSP  llhhhhhhhhlhhhhHLLEEHHHHHHHHHHHHHHhHHHHhlLLLEEEEELHHHHLL------


DSSP  llLLLLLEEEEEL------------lLHHHHHHHHHHHHHhhllllleeEEELLLEELlllll
Query srTSKDFATVAR------------rDIELIHEQLADAGetgwegftAIFTAPEEIaapgx  121
ident    | |                     |||             |
Sbjct -eSSGDMDVLLThpsftsestkqpkLLHQVVEQLQKVH--------FITDTLSKG-----  220
DSSP  -lEELLEEEEEEllllllllllllHHHHHHHHHHHHLL--------LEEEEEEEL-----


DSSP  lLLLEEEEEEEEEL--LEEEEEEEEEEEEL-LLLLllllleeeeelhhhhllllllleeeee
Query pVKPRRFTAKLSYR--GRAFATVPIEVSSV-EAGNadqfdtltsdalglvgvpaavavpc  178
ident           |             |
Sbjct -ETKFMGVCQLPSKndEKEYPHRRIDIRLIpKDQY-------------------------  254
DSSP  -LLEEEEEELLLLLllLLLLLLEEEEEEEELlHHHH-------------------------


DSSP  llhHHHHhhhhhhhhllllllllllHHHHHHH-------------------hhhhhll
Query xtiPWQIaqklhavtavleepkandraHDLVDLQ-------------------lleglll  219
ident                             |
Sbjct --yCGVL----------------yftgSDIFNKNmrahalekgftineytirplgvtgva  296
DSSP  --hHHHH----------------hhhlLHHHHHHhhhhhhllleeellllleeellllllll


DSSP  hHHHHhhHHHHHhhhhhhllllLLLLLLllllhhhhhhhhhllllllllllhhhhhhhh
Query dADLXptRSACIaifearaqhpWPPRVAtlphwpliyagalegldhlelartvdaaaqav  279
ident    |
Sbjct gEPLP--VDSEK-------difDYIQWK--------------------------------  315
DSSP  lLLLL--LLLHH-------hhhHHLLLL--------------------------------


DSSP  hhhhhhhhhllll
Query qrfvaridratkr  292
ident
Sbjct ----yrepkdrse  324
DSSP  ----lllhhhlll
```

No 161: Query=mol1A Sbjct=4f5pA Z-score=6.9

back to top

```
DSSP  ------------------------------------------------------------
Query ------------------------------------------------------------    0
ident
Sbjct pqetlnggitdmltelanfeknvsqaihkynayrkaasviakyphkiksgaeakklpgvg   60
DSSP  llhhhhhhhhhhhhhhhhhhhhhllllhhhhhhhhhhhhhhhhllllllllhhhhhllllll


DSSP  ---------------------------------------------------lllllllll
Query ---------------------------------------------------aapyssppt    9
ident
Sbjct tkiaekideflatgklrklekirqddtsssinfltrvsgigpsaarkfvdegiktledlr  120
DSSP  hhhhhhhhhhhhhlllhhhhhhhhhlhhhhhhhhhllllllhhhhhhhhhlllllhhhhh


DSSP  lhhhhhhhhhhhhhhhllllhHHHHHHHHHHHHHHHHHHL-LLLLLLLLLEEEEHHHHHHHh
Query nlrslrdrltqvaerqgvvfGRLQRHVAXIVVAQFAAT-LTDDTGAPLLLVKGGSSLELr   68
ident                                                   | |
Sbjct knedklnhhqriglkyfgdfEKRIPREEMLQMQDIVLNeVKKVDSEYIATVCGSFRRGA-  179
DSSP  llhhhllhhhhhhhhlhhhhHLLEEHHHHHHHHHHHHHHhHHHHLLLLEEEELHHHHLLL-


DSSP  hllllllLLLLEEEEEL---------lLHHHHHHHHHHHHHhhllllleeEEELLLEELlll
Query rgipdsrTSKDFATVAR---------rDIELIHEQLADAGetgwegftAIFTAPEEIaap  119
ident         | |                     |||             |
Sbjct ------eSSGDMDVLLThpsftsespkLLHQVVEQLQKVH--------FITDTLSKG---  222
```

```
[illegible]


DSSP  lllLLLEEEEEEEEEL--LEEEEEEEEEEEEL-LLLLllllleeeeelhhhhlllllleee
Query gxpVKPRRFTAKLSYR--GRAFATVPIEVSSV-EAGNadqfdtltsdalglvgvpaavav  176
ident             |             |
Sbjct ---ETKFMGVCQLPSKndEKEYPHRRIDIRLIpKDQY-----------------------  256
DSSP  ---LLEEEEEEELLLLLllLLLLLLEEEEEEEELlHHHH-----------------------


DSSP  eellhHHHHhhhhhhhhllllllllllllHHHHHHH-------------------hhhhh
Query pcxtiPWQIaqklhavtavleepkandraHDLVDLQ-------------------llegl  217
ident                               |
Sbjct ----yCGVL----------------yftgSDIFNKNmkahalekgftineytirplgvtg  296
DSSP  ----hHHHH----------------hhhlLHHHHHHhhhhhhhlleeellllleeelllll


DSSP  llhHHHHhhHHHHHhhhhhhllllLLLLLllllllhhhhhhhhhllllllllllhhhhhh
Query lldADLXptRSACIaifearaqhpWPPRVatlphwpliyagalegldhlelartvdaaaq  277
ident      |
Sbjct vagEPLP--VDSEK-------difDYIQW-------------------------------  316
DSSP  lllLLLL--LLLHH-------hhhHHLLL-------------------------------


DSSP  hhhhhhhhhhhllll
Query avqrfvaridratkr  292
ident
Sbjct -----kyrepkdrse  326
DSSP  -----llllhhhlll
```

No 162: Query=mol1A Sbjct=5j2hA Z-score=6.9

back to top

```
DSSP  ------------------------------------------------------------
Query ------------------------------------------------------------    0
ident
Sbjct tlnggitdmltelanfeknvsqaihkynayrkaasviakyphkiksgaeakklpgvgtki   60
DSSP  lllhhhhhhhhhhhhhhhhhllllhhhhhhhhhhhhhhhhhlllllllhhhhhlllllhhh


DSSP  ------------------------------------------------lllllllllhh
Query ------------------------------------------------aapyssppptnlr   12
ident
Sbjct aekideflatgklrklekirqddtsssinfltrvsgigpsaarkfvdegiktledlrkne  120
DSSP  hhhhhhhhhhlllhhhhhhhhlhhhhhhhhhllllllhhhhhhhhhlllllhhhhhllh


DSSP  hhhhhhhhhhhlllllhhhhhHHHHHHHHHHHHHH-LLLLLlLLLLEEEEHHHHHHHHhll
Query slrdrltqvaerqgvvfgrlqRHVAXIVVAQFAA-TLTDdtGAPLLLVKGGSSLELrrgi   71
ident                                                |  |
Sbjct dklnhhqriglkyfgdfekriPREEMLQMQDIVLnEVKKvdSEYIATVCGSFRRGA----  176
DSSP  hhllhhhhhhhhlllhhhlleEHHHHHHHHHHHHHhHHHHhlLLLEEEEELHHHHLLL----


DSSP  llllLLLLEEEEEL------------lLHHHHHHHHHHHHhhllllleeEEELLLEELlll
Query pdsrTSKDFATVAR------------rDIELIHEQLADAGetgwegftAIFTAPEEIaap  119
ident      | |                         |||             |
Sbjct ---eSSGDMDVLLThpsftsestkqpkLLHQVVEQLQKVH--------FITDTLSKG---  222
DSSP  ---lEELLEEEEEEllllllllllllllHHHHHHHHHHHHLL--------LEEEEEEEL---


DSSP  lllLLLEEEEEEEEEL--LEEEEEEEEEEEEL-LLLLllllleeeeelhhhhlllllleee
Query gxpVKPRRFTAKLSYR--GRAFATVPIEVSSV-EAGNadqfdtltsdalglvgvpaavav  176
ident             |             |
Sbjct ---ETKFMGVCQLPSKndEKEYPHRRIDIRLIpKDQY-----------------------  256
DSSP  ---LLEEEEEEELLLLLllLLLLLLEEEEEEEELlHHHH-----------------------


DSSP  eellhHHHHhhhhhhhhllllllllllllHHHHHHH-------------------hhhhh
Query pcxtiPWQIaqklhavtavleepkandraHDLVDLQ-------------------llegl  217
ident                               |
Sbjct ----yCGVL----------------yftgSDIFNKNmrahalekgftineytirplgvtg  296
DSSP  ----hHHHH----------------hhhlLHHHHHHhhhhhhhhleeellllleeelllll


DSSP  llhHHHHhhHHHHHhhhhhhllllLLLLLllllllhhhhhhhhhllllllllllhhhhhh
```

```
Query [illegible]
ident      |
Sbjct vagEPLP--VDSEK-------difDYIQW-------------------------------  316
DSSP  lllLLLL--LLLHH-------hhhHHLLL-------------------------------


DSSP  hhhhhhhhhhllll
Query avqrfvaridratkr  292
ident
Sbjct -----kyrepkdrse  326
DSSP  -----llllhhhlll
```

No 163: Query=mol1A Sbjct=4wseA Z-score=6.8

back to top

```
DSSP  ----------------------llllllllllhHHHHhhhhhhhhhllllhhhhhHHHHh
Query ----------------------aapysspptnlRSLRdrltqvaerqgvvfgrlqRHVAx   38
ident                                                        |
Sbjct ekyqtyyttneyqivkeklpdiirdaeikasevLEPT--------------iyekRAIM-   45
DSSP  lleellllllhhhhhhhhhhhhhhhhhhhhhhhLLLL--------------hhhhHHHH-


DSSP  hHHHHHHHLLllllllllEEEEHHHHHHHHHHL---LLLLLL----lLLEEEEElllhhhH
Query iVVAQFAATLtddtgaplLLVKGGSSLELRRG---IPDSRT----sKDFATVArrdielI   91
ident  |   |              | ||  |          |         |
Sbjct eVIKDFIRDH-------qRKVYGGTALNEALKqvnPKDAIYdnysfSDIEFYS-----pT   93
DSSP  hHHHHHHHHH-------lLEEEHHHHHHHHHHHhhlHHHLLLlllllLLEEEEE-----lL


DSSP  HHHHHHHHHHL---llleeeEELLLEELllllllllEEEEEEEeelleEEEEEEEEEEe
Query HEQLADAGETG---wegftaIFTAPEEIaapgxpvkpRRFTAKLsyrgrAFATVPIEVSs  148
ident   |                                               |
Sbjct PVQDLVDLCNIlyrkgykfvQGKDAQHE---------ETYSIFV-----NFQLYCDITYs  139
DSSP  HHHHHHHHHHHhhhllllllEEEELLLL---------LEEEEEL-----LLLEEEEEEEl


DSSP  lllllLLLL-EEEEelhhhhllllllleEEEELLHHHHHHHHHHHHHHLLLlllllLLLL-H
Query veagnADQF-DTLTsdalglvgvpaavAVPCXTIPWQIAQKLHAVTAVLeepkaNDRA-H  206
ident                                          |  |   |       |
Sbjct ptrvfYGIKtIEID-------------GINYTDPHFMLIDYLRMVNQPL--taaGQRWeK  184
DSSP  lhhhhHHLLeEEEL-------------LEEEELHHHHHHHHHHHHHHLLHH--hhhHHHHhH


DSSP  HHHHHHHHHH-HLLHH---------------------------------------------
Query DLVDLQLLEG-LLLDA---------------------------------------------  221
ident        |
Sbjct AFERMYRLLKdYPIEDfdkrldipeppeeiqsyisrikteflsdnklnesflisgieayn  244
DSSP  HHHHHHHHHHhLLLLLlllllllllllhhhhhhhhhhhhllllllllllleeeehhhhhh


DSSP  ------------------------------------------------------------
Query ------------------------------------------------------------  221
ident
Sbjct fyirhaassvnlnnfianvpfselisvnyredvkntynflrmivedkekisvdeyfplfq  304
DSSP  hhhhhllllllllllllllleeeeellhhhhhhhhhhhhhhhlllhhheeeeeeelllll


DSSP  --------hhhhhhhhhhhhhhhHLLLL----------LLLLllllllhhhhhhhhhlll
Query --------dlxptrsaciaifeaRAQHP----------WPPRvatlphwpliyagalegl  263
ident
Sbjct ftgystvikyddhpiiriyegdgYCIPNvktvktvkyvSFQY------------vlmily  352
DSSP  llllleeeeeellleeeeeeeelllLLEEEellllllleeLHHH------------hhhhhh


DSSP  llllllllhhhhhhhhhhhhhHHHHLLL--------------------------------
Query dhlelartvdaaaqavqrfvaRIDRATK--------------------------------  291
ident
Sbjct inkfrahldknkpmyfnygiaISNLVKArniyldqtgksvldntvfkefrtnctgntisf  412
DSSP  hhhhhhhhlllhhhhhhhhhhHHHHHHHhhhhhhhhlllllllllllllllleelllllh


DSSP  ------------------------------------------------------------
Query ------------------------------------------------------------  291
ident
Sbjct trmnrlrllekrkqgkqtsfvytpedffkkdletqakldpskarfkntsgnkimvpkyll  472
DSSP  hhhhhhhhhhhhhhlllllleelhhhhllllhhhhhhllllllleellllllllllhhhll
```

```
DSSP  ----------------------l
Query ----------------------r  292
ident
Sbjct fkidnngniednihseeaeisek  495
DSSP  eeelllllleeellllllleelll
```

No 164: Query=mol1A Sbjct=4tuqA Z-score=6.8

back to top

```
DSSP  ------------------------------------------------------------
Query ------------------------------------------------------------    0
ident
Sbjct tlnggitdmltelanfeknvsqaihkynayrkaasviakyphkiksgaeakklpgvgtki   60
DSSP  lllhhhhhhhhhhhhhhhhllllhhhhhhhhhhhhhhhlllllllllhhhhhllllllhhh


DSSP  ------------------------------------------------llllllllllhh
Query ------------------------------------------------aapysspptnlr   12
ident
Sbjct aekideflatgklrklekirqddtsssinfltrvsgigpsaarkfvdegiktledlrkne  120
DSSP  hhhhhhhhhhlllhhhhhhhhlhhhhhhhhhllllllhhhhhhhhhlllllhhhhhhlh


DSSP  hhhhhhhhhhhhllllhhhhhHHHHHHHHHHHHH-LLLLllLLLLEEEEHHHHHHHhhll
Query slrdrltqvaerqgvvfgrlqRHVAXIVVAQFAA-TLTDdtGAPLLLVKGGSSLELrrgi   71
ident                                                |  |
Sbjct dklnhhqriglkyfgdfekriPREEMLQMQDIVLnEVKKvdSEYIATVCGSFRRGA----  176
DSSP  hhllhhhhhhhhlllhhhlleEHHHHHHHHHHHHHhHHHHhlLLLEEEELHHHHLLL----


DSSP  llllLLLLEEEEEL------------lLHHHHHHHHHHHHhhlllleeEEELLLEEllll
Query pdsrTSKDFATVAR------------rDIELIHEQLADAGetgwegftAIFTAPEEiaap  119
ident      | |                         |||             |
Sbjct ---eSSGDMDVLLThpsftsestkqpkLLHQVVEQLQKVH--------FITDTLSK----  221
DSSP  ---lEELLEEEEEEllllllllllllHHHHHHHHHHHHLL--------LEEEEEEE----


DSSP  lllLLLEEEEEEEEEL--LEEEEEEEEEEEEL-LLLLllllleeeeelhhhhlllllleee
Query gxpVKPRRFTAKLSYR--GRAFATVPIEVSSV-EAGNadqfdtltsdalglvgvpaavav  176
ident             |             |
Sbjct --gETKFMGVCQLPSKndEKEYPHRRIDIRLIpKDQY-----------------------  256
DSSP  --lLLEEEEEEELLLLLllLLLLLLEEEEEEELlHHHH-----------------------


DSSP  eellhHHHHhhhhhhhhlllllllllllHHHH-------------------hhhhhhhh
Query pcxtiPWQIaqklhavtavleepkandraHDLV-------------------dlqllegl  217
ident                               |
Sbjct ----yCGVL----------------yftgSDIFnknmrahalekgftineytirplgvtg  296
DSSP  ----hHHHH----------------hhhlLHHHhhhhhhhhhhhhleeellllleeellll


DSSP  llhHHHHHhhHHHHhhhhhhlllLLLLLLlllllhhhhhhhhhlllllllllllhhhhhh
Query lldADLXPtrSACIaifearaqhPWPPRVatlphwpliyagalegldhlelartvdaaaq  277
ident      |
Sbjct vagEPLPV--DSEK-----difdYIQWKY-------------------------------  318
DSSP  lllLLLLL--LLHH-----hhhhHLLLLL-------------------------------


DSSP  hhhhhhhhhhhllll
Query avqrfvaridratkr  292
ident
Sbjct -------repkdrse  326
DSSP  -------llhhhlll
```

No 165: Query=mol1A Sbjct=4o5kA Z-score=6.8

back to top

```
DSSP  ------------------------------------------------------------
Query ------------------------------------------------------------    0
ident
```

```
[illegible]
DSSP  lllhhhhhhhhhhhhhhhhllllhhhhhhhhhhhhhhhlllllllllhhhhhllllllhhh


DSSP  ------------------------------------------------llllllllllhh
Query ------------------------------------------------aapysspptnlr   12
ident
Sbjct aekideflatgklrklekirqddtsssinfltrvsgigpsaarkfvdegiktledlrkne  120
DSSP  hhhhhhhhhhlllhhhhhhhhlhhhhhhhhhlllllllhhhhhhhhhlllllhhhhhllh


DSSP  hhhhhhhhhhhhllllhhhhhHHHHHHHHHHHHHH-LLLLllLLLLEEEEHHHHHHHHhhll
Query slrdrltqvaerqgvvfgrlqRHVAXIVVAQFAA-TLTDdtGAPLLLVKGGSSLELrrgi   71
ident                                                |  |
Sbjct dklnhhqriglkyfgdfekriPREEMLQMQDIVLnEVKKvdSEYIATVCGSFRRGA----  176
DSSP  hhllhhhhhhhhlllhhhhlleEHHHHHHHHHHHHHhHHHHhlLLLEEEEELHHHHLLL----


DSSP  llllLLLLEEEEEL------------lLHHHHHHHHHHHHHhhllllleeEEELLLEEllll
Query pdsrTSKDFATVAR------------rDIELIHEQLADAGetgwegftAIFTAPEEiaap  119
ident      | |                         |||             |
Sbjct ---eSSGDMDVLLThpsftsestkqpkLLHQVVEQLQKVH--------FITDTLSK----  221
DSSP  ---lEELLEEEEEEllllllllllllHHHHHHHHHHHHLL--------LEEEEEEE----


DSSP  lllLLLEEEEEEEEEL--LEEEEEEEEEEEEL-LLLLlllleeeeelhhhhllllllleee
Query gxpVKPRRFTAKLSYR--GRAFATVPIEVSSV-EAGNadqfdtltsdalglvgvpaavav  176
ident             |             |
Sbjct --gETKFMGVCQLPSKndEKEYPHRRIDIRLIpKDQY-----------------------  256
DSSP  --lLLEEEEEELLLLLllLLLLLLEEEEEEELlHHHH-----------------------


DSSP  eellhHHHHhhhhhhhhllllllllllllHHHH-------------------hhhhhhhh
Query pcxtiPWQIaqklhavtavleepkandraHDLV-------------------dlqllegl  217
ident                               |
Sbjct ----yCGVL----------------yftgSDIFnknmrahalekgftineytirplgvtg  296
DSSP  ----hHHHH----------------hhhlLHHHhhhhhhhhhhhlleeellllleeellll


DSSP  llhHHHHHhhHHHHhhhhhhlllLLLLLLlllllhhhhhhhhhllllllllllhhhhhh
Query lldADLXPtrSACIaifearaqhPWPPRVatlphwpliyagalegldhlelartvdaaaq  277
ident      |
Sbjct vagEPLPV--DSEK-----difdYIQWKY-------------------------------  318
DSSP  lllLLLLL--LLHH-----hhhhHLLLLL-------------------------------


DSSP  hhhhhhhhhhhllll
Query avqrfvaridratkr  292
ident
Sbjct -------repkdrse  326
DSSP  -------llhhhlll
```

No 166: Query=mol1A Sbjct=5j2eA Z-score=6.8

[back to top]

```
DSSP  ------------------------------------------------------------
Query ------------------------------------------------------------    0
ident
Sbjct tlnggitdmltelanfeknvsqaihkynayrkaasviakyphkiksgaeakklpgvgtki   60
DSSP  lllhhhhhhhhhhhhhhhhllllhhhhhhhhhhhhhhhlllllllllhhhhhllllllhhh


DSSP  ------------------------------------------------llllllllllhh
Query ------------------------------------------------aapysspptnlr   12
ident
Sbjct aekideflatgklrklekirqddtsssinfltrvsgigpsaarkfvdegiktledlrkne  120
DSSP  hhhhhhhhhhlllhhhhhhhhlhhhhhhhhhlllllllhhhhhhhhhlllllhhhhhllh


DSSP  hhhhhhhhhhhhllllhhhhhHHHHHHHHHHHHHH-LLLLllLLLLEEEEHHHHHHHHhhll
Query slrdrltqvaerqgvvfgrlqRHVAXIVVAQFAA-TLTDdtGAPLLLVKGGSSLELrrgi   71
ident                                                |  |
Sbjct dklnhhqriglkyfgdfekriPREEMLQMQDIVLnEVKKvdSEYIATVCGSFRRGA----  176
DSSP  hhllhhhhhhhhlhhhhllleEHHHHHHHHHHHHHhHHHHhlLLLEEEEELHHHHLLL----
```

```
DSSP  [illegible]
Query pdsrTSKDFATVAR------------rDIELIHEQLADAGetgwegftAIFTAPEEiaap  119
ident      | |                         |||             |
Sbjct ---eSSGDMDVLLThpsftsestkqpkLLHQVVEQLQKVH--------FITDTLSK----  221
DSSP  ---lEELLEEEEEEllllllllllllHHHHHHHHHHHHLL--------LEEEEEEE----


DSSP  lllLLLEEEEEEEEEL--LEEEEEEEEEEEL-LLLLlllleeeeelhhhhllllllleee
Query gxpVKPRRFTAKLSYR--GRAFATVPIEVSSV-EAGNadqfdtltsdalglvgvpaavav  176
ident             |             |
Sbjct --gETKFMGVCQLPSKndEKEYPHRRIDIRLIpKDQY-----------------------  256
DSSP  --lLLEEEEEEELLLLLllLLLLLLEEEEEEEELlHHHH-----------------------


DSSP  eellhHHHHhhhhhhhhllllllllllllHHHH-------------------hhhhhhhh
Query pcxtiPWQIaqklhavtavleepkandraHDLV-------------------dlqllegl  217
ident                               |
Sbjct ----yCGVL----------------yftgSDIFnknmrahalekgftineytirplgvtg  296
DSSP  ----hHHHH----------------hhhlLHHHhhhhhhhhhhlleeellllleeellll


DSSP  llhHHHHHhhHHHHhhhhhhlllLLLLLLLlllllhhhhhhhhhllllllllllhhhhhh
Query lldADLXPtrSACIaifearaqhPWPPRVatlphwpliyagalegldhlelartvdaaaq  277
ident      |
Sbjct vagEPLPV--DSEK-----difdYIQWKY-------------------------------  318
DSSP  lllLLLLL--LLHH-----hhhhHLLLLL-------------------------------


DSSP  hhhhhhhhhhhllll
Query avqrfvaridratkr  292
ident
Sbjct -------repkdrse  326
DSSP  -------llhhhlll
```

No 167: Query=mol1A Sbjct=5j2gA Z-score=6.8

back to top

```
DSSP  ------------------------------------------------------------
Query ------------------------------------------------------------    0
ident
Sbjct tlnggitdmltelanfeknvsqaihkynayrkaasviakyphkiksgaeakklpgvgtki   60
DSSP  lllhhhhhhhhhhhhhhhhhllllhhhhhhhhhhhhhhhllllllllhhhhhllllllhhh


DSSP  ------------------------------------------------llllllllllhh
Query ------------------------------------------------aapysspptnlr   12
ident
Sbjct aekideflatgklrklekirqddtsssinfltrvsgigpsaarkfvdegiktledlrkne  120
DSSP  hhhhhhhhhhlllhhhhhhhhlhhhhhhhhhlllllllhhhhhhhhhlllllhhhhhllh


DSSP  hhhhhhhhhhhhllllhhhhhHHHHHHHHHHHHH-LLLLllLLLLEEEEHHHHHHHHhhll
Query slrdrltqvaerqgvvfgrlqRHVAXIVVAQFAA-TLTDdtGAPLLLVKGGSSLELrrgi   71
ident                                                |  |
Sbjct dklnhhqriglkyfgdfekriPREEMLQMQDIVLnEVKKvdSEYIATVCGSFRRGA----  176
DSSP  hhllhhhhhhhhlhhhhhlleEHHHHHHHHHHHHHhHHHHhlLLLEEEEELHHHHLLL----


DSSP  llllLLLLEEEEEEL------------lLHHHHHHHHHHHHHhhllllleeEEELLLEElllll
Query pdsrTSKDFATVAR------------rDIELIHEQLADAGetgwegftAIFTAPEEiaap  119
ident      | |                         |||             |
Sbjct ---eSSGDMDVLLThpsftsestkqpkLLHQVVEQLQKVH--------FITDTLSK----  221
DSSP  ---lEELLEEEEEElllleelleellllHHHHHHHHHHHHLL--------LEEEEEEE----


DSSP  lllLLLEEEEEEEEEL--LEEEEEEEEEEEL-LLLLlllleeeeelhhhhllllllleee
Query gxpVKPRRFTAKLSYR--GRAFATVPIEVSSV-EAGNadqfdtltsdalglvgvpaavav  176
ident             |             |
Sbjct --gETKFMGVCQLPSKndEKEYPHRRIDIRLIpKDQY-----------------------  256
DSSP  --lLLEEEEEEELLLLLllLLLLLLEEEEEEEELlHHHH-----------------------


DSSP  eellhHHHHhhhhhhhhllllllllllllHHHH-------------------hhhhhhhh
Query pcxtiPWQIaqklhavtavleepkandraHDLV-------------------dlqllegl  217
ident                               |
Sbjct ----yCGVL----------------yftgSDIFnknmrahalekgftineytirplgvtg  296
```

[illegible]

```
DSSP  llhHHHHHhhHHHHhhhhhhlllLLLLLLlllllhhhhhhhhhllllllllllhhhhhh
Query lldADLXPtrSACIaifearaqhPWPPRVatlphwpliyagalegldhlelartvdaaaq  277
ident      |
Sbjct vagEPLPV--DSEK-----difdYIQWKY--------------------------------  318
DSSP  lllLLLLL--LLHH-----hhhhHLLLLL--------------------------------


DSSP  hhhhhhhhhhhllll
Query avqrfvaridratkr  292
ident
Sbjct -------repkdrse  326
DSSP  -------llhhhlll
```

No 168: Query=mol1A Sbjct=5j2kA Z-score=6.8

back to top

```
DSSP  ------------------------------------------------------------
Query ------------------------------------------------------------    0
ident
Sbjct tlnggitdmltelanfeknvsqaihkynayrkaasviakyphkiksgaeakklpgvgtki   60
DSSP  lllhhhhhhhhhhhhhhhhllllhhhhhhhhhhhhhhhllllllllhhhhhhllllllhhh


DSSP  ------------------------------------------------llllllllllhh
Query ------------------------------------------------aapysspptnlr   12
ident
Sbjct aekideflatgklrklekirqddtsssinfltrvsgigpsaarkfvdegiktledlrkne  120
DSSP  hhhhhhhhhhlllhhhhhhhhlhhhhhhhhhlllllllhhhhhhhhhlllllhhhhhhlh


DSSP  hhhhhhhhhhhhllllhhhhhHHHHHHHHHHHHHH-LLLLllLLLLEEEEHHHHHHHhhll
Query slrdrltqvaerqgvvfgrlqRHVAXIVVAQFAA-TLTDdtGAPLLLVKGGSSLELrrgi   71
ident                                                |  |
Sbjct dklnhhqriglkyfgdfekriPREEMLQMQDIVLnEVKKvdSEYIATVCGSFRRGA----  176
DSSP  hhllhhhhhhhhlllhhhlleEHHHHHHHHHHHHHhHHHHhlLLLEEEELHHHHLLL----


DSSP  llllLLLLEEEEEL------------lLHHHHHHHHHHHHhhlllleeEEELLLEElll
Query pdsrTSKDFATVAR------------rDIELIHEQLADAGetgwegftAIFTAPEEiaap  119
ident      | |                         |||             |
Sbjct ---eSSGDMDVLLThpsftsestkqpkLLHQVVEQLQKVH--------FITDTLSK----  221
DSSP  ---lEELLEEEEEEllllllllllllHHHHHHHHHHHHLL--------LEEEEEEE----


DSSP  lllLLLEEEEEEEEEL--LEEEEEEEEEEEEL-LLLLlllleeeeelhhhhllllllleee
Query gxpVKPRRFTAKLSYR--GRAFATVPIEVSSV-EAGNadqfdtltsdalglvgvpaavav  176
ident             |             |
Sbjct --gETKFMGVCQLPSKndEKEYPHRRIDIRLIpKDQY-----------------------  256
DSSP  --lLLEEEEEEELLLLLllLLLLLLEEEEEEEELlHHHH-----------------------


DSSP  eellhHHHHhhhhhhhhllllllllllHHHH-------------------hhhhhhhh
Query pcxtiPWQIaqklhavtavleepkandraHDLV-------------------dlqllegl  217
ident                               |
Sbjct ----yCGVL----------------yftgSDIFnknmrahalekgftineytirplgvtg  296
DSSP  ----hHHHH----------------hhhlLHHHhhhhhhhhhhhhleeellllleeelllll


DSSP  llhHHHHHhhHHHHhhhhhhlllLLLLLLlllllhhhhhhhhhllllllllllhhhhhh
Query lldADLXPtrSACIaifearaqhPWPPRVatlphwpliyagalegldhlelartvdaaaq  277
ident      |
Sbjct vagEPLPV--DSEK-----difdYIQWKY--------------------------------  318
DSSP  lllLLLLL--LLHH-----hhhhHLLLLL--------------------------------


DSSP  hhhhhhhhhhhllll
Query avqrfvaridratkr  292
ident
Sbjct -------repkdrse  326
DSSP  -------llhhhlll
```

No 169: Query=mol1A Sbjct=4k1nA Z-score=6.8

back to top

```
DSSP  ------------------------------------------------------------
Query ------------------------------------------------------------    0
ident
Sbjct tlnggitdmltelanfeknvsqaihkynayrkaasviakyphkiksgaeakklpgvgtki   60
DSSP  lllhhhhhhhhhhhhhhhhllllhhhhhhhhhhhhhhhhlllllllhhhhhlllllllhhh


DSSP  ------------------------------------------------llllllllllhh
Query ------------------------------------------------aapyssppptnlr   12
ident
Sbjct aekideflatgklrklekirqddtsssinfltrvsgigpsaarkfvdegiktledlrkne  120
DSSP  hhhhhhhhhhlllhhhhhhhhlhhhhhhhhhlllllllhhhhhhhhhhllllhhhhhhlh


DSSP  hhhhhhhhhhhlllllhhhhhHHHHHHHHHHHHHH-LLLLllLLLLEEEEHHHHHHHHhhll
Query slrdrltqvaerqgvvfgrlqRHVAXIVVAQFAA-TLTDdtGAPLLLVKGGSSLELrrgi   71
ident                                                  | |
Sbjct dklnhhqriglkyfgdfekriPREEMLQMQDIVLnEVKKvdSEYIATVCGSFRRGA----  176
DSSP  hhllhhhhhhhhlhhhhllleEHHHHHHHHHHHHHhHHHHhlLLLEEEEEELLLLLL----


DSSP  llllLLLLEEEEEL------------lLHHHHHHHHHHHHHhhlllleeEEELLLEELlll
Query pdsrTSKDFATVAR------------rDIELIHEQLADAGetgwegftAIFTAPEEIaap  119
ident       | |                           |||              |
Sbjct ---eSSGDMDVLLThpsftsestkqpkLLHQVVEQLQKVH--------FITDTLSKG---  222
DSSP  ---lEELLEEEEEEllllllllllllHHHHHHHHHHHLL--------LEEEEEEEL---


DSSP  lllLLLEEEEEEEEEL--LEEEEEEEEEEEEL-LLLLllllleeeeelhhhhllllllleee
Query gxpVKPRRFTAKLSYR--GRAFATVPIEVSSV-EAGNadqfdtltsdalglvgvpaavav  176
ident              |              |
Sbjct ---ETKFMGVCQLPSKndEKEYPHRRIDIRLIpKDQY----------------------  256
DSSP  ---LLEEEEEEELLLLLllLLLLLLLEEEEEEEELlHHHH----------------------


DSSP  eellhHHHHhhhhhhhhlllllllllllHHHHHHH------------------hhhhh
Query pcxtiPWQIaqklhavtavleepkandraHDLVDLQ------------------llegl  217
ident                               |
Sbjct ----yCGVL----------------yftgSDIFNKNmrahalekgftineytirplgvtg  296
DSSP  ----hHHHH----------------hhhlLHHHHHHhhhhhhhlleeellllleeellllll


DSSP  llhHHHHhhHHHHHhhhhhhllllLLLLLllllllhhhhhhhhhllllllllllllhhhhhh
Query lldADLXptRSACIaifearaqhpWPPRVatlphwpliyagalegldhlelartvdaaaq  277
ident      |
Sbjct vagEPLP--VDSEK-------difDYIQW------------------------------  316
DSSP  lllLLLL--LLLHH-------hhhHHLLL------------------------------


DSSP  hhhhhhhhhhhllll
Query avqrfvaridratkr  292
ident
Sbjct -----kyrepkdrse  326
DSSP  -----llllhhhlll
```

No 170: Query=mol1A Sbjct=4jwmA Z-score=6.8

back to top

```
DSSP  ------------------------------------------------------------
Query ------------------------------------------------------------    0
ident
Sbjct tlnggitdmltelanfeknvsqaihkynayrkaasviakyphkiksgaeakklpgvgtki   60
DSSP  lllhhhhhhhhhhhhhhhhllllhhhhhhhhhhhhhhhhlllllllhhhhhlllllllhhh


DSSP  ------------------------------------------------llllllllllhh
Query ------------------------------------------------aapyssppptnlr   12
ident
Sbjct aekideflatgklrklekirqddtsssinfltrvsgigpsaarkfvdegiktledlrkne  120
DSSP  hhhhhhhhhhlllhhhhhhhhlhhhhhhhhhlllllllhhhhhhhhhlllllhhhhhhlh
```

```
DSSP  hhhhhhhhhhhhllllhhhhhHHHHHHHHHHHHH-LLLLllLLLLEEEEHHHHHHHhhll
Query slrdrltqvaerqgvvfgrlqRHVAXIVVAQFAA-TLTDdtGAPLLLVKGGSSLELrrgi   71
ident                                                |  |
Sbjct dklnhhqriglkyfgdfekriPREEMLQMQDIVLnEVKKvdSEYIATVCGSFRRGA----  176
DSSP  hhllhhhhhhhhlhhhhhlleEHHHHHHHHHHHHhHHHHhlLLLEEEELHHHHLLL----


DSSP  llllLLLLEEEEEL------------lLHHHHHHHHHHHHhhllllleeEEELLLEELlll
Query pdsrTSKDFATVAR------------rDIELIHEQLADAGetgwegftAIFTAPEEIaap  119
ident      | |                         |||             |
Sbjct ---eSSGDMDVLLThpsftsestkqpkLLHQVVEQLQKVH--------FITDTLSKG---  222
DSSP  ---lEELLEEEEEElllleelleellllHHHHHHHHHHHHLL--------LEEEEEEEL---


DSSP  lllLLLEEEEEEEEEL--LEEEEEEEEEEEL-LLLLllllleeeeelhhhhllllllleee
Query gxpVKPRRFTAKLSYR--GRAFATVPIEVSSV-EAGNadqfdtltsdalglvgvpaavav  176
ident             |             ||
Sbjct ---ETKFMGVCQLPSKndEKEYPHRRIEIRLIpKDQY-----------------------  256
DSSP  ---LLEEEEEEELLLLllLLLLLLLEEEEEEEELlHHHH-----------------------


DSSP  eellhHHHHhhhhhhhhlllllllllllHHHHHHH-------------------hhhhh
Query pcxtiPWQIaqklhavtavleepkandraHDLVDLQ-------------------llegl  217
ident                               |
Sbjct ----yCGVL----------------yftgSDIFNKNmrahalekgftineytirplgvtg  296
DSSP  ----hHHHH----------------hhhlLHHHHHHhhhhhhhhleeellllleeellll


DSSP  llhHHHHhhHHHHHhhhhhhllllLLLLLlllllhhhhhhhhhlllllllllllhhhhhh
Query lldADLXptRSACIaifearaqhpWPPRVatlphwpliyagalegldhlelartvdaaaq  277
ident      |
Sbjct vagEPLP--VDSEK-------difDYIQW-------------------------------  316
DSSP  lllLLLL--LLLHH-------hhhHHLLL-------------------------------


DSSP  hhhhhhhhhhhllll
Query avqrfvaridratkr  292
ident
Sbjct -----kyrepkdrse  326
DSSP  -----llllhhhlll
```

No 171: Query=mol1A Sbjct=3rjiA Z-score=6.8

back to top

```
DSSP  ------------------------------------------------------------
Query ------------------------------------------------------------    0
ident
Sbjct tlnggitdmltelanfeknvsqaihkynayrkaasviakyphkiksgaeakklpgvgtki   60
DSSP  lllhhhhhhhhhhhhhhhhllllhhhhhhhhhhhhhhhhllllllllhhhhhllllllhhh


DSSP  ------------------------------------------------llllllllllhh
Query ------------------------------------------------aapysspptnlr   12
ident
Sbjct aekideflatgklrklekirqddtsssinfltrvsgigpsaarkfvdegiktledlrkne  120
DSSP  hhhhhhhhhhlllhhhhhhhhlhhhhhhhhhlllllllhhhhhhhhhhllllhhhhhllh


DSSP  hhhhhhhhhhhhllllhhhhhHHHHHHHHHHHHH-LLLLllLLLLEEEEHHHHHHHhhll
Query slrdrltqvaerqgvvfgrlqRHVAXIVVAQFAA-TLTDdtGAPLLLVKGGSSLELrrgi   71
ident                                                |  |
Sbjct dklnhhqriglkyfgdfekriPREEMLQMQDIVLnEVKKvdSEYIATVCGSFRRGA----  176
DSSP  hhllhhhhhhhhlhhhhhlleEHHHHHHHHHHHHhHHHHhlLLLEEEELHHHHLLL----


DSSP  llllLLLLEEEEEL------------lLHHHHHHHHHHHHhhllllleeEEELLLEELlll
Query pdsrTSKDFATVAR------------rDIELIHEQLADAGetgwegftAIFTAPEEIaap  119
ident      | |                         |||             |
Sbjct ---eSSGDMDVLLThpsftsestkqpkLLHQVVEQLQKVH--------FITDTLSKG---  222
DSSP  ---lEELLEEEEEEllllllllllllllHHHHHHHHHHHHLL--------LEEEEEEEL---


DSSP  lllLLLEEEEEEEEEL--LEEEEEEEEEEEL-LLLLllllleeeeelhhhhllllllleee
Query gxpVKPRRFTAKLSYR--GRAFATVPIEVSSV-EAGNadqfdtltsdalglvgvpaavav  176
ident             |             |
```

```
[illegible]
DSSP  ---LLEEEEEEELLLLLllLLLLLLEEEEEEEELlHHHH----------------------


DSSP  eellhHHHHhhhhhhhhlllllllllllHHHHHHH-------------------hhhhh
Query pcxtiPWQIaqklhavtavleepkandraHDLVDLQ-------------------llegl  217
ident                               |
Sbjct ----yCGVL----------------yftgSDIFNKNmrahalekgftineytirplgvtg  296
DSSP  ----hHHHH----------------hhhlLHHHHHHhhhhhhhleeellllleeellll


DSSP  llhHHHHHhhHHHHhhhhhhllllLLLLLllllllhhhhhhhhlllllllllllhhhhhh
Query lldADLXPtrSACIaifearaqhpWPPRVatlphwpliyagalegldhlelartvdaaaq  277
ident      |
Sbjct vagEPLPV--DSEK-------difDYIQW-------------------------------  316
DSSP  lllLLLLL--LLHH-------hhhHHLLL-------------------------------


DSSP  hhhhhhhhhhhllll
Query avqrfvaridratkr  292
ident
Sbjct -----kyrepkdrse  326
DSSP  -----llllhhhlll
```

No 172: Query=mol1A Sbjct=4jwnA Z-score=6.8

back to top

```
DSSP  ------------------------------------------------------------
Query ------------------------------------------------------------    0
ident
Sbjct tlnggitdmltelanfeknvsqaihkynayrkaasviakyphkiksgaeakklpgvgtki   60
DSSP  lllhhhhhhhhhhhhhhhhllllhhhhhhhhhhhhhhhlllllllhhhhhlllllllhhh


DSSP  ------------------------------------------------llllllllllhh
Query ------------------------------------------------aapysspptnlr   12
ident
Sbjct aekideflatgklrklekirqddtsssinfltrvsgigpsaarkfvdegiktledlrkne  120
DSSP  hhhhhhhhhhlllhhhhhhhhlhhhhhhhhhlllllllhhhhhhhhhhllllhhhhhllh


DSSP  hhhhhhhhhhhllllhhhhhHHHHHHHHHHHHHH-LLLLllLLLLEEEEHHHHHHHhhll
Query slrdrltqvaerqgvvfgrlqRHVAXIVVAQFAA-TLTDdtGAPLLLVKGGSSLELrrgi   71
ident                                                |  |
Sbjct dklnhhqriglkyfgdfekriPREEMLQMQDIVLnEVKKvdSEYIATVCGSFRRGA----  176
DSSP  hhllhhhhhhhhlhhhhllleEHHHHHHHHHHHHHhHHHHhlLLLEEEEELHHHHLLL----


DSSP  llllLLLLEEEEEEL------------lLHHHHHHHHHHHHHhhlllleeEEELLLEELlll
Query pdsrTSKDFATVAR------------rDIELIHEQLADAGetgwegftAIFTAPEEIaap  119
ident      | |                         |||             |
Sbjct ---eSSGDMDVLLThpsftsestkqpkLLHQVVEQLQKVH--------FITDTLSKG---  222
DSSP  ---lEELLEEEEEElllleelleellllHHHHHHHHHHHHLL--------LEEEEEEEL---


DSSP  lllLLLEEEEEEEEEEL--LEEEEEEEEEEEEL-LLLLlllleeeeelhhhhllllllleee
Query gxpVKPRRFTAKLSYR--GRAFATVPIEVSSV-EAGNadqfdtltsdalglvgvpaavav  176
ident             |             |
Sbjct ---ETKFMGVCQLPSKndEKEYPHRRIAIRLIpKDQY-----------------------  256
DSSP  ---LLEEEEEEELLLLLllLLLLLLEEEEEEEELlHHHH----------------------


DSSP  eellhHHHHhhhhhhhhlllllllllllHHHHHHH-------------------hhhhh
Query pcxtiPWQIaqklhavtavleepkandraHDLVDLQ-------------------llegl  217
ident                               |
Sbjct ----yCGVL----------------yftgSDIFNKNmrahalekgftineytirplgvtg  296
DSSP  ----hHHHH----------------hhhlLHHHHHHhhhhhhhhleeellllleeellll


DSSP  llhHHHHHhhHHHHHhhhhhhllllLLLLLllllllhhhhhhhhlllllllllllhhhhhh
Query lldADLXptRSACIaifearaqhpWPPRVatlphwpliyagalegldhlelartvdaaaq  277
ident      |
Sbjct vagEPLP--VDSEK-------difDYIQW-------------------------------  316
DSSP  lllLLLL--LLLHH-------hhhHHLLL-------------------------------
```

```
DSSP  [illegible]
Query avqrfvaridratkr  292
ident
Sbjct -----kyrepkdrse  326
DSSP  -----llllhhhhll
```

No 173: Query=mol1A Sbjct=3rjhA Z-score=6.8

back to top

```
DSSP  ------------------------------------------------------------
Query ------------------------------------------------------------    0
ident
Sbjct tlnggitdmltelanfeknvsqaihkynayrkaasviakyphkiksgaeakklpgvgtki   60
DSSP  lllhhhhhhhhhhhhhhhhhllllhhhhhhhhhhhhhhhhlllllllhhhhhlllllhhh


DSSP  ------------------------------------------------llllllllllhh
Query ------------------------------------------------aapysspptnlr   12
ident
Sbjct aekideflatgklrklekirqddtsssinfltrvsgigpsaarkfvdegiktledlrkne  120
DSSP  hhhhhhhhhlllhhhhhhhhlhhhhhhhhhllllllhhhhhhhhllllllhhhhhllh


DSSP  hhhhhhhhhhhhllllhhhhhHHHHHHHHHHHHH-LLLLllLLLLEEEEHHHHHHHHhhll
Query slrdrltqvaerqgvvfgrlqRHVAXIVVAQFAA-TLTDdtGAPLLLVKGGSSLELrrgi   71
ident                                                | |
Sbjct dklnhhqriglkyfgdfekriPREEMLQMQDIVLnEVKKvdSEYIATVCGSFRRGA----  176
DSSP  hhllhhhhhhhhlhhhhhlleEHHHHHHHHHHHHHhHHHHhlLLLEEEELHHHHLLL----


DSSP  llllLLLLEEEEEL------------lLHHHHHHHHHHHHhhlllleeEEELLLEELlll
Query pdsrTSKDFATVAR------------rDIELIHEQLADAGetgwegftAIFTAPEEIaap  119
ident      | |                         |||             |
Sbjct ---eSSGDMDVLLThpsftsestkqpkLLHQVVEQLQKVH--------FITDTLSKG---  222
DSSP  ---lEELLEEEEEEllleelleellllHHHHHHHHHHHLL--------LEEEEEEEL---


DSSP  lllLLLEEEEEEEEEL--LEEEEEEEEEEEEL-LLLLlllleeeeelhhhhllllleee
Query gxpVKPRRFTAKLSYR--GRAFATVPIEVSSV-EAGNadqfdtltsdalglvgvpaavav  176
ident             |             |
Sbjct ---ETKFMGVCQLPSKndEKEYPHRRIDIRLIpKDQY-----------------------  256
DSSP  ---LLEEEEEEELLLLLllLLLLLLEEEEEEELlHHHH-----------------------


DSSP  eellhHHHhhhhhhhhllllllllllllHHHHHHH--------------------hhhhh
Query pcxtiPWQIaqklhavtavleepkandraHDLVDLQ--------------------llegl  217
ident                               |
Sbjct ----yCGVL----------------yftgSDIFNKNmrahalekgftineytirplgvtg  296
DSSP  ----hHHHH----------------hhhlLHHHHHHhhhhhhhlleeelllleeelllll


DSSP  llhHHHHhhHHHHHhhhhhhllllLLLLLllllhhhhhhhhhllllllllllhhhhhh
Query lldADLXptRSACIaifearaqhpWPPRVatlphwpliyagalegldhlelartvdaaaq  277
ident      |
Sbjct vagEPLP--VDSEK-------difDYIQW-------------------------------  316
DSSP  lllLLLL--LLLHH-------hhhHHLLL-------------------------------


DSSP  hhhhhhhhhhhllll
Query avqrfvaridratkr  292
ident
Sbjct -----kyrepkdrse  326
DSSP  -----llllhhhlll
```

No 174: Query=mol1A Sbjct=3uxpB Z-score=6.8

back to top

```
DSSP  ------------------------------------------------------------
Query ------------------------------------------------------------    0
ident
Sbjct etlnggitdmlvelanfeknvsqaihkynayrkaasviakyphkiksgaeakklpgvgtk   60
DSSP  llllhhhhhhhhhhhhhhhhhllllhhhhhhhhhhhhhhhhlllllllhhhhhllllllhh
```

```
DSSP  -------------------------------------------------lllllllllh
Query -------------------------------------------------aapysspptnl   11
ident
Sbjct iaekideflatgklrklekirqddtsssinfltrvtgigpsaarklvdegiktledlrkn  120
DSSP  hhhhhhhhhhhlllhhhhhhlllhhhhhhhhhlllllllhhhhhhhlllllhhhhhhl


DSSP  hhhhhhhhhhhhhllllhhhhhHHHHHHHHHHHHH-LLLLllLLLLEEEEHHHHHHHhhl
Query rslrdrltqvaerqgvvfgrlqRHVAXIVVAQFAA-TLTDdtGAPLLLVKGGSSLELrrg   70
ident                                                 | |
Sbjct edklnhhqriglkyfedfekriPREEMLQMQDIVLnEVKKldPEYIATVCGSFRRGA---  177
DSSP  hhhllhhhhhhhhlhhhhhlleEHHHHHHHHHHHHhHHHHhlLLLEEEELLLLLLL---


DSSP  lllllLLLLEEEEEL------------lLHHHHHHHHHHHHHhhllllleeEEELLLEELll
Query ipdsrTSKDFATVAR------------rDIELIHEQLADAGetgwegftAIFTAPEEIaa  118
ident       | |                         |||             |
Sbjct ----eSSGDMDVLLThpnftsesskqpkLLHRVVEQLQKVR--------FITDTLSKG--  223
DSSP  ----lEELLLEEEEElllllllllllhHHHHHHHHHHHHLL--------LEEEEEEEL--


DSSP  llllLLLEEEEEEEEEL--LEEEEEEEEEEEL-LLLLllllleeeeelhhhhllllllee
Query pgxpVKPRRFTAKLSYR--GRAFATVPIEVSSV-EAGNadqfdtltsdalglvgvpaava  175
ident              |             |
Sbjct ----ETKFMGVCQLPSEndENEYPHRRIDIRLQpKDQY----------------------  257
DSSP  ----LLEEEEEELLLLllLLLLLLLEEEEEEELlLLLH----------------------


DSSP  eeellhHHHHhhhhhhhhllllllllllHHHHHHH-------------------hhhh
Query vpcxtiPWQIaqklhavtavleepkandraHDLVDLQ-------------------lleg  216
ident                                |
Sbjct -----yCGVL----------------yftgSDIFNKNmrahalekgftineytirplgvt  296
DSSP  -----hHHHH----------------hhhlLHHHHHHhhhhlllllleeellllleeellll


DSSP  hllhHHHHhhHHHHHhhhhhhllllLLLLLlllllhhhhhhhhhllllllllllhhhhh
Query llldADLXptRSACIaifearaqhpWPPRVatlphwpliyagalegldhlelartvdaaa  276
ident       |
Sbjct gvagEPLP--VDSEQ-------difDYIQW------------------------------  317
DSSP  llllLLLL--LLLHH-------hhhHHLLL------------------------------


DSSP  hhhhhhhhhhhhllll
Query qavqrfvaridratkr  292
ident
Sbjct -------ryrepkdrs  326
DSSP  -------llllhhhll
```

No 175: Query=mol1A Sbjct=3jpqA Z-score=6.8

back to top

```
DSSP  ------------------------------------------------------------
Query ------------------------------------------------------------    0
ident
Sbjct tlnggitdmltelanfeknvsqaihkynayrkaasviakyphkiksgaeakklpgvgtki   60
DSSP  lllhhhhhhhhhhhhhhhhllllhhhhhhhhhhhhhhhhlllllllhhhhhlllllhhh


DSSP  ------------------------------------------------lllllllllhh
Query ------------------------------------------------aapysspptnlr   12
ident
Sbjct aekideflatgklrklekirqddtsssinfltrvsgigpsaarkfvdegiktledlrkne  120
DSSP  hhhhhhhhhhlllhhhhhhhhlhhhhhhhhhlllllllhhhhhhhhhlllllhhhhhhlh


DSSP  hhhhhhhhhhhhllllhhhhhHHHHHHHHHHHHH-LLLLllLLLLEEEEHHHHHHHhhll
Query slrdrltqvaerqgvvfgrlqRHVAXIVVAQFAA-TLTDdtGAPLLLVKGGSSLELrrgi   71
ident                                                | |
Sbjct dklnhhqriglkyfgdfekriPREEMLQMQDIVLnEVKKvdSEYIATVCGSFRRGA----  176
DSSP  hhllhhhhhhhhlllhhhlleEHHHHHHHHHHHHhHHHHhlLLLEEEELHHHHLLL----


DSSP  llllLLLLEEEEEL------------lLHHHHHHHHHHHHHhhllllleeEEELLLEELlll
Query pdsrTSKDFATVAR------------rDIELIHEQLADAGetgwegftAIFTAPEEIaap  119
```

```
ident      | |                         |||             |
Sbjct ---eSSGDMDVLLThpsftsestkqpkLLHQVVEQLQKVH--------FITDTLSKG---  222
DSSP  ---lEELLEEEEEElllllllllllllHHHHHHHHHHHLL--------LEEEEEEEL---


DSSP  lllLLLEEEEEEEEEL--LEEEEEEEEEEEEL-LLLLlllleeeeelhhhhlllllleee
Query gxpVKPRRFTAKLSYR--GRAFATVPIEVSSV-EAGNadqfdtltsdalglvgvpaavav  176
ident             |             |
Sbjct ---ETKFMGVCQLPSKndEKEYPHRRIDIRLIpKDQY-----------------------  256
DSSP  ---LLEEEEEELLLLLllLLLLLLEEEEEEELlHHHH-----------------------


DSSP  eellhHHHHhhhhhhhhllllllllllllHHHHHHH-------------------hhhhh
Query pcxtiPWQIaqklhavtavleepkandraHDLVDLQ-------------------llegl  217
ident                               |
Sbjct ----yCGVL----------------yftgSDIFNKNmrahalekgftineytirplgvtg  296
DSSP  ----hHHHH----------------hhhlLHHHHHHhhhhhhhlleeellllleeellll


DSSP  llhHHHHhhHHHHHhhhhhhllllLLLLLlllllhhhhhhhhhlllllllllllhhhhhh
Query lldADLXptRSACIaifearaqhpWPPRVatlphwpliyagalegldhlelartvdaaaq  277
ident      |
Sbjct vagEPLP--VDSEK-------difDYIQW-------------------------------  316
DSSP  lllLLLL--LLLHH-------hhhHHLLL-------------------------------


DSSP  hhhhhhhhhhhllll
Query avqrfvaridratkr  292
ident
Sbjct -----kyrepkdrse  326
DSSP  -----llllhhhlll
```

No 176: Query=mol1A Sbjct=3rjjA Z-score=6.8

back to top

```
DSSP  ------------------------------------------------------------
Query ------------------------------------------------------------    0
ident
Sbjct tlnggitdmltelanfeknvsqaihkynayrkaasviakyphkiksgaeakklpgvgtki   60
DSSP  lllhhhhhhhhhhhhhhhhllllhhhhhhhhhhhhhhhllllllllhhhhhhlllllhhh


DSSP  ------------------------------------------------llllllllllhh
Query ------------------------------------------------aapysspptnlr   12
ident
Sbjct aekideflatgklrklekirqddtsssinfltrvsgigpsaarkfvdegiktledlrkne  120
DSSP  hhhhhhhhhhlllhhhhhhhhhlhhhhhhhhhllllllhhhhhhhhhlllllhhhhhhlh


DSSP  hhhhhhhhhhhhllllhhhhhHHHHHHHHHHHHHH-LLLLllLLLLEEEEHHHHHHHHhhll
Query slrdrltqvaerqgvvfgrlqRHVAXIVVAQFAA-TLTDdtGAPLLLVKGGSSLELrrgi   71
ident                                                | |
Sbjct dklnhhqriglkyfgdfekriPREEMLQMQDIVLnEVKKvdSEYIATVCGSFRRGA----  176
DSSP  hhllhhhhhhhhlhhhhllleEHHHHHHHHHHHHhHHHHhlLLLEEEELHHHHHLL----


DSSP  llllLLLLEEEEEL------------lLHHHHHHHHHHHHhhlllleeEEELLLEELlll
Query pdsrTSKDFATVAR------------rDIELIHEQLADAGetgwegftAIFTAPEEIaap  119
ident      | |                         |||             |
Sbjct ---eSSGDMDVLLThpsftsestkqpkLLHQVVEQLQKVH--------FITDTLSKG---  222
DSSP  ---lEELLEEEEEEllllllllllhhhHHHHHHHHHHHLL--------LEEEEEEEL---


DSSP  lllLLLEEEEEEEEEL--LEEEEEEEEEEEEL-LLLLlllleeeeelhhhhlllllleee
Query gxpVKPRRFTAKLSYR--GRAFATVPIEVSSV-EAGNadqfdtltsdalglvgvpaavav  176
ident             |             |
Sbjct ---ETKFMGVCQLPSKndEKEYPHRRIDIRLIpKDQY-----------------------  256
DSSP  ---LLEEEEEELLLLLllLLLLLLEEEEEEELlHHHH-----------------------


DSSP  eellhHHHHhhhhhhhhllllllllllllhhhhHHHHHH---------------------
Query pcxtiPWQIaqklhavtavleepkandrahdlvDLQLLE---------------------  215
ident
Sbjct ----yCGVL--------------------yftgSDIFNKnmrahalekgftineytirpl  292
DSSP  ----hHHHH--------------------hhhlLHHHHHhhhhhhhhlleeelllleeel
```

```
DSSP  -hhllhhhhhhhhHHHHhhhhhhllllLLLLLlllllhhhhhhhhhlllllllllllhhh
Query -glllldadlxptrSACIaifearaqhpWPPRVatlphwpliyagalegldhlelartvda  274
ident              |
Sbjct gvtgvageplpvdSEKD-------ifdYIQWK----------------------------  317
DSSP  lllllllllllllLHHH-------hhhHLLLL----------------------------


DSSP  hhhhhhhhhhhhhhhllll
Query aaqavqrfvaridratkr  292
ident
Sbjct ---------yrepkdrse  326
DSSP  ---------lllhhhlll
```

No 177: Query=mol1A Sbjct=9icpA Z-score=6.8

[back to top](#)

```
DSSP  ------------------------------------------------------------
Query ------------------------------------------------------------    0
ident
Sbjct etlnggitdmltelanfeknvsqaihkynayrkaasviakyphkiksgaeakklpgvgtk   60
DSSP  llllhhhhhhhhhhhhhhhhllllhhhhhhhhhhhhhhhhlllllllhhhhhllllllhh


DSSP  -------------------------------------------------lllllllllh
Query -------------------------------------------------aapysspptnl   11
ident
Sbjct iaekideflatgklrklekirqddtsssinfltrvsgigpsaarkfvdegiktledlrkn  120
DSSP  hhhhhhhhhhllllhhhhhhlllhhhhhhhhhhlllllllhhhhhhhhhlllllhhhhhhl


DSSP  hhhhhhhhhhhhhllllhhhhhHHHHHHHHHHHHH-LLLLllLLLLEEEEHHHHHHHhhl
Query rslrdrltqvaerqggvvfgrlqRHVAXIVVAQFAA-TLTDdtGAPLLLVKGGSSLELrrg   70
ident                                                 | |
Sbjct edklnhhqriglkyfgdfekriPREEMLQMQDIVLnEVKKvdSEYIATVCGSFRRGA---  177
DSSP  lllllhhhhhhhhlhhhhhlleEHHHHHHHHHHHHHhHHHHhlLLLEEEELLHHHLLL---


DSSP  lllllLLLLEEEEEL------------lLHHHHHHHHHHHHHhhlllleeEEELLLEELll
Query ipdsrTSKDFATVAR------------rDIELIHEQLADAGetgwegftAIFTAPEEIaa  118
ident       | |                         |||             |
Sbjct ----eSSGDMDVLLThpsftsestkqpkLLHQVVEQLQKVH--------FITDTLSKG--  223
DSSP  ----lEELLEEEEEEllllllllllllhHHHHHHHHHHHHL--------LEEEEEEEL--


DSSP  llllLLLEEEEEEEEELL--EEEEEEEEEEEEL-LLLLllllleeeeelhhhhlllllllee
Query pgxpVKPRRFTAKLSYRG--RAFATVPIEVSSV-EAGNadqfdtltsdalglvgvpaava  175
ident              |             |
Sbjct ----ETKFMGVCQLPSKNdeKEYPHRRIDIRLIpKDQY----------------------  257
DSSP  ----LLEEEEEEELLLLLllLLLLLLEEEEEEEELlHHHH----------------------


DSSP  eeellhHHHHhhhhhhhhlllllllllllhhhhHHHHHH--------------------
Query vpcxtiPWQIaqklhavtavleepkandrahdlvDLQLLE--------------------  215
ident
Sbjct -----yCGVL--------------------yftgSDIFNKnmrahalekgftineytirp  292
DSSP  -----hHHHH--------------------hhhlLHHHHHhhhhhhhhlleeeelleeee


DSSP  -hhllhhhhhhhhHHHHHhhhhhllllLLLLLllllllhhhhhhhhhlllllllllllhhh
Query -glllldadlxptrSACIAifearaqhPWPPRvatlphwpliyagalegldhlelartvda  274
ident
Sbjct lgvtgvageplpvDSEKD-----ifdYIQWK-----------------------------  318
DSSP  lllllllllllllLLHHH-----hhhHLLLL-----------------------------


DSSP  hhhhhhhhhhhhhhhllll
Query aaqavqrfvaridratkr  292
ident
Sbjct ---------yrepkdrse  327
DSSP  ---------lllhhhhll
```

No 178: Query=mol1A Sbjct=8icsA Z-score=6.8

back to top

```
DSSP  ------------------------------------------------------------
Query ------------------------------------------------------------    0
ident 
Sbjct etlnggitdmltelanfeknvsqaihkynayrkaasviakyphkiksgaeakklpgvgtk   60
DSSP  llllhhhhhhhhhhhhhhhhllllhhhhhhhhhhhhhhhhlllllllhhhhhlllllllhh


DSSP  -------------------------------------------------llllllllllh
Query -------------------------------------------------aapysspptnl   11
ident 
Sbjct iaekideflatgklrklekirqddtsssinfltrvsgigpsaarkfvdegiktledlrkn  120
DSSP  hhhhhhhhhhllllhhhhhhhhlhhhhhhhhhlllllllhhhhhhhhlllllllhhhhhhl


DSSP  hhhhhhhhhhhhhllllhhhhhHHHHHHHHHHHHH-LLLLllLLLLLEEEEHHHHHHHhhl
Query rslrdrltqvaerqgvvfgrlqRHVAXIVVAQFAA-TLTDdtGAPLLLVKGGSSLELrrg   70
ident                                                 | |
Sbjct edklnhhqriglkyfgdfekriPREEMLQMQDIVLnEVKKvdSEYIATVCGSFRRGA---  177
DSSP  hhhllhhhhhhhhlhhhhhlleEHHHHHHHHHHHHHhHHHHhlLLLEEEELLLLLLLL---


DSSP  lllllLLLLEEEEEL------------lLHHHHHHHHHHHHHhhllllleeEEELLLEELll
Query ipdsrTSKDFATVAR------------rDIELIHEQLADAGetgwegftAIFTAPEEIaa  118
ident       | |                         |||             |
Sbjct ----eSSGDMDVLLThpsftsestkqpkLLHQVVEQLQKVH--------FITDTLSKG--  223
DSSP  ----lEELLEEEEEEellllllllllhHHHHHHHHHHHHLL--------LEEEEEEEL--


DSSP  llllLLLEEEEEEEEEELL--EEEEEEEEEEEEL-LLLLlllleeeeelhhhhllllllee
Query pgxpVKPRRFTAKLSYRG--RAFATVPIEVSSV-EAGNadqfdtltsdalglvgvpaava  175
ident              |             |
Sbjct ----ETKFMGVCQLPSKNdeKEYPHRRIDIRLIpKDQY----------------------  257
DSSP  ----LLEEEEEEELLLLLllLLLLLLEEEEEEEEEeLLLH----------------------


DSSP  eeellhHHHHhhhhhhhhllllllllllllhhhhHHHHHH--------------------
Query vpcxtiPWQIaqklhavtavleepkandrahdlvDLQLLE--------------------  215
ident 
Sbjct -----yCGVL--------------------yftgSDIFNKnmrahalekgftineytirp  292
DSSP  -----hHHHH--------------------hhhlLHHHHHhhhhhhhhhllllleelleell


DSSP  -hhllhhhhhhhHHHHHhhhhhhlllLLLLLLlllllhhhhhhhhhllllllllllllhhh
Query -glllddadlxptRSACIaifearaqhPWPPRVatlphwpliyagalegldhlelartvda  274
ident 
Sbjct lgvtgvageplpVDSEK------difDYIQWK----------------------------  318
DSSP  llllllllllllLLLHH------hhhHHLLLL----------------------------


DSSP  hhhhhhhhhhhhhhhllll
Query aaqavqrfvaridratkr  292
ident 
Sbjct ---------yrepkdrse  327
DSSP  ---------lllllllll
```

No 179: Query=mol1A Sbjct=9icoA Z-score=6.8

back to top

```
DSSP  ------------------------------------------------------------
Query ------------------------------------------------------------    0
ident 
Sbjct etlnggitdmltelanfeknvsqaihkynayrkaasviakyphkiksgaeakklpgvgtk   60
DSSP  llllhhhhhhhhhhhhhhhhllllhhhhhhhhhhhhhhhhlllllllhhhhhlllllllhh


DSSP  -------------------------------------------------llllllllllh
Query -------------------------------------------------aapysspptnl   11
ident 
Sbjct iaekideflatgklrklekirqddtsssinfltrvsgigpsaarkfvdegiktledlrkn  120
DSSP  hhhhhhhhhhllllhhhhhhhlllhhhhhhhhhlllllllhhhhhhhhlllllllhhhhhhl


DSSP  hhhhhhhhhhhhhllllhhhhhHHHHHHHHHHHHH-LLLLllLLLLLEEEEHHHHHHHhhl
Query rslrdrltqvaerqgvvfgrlqRHVAXIVVAQFAA-TLTDdtGAPLLLVKGGSSLELrrg   70
```

```
ident                                                   | |
Sbjct edklnhhqriglkyfgdfekriPREEMLQMQDIVLnEVKKvdSEYIATVCGSFRRGA---  177
DSSP  lllllhhhhhhhhlhhhhhlleEHHHHHHHHHHHHhHHHHhlLLLEEEELLLLLLLL---


DSSP  lllllLLLLEEEEEL------------lLHHHHHHHHHHHHhhlllleeEEELLLEELll
Query ipdsrTSKDFATVAR------------rDIELIHEQLADAGetgwegftAIFTAPEEIaa  118
ident       | |                         |||             |
Sbjct ----eSSGDMDVLLThpsftsestkqpkLLHQVVEQLQKVH--------FITDTLSKG--  223
DSSP  ----lEELLEEEEEEelllllllllllhHHHHHHHHHHHHLL--------LEEEEEEEL--


DSSP  llllLLLEEEEEEEEEELL--EEEEEEEEEEEEL-LLLLllllleeeeelhhhhllllllee
Query pgxpVKPRRFTAKLSYRG--RAFATVPIEVSSV-EAGNadqfdtltsdalglvgvpaava  175
ident              |             |
Sbjct ----ETKFMGVCQLPSKNdeKEYPHRRIDIRLIpKDQY----------------------  257
DSSP  ----LLEEEEEEELLLLLLlLLLLLLEEEEEEEEEeHHHH----------------------


DSSP  eeellhHHHHhhhhhhhhllllllllllllhhhhHHHHHH--------------------
Query vpcxtiPWQIaqklhavtavleepkandrahdlvDLQLLE--------------------  215
ident
Sbjct -----yCGVL--------------------yftgSDIFNKnmrahalekgftineytirp  292
DSSP  -----hHHHH--------------------hhhlLHHHHHhhhhhhhhlleeeelleeee


DSSP  -hhllhhhhhhhHHHHHhhhhhhlllLLLLLLlllllhhhhhhhhhlllllllllllhhh
Query -glllldadlxptRSACIaifearaqhPWPPRVatlphwpliyagalegldhlelartvda  274
ident
Sbjct lgvtgvageplpVDSEK------difDYIQWK----------------------------  318
DSSP  llllllllllllLLLHH------hhhHHLLLL----------------------------


DSSP  hhhhhhhhhhhhhhllll
Query aaqavqrfvaridratkr  292
ident
Sbjct ---------yrepkdrse  327
DSSP  ---------llllhhhll
```

No 180: Query=mol1A Sbjct=1zqaA Z-score=6.8

back to top

```
DSSP  ------------------------------------------------------------
Query ------------------------------------------------------------    0
ident
Sbjct etlnggitdmltelanfeknvsqaihkynayrkaasviakyphkiksgaeakklpgvgtk   60
DSSP  llllhhhhhhhhhhhhhhhhhllllhhhhhhhhhhhhhhhhhllllllllhhhhhllllllhh


DSSP  -------------------------------------------------llllllllllh
Query -------------------------------------------------aapyssspptnl   11
ident
Sbjct iaekideflatgklrklekirqddtsssinfltrvsgigpsaarkfvdegiktledlrkn  120
DSSP  hhhhhhhhhhllllhhhhhhhhlhhhhhhhhhllllllhhhhhhhhhlllllhhhhhhl


DSSP  hhhhhhhhhhhhhllllhhhhhHHHHHHHHHHHHHH-LLLLllLLLLEEEEHHHHHHHHhhl
Query rslrdrltqvaerqgvvfgrlqRHVAXIVVAQFAA-TLTDdtGAPLLLVKGGSSLELrrg   70
ident                                                 | |
Sbjct edklnhhqriglkyfgdfekriPREEMLQMQDIVLnEVKKvdSEYIATVCGSFRRGA---  177
DSSP  hhhllhhhhhhhhlhhhhhlleEHHHHHHHHHHHHhHHHHhlLLLEEEELHHHLLLL---


DSSP  lllllLLLLEEEEEL------------lLHHHHHHHHHHHHhhlllleeEEELLLEELll
Query ipdsrTSKDFATVAR------------rDIELIHEQLADAGetgwegftAIFTAPEEIaa  118
ident       | |                         |||             |
Sbjct ----eSSGDMDVLLThpsftsestkqpkLLHQVVEQLQKVH--------FITDTLSKG--  223
DSSP  ----lEELLEEEEEEEllllllllllllhhHHHHHHHHHHHHLL--------LEEEEEEEL--


DSSP  llllLLLEEEEEEEEEELL--EEEEEEEEEEEEL-LLLLllllleeeeelhhhhllllllee
Query pgxpVKPRRFTAKLSYRG--RAFATVPIEVSSV-EAGNadqfdtltsdalglvgvpaava  175
ident              |             |
Sbjct ----ETKFMGVCQLPSKNdeKEYPHRRIDIRLIpKDQY----------------------  257
DSSP  ----LLEEEEEEELLLLLLlLLLLLLEEEEEEELLlHHHH----------------------
```

```
DSSP  eeellhHHHHhhhhhhhhllllllllllllhhhhHHHHHH--------------------
Query vpcxtiPWQIaqklhavtavleepkandrahdlvDLQLLE--------------------  215
ident
Sbjct -----yCGVL--------------------yftgSDIFNKnmrahalekgftineytirp  292
DSSP  -----hHHHH--------------------hhllLHHHHHhhhhhhhhlleeeelleeee


DSSP  -hhllhhhhhhhHHHHHhhhhhhlllLLLLLLlllllhhhhhhhhhllllllllllllhhh
Query -gllldadlxptRSACIaifearaqhPWPPRVatlphwpliyagalegldhlelartvda  274
ident
Sbjct lgvtgvageplpVDSEK------difDYIQWK----------------------------  318
DSSP  llllllllllllLLLHH------hhhHHLLLL----------------------------


DSSP  hhhhhhhhhhhhhhllll
Query aaqavqrfvaridratkr  292
ident
Sbjct ---------yrepkdrse  327
DSSP  ---------lllhhhlll
```

No 181: Query=mol1A Sbjct=1zqnA Z-score=6.8

[back to top]

```
DSSP  ------------------------------------------------------------
Query ------------------------------------------------------------    0
ident
Sbjct etlnggitdmltelanfeknvsqaihkynayrkaasviakyphkiksgaeakklpgvgtk   60
DSSP  llllhhhhhhhhhhhhhhhllllllhhhhhhhhhhhhhhlllllllllhhhhhllllllhh


DSSP  -------------------------------------------------llllllllllh
Query -------------------------------------------------aapysspptnl   11
ident
Sbjct iaekideflatgklrklekirqddtsssinfltrvsgigpsaarkfvdegiktledlrkn  120
DSSP  hhhhhhhhhhllllhhhhhhhhlhhhhhhhhhhllllllhhhhhhhhhlllllhhhhhhl


DSSP  hhhhhhhhhhhhhllllhhhhhHHHHHHHHHHHHH-LLLLllLLLLLEEEEHHHHHHHHhhl
Query rslrdrltqvaerqgvvfgrlqRHVAXIVVAQFAA-TLTDdtGAPLLLVKGGSSLELrrg   70
ident                                                 | |
Sbjct edklnhhqriglkyfgdfekriPREEMLQMQDIVLnEVKKvdSEYIATVCGSFRRGA---  177
DSSP  hhhllhhhhhhhhlhhhhhlleEHHHHHHHHHHHHHhHHHHhlLLLEEEELLLLLLL---


DSSP  lllllLLLLEEEEEL------------lLHHHHHHHHHHHHHhhllllleeEEEELLLEELll
Query ipdsrTSKDFATVAR------------rDIELIHEQLADAGetgwegftAIFTAPEEIaa  118
ident       | |                         |||             |
Sbjct ----eSSGDMDVLLThpsftsestkqpkLLHQVVEQLQKVH--------FITDTLSKG--  223
DSSP  ----lEELLEEEEEEellllllllllhhHHHHHHHHHHHHLL--------LEEEEEEEL--


DSSP  llllLLLEEEEEEEEEELL--EEEEEEEEEEEEL-LLLLllllleeeeelhhhhllllllee
Query pgxpVKPRRFTAKLSYRG--RAFATVPIEVSSV-EAGNadqfdtltsdalglvgvpaava  175
ident              |             |
Sbjct ----ETKFMGVCQLPSKNdeKEYPHRRIDIRLIpKDQY----------------------  257
DSSP  ----LLEEEEEEELLLLLLllLLLLLLEEEEEEEEEeLLLH----------------------


DSSP  eeellhHHHHHHhhhhhhllllllllllllhhhhHHHHHH--------------------
Query vpcxtiPWQIAQklhavtavleepkandrahdlvDLQLLE--------------------  215
ident
Sbjct -----yCGVLYF--------------------tgSDIFNKnmrahalekgftineytirp  292
DSSP  -----hHHHHHH--------------------llLHHHHHhhhhhhhhlleeeelleeee


DSSP  --hhllhhhhhhhhHHHhhhhhhllllLLLLLlllllhhhhhhhhhllllllllllllhh
Query --gllldadlxptrSACIaifearaqhpWPPRVatlphwpliyagalegldhlelartvd  273
ident               |
Sbjct lgvtgvageplpvdSEKD-------ifdYIQWK---------------------------  318
DSSP  llllllllllllllLHHH-------hhhHLLLL---------------------------


DSSP  hhhhhhhhhhhhhhhllll
Query aaaqavqrfvaridratkr  292
ident
```

```
[illegible]
DSSP  ----------lllhhhlll
```

No 182: Query=mol1A Sbjct=9icyA Z-score=6.8

back to top

```
DSSP  ------------------------------------------------------------
Query ------------------------------------------------------------    0
ident 
Sbjct etlnggitdmltelanfeknvsqaihkynayrkaasviakyphkiksgaeakklpgvgtk   60
DSSP  llllhhhhhhhhhhhhhhhhllllhhhhhhhhhhhhhhhhlllllllhhhhhlllllllhh


DSSP  -------------------------------------------------lllllllllh
Query -------------------------------------------------aapysspptnl   11
ident 
Sbjct iaekideflatgklrklekirqddtsssinfltrvsgigpsaarkfvdegiktledlrkn  120
DSSP  hhhhhhhhhhllllhhhhhhhhlhhhhhhhhhlllllllhhhhhhhhhhllllhhhhhhl


DSSP  hhhhhhhhhhhhllllhhhhhHHHHHHHHHHHHHH-LLLLllLLLLEEEEHHHHHHHHhhl
Query rslrdrltqvaerqggvvfgrlqRHVAXIVVAQFAA-TLTDdtGAPLLLVKGGSSLELrrg   70
ident                                                  | |
Sbjct edklnhhqriglkyfgdfekriPREEMLQMQDIVLnEVKKvdSEYIATVCGSFRRGA---  177
DSSP  hhhllhhhhhhhhlhhhhhlleEHHHHHHHHHHHHHhHHHHhlLLLEEEEEELLLLLL---


DSSP  lllllLLLLEEEEEL------------lLHHHHHHHHHHHHhhlllleeEEELLLEELll
Query ipdsrTSKDFATVAR------------rDIELIHEQLADAGetgwegftAIFTAPEEIaa  118
ident       | |                         |||             |
Sbjct ----eSSGDMDVLLThpsftsestkqpkLLHQVVEQLQKVH--------FITDTLSKG--  223
DSSP  ----lEELLEEEEEEelllllllllhHHHHHHHHHHHLL--------LEEEEEEEL--


DSSP  llllLLLEEEEEEEEELL--EEEEEEEEEEEEL-LLLLllllleeeeelhhhhllllllee
Query pgxpVKPRRFTAKLSYRG--RAFATVPIEVSSV-EAGNadqfdtltsdalglvgvpaava  175
ident              |             |
Sbjct ----ETKFMGVCQLPSKNdeKEYPHRRIDIRLIpKDQY----------------------  257
DSSP  ----LLEEEEEEELLLLLLllLLLLLLEEEEEEEEEeHHHH----------------------


DSSP  eeellhHHHHhhhhhhhhllllllllllhhhhHHHHHH--------------------
Query vpcxtiPWQIaqklhavtavleepkandrahdlvDLQLLE--------------------  215
ident 
Sbjct -----yCGVL--------------------yftgSDIFNKnmrahalekgftineytirp  292
DSSP  -----hHHHH--------------------hhllLHHHHHhhhhhhhhlleeeelleeee


DSSP  -hhllhhhhhhhHHHHHHhhhhhllllLLLLLlllllhhhhhhhhhllllllllllhhh
Query -glllldadlxptRSACIAifearaqhpWPPRVatlphwpliyagaleglldhlelartvda  274
ident 
Sbjct lgvtgvageplpVDSEKD------ifdYIQWK----------------------------  318
DSSP  llllllllllllLLLHHH------hhhHLLLL----------------------------


DSSP  hhhhhhhhhhhhhhllll
Query aaqavqrfvaridratkr  292
ident 
Sbjct ---------yrepkdrse  327
DSSP  ---------lllhhhlll
```

No 183: Query=mol1A Sbjct=9icxA Z-score=6.8

back to top

```
DSSP  ------------------------------------------------------------
Query ------------------------------------------------------------    0
ident 
Sbjct etlnggitdmltelanfeknvsqaihkynayrkaasviakyphkiksgaeakklpgvgtk   60
DSSP  llllhhhhhhhhhhhhhhhhllllhhhhhhhhhhhhhhhhlllllllhhhhhlllllllhh


DSSP  -------------------------------------------------lllllllllh
```

```
[illegible]
ident
Sbjct iaekideflatgklrklekirqddtsssinfltrvsgigpsaarkfvdegiktledlrkn  120
DSSP  hhhhhhhhhhllllhhhhhhhhlhhhhhhhhhllllllhhhhhhhhllllllhhhhhhl


DSSP  hhhhhhhhhhhhhllllhhhhhHHHHHHHHHHHHHH-LLLLllLLLLEEEEHHHHHHHHhhl
Query rslrdrltqvaerqgvvfgrlqRHVAXIVVAQFAA-TLTDdtGAPLLLVKGGSSLELrrg   70
ident                                                 | |
Sbjct edklnhhqriglkyfgdfekriPREEMLQMQDIVLnEVKKvdSEYIATVCGSFRRGA---  177
DSSP  hhhllhhhhhhhhlllhhhlleEHHHHHHHHHHHHHhHHHHhlLLLEEEELLLLLLL---


DSSP  lllllLLLLEEEEEL------------lLHHHHHHHHHHHHhhlllleeEEELLLEELll
Query ipdsrTSKDFATVAR------------rDIELIHEQLADAGetgwegftAIFTAPEEIaa  118
ident       | |                         |||             |
Sbjct ----eSSGDMDVLLThpsftsestkqpkLLHQVVEQLQKVH--------FITDTLSKG--  223
DSSP  ----lEELLEEEEEEelllllllllhHHHHHHHHHHHHLL--------LEEEEEEEL--


DSSP  llllLLLEEEEEEEEEELL--EEEEEEEEEEEEL-LLLLllllleeeeelhhhhllllllee
Query pgxpVKPRRFTAKLSYRG--RAFATVPIEVSSV-EAGNadqfdtltsdalglvgvpaava  175
ident              |             |
Sbjct ----ETKFMGVCQLPSKNdeKEYPHRRIDIRLIpKDQY----------------------  257
DSSP  ----LLEEEEEELLLLLLlLLLLLLEEEEEEEEeHHHH----------------------


DSSP  eeellhHHHHhhhhhhhhlllllllllllhhhhHHHHHH--------------------
Query vpcxtiPWQIaqklhavtavleepkandrahdlvDLQLLE--------------------  215
ident
Sbjct -----yCGVL--------------------yftgSDIFNKnmrahalekgftineytirp  292
DSSP  -----hHHHH--------------------hhllLHHHHHhhhhhhhhlleeellllleee


DSSP  -hhllhhhhhhhHHHHHHhhhhhllllLLLLLlllllhhhhhhhhhllllllllllllhhh
Query -gllldadlxptRSACIAifearaqhpWPPRVatlphwpliyagalegldhlelartvda  274
ident
Sbjct lgvtgvageplpVDSEKD------ifdYIQWK----------------------------  318
DSSP  llllllllllllLLLHHH------hhhHLLLL----------------------------


DSSP  hhhhhhhhhhhhhhllll
Query aaqavqrfvaridratkr  292
ident
Sbjct ---------yrepkdrse  327
DSSP  ---------lllhhhhll
```

No 184: Query=mol1A Sbjct=1zqoA Z-score=6.8

back to top

```
DSSP  ------------------------------------------------------------
Query ------------------------------------------------------------    0
ident
Sbjct etlnggitdmltelanfeknvsqaihkynayrkaasviakyphkiksgaeakklpgvgtk   60
DSSP  llllhhhhhhhhhhhhhhhllllllhhhhhhhhhhhhhlllllllllllhhhhhllllllhh


DSSP  -------------------------------------------------llllllllllh
Query -------------------------------------------------aapyssspptnl   11
ident
Sbjct iaekideflatgklrklekirqddtsssinfltrvsgigpsaarkfvdegiktledlrkn  120
DSSP  hhhhhhhhhhlllllhhhhhhhhlhhhhhhhhhllllllhhhhhhhhhhllllhhhhhhl


DSSP  hhhhhhhhhhhhhllllhhhhhHHHHHHHHHHHHHHH-LLLLllLLLLEEEEHHHHHHHHhhl
Query rslrdrltqvaerqgvvfgrlqRHVAXIVVAQFAA-TLTDdtGAPLLLVKGGSSLELrrg   70
ident                                                 | |
Sbjct edklnhhqriglkyfgdfekriPREEMLQMQDIVLnEVKKvdSEYIATVCGSFRRGA---  177
DSSP  hhhllhhhhhhhhlhhhhhlleEHHHHHHHHHHHHHhHHHHhlLLLEEEEELLLLLLL---


DSSP  lllllLLLLEEEEEL------------lLHHHHHHHHHHHHhhlllleeEEELLLEELll
Query ipdsrTSKDFATVAR------------rDIELIHEQLADAGetgwegftAIFTAPEEIaa  118
ident       | |                         |||             |
Sbjct ----eSSGDMDVLLThpsftsestkqpkLLHQVVEQLQKVH--------FITDTLSKG--  223
DSSP  ----lEELLEEEEEEellllllllllhhHHHHHHHHHHHHHL--------LEEEEEEEL--
```

```
DSSP  llllLLLEEEEEEEEEELL--EEEEEEEEEEEEL-LLLLlllleeeeelhhhhllllllee
Query pgxpVKPRRFTAKLSYRG--RAFATVPIEVSSV-EAGNadqfdtltsdalglvgvpaava  175
ident              |             |
Sbjct ----ETKFMGVCQLPSKNdeKEYPHRRIDIRLIpKDQY----------------------  257
DSSP  ----LLEEEEEELLLLLLllLLLLLEEEEEEEEeLLLH----------------------


DSSP  eeellhHHHHhhhhhhhhllllllllllhhhhHHHHHH--------------------
Query vpcxtiPWQIaqklhavtavleepkandrahdlvDLQLLE--------------------  215
ident
Sbjct -----yCGVL--------------------yftgSDIFNKnmrahalekgftineytirp  292
DSSP  -----hHHHH--------------------hhllLHHHHHhhhhhhhhlleeeelleeee


DSSP  --hhllhhhhhhhhHHHHhhhhhhllllLLLLLlllllhhhhhhhhhllllllllllhh
Query --gllldadlxptrSACIaifearaqhpWPPRVatlphwpliyagalegldhlelartvd  273
ident               |
Sbjct lgvtgvageplpvdSEKD-------ifdYIQWK---------------------------  318
DSSP  llllllllllllllLHHH-------hhhHLLLL---------------------------


DSSP  hhhhhhhhhhhhhhhhllll
Query aaaqavqrfvaridratkr  292
ident
Sbjct ----------yrepkdrse  327
DSSP  ----------lllhhhlll
```

No 185: Query=mol1A Sbjct=8icgA Z-score=6.8

back to top

```
DSSP  ------------------------------------------------------------
Query ------------------------------------------------------------    0
ident
Sbjct etlnggitdmltelanfeknvsqaihkynayrkaasviakyphkiksgaeakklpgvgtk   60
DSSP  llllhhhhhhhhhhhhhhhhhllllhhhhhhhhhhhhhhhhhllllllllhhhhhllllllhh


DSSP  -------------------------------------------------llllllllllh
Query -------------------------------------------------aapysspptnl   11
ident
Sbjct iaekideflatgklrklekirqddtsssinfltrvsgigpsaarkfvdegiktledlrkn  120
DSSP  hhhhhhhhhhllllhhhhhhhhlhhhhhhhhhllllllhhhhhhhhhlllllhhhhhhl


DSSP  hhhhhhhhhhhhllllhhhhhHHHHHHHHHHHHH-LLLLllLLLLEEEEHHHHHHHHhhl
Query rslrdrltqvaerqgvvfgrlqRHVAXIVVAQFAA-TLTDdtGAPLLLVKGGSSLELrrg   70
ident                                                 | |
Sbjct edklnhhqriglkyfgdfekriPREEMLQMQDIVLnEVKKvdSEYIATVCGSFRRGA---  177
DSSP  hhhllhhhhhhhhlhhhhhlleEHHHHHHHHHHHHHhHHHHhlLLLEEEELLLLLLLL---


DSSP  lllllLLLLEEEEEEL------------lLHHHHHHHHHHHHhhllllleeEEELLLEELll
Query ipdsrTSKDFATVAR------------rDIELIHEQLADAGetgwegftAIFTAPEEIaa  118
ident       | |                         |||              |
Sbjct ----eSSGDMDVLLThpsftsestkqpkLLHQVVEQLQKVH--------FITDTLSKG--  223
DSSP  ----lEELLEEEEEEelllllllllllhHHHHHHHHHHHHHL--------LEEEEEEEL--


DSSP  llllLLLEEEEEEEEEELL--EEEEEEEEEEEEL-LLLLlllleeeeelhhhhllllllee
Query pgxpVKPRRFTAKLSYRG--RAFATVPIEVSSV-EAGNadqfdtltsdalglvgvpaava  175
ident              |             |
Sbjct ----ETKFMGVCQLPSKNdeKEYPHRRIDIRLIpKDQY----------------------  257
DSSP  ----LLEEEEEELLLLLLllLLLLLEEEEEEEEeHHHH----------------------


DSSP  eeellhHHHHhhhhhhhhllllllllllhhhhHHHHHH--------------------
Query vpcxtiPWQIaqklhavtavleepkandrahdlvDLQLLE--------------------  215
ident
Sbjct -----yCGVL--------------------yftgSDIFNKnmrahalekgftineytirp  292
DSSP  -----hHHHH--------------------hhhlLHHHHHhhhhhhhhllllllllll


DSSP  --hhllhhhhhhhhHHHHhhhhhhllllLLLLLlllllhhhhhhhhhllllllllllhh
Query --gllldadlxptrSACIaifearaqhpWPPRVatlphwpliyagalegldhlelartvd  273
```

```
Sbjct lgvtgvageplpvdSEKD-------ifdYIQWK---------------------------  318
DSSP  llllllllllllllLHHH-------hhhHLLLL---------------------------


DSSP  hhhhhhhhhhhhhhhllll
Query aaaqavqrfvaridratkr  292
ident
Sbjct ----------yrepkdrse  327
DSSP  ----------lllhhhlll
```

No 186: Query=mol1A Sbjct=1zqeA Z-score=6.8

back to top

```
DSSP  ------------------------------------------------------------
Query ------------------------------------------------------------    0
ident
Sbjct etlnggitdmltelanfeknvsqaihkynayrkaasviakyphkiksgaeakklpgvgtk   60
DSSP  llllhhhhhhhhhhhhhhhlllllhhhhhhhhhhhhhhhhlllllllhhhhhllllllhh


DSSP  -------------------------------------------------lllllllllh
Query -------------------------------------------------aapysspptnl   11
ident
Sbjct iaekideflatgklrklekirqddtsssinfltrvsgigpsaarkfvdegiktledlrkn  120
DSSP  hhhhhhhhhhllllhhhhhhhhlhhhhhhhhhllllllllhhhhhhhhhlllllhhhhhhl


DSSP  hhhhhhhhhhhhhllllhhhhhHHHHHHHHHHHHH-LLLLllLLLLLEEEEHHHHHHHHhl
Query rslrdrltqvaerqgvvfgrlqRHVAXIVVAQFAA-TLTDdtGAPLLLVKGGSSLELrrg   70
ident                                                 | |
Sbjct edklnhhqriglkyfgdfekriPREEMLQMQDIVLnEVKKvdSEYIATVCGSFRRGA---  177
DSSP  lllllhhhhhhhhlhhhhhlleEHHHHHHHHHHHHhHHHHhlLLLEEEEELLLLLL---


DSSP  lllllLLLLEEEEEL------------lLHHHHHHHHHHHHHhhlllleeEEELLLEELll
Query ipdsrTSKDFATVAR------------rDIELIHEQLADAGetgwegftAIFTAPEEIaa  118
ident       | |                         |||             |
Sbjct ----eSSGDMDVLLThpsftsestkqpkLLHQVVEQLQKVH--------FITDTLSKG--  223
DSSP  ----lEELLEEEEEEllllllllllhhHHHHHHHHHHHHLL--------LEEEEEELL--


DSSP  llllLLLEEEEEEEEELL--EEEEEEEEEEEEL-LLLLllllleeeeelhhhhllllllee
Query pgxpVKPRRFTAKLSYRG--RAFATVPIEVSSV-EAGNadqfdtltsdalglvgvpaava  175
ident              |             |
Sbjct ----ETKFMGVCQLPSKNdeKEYPHRRIDIRLIpKDQY----------------------  257
DSSP  ----LLEEEEEEELLLLLLllLLLLLLEEEEEEELLlHHHH----------------------


DSSP  eeellhHHHHhhhhhhhhllllllllllllhhhhHHHHHH--------------------
Query vpcxtiPWQIaqklhavtavleepkandrahdlvDLQLLE--------------------  215
ident
Sbjct -----yCGVL--------------------yftgSDIFNKnmrahalekgftineytirp  292
DSSP  -----hHHHH--------------------hhllLHHHHHhhhhhhllllleeeelleeee


DSSP  --hhllhhhhhhhhHHHHhhhhhhllllLLLLLlllllhhhhhhhhhllllllllllhh
Query --glllda dlxptrSACIaifearaqhpWPPRVatlphwpliyagalegldhlelartvd  273
ident               |
Sbjct lgvtgvageplpvdSEKD-------ifdYIQWK---------------------------  318
DSSP  llllllllllllllLHHH-------hhhHHLLL---------------------------


DSSP  hhhhhhhhhhhhhhhllll
Query aaaqavqrfvaridratkr  292
ident
Sbjct ----------yrepkdrse  327
DSSP  ----------lllhhhlll
```

No 187: Query=mol1A Sbjct=1zqgA Z-score=6.8

back to top

```
----
Query ------------------------------------------------------------    0
ident
Sbjct etlnggitdmltelanfeknvsqaihkynayrkaasviakyphkiksgaeakklpgvgtk   60
DSSP  llllhhhhhhhhhhhhhhhhllllhhhhhhhhhhhhhhhhlllllllhhhhhllllllhh


DSSP  -------------------------------------------------llllllllllh
Query -------------------------------------------------aapysspptnl   11
ident
Sbjct iaekideflatgklrklekirqddtsssinfltrvsgigpsaarkfvdegiktledlrkn  120
DSSP  hhhhhhhhhhllllhhhhhhhhlhhhhhhhhhlllllllhhhhhhhhhhhllllhhhhhl


DSSP  hhhhhhhhhhhhhllllhhhhhHHHHHHHHHHHHH-LLLLllLLLLEEEHHHHHHHHhhl
Query rslrdrltqvaerqgvvfgrlqRHVAXIVVAQFAA-TLTDdtGAPLLLVKGGSSLELrrg   70
ident                                                 | |
Sbjct edklnhhqriglkyfgdfekriPREEMLQMQDIVLnEVKKvdSEYIATVCGSFRRGA---  177
DSSP  hhhllhhhhhhhhlhhhhhlleEHHHHHHHHHHHHhHHHHhlLLLEEEEELLLLLLL---


DSSP  lllllLLLLEEEEEL------------lLHHHHHHHHHHHHhhlllleeEEELLLEELll
Query ipdsrTSKDFATVAR------------rDIELIHEQLADAGetgwegftAIFTAPEEIaa  118
ident       | |                         |||             |
Sbjct ----eSSGDMDVLLThpsftsestkqpkLLHQVVEQLQKVH--------FITDTLSKG--  223
DSSP  ----lEELLEEEEEEllllllllllllhHHHHHHHHHHHHL--------LEEEEEEEL--


DSSP  llllLLLEEEEEEEEELL--EEEEEEEEEEEELLLLLLllleeeeelhhhhlllllleee
Query pgxpVKPRRFTAKLSYRG--RAFATVPIEVSSVEAGNAdqfdtltsdalglvgvpaavav  176
ident              |             |
Sbjct ----ETKFMGVCQLPSKNdeKEYPHRRIDIRLIPKDQY----------------------  257
DSSP  ----LLEEEEEELLLLLLllLLLLLEEEEEELLLLLLH----------------------


DSSP  eellhHHHHhhhhhhhhhlllllllllllhhhhHHHHHH---------------------
Query pcxtiPWQIaqklhavtavleepkandrahdlvDLQLLE---------------------  215
ident
Sbjct ----yCGVL--------------------yftgSDIFNKnmrahalekgftineytirpl  293
DSSP  ----hHHHH--------------------hhllLHHHHHhhhhhhhhlleeeelleeeel


DSSP  -hhllhhhhhhhhHHHHhhhhhhllllLLLLLlllllhhhhhhhhhlllllllllllhhh
Query -gllldadlxptrSACIaifearaqhpWPPRVatlphwpliyagalegldhlelartvda  274
ident              |
Sbjct gvtgvageplpvdSEKD-------ifdYIQWK----------------------------  318
DSSP  lllllllllllllLHHH-------hhhHLLLL----------------------------


DSSP  hhhhhhhhhhhhhhllll
Query aaqavqrfvaridratkr  292
ident
Sbjct ---------yrepkdrse  327
DSSP  ---------lllhhhlll
```

No 188: Query=mol1A Sbjct=1zqrA Z-score=6.8

back to top

```
DSSP  ------------------------------------------------------------
Query ------------------------------------------------------------    0
ident
Sbjct etlnggitdmltelanfeknvsqaihkynayrkaasviakyphkiksgaeakklpgvgtk   60
DSSP  llllhhhhhhhhhhhhhhhlllllhhhhhhhhhhhhhhhhlllllllhhhhhllllllhh


DSSP  -------------------------------------------------llllllllllh
Query -------------------------------------------------aapysspptnl   11
ident
Sbjct iaekideflatgklrklekirqddtsssinfltrvsgigpsaarkfvdegiktledlrkn  120
DSSP  hhhhhhhhhhllllhhhhhhhhlhhhhhhhhhlllllllhhhhhhhhhhhllllhhhhll


DSSP  hhhhhhhhhhhhhllllhhhhhHHHHHHHHHHHHH-LLLLllLLLLEEEHHHHHHHHhhl
Query rslrdrltqvaerqgvvfgrlqRHVAXIVVAQFAA-TLTDdtGAPLLLVKGGSSLELrrg   70
ident                                                 | |
Sbjct edklnhhqriglkyfgdfekriPREEMLQMQDIVLnEVKKvdSEYIATVCGSFRRGA---  177
```

```
DSSP  [illegible]


DSSP  lllllLLLLEEEEEL------------lLHHHHHHHHHHHHhhlllleeEEELLLEELll
Query ipdsrTSKDFATVAR------------rDIELIHEQLADAGetgwegftAIFTAPEEIaa  118
ident       | |                         |||             |
Sbjct ----eSSGDMDVLLThpsftsestkqpkLLHQVVEQLQKVH--------FITDTLSKG--  223
DSSP  ----lEELLEEEEEEllllllllllhhHHHHHHHHHHHHLL--------LEEEEEEEL--


DSSP  llllLLLEEEEEEEEELL--EEEEEEEEEEEEL-LLLLlllleeeeelhhhhllllllee
Query pgxpVKPRRFTAKLSYRG--RAFATVPIEVSSV-EAGNadqfdtltsdalglvgvpaava  175
ident              |             |
Sbjct ----ETKFMGVCQLPSKNdeKEYPHRRIDIRLIpKDQY----------------------  257
DSSP  ----LLLEEEEELLLLLllLLLLLEEEEEELLlHHHH----------------------


DSSP  eeellhHHHHhhhhhhhhllllllllllhhhhHHHHHH--------------------
Query vpcxtiPWQIaqklhavtavleepkandrahdlvDLQLLE--------------------  215
ident
Sbjct -----yCGVL--------------------yftgSDIFNKnmrahalekgftineytirp  292
DSSP  -----hHHHH--------------------hhllLHHHHHhhhhhhhhlleeeelleeee


DSSP  --hhllhhhhhhhhHHHHhhhhhhllllLLLLLllllhhhhhhhhhllllllllllhh
Query --glllladadlxptrSACIaifearaqhpWPPRVatlphwpliyagalegldhlelartvd  273
ident               |
Sbjct lgvtgvageplpvdSEKD-------ifdYIQWK---------------------------  318
DSSP  llllllllllllllLHHH-------hhhHLLLL---------------------------


DSSP  hhhhhhhhhhhhhhhllll
Query aaaqavqrfvaridratkr  292
ident
Sbjct ----------yrepkdrse  327
DSSP  ----------lllhhhlll
```

No 189: Query=mol1A Sbjct=1zqqA Z-score=6.8

back to top

```
DSSP  ------------------------------------------------------------
Query ------------------------------------------------------------    0
ident
Sbjct etlnggitdmltelanfeknvsqaihkynayrkaasviakyphkiksgaeakklpgvgtk   60
DSSP  llllhhhhhhhhhhhhhhhhhlllllhhhhhhhhhhhhhhhhhllllllllhhhhllllllllhh


DSSP  -------------------------------------------------llllllllllh
Query -------------------------------------------------aapysspptnl   11
ident
Sbjct iaekideflatgklrklekirqddtsssinfltrvsgigpsaarkfvdegiktledlrkn  120
DSSP  hhhhhhhhhhllllhhhhhhhhlhhhhhhhhhhlllllhhhhhhhhhlllllhhhhhhl


DSSP  hhhhhhhhhhhhhllllhhhhhHHHHHHHHHHHHHH-LLLLllLLLLEEEEHHHHHHHHhhl
Query rslrdrltqvaerqgvvfgrlqRHVAXIVVAQFAA-TLTDdtGAPLLLVKGGSSLELrrg   70
ident                                                 | |
Sbjct edklnhhqriglkyfgdfekriPREEMLQMQDIVLnEVKKvdSEYIATVCGSFRRGA---  177
DSSP  lllllhhhhhhhhlhhhhhhlleEHHHHHHHHHHHHHhHHHHhlLLLEEEEELHHHHLLL---


DSSP  lllllLLLLEEEEEL------------lLHHHHHHHHHHHHhhlllleeEEELLLEELll
Query ipdsrTSKDFATVAR------------rDIELIHEQLADAGetgwegftAIFTAPEEIaa  118
ident       | |                         |||             |
Sbjct ----eSSGDMDVLLThpsftsestkqpkLLHQVVEQLQKVH--------FITDTLSKG--  223
DSSP  ----lEELLEEEEEEellllllllllhHHHHHHHHHHHHHL--------LEEEEEEEL--


DSSP  llllLLLEEEEEEEEELL--EEEEEEEEEEEEL-LLLLlllleeeeelhhhhllllllee
Query pgxpVKPRRFTAKLSYRG--RAFATVPIEVSSV-EAGNadqfdtltsdalglvgvpaava  175
ident              |             |
Sbjct ----ETKFMGVCQLPSKNdeKEYPHRRIDIRLIpKDQY----------------------  257
DSSP  ----LLEEEEEELLLLLLllLLLLLEEEEEEEEeLLLH----------------------


DSSP  eeellhHHHHhhhhhhhhllllllllllhhhhHHHHHH--------------------
```

```
ident
Sbjct -----yCGVL--------------------yftgSDIFNKnmrahalekgftineytirp  292
DSSP  -----hHHHH--------------------hhllLHHHHHhhhhhhhhlleeeelleeee


DSSP  -hhllhhhhhhhHHHHHhhhhhhllllLLLLLlllllhhhhhhhhhlllllllllllhhh
Query -glllldadlxptRSACIaifearaqhpWPPRVatlphwpliyagalegldhlelartvda  274
ident
Sbjct lgvtgvageplpVDSEK------difdYIQWK----------------------------  318
DSSP  llllllllllllLLLHH------hhhhHLLLL----------------------------


DSSP  hhhhhhhhhhhhhhllll
Query aaqavqrfvaridratkr  292
ident
Sbjct ---------yrepkdrse  327
DSSP  ---------lllhhhlll
```

No 190: Query=mol1A Sbjct=7icpA Z-score=6.8

back to top

```
DSSP  ------------------------------------------------------------
Query ------------------------------------------------------------    0
ident
Sbjct etlnggitdmltelanfeknvsqaihkynayrkaasviakyphkiksgaeakklpgvgtk   60
DSSP  llllhhhhhhhhhhhhhhhhllllhhhhhhhhhhhhhhhhlllllllhhhhhllllllhh


DSSP  -------------------------------------------------llllllllllh
Query -------------------------------------------------aapysspptnl   11
ident
Sbjct iaekideflatgklrklekirqddtsssinfltrvsgigpsaarkfvdegiktledlrkn  120
DSSP  hhhhhhhhhhllllhhhhhhlllhhhhhhhhhlllllllhhhhhhhhhhllllhhhhhhl


DSSP  hhhhhhhhhhhhhllllhhhhhHHHHHHHHHHHHHH-LLLLllLLLLEEEHHHHHHHHhhl
Query rslrdrltqvaerqgvvfgrlqRHVAXIVVAQFAA-TLTDdtGAPLLLVKGGSSLELrrg   70
ident                                                 | |
Sbjct edklnhhqriglkyfgdfekriPREEMLQMQDIVLnEVKKvdSEYIATVCGSFRRGA---  177
DSSP  lllllhhhhhhhhlhhhhhlleEHHHHHHHHHHHHhHHHHhlLLLEEEELLHHHLLL---


DSSP  lllllLLLLEEEEEL------------lLHHHHHHHHHHHHhhlllleeEEELLLEELll
Query ipdsrTSKDFATVAR------------rDIELIHEQLADAGetgwegftAIFTAPEEIaa  118
ident       | |                         |||             |
Sbjct ----eSSGDMDVLLThpsftsestkqpkLLHQVVEQLQKVH--------FITDTLSKG--  223
DSSP  ----lEELLEEEEEEllllllllllllhHHHHHHHHHHHHL--------LEEEEEEEL--


DSSP  llllLLLEEEEEEEEELL--EEEEEEEEEEEEL-LLLLlllleeeeelhhhhllllllee
Query pgxpVKPRRFTAKLSYRG--RAFATVPIEVSSV-EAGNadqfdtltsdalglvgvpaava  175
ident              |             |
Sbjct ----ETKFMGVCQLPSKNdeKEYPHRRIDIRLIpKDQY----------------------  257
DSSP  ----LLEEEEEEELLLLLllLLLLLEEEEEEELlHHHH----------------------


DSSP  eeellhHHHHhhhhhhhhhlllllllllllhhhhHHHHHH--------------------
Query vpcxtiPWQIaqklhavtavleepkandrahdlvDLQLLE--------------------  215
ident
Sbjct -----yCGVL--------------------yftgSDIFNKnmrahalekgftineytirp  292
DSSP  -----hHHHH--------------------hhhlLHHHHHhhhhhhhhlleeeelleeee


DSSP  -hhllhhhhhhhHHHHHhhhhhhllllLLLLLlllllhhhhhhhhhlllllllllllhhh
Query -glllldadlxptRSACIAifearaqhpWPPRVatlphwpliyagalegldhlelartvda  274
ident
Sbjct lgvtgvageplpVDSEKD------ifdYIQWK----------------------------  318
DSSP  llllllllllllLLLHHH------hhhHLLLL----------------------------


DSSP  hhhhhhhhhhhhhhllll
Query aaqavqrfvaridratkr  292
ident
Sbjct ---------yrepkdrse  327
DSSP  ---------llllhhhll
```

No 191: Query=mol1A Sbjct=1zqiA Z-score=6.8

back to top

```
DSSP  ------------------------------------------------------------
Query ------------------------------------------------------------   0
ident 
Sbjct etlnggitdmltelanfeknvsqaihkynayrkaasviakyphkiksgaeakklpgvgtk   60
DSSP  llllhhhhhhhhhhhhhhhlllllhhhhhhhhhhhhhhlllllllllhhhhhllllllhh


DSSP  -------------------------------------------------llllllllllh
Query -------------------------------------------------aapysspptnl   11
ident 
Sbjct iaekideflatgklrklekirqddtsssinfltrvsgigpsaarkfvdegiktledlrkn  120
DSSP  hhhhhhhhhhllllhhhhhhhhlhhhhhhhhhllllllllhhhhhhhhhllllhhhhhhl


DSSP  hhhhhhhhhhhhhllllhhhhhHHHHHHHHHHHHH-LLLLllLLLLEEEEHHHHHHHHhhl
Query rslrdrltqvaerqgvvfgrlqRHVAXIVVAQFAA-TLTDdtGAPLLLVKGGSSLELrrg   70
ident                                                 | |
Sbjct edklnhhqriglkyfgdfekriPREEMLQMQDIVLnEVKKvdSEYIATVCGSFRRGA---  177
DSSP  hhhllhhhhhhhhlhhhhhlleEHHHHHHHHHHHHHhHHHHhlLLLEEEEEELLLLLLL---


DSSP  lllllLLLLEEEEEEL------------lLHHHHHHHHHHHHhhlllleeEEELLLEELll
Query ipdsrTSKDFATVAR------------rDIELIHEQLADAGetgwegftAIFTAPEEIaa  118
ident       | |                         |||             |
Sbjct ----eSSGDMDVLLThpsftsestkqpkLLHQVVEQLQKVH--------FITDTLSKG--  223
DSSP  ----lEELLEEEEEEllllllllllhhHHHHHHHHHHHHLL--------LEEEEEEEL--


DSSP  llllLLLEEEEEEEEEELL--EEEEEEEEEEEEL-LLLLllllleeeeelhhhhllllllee
Query pgxpVKPRRFTAKLSYRG--RAFATVPIEVSSV-EAGNadqfdtltsdalglvgvpaava  175
ident              |             |
Sbjct ----ETKFMGVCQLPSKNdeKEYPHRRIDIRLIpKDQY----------------------  257
DSSP  ----LLEEEEEELLLLLLlLLLLLEEEEEELLlLLLH----------------------


DSSP  eeellhHHHHhhhhhhhhllllllllllhhhhHHHHHH--------------------
Query vpcxtiPWQIaqklhavtavleepkandrahdlvDLQLLE--------------------  215
ident 
Sbjct -----yCGVL--------------------yftgSDIFNKnmrahalekgftineytirp  292
DSSP  -----hHHHH--------------------hhllLHHHHHhhhhhhhhlleeeelleeee


DSSP  --hhllhhhhhhhhHHHHhhhhhhllllLLLLLllllhhhhhhhhhllllllllllllhh
Query --glllldadlxptrSACIaifearaqhpWPPRVatlphwpliyagalegldhlelartvd  273
ident               |
Sbjct lgvtgvageplpvdSEKD-------ifdYIQWK---------------------------  318
DSSP  llllllllllllllLHHH-------hhhHLLLL---------------------------


DSSP  hhhhhhhhhhhhhhhhllll
Query aaaqavqrfvaridratkr  292
ident 
Sbjct ----------yrepkdrse  327
DSSP  ----------lllhhhlll
```

No 192: Query=mol1A Sbjct=1zqfA Z-score=6.8

back to top

```
DSSP  ------------------------------------------------------------
Query ------------------------------------------------------------   0
ident 
Sbjct etlnggitdmltelanfeknvsqaihkynayrkaasviakyphkiksgaeakklpgvgtk   60
DSSP  llllhhhhhhhhhhhhhhhhlllllhhhhhhhhhhhhhhhhlllllllhhhhhllllllhh


DSSP  -------------------------------------------------llllllllllh
Query -------------------------------------------------aapysspptnl   11
ident 
```

```
DSSP  hhhhhhhhhhllllhhhhhhhhlhhhhhhhhhllllllhhhhhhhhhhllllhhhhhhl


DSSP  hhhhhhhhhhhhhllllhhhhhHHHHHHHHHHHHH-LLLLllLLLLLEEEEHHHHHHHHhhl
Query rslrdrltqvaerqgvvfgrlqRHVAXIVVAQFAA-TLTDdtGAPLLLVKGGSSLELrrg   70
ident                                                 | |
Sbjct edklnhhqriglkyfgdfekriPREEMLQMQDIVLnEVKKvdSEYIATVCGSFRRGA---  177
DSSP  hhhllhhhhhhhhlhhhhhlleEHHHHHHHHHHHHHhHHHHhlLLLEEEEELLLLLLLL---


DSSP  lllllLLLLEEEEEL------------lLHHHHHHHHHHHHHhhllllleeEEELLLEELll
Query ipdsrTSKDFATVAR------------rDIELIHEQLADAGetgwegftAIFTAPEEIaa  118
ident       | |                         |||             |
Sbjct ----eSSGDMDVLLThpsftsestkqpkLLHQVVEQLQKVH--------FITDTLSKG--  223
DSSP  ----lEELLEEEEEEellllllllllhhHHHHHHHHHHHHLL--------LEEEEEEEL--


DSSP  llllLLLEEEEEEEEELL--EEEEEEEEEEEL-LLLLllllleeeeelhhhhllllllee
Query pgxpVKPRRFTAKLSYRG--RAFATVPIEVSSV-EAGNadqfdtltsdalglvgvpaava  175
ident              |             |
Sbjct ----ETKFMGVCQLPSKNdeKEYPHRRIDIRLIpKDQY----------------------  257
DSSP  ----LLEEEEEELLLLLllLLLLLLEEEEEEEEeHHHH----------------------


DSSP  eeellhHHHHhhhhhhhhllllllllllhhhhHHHHHH--------------------
Query vpcxtiPWQIaqklhavtavleepkandrahdlvDLQLLE--------------------  215
ident
Sbjct -----yCGVL--------------------yftgSDIFNKnmrahalekgftineytirp  292
DSSP  -----hHHHH--------------------hhhlLHHHHHhhhhhhhhlleeeelleeee


DSSP  -hhllhhhhhhhHHHHHhhhhhhllllLLLLLlllllhhhhhhhhhllllllllllhhh
Query -gllldadlxptRSACIaifearaqhpWPPRVatlphwpliyagalegldhlelartvda  274
ident
Sbjct lgvtgvageplpVDSEK-------difDYIQW----------------------------  317
DSSP  llllllllllllLLLHH-------hhhHHLLL----------------------------


DSSP  hhhhhhhhhhhhhhllll
Query aaqavqrfvaridratkr  292
ident
Sbjct --------kyrepkdrse  327
DSSP  --------llllhhhlll
```

No 193: Query=mol1A Sbjct=1zqpA Z-score=6.8

back to top

```
DSSP  ------------------------------------------------------------
Query ------------------------------------------------------------    0
ident
Sbjct etlnggitdmltelanfeknvsqaihkynayrkaasviakyphkiksgaeakklpgvgtk   60
DSSP  llllhhhhhhhhhhhhhhhhllllhhhhhhhhhhhhhhhhlllllllhhhhhlllllllhh


DSSP  -------------------------------------------------llllllllllh
Query -------------------------------------------------aapysspptnl   11
ident
Sbjct iaekideflatgklrklekirqddtsssinfltrvsgigpsaarkfvdegiktledlrkn  120
DSSP  hhhhhhhhhhllllhhhhhhhhlhhhhhhhhhllllllhhhhhhhhhhllllhhhhhhl


DSSP  hhhhhhhhhhhhhllllhhhhhHHHHHHHHHHHHH-LLLLllLLLLLEEEEHHHHHHHHhhl
Query rslrdrltqvaerqgvvfgrlqRHVAXIVVAQFAA-TLTDdtGAPLLLVKGGSSLELrrg   70
ident                                                 | |
Sbjct edklnhhqriglkyfgdfekriPREEMLQMQDIVLnEVKKvdSEYIATVCGSFRRGA---  177
DSSP  hhhllhhhhhhhhlhhhhhlleEHHHHHHHHHHHHHhHHHHhlLLLEEEEELLLLLLLL---


DSSP  lllllLLLLEEEEEL------------lLHHHHHHHHHHHHHhhllllleeEEELLLEELll
Query ipdsrTSKDFATVAR------------rDIELIHEQLADAGetgwegftAIFTAPEEIaa  118
ident       | |                         |||             |
Sbjct ----eSSGDMDVLLThpsftsestkqpkLLHQVVEQLQKVH--------FITDTLSKG--  223
DSSP  ----lEELLEEEEEEellllllllllhhHHHHHHHHHHHHLL--------LEEEEEEEL--
```

```
Query pgxpVKPRRFTAKLSYRG--RAFATVPIEVSSV-EAGNadqfdtltsdalglvgvpaava  175
ident              |             |
Sbjct ----ETKFMGVCQLPSKNdeKEYPHRRIDIRLIpKDQY----------------------  257
DSSP  ----LLEEEEEELLLLLLllLLLLLLEEEEEEEEeHHHH----------------------


DSSP  eeellhHHHHhhhhhhhhlllllllllllhhhhHHHHHH--------------------
Query vpcxtiPWQIaqklhavtavleepkandrahdlvDLQLLE--------------------  215
ident
Sbjct -----yCGVL--------------------yftgSDIFNKnmrahalekgftineytirp  292
DSSP  -----hHHHH--------------------hhhlLHHHHHhhhhhhhhlleeeelleeee


DSSP  hhllhhhhhhhHHHHhhhhhhhllllLLLLLLllllhhhhhhhhhllllllllllhhhh
Query glllddadlxptRSACiaifearaqhpWPPRVAtlphwpliyagalegldhlelartvdaa  275
ident
Sbjct lgvtgvageplPVDS------ekdifDYIQWK----------------------------  318
DSSP  lllllllllllLLLL------hhhhhHHLLLL----------------------------


DSSP  hhhhhhhhhhhhhllll
Query aqavqrfvaridratkr  292
ident
Sbjct --------yrepkdrse  327
DSSP  --------lllhhhlll
```

No 194: Query=mol1A Sbjct=9iceA Z-score=6.8

back to top

```
DSSP  ------------------------------------------------------------
Query ------------------------------------------------------------    0
ident
Sbjct etlnggitdmltelanfeknvsqaihkynayrkaasviakyphkiksgaeakklpgvgtk   60
DSSP  llllhhhhhhhhhhhhhhhhhhlllhhhhhhhhhhhhhhhhlllllllhhhhhlllllhh


DSSP  -------------------------------------------------llllllllllh
Query -------------------------------------------------aapyssppptnl   11
ident
Sbjct iaekideflatgklrklekirqddtsssinfltrvsgigpsaarkfvdegiktledlrkn  120
DSSP  hhhhhhhhhhllllhhhhhhhhlhhhhhhhhhlllllllhhhhhhhhhlllllhhhhhhl


DSSP  hhhhhhhhhhhhhllllhhhhhHHHHHHHHHHHHHH-LLLLllLLLLLEEEEHHHHHHHhhl
Query rslrdrltqvaerqgvvfgrlqRHVAXIVVAQFAA-TLTDdtGAPLLLVKGGSSLELrrg   70
ident                                                 | |
Sbjct edklnhhqriglkyfgdfekriPREEMLQMQDIVLnEVKKvdSEYIATVCGSFRRGA---  177
DSSP  hhhllhhhhhhhhlhhhhhlleEHHHHHHHHHHHHHhHHHHhlLLLEEEEELLLLLLL---


DSSP  lllllLLLLEEEEEL------------lLHHHHHHHHHHHHhhlllleeEEELLLEELll
Query ipdsrTSKDFATVAR------------rDIELIHEQLADAGetgwegftAIFTAPEEIaa  118
ident       | |                         |||             |
Sbjct ----eSSGDMDVLLThpsftsestkqpkLLHQVVEQLQKVH--------FITDTLSKG--  223
DSSP  ----lEELLEEEEEElllllllllllllhHHHHHHHHHHHHHLL--------LEEEEEEEL--


DSSP  llllLLLEEEEEEEEEELL--EEEEEEEEEEEEL-LLLLlllleeeeelhhhhllllllee
Query pgxpVKPRRFTAKLSYRG--RAFATVPIEVSSV-EAGNadqfdtltsdalglvgvpaava  175
ident              |             |
Sbjct ----ETKFMGVCQLPSKNdeKEYPHRRIDIRLIpKDQY----------------------  257
DSSP  ----LLEEEEEELLLLLLllLLLLLLEEEEEELLlLLLH----------------------


DSSP  eeellhHHHHhhhhhhhhlllllllllllhhhhHHHHHH--------------------
Query vpcxtiPWQIaqklhavtavleepkandrahdlvDLQLLE--------------------  215
ident
Sbjct -----yCGVL--------------------yftgSDIFNKnmrahalekgftineytirp  292
DSSP  -----hHHHH--------------------hhllLHHHHHhhhhhhhlllllleelleell


DSSP  -hhllhhhhhhhHHHHhhhhhhhllllLLLLLlllllhhhhhhhhhllllllllllhhh
Query -glllddadlxptRSACIaifearaqhpWPPRVatlphwpliyagalegldhlelartvda  274
ident
Sbjct lgvtgvageplpVDSEK-------difDYIQW----------------------------  317
```

```
DSSP  hhhhhhhhhhhhhhllll
Query aaqavqrfvaridratkr  292
ident
Sbjct --------kyrepkdrse  327
DSSP  --------llllllllll
```

No 195: Query=mol1A Sbjct=9icaA Z-score=6.8

back to top

```
DSSP  ------------------------------------------------------------
Query ------------------------------------------------------------    0
ident
Sbjct etlnggitdmltelanfeknvsqaihkynayrkaasviakyphkiksgaeakklpgvgtk   60
DSSP  llllhhhhhhhhhhhhhhlllllhhhhhhhhhhhhhhhlllllllhhhhhllllllhh


DSSP  -------------------------------------------------lllllllllh
Query -------------------------------------------------aapysspptnl   11
ident
Sbjct iaekideflatgklrklekirqddtsssinfltrvsgigpsaarkfvdegiktledlrkn  120
DSSP  hhhhhhhhhllllhhhhhhhhlhhhhhhhhhllllllhhhhhhhhhhllllhhhhhhl


DSSP  hhhhhhhhhhhhllllhhhhhHHHHHHHHHHHHHH-LLLLllLLLLEEEEHHHHHHHhhl
Query rslrdrltqvaerqgvvfgrlqRHVAXIVVAQFAA-TLTDdtGAPLLLVKGGSSLELrrg   70
ident                                                 | |
Sbjct edklnhhqriglkyfgdfekriPREEMLQMQDIVLnEVKKvdSEYIATVCGSFRRGA---  177
DSSP  hhhllhhhhhhhhlllllllleEHHHHHHHHHHHHHhHHHHhlLLLEEEELLLLLLLL---


DSSP  lllllLLLLEEEEEL------------lLHHHHHHHHHHHHhhlllleeEEELLLEELll
Query ipdsrTSKDFATVAR------------rDIELIHEQLADAGetgwegftAIFTAPEEIaa  118
ident       | |                         |||             |
Sbjct ----eSSGDMDVLLThpsftsestkqpkLLHQVVEQLQKVH--------FITDTLSKG--  223
DSSP  ----lEELLEEEEEEelllllllllllhHHHHHHHHHHHHLL--------LEEEEEEEL--


DSSP  llllLLLEEEEEEEEEELL--EEEEEEEEEEEEL-LLLLlllleeeeelhhhhlllllee
Query pgxpVKPRRFTAKLSYRG--RAFATVPIEVSSV-EAGNadqfdtltsdalglvgvpaava  175
ident              |             |
Sbjct ----ETKFMGVCQLPSKNdeKEYPHRRIDIRLIpKDQY----------------------  257
DSSP  ----LLEEEEEELLLLLLlLLLLLLEEEEEEEEEeHHHH----------------------


DSSP  eeellhHHHHhhhhhhhhllllllllllllhhhhHHHHHH--------------------
Query vpcxtiPWQIaqklhavtavleepkandrahdlvDLQLLE--------------------  215
ident
Sbjct -----yCGVL--------------------yftgSDIFNKnmrahalekgftineytirp  292
DSSP  -----hHHHH--------------------hhhlLHHHHHhhhhhhhhlleeeelleeee


DSSP  -hhllhhhhhhhHHHHHHhhhhhllllLLLLLllllllhhhhhhhhlllllllllllhhh
Query -glllldadlxptRSACIAifearaqhpWPPRVatlphwpliyagalegldhlelartvda  274
ident
Sbjct lgvtgvageplpVDSEKD------ifdYIQWK----------------------------  318
DSSP  lllllllllllLLLHHH------hhhHLLLL----------------------------


DSSP  hhhhhhhhhhhhhhllll
Query aaqavqrfvaridratkr  292
ident
Sbjct ---------yrepkdrse  327
DSSP  ---------lllhhhlll
```

No 196: Query=mol1A Sbjct=3c2mA Z-score=6.8

back to top

```
DSSP  ------------------------------------------------------------
Query ------------------------------------------------------------    0
```

```
Sbjct tlnggitdmltelanfeknvsqaihkynayrkaasviakyphkiksgaeakklpgvgtki   60
DSSP  lllhhhhhhhhhhhhhhhhllllhhhhhhhhhhhhhhhhllllllhhhhhlllllhhh


DSSP  ------------------------------------------------llllllllllhh
Query ------------------------------------------------aapysspptnlr   12
ident
Sbjct aekideflatgklrklekirqddtsssinfltrvsgigpsaarkfvdegiktledlrkne  120
DSSP  hhhhhhhhhlllhhhhhhhhlhhhhhhhhhllllllhhhhhhhhhlllllhhhhhhlh


DSSP  hhhhhhhhhhhllllhhhhhHHHHHHHHHHHHHH-LLLLLLLLLLEEEEHHHHHHHhhll
Query slrdrltqvaerqgvvfgrlqRHVAXIVVAQFAA-TLTDDTGAPLLLVKGGSSLELrrgi   71
ident                                                | |
Sbjct dklnhhqriglkyfgdfekriPREEMLQMQDIVLnEVKKVDSEYIATVCGSFRRGA----  176
DSSP  hhllhhhhhhhhlhhhhhlleEHHHHHHHHHHHHHhHHHHHLLLLEEEELHHHHLLL----


DSSP  llllLLLLEEEEEL------------lLHHHHHHHHHHHHhhlllleeEEELLLEELlll
Query pdsrTSKDFATVAR------------rDIELIHEQLADAGetgwegftAIFTAPEEIaap  119
ident      | |                         |||             |
Sbjct ---eSSGDMDVLLThpsftsestkqpkLLHQVVEQLQKVH--------FITDTLSKG---  222
DSSP  ---lEELLEEEEEElllllllllllHHHHHHHHHHHLL--------LEEEEEEEL---


DSSP  lllLLLEEEEEEEEEL--LEEEEEEEEEEEEL-LLLLllllleeeeelhhhhllllllleee
Query gxpVKPRRFTAKLSYR--GRAFATVPIEVSSV-EAGNadqfdtltsdalglvgvpaavav  176
ident             |             |
Sbjct ---ETKFMGVCQLPSKndEKEYPHRRIDIRLIpKDQY-----------------------  256
DSSP  ---LLEEEEEELLLLLllLLLLLLEEEEEEELlHHHH-----------------------


DSSP  eellhHHHHhhhhhhhlllllllllllhhhhHHHHHHHHL------lHHHH-------
Query pcxtiPWQIaqklhavtavleepkandrahdlvDLQLLEGLL------lDADL-------  223
ident
Sbjct ----yCGVL--------------------yftgSDIFNKNMRahalekgFTINeytirpl  292
DSSP  ----hHHHH--------------------hhhlLHHHHHHHHhhhhhllEEELllleeel


DSSP  --------hhhHHHHHhhhhhhllllLLLLLlllllhhhhhhhhhlllllllllllhhhh
Query --------xptRSACIaifearaqhpWPPRVatlphwpliyagalegldhlelartvdaa  275
ident
Sbjct gvtgvageplpVDSEK-------difDYIQW-----------------------------  316
DSSP  llllllllllLLLHH-------hhhHHLLL-----------------------------


DSSP  hhhhhhhhhhhhhllll
Query aqavqrfvaridratkr  292
ident
Sbjct -------kyrepkdrse  326
DSSP  -------llllhhhlll
```

No 197: Query=mol1A Sbjct=1huzA Z-score=6.8

back to top

```
DSSP  ------------------------------------------------------------
Query ------------------------------------------------------------    0
ident
Sbjct tlnggitdmlvelanfeknvsqaihkynayrkaasviakyphkiksgaeakklpgvgtki   60
DSSP  lllhhhhhhhhhhhhhhhhllllhhhhhhhhhhhhhhhlllllllllhhhhllllllllhhh


DSSP  ------------------------------------------------llllllllllhh
Query ------------------------------------------------aapysspptnlr   12
ident
Sbjct aekideflatgklrklekirqddtsssinfltrvtgigpsaarklvdegiktledlrkne  120
DSSP  hhhhhhhhhlllhhhhhhhhlhhhhhhhhhllllllhhhhhhhhhlllllhhhhllll


DSSP  hhhhhhhhhhhllllhhhhhHHHHHHHHHHHHHHL-LLLLLLLLLEEEEHHHHHHHhhll
Query slrdrltqvaerqgvvfgrlqRHVAXIVVAQFAAT-LTDDTGAPLLLVKGGSSLELrrgi   71
ident                                                | |
Sbjct dklnhhqriglkyfedfekriPREEMLQMQDIVLNeVKKLDPEYIATVCGSFRRGA----  176
DSSP  llllhhhhhhhhlhhhhhlleEHHHHHHHHHHHHHHhHHHHLLLLEEEELLLLLLLL----
```

```
DSSP  llllLLLLEEEEEL------------lLHHHHHHHHHHHHhhlllleeEEELLLEELlll
Query pdsrTSKDFATVAR------------rDIELIHEQLADAGetgwegftAIFTAPEEIaap  119
ident      | |                         |||             |
Sbjct ---eSSGDMDVLLThpnftsesskqpkLLHRVVEQLQKVR--------FITDTLSKG---  222
DSSP  ---lEELLEEEEEEllllllllllhHHHHHHHHHHHLL--------LEEEEEEEL---


DSSP  lllLLLEEEEEEEEEL--lEEEEEEEEEEEEL-LLLLlllleeeeelhhhhllllleee
Query gxpVKPRRFTAKLSYR--gRAFATVPIEVSSV-EAGNadqfdtltsdalglvgvpaavav  176
ident             |             |
Sbjct ---ETKFMGVCQLPSEndeNEYPHRRIDIRLIpKDQY-----------------------  256
DSSP  ---LLEEEEEEELLLLLlllLLLLLEEEEEEEELlHHHH-----------------------


DSSP  eellhHHHHhhhhhhhhllllllllllHHHHHHH-------------------hhhhh
Query pcxtiPWQIaqklhavtavleepkandraHDLVDLQ-------------------llegl  217
ident                               |
Sbjct ----yCGVL----------------yftgSDIFNKNmrahalekgftineytirplgvtg  296
DSSP  ----hHHHH----------------hlllLHHHHHHhhhhhhhhleeellllleeelllll


DSSP  llhHHHHhhHHHHHhhhhhhllllLLLLLLllllhhhhhhhhhllllllllllhhhhhh
Query lldADLXptRSACIaifearaqhpWPPRVAtlphwpliyagalegldhlelartvdaaaq  277
ident      |
Sbjct vagEPLP--VDSEQ-------difDYIQWR------------------------------  317
DSSP  lllLLLL--LLLHH-------hhhHHLLLL------------------------------


DSSP  hhhhhhhhhhhllll
Query avqrfvaridratkr  292
ident
Sbjct -------yrepkdrs  325
DSSP  -------lllhhhll
```

No 198: Query=mol1A Sbjct=4pgxA Z-score=6.8

back to top

```
DSSP  ------------------------------------------------------------
Query ------------------------------------------------------------    0
ident
Sbjct tlnggitdmltelanfeknvsqaihkynayrkaasviakyphkiksgaeakklpgvgtki   60
DSSP  lllhhhhhhhhhhhhhhhhllllhhhhhhhhhhhhhhhlllllllhhhhhhllllllhhh


DSSP  ------------------------------------------------lllllllllhh
Query ------------------------------------------------aapysspptnlr   12
ident
Sbjct aekideflatgklrklekirqddtsssinfltrvsgigpsaarkfvdegiktledlrkne  120
DSSP  hhhhhhhhhhlllhhhhhhhhlhhhhhhhhhlllllllhhhhhhhhhllllhhhhhhllh


DSSP  hhhhhhhhhhhhllllhhhhhHHHHHHHHHHHHH-LLLLllLLLLEEEEHHHHHHHHhhll
Query slrdrltqvaerqgvvfgrlqRHVAXIVVAQFAA-TLTDdtGAPLLLVKGGSSLELrrgi   71
ident                                                | |
Sbjct dklnhhqriglkyfgdfekriPREEMLQMQDIVLnEVKKvdSEYIATVCGSFRRGA----  176
DSSP  hhllhhhhhhhhlhhhhhlleEHHHHHHHHHHHHHhHHHHhlLLLEEEEELHHHHLLL----


DSSP  llllLLLLEEEEEL------------lLHHHHHHHHHHHHhhlllleeEEELLLEElllll
Query pdsrTSKDFATVAR------------rDIELIHEQLADAGetgwegftAIFTAPEEiaap  119
ident      | |                         |||             |
Sbjct ---eSSGDMDVLLThpsftsestkqpkLLHQVVEQLQKVH--------FITDTLSK----  221
DSSP  ---lEELLEEEEEElllllllllllllHHHHHHHHHHHLL--------LEEEEEEE----


DSSP  lllLLLEEEEEEEEEL--LEEEEEEEEEEEEL-LLLLlllleeeeelhhhhllllleee
Query gxpVKPRRFTAKLSYR--GRAFATVPIEVSSV-EAGNadqfdtltsdalglvgvpaavav  176
ident             |             |
Sbjct --gETKFMGVCQLPSKndEKEYPHRRIDIRLIpKDQY-----------------------  256
DSSP  --lLLEEEEEEELLLLLllLLLLLLEEEEEEEELlHHHH-----------------------


DSSP  eellhHHHHhhhhhhhhllllllllllHHHH-------------------hhhhhhhh
Query pcxtiPWQIaqklhavtavleepkandraHDLV-------------------dlqllegl  217
ident                               |
```

```
DSSP  ----hHHHH----------------hhhlLHHHhhhhhhhhhhhleeellllleeellllll


DSSP  llhHHHHHhhHHHHhhhhhhlllLLLLLLlllllhhhhhhhhhlllllllllllhhhhhh
Query lldADLXPtrSACIaifearaqhPWPPRVatlphwpliyagalegldhlelartvdaaaq  277
ident      |
Sbjct vagEPLPV--DSEK-----difdYIQWKY-------------------------------  318
DSSP  lllLLLLL--LLHH-----hhhhHLLLLL-------------------------------


DSSP  hhhhhhhhhhhllll
Query avqrfvaridratkr  292
ident
Sbjct -------repkdrse  326
DSSP  -------llhhhlll
```

No 199: Query=mol1A Sbjct=4ubbA Z-score=6.8

back to top

```
DSSP  ------------------------------------------------------------
Query ------------------------------------------------------------    0
ident
Sbjct tlnggitdmltelanfeknvsqaihkynayrkaasviakyphkiksgaeakklpgvgtki   60
DSSP  lllhhhhhhhhhhhhhhhhhllllhhhhhhhhhhhhhhhhhllllllhhhhhhlllllhhh


DSSP  ------------------------------------------------llllllllllhh
Query ------------------------------------------------aapysspptnlr   12
ident
Sbjct aekideflatgklrklekirqddtsssinfltrvsgigpsaarkfvdegiktledlrkne  120
DSSP  hhhhhhhhhhlllhhhhhhhhlhhhhhhhhhlllllllhhhhhhhhhlllllhhhhhhhlh


DSSP  hhhhhhhhhhhllllhhhhhHHHHHHHHHHHHH-LLLLllLLLLLEEEEHHHHHHHHhhll
Query slrdrltqvaerqgvvfgrlqRHVAXIVVAQFAA-TLTDdtGAPLLLVKGGSSLELrrgi   71
ident                                                |  |
Sbjct dklnhhqriglkyfgdfekriPREEMLQMQDIVLnEVKKvdSEYIATVCGSFRRGA----  176
DSSP  hhllhhhhhhhhlhhhhhlleEHHHHHHHHHHHHHhHHHHhlLLLEEEEELHHHHLLL----


DSSP  llllLLLLEEEEEL------------lLHHHHHHHHHHHHhhlllleeEEELLLEEllll
Query pdsrTSKDFATVAR------------rDIELIHEQLADAGetgwegftAIFTAPEEiaap  119
ident      | |                         |||             |
Sbjct ---eSSGDMDVLLThpsftsestkqpkLLHQVVEQLQKVH--------FITDTLSK----  221
DSSP  ---lEELLEEEEEEllllllllllllHHHHHHHHHHHHLL--------LEEEEEEE----


DSSP  lllLLLEEEEEEEEEL--LEEEEEEEEEEEEL-LLLLllllleeeeelhhhhllllllleee
Query gxpVKPRRFTAKLSYR--GRAFATVPIEVSSV-EAGNadqfdtltsdalglvgvpaavav  176
ident             |             |
Sbjct --gETKFMGVCQLPSKndEKEYPHRRIDIRLIpKDQY-----------------------  256
DSSP  --lLLEEEEEEELLLLLllLLLLLLEEEEEEEELlHHHH-----------------------


DSSP  eellhHHHHhhhhhhhhllllllllllllHHHH-------------------hhhhhhhh
Query pcxtiPWQIaqklhavtavleepkandraHDLV-------------------dlqllegl  217
ident                               |
Sbjct ----yCGVL----------------yftgSDIFnknmrahalekgftineytirplgvtg  296
DSSP  ----hHHHH----------------hhhlLHHHhhhhhhhhhhhleeellllleeellllll


DSSP  llhHHHHHhhHHHHhhhhhhlllLLLLLLlllllhhhhhhhhhlllllllllllhhhhhh
Query lldADLXPtrSACIaifearaqhPWPPRVatlphwpliyagalegldhlelartvdaaaq  277
ident      |
Sbjct vagEPLPV--DSEK-----difdYIQWKY-------------------------------  318
DSSP  lllLLLLL--LLHH-----hhhhHLLLLL-------------------------------


DSSP  hhhhhhhhhhhllll
Query avqrfvaridratkr  292
ident
Sbjct -------repkdrse  326
DSSP  -------llhhhlll
```

No 200: Query=mol1A Sbjct=1ymoA Z-score=6.8

back to top

```
DSSP  ------------------------------------------------------------
Query ------------------------------------------------------------   0
ident                                                               
Sbjct tlnggitdmltelanfeknvsqaihkynayrkaasviakyphkiksgaeakklpgvgtki  60
DSSP  lllhhhhhhhhhhhhhhhhllllhhhhhhhhhhhhhhhhlllllllhhhhhlllllllhhh


DSSP  ------------------------------------------------llllllllllhh
Query ------------------------------------------------aapysspptnlr  12
ident                                                               
Sbjct aekideflatgklrklekirqddtsssinfltrvsgigpsaarkfvdegiktledlrkne 120
DSSP  hhhhhhhhhhllhhhhhhhhlhhhhhhhhhllllllllhhhhhhhhhlllllhhhhhhlh


DSSP  hhhhhhhhhhhlllllhhhhhHHHHHHHHHHHHH-LLLLllLLLLEEEEHHHHHHHhhll
Query slrdrltqvaerqgvvfgrlqRHVAXIVVAQFAA-TLTDdtGAPLLLVKGGSSLELrrgi  71
ident                                                | |          
Sbjct dklnhhqriglkyfgdfekriPREEMLQMQDIVLnEVKKvdSEYIATVCGSFRRGA---- 176
DSSP  hhllhhhhhhhhlhhhhhlleEHHHHHHHHHHHHhHHHHhlLLLEEEELHHHHLLL----


DSSP  llllLLLLEEEEEL------------lLHHHHHHHHHHHHhhlllleeEEELLLEElllll
Query pdsrTSKDFATVAR------------rDIELIHEQLADAGetgwegftAIFTAPEEiaap 119
ident      | |                         |||             |          
Sbjct ---eSSGDMDVLLThpsftsestkqpkLLHQVVEQLQKVH--------FITDTLSK---- 221
DSSP  ---lEELLEEEEEEllllllllllllHHHHHHHHHHHLL--------LEEEEEEE----


DSSP  lllLLLEEEEEEEEEL--LEEEEEEEEEEEEL-LLLLllllleeeeelhhhhlllllleee
Query gxpVKPRRFTAKLSYR--GRAFATVPIEVSSV-EAGNadqfdtltsdalglvgvpaavav 176
ident             |             |                                 
Sbjct --gETKFMGVCQLPSKndEKEYPHRRIDIRLIpKDQY----------------------- 256
DSSP  --lLLEEEEEELLLLLllLLLLLLEEEEEEEELlHHHH----------------------


DSSP  eellhHHHHhhhhhhhhlllllllllllHHHH-------------------hhhhhhhh
Query pcxtiPWQIaqklhavtavleepkandraHDLV-------------------dlqllegl 217
ident                               |                             
Sbjct ----yCGVL----------------yftgSDIFnknmrahalekgftineytirplgvtg 296
DSSP  ----hHHHH----------------hhhlLHHHhhhhhhhhhhlleeellllleeellll


DSSP  llhHHHHHhhHHHHhhhhhhlllLLLLLLLlllllhhhhhhhhhlllllllllllhhhhhh
Query lldADLXPtrSACIaifearaqhPWPPRVatlphwpliyagalegldhlelartvdaaaq 277
ident      |                                                      
Sbjct vagEPLPV--DSEK-----difdYIQWKY------------------------------- 318
DSSP  lllLLLLL--LLHH-----hhhhHLLLLL-------------------------------


DSSP  hhhhhhhhhhhllll
Query avqrfvaridratkr  292
ident                 
Sbjct -------repkdrse  326
DSSP  -------llhhhlll
```

No 201: Query=mol1A Sbjct=7iceA Z-score=6.8

back to top

```
DSSP  ------------------------------------------------------------
Query ------------------------------------------------------------   0
ident                                                               
Sbjct etlnggitdmltelanfeknvsqaihkynayrkaasviakyphkiksgaeakklpgvgtk  60
DSSP  llllhhhhhhhhhhhhhhhhllllhhhhhhhhhhhhhhhhlllllllhhhhhllllllhh


DSSP  -------------------------------------------------llllllllllh
Query -------------------------------------------------aapysspptnl  11
ident                                                               
Sbjct iaekideflatgklrklekirqddtsssinfltrvsgigpsaarkfvdegiktledlrkn 120
DSSP  hhhhhhhhhhllllhhhhhhhhlhhhhhhhhhlllllllhhhhhhhhllllllhhhhhhl
```

```
DSSP  hhhhhhhhhhhhhllllhhhhhHHHHHHHHHHHHH-LLLLllLLLLEEEEHHHHHHHHhhl
Query rslrdrltqvaerqgvvfgrlqRHVAXIVVAQFAA-TLTDdtGAPLLLVKGGSSLELrrg   70
ident                                                  | |
Sbjct edklnhhqriglkyfgdfekriPREEMLQMQDIVLnEVKKvdSEYIATVCGSFRRGA---  177
DSSP  hhhllhhhhhhhhlhhhhhlleEHHHHHHHHHHHHhHHHHhlLLLEEEELLHHHHLL---


DSSP  lllllLLLLEEEEEL------------lLHHHHHHHHHHHHhhllllleeEEELLLEELll
Query ipdsrTSKDFATVAR------------rDIELIHEQLADAGetgwegftAIFTAPEEIaa  118
ident       | |                          |||             |
Sbjct ----eSSGDMDVLLThpsftsestkqpkLLHQVVEQLQKVH--------FITDTLSKG--  223
DSSP  ----lEELLEEEEEEelllllllllhhHHHHHHHHHHHLL--------LEEEEEEEL--


DSSP  llllLLLEEEEEEEEEELL--EEEEEEEEEEEEL-LLLLllllleeeeelhhhhllllllee
Query pgxpVKPRRFTAKLSYRG--RAFATVPIEVSSV-EAGNadqfdtltsdalglvgvpaava  175
ident              |              |
Sbjct ----ETKFMGVCQLPSKNdeKEYPHRRIDIRLIpKDQY----------------------  257
DSSP  ----LLEEEEEELLLLLLlLLLLLLEEEEEEEEEeHHHH----------------------


DSSP  eeellhHHHHHHhhhhhhlllllllllllhhhhHHHHHH--------------------
Query vpcxtiPWQIAQklhavtavleepkandrahdlvDLQLLE--------------------  215
ident
Sbjct -----yCGVLYF--------------------tgSDIFNKnmrahalekgftineytirp  292
DSSP  -----hHHHHHH--------------------hlLHHHHHhhhhhhhhlleeellllleee


DSSP  --hhllhhhhhhhhhHHHHhhhhhhllllLLLLLlllllhhhhhhhhhllllllllllhh
Query --glllldadlxptrSACIaifearaqhpWPPRVatlphwpliyagalegldhlelartvd  273
ident               |
Sbjct lgvtgvageplpvdSEKD-------ifdYIQWK---------------------------  318
DSSP  llllllllllllllLHHH-------hhhHLLLL---------------------------


DSSP  hhhhhhhhhhhhhhhhllll
Query aaaqavqrfvaridratkr  292
ident
Sbjct ----------yrepkdrse  327
DSSP  ----------lllhhhlll
```

No 202: Query=mol1A Sbjct=4r64A Z-score=6.8

back to top

```
DSSP  ------------------------------------------------------------
Query ------------------------------------------------------------    0
ident
Sbjct tlnggitdmltelanfeknvsqaihkynayrkaasviakyphkiksgaeakklpgvgtki   60
DSSP  lllhhhhhhhhhhhhhhhhhllllhhhhhhhhhhhhhhhhhlllllllhhhhhlllllhhhh


DSSP  ------------------------------------------------llllllllllhh
Query ------------------------------------------------aapysspptnlr   12
ident
Sbjct aekideflatgklrklekirqddtsssinfltrvsgigpsaarkfvdegiktledlrkne  120
DSSP  hhhhhhhhhhlllhhhhhhhhhlhhhhhhhhhlllllllhhhhhhhhhlllllhhhhhllh


DSSP  hhhhhhhhhhhhllllhhhhhHHHHHHHHHHHHHH-LLLLllLLLLEEEEHHHHHHHHhhll
Query slrdrltqvaerqgvvfgrlqRHVAXIVVAQFAA-TLTDdtGAPLLLVKGGSSLELrrgi   71
ident                                                 | |
Sbjct dklnhhqriglkyfgdfekriPREEMLQMQDIVLnEVKKvdSEYIATVCGSFRRGA----  176
DSSP  hhllhhhhhhhhlhhhhhlleEHHHHHHHHHHHHhHHHHhlLLLEEEELHHHHLLL----


DSSP  llllLLLLEEEEEL------------lLHHHHHHHHHHHHhhllllleeEEELLLEElllll
Query pdsrTSKDFATVAR------------rDIELIHEQLADAGetgwegftAIFTAPEEiaap  119
ident      | |                          |||             |
Sbjct ---eSSGDMDVLLThpsftsestkqpkLLHQVVEQLQKVH--------FITDTLSK----  221
DSSP  ---lEELLEEEEEEllllllllllllHHHHHHHHHHHLL--------LEEEEEEE----


DSSP  lllLLLEEEEEEEEEELL--EEEEEEEEEEEEL-LLLLllllleeeeelhhhhllllllleee
Query gxpVKPRRFTAKLSYRG--RAFATVPIEVSSV-EAGNadqfdtltsdalglvgvpaavav  176
ident             |              |
```

```
DSSP  --lLLEEEEEEELLLLLllLLLLLEEEEEEELlHHHH-----------------------


DSSP  eellhHHHhhhhhhhhhllllllllllllHHHH-------------------hhhhhhhh
Query pcxtiPWQIaqklhavtavleepkandraHDLV-------------------dlqllegl  217
ident                               |
Sbjct ----yCGVL----------------yftgSDIFnknmrahalekgftinkytirplgvtg  296
DSSP  ----hHHHH----------------hhhlLHHHhhhhhhhhhhlleeellllleeelllll


DSSP  llhhHHHHhhHHHHHhhhhhlllLLLLLllllllhhhhhhhhhllllllllllhhhhhhh
Query lldaDLXPtrSACIAifearaqhPWPPRvatlphwpliyagalegldhlelartvdaaaq  277
ident      |
Sbjct vagePLPV--DSEKD-----ifdYIQWK--------------------------------  317
DSSP  llllLLLL--LLHHH-----hhhHLLLL--------------------------------


DSSP  hhhhhhhhhhhllll
Query avqrfvaridratkr  292
ident
Sbjct ------yrepkdrse  326
DSSP  ------lllhhhlll
```

No 203: Query=mol1A Sbjct=4ymmA Z-score=6.8

back to top

```
DSSP  ------------------------------------------------------------
Query ------------------------------------------------------------    0
ident
Sbjct pqetlnggitdmltelanfeknvsqaihkynayrkaasviakyphkiksgaeakklpgvg   60
DSSP  llhhhhhhhhhhhhhhhhhhhhhllllhhhhhhhhhhhhhhhhllllllllhhhhhllllll


DSSP  ---------------------------------------------------lllllllll
Query ---------------------------------------------------aapysssppt    9
ident
Sbjct tkiaekideflatgklrklekirqddtsssinfltrvsgigpsaarkfvdegiktledlr  120
DSSP  hhhhhhhhhhhhhlllhhhhhhhhlhhhhhhhhhlllllllhhhhhhhhhlllllhhhhh


DSSP  lhhhhhhhhhhhhhhllllhhhhhHHHHHHHHHHHHHH-LLLLllLLLLEEEEHHHHHHHh
Query nlrslrdrltqvaerqgvvfgrlqRHVAXIVVAQFAA-TLTDdtGAPLLLVKGGSSLELr   68
ident                                                   | |
Sbjct knedklnhhqriglkyfgdfekriPREEMLQMQDIVLnEVKKvdSEYIATVCGSFRRGA-  179
DSSP  hlhhhllhhhhhhhhlhhhhllleEHHHHHHHHHHHHHhHHHHhlLLLEEEELHHHHLLL-


DSSP  hllllllLLLLEEEEEEL----------lLHHHHHHHHHHHHhhllllleeEEELLLEElll
Query rgipdsrTSKDFATVAR----------rDIELIHEQLADAGetgwegftAIFTAPEEiaa  118
ident         | |                       |||             |
Sbjct ------eSSGDMDVLLThpsftsesqpkLLHQVVEQLQKVH--------FITDTLSK---  222
DSSP  ------lEELLEEEEEEllllllllllHHHHHHHHHHHLL--------LEEEEEEE---


DSSP  llllLLLEEEEEEEEEEL--LEEEEEEEEEEEEL-LLLLlllleeeeelhhhhlllllllee
Query pgxpVKPRRFTAKLSYR--GRAFATVPIEVSSV-EAGNadqfdtltsdalglvgvpaava  175
ident              |             |
Sbjct ---gETKFMGVCQLPSKndEKEYPHRRIDIRLIpKDQY----------------------  257
DSSP  ---lLLEEEEEEELLLLLllLLLLLEEEEEEELlHHHH----------------------


DSSP  eeellhHHHHhhhhhhhhhllllllllllllHHHH-------------------hhhhhhh
Query vpcxtiPWQIaqklhavtavleepkandraHDLV-------------------dlqlleg  216
ident                                |
Sbjct -----yCGVL----------------yftgSDIFnknmrahalekgftineytirplgvt  296
DSSP  -----hHHHH----------------hlllLHHHhhhhhhhhhllleeellllleeellll


DSSP  hllhhHHHHhhHHHHHhhhhhlllLLLLLllllllhhhhhhhhhlllllllllllhhhhh
Query llldaDLXPtrSACIAifearaqhPWPPRvatlphwpliyagalegldhlelartvdaaa  276
ident       |
Sbjct gvagePLPV--DSEKD-----ifdYIQWK-------------------------------  318
DSSP  lllllLLLL--LLHHH-----hhhHLLLL-------------------------------
```

```
Query qavqrfvaridratkr  292
ident
Sbjct -------yrepkdrse  327
DSSP  -------lllhhhlll
```

No 204: Query=mol1A Sbjct=1bpeA Z-score=6.8

back to top

```
DSSP  ------------------------------------------------------------
Query ------------------------------------------------------------    0
ident
Sbjct nggitdmlvelanfgaeakklpekideflatgklrklekirrddtsssinfltrvtgigp   60
DSSP  lhhhhhhhhhhhhlllllllllllllhhhhlllhhhhllllllllhhhhhhhhllllllh


DSSP  ---------lllllllllhhhhhhhhhhhhhhhllllhHHHHHHHHHHHHHHHHH-LLLL
Query ---------aapysspptnlrslrdrltqvaerqgvvfGRLQRHVAXIVVAQFAA-TLTD   50
ident
Sbjct saarklvdegiktledlrknedklnhhqriglkyfedfEKRIPREEMLQMQDIVLnEVKK  120
DSSP  hhhhhhhhlllllhhhhhhlhhhllhhhhhhhhlhhhhLLLLLHHHHHHHHHHHHhHHHH


DSSP  llLLLLEEEEHHHHHHHhhllllllLLLLEEEEEL------------lLHHHHHHHHHHH
Query dtGAPLLLVKGGSSLELrrgipdsrTSKDFATVAR------------rDIELIHEQLADA   98
ident         | |               | |                         |||
Sbjct ldPEYIATVCGSFRRGA-------eSSGDMDVLLThpnftsesskqpkLLHRVVEQLQKV  173
DSSP  hlLLLEEEELHHHHHLL-------lLLLLLLEEEEllllllllllllhHHHHHHHHHHHL


DSSP  HhhlllleeEEELLLEELllllllLLLEEEEEEEEELLeEEEEEEEEEEEL-LLLLllll
Query GetgwegftAIFTAPEEIaapgxpVKPRRFTAKLSYRGrAFATVPIEVSSV-EAGNadqf  157
ident           |                      |           |
Sbjct R--------FITDTLSKG------ETKFMGVCQLPSEN-EYPHRRIDIRLIpKDQY----  214
DSSP  L--------LLLEELLLL------LLEEEEELLLLLLL-LLLLLEEEEEELlLLLH----


DSSP  eeeeelhhhhlllllleeeeellhHHHHhhhhhhhhhllllllllllllhhhhHHHHHH--
Query dtltsdalglvgvpaavavpcxtiPWQIaqklhavtavleepkandrahdlvDLQLLE--  215
ident
Sbjct -----------------------yCGVL--------------------yftgSDIFNKnm  231
DSSP  -----------------------hHHHH--------------------hhhlLLLHHHhh


DSSP  -------------------hhllhhhhhhhHHHHHhhhhhhllllLLLLLLllllhhhhh
Query -------------------gllldadlxptRSACIaifearaqhpWPPRVAtlphwpliy  256
ident
Sbjct rahalekgftineytirplgvtgvageplpVDSEQ-------difDYIQWR---------  275
DSSP  hhhhhhlleellllllleellllllllllllLLLLH-------hhhHHLLLL---------


DSSP  hhhhlllllllllllhhhhhhhhhhhhhhhhhllll
Query agalegldhlelartvdaaaqavqrfvaridratkr  292
ident
Sbjct ---------------------------yrepkdrse  284
DSSP  ---------------------------lllhhhlll
```

No 205: Query=mol1A Sbjct=4ubcA Z-score=6.8

back to top

```
DSSP  ------------------------------------------------------------
Query ------------------------------------------------------------    0
ident
Sbjct tlnggitdmltelanfeknvsqaihkynayrkaasviakyphkiksgaeakklpgvgtki   60
DSSP  lllhhhhhhhhhhhhhhhhllllhhhhhhhhhhhhhhhhllllllhhhhhlllllllhhh


DSSP  ------------------------------------------------llllllllllhh
Query ------------------------------------------------aapysspptnlr   12
ident
Sbjct aekideflatgklrklekirqddtsssinfltrvsgigpsaarkfvdegiktledlrkne  120
DSSP  hhhhhhhhhhlllhhhhhhhhlhhhhhhhhhllllllhhhhhhhhhhlllllhhhhhhlh
```

```
DSSP  hhhhhhhhhhhhllllhhhhhHHHHHHHHHHHHH-LLLLllLLLLEEEEHHHHHHHHhhll
Query slrdrltqvaerqgvvfgrlqRHVAXIVVAQFAA-TLTDdtGAPLLLVKGGSSLELrrgi   71
ident                                                |  |
Sbjct dklnhhqriglkyfgdfekriPREEMLQMQDIVLnEVKKvdSEYIATVCGSFRRGA----  176
DSSP  hhllhhhhhhhhlhhhhhlleEHHHHHHHHHHHHHhHHHHhlLLLEEEELHHHHLLL----
```

```
DSSP  llllLLLLEEEEEL------------lLHHHHHHHHHHHHhhlllleeEEELLLEElll l
Query pdsrTSKDFATVAR------------rDIELIHEQLADAGetgwegftAIFTAPEEiaap  119
ident      | |                         |||             |
Sbjct ---eSSGDMDVLLThpsftsestkqpkLLHQVVEQLQKVH--------FITDTLSK----  221
DSSP  ---lEELLEEEEEEllllllllllllHHHHHHHHHHHLL--------LEEEEEEE----
```

```
DSSP  lllLLLEEEEEEEEEL--LEEEEEEEEEEEEL-LLLLlllleeeeelhhhhllllleee
Query gxpVKPRRFTAKLSYR--GRAFATVPIEVSSV-EAGNadqfdtltsdalglvgvpaavav  176
ident             |             |
Sbjct --gETKFMGVCQLPSKndEKEYPHRRIDIRLIpKDQY-----------------------  256
DSSP  --lLLEEEEEELLLLLllLLLLLLEEEEEEEELlHHHH-----------------------
```

```
DSSP  eellhHHHHhhhhhhhhllllllllllHHHH-------------------hhhhhhhh
Query pcxtiPWQIaqklhavtavleepkandraHDLV-------------------dlqllegl  217
ident                               |
Sbjct ----yCGVL----------------yftgSDIFnknmrahalekgftineytirplgvtg  296
DSSP  ----hHHHH----------------hhhlLHHHhhhhhhhhhhlleeellllleeelllll
```

```
DSSP  llhHHHHHhhHHHHhhhhhhlllLLLLLLlllllhhhhhhhhhllllllllllhhhhhh
Query lldADLXPtrSACIaifearaqhPWPPRVatlphwpliyagalegldhlelartvdaaaq  277
ident      |
Sbjct vagEPLPV--DSEK-----difdYIQWKY-------------------------------  318
DSSP  lllLLLLL--LLHH-----hhhhHLLLLL-------------------------------
```

```
DSSP  hhhhhhhhhhhllll
Query avqrfvaridratkr  292
ident
Sbjct -------repkdrse  326
DSSP  -------llhhhlll
```

No 206: Query=mol1A Sbjct=5j2bA Z-score=6.8

back to top

```
DSSP  ------------------------------------------------------------
Query ------------------------------------------------------------    0
ident
Sbjct tlnggitdmltelanfeknvsqaihkynayrkaasviakyphkiksgaeakklpgvgtki   60
DSSP  lllhhhhhhhhhhhhhhhhlllllhhhhhhhhhhhhhhhhlllllllhhhhhhllllhhh
```

```
DSSP  ------------------------------------------------llllllllllhh
Query ------------------------------------------------aapysspptnlr   12
ident
Sbjct aekideflatgklrklekirqddtsssinfltrvsgigpsaarkfvdegiktledlrkne  120
DSSP  hhhhhhhhhhlllhhhhhhhhlhhhhhhhhhlllllllhhhhhhhhhhllllhhhhhllh
```

```
DSSP  hhhhhhhhhhhhllllhhhhhHHHHHHHHHHHHH-LLLLllLLLLEEEEHHHHHHHHhhll
Query slrdrltqvaerqgvvfgrlqRHVAXIVVAQFAA-TLTDdtGAPLLLVKGGSSLELrrgi   71
ident                                                |  |
Sbjct dklnhhqriglkyfgdfekriPREEMLQMQDIVLnEVKKvdSEYIATVCGSFRRGA----  176
DSSP  hhllhhhhhhhhlllhhhlleEHHHHHHHHHHHHHhHHHHhlLLLEEEELHHHHLLL----
```

```
DSSP  llllLLLLEEEEEL------------lLHHHHHHHHHHHHhhlllleeEEELLLEElll l
Query pdsrTSKDFATVAR------------rDIELIHEQLADAGetgwegftAIFTAPEEiaap  119
ident      | |                         |||             |
Sbjct ---eSSGDMDVLLThpsftsestkqpkLLHQVVEQLQKVH--------FITDTLSK----  221
DSSP  ---lEELLEEEEEEllllllllllllHHHHHHHHHHHLL--------LEEEEEEE----
```

```
DSSP  lllLLLEEEEEEEEEL--LEEEEEEEEEEEEL-LLLLlllleeeeelhhhhllllleee
Query gxpVKPRRFTAKLSYR--GRAFATVPIEVSSV-EAGNadqfdtltsdalglvgvpaavav  176
```

```
Sbjct --gETKFMGVCQLPSKndEKEYPHRRIDIRLIpKDQY-----------------------  256
DSSP  --lLLEEEEEELLLLLllLLLLLLEEEEEEELlHHHH-----------------------


DSSP  eellhHHHHhhhhhhhhllllllllllllHHHH-------------------hhhhhhhh
Query pcxtiPWQIaqklhavtavleepkandraHDLV-------------------dlqllegl  217
ident                               |
Sbjct ----yCGVL----------------yftgSDIFnknmrahalekgftineytirplgvtg  296
DSSP  ----hHHHH----------------hhhlLHHHhhhhhhhhhhhleeellllleeelllll


DSSP  llhHHHHHhhHHHHhhhhhhlllLLLLLLlllllhhhhhhhhhllllllllllhhhhhhh
Query lldADLXPtrSACIaifearaqhPWPPRVatlphwpliyagalegldhlelartvdaaaq  277
ident      |
Sbjct vagEPLPV--DSEK-----difdYIQWKY-------------------------------  318
DSSP  lllLLLLL--LLHH-----hhhhHHLLLL-------------------------------


DSSP  hhhhhhhhhhllll
Query avqrfvaridratkr  292
ident
Sbjct -------repkdrse  326
DSSP  -------llhhhhll
```

No 207: Query=mol1A Sbjct=4mf2A Z-score=6.8

back to top

```
DSSP  ------------------------------------------------------------
Query ------------------------------------------------------------    0
ident
Sbjct lnggitdmltelanfeknvsqaihkynayrkaasviakyphkiksgaeakklpgvgtkia   60
DSSP  llhhhhhhhhhhhhhhhhhllllhhhhhhhhhhhhhhhhlllllllhhhhhlllllhhhh


DSSP  -----------------------------------------------llllllllllhhh
Query -----------------------------------------------aapysspptnlrs   13
ident
Sbjct ekideflatgklrklekirqddtsssinfltrvsgigpsaarkfvdegiktledlrkned  120
DSSP  hhhhhhhhhlllhhhhhhhllhhhhhhhhhhlllllllhhhhhhhhhlllllhhhhhhlhh


DSSP  hhhhhhhhhhhllllhHHHHHHHHHHHHHHHHHL-LLLLLLLLLEEEEHHHHHHHhhlll
Query lrdrltqvaerqgvvfGRLQRHVAXIVVAQFAAT-LTDDTGAPLLLVKGGSSLELrrgip   72
ident                                               | |
Sbjct klnhhqriglkyfgdfEKRIPREEMLQMQDIVLNeVKKVDSEYIATVCGSFRRGA-----  175
DSSP  hllhhhhhhhhlhhhhLLLEEHHHHHHHHHHHHHHhHHHHLLLLEEEEELHHHHLLL-----


DSSP  lllLLLLEEEEEL--------lLHHHHHHHHHHHHhhllllleeEEELLLEELllllllLL
Query dsrTSKDFATVAR--------rDIELIHEQLADAGetgwegftAIFTAPEEIaapgxpVK  124
ident     | |                     |||              |
Sbjct --eSSGDMDVLLThpsftsepkLLHQVVEQLQKVH--------FITDTLSKG------ET  219
DSSP  --lEELLEEEEEEllllllllHHHHHHHHHHHHLL--------LEEEEEEEL------LL


DSSP  LEEEEEEEEEL--LEEEEEEEEEEEEL-LLLLlllleeeeelhhhhlllllleeeeellh
Query PRRFTAKLSYR--GRAFATVPIEVSSV-EAGNadqfdtltsdalglvgvpaavavpcxti  181
ident        |                 |
Sbjct KFMGVCQLPSKndEKEYPHRRIDIRLIpKDQY---------------------------y  252
DSSP  EEEEEELLLLLllLLLLLLEEEEEEELlHHHH---------------------------h


DSSP  HHHHhhhhhhhhllllllllllllhhhhHHHHHH----------------------hhll
Query PWQIaqklhavtavleepkandrahdlvDLQLLE----------------------glll  219
ident
Sbjct CGVL--------------------yftgSDIFNKnmrahalekgftineytirplgvtgv  292
DSSP  HHHH--------------------hhhlLHHHHHhhhhhhhhlleeellllleeellllll


DSSP  hhhhhhhhHHHHhhhhhhllllLLLLLllllhhhhhhhhhlllllllllllhhhhhhhhh
Query dadlxptrSACIaifearaqhpWPPRVatlphwpliyagalegldhlelartvdaaaqav  279
ident         |
Sbjct ageplpvdSEKD-------ifdYIQWK---------------------------------  312
DSSP  llllllllLHHH-------hhhHLLLL---------------------------------
```

```
DSSP  hhhhhhhhhllll
Query qrfvaridratkr  292
ident
Sbjct ----yrepkdrse  321
DSSP  ----lllhhhlll
```

No 208: Query=mol1A Sbjct=5cr0A Z-score=6.8

back to top

```
DSSP  -------------------------llllllllllhhhhhhhhhhhhhhllllhHHHHHH
Query -------------------------aapysspptnlrslrdrltqvaerqgvvfGRLQRH   35
ident
Sbjct esvpvlelfsniwgagtktaqmwyqqgfrsledirsqaslttqqaiglkhysdfLERMPR   60
DSSP  lhhhhhhhhhllllllhhhhhhhhhlllllhhhhhhhlllllhhhhhhhhlhhhhLLLEEH


DSSP  HHHHHHHHHHHLL-LLLLLLLLEEEEHHHHHHHhhllllllLLLLEEEEEL--------l
Query VAXIVVAQFAATL-TDDTGAPLLLVKGGSSLELrrgipdsrTSKDFATVAR--------r   86
ident        |              |     |              |  |
Sbjct EEATEIEQTVQKAaQAFNSGLLCVACGSYRRGK-------aTCGDVDVAIThpdgrshrg  113
DSSP  HHHHHHHHHHHHHHhHHHLLLLEEEEEHHHHHLL-------lEELLEEEEEEllllllll


DSSP  LHHHHHHHHHHHHHhhllllleeEEELLLEELLLllLLLLLEEEEEEEEEllEEEEEEEEEE
Query DIELIHEQLADAGetgwegftAIFTAPEEIAApgXPVKPRRFTAKLSYrgRAFATVPIEV  146
ident         |   |                                 |
Sbjct IFSRLLDSLRQEG--------FLTDDLVSQEE-nGQQQKYLGVCRLPG--PGRRHRRLDI  162
DSSP  HHHHHHHHHHHHL--------LEEEEEEEEEE-eLLEEEEEEEEELLLL--LLLLLEEEEE


DSSP  EEL-LLLLlllleeeeelhhhhllllllleeeeellhHHHHhhhhhhhlllllllllll
Query SSV-EAGNadqfdtltsdalglvgvpaavavpcxtiPWQIaqklhavtavleepkandra  205
ident    |
Sbjct IVVpYSEF---------------------------aCALL----------------yftg  179
DSSP  EELlLLLH---------------------------hHHHH----------------hhhl


DSSP  HHHHHHH-----------------------hhhhhllhhhhhhhHHHHHhhhhhhllllL
Query HDLVDLQ-----------------------lleglllldadlxptRSACIaifearaqhpW  242
ident
Sbjct SAHFNRSmralaktkgmslsehalstavvrnthgckvgpgrvlpTPTEK-------dvfR  232
DSSP  LHHHHHHhhhhhhhllleellllleeellllllllllllleeelLLLHH-------hhhH


DSSP  LLLLLllllhhhhhhhhhllllllllllhhhhhhhhhhhhhhhhllll
Query PPRVAtlphwpliyagalegldhlelartvdaaaqavqrfvaridratkr  292
ident
Sbjct LLGLP------------------------------------yrepaerdw  246
DSSP  HLLLL------------------------------------lllhhhhll
```

No 209: Query=mol1A Sbjct=5cp2B Z-score=6.8

back to top

```
DSSP  -------------------------llllllllllhhhhhhhhhhhhhhllllhHHHHHH
Query -------------------------aapysspptnlrslrdrltqvaerqgvvfGRLQRH   35
ident
Sbjct esvpvlelfsniwgagtktaqmwyqqgfrsledirsqaslttqqaiglkhysdfLERMPR   60
DSSP  lhhhhhhhhhllllllhhhhhhhhhlllllhhhhhhhlllllhhhhhhhhlhhhhHLLEEH


DSSP  HHHHHHHHHHHLL-LLLLLLLLEEEEHHHHHHHhhllllllLLLLEEEEEL--------l
Query VAXIVVAQFAATL-TDDTGAPLLLVKGGSSLELrrgipdsrTSKDFATVAR--------r   86
ident        |              |     |              |  |
Sbjct EEATEIEQTVQKAaQAFNSGLLCVACGSYRRGK-------aTCGDVDVAIThpdgrshrg  113
DSSP  HHHHHHHHHHHHHHhHHHLLLLEEEELHHHHLLL-------lEELLEEEEEEllllllll


DSSP  LHHHHHHHHHHHHHhhllllleeEEELLLEELLLllLLLLLEEEEEEEEEllEEEEEEEEEE
Query DIELIHEQLADAGetgwegftAIFTAPEEIAApgXPVKPRRFTAKLSYrgRAFATVPIEV  146
ident         |   |                                 |
Sbjct IFSRLLDSLRQEG--------FLTDDLVSQEE-nGQQQKYLGVCRLPG--PGRRHRRLDI  162
```

```
DSSP  EEL-LLLLllllleeeeelhhhhlllllleeeeellhHHHHhhhhhhhhhllllllllll
Query SSV-EAGNadqfdtltsdalglvgvpaavavpcxtiPWQIaqklhavtavleepkandra  205
ident   |
Sbjct IVVpYSEF---------------------------aCALL----------------yftg  179
DSSP  EELlHHHH---------------------------hHHHH----------------hhhl


DSSP  HHHHHHH-------------------------hhhhhllhHHHHhhHHHHHhhhhhhlll
Query HDLVDLQ-------------------------llegllldADLXptRSACIaifearaqh  240
ident                                           |
Sbjct SAHFNRSmralaktkgmslsehalstavvrnthgckvgpgRVLP--TPTEK-------dv  230
DSSP  LHHHHHHhhhhhhhllleellllleeelleellllleeellEEEL--LLLHH-------hh


DSSP  lLLLLLLllllhhhhhhhhhllllllllllhhhhhhhhhhhhhhhhllll
Query pWPPRVAtlphwpliyagalegldhlelartvdaaaqavqrfvaridratkr  292
ident
Sbjct fRLLGLP------------------------------------yrepaerdw  246
DSSP  hHHLLLL------------------------------------lllhhhhll
```

No 210: Query=mol1A Sbjct=5j2iA Z-score=6.7

back to top

```
DSSP  ------------------------------------------------------------
Query ------------------------------------------------------------    0
ident
Sbjct tlnggitdmltelanfeknvsqaihkynayrkaasviakyphkiksgaeakklpgvgtki   60
DSSP  lllhhhhhhhhhhhhhhhhhllllhhhhhhhhhhhhhhhhlllllllhhhhhlllllhhh


DSSP  ------------------------------------------------llllllllllhh
Query ------------------------------------------------aapyssppptnlr   12
ident
Sbjct aekideflatgklrklekirqddtsssinfltrvsgigpsaarkfvdegiktledlrkne  120
DSSP  hhhhhhhhhhlllhhhhhhhhlhhhhhhhhhllllllhhhhhhhhhlllllhhhhhhlh


DSSP  hhhhhhhhhhhhllllhhhhhHHHHHHHHHHHHHH-LLLLLLLLLLEEEEHHHHHHHhhll
Query slrdrltqvaerqgvvfgrlqRHVAXIVVAQFAA-TLTDDTGAPLLLVKGGSSLELrrgi   71
ident                                                |  |
Sbjct dklnhhqriglkyfgdfekriPREEMLQMQDIVLnEVKKVDSEYIATVCGSFRRGA----  176
DSSP  hhllhhhhhhhhlhhhhhlleEHHHHHHHHHHHHHhHHHHHLLLLEEEELHHHHLLL----


DSSP  lllLLLLLLEEEEEL------------lLHHHHHHHHHHHHhhlllleeEEELLLLEEllll
Query pdsRTSKDFATVAR------------rDIELIHEQLADAGetgwegftAIFTAPEEiaap  119
ident      | |                         |||             |
Sbjct ---ESSGDMDVLLThpsftsestkqpkLLHQVVEQLQKVH--------FITDTLSK----  221
DSSP  ---LEELLEEEEEEllllllllllllHHHHHHHHHHHHLL--------LEEEEEEE----


DSSP  lllLLLEEEEEEEEEL--LEEEEEEEEEEEEL-LLLLllllleeeeelhhhhlllllleee
Query gxpVKPRRFTAKLSYR--GRAFATVPIEVSSV-EAGNadqfdtltsdalglvgvpaavav  176
ident             |             |
Sbjct --gETKFMGVCQLPSKndEKEYPHRRIDIRLIpKDQY-----------------------  256
DSSP  --lLLEEEEEEELLLLLllLLLLLLLEEEEEEEELlHHHH-----------------------


DSSP  eellhHHHH---hhHHHHH-HLLLlllllllllhhhhhhhhhhhllhhHHHHhhHHHHh
Query pcxtiPWQI---aqKLHAV-TAVLeepkandrahdlvdlqllegllldaDLXPtrSACIa  232
ident                                                   |
Sbjct ----yCGVLyftgsDIFNKnMRAH-alekgftineytirplgvtgvagePLPV--DSEK-  308
DSSP  ----hHHHHhhhllHHHHHhHHHH-hhhlleeellllleeellllllllLLLL--LLHH-


DSSP  hhhhhlllLLLLLllllllhhhhhhhhhllllllllllhhhhhhhhhhhhhhhhllll
Query ifearaqhPWPPRvatlphwpliyagalegldhlelartvdaaaqavqrfvaridratkr  292
ident
Sbjct ----difdYIQWK--------------------------------------yrepkdrse  326
DSSP  ----hhhhHLLLL--------------------------------------lllhhhhll
```

No 211: Query=mol1A Sbjct=[illegible] Z-score=[illegible]

back to top

```
DSSP  ------------------------------------------------------------
Query ------------------------------------------------------------    0
ident 
Sbjct lnggitdmltelanfeknvsqaihkynayrkaasviakyphkiksgaeakklpgvgtkia   60
DSSP  llhhhhhhhhhhhhhhhhllllhhhhhhhhhhhhhhhhlllllllhhhhhllllllhhhh


DSSP  -----------------------------------------------lllllllllhhh
Query -----------------------------------------------aapysspptnlrs   13
ident 
Sbjct ekideflatgklrklekirqddtsssinfltrvsgigpsaarkfvdegiktledlrkned  120
DSSP  hhhhhhhhhlllhhhhhhhhlhhhhhhhhhlllllllhhhhhhhhhhllllhhhhhhlhh


DSSP  hhhhhhhhhhhllllhhhhhHHHHHHHHHHHHHH-LLLLllLLLLEEEEHHHHHHHhhlll
Query lrdrltqvaerqgvvfgrlqRHVAXIVVAQFAA-TLTDdtGAPLLLVKGGSSLELrrgip   72
ident                                               | |
Sbjct klnhhqriglkyfgdfekriPREEMLQMQDIVLnEVKKvdSEYIATVCGSFRRGA-----  175
DSSP  hllhhhhhhhhlllhhhlleEHHHHHHHHHHHHHhHHHHhlLLLEEEELHHHHLLL-----


DSSP  lllLLLLEEEEEL------------lLHHHHHHHHHHHHhhlllleeEEELLLEElllll
Query dsrTSKDFATVAR------------rDIELIHEQLADAGetgwegftAIFTAPEEiaapg  120
ident     | |                         |||             |
Sbjct --eSSGDMDVLLThpsftsestkqpkLLHQVVEQLQKVH--------FITDTLSK-----  220
DSSP  --lEELLEEEEEEllllllllllllHHHHHHHHHHHLL--------LEEEEEEE-----


DSSP  llLLLEEEEEEEEEL--LEEEEEEEEEEEEL-LLLLlllleeeeelhhhhllllllleeee
Query xpVKPRRFTAKLSYR--GRAFATVPIEVSSV-EAGNadqfdtltsdalglvgvpaavavp  177
ident            |             |
Sbjct -gETKFMGVCQLPSKndEKEYPHRRIDIRLIpKDQY------------------------  255
DSSP  -lLLEEEEEELLLLLllLLLLLLEEEEEEELlHHHH------------------------


DSSP  ellhHHHHhhhhhhhhlllllllllllHHHH-----------------hhhhhhhhllh
Query cxtiPWQIaqklhavtavleepkandraHDLV-----------------dlqlleglllld  220
ident                              |
Sbjct ---yCGVL----------------yftgSDIFnknmrahalekgftineytirpltgvag  296
DSSP  ---hHHHH----------------hhhlLHHHhhhhhhhhhhhhleeellllleeellllll


DSSP  HHHHhhHHHHhhhhhhlllLLLLLLlllllhhhhhhhhlllllllllllhhhhhhhhh
Query ADLXPtrSACIaifearaqhPWPPRVatlphwpliyagalegldhlelartvdaaaqavq  280
ident   |
Sbjct EPLPV--DSEK-----difdYIQWKY----------------------------------  315
DSSP  LLLLL--LLHH-----hhhhHLLLLL----------------------------------


DSSP  hhhhhhhhllll
Query rfvaridratkr  292
ident 
Sbjct ----repkdrse  323
DSSP  ----llhhhlll
```

No 212: Query=mol1A Sbjct=4rq5A Z-score=6.7

back to top

```
DSSP  ------------------------------------------------------------
Query ------------------------------------------------------------    0
ident 
Sbjct tlnggitdmltelanfeknvsqaihkynayrkaasviakyphkiksgaeakklpgvgtki   60
DSSP  lllhhhhhhhhhhhhhhhhllllhhhhhhhhhhhhhhhhlllllllhhhhhhllllllhhh


DSSP  ------------------------------------------------llllllllllhh
Query ------------------------------------------------aapyssspptnlr   12
ident 
Sbjct aekideflatgklrklekirqddtsssinfltrvsgigpsaarkfvdegiktledlrkne  120
DSSP  hhhhhhhhhhlllhhhhhhhhllhhhhhhhhhhllllllhhhhhhhhhllllllhhhhhhlh
```

```
DSSP  hhhhhhhhhhhhllllhhhhhHHHHHHHHHHHHH-LLLLllLLLLEEEEHHHHHHHHhhll
Query slrdrltqvaerqgvvfgrlqRHVAXIVVAQFAA-TLTDdtGAPLLLVKGGSSLELrrgi   71
ident                                                | |
Sbjct dklnhhqriglkyfgdfekriPREEMLQMQDIVLnEVKKvdSEYIATVCGSFRRGA----  176
DSSP  hhllhhhhhhhhhlhhhhhlleEHHHHHHHHHHHHHhHHHHhlLLLEEEELHHHHLLL----


DSSP  llllLLLLEEEEEL------------lLHHHHHHHHHHHHhhlllleeEEELLLEEllll
Query pdsrTSKDFATVAR------------rDIELIHEQLADAGetgwegftAIFTAPEEiaap  119
ident      | |                         |||             |
Sbjct ---eSSGDMDVLLThpsftsestkqpkLLHQVVEQLQKVH--------FITDTLSK----  221
DSSP  ---lEELLEEEEEElllllllllllllHHHHHHHHHHHLL--------LEEEEEEE----


DSSP  lllLLLEEEEEEEEEL--LEEEEEEEEEEEEL-LLLLlllleeeeelhhhhlllllleee
Query gxpVKPRRFTAKLSYR--GRAFATVPIEVSSV-EAGNadqfdtltsdalglvgvpaavav  176
ident             |             |
Sbjct --gETKFMGVCQLPSKndEKEYPHRRIDIRLIpKDQY-----------------------  256
DSSP  --lLLEEEEEEELLLLllLLLLLLEEEEEEELlHHHH-----------------------


DSSP  eellhHHHHhhhhhhhhllllllllllllHHHH-------------------hhhhhhhh
Query pcxtiPWQIaqklhavtavleepkandraHDLV-------------------dlqllegl  217
ident                               |
Sbjct ----yCGVL----------------yftgSDIFnknmrahalekgftineytirplgvtg  296
DSSP  ----hHHHH----------------hhhlLHHHhhhhhhhhhhlleeelllleeelllll


DSSP  llhHHHHHhhHHHHhhhhhhlllLLLLLlllllhhhhhhhhhllllllllllllhhhhhh
Query lldADLXPtrSACIaifearaqhPWPPRvatlphwpliyagalegldhlelartvdaaaq  277
ident      |
Sbjct vagEPLPV--DSEK-----difdYIQWK--------------------------------  317
DSSP  lllLLLLL--LLHH-----hhhhHLLLL--------------------------------


DSSP  hhhhhhhhhhhllll
Query avqrfvaridratkr  292
ident
Sbjct ------yrepkdrse  326
DSSP  ------lllhhhhll
```

No 213: Query=mol1A Sbjct=4rpyA Z-score=6.7

back to top

```
DSSP  ------------------------------------------------------------
Query ------------------------------------------------------------    0
ident
Sbjct tlnggitdmltelanfeknvsqaihkynayrkaasviakyphkiksgaeakklpgvgtki   60
DSSP  lllhhhhhhhhhhhhhhhhhllllhhhhhhhhhhhhhhhlllllllhhhhhllllllhhh


DSSP  ------------------------------------------------llllllllllhh
Query ------------------------------------------------aapyssspptnlr   12
ident
Sbjct aekideflatgklrklekirqddtsssinfltrvsgigpsaarkfvdegiktledlrkne  120
DSSP  hhhhhhhhhhlllhhhhhhhhlhhhhhhhhhlllllllhhhhhhhhhllllllhhhhhhlh


DSSP  hhhhhhhhhhhhllllhhhhhHHHHHHHHHHHHH-LLLLllLLLLEEEEHHHHHHHHhhll
Query slrdrltqvaerqgvvfgrlqRHVAXIVVAQFAA-TLTDdtGAPLLLVKGGSSLELrrgi   71
ident                                                | |
Sbjct dklnhhqriglkyfgdfekriPREEMLQMQDIVLnEVKKvdSEYIATVCGSFRRGA----  176
DSSP  hhllhhhhhhhhhlhhhhhlleEHHHHHHHHHHHHHhHHHHhlLLLEEEELHHHHLLL----


DSSP  lllLLLLLEEEEEL------------lLHHHHHHHHHHHHhhlllleeEEELLLEEllll
Query pdsRTSKDFATVAR------------rDIELIHEQLADAGetgwegftAIFTAPEEiaap  119
ident      | |                         |||             |
Sbjct ---ESSGDMDVLLThpsftsestkqpkLLHQVVEQLQKVH--------FITDTLSK----  221
DSSP  ---LEELLEEEEEElllllllllllllHHHHHHHHHHHLL--------LEEEEEEE----


DSSP  lllLLLEEEEEEEEEL--lEEEEEEEEEEEEL-LLLLlllleeeeelhhhhlllllleee
Query gxpVKPRRFTAKLSYR--gRAFATVPIEVSSV-EAGNadqfdtltsdalglvgvpaavav  176
ident             |             |
```

```
DSSP  --lLLEEEEEEELLLLLlllLLLLLEEEEEEEElHHHH-----------------------


DSSP  eellhHHHHhhhhhhhhhlllllllllllHHHH-------------------hhhhhhhh
Query pcxtiPWQIaqklhavtavleepkandraHDLV-------------------dlqllegl  217
ident                               |
Sbjct ----yCGVL----------------yftgSDIFnknmrahalekgftineytirplgvtg  296
DSSP  ----hHHHH----------------hhhlLHHHhhhhhhhhhhlleeellllleeellll


DSSP  llhHHHHHhhHHHHhhhhhhlllLLLLLLlllllhhhhhhhhhlllllllllllhhhhhh
Query lldADLXPtrSACIaifearaqhPWPPRVatlphwpliyagalegldhlelartvdaaaq  277
ident      |
Sbjct vagEPLPV--DSEK-----difdYIQWKY-------------------------------  318
DSSP  lllLLLLL--LLHH-----hhhhHLLLLL-------------------------------


DSSP  hhhhhhhhhhhllll
Query avqrfvaridratkr  292
ident
Sbjct -------repkdrse  326
DSSP  -------llhhhhll
```

No 214: Query=mol1A Sbjct=4rq0A Z-score=6.7

back to top

```
DSSP  ------------------------------------------------------------
Query ------------------------------------------------------------    0
ident
Sbjct etlnggitdmltelanfeknvsqaihkynayrkaasviakyphkiksgaeakklpgvgtk   60
DSSP  llllhhhhhhhhhhhhhhhhhllllhhhhhhhhhhhhhhhhhllllllllhhhhhllllllhh


DSSP  -------------------------------------------------llllllllllh
Query -------------------------------------------------aapyssspptnl   11
ident
Sbjct iaekideflatgklrklekirqddtsssinfltrvsgigpsaarkfvdegiktledlrkn  120
DSSP  hhhhhhhhhhhlllhhhhhhhhlhhhhhhhhhlllllllhhhhhhhhhlllllhhhhhhl


DSSP  hhhhhhhhhhhhllllhhhhhHHHHHHHHHHHHH-LLLLllLLLLEEEEHHHHHHHHhhl
Query rslrdrltqvaerqgvvfgrlqRHVAXIVVAQFAA-TLTDdtGAPLLLVKGGSSLELrrg   70
ident                                                 | |
Sbjct edklnhhqriglkyfgdfekriPREEMLQMQDIVLnEVKKvdSEYIATVCGSFRRGA---  177
DSSP  hhhllhhhhhhhhlhhhhhlleEHHHHHHHHHHHHHhHHHHhlLLLEEEEELHHHHLLL---


DSSP  llllLLLLLEEEEEL------------lLHHHHHHHHHHHHhhllllleeEEELLLEElll
Query ipdsRTSKDFATVAR------------rDIELIHEQLADAGetgwegftAIFTAPEEiaa  118
ident       | |                         |||             |
Sbjct ----ESSGDMDVLLThpsftsestkqpkLLHQVVEQLQKVH--------FITDTLSK---  222
DSSP  ----LEELLEEEEEEllllllllllllHHHHHHHHHHHHLL--------LEEEEEEE---


DSSP  llllLLLEEEEEEEEEEL--lEEEEEEEEEEEEL-LLLLlllleeeeelhhhhllllllee
Query pgxpVKPRRFTAKLSYR--gRAFATVPIEVSSV-EAGNadqfdtltsdalglvgvpaava  175
ident              |             |
Sbjct ---gETKFMGVCQLPSKndeKEYPHRRIDIRLIpKDQY----------------------  257
DSSP  ---lLLEEEEEEELLLLLlllLLLLLEEEEEEEElHHHH----------------------


DSSP  eeellhHHHHhhhhhhhhhlllllllllllHHHH-------------------hhhhhhh
Query vpcxtiPWQIaqklhavtavleepkandraHDLV-------------------dlqlleg  216
ident                                |
Sbjct -----yCGVL----------------yftgSDIFnknmrahalekgftineytirplgvt  296
DSSP  -----hHHHH----------------hhhlLHHHhhhhhhhhhhlleeellllleeellll


DSSP  hllhHHHHHhhHHHHhhhhhhlllLLLLLLlllllhhhhhhhhhlllllllllllhhhhh
Query llldADLXPtrSACIaifearaqhPWPPRVatlphwpliyagalegldhlelartvdaaa  276
ident       |
Sbjct gvagEPLPV--DSEK-----difdYIQWKY------------------------------  319
DSSP  llllLLLLL--LLHH-----hhhhHLLLLL------------------------------
```

```
Query qavqrfvaridratkr  292
ident
Sbjct --------repkdrse  327
DSSP  --------llhhhlll
```

No 215: Query=mol1A Sbjct=3k75D Z-score=6.7

back to top

```
DSSP  ---------------------------llllllllllhhhhhhhhhhhhhhllllhhhhh
Query ---------------------------aapysspptnlrslrdrltqvaerqgvvfgrlq   33
ident
Sbjct ddtsssinfltrvtgigpsaarklvdegiktledlrknedklnhhqriglkyfedfekri   60
DSSP  llhhhhhhhhllllllhhhhhhhhhlllllhhhhlllhhhllhhhhhhhhhlhhhhhlle


DSSP  HHHHHHHHHHHHH-LLLLllLLLLEEEEHHHHHHHhhllllllLLLLEEEEEL-------
Query RHVAXIVVAQFAA-TLTDdtGAPLLLVKGGSSLELrrgipdsrTSKDFATVAR-------   85
ident                           | |               | |
Sbjct PREEMLQMQDIVLnEVKKldPEYIATVCGSFRRGA-------eSSGDMDVLLThpnftse  113
DSSP  EHHHHHHHHHHHHhHHHHhlLLLEEEELHHHHLLL-------lEELLEEEEEElllllll


DSSP  -----lLHHHHHHHHHHHHhhlllleeEEELLLEELllllllLLLEEEEEEEEEL--LEE
Query -----rDIELIHEQLADAGetgwegftAIFTAPEEIaapgxpVKPRRFTAKLSYR--GRA  138
ident             |||             |                      |
Sbjct sskqpkLLHRVVEQLQKVR--------FITDTLSKG------ETKFMGVCQLPSEndENE  159
DSSP  llllhhHHHHHHHHHHHLL--------LEEEEEEEL------LLEEEEEEELLLLllLLL


DSSP  EEEEEEEEEELLLLLLllleeeeelhhhhlllllleeeeellhHHHHhhhhhhhhlllll
Query FATVPIEVSSVEAGNAdqfdtltsdalglvgvpaavavpcxtiPWQIaqklhavtavlee  198
ident      |
Sbjct YPHRRIDIRLIPKDQY--------------------------yCGVL-------------  180
DSSP  LLLEEEEEEELLHHHH--------------------------hHHHH-------------


DSSP  llllllhhhhhHHHHHH----------------------hhllhhhhhhhhHHHHhhhhh
Query pkandrahdlvDLQLLE----------------------gllldadlxptrSACIaifea  236
ident                                                    |
Sbjct -------yftgSDIFNKnmrahalekgftineytirplgvtgvageplpvdSEQD-----  228
DSSP  -------hhhlLHHHHHhhhhhhhhlleeellleeelllllllllllllllLHHH-----


DSSP  hllllLLLLLlllllhhhhhhhhhlllllLLLLllhhhhhhhhhhhhhhhhhllll
Query raqhpWPPRVatlphwpliyagalegldhLELArtvdaaaqavqrfvaridratkr  292
ident
Sbjct --ifdYIQWR-----------yrepkdrsEHHH-----------------------  248
DSSP  --hhhHHLLL-----------lllhhhllLLLL-----------------------
```

No 216: Query=mol1A Sbjct=3jptA Z-score=6.7

back to top

```
DSSP  ------------------------------------------------------------
Query ------------------------------------------------------------    0
ident
Sbjct tlnggitdmltelanfeknvsqaihkynayrkaasviakyphkiksgaeakklpgvgtki   60
DSSP  lllhhhhhhhhhhhhhhhhllllhhhhhhhhhhhhhhhhlllllllhhhhhllllllhhh


DSSP  ------------------------------------------------llllllllllhh
Query ------------------------------------------------aapysspptnlr   12
ident
Sbjct aekideflatgklrklekirqddtsssinfltrvsgigpsaarkfvdegiktledlrkne  120
DSSP  hhhhhhhhhhlllhhhhhhhhlhhhhhhhhhllllllhhhhhhhhhhlllllhhhhhhlh


DSSP  hhhhhhhhhhhhllllhhhhhHHHHHHHHHHHHH-LLLLllLLLLEEEEHHHHHHHhhll
Query slrdrltqvaerqgvvfgrlqRHVAXIVVAQFAA-TLTDdtGAPLLLVKGGSSLELrrgi   71
ident                                                | |
Sbjct dklnhhqriglkyfgdfekriPREEMLQMQDIVLnEVKKvdSEYIATVCGSFRRGA----  176
DSSP  hhllhhhhhhhhlllhhhlleEHHHHHHHHHHHHhHHHHhlLLLEEEELHHHHLLL----
```

```
DSSP  llllLLLLEEEEEL------------lLHHHHHHHHHHHHhhlllleeEEELLLEELlll
Query pdsrTSKDFATVAR------------rDIELIHEQLADAGetgwegftAIFTAPEEIaap  119
ident      | |                         |||             |
Sbjct ---eSSGDMDVLLThpsftsestkqpkLLHQVVEQLQKVH--------FITDTLSKG---  222
DSSP  ---lEELLEEEEELllllllllllllHHHHHHHHHHHLL--------LEEEEEEEL---


DSSP  lllLLLEEEEEEEEEL--LEEEEEEEEEEEEL-LLLLlllleeeeelhhhhlllllleee
Query gxpVKPRRFTAKLSYR--GRAFATVPIEVSSV-EAGNadqfdtltsdalglvgvpaavav  176
ident             |             |
Sbjct ---ETKFMGVCQLPSKndEKEYPHRRIDIRLIpKDQY-----------------------  256
DSSP  ---LLEEEEEEELLLLllLLLLLLEEEEEEELlHHHH-----------------------


DSSP  eellhHHHHhhhhhhhhllllllllllllHHHHHHH-------------------hhhhh
Query pcxtiPWQIaqklhavtavleepkandraHDLVDLQ-------------------llegl  217
ident                               |
Sbjct ----yCGVL----------------yftgSDIFNKNmrahalekgftineytirplgvtg  296
DSSP  ----hHHHH----------------hhhlLHHHHHHhhhhhhhlleeellllleeelllll


DSSP  llhHHHHhhHHHHHhhhhhhllllLLLLLlllllhhhhhhhhhllllllllllllhhhhhh
Query lldADLXptRSACIaifearaqhpWPPRVatlphwpliyagalegldhlelartvdaaaq  277
ident      |
Sbjct vagEPLP--VDSEK-------difDYIQW-------------------------------  316
DSSP  lllLLLL--LLLHH-------hhhHHLLL-------------------------------


DSSP  hhhhhhhhhhhllll
Query avqrfvaridratkr  292
ident
Sbjct -----kyrepkdrse  326
DSSP  -----llllhhhlll
```

No 217: Query=mol1A Sbjct=3isdA Z-score=6.7

back to top

```
DSSP  ------------------------------------------------------------
Query ------------------------------------------------------------    0
ident
Sbjct tlnggitdmltelanfeknvsqaihkynayrkaasviakyphkiksgaeakklpgvgtki   60
DSSP  lllhhhhhhhhhhhhhhhhllllhhhhhhhhhhhhhhhllllllllhhhhhllllllhhh


DSSP  ------------------------------------------------llllllllllhh
Query ------------------------------------------------aapyssspptnlr   12
ident
Sbjct aekideflatgklrklekirqddtsssinfltrvsgigpsaarkfvdegiktledlrkne  120
DSSP  hhhhhhhhhhlllhhhhhhhhlhhhhhhhhhllllllhhhhhhhhhhlllllhhhhhhlh


DSSP  hhhhhhhhhhhhllllhhhhhHHHHHHHHHHHHHH-LLLLllLLLLEEEEHHHHHHHhhll
Query slrdrltqvaerqgvvfgrlqRHVAXIVVAQFAA-TLTDdtGAPLLLVKGGSSLELrrgi   71
ident                                                |  |
Sbjct dklnhhqriglkyfgdfekriPREEMLQMQDIVLnEVKKvdSEYIATVCGSFRRGA----  176
DSSP  hhllhhhhhhhhlhhhhllleEHHHHHHHHHHHHhHHHHhlLLLLLEELHHHHLLL----


DSSP  llllLLLLEEEEEL------------lLHHHHHHHHHHHHhhlllleeEEELLLEELlll
Query pdsrTSKDFATVAR------------rDIELIHEQLADAGetgwegftAIFTAPEEIaap  119
ident      | |                         |||             |
Sbjct ---eSSGDMDVLLThpsftsestkqpkLLHQVVEQLQKVH--------FITDTLSKG---  222
DSSP  ---lEELLEEEEELllllllllllllHHHHHHHHHHHLL--------LEEEEEEEL---


DSSP  lllLLLEEEEEEEEELL--EEEEEEEEEEEEL-LLLLlllleeeeelhhhhlllllleee
Query gxpVKPRRFTAKLSYRG--RAFATVPIEVSSV-EAGNadqfdtltsdalglvgvpaavav  176
ident             |             |
Sbjct ---ETKFMGVCQLPSKNdeKEYPHRRIDIRLIpKDQY-----------------------  256
DSSP  ---LLEEEEEEELLLLLllLLLLLEEEEEEELlHHHH-----------------------


DSSP  eellhHHHHhhhhhhhhllllllllllllHHHHHHH-------------------hhhhh
Query pcxtiPWQIaqklhavtavleepkandraHDLVDLQ-------------------llegl  217
```

```
Sbjct ----yCGVL----------------yftgSDIFNKNmrahalekgftineytirplgvtg  296
DSSP  ----hHHHH----------------hhhlLHHHHHHhhhhhhhlleeellllleeelllll
```

```
DSSP  llhHHHHhhHHHHHhhhhhhllllLLLLLllllllhhhhhhhhhllllllllllhhhhhh
Query lldADLXptRSACIaifearaqhpWPPRVatlphwpliyagalegldhlelartvdaaaq  277
ident      |
Sbjct vagEPLP--VDSEK-------difDYIQW-------------------------------  316
DSSP  lllLLLL--LLLHH-------hhhHHLLL-------------------------------
```

```
DSSP  hhhhhhhhhhhllll
Query avqrfvaridratkr  292
ident
Sbjct -----kyrepkdrse  326
DSSP  -----llllhhhlll
```

No 218: Query=mol1A Sbjct=4doaA Z-score=6.7

back to top

```
DSSP  ------------------------------------------------------------
Query ------------------------------------------------------------     0
ident
Sbjct tlnggitdmltelanfeknvsqaihkynayrkaasviakyphkiksgaeakklpgvgtki    60
DSSP  lllhhhhhhhhhhhhhhhhllllhhhhhhhhhhhhhhhhlllllllhhhhhlllllhhh
```

```
DSSP  ------------------------------------------------llllllllllhh
Query ------------------------------------------------aapysspptnlr    12
ident
Sbjct aekideflatgklrklekirqddtsssinfltrvsgigpsaarkfvdegiktledlrkne   120
DSSP  hhhhhhhhhhlllhhhhhhhhlhhhhhhhhhlllllllhhhhhhhhhlllllhhhhhhlh
```

```
DSSP  hhhhhhhhhhhhllllhhhhhHHHHHHHHHHHHH-LLLLllLLLLEEEEHHHHHHHhhll
Query slrdrltqvaerqgvvfgrlqRHVAXIVVAQFAA-TLTDdtGAPLLLVKGGSSLELrrgi    71
ident                                                |  |
Sbjct dklnhhqriglkyfgdfekriPREEMLQMQDIVLnEVKKvdSEYIATVCGSFRRGA----   176
DSSP  hhllhhhhhhhhlhhhhlllleEHHHHHHHHHHHHHhHHHHhlLLLEEEEELHHHHLLL----
```

```
DSSP  llllLLLLEEEEEL------------lLHHHHHHHHHHHHHhhllllleeEEELLLEELlll
Query pdsrTSKDFATVAR------------rDIELIHEQLADAGetgwegftAIFTAPEEIaap   119
ident      | |                         |||             |
Sbjct ---eSSGDMDVLLThpsftsestkqpkLLHQVVEQLQKVH--------FITDTLSKG---   222
DSSP  ---lEELLEEEEEEllllllllllllHHHHHHHHHHHHLL--------LEEEEEEEL---
```

```
DSSP  lllLLLEEEEEEEEEL--LEEEEEEEEEEEEL-LLLLllllleeeeelhhhhlllllleee
Query gxpVKPRRFTAKLSYR--GRAFATVPIEVSSV-EAGNadqfdtltsdalglvgvpaavav   176
ident             |             |
Sbjct ---ETKFMGVCQLPSKndEKEYPHRRIDIRLIpKDQY-----------------------   256
DSSP  ---LLEEEEEEELLLLLllLLLLLLLEEEEEEEELlHHHH-----------------------
```

```
DSSP  eellhHHHHhhhhhhhhllllllllllllHHHHHHH------------------hhhhh
Query pcxtiPWQIaqklhavtavleepkandraHDLVDLQ------------------llegl   217
ident                               |
Sbjct ----yCGVL----------------yftgSDIFNKNmrahalekgftineytirplgvtg   296
DSSP  ----hHHHH----------------hhhlLHHHHHHhhhhhhhlleeellllleeelllll
```

```
DSSP  llhHHHHhhHHHHHhhhhhhllllLLLLLllllllhhhhhhhhhllllllllllhhhhhh
Query lldADLXptRSACIaifearaqhpWPPRVatlphwpliyagalegldhlelartvdaaaq   277
ident      |
Sbjct vagEPLP--VDSEK-------difDYIQW-------------------------------   316
DSSP  lllLLLL--LLLHH-------hhhHHLLL-------------------------------
```

```
DSSP  hhhhhhhhhhhllll
Query avqrfvaridratkr  292
ident
Sbjct -----kyrepkdrse  326
DSSP  -----llllhhhlll
```

No 219: Query=mol1A Sbjct=3rh6A Z-score=6.7

back to top

```
DSSP  ------------------------------------------------------------
Query ------------------------------------------------------------    0
ident 
Sbjct tlnggitdmltelanfeknvsqaihkynayrkaasviakyphkiksgaeakklpgvgtki   60
DSSP  lllhhhhhhhhhhhhhhhhhllllhhhhhhhhhhhhhhhhlllllllhhhhllllllhhh


DSSP  ------------------------------------------------llllllllllhh
Query ------------------------------------------------aapysspptnlr   12
ident 
Sbjct aekideflatgklrklekirqddtsssinfltrvsgigpsaarkfvdegiktledlrkne  120
DSSP  hhhhhhhhhlllhhhhhhhhhlhhhhhhhhhllllllhhhhhhhhhhllllhhhhhhllh


DSSP  hhhhhhhhhhhhllllhhhhhHHHHHHHHHHHHH-LLLLllLLLLEEEEHHHHHHHhhll
Query slrdrltqvaerqgvvfgrlqRHVAXIVVAQFAA-TLTDdtGAPLLLVKGGSSLELrrgi   71
ident                                                | |          
Sbjct dklnhhqriglkyfgdfekriPREEMLQMQDIVLnEVKKvdSEYIATVCGSFRRGA----  176
DSSP  hhllhhhhhhhhlhhhhhlleEHHHHHHHHHHHHhHHHHhlLLLEEEELHHHHLLL----


DSSP  llllLLLLEEEEEL------------lLHHHHHHHHHHHHhhlllleeEEELLLEELlll
Query pdsrTSKDFATVAR------------rDIELIHEQLADAGetgwegftAIFTAPEEIaap  119
ident      | |                         |||             |          
Sbjct ---eSSGDMDVLLThpsftsestkqpkLLHQVVEQLQKVH--------FITDTLSKG---  222
DSSP  ---lEELLEEEEEEllllllllllllHHHHHHHHHHHLL--------LEEEEEEEL---


DSSP  lllLLLEEEEEEEEEL--LEEEEEEEEEEEEL-LLLLlllleeeeelhhhhlllllleee
Query gxpVKPRRFTAKLSYR--GRAFATVPIEVSSV-EAGNadqfdtltsdalglvgvpaavav  176
ident             |             |                                 
Sbjct ---ETKFMGVCQLPSKndEKEYPHRRIDIRLIpKDQY-----------------------  256
DSSP  ---LLEEEEEEELLLLllLLLLLLEEEEEEELlHHHH-----------------------


DSSP  eellhHHHHhhhhhhhhllllllllllllHHHHHHH-------------------hhhhh
Query pcxtiPWQIaqklhavtavleepkandraHDLVDLQ-------------------llegl  217
ident                               |                             
Sbjct ----yCGVL----------------aftgSDIFNKNmrahalekgftineytirplgvtg  296
DSSP  ----hHHHH----------------hhhlLHHHHHHhhhhhhhhleeellllleeelllll


DSSP  llhHHHHhhHHHHHhhhhhhllllLLLLLlllllhhhhhhhhhllllllllllllhhhhhh
Query lldADLXptRSACIaifearaqhpWPPRVatlphwpliyagalegldhlelartvdaaaq  277
ident      |                                                      
Sbjct vagEPLP--VDSEK-------difDYIQW-------------------------------  316
DSSP  lllLLLL--LLLHH-------hhhHHLLL-------------------------------


DSSP  hhhhhhhhhhhllll
Query avqrfvaridratkr  292
ident 
Sbjct -----kyrepkdrse  326
DSSP  -----llllhhhhll
```

No 220: Query=mol1A Sbjct=3jpsA Z-score=6.7

back to top

```
DSSP  ------------------------------------------------------------
Query ------------------------------------------------------------    0
ident 
Sbjct tlnggitdmltelanfeknvsqaihkynayrkaasviakyphkiksgaeakklpgvgtki   60
DSSP  lllhhhhhhhhhhhhhhhhhllllhhhhhhhhhhhhhhhhlllllllhhhhhlllllhhh


DSSP  ------------------------------------------------llllllllllhh
Query ------------------------------------------------aapysspptnlr   12
ident 
Sbjct aekideflatgklrklekirqddtsssinfltrvsgigpsaarkfvdegiktledlrkne  120
```

```
DSSP  hhhhhhhhhhhhllllhhhhhHHHHHHHHHHHHHH-LLLLllLLLLEEEEHHHHHHHHhhll
Query slrdrltqvaerqgvvfgrlqRHVAXIVVAQFAA-TLTDdtGAPLLLVKGGSSLELrrgi   71
ident                                                |  |
Sbjct dklnhhqriglkyfgdfekriPREEMLQMQDIVLnEVKKvdSEYIATVCGSFRRGA----  176
DSSP  hhllhhhhhhhhlllhhhlleEHHHHHHHHHHHHHhHHHHhlLLLEEEELHHHHLLL----


DSSP  llllLLLLEEEEEL------------lLHHHHHHHHHHHHHhhlllleeEEELLLEELlll
Query pdsrTSKDFATVAR------------rDIELIHEQLADAGetgwegftAIFTAPEEIaap  119
ident      | |                         |||             |
Sbjct ---eSSGDMDVLLThpsftsestkqpkLLHQVVEQLQKVH--------FITDTLSKG---  222
DSSP  ---lEELLEEEEEElllllllllllllHHHHHHHHHHHLL--------LEEEEEEEL---


DSSP  lllLLLEEEEEEEEEL--LEEEEEEEEEEEL-LLLLlllleeeeelhhhhllllllleee
Query gxpVKPRRFTAKLSYR--GRAFATVPIEVSSV-EAGNadqfdtltsdalglvgvpaavav  176
ident             |             |
Sbjct ---ETKFMGVCQLPSKndEKEYPHRRIDIRLIpKDQY-----------------------  256
DSSP  ---LLEEEEEEELLLLLllLLLLLLEEEEEEEELlHHHH-----------------------


DSSP  eellhHHHHhhhhhhhhlllllllllllHHHHHHH-------------------hhhhh
Query pcxtiPWQIaqklhavtavleepkandraHDLVDLQ-------------------llegl  217
ident                               |
Sbjct ----yCGVL----------------yftgSDIFNKNmrahalekgftineytirplgvtg  296
DSSP  ----hHHHH----------------hhhlLHHHHHHhhhhhhhlleeellllleeellll


DSSP  llhHHHHhhHHHHHhhhhhhllllLLLLLlllllhhhhhhhhhlllllllllllhhhhhh
Query lldADLXptRSACIaifearaqhpWPPRVatlphwpliyagalegldhlelartvdaaaq  277
ident      |
Sbjct vagEPLP--VDSEK-------difDYIQW-------------------------------  316
DSSP  lllLLLL--LLLHH-------hhhHHLLL-------------------------------


DSSP  hhhhhhhhhhhllll
Query avqrfvaridratkr  292
ident 
Sbjct -----kyrepkdrse  326
DSSP  -----llllhhhlll
```

No 221: Query=mol1A Sbjct=3uxnB Z-score=6.7

back to top

```
DSSP  ------------------------------------------------------------
Query ------------------------------------------------------------    0
ident 
Sbjct tlnggitdmlvelanfeknvsqaihkynayrkaasviakyphkiksgaeakklpgvgtki   60
DSSP  lllhhhhhhhhhhhhhhhhhllllhhhhhhhhhhhhhhhlllllllllllllllllhhhh


DSSP  ------------------------------------------------llllllllllhh
Query ------------------------------------------------aapysspptnlr   12
ident 
Sbjct aekideflatgklrklekirqddtsssinfltrvtgigpsaarklvdegiktledlrkne  120
DSSP  hhhhhhhhhlllllllllllllhhhhllllllllllhhhhhhhhhlllllllllll


DSSP  hhhhhhhhhhhhllllhhhhhHHHHHHHHHHHHHHH-LLLLllLLLLEEEEHHHHHHHHhhll
Query slrdrltqvaerqgvvfgrlqRHVAXIVVAQFAA-TLTDdtGAPLLLVKGGSSLELrrgi   71
ident                                                |  |
Sbjct dklnhhqriglkyfedfekriPREEMLQMQDIVLnEVKKldPEYIATVCGSFRRGA----  176
DSSP  lllllllhhhhhlhhhhllleEHHHHHHHHHHHHHhHHHHhlLLLEEEELHHHLLLL----


DSSP  llllLLLLEEEEEL------------lLHHHHHHHHHHHHHhhlllleeEEELLLEELlll
Query pdsrTSKDFATVAR------------rDIELIHEQLADAGetgwegftAIFTAPEEIaap  119
ident      | |                         |||             |
Sbjct ---eSSGDMDVLLThpnftsesskqpkLLHRVVEQLQKVR--------FITDTLSKG---  222
DSSP  ---lEELLEEEEEEllllllllllllhHHHHHHHHHHLLLL--------LEEEEEEEL---


DSSP  lllLLLEEEEEEEEEELLE--EEEEEEEEEEEL-LLLLlllleeeeelhhhhllllllleee
```

```
ident             |             |
Sbjct ---ETKFMGVCQLPSENDenEYPHRRIDIRLIpKDQY-----------------------  256
DSSP  ---LLEEEEEELLLLLLLlLLLLEEEEEEELlHHHH-----------------------


DSSP  eellhHHHHhhhhhhhhlllllllllllHHHHHHH-------------------hhhhh
Query pcxtiPWQIaqklhavtavleepkandraHDLVDLQ-------------------llegl  217
ident                               |
Sbjct ----yCGVL----------------yftgSDIFNKNmrahalekgftineytirplgvtg  296
DSSP  ----hHHHH----------------hhhlLHHHHHHhhhhhhlleeellllleeellll


DSSP  llhHHHHhhHHHHHhhhhhhllllLLLLLlllllhhhhhhhhhllllllllllhhhhhh
Query lldADLXptRSACIaifearaqhpWPPRVatlphwpliyagalegldhlelartvdaaaq  277
ident      |
Sbjct vagEPLP--VDSEQ-------difDYIQW-------------------------------  316
DSSP  lllLLLL--LLLHH-------hhhHHLLL-------------------------------


DSSP  hhhhhhhhhhllll
Query avqrfvaridratkr  292
ident
Sbjct ------ryrepkdrs  325
DSSP  ------llllhhhll
```

No 222: Query=mol1A Sbjct=9icnA Z-score=6.7

back to top

```
DSSP  ------------------------------------------------------------
Query ------------------------------------------------------------    0
ident
Sbjct etlnggitdmltelanfeknvsqaihkynayrkaasviakyphkiksgaeakklpgvgtk   60
DSSP  llllhhhhhhhhhhhhhhhhllllhhhhhhhhhhhhhhhhllllllhhhhhlllllllhh


DSSP  ------------------------------------------------lllllllllllh
Query ------------------------------------------------aapyssppptnl   11
ident
Sbjct iaekideflatgklrklekirqddtsssinfltrvsgigpsaarkfvdegiktledlrkn  120
DSSP  hhhhhhhhhllllhhhhhhlllhhhhhhhhhlllllllhhhhhhhhllllllhhhhhhl


DSSP  hhhhhhhhhhhhhllllhhhhhHHHHHHHHHHHHH-LLLLllLLLLEEEEHHHHHHHhhl
Query rslrdrltqvaerqgvvfgrlqRHVAXIVVAQFAA-TLTDdtGAPLLLVKGGSSLELrrg   70
ident                                                 | |
Sbjct edklnhhqriglkyfgdfekriPREEMLQMQDIVLnEVKKvdSEYIATVCGSFRRGA---  177
DSSP  llllllhhhhhhhlhhhhhlleEHHHHHHHHHHHHHhHHHHhlLLLEEEELLLLLLLL---


DSSP  lllllLLLLEEEEEL------------lLHHHHHHHHHHHHhhlllleeEEELLLEELll
Query ipdsrTSKDFATVAR------------rDIELIHEQLADAGetgwegftAIFTAPEEIaa  118
ident       | |                         |||              |
Sbjct ----eSSGDMDVLLThpsftsestkqpkLLHQVVEQLQKVH--------FITDTLSKG--  223
DSSP  ----lEELLEEEEEEellllllllllhHHHHHHHHHHHHLL--------LEEEEEEEL--


DSSP  llllLLLEEEEEEEEEEELL--EEEEEEEEEEEEL-LLLLlllleeeeelhhhhllllllee
Query pgxpVKPRRFTAKLSYRG--RAFATVPIEVSSV-EAGNadqfdtltsdalglvgvpaava  175
ident              |             |
Sbjct ----ETKFMGVCQLPSKNdeKEYPHRRIDIRLIpKDQY----------------------  257
DSSP  ----LLEEEEEELLLLLLlLLLLLLEEEEEEEEEeHHHH----------------------


DSSP  eeellhHHHHhhhhhhhhlllllllllllhhhhHHHHHH--------------------
Query vpcxtiPWQIaqklhavtavleepkandrahdlvDLQLLE--------------------  215
ident
Sbjct -----yCGVL--------------------yftgSDIFNKnmrahalekgftineytirp  292
DSSP  -----hHHHH--------------------hhhlLHHHHHhhhhhhhhllllleelleell


DSSP  -hhllhhhhhhhHHHHHHhhhhhllllLLLLLlllllhhhhhhhhhllllllllllhhh
Query -glllldadlxptRSACIAifearaqhpWPPRVatlphwpliyagalegldhlelartvda  274
ident
Sbjct lgvtgvageplpVDSEKD------ifdYIQWK----------------------------  318
DSSP  lllllllllllLLLHHH------hhhHLLLL----------------------------
```

```
DSSP  hhhhhhhhhhhhhhhllll
Query aaqavqrfvaridratkr  292
ident
Sbjct ---------yrepkdrse  327
DSSP  ---------lllhhhhll
```

No 223: Query=mol1A Sbjct=2fmsA Z-score=6.7

back to top

```
DSSP  ------------------------------------------------------------
Query ------------------------------------------------------------    0
ident
Sbjct tlnggitdmltelanfeknvsqaihkynayrkaasviakyphkiksgaeakklpgvgtki   60
DSSP  lllhhhhhhhhhhhhhhhhllllhhhhhhhhhhhhhhhlllllllhhhhhlllllhhh


DSSP  ------------------------------------------------llllllllllhh
Query ------------------------------------------------aapysspptnlr   12
ident
Sbjct aekideflatgklrklekirqddtsssinfltrvsgigpsaarkfvdegiktledlrkne  120
DSSP  hhhhhhhhhlllhhhhhhhhlhhhhhhhhhllllllhhhhhhhhhlllllhhhhhhlh


DSSP  hhhhhhhhhhhllllhhhhhHHHHHHHHHHHHHH-LLLLLLLLLLEEEEHHHHHHHhhll
Query slrdrltqvaerqgvvfgrlqRHVAXIVVAQFAA-TLTDDTGAPLLLVKGGSSLELrrgi   71
ident                                                | |
Sbjct dklnhhqriglkyfgdfekriPREEMLQMQDIVLnEVKKVDSEYIATVCGSFRRGA----  176
DSSP  hhllhhhhhhhhlllhhhlleEHHHHHHHHHHHHHhHHHHHLLLLEEEELHHHHLLL----


DSSP  llllLLLLEEEEEL------------lLHHHHHHHHHHHHhhlllleeEEELLLEELlll
Query pdsrTSKDFATVAR------------rDIELIHEQLADAGetgwegftAIFTAPEEIaap  119
ident      | |                         |||             |
Sbjct ---eSSGDMDVLLThpsftsestkqpkLLHQVVEQLQKVH--------FITDTLSKG---  222
DSSP  ---lEELLEEEEEElllee1leellllHHHHHHHHHHHLL--------LEEEEEEL---


DSSP  lllLLLEEEEEEEEEL--LEEEEEEEEEEEL-LLLLlllleeeeelhhhhllllllleee
Query gxpVKPRRFTAKLSYR--GRAFATVPIEVSSV-EAGNadqfdtltsdalglvgvpaavav  176
ident             |             |
Sbjct ---ETKFMGVCQLPSKndEKEYPHRRIDIRLIpKDQY-----------------------  256
DSSP  ---LLEEEEEELLLLLllLLLLLLLEEEEEEEELlHHHH-----------------------


DSSP  eellhHHHHhhhhhhhhllllllllllllHHHHHHH-------------------hhhhh
Query pcxtiPWQIaqklhavtavleepkandraHDLVDLQ-------------------llegl  217
ident                               |
Sbjct ----yCGVL----------------yftgSDIFNKNmrahalekgftineytirplgvtg  296
DSSP  ----hHHHH----------------hhhlLHHHHHHhhhhhhhhleeellllleeellll


DSSP  llhHHHHhhHHHHHhhhhhhllllLLLLLLllllhhhhhhhhhllllllllllhhhhhh
Query lldADLXptRSACIaifearaqhpWPPRVAtlphwpliyagalegldhlelartvdaaaq  277
ident      |
Sbjct vagEPLP--VDSEK-------difDYIQWK------------------------------  317
DSSP  lllLLLL--LLLHH-------hhhHHLLLL------------------------------


DSSP  hhhhhhhhhhhllll
Query avqrfvaridratkr  292
ident
Sbjct ------yrepkdrse  326
DSSP  ------lllhhhlll
```

No 224: Query=mol1A Sbjct=1huoB Z-score=6.7

back to top

```
DSSP  ------------------------------------------------------------
Query ------------------------------------------------------------    0
ident
```

```
DSSP  lllhhhhhhhhhhhhhhhhhllllhhhhhhhhhhhhhhhhlllllllhhhhhlllllllhhh


DSSP  -------------------------------------------------llllllllllhh
Query -------------------------------------------------aapyssspptnlr   12
ident 
Sbjct aekideflatgklrklekirqddtsssinfltrvtgigpsaarklvdegiktledlrkne  120
DSSP  hhhhhhhhhllllhhhhhhhhlhhhhhhhhhhlllllllhhhhhhhhhlllllhhhhlllh


DSSP  hhhhhhhhhhhhllllhhhhhHHHHHHHHHHHHH-LLLLllLLLLEEEEHHHHHHHHhhll
Query slrdrltqvaerqgvvfgrlqRHVAXIVVAQFAA-TLTDdtGAPLLLVKGGSSLELrrgi   71
ident                                                |  |
Sbjct dklnhhqriglkyfedfekriPREEMLQMQDIVLnEVKKldPEYIATVCGSFRRGA----  176
DSSP  hhllhhhhhhhhhhhhhhhlleEHHHHHHHHHHHHHhHHHHhlLLLEEEEELLHHHHLL----


DSSP  llllLLLLEEEEEL------------lLHHHHHHHHHHHHhhlllleeEEELLLEELlll
Query pdsrTSKDFATVAR------------rDIELIHEQLADAGetgwegftAIFTAPEEIaap  119
ident      | |                         |||             |
Sbjct ---eSSGDMDVLLThpnftsesskqpkLLHRVVEQLQKVR--------FITDTLSKG---  222
DSSP  ---lEELLEEEEEEllllllllllhhHHHHHHHHHHHHLL--------LLLEEEEEL---


DSSP  lllLLLEEEEEEEEEL--LEEEEEEEEEEEEL-LLLLllllleeeeelhhhhlllllleee
Query gxpVKPRRFTAKLSYR--GRAFATVPIEVSSV-EAGNadqfdtltsdalglvgvpaavav  176
ident             |             |
Sbjct ---ETKFMGVCQLPSEndENEYPHRRIDIRLIpKDQY-----------------------  256
DSSP  ---LLEEEEEEELLLLLllLLLLLLEEEEEEEELlHHHH-----------------------


DSSP  eellhHHHHhhhhhhhhlllllllllllHHHHHHH-------------------hhhhh
Query pcxtiPWQIaqklhavtavleepkandraHDLVDLQ-------------------llegl  217
ident                               |
Sbjct ----yCGVL----------------yftgSDIFNKNmrahalekgftineytirplgvtg  296
DSSP  ----hHHHH----------------hhhlLHHHHHHhhhhhhhhleeellllleeelllll


DSSP  llhHHHHhhHHHHHhhhhhhllllLLLLLLllllhhhhhhhhhllllllllllhhhhhh
Query lldADLXptRSACIaifearaqhpWPPRVAtlphwpliyagalegldhlelartvdaaaq  277
ident      |
Sbjct vagEPLP--VDSEQ-------difDYIQWR------------------------------  317
DSSP  lllLLLL--LLLHH-------hhhHHLLLL------------------------------


DSSP  hhhhhhhhhhhllll
Query avqrfvaridratkr  292
ident 
Sbjct -------yrepkdrs  325
DSSP  -------llllllll
```

No 225: Query=mol1A Sbjct=2pxiA Z-score=6.7

back to top

```
DSSP  ------------------------------------------------------------
Query ------------------------------------------------------------    0
ident 
Sbjct tlnggitdmltelanfeknvsqaihkynayrkaasviakyphkiksgaeakklpgvgtki   60
DSSP  lllhhhhhhhhhhhhhhhhhllllhhhhhhhhhhhhhhhhllllllllhhhhhlllllllhhh


DSSP  -------------------------------------------------llllllllllhh
Query -------------------------------------------------aapyssspptnlr   12
ident 
Sbjct aekideflatgklrklekirqddtsssinfltrvsgigpsaarkfvdegiktledlrkne  120
DSSP  hhhhhhhhhhlllhhhhhhhhlhhhhhhhhhhlllllllhhhhhhhhhlllllhhhhhhllh


DSSP  hhhhhhhhhhhhllllhhhhhHHHHHHHHHHHHH-LLLLllLLLLEEEEHHHHHHHHhhll
Query slrdrltqvaerqgvvfgrlqRHVAXIVVAQFAA-TLTDdtGAPLLLVKGGSSLELrrgi   71
ident                                                |  |
Sbjct dklnhhqriglkyfgdfekriPREEMLQMQDIVLnEVKKvdSEYIATVCGSFRRGA----  176
DSSP  hhllhhhhhhhhhlllhhhhlleEHHHHHHHHHHHHHhHHHHhlLLLEEEEELHHHHLLL----
```

```
Query pdsrTSKDFATVAR------------rDIELIHEQLADAGetgwegftAIFTAPEEIaap  119
ident      | |                         |||             |
Sbjct ---eSSGDMDVLLThpsftsestkqpkLLHQVVEQLQKVH--------FITDTLSKG---  222
DSSP  ---lEELLEEEEEElllllllllllHHHHHHHHHHHLL--------LEEEEEEEL---


DSSP  lllLLLLEEEEEEEEEL--LEEEEEEEEEEEL-LLLLllllleeeeelhhhhllllleee
Query gxpVKPRRFTAKLSYR--GRAFATVPIEVSSV-EAGNadqfdtltsdalglvgvpaavav  176
ident             |             |
Sbjct ---ETKFMGVCQLPSKndEKEYPHRRIDIRLIpKDQY----------------------  256
DSSP  ---LLEEEEEELLLLLllLLLLLLEEEEEEELlHHHH----------------------


DSSP  eellhHHHHhhhhhhhhlllllllllllHHHHHHH------------------hhhhh
Query pcxtiPWQIaqklhavtavleepkandraHDLVDLQ------------------llegl  217
ident                               |
Sbjct ----yCGVL----------------yftgSDIFNKNmrahalekgftineytirplgvtg  296
DSSP  ----hHHHH----------------hhhlLHHHHHHhhhhhhhlleeellllleeelllll


DSSP  llhHHHHhhHHHHHhhhhhhllllLLLLLLllllhhhhhhhhhllllllllllhhhhhh
Query lldADLXptRSACIaifearaqhpWPPRVAtlphwpliyagalegldhlelartvdaaaq  277
ident      |
Sbjct vagEPLP--VDSEK-------difDYIQWK------------------------------  317
DSSP  lllLLLL--LLLHH-------hhhHHLLLL------------------------------


DSSP  hhhhhhhhhhhllll
Query avqrfvaridratkr  292
ident
Sbjct ------yrepkdrse  326
DSSP  ------lllhhhlll
```

No 226: Query=mol1A Sbjct=9icmA Z-score=6.7

back to top

```
DSSP  ------------------------------------------------------------
Query ------------------------------------------------------------    0
ident
Sbjct etlnggitdmltelanfeknvsqaihkynayrkaasviakyphkiksgaeakklpgvgtk   60
DSSP  llllhhhhhhhhhhhhhhhhllllhhhhhhhhhhhhhhhhlllllllhhhhhlllllhh


DSSP  -------------------------------------------------lllllllllh
Query -------------------------------------------------aapysspptnl   11
ident
Sbjct iaekideflatgklrklekirqddtsssinfltrvsgigpsaarkfvdegiktledlrkn  120
DSSP  hhhhhhhhhhllllhhhhhhlllhhhhhhhhhlllllllhhhhhhhhhlllllhhhhhhl


DSSP  hhhhhhhhhhhhhllllhhhhhHHHHHHHHHHHHH-LLLLllLLLLLEEEEHHHHHHHHhl
Query rslrdrltqvaerqgvvfgrlqRHVAXIVVAQFAA-TLTDdtGAPLLLVKGGSSLELrrg   70
ident                                                 | |
Sbjct edklnhhqriglkyfgdfekriPREEMLQMQDIVLnEVKKvdSEYIATVCGSFRRGA---  177
DSSP  hhhllhhhhhhhhlhhhhhlleEHHHHHHHHHHHHhHHHHhlLLLEEEELLLLLLLL---


DSSP  lllllLLLLEEEEEL------------lLHHHHHHHHHHHHhhllllleeEEELLLEELll
Query ipdsrTSKDFATVAR------------rDIELIHEQLADAGetgwegftAIFTAPEEIaa  118
ident       | |                         |||             |
Sbjct ----eSSGDMDVLLThpsftsestkqpkLLHQVVEQLQKVH--------FITDTLSKG--  223
DSSP  ----lEELLEEEEEElllllllllllhHHHHHHHHHHHLL--------LEEEEEEEL--


DSSP  llllLLLEEEEEEEEELL--EEEEEEEEEEEEL-LLLLllllleeeeelhhhhllllllee
Query pgxpVKPRRFTAKLSYRG--RAFATVPIEVSSV-EAGNadqfdtltsdalglvgvpaava  175
ident              |             |
Sbjct ----ETKFMGVCQLPSKNdeKEYPHRRIDIRLIpKDQY----------------------  257
DSSP  ----LLEEEEEELLLLLllLLLLLLEEEEEEEEeHHHH----------------------


DSSP  eeellhHHHHhhhhhhhhlllllllllllhhhhHHHHHH--------------------
Query vpcxtiPWQIaqklhavtavleepkandrahdlvDLQLLE--------------------  215
ident
Sbjct -----yCGVL--------------------yftgSDIFNKnmrahalekgftineytirp  292
```

```
DSSP  -hhllhhhhhhhHHHHHHhhhhhllllLLLLLlllllhhhhhhhhhllllllllllhhh
Query -glllldadlxptRSACIAifearaqhpWPPRVatlphwpliyagalegldhlelartvda  274
ident
Sbjct lgvtgvageplpVDSEKD------ifdYIQWK----------------------------  318
DSSP  lllllllllllLLLHHH------hhhHLLLL----------------------------


DSSP  hhhhhhhhhhhhhhllll
Query aaqavqrfvaridratkr  292
ident
Sbjct ---------yrepkdrse  327
DSSP  ---------lllhhhlll
```

No 227: Query=mol1A Sbjct=8icoA Z-score=6.7

[back to top]

```
DSSP  ------------------------------------------------------------
Query ------------------------------------------------------------    0
ident
Sbjct etlnggitdmltelanfeknvsqaihkynayrkaasviakyphkiksgaeakklpgvgtk   60
DSSP  llllhhhhhhhhhhhhhhhhlllllhhhhhhhhhhhhhhhhlllllllhhhhhllllllhh


DSSP  -------------------------------------------------lllllllllh
Query -------------------------------------------------aapysspptnl   11
ident
Sbjct iaekideflatgklrklekirqddtsssinfltrvsgigpsaarkfvdegiktledlrkn  120
DSSP  hhhhhhhhhhllllhhhhhhhhlhhhhhhhhhlllllllhhhhhhhhhhllllhhhhhhl


DSSP  hhhhhhhhhhhhhlllllhhhhhHHHHHHHHHHHHHH-LLLLllLLLLLEEEEHHHHHHHHhhl
Query rslrdrltqvaerqgvvfgrlqRHVAXIVVAQFAA-TLTDdtGAPLLLVKGGSSLELrrg   70
ident                                                 | |
Sbjct edklnhhqriglkyfgdfekriPREEMLQMQDIVLnEVKKvdSEYIATVCGSFRRGA---  177
DSSP  hhhllhhhhhhhhlhhhhhlleEHHHHHHHHHHHHHhHHHHhlLLLEEEELHHHHLLL---


DSSP  lllllLLLLEEEEEL------------lLHHHHHHHHHHHHHhhllllleeEEEELLLEELll
Query ipdsrTSKDFATVAR------------rDIELIHEQLADAGetgwegftAIFTAPEEIaa  118
ident       | |                         |||             |
Sbjct ----eSSGDMDVLLThpsftsestkqpkLLHQVVEQLQKVH--------FITDTLSKG--  223
DSSP  ----lEELLEEEEEEelllllllllllhHHHHHHHHHHHHLL--------LEEEEEEEL--


DSSP  llllLLLEEEEEEEEEELL--EEEEEEEEEEEEL-LLLLllllleeeeelhhhhllllllee
Query pgxpVKPRRFTAKLSYRG--RAFATVPIEVSSV-EAGNadqfdtltsdalglvgvpaava  175
ident              |             |
Sbjct ----ETKFMGVCQLPSKNdeKEYPHRRIDIRLIpKDQY----------------------  257
DSSP  ----LLEEEEEEELLLLLLllLLLLLLEEEEEEEEEeHHHH----------------------


DSSP  eeellhHHHHhhhhhhhhlllllllllllhhhhHHHHHH--------------------
Query vpcxtiPWQIaqklhavtavleepkandrahdlvDLQLLE--------------------  215
ident
Sbjct -----yCGVL--------------------yftgSDIFNKnmrahalekgftineytirp  292
DSSP  -----hHHHH--------------------hhhlLHHHHHhhhhhhhhhllllllllll


DSSP  -hhllhhhhhhhHHHHHHhhhhhllllLLLLLlllllhhhhhhhhhllllllllllhhh
Query -glllldadlxptRSACIAifearaqhpWPPRVatlphwpliyagalegldhlelartvda  274
ident
Sbjct lgvtgvageplpVDSEKD------ifdYIQWK----------------------------  318
DSSP  lllllllllllLLLHHH------hhhHLLLL----------------------------


DSSP  hhhhhhhhhhhhhhllll
Query aaqavqrfvaridratkr  292
ident
Sbjct ---------yrepkdrse  327
DSSP  ---------lllhhhlll
```

[illegible]

back to top

```
DSSP  ------------------------------------------------------------
Query ------------------------------------------------------------    0
ident 
Sbjct etlnggitdmltelanfeknvsqaihkynayrkaasviakyphkiksgaeakklpgvgtk   60
DSSP  llllhhhhhhhhhhhhhhhhhlllhhhhhhhhhhhhhhhhlllllllhhhhhllllllhh


DSSP  -------------------------------------------------llllllllllh
Query -------------------------------------------------aapysspptnl   11
ident 
Sbjct iaekideflatgklrklekirqddtsssinfltrvsgigpsaarkfvdegiktledlrkn  120
DSSP  hhhhhhhhhhllllhhhhhhhhlhhhhhhhhhlllllllhhhhhhhhhhllllhhhhhhl


DSSP  hhhhhhhhhhhhhllllhhhhhHHHHHHHHHHHHHH-LLLLllLLLLEEEEHHHHHHHHhhl
Query rslrdrltqvaerqgvvfgrlqRHVAXIVVAQFAA-TLTDdtGAPLLLVKGGSSLELrrg   70
ident                                                 | |
Sbjct edklnhhqriglkyfgdfekriPREEMLQMQDIVLnEVKKvdSEYIATVCGSFRRGA---  177
DSSP  hhhllhhhhhhhhlhhhhhlleEHHHHHHHHHHHHHHhHHHHhlLLLEEEEEELLLLLL---


DSSP  lllllLLLLEEEEEL------------lLHHHHHHHHHHHHHhhllllleeEEELLLEELll
Query ipdsrTSKDFATVAR------------rDIELIHEQLADAGetgwegftAIFTAPEEIaa  118
ident       | |                         |||             |
Sbjct ----eSSGDMDVLLThpsftsestkqpkLLHQVVEQLQKVH--------FITDTLSKG--  223
DSSP  ----lEELLEEEEEEllllllllllllhHHHHHHHHHHHHLL--------LEEEEEEEL--


DSSP  llllLLLEEEEEEEEEELL--EEEEEEEEEEEEL-LLLLllllleeeeelhhhhllllllee
Query pgxpVKPRRFTAKLSYRG--RAFATVPIEVSSV-EAGNadqfdtltsdalglvgvpaava  175
ident              |             |
Sbjct ----ETKFMGVCQLPSKNdeKEYPHRRIDIRLIpKDQY----------------------  257
DSSP  ----LLEEEEEEELLLLLLllLLLLLLEEEEEEEEElLLLH----------------------


DSSP  eeellhHHHHhhhhhhhhllllllllllllhhhhHHHHHH--------------------
Query vpcxtiPWQIaqklhavtavleepkandrahdlvDLQLLE--------------------  215
ident 
Sbjct -----yCGVL--------------------yftgSDIFNKnmrahalekgftineytirp  292
DSSP  -----hHHHH--------------------hhllLHHHHHhhhhhhhhlleeeelleeee


DSSP  -hhllhhhhhhhHHHHHhhhhhhlllLLLLLLlllllhhhhhhhhhllllllllllhhh
Query -gllldadlxptRSACIaifearaqhPWPPRVatlphwpliyagalegldhlelartvda  274
ident 
Sbjct lgvtgvageplpVDSEK------difDYIQWK----------------------------  318
DSSP  llllllllllllLLLHH------hhhHHLLLL----------------------------


DSSP  hhhhhhhhhhhhhhllll
Query aaqavqrfvaridratkr  292
ident 
Sbjct ---------yrepkdrse  327
DSSP  ---------lllhhhlll
```


No 229: Query=mol1A Sbjct=9iclA Z-score=6.7

back to top

```
DSSP  ------------------------------------------------------------
Query ------------------------------------------------------------    0
ident 
Sbjct etlnggitdmltelanfeknvsqaihkynayrkaasviakyphkiksgaeakklpgvgtk   60
DSSP  llllhhhhhhhhhhhhhhhhhllllhhhhhhhhhhhhhhhhlllllllhhhhhllllllhh


DSSP  -------------------------------------------------llllllllllh
Query -------------------------------------------------aapysspptnl   11
ident 
Sbjct iaekideflatgklrklekirqddtsssinfltrvsgigpsaarkfvdegiktledlrkn  120
DSSP  hhhhhhhhhhllllhhhhhhhhlhhhhhhhhhlllllllhhhhhhhhhllllllhhhhhhl
```

```
DSSP  hhhhhhhhhhhhhllllhhhhhHHHHHHHHHHHHH-LLLLllLLLLEEEEHHHHHHHHhhl
Query rslrdrltqvaerqgvvfgrlqRHVAXIVVAQFAA-TLTDdtGAPLLLVKGGSSLELrrg   70
ident                                                 | |
Sbjct edklnhhqriglkyfgdfekriPREEMLQMQDIVLnEVKKvdSEYIATVCGSFRRGA---  177
DSSP  hhhllhhhhhhhhlhhhhhlleEHHHHHHHHHHHHhHHHHhlLLLEEEELLLLLLL---


DSSP  lllllLLLLEEEEEL------------lLHHHHHHHHHHHHhhllllleeEEELLLEELll
Query ipdsrTSKDFATVAR------------rDIELIHEQLADAGetgwegftAIFTAPEEIaa  118
ident       | |                         |||             |
Sbjct ----eSSGDMDVLLThpsftsestkqpkLLHQVVEQLQKVH--------FITDTLSKG--  223
DSSP  ----lEELLEEEEEEellllllllllhHHHHHHHHHHHHLL--------LEEEEEEEL--


DSSP  llllLLLEEEEEEEEELL--EEEEEEEEEEEEL-LLLLllllleeeeelhhhhllllllee
Query pgxpVKPRRFTAKLSYRG--RAFATVPIEVSSV-EAGNadqfdtltsdalglvgvpaava  175
ident              |             |
Sbjct ----ETKFMGVCQLPSKNdeKEYPHRRIDIRLIpKDQY----------------------  257
DSSP  ----LLEEEEEEELLLLLllLLLLLLEEEEEEEEEeHHHH----------------------


DSSP  eeellhHHHHhhhhhhhhlllllllllllhhhhHHHHHH--------------------
Query vpcxtiPWQIaqklhavtavleepkandrahdlvDLQLLE--------------------  215
ident
Sbjct -----yCGVL--------------------yftgSDIFNKnmrahalekgftineytirp  292
DSSP  -----hHHHH--------------------hhhlLHHHHHhhhhhhhhlleeellllleee


DSSP  --hhllhhhhhhhhHHHHhhhhhhllllLLLLLlllllhhhhhhhhhllllllllllhh
Query --glllddadlxptrSACIaifearaqhpWPPRVatlphwpliyagalegldhlelartvd  273
ident               |
Sbjct lgvtgvageplpvdSEKD-------ifdYIQWK---------------------------  318
DSSP  lllllllllllllLHHH-------hhhHLLLL---------------------------


DSSP  hhhhhhhhhhhhhhhllll
Query aaaqavqrfvaridratkr  292
ident
Sbjct ----------yrepkdrse  327
DSSP  ----------lllhhhhll
```

No 230: Query=mol1A Sbjct=8iccA Z-score=6.7

back to top

```
DSSP  ------------------------------------------------------------
Query ------------------------------------------------------------    0
ident
Sbjct etlnggitdmltelanfeknvsqaihkynayrkaasviakyphkiksgaeakklpgvgtk   60
DSSP  llllhhhhhhhhhhhhhhhhhlllhhhhhhhhhhhhhhhhlllllllhhhhhlllllllhh


DSSP  -------------------------------------------------lllllllllh
Query -------------------------------------------------aapysspptnl   11
ident
Sbjct iaekideflatgklrklekirqddtsssinfltrvsgigpsaarkfvdegiktledlrkn  120
DSSP  hhhhhhhhhhllllhhhhhhhhlhhhhhhhhhlllllllhhhhhhhhhhllllhhhhhhl


DSSP  hhhhhhhhhhhhhllllhhhhhHHHHHHHHHHHHH-LLLLllLLLLEEEEHHHHHHHHhhl
Query rslrdrltqvaerqgvvfgrlqRHVAXIVVAQFAA-TLTDdtGAPLLLVKGGSSLELrrg   70
ident                                                 | |
Sbjct edklnhhqriglkyfgdfekriPREEMLQMQDIVLnEVKKvdSEYIATVCGSFRRGA---  177
DSSP  hhhllhhhhhhhhlhhhhhlleEHHHHHHHHHHHHhHHHHhlLLLEEEELLLLLLL---


DSSP  lllllLLLLEEEEEL------------lLHHHHHHHHHHHHhhllllleeEEELLLEELll
Query ipdsrTSKDFATVAR------------rDIELIHEQLADAGetgwegftAIFTAPEEIaa  118
ident       | |                         |||             |
Sbjct ----eSSGDMDVLLThpsftsestkqpkLLHQVVEQLQKVH--------FITDTLSKG--  223
DSSP  ----lEELLEEEEEEellllllllllhHHHHHHHHHHHHLL--------LEEEEEEEL--


DSSP  llllLLLEEEEEEEEELL--EEEEEEEEEEEEL-LLLLllllleeeeelhhhhllllllee
Query pgxpVKPRRFTAKLSYRG--RAFATVPIEVSSV-EAGNadqfdtltsdalglvgvpaava  175
ident              |             |
```

```
DSSP  ----LLEEEEEEELLLLLLllLLLLLLEEEEEEEEEeHHHH----------------------


DSSP  eeellhHHHHhhhhhhhhlllllllllllhhhhHHHHHH--------------------
Query vpcxtiPWQIaqklhavtavleepkandrahdlvDLQLLE--------------------  215
ident
Sbjct -----yCGVL--------------------yftgSDIFNKnmrahalekgftineytirp  292
DSSP  -----hHHHH--------------------hhhlLHHHHHhhhhhhhhllllleelleell


DSSP  --hhllhhhhhhhhHHHHhhhhhhllllLLLLLlllllhhhhhhhhhllllllllllhh
Query --glllldadlxptrSACIaifearaqhpWPPRVatlphwpliyagalegldhlelartvd  273
ident               |
Sbjct lgvtgvageplpvdSEKD-------ifdYIQWK---------------------------  318
DSSP  llllllllllllllLHHH-------hhhHLLLL---------------------------


DSSP  hhhhhhhhhhhhhhhllll
Query aaaqavqrfvaridratkr  292
ident
Sbjct ----------yrepkdrse  327
DSSP  ----------lllhhhlll
```

No 231: Query=mol1A Sbjct=9icwA Z-score=6.7

back to top

```
DSSP  ------------------------------------------------------------
Query ------------------------------------------------------------    0
ident
Sbjct etlnggitdmltelanfeknvsqaihkynayrkaasviakyphkiksgaeakklpgvgtk   60
DSSP  llllhhhhhhhhhhhhhhhhllllhhhhhhhhhhhhhhhhlllllllhhhhhllllllhh


DSSP  ------------------------------------------------llllllllllh
Query ------------------------------------------------aapyssppptnl   11
ident
Sbjct iaekideflatgklrklekirqddtsssinfltrvsgigpsaarkfvdegiktledlrkn  120
DSSP  hhhhhhhhhhllllhhhhhhlllhhhhhhhhhllllllhhhhhhhhhllllhhhhhhl


DSSP  hhhhhhhhhhhhhllllhhhhhHHHHHHHHHHHHH-LLLLllLLLLLEEEEHHHHHHHhhl
Query rslrdrltqvaerqgvvfgrlqRHVAXIVVAQFAA-TLTDdtGAPLLLVKGGSSLELrrg   70
ident                                                 | |
Sbjct edklnhhqriglkyfgdfekriPREEMLQMQDIVLnEVKKvdSEYIATVCGSFRRGA---  177
DSSP  hhhllhhhhhhhhlllhhhlleEHHHHHHHHHHHHHhHHHHhlLLLEEEEELLLLLLL---


DSSP  lllllLLLLEEEEEEL------------lLHHHHHHHHHHHHhhllllleeEEELLLEELll
Query ipdsrTSKDFATVAR------------rDIELIHEQLADAGetgwegftAIFTAPEEIaa  118
ident       | |                         |||             |
Sbjct ----eSSGDMDVLLThpsftsestkqpkLLHQVVEQLQKVH--------FITDTLSKG--  223
DSSP  ----lEELLEEEEEEellllllllllhHHHHHHHHHHHHLL--------LEEEEEEEL--


DSSP  llllLLLEEEEEEEEEELL--EEEEEEEEEEEEL-LLLLlllleeeeelhhhhllllllee
Query pgxpVKPRRFTAKLSYRG--RAFATVPIEVSSV-EAGNadqfdtltsdalglvgvpaava  175
ident              |             |
Sbjct ----ETKFMGVCQLPSKNdeKEYPHRRIDIRLIpKDQY----------------------  257
DSSP  ----LLEEEEEEELLLLLLllLLLLLLEEEEEEEEEeHHHH----------------------


DSSP  eeellhHHHHhhhhhhhhlllllllllllhhhhHHHHHH--------------------
Query vpcxtiPWQIaqklhavtavleepkandrahdlvDLQLLE--------------------  215
ident
Sbjct -----yCGVL--------------------yftgSDIFNKnmrahalekgftineytirp  292
DSSP  -----hHHHH--------------------hhhlLHHHHHhhhhhhhhlleeeelleeee


DSSP  -hhllhhhhhhhHHHHhhhhhhllllLLLLLlllllhhhhhhhhhllllllllllhhh
Query -glllldadlxptRSACIaifearaqhpWPPRVatlphwpliyagalegldhlelartvda  274
ident
Sbjct lgvtgvageplpVDSEK-------difDYIQW----------------------------  317
DSSP  lllllllllllLLLHH-------hhhHHLLL----------------------------
```

```
Query aaqavqrfvaridratkr  292
ident
Sbjct --------kyrepkdrse  327
DSSP  --------llllhhhlll
```

No 232: Query=mol1A Sbjct=7iciA Z-score=6.7

back to top

```
DSSP  ------------------------------------------------------------
Query ------------------------------------------------------------    0
ident
Sbjct etlnggitdmltelanfeknvsqaihkynayrkaasviakyphkiksgaeakklpgvgtk   60
DSSP  llllhhhhhhhhhhhhhhhhllllhhhhhhhhhhhhhhhhllllllllhhhhhlllllllhh


DSSP  -------------------------------------------------lllllllllh
Query -------------------------------------------------aapysspptnl   11
ident
Sbjct iaekideflatgklrklekirqddtsssinfltrvsgigpsaarkfvdegiktledlrkn  120
DSSP  hhhhhhhhhhllllhhhhhhlllhhhhhhhhhllllllhhhhhhhhllllllhhhhhhl


DSSP  hhhhhhhhhhhhllllhhhhhHHHHHHHHHHHHH-LLLLllLLLLEEEEHHHHHHHHhhl
Query rslrdrltqvaerqgvvfgrlqRHVAXIVVAQFAA-TLTDdtGAPLLLVKGGSSLELrrg   70
ident                                                 | |
Sbjct edklnhhqriglkyfgdfekriPREEMLQMQDIVLnEVKKvdSEYIATVCGSFRRGA---  177
DSSP  hhhllhhhhhhhhlhhhhhlleEHHHHHHHHHHHHhHHHHhlLLLEEEELLLLLLL---


DSSP  lllllLLLLEEEEEL------------lLHHHHHHHHHHHHhhlllleeEEELLLEELll
Query ipdsrTSKDFATVAR------------rDIELIHEQLADAGetgwegftAIFTAPEEIaa  118
ident       | |                         |||             |
Sbjct ----eSSGDMDVLLThpsftsestkqpkLLHQVVEQLQKVH--------FITDTLSKG--  223
DSSP  ----lEELLEEEEEEellllllllllhHHHHHHHHHHHHLL--------LEEEEEEEL--


DSSP  llllLLLEEEEEEEEEELL--EEEEEEEEEEEEL-LLLLllllleeeeelhhhhllllllee
Query pgxpVKPRRFTAKLSYRG--RAFATVPIEVSSV-EAGNadqfdtltsdalglvgvpaava  175
ident              |             |
Sbjct ----ETKFMGVCQLPSKNdeKEYPHRRIDIRLIpKDQY----------------------  257
DSSP  ----LLEEEEEEELLLLLllLLLLLEEEEEEEEEeHHHH----------------------


DSSP  eeellhHHHHhhhhhhhhllllllllllllhhhhHHHHHH--------------------
Query vpcxtiPWQIaqklhavtavleepkandrahdlvDLQLLE--------------------  215
ident
Sbjct -----yCGVL--------------------yftgSDIFNKnmrahalekgftineytirp  292
DSSP  -----hHHHH--------------------hhhlLHHHHHhhhhhhhhlleeeelleeee


DSSP  -hhllhhhhhhhHHHHHHhhhhhllllLLLLLlllllhhhhhhhhhlllllllllllhhh
Query -glllдadlxptRSACIAifearaqhpWPPRVatlphwpliyagalegldhlelartvda  274
ident
Sbjct lgvtgvageplpVDSEKD------ifdYIQWK----------------------------  318
DSSP  lllllllllllLLLHHH------hhhHLLLL----------------------------


DSSP  hhhhhhhhhhhhhhllll
Query aaqavqrfvaridratkr  292
ident
Sbjct ---------yrepkdrse  327
DSSP  ---------lllhhhhll
```

No 233: Query=mol1A Sbjct=7icrA Z-score=6.7

back to top

```
DSSP  ------------------------------------------------------------
Query ------------------------------------------------------------    0
ident
Sbjct etlnggitdmltelanfeknvsqaihkynayrkaasviakyphkiksgaeakklpgvgtk   60
DSSP  llllhhhhhhhhhhhhhhhhllllhhhhhhhhhhhhhhhhllllllllhhhhhlllllllhh
```

```
DSSP  -------------------------------------------------llllllllllh
Query -------------------------------------------------aapysspptnl   11
ident 
Sbjct iaekideflatgklrklekirqddtsssinfltrvsgigpsaarkfvdegiktledlrkn  120
DSSP  hhhhhhhhhhllllhhhhhhhhlhhhhhhhhhllllllhhhhhhhhllllllhhhhhhl
```

```
DSSP  hhhhhhhhhhhhhllllhhhhhHHHHHHHHHHHHHH-LLLLllLLLLEEEEHHHHHHHHhhl
Query rslrdrltqvaerqgvvfgrlqRHVAXIVVAQFAA-TLTDdtGAPLLLVKGGSSLELrrg   70
ident                                                 | |
Sbjct edklnhhqriglkyfgdfekriPREEMLQMQDIVLnEVKKvdSEYIATVCGSFRRGA---  177
DSSP  lllllhhhhhhhhlhhhhhlleEHHHHHHHHHHHHHhHHHHhlLLLEEEEELLHHHHLL---
```

```
DSSP  lllllLLLLEEEEEL------------lLHHHHHHHHHHHHhhlllleeEEELLLEELll
Query ipdsrTSKDFATVAR------------rDIELIHEQLADAGetgwegfftAIFTAPEEIaa  118
ident       | |                         |||             |
Sbjct ----eSSGDMDVLLThpsftsestkqpkLLHQVVEQLQKVH--------FITDTLSKG--  223
DSSP  ----lEELLEEEEEEellllllllllhHHHHHHHHHHHHLL--------LEEEEEEEL--
```

```
DSSP  llllLLLEEEEEEEEEELL--EEEEEEEEEEEEL-LLLLllllleeeeelhhhhllllllee
Query pgxpVKPRRFTAKLSYRG--RAFATVPIEVSSV-EAGNadqfdtltsdalglvgvpaava  175
ident              |             |
Sbjct ----ETKFMGVCQLPSKNdeKEYPHRRIDIRLIpKDQY----------------------  257
DSSP  ----LLEEEEEELLLLLLllLLLLLLEEEEEEEEEeHHHH----------------------
```

```
DSSP  eeellhHHHHhhhhhhhhlllllllllllhhhhHHHHHH--------------------
Query vpcxtiPWQIaqklhavtavleepkandrahdlvDLQLLE--------------------  215
ident 
Sbjct -----yCGVL--------------------yftgSDIFNKnmrahalekgftineytirp  292
DSSP  -----hHHHH--------------------hhhlLHHHHHhhhhhhhhlleeellllleee
```

```
DSSP  -hhllhhhhhhhHHHHHHhhhhhllllLLLLLlllllhhhhhhhhhlllllllllllhhh
Query -gllldadlxptRSACIAifearaqhpWPPRVatlphwpliyagalegldhlelartvda  274
ident 
Sbjct lgvtgvageplpVDSEKD------ifdYIQWK----------------------------  318
DSSP  llllllllllllLLLHHH------hhhHLLLL----------------------------
```

```
DSSP  hhhhhhhhhhhhhhhllll
Query aaqavqrfvaridratkr  292
ident 
Sbjct ---------yrepkdrse  327
DSSP  ---------lllhhhlll
```

No 234: Query=mol1A Sbjct=8icbA Z-score=6.7

back to top

```
DSSP  ------------------------------------------------------------
Query ------------------------------------------------------------    0
ident 
Sbjct etlnggitdmltelanfeknvsqaihkynayrkaasviakyphkiksgaeakklpgvgtk   60
DSSP  llllhhhhhhhhhhhhhhhhllllhhhhhhhhhhhhhhhllllllllllhhhhhllllllhh
```

```
DSSP  -------------------------------------------------llllllllllh
Query -------------------------------------------------aapysspptnl   11
ident 
Sbjct iaekideflatgklrklekirqddtsssinfltrvsgigpsaarkfvdegiktledlrkn  120
DSSP  hhhhhhhhhhllllhhhhhhhhlhhhhhhhhhllllllhhhhhhhhhhllllhhhhhhl
```

```
DSSP  hhhhhhhhhhhhhllllhhhhhHHHHHHHHHHHHHH-LLLLllLLLLEEEEHHHHHHHHhhl
Query rslrdrltqvaerqgvvfgrlqRHVAXIVVAQFAA-TLTDdtGAPLLLVKGGSSLELrrg   70
ident                                                 | |
Sbjct edklnhhqriglkyfgdfekriPREEMLQMQDIVLnEVKKvdSEYIATVCGSFRRGA---  177
DSSP  hhhllhhhhhhhhlhhhhhlleEHHHHHHHHHHHHHhHHHHhlLLLEEEEELLLLLLL---
```

```
DSSP  lllllLLLLEEEEEL------------lLHHHHHHHHHHHHhhlllleeEEELLLEELll
Query ipdsrTSKDFATVAR------------rDIELIHEQLADAGetgwegfftAIFTAPEEIaa  118
```

```
Sbjct ----eSSGDMDVLLThpsftsestkqpkLLHQVVEQLQKVH--------FITDTLSKG--  223
DSSP  ----lEELLEEEEEElllllllllllhHHHHHHHHHHHHLL--------LEEEEEEEL--


DSSP  llllLLLEEEEEEEEELL--EEEEEEEEEEEELLLLLLllleeeeelhhhhllllllleee
Query pgxpVKPRRFTAKLSYRG--RAFATVPIEVSSVEAGNAdqfdtltsdalglvgvpaavav  176
ident              |             |
Sbjct ----ETKFMGVCQLPSKNdeKEYPHRRIDIRLIPKDQY----------------------  257
DSSP  ----LLEEEEEELLLLLLlLLLLLLEEEEEEELLLLLLH----------------------


DSSP  eellhHHHHhhhhhhhhllllllllllllhhhhHHHHHH---------------------
Query pcxtiPWQIaqklhavtavleepkandrahdlvDLQLLE---------------------  215
ident
Sbjct ----yCGVL--------------------yftgSDIFNKnmrahalekgftineytirpl  293
DSSP  ----hHHHH--------------------hhhlLHHHHHhhhhhhhhlleeeelleeeel


DSSP  -hhllhhhhhhhhHHHHhhhhhhllllLLLLLlllllhhhhhhhhhllllllllllhhh
Query -gllldadlxptrSACIaifearaqhpWPPRVatlphwpliyagalegldhlelartvda  274
ident              |
Sbjct gvtgvageplpvdSEKD-------ifdYIQWK----------------------------  318
DSSP  lllllllllllllLHHH-------hhhHHLLL----------------------------


DSSP  hhhhhhhhhhhhhhllll
Query aaqavqrfvaridratkr  292
ident
Sbjct ---------yrepkdrse  327
DSSP  ---------lllhhhlll
```

No 235: Query=mol1A Sbjct=8icvA Z-score=6.7

back to top

```
DSSP  ------------------------------------------------------------
Query ------------------------------------------------------------    0
ident
Sbjct etlnggitdmltelanfeknvsqaihkynayrkaasviakyphkiksgaeakklpgvgtk   60
DSSP  llllhhhhhhhhhhhhhhhlllllhhhhhhhhhhhhhhhhlllllllhhhhhlllllhh


DSSP  -------------------------------------------------llllllllllh
Query -------------------------------------------------aapysspptnl   11
ident
Sbjct iaekideflatgklrklekirqddtsssinfltrvsgigpsaarkfvdegiktledlrkn  120
DSSP  hhhhhhhhhhllllhhhhhhhhlhhhhhhhhhlllllllhhhhhhhhhhllllhhhhhhl


DSSP  hhhhhhhhhhhhhllllhhhhhHHHHHHHHHHHHH-LLLLllLLLLLEEEEHHHHHHHhhl
Query rslrdrltqvaerqgvvfgrlqRHVAXIVVAQFAA-TLTDdtGAPLLLVKGGSSLELrrg   70
ident                                                 | |
Sbjct edklnhhqriglkyfgdfekriPREEMLQMQDIVLnEVKKvdSEYIATVCGSFRRGA---  177
DSSP  hhhllhhhhhhhhlhhhhhlleEHHHHHHHHHHHHhHHHHhlLLLEEEEEELLLLLLL---


DSSP  lllllLLLLEEEEEL------------lLHHHHHHHHHHHHhhllllleeEEELLLEELll
Query ipdsrTSKDFATVAR------------rDIELIHEQLADAGetgwegftAIFTAPEEIaa  118
ident       | |                         |||             |
Sbjct ----eSSGDMDVLLThpsftsestkqpkLLHQVVEQLQKVH--------FITDTLSKG--  223
DSSP  ----lEELLEEEEEElllllllllllhHHHHHHHHHHHHLL--------LEEEEEEEL--


DSSP  llllLLLEEEEEEEEELL--EEEEEEEEEEEEL-LLLLllllleeeeelhhhhllllllee
Query pgxpVKPRRFTAKLSYRG--RAFATVPIEVSSV-EAGNadqfdtltsdalglvgvpaava  175
ident              |             |
Sbjct ----ETKFMGVCQLPSKNdeKEYPHRRIDIRLIpKDQY----------------------  257
DSSP  ----LLEEEEEELLLLLLlLLLLLLEEEEEEELLlHHHH----------------------


DSSP  eeellhHHHHhhhhhhhhllllllllllllhhhhHHHHHH--------------------
Query vpcxtiPWQIaqklhavtavleepkandrahdlvDLQLLE--------------------  215
ident
Sbjct -----yCGVL--------------------yftgSDIFNKnmrahalekgftineytirp  292
DSSP  -----hHHHH--------------------hhhlLHHHHHhhhhhhhhllllleelleell
```

```
DSSP  --hhllhhhhhhhhHHHhhhhhhhllllLLLLLlllllhhhhhhhhhllllllllllhh
Query --glllldadlxptrSACIaifearaqhpWPPRVatlphwpliyagalegldhlelartvd  273
ident               |
Sbjct lgvtgvageplpvdSEKD-------ifdYIQWK---------------------------  318
DSSP  llllllllllllllLHHH-------hhhHHLLL---------------------------


DSSP  hhhhhhhhhhhhhhhllll
Query aaaqavqrfvaridratkr  292
ident
Sbjct ----------yrepkdrse  327
DSSP  ----------lllllllll
```

No 236: Query=mol1A Sbjct=9icuA Z-score=6.7

back to top

```
DSSP  ------------------------------------------------------------
Query ------------------------------------------------------------    0
ident
Sbjct etlnggitdmltelanfeknvsqaihkynayrkaasviakyphkiksgaeakklpgvgtk   60
DSSP  llllhhhhhhhhhhhhhhhhllllhhhhhhhhhhhhhhhhlllllllhhhhhllllllhh


DSSP  -------------------------------------------------llllllllllh
Query -------------------------------------------------aapysspptnl   11
ident
Sbjct iaekideflatgklrklekirqddtsssinfltrvsgigpsaarkfvdegiktledlrkn  120
DSSP  hhhhhhhhhhllllhhhhhhhhlhhhhhhhhhlllllllhhhhhhhhhhllllhhhhhhl


DSSP  hhhhhhhhhhhhhllllhhhhhHHHHHHHHHHHHHH-LLLLLLLLLLEEEEHHHHHHHHhl
Query rslrdrltqvaerqgvvfgrlqRHVAXIVVAQFAA-TLTDDTGAPLLLVKGGSSLELrrg   70
ident                                                 | |
Sbjct edklnhhqriglkyfgdfekriPREEMLQMQDIVLnEVKKVDSEYIATVCGSFRRGA---  177
DSSP  hhhllhhhhhhhhlhhhhhlleEHHHHHHHHHHHHHhHHHHHLLLLEEEEELLLLLLL---


DSSP  lllllLLLLEEEEEEL------------lLHHHHHHHHHHHHhhllleeEEELLLEELll
Query ipdsrTSKDFATVAR------------rDIELIHEQLADAGetgwegftAIFTAPEEIaa  118
ident       | |                         |||             |
Sbjct ----eSSGDMDVLLThpsftsestkqpkLLHQVVEQLQKVH--------FITDTLSKG--  223
DSSP  ----lEELLEEEEEEellllllllllhhHHHHHHHHHHHHLL--------LEEEEEEEL--


DSSP  llllLLLEEEEEEEEELL--EEEEEEEEEEEEL-LLLLlllleeeeelhhhhllllllee
Query pgxpVKPRRFTAKLSYRG--RAFATVPIEVSSV-EAGNadqfdtltsdalglvgvpaava  175
ident              |             |
Sbjct ----ETKFMGVCQLPSKNdeKEYPHRRIDIRLIpKDQY----------------------  257
DSSP  ----LLEEEEEEELLLLLllLLLLLLEEEEEEEEeHHHH----------------------


DSSP  eeellhHHHHhhhhhhhhllllllllllllhhhhHHHHHH--------------------
Query vpcxtiPWQIaqklhavtavleepkandrahdlvDLQLLE--------------------  215
ident
Sbjct -----yCGVL--------------------yftgSDIFNKnmrahalekgftineytirp  292
DSSP  -----hHHHH--------------------hhllLHHHHHhhhhhhhhlleeellllleee


DSSP  -hhllhhhhhhhHHHHHhhhhhhllllLLLLLlllllhhhhhhhhhllllllllllhhh
Query -glllldadlxptRSACIaifearaqhpWPPRVatlphwpliyagalegldhlelartvda  274
ident
Sbjct lgvtgvageplpVDSEK-------difDYIQW----------------------------  317
DSSP  llllllllllllLLLHH-------hhhHHLLL----------------------------


DSSP  hhhhhhhhhhhhhhllll
Query aaqavqrfvaridratkr  292
ident
Sbjct --------kyrepkdrse  327
DSSP  --------llllllllll
```

No 237: Query=mol1A Sbjct=1zqkA Z-score=6.7

```
DSSP  ------------------------------------------------------------
Query ------------------------------------------------------------    0
ident
Sbjct etlnggitdmltelanfeknvsqaihkynayrkaasviakyphkiksgaeakklpgvgtk   60
DSSP  llllhhhhhhhhhhhhhhhllllhhhhhhhhhhhhhhhlllllllhhhhhlllllhh


DSSP  ------------------------------------------------llllllllllh
Query ------------------------------------------------aapysspptnl   11
ident
Sbjct iaekideflatgklrklekirqddtsssinfltrvsgigpsaarkfvdegiktledlrkn  120
DSSP  hhhhhhhhhhllllhhhhhhhhlhhhhhhhhhlllllllhhhhhhhhhhllllhhhhhhl


DSSP  hhhhhhhhhhhhhllllhhhhhHHHHHHHHHHHHH-LLLLllLLLLEEEEHHHHHHHhhl
Query rslrdrltqvaerqgvvfgrlqRHVAXIVVAQFAA-TLTDdtGAPLLLVKGGSSLELrrg   70
ident                                                 | |
Sbjct edklnhhqriglkyfgdfekriPREEMLQMQDIVLnEVKKvdSEYIATVCGSFRRGA---  177
DSSP  hhhllhhhhhhhhlhhhhhlleEHHHHHHHHHHHHhHHHHhlLLLEEEEELLLLLL---


DSSP  lllllLLLLEEEEEL------------lLHHHHHHHHHHHHHhhlllleeEEELLLEELll
Query ipdsrTSKDFATVAR------------rDIELIHEQLADAGetgwegftAIFTAPEEIaa  118
ident       | |                         |||             |
Sbjct ----eSSGDMDVLLThpsftsestkqpkLLHQVVEQLQKVH--------FITDTLSKG--  223
DSSP  ----lEELLEEEEEEllllllllllhHHHHHHHHHHHHLL--------LEEEEEEEL--


DSSP  llllLLLEEEEEEEEELL--EEEEEEEEEEEL-LLLLllllleeeeelhhhhllllllee
Query pgxpVKPRRFTAKLSYRG--RAFATVPIEVSSV-EAGNadqfdtltsdalglvgvpaava  175
ident              |             |
Sbjct ----ETKFMGVCQLPSKNdeKEYPHRRIDIRLIpKDQY----------------------  257
DSSP  ----LLEEEEEELLLLLLllLLLLLEEEEEELLlHHHH----------------------


DSSP  eeellhHHHHhhhhhhhhllllllllllllhhhhHHHHHH--------------------
Query vpcxtiPWQIaqklhavtavleepkandrahdlvDLQLLE--------------------  215
ident
Sbjct -----yCGVL--------------------yftgSDIFNKnmrahalekgftineytirp  292
DSSP  -----hHHHH--------------------hhllLHHHHHhhhhhhhhlleeeelleeee


DSSP  -hhllhhhhhhhhHHHHHhhhhhhllllLLLLLLllllhhhhhhhhhlllllllllllhhh
Query -gllldadlxptRSACIaifearaqhpWPPRVAtlphwpliyagalegldhlelartvda  274
ident
Sbjct lgvtgvageplpVDSEK-------difDYIQWK---------------------------  318
DSSP  llllllllllllLLLHH-------hhhHHLLLL---------------------------


DSSP  hhhhhhhhhhhhhhllll
Query aaqavqrfvaridratkr  292
ident
Sbjct ---------yrepkdrse  327
DSSP  ---------lllhhhlll
```

No 238: Query=mol1A Sbjct=7icsA Z-score=6.7

back to top

```
DSSP  ------------------------------------------------------------
Query ------------------------------------------------------------    0
ident
Sbjct etlnggitdmltelanfeknvsqaihkynayrkaasviakyphkiksgaeakklpgvgtk   60
DSSP  llllhhhhhhhhhhhhhhhllllhhhhhhhhhhhhhhhlllllllhhhhhlllllhh


DSSP  ------------------------------------------------llllllllllh
Query ------------------------------------------------aapysspptnl   11
ident
Sbjct iaekideflatgklrklekirqddtsssinfltrvsgigpsaarkfvdegiktledlrkn  120
DSSP  hhhhhhhhhhllllhhhhhhhhlhhhhhhhhhlllllllhhhhhhhhhhllllhhhhhhl


DSSP  hhhhhhhhhhhhhllllhhhhhHHHHHHHHHHHHH-LLLLllLLLLEEEEHHHHHHHhhl
Query rslrdrltqvaerqgvvfgrlqRHVAXIVVAQFAA-TLTDdtGAPLLLVKGGSSLELrrg   70
```

```
Sbjct edklnhhqriglkyfgdfekriPREEMLQMQDIVLnEVKKvdSEYIATVCGSFRRGA---  177
DSSP  hhhllhhhhhhhhhhhhhlleEHHHHHHHHHHHHhHHHHhlLLLEEEELLHHHHLL---


DSSP  lllllLLLLEEEEEL------------lLHHHHHHHHHHHHHhhllllleeEEELLLEELll
Query ipdsrTSKDFATVAR------------rDIELIHEQLADAGetgwegftAIFTAPEEIaa  118
ident       | |                       |||           |
Sbjct ----eSSGDMDVLLThpsftsestkqpkLLHQVVEQLQKVH--------FITDTLSKG--  223
DSSP  ----lEELLEEEEEEellllllllllhHHHHHHHHHHHHL--------LEEEEEEEL--


DSSP  llllLLLEEEEEEEEELL--EEEEEEEEEEEEL-LLLLllllleeeeelhhhhlllllllee
Query pgxpVKPRRFTAKLSYRG--RAFATVPIEVSSV-EAGNadqfdtltsdalglvgvpaava  175
ident              |              |
Sbjct ----ETKFMGVCQLPSKNdeKEYPHRRIDIRLIpKDQY----------------------  257
DSSP  ----LLEEEEEEELLLLLLllLLLLLLEEEEEEEEEeHHHH----------------------


DSSP  eeellhHHHHhhhhhhhhllllllllllhhhhHHHHHH--------------------
Query vpcxtiPWQIaqklhavtavleepkandrahdlvDLQLLE--------------------  215
ident
Sbjct -----yCGVL--------------------yftgSDIFNKnmrahalekgftineytirp  292
DSSP  -----hHHHH--------------------hhhlLHHHHHhhhhhhhhlleeellllleee


DSSP  -hhllhhhhhhhHHHHHHhhhhhllllLLLLLlllllhhhhhhhhhllllllllllhhh
Query -gllldadlxptRSACIAifearaqhpWPPRVatlphwpliyagalegldhlelartvda  274
ident
Sbjct lgvtgvageplpVDSEKD------ifdYIQWK----------------------------  318
DSSP  lllllllllllLLLHHH------hhhHLLLL----------------------------


DSSP  hhhhhhhhhhhhhhllll
Query aaqavqrfvaridratkr  292
ident
Sbjct ---------yrepkdrse  327
DSSP  ---------lllllllll
```

No 239: Query=mol1A Sbjct=9icgA Z-score=6.7

back to top

```
DSSP  ------------------------------------------------------------
Query ------------------------------------------------------------    0
ident
Sbjct etlnggitdmltelanfeknvsqaihkynayrkaasviakyphkiksgaeakklpgvgtk   60
DSSP  llllhhhhhhhhhhhhhhhhllllhhhhhhhhhhhhhhhhhllllllhhhhlllllllhh


DSSP  -------------------------------------------------llllllllllh
Query -------------------------------------------------aapyssppptnl   11
ident
Sbjct iaekideflatgklrklekirqddtsssinfltrvsgigpsaarkfvdegiktledlrkn  120
DSSP  hhhhhhhhhhllllhhhhhhhhlhhhhhhhhhllllllhhhhhhhhllllllhhhhhhl


DSSP  hhhhhhhhhhhhllllhhhhhHHHHHHHHHHHHH-LLLLllLLLLEEEEHHHHHHHhhl
Query rslrdrltqvaerqgvvfgrlqRHVAXIVVAQFAA-TLTDdtGAPLLLVKGGSSLELrrg   70
ident                                                 | |
Sbjct edklnhhqriglkyfgdfekriPREEMLQMQDIVLnEVKKvdSEYIATVCGSFRRGA---  177
DSSP  hhhllhhhhhhhllhhhhhlllLHHHHHHHHHHHHhHHHHhlLLLEEEELHHHHLLL---


DSSP  lllllLLLLEEEEEL------------lLHHHHHHHHHHHHHhhllllleeEEELLLEELll
Query ipdsrTSKDFATVAR------------rDIELIHEQLADAGetgwegftAIFTAPEEIaa  118
ident       | |                       |||           |
Sbjct ----eSSGDMDVLLThpsftsestkqpkLLHQVVEQLQKVH--------FITDTLSKG--  223
DSSP  ----lLLLLEEEEEEellllllllllhHHHHHHHHHHHHLL--------LEEEEEEEL--


DSSP  llllLLLEEEEEEEEELL--EEEEEEEEEEEEL-LLLLllllleeeeelhhhhlllllllee
Query pgxpVKPRRFTAKLSYRG--RAFATVPIEVSSV-EAGNadqfdtltsdalglvgvpaava  175
ident              |              |
Sbjct ----ETKFMGVCQLPSKNdeKEYPHRRIDIRLIpKDQY----------------------  257
DSSP  ----LLEEEEEEELLLLLLllLLLLLLEEEEEEEEEeHHHH----------------------
```

```
DSSP  eeellhHHHHhhhhhhhhllllllllllllhhhhHHHHHH--------------------
Query vpcxtiPWQIaqklhavtavleepkandrahdlvDLQLLE--------------------  215
ident
Sbjct -----yCGVL--------------------yftgSDIFNKnmrahalekgftineytirp  292
DSSP  -----hHHHH--------------------hhllLHHHHHhhhhhhhhllllllllllll

DSSP  -hhllhhhhhhhHHHHHhhhhhllllLLLLLllllhhhhhhhhhllllllllllllhhh
Query -gllldadlxptRSACIAifearaqhpWPPRVatlphwpliyagalegldhlelartvda  274
ident
Sbjct lgvtgvageplpVDSEKD------ifdYIQWK----------------------------  318
DSSP  lllllllllllLLLHHH------hhhHLLLL----------------------------

DSSP  hhhhhhhhhhhhhhllll
Query aaqavqrfvaridratkr  292
ident
Sbjct ---------yrepkdrse  327
DSSP  ---------lllhhhlll
```

No 240: Query=mol1A Sbjct=3c2kA Z-score=6.7

back to top

```
DSSP  ------------------------------------------------------------
Query ------------------------------------------------------------    0
ident
Sbjct tlnggitdmltelanfeknvsqaihkynayrkaasviakyphkiksgaeakklpgvgtki   60
DSSP  lllhhhhhhhhhhhhhhhhllllhhhhhhhhhhhhhhhhlllllllhhhhhllllllhhh

DSSP  ------------------------------------------------llllllllllhh
Query ------------------------------------------------aapysspptnlr   12
ident
Sbjct aekideflatgklrklekirqddtsssinfltrvsgigpsaarkfvdegiktledlrkne  120
DSSP  hhhhhhhhhhlllhhhhhhlllhhhhhhhhhllllllhhhhhhhhhlllllhhhhhhlh

DSSP  hhhhhhhhhhhhllllhhhhhHHHHHHHHHHHHHH-LLLLllLLLLEEEEHHHHHHHhhll
Query slrdrltqvaerqgvvfgrlqRHVAXIVVAQFAA-TLTDdtGAPLLLVKGGSSLELrrgi   71
ident                                                | |
Sbjct dklnhhqriglkyfgdfekriPREEMLQMQDIVLnEVKKvdSEYIATVCGSFRRGA----  176
DSSP  hhllhhhhhhhhhhhhhhhlleEHHHHHHHHHHHHHhHHHHhlLLLEEEELHHHHLLL----

DSSP  llllLLLLEEEEEL------------lLHHHHHHHHHHHHhhlllleeEEELLLEELlll
Query pdsrTSKDFATVAR------------rDIELIHEQLADAGetgwegftAIFTAPEEIaap  119
ident      | |                         |||             |
Sbjct ---eSSGDMDVLLThpsftsestkqpkLLHQVVEQLQKVH--------FITDTLSKG---  222
DSSP  ---lEELLEEEEEEllleelleellllHHHHHHHHHHHHLL--------LEEEEEEEL---

DSSP  lllLLLEEEEEEEEEEL--LEEEEEEEEEEEEEL-LLLLlllleeeeelhhhhllllllleee
Query gxpVKPRRFTAKLSYR--GRAFATVPIEVSSV-EAGNadqfdtltsdalglvgvpaavav  176
ident             |             |
Sbjct ---ETKFMGVCQLPSKndEKEYPHRRIDIRLIpKDQY-----------------------  256
DSSP  ---LLEEEEEEELLLLLllLLLLLLEEEEEEEELlHHHH-----------------------

DSSP  eellhHHHHhhhhhhhhllllllllllllhhhhHHHHHHHHHL------------------
Query pcxtiPWQIaqklhavtavleepkandrahdlvDLQLLEGLL------------------  218
ident
Sbjct ----yCGVL--------------------yftgSDIFNKNMRahalekgftineytirpl  292
DSSP  ----hHHHH--------------------hhhlLHHHHHHHHhhhhhhleeellllleeel

DSSP  ---lhhhhhhhHHHHHhhhhhhllllLLLLLllllhhhhhhhhhllllllllllllhhhh
Query ---ldadlxptRSACIaifearaqhpWPPRVatlphwpliyagalegldhlelartvdaa  275
ident
Sbjct gvtgvageplpVDSEK-------difDYIQW-----------------------------  316
DSSP  lllllllllllLLLHH-------hhhHHLLL-----------------------------

DSSP  hhhhhhhhhhhhhllll
Query aqavqrfvaridratkr  292
ident
```

```
DSSP  -------llllhhhlll
```

No 241: Query=mol1A Sbjct=4r65A Z-score=6.7

back to top

```
DSSP  ------------------------------------------------------------
Query ------------------------------------------------------------    0
ident 
Sbjct tlnggitdmltelanfeknvsqaihkynayrkaasviakyphkiksgaeakklpgvgtki   60
DSSP  lllhhhhhhhhhhhhhhhhhllllhhhhhhhhhhhhhhhhllllllhhhhlllllllhhh


DSSP  ------------------------------------------------llllllllllhh
Query ------------------------------------------------aapysspptnlr   12
ident 
Sbjct aekideflatgklrklekirqddtsssinfltrvsgigpsaarkfvdegiktledlrkne  120
DSSP  hhhhhhhhhhlllhhhhhhhhlhhhhhhhhhllllllhhhhhhhhhhlllllhhhhhhlh


DSSP  hhhhhhhhhhhhllllhhhhhHHHHHHHHHHHHH-LLLLLLLLLLEEEEHHHHHHHhhll
Query slrdrltqvaerqgvvfgrlqRHVAXIVVAQFAA-TLTDDTGAPLLLVKGGSSLELrrgi   71
ident                                                | |
Sbjct dklnhhqriglkyfgdfekriPREEMLQMQDIVLnEVKKVDSEYIATVCGSFRRGA----  176
DSSP  hhllhhhhhhhhlhhhhhlleEHHHHHHHHHHHHhHHHHHLLLLEEEELHHHHLLL----


DSSP  llllLLLLEEEEEL------------lLHHHHHHHHHHHHhhlllleeEEELLLEEllll
Query pdsrTSKDFATVAR------------rDIELIHEQLADAGetgwegftAIFTAPEEiaap  119
ident      | |                         |||             |
Sbjct ---eSSGDMDVLLThpsftsestkqpkLLHQVVEQLQKVH--------FITDTLSK----  221
DSSP  ---lEELLEEEEEEllleelleellllHHHHHHHHHHHLL--------LEEEEEEE----


DSSP  lllLLLEEEEEEEEEL--LEEEEEEEEEEEEL-LLLLlllleeeeelhhhhlllllleee
Query gxpVKPRRFTAKLSYR--GRAFATVPIEVSSV-EAGNadqfdtltsdalglvgvpaavav  176
ident             |             |
Sbjct --gETKFMGVCQLPSKndEKEYPHRRIDIALIpKDQY-----------------------  256
DSSP  --lLLEEEEEEELLLLllLLLLLLEEEEEEELlHHHH-----------------------


DSSP  eellhHHHHhhhhhhhhhlllllllllllHHHH-------------------hhhhhhhh
Query pcxtiPWQIaqklhavtavleepkandraHDLV-------------------dlqllegl  217
ident                               |
Sbjct ----yCGVL----------------yftgSDIFnknmrahalekgftineytirplgvtg  296
DSSP  ----hHHHH----------------hhhlLHHHhhhhhhhhhhhleeellllleeelllll


DSSP  llhHHHHhhHHHHHHhhhhhlllLLLLLLllllllhhhhhhhhhlllllllllllhhhhhh
Query lldADLXptRSACIAifearaqhPWPPRVatlphwpliyagalegldhlelartvdaaaq  277
ident      |
Sbjct vagEPLP--VDSEKD-----ifdYIQWKY-------------------------------  318
DSSP  lllLLLL--LLLHHH-----hhhHLLLLL-------------------------------


DSSP  hhhhhhhhhhhllll
Query avqrfvaridratkr  292
ident 
Sbjct -------repkdrse  326
DSSP  -------llhhhhll
```

No 242: Query=mol1A Sbjct=2bpgA Z-score=6.7

back to top

```
DSSP  ------------------------------------------------------------
Query ------------------------------------------------------------    0
ident 
Sbjct etlnggitdmlvelanfeknvsqaihkynayrkaasviakyphkiksgaeakklpgvgtk   60
DSSP  lllhhhhhhhhhhhhhhhhhllllhhhhhhhhhhhhhhhhlllllllhhhhhhlllllhh


DSSP  -------------------------------------------------llllllllllh
```

```
ident 
Sbjct iaekideflatgklrklekirqddtsssinfltrvtgigpsaarklvdegiktledlrkn  120
DSSP  hhhhhhhhhhllllhhhhhhhhlhhhhhhhhhhlllllhhhhhhhhlllllhhhhhhl


DSSP  hhhhhhhhhhhhllllhHHHHHHHHHHHHHHHHH-LLLLllLLLLEEEEHHHHHHHhhl
Query rslrdrltqvaerqgvvfGRLQRHVAXIVVAQFAA-TLTDdtGAPLLLVKGGSSLELrrg   70
ident                                                 | |
Sbjct edklnhhqriglkyfedfEKRIPREEMLQMQDIVLnEVKKldPEYIATVCGSFRRGA---  177
DSSP  hhhllhhhhhhhhhllhhHLLEEHHHHHHHHHHHHHhHHHHhlLLLEEEELLLLLLL---


DSSP  lllllLLLLEEEEEL------------lLHHHHHHHHHHHHhhlllleeEEELLLEELll
Query ipdsrTSKDFATVAR------------rDIELIHEQLADAGetgwegftAIFTAPEEIaa  118
ident       | |                         |||             |
Sbjct ----eSSGDMDVLLThpnftsesskqpkLLHRVVEQLQKVR--------FITDTLSKG--  223
DSSP  ----lEELLEEEEEEllllllllllhHHHHHHHHHHHHLL--------LEEEEEELL--


DSSP  llllLLLEEEEEEEEEELLeEEEEEEEEEEEL-LLLLlllleeeeelhhhhlllllleeee
Query pgxpVKPRRFTAKLSYRGrAFATVPIEVSSV-EAGNadqfdtltsdalglvgvpaavavp  177
ident              |           |
Sbjct ----ETKFMGVCQLPSEN-EYPHRRIDIRLIpKDQY------------------------  254
DSSP  ----LLEEEEEEEELLLLL-LLLEEEEEEEEELlHHHH------------------------


DSSP  ellhHHHHhhhhhhhhllllllllllHHHHHHH-------------------hhhhhl
Query cxtiPWQIaqklhavtavleepkandraHDLVDLQ-------------------llegll  218
ident                              |
Sbjct ---yCGVL----------------yftgSDIFNKNmrahalekgftineytirplgvtgv  295
DSSP  ---hHHHH----------------hhhlLLHHHHHhhhhhhhlleeellllleeellllll


DSSP  lhHHHHhhHHHHHhhhhhhllllLLLLLlllllhhhhhhhhhlllllllllllhhhhhhh
Query ldADLXptRSACIaifearaqhpWPPRVatlphwpliyagalegldhlelartvdaaaqa  278
ident     |
Sbjct agEPLP--VDSEQ-------difDYIQW--------------------------------  314
DSSP  llLLLL--LLLHH-------hhhHHLLL--------------------------------


DSSP  hhhhhhhhhhllll
Query vqrfvaridratkr  292
ident 
Sbjct ----ryrepkdrse  324
DSSP  ----llllhhhlll
```

No 243: Query=mol1A Sbjct=4rpxA Z-score=6.7

back to top

```
DSSP  ------------------------------------------------------------
Query ------------------------------------------------------------    0
ident 
Sbjct tlnggitdmltelanfeknvsqaihkynayrkaasviakyphkiksgaeakklpgvgtki   60
DSSP  lllhhhhhhhhhhhhhhhhhllllhhhhhhhhhhhhhhhhllllllllhhhhhlllllhhh


DSSP  ------------------------------------------------lllllllllhh
Query ------------------------------------------------aapyssppptnlr   12
ident 
Sbjct aekideflatgklrklekirqddtsssinfltrvsgigpsaarkfvdegiktledlrkne  120
DSSP  hhhhhhhhhlllhhhhhhhllhhhhhhhhhhlllllhhhhhhhhhlllllhhhhhhlh


DSSP  hhhhhhhhhhhhllllhhhhhHHHHHHHHHHHHHH-LLLLllLLLLEEEEHHHHHHHhhll
Query slrdrltqvaerqgvvfgrlqRHVAXIVVAQFAA-TLTDdtGAPLLLVKGGSSLELrrgi   71
ident                                                | |
Sbjct dklnhhqriglkyfgdfekriPREEMLQMQDIVLnEVKKvdSEYIATVCGSFRRGA----  176
DSSP  hhllhhhhhhhhhlhhhhhlleEHHHHHHHHHHHHHhHHHHhlLLLEEEELHHHHLLL----


DSSP  llllLLLLEEEEEL------------lLHHHHHHHHHHHHhhlllleeEEELLLEEllll
Query pdsrTSKDFATVAR------------rDIELIHEQLADAGetgwegftAIFTAPEEiaap  119
ident      | |                         |||             |
Sbjct ---eSSGDMDVLLThpsftsestkqpkLLHQVVEQLQKVH--------FITDTLSK----  221
DSSP  ---lEELLEEEEEEllllllllllllHHHHHHHHHHHHLL--------LEEEEEEE----
```

```
DSSP  lllLLLEEEEEEEEEL--lEEEEEEEEEEEEL-LLLLllllleeeeelhhhhlllllleee
Query gxpVKPRRFTAKLSYR--gRAFATVPIEVSSV-EAGNadqfdtltsdalglvgvpaavav  176
ident             |             |
Sbjct --gETKFMGVCQLPSKndeKEYPHRRIDIRLIpKDQY-----------------------  256
DSSP  --lLLEEEEEEELLLLLlllLLLLLEEEEEEEELlHHHH-----------------------


DSSP  eellhHHHHhhhhhhhhlllllllllllHHHH-------------------hhhhhhhh
Query pcxtiPWQIaqklhavtavleepkandraHDLV-------------------dlqllegl  217
ident                               |
Sbjct ----yCGVL----------------yftgSDIFnknmrahalekgftineytirplgvtg  296
DSSP  ----hHHHH----------------hhhlLHHHhhhhhhhhhhlleeellllleeellll


DSSP  llhHHHHHhhHHHHhhhhhhlllLLLLLLlllllhhhhhhhhhllllllllllhhhhhh
Query lldADLXPtrSACIaifearaqhPWPPRVatlphwpliyagalegldhlelartvdaaaq  277
ident      |
Sbjct vagEPLPV--DSEK-----difdYIQWKY-------------------------------  318
DSSP  lllLLLLL--LLHH-----hhhhHLLLLL-------------------------------


DSSP  hhhhhhhhhhhllll
Query avqrfvaridratkr  292
ident
Sbjct -------repkdrse  326
DSSP  -------llhhhlll
```

No 244: Query=mol1A Sbjct=8icqA Z-score=6.7

back to top

```
DSSP  ------------------------------------------------------------
Query ------------------------------------------------------------    0
ident
Sbjct etlnggitdmltelanfeknvsqaihkynayrkaasviakyphkiksgaeakklpgvgtk   60
DSSP  llllhhhhhhhhhhhhhhhlllllhhhhhhhhhhhhhhlllllllllhhhhhllllllhh


DSSP  -------------------------------------------------llllllllllh
Query -------------------------------------------------aapysspptnl   11
ident
Sbjct iaekideflatgklrklekirqddtsssinfltrvsgigpsaarkfvdegiktledlrkn  120
DSSP  hhhhhhhhhhllllhhhhhhhhlhhhhhhhhhlllllllhhhhhhhhhhllllhhhhhhl


DSSP  hhhhhhhhhhhhhllllhhhhhHHHHHHHHHHHHHH-LLLLllLLLLEEEEHHHHHHHhhl
Query rslrdrltqvaerqgvvfgrlqRHVAXIVVAQFAA-TLTDdtGAPLLLVKGGSSLELrrg   70
ident                                                 | |
Sbjct edklnhhqriglkyfgdfekriPREEMLQMQDIVLnEVKKvdSEYIATVCGSFRRGA---  177
DSSP  hhhllhhhhhhhhlhhhhhlleEHHHHHHHHHHHHHhHHHHhlLLLEEEELLLLLLL---


DSSP  lllllLLLLEEEEEEL------------lLHHHHHHHHHHHHHhhllllleeEEELLLEELll
Query ipdsrTSKDFATVAR------------rDIELIHEQLADAGetgwegftAIFTAPEEIaa  118
ident       | |                         |||             |
Sbjct ----eSSGDMDVLLThpsftsestkqpkLLHQVVEQLQKVH--------FITDTLSKG--  223
DSSP  ----lEELLEEEEEEelllllllllllhHHHHHHHHHHHHHLL--------LEEEEEEEL--


DSSP  llllLLLEEEEEEEEELL--EEEEEEEEEEEEL-LLLLllllleeeeelhhhhlllllllee
Query pgxpVKPRRFTAKLSYRG--RAFATVPIEVSSV-EAGNadqfdtltsdalglvgvpaava  175
ident              |             |
Sbjct ----ETKFMGVCQLPSKNdeKEYPHRRIDIRLIpKDQY----------------------  257
DSSP  ----LLEEEEEEELLLLLllLLLLLEEEEEEEEEeHHHH----------------------


DSSP  eeellhHHHHhhhhhhhhlllllllllllhhhhHHHHHH--------------------
Query vpcxtiPWQIaqklhavtavleepkandrahdlvDLQLLE--------------------  215
ident
Sbjct -----yCGVL--------------------yftgSDIFNKnmrahalekgftineytirp  292
DSSP  -----hHHHH--------------------hhhlLHHHHHhhhhhhhhhlleeellllleee


DSSP  -hhllhhhhhhhHHHHHhhhhhhllllLLLLLlllllhhhhhhhhhllllllllllhhh
Query -glllldadlxptRSACIaifearaqhpWPPRVatlphwpliyagalegldhlelartvda  274
```

```
Sbjct lgvtgvageplpVDSEK-------difDYIQW----------------------------  317
DSSP  llllllllllllLLLHH-------hhhHHLLL----------------------------


DSSP  hhhhhhhhhhhhhhllll
Query aaqavqrfvaridratkr  292
ident
Sbjct --------kyrepkdrse  327
DSSP  --------llllhhhlll
```

No 245: Query=mol1A Sbjct=1zqvA Z-score=6.7

back to top

```
DSSP  --------------------------lllllllllhhhhhhhhhhhhhhllllhhhhh
Query --------------------------aapyssspptnlrslrdrltqvaerqgvvfgrlq   33
ident
Sbjct ddtsssinfltrvtgigpsaarklvdegiktledlrknedklnhhqriglkyfedfekri   60
DSSP  lllhhhhhhhlllllllhhhhhhhhhlllllhhhhhhllhhhllhhhhhhhhhlllhhhlle


DSSP  HHHHHHHHHHHHHLL-LLLLLLLLEEEEHHHHHHHhhllllllLLLLEEEEEL-------
Query RHVAXIVVAQFAATL-TDDTGAPLLLVKGGSSLELrrgipdsrTSKDFATVAR-------   85
ident                           | |               | |
Sbjct PREEMLQMQDIVLNEvKKLDPEYIATVCGSFRRGA-------eSSGDMDVLLThpnftse  113
DSSP  EHHHHHHHHHHHHHHhHHHLLLLEEEEELLHHHHLL-------lEELLEEEEEElllllll


DSSP  -----lLHHHHHHHHHHHHhhlllleeEEELLLEELllllllLLLEEEEEEEEEELLeEEE
Query -----rDIELIHEQLADAGetgwegftAIFTAPEEIaapgxpVKPRRFTAKLSYRGrAFA  140
ident             |||             |                      |
Sbjct sskqpkLLHRVVEQLQKVR--------FITDTLSKG------ETKFMGVCQLPSEN-EYP  158
DSSP  llllhhHHHHHHHHHHHLL--------LEEEEEEEL------LLLEEEEEELLLLLL-LLL


DSSP  EEEEEEEEL-LLLLllleeeeelhhhhllllllleeeeellhHHHHhhhhhhhhllllll
Query TVPIEVSSV-EAGNadqfdtltsdalglvgvpaavavpcxtiPWQIaqklhavtavleep  199
ident    |
Sbjct HRRIDIRLIpKDQY---------------------------yCGVL--------------  177
DSSP  LLLEEEEELlLLLH---------------------------hHHHH--------------


DSSP  llllllhhhhHHHHHH----------------------hhllhhhhhhhhHHHHhhhhhh
Query kandrahdlvDLQLLE----------------------gllldadlxptrSACIaifear  237
ident                                                   |
Sbjct ------yftgSDIFNKnmrahalekgftineytirplgvtgvageplpvdSEQD------  225
DSSP  ------hhhlLHHHHHhhhhhhhhlleeellllleeellllllllllllLHHH------


DSSP  llllLLLLLlllllhhhhhhhhhlllllllllllhhhhhhhhhhhhhhhhhllll
Query aqhpWPPRVatlphwpliyagalegldhlelartvdaaaqavqrfvaridratkr  292
ident
Sbjct -ifdYIQWR------------------------------------yrepkdrse  242
DSSP  -hhhHLLLL------------------------------------lllllllll
```

No 246: Query=mol1A Sbjct=8icrA Z-score=6.7

back to top

```
DSSP  ------------------------------------------------------------
Query ------------------------------------------------------------    0
ident
Sbjct etlnggitdmltelanfeknvsqaihkynayrkaasviakyphkiksgaeakklpgvgtk   60
DSSP  llllhhhhhhhhhhhhhhhllllhhhhhhhhhhhhhhhhlllllllhhhhhlllllllhh


DSSP  --------------------------------------------------llllllllllh
Query --------------------------------------------------aapyssspptnl   11
ident
Sbjct iaekideflatgklrklekirqddtsssinfltrvsgigpsaarkfvdegiktledlrkn  120
DSSP  hhhhhhhhhhllllhhhhhhhhlhhhhhhhhhllllllllhhhhhhhhhlllllhhhhhhl
```

```
Query rslrdrltqvaerqgvvfgrlqRHVAXIVVAQFAA-TLTDdtGAPLLLVKGGSSLELrrg   70
ident                                                 | |
Sbjct edklnhhqriglkyfgdfekriPREEMLQMQDIVLnEVKKvdSEYIATVCGSFRRGA---  177
DSSP  hhhllhhhhhhhhlhhhhhlleEHHHHHHHHHHHHhHHHHhlLLLEEEELHHHHLLL---


DSSP  lllllLLLLEEEEEL------------lLHHHHHHHHHHHHhhlllleeEEELLLEELll
Query ipdsrTSKDFATVAR------------rDIELIHEQLADAGetgwegftAIFTAPEEIaa  118
ident       | |                          |||             |
Sbjct ----eSSGDMDVLLThpsftsestkqpkLLHQVVEQLQKVH--------FITDTLSKG--  223
DSSP  ----lEELLEEEEEEellllllllllllhHHHHHHHHHHLL--------LEEEEEEEL--


DSSP  llllLLLEEEEEEEEELL--EEEEEEEEEEEEL-LLLLllllleeeeelhhhhllllllee
Query pgxpVKPRRFTAKLSYRG--RAFATVPIEVSSV-EAGNadqfdtltsdalglvgvpaava  175
ident              |             |
Sbjct ----ETKFMGVCQLPSKNdeKEYPHRRIDIRLIpKDQY----------------------  257
DSSP  ----LLEEEEEEELLLLLllLLLLLEEEEEEEEeHHHH----------------------


DSSP  eeellhHHHHhhhhhhhhllllllllllllhhhhHHHHHH--------------------
Query vpcxtiPWQIaqklhavtavleepkandrahdlvDLQLLE--------------------  215
ident
Sbjct -----yCGVL--------------------yftgSDIFNKnmrahalekgftineytirp  292
DSSP  -----hHHHH--------------------hhllLHHHHHhhhhhhhhlllleelleell


DSSP  --hhllhhhhhhhhHHHHhhhhhhllllLLLLLlllllhhhhhhhhhllllllllllllhh
Query --gllldadlxptrSACIaifearaqhpWPPRVatlphwpliyagalegldhlelartvd  273
ident               |
Sbjct lgvtgvageplpvdSEKD-------ifdYIQWK---------------------------  318
DSSP  llllllllllllllLHHH-------hhhHLLLL---------------------------


DSSP  hhhhhhhhhhhhhhhllll
Query aaaqavqrfvaridratkr  292
ident
Sbjct ----------yrepkdrse  327
DSSP  ----------lllhhhlll
```

No 247: Query=mol1A Sbjct=8ickA Z-score=6.7

back to top

```
DSSP  ------------------------------------------------------------
Query ------------------------------------------------------------    0
ident
Sbjct etlnggitdmltelanfeknvsqaihkynayrkaasviakyphkiksgaeakklpgvgtk   60
DSSP  llllhhhhhhhhhhhhhhhhhlllhhhhhhhhhhhhhhhhhllllllhhhhhllllllhh


DSSP  -------------------------------------------------lllllllllh
Query -------------------------------------------------aapysspptnl   11
ident
Sbjct iaekideflatgklrklekirqddtsssinfltrvsgigpsaarkfvdegiktledlrkn  120
DSSP  hhhhhhhhhhllllhhhhhhhhlhhhhhhhhhllllllhhhhhhhhhhllllhhhhhhl


DSSP  hhhhhhhhhhhhhllllhhhhhHHHHHHHHHHHHH-LLLLllLLLLEEEEHHHHHHHhhl
Query rslrdrltqvaerqgvvfgrlqRHVAXIVVAQFAA-TLTDdtGAPLLLVKGGSSLELrrg   70
ident                                                 | |
Sbjct edklnhhqriglkyfgdfekriPREEMLQMQDIVLnEVKKvdSEYIATVCGSFRRGA---  177
DSSP  hhhllhhhhhhhhlhhhhhlleEHHHHHHHHHHHHhHHHHhlLLLEEEELLLLLLLL---


DSSP  lllllLLLLEEEEEL------------lLHHHHHHHHHHHHhhlllleeEEELLLEELll
Query ipdsrTSKDFATVAR------------rDIELIHEQLADAGetgwegftAIFTAPEEIaa  118
ident       | |                          |||             |
Sbjct ----eSSGDMDVLLThpsftsestkqpkLLHQVVEQLQKVH--------FITDTLSKG--  223
DSSP  ----lEELLEEEEEEellllllllllllhHHHHHHHHHHLL--------LEEEEEEEL--


DSSP  llllLLLEEEEEEEEELL--EEEEEEEEEEEEL-LLLLllllleeeeelhhhhllllllee
Query pgxpVKPRRFTAKLSYRG--RAFATVPIEVSSV-EAGNadqfdtltsdalglvgvpaava  175
ident              |             |
Sbjct ----ETKFMGVCQLPSKNdeKEYPHRRIDIRLIpKDQY----------------------  257
```

```
DSSP  eeellhHHHHhhhhhhhhhlllllllllllhhhhHHHHHH--------------------
Query vpcxtiPWQIaqklhavtavleepkandrahdlvDLQLLE--------------------  215
ident
Sbjct -----yCGVL--------------------yftgSDIFNKnmrahalekgftineytirp  292
DSSP  -----hHHHH--------------------hhhlLHHHHHhhhhhhhhlleeeelleeee


DSSP  -hhllhhhhhhhHHHHHHhhhhhllllLLLLLlllllhhhhhhhhhlllllllllllhhh
Query -gllldadlxptRSACIAifearaqhpWPPRVatlphwpliyagalegldhlelartvda  274
ident
Sbjct lgvtgvageplpVDSEKD------ifdYIQWK----------------------------  318
DSSP  llllllllllllLLLHHH------hhhHLLLL----------------------------


DSSP  hhhhhhhhhhhhhllll
Query aaqavqrfvaridratkr  292
ident
Sbjct ---------yrepkdrse  327
DSSP  ---------lllhhhlll
```

No 248: Query=mol1A Sbjct=4nlkA Z-score=6.7

back to top

```
DSSP  ------------------------------------------------------------
Query ------------------------------------------------------------    0
ident
Sbjct tlnggitdmltelanfeknvsqaihkynayrkaasviakyphkiksgaeakklpgvgtki   60
DSSP  lllhhhhhhhhhhhhhhhhhllllhhhhhhhhhhhhhhhlllllllhhhhhlllllllhhh


DSSP  ------------------------------------------------llllllllllhh
Query ------------------------------------------------aapyssppptnlr   12
ident
Sbjct aekideflatgklrklekirqddtsssinfltrvsgigpsaarkfvdegiktledlrkne  120
DSSP  hhhhhhhhhlllhhhhhhhhlhhhhhhhhhhlllllhhhhhhhhhlllllhhhhhhllh


DSSP  hhhhhhhhhhhllllhHHHHHHHHHHHHHHHHHH-LLLLllLLLLEEEEHHHHHHHHhhll
Query slrdrltqvaerqgvvfGRLQRHVAXIVVAQFAA-TLTDdtGAPLLLVKGGSSLELrrgi   71
ident                                               | |
Sbjct dklnhhqriglkyfgdfEKRIPREEMLQMQDIVLnEVKKvdSEYIATVCGSFRRGA----  176
DSSP  hhllhhhhhhhhlhhhhLLLEEHHHHHHHHHHHHHhHHHHhlLLLEEEELHHHHLLL----


DSSP  llllLLLLEEEEEL------------lLHHHHHHHHHHHHhhlllleeEEELLLEELlll
Query pdsrTSKDFATVAR------------rDIELIHEQLADAGetgwegftAIFTAPEEIaap  119
ident       | |                         |||              |
Sbjct ---eSSGDMDVLLThpsftsestkqpkLLHQVVEQLQKVH--------FITDTLSKG---  222
DSSP  ---lEELLEEEEEllllllllllllLLHHHHHHHHHLL--------LEEEEEEEL---


DSSP  lllLLLEEEEEEEEEEL--LEEEEEEEEEEEEL-LLLLlllleeeeelhhhhllllllleee
Query gxpVKPRRFTAKLSYR--GRAFATVPIEVSSV-EAGNadqfdtltsdalglvgvpaavav  176
ident              |             |
Sbjct ---ETKFMGVCQLPSKndEKEYPHRRIDIRLIpKDQY-----------------------  256
DSSP  ---LLEEEEEELLLLLllLLLLLLEEEEEEELlHHHH-----------------------


DSSP  eellhHHHHhhhhhhhhlllllllllllHHHHHHH------------------hhhhh
Query pcxtiPWQIaqklhavtavleepkandraHDLVDLQ------------------llegl  217
ident                                 |
Sbjct ----yCGVL----------------yftgSDIFNKNmrahalekgftineytirplgvtg  296
DSSP  ----hHHHH----------------hhhlLHHHHHHhhhhhhhlleeellllleeelllll


DSSP  llhHHHHhhHHHHHhhhhhhllllLLLLLLllllhhhhhhhhhlllllllllllhhhhhh
Query lldADLXptRSACIaifearaqhpWPPRVAtlphwpliyagalegldhlelartvdaaaq  277
ident       |
Sbjct vagEPLP--VDSEK-------difDYIQWK------------------------------  317
DSSP  lllLLLL--LLLHH-------hhhHHLLLL------------------------------


DSSP  hhhhhhhhhhhllll
```

```
ident
Sbjct ------yrepkdrse  326
DSSP  ------lllhhhlll
```

No 249: Query=mol1A Sbjct=3v72A Z-score=6.7

back to top

```
DSSP  ------------------------------------------------------------
Query ------------------------------------------------------------    0
ident
Sbjct etlnggitdmlvelanfeknvsqaihkynayrkaasviakyphkiksgaeakklpgvgtk   60
DSSP  llllhhhhhhhhhhhhhhhhllllhhhhhhhhhhhhhhhllllllllhhhhhllllllhh


DSSP  -------------------------------------------------llllllllllh
Query -------------------------------------------------aapysspptnl   11
ident
Sbjct iaekideflatgklrklekirqddtsssinfltrvtgigpsaarklvdegiktledlrkn  120
DSSP  hhhhhhhhhhhlllhhhhhhhhlhhhhhhhhhllllllhhhhhhhhhhllllhhhhhll


DSSP  hhhhhhhhhhhhllllhHHHHHHHHHHHHHHHHHHL-LLLLLLLLLEEEEHHHHHHHhhl
Query rslrdrltqvaerqgvvfGRLQRHVAXIVVAQFAAT-LTDDTGAPLLLVKGGSSLELrrg   70
ident                                                 | |
Sbjct edklnhhqriglkyfedfEKRIPREEMLQMQDIVLNeVKKLDPEYIATVCGSFRRGA---  177
DSSP  hhhllhhhhhhhhlhhhhHLLEEHHHHHHHHHHHHHHhHHHHLLLLEEEELLHHHHLL---


DSSP  lllllLLLLEEEEEEL------------lLHHHHHHHHHHHHHhhlllleeEEELLLEELll
Query ipdsrTSKDFATVAR------------rDIELIHEQLADAGetgwegftAIFTAPEEIaa  118
ident       | |                         |||             |
Sbjct ----eSSGDMDVLLThpnftsesskqpkLLHRVVEQLQKVR--------FITDTLSKG--  223
DSSP  ----lEELLEEEEEEllllllllllllhHHHHHHHHHHHHLL--------LEEEEEEEL--


DSSP  llllLLLEEEEEEEEELL--EEEEEEEEEEEL-LLLLllllleeeeelhhhhlllllllee
Query pgxpVKPRRFTAKLSYRG--RAFATVPIEVSSV-EAGNadqfdtltsdalglvgvpaava  175
ident              |             |
Sbjct ----ETKFMGVCQLPSENdeNEYPHRRIDIRLIpKDQY----------------------  257
DSSP  ----LLEEEEEEELLLLLllLLLLLLEEEEEEEELlHHHH----------------------


DSSP  eeellhHHHHhhhhhhhhllllllllllllhhhhHHHHHH--------------------
Query vpcxtiPWQIaqklhavtavleepkandrahdlvDLQLLE--------------------  215
ident
Sbjct -----yCGVL--------------------yftgSDIFNKnmrahalekgftinkytirp  292
DSSP  -----hHHHH--------------------hlllLHHHHHhhhhhhhhlleeellllleee


DSSP  -hhllhhhhhhhhhHHHHhhhhhlllLLLLLllllllhhhhhhhhhllllllllllhhh
Query -glllldadlxptrsACIAifearaqhPWPPRvatlphwpliyagalegldhlelartvda  274
ident                              |
Sbjct lgvtgvageplpvdSEQD-----ifdYIQWR-----------------------------  318
DSSP  llllllllllllllLHHH-----hhhHLLLL-----------------------------


DSSP  hhhhhhhhhhhhhhllll
Query aaqavqrfvaridratkr  292
ident
Sbjct ---------yrepkdrse  327
DSSP  ---------lllhhhhll
```

No 250: Query=mol1A Sbjct=4nm1A Z-score=6.7

back to top

```
DSSP  ------------------------------------------------------------
Query ------------------------------------------------------------    0
ident
Sbjct tlnggitdmltelanfeknvsqaihkynayrkaasviakyphkiksgaeakklpgvgtki   60
DSSP  lllhhhhhhhhhhhhhhhhllllhhhhhhhhhhhhhhhllllllllhhhhlllllllhhh
```

```
DSSP  ------------------------------------------------llllllllllhh
Query ------------------------------------------------aapysspptnlr   12
ident 
Sbjct aekideflatgklrklekirqddtsssinfltrvsgigpsaarkfvdegiktledlrkne  120
DSSP  hhhhhhhhhlllhhhhhhhhlhhhhhhhhhllllllhhhhhhhhlllllhhhhhhlh


DSSP  hhhhhhhhhhhhllllhHHHHHHHHHHHHHHHHHHL-LLLLLLLLLEEEEHHHHHHHHhhll
Query slrdrltqvaerqgvvfGRLQRHVAXIVVAQFAAT-LTDDTGAPLLLVKGGSSLELrrgi   71
ident                                                | |
Sbjct dklnhhqriglkyfgdfEKRIPREEMLQMQDIVLNeVKKVDSEYIATVCGSFRRGA----  176
DSSP  hhllhhhhhhhhlhhhhLLLEEHHHHHHHHHHHHHHhHHHHLLLLEEEELHHHHLLL----


DSSP  llllLLLLEEEEEL------------lLHHHHHHHHHHHHhhlllleeEEELLLEELlll
Query pdsrTSKDFATVAR------------rDIELIHEQLADAGetgwegftAIFTAPEEIaap  119
ident      | |                         |||             |
Sbjct ---eSSGDMDVLLThpsftsestkqpkLLHQVVEQLQKVH--------FITDTLSKG---  222
DSSP  ---lEELLEEEEEEllllllllllllHHHHHHHHHHHLL--------LEEEEEEEL---


DSSP  lllLLLEEEEEEEEEL--LEEEEEEEEEEEEL-LLLLllllleeeeelhhhhllllllleee
Query gxpVKPRRFTAKLSYR--GRAFATVPIEVSSV-EAGNadqfdtltsdalglvgvpaavav  176
ident             |             |
Sbjct ---ETKFMGVCQLPSKndEKEYPHRRIDIRLIpKDQY-----------------------  256
DSSP  ---LLEEEEEELLLLLllLLLLLLEEEEEEELlHHHH-----------------------


DSSP  eellhHHHHhhhhhhhhllllllllllllHHHHHHH-------------------hhhhh
Query pcxtiPWQIaqklhavtavleepkandraHDLVDLQ-------------------llegl  217
ident                               |
Sbjct ----yCGVL----------------yftgSDIFNKNmrahalekgftineytirplgvtg  296
DSSP  ----hHHHH----------------hhhlLHHHHHHhhhhhhhlleeellllleeellll


DSSP  llhHHHHhhHHHHHhhhhhhllllLLLLLLllllhhhhhhhhhllllllllllhhhhhh
Query lldADLXptRSACIaifearaqhpWPPRVAtlphwpliyagalegldhlelartvdaaaq  277
ident      |
Sbjct vagEPLP--VDSEK-------difDYIQWK------------------------------  317
DSSP  lllLLLL--LLLHH-------hhhHHLLLL------------------------------


DSSP  hhhhhhhhhhhllll
Query avqrfvaridratkr  292
ident 
Sbjct ------yrepkdrse  326
DSSP  ------lllhhhlll
```

No 251: Query=mol1A Sbjct=4nxzA Z-score=6.7

back to top

```
DSSP  ------------------------------------------------------------
Query ------------------------------------------------------------    0
ident 
Sbjct tlnggitdmltelanfeknvsqaihkynayrkaasviakyphkiksgaeakklpgvgtki   60
DSSP  lllhhhhhhhhhhhhhhhhllllhhhhhhhhhhhhhhhllllllllhhhhhhlllllhhh


DSSP  ------------------------------------------------llllllllllhh
Query ------------------------------------------------aapysspptnlr   12
ident 
Sbjct aekideflatgklrklekirqddtsssinfltrvsgigpsaarkfvdegiktledlrkne  120
DSSP  hhhhhhhhhlllhhhhhhhhlhhhhhhhhhllllllhhhhhhhhlllllhhhhhhlh


DSSP  hhhhhhhhhhhhllllhHHHHHHHHHHHHHHHHHHL-LLLLlLLLLEEEEHHHHHHHHhhll
Query slrdrltqvaerqgvvfGRLQRHVAXIVVAQFAAT-LTDDtGAPLLLVKGGSSLELrrgi   71
ident                                                | |
Sbjct dklnhhqriglkyfgdfEKRIPREEMLQMQDIVLNeVKKVdSEYIATVCGSFRRGA----  176
DSSP  hhllhhhhhhhhlllhhHLLEEHHHHHHHHHHHHHHhHHLLlLLLEEEELHHHHLLL----


DSSP  llllLLLLEEEEEL------------lLHHHHHHHHHHHHhhlllleeEEELLLEELlll
Query pdsrTSKDFATVAR------------rDIELIHEQLADAGetgwegftAIFTAPEEIaap  119
ident      | |                         |||             |
```

```
DSSP  ---lEELLEEEEEEllllllllllllHHHHHHHHHHHHLL--------LEEEEEEEL---


DSSP  lllLLLEEEEEEEEEL--LEEEEEEEEEEEEL-LLLLllllleeeeelhhhhlllllleee
Query gxpVKPRRFTAKLSYR--GRAFATVPIEVSSV-EAGNadqfdtltsdalglvgvpaavav  176
ident             |             |
Sbjct ---ETKFMGVCQLPSKndEKEYPHRRIDIRLIpKDQY-----------------------  256
DSSP  ---LLEEEEEEELLLLLllLLLLLLLEEEEEEEELlHHHH-----------------------


DSSP  eellhHHHHhhhhhhhllllllllllllHHHHHHH------------------hhhhh
Query pcxtiPWQIaqklhavtavleepkandraHDLVDLQ------------------llegl  217
ident                               |
Sbjct ----yCGVL----------------yftgSDIFNKNmrahalekgftineytirplgvtg  296
DSSP  ----hHHHH----------------hhhlLHHHHHHhhhhhhhhleeellllleeelllll


DSSP  llhHHHHhhHHHHHhhhhhhllllLLLLLLllllhhhhhhhhhllllllllllhhhhhh
Query lldADLXptRSACIaifearaqhpWPPRVAtlphwpliyagalegldhlelartvdaaaq  277
ident      |
Sbjct vagEPLP--VDSEK-------difDYIQWK------------------------------  317
DSSP  lllLLLL--LLLHH-------hhhHHLLLL------------------------------


DSSP  hhhhhhhhhhhllll
Query avqrfvaridratkr  292
ident
Sbjct ------yrepkdrse  326
DSSP  ------lllhhhhll
```

No 252: Query=mol1A Sbjct=4uayA Z-score=6.7

back to top

```
DSSP  ------------------------------------------------------------
Query ------------------------------------------------------------    0
ident
Sbjct tlnggitdmltelanfeknvsqaihkynayrkaasviakyphkiksgaeakklpgvgtki   60
DSSP  lllhhhhhhhhhhhhhhhhllllhhhhhhhhhhhhhhhllllllllhhhhhlllllllhhh


DSSP  ------------------------------------------------llllllllllhh
Query ------------------------------------------------aapysspptnlr   12
ident
Sbjct aekideflatgklrklekirqddtsssinfltrvsgigpsaarkfvdegiktledlrkne  120
DSSP  hhhhhhhhhhlllhhhhhhhhlhhhhhhhhhlllllllhhhhhhhhhllllllhhhhhhhlh


DSSP  hhhhhhhhhhhhllllhhhhhHHHHHHHHHHHHHH-LLLLllLLLLLEEEEHHHHHHHHhhll
Query slrdrltqvaerqgvvfgrlqRHVAXIVVAQFAA-TLTDdtGAPLLLVKGGSSLELrrgi   71
ident                                                | |
Sbjct dklnhhqriglkyfgdfekriPREEMLQMQDIVLnEVKKvdSEYIATVCGSFRRGA----  176
DSSP  hhllhhhhhhhhlhhhhhlleEHHHHHHHHHHHHHhHHHHhlLLLEEEEELHHHHLLL----


DSSP  llllLLLLEEEEEL------------lLHHHHHHHHHHHHhhllllleeEEELLLEEllll
Query pdsrTSKDFATVAR------------rDIELIHEQLADAGetgwegftAIFTAPEEiaap  119
ident      | |                         |||             |
Sbjct ---eSSGDMDVLLThpsftsestkqpkLLHQVVEQLQKVH--------FITDTLSK----  221
DSSP  ---lEELLEEEEEEllllllllllllHHHHHHHHHHHHLL--------LEEEEEEE----


DSSP  lllLLLEEEEEEEEEL--LEEEEEEEEEEEEL-LLLLllllleeeeelhhhhlllllleee
Query gxpVKPRRFTAKLSYR--GRAFATVPIEVSSV-EAGNadqfdtltsdalglvgvpaavav  176
ident             |             |
Sbjct --gETKFMGVCQLPSKndEKEYPHRRIDIRLIpKDQY-----------------------  256
DSSP  --lLLEEEEEEELLLLLllLLLLLLLEEEEEEEELlHHHH-----------------------


DSSP  eellhHHHHhhhhhhhllllllllllllHHHH------------------hhhhhhhh
Query pcxtiPWQIaqklhavtavleepkandraHDLV------------------dlqllegl  217
ident                               |
Sbjct ----yCGVL----------------yftgSDIFnknmrahalekgftineytirplgvtg  296
DSSP  ----hHHHH----------------hhhlLHHHhhhhhhhhhhhlleeellllleeelllll
```

```
Query lldADLXPtrSACIaifearaqhPWPPRVatlphwpliyagalegldhlelartvdaaaq  277
ident      |
Sbjct vagEPLPV--DSEK-----difdYIQWKY-------------------------------  318
DSSP  lllLLLLL--LLHH-----hhhhHLLLLL-------------------------------


DSSP  hhhhhhhhhhhllll
Query avqrfvaridratkr  292
ident
Sbjct -------repkdrse  326
DSSP  -------llhhhlll
```

No 253: Query=mol1A Sbjct=4ph5A Z-score=6.7

back to top

```
DSSP  ------------------------------------------------------------
Query ------------------------------------------------------------    0
ident
Sbjct tlnggitdmltelanfeknvsqaihkynayrkaasviakyphkiksgaeakklpgvgtki   60
DSSP  lllhhhhhhhhhhhhhhhllllhhhhhhhhhhhhhhllllllllhhhhhllllllhhh


DSSP  ------------------------------------------------llllllllllhh
Query ------------------------------------------------aapysspptnlr   12
ident
Sbjct aekideflatgklrklekirqddtsssinfltrvsgigpsaarkfvdegiktledlrkne  120
DSSP  hhhhhhhhhhlllhhhhhhhhlhhhhhhhhhllllllhhhhhhhhllllllhhhhhllh


DSSP  hhhhhhhhhhhhllllhhhhhHHHHHHHHHHHHH-LLLLllLLLLEEEEHHHHHHHHhhll
Query slrdrltqvaerqgvvfgrlqRHVAXIVVAQFAA-TLTDdtGAPLLLVKGGSSLELrrgi   71
ident                                                | |
Sbjct dklnhhqriglkyfgdfekriPREEMLQMQDIVLnEVKKvdSEYIATVCGSFRRGA----  176
DSSP  hhllhhhhhhhhlllhhhlleEHHHHHHHHHHHHHhHHHHhlLLLEEEEELHHHHLLL----


DSSP  lllLLLLLEEEEEL------------lLHHHHHHHHHHHHhhlllleeEEELLLEEllll
Query pdsRTSKDFATVAR------------rDIELIHEQLADAGetgwegftAIFTAPEEiaap  119
ident      | |                         |||             |
Sbjct ---ESSGDMDVLLThpsftsestkqpkLLHQVVEQLQKVH--------FITDTLSK----  221
DSSP  ---LEELLEEEEEEllllllllllllHHHHHHHHHHHHLL--------LEEEEEEE----


DSSP  lllLLLEEEEEEEEEL--LEEEEEEEEEEEL-LLLLllllleeeeelhhhhlllllleee
Query gxpVKPRRFTAKLSYR--GRAFATVPIEVSSV-EAGNadqfdtltsdalglvgvpaavav  176
ident             |             |
Sbjct --gETKFMGVCQLPSKndEKEYPHRRIDIRLIpKDQY-----------------------  256
DSSP  --lLLEEEEEEELLLLLllLLLLLLLEEEEEEELlHHHH-----------------------


DSSP  eellhHHHHhhhhhhhhllllllllllllHHHH-------------------hhhhhhhh
Query pcxtiPWQIaqklhavtavleepkandraHDLV-------------------dlqllegl  217
ident                               |
Sbjct ----yCGVL----------------yftgSDIFnknmrahalekgftineytirplgvtg  296
DSSP  ----hHHHH----------------hhhlLHHHhhhhhhhhhhhlleeellllleeellll


DSSP  llhHHHHHhhHHHHhhhhhhlllLLLLLLlllllhhhhhhhhhlllllllllllhhhhhh
Query lldADLXPtrSACIaifearaqhPWPPRVatlphwpliyagalegldhlelartvdaaaq  277
ident      |
Sbjct vagEPLPV--DSEK-----difdYIQWKY-------------------------------  318
DSSP  lllLLLLL--LLHH-----hhhhHLLLLL-------------------------------


DSSP  hhhhhhhhhhhllll
Query avqrfvaridratkr  292
ident
Sbjct -------repkdrse  326
DSSP  -------llhhhlll
```

No 254: Query=mol1A Sbjct=4k4hI Z-score=6.7

```
DSSP  ------------------------------------------------------------
Query ------------------------------------------------------------    0
ident
Sbjct atnhnlhiteklevlakaysvqgdkwralgyakainalksfhkpvtsyqeacsipgigkr   60
DSSP  lllllhhhhhhhhhhhhhhhhhlllhhhhhhhhhhhhhhhlllllllhhhhhlllllllhh


DSSP  ----------------------------------------------llllllllllhhhh
Query ----------------------------------------------aapysspptnlrsl   14
ident
Sbjct maekiieilesghlrkldhisesvpvlelfsniwgagtktaqmwyqqgfrsledirsqas  120
DSSP  hhhhhhhhhhhlllhhhhhllllhhhhhhhhlllllhhhhhhhhhlllllhhhhhhhhll


DSSP  hhhhhhhhhhllllhhhhhHHHHHHHHHHHHH-LLLLllLLLLEEEEHHHHHHhhhllll
Query rdrltqvaerqgvvfgrlqRHVAXIVVAQFAA-TLTDdtGAPLLLVKGGSSLElrrgipd   73
ident                             |             |    |
Sbjct lttqqaiglkhysdflermPREEATEIEQTVQkAAQAfnSGLLCVACGSYRRG-------  173
DSSP  llhhhhhhhhlhhhhhlleEHHHHHHHHHHHHhHHHHhlLLLEEEELHHHHLL-------


DSSP  LLLLLLEEEEEL--------lLHHHHHHHHHHHHhhlllleeEEELLLE-ELLLllLLLL
Query SRTSKDFATVAR--------rDIELIHEQLADAGetgwegftAIFTAPE-EIAApgXPVK  124
ident   |  |                       |   |
Sbjct KATCGDVDVLIThpdgrshrgIFSRLLDSLRQEG--------FLTDDLVsQEEN--GQQQ  223
DSSP  LLEELLEEEEEElllllllllHHHHHHHHHHHLL--------LEEEEEEeEEEE--LLEE


DSSP  LEEEEEEEEEllEEEEEEEEEEEEL-LLLLlllleeeeelhhhhlllllleeeeellhHH
Query PRRFTAKLSYrgRAFATVPIEVSSV-EAGNadqfdtltsdalglvgvpaavavpcxtiPW  183
ident        |                |
Sbjct KYLGVCRLPG--PGRRHRRLDIIVVpYSEF---------------------------aCA  254
DSSP  EEEEEELLLL--LLLLLEEEEEEELlHHHH---------------------------hHH


DSSP  HHhhhhhhhhllllllllllllHHHH-------------------------hhhhhhhhl
Query QIaqklhavtavleepkandraHDLV-------------------------dlqllegll  218
ident
Sbjct LL----------------yftgSAHFnrsmralaktkgmslsehalstavvrnthgckvg  298
DSSP  HH----------------hhhlLHHHhhhhhhhhhhlleeellllleeellllllllll


DSSP  lhhHHHHhhHHHHhhhhhhlllLLLLLlllllhhhhhhhhhllllllllllllhhhHHHH
Query ldaDLXPtrSACIaifearaqhPWPPRvatlphwpliyagalegldhlelartvdaAAQA  278
ident     |
Sbjct pgrVLPT--PTEK-----dvfrLLGLP--------------------yrepaerdwLEHH  331
DSSP  lllEELL--LLHH-----hhhhHLLLL--------------------lllhhhlllLLLL


DSSP  hhhhhhhhhllll
Query vqrfvaridratkr  292
ident
Sbjct -------------h  332
DSSP  -------------l
```

No 255: Query=mol1A Sbjct=3rh5A Z-score=6.7

back to top

```
DSSP  ------------------------------------------------------------
Query ------------------------------------------------------------    0
ident
Sbjct tlnggitdmltelanfeknvsqaihkynayrkaasviakyphkiksgaeakklpgvgtki   60
DSSP  lllhhhhhhhhhhhhhhhllllhhhhhhhhhhhhhhhhlllllllhhhhllllllllhhh


DSSP  ------------------------------------------------llllllllllhh
Query ------------------------------------------------aapysspptnlr   12
ident
Sbjct aekideflatgklrklekirqddtsssinfltrvsgigpsaarkfvdegiktledlrkne  120
DSSP  hhhhhhhhhlllhhhhhhlllhhhhhhhhhlllllllhhhhhhhhhhlllllhhhhhllh


DSSP  hhhhhhhhhhhhllllhHHHHHHHHHHHHHHHH-LLLLllLLLLEEEEHHHHHHHhhll
Query slrdrltqvaerqgvvfGRLQRHVAXIVVAQFAA-TLTDdtGAPLLLVKGGSSLELrrgi   71
```

```
Sbjct dklnhhqriglkyfgdfEKRIPREEMLQMQDIVLnEVKKvdSEYIATVCGSFRRGA----  176
DSSP  hhllhhhhhhhhlhhhhHLLEEHHHHHHHHHHHHHhHHHHhlLLLEEEELHHHHLLL----


DSSP  llllLLLLEEEEEL------------lLHHHHHHHHHHHHhhlllleeEEELLLEELlll
Query pdsrTSKDFATVAR------------rDIELIHEQLADAGetgwegftAIFTAPEEIaap  119
ident      | |                         |||             |
Sbjct ---eSSGDMDVLLThpsftsestkqpkLLHQVVEQLQKVH--------FITDTLSKG---  222
DSSP  ---lEELLEEEEEElllllllllllllHHHHHHHHHHHLL--------LEEEEEEEL---


DSSP  lllLLLEEEEEEEEEL--LEEEEEEEEEEEEL-LLLLlllleeeeelhhhhlllllleee
Query gxpVKPRRFTAKLSYR--GRAFATVPIEVSSV-EAGNadqfdtltsdalglvgvpaavav  176
ident             |             |
Sbjct ---ETKFMGVCQLPSKndEKEYPHRRIDIRLIpKDQY-----------------------  256
DSSP  ---LLEEEEEEELLLLllLLLLLLEEEEEEELlHHHH-----------------------


DSSP  eellhHHHHhhhhhhhhllllllllllllHHHHHHH-------------------hhhhh
Query pcxtiPWQIaqklhavtavleepkandraHDLVDLQ-------------------llegl  217
ident                               |
Sbjct ----yCGVL----------------aftgSDIFNKNmrahalekgftineytirplgvtg  296
DSSP  ----hHHHH----------------hhhlLHHHHHHhhhhhhhlleeellllleeellll


DSSP  llhHHHHhhHHHHHhhhhhhllllLLLLLLllllhhhhhhhhhllllllllllhhhhhh
Query lldADLXptRSACIaifearaqhpWPPRVAtlphwpliyagalegldhlelartvdaaaq  277
ident      |
Sbjct vagEPLP--VDSEK-------difDYIQWK------------------------------  317
DSSP  lllLLLL--LLLHH-------hhhHHLLLL------------------------------


DSSP  hhhhhhhhhhhllll
Query avqrfvaridratkr  292
ident
Sbjct ------yrepkdrse  326
DSSP  ------lllhhhhll
```

No 256: Query=mol1A Sbjct=4ycxA Z-score=6.7

back to top

```
DSSP  ------------------------------------------------------------
Query ------------------------------------------------------------    0
ident
Sbjct wmpayacqrptplthhntglsealeilaeaagfegsegrlltfcraasvlkalpspvttl   60
DSSP  lllllhhhllllllllllhhhhhhhhhhhhhhhhlllhhhhhhhhhhhhhhhhhlllllh


DSSP  ------------------------------------------------------------
Query ------------------------------------------------------------    0
ident
Sbjct sqlqglphfgehssrvvqellehgvceevervrrseryqtmklftqifgvgvktadrwyr  120
DSSP  hhhllllllllhhhhhhhhhhhhhlllhhhhhhhhlhhhhhhhhhhllllllhhhhhhhh


DSSP  -llllllllllhhhhhhhhhhhhhhlllllhHHHHHHHHHHHHHHHHHHL-LLLLlLLLLEE
Query -aapysspptnlrslrdrltqvaerqgvvfGRLQRHVAXIVVAQFAAT-LTDDtGAPLLL   58
ident                                            |
Sbjct eglrtlddlreqpqkltqqqkaglqhhqdlSTPVLRSDVDALQQVVEEaVGQAlPGATVT  180
DSSP  llllhhhhhllhhhllhhhhhhhhlhhhhLLLEEHHHHHHHHHHHHHHhHHHHlLLLEEE


DSSP  EEHHHHHHHhhlllllllLLLLEEEEEL--------lLHHHHHHHHHHHHhhlllleeeEE
Query VKGGSSLELrrgipdsrTSKDFATVAR--------rDIELIHEQLADAGetgwegftaIF  110
ident   ||                |                       | | |
Sbjct LTGGFRRGK-------lQGHDVDFLIThpkegqeagLLPRVMCRLQDQG---------LI  224
DSSP  ELHHHHLLL-------lEELLEEEEEEllllllllLHHHHHHHHHHLL---------LE


DSSP  LLLEELllllLLLLLEEEEEEEEEELLEeEEEEEEEEEEEL-LLLLlllleeeeelhhhhll
Query TAPEEIaapgXPVKPRRFTAKLSYRGRaFATVPIEVSSV-EAGNadqfdtltsdalglvg  169
ident                      |   |     |
Sbjct LYHQHQ---hSAFERSFCIFRLPQPGS-WKAVRVDLVVApVSQF----------------  264
DSSP  EEEEEE---lLLLEEEEEEEEEEELLE-EEEEEEEEEEELlLLLH----------------
```

```
DSSP  llllleeeeellhHHHHhhhhhhhhlllllllllllHHHHHHH----------------h
Query vpaavavpcxtiPWQIaqklhavtavleepkandraHDLVDLQ----------------l  213
ident                                       |
Sbjct -----------pFALL----------------gwtgSKLFQRElrrfsrkekglwlnshg  297
DSSP  -----------hHHHH----------------hhhlLHHHHHHhhhhhhhhhlleellll


DSSP  hhhhllhHHHHhhHHHHHhhhhhhllllLLLLLLllllhhhhhhhhhlllllllllllhh
Query leglllldADLXptRSACIaifearaqhpWPPRVAtlphwpliyagalegldhlelartvd  273
ident
Sbjct lfdpeqkTFFQ--AASEE-------difRHLGLE--------------------------  322
DSSP  eeellllEELL--LLLHH-------hhhHHLLLL--------------------------


DSSP  hhhhhhhhhhhhhhhllll
Query aaaqavqrfvaridratkr  292
ident
Sbjct ----------ylppeqrna  331
DSSP  ----------lllhhhlll
```

No 257: Query=mol1A Sbjct=5ca7B Z-score=6.7

back to top

```
DSSP  --------------------------lllllllllhhhhhhhhhhhhhhllllhHHHHH
Query --------------------------aapyssspptnlrslrdrltqvaerqgvvfGRLQR   34
ident
Sbjct sesvpvlelfsniwgagtktaqmwyqqgfrsledirsqasltttqqaiglkhysdfLERMP   60
DSSP  llhhhhhhhhhllllllhhhhhhhhhlllllhhhhhhhllllhhhhhhhhhlhhhhHLLEE


DSSP  HHHHHHHHHHHHHLL-LLLLLLLLEEEEHHHHHHHhhllllllLLLLEEEEEL--------
Query HVAXIVVAQFAATL-TDDTGAPLLLVKGGSSLELrrgipdsrTSKDFATVAR--------   85
ident         |             |    |              |  |
Sbjct REEATEIEQTVQKAaQAFNSGLLCVACGSYRRGK-------aTCGDVDVLIThpdgrshr  113
DSSP  HHHHHHHHHHHHHHHhHHHLLLLEEEELHHHHLLL-------lEELLEEEEEElllllll


DSSP  lLHHHHHHHHHHHHhhllllleeEEELLLEELLLllLLLLLEEEEEEEEEllEEEEEEEEE
Query rDIELIHEQLADAGetgwegftAIFTAPEEIAApgXPVKPRRFTAKLSYrgRAFATVPIE  145
ident          |   |                                |
Sbjct gIFSRLLDSLRQEG--------FLTDDLVSQEE-nGQQQKYLGVCRLPG--PGRRHRRLD  162
DSSP  lHHHHHHHHHHHHLL--------LEEEEEEEEEE-eLLEEEEEEEEELLLL--LLLLLEEEE


DSSP  EEEL-LLLLllllleeeeelhhhhllllllleeeeellhHHHHhhhhhhhhllllllllllll
Query VSSV-EAGNadqfdtltsdalglvgvpaavavpcxtiPWQIaqklhavtavleepkandr  204
ident    |
Sbjct IIVVpYSEF---------------------------aCALL----------------yft  179
DSSP  EEELlHHHH---------------------------hHHHH----------------hhh


DSSP  lHHHHHHH------------------------hhhhhllhHHHHhhHHHHHhhhhhhll
Query aHDLVDLQ------------------------lleglllldADLXptRSACIaifearaq  239
ident                                            |
Sbjct gSAHFNRSmralaktkgmslsehalstavvrnthgckvgpgRVLP--TPTEK-------d  230
DSSP  lLHHHHHHhhhhhhhllleellllleeelleellllleeellEEEL--LLLHH-------h


DSSP  llLLLLLLLllllhhhhhhhhhllllllllllhhhhhhhhhhhhhhhhllll
Query hpWPPRVAtlphwpliyagalegldhlelartvdaaaqavqrfvaridratkr  292
ident
Sbjct vfRLLGLP------------------------------------yrepaerdw  247
DSSP  hhHHLLLL------------------------------------lllhhhhll
```

No 258: Query=mol1A Sbjct=4xq8B Z-score=6.7

back to top

```
DSSP  --------------------------lllllllllhhhhhhhhhhhhhhllllhHHHHH
Query --------------------------aapyssspptnlrslrdrltqvaerqgvvfGRLQR   34
ident
Sbjct sesvpvlelfsniwgagtktaqmwyqqgfrsledirsqasltttqqaiglkhysdfLERMP   60
```

```
DSSP  HHHHHHHHHHHHLL-LLLLLLLLEEEEHHHHHHHhhllllllLLLLEEEEEL--------
Query HVAXIVVAQFAATL-TDDTGAPLLLVKGGSSLELrrgipdsrTSKDFATVAR--------   85
ident         |             |    |              |  |
Sbjct REEATEIEQTVQKAaQAFNSGLLCVACGSYRRGK-------aTCGDVDVLIThpdgrshr  113
DSSP  HHHHHHHHHHHHHHhHHHLLLLEEEELHHHHLLL-------lEELEEEEEEEllllllll


DSSP  lLHHHHHHHHHHHHhhlllleeEEELLLEELLLllLLLLLEEEEEEEEEllEEEEEEEEE
Query rDIELIHEQLADAGetgwegftAIFTAPEEIAApgXPVKPRRFTAKLSYrgRAFATVPIE  145
ident          |   |                                |
Sbjct gIFSRLLDSLRQEG--------FLTDDLVSQEE-nGQQQKYLGVCRLPG--PGRRHRRLD  162
DSSP  lLHHHHHHHHHHLL--------LEEEEEEEEEE-eLLEEEEEEEELLLL--LLLLLEEEE


DSSP  EEEL-LLLLlllleeeeelhhhhlllllleeeeellhHHHhhhhhhhhhhllllllllll
Query VSSV-EAGNadqfdtltsdalglvgvpaavavpcxtiPWQIaqklhavtavleepkandr  204
ident    |
Sbjct IIVVpYSEF---------------------------aCALL----------------yft  179
DSSP  EEELlHHHH---------------------------hHHHH----------------hhh


DSSP  lHHHHHHH-------------------------hhhhhllhHHHHhhHHHHHhhhhhhll
Query aHDLVDLQ-------------------------llegllldADLXptRSACIaifearaq  239
ident                                            |
Sbjct gSAHFNRSmralaktkgmslsehalstavvrnthgckvgpgRVLP--TPTEK-------d  230
DSSP  lLHHHHHHhhhhhhhllleellllleeelleellllleeellEEEL--LLLHH-------h


DSSP  llLLLLLLllllhhhhhhhhhllllllllllhhhhhhhhhhhhhhhhhhllll
Query hpWPPRVAtlphwpliyagalegldhlelartvdaaaqavqrfvaridratkr  292
ident
Sbjct vfRLLGLP------------------------------------yrepaerdw  247
DSSP  hhHHLLLL------------------------------------lllhhhlll
```

No 259: Query=mol1A Sbjct=5chgA Z-score=6.7

back to top

```
DSSP  -------------------------lllllllllhhhhhhhhhhhhhhhllllhHHHHHH
Query -------------------------aapysspptnlrslrdrltqvaerqgvvfGRLQRH   35
ident
Sbjct esvpvlelfsniwgagtktaqmwyqqgfrsledirsqasltttqqaiglkhysdfLERMPR   60
DSSP  lhhhhhhhhllllllhhhhhhhhhhllllhhhhhhhhllllhhhhhhhhlhhhhHLLEEH


DSSP  HHHHHHHHHHHLL-LLLLLLLLEEEEHHHHHHHhhllllllLLLLEEEEEL--------l
Query VAXIVVAQFAATL-TDDTGAPLLLVKGGSSLELrrgipdsrTSKDFATVAR--------r   86
ident        |             |    |              |  |
Sbjct EEATEIEQTVQKAaQAFNSGLLCVACGSYRRGK-------aTCGDVDVAIThpdgrshrg  113
DSSP  HHHHHHHHHHHHHhHLLLLLLEEEELLLLLLLLL-------lEELLEEEEEEllllllll


DSSP  LHHHHHHHHHHHHhhlllleeEEELLL-EELLLllLLLLLEEEEEEEEEllEEEEEEEEE
Query DIELIHEQLADAGetgwegftAIFTAP-EEIAApgXPVKPRRFTAKLSYrgRAFATVPIE  145
ident         |   |                                 |
Sbjct IFSRLLDSLRQEG--------FLTDDLvSQEEN--GQQQKYLGVCRLPG--PGRRHRRLD  161
DSSP  HHHHHHHHHHHHL--------LEEEEElLLLEE--LLEEEEEEEELLLL--LLLLLEEEE


DSSP  EEEL-LLLLlllleeeeelhhhhlllllleeeeellhHHHhhhhhhhhhhllllllllll
Query VSSV-EAGNadqfdtltsdalglvgvpaavavpcxtiPWQIaqklhavtavleepkandr  204
ident    |
Sbjct IIVVpYSEF---------------------------aCALL----------------yft  178
DSSP  EEELlHHHH---------------------------hHHHH----------------hhh


DSSP  lHHHHHHH-----------------------hhhhhllhhhhhhhHHHHHhhhhhhllll
Query aHDLVDLQ-----------------------llegllldadlxptRSACIaifearaqhp  241
ident
Sbjct gSAHFNRSmralaktkgmslsehalstavvrnthgckvgpgrvlpTPTEK-------dvf  231
DSSP  lLHHHHHHhhhhhhhllleellllleeellllllllllllllleeelLLLHH-------hhh


DSSP  LLLLLLllllhhhhhhhhhllllllllllhhhhhhhhhhhhhhhhhhllll
```

```
ident
Sbjct RLLGLP------------------------------------yrepaerdw  246
DSSP  HHLLLL------------------------------------lllhhhlll
```

No 260: Query=mol1A Sbjct=4z6fA Z-score=6.6

back to top

```
DSSP  ------------------------------------------------------------
Query ------------------------------------------------------------    0
ident
Sbjct tlnggitdmltelanfeknvsqaihkynayrkaasviakyphkiksgaeakklpgvgtki   60
DSSP  lllhhhhhhhhhhhhhhhhllllhhhhhhhhhhhhhhhhlllllllhhhhhlllllhhh


DSSP  ------------------------------------------------llllllllllhh
Query ------------------------------------------------aapysspptnlr   12
ident
Sbjct aekideflatgklrklekirqddtsssinfltrvsgigpsaarkfvdegiktledlrkne  120
DSSP  hhhhhhhhhhlllhhhhhhhhlhhhhhhhhhlllllllhhhhhhhhhhllllhhhhhllh


DSSP  hhhhhhhhhhhhllllhhhhhHHHHHHHHHHHHHH-LLLLLLLLLLEEEEHHHHHHHhhll
Query slrdrltqvaerqgvvfgrlqRHVAXIVVAQFAA-TLTDDTGAPLLLVKGGSSLELrrgi   71
ident                                                | |
Sbjct dklnhhqriglkyfgdfekriPREEMLQMQDIVLnEVKKVDSEYIATVCGSFRRGA----  176
DSSP  hhllhhhhhhhhlhhhhhlleEHHHHHHHHHHHHHhHHHHHLLLLEEEELHHHHLLL----


DSSP  lllLLLLLEEEEEL------------lLHHHHHHHHHHHHhhlllleeEEELLLEElll
Query pdsRTSKDFATVAR------------rDIELIHEQLADAGetgwegftAIFTAPEEiaap  119
ident      | |                         |||             |
Sbjct ---ESSGDMDVLLThpsftsestkqpkLLHQVVEQLQKVH--------FITDTLSK----  221
DSSP  ---LEELLEEEEEElllllllllllHHHHHHHHHHHLL--------LEEEEEEE----


DSSP  lllLLLEEEEEEEEEL--LEEEEEEEEEEEEL-LLLLllleeeeelhhhhllllllleee
Query gxpVKPRRFTAKLSYR--GRAFATVPIEVSSV-EAGNadqfdtltsdalglvgvpaavav  176
ident             |             |
Sbjct --gETKFMGVCQLPSKndEKEYPHRRIDIRLIpKDQY-----------------------  256
DSSP  --lLLEEEEEEELLLLLllLLLLLLEEEEEEEELlHHHH-----------------------


DSSP  eellhHHHH---hhHHHHHHLLLlllllllllhhhhhhhhhhhllhhHHHHhhHHHHhh
Query pcxtiPWQI---aqKLHAVTAVLeepkandrahdlvdlqllegllldaDLXPtrSACIai  233
ident                  |                               |
Sbjct ----yCGVLyftgsDIFAKNMRAhalekgftineytirplgvtgvagePLPV--DSEK--  308
DSSP  ----hHHHHhhhllHHHHHHHHHHhhhhhleeellllleeellllllllLLLL--LLHH--


DSSP  hhhhlllLLLLLlllllhhhhhhhhhllllllllllhhhhhhhhhhhhhhhhhllll
Query fearaqhPWPPRvatlphwpliyagalegldhlelartvdaaaqavqrfvaridratkr  292
ident
Sbjct ---difdYIQWK--------------------------------------yrepkdrse  326
DSSP  ---hhhhHLLLL--------------------------------------lllhhhlll
```

No 261: Query=mol1A Sbjct=1zjnA Z-score=6.6

back to top

```
DSSP  ------------------------------------------------------------
Query ------------------------------------------------------------    0
ident
Sbjct tlnggitdmltelanfeknvsqaihkynayrkaasviakyphkiksgaeakklpgvgtki   60
DSSP  lllhhhhhhhhhhhhhhhhllllhhhhhhhhhhhhhhhhlllllllhhhhhlllllhhh


DSSP  ------------------------------------------------llllllllllhh
Query ------------------------------------------------aapysspptnlr   12
ident
Sbjct aekideflatgklrklekirqddtsssinfltrvsgigpsaarkfvdegiktledlrkne  120
DSSP  hhhhhhhhhhlllhhhhhhhhllhhhhhhhhhlllllllhhhhhhhhhlllllhhhhhhlh
```

```
DSSP  hhhhhhhhhhhhllllhhhhhHHHHHHHHHHHHHH-LLLLllLLLLEEEEHHHHHHHHhhll
Query slrdrltqvaerqgvvfgrlqRHVAXIVVAQFAA-TLTDdtGAPLLLVKGGSSLELrrgi   71
ident                                                | |
Sbjct dklnhhqriglkyfgdfekriPREEMLQMQDIVLnEVKKvdSEYIATVCGSFRRGA----  176
DSSP  hhllhhhhhhhhlhhhhhlleEHHHHHHHHHHHHHhHHHLllLLLEEEELHHHHLLL----
```

```
DSSP  llllLLLLEEEEEL------------lLHHHHHHHHHHHHhhlllleeEEELLLEEllll
Query pdsrTSKDFATVAR------------rDIELIHEQLADAGetgwegftAIFTAPEEiaap  119
ident      | |                         |||             |
Sbjct ---eSSGDMDVLLThpsftsestkqpkLLHQVVEQLQKVH--------FITDTLSK----  221
DSSP  ---lEELLEEEEEEllllllllllllHHHHHHHHHHHHLL--------LEEEEEEE----
```

```
DSSP  lllLLLEEEEEEEEEL--LEEEEEEEEEEEEL-LLLLlllleeeeelhhhhllllllleee
Query gxpVKPRRFTAKLSYR--GRAFATVPIEVSSV-EAGNadqfdtltsdalglvgvpaavav  176
ident             |             |
Sbjct --gETKFMGVCQLPSKndEKEYPHRRIDIRLIpKDQY-----------------------  256
DSSP  --lLLEEEEEEELLLLLllLLLLLLEEEEEEELlHHHH-----------------------
```

```
DSSP  eellhHHHHHHhhhhhhlllllllllllHHHH-------------------hhhhhhhh
Query pcxtiPWQIAQklhavtavleepkandraHDLV-------------------dlqllegl  217
ident                               |
Sbjct -----YCGVLY---------------ftgSDIFnknmrahalekgftineytirplgvtg  296
DSSP  -----HHHHHH---------------hhlLHHHhhhhhhhhhhhleeellllleeelllll
```

```
DSSP  llhHHHHHhhHHHHhhhhhhlllLLLLLllllllhhhhhhhhhlllllllllllhhhhhh
Query lldADLXPtrSACIaifearaqhPWPPRvatlphwpliyagalegldhlelartvdaaaq  277
ident      |
Sbjct vagEPLPV--DSEK-----difdYIQWK--------------------------------  317
DSSP  lllLLLLL--LLHH-----hhhhHLLLL--------------------------------
```

```
DSSP  hhhhhhhhhhhllll
Query avqrfvaridratkr  292
ident
Sbjct -------yrepkdrs  325
DSSP  -------lllhhhll
```

No 262: Query=mol1A Sbjct=4rpzA Z-score=6.6

back to top

```
DSSP  ------------------------------------------------------------
Query ------------------------------------------------------------    0
ident
Sbjct tlnggitdmltelanfeknvsqaihkynayrkaasviakyphkiksgaeakklpgvgtki   60
DSSP  lllhhhhhhhhhhhhhhhhhllllhhhhhhhhhhhhhhhhhlllllllhhhhhlllllllhhh
```

```
DSSP  ------------------------------------------------llllllllllhh
Query ------------------------------------------------aapysspptnlr   12
ident
Sbjct aekideflatgklrklekirqddtsssinfltrvsgigpsaarkfvdegiktledlrkne  120
DSSP  hhhhhhhhhhlllhhhhhhhhlhhhhhhhhhlllllllhhhhhhhhhlllllhhhhhhlh
```

```
DSSP  hhhhhhhhhhhhllllhhhhhHHHHHHHHHHHHHH-LLLLllLLLLEEEEHHHHHHHHhhll
Query slrdrltqvaerqgvvfgrlqRHVAXIVVAQFAA-TLTDdtGAPLLLVKGGSSLELrrgi   71
ident                                                | |
Sbjct dklnhhqriglkyfgdfekriPREEMLQMQDIVLnEVKKvdSEYIATVCGSFRRGA----  176
DSSP  hhllhhhhhhhhlhhhhllleEHHHHHHHHHHHHHhHHHHhlLLLEEEELHHHHLLL----
```

```
DSSP  llllLLLLEEEEEL------------lLHHHHHHHHHHHHhhlllleeEEELLLEEllll
Query pdsrTSKDFATVAR------------rDIELIHEQLADAGetgwegftAIFTAPEEiaap  119
ident      | |                         |||             |
Sbjct ---eSSGDMDVLLThpsftsestkqpkLLHQVVEQLQKVH--------FITDTLSK----  221
DSSP  ---lEELLEEEEEEllleelleellllHHHHHHHHHHHHLL--------LEEEEEEE----
```

```
DSSP  lllLLLEEEEEEEEEL--lEEEEEEEEEEEEL-LLLLlllleeeeelhhhhllllllleee
Query gxpVKPRRFTAKLSYR--gRAFATVPIEVSSV-EAGNadqfdtltsdalglvgvpaavav  176
ident             |             |
```

```
DSSP  --lLLEEEEEELLLLLllLLLLLLEEEEEEELlHHHH-----------------------


DSSP  eellhHHHhhhhhhhhlllllllllllllHHHH-------------------hhhhhhhh
Query pcxtiPWQIaqklhavtavleepkandraHDLV-------------------dlqllegl  217
ident                                |
Sbjct ----yCGVL----------------yftgSDIFnknmrahalekgftineytirplgvtg  296
DSSP  ----hHHHH----------------hhhlLHHHhhhhhhhhhhlleeelllleeelllll


DSSP  llhHHHHHhhHHHHhhhhhhlllLLLLLLlllllhhhhhhhhhhllllllllllhhhhhh
Query lldADLXPtrSACIaifearaqhPWPPRVatlphwpliyagalegldhlelartvdaaaq  277
ident      |
Sbjct vagEPLPV--DSEK-----difdYIQWKY-------------------------------  318
DSSP  lllLLLLL--LLHH-----hhhhHLLLLL-------------------------------


DSSP  hhhhhhhhhhhllll
Query avqrfvaridratkr  292
ident
Sbjct -------repkdrse  326
DSSP  -------llhhhhll
```

No 263: Query=mol1A Sbjct=3lk9A Z-score=6.6

back to top

```
DSSP  ------------------------------------------------------------
Query ------------------------------------------------------------    0
ident
Sbjct tlnggitdmltelanfeknvsqaihkynayrkaasviakyphkiksgaeakklpgvgtki   60
DSSP  lllhhhhhhhhhhhhhhhhllllhhhhhhhhhhhhhhhllllllllhhhhllllllllhhh


DSSP  ------------------------------------------------llllllllllhh
Query ------------------------------------------------aapysspptnlr   12
ident
Sbjct aekideflatgklrklekirqddtsssinfltrvsgigpsaarkfvdegiktledlrkne  120
DSSP  hhhhhhhhhhlllhhhhhhhhlhhhhhhhhhhlllllhhhhhhhhhhlllllhhhhhhlh


DSSP  hhhhhhhhhhhhllllhhhhhHHHHHHHHHHHHHH-LLLLllLLLLEEEEHHHHHHHhhll
Query slrdrltqvaerqgvvfgrlqRHVAXIVVAQFAA-TLTDdtGAPLLLVKGGSSLELrrgi   71
ident                                                |  |
Sbjct dklnhhqriglkyfgdfekriPREEMLQMQDIVLnEVKKvdSEYIATVCGSFRRGA----  176
DSSP  hhllhhhhhhhhlllhhhlleEHHHHHHHHHHHHhHHLLllLLLEEEELHHHHLLL----


DSSP  llllLLLLEEEEEL------------lLHHHHHHHHHHHHhhlllleeeEELLLEELlll
Query pdsrTSKDFATVAR------------rDIELIHEQLADAGetgwegftaIFTAPEEIaap  119
ident      | |                         |||               |
Sbjct ---eSSGDMDVLLThpsftsestkqpkLLHQVVEQLQKVH---------FITDTLSK---  221
DSSP  ---lEELLEEEEEElllee lleellllHHHHHHHHHHHLL---------LEEEEEEE---


DSSP  lllLLLEEEEEEEEEL--LEEEEEEEEEEEEL-LLLLlllleeeeelhhhhllllllleee
Query gxpVKPRRFTAKLSYR--GRAFATVPIEVSSV-EAGNadqfdtltsdalglvgvpaavav  176
ident             |             |
Sbjct --gETKFMGVCQLPSKndEKEYPHRRIDIRLIpKDQY-----------------------  256
DSSP  --lLLEEEEEELLLLLllLLLLLLEEEEEEELlLLLH-----------------------


DSSP  eellhHHHhhhhhhhhlllllllllllllHHHHHHH-------------------hhhhh
Query pcxtiPWQIaqklhavtavleepkandraHDLVDLQ-------------------llegl  217
ident                                |
Sbjct ----yCGVL----------------yftgSDIFNKNmrahalekgftineytirplgvtg  296
DSSP  ----hHHHH----------------hhhlLHHHHHHhhhhhhhhleeelllleeelllll


DSSP  llhHHHHhhHHHHHhhhhhhllllLLLLLlllllhhhhhhhhhhllllllllllhhhhhh
Query lldADLXptRSACIaifearaqhpWPPRVatlphwpliyagalegldhlelartvdaaaq  277
ident      |
Sbjct vagEPLP--VDSEK-------difDYIQW-------------------------------  316
DSSP  lllLLLL--LLLHH-------hhhHHLLL-------------------------------
```

```
Query avqrfvaridratkr  292
ident
Sbjct -----kyrepkdrse  326
DSSP  -----llllhhhlll
```

No 264: Query=mol1A Sbjct=9icrA Z-score=6.6

back to top

```
DSSP  ------------------------------------------------------------
Query ------------------------------------------------------------    0
ident
Sbjct etlnggitdmltelanfeknvsqaihkynayrkaasviakyphkiksgaeakklpgvgtk   60
DSSP  llllhhhhhhhhhhhhhhhhllllhhhhhhhhhhhhhhhhllllllllhhhhhllllllhh


DSSP  -------------------------------------------------llllllllllh
Query -------------------------------------------------aapysspptnl   11
ident
Sbjct iaekideflatgklrklekirqddtsssinfltrvsgigpsaarkfvdegiktledlrkn  120
DSSP  hhhhhhhhhhllllhhhhhhlllhhhhhhhhhlllllllhhhhhhhhhlllllhhhhhhl


DSSP  hhhhhhhhhhhhhllllhhhhhHHHHHHHHHHHHH-LLLLllLLLLLEEEEHHHHHHHhhl
Query rslrdrltqvaerqgvvfgrlqRHVAXIVVAQFAA-TLTDdtGAPLLLVKGGSSLELrrg   70
ident                                                 | |
Sbjct edklnhhqriglkyfgdfekriPREEMLQMQDIVLnEVKKvdSEYIATVCGSFRRGA---  177
DSSP  lllllhhhhhhhhlhhhhhlleEHHHHHHHHHHHHHhHHHHhlLLLEEEELLLLLLL---


DSSP  lllllLLLLEEEEEL------------lLHHHHHHHHHHHHhhlllleeEEELLLEELll
Query ipdsrTSKDFATVAR------------rDIELIHEQLADAGetgwegftAIFTAPEEIaa  118
ident       | |                         |||             |
Sbjct ----eSSGDMDVLLThpsftsestkqpkLLHQVVEQLQKVH--------FITDTLSKG--  223
DSSP  ----lEELLEEEEEEllllllllllllhHHHHHHHHHHHHLL--------LEEEEEEEL--


DSSP  llllLLLEEEEEEEEEELL--EEEEEEEEEEEEL-LLLLllllleeeeelhhhhllllllee
Query pgxpVKPRRFTAKLSYRG--RAFATVPIEVSSV-EAGNadqfdtltsdalglvgvpaava  175
ident              |             |
Sbjct ----ETKFMGVCQLPSKNdeKEYPHRRIDIRLIpKDQY----------------------  257
DSSP  ----LLEEEEEEELLLLLllLLLLLLEEEEEEEELlHHHH----------------------


DSSP  eeellhHHHHhhhhhhhhllllllllllllhhhhHHHHHH--------------------
Query vpcxtiPWQIaqklhavtavleepkandrahdlvDLQLLE--------------------  215
ident
Sbjct -----yCGVL--------------------yftgSDIFNKnmrahalekgftineytirp  292
DSSP  -----hHHHH--------------------hhllLHHHHHhhhhhhhhlleeellllleee


DSSP  -hhllhhhhhhhHHHHHHhhhhhllllLLLLLlllllhhhhhhhhhlllllllllllhhh
Query -glllddadlxptRSACIAifearaqhpWPPRVatlphwpliyagalegldhlelartvda  274
ident
Sbjct lgvtgvageplpVDSEKD------ifdYIQWK----------------------------  318
DSSP  lllllllllllLLLHHH------hhhHLLLL----------------------------


DSSP  hhhhhhhhhhhhhhllll
Query aaqavqrfvaridratkr  292
ident
Sbjct ---------yrepkdrse  327
DSSP  ---------llllhhhll
```

No 265: Query=mol1A Sbjct=9icqA Z-score=6.6

back to top

```
DSSP  ------------------------------------------------------------
Query ------------------------------------------------------------    0
ident
Sbjct etlnggitdmltelanfeknvsqaihkynayrkaasviakyphkiksgaeakklpgvgtk   60
DSSP  llllhhhhhhhhhhhhhhhhllllhhhhhhhhhhhhhhhhllllllllhhhhhllllllhh
```

```
DSSP  -------------------------------------------------llllllllllh
Query -------------------------------------------------aapysspptnl   11
ident
Sbjct iaekideflatgklrklekirqddtsssinfltrvsgigpsaarkfvdegiktledlrkn  120
DSSP  hhhhhhhhhhllllhhhhhhlllhhhhhhhhhllllllhhhhhhhhllllllhhhhhhl
```

```
DSSP  hhhhhhhhhhhhhllllhhhhhHHHHHHHHHHHHH-LLLLllLLLLEEEEHHHHHHHHhhl
Query rslrdrltqvaerqgvvfgrlqRHVAXIVVAQFAA-TLTDdtGAPLLLVKGGSSLELrrg   70
ident                                                 | |
Sbjct edklnhhqriglkyfgdfekriPREEMLQMQDIVLnEVKKvdSEYIATVCGSFRRGA---  177
DSSP  llllhhhhhhhhlhhhhhlleEHHHHHHHHHHHHhHHHHhlLLLEEEELLHHHHLL---
```

```
DSSP  lllllLLLLEEEEEL------------lLHHHHHHHHHHHhhlllleeEEELLLEELll
Query ipdsrTSKDFATVAR------------rDIELIHEQLADAGetgwegftAIFTAPEEIaa  118
ident       | |                         |||             |
Sbjct ----eSSGDMDVLLThpsftsestkqpkLLHQVVEQLQKVH--------FITDTLSKG--  223
DSSP  ----lEELLEEEEEEellllllllllhHHHHHHHHHHHLL--------LEEEEEEEL--
```

```
DSSP  llllLLLEEEEEEEEELL--EEEEEEEEEEEEL-LLLLllllleeeeelhhhhllllllee
Query pgxpVKPRRFTAKLSYRG--RAFATVPIEVSSV-EAGNadqfdtltsdalglvgvpaava  175
ident              |             |
Sbjct ----ETKFMGVCQLPSKNdeKEYPHRRIDIRLIpKDQY----------------------  257
DSSP  ----LLEEEEEELLLLLLllLLLLLLEEEEEEEEeHHHH----------------------
```

```
DSSP  eeellhHHHHhhhhhhhhlllllllllllhhhhHHHHHH--------------------
Query vpcxtiPWQIaqklhavtavleepkandrahdlvDLQLLE--------------------  215
ident
Sbjct -----yCGVL--------------------yftgSDIFNKnmrahalekgftineytirp  292
DSSP  -----hHHHH--------------------hhllLHHHHHhhhhhhhhlleeeelleeee
```

```
DSSP  -hhllhhhhhhhHHHHHHhhhhhllllLLLLLlllllhhhhhhhhhlllllllllllhhh
Query -glllldadlxptRSACIAifearaqhpWPPRVatlphwpliyagalegldhlelartvda  274
ident
Sbjct lgvtgvageplpVDSEKD------ifdYIQWK----------------------------  318
DSSP  llllllllllllLLLHHH------hhhHLLLL----------------------------
```

```
DSSP  hhhhhhhhhhhhhhllll
Query aaqavqrfvaridratkr  292
ident
Sbjct ---------yrepkdrse  327
DSSP  ---------lllhhhhll
```

No 266: Query=mol1A Sbjct=1huzB Z-score=6.6

back to top

```
DSSP  ------------------------------------------------------------
Query ------------------------------------------------------------    0
ident
Sbjct tlnggitdmlvelanfeknvsqaihkynayrkaasviakyphkiksgaeakklpgvgtki   60
DSSP  lllhhhhhhhhhhhhhhhhhllllhhhhhhhhhhhhhhhlllllllllhhhhhllllllhhh
```

```
DSSP  ------------------------------------------------lllllllllhh
Query ------------------------------------------------aapysspptnlr   12
ident
Sbjct aekideflatgklrklekirqddtsssinfltrvtgigpsaarklvdegiktledlrkne  120
DSSP  hhhhhhhhhhlllhhhhhhhhlhhhhhhhhhllllllhhhhhhhhllllllhhhhhllh
```

```
DSSP  hhhhhhhhhhhhllllhhhhhHHHHHHHHHHHHH-LLLLLLLLLLEEEEHHHHHHHHhhll
Query slrdrltqvaerqgvvfgrlqRHVAXIVVAQFAA-TLTDDTGAPLLLVKGGSSLELrrgi   71
ident                                                | |
Sbjct dklnhhqriglkyfedfekriPREEMLQMQDIVLnEVKKLDPEYIATVCGSFRRGA----  176
DSSP  hhllhhhhhhhhlhhhhhlllLHHHHHHHHHHHHhHHHHHLLLLEEEELHHHHLLL----
```

```
DSSP  llllLLLLEEEEEL------------lLHHHHHHHHHHHhhlllleeEEELLLEELlll
Query pdsrTSKDFATVAR------------rDIELIHEQLADAGetgwegftAIFTAPEEIaap  119
```

```
Sbjct ---eSSGDMDVLLThpnftsesskqpkLLHRVVEQLQKVR--------FITDTLSKG---  222
DSSP  ---lLLLLEEEEEEllllllllllllhHHHHHHHHHHHHLL--------LEEEEEEEL---
```

```
DSSP  lllLLLEEEEEEEEEELL--EEEEEEEEEEEEL-LLLLllllleeeeelhhhhlllllleee
Query gxpVKPRRFTAKLSYRG--RAFATVPIEVSSV-EAGNadqfdtltsdalglvgvpaavav  176
ident             |             |
Sbjct ---ETKFMGVCQLPSENdeNEYPHRRIDIRLIpKDQY-----------------------  256
DSSP  ---LLEEEEEEELLLLLLllLLLLLLEEEEEEEELlHHHH-----------------------
```

```
DSSP  eellhHHHHhhhhhhhhllllllllllllHHHHHHH------------------hhhhh
Query pcxtiPWQIaqklhavtavleepkandraHDLVDLQ------------------llegl  217
ident                               |
Sbjct ----yCGVL----------------yftgSDIFNKNmrahalekgftineytirplgvtg  296
DSSP  ----hHHHH----------------hhhlLHHHHHHhhhhhhhhleeellllleeellllll
```

```
DSSP  llhHHHHhhHHHHHhhhhhhllllLLLLLLllllhhhhhhhhhllllllllllhhhhhh
Query lldADLXptRSACIaifearaqhpWPPRVAtlphwpliyagalegldhlelartvdaaaq  277
ident      |
Sbjct vagEPLP--VDSEQ-------difDYIQWR------------------------------  317
DSSP  lllLLLL--LLLHH-------hhhHHLLLL------------------------------
```

```
DSSP  hhhhhhhhhhhllll
Query avqrfvaridratkr  292
ident
Sbjct -------yrepkdrs  325
DSSP  -------lllhhhll
```

No 267: Query=mol1A Sbjct=8iceA Z-score=6.6

back to top

```
DSSP  ------------------------------------------------------------
Query ------------------------------------------------------------    0
ident
Sbjct etlnggitdmltelanfeknvsqaihkynayrkaasviakyphkiksgaeakklpgvgtk   60
DSSP  llllhhhhhhhhhhhhhhhhhhhlllhhhhhhhhhhhhhhhhhlllllllhhhhhlllllhh
```

```
DSSP  -------------------------------------------------llllllllllh
Query -------------------------------------------------aapysspptnl   11
ident
Sbjct iaekideflatgklrklekirqddtsssinfltrvsgigpsaarkfvdegiktledlrkn  120
DSSP  hhhhhhhhhhllllhhhhhhhhlhhhhhhhhhllllllhhhhhhhhhhllllllhhhhl
```

```
DSSP  hhhhhhhhhhhhhllllhhhhhHHHHHHHHHHHHH-LLLLllLLLLLEEEEHHHHHHHHhhl
Query rslrdrltqvaerqgvvfgrlqRHVAXIVVAQFAA-TLTDdtGAPLLLVKGGSSLELrrg   70
ident                                                 | |
Sbjct edklnhhqriglkyfgdfekriPREEMLQMQDIVLnEVKKvdSEYIATVCGSFRRGA---  177
DSSP  hhhllhhhhhhhhhlllllllleEHHHHHHHHHHHHHHhHHHHhlLLLEEEEEELLHHHLL---
```

```
DSSP  lllllLLLLEEEEEL------------lLHHHHHHHHHHHHHhhllllleeEEELLLEELll
Query ipdsrTSKDFATVAR------------rDIELIHEQLADAGetgwegftAIFTAPEEIaa  118
ident       | |                         |||             |
Sbjct ----eSSGDMDVLLThpsftsestkqpkLLHQVVEQLQKVH--------FITDTLSKG--  223
DSSP  ----lEELLEEEEEEellllllllllllhHHHHHHHHHHHHLL--------LEEEEEEEL--
```

```
DSSP  llllLLLEEEEEEEEEELL--EEEEEEEEEEEEL-LLLLllllleeeeelhhhhllllllee
Query pgxpVKPRRFTAKLSYRG--RAFATVPIEVSSV-EAGNadqfdtltsdalglvgvpaava  175
ident              |             |
Sbjct ----ETKFMGVCQLPSKNdeKEYPHRRIDIRLIpKDQY----------------------  257
DSSP  ----LLEEEEEEELLLLLLllLLLLLLEEEEEEEEeHHHH----------------------
```

```
DSSP  eeellhHHHHhhhhhhhhlllllllllllhhhhHHHHHH--------------------
Query vpcxtiPWQIaqklhavtavleepkandrahdlvDLQLLE--------------------  215
ident
Sbjct -----yCGVL--------------------yftgSDIFNKNmrahalekgftineytirp  292
DSSP  -----hHHHH--------------------hhhlLHHHHHHhhhhhhhhlleeeelleeee
```

```
DSSP  -hhllhhhhhhhHHHHHhhhhhhllllLLLLLlllllhhhhhhhhhllllllllllhhh
Query -glldadlxptRSACIaifearaqhpWPPRVatlphwpliyagaleglдhlelartvda  274
ident
Sbjct lgvtgvageplpVDSEK-------difDYIQW---------------------------  317
DSSP  lllllllllllLLLHH-------hhhHHLLL---------------------------


DSSP  hhhhhhhhhhhhhhllll
Query aaqavqrfvaridratkr  292
ident
Sbjct --------kyrepkdrse  327
DSSP  --------llllhhhlll
```

No 268: Query=mol1A Sbjct=9iciA Z-score=6.6

back to top

```
DSSP  ------------------------------------------------------------
Query ------------------------------------------------------------    0
ident
Sbjct etlnggitdmltelanfeknvsqaihkynayrkaasviakyphkiksgaeakklpgvgtk   60
DSSP  llllhhhhhhhhhhhhhhlllllhhhhhhhhhhhhhhhlllllllhhhhhllllllhh


DSSP  -------------------------------------------------lllllllllh
Query -------------------------------------------------aapysspptnl   11
ident
Sbjct iaekideflatgklrklekirqddtsssinfltrvsgigpsaarkfvdegiktledlrkn  120
DSSP  hhhhhhhhhhllllhhhhhhhhlhhhhhhhhhllllllhhhhhhhhhhllllhhhhhhl


DSSP  hhhhhhhhhhhhllllhhhhhHHHHHHHHHHHHH-LLLLllLLLLLEEEEHHHHHHHhhl
Query rslrdrltqvaerqgvvfgrlqRHVAXIVVAQFAA-TLTDdtGAPLLLVKGGSSLELrrg   70
ident                                                 | |
Sbjct edklnhhqriglkyfgdfekriPREEMLQMQDIVLnEVKKvdSEYIATVCGSFRRGA---  177
DSSP  hhhllhhhhhhhhlllllllleEHHHHHHHHHHHHhHHHHhlLLLEEEEELLHHHHLL---


DSSP  lllllLLLLEEEEEL------------lLHHHHHHHHHHHHHhhllllleeEEEELLLEELll
Query ipdsrTSKDFATVAR------------rDIELIHEQLADAGetgwegftAIFTAPEEIaa  118
ident       | |                         |||              |
Sbjct ----eSSGDMDVLLThpsftsestkqpkLLHQVVEQLQKVH--------FITDTLSKG--  223
DSSP  ----lEELLEEEEEEelllllllllllhHHHHHHHHHHHHHL--------LEEEEEEEL--


DSSP  llllLLLEEEEEEEEELL--EEEEEEEEEEEEL-LLLLllllleeeeelhhhhllllllee
Query pgxpVKPRRFTAKLSYRG--RAFATVPIEVSSV-EAGNadqfdtltsdalglvgvpaava  175
ident              |             |
Sbjct ----ETKFMGVCQLPSKNdeKEYPHRRIDIRLIpKDQY----------------------  257
DSSP  ----LLEEEEEEELLLLLLllLLLLLLEEEEEEEEEeHHHH----------------------


DSSP  eeellhHHHHhhhhhhhhlllllllllllhhhhHHHHHH--------------------
Query vpcxtiPWQIaqklhavtavleepkandrahdlvDLQLLE--------------------  215
ident
Sbjct -----yCGVL--------------------yftgSDIFNKnmrahalekgftineytirp  292
DSSP  -----hHHHH--------------------hhhlLHHHHHhhhhhhhhlleeelllleee


DSSP  --hhllhhhhhhhhHHHHhhhhhhllllLLLLLlllllhhhhhhhhhlllllllllllhh
Query --glldadlxptrSACIaifearaqhpWPPRVatlphwpliyagalegldhlelartvd  273
ident               |
Sbjct lgvtgvageplpvdSEKD-------ifdYIQWK---------------------------  318
DSSP  llllllllllllLHHH-------hhhHLLLL---------------------------


DSSP  hhhhhhhhhhhhhhhllll
Query aaaqavqrfvaridratkr  292
ident
Sbjct ----------yrepkdrse  327
DSSP  ----------lllhhhlll
```

No 269: Query=mol1A Sbjct=8icyA Z-score=6.6

```
DSSP  ------------------------------------------------------------
Query ------------------------------------------------------------    0
ident 
Sbjct etlnggitdmltelanfeknvsqaihkynayrkaasviakypkhkiksgaeakklpgvgtk   60
DSSP  llllhhhhhhhhhhhhhhhllllhhhhhhhhhhhhhhhlllllllhhhhhllllllhh


DSSP  -------------------------------------------------llllllllllh
Query -------------------------------------------------aapysspptnl   11
ident 
Sbjct iaekideflatgklrklekirqddtsssinfltrvsgigpsaarkfvdegiktledlrkn  120
DSSP  hhhhhhhhhhllllhhhhhhhhlhhhhhhhhhllllllhhhhhhhhhhllllhhhhhhl


DSSP  hhhhhhhhhhhhllllhhhhhHHHHHHHHHHHHH-LLLLllLLLLEEEEHHHHHHHhhl
Query rslrdrltqvaerqgvvfgrlqRHVAXIVVAQFAA-TLTDdtGAPLLLVKGGSSLELrrg   70
ident                                                 | |
Sbjct edklnhhqriglkyfgdfekriPREEMLQMQDIVLnEVKKvdSEYIATVCGSFRRGA---  177
DSSP  hhhllhhhhhhhhlllhhhlleEHHHHHHHHHHHHhHHHHhlLLLEEEELLLLLLL---


DSSP  lllllLLLLEEEEEL------------lLHHHHHHHHHHHHhhllllleeEEELLLEELll
Query ipdsrTSKDFATVAR------------rDIELIHEQLADAGetgwegftAIFTAPEEIaa  118
ident       | |                         |||             |
Sbjct ----eSSGDMDVLLThpsftsestkqpkLLHQVVEQLQKVH--------FITDTLSKG--  223
DSSP  ----lEELLEEEEEEellllllllllhHHHHHHHHHHHHLL--------LEEEEEELL--


DSSP  llllLLLEEEEEEEEELL--EEEEEEEEEEEEL-LLLLllllleeeeelhhhhllllllee
Query pgxpVKPRRFTAKLSYRG--RAFATVPIEVSSV-EAGNadqfdtltsdalglvgvpaava  175
ident              |             |
Sbjct ----ETKFMGVCQLPSKNdeKEYPHRRIDIRLIpKDQY----------------------  257
DSSP  ----LLEEEEEEELLLLLllLLLLEEEEEEEEEeHHHH----------------------


DSSP  eeellhHHHHhhhhhhhhllllllllllllhhhhHHHHHH--------------------
Query vpcxtiPWQIaqklhavtavleepkandrahdlvDLQLLE--------------------  215
ident 
Sbjct -----yCGVL--------------------yftgSDIFNKnmrahalekgftineytirp  292
DSSP  -----hHHHH--------------------hhllLHHHHHhhhhhhllllleeellllee


DSSP  -hhllhhhhhhhHHHHHHhhhhhllllLLLLLLllllhhhhhhhhhllllllllllhhh
Query -glllddadlxptRSACIAifearaqhpWPPRVatlphwpliyagalegldhlelartvda  274
ident 
Sbjct lgvtgvageplpVDSEKD------ifdYIQWK----------------------------  318
DSSP  lllllllllllLLLHHH------hhhHLLLL----------------------------


DSSP  hhhhhhhhhhhhhhllll
Query aaqavqrfvaridratkr  292
ident 
Sbjct ---------yrepkdrse  327
DSSP  ---------lllllllll
```

No 270: Query=mol1A Sbjct=9icjA Z-score=6.6

back to top

```
DSSP  ------------------------------------------------------------
Query ------------------------------------------------------------    0
ident 
Sbjct etlnggitdmltelanfeknvsqaihkynayrkaasviakypkhkiksgaeakklpgvgtk   60
DSSP  llllhhhhhhhhhhhhhhhllllhhhhhhhhhhhhhhhlllllllhhhhhllllllhh


DSSP  -------------------------------------------------llllllllllh
Query -------------------------------------------------aapysspptnl   11
ident 
Sbjct iaekideflatgklrklekirqddtsssinfltrvsgigpsaarkfvdegiktledlrkn  120
DSSP  hhhhhhhhhhllllhhhhhhhhlhhhhhhhhhllllllhhhhhhhhhhllllhhhhhhl


DSSP  hhhhhhhhhhhhllllhhhhhHHHHHHHHHHHHH-LLLLllLLLLEEEEHHHHHHHhhl
Query rslrdrltqvaerqgvvfgrlqRHVAXIVVAQFAA-TLTDdtGAPLLLVKGGSSLELrrg   70
```

```
Sbjct edklnhhqriglkyfgdfekriPREEMLQMQDIVLnEVKKvdSEYIATVCGSFRRGA---  177
DSSP  hhhllhhhhhhhhlhhhhhlleEHHHHHHHHHHHHHhHHHHhlLLLEEEEELLLLLLL---


DSSP  lllllLLLLEEEEEL------------lLHHHHHHHHHHHHHhhllllleeEEELLLEELll
Query ipdsrTSKDFATVAR------------rDIELIHEQLADAGetgwegftAIFTAPEEIaa  118
ident       | |                         |||             |
Sbjct ----eSSGDMDVLLThpsftsestkqpkLLHQVVEQLQKVH--------FITDTLSKG--  223
DSSP  ----lEELLEEEEEEellllllllllhHHHHHHHHHHHHLL--------LEEEEEEEL--


DSSP  llllLLLEEEEEEEEELL--EEEEEEEEEEEL-LLLLllllleeeeelhhhhllllllee
Query pgxpVKPRRFTAKLSYRG--RAFATVPIEVSSV-EAGNadqfdtltsdalglvgvpaava  175
ident              |             |
Sbjct ----ETKFMGVCQLPSKNdeKEYPHRRIDIRLIpKDQY----------------------  257
DSSP  ----LLEEEEEEELLLLLLllLLLLLLEEEEEEEEEeHHHH----------------------


DSSP  eeellhHHHHhhhhhhhhlllllllllllhhhhHHHHHH--------------------
Query vpcxtiPWQIaqklhavtavleepkandrahdlvDLQLLE--------------------  215
ident
Sbjct -----yCGVL--------------------yftgSDIFNKnmrahalekgftineytirp  292
DSSP  -----hHHHH--------------------hhllLHHHHHhhhhhhhhlleeeelleeee


DSSP  -hhllhhhhhhhHHHHHHhhhhhllllLLLLLlllllhhhhhhhhhlllllllllhhh
Query -gllldadlxptRSACIAifearaqhpWPPRVatlphwpliyagaleglдhlelartvda  274
ident
Sbjct lgvtgvageplpVDSEKD------ifdYIQWK----------------------------  318
DSSP  llllllllllllLLLHHH------hhhHLLLL----------------------------


DSSP  hhhhhhhhhhhhhhllll
Query aaqavqrfvaridratkr  292
ident
Sbjct ---------yrepkdrse  327
DSSP  ---------lllhhhlll
```

No 271: Query=mol1A Sbjct=8icmA Z-score=6.6

back to top

```
DSSP  ------------------------------------------------------------
Query ------------------------------------------------------------    0
ident
Sbjct etlnggitdmltelanfeknvsqaihkynayrkaasviakyphkiksgaeakklpgvgtk   60
DSSP  llllhhhhhhhhhhhhhhhhlllllhhhhhhhhhhhhhhlllllllllllhhhhhlllllllhh


DSSP  -------------------------------------------------llllllllllh
Query -------------------------------------------------aapysspptnl   11
ident
Sbjct iaekideflatgklrklekirqddtsssinfltrvsgigpsaarkfvdegiktledlrkn  120
DSSP  hhhhhhhhhhllllhhhhhhhhlhhhhhhhhhllllllhhhhhhhhhhllllhhhhhll


DSSP  hhhhhhhhhhhhhllllhhhhhHHHHHHHHHHHHHH-LLLLllLLLLLEEEEHHHHHHHHhhl
Query rslrdrltqvaerqgvvfgrlqRHVAXIVVAQFAA-TLTDdtGAPLLLVKGGSSLELrrg   70
ident                                                 | |
Sbjct edklnhhqriglkyfgdfekriPREEMLQMQDIVLnEVKKvdSEYIATVCGSFRRGA---  177
DSSP  hhhllhhhhhhhhlhhhhhlleEHHHHHHHHHHHHHhHHHHhlLLLEEEEELLLLLLL---


DSSP  lllllLLLLEEEEEL------------lLHHHHHHHHHHHHHhhllllleeEEELLLEELll
Query ipdsrTSKDFATVAR------------rDIELIHEQLADAGetgwegftAIFTAPEEIaa  118
ident       | |                         |||             |
Sbjct ----eSSGDMDVLLThpsftsestkqpkLLHQVVEQLQKVH--------FITDTLSKG--  223
DSSP  ----lEELLEEEEEEellllllllllhhHHHHHHHHHHHHLL--------LEEEEEEEL--


DSSP  llllLLLEEEEEEEEELL--EEEEEEEEEEEL-LLLLllllleeeeelhhhhllllllee
Query pgxpVKPRRFTAKLSYRG--RAFATVPIEVSSV-EAGNadqfdtltsdalglvgvpaava  175
ident              |             |
Sbjct ----ETKFMGVCQLPSKNdeKEYPHRRIDIRLIpKDQY----------------------  257
DSSP  ----LLEEEEEEELLLLLLllLLLLLLEEEEEEEEEeHHHH----------------------
```

```
DSSP  eeellhHHHHhhhhhhhhhlllllllllllhhhhHHHHHH--------------------
Query vpcxtiPWQIaqklhavtavleepkandrahdlvDLQLLE--------------------  215
ident 
Sbjct -----yCGVL--------------------yftgSDIFNKnmrahalekgftineytirp  292
DSSP  -----hHHHH--------------------hhhlLHHHHhhhhhhhhlllllllllllll


DSSP  --hhllhhhhhhhhhHHHHhhhhhhllllLLLLLlllllhhhhhhhhhllllllllllhh
Query --glllldadlxptrSACIaifearaqhpWPPRVatlphwpliyagalegldhlelartvd  273
ident               |
Sbjct lgvtgvageplpvdSEKD-------ifdYIQWK---------------------------  318
DSSP  llllllllllllllLHHH-------hhhHLLLL---------------------------


DSSP  hhhhhhhhhhhhhhhllll
Query aaaqavqrfvaridratkr  292
ident 
Sbjct ----------yrepkdrse  327
DSSP  ----------lllhhhlll
```

No 272: Query=mol1A Sbjct=7ictA Z-score=6.6

back to top

```
DSSP  ------------------------------------------------------------
Query ------------------------------------------------------------    0
ident 
Sbjct etlnggitdmltelanfeknvsqaihkynayrkaasviakyphkiksgaeakklpgvgtk   60
DSSP  llllhhhhhhhhhhhhhhhhllllhhhhhhhhhhhhhhhhlllllllhhhhhllllllhh


DSSP  -------------------------------------------------llllllllllh
Query -------------------------------------------------aapysspptnl   11
ident 
Sbjct iaekideflatgklrklekirqddtsssinfltrvsgigpsaarkfvdegiktledlrkn  120
DSSP  hhhhhhhhhhllllhhhhhhhhlhhhhhhhhhllllllhhhhhhhhhhhllllhhhhhhl


DSSP  hhhhhhhhhhhhhllllhhhhhHHHHHHHHHHHHHH-LLLLllLLLLEEEEHHHHHHHhhl
Query rslrdrltqvaerqgvvfgrlqRHVAXIVVAQFAA-TLTDdtGAPLLLVKGGSSLELrrg   70
ident                                                 | |
Sbjct edklnhhqriglkyfgdfekriPREEMLQMQDIVLnEVKKvdSEYIATVCGSFRRGA---  177
DSSP  hhhllhhhhhhhhlhhhhhlleEHHHHHHHHHHHHhHHHHhlLLLEEEELLLLLLLL---


DSSP  lllllLLLLEEEEEL------------lLHHHHHHHHHHHHhhlllleeEEELLLEELll
Query ipdsrTSKDFATVAR------------rDIELIHEQLADAGetgwegftAIFTAPEEIaa  118
ident       | |                         |||             |
Sbjct ----eSSGDMDVLLThpsftsestkqpkLLHQVVEQLQKVH--------FITDTLSKG--  223
DSSP  ----lEELLEEEEEEellllllllllllhHHHHHHHHHHHLL--------LEEEEEEEL--


DSSP  llllLLLEEEEEEEEELL--EEEEEEEEEEEEL-LLLLlllleeeeelhhhhlllllllee
Query pgxpVKPRRFTAKLSYRG--RAFATVPIEVSSV-EAGNadqfdtltsdalglvgvpaava  175
ident              |             |
Sbjct ----ETKFMGVCQLPSKNdeKEYPHRRIDIRLIpKDQY----------------------  257
DSSP  ----LLEEEEEELLLLLLllLLLLLEEEEEEEEeHHHH----------------------


DSSP  eeellhHHHHhhhhhhhhhlllllllllllhhhhHHHHHH--------------------
Query vpcxtiPWQIaqklhavtavleepkandrahdlvDLQLLE--------------------  215
ident 
Sbjct -----yCGVL--------------------yftgSDIFNKnmrahalekgftineytirp  292
DSSP  -----hHHHH--------------------hhllLHHHHhhhhhhhhlleeellllleee


DSSP  --hhllhhhhhhhhhHHHHhhhhhhllllLLLLLlllllhhhhhhhhhllllllllllhh
Query --glllldadlxptrSACIaifearaqhpWPPRVatlphwpliyagalegldhlelartvd  273
ident               |
Sbjct lgvtgvageplpvdSEKD-------ifdYIQWK---------------------------  318
DSSP  llllllllllllllLHHH-------hhhHLLLL---------------------------


DSSP  hhhhhhhhhhhhhhhllll
Query aaaqavqrfvaridratkr  292
ident 
```

```
DSSP  ----------lllhhhhll
```

No 273: Query=mol1A Sbjct=7icuA Z-score=6.6

back to top

```
DSSP  ------------------------------------------------------------
Query ------------------------------------------------------------    0
ident 
Sbjct etlnggitdmltelanfeknvsqaihkynayrkaasviakyphkiksgaeakklpgvgtk   60
DSSP  lllhhhhhhhhhhhhhhhhhllllhhhhhhhhhhhhhhhhlllllllhhhhhllllllhh


DSSP  -------------------------------------------------llllllllllh
Query -------------------------------------------------aapysspptnl   11
ident 
Sbjct iaekideflatgklrklekirqddtsssinfltrvsgigpsaarkfvdegiktledlrkn  120
DSSP  hhhhhhhhhhllllhhhhhhlllhhhhhhhhhhlllllhhhhhhhhhhlllllhhhhhhl


DSSP  hhhhhhhhhhhhhllllhhhhhHHHHHHHHHHHHHH-LLLLllLLLLEEEEHHHHHHHhhl
Query rslrdrltqvaerqgvvfgrlqRHVAXIVVAQFAA-TLTDdtGAPLLLVKGGSSLELrrg   70
ident                                                 | |
Sbjct edklnhhqriglkyfgdfekriPREEMLQMQDIVLnEVKKvdSEYIATVCGSFRRGA---  177
DSSP  hhhllhhhhhhhhlhhhhhlleEHHHHHHHHHHHHhHHHHhlLLLEEEELLLLLLLL---


DSSP  lllllLLLLEEEEEL------------lLHHHHHHHHHHHHhhlllleeEEELLLEELll
Query ipdsrTSKDFATVAR------------rDIELIHEQLADAGetgwegftAIFTAPEEIaa  118
ident       | |                         |||             |
Sbjct ----eSSGDMDVLLThpsftsestkqpkLLHQVVEQLQKVH--------FITDTLSKG--  223
DSSP  ----lEELLEEEEEEelllllllllllhHHHHHHHHHHHLL--------LEEEEEEEL--


DSSP  llllLLLEEEEEEEEELL--EEEEEEEEEEEEL-LLLLllllleeeeelhhhhllllllee
Query pgxpVKPRRFTAKLSYRG--RAFATVPIEVSSV-EAGNadqfdtltsdalglvgvpaava  175
ident              |             |
Sbjct ----ETKFMGVCQLPSKNdeKEYPHRRIDIRLIpKDQY----------------------  257
DSSP  ----LLEEEEEEELLLLLllLLLLLEEEEEEEEeHHHH----------------------


DSSP  eeellhHHHHHhhhhhhlllllllllllllhhhhHHHHHH--------------------
Query vpcxtiPWQIAQklhavtavleepkandrahdlvDLQLLE--------------------  215
ident 
Sbjct -----yCGVLYF--------------------tgSDIFNKnmrahalekgftineytirp  292
DSSP  -----hHHHHHL--------------------llLHHHHHhhhhhhhhlleeellllleee


DSSP  --hhllhhhhhhhhHHHHhhhhhhllllLLLLLlllllhhhhhhhhhllllllllllllhh
Query --glllldadlxptrSACIaifearaqhpWPPRVatlphwpliyagalegldhlelartvd  273
ident               |
Sbjct lgvtgvageplpvdSEKD-------ifdYIQWK---------------------------  318
DSSP  llllllllllllllLHHH-------hhhHLLLL---------------------------


DSSP  hhhhhhhhhhhhhhhllll
Query aaaqavqrfvaridratkr  292
ident 
Sbjct ----------yrepkdrse  327
DSSP  ----------lllhhhlll
```

No 274: Query=mol1A Sbjct=8ichA Z-score=6.6

back to top

```
DSSP  ------------------------------------------------------------
Query ------------------------------------------------------------    0
ident 
Sbjct etlnggitdmltelanfeknvsqaihkynayrkaasviakyphkiksgaeakklpgvgtk   60
DSSP  lllhhhhhhhhhhhhhhhhhllllhhhhhhhhhhhhhhhhlllllllhhhhhllllllhh


DSSP  -------------------------------------------------llllllllllh
```

```
ident
Sbjct iaekideflatgklrklekirqddtsssinfltrvsgigpsaarkfvdegiktledlrkn  120
DSSP  hhhhhhhhhhllllhhhhhhhhlhhhhhhhhhlllllllhhhhhhhhhhllllhhhhhhl


DSSP  hhhhhhhhhhhhhllllhhhhhHHHHHHHHHHHHH-LLLLllLLLLLEEEEHHHHHHHHhl
Query rslrdrltqvaerqgvvfgrlqRHVAXIVVAQFAA-TLTDdtGAPLLLVKGGSSLELrrg   70
ident                                                 | |
Sbjct edklnhhqriglkyfgdfekriPREEMLQMQDIVLnEVKKvdSEYIATVCGSFRRGA---  177
DSSP  hhhllhhhhhhhhlhhhhhlleEHHHHHHHHHHHHHhHHHHhlLLLEEEEELLLLLLLL---


DSSP  lllllLLLLEEEEEL------------lLHHHHHHHHHHHHhhlllleeEEELLLEELll
Query ipdsrTSKDFATVAR------------rDIELIHEQLADAGetgwegftAIFTAPEEIaa  118
ident       | |                         |||             |
Sbjct ----eSSGDMDVLLThpsftsestkqpkLLHQVVEQLQKVH--------FITDTLSKG--  223
DSSP  ----lEELLEEEEEEellllllllllhHHHHHHHHHHHHLL--------LEEEEEEEL--


DSSP  llllLLLEEEEEEEEELL--EEEEEEEEEEEEL-LLLLlllleeeeelhhhhllllllee
Query pgxpVKPRRFTAKLSYRG--RAFATVPIEVSSV-EAGNadqfdtltsdalglvgvpaava  175
ident              |             |
Sbjct ----ETKFMGVCQLPSKNdeKEYPHRRIDIRLIpKDQY----------------------  257
DSSP  ----LLEEEEEELLLLLllLLLLLEEEEEEEEeHHHH----------------------


DSSP  eeellhHHHHhhhhhhhhllllllllllhhhhHHHHHH--------------------
Query vpcxtiPWQIaqklhavtavleepkandrahdlvDLQLLE--------------------  215
ident
Sbjct -----yCGVL--------------------yftgSDIFNKnmrahalekgftineytirp  292
DSSP  -----hHHHH--------------------hhhlLHHHHHhhhhhhhhllllleelleell


DSSP  -hhllhhhhhhhHHHHHHhhhhhllllLLLLLlllllhhhhhhhhhllllllllllhhh
Query -glllddadlxptRSACIAifearaqhpWPPRVatlphwpliyagaleglldhlelartvda  274
ident
Sbjct lgvtgvageplpVDSEKD------ifdYIQWK----------------------------  318
DSSP  lllllllllllLLLHHH------hhhHLLLL----------------------------


DSSP  hhhhhhhhhhhhhhllll
Query aaqavqrfvaridratkr  292
ident
Sbjct ---------yrepkdrse  327
DSSP  ---------lllhhhlll
```

No 275: Query=mol1A Sbjct=8iciA Z-score=6.6

back to top

```
DSSP  ------------------------------------------------------------
Query ------------------------------------------------------------    0
ident
Sbjct etlnggitdmltelanfeknvsqaihkynayrkaasviakyphkiksgaeakklpgvgtk   60
DSSP  llllhhhhhhhhhhhhhhhlllllhhhhhhhhhhhhhhhlllllllhhhhhlllllllhh


DSSP  -------------------------------------------------llllllllllh
Query -------------------------------------------------aapysspptnl   11
ident
Sbjct iaekideflatgklrklekirqddtsssinfltrvsgigpsaarkfvdegiktledlrkn  120
DSSP  hhhhhhhhhhllllhhhhhhhhlhhhhhhhhhlllllllhhhhhhhhhhllllhhhhhhl


DSSP  hhhhhhhhhhhhhllllhhhhhHHHHHHHHHHHHH-LLLLllLLLLLEEEEHHHHHHHHhl
Query rslrdrltqvaerqgvvfgrlqRHVAXIVVAQFAA-TLTDdtGAPLLLVKGGSSLELrrg   70
ident                                                 | |
Sbjct edklnhhqriglkyfgdfekriPREEMLQMQDIVLnEVKKvdSEYIATVCGSFRRGA---  177
DSSP  hhhllhhhhhhhhlhhhhhlleEHHHHHHHHHHHHHhHHHHhlLLLEEEEELLLLLLLL---


DSSP  lllllLLLLEEEEEL------------lLHHHHHHHHHHHHhhlllleeEEELLLEELll
Query ipdsrTSKDFATVAR------------rDIELIHEQLADAGetgwegftAIFTAPEEIaa  118
ident       | |                         |||             |
Sbjct ----eSSGDMDVLLThpsftsestkqpkLLHQVVEQLQKVH--------FITDTLSKG--  223
DSSP  ----lEELLEEEEEEellllllllllhHHHHHHHHHHHHLL--------LEEEEEEEL--
```

```
DSSP  llllLLLEEEEEEEEELL--EEEEEEEEEEEEL-LLLLllllleeeeelhhhhlllllee
Query pgxpVKPRRFTAKLSYRG--RAFATVPIEVSSV-EAGNadqfdtltsdalglvgvpaava  175
ident              |             |
Sbjct ----ETKFMGVCQLPSKNdeKEYPHRRIDIRLIpKDQY----------------------  257
DSSP  ----LLEEEEEELLLLLLllLLLLLEEEEEEEEeHHHH----------------------


DSSP  eeellhHHHHhhhhhhhhllllllllllllhhhhHHHHHH--------------------
Query vpcxtiPWQIaqklhavtavleepkandrahdlvDLQLLE--------------------  215
ident
Sbjct -----yCGVL--------------------yftgSDIFNKnmrahalekgftineytirp  292
DSSP  -----hHHHH--------------------hhllLHHHHHhhhhhhhhlleeellllleee


DSSP  --hhllhhhhhhhhHHHHhhhhhhllllLLLLLlllllhhhhhhhhhlllllllllllhh
Query --gllldadlxptrSACIaifearaqhpWPPRVatlphwpliyagalegldhlelartvd  273
ident               |
Sbjct lgvtgvageplpvdSEKD-------ifdYIQWK---------------------------  318
DSSP  llllllllllllllLHHH-------hhhHLLLL---------------------------


DSSP  hhhhhhhhhhhhhhhllll
Query aaaqavqrfvaridratkr  292
ident
Sbjct ----------yrepkdrse  327
DSSP  ----------lllhhhlll
```

No 276: Query=mol1A Sbjct=8icpA Z-score=6.6

back to top

```
DSSP  ------------------------------------------------------------
Query ------------------------------------------------------------    0
ident
Sbjct etlnggitdmltelanfeknvsqaihkynayrkaasviakyphkiksgaeakklpgvgtk   60
DSSP  llllhhhhhhhhhhhhhhhhhllllhhhhhhhhhhhhhhhhlllllllhhhhhllllllhh


DSSP  ------------------------------------------------lllllllllllh
Query ------------------------------------------------aapyssppptnl   11
ident
Sbjct iaekideflatgklrklekirqddtsssinfltrvsgigpsaarkfvdegiktledlrkn  120
DSSP  hhhhhhhhhhllllhhhhhhhhlhhhhhhhhhllllllhhhhhhhhhhllllhhhhhhhl


DSSP  hhhhhhhhhhhhhllllhhhhhHHHHHHHHHHHHH-LLLLllLLLLEEEEHHHHHHHhhl
Query rslrdrltqvaerqgvvfgrlqRHVAXIVVAQFAA-TLTDdtGAPLLLVKGGSSLELrrg   70
ident                                                 | |
Sbjct edklnhhqriglkyfgdfekriPREEMLQMQDIVLnEVKKvdSEYIATVCGSFRRGA---  177
DSSP  hhhllhhhhhhhhlhhhhhlleEHHHHHHHHHHHHhHHHHhlLLLEEEEELLLLLLL---


DSSP  lllllLLLLEEEEEL------------lLHHHHHHHHHHHHhhlllleeEEELLLEELll
Query ipdsrTSKDFATVAR------------rDIELIHEQLADAGetgwegftAIFTAPEEIaa  118
ident       | |                         |||             |
Sbjct ----eSSGDMDVLLThpsftsestkqpkLLHQVVEQLQKVH--------FITDTLSKG--  223
DSSP  ----lEELLEEEEEEelllllllllllhHHHHHHHHHHHLL--------LEEEEEEEL--


DSSP  llllLLLEEEEEEEEELL--EEEEEEEEEEEEL-LLLLllllleeeeelhhhhlllllee
Query pgxpVKPRRFTAKLSYRG--RAFATVPIEVSSV-EAGNadqfdtltsdalglvgvpaava  175
ident              |             |
Sbjct ----ETKFMGVCQLPSKNdeKEYPHRRIDIRLIpKDQY----------------------  257
DSSP  ----LLEEEEEELLLLLLllLLLLLEEEEEEEEeHHHH----------------------


DSSP  eeellhHHHHhhhhhhhhllllllllllllhhhhHHHHHH--------------------
Query vpcxtiPWQIaqklhavtavleepkandrahdlvDLQLLE--------------------  215
ident
Sbjct -----yCGVL--------------------yftgSDIFNKnmrahalekgftineytirp  292
DSSP  -----hHHHH--------------------hhllLHHHHHhhhhhhhhllllllllllll


DSSP  -hhllhhhhhhhHHHHHHhhhhhllllLLLLLlllllhhhhhhhhhlllllllllllhhh
Query -gllldadlxptRSACIAifearaqhpWPPRVatlphwpliyagalegldhlelartvda  274
```

```
Sbjct lgvtgvageplpVDSEKD------ifdYIQWK----------------------------  318
DSSP  llllllllllllLLLHHH------hhhHLLLL----------------------------


DSSP  hhhhhhhhhhhhhllll
Query aaqavqrfvaridratkr  292
ident
Sbjct ---------yrepkdrse  327
DSSP  ---------lllhhhlll
```

No 277: Query=mol1A Sbjct=7icmA Z-score=6.6

back to top

```
DSSP  ------------------------------------------------------------
Query ------------------------------------------------------------    0
ident
Sbjct etlnggitdmltelanfeknvsqaihkynayrkaasviakyphkiksgaeakklpgvgtk   60
DSSP  llllhhhhhhhhhhhhhhhhllllhhhhhhhhhhhhhhhhlllllllhhhhhllllllhh


DSSP  -------------------------------------------------llllllllllh
Query -------------------------------------------------aapysspptnl   11
ident
Sbjct iaekideflatgklrklekirqddtsssinfltrvsgigpsaarkfvdegiktledlrkn  120
DSSP  hhhhhhhhhllllhhhhhhhhlhhhhhhhhhllllllhhhhhhhhlllllllhhhhhhl


DSSP  hhhhhhhhhhhhllllhhhhhHHHHHHHHHHHHH-LLLLllLLLLEEEEHHHHHHHHhhl
Query rslrdrltqvaerqgvvfgrlqRHVAXIVVAQFAA-TLTDdtGAPLLLVKGGSSLELrrg   70
ident                                                 | |
Sbjct edklnhhqriglkyfgdfekriPREEMLQMQDIVLnEVKKvdSEYIATVCGSFRRGA---  177
DSSP  hhhllhhhhhhhhlllhhhlleEHHHHHHHHHHHHHhHHHHhlLLLEEEELLHHHHLL---


DSSP  lllllLLLLEEEEEEL------------lLHHHHHHHHHHHHhhllllleeEEELLLEELll
Query ipdsrTSKDFATVAR------------rDIELIHEQLADAGetgwegftAIFTAPEEIaa  118
ident       | |                         |||             |
Sbjct ----eSSGDMDVLLThpsftsestkqpkLLHQVVEQLQKVH--------FITDTLSKG--  223
DSSP  ----lEELLEEEEEEellllllllllhHHHHHHHHHHHHHL--------LEEEEEEEL--


DSSP  llllLLLEEEEEEEEEELL--EEEEEEEEEEEEL-LLLLlllleeeeelhhhhlllllllee
Query pgxpVKPRRFTAKLSYRG--RAFATVPIEVSSV-EAGNadqfdtltsdalglvgvpaava  175
ident              |             |
Sbjct ----ETKFMGVCQLPSKNdeKEYPHRRIDIRLIpKDQY----------------------  257
DSSP  ----LLEEEEEEELLLLLLllLLLLLEEEEEEEEEeHHHH----------------------


DSSP  eeellhHHHHhhhhhhhhlllllllllllhhhhHHHHHH--------------------
Query vpcxtiPWQIaqklhavtavleepkandrahdlvDLQLLE--------------------  215
ident
Sbjct -----yCGVL--------------------yftgSDIFNKnmrahalekgftineytirp  292
DSSP  -----hHHHH--------------------hhhlLHHHHHhhhhhhhhlleeeelleeee


DSSP  -hhllhhhhhhhHHHHHHhhhhhllllLLLLLlllllhhhhhhhhhllllllllllllhhh
Query -glllldadlxptRSACIAifearaqhpWPPRVatlphwpliyagalegldhlelartvda  274
ident
Sbjct lgvtgvageplpVDSEKD------ifdYIQWK----------------------------  318
DSSP  llllllllllllLLLHHH------hhhHLLLL----------------------------


DSSP  hhhhhhhhhhhhhllll
Query aaqavqrfvaridratkr  292
ident
Sbjct ---------yrepkdrse  327
DSSP  ---------lllhhhhll
```

No 278: Query=mol1A Sbjct=1zqtA Z-score=6.6

back to top

```
Query ------------------------------------------------------------    0
ident
Sbjct etlnggitdmltelanfeknvsqaihkynayrkaasviakyphkiksgaeakklpgvgtk   60
DSSP  llllhhhhhhhhhhhhhhhllllhhhhhhhhhhhhhhhhlllllllhhhhhllllllhh


DSSP  -------------------------------------------------llllllllllh
Query -------------------------------------------------aapyssppptnl   11
ident
Sbjct iaekideflatgklrklekirqddtsssinfltrvsgigpsaarkfvdegiktledlrkn  120
DSSP  hhhhhhhhhhllllhhhhhhhhlhhhhhhhhhhllllllhhhhhhhhhhllllhhhhhhl


DSSP  hhhhhhhhhhhhhllllhhhhhHHHHHHHHHHHHH-LLLLllLLLLLEEEEHHHHHHHHhhl
Query rslrdrltqvaerqgvvfgrlqRHVAXIVVAQFAA-TLTDdtGAPLLLVKGGSSLELrrg   70
ident                                                 | |
Sbjct edklnhhqriglkyfgdfekriPREEMLQMQDIVLnEVKKvdSEYIATVCGSFRRGA---  177
DSSP  hhhllhhhhhhhhlhhhhhlleEHHHHHHHHHHHHHhHHHHhlLLLEEEEEELLLLLL---


DSSP  lllllLLLLEEEEEL------------lLHHHHHHHHHHHHHhhllllleeEEELLLEELll
Query ipdsrTSKDFATVAR------------rDIELIHEQLADAGetgwegftAIFTAPEEIaa  118
ident       | |                         |||             |
Sbjct ----eSSGDMDVLLThpsftsestkqpkLLHQVVEQLQKVH--------FITDTLSKG--  223
DSSP  ----lEELLEEEEEEellllllllllhhHHHHHHHHHHHHLL--------LEEEEEEEL--


DSSP  llllLLLEEEEEEEEEELL--EEEEEEEEEEEEL-LLLLllllleeeeelhhhhllllllee
Query pgxpVKPRRFTAKLSYRG--RAFATVPIEVSSV-EAGNadqfdtltsdalglvgvpaava  175
ident              |             |
Sbjct ----ETKFMGVCQLPSKNdeKEYPHRRIDIRLIpKDQY----------------------  257
DSSP  ----LLEEEEEEELLLLLLllLLLLLEEEEEEEEEeHHHH----------------------


DSSP  eeellhHHHHhhhhhhhhllllllllllhhhhHHHHHH--------------------
Query vpcxtiPWQIaqklhavtavleepkandrahdlvDLQLLE--------------------  215
ident
Sbjct -----yCGVL--------------------yftgSDIFNKnmrahalekgftineytirp  292
DSSP  -----hHHHH--------------------hhllLHHHHHhhhhhhhhlleeeelleeee


DSSP  --hhllhhhhhhhhHHHHhhhhhhllllLLLLLlllllhhhhhhhhhllllllllllhh
Query --gllldadlxptrSACIaifearaqhpWPPRVatlphwpliyagalegldhlelartvd  273
ident               |
Sbjct lgvtgvageplpvdSEKD-------ifdYIQWK---------------------------  318
DSSP  llllllllllllllLHHH-------hhhHHLLL---------------------------


DSSP  hhhhhhhhhhhhhhhllll
Query aaaqavqrfvaridratkr  292
ident
Sbjct ----------yrepkdrse  327
DSSP  ----------lllhhhlll
```

No 279: Query=mol1A Sbjct=8icxA Z-score=6.6

back to top

```
DSSP  ------------------------------------------------------------
Query ------------------------------------------------------------    0
ident
Sbjct etlnggitdmltelanfeknvsqaihkynayrkaasviakyphkiksgaeakklpgvgtk   60
DSSP  llllhhhhhhhhhhhhhhhlllllhhhhhhhhhhhhhhhhlllllllhhhhhllllllhh


DSSP  -------------------------------------------------llllllllllh
Query -------------------------------------------------aapyssppptnl   11
ident
Sbjct iaekideflatgklrklekirqddtsssinfltrvsgigpsaarkfvdegiktledlrkn  120
DSSP  hhhhhhhhhhllllhhhhhhhhlhhhhhhhhhhlllllllhhhhhhhhhhllllhhhhhhl


DSSP  hhhhhhhhhhhhhllllhhhhhHHHHHHHHHHHHH-LLLLllLLLLLEEEEHHHHHHHHhhl
Query rslrdrltqvaerqgvvfgrlqRHVAXIVVAQFAA-TLTDdtGAPLLLVKGGSSLELrrg   70
ident                                                 | |
Sbjct edklnhhqriglkyfgdfekriPREEMLQMQDIVLnEVKKvdSEYIATVCGSFRRGA---  177
```

```
DSSP  lllllLLLLEEEEEL------------lLHHHHHHHHHHHHhhlllleeEEELLLEELll
Query ipdsrTSKDFATVAR------------rDIELIHEQLADAGetgwegftAIFTAPEEIaa  118
ident       | |                         |||             |
Sbjct ----eSSGDMDVLLThpsftsestkqpkLLHQVVEQLQKVH--------FITDTLSKG--  223
DSSP  ----lEELLEEEEEElllllllllllhHHHHHHHHHHHLL--------LEEEEEEEL--


DSSP  llllLLLEEEEEEEEELL--EEEEEEEEEEEEL-LLLLlllleeeeelhhhhllllllee
Query pgxpVKPRRFTAKLSYRG--RAFATVPIEVSSV-EAGNadqfdtltsdalglvgvpaava  175
ident              |             |
Sbjct ----ETKFMGVCQLPSKNdeKEYPHRRIDIRLIpKDQY----------------------  257
DSSP  ----LLEEEEEEELLLLLllLLLLLLEEEEEELLlHHHH----------------------


DSSP  eeellhHHHHhhhhhhhhlllllllllllhhhhHHHHHH--------------------
Query vpcxtiPWQIaqklhavtavleepkandrahdlvDLQLLE--------------------  215
ident
Sbjct -----yCGVL--------------------yftgSDIFNKnmrahalekgftineytirp  292
DSSP  -----hHHHH--------------------hhhlLHHHHHhhhhhhhhlleeeelleeee


DSSP  -hhllhhhhhhhHHHHHhhhhhhllllLLLLLlllllhhhhhhhhhllllllllllhhh
Query -glllddadlxptRSACIaifearaqhpWPPRVatlphwpliyagalegldhlelartvda  274
ident
Sbjct lgvtgvageplpVDSEK-------difDYIQW----------------------------  317
DSSP  llllllllllllLLLHH-------hhhHHLLL----------------------------


DSSP  hhhhhhhhhhhhhhllll
Query aaqavqrfvaridratkr  292
ident
Sbjct --------kyrepkdrse  327
DSSP  --------llllhhhlll
```

No 280: Query=mol1A Sbjct=8icaA Z-score=6.6

back to top

```
DSSP  ------------------------------------------------------------
Query ------------------------------------------------------------    0
ident
Sbjct etlnggitdmltelanfeknvsqaihkynayrkaasviakyphkiksgaeakklpgvgtk   60
DSSP  llllhhhhhhhhhhhhhhhllllhhhhhhhhhhhhhhhhllllllhhhhhllllllhh


DSSP  -------------------------------------------------lllllllllh
Query -------------------------------------------------aapysspptnl   11
ident
Sbjct iaekideflatgklrklekirqddtsssinfltrvsgigpsaarkfvdegiktledlrkn  120
DSSP  hhhhhhhhhhllllhhhhhhhhlhhhhhhhhhllllllhhhhhhhhhhllllhhhhhhl


DSSP  hhhhhhhhhhhhllllhhhhhHHHHHHHHHHHHH-LLLLllLLLLEEEEHHHHHHHhhl
Query rslrdrltqvaerqggvvfgrlqRHVAXIVVAQFAA-TLTDdtGAPLLLVKGGSSLELrrg   70
ident                                                 | |
Sbjct edklnhhqriglkyfgdfekriPREEMLQMQDIVLnEVKKvdSEYIATVCGSFRRGA---  177
DSSP  hhhllhhhhhhhhlhhhhhlleEHHHHHHHHHHHHhHHHHhlLLLEEEELHHHHLLL---


DSSP  lllllLLLLEEEEEL------------lLHHHHHHHHHHHHhhlllleeEEELLLEELll
Query ipdsrTSKDFATVAR------------rDIELIHEQLADAGetgwegftAIFTAPEEIaa  118
ident       | |                         |||             |
Sbjct ----eSSGDMDVLLThpsftsestkqpkLLHQVVEQLQKVH--------FITDTLSKG--  223
DSSP  ----lEELLEEEEEEellllllllllhHHHHHHHHHHHLL--------LEEEEEEEL--


DSSP  llllLLLEEEEEEEEELL--EEEEEEEEEEEEL-LLLLlllleeeeelhhhhllllllee
Query pgxpVKPRRFTAKLSYRG--RAFATVPIEVSSV-EAGNadqfdtltsdalglvgvpaava  175
ident              |             |
Sbjct ----ETKFMGVCQLPSKNdeKEYPHRRIDIRLIpKDQY----------------------  257
DSSP  ----LLEEEEEEELLLLLllLLLLLLEEEEEEEeHHHH----------------------


DSSP  eeellhHHHHhhhhhhhhlllllllllllhhhhHHHHHH--------------------
```

```
ident
Sbjct -----yCGVL--------------------yftgSDIFNKnmrahalekgftineytirp  292
DSSP  -----hHHHH--------------------hhhlLHHHHHhhhhhhhhllllleelleell


DSSP  -hhllhhhhhhhHHHHHhhhhhhllllLLLLLllllhhhhhhhhhllllllllllhhh
Query -glllddadlxptRSACIaifearaqhpWPPRVatlphwpliyagalegldhlelartvda  274
ident
Sbjct lgvtgvageplpVDSEK-------difDYIQW----------------------------  317
DSSP  llllllllllllLLLHH-------hhhHHLLL----------------------------


DSSP  hhhhhhhhhhhhhhllll
Query aaqavqrfvaridratkr  292
ident
Sbjct --------kyrepkdrse  327
DSSP  --------llllhhhlll
```

No 281: Query=mol1A Sbjct=8icnA Z-score=6.6

back to top

```
DSSP  ------------------------------------------------------------
Query ------------------------------------------------------------    0
ident
Sbjct etlnggitdmltelanfeknvsqaihkynayrkaasviakyphkiksgaeakklpgvgtk   60
DSSP  llllhhhhhhhhhhhhhhhhllllhhhhhhhhhhhhhhhhlllllllhhhhhlllllllhh


DSSP  -------------------------------------------------llllllllllh
Query -------------------------------------------------aapysspptnl   11
ident
Sbjct iaekideflatgklrklekirqddtsssinfltrvsgigpsaarkfvdegiktledlrkn  120
DSSP  hhhhhhhhhhllllhhhhhhhhlhhhhhhhhhllllllhhhhhhhhhhllllhhhhhhl


DSSP  hhhhhhhhhhhhhllllhhhhhHHHHHHHHHHHHH-LLLLllLLLLLEEEEHHHHHHHHhhl
Query rslrdrltqvaerqgvvfgrlqRHVAXIVVAQFAA-TLTDdtGAPLLLVKGGSSLELrrg   70
ident                                                 | |
Sbjct edklnhhqriglkyfgdfekriPREEMLQMQDIVLnEVKKvdSEYIATVCGSFRRGA---  177
DSSP  hhhllhhhhhhhhlhhhhhlleEHHHHHHHHHHHHHhHHHHhlLLLEEEELLLLLLL---


DSSP  lllllLLLLEEEEEL------------lLHHHHHHHHHHHHHhhlllleeEEELLLEELll
Query ipdsrTSKDFATVAR------------rDIELIHEQLADAGetgwegftAIFTAPEEIaa  118
ident       | |                         |||             |
Sbjct ----eSSGDMDVLLThpsftsestkqpkLLHQVVEQLQKVH--------FITDTLSKG--  223
DSSP  ----lEELLEEEEEEellllllllllhHHHHHHHHHHHHLL--------LEEEEEEEL--


DSSP  llllLLLEEEEEEEEEELL--EEEEEEEEEEEEL-LLLLllllleeeeelhhhhllllllee
Query pgxpVKPRRFTAKLSYRG--RAFATVPIEVSSV-EAGNadqfdtltsdalglvgvpaava  175
ident              |             |
Sbjct ----ETKFMGVCQLPSKNdeKEYPHRRIDIRLIpKDQY----------------------  257
DSSP  ----LLEEEEEELLLLLllLLLLLLEEEEEEEEEeHHHH----------------------


DSSP  eeellhHHHHhhhhhhhhlllllllllllhhhhHHHHHH--------------------
Query vpcxtiPWQIaqklhavtavleepkandrahdlvDLQLLE--------------------  215
ident
Sbjct -----yCGVL--------------------yftgSDIFNKnmrahalekgftineytirp  292
DSSP  -----hHHHH--------------------hhhlLHHHHHhhhhhhhhllllleelleell


DSSP  --hhllhhhhhhhhHHHHhhhhhhllllLLLLLllllhhhhhhhhhllllllllllhh
Query --glllddadlxptrSACIaifearaqhpWPPRVatlphwpliyagalegldhlelartvd  273
ident               |
Sbjct lgvtgvageplpvdSEKD-------ifdYIQWK---------------------------  318
DSSP  llllllllllllllLHHH-------hhhHHLLL---------------------------


DSSP  hhhhhhhhhhhhhhhllll
Query aaaqavqrfvaridratkr  292
ident
Sbjct ----------yrepkdrse  327
DSSP  ----------lllhhhlll
```

No 282: Query=mol1A Sbjct=8iczA Z-score=6.6

back to top

```
DSSP  ------------------------------------------------------------
Query ------------------------------------------------------------   0
ident 
Sbjct etlnggitdmltelanfeknvsqaihkynayrkaasviakyphkiksgaeakklpgvgtk   60
DSSP  llllhhhhhhhhhhhhhhhhllllhhhhhhhhhhhhhhhhlllllllhhhhhlllllhh


DSSP  -------------------------------------------------lllllllllh
Query -------------------------------------------------aapyssppptnl   11
ident 
Sbjct iaekideflatgklrklekirqddtsssinfltrvsgigpsaarkfvdegiktledlrkn  120
DSSP  hhhhhhhhhhllllhhhhhhhhlhhhhhhhhhlllllllhhhhhhhhlllllllhhhhhhl


DSSP  hhhhhhhhhhhhllllhhhhhHHHHHHHHHHHHH-LLLLllLLLLEEEEHHHHHHHhhl
Query rslrdrltqvaerqgvvfgrlqRHVAXIVVAQFAA-TLTDdtGAPLLLVKGGSSLELrrg   70
ident                                                 | |
Sbjct edklnhhqriglkyfgdfekriPREEMLQMQDIVLnEVKKvdSEYIATVCGSFRRGA---  177
DSSP  hhhllhhhhhhhhllllllleEHHHHHHHHHHHHHhHHHHhlLLLEEEELLLLLLL---


DSSP  lllllLLLLEEEEEL------------lLHHHHHHHHHHHHHhhlllleeEEELLLEELll
Query ipdsrTSKDFATVAR------------rDIELIHEQLADAGetgwegftAIFTAPEEIaa  118
ident       | |                         |||             |
Sbjct ----eSSGDMDVLLThpsftsestkqpkLLHQVVEQLQKVH--------FITDTLSKG--  223
DSSP  ----lEELLEEEEEEelllllllllhhHHHHHHHHHHHHLL--------LEEEEEEEE--


DSSP  llllLLLEEEEEEEEEELL--EEEEEEEEEEEEL-LLLLlllleeeeelhhhhllllllee
Query pgxpVKPRRFTAKLSYRG--RAFATVPIEVSSV-EAGNadqfdtltsdalglvgvpaava  175
ident              |             |
Sbjct ----ETKFMGVCQLPSKNdeKEYPHRRIDIRLIpKDQY----------------------  257
DSSP  ----LLEEEEEELLLLLLllLLLLLLEEEEEEEEEeHHHH----------------------


DSSP  eeellhHHHHhhhhhhhhlllllllllllhhhhHHHHHH--------------------
Query vpcxtiPWQIaqklhavtavleepkandrahdlvDLQLLE--------------------  215
ident 
Sbjct -----yCGVL--------------------yftgSDIFNKnmrahalekgftineytirp  292
DSSP  -----hHHHH--------------------hhllLHHHHHhhhhhhhhllllleelleell


DSSP  --hhllhhhhhhhhHHHHhhhhhhllllLLLLLlllllhhhhhhhhhllllllllllhh
Query --glllldadlxptrSACIaifearaqhpWPPRVatlphwpliyagalegldhlelartvd  273
ident               |
Sbjct lgvtgvageplpvdSEKD-------ifdYIQWK---------------------------  318
DSSP  llllllllllllllLHHH-------hhhHLLLL---------------------------


DSSP  hhhhhhhhhhhhhhhllll
Query aaaqavqrfvaridratkr  292
ident 
Sbjct ----------yrepkdrse  327
DSSP  ----------lllhhhlll
```

No 283: Query=mol1A Sbjct=9icbA Z-score=6.6

back to top

```
DSSP  ------------------------------------------------------------
Query ------------------------------------------------------------   0
ident 
Sbjct etlnggitdmltelanfeknvsqaihkynayrkaasviakyphkiksgaeakklpgvgtk   60
DSSP  llllhhhhhhhhhhhhhhhhllllhhhhhhhhhhhhhhhhlllllllhhhhhlllllhh


DSSP  -------------------------------------------------lllllllllh
Query -------------------------------------------------aapyssppptnl   11
ident 
```

```
DSSP  hhhhhhhhhhllllhhhhhhhhlhhhhhhhhhllllllhhhhhhhhhllllllhhhhhhl


DSSP  hhhhhhhhhhhhhllllhhhhhHHHHHHHHHHHHHH-LLLLllLLLLLEEEEHHHHHHHHhhl
Query rslrdrltqvaerqgvvfgrlqRHVAXIVVAQFAA-TLTDdtGAPLLLVKGGSSLELrrg   70
ident                                                 | |
Sbjct edklnhhqriglkyfgdfekriPREEMLQMQDIVLnEVKKvdSEYIATVCGSFRRGA---  177
DSSP  hhhllhhhhhhhhhlhhhhhlleEHHHHHHHHHHHHHhHHHHhlLLLEEEELLLLLLLL---


DSSP  lllllLLLLEEEEEL------------lLHHHHHHHHHHHHHhhllllleeEEELLLEELll
Query ipdsrTSKDFATVAR------------rDIELIHEQLADAGetgwegftAIFTAPEEIaa  118
ident       | |                         |||             |
Sbjct ----eSSGDMDVLLThpsftsestkqpkLLHQVVEQLQKVH--------FITDTLSKG--  223
DSSP  ----lEELLEEEEEEellllllllllhHHHHHHHHHHHHLL--------LEEEEEEEL--


DSSP  llllLLLEEEEEEEEEELL--EEEEEEEEEEEEL-LLLLllllleeeeelhhhhllllllee
Query pgxpVKPRRFTAKLSYRG--RAFATVPIEVSSV-EAGNadqfdtltsdalglvgvpaava  175
ident              |             |
Sbjct ----ETKFMGVCQLPSKNdeKEYPHRRIDIRLIpKDQY----------------------  257
DSSP  ----LLEEEEEEELLLLLllLLLLLEEEEEEEEEeHHHH----------------------


DSSP  eeellhHHHHhhhhhhhhlllllllllllhhhhHHHHHH--------------------
Query vpcxtiPWQIaqklhavtavleepkandrahdlvDLQLLE--------------------  215
ident
Sbjct -----yCGVL--------------------yftgSDIFNKnmrahalekgftineytirp  292
DSSP  -----hHHHH--------------------hhhlLHHHHHhhhhhhhhhlleeeelleeee


DSSP  --hhllhhhhhhhhHHHHhhhhhhllllLLLLLllllllhhhhhhhhhlllllllllllhh
Query --glllldadlxptrSACIaifearaqhpWPPRVatlphwpliyagalegldhlelartvd  273
ident               |
Sbjct lgvtgvageplpvdSEKD-------ifdYIQWK---------------------------  318
DSSP  llllllllllllllLHHH-------hhhHLLLL---------------------------


DSSP  hhhhhhhhhhhhhhhllll
Query aaaqavqrfvaridratkr  292
ident
Sbjct ----------yrepkdrse  327
DSSP  ----------lllhhhlll
```

No 284: Query=mol1A Sbjct=9ichA Z-score=6.6

back to top

```
DSSP  ------------------------------------------------------------
Query ------------------------------------------------------------    0
ident
Sbjct etlnggitdmltelanfeknvsqaihkynayrkaasviakyphkiksgaeakklpgvgtk   60
DSSP  llllhhhhhhhhhhhhhhhhllllhhhhhhhhhhhhhhhhlllllllhhhhhlllllllhh


DSSP  --------------------------------------------------llllllllllh
Query --------------------------------------------------aapysspptnl   11
ident
Sbjct iaekideflatgklrklekirqddtsssinfltrvsgigpsaarkfvdegiktledlrkn  120
DSSP  hhhhhhhhhhllllhhhhhhhhlhhhhhhhhhllllllhhhhhhhhhllllllhhhhhhl


DSSP  hhhhhhhhhhhhhllllhhhhhHHHHHHHHHHHHHH-LLLLllLLLLLEEEEHHHHHHHHhhl
Query rslrdrltqvaerqgvvfgrlqRHVAXIVVAQFAA-TLTDdtGAPLLLVKGGSSLELrrg   70
ident                                                 | |
Sbjct edklnhhqriglkyfgdfekriPREEMLQMQDIVLnEVKKvdSEYIATVCGSFRRGA---  177
DSSP  hhhllhhhhhhhhhlhhhhhlleEHHHHHHHHHHHHHhHHHHhlLLLEEEELLLLLLLL---


DSSP  lllllLLLLEEEEEL------------lLHHHHHHHHHHHHHhhllllleeEEELLLEELll
Query ipdsrTSKDFATVAR------------rDIELIHEQLADAGetgwegftAIFTAPEEIaa  118
ident       | |                         |||             |
Sbjct ----eSSGDMDVLLThpsftsestkqpkLLHQVVEQLQKVH--------FITDTLSKG--  223
DSSP  ----lEELLEEEEEEellllllllllhHHHHHHHHHHHHLL--------LEEEEEEEL--
```

```
Query pgxpVKPRRFTAKLSYRG--RAFATVPIEVSSV-EAGNadqfdtltsdalglvgvpaava  175
ident              |             |
Sbjct ----ETKFMGVCQLPSKNdeKEYPHRRIDIRLIpKDQY----------------------  257
DSSP  ----LLEEEEEELLLLLLllLLLLLLEEEEEEEEEeHHHH----------------------


DSSP  eeellhHHHHhhhhhhhhllllllllllllhhhhHHHHHH--------------------
Query vpcxtiPWQIaqklhavtavleepkandrahdlvDLQLLE--------------------  215
ident
Sbjct -----yCGVL--------------------yftgSDIFNKnmrahalekgftineytirp  292
DSSP  -----hHHHH--------------------hhhlLHHHHHhhhhhhhhhlleeeelleeee


DSSP  -hhllhhhhhhhHHHHHhhhhhhllllLLLLLLllllhhhhhhhhhllllllllllhhh
Query -glllddadlxptRSACIaifearaqhpWPPRVAtlphwpliyagalegldhlelartvda  274
ident
Sbjct lgvtgvageplpVDSEK-------difDYIQWK---------------------------  318
DSSP  llllllllllllLLLHH-------hhhHHLLLL---------------------------


DSSP  hhhhhhhhhhhhhhllll
Query aaqavqrfvaridratkr  292
ident
Sbjct ---------yrepkdrse  327
DSSP  ---------lllhhhlll
```

No 285: Query=mol1A Sbjct=9icfA Z-score=6.6

back to top

```
DSSP  ------------------------------------------------------------
Query ------------------------------------------------------------    0
ident
Sbjct etlnggitdmltelanfeknvsqaihkynayrkaasviakyphkiksgaeakklpgvgtk   60
DSSP  llllhhhhhhhhhhhhhhhhhllllhhhhhhhhhhhhhhhhllllllhhhhhlllllllhh


DSSP  -------------------------------------------------llllllllllh
Query -------------------------------------------------aapysspptnl   11
ident
Sbjct iaekideflatgklrklekirqddtsssinfltrvsgigpsaarkfvdegiktledlrkn  120
DSSP  hhhhhhhhhhllllhhhhhhhhlhhhhhhhhhllllllhhhhhhhhhhhllllhhhhhhl


DSSP  hhhhhhhhhhhhlllhhhhhHHHHHHHHHHHHH-LLLLllLLLLEEEEHHHHHHHHhhl
Query rslrdrltqvaerqggvvfgrlqRHVAXIVVAQFAA-TLTDdtGAPLLLVKGGSSLELrrg   70
ident                                                 | |
Sbjct edklnhhqriglkyfgdfekriPREEMLQMQDIVLnEVKKvdSEYIATVCGSFRRGA---  177
DSSP  hhhllhhhhhhhhlhhhhhlleEHHHHHHHHHHHHHhHHHHhlLLLEEEELLLLLLL---


DSSP  lllllLLLLEEEEEL------------lLHHHHHHHHHHHHhhllllleeEEELLLEELll
Query ipdsrTSKDFATVAR------------rDIELIHEQLADAGetgwegftAIFTAPEEIaa  118
ident       | |                         |||             |
Sbjct ----eSSGDMDVLLThpsftsestkqpkLLHQVVEQLQKVH--------FITDTLSKG--  223
DSSP  ----lEELLEEEEEEellllllllllhHHHHHHHHHHHHLL--------LEEEEEEEL--


DSSP  llllLLLEEEEEEEEEELL--EEEEEEEEEEEEL-LLLLllllleeeeelhhhhllllllee
Query pgxpVKPRRFTAKLSYRG--RAFATVPIEVSSV-EAGNadqfdtltsdalglvgvpaava  175
ident              |             |
Sbjct ----ETKFMGVCQLPSKNdeKEYPHRRIDIRLIpKDQY----------------------  257
DSSP  ----LLEEEEEEELLLLLLllLLLLLLEEEEEEEEEeHHHH----------------------


DSSP  eeellhHHHHhhhhhhhhllllllllllllhhhhHHHHHH--------------------
Query vpcxtiPWQIaqklhavtavleepkandrahdlvDLQLLE--------------------  215
ident
Sbjct -----yCGVL--------------------yftgSDIFNKnmrahalekgftineytirp  292
DSSP  -----hHHHH--------------------hhhlLHHHHHhhhhhhhhhlleeeelleeee


DSSP  -hhllhhhhhhhHHHHHhhhhhhllllLLLLLLllllhhhhhhhhhllllllllllhhh
Query -glllddadlxptRSACIAifearaqhpWPPRVatlphwpliyagalegldhlelartvda  274
ident
Sbjct lgvtgvageplpVDSEKD------ifdYIQWK----------------------------  318
```

```
DSSP  hhhhhhhhhhhhhhllll
Query aaqavqrfvaridratkr  292
ident
Sbjct ---------yrepkdrse  327
DSSP  ---------lllllllll
```

No 286: Query=mol1A Sbjct=4phpA Z-score=6.6

back to top

```
DSSP  ------------------------------------------------------------
Query ------------------------------------------------------------    0
ident
Sbjct tlnggitdmltelanfeknvsqaihkynayrkaasviakyphkiksgaeakklpgvgtki   60
DSSP  lllhhhhhhhhhhhhhhhhllllhhhhhhhhhhhhhhhhhlllllllhhhhhlllllhhh


DSSP  -----------------------------------------------lllllllllllhh
Query -----------------------------------------------aapyssppptnlr   12
ident
Sbjct aekideflatgklrklekirqddtsssinfltrvsgigpsaarkfvdegiktledlrkne  120
DSSP  hhhhhhhhhhlllhhhhhhhhhlhhhhhhhhhhllllllhhhhhhhhlllllhhhhhhlh


DSSP  hhhhhhhhhhhhllllhhhhhHHHHHHHHHHHHHH-LLLLllLLLLEEEEHHHHHHHhhll
Query slrdrltqvaerqgvvfgrlqRHVAXIVVAQFAA-TLTDdtGAPLLLVKGGSSLELrrgi   71
ident                                                |  |
Sbjct dklnhhqriglkyfgdfekriPREEMLQMQDIVLnEVKKvdSEYIATVCGSFRRGA----  176
DSSP  hhllhhhhhhhhlllhhhlleEHHHHHHHHHHHHhHHHHhlLLLEEEELHHHHLLL----


DSSP  llllLLLLEEEEEL------------lLHHHHHHHHHHHHhhlllleeEEELLLEEllll
Query pdsrTSKDFATVAR------------rDIELIHEQLADAGetgwegftAIFTAPEEiaap  119
ident      | |                         |||             |
Sbjct ---eSSGDMDVLLThpsftsestkqpkLLHQVVEQLQKVH--------FITDTLSK----  221
DSSP  ---lEELLEEEEEElllllllllllllLLHHHHHHHHHLL--------LEEEEEEE----


DSSP  lllLLLEEEEEEEEEL--LEEEEEEEEEEEEL-LLLLlllleeeeelhhhhllllleee
Query gxpVKPRRFTAKLSYR--GRAFATVPIEVSSV-EAGNadqfdtltsdalglvgvpaavav  176
ident             |             |
Sbjct --gETKFMGVCQLPSKndEKEYPHRRIDIRLIpKDQY-----------------------  256
DSSP  --lLLEEEEEELLLLLllLLLLLLEEEEEEELlHHHH-----------------------


DSSP  eellhHHHHhhhhhhhhllllllllllllHHHH-------------------hhhhhhhh
Query pcxtiPWQIaqklhavtavleepkandraHDLV-------------------dlqllegl  217
ident                               |
Sbjct ----yCGVL----------------yftgSDIFnknmrahalekgftineytirplgvtg  296
DSSP  ----hHHHH----------------hhhlLHHHhhhhhhhhhhhleeellllleeelllll


DSSP  llhHHHHHhhHHHHhhhhhhlllLLLLLLlllllhhhhhhhhhlllllllllllhhhhhh
Query lldADLXPtrSACIaifearaqhPWPPRVatlphwpliyagalegldhlelartvdaaaq  277
ident      |
Sbjct vagEPLPV--DSEK-----difdYIQWKY-------------------------------  318
DSSP  lllLLLLL--LLHH-----hhhhHLLLLL-------------------------------


DSSP  hhhhhhhhhhhllll
Query avqrfvaridratkr  292
ident
Sbjct -------repkdrse  326
DSSP  -------llhhhlll
```

No 287: Query=mol1A Sbjct=1zquA Z-score=6.6

back to top

```
DSSP  --------------------------lllllllllllhhhhhhhhhhhhhhllllhhhhh
Query --------------------------aapyssppptnlrslrdrltqvaerqgvvfgrlq   33
```

```
Sbjct ddtsssinfltrvtgigpsaarklvdegiktledlrknedklnhhqriglkyfedfekri   60
DSSP  lllhhhhhhhlllllllhhhhhhhhhlllllhhhhhllhhhllhhhhhhhhlllhhhlle


DSSP  HHHHHHHHHHHHHLL-LLLLLLLEEEEHHHHHHHHhhllllllLLLLEEEEEL-------
Query RHVAXIVVAQFAATL-TDDTGAPLLLVKGGSSLELrrgipdsrTSKDFATVAR-------   85
ident                           | |               | |
Sbjct PREEMLQMQDIVLNEvKKLDPEYIATVCGSFRRGA-------eSSGDMDVLLThpnftse  113
DSSP  EHHHHHHHHHHHHHHhHHHLLLLEEEELHHHHLLL-------lEELLEEEEEElllllll


DSSP  -----lLHHHHHHHHHHHHhhlllleeEEELLLEELllllllLLLEEEEEEEEELLeEEE
Query -----rDIELIHEQLADAGetgwegftAIFTAPEEIaapgxpVKPRRFTAKLSYRGrAFA  140
ident             |||             |                      |
Sbjct sskqpkLLHRVVEQLQKVR--------FITDTLSKG------ETKFMGVCQLPSEN-EYP  158
DSSP  llllhhHHHHHHHHHHHLL--------LEEEEEEEL------LLEEEEEELLLLLL-LLL


DSSP  EEEEEEEEL-LLLLlllleeeeelhhhhlllllleeeeellhHHHHhhhhhhhhllllll
Query TVPIEVSSV-EAGNadqfdtltsdalglvgvpaavavpcxtiPWQIaqklhavtavleep  199
ident    |
Sbjct HRRIDIRLIpKDQY---------------------------yCGVL--------------  177
DSSP  LEEEEEEELlHHHH---------------------------hHHHH--------------


DSSP  llllllhhhhHHHHHH---------------------hhllhhhhhhhhhHHHHhhhhhh
Query kandrahdlvDLQLLE---------------------glllddadlxptrSACIaifear  237
ident                                                   |
Sbjct ------yftgSDIFNKnmrahalekgftineytirplgvtgvageplpvdSEQD------  225
DSSP  ------hhhlLHHHHHhhhhhhhhlleeellllleeellllllllllllllLHHH------


DSSP  llllLLLLLlllllhhhhhhhhhllllllllllllhhhhhhhhhhhhhhhhhllll
Query aqhpWPPRVatlphwpliyagalegldhlelartvdaaaqavqrfvaridratkr  292
ident
Sbjct -ifdYIQWR-------------------------------------yrepkdrse  242
DSSP  -hhhHLLLL-------------------------------------lllhhhhll
```

No 288: Query=mol1A Sbjct=1xslI Z-score=6.6

back to top

```
DSSP  ------------------------------------------------------------
Query ------------------------------------------------------------    0
ident
Sbjct tnhnlhiteklevlakaysvqgdkwralgyakainalksfhkpvtsyqeacsipgigkrm   60
DSSP  llllhhhhhhhhhhhhhhhhlllhhhhhhhhhhhhhhhlllllllhhhhhlllllllhhh


DSSP  ---------------------------------------------llllllllllhhhhh
Query ---------------------------------------------aapysspptnlrslr   15
ident
Sbjct aekiieilesghlrkldhisesvpvlelfsniwgagtktaqmwyqqgfrsledirsqasl  120
DSSP  hhhhhhhhhhlllhhhhhllllhhhhhhhhlllllhhhhhhhhhhlllllhhhhhhhlll


DSSP  hhhhhhhhllllhHHHHHHHHHHHHHHHHHHL-LLLLlLLLEEEEHHHHHHHHhhlllll
Query drltqvaerqgvvfGRLQRHVAXIVVAQFAAT-LTDDtGAPLLLVKGGSSLELrrgipds   74
ident                            |             |    |
Sbjct ttqqaiglkhysdfLERMPREEATEIEQTVQKaAQAFnSGLLCVACGSYRRGK-------  173
DSSP  lhhhhhhhlhhhhHLLEEHHHHHHHHHHHHHHhHLLLlLLLEEEELHHHHLLL-------


DSSP  lLLLLEEEEEL--------lLHHHHHHHHHHHHhhlllleeEEELLLE-ELLLllLLLLL
Query rTSKDFATVAR--------rDIELIHEQLADAGetgwegftAIFTAPE-EIAApgXPVKP  125
ident  |  |                       |   |
Sbjct aTCGDVDVLIThpdgrshrgIFSRLLDSLRQEG--------FLTDDLVsQEEN--GQQQK  223
DSSP  lEELLEEEEEElllllllllHHHHHHHHHHHLL--------LEEEEEEeEEEE--LLEEE


DSSP  EEEEEEEEEllEEEEEEEEEEEEL-LLLLlllleeeeelhhhhlllllleeeeellhHHH
Query RRFTAKLSYrgRAFATVPIEVSSV-EAGNadqfdtltsdalglvgvpaavavpcxtiPWQ  184
ident       |                |
Sbjct YLGVCRLPG--PGRRHRRLDIIVVpYSEF---------------------------aCAL  254
DSSP  EEEEELLLL--LLLLLEEEEEEELlHHHH---------------------------hHHH
```

```
DSSP  Hhhhhhhhhllllllllllll HHHHHHH-------------------------hhhhhll
Query Iaqklhavtavleepkandra HDLVDLQ-------------------------lleglll  219
ident
Sbjct L----------------yftgSAHFNRSmralaktkgmslsehalstavvrnthgckvgp  298
DSSP  H----------------hhhlLHHHHHHhhhhhhhlleeellllleeelleellllleeel


DSSP  hhHHHHhhHHHHHhhhhhlllLLLLLlllllhhhhhhhhhllllllllllhhhhhhh
Query daDLXPtrSACIAifearaqhPWPPRvatlphwpliyagalegldhlelartvdaaaqav  279
ident    |
Sbjct grVLPT--PTEKD-----vfrLLGLP----------------------------------  317
DSSP  llEELL--LLHHH-----hhhHLLLL----------------------------------


DSSP  hhhhhhhhhllll
Query qrfvaridratkr  292
ident
Sbjct ----yrepaerdw  326
DSSP  ----lllhhhlll
```

No 289: Query=mol1A Sbjct=2gwsM Z-score=6.6

back to top

```
DSSP  ------------------------------------------------------------
Query ------------------------------------------------------------    0
ident
Sbjct tnhnlhiteklevlakaysvqgdkwralgyakainalksfhkpvtsyqeacsipgigkrm   60
DSSP  llllhhhhhhhhhhhhhhhhhllhhhhhhhhhhhhhhhhlllllllhhhhhhlllllhhh


DSSP  ---------------------------------------------llllllllllhhhhh
Query ---------------------------------------------aapysspptnlrslr   15
ident
Sbjct aekiieilesghlrkldhisesvpvlelfsniwgagtktaqmwyqqgfrsledirsqasl  120
DSSP  hhhhhhhhhlllhhhhhlllhhhhhhhhlllllhhhhhhhhhlllllhhhhhhhhlll


DSSP  hhhhhhhhllllhHHHHHHHHHHHHHHHHHHLL-LLLLLLLLEEEEHHHHHHHhhlllll
Query drltqvaerqgvvfGRLQRHVAXIVVAQFAATL-TDDTGAPLLLVKGGSSLELrrgipds   74
ident                            |             |    |
Sbjct ttqqaiglkhysdfLERMPREEATEIEQTVQKAaQAFNSGLLCVACGSYRRGK-------  173
DSSP  lhhhhhhhhlhhhhHLLEEHHHHHHHHHHHHHHHhHLLLLLLEEEELHHHHLLL-------


DSSP  lLLLLEEEEEL--------lLHHHHHHHHHHHHhhlllleeEEELLLE-ELLLllLLLLL
Query rTSKDFATVAR--------rDIELIHEQLADAGetgwegftAIFTAPE-EIAApgXPVKP  125
ident  |  |                       |   |
Sbjct aTCGDVDVLIThpdgrshrgIFSRLLDSLRQEG--------FLTDDLVsQEEN--GQQQK  223
DSSP  lEELLEEEEEEllllllllLHHHHHHHHHHHLL--------LEEEEEEeEEEE--LLEEE


DSSP  EEEEEEEEEllEEEEEEEEEEEEL-LLLLlllleeeeelhhhhllllllleeeeellhHHH
Query RRFTAKLSYrgRAFATVPIEVSSV-EAGNadqfdtltsdalglvgvpaavavpcxtiPWQ  184
ident       |                |
Sbjct YLGVCRLPG--PGRRHRRLDIIVVpYSEF---------------------------aCAL  254
DSSP  EEEEELLLL--LLLLLEEEEEEELlHHHH---------------------------hHHH


DSSP  Hhhhhhhhhllllllllllll HHHHHHH-------------------------hhhhhll
Query Iaqklhavtavleepkandra HDLVDLQ-------------------------lleglll  219
ident
Sbjct L----------------yftgSAHFNRSmralaktkgmslsehalstavvrnthgckvgp  298
DSSP  H----------------hhhlLHHHHHHhhhhhhhlleeellllleeelleellllleeel


DSSP  hHHHHhhHHHHHhhhhhhllllLLLLLlllllhhhhhhhhhllllllllllhhhhhhh
Query dADLXptRSACIaifearaqhpWPPRVatlphwpliyagalegldhlelartvdaaaqav  279
ident    |
Sbjct gRVLP--TPTEK------dvfrLLGLP---------------------------------  317
DSSP  lLEEL--LLLHH------hhhhHLLLL---------------------------------


DSSP  hhhhhhhhhllll
Query qrfvaridratkr  292
ident
```

```
DSSP  ----lllhhhlll
```

No 290: Query=mol1A Sbjct=8icuA Z-score=6.6

back to top

```
DSSP  ------------------------------------------------------------
Query ------------------------------------------------------------    0
ident 
Sbjct etlnggitdmltelanfeknvsqaihkynayrkaasviakyphkiksgaeakklpgvgtk   60
DSSP  llllhhhhhhhhhhhhhhhhhlllhhhhhhhhhhhhhhhhllllllllhhhhhllllllhh


DSSP  -------------------------------------------------llllllllllh
Query -------------------------------------------------aapysspptnl   11
ident 
Sbjct iaekideflatgklrklekirqddtsssinfltrvsgigpsaarkfvdegiktledlrkn  120
DSSP  hhhhhhhhhhllllhhhhhhhhlhhhhhhhhhlllllllhhhhhhhhhhllllhhhhhhl


DSSP  hhhhhhhhhhhhhllllhhhhhHHHHHHHHHHHHH-LLLLllLLLLLEEEEHHHHHHHhhl
Query rslrdrltqvaerqggvvfgrlqRHVAXIVVAQFAA-TLTDdtGAPLLLVKGGSSLELrrg   70
ident                                                 | |
Sbjct edklnhhqriglkyfgdfekriPREEMLQMQDIVLnEVKKvdSEYIATVCGSFRRGA---  177
DSSP  hhhllhhhhhhhhlhhhhhlleEHHHHHHHHHHHHHhHHHHhlLLLEEEELLLLLLL---


DSSP  lllllLLLLEEEEEL------------lLHHHHHHHHHHHHhhlllleeEEELLLEELll
Query ipdsrTSKDFATVAR------------rDIELIHEQLADAGetgwegftAIFTAPEEIaa  118
ident       | |                         |||             |
Sbjct ----eSSGDMDVLLThpsftsestkqpkLLHQVVEQLQKVH--------FITDTLSKG--  223
DSSP  ----lEELLEEEEEEellllllllllhHHHHHHHHHHHHLL--------LEEEELLLL--


DSSP  llllLLLEEEEEEEEELL--EEEEEEEEEEEL-LLLLllllleeeeelhhhhllllllee
Query pgxpVKPRRFTAKLSYRG--RAFATVPIEVSSV-EAGNadqfdtltsdalglvgvpaava  175
ident              |             |
Sbjct ----ETKFMGVCQLPSKNdeKEYPHRRIDIRLIpKDQY----------------------  257
DSSP  ----LLEEEEEELLLLLllLLLLLEEEEEEEEeHHHH----------------------


DSSP  eeellhHHHHhhhhhhhhlllllllllllhhhhHHHHHH--------------------
Query vpcxtiPWQIaqklhavtavleepkandrahdlvDLQLLE--------------------  215
ident 
Sbjct -----yCGVL--------------------yftgSDIFNKnmrahalekgftineytirp  292
DSSP  -----hHHHH--------------------hhhlLHHHHHhhhhhhhhlleeeelleeee


DSSP  -hhllhhhhhhhHHHHHHhhhhhllllLLLLLlllllhhhhhhhhhlllllllllllhhh
Query -glldadlxptRSACIAifearaqhpWPPRVatlphwpliyagalegldhlelartvda  274
ident 
Sbjct lgvtgvageplpVDSEKD------ifdYIQWK----------------------------  318
DSSP  lllllllllllLLLHHH------hhhHLLLL----------------------------


DSSP  hhhhhhhhhhhhhhllll
Query aaqavqrfvaridratkr  292
ident 
Sbjct ---------yrepkdrse  327
DSSP  ---------lllhhhlll
```

No 291: Query=mol1A Sbjct=7ickA Z-score=6.6

back to top

```
DSSP  ------------------------------------------------------------
Query ------------------------------------------------------------    0
ident 
Sbjct etlnggitdmltelanfeknvsqaihkynayrkaasviakyphkiksgaeakklpgvgtk   60
DSSP  llllhhhhhhhhhhhhhhhhllllhhhhhhhhhhhhhhhhllllllllhhhhhllllllhh


DSSP  -------------------------------------------------llllllllllh
```

```
ident
Sbjct iaekideflatgklrklekirqddtsssinfltrvsgigpsaarkfvdegiktledlrkn  120
DSSP  hhhhhhhhhhllllhhhhhhhhlhhhhhhhhhllllllhhhhhhhhhhllllhhhhhhl


DSSP  hhhhhhhhhhhhhllllhhhhhHHHHHHHHHHHHH-LLLLllLLLLEEEEHHHHHHHhhl
Query rslrdrltqvaerqggvvfgrlqRHVAXIVVAQFAA-TLTDdtGAPLLLVKGGSSLELrrg   70
ident                                                 | |
Sbjct edklnhhqriglkyfgdfekriPREEMLQMQDIVLnEVKKvdSEYIATVCGSFRRGA---  177
DSSP  hhhllhhhhhhhhlhhhhhlleEHHHHHHHHHHHHHhHHHHhlLLLEEEEELLHHHHLL---


DSSP  lllllLLLLEEEEEL------------lLHHHHHHHHHHHHhhlllleeEEELLLEELll
Query ipdsrTSKDFATVAR------------rDIELIHEQLADAGetgwegftAIFTAPEEIaa  118
ident       | |                         |||             |
Sbjct ----eSSGDMDVLLThpsftsestkqpkLLHQVVEQLQKVH--------FITDTLSKG--  223
DSSP  ----lEELLEEEEEEellllllllhhHHHHHHHHHHHHHLL--------LEEEEEEEL--


DSSP  llllLLLEEEEEEEEEELL--EEEEEEEEEEEEL-LLLLllllleeeeelhhhhllllllee
Query pgxpVKPRRFTAKLSYRG--RAFATVPIEVSSV-EAGNadqfdtltsdalglvgvpaava  175
ident              |             |
Sbjct ----ETKFMGVCQLPSKNdeKEYPHRRIDIRLIpKDQY----------------------  257
DSSP  ----LLEEEEEEELLLLLllLLLLLEEEEEEEEeHHHH----------------------


DSSP  eeellhHHHHhhhhhhhhlllllllllllhhhhHHHHHH--------------------
Query vpcxtiPWQIaqklhavtavleepkandrahdlvDLQLLE--------------------  215
ident
Sbjct -----yCGVL--------------------yftgSDIFNKnmrahalekgftineytirp  292
DSSP  -----hHHHH--------------------hhhlLHHHHHhhhhhhhhlleeeelleeee


DSSP  -hhllhhhhhhhHHHHHHhhhhhllllLLLLLllllllhhhhhhhhhllllllllllhhh
Query -glllddadlxptRSACIAifearaqhpWPPRVatlphwpliyagalegldhlelartvda  274
ident
Sbjct lgvtgvageplpVDSEKD------ifdYIQWK----------------------------  318
DSSP  llllllllllllLLLHHH------hhhHLLLL----------------------------


DSSP  hhhhhhhhhhhhhhllll
Query aaqavqrfvaridratkr  292
ident
Sbjct ---------yrepkdrse  327
DSSP  ---------lllhhhhll
```

No 292: Query=mol1A Sbjct=8icwA Z-score=6.6

back to top

```
DSSP  ------------------------------------------------------------
Query ------------------------------------------------------------    0
ident
Sbjct etlnggitdmltelanfeknvsqaihkynayrkaasviakyphkiksgaeakklpgvgtk   60
DSSP  llllhhhhhhhhhhhhhhhhlllllhhhhhhhhhhhhhhhhlllllllhhhhhlllllllhh


DSSP  -------------------------------------------------lllllllllh
Query -------------------------------------------------aapysspptnl   11
ident
Sbjct iaekideflatgklrklekirqddtsssinfltrvsgigpsaarkfvdegiktledlrkn  120
DSSP  hhhhhhhhhhllllhhhhhhhhlhhhhhhhhhllllllhhhhhhhhhhllllhhhhhhl


DSSP  hhhhhhhhhhhhhllllhhhhhHHHHHHHHHHHHH-LLLLllLLLLEEEEHHHHHHHhhl
Query rslrdrltqvaerqggvvfgrlqRHVAXIVVAQFAA-TLTDdtGAPLLLVKGGSSLELrrg   70
ident                                                 | |
Sbjct edklnhhqriglkyfgdfekriPREEMLQMQDIVLnEVKKvdSEYIATVCGSFRRGA---  177
DSSP  hhhllhhhhhhhhlhhhhhlllLHHHHHHHHHHHHHhHHHHhlLLLEEEEELLLLLLL---


DSSP  lllllLLLLEEEEEL------------lLHHHHHHHHHHHHhhlllleeEEELLLEELll
Query ipdsrTSKDFATVAR------------rDIELIHEQLADAGetgwegftAIFTAPEEIaa  118
ident       | |                         |||             |
Sbjct ----eSSGDMDVLLThpsftsestkqpkLLHQVVEQLQKVH--------FITDTLSKG--  223
DSSP  ----lLLLLEEEEEElllllllllllhHHHHHHHHHHHHLL--------LEEEEEEEL--
```

```
DSSP  llllLLLEEEEEEEEEELL--EEEEEEEEEEEEL-LLLLllllleeeeelhhhhllllllee
Query pgxpVKPRRFTAKLSYRG--RAFATVPIEVSSV-EAGNadqfdtltsdalglvgvpaava  175
ident              |             |
Sbjct ----ETKFMGVCQLPSKNdeKEYPHRRIDIRLIpKDQY----------------------  257
DSSP  ----LLEEEEEEELLLLLllLLLLLEEEEEEELLlLLLL----------------------


DSSP  eeellhHHHHHHhhhhhhllllllllllllhhhhHHHHHH--------------------
Query vpcxtiPWQIAQklhavtavleepkandrahdlvDLQLLE--------------------  215
ident
Sbjct -----yCGVLYF--------------------tgSDIFNKnmrahalekgftineytirp  292
DSSP  -----hHHHHHL--------------------llLHHHHHhhhhhhllllleeellllleee


DSSP  -hhllhhhhhhhHHHHhhhhhhllllLLLLLllllllhhhhhhhhhlllllllllllhhh
Query -glllldadlxptRSACIaifearaqhpWPPRVatlphwpliyagalegldhlelartvda  274
ident
Sbjct lgvtgvageplpVDSEK------difdYIQWK----------------------------  318
DSSP  llllllllllllLLLHH------hhhhHLLLL----------------------------


DSSP  hhhhhhhhhhhhhhhllll
Query aaqavqrfvaridratkr  292
ident
Sbjct ---------yrepkdrse  327
DSSP  ---------llllllll
```

No 293: Query=mol1A Sbjct=1xsnA Z-score=6.6

back to top

```
DSSP  ------------------------------------------------------------
Query ------------------------------------------------------------    0
ident
Sbjct hnlhiteklevlakaysvqgdkwralgyakainalksfhkpvtsyqeacsipgigkrmae   60
DSSP  llllhhhhhhhhhhhhhlllhhhhhhhhhhhhhhllllllllllhhhhhllllllhhhhh


DSSP  ------------------------------------------lllllllllhhhhhhh
Query ------------------------------------------aapyssspptnlrslrdr   17
ident
Sbjct kiieilesghlrkldhisesvpvlelfsniwgagtktaqmwyqqgfrsledirsqasltt  120
DSSP  hhhlllllllllhhhhhllllhhhhhhhhhlllllllhhhhhhhhhlllllhhhhhhhhllllh


DSSP  hhhhhhhllllhhhhHHHHHHHHHHHHHHHLL-LLLLLLLLEEEEHHHHHHHhhllllllL
Query ltqvaerqgvvfgrlQRHVAXIVVAQFAATL-TDDTGAPLLLVKGGSSLELrrgipdsrT   76
ident                          |             |    |              |
Sbjct qqaiglkhysdflerMPREEATEIEQTVQKAaQAFNSGLLCVACGSYRRGK-------aT  173
DSSP  hhhhhhhlllhhhllEEHHHHHHHHHHHHHHHHhHLLLLLLEEEELHHHHLLL-------lE


DSSP  LLLEEEEEL--------lLHHHHHHHHHHHHHhhllllleeeEELLLEELLllllLLLLEEE
Query SKDFATVAR--------rDIELIHEQLADAGetgwegftaIFTAPEEIAapgxPVKPRRF  128
ident   |                       |   |           |
Sbjct CGDVDVLIThpdgrshrgIFSRLLDSLRQEG---------FLTDDLVSQeengQQQKYLG  224
DSSP  ELEEEEEEEllllllllLHHHHHHHHHHHLL---------LEEEEEELLllllLLLEEEE


DSSP  EEEEEEllEEEEEEEEEEEEL-LLLLllllleeeeelhhhhllllllleeeeellhHHHHhh
Query TAKLSYrgRAFATVPIEVSSV-EAGNadqfdtltsdalglvgvpaavavpcxtiPWQIaq  187
ident    |                |
Sbjct VCRLPG--PGRRHRRLDIIVVpYSEF---------------------------aCALL--  253
DSSP  EELLLL--LLLLLEEEEEEEELlHHHH---------------------------hHHHH--


DSSP  hhhhhhlllllllllllHHHHHHH-------------------------hhhhhllhHH
Query klhavtavleepkandraHDLVDLQ-------------------------lleglllldAD  222
ident
Sbjct --------------yftgSAHFNRSmralaktkgmslsehalstavvrnthgakvgpgRV  299
DSSP  --------------hhhlLHHHHHHhhhhhhhlleeellllleeelleellllleeellEE


DSSP  HHhhHHHHHhhhhhhllllLLLLLllllllhhhhhhhhhlllllllllllhhhhhhhhhhh
Query LXptRSACIaifearaqhpWPPRVatlphwpliyagalegldhlelartvdaaaqavqrf  282
```

```
Sbjct LP--TPTEK-------dvfRLLGL------------------------------------  314
DSSP  LL--LLLHH-------hhhHHLLL------------------------------------


DSSP  hhhhhhllll
Query varidratkr  292
ident 
Sbjct pyrepaerdw  324
DSSP  llllhhhhll
```

No 294: Query=mol1A Sbjct=3pncA Z-score=6.6

back to top

```
DSSP  ------------------------------------------------------------
Query ------------------------------------------------------------    0
ident 
Sbjct tnhnlhiteklevlakaysvqgdkwralgyakainalksfhkpvtsyqeacsipgigkrm   60
DSSP  llllhhhhhhhhhhhhhhhhhllhhhhhhhhhhhhhhhhlllllllhhhhhhlllllhhh


DSSP  ---------------------------------------------llllllllllhhhhh
Query ---------------------------------------------aapysspptnlrslr   15
ident 
Sbjct aekiieilesghlrkldhisesvpvlelfsniwgagtktaqmwyqqgfrsledirsqasl  120
DSSP  hhhhhhhhhlllhhhhhllllhhhhhhhhhllllllhhhhhhhhhlllllhhhhhhhlll


DSSP  hhhhhhhhhllllhHHHHHHHHHHHHHHHHHLL-LLLLLLLLEEEEHHHHHHHhhlllll
Query drltqvaerqgvvfGRLQRHVAXIVVAQFAATL-TDDTGAPLLLVKGGSSLELrrgipds   74
ident                            |             |    |
Sbjct ttqqaiglkhysdfLERMPREEATEIEQTVQKAaQAFNSGLLCVACGSYRRGK-------  173
DSSP  lhhhhhhhhlhhhhHLLEEHHHHHHHHHHHHHHhHLLLLLLEEEELHHHHLLL-------


DSSP  lLLLLEEEEEL--------lLHHHHHHHHHHHHhhlllleeEEELLLEELllllllLLLE
Query rTSKDFATVAR--------rDIELIHEQLADAGetgwegftAIFTAPEEIaapgxpVKPR  126
ident  |  |                       |   |
Sbjct aTCGDVDVLIThpdgrshrgIFSRLLDSLRQEG--------FLTDDLVKG------ETKY  219
DSSP  lEELLEEEEEElllllllllLHHHHHHHHHHLL--------LEEEEEEEL------LLEE


DSSP  EEEEEEEEllEEEEEEEEEEEEL-LLLLlllleeeeelhhhhlllllleeeeellhHHHH
Query RFTAKLSYrgRAFATVPIEVSSV-EAGNadqfdtltsdalglvgvpaavavpcxtiPWQI  185
ident      |                |
Sbjct LGVCRLPG--PGRRHRRLDIIVVpYSEF---------------------------aCALL  250
DSSP  EEEELLLL--LLLLLEEEEEEELlHHHH---------------------------hHHHH


DSSP  hhhhhhhlllllllllllllHHHHHHH--------------------hhhhhllhHHHHh
Query aqklhavtavleepkandraHDLVDLQ--------------------llegllldADLXp  225
ident                                                          |
Sbjct ----------------yftgSAHFNRSmralaktkgmslsehalstavvrkvgpgRVLP-  293
DSSP  ----------------hhhlLHHHHHHhhhhhhhlleeellllleeelllllllllEEEL-


DSSP  hHHHHHhhhhhhllllLLLLLLllllhhhhhhhhhlllllllllllhhhhhhhhhhhhhh
Query tRSACIaifearaqhpWPPRVAtlphwpliyagalegldhlelartvdaaaqavqrfvar  285
ident 
Sbjct -TPTEK-------dvfRLLGLP------------------------------------yr  309
DSSP  -LLLHH-------hhhHHLLLL------------------------------------ll


DSSP  hhhllll
Query idratkr  292
ident 
Sbjct epaerdw  316
DSSP  lhhhlll
```

No 295: Query=mol1A Sbjct=4uawA Z-score=6.6

back to top

```
Query ------------------------------------------------------------    0
ident
Sbjct tlnggitdmltelanfeknvsqaihkynayrkaasviakyphkiksgaeakklpgvgtki   60
DSSP  lllhhhhhhhhhhhhhhhllllhhhhhhhhhhhhhhhhllllllllhhhhlllllllhhh


DSSP  ------------------------------------------------llllllllllhh
Query ------------------------------------------------aapysspptnlr   12
ident
Sbjct aekideflatgklrklekirqddtsssinfltrvsgigpsaarkfvdegiktledlrkne  120
DSSP  hhhhhhhhhhlllhhhhhhhhlhhhhhhhhhllllllhhhhhhhhhlllllhhhhhllh


DSSP  hhhhhhhhhhhhllllhhhhhHHHHHHHHHHHHHH-LLLLLLLLLEEEEHHHHHHHHhhll
Query slrdrltqvaerqgvvfgrlqRHVAXIVVAQFAA-TLTDDTGAPLLLVKGGSSLELrrgi   71
ident                                                | |
Sbjct dklnhhqriglkyfgdfekriPREEMLQMQDIVLnEVKKVDSEYIATVCGSFRRGA----  176
DSSP  hhllhhhhhhhhlhhhhhlleEHHHHHHHHHHHHhHHHHHLLLLEEEELHHHHLLL----


DSSP  llllLLLLEEEEEL------------lLHHHHHHHHHHHHhhlllleeEEELLLEEllll
Query pdsrTSKDFATVAR------------rDIELIHEQLADAGetgwegftAIFTAPEEiaap  119
ident      | |                         |||             |
Sbjct ---eSSGDMDVLLThpsftsestkqpkLLHQVVEQLQKVH--------FITDTLSK----  221
DSSP  ---lEELLEEEEEEllllllllllllllHHHHHHHHHHHLL--------LEEEEEEE----


DSSP  lllLLLEEEEEEEEEL--LEEEEEEEEEEEEL-LLLLlllleeeeelhhhhlllllleee
Query gxpVKPRRFTAKLSYR--GRAFATVPIEVSSV-EAGNadqfdtltsdalglvgvpaavav  176
ident             |             |
Sbjct --gETKFMGVCQLPSKndEKEYPHRRIDIRLIpKDQY-----------------------  256
DSSP  --lLLEEEEEEELLLLllLLLLLEEEEEEEELlHHHH-----------------------


DSSP  eellhHHHHhhhhhhhhllllllllllllHHHH-------------------hhhhhhhh
Query pcxtiPWQIaqklhavtavleepkandraHDLV-------------------dlqllegl  217
ident                               |
Sbjct ----yCGVL----------------yftgSDIFnknmrahalekgftineytirplgvtg  296
DSSP  ----hHHHH----------------hhhlLHHHhhhhhhhhhhlleeellllleeelllll


DSSP  llhHHHHHhhHHHHhhhhhhlllLLLLLLlllllhhhhhhhhhlllllllllllhhhhhh
Query lldADLXPtrSACIaifearaqhPWPPRVatlphwpliyagalegldhlelartvdaaaq  277
ident      |
Sbjct vagEPLPV--DSEK-----difdYIQWKY-------------------------------  318
DSSP  lllLLLLL--LLHH-----hhhhHLLLLL-------------------------------


DSSP  hhhhhhhhhhhllll
Query avqrfvaridratkr  292
ident
Sbjct -------repkdrse  326
DSSP  -------llhhhlll
```

No 296: Query=mol1A Sbjct=4k4gI Z-score=6.6

back to top

```
DSSP  ------------------------------------------------------------
Query ------------------------------------------------------------    0
ident
Sbjct tnhnlhiteklevlakaysvqgdkwralgyakainalksfhkpvtsyqeacsipgigkrm   60
DSSP  llllhhhhhhhhhhhhhhhhlllhhhhhhhhhhhhhhhllllllllhhhhhlllllllhhh


DSSP  ---------------------------------------------lllllllllhhhhh
Query ---------------------------------------------aapysspptnlrslr   15
ident
Sbjct aekiieilesghlrkldhisesvpvlelfsniwgagtktaqmwyqqgfrsledirsqasl  120
DSSP  hhhhhhhhhllllhhhhllllhhhhhhhhlllllhhhhhhhhhlllllhhhhhhhhlll


DSSP  hhhhhhhhhllllhhhhhHHHHHHHHHHHHH-LLLLllLLLLEEEEHHHHHHhhhllllL
Query drltqvaerqgvvfgrlqRHVAXIVVAQFAA-TLTDdtGAPLLLVKGGSSLElrrgipdS   74
ident                            |             |    |
Sbjct ttqqaiglkhysdflermPREEATEIEQTVQkAAQAfnSGLLCVACGSYRRG-------K  173
```

```
DSSP  LLLLLEEEEEL--------lLHHHHHHHHHHHHHhhlllleeEEELLLE-ELLLllLLLLL
Query RTSKDFATVAR--------rDIELIHEQLADAGetgwegftAIFTAPE-EIAApgXPVKP  125
ident  |  |                       |   |
Sbjct ATCGDVDVLIThpdgrshrgIFSRLLDSLRQEG--------FLTDDLVsQEEN--GQQQK  223
DSSP  LEELLEEEEEEllllllllHHHHHHHHHHHHLL--------LEEEEEEeEEEE--LLEEE


DSSP  EEEEEEEEEllEEEEEEEEEEEEL-LLLLlllleeeeelhhhhllllllleeeeellhHHH
Query RRFTAKLSYrgRAFATVPIEVSSV-EAGNadqfdtltsdalglvgvpaavavpcxtiPWQ  184
ident       |                |
Sbjct YLGVCRLPG--PGRRHRRLDIIVVpYSEF---------------------------aCAL  254
DSSP  EEEEELLLL--LLLLLEEEEEEEELlHHHH---------------------------hHHH


DSSP  HhhhhhhhhllllllllllllHHHH--------------------hhhhhhhhllhhHHH
Query IaqklhavtavleepkandraHDLV--------------------dlqlleglllldaDLX  224
ident                                                           |
Sbjct L-----------------yftgSAHFnrsmralaktkgmslsehalstavvckvgpgrVLP  298
DSSP  H-----------------hhhlLHHHhhhhhhhhhllleeellllleeellllllllllEEL


DSSP  HhhHHHHhhhhhhlllLLLLLllllllhhhhhhhhhllllllllllhhhHHHHhhhhhh
Query PtrSACIaifearaqhPWPPRvatlphwpliyagalegldhlelartvdaAAQAvqrfva  284
ident
Sbjct T--PTEK-----dvfrLLGLP--------------------yrepaerdwLEHH------  325
DSSP  L--LLHH-----hhhhHLLLL--------------------lllhhhlllLLLL------


DSSP  hhhhllll
Query ridratkr  292
ident
Sbjct ------hh  327
DSSP  ------ll
```

No 297: Query=mol1A Sbjct=4rq1A Z-score=6.6

back to top

```
DSSP  ------------------------------------------------------------
Query ------------------------------------------------------------    0
ident
Sbjct tlnggitdmltelanfeknvsqaihkynayrkaasviakyphkiksgaeakklpgvgtki   60
DSSP  lllhhhhhhhhhhhhhhhhllllhhhhhhhhhhhhhhhhlllllllhhhhhllllllhhh


DSSP  -------------------------------------------------lllllllllh
Query -------------------------------------------------aapyssspptnl   11
ident
Sbjct aekideflatgklrklekirqddtsssinfltrvsgigpsaarkfvdegiktledlrkne  120
DSSP  hhhhhhhhhhlllhhhhhhhhllhhhhhhhhhhllllllhhhhhhhhhhllllllhhhhhhlh


DSSP  hhhhhhhhhhhhhllllhHHHHhHHHHHHHHHHHHH-LLLLllLLLLEEEEHHHHHHhhhl
Query rslrdrltqvaerqgvvfGRLQrHVAXIVVAQFAA-TLTDdtGAPLLLVKGGSSLElrrg   70
ident                    |                            | |
Sbjct dklnhhqriglkyfgdfeKRIP-REEMLQMQDIVLnEVKKvdSEYIATVCGSFRRG----  175
DSSP  hhllhhhhhhhhlhhhhhLLEE-HHHHHHHHHHHHHhHHHHhlLLLLLEELLHHHHL----


DSSP  lllLLLLLLEEEEEL------------lLHHHHHHHHHHHHHhhlllleeEEELLLEElll
Query ipdSRTSKDFATVAR------------rDIELIHEQLADAGetgwegftAIFTAPEEiaa  118
ident       | |                         |||             |
Sbjct ---AESSGDMDVLLThpsftsestkqpkLLHQVVEQLQKVH--------FITDTLSK---  221
DSSP  ---LLEELLEEEEELllllllllllllHHHHHHHHHHHHLL--------LEEEEEEE---


DSSP  llllLLLEEEEEEEEEELL--EEEEEEEEEEEEL-LLLLllllleeeeelhhhhllllllee
Query pgxpVKPRRFTAKLSYRG--RAFATVPIEVSSV-EAGNadqfdtltsdalglvgvpaava  175
ident              |             |
Sbjct ---gETKFMGVCQLPSKNdeKEYPHRRIDIRLIpKDQY----------------------  256
DSSP  ---lLLEEEEEEELLLLLLllLLLLLEEEEEEEELlHHHH----------------------


DSSP  eeellhHHHHhhhhhhhhhllllllllllllHHHH-------------------hhhhhhh
```

```
ident                                |
Sbjct -----yCGVL----------------yftgSDIFnknmrahalekgftineytirplgvt  295
DSSP  -----hHHHH----------------hhllLHHHhhhhhhhhhhhleeellllleeellll


DSSP  hllhhHHHHhhHHHHhhhhhhlllLLLLLLlllllhhhhhhhhhlllllllllllhhhhh
Query llldaDLXPtrSACIaifearaqhPWPPRVatlphwpliyagalegldhlelartvdaaa  276
ident       |
Sbjct gvagePLPV--DSEK-----difdYIQWKY------------------------------  318
DSSP  lllllLLLL--LLHH-----hhhhHLLLLL------------------------------


DSSP  hhhhhhhhhhhhllll
Query qavqrfvaridratkr  292
ident
Sbjct ----------repkdr  324
DSSP  ----------llhhhl
```

No 298: Query=mol1A Sbjct=3pmnA Z-score=6.6

back to top

```
DSSP  ------------------------------------------------------------
Query ------------------------------------------------------------    0
ident
Sbjct atnhnlhiteklevlakaysvqgdkwralgyakainalksfhkpvtsyqeacsipgigkr   60
DSSP  llllllhhhhhhhhhhhhhhhlllhhhhhhhhhhhhhhhlllllllllhhhhhhlllllhh


DSSP  ---------------------------------------------llllllllllhhhh
Query ---------------------------------------------aapyssppptnlrsl   14
ident
Sbjct maekiieilesghlrkldhisesvpvlelfsniwgagtktaqmwyqqgfrsledirsqas  120
DSSP  hhhhhhhhhhhlllhhhhhllllhhhhhhhhllllllhhhhhhhhhlllllhhhhhhhhll


DSSP  hhhhhhhhhllllhHHHHHHHHHHHHHHHHHHHHLL-LLLLLLLLEEEEHHHHHHHHhhllll
Query rdrltqvaerqgvvfGRLQRHVAXIVVAQFAATL-TDDTGAPLLLVKGGSSLELrrgipd   73
ident                             |             |    |
Sbjct lttqqaiglkhysdfLERMPREEATEIEQTVQKAaQAFNSGLLCVACGSYRRGK------  174
DSSP  llhhhhhhhhlllhhHLLEEHHHHHHHHHHHHHHHHhHLLLLLLEEEELHHHHLLL------


DSSP  llLLLLEEEEEL--------lLHHHHHHHHHHHHhhlllleeEEELLLEELllllllLLL
Query srTSKDFATVAR--------rDIELIHEQLADAGetgwegftAIFTAPEEIaapgxpVKP  125
ident   |  |                       |   |
Sbjct -aTCGDVDVLIThpdgrshrgIFSRLLDSLRQEG--------FLTDDLVKG------ETK  219
DSSP  -lEELLEEEEEEllllllllLHHHHHHHHHHHLL--------LEEEEEEEL------LLE


DSSP  EEEEEEEEEllEEEEEEEEEEEEEL-LLLLllllleeeeelhhhhllllllleeeeellhHHH
Query RRFTAKLSYrgRAFATVPIEVSSV-EAGNadqfdtltsdalglvgvpaavavpcxtiPWQ  184
ident       |                |
Sbjct YLGVCRLPG--PGRRHRRLDIIVVpYSEF---------------------------aCAL  250
DSSP  EEEEELLLL--LLLLLEEEEEEEELlHHHH---------------------------hHHH


DSSP  HhhhhhhhhllllllllllllHHHHHHH---------------hhhhhllhHHHHhhHHH
Query IaqklhavtavleepkandraHDLVDLQ---------------lleglllldADLXptRSA  229
ident                                                      |
Sbjct L----------------yftgSAHFNRSmralaktkgmslsehalstavpgRVLP--TPT  292
DSSP  H----------------hhhlLHHHHHHhhhhhhhlleeellllleeellllLEEL--LLL


DSSP  HHhhhhhhllllLLLLLLllllhhhhhhhhhllllllllllhhhhhhhhhhhhhhhhhl
Query CIaifearaqhpWPPRVAtlphwpliyagalegldhlelartvdaaaqavqrfvaridra  289
ident
Sbjct EK-------dvfRLLGLP-------------------------------------yrepae  309
DSSP  HH-------hhhHHLLLL-------------------------------------lllhhh


DSSP  lll
Query tkr  292
ident
Sbjct rdw  312
DSSP  lll
```

No 299: Query=mol1A Sbjct=4z6eA Z-score=6.6

back to top

```
DSSP  ------------------------------------------------------------
Query ------------------------------------------------------------   0
ident 
Sbjct tlnggitdmltelanfeknvsqaihkynayrkaasviakyphkiksgaeakklpgvgtki  60
DSSP  lllhhhhhhhhhhhhhhhhllllhhhhhhhhhhhhhhhllllllllhhhhhllllllhhh


DSSP  ------------------------------------------------llllllllllhh
Query ------------------------------------------------aapyssspptnlr  12
ident 
Sbjct aekideflatgklrklekirqddtsssinfltrvsgigpsaarkfvdegiktledlrkne 120
DSSP  hhhhhhhhhhlllhhhhhhhhlhhhhhhhhhllllllhhhhhhhhhlllllhhhhhhlh


DSSP  hhhhhhhhhhhhllllhHHHHHHHHHHHHHHHHHHL-LLLLLLLLLEEEEHHHHHHHHhhll
Query slrdrltqvaerqgvvfGRLQRHVAXIVVAQFAAT-LTDDTGAPLLLVKGGSSLELrrgi  71
ident                                                | |
Sbjct dklnhhqriglkyfgdfEKRIPREEMLQMQDIVLNeVKKVDSEYIATVCGSFRRGA---- 176
DSSP  hhllhhhhhhhhlhhhhLLLEEHHHHHHHHHHHHHHhHHHHLLLLEEEEEELLLLLLL----


DSSP  llllLLLLEEEEEL------------lLHHHHHHHHHHHHHhhlllleeEEELLLEELlll
Query pdsrTSKDFATVAR------------rDIELIHEQLADAGetgwegftAIFTAPEEIaap 119
ident      | |                         |||             |
Sbjct ---eSSGDMDVLLThpsftsestkqpkLLHQVVEQLQKVH--------FITDTLSKG--- 222
DSSP  ---lEELLEEEEEEllllllllllllHHHHHHHHHHHHLL--------LEEEEEEEL---


DSSP  lllLLLEEEEEEEEEL--LEEEEEEEEEEEEL-LLLLllllleeeeelhhhhlllllleee
Query gxpVKPRRFTAKLSYR--GRAFATVPIEVSSV-EAGNadqfdtltsdalglvgvpaavav 176
ident             |             |
Sbjct ---ETKFMGVCQLPSKndEKEYPHRRIDIRLIpKDQY----------------------- 256
DSSP  ---LLEEEEEEELLLLLllLLLLLLLEEEEEEEELlHHHH-----------------------


DSSP  eellhHHHHhhhhhhhhllllllllllllHHHHHHH------------------hhhhh
Query pcxtiPWQIaqklhavtavleepkandraHDLVDLQ------------------llegl 217
ident                               |
Sbjct ----yCGVL----------------yftgSDIFAKNmrahalekgftineytirplgvtg 296
DSSP  ----hHHHH----------------hhhlLHHHHHHhhhhhhhlleeellllleeellll


DSSP  llhHHHHhhHHHHHhhhhhhllllLLLLLlllllhhhhhhhhhllllllllllhhhhhh
Query lldADLXptRSACIaifearaqhpWPPRVatlphwpliyagalegldhlelartvdaaaq 277
ident      |
Sbjct vagEPLP--VDSEK-------difDYIQW------------------------------- 316
DSSP  lllLLLL--LLLHH-------hhhHHLLL-------------------------------


DSSP  hhhhhhhhhhhllll
Query avqrfvaridratkr  292
ident 
Sbjct -----kyrepkdrse  326
DSSP  -----llllhhhlll
```

No 300: Query=mol1A Sbjct=4rq2A Z-score=6.5

back to top

```
DSSP  ------------------------------------------------------------
Query ------------------------------------------------------------   0
ident 
Sbjct etlnggitdmltelanfeknvsqaihkynayrkaasviakyphkiksgaeakklpgvgtk  60
DSSP  llllhhhhhhhhhhhhhhhhllllhhhhhhhhhhhhhhhhlllllllhhhhhhlllllhh


DSSP  -------------------------------------------------llllllllllh
Query -------------------------------------------------aapyssspptnl  11
ident 
```

```
DSSP  hhhhhhhhhhhlllhhhhhhhhlhhhhhhhhhhlllllhhhhhhhhhllllhhhhhhl


DSSP  hhhhhhhhhhhhhllllhhhhhHHHHHHHHHHHHH-LLLLllLLLLEEEEHHHHHHHhhl
Query rslrdrltqvaerqgvvfgrlqRHVAXIVVAQFAA-TLTDdtGAPLLLVKGGSSLELrrg   70
ident                                                 | |
Sbjct edklnhhqriglkyfgdfekriPREEMLQMQDIVLnEVKKvdSEYIATVCGSFRRGA---  177
DSSP  hhhllhhhhhhhhlhhhhhlleEHHHHHHHHHHHHhHHHHhlLLLEEEELHHHLLLL---


DSSP  lllllLLLLEEEEEL------------lLHHHHHHHHHHHHhhlllleeEEELLLEELll
Query ipdsrTSKDFATVAR------------rDIELIHEQLADAGetgwegftAIFTAPEEIaa  118
ident       | |                         |||             |
Sbjct ----eSSGDMDVLLThpsftsestkqpkLLHQVVEQLQKVH--------FITDTLSKG--  223
DSSP  ----lEELLEEEEEElllee1leellllHHHHHHHHHHHLL--------LEEEEEEEL--


DSSP  llllLLLEEEEEEEEEEL--LEEEEEEEEEEEEL-LLLLllllleeeeelhhhhllllllee
Query pgxpVKPRRFTAKLSYR--GRAFATVPIEVSSV-EAGNadqfdtltsdalglvgvpaava  175
ident              |             |
Sbjct ----ETKFMGVCQLPSKndEKEYPHRRIDIRLIpKDQY----------------------  257
DSSP  ----LLEEEEEEELLLLLllLLLLLLEEEEEEELlHHHH----------------------


DSSP  eeellhHHHHhhhhhhhhllllllllllHHHHHHHhhhhhllhhhhhhhhhHHHH---
Query vpcxtiPWQIaqklhavtavleepkandraHDLVDLQlleglllldadlxptrsACIA---  232
ident                                |                       |
Sbjct -----yCGVL----------------yftgSDIFNKN--mrahalekgftineYTIRplg  294
DSSP  -----hHHHH----------------hhhlLHHHHHH--hhhhhhhlleeellLLEEell


DSSP  ---------hhhhhlllllLLLLLllllhhhhhhhHHLLlllllllllhhhhhhhhhhh
Query ---------ifearaqhpwPPRVAtlphwpliyagALEGldhlelartvdaaaqavqrfv  283
ident
Sbjct vtgvageplpvdsekdifdYIQWK----yrepkdrSEHH---------------------  329
DSSP  lllllllllllllhhhhhhHLLLL----lllhhhhLLLL---------------------


DSSP  hhhhhllll
Query aridratkr  292
ident
Sbjct -----hhhh  333
DSSP  -----llll
```

No 301: Query=mol1A Sbjct=5j2aA Z-score=6.5

back to top

```
DSSP  ------------------------------------------------------------
Query ------------------------------------------------------------    0
ident
Sbjct tlnggitdmltelanfeknvsqaihkynayrkaasviakyphkiksgaeakklpgvgtki   60
DSSP  lllhhhhhhhhhhhhhhhhllllhhhhhhhhhhhhhhhhhlllllllhhhhlllllllhhh


DSSP  ------------------------------------------------llllllllllhh
Query ------------------------------------------------aapysspptnlr   12
ident
Sbjct aekideflatgklrklekirqddtsssinfltrvsgigpsaarkfvdegiktledlrkne  120
DSSP  hhhhhhhhhlllhhhhhhhhlhhhhhhhhhlllllllhhhhhhhhhhllllhhhhhllh


DSSP  hhhhhhhhhhhhllllhhhhhHHHHHHHHHHHHH-LLLLllLLLLEEEEHHHHHHHhhll
Query slrdrltqvaerqgvvfgrlqRHVAXIVVAQFAA-TLTDdtGAPLLLVKGGSSLELrrgi   71
ident                                                | |
Sbjct dklnhhqriglkyfgdfekriPREEMLQMQDIVLnEVKKvdSEYIATVCGSFRRGA----  176
DSSP  hhllhhhhhhhhlhhhhhlleEHHHHHHHHHHHHhHHHHhlLLLEEEEEHHHLLLL----


DSSP  lllLLLLLEEEEEL------------lLHHHHHHHHHHHHhhlllleeEEELLLEEllll
Query pdsRTSKDFATVAR------------rDIELIHEQLADAGetgwegftAIFTAPEEiaap  119
ident      | |                         |||             |
Sbjct ---ESSGDMDVLLThpsftsestkqpkLLHQVVEQLQKVH--------FITDTLSK----  221
DSSP  ---LEELLEEEEEElllee1leellllHHHHHHHHHHHLL--------LEEEEEEE----
```

```
Query gxpVKPRRFTAKLSYR--GRAFATVPIEVSSV-EAGNadqfdtltsdalglvgvpaavav  176
ident             |             |
Sbjct --gETKFMGVCQLPSKndEKEYPHRRIDIRLIpKDQY-----------------------  256
DSSP  --lLLEEEEEELLLLLllLLLLLLEEEEEEELlHHHH-----------------------


DSSP  eellhHHHHHhhhhhhllllllllllllHHHH-------------------hhhhhhhh
Query pcxtiPWQIAQklhavtavleepkandraHDLV-------------------dlqllegl  217
ident                               |
Sbjct -----YCGVLY---------------ftgSDIFnknmrahalekgftineytirplgvtg  296
DSSP  -----HHHHHH---------------hhlLHHHhhhhhhhhhhhleeellllleeelllll


DSSP  llhHHHHHhhHHHHhhhhhhlllLLLLLLlllllhhhhhhhhhllllllllllhhhhhh
Query lldADLXPtrSACIaifearaqhPWPPRVatlphwpliyagalegldhlelartvdaaaq  277
ident      |
Sbjct vagEPLPV--DSEK-----difdYIQWKY-------------------------------  318
DSSP  lllLLLLL--LLHH-----hhhhHLLLLL-------------------------------


DSSP  hhhhhhhhhhhllll
Query avqrfvaridratkr  292
ident
Sbjct -------repkdrse  326
DSSP  -------llhhhhll
```

No 302: Query=mol1A Sbjct=3mbyA Z-score=6.5

back to top

```
DSSP  ------------------------------------------------------------
Query ------------------------------------------------------------    0
ident
Sbjct tlnggitdmltelanfeknvsqaihkynayrkaasviakyphkiksgaeakklpgvgtki   60
DSSP  lllhhhhhhhhhhhhhhhhhllllhhhhhhhhhhhhhhhhlllllllllhhhhhllllllhhh


DSSP  ------------------------------------------------llllllllllhh
Query ------------------------------------------------aapysspptnlr   12
ident
Sbjct aekideflatgklrklekirqddtsssinfltrvsgigpsaarkfvdegiktledlrkne  120
DSSP  hhhhhhhhhlllhhhhhhhhlhhhhhhhhhllllllhhhhhhhhhlllllhhhhhhlh


DSSP  hhhhhhhhhhhhllllhhhhhHHHHHHHHHHHHHH-LLLLllLLLLEEEEHHHHHHHHhhll
Query slrdrltqvaerqgvvfgrlqRHVAXIVVAQFAA-TLTDdtGAPLLLVKGGSSLELrrgi   71
ident                                                |  |
Sbjct dklnhhqriglkyfgdfekriPREEMLQMQDIVLnEVKKvdSEYIATVCGSFRRGA----  176
DSSP  hhllhhhhhhhhlllhhhlleEHHHHHHHHHHHHHhHHHHhlLLLEEEELHHHHLLL----


DSSP  llllLLLLEEEEEL------------lLHHHHHHHHHHHHHhhllllleeEEELLLEELlll
Query pdsrTSKDFATVAR------------rDIELIHEQLADAGetgwegftAIFTAPEEIaap  119
ident      | |                         |||             |
Sbjct ---eSSGDMDVLLThpsftsestkqpkLLHQVVEQLQKVH--------FITDTLSKG---  222
DSSP  ---lEELLEEEEEElllllllllllllHHHHHHHHHHHHLL--------LEEEEEEEL---


DSSP  lllLLLEEEEEEEEEEL--LEEEEEEEEEEEEEL-LLLLllllleeeeelhhhhllllllleee
Query gxpVKPRRFTAKLSYR--GRAFATVPIEVSSV-EAGNadqfdtltsdalglvgvpaavav  176
ident             |             |
Sbjct ---ETKFMGVCQLPSKndEKEYPHRRIDIRLIpKDQY-----------------------  256
DSSP  ---LLEEEEEEELLLLLllLLLLLLEEEEEEELlHHHH-----------------------


DSSP  eellhHHHHhhhhhhhhhllllllllllllHHHHHHHH-------------------hhhhh
Query pcxtiPWQIaqklhavtavleepkandraHDLVDLQ-------------------llegl  217
ident                               |
Sbjct ----yCGVL----------------yftgSDIFNKNmrahalekgftineytirplgvtg  296
DSSP  ----hHHHH----------------hhhlLHHHHHHhhhhhhhhlleeellllleeelllll


DSSP  llhHHHHhhHHHHHhhhhhhllllLLLLLlllllhhhhhhhhhllllllllllhhhhhh
Query lldADLXptRSACIaifearaqhpWPPRVatlphwpliyagalegldhlelartvdaaaq  277
ident      |
Sbjct vagEPLP--VDSEK-------difDYIQW-------------------------------  316
```

```
DSSP  hhhhhhhhhhhllll
Query avqrfvaridratkr  292
ident 
Sbjct -----kyrepkdrse  326
DSSP  -----llllhhhlll
```

No 303: Query=mol1A Sbjct=3jprA Z-score=6.5

back to top

```
DSSP  ------------------------------------------------------------
Query ------------------------------------------------------------    0
ident 
Sbjct tlnggitdmltelanfeknvsqaihkynayrkaasviakyphkiksgaeakklpgvgtki   60
DSSP  lllhhhhhhhhhhhhhhhhllllhhhhhhhhhhhhhhhhlllllllhhhhlllllllhhh


DSSP  ------------------------------------------------llllllllllhh
Query ------------------------------------------------aapysspptnlr   12
ident 
Sbjct aekideflatgklrklekirqddtsssinfltrvsgigpsaarkfvdegiktledlrkne  120
DSSP  hhhhhhhhhhlllhhhhhhlllhhhhhhhhhllllllhhhhhhhhhlllllhhhhhhhlh


DSSP  hhhhhhhhhhhhllllhhhhhHHHHHHHHHHHHH-LLLLllLLLLEEEEHHHHHHHhhll
Query slrdrltqvaerqgvvfgrlqRHVAXIVVAQFAA-TLTDdtGAPLLLVKGGSSLELrrgi   71
ident                                                |  |
Sbjct dklnhhqriglkyfgdfekriPREEMLQMQDIVLnEVKKvdSEYIATVCGSFRRGA----  176
DSSP  hhllhhhhhhhhlllhhhlleEHHHHHHHHHHHHhHHHHhlLLLEEEELHHHHLLL----


DSSP  llllLLLLEEEEEL------------lLHHHHHHHHHHHHhhlllleeEEELLLEELlll
Query pdsrTSKDFATVAR------------rDIELIHEQLADAGetgwegftAIFTAPEEIaap  119
ident      | |                         |||             |
Sbjct ---eSSGDMDVLLThpsftsestkqpkLLHQVVEQLQKVH--------FITDTLSKG---  222
DSSP  ---lEELLEEEEEEllleelleellllHHHHHHHHHHHLL--------LEEEEEEEL---


DSSP  lllLLLEEEEEEEEEL--LEEEEEEEEEEEEL-LLLLllleeeeelhhhhllllllleee
Query gxpVKPRRFTAKLSYR--GRAFATVPIEVSSV-EAGNadqfdtltsdalglvgvpaavav  176
ident             |             |
Sbjct ---ETKFMGVCQLPSKndEKEYPHRRIDIRLIpKDQY-----------------------  256
DSSP  ---LLEEEEEELLLLLllLLLLLLEEEEEEELlHHHH-----------------------


DSSP  eellhHHHHhhhhhhhhhhllllllllllHHHHHHH-------------------hhhhh
Query pcxtiPWQIaqklhavtavleepkandraHDLVDLQ-------------------llegl  217
ident                               |
Sbjct ----yCGVL----------------yftgSDIFNKNmrahalekgftineytirplgvtg  296
DSSP  ----hHHHH----------------hhhlLHHHHHHhhhhhhhlleeellllleeelllll


DSSP  llhHHHHhhHHHHHhhhhhhllllLLLLLlllllhhhhhhhhhllllllllllllhhhhhh
Query lldADLXptRSACIaifearaqhpWPPRVatlphwpliyagalegldhlelartvdaaaq  277
ident      |
Sbjct vagEPLP--VDSEK-------difDYIQW-------------------------------  316
DSSP  lllLLLL--LLLHH-------hhhHHLLL-------------------------------


DSSP  hhhhhhhhhhhllll
Query avqrfvaridratkr  292
ident 
Sbjct -----kyrepkdrse  326
DSSP  -----llllhhhlll
```

No 304: Query=mol1A Sbjct=3rjfA Z-score=6.5

back to top

```
DSSP  ------------------------------------------------------------
Query ------------------------------------------------------------    0
```

```
Sbjct tlnggitdmltelanfeknvsqaihkynayrkaasviakyphkiksgaeakklpgvgtki   60
DSSP  lllhhhhhhhhhhhhhhhllllhhhhhhhhhhhhhhhllllllllhhhhhllllllhhh


DSSP  ------------------------------------------------llllllllllhh
Query ------------------------------------------------aapysspptnlr   12
ident 
Sbjct aekideflatgklrklekirqddtsssinfltrvsgigpsaarkfvdegiktledlrkne  120
DSSP  hhhhhhhhhhlllhhhhhhhhlhhhhhhhhhlllllllhhhhhhhhhhllllhhhhhllh


DSSP  hhhhhhhhhhhhllllhhhhhHHHHHHHHHHHHHH-LLLLllLLLLEEEEHHHHHHHHhhll
Query slrdrltqvaerqgvvfgrlqRHVAXIVVAQFAA-TLTDdtGAPLLLVKGGSSLELrrgi   71
ident                                                | |
Sbjct dklnhhqriglkyfgdfekriPREEMLQMQDIVLnEVKKvdSEYIATVCGSFRRGA----  176
DSSP  hhllhhhhhhhhlhhhhhlleEHHHHHHHHHHHHHhHHHHhlLLLEEEELHHHHLLL----


DSSP  llllLLLLEEEEEL------------lLHHHHHHHHHHHHHhhlllleeEEELLLEELlll
Query pdsrTSKDFATVAR------------rDIELIHEQLADAGetgwegftAIFTAPEEIaap  119
ident      | |                         |||             |
Sbjct ---eSSGDMDVLLThpsftsestkqpkLLHQVVEQLQKVH--------FITDTLSKG---  222
DSSP  ---lEELLEEEEEllleelleellllHHHHHHHHHHHLL--------LEEEEEEEL---


DSSP  lllLLLEEEEEEEEEL--LEEEEEEEEEEEEL-LLLLllllleeeeelhhhhllllleee
Query gxpVKPRRFTAKLSYR--GRAFATVPIEVSSV-EAGNadqfdtltsdalglvgvpaavav  176
ident             |             |
Sbjct ---ETKFMGVCQLPSKndEKEYPHRRIDIRLIpKDQY-----------------------  256
DSSP  ---LLEEEEEEELLLLLllLLLLLLLEEEEEEEELlHHHH-----------------------


DSSP  eellhHHHHhhhhhhhhllllllllllllHHHHHHH------------------hhhhh
Query pcxtiPWQIaqklhavtavleepkandraHDLVDLQ------------------llegl  217
ident                               |
Sbjct ----yCGVL----------------yftgSDIFNKNmrahalekgftineytirplgvtg  296
DSSP  ----hHHHH----------------hhhlLHHHHHHhhhhhhhlleeellllleeelllll


DSSP  llhHHHHHhhHHHHhhhhhhlllLLLLLLlllllhhhhhhhhhllllllllllhhhhhh
Query lldADLXPtrSACIaifearaqhPWPPRVatlphwpliyagalegldhlelartvdaaaq  277
ident      |
Sbjct vagEPLPV--DSEK-----difdYIQWKY-------------------------------  318
DSSP  lllLLLLL--LLHH-----hhhhHLLLLL-------------------------------


DSSP  hhhhhhhhhhhllll
Query avqrfvaridratkr  292
ident 
Sbjct -------repkdrse  326
DSSP  -------llhhhlll
```

No 305: Query=mol1A Sbjct=3jpnA Z-score=6.5

back to top

```
DSSP  ------------------------------------------------------------
Query ------------------------------------------------------------    0
ident 
Sbjct tlnggitdmltelanfeknvsqaihkynayrkaasviakyphkiksgaeakklpgvgtki   60
DSSP  lllhhhhhhhhhhhhhhhllllhhhhhhhhhhhhhhhllllllllhhhhhllllllhhh


DSSP  ------------------------------------------------llllllllllhh
Query ------------------------------------------------aapysspptnlr   12
ident 
Sbjct aekideflatgklrklekirqddtsssinfltrvsgigpsaarkfvdegiktledlrkne  120
DSSP  hhhhhhhhhhlllhhhhhhhhlhhhhhhhhhlllllllhhhhhhhhhhlllllhhhhhllh


DSSP  hhhhhhhhhhhhllllhhhhhHHHHHHHHHHHHHH-LLLLllLLLLEEEEHHHHHHHHhhll
Query slrdrltqvaerqgvvfgrlqRHVAXIVVAQFAA-TLTDdtGAPLLLVKGGSSLELrrgi   71
ident                                                | |
Sbjct dklnhhqriglkyfgdfekriPREEMLQMQDIVLnEVKKvdSEYIATVCGSFRRGA----  176
DSSP  hhllhhhhhhhhlhhhhhlleEHHHHHHHHHHHHHhHHHHhlLLLEEEELHHHHLLL----
```

```
DSSP  llllLLLLEEEEEL------------lLHHHHHHHHHHHHhhlllleeEEELLLEELlll
Query pdsrTSKDFATVAR------------rDIELIHEQLADAGetgwegftAIFTAPEEIaap  119
ident      | |                         |||             |
Sbjct ---eSSGDMDVLLThpsftsestkqpkLLHQVVEQLQKVH--------FITDTLSKG---  222
DSSP  ---lEELLEEEEEElllllllllllllHHHHHHHHHHHLL--------LEEEEEEEL---


DSSP  lllLLLEEEEEEEEEL--LEEEEEEEEEEEEL-LLLLlllleeeeelhhhhlllllleee
Query gxpVKPRRFTAKLSYR--GRAFATVPIEVSSV-EAGNadqfdtltsdalglvgvpaavav  176
ident             |             |
Sbjct ---ETKFMGVCQLPSKndEKEYPHRRIDIRLIpKDQY-----------------------  256
DSSP  ---LLEEEEEELLLLLllLLLLLLEEEEEEELlHHHH-----------------------


DSSP  eellhHHHhhhhhhhhhhlllllllllllHHHHHHH-------------------hhhhh
Query pcxtiPWQIaqklhavtavleepkandraHDLVDLQ-------------------llegl  217
ident                               |
Sbjct ----yCGVL----------------yftgSDIFNKNmrahalekgftineytirplgvtg  296
DSSP  ----hHHHH----------------hhhlLHHHHHHhhhhhhhlleeellllleeelllll


DSSP  llhHHHHhhHHHHHhhhhhhllllLLLLLlllllhhhhhhhhhlllllllllllhhhhhh
Query lldADLXptRSACIaifearaqhpWPPRVatlphwpliyagalegldhlelartvdaaaq  277
ident      |
Sbjct vagEPLP--VDSEK-------difDYIQW-------------------------------  316
DSSP  lllLLLL--LLLHH-------hhhHHLLL-------------------------------


DSSP  hhhhhhhhhhhllll
Query avqrfvaridratkr  292
ident
Sbjct -----kyrepkdrse  326
DSSP  -----llllhhhlll
```

No 306: Query=mol1A Sbjct=4do9A Z-score=6.5

back to top

```
DSSP  ------------------------------------------------------------
Query ------------------------------------------------------------    0
ident
Sbjct tlnggitdmltelanfeknvsqaihkynayrkaasviakyphkiksgaeakklpgvgtki   60
DSSP  lllhhhhhhhhhhhhhhhhllllhhhhhhhhhhhhhhhhlllllllhhhhlllllllhhh


DSSP  ------------------------------------------------llllllllllhh
Query ------------------------------------------------aapysspptnlr   12
ident
Sbjct aekideflatgklrklekirqddtsssinfltrvsgigpsaarkfvdegiktledlrkne  120
DSSP  hhhhhhhhhhlllhhhhhhhhlhhhhhhhhhlllllllhhhhhhhhhlllllhhhhhhlh


DSSP  hhhhhhhhhhhhllllhhhhhHHHHHHHHHHHHHH-LLLLllLLLEEEEHHHHHHHHhhll
Query slrdrltqvaerqgvvfgrlqRHVAXIVVAQFAA-TLTDdtGAPLLLVKGGSSLELrrgi   71
ident                                                | |
Sbjct dklnhhqriglkyfgdfekriPREEMLQMQDIVLnEVKKvdSEYIATVCGSFRRGA----  176
DSSP  hhllhhhhhhhhlllhhhlleEHHHHHHHHHHHHhHHHHhlLLLEEEELHHHHLLL----


DSSP  llllLLLLEEEEEL------------lLHHHHHHHHHHHHhhlllleeEEELLLEELlll
Query pdsrTSKDFATVAR------------rDIELIHEQLADAGetgwegftAIFTAPEEIaap  119
ident      | |                         |||             |
Sbjct ---eSSGDMDVLLThpsftsestkqpkLLHQVVEQLQKVH--------FITDTLSKG---  222
DSSP  ---lEELLEEEEEElllllllllllllHHHHHHHHHHHLL--------LEEEEEEEL---


DSSP  lllLLLEEEEEEEEEL--LEEEEEEEEEEEEL-LLLLlllleeeeelhhhhlllllleee
Query gxpVKPRRFTAKLSYR--GRAFATVPIEVSSV-EAGNadqfdtltsdalglvgvpaavav  176
ident             |             |
Sbjct ---ETKFMGVCQLPSKndEKEYPHRRIDIRLIpKDQY-----------------------  256
DSSP  ---LLEEEEEELLLLLllLLLLLLEEEEEEELlHHHH-----------------------


DSSP  eellhHHHhhhhhhhhhhlllllllllllHHHHHHH-------------------hhhhh
Query pcxtiPWQIaqklhavtavleepkandraHDLVDLQ-------------------llegl  217
ident                               |
```

```
DSSP  ----hHHHH----------------hhhlLHHHHHHhhhhhhhlleeellllleeellllll
```

```
DSSP  llhHHHHhhHHHHHhhhhhhllllLLLLLlllllhhhhhhhhhlllllllllllhhhhhh
Query lldADLXptRSACIaifearaqhpWPPRVatlphwpliyagalegldhlelartvdaaaq  277
ident      |
Sbjct vagEPLP--VDSEK-------difDYIQW-------------------------------  316
DSSP  lllLLLL--LLLHH-------hhhHHLLL-------------------------------
```

```
DSSP  hhhhhhhhhhhllll
Query avqrfvaridratkr  292
ident
Sbjct -----kyrepkdrse  326
DSSP  -----llllhhhlll
```

No 307: Query=mol1A Sbjct=3jpoA Z-score=6.5

back to top

```
DSSP  ------------------------------------------------------------
Query ------------------------------------------------------------    0
ident
Sbjct tlnggitdmltelanfeknvsqaihkynayrkaasviakyphkiksgaeakklpgvgtki   60
DSSP  lllhhhhhhhhhhhhhhhhhllllhhhhhhhhhhhhhhhhllllllllhhhhhllllllhhh
```

```
DSSP  ------------------------------------------------llllllllllhh
Query ------------------------------------------------aapyssspptnlr   12
ident
Sbjct aekideflatgklrklekirqddtsssinfltrvsgigpsaarkfvdegiktledlrkne  120
DSSP  hhhhhhhhhhhlllhhhhhhhhlhhhhhhhhhlllllllhhhhhhhhhhlllllhhhhhllh
```

```
DSSP  hhhhhhhhhhhhllllhhhhhHHHHHHHHHHHHHH-LLLLllLLLLEEEEHHHHHHHhhll
Query slrdrltqvaerqgvvfgrlqRHVAXIVVAQFAA-TLTDdtGAPLLLVKGGSSLELrrgi   71
ident                                                |  |
Sbjct dklnhhqriglkyfgdfekriPREEMLQMQDIVLnEVKKvdSEYIATVCGSFRRGA----  176
DSSP  hhllhhhhhhhhlhhhhhlleEHHHHHHHHHHHHHhHHHHhlLLLEEEELHHHHLLL----
```

```
DSSP  llllLLLLEEEEEL------------lLHHHHHHHHHHHHhhlllleeEEELLLEELlll
Query pdsrTSKDFATVAR------------rDIELIHEQLADAGetgwegftAIFTAPEEIaap  119
ident      | |                         |||             |
Sbjct ---eSSGDMDVLLThpsftsestkqpkLLHQVVEQLQKVH--------FITDTLSKG---  222
DSSP  ---lEELLEEEEEEllllllllllllllHHHHHHHHHHHHLL--------LEEEEEEEL---
```

```
DSSP  lllLLLEEEEEEEEEL--LEEEEEEEEEEEEL-LLLLlllleeeeelhhhhllllllleee
Query gxpVKPRRFTAKLSYR--GRAFATVPIEVSSV-EAGNadqfdtltsdalglvgvpaavav  176
ident             |             |
Sbjct ---ETKFMGVCQLPSKndEKEYPHRRIDIRLIpKDQY-----------------------  256
DSSP  ---LLEEEEEEELLLLLllLLLLLLEEEEEEEELlHHHH-----------------------
```

```
DSSP  eellhHHHHhhhhhhhhllllllllllllHHHHHHH-------------------hhhhh
Query pcxtiPWQIaqklhavtavleepkandraHDLVDLQ-------------------llegl  217
ident                               |
Sbjct ----yCGVL----------------yftgSDIFNKNmrahalekgftineytirplgvtg  296
DSSP  ----hHHHH----------------hhhlLHHHHHHhhhhhhhlleeellllleeellllll
```

```
DSSP  llhHHHHhhHHHHHhhhhhhllllLLLLLlllllhhhhhhhhhlllllllllllhhhhhh
Query lldADLXptRSACIaifearaqhpWPPRVatlphwpliyagalegldhlelartvdaaaq  277
ident      |
Sbjct vagEPLP--VDSEK-------difDYIQW-------------------------------  316
DSSP  lllLLLL--LLLHH-------hhhHHLLL-------------------------------
```

```
DSSP  hhhhhhhhhhhllll
Query avqrfvaridratkr  292
ident
Sbjct -----kyrepkdrse  326
DSSP  -----llllhhhlll
```

back to top

```
DSSP  ------------------------------------------------------------
Query ------------------------------------------------------------   0
ident 
Sbjct tlnggitdmltelanfeknvsqaihkynayrkaasviakyphkiksgaeakklpgvgtki  60
DSSP  lllhhhhhhhhhhhhhhhhlllllhhhhhhhhhhhhhhhhlllllllhhhhhlllllhhh


DSSP  ------------------------------------------------llllllllllhh
Query ------------------------------------------------aapysspptnlr  12
ident 
Sbjct aekideflatgklrklekirqddtsssinfltrvsgigpsaarkfvdegiktledlrkne 120
DSSP  hhhhhhhhhhlllhhhhhhhhlhhhhhhhhhlllllllhhhhhhhhhlllllhhhhhhlh


DSSP  hhhhhhhhhhhhllllhhhhhHHHHHHHHHHHHHH-LLLLllLLLLEEEEHHHHHHHhhll
Query slrdrltqvaerqgvvfgrlqRHVAXIVVAQFAA-TLTDdtGAPLLLVKGGSSLELrrgi  71
ident                                                |  |
Sbjct dklnhhqriglkyfgdfekriPREEMLQMQDIVLnEVKKvdSEYIATVCGSFRRGA---- 176
DSSP  hhllhhhhhhhhhlhhhhhlleEHHHHHHHHHHHHHhHHHHhlLLLEEEELHHHHLLL----


DSSP  llllLLLLEEEEEL------------lLHHHHHHHHHHHHhhlllleeEEELLLEELlll
Query pdsrTSKDFATVAR------------rDIELIHEQLADAGetgwegftAIFTAPEEIaap 119
ident      | |                         |||             |
Sbjct ---eSSGDMDVLLThpsftsestkqpkLLHQVVEQLQKVH--------FITDTLSKG--- 222
DSSP  ---lEELLEEEEEEllllllllllllHHHHHHHHHHHHLL--------LEEEEEEEL---


DSSP  lllLLLEEEEEEEEEEL--LEEEEEEEEEEEEL-LLLLllllleeeeelhhhhllllllleee
Query gxpVKPRRFTAKLSYR--GRAFATVPIEVSSV-EAGNadqfdtltsdalglvgvpaavav 176
ident             |             |
Sbjct ---ETKFMGVCQLPSKndEKEYPHRRIDIRLIpKDQY----------------------- 256
DSSP  ---LLEEEEEELLLLLllLLLLLLEEEEEEELlHHHH-----------------------


DSSP  eellhHHHHhhhhhhhhllllllllllllHHHHHHHH-------------------hhhh
Query pcxtiPWQIaqklhavtavleepkandraHDLVDLQL-------------------legl 217
ident                               |
Sbjct ----yCGVL----------------yftgSDIFNKNMrahalekgftineytirplgvtg 296
DSSP  ----hHHHH----------------hhhlLHHHHHHHHhhhhhhlleeellllleeellll


DSSP  llhHHHHhhHHHHHhhhhhhllllLLLLLlllllhhhhhhhhhllllllllllhhhhhh
Query lldADLXptRSACIaifearaqhpWPPRVatlphwpliyagalegldhlelartvdaaaq 277
ident      |
Sbjct vagEPLP--VDSEK-------difDYIQW------------------------------- 316
DSSP  lllLLLL--LLLHH-------hhhHHLLL-------------------------------


DSSP  hhhhhhhhhhhllll
Query avqrfvaridratkr  292
ident 
Sbjct -----kyrepkdrse  326
DSSP  -----llllhhhlll
```

No 309: Query=mol1A Sbjct=3c5gB Z-score=6.5

back to top

```
DSSP  ------------------------------------------------------------
Query ------------------------------------------------------------   0
ident 
Sbjct ssqkatnhnlhiteklevlakaysvqgdkwralgyakainalksfhkpvtsyqeacsipg  60
DSSP  lllllllllhhhhhhhhhhhhhhhhlllhhhhhhhhhhhhhhhhlllllllllhhhhhhlll


DSSP  --------------------------------------------------llllllllll
Query --------------------------------------------------aapysspptn  10
ident 
Sbjct igkrmaekiieilesghlrkldhisesvpvlelfsniwgagtktaqmwyqqgfrsledir 120
DSSP  llhhhhhhhhhhhhhhlllhhhhlllllhhhhhhhhlllllllhhhhhhhhhlllllhhhhh
```

```
DSSP  hhhhhhhhhhhhhhllllhHHHHHHHHHHHHHHHHHLL-LLLLLLLEEEEHHHHHHHHhh
Query lrslrdrltqvaerqgvvfGRLQRHVAXIVVAQFAATL-TDDTGAPLLLVKGGSSLELrr   69
ident                                 |             |    |
Sbjct sqasl ttqqaiglkhysdfLERMPREEATEIEQTVQKAaQAFNSGLLCVACGSYRRGK--  178
DSSP  hhllllhhhhhhhhlhhhhHLLEEHHHHHHHHHHHHHHhHLLLLLLEEEELHHHHLLL--


DSSP  llllllLLLLEEEEEL--------lLHHHHHHHHHHHHhhlllleeeEELLL-EELLLLl
Query gipdsrTSKDFATVAR--------rDIELIHEQLADAGetgwegftaIFTAP-EEIAAPg  120
ident       |  |                       |   |           |
Sbjct -----aTCGDVDVLIThpdgrshrgIFSRLLDSLRQEG---------FLTDDlVSQEEN-  223
DSSP  -----lEELLEEEEEEllllllllHHHHHHHHHHHHLL---------LEEEEeEELLLL-


DSSP  LLLLLEEEEEEEEEllEEEEEEEEEEEEL-LLLLlllleeeeelhhhhlllllleeeeel
Query XPVKPRRFTAKLSYrgRAFATVPIEVSSV-EAGNadqfdtltsdalglvgvpaavavpcx  179
ident            |                |
Sbjct GQQQKYLGVCRLPG--PGRRHRRLDIIVVpYSEF--------------------------  255
DSSP  LLLEEEEEEEELLL--LLLLEEEEEEEELlHHHH--------------------------


DSSP  lhHHHHhhhhhhhhllllllllllllHHHHHHH-----------------------hhhh
Query tiPWQIaqklhavtavleepkandraHDLVDLQ-----------------------lleg  216
ident
Sbjct -aCALL----------------yftgSAHFNRSmkalaktkgmslsehalstavvrhgck  298
DSSP  -hHHHH----------------hhhlLHHHHHHhhhhhhlleeellllleeellllllll


DSSP  hllhHHHHhhHHHHHhhhhhhllllLLLLLlllllhhhhhhhhhlllllllllllhhhhh
Query llldADLXptRSACIaifearaqhpWPPRVatlphwpliyagalegldhlelartvdaaa  276
ident       |
Sbjct vgpgRVLP--TPTEK-------dvfRLLGL------------------------------  319
DSSP  llllLEEL--LLLHH-------hhhHHLLL------------------------------


DSSP  hhhhhhhhhhhhllll
Query qavqrfvaridratkr  292
ident
Sbjct ------pyrepaerdw  329
DSSP  ------llllhhhhll
```

No 310: Query=mol1A Sbjct=3mdaA Z-score=6.5

back to top

```
DSSP  ------------------------------------------------------------
Query ------------------------------------------------------------    0
ident
Sbjct ahnlhiteklevlakaysvqgdkwralgyakainalksfhkpvtsyqeacsipgigkrma   60
DSSP  lllhhhhhhhhhhhhhhhhhlllhhhhhhhhhhhhhhhhllllllllhhhhhhllllhhhh


DSSP  --------------------------------------------llllllllllhhhhhh
Query --------------------------------------------aapysspptnlrslrd   16
ident
Sbjct ekiieilesghlrkldhisesvpvlelfsniwgagtktaqmwyqqgfrsledirsqaslt  120
DSSP  hhhhhhhhllllhhhhlllllhhhhhhhhllllllhhhhhhhhhlllllhhhhhhhllll


DSSP  hhhhhhhhllllhhhhHHHHHHHHHHHHHHL-LLLLLLLLEEEEHHHHHHHHhhllllll
Query rltqvaerqgvvfgrlQRHVAXIVVAQFAAT-LTDDTGAPLLLVKGGSSLELrrgipdsr   75
ident                           |             |    |
Sbjct tqqaiglkhysdflerMPREEATEIEQTVQKaAQAFNSGLLCVACGSYRRGK-------a  173
DSSP  hhhhhhhhlllhhhllEEHHHHHHHHHHHHHhHHLLLLLLEEEELHHHHLLL-------l


DSSP  LLLLEEEEEL--------lLHHHHHHHHHHHHhhlllleeEEELLLEELlllllLLLLEE
Query TSKDFATVAR--------rDIELIHEQLADAGetgwegftAIFTAPEEIaapgxPVKPRR  127
ident |  |                       |   |
Sbjct TCGDVDVLIThpdgrshrgIFSRLLDSLRQEG--------FLTDDLVSQ-eengQQQKYL  224
DSSP  EELLEEEEEEllllllllLHHHHHHHHHHHLL--------LEEEEEELL-llllLLLEEE


DSSP  EEEEEEEllEEEEEEEEEEEELLLLLlllleeeeelhhhhlllllleeeeellhHHHHhh
Query FTAKLSYrgRAFATVPIEVSSVEAGNadqfdtltsdalglvgvpaavavpcxtiPWQIaq  187
ident     |                |
```

```
DSSP  EEELLLL--LLLLLEEEEEEELLHHH--------------------------hhHHHH--


DSSP  hhhhhhllllllllllllHHHHHHH-------------------------hhhhhllhHH
Query klhavtavleepkandraHDLVDLQ-------------------------llegllldAD  222
ident
Sbjct --------------yftgSAHFNRSmralaktkgmslsehalstavvrnthgckvgpgRV  300
DSSP  --------------hhhlLHHHHHHhhhhhhhlleellllleeelleellllleeellEE


DSSP  HHhhHHHHHhhhhhhllllLLLLLlllllhhhhhhhhhllllllllllhhhhhhhhhhhh
Query LXptRSACIaifearaqhpWPPRVatlphwpliyagalegldhlelartvdaaaqavqrf  282
ident |
Sbjct LP--TPTEK-------dvfRLLGL------------------------------------  315
DSSP  EL--LLLHH-------hhhHHLLL------------------------------------


DSSP  hhhhhhllll
Query varidratkr  292
ident
Sbjct pyrepaerdw  325
DSSP  llllhhhlll
```

No 311: Query=mol1A Sbjct=3uq0A Z-score=6.5

back to top

```
DSSP  ------------------------------------------------------------
Query ------------------------------------------------------------    0
ident
Sbjct nhnlhiteklevlakaysvqgdkwralgyakainalksfhkpvtsyqeacsipgigkrma   60
DSSP  lllhhhhhhhhhhhhhhhhlllhhhhhhhhhhhhhhlllllllllhhhhhhlllllhhhh


DSSP  --------------------------------------------llllllllllhhhhhh
Query --------------------------------------------aapysspptnlrslrd   16
ident
Sbjct ekiieilesghlrkldhisesvpvlelfsniwgagtktaqmwyqqgfrsledirsqaslt  120
DSSP  hhhhhhhhhlllhhhhhlllllhhhhhhhhlllllhhhhhhhhhlllllhhhhhhhllll


DSSP  hhhhhhhhllllhhhhHHHHHHHHHHHHHH-LLLLllLLLLEEEEHHHHHHHhhllllll
Query rltqvaerqgvvfgrlQRHVAXIVVAQFAA-TLTDdtGAPLLLVKGGSSLELrrgipdsr   75
ident                           |             |    |
Sbjct tqqaiglkhysdflerMPREEATEIEQTVQkAAQAfnSGLLCVACGSYRRGK-------a  173
DSSP  hhhhhhhhlhhhhhllEEHHHHHHHHHHHHhHHHLllLLLEEEELHHHHLLL-------l


DSSP  LLLLEEEEEL--------lLHHHHHHHHHHHHhhlllleeEEELLLEELllllllLLLEE
Query TSKDFATVAR--------rDIELIHEQLADAGetgwegftAIFTAPEEIaapgxpVKPRR  127
ident |  |                       |   |
Sbjct TCGDVDVLIThpdgrshrgIFSRLLDSLRQEG--------FLTDDLVKG------ETKYL  219
DSSP  EELLEEEEEElllllllllHHHHHHHHHHHLL--------LEEEEEEEL------LLEEE


DSSP  EEEEEEEllEEEEEEEEEEEELLLLLLllleeeeelhhhhlllllleeeeellhHHHHhh
Query FTAKLSYrgRAFATVPIEVSSVEAGNAdqfdtltsdalglvgvpaavavpcxtiPWQIaq  187
ident     |                |
Sbjct GVCRLPG--PGRRHRRLDIIVVPYSEF--------------------------aCALL--  249
DSSP  EEELLLL--LLLLLEEEEEEELLHHHH--------------------------hHHHH--


DSSP  hhhhhhllllllllllllHHHHHHH---------------hhhhhllhHHHHhhHHHHHh
Query klhavtavleepkandraHDLVDLQ---------------llegllldADLXptRSACIa  232
ident                                                   |
Sbjct --------------yftgSAHFNRSmralaktkgmslsehalstavpgRVLP--TPTEK-  292
DSSP  --------------hhhlLHHHHHHhhhhhhhlleeelllleeellllLLLL--LLLHH-


DSSP  hhhhhllllLLLLLlllllhhhhhhhhhllllllllllhhhhhhhhhhhhhhhhhhllll
Query ifearaqhpWPPRVatlphwpliyagalegldhlelartvdaaaqavqrfvaridratkr  292
ident
Sbjct ------dvfRLLGL------------------------------------pyrepaerdw  310
DSSP  ------hhhHHLLL------------------------------------llllhhhhll
```

back to top

```
DSSP  ------------------------------------------------------------
Query ------------------------------------------------------------   0
ident 
Sbjct tnhnlhiteklevlakaysvqgdkwralgyakainalksfhkpvtsyqeacsipgigkrm   60
DSSP  lllhhhhhhhhhhhhhhhhllllhhhhhhhhhhhhhhhhhlllllllhhhhhhllllllhhh


DSSP  ---------------------------------------------llllllllllhhhhh
Query ---------------------------------------------aapysspptnlrslr   15
ident 
Sbjct aekiieilesghlrkldhisesvpvlelfsniwgagtktaqmwyqqgfrsledirsqasl  120
DSSP  hhhhhhhhhlllhhhllllllhhhhhhhllllllhhhhhhhhhhllllhhhhhhhllll


DSSP  hhhhhhhhllllhHHHHHHHHHHHHHHHHHHLL--LLLLlLLLEEEEHHHHHHHHhhllll
Query drltqvaerqgvvfGRLQRHVAXIVVAQFAATL--TDDTgAPLLLVKGGSSLELrrgipd   73
ident                            |              |    |
Sbjct ttqqaiglkhysdfLERMPREEATEIEQTVQKAaqAFNS-GLLCVACGSYRRGK------  173
DSSP  lhhhhhhhhlllhhHLLEEHHHHHHHHHHHHHHHHhhLLLL-LLEEEELHHHHLLL------


DSSP  llLLLLEEEEEL--------lLHHHHHHHHHHHHhhlllleeEEELLLEELllllllLLL
Query srTSKDFATVAR--------rDIELIHEQLADAGetgwegftAIFTAPEEIaapgxpVKP  125
ident   |  |                       |   |
Sbjct -aTCGDVDVLIThpdgrshrgIFSRLLDSLRQEG--------FLTDDLVKG------ETK  218
DSSP  -lEELLEEEEEEllllllllLHHHHHHHHHHHLL--------LEEEEEEEL------LLE


DSSP  EEEEEEEEEllEEEEEEEEEEEEELLLLLlllleeeeelhhhhlllllleeeeellhHHHH
Query RRFTAKLSYrgRAFATVPIEVSSVEAGNadqfdtltsdalglvgvpaavavpcxtiPWQI  185
ident       |                |
Sbjct YLGVCRLPG--PGRRHRRLDIIVVPYSE--------------------------faCALL  250
DSSP  EEEEELLLL--LLLLLEEEEEEELLHHH--------------------------hhHHHH


DSSP  hhhhhhhlllllllllllllHHHHHHHH-------------------------hhhhllh
Query aqklhavtavleepkandraHDLVDLQL-------------------------legllld  220
ident 
Sbjct ----------------yftgSAHFNRSMralaktkgmslsehalstavvrnthgckvgpg  294
DSSP  ----------------hhhlLHHHHHHHhhhhhhlleeellllleeellllllllllllll


DSSP  HHHHhhHHHHHhhhhhhllllLLLLLllllhhhhhhhhhlllllllllllhhhhhhhhh
Query ADLXptRSACIaifearaqhpWPPRVatlphwpliyagalegldhlelartvdaaaqavq  280
ident   |
Sbjct RVLP--TPTEK-------dvfRLLGL----------------------------------  311
DSSP  LEEL--LLLHH-------hhhHHLLL----------------------------------


DSSP  hhhhhhhhllll
Query rfvaridratkr  292
ident 
Sbjct --pyrepaerdw  321
DSSP  --llllhhhlll
```

No 313: Query=mol1A Sbjct=2ispA Z-score=6.5

back to top

```
DSSP  ------------------------------------------------------------
Query ------------------------------------------------------------   0
ident 
Sbjct tlnggitdmltelanfeknvsqaihkynayrkaasviakyphkiksgaeakklpgvgtki   60
DSSP  lllhhhhhhhhhhhhhhhhhllllhhhhhhhhhhhhhhhhhlllllllhhhhhhllllllhhh


DSSP  ------------------------------------------------llllllllllhh
Query ------------------------------------------------aapysspptnlr   12
ident 
Sbjct aekideflatgklrklekirqddtsssinfltrvsgigpsaarkfvdegiktledlrkne  120
DSSP  hhhhhhhhhlllhhhhhhhhlhhhhhhhhhlllllllhhhhhhhhhlllllhhhhhhllh
```

```
DSSP  hhhhhhhhhhhhllllhhhhhHHHHHHHHHHHHHH-LLLLlLLLLLEEEEHHHHHHHHHhll
Query slrdrltqvaerqgvvfgrlqRHVAXIVVAQFAA-TLTDdtGAPLLLVKGGSSLELrrgi   71
ident                                                |  |
Sbjct dklnhhqriglkyfgdfekriPREEMLQMQDIVLnEVKKvdSEYIATVCGSFRRGA----  176
DSSP  hhllhhhhhhhhhlllhhhlleEHHHHHHHHHHHHHhHHHHhlLLLEEEELHHHHLLL----


DSSP  llllLLLLEEEEEL------------lLHHHHHHHHHHHHhhllllleeEEELLLEELlll
Query pdsrTSKDFATVAR------------rDIELIHEQLADAGetgwegftAIFTAPEEIaap  119
ident      | |                         |||             |
Sbjct ---eSSGDMDVLLThpsftsestkqpkLLHQVVEQLQKVH--------FITDTLSKG---  222
DSSP  ---lEELLEEEEEElllee llee llllHHHHHHHHHHHHLL--------LEEEEEEEL---


DSSP  lllLLLEEEEEEEEEEL--LEEEEEEEEEEEEL-LLLLlllleeeeelhhhhlllllleee
Query gxpVKPRRFTAKLSYR--GRAFATVPIEVSSV-EAGNadqfdtltsdalglvgvpaavav  176
ident             |             |
Sbjct ---ETKFMGVCQLPSKndEKEYPHRRIDIRLIpKDQY-----------------------  256
DSSP  ---LLEEEEEEELLLLLllLLLLLLLEEEEEEEELlHHHH-----------------------


DSSP  eellhHHHHhhhhhhhhlllllllllllHHHHHHH------------------hhhhh
Query pcxtiPWQIaqklhavtavleepkandraHDLVDLQ------------------llegl  217
ident                               |
Sbjct ----yCGVL----------------yftgSDIFNKNmrahalekgftineytirplgvtg  296
DSSP  ----hHHHH----------------hhhlLHHHHHHhhhhhhhhleeellllleeellll


DSSP  llhHHHHhhHHHHHhhhhhhllllLLLLLlllllhhhhhhhhhllllllllllhhhhhh
Query lldADLXptRSACIaifearaqhpWPPRVatlphwpliyagalegldhlelartvdaaaq  277
ident      |
Sbjct vagEPLP--VDSEK-------difDYIQW-------------------------------  316
DSSP  lllLLLL--LLLHH-------hhhHHLLL-------------------------------


DSSP  hhhhhhhhhhhllll
Query avqrfvaridratkr  292
ident
Sbjct -----kyrepkdrse  326
DSSP  -----llllhhhlll
```

No 314: Query=mol1A Sbjct=7icgA Z-score=6.5

back to top

```
DSSP  ------------------------------------------------------------
Query ------------------------------------------------------------    0
ident
Sbjct etlnggitdmltelanfeknvsqaihkynayrkaasviakyphkiksgaeakklpgvgtk   60
DSSP  llllhhhhhhhhhhhhhhhhhhlllhhhhhhhhhhhhhhhhhlllllllhhhhhlllllhh


DSSP  --------------------------------------------------llllllllllh
Query --------------------------------------------------aapysspptnl   11
ident
Sbjct iaekideflatgklrklekirqddtsssinfltrvsgigpsaarkfvdegiktledlrkn  120
DSSP  hhhhhhhhhhllllhhhhhhhhlhhhhhhhhhllllllhhhhhhhhlllllhhhhhhl


DSSP  hhhhhhhhhhhhllllhhhhhHHHHHHHHHHHHHH-LLLLLLLLLLLEEEEHHHHHHHHhl
Query rslrdrltqvaerqgvvfgrlqRHVAXIVVAQFAA-TLTDDTGAPLLLVKGGSSLELrrg   70
ident                                                 |  |
Sbjct edklnhhqriglkyfgdfekriPREEMLQMQDIVLnEVKKVDSEYIATVCGSFRRGA---  177
DSSP  hhhllhhhhhhhhlhhhhhlleEHHHHHHHHHHHHHhHHHHHLLLLEEEELLHHHHLL---


DSSP  lllllLLLLEEEEEL------------lLHHHHHHHHHHHHhhlllleeEEELLLEELll
Query ipdsrTSKDFATVAR------------rDIELIHEQLADAGetgwegftAIFTAPEEIaa  118
ident       | |                         |||             |
Sbjct ----eSSGDMDVLLThpsftsestkqpkLLHQVVEQLQKVH--------FITDTLSKG--  223
DSSP  ----lEELLEEEEEEellllllllllhHHHHHHHHHHHHLL--------LEEEEEEEL--


DSSP  llllLLLEEEEEEEEEELL--EEEEEEEEEEEEL-LLLLlllleeeeelhhhhllllllee
Query pgxpVKPRRFTAKLSYRG--RAFATVPIEVSSV-EAGNadqfdtltsdalglvgvpaava  175
ident              |             |
```

```
DSSP  ----LLEEEEEEELLLLLllLLLLLEEEEEEEEeHHHH----------------------


DSSP  eeellhHHHHhhhhhhhhllllllllllllhhhhHHHHHH--------------------
Query vpcxtiPWQIaqklhavtavleepkandrahdlvDLQLLE--------------------  215
ident
Sbjct -----yCGVL--------------------yftgSDIFNKnmrahalekgftineytirp  292
DSSP  -----hHHHH--------------------hhhlLHHHHHhhhhhhhhhlleeeelleeee


DSSP  -hhllhhhhhhhHHHHHHhhhhhllllLLLLLlllllhhhhhhhhhllllllllllhhh
Query -gllldadlxptRSACIAifearaqhpWPPRVatlphwpliyagaleglhelartvda  274
ident
Sbjct lgvtgvageplpVDSEKD------ifdYIQWK----------------------------  318
DSSP  llllllllllllLLLHHH------hhhHLLLL----------------------------


DSSP  hhhhhhhhhhhhhhllll
Query aaqavqrfvaridratkr  292
ident
Sbjct ---------yrepkdrse  327
DSSP  ---------lllhhhhll
```

No 315: Query=mol1A Sbjct=8iclA Z-score=6.5

back to top

```
DSSP  ------------------------------------------------------------
Query ------------------------------------------------------------    0
ident
Sbjct etlnggitdmltelanfeknvsqaihkynayrkaasviakyphkiksgaeakklpgvgtk   60
DSSP  llllhhhhhhhhhhhhhhhllllllhhhhhhhhhhhhhhhhhllllllllhhhhhllllllhh


DSSP  -------------------------------------------------llllllllllh
Query -------------------------------------------------aapyssppptnl   11
ident
Sbjct iaekideflatgklrklekirqddtsssinfltrvsgigpsaarkfvdegiktledlrkn  120
DSSP  hhhhhhhhhhllllhhhhhhhhlhhhhhhhhhllllllhhhhhhhhhhhllllhhhhhhl


DSSP  hhhhhhhhhhhhhllllhhhhhHHHHHHHHHHHHH-LLLLLLLLLEEEEHHHHHHHHhhl
Query rslrdrltqvaerqgvvfgrlqRHVAXIVVAQFAA-TLTDDTGAPLLLVKGGSSLELrrg   70
ident                                                 | |
Sbjct edklnhhqriglkyfgdfekriPREEMLQMQDIVLnEVKKVDSEYIATVCGSFRRGA---  177
DSSP  hhhllhhhhhhhhlhhhhhlleEHHHHHHHHHHHHHhHHHHHLLLLEEEELHHHHLLL---


DSSP  lllllLLLLEEEEEEL------------lLHHHHHHHHHHHHhhlllleeEEELLLEELll
Query ipdsrTSKDFATVAR------------rDIELIHEQLADAGetgwegftAIFTAPEEIaa  118
ident       | |                         |||             |
Sbjct ----eSSGDMDVLLThpsftsestkqpkLLHQVVEQLQKVH--------FITDTLSKG--  223
DSSP  ----lEELLEEEEEEellllllllllhhHHHHHHHHHHHHLL--------LEEEEEEEL--


DSSP  llllLLLEEEEEEEEEELL--EEEEEEEEEEEEL-LLLLlllleeeeelhhhhllllllee
Query pgxpVKPRRFTAKLSYRG--RAFATVPIEVSSV-EAGNadqfdtltsdalglvgvpaava  175
ident              |             |
Sbjct ----ETKFMGVCQLPSKNdeKEYPHRRIDIRLIpKDQY----------------------  257
DSSP  ----LLEEEEEEELLLLLllLLLLLEEEEEEEEeLLLH----------------------


DSSP  eeellhHHHHhhhhhhhhllllllllllllhhhhHHHHHH--------------------
Query vpcxtiPWQIaqklhavtavleepkandrahdlvDLQLLE--------------------  215
ident
Sbjct -----yCGVL--------------------yftgSDIFNKnmrahalekgftineytirp  292
DSSP  -----hHHHH--------------------hhhlLHHHHHhhhhhhhhhlleeeelleeee


DSSP  -hhllhhhhhhhHHHHHHhhhhhllllLLLLLlllllhhhhhhhhhllllllllllhhh
Query -gllldadlxptRSACIAifearaqhpWPPRVatlphwpliyagaleglhelartvda  274
ident
Sbjct lgvtgvageplpVDSEK-------difDYIQW----------------------------  317
DSSP  llllllllllllLLLHH-------hhhHHHLL----------------------------
```

```
Query aaqavqrfvaridratkr  292
ident
Sbjct --------kyrepkdrse  327
DSSP  --------llllhhhlll
```

No 316: Query=mol1A Sbjct=1zqbA Z-score=6.5

back to top

```
DSSP  ------------------------------------------------------------
Query ------------------------------------------------------------   0
ident
Sbjct etlnggitdmltelanfeknvsqaihkynayrkaasviakyphkiksgaeakklpgvgtk   60
DSSP  llllhhhhhhhhhhhhhhhhllllhhhhhhhhhhhhhhhhlllllllhhhhhllllllhh


DSSP  -------------------------------------------------llllllllllh
Query -------------------------------------------------aapysspptnl   11
ident
Sbjct iaekideflatgklrklekirqddtsssinfltrvsgigpsaarkfvdegiktledlrkn  120
DSSP  hhhhhhhhhhllllhhhhhhhhlhhhhhhhhhlllllllhhhhhhhhhhllllhhhhhhl


DSSP  hhhhhhhhhhhhhllllhhhhhHHHHHHHHHHHHH-LLLLllLLLLLEEEEHHHHHHHHhl
Query rslrdrltqvaerqgvvfgrlqRHVAXIVVAQFAA-TLTDdtGAPLLLVKGGSSLELrrg   70
ident                                                  | |
Sbjct edklnhhqriglkyfgdfekriPREEMLQMQDIVLnEVKKvdSEYIATVCGSFRRGA---  177
DSSP  hhhllhhhhhhhhlhhhhhlleEHHHHHHHHHHHHHhHHHHhlLLLEEEEEELLLLLLL---


DSSP  lllllLLLLEEEEEL------------lLHHHHHHHHHHHHhhllllleeEEELLLEELll
Query ipdsrTSKDFATVAR------------rDIELIHEQLADAGetgwegftAIFTAPEEIaa  118
ident       | |                            |||             |
Sbjct ----eSSGDMDVLLThpsftsestkqpkLLHQVVEQLQKVH--------FITDTLSKG--  223
DSSP  ----lEELLEEEEEEelllllllllhhHHHHHHHHHHHHLL--------LEEEEEEEL--


DSSP  llllLLLEEEEEEEEEELL--EEEEEEEEEEEEL-LLLLllllleeeeelhhhhlllllllee
Query pgxpVKPRRFTAKLSYRG--RAFATVPIEVSSV-EAGNadqfdtltsdalglvgvpaava  175
ident              |              |
Sbjct ----ETKFMGVCQLPSKNdeKEYPHRRIDIRLIpKDQY----------------------  257
DSSP  ----LLLEEEEELLLLLLllLLLLLEEEEEEEEeLLLH----------------------


DSSP  eeellhHHHHhhhhhhhhllllllllllllhhhhHHHHHH--------------------
Query vpcxtiPWQIaqklhavtavleepkandrahdlvDLQLLE--------------------  215
ident
Sbjct -----yCGVL--------------------yftgSDIFNKnmrahalekgftineytirp  292
DSSP  -----hHHHH--------------------hhllLHHHHHhhhhhhhhlleeeelleeee


DSSP  --hhllhhhhhhhhHHHHhhhhhhllllLLLLLlllllhhhhhhhhhllllllllllhh
Query --glllldadlxptrSACIaifearaqhpWPPRVatlphwpliyagalegldhlelartvd  273
ident                |
Sbjct lgvtgvageplpvdSEKD-------ifdYIQWK---------------------------  318
DSSP  llllllllllllllLHHH-------hhhHLLLL---------------------------


DSSP  hhhhhhhhhhhhhhllll
Query aaaqavqrfvaridratkr  292
ident
Sbjct ----------yrepkdrse  327
DSSP  ----------lllhhhlll
```

No 317: Query=mol1A Sbjct=7icnA Z-score=6.5

back to top

```
DSSP  ------------------------------------------------------------
Query ------------------------------------------------------------   0
ident
Sbjct etlnggitdmltelanfeknvsqaihkynayrkaasviakyphkiksgaeakklpgvgtk   60
DSSP  llllhhhhhhhhhhhhhhhhllllhhhhhhhhhhhhhhhhlllllllhhhhhllllllhh
```

```
DSSP  -------------------------------------------------llllllllllh
Query -------------------------------------------------aapysspptnl   11
ident
Sbjct iaekideflatgklrklekirqddtsssinfltrvsgigpsaarkfvdegiktledlrkn  120
DSSP  hhhhhhhhhhllllhhhhhhlllhhhhhhhhhlllllllhhhhhhhhllllllhhhhhhl


DSSP  hhhhhhhhhhhhhllllhhhhhHHHHHHHHHHHHH-LLLLllLLLLEEEEHHHHHHHhhl
Query rslrdrltqvaerqgvvfgrlqRHVAXIVVAQFAA-TLTDdtGAPLLLVKGGSSLELrrg   70
ident                                                 | |
Sbjct edklnhhqriglkyfgdfekriPREEMLQMQDIVLnEVKKvdSEYIATVCGSFRRGA---  177
DSSP  hhhllhhhhhhhhlhhhhhlleEHHHHHHHHHHHHhHHHHhlLLLEEEELLLLLLLL---


DSSP  lllllLLLLEEEEEL------------lLHHHHHHHHHHHHhhlllleeEEELLLEELll
Query ipdsrTSKDFATVAR------------rDIELIHEQLADAGetgwegftAIFTAPEEIaa  118
ident       | |                         |||             |
Sbjct ----eSSGDMDVLLThpsftsestkqpkLLHQVVEQLQKVH--------FITDTLSKG--  223
DSSP  ----lEELLEEEEEElllllllllllhHHHHHHHHHHHHLL--------LEEEEEEEL--


DSSP  llllLLLEEEEEEEEEELL--EEEEEEEEEEEEL-LLLLlllleeeeelhhhhllllllee
Query pgxpVKPRRFTAKLSYRG--RAFATVPIEVSSV-EAGNadqfdtltsdalglvgvpaava  175
ident              |             |
Sbjct ----ETKFMGVCQLPSKNdeKEYPHRRIDIRLIpKDQY----------------------  257
DSSP  ----LLEEEEEELLLLLllLLLLLLEEEEEEEELlHHHH----------------------


DSSP  eeellhHHHHhhhhhhhhllllllllllllhhhhHHHHHH--------------------
Query vpcxtiPWQIaqklhavtavleepkandrahdlvDLQLLE--------------------  215
ident
Sbjct -----yCGVL--------------------yftgSDIFNKnmrahalekgftineytirp  292
DSSP  -----hHHHH--------------------hhhlLHHHHHhhhhhhhhlleeeelleeee


DSSP  -hhllhhhhhhhHHHHHhhhhhhllllLLLLLllllhhhhhhhhhllllllllllhhh
Query -glllldadlxptRSACIaifearaqhpWPPRVatlphwpliyagalegldhlelartvda  274
ident
Sbjct lgvtgvageplpVDSEK-------difDYIQW----------------------------  317
DSSP  llllllllllllLLLHH-------hhhHHLLL----------------------------


DSSP  hhhhhhhhhhhhhhllll
Query aaqavqrfvaridratkr  292
ident
Sbjct --------kyrepkdrse  327
DSSP  --------llllhhhhll
```

No 318: Query=mol1A Sbjct=1zqdA Z-score=6.5

back to top

```
DSSP  ------------------------------------------------------------
Query ------------------------------------------------------------    0
ident
Sbjct etlnggitdmltelanfeknvsqaihkynayrkaasviakyphkiksgaeakklpgvgtk   60
DSSP  llllhhhhhhhhhhhhhhhhllllhhhhhhhhhhhhhhhhllllllhhhhlllllllhh


DSSP  -------------------------------------------------llllllllllh
Query -------------------------------------------------aapysspptnl   11
ident
Sbjct iaekideflatgklrklekirqddtsssinfltrvsgigpsaarkfvdegiktledlrkn  120
DSSP  hhhhhhhhhhllllhhhhhhhhlhhhhhhhhhlllllllhhhhhhhhhhllllhhhhhhl


DSSP  hhhhhhhhhhhhhllllhhhhhHHHHHHHHHHHHH-LLLLllLLLLEEEEHHHHHHHhhl
Query rslrdrltqvaerqgvvfgrlqRHVAXIVVAQFAA-TLTDdtGAPLLLVKGGSSLELrrg   70
ident                                                 | |
Sbjct edklnhhqriglkyfgdfekriPREEMLQMQDIVLnEVKKvdSEYIATVCGSFRRGA---  177
DSSP  hhhllhhhhhhhhlhhhhhlleEHHHHHHHHHHHHhHHHHhlLLLEEEELHHHHLLL---


DSSP  lllllLLLLEEEEEL------------lLHHHHHHHHHHHHhhlllleeEEELLLEELll
Query ipdsrTSKDFATVAR------------rDIELIHEQLADAGetgwegftAIFTAPEEIaa  118
```

```
Sbjct ----eSSGDMDVLLThpsftsestkqpkLLHQVVEQLQKVH--------FITDTLSKG--  223
DSSP  ----lEELLEEEEEEellllllllllhhHHHHHHHHHHHHL--------LEEEEEEEL--


DSSP  llllLLLEEEEEEEEEELL--EEEEEEEEEEEEL-LLLLllllleeeeelhhhhllllllee
Query pgxpVKPRRFTAKLSYRG--RAFATVPIEVSSV-EAGNadqfdtltsdalglvgvpaava  175
ident              |             |
Sbjct ----ETKFMGVCQLPSKNdeKEYPHRRIDIRLIpKDQY----------------------  257
DSSP  ----LLEEEEEELLLLLLllLLLLLEEEEEEEEeLLLH----------------------


DSSP  eeellhHHHHhhhhhhhhlllllllllllhhhhHHHHHH--------------------
Query vpcxtiPWQIaqklhavtavleepkandrahdlvDLQLLE--------------------  215
ident
Sbjct -----yCGVL--------------------yftgSDIFNKnmrahalekgftineytirp  292
DSSP  -----hHHHH--------------------hhllLHHHHHhhhhhhhhlleeeelleeee


DSSP  -hhllhhhhhhhHHHHHhhhhhhllllLLLLLllllllhhhhhhhhhllllllllllhhh
Query -gllldadlxptRSACIaifearaqhpWPPRVatlphwpliyagalegldhlelartvda  274
ident
Sbjct lgvtgvageplpVDSEK-------difDYIQW----------------------------  317
DSSP  llllllllllllLLLHH-------hhhHHHLL----------------------------


DSSP  hhhhhhhhhhhhhhllll
Query aaqavqrfvaridratkr  292
ident
Sbjct --------kyrepkdrse  327
DSSP  --------llllhhhlll
```

No 319: Query=mol1A Sbjct=7icfA Z-score=6.5

back to top

```
DSSP  ------------------------------------------------------------
Query ------------------------------------------------------------    0
ident
Sbjct etlnggitdmltelanfeknvsqaihkynayrkaasviakyphkiksgaeakklpgvgtk   60
DSSP  llllhhhhhhhhhhhhhhhhllllhhhhhhhhhhhhhhhhlllllllhhhhhlllllllh


DSSP  -------------------------------------------------llllllllllh
Query -------------------------------------------------aapyssspptnl   11
ident
Sbjct iaekideflatgklrklekirqddtsssinfltrvsgigpsaarkfvdegiktledlrkn  120
DSSP  hhhhhhhhhhllllhhhhhhlllhhhhhhhhhllllllhhhhhhhhhhllllhhhhhhl


DSSP  hhhhhhhhhhhhhllllhhhhhHHHHHHHHHHHHHH-LLLLlLLLLEEEEHHHHHHHHhhl
Query rslrdrltqvaerqgvvfgrlqRHVAXIVVAQFAA-TLTDdtGAPLLLVKGGSSLELrrg   70
ident                                                 | |
Sbjct edklnhhqriglkyfgdfekriPREEMLQMQDIVLnEVKKvdSEYIATVCGSFRRGA---  177
DSSP  hhhllhhhhhhhhlhhhhhlleEHHHHHHHHHHHHHHhHHHHhlLLLEEEELLHHHHLL---


DSSP  lllllLLLLEEEEEEL------------lLHHHHHHHHHHHHHhhllllleeEEELLLEELll
Query ipdsrTSKDFATVAR------------rDIELIHEQLADAGetgwegftAIFTAPEEIaa  118
ident       | |                         |||             |
Sbjct ----eSSGDMDVLLThpsftsestkqpkLLHQVVEQLQKVH--------FITDTLSKG--  223
DSSP  ----lEELLEEEEEEellllllllllhHHHHHHHHHHHHLL--------LEEEEEEEL--


DSSP  llllLLLEEEEEEEEEELL--EEEEEEEEEEEEL-LLLLllllleeeeelhhhhllllllee
Query pgxpVKPRRFTAKLSYRG--RAFATVPIEVSSV-EAGNadqfdtltsdalglvgvpaava  175
ident              |             |
Sbjct ----ETKFMGVCQLPSKNdeKEYPHRRIDIRLIpKDQY----------------------  257
DSSP  ----LLEEEEEELLLLLLllLLLLLEEEEEEEEeLLLH----------------------


DSSP  eeellhHHHHHHhhhhhhlllllllllllhhhhHHHHHH--------------------
Query vpcxtiPWQIAQklhavtavleepkandrahdlvDLQLLE--------------------  215
ident
Sbjct -----yCGVLYF--------------------tgSDIFNKnmrahalekgftineytirp  292
DSSP  -----hHHHHHL--------------------llLHHHHHhhhhhhhhlleeellllleee
```

```
DSSP  --hhllhhhhhhhhHHHHhhhhhhllllLLLLLlllllhhhhhhhhhlllllllllllhh
Query --gllldadlxptrSACIaifearaqhpWPPRVatlphwpliyagalegldhlelartvd  273
ident               |
Sbjct lgvtgvageplpvdSEKD-------ifdYIQWK---------------------------  318
DSSP  llllllllllllllLHHH-------hhhHLLLL---------------------------
```

```
DSSP  hhhhhhhhhhhhhhhllll
Query aaaqavqrfvaridratkr  292
ident
Sbjct ----------yrepkdrse  327
DSSP  ----------lllllllll
```

No 320: Query=mol1A Sbjct=9iccA Z-score=6.5

back to top

```
DSSP  ------------------------------------------------------------
Query ------------------------------------------------------------    0
ident
Sbjct etlnggitdmltelanfeknvsqaihkynayrkaasviakyphkiksgaeakklpgvgtk   60
DSSP  llllhhhhhhhhhhhhhhhlllllhhhhhhhhhhhhhhhhllllllllhhhhhllllllhh
```

```
DSSP  -------------------------------------------------llllllllllh
Query -------------------------------------------------aapyssspptnl   11
ident
Sbjct iaekideflatgklrklekirqddtsssinfltrvsgigpsaarkfvdegiktledlrkn  120
DSSP  hhhhhhhhhhllllhhhhhhhhlhhhhhhhhhlllllllhhhhhhhhhlllllhhhhhhl
```

```
DSSP  hhhhhhhhhhhhhllllhhhhhHHHHHHHHHHHHH-LLLLllLLLLEEEEHHHHHHHhhl
Query rslrdrltqvaerqgvvfgrlqRHVAXIVVAQFAA-TLTDdtGAPLLLVKGGSSLELrrg   70
ident                                                 | |
Sbjct edklnhhqriglkyfgdfekriPREEMLQMQDIVLnEVKKvdSEYIATVCGSFRRGA---  177
DSSP  hhhllhhhhhhhhlhhhhhlleEHHHHHHHHHHHHhHHHHhlLLLEEEEEELLLLLL---
```

```
DSSP  lllllLLLLEEEEEL------------lLHHHHHHHHHHHHhhlllleeEEELLLEELll
Query ipdsrTSKDFATVAR------------rDIELIHEQLADAGetgwegftAIFTAPEEIaa  118
ident       | |                         |||             |
Sbjct ----eSSGDMDVLLThpsftsestkqpkLLHQVVEQLQKVH--------FITDTLSKG--  223
DSSP  ----lEELLEEEEEEellllllllllllhHHHHHHHHHHLL--------LEEEEEEEL--
```

```
DSSP  llllLLLEEEEEEEEELL--EEEEEEEEEEEEL-LLLLlllleeeeelhhhhllllllee
Query pgxpVKPRRFTAKLSYRG--RAFATVPIEVSSV-EAGNadqfdtltsdalglvgvpaava  175
ident              |             |
Sbjct ----ETKFMGVCQLPSKNdeKEYPHRRIDIRLIpKDQY----------------------  257
DSSP  ----LLEEEEEEELLLLLllLLLLLEEEEEEEEeHHHH----------------------
```

```
DSSP  eeellhHHHHhhhhhhhhllllllllllllhhhhHHHHHH--------------------
Query vpcxtiPWQIaqklhavtavleepkandrahdlvDLQLLE--------------------  215
ident
Sbjct -----yCGVL--------------------yftgSDIFNKnmrahalekgftineytirp  292
DSSP  -----hHHHH--------------------hhhlLHHHHHhhhhhhhhlleeeelleeee
```

```
DSSP  -hhllhhhhhhhHHHHHhhhhhhllllLLLLLllllhhhhhhhhhllllllllllllhhh
Query -gllldadlxptRSACIaifearaqhpWPPRVatlphwpliyagalegldhlelartvda  274
ident
Sbjct lgvtgvageplpVDSEK-------difDYIQW----------------------------  317
DSSP  llllllllllllLLLHH-------hhhHHLLL----------------------------
```

```
DSSP  hhhhhhhhhhhhhhllll
Query aaqavqrfvaridratkr  292
ident
Sbjct --------kyrepkdrse  327
DSSP  --------llllhhhlll
```

No 321: Query=mol1A Sbjct=8icjA Z-score=6.5

```
DSSP  ------------------------------------------------------------
Query ------------------------------------------------------------   0
ident
Sbjct etlnggitdmltelanfeknvsqaihkynayrkaasviakyphkiksgaeakklpgvgtk   60
DSSP  lllhhhhhhhhhhhhhhhhhllllhhhhhhhhhhhhhhhhhllllllhhhhhllllllhh


DSSP  -------------------------------------------------llllllllllh
Query -------------------------------------------------aapyssppptnl   11
ident
Sbjct iaekideflatgklrklekirqddtsssinfltrvsgigpsaarkfvdegiktledlrkn  120
DSSP  hhhhhhhhhhllllhhhhhhhhlhhhhhhhhhllllllhhhhhhhhhlllllhhhhhhl


DSSP  hhhhhhhhhhhhllllhhhhhHHHHHHHHHHHHH-LLLLllLLLLEEEEHHHHHHHHhhl
Query rslrdrltqvaerqgvvfgrlqRHVAXIVVAQFAA-TLTDdtGAPLLLVKGGSSLELrrg   70
ident                                                 | |
Sbjct edklnhhqriglkyfgdfekriPREEMLQMQDIVLnEVKKvdSEYIATVCGSFRRGA---  177
DSSP  hhhllhhhhhhhhlhhhhhlleEHHHHHHHHHHHHHhHHHHhlLLLEEEEELLLLLLL---


DSSP  lllllLLLLEEEEEL------------lLHHHHHHHHHHHHhhllllleeEEELLLEELll
Query ipdsrTSKDFATVAR------------rDIELIHEQLADAGetgwegftAIFTAPEEIaa  118
ident       | |                         |||             |
Sbjct ----eSSGDMDVLLThpsftsestkqpkLLHQVVEQLQKVH--------FITDTLSKG--  223
DSSP  ----lEELLEEEEEEellllllllllhHHHHHHHHHHHHLL--------LEEEEEEEL--


DSSP  llllLLLEEEEEEEEEELL--EEEEEEEEEEEEL-LLLLllllleeeeelhhhhllllllee
Query pgxpVKPRRFTAKLSYRG--RAFATVPIEVSSV-EAGNadqfdtltsdalglvgvpaava  175
ident              |            |
Sbjct ----ETKFMGVCQLPSKNdeKEYPHRRIDIRLIpKDQY----------------------  257
DSSP  ----LLEEEEEEELLLLLLlLLLLLEEEEEEEEeHHHH----------------------


DSSP  eeellhHHHHhhhhhhhhhllllllllllllhhhhHHHHHH--------------------
Query vpcxtiPWQIaqklhavtavleepkandrahdlvDLQLLE--------------------  215
ident
Sbjct -----yCGVL--------------------yftgSDIFNKnmrahalekgftineytirp  292
DSSP  -----hHHHH--------------------hhhlLHHHHHhhhhhhhhllllllllllll


DSSP  --hhllhhhhhhhhHHHhhhhhhllllLLLLLllllhhhhhhhhhlllllllllllhh
Query --gllldadlxptrSACIaifearaqhpWPPRVatlphwpliyagalegldhlelartvd  273
ident               |
Sbjct lgvtgvageplpvdSEKD-------ifdYIQWK---------------------------  318
DSSP  llllllllllllllLHHH-------hhhHLLLL---------------------------


DSSP  hhhhhhhhhhhhhhhhllll
Query aaaqavqrfvaridratkr  292
ident
Sbjct ----------yrepkdrse  327
DSSP  ----------lllllllll
```

No 322: Query=mol1A Sbjct=3jppA Z-score=6.5

back to top

```
DSSP  ------------------------------------------------------------
Query ------------------------------------------------------------   0
ident
Sbjct tlnggitdmltelanfeknvsqaihkynayrkaasviakyphkiksgaeakklpgvgtki   60
DSSP  lllhhhhhhhhhhhhhhhhhllllhhhhhhhhhhhhhhhhhllllllhhhhhlllllhhh


DSSP  ------------------------------------------------llllllllllhh
Query ------------------------------------------------aapyssppptnlr   12
ident
Sbjct aekideflatgklrklekirqddtsssinfltrvsgigpsaarkfvdegiktledlrkne  120
DSSP  hhhhhhhhhlllhhhhhhhhlhhhhhhhhhllllllhhhhhhhhhlllllhhhhhhllh


DSSP  hhhhhhhhhhhllllhhhhhHHHHHHHHHHHHH-LLLLllLLLLEEEEHHHHHHHHhhll
Query slrdrltqvaerqgvvfgrlqRHVAXIVVAQFAA-TLTDdtGAPLLLVKGGSSLELrrgi   71
```

```
Sbjct dklnhhqriglkyfgdfekriPREEMLQMQDIVLnEVKKvdSEYIATVCGSFRRGA----  176
DSSP  hhllhhhhhhhhlhhhhhlleEHHHHHHHHHHHHhHHHHhlLLLEEEELHHHHLLL----


DSSP  llllLLLLEEEEEL------------lLHHHHHHHHHHHHhhllllleeEEELLLEELlll
Query pdsrTSKDFATVAR------------rDIELIHEQLADAGetgwegftAIFTAPEEIaap  119
ident      | |                         |||             |
Sbjct ---eSSGDMDVLLThpsftsestkqpkLLHQVVEQLQKVH--------FITDTLSKG---  222
DSSP  ---lEELLEEEEEEllllllllllllHHHHHHHHHHHLL--------LEEEEEEEL---


DSSP  lllLLLEEEEEEEEEL--LEEEEEEEEEEEEL-LLLLlllleeeeelhhhhllllllleee
Query gxpVKPRRFTAKLSYR--GRAFATVPIEVSSV-EAGNadqfdtltsdalglvgvpaavav  176
ident             |             |
Sbjct ---ETKFMGVCQLPSKndEKEYPHRRIDIRLIpKDQY-----------------------  256
DSSP  ---LLEEEEEEELLLLLllLLLLLLLEEEEEEEELlHHHH-----------------------


DSSP  eellhHHHHhhhhhhhhllllllllllllHHHHHHH-------------------hhhhh
Query pcxtiPWQIaqklhavtavleepkandraHDLVDLQ-------------------llegl  217
ident                               |
Sbjct ----yCGVL----------------yftgSDIFNKNmrahalekgftineytirplgvtg  296
DSSP  ----hHHHH----------------hhhlLHHHHHHhhhhhhhlleeellllleeellll


DSSP  llhHHHHhhHHHHHhhhhhhllllLLLLLlllllhhhhhhhhhlllllllllllhhhhhh
Query lldADLXptRSACIaifearaqhpWPPRVatlphwpliyagalegldhlelartvdaaaq  277
ident      |
Sbjct vagEPLP--VDSEK-------difDYIQW-------------------------------  316
DSSP  lllLLLL--LLLHH-------hhhHHLLL-------------------------------


DSSP  hhhhhhhhhhhllll
Query avqrfvaridratkr  292
ident
Sbjct -----kyrepkdrse  326
DSSP  -----llllhhhlll
```

No 323: Query=mol1A Sbjct=4kldA Z-score=6.5

back to top

```
DSSP  ------------------------------------------------------------
Query ------------------------------------------------------------    0
ident
Sbjct tlnggitdmltelanfeknvsqaihkynayrkaasviakyphkiksgaeakklpgvgtki   60
DSSP  lllhhhhhhhhhhhhhhhhhllllhhhhhhhhhhhhhhhlllllllllhhhhhllllllhhh


DSSP  -----------------------------------------------llllllllllhh
Query -----------------------------------------------aapysspptnlr   12
ident
Sbjct aekideflatgklrklekirqddtsssinfltrvsgigpsaarkfvdegiktledlrkne  120
DSSP  hhhhhhhhhhlllhhhhhhhhlhhhhhhhhhlllllllhhhhhhhhhlllllhhhhhllh


DSSP  hhhhhhhhhhhllllhhhhhHHHHHHHHHHHHHH-LLLLllLLLLEEEEHHHHHHHHhhll
Query slrdrltqvaerqgvvfgrlqRHVAXIVVAQFAA-TLTDdtGAPLLLVKGGSSLELrrgi   71
ident                                                | |
Sbjct dklnhhqriglkyfgdfekriPREEMLQMQDIVLnEVKKvdSEYIATVCGSFRRGA----  176
DSSP  hhllhhhhhhhhlhhhhhlleEHHHHHHHHHHHHhHHHHhlLLLEEEELLLLLLL----


DSSP  llllLLLLEEEEEL------------lLHHHHHHHHHHHHhhllllleeEEELLLEELlll
Query pdsrTSKDFATVAR------------rDIELIHEQLADAGetgwegftAIFTAPEEIaap  119
ident      | |                         |||             |
Sbjct ---eSSGDMDVLLThpsftsestkqpkLLHQVVEQLQKVH--------FITDTLSKG---  222
DSSP  ---lEELLEEEEEEllllllllllllHHHHHHHHHHHLL--------LEEEEEEEL---


DSSP  lllLLLEEEEEEEEEL--LEEEEEEEEEEEEL-LLLLlllleeeeelhhhhllllllleee
Query gxpVKPRRFTAKLSYR--GRAFATVPIEVSSV-EAGNadqfdtltsdalglvgvpaavav  176
ident             |             |
Sbjct ---ETKFMGVCQLPSKndEKEYPHRRIDIRLIpKDQY-----------------------  256
DSSP  ---LLEEEEEEELLLLLllLLLLLLLEEEEEEEELlHHHH-----------------------
```

```
DSSP  eellhHHHHhhhhhhhhllllllllllllHHHHHHH-------------------hhhhh
Query pcxtiPWQIaqklhavtavleepkandraHDLVDLQ-------------------llegl  217
ident                               |
Sbjct ----yCGVL----------------yftgSDIFNKNmrahalekgftineytirplgvtg  296
DSSP  ----hHHHH----------------hhhlLHHHHHHhhhhhhhhleeellllleeellll


DSSP  llhHHHHhhHHHHHhhhhhhllllLLLLLlllllhhhhhhhhhlllllllllllhhhhhh
Query lldADLXptRSACIaifearaqhpWPPRVatlphwpliyagalegldhlelartvdaaaq  277
ident      |
Sbjct vagEPLP--VDSEK-------difDYIQW-------------------------------  316
DSSP  lllLLLL--LLLHH-------hhhHHLLL-------------------------------


DSSP  hhhhhhhhhhllll
Query avqrfvaridratkr  292
ident
Sbjct -----kyrepkdrse  326
DSSP  -----llllhhhlll
```

No 324: Query=mol1A Sbjct=4rq6A Z-score=6.5

back to top

```
DSSP  ------------------------------------------------------------
Query ------------------------------------------------------------    0
ident
Sbjct tlnggitdmltelanfeknvsqaihkynayrkaasviakyphkiksgaeakklpgvgtki   60
DSSP  lllhhhhhhhhhhhhhhhhllllhhhhhhhhhhhhhhhhhllllllhhhhhllllllhhh


DSSP  ------------------------------------------------llllllllllhh
Query ------------------------------------------------aapysspptnlr   12
ident
Sbjct aekideflatgklrklekirqddtsssinfltrvsgigpsaarkfvdegiktledlrkne  120
DSSP  hhhhhhhhhhlllhhhhhhhhlhhhhhhhhhhllllllhhhhhhhhhhllllllhhhhhllh


DSSP  hhhhhhhhhhhhllllhhhhhHHHHHHHHHHHHHH-LLLLllLLLLEEEEHHHHHHHhhll
Query slrdrltqvaerqgvvfgrlqRHVAXIVVAQFAA-TLTDdtGAPLLLVKGGSSLELrrgi   71
ident                                                |  |
Sbjct dklnhhqriglkyfgdfekriPREEMLQMQDIVLnEVKKvdSEYIATVCGSFRRGA----  176
DSSP  hhllhhhhhhhhlhhhhllleEHHHHHHHHHHHHhHHHHhlLLLEEEELHHHHLLL----


DSSP  llllLLLLEEEEEL------------lLHHHHHHHHHHHHhhlllleeEEELLLEEllll
Query pdsrTSKDFATVAR------------rDIELIHEQLADAGetgwegftAIFTAPEEiaap  119
ident      | |                        |||              |
Sbjct ---eSSGDMDVLLThpsftsestkqpkLLHQVVEQLQKVH--------FITDTLSK----  221
DSSP  ---lEELLEEEEEElllllllllllllHHHHHHHHHHHLL--------LEEEEEEE----


DSSP  lllLLLEEEEEEEEEL--LEEEEEEEEEEEEL-LLLLlllleeeeelhhhhllllllleee
Query gxpVKPRRFTAKLSYR--GRAFATVPIEVSSV-EAGNadqfdtltsdalglvgvpaavav  176
ident             |             |
Sbjct --gETKFMGVCQLPSKndEKEYPHRRIDIRLIpKDQY-----------------------  256
DSSP  --lLLEEEEEELLLLLllLLLLLLEEEEEEELlHHHH-----------------------


DSSP  eellhHHHHhhhhhhhhllllllllllllHHHH-------------------hhhhhhhh
Query pcxtiPWQIaqklhavtavleepkandraHDLV-------------------dlqllegl  217
ident                               |
Sbjct ----yCGVL----------------yftgSDIFnknmrahalekgftineytirplgvtg  296
DSSP  ----hHHHH----------------hhhlLHHHhhhhhhhhhhhleeelllleeelllll


DSSP  llhHHHHhhHHHHhhhhhhlllLLLLLLlllllhhhhhhhhhlllllllllllhhhhhh
Query lldADLXPtrSACIaifearaqhPWPPRVatlphwpliyagalegldhlelartvdaaaq  277
ident      |
Sbjct vagEPLPV--DSEK-----difdYIQWKY-------------------------------  318
DSSP  lllLLLLL--LLHH-----hhhhHLLLLL-------------------------------


DSSP  hhhhhhhhhhllll
Query avqrfvaridratkr  292
ident
```

```
DSSP  -------llhhhhll
```

No 325: Query=mol1A Sbjct=1xslM Z-score=6.5

back to top

```
DSSP  ------------------------------------------------------------
Query ------------------------------------------------------------    0
ident
Sbjct atnhnlhiteklevlakaysvqgdkwralgyakainalksfhkpvtsyqeacsipgigkr   60
DSSP  lllllhhhhhhhhhhhhhhhhlllhhhhhhhhhhhhhhhhlllllllhhhhhhlllllhh


DSSP  ----------------------------------------------llllllllllhhhh
Query ----------------------------------------------aapysspptnlrsl   14
ident
Sbjct maekiieilesghlrkldhisesvpvlelfsniwgagtktaqmwyqqgfrsledirsqas  120
DSSP  hhhhhhhhhhhlllhhhhlllllhhhhhhhhllllllhhhhhhhhhlllllhhhhhhhll


DSSP  hhhhhhhhhhllllhHHHHHHHHHHHHHHHHHLL-LLLLLLLEEEEHHHHHHHHhhllll
Query rdrltqvaerqgvvfGRLQRHVAXIVVAQFAATL-TDDTGAPLLLVKGGSSLELrrgipd   73
ident                             |             |    |
Sbjct lttqqaiglkhysdfLERMPREEATEIEQTVQKAaQAFNSGLLCVACGSYRRGK------  174
DSSP  llhhhhhhhhlhhhhHLLEEHHHHHHHHHHHHHHhHLLLLLLEEEELHHHHLLL------


DSSP  llLLLLEEEEEL--------lLHHHHHHHHHHHHhhlllleeeEELLL-EELLLlLLLLL
Query srTSKDFATVAR--------rDIELIHEQLADAGetgwegftaIFTAP-EEIAApGXPVK  124
ident   |  |                       |   |           |
Sbjct -aTCGDVDVLIThpdgrshrgIFSRLLDSLRQEG---------FLTDDlVSQEE-NGQQQ  223
DSSP  -lEELLEEEEEElllllllllLHHHHHHHHHHLL---------LEEEEeEEEEE-ELLEE


DSSP  LEEEEEEEEEllEEEEEEEEEEEEL-LLLLlllleeeeelhhhhlllllleeeeellhHH
Query PRRFTAKLSYrgRAFATVPIEVSSV-EAGNadqfdtltsdalglvgvpaavavpcxtiPW  183
ident        |                |
Sbjct KYLGVCRLPG--PGRRHRRLDIIVVpYSEF---------------------------aCA  254
DSSP  EEEEEELLLL--LLLLLEEEEEEELlHHHH---------------------------hHH


DSSP  HHhhhhhhhlllllllllllllHHHHHHH-------------------------hhhhhl
Query QIaqklhavtavleepkandraHDLVDLQ-------------------------llegll  218
ident
Sbjct LL----------------yftgSAHFNRSmralaktkgmslsehalstavvrnthgckvg  298
DSSP  HH----------------hhhlLHHHHHHhhhhhhhlleeellllleeelleellllleee


DSSP  lhHHHHhhHHHHHhhhhhhllllLLLLLllllhhhhhhhhhllllllllllhhhhhhh
Query ldADLXptRSACIaifearaqhpWPPRVatlphwpliyagalegldhlelartvdaaaqa  278
ident     |
Sbjct pgRVLP--TPTEK-------dvfRLLGL--------------------------------  317
DSSP  llLEEL--LLLHH-------hhhHHLLL--------------------------------


DSSP  hhhhhhhhhhllll
Query vqrfvaridratkr  292
ident
Sbjct ----pyrepaerdw  327
DSSP  ----llllhhhlll
```

No 326: Query=mol1A Sbjct=2bcrA Z-score=6.5

back to top

```
DSSP  ------------------------------------------------------------
Query ------------------------------------------------------------    0
ident
Sbjct nhnlhiteklevlakaysvqgdkwralgyakainalksfhkpvtsyqeacsipgigkrma   60
DSSP  lllhhhhhhhhhhhhhhhhlllhhhhhhhhhhhhhhhlllllllllhhhhhlllllhhhh


DSSP  --------------------------------------------llllllllllhhhhhh
```

```
ident
Sbjct ekiieilesghlrkldhisesvpvlelfsniwgagtktaqmwyqqgfrsledirsqaslt  120
DSSP  hhhhhhhlllllhhhhlllllhhhhhhhhhlllllhhhhhhhhhlllllhhhhhhhllll


DSSP  hhhhhhhhllllhhhhHHHHHHHHHHHHHHHL-LLLLLLLLLEEEEHHHHHHHHhhllllll
Query rltqvaerqgvvfgrlQRHVAXIVVAQFAAT-LTDDTGAPLLLVKGGSSLELrrgipdsr   75
ident                           |             |    |
Sbjct tqqaiglkhysdflerMPREEATEIEQTVQKaAQAFNSGLLCVACGSYRRGK-------a  173
DSSP  hhhhhhhhlllhhhllEEHHHHHHHHHHHHHHhHHHHLLLLEEEELHHHHLLL-------l


DSSP  LLLLEEEEEL--------lLHHHHHHHHHHHHhhlllleeeEELLLEELLllllLLLLEE
Query TSKDFATVAR--------rDIELIHEQLADAGetgwegftaIFTAPEEIAapgxPVKPRR  127
ident |  |                       |   |           |
Sbjct TCGDVDVLIThpdgrshrgIFSRLLDSLRQEG---------FLTDDLVSQeengQQQKYL  224
DSSP  EELLEEEEEEllllllllLHHHHHHHHHHHLL---------LEEEEEEELLllllLLLEEE


DSSP  EEEEEEEllEEEEEEEEEEEEL-LLLLllleeeeelhhhhllllllleeeeellhHHHHh
Query FTAKLSYrgRAFATVPIEVSSV-EAGNadqfdtltsdalglvgvpaavavpcxtiPWQIa  186
ident     |                |
Sbjct GVCRLPG--PGRRHRRLDIIVVpYSEF---------------------------aCALL-  254
DSSP  EEELLLL--LLLLLEEEEEEEELlHHHH---------------------------hHHHH-


DSSP  hhhhhhhllllllllllHHHHHHH-------------------------hhhhhllhH
Query qklhavtavleepkandraHDLVDLQ-------------------------llegllldA  221
ident
Sbjct ---------------yftgSAHFNRSmralaktkgmslsehalstavvrnthgckvgpgR  299
DSSP  ---------------hhhlLHHHHHHhhhhhhhllleellllleeelleellllleeellE


DSSP  HHHhhHHHHHhhhhhhllllLLLLLlllllhhhhhhhhhllllllllllhhhhhhhhhh
Query DLXptRSACIaifearaqhpWPPRVatlphwpliyagalegldhlelartvdaaaqavqr  281
ident  |
Sbjct VLP--TPTEK-------dvfRLLGL-----------------------------------  315
DSSP  ELL--LLLHH-------hhhHHLLL-----------------------------------


DSSP  hhhhhhhllll
Query fvaridratkr  292
ident
Sbjct -pyrepaerdw  325
DSSP  -llllhhhhll
```

No 327: Query=mol1A Sbjct=2pfnA Z-score=6.5

back to top

```
DSSP  ------------------------------------------------------------
Query ------------------------------------------------------------    0
ident
Sbjct nhnlhiteklevlakaysvqgdkwralgyakainalksfhkpvtsyqeacsipgigkrma   60
DSSP  lllhhhhhhhhhhhhhhhhlllhhhhhhhhhhhhhhhllllllllhhhhhlllllllhhhh


DSSP  --------------------------------------------llllllllllhhhhhh
Query --------------------------------------------aapysspptnlrslrd   16
ident
Sbjct ekiieilesghlrkldhisesvpvlelfsniwgagtktaqmwyqqgfrsledirsqaslt  120
DSSP  hhhhhhhlllllhhhhhlllhhhhhhhhhlllllhhhhhhhhhlllllhhhhhhhhllll


DSSP  hhhhhhhhllllhhhhHHHHHHHHHHHHHHHL-LLLLlLLLLEEEEHHHHHHHHhhllllll
Query rltqvaerqgvvfgrlQRHVAXIVVAQFAAT-LTDDtGAPLLLVKGGSSLELrrgipdsr   75
ident                           |             |    |
Sbjct tqqaiglkhysdflerMPREEATEIEQTVQKaAQAFnSGLLCVACGSYRRGK-------a  173
DSSP  hhhhhhhhlhhhhhllEEHHHHHHHHHHHHHHhHHHHlLLLEEEELHHHHLLL-------l


DSSP  LLLLEEEEEL--------lLHHHHHHHHHHHHhhlllleeeEELLLEELlllllLLLLEE
Query TSKDFATVAR--------rDIELIHEQLADAGetgwegftaIFTAPEEIaapgxPVKPRR  127
ident |  |                       |   |           |
Sbjct TCGDVDVLIThpdgrshrgIFSRLLDSLRQEG---------FLTDDLVSqeengQQQKYL  224
DSSP  EELLEEEEEEllllllllLHHHHHHHHHHHLL---------LEEEEEEELlllllLLLEEE
```

```
DSSP  EEEEEEEllEEEEEEEEEEEEL-LLLLlllleeeeelhhhhlllllleeeeellhHHHHh
Query FTAKLSYrgRAFATVPIEVSSV-EAGNadqfdtltsdalglvgvpaavavpcxtiPWQIa  186
ident     |                |
Sbjct GVCRLPG--PGRRHRRLDIIVVpYSEF---------------------------aCALL-  254
DSSP  EEELLLL--LLLLLEEEEEEELlHHHH---------------------------hHHHH-


DSSP  hhhhhhhllllllllllllHHHHHHH-------------------------hhhhhllhH
Query qklhavtavleepkandraHDLVDLQ-------------------------llegllldA  221
ident
Sbjct ---------------yftgSAHFNRSmralaktkgmslsehalstavvrnthgakvgpgR  299
DSSP  ---------------hhhlLHHHHHHhhhhhhhlleeellllleeellllllllllllllE


DSSP  HHHhhHHHHHhhhhhhllllLLLLLlllllhhhhhhhhhllllllllllhhhhhhhhhhh
Query DLXptRSACIaifearaqhpWPPRVatlphwpliyagalegldhlelartvdaaaqavqr  281
ident  |
Sbjct VLP--TPTEK-------dvfRLLGL-----------------------------------  315
DSSP  EEL--LLLHH-------hhhHHLLL-----------------------------------


DSSP  hhhhhhhllll
Query fvaridratkr  292
ident
Sbjct -pyrepaerdw  325
DSSP  -llllhhhhll
```

No 328: Query=mol1A Sbjct=1rztA Z-score=6.5

back to top

```
DSSP  ------------------------------------------------------------
Query ------------------------------------------------------------    0
ident
Sbjct atnhnlhiteklevlakaysvqgdkwralgyakainalksfhkpvtsyqeacsipgigkr   60
DSSP  lllllhhhhhhhhhhhhhhhhhlllhhhhhhhhhhhhhhhlllllllhhhhhhlllllhh


DSSP  ---------------------------------------------lllllllllllhhhh
Query ---------------------------------------------aapyssspptnlrsl   14
ident
Sbjct maekiieilesghlrkldhisesvpvlelfsniwgagtktaqmwyqqgfrsledirsqas  120
DSSP  hhhhhhhhhhhlllhhhhhhllllhhhhhhhhhlllllhhhhhhhhhlllllhhhhhhll


DSSP  hhhhhhhhhhllllhHHHHHHHHHHHHHHHHHLL-LLLLLLLLEEEEHHHHHHHhhllll
Query rdrltqvaerqgvvfGRLQRHVAXIVVAQFAATL-TDDTGAPLLLVKGGSSLELrrgipd   73
ident                             |             |    |
Sbjct lttqqaiglkhysdfLERMPREEATEIEQTVQKAaQAFNSGLLCVACGSYRRGK------  174
DSSP  llhhhhhhhhlhhhhHLLEEHHHHHHHHHHHHHHhHLLLLLLEEEELHHHHLLL------


DSSP  llLLLLEEEEEL--------lLHHHHHHHHHHHHhhlllleeeEELLL-EELLLlLLLLL
Query srTSKDFATVAR--------rDIELIHEQLADAGetgwegftaIFTAP-EEIAApGXPVK  124
ident   |  |                       |   |           |
Sbjct -aTCGDVDVLIThpdgrshrgIFSRLLDSLRQEG---------FLTDDlVSQEE-NGQQQ  223
DSSP  -lEELLEEEEEElllllllllLHHHHHHHHHHLL---------LEEEEeEEEEE-ELLEE


DSSP  LEEEEEEEEEllEEEEEEEEEEEEL-LLLLlllleeeeelhhhhlllllleeeeellhHH
Query PRRFTAKLSYrgRAFATVPIEVSSV-EAGNadqfdtltsdalglvgvpaavavpcxtiPW  183
ident        |                |
Sbjct KYLGVCRLPG--PGRRHRRLDIIVVpYSEF---------------------------aCA  254
DSSP  EEEEEELLLL--LLLLLEEEEEEELlHHHH---------------------------hHH


DSSP  HHhhhhhhhhllllllllllllHHHHHHH-----------------------hhhhhllh
Query QIaqklhavtavleepkandraHDLVDLQ-----------------------llegllld  220
ident
Sbjct LL----------------yftgSAHFNRSmralaktkgmslsehalstavvrnthgckvg  298
DSSP  HH----------------hhhlLHHHHHHhhhhhhhlleeellllleeelleelllllleee


DSSP  hhhhhhHHHHHhhhhhhllllLLLLLlllllhhhhhhhhhllllllllllhhhhhhhhhh
Query adlxptRSACIaifearaqhpWPPRVatlphwpliyagalegldhlelartvdaaaqavq  280
```

```
Sbjct pgrvipIPIEK-------dvIRLLGL----------------------------------  317
DSSP  llllllLLLHH-------hhhHHLLL----------------------------------


DSSP  hhhhhhhhllll
Query rfvaridratkr  292
ident
Sbjct --pyrepaerdw  327
DSSP  --llllhhhlll
```

No 329: Query=mol1A Sbjct=7icoA Z-score=6.5

back to top

```
DSSP  ------------------------------------------------------------
Query ------------------------------------------------------------    0
ident
Sbjct etlnggitdmltelanfeknvsqaihkynayrkaasviakyphkiksgaeakklpgvgtk   60
DSSP  lllllhhhhhhhhhhhhhhhllllhhhhhhhhhhhhhhhhllllllhhhhhlllllllhh


DSSP  -------------------------------------------------llllllllllh
Query -------------------------------------------------aapysspptnl   11
ident
Sbjct iaekideflatgklrklekirqddtsssinfltrvsgigpsaarkfvdegiktledlrkn  120
DSSP  hhhhhhhhhhllllhhhhhhhhlhhhhhhhhhllllllhhhhhhhhllllllhhhhhhl


DSSP  hhhhhhhhhhhhhllllhhhhhHHHHHHHHHHHHH-LLLLllLLLLEEEEHHHHHHHhhl
Query rslrdrltqvaerqggvvfgrlqRHVAXIVVAQFAA-TLTDdtGAPLLLVKGGSSLELrrg   70
ident                                                 | |
Sbjct edklnhhqriglkyfgdfekriPREEMLQMQDIVLnEVKKvdSEYIATVCGSFRRGA---  177
DSSP  lllllhhhhhhhhlllhhhlleEHHHHHHHHHHHHhHHHHhlLLLLLLLLLHHHHLL---


DSSP  lllllLLLLEEEEEL------------lLHHHHHHHHHHHHhhlllleeEEELLLEELll
Query ipdsrTSKDFATVAR------------rDIELIHEQLADAGetgwegftAIFTAPEEIaa  118
ident       | |                         |||             |
Sbjct ----eSSGDMDVLLThpsftsestkqpkLLHQVVEQLQKVH--------FITDTLSKG--  223
DSSP  ----lEELLEEEEEEelllllllllllhHHHHHHHHHHHLL--------LEEEEEEEL--


DSSP  llllLLLEEEEEEEEEELL--EEEEEEEEEEEEL-LLLLlllleeeeelhhhhlllllee
Query pgxpVKPRRFTAKLSYRG--RAFATVPIEVSSV-EAGNadqfdtltsdalglvgvpaava  175
ident              |             |
Sbjct ----ETKFMGVCQLPSKNdeKEYPHRRIDIRLIpKDQY----------------------  257
DSSP  ----LLEEEEEEELLLLLllLLLLLEEEEEEEEeHHHL----------------------


DSSP  eeellhHHHHHHhhhhhhllllllllllllhhhhHHHHHH--------------------
Query vpcxtiPWQIAQklhavtavleepkandrahdlvDLQLLE--------------------  215
ident
Sbjct -----yCGVLYF--------------------tgSDIFNKnmrahalekgftineytirp  292
DSSP  -----hHHHHHH--------------------hlLHHHHHhhhhhhhhlleeeelleeee


DSSP  -hhllhhhhhhhHHHHHhhhhhhllllLLLLLLllllhhhhhhhhhllllllllllllhhh
Query -glllldadlxptRSACIaifearaqhpWPPRVAtlphwpliyagalegldhlelartvda  274
ident
Sbjct lgvtgvageplpVDSEK-------difDYIQWK---------------------------  318
DSSP  llllllllllllLLLHH-------hhhHHLLLL---------------------------


DSSP  hhhhhhhhhhhhhhllll
Query aaqavqrfvaridratkr  292
ident
Sbjct ---------yrepkdrse  327
DSSP  ---------lllllllll
```

No 330: Query=mol1A Sbjct=3hw8A Z-score=6.5

back to top

```
Query ------------------------------------------------------------    0
ident
Sbjct atnhnlhiteklevlakaysvqgdkwralgyakainalksfhkpvtsyqeacsipgigkr   60
DSSP  lllllhhhhhhhhhhhhhhhhlllhhhhhhhhhhhhhhhhlllllllhhhhhlllllllhh


DSSP  ----------------------------------------------lllllllllhhhh
Query ----------------------------------------------aapyssspptnlrsl   14
ident
Sbjct maekiieilesghlrkldhisesvpvlelfsniwgagtktaqmwyqqgfrsledirsqas  120
DSSP  hhhhhhhhhhllllhhhhllllllhhhhhhhhllllllhhhhhhhhhlllllhhhhhhhll


DSSP  hhhhhhhhhhllllhhHHHHHHHHHHHHHHHHHLL-LLLLLLLLEEEEHHHHHHHhhllll
Query rdrltqvaerqgvvfgRLQRHVAXIVVAQFAATL-TDDTGAPLLLVKGGSSLELrrgipd   73
ident                             |             |    |
Sbjct lttqqaiglkhysdflERMPREEATEIEQTVQKAaQAFNSGLLCVACGSYRRGK------  174
DSSP  llhhhhhhhhlhhhhlLLEEHHHHHHHHHHHHHHHHhHHLLLLLLEEEELHHHHLLL------


DSSP  llLLLLLEEEEEL--------lLHHHHHHHHHHHHHhhllllleeeEELLLEELLllllLLLL
Query srTSKDFATVAR--------rDIELIHEQLADAGetgwegftaIFTAPEEIAapgxPVKP  125
ident   |  |                       |   |           |
Sbjct -aTCGDVDVLIThpdgrshrgIFSRLLDSLRQEG---------FLTDDLVSQeengQQQK  224
DSSP  -lEELLEEEEEElllllllllLHHHHHHHHHHHLL---------LEEEEEELLllllLLLE


DSSP  EEEEEEEEEllEEEEEEEEEEEEL-LLLLllllleeeeelhhhhlllllllleeeeellhHHH
Query RRFTAKLSYrgRAFATVPIEVSSV-EAGNadqfdtltsdalglvgvpaavavpcxtiPWQ  184
ident       |                |
Sbjct YLGVCRLPG--PGRRHRRLDIIVVpYSEF---------------------------aCAL  255
DSSP  EEEEELLLL--LLLLLEEEEEEEELlHHHH---------------------------hHHH


DSSP  HhhhhhhhhllllllllllllHHHHHHH-------------------------hhhhhll
Query IaqklhavtavleepkandraHDLVDLQ-------------------------lleglll  219
ident
Sbjct L----------------yftgSAHFNRSmralaktkgmslsehalstavvrnthgakvgp  299
DSSP  H----------------hhhlLHHHHHHhhhhhhhlleeellllleeellllllllllll


DSSP  hHHHHhhHHHHHhhhhhhllllLLLLLllllhhhhhhhhhllllllllllhhhhhhhh
Query dADLXptRSACIaifearaqhpWPPRVatlphwpliyagalegldhlelartvdaaaqav  279
ident    |
Sbjct gRVLP--TPTEK-------dvfRLLGL----------------------------------  317
DSSP  lEELL--LLLHH-------hhhHHLLL----------------------------------


DSSP  hhhhhhhhhllll
Query qrfvaridratkr  292
ident
Sbjct ---pyrepaerdw  327
DSSP  ---llllhhhhll
```

No 331: Query=mol1A Sbjct=4uazA Z-score=6.5

back to top

```
DSSP  ------------------------------------------------------------
Query ------------------------------------------------------------    0
ident
Sbjct tlnggitdmltelanfeknvsqaihkynayrkaasviakyphkiksgaeakklpgvgtki   60
DSSP  lllhhhhhhhhhhhhhhhhhllllhhhhhhhhhhhhhhhhlllllllhhhhhlllllhhh


DSSP  ------------------------------------------------lllllllllhh
Query ------------------------------------------------aapyssspptnlr   12
ident
Sbjct aekideflatgklrklekirqddtsssinfltrvsgigpsaarkfvdegiktledlrkne  120
DSSP  hhhhhhhhhhlllhhhhhhhhlhhhhhhhhhllllllhhhhhhhhhlllllhhhhhhlh


DSSP  hhhhhhhhhhhhllllhhhhhHHHHHHHHHHHHHH-LLLLllLLLLEEEEHHHHHHHhhll
Query slrdrltqvaerqgvvfgrlqRHVAXIVVAQFAA-TLTDdtGAPLLLVKGGSSLELrrgi   71
ident                                                |ˌ|
Sbjct dklnhhqriglkyfgdfekriPREEMLQMQDIVLnEVKKvdSEYIATVCGSFRRGA----  176
```

```
DSSP  llllLLLLEEEEEL------------lLHHHHHHHHHHHHhhlllleeEEELLLEEllll
Query pdsrTSKDFATVAR------------rDIELIHEQLADAGetgwegftAIFTAPEEiaap  119
ident      | |                         |||             |
Sbjct ---eSSGDMDVLLThpsftsestkqpkLLHQVVEQLQKVH--------FITDTLSK----  221
DSSP  ---lEELLEEEEEElllllllllllllHHHHHHHHHHHLL--------LEEEEEEE----


DSSP  lllLLLEEEEEEEEEL--LEEEEEEEEEEEEL-LLLLlllleeeeelhhhhlllllleee
Query gxpVKPRRFTAKLSYR--GRAFATVPIEVSSV-EAGNadqfdtltsdalglvgvpaavav  176
ident             |             |
Sbjct --gETKFMGVCQLPSKndEKEYPHRRIDIRLIpKDQY-----------------------  256
DSSP  --lLLEEEEEEELLLLllLLLLLLEEEEEEELlHHHH-----------------------


DSSP  eellhHHHHhhhhhhhhllllllllllllHHHH------------------hhhhhhhhl
Query pcxtiPWQIaqklhavtavleepkandraHDLV------------------dlqllegll  218
ident                               |
Sbjct ----yCGVL----------------yftgSDIFnknmrahalekgftineytirplgtgv  296
DSSP  ----hHHHH----------------hhhlLHHHhhhhhhhhhhlleeellllleeelllll


DSSP  lhHHHHHhhHHHHhhhhhhlllLLLLLLlllllhhhhhhhhhlllllllllllhhhhhhh
Query ldADLXPtrSACIaifearaqhPWPPRVatlphwpliyagalegldhlelartvdaaaqa  278
ident     |
Sbjct agEPLPV--DSEK-----difdYIQWKY--------------------------------  317
DSSP  llLLLLL--LLHH-----hhhhHLLLLL--------------------------------


DSSP  hhhhhhhhhhllll
Query vqrfvaridratkr  292
ident
Sbjct ------repkdrse  325
DSSP  ------llhhhlll
```

No 332: Query=mol1A Sbjct=4rq3A Z-score=6.5

back to top

```
DSSP  ------------------------------------------------------------
Query ------------------------------------------------------------    0
ident
Sbjct tlnggitdmltelanfeknvsqaihkynayrkaasviakyphkiksgaeakklpgvgtki   60
DSSP  lllhhhhhhhhhhhhhhhhllllhhhhhhhhhhhhhhhhllllllhhhhhlllllllhhh


DSSP  ------------------------------------------------llllllllllhh
Query ------------------------------------------------aapysspptnlr   12
ident
Sbjct aekideflatgklrklekirqddtsssinfltrvsgigpsaarkfvdegiktledlrkne  120
DSSP  hhhhhhhhhhlllhhhhhhhhllhhhhhhhhhllllllhhhhhhhhhlllllhhhhhllh


DSSP  hhhhhhhhhhhhllllhhhhhHHHHHHHHHHHHH-LLLLllLLLLEEEEHHHHHHHhhll
Query slrdrltqvaerqgvvfgrlqRHVAXIVVAQFAA-TLTDdtGAPLLLVKGGSSLELrrgi   71
ident                                                |  |
Sbjct dklnhhqriglkyfgdfekriPREEMLQMQDIVLnEVKKvdSEYIATVCGSFRRGA----  176
DSSP  hhllhhhhhhhhlhhhhhlleEHHHHHHHHHHHHhHHHHhlLLLEEEELHHHHLLL----


DSSP  llllLLLLEEEEEL------------lLHHHHHHHHHHHHhhlllleeEEELLLEEllll
Query pdsrTSKDFATVAR------------rDIELIHEQLADAGetgwegftAIFTAPEEiaap  119
ident      | |                         |||             |
Sbjct ---eSSGDMDVLLThpsftsestkqpkLLHQVVEQLQKVH--------FITDTLSK----  221
DSSP  ---lEELLEEEEEElllleelleellllHHHHHHHHHHHLL--------LEEEEEEE----


DSSP  lllLLLEEEEEEEEEL--lEEEEEEEEEEEEL-LLLLlllleeeeelhhhhlllllleee
Query gxpVKPRRFTAKLSYR--gRAFATVPIEVSSV-EAGNadqfdtltsdalglvgvpaavav  176
ident             |             |
Sbjct --gETKFMGVCQLPSKndeKEYPHRRIDIRLIpKDQY-----------------------  256
DSSP  --lLLEEEEEEELLLLlllLLLLLEEEEEEELlHHHH-----------------------


DSSP  eellhHHHHhhhhhhhhllllllllllllHHHH-------------------hhhhhhhh
```

```
ident                               |
Sbjct ----yCGVL----------------yftgSDIFnknmrahalekgftineytirplgvtg  296
DSSP  ----hHHHH----------------hhhlLHHHhhhhhhhhhhlleeellllleeellllll


DSSP  llhHHHHHhhHHHHhhhhhhlllLLLLLLllllhhhhhhhhhllllllllllhhhhhh
Query lldADLXPtrSACIaifearaqhPWPPRVatlphwpliyagalegldhlelartvdaaaq  277
ident      |
Sbjct vagEPLPV--DSEK-----difdYIQWKY------------------------------  318
DSSP  lllLLLLL--LLHH-----hhhhHLLLLL------------------------------


DSSP  hhhhhhhhhhllll
Query avqrfvaridratkr  292
ident
Sbjct -------repkdrse  326
DSSP  -------llhhhhll
```

No 333: Query=mol1A Sbjct=5cr0B Z-score=6.5

back to top

```
DSSP  ----------------------llllllllllhhhhhhhhhhhhhhllllhHHHHHHHHH
Query ----------------------aapysspptnlrslrdrltqvaerqgvvfGRLQRHVAX   38
ident
Sbjct pvlelfsniwgagtktaqmwyqqgfrsledirsqaslttqqaiglkhysdfLERMPREEA   60
DSSP  llhhhhhllllllhhhhhhhhhlllllhhhhhhlllllhhhhhhhhlhhhhLLLEEHHHH


DSSP  HHHHHHHHLL-LLLLLLLLEEEEHHHHHHHhhllllllLLLLEEEEEL--------lLHH
Query IVVAQFAATL-TDDTGAPLLLVKGGSSLELrrgipdsrTSKDFATVAR--------rDIE   89
ident     |             |    |              |  |
Sbjct TEIEQTVQKAaQAFNSGLLCVACGSYRRGK-------aTCGDVDVAIThpdgrshrgIFS  113
DSSP  HHHHHHHHHHhHHHLLLLEEEELLLLLLL-------lEELLEEEEEEllllllllHHH


DSSP  HHHHHHHHHHhhllllleeEEELLLE-ELLLllLLLLLEEEEEEEEEllEEEEEEEEEEE
Query LIHEQLADAGetgwegftAIFTAPE-EIAApgXPVKPRRFTAKLSYrgRAFATVPIEVSS  148
ident      |   |                                 |
Sbjct RLLDSLRQEG--------FLTDDLVsQEEN--GQQQKYLGVCRLPG--PGRRHRRLDIIV  161
DSSP  HHHHHHHHLL--------LEEEEEEeEEEE--LLEEEEEEEELLLL--LLLLLEEEEEEE


DSSP  L-LLLLllllleeeeelhhhhllllllleeeeellhHHHHhhhhhhhhlllllllllllHH
Query V-EAGNadqfdtltsdalglvgvpaavavpcxtiPWQIaqklhavtavleepkandraHD  207
ident |
Sbjct VpYSEF---------------------------aCALL----------------yftgSA  178
DSSP  LlHHHH---------------------------hHHHH----------------hhhlLH


DSSP  HHHHH-------------------------hhhhhllhHHHHhhHHHHHhhhhhhllllL
Query LVDLQ-------------------------lleglllldADLXptRSACIaifearaqhpW  242
ident                                         |
Sbjct HFNRSmralaktkgmslsehalstavvrnthgckvgpgRVLP--TPTEK-------dvfR  229
DSSP  HHHHHhhhhhhllleeellllleeeleeellllleeeelLLLL--LLLHH-------hhhH


DSSP  LLLLlllllhhhhhhhhhlllllllllllhhhhhhhhhhhhhhhhllll
Query PPRVatlphwpliyagalegldhlelartvdaaaqavqrfvaridratkr  292
ident
Sbjct LLGL------------------------------------pyrepaerdw  243
DSSP  HHLL------------------------------------llllhhhhll
```

No 334: Query=mol1A Sbjct=5iimA Z-score=6.5

back to top

```
DSSP  ------------------------------------------------------------
Query ------------------------------------------------------------    0
ident
Sbjct tnhnlhiteklevlakaysvqgdkwralgyakainalksfhkpvtsyqeacsipgigkrm   60
DSSP  llllhhhhhhhhhhhhhhhhlllhhhhhhhhhhhhhhllllllllhhhhhlllllhhh
```

```
DSSP  ---------------------------------------------llllllllllhhhhh
Query ---------------------------------------------aapysspptnlrslr   15
ident
Sbjct aekiieilesghlrkldhisesvpvlelfsniwgagtktaqmwyqqgfrsledirsqasl  120
DSSP  hhhhhhhhhhlllhhhhhllllhhhhhhhhhlllllhhhhhhhhhlllllhhhhhhhlll


DSSP  hhhhhhhhhllllhhHHHHHHHHHHHHHHHHHLL-LLLLLLLEEEEHHHHHHHhhlllll
Query drltqvaerqgvvfgRLQRHVAXIVVAQFAATL-TDDTGAPLLLVKGGSSLELrrgipds   74
ident                            |             |    |
Sbjct ttqqaiglkhysdflERMPREEATEIEQTVQKAaQAFNSGLLCVACGSYRRGK-------  173
DSSP  lhhhhhhhhlhhhhhLLEEHHHHHHHHHHHHHHHhHHHLLLLEEEELHHHHLLL-------


DSSP  lLLLLEEEEEL--------lLHHHHHHHHHHHHHhhllllleeeEELLLEELLllllLLLLE
Query rTSKDFATVAR--------rDIELIHEQLADAGetgwegftaIFTAPEEIAapgxPVKPR  126
ident  |  |                       |   |           |
Sbjct aTCGDVDVLIThpdgrshrgIFSRLLDSLRQEG---------FLTDDLVSQeengQQQKY  224
DSSP  lEELLEEEEEElllllllllHHHHHHHHHHHHLL---------LEEEEEELLllllLLLEE


DSSP  EEEEEEEEllEEEEEEEEEEEELLLLLLlllleeeeelhhhhllllleeeeellhHHHHh
Query RFTAKLSYrgRAFATVPIEVSSVEAGNAdqfdtltsdalglvgvpaavavpcxtiPWQIa  186
ident      |                |
Sbjct LGVCRLPG--PGRRHRRLDIIVVPYSEF--------------------------aCALL-  255
DSSP  EEEELLLL--LLLLLLEEEEEEEELLHHHH--------------------------hHHHH-


DSSP  hhhhhhhlllllllllllHHHHHHH-------------------------hhhhhllhH
Query qklhavtavleepkandraHDLVDLQ-------------------------lleglllldA  221
ident
Sbjct ---------------yftgSAHFNRSmralaktkgmslsehalstavvrnthgckvgpgR  300
DSSP  ---------------hhhlLHHHHHHhhhhhhhllleellllleeelleelllllleeellE


DSSP  HHHhhHHHHHhhhhhhllllLLLLLlllllhhhhhhhhhlllllllllhhhhhhhhhh
Query DLXptRSACIaifearaqhpWPPRVatlphwpliyagalegldhlelartvdaaaqavqr  281
ident  |
Sbjct VLP--TPTEK-------dvfRLLGL-----------------------------------  316
DSSP  ELL--LLLHH-------hhhHHLLL-----------------------------------


DSSP  hhhhhhhllll
Query fvaridratkr  292
ident
Sbjct -pyrepaerdw  326
DSSP  -llllhhhlll
```

No 335: Query=mol1A Sbjct=5iinA Z-score=6.5

back to top

```
DSSP  ------------------------------------------------------------
Query ------------------------------------------------------------    0
ident
Sbjct tnhnlhiteklevlakaysvqgdkwralgyakainalksfhkpvtsyqeacsipgigkrm   60
DSSP  llllhhhhhhhhhhhhhhhhlllhhhhhhhhhhhhhhhhlllllllhhhhhlllllllhhh


DSSP  ---------------------------------------------llllllllllhhhhh
Query ---------------------------------------------aapysspptnlrslr   15
ident
Sbjct aekiieilesghlrkldhisesvpvlelfsniwgagtktaqmwyqqgfrsledirsqasl  120
DSSP  hhhhhhhhhhlllhhhhhlllhhhhhhhhhlllllhhhhhhhhhlllllhhhhhhhlll


DSSP  hhhhhhhhhllllhhHHHHHHHHHHHHHHHHHHL-LLLLLLLLEEEEHHHHHHHhhlllll
Query drltqvaerqgvvfgRLQRHVAXIVVAQFAAT-LTDDTGAPLLLVKGGSSLELrrgipds   74
ident                            |             |    |
Sbjct ttqqaiglkhysdflERMPREEATEIEQTVQKaAQAFNSGLLCVACGSYRRGK-------  173
DSSP  lhhhhhhhhlhhhhhLLEEHHHHHHHHHHHHHHHhHHLLLLLLLEEEELHHHHLLL-------


DSSP  lLLLLEEEEEL--------lLHHHHHHHHHHHHHhhllllleeeEELLLEELLllllLLLLE
Query rTSKDFATVAR--------rDIELIHEQLADAGetgwegftaIFTAPEEIAapgxPVKPR  126
ident  |  |                       |   |           |
```

```
DSSP  lEELLEEEEEElllllllllLHHHHHHHHHHLL---------LEEEEEEELLllllLLLEE


DSSP  EEEEEEEEllEEEEEEEEEEEEELLLLLLlleeeeelhhhhllllleeeeellhHHHHh
Query RFTAKLSYrgRAFATVPIEVSSVEAGNAdqfdtltsdalglvgvpaavavpcxtiPWQIa  186
ident      |                |
Sbjct LGVCRLPG--PGRRHRRLDIIVVPYSEF--------------------------aCALL-  255
DSSP  EEEELLLL--LLLLLEEEEEEELLHHHH--------------------------hHHHH-


DSSP  hhhhhhhllllllllllllHHHHHHH-------------------------hhhhhllhH
Query qklhavtavleepkandraHDLVDLQ-------------------------lleglllldA  221
ident
Sbjct ---------------yftgSAHFNRSmralaktkgmslsehalstavvrnthgckvgpgR  300
DSSP  ---------------hhhlLHHHHHHhhhhhhhllleellllleeelleelllllleeellE


DSSP  HHHhhHHHHHhhhhhhllllLLLLLlllllhhhhhhhhhlllllllllllhhhhhhhhh
Query DLXptRSACIaifearaqhpWPPRVatlphwpliyagalegldhlelartvdaaaqavqr  281
ident  |
Sbjct VLP--TPTEK-------dvfRLLGL-----------------------------------  316
DSSP  ELL--LLLHH-------hhhHHLLL-----------------------------------


DSSP  hhhhhhhllll
Query fvaridratkr  292
ident
Sbjct -pyrepaerdw  326
DSSP  -llllhhhlll
```

No 336: Query=mol1A Sbjct=4rq8A Z-score=6.4

back to top

```
DSSP  ------------------------------------------------------------
Query ------------------------------------------------------------    0
ident
Sbjct etlnggitdmltelanfeknvsqaihkynayrkaasviakyphkiksgaeakklpgvgtk   60
DSSP  llllhhhhhhhhhhhhhhhhlllllhhhhhhhhhhhhhhhhllllllllhhhhhllllllhh


DSSP  -------------------------------------------------lllllllllh
Query -------------------------------------------------aapyssspptnl   11
ident
Sbjct iaekideflatgklrklekirqddtsssinfltrvsgigpsaarkfvdegiktledlrkn  120
DSSP  hhhhhhhhhhhllllhhhhhhhhlhhhhhhhhhllllllllhhhhhhhhhlllllhhhhhhl


DSSP  hhhhhhhhhhhhllllhhhhhHHHHHHHHHHHHH-LLLLLLLLLLEEEEHHHHHHHHhhl
Query rslrdrltqvaerqgvvfgrlqRHVAXIVVAQFAA-TLTDDTGAPLLLVKGGSSLELrrg   70
ident                                                 | |
Sbjct edklnhhqriglkyfgdfekriPREEMLQMQDIVLnEVKKVDSEYIATVCGSFRRGA---  177
DSSP  hhhllhhhhhhhhlhhhhhlleEHHHHHHHHHHHHHhHHHHHLLLLEEEELHHHHLLL---


DSSP  llllllLLLLEEEEEEL------------lLHHHHHHHHHHHHHhhllllleeEEELLLEELll
Query ipdsrTSKDFATVAR------------rDIELIHEQLADAGetgwegftAIFTAPEEIaa  118
ident       | |                         |||             |
Sbjct ----eSSGDMDVLLThpsftsestkqpkLLHQVVEQLQKVH--------FITDTLSKG--  223
DSSP  ----lEELLEEEEEEllleelleellllHHHHHHHHHHHHHLL--------LEEEEEEEL--


DSSP  llllLLLEEEEEEEEEEEL--LEEEEEEEEEEEEEL-LLLLllllleeeeelhhhhlllllllee
Query pgxpVKPRRFTAKLSYR--GRAFATVPIEVSSV-EAGNadqfdtltsdalglvgvpaava  175
ident              |             |
Sbjct ----ETKFMGVCQLPSKndEKEYPHRRIDIRLIpKDQY----------------------  257
DSSP  ----LLEEEEEEELLLLLllLLLLLLEEEEEEEELlHHHH----------------------


DSSP  eeellhHHHHhhhhhhhhlllllllllllHHHHHHHhhhhhllhhhhhhhhhHHHH---
Query vpcxtiPWQIaqklhavtavleepkandraHDLVDLQlleglllldadlxptrsACIA---  232
ident                                |                       |
Sbjct -----yCGVL----------------yftgSDIFNKN--mrahalekgftineYTIRplg  294
DSSP  -----hHHHH----------------hhhlLHHHHHH--hhhhhhhhleeellLLEEell
```

```
Query ------lFEAR---aqnpwPPRVAtlphwpllyagALEGldhlelartvdaaaqavqrfv  283
ident
Sbjct vtgvagePLPVdsekdifdYIQWK----yrepkdrSEHH---------------------  329
DSSP  lllllllLLLLllhhhhhhHLLLL----lllhhhhLLLL---------------------


DSSP  hhhhhllll
Query aridratkr  292
ident
Sbjct -----hhhh  333
DSSP  -----llll
```

No 337: Query=mol1A Sbjct=4fhxA Z-score=6.4

back to top

```
DSSP  lllllllllhhhhhhhhhhhhhhlLLLHHHHHHHHHHHHHHHHHHHLLLlllllLLEEEE
Query aapysspptnlrslrdrltqvaerqGVVFGRLQRHVAXIVVAQFAATLTddtgaPLLLVK   60
ident                                       |                 |
Sbjct --------shkeftkfcyevyneikISDKEFKEKRAALDTLRLCLKRIS-----PDAELV   47
DSSP  --------llhhhhhhhhhhhhhhlLLHHHHHHHHHHHHHHHHHHHHHLLL-----LLLEEE


DSSP  HHhhhhhhhllllllllLLEEEEEL--LLHHHHHHHHHHHHHHLllleEEEELLLEELll
Query GGsslelrrgipdsrtsKDFATVAR--RDIELIHEQLADAGETGwegfTAIFTAPEEIaa  118
ident                   |             |  |
Sbjct AF--gslesglalknsdMDLCVLMDsrVQSDTIALQFYEELIAE----GFEGKFLQRA--   99
DSSP  EE--lhhhhllllllllEEEEEELLllLLHHHHHHHHHHHHHHL----LEEEEEEEEL--


DSSP  lllllLLEEEEEEEEEELLEEE--EEEEEEEEellllllllleeeeelhhhhllllllleee
Query pgxpvKPRRFTAKLSYRGRAF--ATVPIEVSsveagnadqfdtltsdalglvgvpaavav  176
ident                            |
Sbjct -----RIPIIKLTSDTKNGFGasFQCDIGFN-----------------------------  125
DSSP  -----LEEEEEEEEELLLLLLllLLEEEEEL-----------------------------


DSSP  eellhhhhhhHHHHHHhllllllllLLLLhhhhhhhhhhhhllhhhhhhhhhhhhhhhh
Query pcxtipwqiaQKLHAVtavleepkaNDRAhdlvdlqlleglllldadlxptrsaciaifea  236
ident
Sbjct ------nrlaIHNTLL---------LSSY-------------------------------  139
DSSP  ------lhhhHHHHHH---------HHHH-------------------------------


DSSP  hllllllllllllllHHHHhHHHHLLL-LLLL------------------lllLHHHHHH
Query raqhpwpprvatlphWPLIyAGALEGL-DHLE------------------larTVDAAAQ  277
ident                           |  |
Sbjct -------------tkLDAR-LKPMVLLvKHWAkrkqinspyfgtlssygyvlmVLYYLIH  185
DSSP  -------------hhHLLL-HHHHHHHhHHHHhhlllllhhhllllhhhhhhhHHHHHHH


DSSP  ------------------------------------------------------------
Query ------------------------------------------------------------  277
ident
Sbjct vikppvfpnlllsplkqekivdgfdvgfddkledippsqnysslgsllhgffrfyaykfe  245
DSSP  llllllllhhhlllllllleelleellllllhhhlllllllllhhhhhhhhhhhhhhlll


DSSP  -----hhHHHH---hhhhHLLLL-------------------------------------
Query -----avQRFV---aridRATKR-------------------------------------  292
ident         |
Sbjct prekvvtFRRPdgyltkqEKGWTryilaiedpfeisnnvgrtvsssglyrirgefmaasr  305
DSSP  lllllllLLLLlllllhhHHLLLlllllllllllllhhhhllhhhhhhhhhhhhhhhh


DSSP  ------------------
Query ------------------  292
ident
Sbjct llnsrsypipydslfeea  323
DSSP  hhllllllllhhhhhlll
```

No 338: Query=mol1A Sbjct=4rq4A Z-score=6.4

```
DSSP  ------------------------------------------------------------
Query ------------------------------------------------------------    0
ident
Sbjct tlnggitdmltelanfeknvsqaihkynayrkaasviakyphkiksgaeakklpgvgtki   60
DSSP  lllhhhhhhhhhhhhhhhhllllhhhhhhhhhhhhhhhhllllllllhhhhhlllllllhhh


DSSP  ------------------------------------------------llllllllllhh
Query ------------------------------------------------aapysspptnlr   12
ident
Sbjct aekideflatgklrklekirqddtsssinfltrvsgigpsaarkfvdegiktledlrkne  120
DSSP  hhhhhhhhhhlllhhhhhhhhlhhhhhhhhhhlllllhhhhhhhhhlllllhhhhhhlh


DSSP  hhhhhhhhhhhhllllhhhhhHHHHHHHHHHHHHH-LLLLllLLLLEEEEHHHHHHHHhhll
Query slrdrltqvaerqgvvfgrlqRHVAXIVVAQFAA-TLTDdtGAPLLLVKGGSSLELrrgi    71
ident                                                 | |
Sbjct dklnhhqriglkyfgdfekriPREEMLQMQDIVLnEVKKvdSEYIATVCGSFRRGA----  176
DSSP  hhllhhhhhhhhlhhhhhlleEHHHHHHHHHHHHHhHHHHhlLLLEEEELHHHHLLL----


DSSP  llllLLLLEEEEEL------------lLHHHHHHHHHHHHHhhlllleeEEELLLEElllll
Query pdsrTSKDFATVAR------------rDIELIHEQLADAGetgwegftAIFTAPEEiaap  119
ident      | |                         |||              |
Sbjct ---eSSGDMDVLLThpsftsestkqpkLLHQVVEQLQKVH--------FITDTLSK----  221
DSSP  ---lEELLEEEEEllllllllllllHHHHHHHHHHHLL--------LEEEEEEE----


DSSP  lllLLLEEEEEEEEEL--LEEEEEEEEEEEEL-LLLLllllleeeeelhhhhllllllleee
Query gxpVKPRRFTAKLSYR--GRAFATVPIEVSSV-EAGNadqfdtltsdalglvgvpaavav  176
ident              |             |
Sbjct --gETKFMGVCQLPSKndEKEYPHRRIDIRLIpKDQY----------------------  256
DSSP  --lLLEEEEEELLLLLllLLLLLLEEEEEEEELlHHHH----------------------


DSSP  eellhHHHHhhhhhhhhlllllllllllHHHH------------------hhhhhhhh
Query pcxtiPWQIaqklhavtavleepkandraHDLV------------------dlqllegl  217
ident                              |
Sbjct ----yCGVL----------------yftgSDIFnknmrahalekgftineytirplgvtg  296
DSSP  ----hHHHH----------------hhhlLHHHhhhhhhhhhhlleeellllleeellll


DSSP  llhHHHHHhhHHHHhhhhhhlllLLLLLLlllllhhhhhhhhhlllllllllllhhhhhh
Query lldADLXPtrSACIaifearaqhPWPPRVatlphwpliyagalegldhlelartvdaaaq  277
ident      |
Sbjct vagEPLPV--DSEK-----difdYIQWKY-------------------------------  318
DSSP  lllLLLLL--LLHH-----hhhhHLLLLL-------------------------------


DSSP  hhhhhhhhhhhllll
Query avqrfvaridratkr  292
ident
Sbjct -------repkdrse  326
DSSP  -------llhhhhll
```

No 339: Query=mol1A Sbjct=3pmlA Z-score=6.4

back to top

```
DSSP  ------------------------------------------------------------
Query ------------------------------------------------------------    0
ident
Sbjct atnhnlhiteklevlakaysvqgdkwralgyakainalksfhkpvtsyqeacsipgigkr   60
DSSP  llllllhhhhhhhhhhhhhhhhhlllhhhhhhhhhhhhhhhlllllllhhhhhlllllllhh


DSSP  ----------------------------------------------llllllllllhhhh
Query ----------------------------------------------aapysspptnlrsl   14
ident
Sbjct maekiieilesghlrkldhisesvpvlelfsniwgagtktaqmwyqqgfrsledirsqas  120
DSSP  hhhhhhhhhhhlllhhhhlllllhhhhhhhhlllllhhhhhhhhhlllllhhhhhhlll


DSSP  hhhhhhhhhllllhHHHHHHHHHHHHHHHHHH-LLLLLLLLLLEEEEHHHHHHHHhhllll
Query rdrltqvaerqgvvfGRLQRHVAXIVVAQFAA-TLTDDTGAPLLLVKGGSSLELrrgipd   73
```

```
Sbjct lttqqaiglkhysdfLERMPREEATEIEQTVQkAAQAFNSGLLCVACGSYRRGK------  174
DSSP  llhhhhhhhhlhhhhHLLEEHHHHHHHHHHHHhHHHLLLLLLEEEELHHHHLLL------


DSSP  llLLLLEEEEEL--------lLHHHHHHHHHHHHhhlllleeEEELLLEELllllllLLL
Query srTSKDFATVAR--------rDIELIHEQLADAGetgwegftAIFTAPEEIaapgxpVKP  125
ident   |  |                       |   |
Sbjct -aTCGDVDVLIThpdgrshrgIFSRLLDSLRQEG--------FLTDDLVKG------ETK  219
DSSP  -lEELLEEEEEEllllllllllLHHHHHHHHHHLL--------LEEEEEEEL------LLE


DSSP  EEEEEEEEEllEEEEEEEEEEEEL-LLLLlllleeeeelhhhhllllllleeeeellhHHH
Query RRFTAKLSYrgRAFATVPIEVSSV-EAGNadqfdtltsdalglvgvpaavavpcxtiPWQ  184
ident       |                |
Sbjct YLGVCRLPG--PGRRHRRLDIIVVpYSEF---------------------------aCAL  250
DSSP  EEEEELLLL--LLLLLEEEEEEELlHHHH---------------------------hHHH


DSSP  HhhhhhhhhllllllllllllHHHHHHH-------------------------hhhhhll
Query IaqklhavtavleepkandraHDLVDLQ-------------------------lleglll  219
ident
Sbjct L----------------yftgSAHFNRSmralaktkgmslsehalstavvrnthgckvgp  294
DSSP  H----------------hhhlLHHHHHHhhhhhhlleeellllleeelleellllleeel


DSSP  hHHHHhhHHHHHhhhhhhllllLLLLLlllllhhhhhhhhhllllllllllhhhhhhhh
Query dADLXptRSACIaifearaqhpWPPRVatlphwpliyagalegldhlelartvdaaaqav  279
ident    |
Sbjct gRVLP--TPTEK-------dvfRLLGL---------------------------------  312
DSSP  lLLLL--LLLHH-------hhhHHLLL---------------------------------


DSSP  hhhhhhhhhllll
Query qrfvaridratkr  292
ident
Sbjct ---pyrepaerdw  322
DSSP  ---llllhhhlll
```

No 340: Query=mol1A Sbjct=3mgiA Z-score=6.4

back to top

```
DSSP  ------------------------------------------------------------
Query ------------------------------------------------------------    0
ident
Sbjct hnlhiteklevlakaysvqgdkwralgyakainalksfhkpvtsyqeacsipgigkrmae   60
DSSP  llllhhhhhhhhhhhhhlllhhhhhhhhhhhhhhhhhlllllllhhhhhllllllhhhhh


DSSP  -------------------------------------------llllllllllhhhhhhh
Query -------------------------------------------aapysspptnlrslrdr   17
ident
Sbjct kiieilesghlrkldhisesvpvlelfsniwgagtktaqmwyqqgfrsledirsqasltt  120
DSSP  hhhhhhhllllhhhhhllllhhhhhhhhhlllllllhhhhhhhhhllllllhhhhhhhllllh


DSSP  hhhhhhhllllhhhhHHHHHHHHHHHHHHHHL-LLLLLLLLEEEEHHHHHHHHhhllllllL
Query ltqvaerqgvvfgrlQRHVAXIVVAQFAAT-LTDDTGAPLLLVKGGSSLELrrgipdsrT   76
ident                          |             |    |              |
Sbjct qqaiglkhysdflerMPREEATEIEQTVQKaAQAFNSGLLCVACGSYRRGK-------aT  173
DSSP  hhhhhhhlllhhhllEEHHHHHHHHHHHHHHHhHHHLLLLLLEEEELHHHHLLL-------lE


DSSP  LLLEEEEEL--------lLHHHHHHHHHHHHhhlllleeEEELLLEELllllllLLLEEE
Query SKDFATVAR--------rDIELIHEQLADAGetgwegftAIFTAPEEIaapgxpVKPRRF  128
ident   |                       |   |
Sbjct CGDVDVLIThpdgrshrgIFSRLLDSLRQEG--------FLTDDLVKG------ETKYLG  219
DSSP  ELLEEEEEEllllllllllHHHHHHHHHHHLL--------LEEEEEEEL------LLEEEE


DSSP  EEEEEEllEEEEEEEEEEEEL-LLLLlllleeeeelhhhhllllllleeeeellhHHHHhh
Query TAKLSYrgRAFATVPIEVSSV-EAGNadqfdtltsdalglvgvpaavavpcxtiPWQIaq  187
ident    |                |
Sbjct VCRLPG--PGRRHRRLDIIVVpYSEF---------------------------aCALL--  248
DSSP  EELLLL--LLLLLEEEEEEELlHHHH---------------------------hHHHH--
```

```
DSSP  hhhhhhllllllllllllHHHHHHH-------------------------hhhhhllhHH
Query klhavtavleepkandraHDLVDLQ-------------------------llegllldAD  222
ident
Sbjct --------------yftgSAHFNRSmralaktkgmslsehalstavvrnthgckvgpgRV  294
DSSP  --------------hhhlLHHHHHHhhhhhhhllleellllleeelleellllleeellEE


DSSP  HHhhHHHHHhhhhhhllllLLLLLlllllhhhhhhhhhllllllllllhhhhhhhhhhhh
Query LXptRSACIaifearaqhpWPPRVatlphwpliyagalegldhlelartvdaaaqavqrf  282
ident |
Sbjct LP--TPTEK-------dvfRLLGL------------------------------------  309
DSSP  EL--LLLHH-------hhhHHLLL------------------------------------


DSSP  hhhhhhllll
Query varidratkr  292
ident
Sbjct -pyrepaerd  318
DSSP  -llllhhhhl
```

No 341: Query=mol1A Sbjct=3uq2A Z-score=6.4

back to top

```
DSSP  ------------------------------------------------------------
Query ------------------------------------------------------------    0
ident
Sbjct nlhiteklevlakaysvqgdkwralgyakainalksfhkpvtsyqeacsipgigkrmaek   60
DSSP  llllhhhhhhhhhhhhhlllhhhhhhhhhhhhhhhhlllllllhhhhhhlllllhhhhhh


DSSP  ------------------------------------------llllllllllhhhhhhhh
Query ------------------------------------------aapysspptnlrslrdrl   18
ident
Sbjct iieilesghlrkldhisesvpvlelfsniwgagtktaqmwyqqgfrsledirsqaslttq  120
DSSP  hhhhhhllllhhhhlllllhhhhhhhhlllllhhhhhhhhhlllllhhhhhhhhllllhh


DSSP  hhhhhhllllhhhhHHHHHHHHHHHHHHH-LLLLllLLLLEEEEHHHHHHHhhllllllLL
Query tqvaerqggvvfgrlQRHVAXIVVAQFAA-TLTDdtGAPLLLVKGGSSLELrrgipdsrTS   77
ident                         |             |    |              |
Sbjct qaiglkhysdflerMPREEATEIEQTVQkAAQAfnSGLLCVACGSYRRGK-------aTC  173
DSSP  hhhhhhlhhhhlllEEHHHHHHHHHHHHHhHHLLllLLLEEEELHHHHLLL-------lEE


DSSP  LLEEEEEL--------lLHHHHHHHHHHHHhhlllleeEEELLLEELllllllLLLEEEE
Query KDFATVAR--------rDIELIHEQLADAGetgwegftAIFTAPEEIaapgxpVKPRRFT  129
ident  |                       |   |
Sbjct GDVDVLIThpdgrshrgIFSRLLDSLRQEG--------FLTDDLVKG------ETKYLGV  219
DSSP  LLEEEEEEelllllllhHHHHHHHHHHHLL--------LEEEEEEEL------LLEEEEE


DSSP  EEEEEllEEEEEEEEEEEEL-LLLLlllleeeeelhhhhlllllleeeeellhHHHHhhh
Query AKLSYrgRAFATVPIEVSSV-EAGNadqfdtltsdalglvgvpaavavpcxtiPWQIaqk  188
ident   |                |
Sbjct CRLPG--PGRRHRRLDIIVVpYSEF---------------------------aCALL---  247
DSSP  ELLLL--LLLLLEEEEEEEEeHHHH---------------------------hHHHH---


DSSP  hhhhhllllllllllllHHHHHHH-------------------------hhhhhllhHHH
Query lhavtavleepkandraHDLVDLQ-------------------------llegllldADL  223
ident                                                            |
Sbjct -------------yftgSAHFNRSmralaktkgmslsehalstavvrnthgakvgpgRVL  294
DSSP  -------------hhhlLHHHHHHhhhhhhhllleellllleeelleellllleeellEEL


DSSP  HhhHHHHHhhhhhhllllLLLLLlllllhhhhhhhhhllllllllllhhhhhhhhhhhhh
Query XptRSACIaifearaqhpWPPRVatlphwpliyagalegldhlelartvdaaaqavqrfv  283
ident
Sbjct P--TPTEK-------dvfRLLGL------------------------------------p  309
DSSP  L--LLLHH-------hhhHHHLL------------------------------------l


DSSP  hhhhhllll
Query aridratkr  292
ident
```

```
DSSP  lllhhhhll
```

No 342: Query=mol1A Sbjct=2isoA Z-score=6.4

back to top

```
DSSP  ------------------------------------------------------------
Query ------------------------------------------------------------    0
ident 
Sbjct tlnggitdmltelanfeknvsqaihkynayrkaasviakyphkiksgaeakklpgvgtki   60
DSSP  lllhhhhhhhhhhhhhhhhhllllhhhhhhhhhhhhhhhhlllllllhhhhhlllllhhh


DSSP  ------------------------------------------------llllllllllhh
Query ------------------------------------------------aapysspptnlr   12
ident 
Sbjct aekideflatgklrklekirqddtsssinfltrvsgigpsaarkfvdegiktledlrkne  120
DSSP  hhhhhhhhhhlllhhhhhhhhlhhhhhhhhhlllllllhhhhhhhhhhllllhhhhhhlh


DSSP  hhhhhhhhhhhhllllhhhhhHHHHHHHHHHHHH-LLLLLLLLLEEEEHHHHHHHHhhll
Query slrdrltqvaerqgvvfgrlqRHVAXIVVAQFAA-TLTDDTGAPLLLVKGGSSLELrrgi   71
ident                                                | |
Sbjct dklnhhqriglkyfgdfekriPREEMLQMQDIVLnEVKKVDSEYIATVCGSFRRGA----  176
DSSP  hhllhhhhhhhhlhhhhllleEHHHHHHHHHHHHhHHHHHLLLLEEEELHHHHLLL----


DSSP  llllLLLLEEEEEL------------lLHHHHHHHHHHHHhhlllleeEEELLLEELlll
Query pdsrTSKDFATVAR------------rDIELIHEQLADAGetgwegftAIFTAPEEIaap  119
ident      | |                         |||             |
Sbjct ---eSSGDMDVLLThpsftsestkqpkLLHQVVEQLQKVH--------FITDTLSKG---  222
DSSP  ---lEELLEEEEEEllllllllllllllHHHHHHHHHHHLL--------LEEEEEEEL---


DSSP  lllLLLEEEEEEEEEL--LEEEEEEEEEEEEL-LLLLlllleeeeelhhhhlllllleee
Query gxpVKPRRFTAKLSYR--GRAFATVPIEVSSV-EAGNadqfdtltsdalglvgvpaavav  176
ident             |             |
Sbjct ---ETKFMGVCQLPSKndEKEYPHRRIDIRLIpKDQY-----------------------  256
DSSP  ---LLEEEEEEELLLLllLLLLLLEEEEEEELlHHHH-----------------------


DSSP  eellhHHHHhhhhhhhhhlllllllllllHHHHHHH-------------------hhhhh
Query pcxtiPWQIaqklhavtavleepkandraHDLVDLQ-------------------llegl  217
ident                               |
Sbjct ----yCGVL----------------yftgSDIFNKNmrahalekgftineytirplgvtg  296
DSSP  ----hHHHH----------------hhhlLHHHHHHhhhhhhhhlleeelllleeellll


DSSP  llhHHHHhhHHHHHhhhhhhllllLLLLLLllllhhhhhhhhhlllllllllllhhhhhh
Query lldADLXptRSACIaifearaqhpWPPRVAtlphwpliyagalegldhlelartvdaaaq  277
ident      |
Sbjct vagEPLP--VDSEK-------difDYIQWK------------------------------  317
DSSP  lllLLLL--LLLHH-------hhhHHLLLL------------------------------


DSSP  hhhhhhhhhhhllll
Query avqrfvaridratkr  292
ident 
Sbjct ------yrepkdrse  326
DSSP  ------lllhhhlll
```

No 343: Query=mol1A Sbjct=1mq3A Z-score=6.4

back to top

```
DSSP  ------------------------------------------------------------
Query ------------------------------------------------------------    0
ident 
Sbjct tlnggitdmltelanfeknvsqaihkynayrkaasviakyphkiksgaeakklpgvgtki   60
DSSP  lllhhhhhhhhhhhhhhhhhllllhhhhhhhhhhhhhhhhlllllllhhhhhllllllhh


DSSP  ------------------------------------------------llllllllllhh
```

```
ident
Sbjct aekideflatgklrklekirqddtsssinfltrvsgigpsaarkfvdegiktledlrkne  120
DSSP  hhhhhhhhhhlllhhhhhhhhlllhhhhhhhllllllhhhhhhhhhllllllhhhhhhll


DSSP  hhhhhhhhhhhhllllhhhhhHHHHHHHHHHHHH-LLLLllLLLLLEEEEHHHHHHHHhhll
Query slrdrltqvaerqgvvfgrlqRHVAXIVVAQFAA-TLTDdtGAPLLLVKGGSSLELrrgi   71
ident                                                |  |
Sbjct dklnhhqriglkyfgdfekriPREEMLQMQDIVLnEVKKvdSEYIATVCGSFRRGA----  176
DSSP  llllhhhhhhhhhllhhhlleEHHHHHHHHHHHHHhHHHHhlLLLEEEEELHHHHLLL----


DSSP  llllLLLLEEEEEL------------lLHHHHHHHHHHHHhhllllleeEEELLLEELlll
Query pdsrTSKDFATVAR------------rDIELIHEQLADAGetgwegftAIFTAPEEIaap  119
ident      | |                         |||             |
Sbjct ---eSSGDMDVLLThpsftsestkqpkLLHQVVEQLQKVH--------FITDTLSKG---  222
DSSP  ---lEELLEEEEEElllleelleellllHHHHHHHHHHHHLL--------LEEEEEEEL---


DSSP  lllLLLEEEEEEEEEL--LEEEEEEEEEEEL-LLLLllllleeeeelhhhhllllllleee
Query gxpVKPRRFTAKLSYR--GRAFATVPIEVSSV-EAGNadqfdtltsdalglvgvpaavav  176
ident             |             |
Sbjct ---ETKFMGVCQLPSKndEKEYPHRRIDIRLIpKDQY-----------------------  256
DSSP  ---LLEEEEEELLLLLllLLLLLLEEEEEEELlHHHH-----------------------


DSSP  eellhHHHHhhhhhhhhlllllllllllHHHHHHH------------------hhhhh
Query pcxtiPWQIaqklhavtavleepkandraHDLVDLQ------------------llegl  217
ident                               |
Sbjct ----yCGVL----------------yftgSDIFNKNmrahalekgftineytirplgvtg  296
DSSP  ----hHHHH----------------hhhlLHHHHHHhhhhhhhhleeellllleeellll


DSSP  llhHHHHhhHHHHHhhhhhhllllLLLLLLllllhhhhhhhhhllllllllllhhhhhh
Query lldADLXptRSACIaifearaqhpWPPRVAtlphwpliyagalegldhlelartvdaaaq  277
ident      |
Sbjct vagEPLP--VDSEK-------difDYIQWK------------------------------  317
DSSP  lllLLLL--LLLHH-------hhhHHLLLL------------------------------


DSSP  hhhhhhhhhhhllll
Query avqrfvaridratkr  292
ident
Sbjct ------yrepkdrse  326
DSSP  ------lllhhhlll
```

No 344: Query=mol1A Sbjct=1zqsA Z-score=6.4

back to top

```
DSSP  ------------------------------------------------------------
Query ------------------------------------------------------------    0
ident
Sbjct etlnggitdmltelanfeknvsqaihkynayrkaasviakyphkiksgaeakklpgvgtk   60
DSSP  llllhhhhhhhhhhhhhhhhhllllhhhhhhhhhhhhhhhhlllllllhhhhhllllllhh


DSSP  -------------------------------------------------llllllllllh
Query -------------------------------------------------aapysspptnl   11
ident
Sbjct iaekideflatgklrklekirqddtsssinfltrvsgigpsaarkfvdegiktledlrkn  120
DSSP  hhhhhhhhhhllllhhhhhhhhlhhhhhhhhhlllllllhhhhhhhhhhllllhhhhhhl


DSSP  hhhhhhhhhhhhhllllhhhhhHHHHHHHHHHHHH-LLLLllLLLLLEEEEHHHHHHHHhhl
Query rslrdrltqvaerqgvvfgrlqRHVAXIVVAQFAA-TLTDdtGAPLLLVKGGSSLELrrg   70
ident                                                 |  |
Sbjct edklnhhqriglkyfgdfekriPREEMLQMQDIVLnEVKKvdSEYIATVCGSFRRGA---  177
DSSP  lllllhhhhhhhhlhhhhhlleEHHHHHHHHHHHHHhHHHHhlLLLEEEEELLLLLLL---


DSSP  lllllLLLLEEEEEL------------lLHHHHHHHHHHHHhhllllleeEEELLLEELll
Query ipdsrTSKDFATVAR------------rDIELIHEQLADAGetgwegftAIFTAPEEIaa  118
ident       | |                         |||             |
Sbjct ----eSSGDMDVLLThpsftsestkqpkLLHQVVEQLQKVH--------FITDTLSKG--  223
DSSP  ----lEELLEEEEEEelllllllllllhHHHHHHHHHHHHLL--------LEEEEEEEL--
```

```
DSSP  llllLLLEEEEEEEEEELL--EEEEEEEEEEEEL-LLLLllllleeeeelhhhhllllllee
Query pgxpVKPRRFTAKLSYRG--RAFATVPIEVSSV-EAGNadqfdtltsdalglvgvpaava  175
ident              |             |
Sbjct ----ETKFMGVCQLPSKNdeKEYPHRRIDIRLIpKDQY----------------------  257
DSSP  ----LLEEEEEEELLLLLLlLLLLLLEEEEEEEEEeLLLH----------------------


DSSP  eeellhHHHHhhhhhhhhlllllllllllhhhhHHHHHH--------------------
Query vpcxtiPWQIaqklhavtavleepkandrahdlvDLQLLE--------------------  215
ident
Sbjct -----yCGVL--------------------yftgSDIFNKnmrahalekgftineytirp  292
DSSP  -----hHHHH--------------------hhllLHHHHHhhhhhhhhlleeeelleeee


DSSP  --hhllhhhhhhhhHHHHhhhhhhllllLLLLLllllllhhhhhhhhhllllllllllhh
Query --glllldadlxptrSACIaifearaqhpWPPRVatlphwpliyagalegldhlelartvd  273
ident               |
Sbjct lgvtgvageplpvdSEKD-------ifdYIQWK---------------------------  318
DSSP  llllllllllllllLHHH-------hhhHLLLL---------------------------


DSSP  hhhhhhhhhhhhhhhllll
Query aaaqavqrfvaridratkr  292
ident
Sbjct ----------yrepkdrse  327
DSSP  ----------lllhhhlll
```

No 345: Query=mol1A Sbjct=1zqhA Z-score=6.4

back to top

```
DSSP  ------------------------------------------------------------
Query ------------------------------------------------------------    0
ident
Sbjct etlnggitdmltelanfeknvsqaihkynayrkaasviakyphkiksgaeakklpgvgtk   60
DSSP  llllhhhhhhhhhhhhhhhhllllhhhhhhhhhhhhhhhhllllllllhhhhhllllllhh


DSSP  --------------------------------------------------llllllllllh
Query --------------------------------------------------aapysspptnl   11
ident
Sbjct iaekideflatgklrklekirqddtsssinfltrvsgigpsaarkfvdegiktledlrkn  120
DSSP  hhhhhhhhhhllllhhhhhhhhlhhhhhhhhhlllllllhhhhhhhhhhllllhhhhhhl


DSSP  hhhhhhhhhhhhllllhhhhhHHHHHHHHHHHHHH-LLLLllLLLLEEEEHHHHHHHHhhl
Query rslrdrltqvaerqgvvfgrlqRHVAXIVVAQFAA-TLTDdtGAPLLLVKGGSSLELrrg   70
ident                                                 | |
Sbjct edklnhhqriglkyfgdfekriPREEMLQMQDIVLnEVKKvdSEYIATVCGSFRRGA---  177
DSSP  hhhllhhhhhhhlllllllleEHHHHHHHHHHHHHhHHHHhlLLLEEEEEELLLLLLL---


DSSP  lllllLLLLEEEEEL------------lLHHHHHHHHHHHHhhlllleeEEELLLEELll
Query ipdsrTSKDFATVAR------------rDIELIHEQLADAGetgwegftAIFTAPEEIaa  118
ident       | |                         |||             |
Sbjct ----eSSGDMDVLLThpsftsestkqpkLLHQVVEQLQKVH--------FITDTLSKG--  223
DSSP  ----lEELLEEEEEEllllllllllhhHHHHHHHHHHHHHL--------LEEEEEEEL--


DSSP  llllLLLEEEEEEEEEELL--EEEEEEEEEEEEL-LLLLllllleeeeelhhhhllllllee
Query pgxpVKPRRFTAKLSYRG--RAFATVPIEVSSV-EAGNadqfdtltsdalglvgvpaava  175
ident              |             |
Sbjct ----ETKFMGVCQLPSKNdeKEYPHRRIDIRLIpKDQY----------------------  257
DSSP  ----LLEEEEEEELLLLLLlLLLLLLLLEEEEELLlHHHH----------------------


DSSP  eeellhHHHHhhhhhhhhlllllllllllhhhhHHHHHH--------------------
Query vpcxtiPWQIaqklhavtavleepkandrahdlvDLQLLE--------------------  215
ident
Sbjct -----yCGVL--------------------yftgSDIFNKnmrahalekgftineytirp  292
DSSP  -----hHHHH--------------------hhhlLHHHHHhhhhhhhhlleeeelleeee


DSSP  --hhllhhhhhhhhHHHHhhhhhhllllLLLLLllllllhhhhhhhhhllllllllllhh
Query --glllldadlxptrSACIaifearaqhpWPPRVatlphwpliyagalegldhlelartvd  273
```

```
Sbjct lgvtgvageplpvdSEKD-------ifdYIQWK---------------------------  318
DSSP  llllllllllllllLHHH-------hhhHHLLL---------------------------


DSSP  hhhhhhhhhhhhhhhllll
Query aaaqavqrfvaridratkr  292
ident
Sbjct ----------yrepkdrse  327
DSSP  ----------lllhhhlll
```

No 346: Query=mol1A Sbjct=1zqjA Z-score=6.4

back to top

```
DSSP  ------------------------------------------------------------
Query ------------------------------------------------------------    0
ident
Sbjct etlnggitdmltelanfeknvsqaihkynayrkaasviakyphkiksgaeakklpgvgtk   60
DSSP  llllhhhhhhhhhhhhhhhlllllhhhhhhhhhhhhhllllllllllhhhhhllllllhh


DSSP  -------------------------------------------------llllllllllh
Query -------------------------------------------------aapyssspptnl   11
ident
Sbjct iaekideflatgklrklekirqddtsssinfltrvsgigpsaarkfvdegiktledlrkn  120
DSSP  hhhhhhhhhhllllhhhhhhhhlhhhhhhhhhllllllhhhhhhhhhhllllhhhhhhl


DSSP  hhhhhhhhhhhhllllhhhhhHHHHHHHHHHHHH-LLLLllLLLLEEEEHHHHHHHHhhl
Query rslrdrltqvaerqgvvfgrlqRHVAXIVVAQFAA-TLTDdtGAPLLLVKGGSSLELrrg   70
ident                                                 | |
Sbjct edklnhhqriglkyfgdfekriPREEMLQMQDIVLnEVKKvdSEYIATVCGSFRRGA---  177
DSSP  hhhllhhhhhhhhlhhhhhlllLHHHHHHHHHHHHhHHHHhlLLLEEEELLLLLLLL---


DSSP  lllllLLLLEEEEEL------------lLHHHHHHHHHHHHhhlllleeEEELLLEELll
Query ipdsrTSKDFATVAR------------rDIELIHEQLADAGetgwegftAIFTAPEEIaa  118
ident       | |                         |||             |
Sbjct ----eSSGDMDVLLThpsftsestkqpkLLHQVVEQLQKVH--------FITDTLSKG--  223
DSSP  ----lLLLLEEEEEEellllllllllhhHHHHHHHHHHHHLL--------LEEEEEELL--


DSSP  llllLLLEEEEEEEEEELL--EEEEEEEEEEEEL-LLLLllllleeeeelhhhhllllllee
Query pgxpVKPRRFTAKLSYRG--RAFATVPIEVSSV-EAGNadqfdtltsdalglvgvpaava  175
ident              |             |
Sbjct ----ETKFMGVCQLPSKNdeKEYPHRRIDIRLIpKDQY----------------------  257
DSSP  ----LLLLEEEELLLLLLllLLLLLLLLEEEEEEeHHHH----------------------


DSSP  eeellhHHHHhhhhhhhhlllllllllllhhhhHHHHHH--------------------
Query vpcxtiPWQIaqklhavtavleepkandrahdlvDLQLLE--------------------  215
ident
Sbjct -----yCGVL--------------------yftgSDIFNKnmrahalekgftineytirp  292
DSSP  -----hHHHH--------------------hhllLHHHHHhhhhhhhhlleeeelleeee


DSSP  -hhllhhhhhhhHHHHHhhhhhhllllLLLLLLllllhhhhhhhhhllllllllllllhhh
Query -glllldadlxptRSACIaifearaqhpWPPRVAtlphwpliyagalegldhlelartvda  274
ident
Sbjct lgvtgvageplpVDSEK-------difDYIQWK---------------------------  318
DSSP  llllllllllllLLLHH-------hhhHHHLLL---------------------------


DSSP  hhhhhhhhhhhhhhllll
Query aaqavqrfvaridratkr  292
ident
Sbjct ---------yrepkdrse  327
DSSP  ---------lllhhhlll
```

No 347: Query=mol1A Sbjct=3hwtA Z-score=6.4

back to top

```
Query ------------------------------------------------------------    0
ident
Sbjct teklevlakaysvqgdkwralgyakainalksfhkpvtsyqeacsipgigkrmaekiiei   60
DSSP  lhhhhhhhhhhhhhllhhhhhhhhhhhhhhhllllllllhhhhhhlllllhhhhhhhhhh


DSSP  --------------------------------------llllllllllhhhhhhhhhhhh
Query --------------------------------------aapysspptnlrslrdrltqva   22
ident
Sbjct lesghlrkldhisesvpvlelfsniwgagtktaqmwyqqgfrsledirsqaslttqqaig  120
DSSP  hhhlllhhhhhllllhhhhhhhhlllllhhhhhhhhhlllllhhhhhhhllllhhhhhhh


DSSP  hhllllhhhhHHHHHHHHHHHHHHL-LLLLLLLLEEEEHHHHHHHhhllllllLLLLEE
Query erqgvvfgrlQRHVAXIVVAQFAAT-LTDDTGAPLLLVKGGSSLELrrgipdsrTSKDFA   81
ident                     |             |    |              |  |
Sbjct lkhysdflerMPREEATEIEQTVQKaAQAFNSGLLCVACGSYRRGK-------aTCGDVD  173
DSSP  hhlllhhhllEEHHHHHHHHHHHHHHhHHHHLLLLEEEELHHHHLLL-------lEELEEE


DSSP  EEEL--------lLHHHHHHHHHHHHhhlllleeEEELLLEELlllllLLLLEEEEEEEE
Query TVAR--------rDIELIHEQLADAGetgwegftAIFTAPEEIaapgxPVKPRRFTAKLS  133
ident                      |   |                                |
Sbjct VLIThpdgrshrgIFSRLLDSLRQEG--------FLTDDLVSQ-eengQQQKYLGVCRLP  224
DSSP  EEEElllllllllLHHHHHHHHHHHLL--------LEEEEEELL-llllLLLEEEEEEELLL


DSSP  EllEEEEEEEEEEEEL-LLLLllllleeeeelhhhhllllllleeeeellhHHHHhhhhhhh
Query YrgRAFATVPIEVSSV-EAGNadqfdtltsdalglvgvpaavavpcxtiPWQIaqklhav  192
ident                |
Sbjct G--PGRRHRRLDIIVVpYSEF---------------------------aCALL-------  248
DSSP  L--LLLLLEEEEEEELlHHHH---------------------------hHHHH-------


DSSP  hllllllllllllHHHHHHH-------------------------hhhhhllhHHHHhhH
Query tavleepkandraHDLVDLQ-------------------------lleglllldADLXptR  227
ident                                                        |
Sbjct ---------yftgSAHFNRSmralaktkgmslsehalstavvrnthgakvgpgRVLP--T  297
DSSP  ---------hhhlLHHHHHHhhhhhhhllleellllleeelleelllllleeellEELL--L


DSSP  HHHHhhhhhhlllLLLLLLlllllhhhhhhhhhlllllllllllhhhhhhhhhhhhhhhh
Query SACIaifearaqhPWPPRVatlphwpliyagalegldhlelartvdaaaqavqrfvarid  287
ident
Sbjct PTEK-----dvfrLLGLPY---------------------------------------rep  314
DSSP  LLHH-----hhhhHLLLLL---------------------------------------llh


DSSP  hllll
Query ratkr  292
ident
Sbjct aerdw  319
DSSP  hhhll
```

No 348: Query=mol1A Sbjct=2pfpA Z-score=6.4

back to top

```
DSSP  ------------------------------------------------------------
Query ------------------------------------------------------------    0
ident
Sbjct tnhnlhiteklevlakaysvqgdkwralgyakainalksfhkpvtsyqeacsipgigkrm   60
DSSP  llllhhhhhhhhhhhhhhhhhlllhhhhhhhhhhhhhllllllllllhhhhhlllllhhh


DSSP  ---------------------------------------------llllllllllhhhhh
Query ---------------------------------------------aapysspptnlrslr   15
ident
Sbjct aekiieilesghlrkldhisesvpvlelfsniwgagtktaqmwyqqgfrsledirsqasl  120
DSSP  hhhhhhhhllllhhhhhllllhhhhhhhhlllllhhhhhhhhhlllllhhhhhhhhlll


DSSP  hhhhhhhhllllhhHHHHHHHHHHHHHHHHHLL-LLLLLLLLEEEEHHHHHHHhhlllll
Query drltqvaerqggvvfgRLQRHVAXIVVAQFAATL-TDDTGAPLLLVKGGSSLELrrgipds   74
ident                            |             |    |
Sbjct ttqqaiglkhysdflERMPREEATEIEQTVQKAaQAFNSGLLCVACGSYRRGK-------  173
```

```
DSSP  lLLLLEEEEEL--------lLHHHHHHHHHHHHhhllllleeeEELLLEELLllllLLLLE
Query rTSKDFATVAR--------rDIELIHEQLADAGetgwegftaIFTAPEEIAapgxPVKPR  126
ident  |  |                          |   |           |
Sbjct aTCGDVDVLIThpdgrshrgIFSRLLDSLRQEG---------FLTDDLVSQeengQQQKY  224
DSSP  lEELLEEEEEElllllllllLHHHHHHHHHHLL---------LEEEEEELLllllLLLEE


DSSP  EEEEEEEEllEEEEEEEEEEEEL-LLLLllllleeeeelhhhhlllllleeeeellhHHHH
Query RFTAKLSYrgRAFATVPIEVSSV-EAGNadqfdtltsdalglvgvpaavavpcxtiPWQI  185
ident       |                 |
Sbjct LGVCRLPG--PGRRHRRLDIIVVpYSEF---------------------------aCALL  255
DSSP  EEEELLLL--LLLLLEEEEEEEELlHHHH---------------------------hHHHH


DSSP  hhhhhhhhllllllllllllHHHHHHH-------------------------hhhhhllh
Query aqklhavtavleepkandraHDLVDLQ-------------------------lleglllld  220
ident
Sbjct ----------------yftgSAHFNRSmralaktkgmslsehalstavvrnthgakvgpg  299
DSSP  ----------------hhhlLHHHHHHhhhhhhhllleellllleeelleellllleeell


DSSP  HHHHhhHHHHHhhhhhhllllLLLLLlllllhhhhhhhhhllllllllllhhhhhhhhh
Query ADLXptRSACIaifearaqhpWPPRVatlphwpliyagaleglдhlelartvdaaaqavq  280
ident    |
Sbjct RVLP--TPTEK-------dvfRLLGL----------------------------------  316
DSSP  EELL--LLLHH-------hhhHHLLL----------------------------------


DSSP  hhhhhhhhllll
Query rfvaridratkr  292
ident
Sbjct --pyrepaerdw  326
DSSP  --llllhhhhll
```

No 349: Query=mol1A Sbjct=2pfqA Z-score=6.4

back to top

```
DSSP  ------------------------------------------------------------
Query ------------------------------------------------------------    0
ident
Sbjct hnlhiteklevlakaysvqgdkwralgyakainalksfhkpvtsyqeacsipgigkrmae   60
DSSP  llllhhhhhhhhhhhhhlllhhhhhhhhhhhhhhlllllllllhhhhhhlllllhhhhh


DSSP  -------------------------------------------lllllllllhhhhhhh
Query -------------------------------------------aapysspptnlrslrdr   17
ident
Sbjct kiieilesghlrkldhisesvpvlelfsniwgagtktaqmwyqqgfrsledirsqasltt  120
DSSP  hhhhhhhllllhhhhlllllhhhhhhhhhllllllhhhhhhhhhlllllhhhhhhhllllh


DSSP  hhhhhhhllllhhhhHHHHHHHHHHHHHHHLL-LLLLLLLLEEEEHHHHHHHhhllllllL
Query ltqvaerqgvvfgrlQRHVAXIVVAQFAATL-TDDTGAPLLLVKGGSSLELrrgipdsrT   76
ident                         |                |     |            |
Sbjct qqaiglkhysdflerMPREEATEIEQTVQKAaQAFNSGLLCVACGSYRRGK-------aT  173
DSSP  hhhhhhhlllhhhllEEHHHHHHHHHHHHHHHhHLLLLLLEEEELHHHHLLL-------lE


DSSP  LLLEEEEEL--------lLHHHHHHHHHHHHhhllllleeeEELLLEELlllllLLLLEEE
Query SKDFATVAR--------rDIELIHEQLADAGetgwegftaIFTAPEEIaapgxPVKPRRF  128
ident    |                          |   |           |
Sbjct CGDVDVLIThpdgrshrgIFSRLLDSLRQEG---------FLTDDLVSqeengQQQKYLG  224
DSSP  ELEEEEEEElllllllllLHHHHHHHHHHLL---------LEEEEEELlllllLLLEEEE


DSSP  EEEEEEllEEEEEEEEEEEEL-LLLLllllleeeeelhhhhlllllleeeeellhHHHHhh
Query TAKLSYrgRAFATVPIEVSSV-EAGNadqfdtltsdalglvgvpaavavpcxtiPWQIaq  187
ident     |                 |
Sbjct VCRLPG--PGRRHRRLDIIVVpYSEF---------------------------aCALL--  253
DSSP  EELLLL--LLLLLEEEEEEEELlHHHH---------------------------hHHHH--


DSSP  hhhhhhllllllllllllHHHHHHH-------------------------hhhhhllhHH
```

```
ident
Sbjct --------------yftgSAHFNRSmralaktkgmslsehalstavvrnthgakvgpgRV  299
DSSP  --------------hhhlLHHHHHHhhhhhhhlleeellllleeelleellllleeellEE


DSSP  HHhhHHHHHhhhhhhllllLLLLLlllllhhhhhhhhhllllllllllhhhhhhhhhh
Query LXptRSACIaifearaqhpWPPRVatlphwpliyagalegldhlelartvdaaaqavqrf  282
ident |
Sbjct LP--TPTEK------dvfrLLGLP------------------------------------  315
DSSP  EL--LLLHH------hhhhHLLLL------------------------------------


DSSP  hhhhhhllll
Query varidratkr  292
ident
Sbjct -yrepaerdw  324
DSSP  -lllhhhhll
```

No 350: Query=mol1A Sbjct=5ddyG Z-score=6.4

back to top

```
DSSP  ------------------------lllllllllhhhhhhhhhhhhhhllllHHHHhHHH
Query ------------------------aapyssspptnlrslrdrltqvaerqgvvFGRLqRHV   36
ident                                                       |
Sbjct vpvlelfsniwgagtktaqmwyqqgfrsledirsqasltttqqaiglkhysdfLERM-PRE   59
DSSP  lhhhhhhllllllhhhhhhhhhhllllhhhhhhlllllhhhhhhhhlhhhhLLLL-LLH


DSSP  HHHHHHHHHHL-LLLLlLLLLEEEEHHHHHHhhhllllLLLLLLEEEEEL--------lL
Query AXIVVAQFAAT-LTDDtGAPLLLVKGGSSLElrrgipdSRTSKDFATVAR--------rD   87
ident       |             |    |              |  |
Sbjct EATEIEQTVQKaAQAFnSGLLCVACGSYRRG-------KATCGDVDVLIThpdgrshrgI  112
DSSP  HHHHHHHHHHHhHHHHlLLLEEEELLLLLL-------LLLLLLEEEEEElllllllllL


DSSP  HHHHHHHHHHHHhhlllleeEEELLLE-ELLLlLLLLLEEEEEEEEEllEEEEEEEEEE
Query IELIHEQLADAGetgwegftAIFTAPE-EIAApgXPVKPRRFTAKLSYrgRAFATVPIEV  146
ident        |   |                                 |
Sbjct FSRLLDSLRQEG--------FLTDDLVsQEEN--GQQQKYLGVCRLPG--PGRRHRRLDI  160
DSSP  HHHHHHHHHHLL--------LEEEEEElLLEE--LLEEEEEEEEELLL--LLLLEEEEEE


DSSP  EEL-LLLLllllleeeeelhhhhlllllleeeeellhHHHHhhhhhhhhllllllllllll
Query SSV-EAGNadqfdtltsdalglvgvpaavavpcxtiPWQIaqklhavtavleepkandra  205
ident   |
Sbjct IVVpYSEF---------------------------aCALL----------------yftg  177
DSSP  EELlLLLH---------------------------hHHHH----------------hhhl


DSSP  HHHHHHhhhhhllhhhhhhhhhhhhHHHH---------------------hhlllllLL
Query HDLVDLQllegllldadlxptrsaciAIFE---------------------araqhpwPP  244
ident
Sbjct SAHFNRS-----mralaktkgmslseHALStavvrnthgckvgpgrvlptptekdvfrLL  232
DSSP  LHHHHHH-----hhhhhhllleeellLLEEellllllllllllleellllhhhhhhLL


DSSP  LLLllllhhhhhhhhhllllllllllhhhhhhhhhhhhhhhhhllll
Query RVAtlphwpliyagalegldhlelartvdaaaqavqrfvaridratkr  292
ident
Sbjct GLP------------------------------------yrepaerdw  244
DSSP  LLL------------------------------------lllhhhhll
```

No 351: Query=mol1A Sbjct=2bcvA Z-score=6.4

back to top

```
DSSP  ------------------------------------------------------------
Query ------------------------------------------------------------    0
ident
Sbjct nlhiteklevlakaysvqgdkwralgyakainalksfhkpvtsyqeacsipgigkrmaek   60
DSSP  lllllhhhhhhhhhhhlllhhhhhhhhhhhhhhhhlllllllhhhhhlllllhhhhhh
```

```
DSSP  ------------------------------------------lllllllllhhhhhhhhh
Query ------------------------------------------aapyssspptnlrslrdrl   18
ident
Sbjct iieilesghlrkldhisesvpvlelfsniwgagtktaqmwyqqgfrsledirsqaslttq  120
DSSP  hhhhhhllllhhhhhllllhhhhhhhhlllllhhhhhhhhhllllhhhhhhhllllhh


DSSP  hhhhhhllllhhHHHHHHHHHHHHHHHHHLL-LLLLLLLLEEEEHHHHHHHhhllllllLL
Query tqvaerqgvvfgRLQRHVAXIVVAQFAATL-TDDTGAPLLLVKGGSSLELrrgipdsrTS   77
ident                         |             |    |              |
Sbjct qaiglkhysdflERMPREEATEIEQTVQKAaQAFNSGLLCVACGSYRRGK-------aTC  173
DSSP  hhhhhhlllhhhLLEEHHHHHHHHHHHHHHHHhHHHLLLLEEEELHHHHLLL-------lEE


DSSP  LLEEEEEL--------lLHHHHHHHHHHHHhhlllleeeEELLLEELLllllLLLLEEEE
Query KDFATVAR--------rDIELIHEQLADAGetgwegftaIFTAPEEIAapgxPVKPRRFT  129
ident  |                       |   |           |
Sbjct GDVDVLIThpdgrshrgIFSRLLDSLRQEG---------FLTDDLVSQeengQQQKYLGV  224
DSSP  LEEEEEEElllllllllLHHHHHHHHHHLL---------LEEEEEELLllllLLLEEEEE


DSSP  EEEEEllEEEEEEEEEEEELLLLLllllleeeeelhhhhllllleeeeellhHHHHhhhh
Query AKLSYrgRAFATVPIEVSSVEAGNadqfdtltsdalglvgvpaavavpcxtiPWQIaqkl  189
ident   |                |
Sbjct CRLPG--PGRRHRRLDIIVVPYSE--------------------------faCALL----  252
DSSP  ELLLL--LLLLLLEEEEEEEELLHHH--------------------------hhHHHH----


DSSP  hhhhllllllllllllHHHHHHH-------------------------hhhhhllhHHHH
Query havtavleepkandraHDLVDLQ-------------------------lleglldADLX  224
ident                                                           |
Sbjct ------------yftgSAHFNRSmralaktkgmslsehalstavvrnthgckvgpgRVLP  300
DSSP  ------------hhhlLHHHHHHhhhhhhlllleellllleeelleellllleeellEELL


DSSP  hhHHHHHhhhhhhllllLLLLLlllllhhhhhhhhhllllllllllhhhhhhhhhhhh
Query ptRSACIaifearaqhpWPPRVatlphwpliyagalegldhlelartvdaaaqavqrfva  284
ident
Sbjct --TPTEK-------dvfRLLGL------------------------------------py  315
DSSP  --LLLHH-------hhhHHLLL------------------------------------ll


DSSP  hhhhllll
Query ridratkr  292
ident
Sbjct repaerdw  323
DSSP  llhhhhll
```

No 352: Query=mol1A Sbjct=2bcqA Z-score=6.4

back to top

```
DSSP  ------------------------------------------------------------
Query ------------------------------------------------------------    0
ident
Sbjct hnlhiteklevlakaysvqgdkwralgyakainalksfhkpvtsyqeacsipgigkrmae   60
DSSP  llhhhhhhhhhhhhhhhhlllhhhhhhhhhhhhhhhllllllllhhhhhlllllllhhhhh


DSSP  -------------------------------------------lllllllllhhhhhhh
Query -------------------------------------------aapyssspptnlrslrdr   17
ident
Sbjct kiieilesghlrkldhisesvpvlelfsniwgagtktaqmwyqqgfrsledirsqaslttt  120
DSSP  hhhhhhhllllhhhhhllllhhhhhhhhlllllhhhhhhhhhllllhhhhhhhllllh


DSSP  hhhhhhhllllhhHHHHHHHHHHHHHHHHHLL-LLLLLLLLEEEEHHHHHHHhhllllllL
Query ltqvaerqgvvfgRLQRHVAXIVVAQFAATL-TDDTGAPLLLVKGGSSLELrrgipdsrT   76
ident                          |             |    |              |
Sbjct qqaiglkhysdflERMPREEATEIEQTVQKAaQAFNSGLLCVACGSYRRGK-------aT  173
DSSP  hhhhhhhlllhhhLLEEHHHHHHHHHHHHHHHHhHLLLLLLLEEEELHHHHLLL-------lE


DSSP  LLLEEEEEL--------lLHHHHHHHHHHHHhhlllleeeEELLLEELLllllLLLLEEE
Query SKDFATVAR--------rDIELIHEQLADAGetgwegftaIFTAPEEIAapgxPVKPRRF  128
ident   |                       |   |           |
```

```
DSSP  ELLEEEEEEElllllllllLHHHHHHHHHHLL---------LEEEEEEELLlllllLLLEEEE


DSSP  EEEEEEllEEEEEEEEEEEEELLLLLlllleeeeelhhhhllllllleeeeellhHHHHhhh
Query TAKLSYrgRAFATVPIEVSSVEAGNadqfdtltsdalglvgvpaavavpcxtiPWQIaqk  188
ident    |                |
Sbjct VCRLPG--PGRRHRRLDIIVVPYSE--------------------------faCALL---  253
DSSP  EELLLL--LLLLLEEEEEEEELLHHH--------------------------hhHHHH---


DSSP  hhhhhlllllllllllHHHHHHH-------------------------hhhhhllhHHH
Query lhavtavleepkandraHDLVDLQ-------------------------lleglllDADL  223
ident                                                            |
Sbjct -------------yftgSAHFNRSmralaktkgmslsehalstavvrnthgckvgpgRVL  300
DSSP  -------------hhhlLHHHHHHhhhhhhllleellllleeelleelllllleeellEEL


DSSP  HhhHHHHHhhhhhhllllLLLLLlllllhhhhhhhhhlllllllllllhhhhhhhhhhh
Query XptRSACIaifearaqhpWPPRVatlphwpliyagalegldhlelartvdaaaqavqrfv  283
ident
Sbjct P--TPTEK-------dvfRLLGL------------------------------------p  315
DSSP  L--LLLHH-------hhhHHLLL------------------------------------l


DSSP  hhhhhllll
Query aridratkr  292
ident
Sbjct yrepaerdw  324
DSSP  lllhhhhll
```

No 353: Query=mol1A Sbjct=3hx0F Z-score=6.4

back to top

```
DSSP  ------------------------------------------------------------
Query ------------------------------------------------------------    0
ident
Sbjct qkatnhnlhiteklevlakaysvqgdkwraagyakainalksfhkpvtsyqeacsipgig   60
DSSP  llllllhhhhhhhhhhhhhhhhhhllhhhhhhhhhhhhhhhhllllllllhhhhlllllll


DSSP  ------------------------------------------------llllllllllhh
Query ------------------------------------------------aapysspptnlr   12
ident
Sbjct krmaekiieilesghlrkldhisesvpvlelfsniwgagtktaqmwyqqgfrsledirsq  120
DSSP  hhhhhhhhhhhhhhlllhhhhllllhhhhhhhhhlllllllhhhhhhhhhlllllhhhhhhl


DSSP  hhhhhhhhhhhhllllhHHHHHHHHHHHHHHHHHHL-LLLLLLLLLEEEEHHHHHHHHhhll
Query slrdrltqvaerqgvvfGRLQRHVAXIVVAQFAAT-LTDDTGAPLLLVKGGSSLELrrgi   71
ident                               |             |    |
Sbjct asIttqqaiglkhysdfLERMPREEATEIEQTVQKaAQAFNSGLLCVACGSYRRGK----  176
DSSP  llllhhhhhhhhhlhhhhHLLEEHHHHHHHHHHHHHHHhHHLLLLLLLEEEELHHHHLLL----


DSSP  llllLLLLEEEEEL--------lLHHHHHHHHHHHHHhhllllleeEEELLLEELlllllLL
Query pdsrTSKDFATVAR--------rDIELIHEQLADAGetgwegftAIFTAPEEIaapgxPV  123
ident     |  |                       |   |
Sbjct ---aTCGDVDVLIThpdgrshrgIFSRLLDSLRQEG--------FLTDDLVSQ-eengQQ  224
DSSP  ---lEELEEEEEEEllllllllLHHHHHHHHHHHLL--------LEEEEEELL-llllLL


DSSP  LLEEEEEEEEEllEEEEEEEEEEEEL-LLLLlllleeeeelhhhhllllllleeeeellhH
Query KPRRFTAKLSYrgRAFATVPIEVSSV-EAGNadqfdtltsdalglvgvpaavavpcxtiP  182
ident         |                |
Sbjct QKYLGVCRLPG--PGRRHRRLDIIVVpYSEF---------------------------aC  255
DSSP  LEEEEEELLLL--LLLLLEEEEEEEELlHHHH---------------------------hH


DSSP  HHHhhhhhhhhlllllllllllHHHHHHH-------------------------hhhhh
Query WQIaqklhavtavleepkandraHDLVDLQ-------------------------llegl  217
ident
Sbjct ALL----------------yftgSAAFNASmralaktkgmslsehalstavvrnthgakv  299
DSSP  HHH----------------hhhlLHHHHHHhhhhhhllleellllleelllllllllll
```

```
Query lldADLXptRSACIaifearaqhpWPPRVatlphwpliyagalegldhlelartvdaaaq  277
ident      |
Sbjct gpgRVLPT--PTEK-----dvfrLLGLPY-------------------------------  321
DSSP  lllLLLLL--LLHH-----hhhhLLLLLL-------------------------------


DSSP  hhhhhhhhhhhllll
Query avqrfvaridratkr  292
ident
Sbjct -------repaerdw  329
DSSP  -------llhhhlll
```

No 354: Query=mol1A Sbjct=3hx0A Z-score=6.4

back to top

```
DSSP  ------------------------------------------------------------
Query ------------------------------------------------------------    0
ident
Sbjct sqkatnhnlhiteklevlakaysvqgdkwraagyakainalksfhkpvtsyqeacsipgi   60
DSSP  lllllllhhhhhhhhhhhhhhhhlllhhhhhhhhhhhhhhhlllllllhhhhhlllll


DSSP  -------------------------------------------------lllllllllh
Query -------------------------------------------------aapysspptnl   11
ident
Sbjct gkrmaekiieilesghlrkldhisesvpvlelfsniwgagtktaqmwyqqgfrsledirs  120
DSSP  lhhhhhhhhhhhhhllllhhhhllllhhhhhhhhlllllhhhhhhhhhlllllhhhhhh


DSSP  hhhhhhhhhhhhlllllhHHHHHHHHHHHHHHHHHHHLL-LLLLLLLLEEEEHHHHHHHHhhl
Query rslrdrltqvaerqgvvfGRLQRHVAXIVVAQFAATL-TDDTGAPLLLVKGGSSLELrrg   70
ident                                |             |    |
Sbjct qaslttqqaiglkhysdfLERMPREEATEIEQTVQKAaQAFNSGLLCVACGSYRRGK---  177
DSSP  hllllhhhhhhhhlhhhhHLLEEHHHHHHHHHHHHHHHHhHHHLLLLEEEELHHHHLLL---


DSSP  lllllLLLLEEEEEL--------lLHHHHHHHHHHHHhhllllleeEEELLLEELlllllL
Query ipdsrTSKDFATVAR--------rDIELIHEQLADAGetgwegftAIFTAPEEIaapgxP  122
ident      |  |                       |   |
Sbjct ----aTCGDVDVLIThpdgrshrgIFSRLLDSLRQEG--------FLTDDLVSQ-eengQ  224
DSSP  ----lEELEEEEEEElllllllllLHHHHHHHHHHHL--------LLLEEEELL-llllL


DSSP  LLLEEEEEEEEEllEEEEEEEEEEEEL-LLLLllllleeeeelhhhhllllllleeeeellh
Query VKPRRFTAKLSYrgRAFATVPIEVSSV-EAGNadqfdtltsdalglvgvpaavavpcxti  181
ident          |                |
Sbjct QQKYLGVCRLPG--PGRRHRRLDIIVVpYSEF---------------------------a  255
DSSP  LLEEEEEELLLL--LLLLLEEEEEEELlLLLH---------------------------h


DSSP  HHHHhhhhhhhhhlllllllllllHHHHHHH-------------------------hhhh
Query PWQIaqklhavtavleepkandraHDLVDLQ-------------------------lleg  216
ident
Sbjct CALL----------------yftgSAAFNASmralaktkgmslsehalstavvrnthgak  299
DSSP  HHHH----------------hhhlLHHHHHHhhhhhllllleellllllleelleellllle


DSSP  hllhHHHHhhHHHHHhhhhhhllllLLLLLllllhhhhhhhhhllllllllllhhhhh
Query llldADLXptRSACIaifearaqhpWPPRVatlphwpliyagalegldhlelartvdaaa  276
ident       |
Sbjct vgpgRVLP--TPTEK-------dvfRLLGL------------------------------  320
DSSP  eellLLLL--LLLHH-------hhhHHHLL------------------------------


DSSP  hhhhhhhhhhhhllll
Query qavqrfvaridratkr  292
ident
Sbjct ------pyrepaerdw  330
DSSP  ------llllhhhlll
```

No 355: Query=mol1A Sbjct=5cb1A Z-score=6.4

```
DSSP  llllllllllhhhhhhhhhhhhHHLL----------------------------------
Query aapysspptnlrslrdrltqvaERQG----------------------------------   26
ident
Sbjct tnhnlhiteklevlakaysvqgDKWRalgyakainalksfhkpvtsyqeacsipgigkrm   60
DSSP  llllhhhhhhhhhhhhhhhhllLHHHhhhhhhhhhhhhllllllllhhhhhllllllhhh


DSSP  ------------------------------------------------------------
Query ------------------------------------------------------------   26
ident
Sbjct aekiieilesghlrkldhisesvpvlelfsniwgagtktaqmwyqqgfrsledirsqasl  120
DSSP  hhhhhhhhhhllllllllhhhhhhhhhhhhlllllllhhhhhhhhhhllllhhhhhhhlll


DSSP  -----------llhHHHHHHHHHHHHHHHHHHHLL-LLLLLLLLEEEEHHHHHHHhhlllll
Query -----------vvfGRLQRHVAXIVVAQFAATL-TDDTGAPLLLVKGGSSLELrrgipds   74
ident                            |             |    |
Sbjct ttqqaiglkhysdfLERMPREEATEIEQTVQKAaQAFNSGLLCVACGSYRRGK-------  173
DSSP  lhhhhhhhhlhhhhHLLEEHHHHHHHHHHHHHHHHhHHHLLLLEEEEEELLLLLLL-------


DSSP  lLLLLEEEEEL--------lLHHHHHHHHHHHHhhlllleeeEELL-LEELLLLLlLLLL
Query rTSKDFATVAR--------rDIELIHEQLADAGetgwegftaIFTA-PEEIAAPGxPVKP  125
ident  |  |                       |   |           |         |
Sbjct aTCGDVDVLIThpdgrshrgIFSRLLDSLRQEG---------FLTDdLVSQEENG-QQQK  223
DSSP  lEELLEEEEEEllllllllHHHHHHHHHHHHHL---------LEELlLLLEEEEL-LEEE


DSSP  EEEEEEEEEllEEEEEEEEEEEEEL-LLLLllllleeeeelhhhhllllllleeeeellhHHH
Query RRFTAKLSYrgRAFATVPIEVSSV-EAGNadqfdtltsdalglvgvpaavavpcxtiPWQ  184
ident       |                |
Sbjct YLGVCRLPG--PGRRHRRLDIIVVpYSEF---------------------------aCAL  254
DSSP  EEEEELLLL--LLLLLEEEEEEEEL1HHHH---------------------------hHHH


DSSP  HhhhhhhhhllllllllllllHHHHHHH------------------------hhhhhllhh
Query IaqklhavtavleepkandraHDLVDLQ------------------------llegllda  221
ident
Sbjct L----------------yftgSAHFNRSmralaktkgmslsehalstavvrnthgckvgp  298
DSSP  H----------------hhhlLHHHHHhhhhhhlleeelllllleeelleellllllleeel


DSSP  hhhhhHHHHhhhhhhllllLLLLLlllllhhhhhhhhhlllllllllllhhhhhhhhhh
Query dlxptRSACIaifearaqhpWPPRVatlphwpliyagalegldhlelartvdaaaqavqr  281
ident
Sbjct grvlpTPTEK-------dvfRLLGL-----------------------------------  316
DSSP  lllllLLLHH-------hhhHHLLL-----------------------------------


DSSP  hhhhhhhllll
Query fvaridratkr  292
ident
Sbjct -pyrepaerdw  326
DSSP  -llllhhhhll
```

No 356: Query=mol1A Sbjct=4k4iE Z-score=6.4

back to top

```
DSSP  ------------------------------------------------------------
Query ------------------------------------------------------------    0
ident
Sbjct atnhnlhiteklevlakaysvqgdkwralgyakainalksfhkpvtsyqeacsipgigkr   60
DSSP  llllllhhhhhhhhhhhhhhhhlllhhhhhhhhhhhhhhhhhlllllllhhhhhllllllhh


DSSP  ----------------------------------------------llllllllllhhhh
Query ----------------------------------------------aapysspptnlrsl   14
ident
Sbjct maekiieilesghlrkldhisesvpvlelfsniwgagtktaqmwyqqgfrsledirsqas  120
DSSP  hhhhhhhhhhhlllhhhhhllllhhhhhhhhllllllhhhhhhhhhhllllllhhhhhhhll


DSSP  hhhhhhhhhhllllhHHHHHHHHHHHHHHHHHHHLL-LLLLLLLLEEEEHHHHHHHhhllll
Query rdrltqvaerqgvvfGRLQRHVAXIVVAQFAATL-TDDTGAPLLLVKGGSSLELrrgipd   73
```

```
Sbjct lttqqaiglkhysdfLERMPREEATEIEQTVQKAaQAFNSGLLCVACGSYRRGK------  174
DSSP  llhhhhhhhlhhhhHLLEEHHHHHHHHHHHHHHHhHLLLLLLEEEELHHHHLLL------


DSSP  llLLLLEEEEEL--------lLHHHHHHHHHHHHhhllllleeEEELLLEELlLLLLlLLL
Query srTSKDFATVAR--------rDIELIHEQLADAGetgwegftAIFTAPEEIaAPGXpVKP  125
ident   |  |                       |   |                    |
Sbjct -aTCGDVDVLIThpdgrshrgIFSRLLDSLRQEG--------FLTDDLVSQeENGQ-QQK  224
DSSP  -lEELLEEEEEEllllllllLHHHHHHHHHHLL--------LEEEEEEEElLLLL-LEE


DSSP  EEEEEEEEEllEEEEEEEEEEEEL-LLLLllllleeeeelhhhhllllleeeeellhHHH
Query RRFTAKLSYrgRAFATVPIEVSSV-EAGNadqfdtltsdalglvgvpaavavpcxtiPWQ  184
ident       |                |
Sbjct YLGVCRLPG--PGRRHRRLDIIVVpYSEF---------------------------aCAL  255
DSSP  EEEEELLLL--LLLLLEEEEEEEELlHHHH---------------------------hHHH


DSSP  HhhhhhhhhlllllllllllHHHHHHH-----------------------hhhhhllhh
Query IaqklhavtavleepkandraHDLVDLQ-----------------------llegllda  221
ident
Sbjct L----------------yftgSAHFNRSmralaktkgmslsehalstavvrnthgckvgp  299
DSSP  H----------------hhhlLHHHHHHhhhhhhhllleellleellllllllllll


DSSP  hhhhhHHHHHhhhhhhllllLLLLLlllllhhhhhhhhhllllllllllhhhhhhhhh
Query dlxptRSACIaifearaqhpWPPRVatlphwpliyagalegldhlelartvdaaaqavqr  281
ident
Sbjct grvlpTPTEK-------dvfRLLGL----------------------------------p  318
DSSP  lllllLLLHH-------hhhHHHLL----------------------------------l


DSSP  hhhhhhhllll
Query fvaridratkr  292
ident
Sbjct yrepaerdwle  329
DSSP  lllhhhlllll
```

No 357: Query=mol1A Sbjct=5dkwA Z-score=6.4

back to top

```
DSSP  ------------------------------------------------------------
Query ------------------------------------------------------------    0
ident
Sbjct atnhnlhiteklevlakaysvqgdkwralgyakainalksfhkpvtsyqeacsipgigkr   60
DSSP  llllhhhhhhhhhhhhhhhhlllhhhhhhhhhhhhhhhhlllllllhhhhhhllllllhh


DSSP  ----------------------------------------------llllllllllhhhh
Query ----------------------------------------------aapysspptnlrsl   14
ident
Sbjct maekiieilesghlrkldhisesvpvlelfsniwgagtktaqmwyqqgfrsledirsqas  120
DSSP  hhhhhhhhhhhlllhhhhlllllhhhhhhhhllllllhhhhhhhhhlllllhhhhhhhll


DSSP  hhhhhhhhhhllllhHHHHHHHHHHHHHHHHHHHLL-LLLLLLLLEEEEHHHHHHHhhllll
Query rdrltqvaerqgvvfGRLQRHVAXIVVAQFAATL-TDDTGAPLLLVKGGSSLELrrgipd   73
ident                             |             |    |
Sbjct lttqqaiglkhysdfLERMPREEATEIEQTVQKAaQAFNSGLLCVACGSYRRGK------  174
DSSP  llhhhhhhhlhhhhHLLEEHHHHHHHHHHHHHHHhLLLLLLLEEEELHHHLLLL------


DSSP  llLLLLEEEEEL--------lLHHHHHHHHHHHHhhllllleeeEELLL-EELLLLLlLLL
Query srTSKDFATVAR--------rDIELIHEQLADAGetgwegftaIFTAP-EEIAAPGxPVK  124
ident   |  |                       |   |           |         |
Sbjct -aTCGDVDVLIThpdgrshrgIFSRLLDSLRQEG---------FLTDDlVSQEENG-QQQ  223
DSSP  -lEELLEEEEEEllllllllLHHHHHHHHHHLL---------LEEEEeEEEEEEL-LEE


DSSP  LEEEEEEEEEllEEEEEEEEEEEEL-LLLLllllleeeeelhhhhllllleeeeellhHH
Query PRRFTAKLSYrgRAFATVPIEVSSV-EAGNadqfdtltsdalglvgvpaavavpcxtiPW  183
ident        |                |
Sbjct KYLGVCRLPG--PGRRHRRLDIIVVpYSEF---------------------------aCA  254
DSSP  EEEEEELLLL--LLLLLEEEEEEEELlHHHH---------------------------hHH
```

```
DSSP  HHhhhhhhhhllllllllllllHHHHHHH-----------------hhhhhllhHHHHhh
Query QIaqklhavtavleepkandraHDLVDLQ-----------------lleglllldADLXpt  226
ident                                                       |
Sbjct LL----------------yftgSAHFNRSmralaktkgmslsehalstavvgpgRVLP--  296
DSSP  HH----------------hhhlLHHHHHHhhhhhhhlleeellllleeellllllLEEL--


DSSP  HHHHhhhhhhllllLLLLLLllllhhhhhhhhhllllllllllhhhhhhhhhhhhhh
Query RSACIaifearaqhpWPPRVAtlphwpliyagalegldhlelartvdaaaqavqrfvari  286
ident
Sbjct TPTEK-------dvfRLLGLP------------------------------------yre  313
DSSP  LLLHH-------hhhHHLLLL------------------------------------lll


DSSP  hhllll
Query dratkr  292
ident
Sbjct paerdw  319
DSSP  hhhlll
```

No 358: Query=mol1A Sbjct=5iilA Z-score=6.4

back to top

```
DSSP  ------------------------------------------------------------
Query ------------------------------------------------------------    0
ident
Sbjct tnhnlhitekleevlakaysvqgdkwralgyakainalksfhkpvtsyqeacsipgigkrm   60
DSSP  llllhhhhhhhhhhhhhhhhlllhhhhhhhhhhhhhhhhllllllllhhhhhhlllllhhh


DSSP  ---------------------------------------------llllllllllhhhhh
Query ---------------------------------------------aapysspptnlrslr   15
ident
Sbjct aekiieilesghlrkldhisesvpvlelfsniwgagtktaqmwyqqgfrsledirsqasl  120
DSSP  hhhhhhhhhhlllhhhhlllllhhhhhhhhlllllllhhhhhhhhhlllllhhhhhhhlll


DSSP  hhhhhhhhhllllhhHHHHHHHHHHHHHHHHHHLL-LLLLLLLLEEEEHHHHHHHHhhlllll
Query drltqvaerqgvvfgRLQRHVAXIVVAQFAATL-TDDTGAPLLLVKGGSSLELrrgipds   74
ident                           |             |    |
Sbjct ttqqaiglkhysdflERMPREEATEIEQTVQKAaQAFNSGLLCVACGSYRRGK-------  173
DSSP  lhhhhhhhhhlllhhhLLEEHHHHHHHHHHHHHHHhHHHLLLLEEEELHHHHLLL-------


DSSP  lLLLLEEEEEL--------lLHHHHHHHHHHHHHhhlllleeeEELLLEELLllllLLLLE
Query rTSKDFATVAR--------rDIELIHEQLADAGetgwegftaIFTAPEEIAapgxPVKPR  126
ident  |  |                        |   |           |
Sbjct aTCGDVDVLIThpdgrshrgIFSRLLDSLRQEG---------FLTDDLVSQeengQQQKY  224
DSSP  lEELEEEEEEEllllllllHHHHHHHHHHHHLL---------LEEEEEEELllllLLLEE


DSSP  EEEEEEEEllEEEEEEEEEEEEELLLLLLlllleeeeelhhhhlllllleeeeellhHHHHh
Query RFTAKLSYrgRAFATVPIEVSSVEAGNAdqfdtltsdalglvgvpaavavpcxtiPWQIa  186
ident       |                |
Sbjct LGVCRLPG--PGRRHRRLDIIVVPYSEF--------------------------aCALL-  255
DSSP  EEEELLLL--LLLLLEEEEEEEELLHHHH--------------------------hHHHH-


DSSP  hhhhhhhllllllllllllHHHHHHH-------------------------hhhhhllhH
Query qklhavtavleepkandraHDLVDLQ-------------------------llegllldA  221
ident
Sbjct ---------------yftgSAHFNRSmralaktkgmslsehalstavvrnthgckvgpgR  300
DSSP  ---------------hhhlLHHHHHHhhhhhhhllleellllleeelleellllleeellE


DSSP  HHHhhHHHHHhhhhhhllllLLLLLLllllhhhhhhhhhllllllllllhhhhhhhhhh
Query DLXptRSACIaifearaqhpWPPRVAtlphwpliyagalegldhlelartvdaaaqavqr  281
ident  |
Sbjct VLP--TPTEK-------dvfRLLGLP----------------------------------  317
DSSP  EEL--LLLHH-------hhhHHLLLL----------------------------------


DSSP  hhhhhhhhllll
Query fvaridratkr  292
ident
```

```
DSSP  lllhhhlll
```

No 359: Query=mol1A Sbjct=5iikA Z-score=6.4

[back to top](#top)

```
DSSP  ------------------------------------------------------------
Query ------------------------------------------------------------    0
ident
Sbjct nhnlhiteklevlakaysvqgdkwralgyakainalksfhkpvtsyqeacsipgigkrma   60
DSSP  lllhhhhhhhhhhhhhhhhlllhhhhhhhhhhhhhhhlllllllllhhhhhhlllllhhhh


DSSP  -------------------------------------------llllllllllhhhhhh
Query -------------------------------------------aapysspptnlrslrd   16
ident
Sbjct ekiieilesghlrkldhisesvpvlelfsniwgagtktaqmwyqqgfrsledirsqaslt  120
DSSP  hhhhhhhhhlllhhhhhllllhhhhhhhhlllllhhhhhhhhhlllllhhhhhhhllll


DSSP  hhhhhhhhllllhhHHHHHHHHHHHHHHHHHLL-LLLLLLLLEEEEHHHHHHHhhllllll
Query rltqvaerqgvvfgRLQRHVAXIVVAQFAATL-TDDTGAPLLLVKGGSSLELrrgipdsr   75
ident                           |             |    |
Sbjct tqqaiglkhysdflERMPREEATEIEQTVQKAaQAFNSGLLCVACGSYRRGK-------a  173
DSSP  hhhhhhhhlllhhhLLEEHHHHHHHHHHHHHHHhHHHLLLLEEEELHHHHLLL-------l


DSSP  LLLLEEEEEL--------lLHHHHHHHHHHHHhhlllleeeEELLLEELLllllLLLLEE
Query TSKDFATVAR--------rDIELIHEQLADAGetgwegftaIFTAPEEIAapgxPVKPRR  127
ident |  |                       |   |           |
Sbjct TCGDVDVLIThpdgrshrgIFSRLLDSLRQEG---------FLTDDLVSQeengQQQKYL  224
DSSP  EELLEEEEEEllllllllHHHHHHHHHHHHLL---------LEEEEEELLllllLLLEEE


DSSP  EEEEEEEllEEEEEEEEEEEELLLLLLllleeeeelhhhhlllllleeeeellhHHHHhh
Query FTAKLSYrgRAFATVPIEVSSVEAGNAdqfdtltsdalglvgvpaavavpcxtiPWQIaq  187
ident     |                |
Sbjct GVCRLPG--PGRRHRRLDIIVVPYSEF--------------------------aCALL--  254
DSSP  EEELLLL--LLLLLEEEEEEEELLHHHH--------------------------hHHHH--


DSSP  hhhhhhllllllllllllHHHHHHH-------------------------hhhhhllhHH
Query klhavtavleepkandraHDLVDLQ-------------------------lleglllldAD  222
ident
Sbjct --------------yftgSAHFNRSmralaktkgmslsehalstavvrnthgckvgpgRV  300
DSSP  --------------hhhlLHHHHHHhhhhhhhllleellllleeelleellllleeellEE


DSSP  HHhhHHHHHhhhhhhllllLLLLLLllllhhhhhhhhhllllllllllhhhhhhhhhh
Query LXptRSACIaifearaqhpWPPRVAtlphwpliyagalegldhlelartvdaaaqavqrf  282
ident |
Sbjct LP--TPTEK-------dvfRLLGLP------------------------------------  316
DSSP  EL--LLLHH-------hhhHHLLLL------------------------------------


DSSP  hhhhhhllll
Query varidratkr  292
ident
Sbjct -yrepaerdw  325
DSSP  -lllhhhlll
```

No 360: Query=mol1A Sbjct=4fh3A Z-score=6.3

[back to top](#top)

```
DSSP  llllllllllhhhhhhhhhhhhhhhllLLHHHHHHHHHHHHHHHHHHHHLLLlllllLLEEEE
Query aapysspptnlrslrdrltqvaerqgVVFGRLQRHVAXIVVAQFAATLTddtgaPLLLVK   60
ident                                     |                 |
Sbjct --------shkeftkfcyevyneikiSDKEFKEKRAALDTLRLCLKRIS-----PDAELV   47
DSSP  --------llhhhhhhhhhhhhhhllLHHHHHHHHHHHHHHHHHHHHHLLL-----LLLEEE


DSSP  HHhhhhhhhllllllLLLLEEEEEL--LLHHHHHHHHHHHHHHHL111eEEEELLLEEL11
```

```
ident                   |             |  |
Sbjct AF--gslesglalknSDMDLCVLMDsrVQSDTIALQFYEELIAE----GFEGKFLQRA--   99
DSSP  EE--lhhhhlllllLLEEEEEELLllLLHHHHHHHHHHHHHHL----LEEEEEEEEL--


DSSP  lllllLLEEEEEEEEEELLE--EEEEEEEEEEelllllllllleeeeelhhhhlllllleee
Query pgxpvKPRRFTAKLSYRGR--AFATVPIEVSsveagnadqfdtltsdalglvgvpaavav  176
ident                      |     |
Sbjct -----RIPIIKLTSDTKNGfgASFQCDIGFN-----------------------------  125
DSSP  -----LEEEEEEEEELLLLllLLLLEEEEEL-----------------------------


DSSP  eellhhhhhhHHHHHHhllllllllLLLLhhhhhhhhhhhhllhhhhhhhhhhhhhhhhh
Query pcxtipwqiaQKLHAVtavleepkaNDRAhdlvdlqlleglllldadlxptrsaciaifea  236
ident
Sbjct ------nrlaIHNTLL---------LSSY-------------------------------  139
DSSP  ------lhhhHHHHHH---------HHHH-------------------------------


DSSP  hllllllllllllLHHHhHHHHHL-lLLLL-----------------llllLHHHHHH-
Query raqhpwpprvatlpHWPLiYAGALE-gLDHL-----------------elarTVDAAAQ-  277
ident
Sbjct ------------tkLDAR-LKPMVLlvKHWAkrkqinspyfgtlssygyvlmVLYYLIHv  186
DSSP  ------------hhHLLL-HHHHHHhhHHHHhhllllhhhllllhhhhhhhHHHHHHHl


DSSP  ------------------------------------------------------------
Query ------------------------------------------------------------  277
ident
Sbjct ikppvfpnlllsplkqekivdgfdvgfddkledippsqnysslgsllhgffrfyaykfep  246
DSSP  llllllllllllllllleelleellllllhhhllllllllllhhhhhhhhhhhhhhhllll


DSSP  ----hhHHHH---hhhhHLLLL--------------------------------------
Query ----avQRFV---aridRATKR--------------------------------------  292
ident        |
Sbjct rekvvtFRRPdgyltkqEKGWTryilaiedpfeishnvgrtvsssglyrirgefmaasrl  306
DSSP  llllllLLLLlllllhhHHLLLllllllllllllllhhhhllhhhhhhhhhhhhhhhhh


DSSP  -----------------
Query -----------------  292
ident
Sbjct lnsrsypipydslfeea  323
DSSP  hhllllllhhhhhlll
```

No 361: Query=mol1A Sbjct=4fh5A Z-score=6.3

back to top

```
DSSP  llllllllllhhhhhhhhhhhhhhllLLHHHHHHHHHHHHHHHHHHHHLLLlllllLLEEEE
Query aapyssptnlrslrdrltqvaerqgVVFGRLQRHVAXIVVAQFAATLTddtgaPLLLVK   60
ident                                     |                 |
Sbjct --------shkeftkfcyevyneikiSDKEFKEKRAALDTLRLCLKRIS-----PDAELV   47
DSSP  --------llhhhhhhhhhhhhhllLHHHHHHHHHHHHHHHHHHHHHHHL-----LLLEEE


DSSP  HHhhhhhhhllllllllLLEEEEEL--LLHHHHHHHHHHHHHHHLlllеEEEELLLEELll
Query GGsslelrrgipdsrtsKDFATVAR--RDIELIHEQLADAGETGwegfTAIFTAPEEIaa  118
ident                   |             |  |
Sbjct AF--gslesglalknsdMDLCVLMDsrVQSDTIALQFYEELIAE----GFEGKFLQRA--   99
DSSP  EE--lhhhhlllllllllEEEEEELLllLLHHHHHHHHHHHHHHL----LEEEEEEEEL--


DSSP  lllllLLEEEEEEEEEELLE--EEEEEEEEEEelllllllllleeeeelhhhhlllllleee
Query pgxpvKPRRFTAKLSYRGR--AFATVPIEVSsveagnadqfdtltsdalglvgvpaavav  176
ident                      |     |
Sbjct -----RIPIIKLTSDTKNGfgASFQCDIGFN-----------------------------  125
DSSP  -----LEEEEEEEEELLLLllLLLLEEEEEL-----------------------------


DSSP  eellhhhhhhHHHHHHhllllllllLLLLhhhhhhhhhhhhllhhhhhhhhhhhhhhhhh
Query pcxtipwqiaQKLHAVtavleepkaNDRAhdlvdlqlleglllldadlxptrsaciaifea  236
ident
Sbjct ------nrlaIHNTLL---------LSSY-------------------------------  139
DSSP  ------lhhhHHHHHH---------HHHH-------------------------------
```

```
DSSP  hllllllllllllLHHHhHHHHHLL-LLLL-----------------llllLHHHHHH-
Query raqhpwpprvatlpHWPLiYAGALEG-LDHL-----------------elarTVDAAAQ-  277
ident
Sbjct ------------tkLDAR-LKPMVLLvKHWAkrkqinspyfgtlssygyvlmVLYYLIHv  186
DSSP  ------------hhHLLL-HHHHHHHhHHHHhhlllllhhhllllhhhhhhhHHHHHHHl


DSSP  ------------------------------------------------------------
Query ------------------------------------------------------------  277
ident
Sbjct ikppvfpnlllsplkqekivdgfdvgfddkledippsqnysslgsllhgffrfyaykfep  246
DSSP  llllllllllllllllleelleelllllhhhllllllllllhhhhhhhhhhhhhhllll


DSSP  ----hhHHHH---hhhhHLLLL--------------------------------------
Query ----avQRFV---aridRATKR--------------------------------------  292
ident        |
Sbjct rekvvtFRRPdgyltkqEKGWTryilaiedpfeishnvgrtvsssglyrirgefmaasrl  306
DSSP  llllllLLLLlllllhhHHLLLllllllllllllllllllllhhhhhhhhhhhhhhhhh


DSSP  -----------------
Query -----------------  292
ident
Sbjct lnsrsypipydslfeea  323
DSSP  hhllllllllhhhhhlll
```

No 362: Query=mol1A Sbjct=4fhwA Z-score=6.3

back to top

```
DSSP  llllllllllhhhhhhhhhhhhhhllLLHHHHHHHHHHHHHHHHHHHLLLlllllLLEEEE
Query aapyssppptnlrslrdrltqvaerqgVVFGRLQRHVAXIVVAQFAATLTddtgaPLLLVK   60
ident                                     |                 |
Sbjct --------shkeftkfcyevyneikiSDKEFKEKRAALDTLRLCLKRIS-----PDAELV   47
DSSP  --------llhhhhhhhhhhhhhhllLHHHHHHHHHHHHHHHHHHHHHLLL-----LLLEEE


DSSP  HHhhhhhhhllllllllLLEEEEEL--LLHHHHHHHHHHHHHHLllleEEEELLLEELll
Query GGsslelrrgipdsrtsKDFATVAR--RDIELIHEQLADAGETGwegfTAIFTAPEEIaa  118
ident                   |             |  |
Sbjct AF--gslesglalknsdMDLCVLMDsrVQSDTIALQFYEELIAE----GFEGKFLQRA--   99
DSSP  EE--lhhhhllllllllEEEEEELLllLLHHHHHHHHHHHHHHHL----LEEEEEEEEL--


DSSP  lllllLLEEEEEEEEEELLE--EEEEEEEEEEellllllllleeeeelhhhhllllllleee
Query pgxpvKPRRFTAKLSYRGR--AFATVPIEVSsveagnadqfdtltsdalglvgvpaavav  176
ident                      |     |
Sbjct -----RIPIIKLTSDTKNGfgASFQCDIGFN-----------------------------  125
DSSP  -----LEEEEEEEEELLLLllLLLLEEEEEL-----------------------------


DSSP  eellhhhhhhHHHHHHhllllllllLLLLhhhhhhhhhhhhllhhhhhhhhhhhhhhhhh
Query pcxtipwqiaQKLHAVtavleepkaNDRAhdlvdlqlleglllldadlxptrsaciaifea  236
ident
Sbjct ------nrlaIHNTLL---------LSSY-------------------------------  139
DSSP  ------lhhhHHHHHHH---------HHHH-------------------------------


DSSP  hllllllllllllLHHHhHHHHHLL-LLLL-----------------llllLHHHHHH-
Query raqhpwpprvatlpHWPLiYAGALEG-LDHL-----------------elarTVDAAAQ-  277
ident
Sbjct ------------tkLDAR-LKPMVLLvKHWAkrkqinspyfgtlssygyvlmVLYYLIHv  186
DSSP  ------------hhHLLL-HHHHHHHhHHHHhhlllllhhhllllhhhhhhhHHHHHHHl


DSSP  ------------------------------------------------------------
Query ------------------------------------------------------------  277
ident
Sbjct ikppvfpnlllsplkqekivdgfdvgfddkledippsqnysslgsllhgffrfyaykfep  246
DSSP  llllllllllllllllleelleelllllhhhllllllllllhhhhhhhhhhhhhhllll


DSSP  ----hhHHHH---hhhhHLLLL--------------------------------------
Query ----avQRFV---aridRATKR--------------------------------------  292
```

```
Sbjct rekvvtFRRPdgyltkqEKGWTryilaiedpfeishnvgrtvsssglyrirgefmaasrl  306
DSSP  llllllLLLLlllllhhHHLLLlllllllllllllllllllhhhhhhhhhhhhhhhhh


DSSP  -----------------
Query -----------------  292
ident
Sbjct lnsrsypipydslfeea  323
DSSP  hhllllllhhhhhlll
```

No 363: Query=mol1A Sbjct=4fhvA Z-score=6.3

back to top

```
DSSP  llllllllllhhhhhhhhhhhhhhllLLHHHHHHHHHHHHHHHHHHHLLLllllllLLEEEE
Query aapyssppptnlrslrdrltqvaerqgVVFGRLQRHVAXIVVAQFAATLTddtgaPLLLVK   60
ident                                     |                 |
Sbjct --------shkeftkfcyevyneikiSDKEFKEKRAALDTLRLCLKRIS-----PDAELV   47
DSSP  --------llhhhhhhhhhhhhhhllLHHHHHHHHHHHHHHHHHHHHHHLLL-----LLLEEE


DSSP  HHhhhhhhhllllllllLLEEEEEL--LLHHHHHHHHHHHHHHHLllleEEEELLLEELll
Query GGsslelrrgipdsrtsKDFATVAR--RDIELIHEQLADAGETGwegfTAIFTAPEEIaa  118
ident                   |             |  |
Sbjct AF--gslesglalknsdMDLCVLMDsrVQSDTIALQFYEELIAE----GFEGKFLQRA--   99
DSSP  EE--lhhhhllllllllEEEEEELLllLLHHHHHHHHHHHHHHHL----LEEEEEEEEL--


DSSP  lllllLLEEEEEEEEEELLE--EEEEEEEEEEellllllllleeeeelhhhhllllllleee
Query pgxpvKPRRFTAKLSYRGR--AFATVPIEVSsveagnadqfdtltsdalglvgvpaavav  176
ident                      |     |
Sbjct -----RIPIIKLTSDTKNGfgASFQCDIGFN-----------------------------  125
DSSP  -----LEEEEEEEEELLLLllLLLLEEEEEL-----------------------------


DSSP  eellhhhhhhHHHHHHhllllllllLLLLhhhhhhhhhhhhllhhhhhhhhhhhhhhhhh
Query pcxtipwqiaQKLHAVtavleepkaNDRAhdlvdlqlleglllldadlxptrsaciaifea  236
ident
Sbjct ------nrlaIHNTLL---------LSSY-------------------------------  139
DSSP  ------lhhhHHHHHHH---------HHHH-------------------------------


DSSP  hllllllllllllLHHHhHHHHHLL-LLLL-----------------llllLHHHHHH-
Query raqhpwpprvatlpHWPLiYAGALEG-LDHL-----------------elarTVDAAAQ-  277
ident
Sbjct ------------tkLDAR-LKPMVLLvKHWAkrkqinspyfgtlssygyvlmVLYYLIHv  186
DSSP  ------------hhHLLL-HHHHHHHhHHHHhhlllllhhhllllhhhhhhhHHHHHHHl


DSSP  ------------------------------------------------------------
Query ------------------------------------------------------------  277
ident
Sbjct ikppvfpnlllsplkqekivdgfdvgfddkledippsqnysslgsllhgffrfyaykfep  246
DSSP  lllllllllllllllllleelleelllllhhhllllllllllhhhhhhhhhhhhhhhllll


DSSP  ----hhHHHH---hhhhHLLLL--------------------------------------
Query ----avQRFV---aridRATKR--------------------------------------  292
ident        |
Sbjct rekvvtFRRPdgyltkqEKGWTryilaiedpfeishnvgrtvsssglyrirgefmaasrl  306
DSSP  llllllLLLLlllllhhHHLLLllllllllllllllhhhhllhhhhhhhhhhhhhhhhhh


DSSP  -----------------
Query -----------------  292
ident
Sbjct lnsrsypipydslfeea  323
DSSP  hhllllllhhhhhlll
```

No 364: Query=mol1A Sbjct=4p4oA Z-score=6.3

back to top

```
Query ----------------------------------------------------------aa    2
ident
Sbjct qemadlnnalgekykvssyhkllkkatkpklkaiqeltqvhgfgpraaaalfdregiftv   60
DSSP  llllhhhhllllhhhhhhhlllllllllhhhhhhhhhllllllhhhhhhhhhhhllllh


DSSP  lllllllhhhhhhhhhhhhhhllllhhhhhHHHHHHHHHHHHH-LLLL-lLLLLLLEEEE
Query pysspptnlrslrdrltqvaerqgvvfgrlqRHVAXIVVAQFAA-TLTD-dTGAPLLLVK   60
ident                                                          |
Sbjct dellqkadsipsltdqqrvgikyfydinekiPMQESVLHENYLReKCMEvlGKDFSILIC  120
DSSP  hhhhhhhhhlllllhhhhhhhhlhhhhllleEHHHHHHHHHHHHhHHHHhhLLLEEEEEL


DSSP  HHHHHHHhhlllllllLLLLEEEEEL---------------lLHHHHHHHHHHHHHhhllll
Query GGSSLELrrgipdsrTSKDFATVAR---------------rDIELIHEQLADAGetgweg  105
ident |               | |                            | |
Sbjct GSYRRRH-------pFSGDVDAILSrtldapplsepvaatgVLGHFVEFLESLK------  167
DSSP  HHHHLLL-------lEELLEEEEEEellllllllllhhhhlHHHHHHHHHHHLL------


DSSP  eeeEELL-LEELllllllLLLEEEEEEE-EEEL-LEEEEEEEEEEEEL-LLLLllllleeee
Query ftaIFTA-PEEIaapgxpVKPRRFTAK-LSYR-GRAFATVPIEVSSV-EAGNadqfdtlt  161
ident       |
Sbjct ---YLEAtMAQG------PLKYMGMGRlPPRInTKVYKARRVDIRLIeTKSV--------  210
DSSP  ---LEEEeEEEL------LLEEEEEELlLLLLlLLLLLLLEEEEEEEEElHHHH--------


DSSP  elhhhhllllllleeeeellhHHHHhhhhhhhhlllllllllllHHHHHHHhhhhhllhh
Query sdalglvgvpaavavpcxtiPWQIaqklhavtavleepkandraHDLVDLQllegllld a  221
ident
Sbjct -------------------pTAML----------------tftgSKNFNVI--mrqaais  233
DSSP  -------------------hHHHH----------------hhhlLHHHHHH--hhhhhhh


DSSP  hhhhhhhHHHH------------------------------------hHHHH--lllllL
Query dlxptrsACIA------------------------------------iFEAR--aqhpwP  243
ident                                                  | |
Sbjct kgyllneYGLFklgtpeearalyerigirgknageelgvpkdeledkrVEVRseqdvfdV  293
DSSP  lleeeelLEEEelllhhhhhhhhhlllllllleelleelhhhhlleELLLlhhhhhhH


DSSP  LLLLllllhhhhhhhhhllllllllllhhhhhhhhhhhhhhhhllll
Query PRVAtlphwpliyagaleglhlelartvdaaaqavqrfvaridratkr  292
ident
Sbjct LGMP------------------------------------yakpenrdp  306
DSSP  LLLL------------------------------------lllhhhlll
```

No 365: Query=mol1A Sbjct=1bpxA Z-score=6.3

[back to top](#)

```
DSSP  ------------------------------------------------------------
Query ------------------------------------------------------------    0
ident
Sbjct kapqetlnggitdmltelanfeknvsqaihkynayrkaasviakyphkiksgaeakklpg   60
DSSP  llllhhhhhhhhhhhhhhhhhhllllllhhhhhhhhhhhhhhhhhlllllllhhhhhllll


DSSP  -----------------------------------------------------lllllll
Query -----------------------------------------------------aapyssp    7
ident
Sbjct vgtkiaekideflatgklrklekirqddtsssinfltrvsgigpsaarkfvdegiktled  120
DSSP  llhhhhhhhhhhhhhhlllhhhhhhhllhhhhhhhhhhhlllllhhhhhhhhhlllllhhh


DSSP  lllhhhhhhhhhhhhhhllllhhhhhHHHHHHHHHHHHH-LLLLllLLLLEEEEHHHHHH
Query ptnlrslrdrltqvaerqgvvfgrlqRHVAXIVVAQFAA-TLTDdtGAPLLLVKGGSSLE   66
ident                                                     | |
Sbjct lrknedklnhhqriglkyfgdfekriPREEMLQMQDIVLnEVKKvdSEYIATVCGSFRRG  180
DSSP  hhllhhhllhhhhhhhhhhhhhlllleEHHHHHHHHHHHHhHHHHhlLLLEEEEELLLLLL


DSSP  HhhllllllLLLLEEEEEL------------lLHHHHHHHHHHHHHhhlllleeEEELLLE
Query LrrgipdsrTSKDFATVAR------------rDIELIHEQLADAGetgwegftAIFTAPE  114
ident           | |                         |||              |
Sbjct A-------eSSGDMDVLLThpsftsestkqpkLLHQVVEQLQKVH--------FITDTLS  225
```

```
DSSP  ElllllllLLLEEEEEEEEEEL--LEEEEEEEEEEEEL-LLLLllllleeeeelhhhhllll
Query EiaapgxpVKPRRFTAKLSYR--GRAFATVPIEVSSV-EAGNadqfdtltsdalglvgvp  171
ident                  |             |
Sbjct K------gETKFMGVCQLPSKndEKEYPHRRIDIRLIpKDQY------------------  261
DSSP  E------lLLEEEEEEELLLLLllLLLLLLEEEEEEEEeHHHH------------------


DSSP  lleeeeellhHHHHhhhhhhhhhlllllllllllHHHH-------------------hhh
Query aavavpcxtiPWQIaqklhavtavleepkandraHDLV-------------------dlq  212
ident                                    |
Sbjct ---------yCGVL----------------yftgSDIFnknmrahalekgftineytirp  296
DSSP  ---------hHHHH----------------hhhlLHHHhhhhhhhhhhlleeelllleee


DSSP  hhhhhllhhHHHHhhHHHHHhhhhhlllLLLLLllllllhhhhhhhhhllllllllllh
Query llegllldaDLXPtrSACIAifearaqhPWPPRvatlphwpliyagalegldhlelartv  272
ident           |
Sbjct lgvtgvagePLPV--DSEKD-----ifdYIQWK---------------------------  322
DSSP  lllllllllLLLL--LLHHH-----hhhHLLLL---------------------------


DSSP  hhhhhhhhhhhhhhhhllll
Query daaaqavqrfvaridratkr  292
ident
Sbjct -----------yrepkdrse  331
DSSP  -----------lllhhhlll
```

No 366: Query=mol1A Sbjct=7iclA Z-score=6.3

back to top

```
DSSP  ------------------------------------------------------------
Query ------------------------------------------------------------    0
ident
Sbjct etlnggitdmltelanfeknvsqaihkynayrkaasviakyphkiksgaeakklpgvgtk   60
DSSP  llllhhhhhhhhhhhhhhhhllllhhhhhhhhhhhhhhhhlllllllhhhhhlllllllhh


DSSP  -------------------------------------------------llllllllllh
Query -------------------------------------------------aapysspptnl   11
ident
Sbjct iaekideflatgklrklekirqddtsssinfltrvsgigpsaarkfvdegiktledlrkn  120
DSSP  hhhhhhhhhhllllhhhhhhhhlhhhhhhhhhlllllllhhhhhhhhhlllllhhhhhhl


DSSP  hhhhhhhhhhhhhllllhhhhhHHHHHHHHHHHHH-LLLLllLLLLEEEEHHHHHHHHhhl
Query rslrdrltqvaerqgvvfgrlqRHVAXIVVAQFAA-TLTDdtGAPLLLVKGGSSLELrrg   70
ident                                                 | |
Sbjct edklnhhqriglkyfgdfekriPREEMLQMQDIVLnEVKKvdSEYIATVCGSFRRGA---  177
DSSP  hhhllhhhhhhhhlhhhhhlleEHHHHHHHHHHHHHhHHHHhlLLLEEEEELLLLLLL---


DSSP  lllllLLLLEEEEEEL------------lLHHHHHHHHHHHHhhllllleeEEELLLEELll
Query ipdsrTSKDFATVAR------------rDIELIHEQLADAGetgwegftAIFTAPEEIaa  118
ident       | |                         |||             |
Sbjct ----eSSGDMDVLLThpsftsestkqpkLLHQVVEQLQKVH--------FITDTLSKG--  223
DSSP  ----lEELLEEEEEEellllllllllhHHHHHHHHHHHHHLL--------LEEEEEEEL--


DSSP  llllLLLEEEEEEEEEEELL--EEEEEEEEEEEEL-LLLLllllleeeeelhhhhllllllee
Query pgxpVKPRRFTAKLSYRG--RAFATVPIEVSSV-EAGNadqfdtltsdalglvgvpaava  175
ident              |             |
Sbjct ----ETKFMGVCQLPSKNdeKEYPHRRIDIRLIpKDQY----------------------  257
DSSP  ----LLEEEEEEELLLLLLllLLLLLLEEEEEEEEeHHHH----------------------


DSSP  eeellhHHHHhhhhhhhhhlllllllllllhhhhHHHHHH--------------------
Query vpcxtiPWQIaqklhavtavleepkandrahdlvDLQLLE--------------------  215
ident
Sbjct -----yCGVL--------------------yftgSDIFNKnmrahalekgftineytirp  292
DSSP  -----hHHHH--------------------hhllLHHHHHhhhhhhhhlleeelllleee


DSSP  -hhllhhhhhhhHHHHhhhhhhllllLLLLLLllllhhhhhhhhhllllllllllhhh
```

```
ident
Sbjct lgvtgvageplpVDSEK-------difDYIQWK---------------------------  318
DSSP  llllllllllllLLLHH-------hhhHHLLLL---------------------------


DSSP  hhhhhhhhhhhhhhllll
Query aaqavqrfvaridratkr  292
ident
Sbjct ---------yrepkdrse  327
DSSP  ---------lllllllll
```

No 367: Query=mol1A Sbjct=1zqlA Z-score=6.3

back to top

```
DSSP  ------------------------------------------------------------
Query ------------------------------------------------------------    0
ident
Sbjct etlnggitdmltelanfeknvsqaihkynayrkaasviakyphkiksgaeakklpgvgtk   60
DSSP  llllhhhhhhhhhhhhhhhhllllhhhhhhhhhhhhhhhhlllllllhhhhlllllllhh


DSSP  -------------------------------------------------llllllllllh
Query -------------------------------------------------aapysspptnl   11
ident
Sbjct iaekideflatgklrklekirqddtsssinfltrvsgigpsaarkfvdegiktledlrkn  120
DSSP  hhhhhhhhhhllllhhhhhhhhlhhhhhhhhhllllllhhhhhhhhhhllllhhhhhhl


DSSP  hhhhhhhhhhhhhllllhhhhhHHHHHHHHHHHHH-LLLLllLLLLEEEEHHHHHHHHhhl
Query rslrdrltqvaerqgvvfgrlqRHVAXIVVAQFAA-TLTDdtGAPLLLVKGGSSLELrrg   70
ident                                                 | |
Sbjct edklnhhqriglkyfgdfekriPREEMLQMQDIVLnEVKKvdSEYIATVCGSFRRGA---  177
DSSP  hhhllhhhhhhhhlhhhhhlleEHHHHHHHHHHHHHhHHHHhlLLLEEEEEELLLLLLL---


DSSP  lllllLLLLEEEEEL------------lLHHHHHHHHHHHHhhllllleeEEELLLEELll
Query ipdsrTSKDFATVAR------------rDIELIHEQLADAGetgwegftAIFTAPEEIaa  118
ident       | |                         |||             |
Sbjct ----eSSGDMDVLLThpsftsestkqpkLLHQVVEQLQKVH--------FITDTLSKG--  223
DSSP  ----lEELLEEEEEEellllllllllhhHHHHHHHHHHHHHLL--------LEEEEEEEL--


DSSP  llllLLLEEEEEEEEEELL--EEEEEEEEEEEEL-LLLLllllleeeeelhhhhllllllee
Query pgxpVKPRRFTAKLSYRG--RAFATVPIEVSSV-EAGNadqfdtltsdalglvgvpaava  175
ident              |             |
Sbjct ----ETKFMGVCQLPSKNdeKEYPHRRIDIRLIpKDQY----------------------  257
DSSP  ----LLEEEEEEELLLLLllLLLLLLEEEEEEEEEeLLLH----------------------


DSSP  eeellhHHHHhhhhhhhhllllllllllllhhhhHHHHHH--------------------
Query vpcxtiPWQIaqklhavtavleepkandrahdlvDLQLLE--------------------  215
ident
Sbjct -----yCGVL--------------------yftgSDIFNKnmrahalekgftineytirp  292
DSSP  -----hHHHH--------------------hhllLHHHHHhhhhhhhhhlleeeelleeee


DSSP  -hhllhhhhhhhHHHHHhhhhhhllllLLLLLlllllhhhhhhhhhllllllllllhhh
Query -glllddadlxptRSACIaifearaqhpWPPRVatlphwpliyagaleglldhlelartvda  274
ident
Sbjct lgvtgvageplpVDSEK-------difDYIQW----------------------------  317
DSSP  llllllllllllLLLHH-------hhhHHLLL----------------------------


DSSP  hhhhhhhhhhhhhhllll
Query aaqavqrfvaridratkr  292
ident
Sbjct --------kyrepkdrse  327
DSSP  --------llllhhhlll
```

No 368: Query=mol1A Sbjct=1zqcA Z-score=6.3

```
DSSP  ------------------------------------------------------------
Query ------------------------------------------------------------    0
ident
Sbjct etlnggitdmltelanfeknvsqaihkynayrkaasviakyphkiksgaeakklpgvgtk   60
DSSP  llllhhhhhhhhhhhhhhhhhllllhhhhhhhhhhhhhhhhlllllllhhhhhllllllhh


DSSP  -------------------------------------------------lllllllllh
Query -------------------------------------------------aapysspptnl   11
ident
Sbjct iaekideflatgklrklekirqddtsssinfltrvsgigpsaarkfvdegiktledlrkn  120
DSSP  hhhhhhhhhhllllhhhhhhhhlhhhhhhhhhlllllllhhhhhhhhhhllllhhhhhhl


DSSP  hhhhhhhhhhhhhllllhhhhhHHHHHHHHHHHHHH-LLLLllLLLLEEEEHHHHHHHhhl
Query rslrdrltqvaerqgvvfgrlqRHVAXIVVAQFAA-TLTDdtGAPLLLVKGGSSLELrrg   70
ident                                                 | |
Sbjct edklnhhqriglkyfgdfekriPREEMLQMQDIVLnEVKKvdSEYIATVCGSFRRGA---  177
DSSP  hhhllhhhhhhhhlhhhhhlleEHHHHHHHHHHHHhHHHHhlLLLEEEEELLLLLLL---


DSSP  lllllLLLLEEEEEL------------lLHHHHHHHHHHHHhhlllleeEEELLLEELll
Query ipdsrTSKDFATVAR------------rDIELIHEQLADAGetgwegftAIFTAPEEIaa  118
ident       | |                         |||             |
Sbjct ----eSSGDMDVLLThpsftsestkqpkLLHQVVEQLQKVH--------FITDTLSKG--  223
DSSP  ----lEELLEEEEEEelllllllllhhHHHHHHHHHHHLL--------LEEEEEEEL--


DSSP  llllLLLEEEEEEEEEELL--EEEEEEEEEEEEL-LLLLlllleeeeelhhhhllllllee
Query pgxpVKPRRFTAKLSYRG--RAFATVPIEVSSV-EAGNadqfdtltsdalglvgvpaava  175
ident              |             |
Sbjct ----ETKFMGVCQLPSKNdeKEYPHRRIDIRLIpKDQY----------------------  257
DSSP  ----LLEEEEEELLLLLLllLLLLLLEEEEEEEEeHHHH----------------------


DSSP  eeellhHHHHhhhhhhhhllllllllllllhhhhHHHHHH--------------------
Query vpcxtiPWQIaqklhavtavleepkandrahdlvDLQLLE--------------------  215
ident
Sbjct -----yCGVL--------------------yftgSDIFNKnmrahalekgftineytirp  292
DSSP  -----hHHHH--------------------hhllLHHHHHhhhhhhhhlleeeelleeee


DSSP  --hhllhhhhhhhhHHHHhhhhhhllllLLLLLlllllhhhhhhhhhlllllllllllhh
Query --glllldadlxptrSACIaifearaqhpWPPRVatlphwpliyagalegldhlelartvd  273
ident               |
Sbjct lgvtgvageplpvdSEKD-------ifdYIQWK---------------------------  318
DSSP  llllllllllllllLHHH-------hhhHLLLL---------------------------


DSSP  hhhhhhhhhhhhhhhhllll
Query aaaqavqrfvaridratkr  292
ident
Sbjct ----------yrepkdrse  327
DSSP  ----------lllhhhlll
```

No 369: Query=mol1A Sbjct=1mq2A Z-score=6.3

back to top

```
DSSP  ------------------------------------------------------------
Query ------------------------------------------------------------    0
ident
Sbjct tlnggitdmltelanfeknvsqaihkynayrkaasviakyphkiksgaeakklpgvgtki   60
DSSP  lllhhhhhhhhhhhhhhhhhllllhhhhhhhhhhhhhhhllllllllllhhhhhllllllhhh


DSSP  ------------------------------------------------llllllllllhh
Query ------------------------------------------------aapysspptnlr   12
ident
Sbjct aekideflatgklrklekirqddtsssinfltrvsgigpsaarkfvdegiktledlrkne  120
DSSP  hhhhhhhhhllllhhhhhhhhlhhhhhhhhhlllllllhhhhhhhhhhlllllhhhhhhlh


DSSP  hhhhhhhhhhhhllllhhhhhHHHHHHHHHHHHHH-LLLLLLLLLLEEEEHHHHHHHhhll
Query slrdrltqvaerqgvvfgrlqRHVAXIVVAQFAA-TLTDDTGAPLLLVKGGSSLELrrgi   71
```

```
Sbjct dklnhhqriglkyfgdfekriPREEMLQMQDIVLnEVKKvdSEYIATVCGSFRRGA     176
DSSP  hhllhhhhhhhhlhhhhhlllLHHHHHHHHHHHHHhHHHHHLLLLEEEELHHHLLLL----


DSSP  llllLLLLEEEEEL------------lLHHHHHHHHHHHHhhlllleeEEELLLEELlll
Query pdsrTSKDFATVAR------------rDIELIHEQLADAGetgwegftAIFTAPEEIaap  119
ident      | |                         |||             |
Sbjct ---eSSGDMDVLLThpsftsestkqpkLLHQVVEQLQKVH--------FITDTLSKG---  222
DSSP  ---lLLLLEEEEEEllllllllllllHHHHHHHHHHHLL--------LEEEEEEEL---


DSSP  lllLLLEEEEEEEEEELL--EEEEEEEEEEEEL-LLLLllllleeeeelhhhhllllllleee
Query gxpVKPRRFTAKLSYRG--RAFATVPIEVSSV-EAGNadqfdtltsdalglvgvpaavav  176
ident             |             |
Sbjct ---ETKFMGVCQLPSKNdeKEYPHRRIDIRLIpKDQY-----------------------  256
DSSP  ---LLEEEEEEELLLLLLllLLLLLLEEEEEEEELlLLLH-----------------------


DSSP  eellhHHHHhhhhhhhhlllllllllllhhhhHHHHHH---------------------
Query pcxtiPWQIaqklhavtavleepkandrahdlvDLQLLE---------------------  215
ident
Sbjct ----yCGVL--------------------yftgSDIFNKnmrahalekgftineytirpl  292
DSSP  ----hHHHH--------------------hhhlLHHHHHhhhhhhhhhhleeellllleeel


DSSP  hhllhhhhhhhHHHHHHhhhhhllllLLLLLlllllhhhhhhhhhllllllllllllhhhh
Query gllldadlxptRSACIAifearaqhpWPPRVatlphwpliyagalegldhlelartvdaa  275
ident
Sbjct gvtgvageplpVDSEKD-------ifDYIQW-----------------------------  316
DSSP  lllllllllllLLLHHH-------hhLLLLL-----------------------------


DSSP  hhhhhhhhhhhhhhllll
Query aqavqrfvaridratkr  292
ident
Sbjct -------kyrepkdrse  326
DSSP  -------llllhhhlll
```

No 370: Query=mol1A Sbjct=1zqmA Z-score=6.3

back to top

```
DSSP  ------------------------------------------------------------
Query ------------------------------------------------------------    0
ident
Sbjct etlnggitdmltelanfeknvsqaihkynayrkaasviakyphkiksgaeakklpgvgtk   60
DSSP  llllhhhhhhhhhhhhhhhhhllllhhhhhhhhhhhhhhhhhlllllllhhhhhlllllllhh


DSSP  -------------------------------------------------llllllllllh
Query -------------------------------------------------aapysspptnl   11
ident
Sbjct iaekideflatgklrklekirqddtsssinfltrvsgigpsaarkfvdegiktledlrkn  120
DSSP  hhhhhhhhhhllllhhhhhhhhlhhhhhhhhhlllllllhhhhhhhhhlllllhhhhhhl


DSSP  hhhhhhhhhhhhhllllhhhhhHHHHHHHHHHHHHH-LLLLllLLLLLEEEEHHHHHHHhhl
Query rslrdrltqvaerqgvvfgrlqRHVAXIVVAQFAA-TLTDdtGAPLLLVKGGSSLELrrg   70
ident                                                 | |
Sbjct edklnhhqriglkyfgdfekriPREEMLQMQDIVLnEVKKvdSEYIATVCGSFRRGA---  177
DSSP  hhhllhhhhhhhhlhhhhhlleEHHHHHHHHHHHHHhHHHHhlLLLEEEEELLLLLLLL---


DSSP  lllllLLLLEEEEEL------------lLHHHHHHHHHHHHhhlllleeEEELLLEELll
Query ipdsrTSKDFATVAR------------rDIELIHEQLADAGetgwegftAIFTAPEEIaa  118
ident       | |                         |||             |
Sbjct ----eSSGDMDVLLThpsftsestkqpkLLHQVVEQLQKVH--------FITDTLSKG--  223
DSSP  ----lEELLEEEEEEelllllllllhhHHHHHHHHHHHHHLL--------LEEEEEEEL--


DSSP  llllLLLEEEEEEEEEELL--EEEEEEEEEEEEL-LLLLllllleeeeelhhhhllllllee
Query pgxpVKPRRFTAKLSYRG--RAFATVPIEVSSV-EAGNadqfdtltsdalglvgvpaava  175
ident              |             |
Sbjct ----ETKFMGVCQLPSKNdeKEYPHRRIDIRLIpKDQY----------------------  257
DSSP  ----LLEEEEEEELLLLLLllLLLLLLEEEEEEEEEeLLLH----------------------
```

```
DSSP  [illegible]
Query vpcxtiPWQIaqklhavtavleepkandrahdlvDLQLLE--------------------  215
ident
Sbjct -----yCGVL--------------------yftgSDIFNKnmrahalekgftineytirp  292
DSSP  -----hHHHH--------------------hhllLHHHHHhhhhhhhhlleeeelleeee


DSSP  -hhllhhhhhhhHHHHHhhhhhhllllLLLLLlllllhhhhhhhhhllllllllllhhh
Query -gllldadlxptRSACIaifearaqhpWPPRVatlphwpliyagalegldhlelartvda  274
ident
Sbjct lgvtgvageplpVDSEK-------difDYIQW----------------------------  317
DSSP  llllllllllllLLLHH-------hhhHHLLL----------------------------


DSSP  hhhhhhhhhhhhhhllll
Query aaqavqrfvaridratkr  292
ident
Sbjct --------kyrepkdrse  327
DSSP  --------llllhhhlll
```

No 371: Query=mol1A Sbjct=8icfA Z-score=6.3

back to top

```
DSSP  ------------------------------------------------------------
Query ------------------------------------------------------------    0
ident
Sbjct etlnggitdmltelanfeknvsqaihkynayrkaasviakyphkiksgaeakklpgvgtk   60
DSSP  llllhhhhhhhhhhhhhhhhllllhhhhhhhhhhhhhhhllllllllhhhhhllllllhh


DSSP  -------------------------------------------------llllllllllh
Query -------------------------------------------------aapysspptnl   11
ident
Sbjct iaekideflatgklrklekirqddtsssinfltrvsgigpsaarkfvdegiktledlrkn  120
DSSP  hhhhhhhhhhllllhhhhhhhhlhhhhhhhhhlllllllhhhhhhhhlllllllhhhhhhl


DSSP  hhhhhhhhhhhhllllhhhhhHHHHHHHHHHHHH-LLLLllLLLLEEEEHHHHHHHHhl
Query rslrdrltqvaerqgvvfgrlqRHVAXIVVAQFAA-TLTDdtGAPLLLVKGGSSLELrrg   70
ident                                                 | |
Sbjct edklnhhqriglkyfgdfekriPREEMLQMQDIVLnEVKKvdSEYIATVCGSFRRGA---  177
DSSP  hhhllhhhhhhhhlhhhhhlleEHHHHHHHHHHHHHhHHHHhlLLLEEEEELLLLLLLL---


DSSP  lllllLLLLEEEEEEL------------lLHHHHHHHHHHHHhhllllleeEEELLLEELll
Query ipdsrTSKDFATVAR------------rDIELIHEQLADAGetgwegftAIFTAPEEIaa  118
ident       | |                         |||             |
Sbjct ----eSSGDMDVLLThpsftsestkqpkLLHQVVEQLQKVH--------FITDTLSKG--  223
DSSP  ----lEELLEEEEEEelllllllllllhHHHHHHHHHHHHLL--------LEEEEEEEL--


DSSP  llllLLLEEEEEEEEELL--EEEEEEEEEEEL-LLLLllllleeeeelhhhhllllllee
Query pgxpVKPRRFTAKLSYRG--RAFATVPIEVSSV-EAGNadqfdtltsdalglvgvpaava  175
ident              |             |
Sbjct ----ETKFMGVCQLPSKNdeKEYPHRRIDIRLIpKDQY----------------------  257
DSSP  ----LLEEEEEEELLLLLLllLLLLLLEEEEEEEEEeLLLH----------------------


DSSP  eeellhHHHHhhhhhhhhlllllllllllhhhhHHHHHH--------------------
Query vpcxtiPWQIaqklhavtavleepkandrahdlvDLQLLE--------------------  215
ident
Sbjct -----yCGVL--------------------yftgSDIFNKnmrahalekgftineytirp  292
DSSP  -----hHHHH--------------------hhllLHHHHHhhhhhhhhlleeellllleee


DSSP  --hhllhhhhhhhhHHHHhhhhhhllllLLLLLlllllhhhhhhhhhllllllllllhh
Query --gllldadlxptrSACIaifearaqhpWPPRVatlphwpliyagalegldhlelartvd  273
ident               |
Sbjct lgvtgvageplpvdSEKD-------ifdYIQWK---------------------------  318
DSSP  llllllllllllllLHHH-------hhhHHLLL---------------------------


DSSP  hhhhhhhhhhhhhhhllll
Query aaaqavqrfvaridratkr  292
ident
```

```
DSSP  ----------llllllll
```

No 372: Query=mol1A Sbjct=2bcuA Z-score=6.3

back to top

```
DSSP  ------------------------------------------------------------
Query ------------------------------------------------------------   0
ident 
Sbjct tnhnlhiteklevlakaysvqgdkwralgyakainalksfhkpvtsyqeacsipgigkrm   60
DSSP  lllllllllhhhhhhhhhhhlllhhhhhhhhhhhhhhlllllllllhhhhhhllllllhhh


DSSP  ---------------------------------------------lllllllllhhhhh
Query ---------------------------------------------aapyssspptnlrslr   15
ident 
Sbjct aekiieilesghlrkldhisesvpvlelfsniwgagtktaqmwyqqgfrsledirsqasl  120
DSSP  hhhhhhhhhlllhhhhhllllhhhhhhhhlllllhhhhhhhhhlllllhhhhhhhlll


DSSP  hhhhhhhhlllllhhhhHHHHHHHHHHHHHHHL-LLLLLLLLEEEEHHHHHHHhhlllll
Query drltqvaerqgvvfgrlQRHVAXIVVAQFAAT-LTDDTGAPLLLVKGGSSLELrrgipds   74
ident                            |             |    |
Sbjct ttqqaiglkhysdflerMPREEATEIEQTVQKaAQAFNSGLLCVACGSYRRGK-------  173
DSSP  lhhhhhhhhlllhhhllEEHHHHHHHHHHHHHHhHHHHLLLLEEEELHHHHLLL-------


DSSP  lLLLLEEEEEL--------lLHHHHHHHHHHHHhhlllleeeEELLLEELLllllLLLLE
Query rTSKDFATVAR--------rDIELIHEQLADAGetgwegftaIFTAPEEIAapgxPVKPR  126
ident  |  |                       |   |           |
Sbjct aTCGDVDVLIThpdgrshrgIFSRLLDSLRQEG---------FLTDDLVSQeengQQQKY  224
DSSP  lEELLEEEEEElllllllllLHHHHHHHHHHLL---------LEEEEEELLllllLLLEE


DSSP  EEEEEEEEllEEEEEEEEEEEEL-LLLLlllleeeeelhhhhlllllleeeeellhHHHH
Query RFTAKLSYrgRAFATVPIEVSSV-EAGNadqfdtltsdalglvgvpaavavpcxtiPWQI  185
ident      |                |
Sbjct LGVCRLPG--PGRRHRRLDIIVVpYSEF----------------------------aCALL  255
DSSP  EEEELLLL--LLLLLEEEEEEEELlHHHH----------------------------hHHHH


DSSP  hhhhhhhhlllllllllllHHHHHHH-------------------------hhhhhllh
Query aqklhavtavleepkandraHDLVDLQ-------------------------lleglllld  220
ident 
Sbjct ----------------yftgSAHFNRSmralaktkgmslsehalstavvrnthgckvgpg  299
DSSP  ----------------hhhlLHHHHHHhhhhhhhllleellllleeelleellllleeell


DSSP  HHHHhhHHHHHhhhhhhllllLLLLLllllhhhhhhhhhlllllllllllhhhhhhhhh
Query ADLXptRSACIaifearaqhpWPPRVatlphwpliyagalegldhlelartvdaaaqavq  280
ident   |
Sbjct RVLP--TPTEK-------dvfRLLGL----------------------------------  316
DSSP  EELL--LLLHH-------hhhHHLLL----------------------------------


DSSP  hhhhhhhhllll
Query rfvaridratkr  292
ident 
Sbjct --pyrepaerdw  326
DSSP  --llllhhhlll
```

No 373: Query=mol1A Sbjct=2ihmA Z-score=6.3

back to top

```
DSSP  ------------------------------------------------------------
Query ------------------------------------------------------------   0
ident 
Sbjct smpayacqrpsplthhntllsealetlaeaagfeanegrllsfsraasvlkslpcpvasl   60
DSSP  llllllhhhlllllllllhhhhhhhhhhhhhhhhlllhhhhhhhhhhhhhhhhlllllllh


DSSP  ------------------------------------------------------------
```

```
ident
Sbjct sqlhglpyfgehstrviqellehgtceevkqvrcseryqtmklftqvfgvgvktanrwyq  120
DSSP  hhhllllllhhhhhhhhhhhhhlllhhhhhhhhlhhhhhhhhhhllllllhhhhhhhhh


DSSP  -llllllllllhhhhhhhhhhhhhhllllhhhhhHHHHHHHHHHHHHL-LLLLlLLLLEE
Query -aapysspptnlrslrdrltqvaerqgvvfgrlqRHVAXIVVAQFAAT-LTDDtGAPLLL   58
ident                                   |        |
Sbjct eglrtldelreqpqrltqqqkaglqyyqdlstpvRRADAEALQQLIEAaVRQTlPGATVT  180
DSSP  llllhhhhhlllllllhhhhhhhhlhhhhhlleEHHHHHHHHHHHHHHhHHLLlLLLEEE


DSSP  EEHHHHHHHhhlllllLLLLEEEEEL--------lLHHHHHHHHHHHHhhlllleeeEE
Query VKGGSSLELrrgipdsrTSKDFATVAR--------rDIELIHEQLADAGetgwegftaIF  110
ident   ||                |                       |   |
Sbjct LTGGFRRGK-------lQGHDVDFLIThpeegqevgLLPKVMSCLQSQG---------LV  224
DSSP  ELHHHHLLL-------lEELEEEEEEEllllllllHHHHHHHHHHHHLL---------LE


DSSP  LLLEELLLLlllLLLEEEEEEEEEllEEEEEEEEEEEEL-LLLLlllleeeeelhhhhll
Query TAPEEIAAPgxpVKPRRFTAKLSYrgRAFATVPIEVSSV-EAGNadqfdtltsdalglvg  169
ident                      |         |
Sbjct LYHQYHRSH-dvFERSFCILGLPQ--PTWKAVRVDLVVTpSSQF----------------  265
DSSP  EEEEEELLL-llLLEEEEEEEEEL--LLEEEEEEEEEELlLLLH----------------


DSSP  lllleeeeellhHHHHHHhhhhhlllllllllllHHHHHHH-----------------
Query vpaavavpcxtiPWQIAQklhavtavleepkandraHDLVDLQ-----------------  212
ident             |
Sbjct ------------PFALLG---------------wtgSQFFERElrrfsrqekglwlnshg  298
DSSP  ------------HHHHHH---------------hhlLHHHHHHhhhhhhhhhlleellll


DSSP  -hhhhhllhhhhhHHHHHhhhhhhhhlllllllllllllhhhhhhhhhllllllllll
Query -lleglllldadlxPTRSAciaifearaqhpwpprvatlphwpliyagalegldhlelart  271
ident
Sbjct lfdpeqkrvfhatSEEDV------------------------------------------  316
DSSP  eellllllllllLHHHH------------------------------------------


DSSP  hhhhhhhhhhHHHH---hhllll
Query vdaaaqavqrfVARI---dratkr  292
ident
Sbjct --------frlLGLKylppeqrna  332
DSSP  --------hhhLLLLlllhhhlll
```

No 374: Query=mol1A Sbjct=1xspA Z-score=6.3

back to top

```
DSSP  ------------------------------------------------------------
Query ------------------------------------------------------------    0
ident
Sbjct atnhnlhiteklevlakaysvqgdkwralgyakainalksfhkpvtsyqeacsipgigkr   60
DSSP  llllhhhhhhhhhhhhhhhhhllhhhhhhhhhhhhhhhhlllllllhhhhhllllllhh


DSSP  ----------------------------------------------llllllllllhhhh
Query ----------------------------------------------aapysspptnlrsl   14
ident
Sbjct maekiieilesghlrkldhisesvpvlelfsniwgagtktaqmwyqqgfrsledirsqas  120
DSSP  hhhhhhhhhllllhhhhhllllhhhhhhhhlllllhhhhhhhhhlllllhhhhhhhll


DSSP  hhhhhhhhhhllllhhHHHHHHHHHHHHHHHHHHLL-LLLLLLLLEEEEHHHHHHHhhllll
Query rdrltqvaerqgvvfgRLQRHVAXIVVAQFAATL-TDDTGAPLLLVKGGSSLELrrgipd   73
ident                             |             |    |
Sbjct lttqqaiglkhysdflERMPREEATEIEQTVQKAaQAFNSGLLCVACGSYRRGK------  174
DSSP  llhhhhhhhhlllhhhLLEEHHHHHHHHHHHHHHHhHLLLLLLEEEELHHHHLLL------


DSSP  llLLLLEEEEEL--------lLHHHHHHHHHHHHhhlllleeeEELLLEELLllllLLLL
Query srTSKDFATVAR--------rDIELIHEQLADAGetgwegftaIFTAPEEIAapgxPVKP  125
ident   |  |                       |   |           |
Sbjct -aTCGDVDVLIThpdgrshrgIFSRLLDSLRQEG---------FLTDDLVSQeengQQQK  224
DSSP  -lEELLEEEEEEllllllllLHHHHHHHHHHLL---------LEEEEEELLllllLLLE
```

```
DSSP  EEEEEEEEEllEEEEEEEEEEEEELLLLLllllleeeeelhhhhlllllleeeeellhHHHH
Query RRFTAKLSYrgRAFATVPIEVSSVEAGNadqfdtltsdalglvgvpaavavpcxtiPWQI  185
ident        |                   |
Sbjct YLGVCRLPG--PGRRHRRLDIIVVPYSE-------------------------faCALL  256
DSSP  EEEEELLLL--LLLLLEEEEEEEELLHHH-------------------------hhHHHH


DSSP  hhhhhhhhlllllllllllHHHHHHH------------------------hhhhhllh
Query aqklhavtavleepkandraHDLVDLQ------------------------lleglllld  220
ident
Sbjct ----------------yftgSAHFNRSmralaktkgmslsehalstavvrnthgakvgpg  300
DSSP  ----------------hhhlLHHHHHHhhhhhhhllleellllleeelleellllleeell


DSSP  HHHHhhHHHHHhhhhhhllllLLLLLlllllhhhhhhhhhllllllllllhhhhhhhhh
Query ADLXptRSACIaifearaqhpWPPRVatlphwpliyagalegldhlelartvdaaaqavq  280
ident    |
Sbjct RVLP--TPTEK-------dvfRLLGL----------------------------------  317
DSSP  EELL--LLLHH-------hhhHHLLL----------------------------------


DSSP  hhhhhhhhllll
Query rfvaridratkr  292
ident
Sbjct --pyrepaerdw  327
DSSP  --llllhhhlll
```

No 375: Query=mol1A Sbjct=2bcsA Z-score=6.3

back to top

```
DSSP  ------------------------------------------------------------
Query ------------------------------------------------------------    0
ident
Sbjct hnlhiteklevlakaysvqgdkwralgyakainalksfhkpvtsyqeacsipgigkrmae   60
DSSP  llhhhhhhhhhhhhhhhhlllhhhhhhhhhhhhhhhlllllllhhhhhlllllllhhhhh


DSSP  -------------------------------------------lllllllllhhhhhhh
Query -------------------------------------------aapysspptnlrslrdr   17
ident
Sbjct kiieilesghlrkldhisesvpvlelfsniwgagtktaqmwyqqgfrsledirsqasltt  120
DSSP  hhhhhhhllllhhhhllllllhhhhhhhhllllllhhhhhhhhhlllllhhhhhhhhllllh


DSSP  hhhhhhhllllhhHHHHHHHHHHHHHHHHHLL-LLLLLLLLEEEEHHHHHHHHhhlllllllL
Query ltqvaerqgvvfgRLQRHVAXIVVAQFAATL-TDDTGAPLLLVKGGSSLELrrgipdsrT   76
ident                          |             |    |              |
Sbjct qqaiglkhysdflERMPREEATEIEQTVQKAaQAFNSGLLCVACGSYRRGK-------aT  173
DSSP  hhhhhhhhlllhhhLLEEHHHHHHHHHHHHHHHHhHHLLLLLLEEEELHHHHLLL-------lE


DSSP  LLLEEEEEL--------lLHHHHHHHHHHHHhhllllleeeEELLLEELLllllLLLLEEE
Query SKDFATVAR--------rDIELIHEQLADAGetgwegftaIFTAPEEIAapgxPVKPRRF  128
ident    |                       |   |            |
Sbjct CGDVDVLIThpdgrshrgIFSRLLDSLRQEG---------FLTDDLVSQeengQQQKYLG  224
DSSP  ELEEEEEEElllllllllLHHHHHHHHHHHLL---------LEEEEEELLllllLLLEEEE


DSSP  EEEEEEllEEEEEEEEEEEEELLLLLllllleeeeelhhhhlllllleeeeellhHHHHhhh
Query TAKLSYrgRAFATVPIEVSSVEAGNadqfdtltsdalglvgvpaavavpcxtiPWQIaqk  188
ident     |                  |
Sbjct VCRLPG--PGRRHRRLDIIVVPYSE-------------------------faCALL---  253
DSSP  EELLLL--LLLLLEEEEEEEELLHHH-------------------------hhHHHH---


DSSP  hhhhhlllllllllllHHHHHHH------------------------hhhhhllhHHH
Query lhavtavleepkandraHDLVDLQ------------------------lleglllldADL  223
ident                                                          |
Sbjct -------------yftgSAHFNRSmralaktkgmslsehalstavvrnthgckvgpgRVL  300
DSSP  -------------hhhlLHHHHHHhhhhhhhlleeellllleeelleellllleeellEEL


DSSP  HhhHHHHHhhhhhhllllLLLLLlllllhhhhhhhhhllllllllllhhhhhhhhhhh
Query XptRSACIaifearaqhpWPPRVatlphwpliyagalegldhlelartvdaaaqavqrfv  283
```

```
[illegible]
DSSP  L--LLLHH-------hhhHHLLL------------------------------------l
```

```
DSSP  hhhhhllll
Query aridratkr  292
ident
Sbjct yrepaerdw  324
DSSP  lllhhhhll
```

No 376: Query=mol1A Sbjct=4k4hA Z-score=6.3

back to top

```
DSSP  ------------------------------------------------------------
Query ------------------------------------------------------------    0
ident
Sbjct atnhnlhiteklevlakaysvqgdkwralgyakainalksfhkpvtsyqeacsipgigkr   60
DSSP  lllllhhhhhhhhhhhhhhhhlllhhhhhhhhhhhhhhhhlllllllhhhhhhlllllhh


DSSP  ----------------------------------------------llllllllllhhhh
Query ----------------------------------------------aapysspptnlrsl   14
ident
Sbjct maekiieilesghlrkldhisesvpvlelfsniwgagtktaqmwyqqgfrsledirsqas  120
DSSP  hhhhhhhhhlllllhhhhlllllhhhhhhhhlllllhhhhhhhhhlllllhhhhhhhhll


DSSP  hhhhhhhhhhllllhHHHHHHHHHHHHHHHHHLL-LLLLLLLEEEEHHHHHHHHhhllll
Query rdrltqvaerqgvvfGRLQRHVAXIVVAQFAATL-TDDTGAPLLLVKGGSSLELrrgipd   73
ident                              |             |    |
Sbjct lttqqaiglkhysdfLERMPREEATEIEQTVQKAaQAFNSGLLCVACGSYRRGK------  174
DSSP  llhhhhhhhhlhhhhHLLEEHHHHHHHHHHHHHHhHLLLLLLEEEELHHHHLLL------


DSSP  llLLLLEEEEEL--------lLHHHHHHHHHHHHhhlllleeeEELLL-EELLllllLLL
Query srTSKDFATVAR--------rDIELIHEQLADAGetgwegftaIFTAP-EEIAapgxPVK  124
ident   |  |                       |   |           |
Sbjct -aTCGDVDVLIThpdgrshrgIFSRLLDSLRQEG---------FLTDDlVSQE-engQQQ  223
DSSP  -lEELLEEEEEElllllllllHHHHHHHHHHHLL---------LEEEEeEELL-lllLLL


DSSP  LEEEEEEEEEllEEEEEEEEEEEELLLLLlllleeeeelhhhhllllllleeeeellhHHH
Query PRRFTAKLSYrgRAFATVPIEVSSVEAGNadqfdtltsdalglvgvpaavavpcxtiPWQ  184
ident        |                |
Sbjct KYLGVCRLPG--PGRRHRRLDIIVVPYSE--------------------------faCAL  255
DSSP  EEEEEELLLL--LLLLLEEEEEEELLHHH--------------------------hhHHH


DSSP  HhhhhhhhhlllllllllllHHHHHHHH--------------------hhhhllhHHHHh
Query IaqklhavtavleepkandraHDLVDLQL--------------------legllldADLXp  225
ident                                                           |
Sbjct L----------------yftgSAHFNRSMralaktkgmslsehalstavvkvgpgRVLP-  298
DSSP  H----------------hhhlLHHHHHHHhhhhhhlleeellllleeelllllllEEEL-


DSSP  hHHHHHhhhhhhllllLLLLLlllllhhhhhhhhhlllllllLLLLHhhhhhhhhhhhhh
Query tRSACIaifearaqhpWPPRVatlphwpliyagalegldhleLARTVdaaaqavqrfvar  285
ident                                            |
Sbjct -TPTEK-------dvfRLLGL-----------pyrepaerdwLEHHH-------------  326
DSSP  -LLLHH-------hhhHHLLL-----------llllhhhlllLLLLL-------------


DSSP  hhhllll
Query idratkr  292
ident
Sbjct ------h  327
DSSP  ------l
```

No 377: Query=mol1A Sbjct=5ddmA Z-score=6.3

back to top

```
Query                      aapysspptnlrslrdrltqvaerqgvvfGRLQRHVAXI   39
ident
Sbjct vlelfsniwgagtktaqmwyqqgfrsledirsqaslttqqaiglkhysdfLERMPREEAT   60
DSSP  llllllllllhhhhhhhhhlllllhhhhhhhllllhhhhhhhhlhhhhLLLEEHHHHH


DSSP  HHHHHHHLL-LLLLLLLLEEEEHHHHHHHhhllllllLLLLEEEEEL--------lLHHH
Query VVAQFAATL-TDDTGAPLLLVKGGSSLELrrgipdsrTSKDFATVAR--------rDIEL   90
ident    |             |    |              |  |
Sbjct EIEQTVQKAaQAFNSGLLCVACGSYRRGK-------aTCGDVDVLIThpdgrshrgIFSR  113
DSSP  HHHHHHHHHhHHHLLLLEEEEELLLLLLL-------lEELLEEEEEEllllllllHHHH


DSSP  HHHHHHHHHhhlllleeeEELLL-EELLLlLLLLLLEEEEEEEEEllEEEEEEEEEEEEL
Query IHEQLADAGetgwegftaIFTAP-EEIAApGXPVKPRRFTAKLSYrgRAFATVPIEVSSV  149
ident     |   |           |                     |                |
Sbjct LLDSLRQEG---------FLTDDlVSQEE-NGQQQKYLGVCRLPG--PGRRHRRLDIIVV  161
DSSP  HHHHHHHLL---------LEEEEeEEEEE-ELLEEEEEEEELLLL--LLLLLEEEEEEEL


DSSP  -LLLLlllleeeeelhhhhllllllleeeeellhHHHHhhhhhhhhllllllllllllHHH
Query -EAGNadqfdtltsdalglvgvpaavavpcxtiPWQIaqklhavtavleepkandraHDL  208
ident
Sbjct pYSEF---------------------------aCALL----------------yftgSAH  178
DSSP  lHHHH---------------------------hHHHH----------------hhhlLHH


DSSP  HHHH-------------------------hhhhhllhHHHHhhHHHHHhhhhhhllllLL
Query VDLQ-------------------------llegllldADLXptRSACIaifearaqhpWP  243
ident                                        |
Sbjct FNRSmralaktkgmslsehalstavvrnthgckvgpgRVLP--TPTEK-------dvfRL  229
DSSP  HHHHhhhhhhhhlleeellllleeellllllllllllllLEEL--LLLHH-------hhhHH


DSSP  LLLllllllhhhhhhhhhhlllllllllllhhhhhhhhhhhhhhhhhllll
Query PRVatlphwpliyagalegldhlelartvdaaaqavqrfvaridratkr  292
ident
Sbjct LGL------------------------------------pyrepaerdw  242
DSSP  HLL------------------------------------llllhhhlll
```

No 378: Query=mol1A Sbjct=5cb1B Z-score=6.3

[back to top]

```
DSSP  ------------------------llllllllllhhhhhhhhhhhhhhllllhHHHHHHH
Query ------------------------aapysspptnlrslrdrltqvaerqgvvfGRLQRHV   36
ident
Sbjct svpvlelfsniwgagtktaqmwyqqgfrsledirsqaslttqqaiglkhysdfLERMPRE   60
DSSP  llhhhhhhhllllllhhhhhhhhhlllllhhhhhhhllllhhhhhhhhlhhhhLLLEEHH


DSSP  HHHHHHHHHHLL-LLLLLLLLEEEEHHHHHHHhhllllllLLLLEEEEEL--------lL
Query AXIVVAQFAATL-TDDTGAPLLLVKGGSSLELrrgipdsrTSKDFATVAR--------rD   87
ident       |             |    |              |  |
Sbjct EATEIEQTVQKAaQAFNSGLLCVACGSYRRGK-------aTCGDVDVLIThpdgrshrgI  113
DSSP  HHHHHHHHHHHHhHHHLLLLEEEEELHHHHLL-------lEELLEEEEEEllllllllH


DSSP  HHHHHHHHHHHHhhlllleeEEELLLEELLLLLlLLLLEEEEEEEEEllEEEEEEEEEEE
Query IELIHEQLADAGetgwegftAIFTAPEEIAAPGxPVKPRRFTAKLSYrgRAFATVPIEVS  147
ident        |   |                    |           |
Sbjct FSRLLDSLRQEG--------FLTDDLVSQEENG-QQQKYLGVCRLPG--PGRRHRRLDII  162
DSSP  HHHHHHHHHHLL--------LLLLLLLLLEEEL-LEEEEEEEELLLL--LLLLLEEEEEE


DSSP  EL-LLLLlllleeeeelhhhhllllllleeeeellhHHHHhhhhhhhhllllllllllllH
Query SV-EAGNadqfdtltsdalglvgvpaavavpcxtiPWQIaqklhavtavleepkandraH  206
ident  |
Sbjct VVpYSEF---------------------------aCALL----------------yftgS  179
DSSP  ELlLLLH---------------------------hHHHH----------------hhhlL


DSSP  HHHHHH----------------------hhhhhllhhhhhhHHHHHhhhhhhllllLL
Query DLVDLQ----------------------llegllldadlxptRSACIaifearaqhpWP  243
ident
Sbjct AHFNRSmralaktkgmslsehalstavvrnthgckvgpgrvlpTPTEK-------dvfRL  232
```

```
DSSP  LLLlllllhhhhhhhhhlllllllllllhhhhhhhhhhhhhhhhhllll
Query PRVatlphwpliyagalegldhlelartvdaaaqavqrfvaridratkr  292
ident
Sbjct LGL------------------------------------pyrepaerdw  245
DSSP  LLL------------------------------------llllhhhhll
```

No 379: Query=mol1A Sbjct=4xusA Z-score=6.3

back to top

```
DSSP  ------------------------------------------------------------
Query ------------------------------------------------------------    0
ident
Sbjct hnlhiteklevlakaysvqgdkwralgyakainalksfhkpvtsyqeacsipgigkrmae   60
DSSP  lllhhhhhhhhhhhhhhhllhhhhhhhhhhhhhhhhlllllllhhhhhlllllllhhhhh


DSSP  -------------------------------------------llllllllllhhhhhhh
Query -------------------------------------------aapysspptnlrslrdr   17
ident
Sbjct kiieilesghlrkldhisesvpvlelfsniwgagtktaqmwyqqgfrsledirsqasltt  120
DSSP  hhhhhhhlllhhhhhllllhhhhhhhhlllllhhhhhhhhhllllhhhhhhhhllllh


DSSP  hhhhhhllllhhhhHHHHHHHHHHHHHHL-LLLLlLLLLEEEEHHHHHHHhhlllllllL
Query ltqvaerqgvvfgrlQRHVAXIVVAQFAAT-LTDDtGAPLLLVKGGSSLELrrgipdsrT   76
ident                          |             |    |              |
Sbjct qqaiglkhysdflerMPREEATEIEQTVQKaAQAFnSGLLCVACGSYRRGK-------aT  173
DSSP  hhhhhhhlhhhhhllEEHHHHHHHHHHHHHHhHHHHlLLLEEEEELHHHHLLL-------lE


DSSP  LLLEEEEEL--------lLHHHHHHHHHHHHhhlllleeeEELLLEELLllllLLLLEEE
Query SKDFATVAR--------rDIELIHEQLADAGetgwegftaIFTAPEEIAapgxPVKPRRF  128
ident   |                       |   |           |
Sbjct CGDVDVLIThpdgrshrgIFSRLLDSLRQEG---------FLTDDLVSQeengQQQKYLG  224
DSSP  ELLEEEEEEllllllllHHHHHHHHHHHHLL---------LEEEEEELLllllLLLEEEE


DSSP  EEEEEEllEEEEEEEEEEEEL-LLLLlllleeeeelhhhhllllllleeeeellhHHHHhh
Query TAKLSYrgRAFATVPIEVSSV-EAGNadqfdtltsdalglvgvpaavavpcxtiPWQIaq  187
ident    |                |
Sbjct VCRLPG--PGRRHRRLDIIVVpYSEF---------------------------aCALL--  253
DSSP  EELLLL--LLLLLEEEEEEELlHHHH---------------------------hHHHH--


DSSP  hhhhhhllllllllllllHHHHHHH------------------------hhhhhllhHHH
Query klhavtavleepkandraHDLVDLQ------------------------llegllldADL  223
ident                                                            |
Sbjct --------------yftgSAHFNRSmralaktkgmslsehalstavvrnhgckvgpgRVL  299
DSSP  --------------hhhlLHHHHHHhhhhhhllleellllleeellllllllllllEEL


DSSP  HhhHHHHhhhhhhllllLLLLLlllllhhhhhhhhhlllllllllllhhhhhhhhhhhh
Query XptRSACIaifearaqhpWPPRVatlphwpliyagalegldhlelartvdaaaqavqrfv  283
ident
Sbjct P--TPTEK-------dvfRLLGL------------------------------------p  314
DSSP  L--LLLHH-------hhhHHLLL------------------------------------l


DSSP  hhhhhllll
Query aridratkr  292
ident
Sbjct yrepaerdw  323
DSSP  lllhhhlll
```

No 380: Query=mol1A Sbjct=4x5vA Z-score=6.3

back to top

```
DSSP  ------------------------------------------------------------
Query ------------------------------------------------------------    0
```

```
Sbjct [illegible]   60
DSSP  lllhhhhhhhhhhhhhhhhlllhhhhhhhhhhhhhhllhhhhhlllllhhhhhhhhhhhh


DSSP  ------------------------------------llllllllllhhhhhhhhhhhhh
Query ------------------------------------aapysspptnlrslrdrltqvaer   24
ident 
Sbjct sghlrkldhisesvpvlelfsniwgagtktaqmwyqqgfrsledirsqasltttqqaiglk  120
DSSP  llllhhhhllllllhhhhhhhhlllllllhhhhhhhhhlllllhhhhhhhllllhhhhhhhh


DSSP  llllhhHHHHHHHHHHHHHHHHHLL-LLLLLLLLEEEEHHHHHHHhhllllllLLLLEEEE
Query qgvvfgRLQRHVAXIVVAQFAATL-TDDTGAPLLLVKGGSSLELrrgipdsrTSKDFATV   83
ident                   |             |    |              |  |
Sbjct hysdflERMPREEATEIEQTVQKAaQAFNSGLLCVACGSYRRGK-------aTCGDVDVL  173
DSSP  lhhhhhLLEEHHHHHHHHHHHHHHHHhHHHLLLLEEEELHHHHLLL-------lEELLEEEE


DSSP  EL--------lLHHHHHHHHHHHHHhhllllleeeEELLLEELLllllLLLLEEEEEEEEEl
Query AR--------rDIELIHEQLADAGetgwegftaIFTAPEEIAapgxPVKPRRFTAKLSYr  135
ident                    |   |           |                    |
Sbjct IThpdgrshrgIFSRLLDSLRQEG---------FLTDDLVSQeengQQQKYLGVCRLPG-  223
DSSP  EEllllllllllHHHHHHHHHHHHLL---------LEEEEEELLllllLLLEEEEEEELLLL-


DSSP  lEEEEEEEEEEEELLLLLLlllleeeeelhhhhllllllleeeeellhHHHHhhhhhhhhll
Query gRAFATVPIEVSSVEAGNAdqfdtltsdalglvgvpaavavpcxtiPWQIaqklhavtav  195
ident              |
Sbjct -PGRRHRRLDIIVVPYSEF--------------------------aCALL----------  246
DSSP  -LLLLLEEEEEEEELLHHHH--------------------------hHHHH----------


DSSP  llllllllllHHHHHHHH-------------------------hhhhhllhHHHHhhHHHH
Query leepkandraHDLVDLQ-------------------------lleglllldADLXptRSAC  230
ident                                                     |
Sbjct ------yftgSAHFNRSmralaktkgmslsehalstavvrnthgckvgpgRVLP--TPTE  298
DSSP  ------hhhlLHHHHHHhhhhhhhllleellllleeelleelllllleeellEELL--LLLH


DSSP  HhhhhhhllllLLLLLllllllhhhhhhhhhllllllllllhhhhhhhhhhhhhhhhhll
Query Iaifearaqhp WPPRVatlphwpliyagalegldhlelartvdaaaqavqrfvaridrat  290
ident 
Sbjct K-------dvfRLLGL-------------------------------------pyrepaer  315
DSSP  H-------hhhHHHLL-------------------------------------llllhhhh


DSSP  ll
Query kr  292
ident 
Sbjct dw  317
DSSP  ll
```

No 381: Query=mol1A Sbjct=5ddyE Z-score=6.3

back to top

```
DSSP  -----------------------lllllllllhhhhhhhhhhhhhhllllhHHHHHHHH
Query -----------------------aapysspptnlrslrdrltqvaerqgvvfGRLQRHVA   37
ident 
Sbjct vpvlelfsniwgagtktaqmwyqqgfrsledirsqasltttqqaiglkhysdfLERMPREE   60
DSSP  lhhhhhhhlllllllhhhhhhhhhhhlllllhhhhhhllllllhhhhhhhhlhhhhLLLEEHHH


DSSP  HHHHHHHHHLL-LLLLLLLLEEEEHHHHHHhhhllllLLLLLLLEEEEEL--------lLH
Query XIVVAQFAATL-TDDTGAPLLLVKGGSSLElrrgipdSRTSKDFATVAR--------rDI   88
ident      |             |    |              |  |
Sbjct ATEIEQTVQKAaQAFNSGLLCVACGSYRRG-------KATCGDVDVLIThpdgrshrgIF  113
DSSP  HHHHHHHHHHHhHHHLLLLEEEELLLLLLL-------LLEELLEEEEEEllllllllllHH


DSSP  HHHHHHHHHHHhhllllleeeEELLL--EELLLllLLLLLEEEEEEEEEllEEEEEEEEEE
Query ELIHEQLADAGetgwegftaIFTAP--EEIAApgXPVKPRRFTAKLSYrgRAFATVPIEV  146
ident       |   |           |                      |
Sbjct SRLLDSLRQEG---------FLTDDlvSQEEN--GQQQKYLGVCRLPG--PGRRHRRLDI  160
DSSP  HHHHHHHHHLL---------LLLLEeeLLLEE--LLEEEEEEEELLLL--LLLLLEEEEE
```

```
DSSP  [illegible]
Query SSV-EAGNadqfdtltsdalglvgvpaavavpcxtiPWQIaqklhavtavleepkandra  205
ident   |
Sbjct IVVpYSEF----------------------------aCALL---------------yftg  177
DSSP  EELlLLLH----------------------------hHHHH---------------hhhl


DSSP  HHHHHHH-----------------------hhhhhllhhhhhhhHHHHHhhhhhhllllL
Query HDLVDLQ-----------------------llegllldadlxptRSACIaifearaqhpW  242
ident
Sbjct SAHFNRSmralaktkgmslsehalstavvrnthgckvgpgrvlpTPTEK-------dvfR  230
DSSP  LHHHHHHhhhhhhhhleeellllleeellllllllllllllleelLLLHH-------hhhH


DSSP  LLLLLllllhhhhhhhhhllllllllllllhhhhhhhhhhhhhhhhhllll
Query PPRVAtlphwpliyagalegldhlelartvdaaaqavqrfvaridratkr  292
ident
Sbjct LLGLP------------------------------------yrepaerdw  244
DSSP  HLLLL------------------------------------lllhhhhll
```

No 382: Query=mol1A Sbjct=1xslA Z-score=6.2

[back to top]

```
DSSP  ------------------------------------------------------------
Query ------------------------------------------------------------    0
ident
Sbjct atnhnlhiteklevlakaysvqgdkwralgyakainalksfhkpvtsyqeacsipgigkr   60
DSSP  lllllhhhhhhhhhhhhhhhhhlllhhhhhhhhhhhhhhhlllllllhhhhhhlllllhh


DSSP  ---------------------------------------------lllllllllllhhhh
Query ---------------------------------------------aapyssspptnlrsl   14
ident
Sbjct maekiieilesghlrkldhisesvpvlelfsniwgagtktaqmwyqqgfrsledirsqas  120
DSSP  hhhhhhhhhhlllhhhhlllllhhhhhhhhllllllhhhhhhhhhlllllhhhhhhhll


DSSP  hhhhhhhhhhllllhHHHHHHHHHHHHHHHHHLL--LLLLlLLLEEEEHHHHHHHhhlll
Query rdrltqvaerqgvvfGRLQRHVAXIVVAQFAATL--TDDTgAPLLLVKGGSSLELrrgip   72
ident                             |              |    |
Sbjct lttqqaiglkhysdfLERMPREEATEIEQTVQKAaqAFNS-GLLCVACGSYRRGK-----  174
DSSP  llhhhhhhhlhhhhHLLEEHHHHHHHHHHHHHHHHhhLLLL-LLEEEELHHHHLLL-----


DSSP  lllLLLLEEEEEL--------lLHHHHHHHHHHHHhhlllleeEEELLLE-ELLLllLLL
Query dsrTSKDFATVAR--------rDIELIHEQLADAGetgwegftAIFTAPE-EIAApgXPV  123
ident    |  |                       |   |
Sbjct --aTCGDVDVLIThpdgrshrgIFSRLLDSLRQEG--------FLTDDLVsQEEN--GQQ  222
DSSP  --lEELLEEEEEElllllllllLHHHHHHHHHHLL--------LEEEEEEeEEEE--LLE


DSSP  LLEEEEEEEEEllEEEEEEEEEEEEL-LLLLllllleeeeelhhhhllllllleeeeellhH
Query KPRRFTAKLSYrgRAFATVPIEVSSV-EAGNadqfdtltsdalglvgvpaavavpcxtiP  182
ident         |                |
Sbjct QKYLGVCRLPG--PGRRHRRLDIIVVpYSEF---------------------------aC  253
DSSP  EEEEEEELLLL--LLLLLEEEEEEELlHHHH---------------------------hH


DSSP  HHHhhhhhhhhllllllllllllHHHHHHH-------------------------hhhhh
Query WQIaqklhavtavleepkandraHDLVDLQ-------------------------llegl  217
ident
Sbjct ALL----------------yftgSAHFNRSmralaktkgmslsehalstavvrnthgckv  297
DSSP  HHH----------------hhhlLHHHHHHhhhhhhhhleeelllleeelllllllllll


DSSP  llhHHHHhhHHHHHHhhhhhlllLLLLLllllllhhhhhhhhhlllllllllllhhhhhh
Query lldADLXptRSACIAifearaqhPWPPRvatlphwpliyagalegldhlelartvdaaaq  277
ident      |
Sbjct gpgRVLP--TPTEKD-----vfrLLGLP--------------------------------  318
DSSP  lllLLLL--LLLHHH-----hhhHLLLL--------------------------------


DSSP  hhhhhhhhhhhllll
Query avqrfvaridratkr  292
ident
```

```
DSSP        lllhhhlll
```

No 383: Query=mol1A Sbjct=3pmlB Z-score=6.2

back to top

```
DSSP  ------------------------------------------------------------
Query ------------------------------------------------------------   0
ident 
Sbjct qkatnhnlhiteklevlakaysvqgdkwralgyakainalksfhkpvtsyqeacsipgig  60
DSSP  llllllhhhhhhhhhhhhhhhlllhhhhhhhhhhhhhhhllllllllhhhhhhlllll


DSSP  ------------------------------------------------llllllllllhh
Query ------------------------------------------------aapysspptnlr  12
ident 
Sbjct krmaekiieilesghlrkldhisesvpvlelfsniwgagtktaqmwyqqgfrsledirsq  120
DSSP  hhhhhhhhhhhhhlllhhhhlllllhhhhhhhlllllllhhhhhhhhllllllhhhhhhh


DSSP  hhhhhhhhhhhhllllhhhhhHHHHHHHHHHHHH-LLLLllLLLLEEEEHHHHHHHhhll
Query slrdrltqvaerqgvvfgrlqRHVAXIVVAQFAA-TLTDdtGAPLLLVKGGSSLELrrgi  71
ident                               |             |    |
Sbjct aslttqqaiglkhysdflermPREEATEIEQTVQkAAQAfnSGLLCVACGSYRRGK----  176
DSSP  llllhhhhhhhhlhhhhllleEHHHHHHHHHHHHHhHHHLllLLLEEEEELHHHHLLL----


DSSP  llllLLLLEEEEEL--------lLHHHHHHHHHHHHhhlllleeEEELLLEELllllllL
Query pdsrTSKDFATVAR--------rDIELIHEQLADAGetgwegftAIFTAPEEIaapgxpV  123
ident     |  |                       |   |
Sbjct ---aTCGDVDVLIThpdgrshrgIFSRLLDSLRQEG--------FLTDDLVKG------E  219
DSSP  ---lEELLEEEEEEllllllllHHHHHHHHHHHHLL--------LEEEEEEEL------L


DSSP  LLEEEEEEEEEllEEEEEEEEEEEEL-LLLLllllleeeeelhhhhlllllleeeeellhH
Query KPRRFTAKLSYrgRAFATVPIEVSSV-EAGNadqfdtltsdalglvgvpaavavpcxtiP  182
ident         |                |
Sbjct TKYLGVCRLPG--PGRRHRRLDIIVVpYSEF---------------------------aC  250
DSSP  LEEEEEELLLL--LLLLLEEEEEEEELlHHHH---------------------------hH


DSSP  HHHhhhhhhhhllllllllllllHHHHHHH-------------------------hhhhh
Query WQIaqklhavtavleepkandraHDLVDLQ-------------------------llegl  217
ident 
Sbjct ALL----------------yftgSAHFNRSmralaktkgmslsehalstavvrnthgckv  294
DSSP  HHH----------------hhhlLHHHHHHhhhhhhhlleeellllleeelleelllllee


DSSP  llhHHHHhhHHHHHhhhhhhlllLLLLLLlllllhhhhhhhhhlllllllllllhhhhhh
Query lldADLXptRSACIaifearaqhPWPPRVatlphwpliyagalegldhlelartvdaaaq  277
ident      |
Sbjct gpgRVLP--TPTEK-----dvfrLLGLPY-------------------------------  316
DSSP  ellLEEL--LLLHH-----hhhhHLLLLL-------------------------------


DSSP  hhhhhhhhhhhhllll
Query avqrfvaridratkr  292
ident 
Sbjct -------repaerdw  324
DSSP  -------llhhhlll
```

No 384: Query=mol1A Sbjct=4p4pA Z-score=6.2

back to top

```
DSSP  ----------------------------------llllllllllhhhhhhhhhhhhhhll
Query ----------------------------------aapysspptnlrslrdrltqvaerqg  26
ident 
Sbjct kykvpklkaiqeltqvhgfgpraaaalfdregiftvdellqkadsipsltdqqrvgikyf  60
DSSP  llllllhhhhhhhhlllllllhhhhhhhhhhhllllhhhhhhhhhhlllllhhhhhhhhlh


DSSP  llhhhhhHHHHHHHHHHHHH-LLLL-llLLLLEEEEHHHHHHhhhllllLLLLLLEEEEE
```

```
ident                                  |  |               | |
Sbjct ydinekiPMQESVLHENYLReKCMEvlgKDFSILICGSYRRR-------HPFSGDVDAIL  113
DSSP  hhhllleEHHHHHHHHHHHHhHHHHhllLLEEEEELHHHHLL-------LLEELLEEEEE


DSSP  L---------------lLHHHHHHHHHHHHhhlllleeEEELLLEElllllllLLLEEEE
Query R---------------rDIELIHEQLADAGetgwegftAIFTAPEEiaapgxpVKPRRFT  129
ident                        | |
Sbjct SrtldapplsepvaatgVLGHFVEFLESLK--------YLEATMAQ------gPLKYMGM  159
DSSP  EellllllllllhhhhlHHHHHHHHHHHHLL--------LEEEEEEE------lLLEEEEE


DSSP  EEEEEL--LEEEEEEEEEEEEL-LLLLllllleeeeelhhhhlllllleeeeellhHHHHh
Query AKLSYR--GRAFATVPIEVSSV-EAGNadqfdtltsdalglvgvpaavavpcxtiPWQIa  186
ident   |  |
Sbjct GRLPPRinTKVYKARRVDIRLIeTKSV---------------------------pTAML-  191
DSSP  ELLLLLllLLLLLLEEEEEEEElHHHH---------------------------hHHHH-


DSSP  hhhhhhhlllllllllllHHHH--------------------------------------
Query qklhavtavleepkandraHDLV-------------------------------------  209
ident
Sbjct ---------------tftgSKNFnvimrqaaiskgyllneyglfklgtpeearalyerig  236
DSSP  ---------------hhhlLHHHhhhhhhhhhhlleeeelleeeelllhhhhhhhhhhhl


DSSP  --------hhhhhhhhllhhHHHHhhHHHHhhhhhhlllLLLLLllllllhhhhhhhhhl
Query --------dlqlleglllldaDLXPtrSACIaifearaqhPWPPRvatlphwpliyagale  261
ident
Sbjct irgknageelgvpkdeledkRVEV--RSEQ-----dvfdVLGMP----------------  273
DSSP  lllllllleelleelhhhhllLLLL--LLHH-----hhhhHLLLL----------------


DSSP  llllllllllhhhhhhhhhhhhhhhhhhllll
Query gldhlelartvdaaaqavqrfvaridratkr  292
ident
Sbjct ----------------------yakpenrdp  282
DSSP  ----------------------lllhhhlll
```

No 385: Query=mol1A Sbjct=4yd2A Z-score=6.2

back to top

```
DSSP  ------------------------------------------------------------
Query ------------------------------------------------------------    0
ident
Sbjct mpayacqrptplthhntglsealeilaeaagfegsegrlltfcraasvlkalpspvttls   60
DSSP  llllhhhllllllllllhhhhhhhhhhhhhhhhhhlllhhhhhhhhhhhhhhhhhllllllllhh


DSSP  ------------------------------------------------------------
Query ------------------------------------------------------------    0
ident
Sbjct qlqglphfgehssrvvqellehgvceevervrrseryqtmklftqifgvgvktadrwyre  120
DSSP  hhlllllllhhhhhhhhhhhhhlllhhhhhhhhlhhhhhhhhhhlllllllhhhhhhhhhl


DSSP  lllllllllllhhhhhhhhhhhhhhlllhhhhhHHHHHHHHHHHHHLLLL-llLLLLEEE
Query aapysspptnlrslrdrltqvaerqgvvfgrlqRHVAXIVVAQFAATLTD-dtGAPLLLV   59
ident                                           |
Sbjct glrtlddlreqpqkltqqqkaglqhhqdlstpvLRSDVDALQQVVEEAVGqalPGATVTL  180
DSSP  llllhhhhhllhhhllhhhhhhhhhlhhhhllleEHHHHHHHHHHHHHHHHHHhhlLLLEEEE


DSSP  EHHHHHHhhhllllLLLLLLEEEEEL--------lLHHHHHHHHHHHHhhlllleeeEEL
Query KGGSSLElrrgipdSRTSKDFATVAR--------rDIELIHEQLADAGetgwegftaIFT  111
ident  ||                |                       | | |
Sbjct TGGFRRG-------KLQGHDVDFLIThpkegqeagLLPRVMCRLQDQG---------LIL  224
DSSP  LHHHHLL-------LLEELLEEEEEElllllllllLHHHHHHHHHHLL---------LEE


DSSP  LLeelllllllllLLEEEEEEEEEELLEeEEEEEEEEEEEL-LLLLllllleeeeelhhhhlll
Query APeeiaapgxpvKPRRFTAKLSYRGRaFATVPIEVSSV-EAGNadqfdtltsdalglvgv  170
ident                     |   |     |
Sbjct YH----------ERSFCIFRLPQPGS-WKAVRVDLVVApVSQF-----------------  256
DSSP  EL----------LEEEEEEEEEEELLE-EEEEEEEEEELlLLLH-----------------
```

```
DSSP  llleeeeellhHHHHhhhhhhhhllllllllllllHHHH----------------hhhhh
Query paavavpcxtiPWQIaqklhavtavleepkandraHDLV----------------dlqll  214
ident                                      |
Sbjct ----------pFALL----------------gwtgSKLFqrelrrfsrkekglwlnshgl  290
DSSP  ----------hHHHH----------------hhhlLHHHhhhhhhhhhhhhhlleellle


DSSP  hhhllhhHHHHhhHHHHHhhhhhlllLLLLLlllllhhhhhhhhhllllllllllllhhh
Query egllldaDLXPtrSACIAifearaqhPWPPRvatlphwpliyagalegldhlelartvda  274
ident
Sbjct fdpeqktFFQA--ASEED-----ifrHLGLE------------------------------  314
DSSP  eelllleELLL--LLHHH-----hhhHLLLL------------------------------


DSSP  hhhhhhhhhhhhhhllll
Query aaqavqrfvaridratkr  292
ident
Sbjct ---------ylppeqrna  323
DSSP  ---------lllhhhlll
```

No 386: Query=mol1A Sbjct=4k4iA Z-score=6.2

back to top

```
DSSP  ------------------------------------------------------------
Query ------------------------------------------------------------    0
ident
Sbjct tnhnlhiteklevlakaysvqgdkwralgyakainalksfhkpvtsyqeacsipgigkrm   60
DSSP  llllhhhhhhhhhhhhhhhhhlllhhhhhhhhhhhhhhhhllllllhhhhhhlllllhhh


DSSP  ---------------------------------------------llllllllllhhhhh
Query ---------------------------------------------aapysspptnlrslr   15
ident
Sbjct aekiieilesghlrkldhisesvpvlelfsniwgagtktaqmwyqqgfrsledirsqasl  120
DSSP  hhhhhhhhhhlllhhhhllllhhhhhhhhhlllllhhhhhhhhhlllllhhhhhhhhlll


DSSP  hhhhhhhhhllllhHHHHHHHHHHHHHHHHHLL-LLLLLLLLEEEEHHHHHHHhhlllll
Query drltqvaerqgvvfGRLQRHVAXIVVAQFAATL-TDDTGAPLLLVKGGSSLELrrgipds   74
ident                            |             |    |
Sbjct ttqqaiglkhysdfLERMPREEATEIEQTVQKAaQAFNSGLLCVACGSYRRGK-------  173
DSSP  lhhhhhhhlhhhhhHLLEEHHHHHHHHHHHHHHhHLLLLLLEEEELHHHHLLL-------


DSSP  lLLLLEEEEEL--------lLHHHHHHHHHHHHhhlllleeeEELLLEELLllllLLLLE
Query rTSKDFATVAR--------rDIELIHEQLADAGetgwegftaIFTAPEEIAapgxPVKPR  126
ident  |  |                       |   |           |
Sbjct aTCGDVDVLIThpdgrshrgIFSRLLDSLRQEG---------FLTDDLVSQeengQQQKY  224
DSSP  lEELLEEEEEElllllllllHHHHHHHHHHHLL---------LEEEEEELLllllLLLEE


DSSP  EEEEEEEEllEEEEEEEEEEEELLLLLlllleeeeelhhhhlllllleeeeellhHHHHh
Query RFTAKLSYrgRAFATVPIEVSSVEAGNadqfdtltsdalglvgvpaavavpcxtiPWQIa  186
ident      |                |
Sbjct LGVCRLPG--PGRRHRRLDIIVVPYSE--------------------------faCALL-  255
DSSP  EEEEELLL--LLLLEEEEEEEELLHHH--------------------------hhHHHH-


DSSP  hhhhhhlllllllllllllHHHHHHH-------------------------hhhhhllhH
Query qklhavtavleepkandraHDLVDLQ-------------------------llegllldA  221
ident
Sbjct ---------------yftgSAHFNRSmralaktkgmslsehalstavvrnthgckvgpgR  300
DSSP  ---------------hhhlLHHHHHHhhhhhhhlleellllleellllllllllllllllL


DSSP  HHHhhHHHHHhhhhhhllllLLLLLlllllhhhhhhhhhlllllllLLLLHHhhhhhhhh
Query DLXptRSACIaifearaqhpWPPRVatlphwpliyagalegldhleLARTVDaaaqavqr  281
ident  |                                            |
Sbjct VLP--TPTEK-------dvfRLLGL-----------pyrepaerdwLEHHHH--------  332
DSSP  EEL--LLLHH-------hhhHHLLL-----------llllhhhlllLLLLLL--------


DSSP  hhhhhhhllll
Query fvaridratkr  292
```

```
Sbjct [illegible]
DSSP  ----------l
```

No 387: Query=mol1A Sbjct=5ddyA Z-score=6.2

back to top

```
DSSP  -lllllllllhhhhhhhhhhhHHLL---------------------------------
Query -aapysspptnlrslrdrltqvaERQG---------------------------------   26
ident 
Sbjct atnhnlhiteklevlakaysvqgDKWRalgyakainalksfhkpvtsyqeacsipgigkr   60
DSSP  lllllhhhhhhhhhhhhhhhhhlLHHHhhhhhhhhhhhhllllllllhhhhhhlllllll


DSSP  ------------------------------------------------------------
Query ------------------------------------------------------------   26
ident 
Sbjct maekiieilesghlrkldhisesvpvlelfsniwgagtktaqmwyqqgfrsledirsqas  120
DSSP  hhhhhhhhhlllllllllhhhhhhhhhhhhlllllhhhhhhhhhhllllhhhhhhlll


DSSP  ------------llhHHHHHHHHHHHHHHHHHHLL-LLLLLLLLEEEEHHHHHHHHhhllll
Query ------------vvfGRLQRHVAXIVVAQFAATL-TDDTGAPLLLVKGGSSLELrrgipd   73
ident                             |             |    |
Sbjct lttqqaiglkhysdfLERMPREEATEIEQTVQKAaQAFNSGLLCVACGSYRRGK------  174
DSSP  llhhhhhhhhlhhhhLLLEEHHHHHHHHHHHHHHHHhHLLLLLLEEEELLLLLLLL------


DSSP  llLLLLEEEEEL--------lLHHHHHHHHHHHHhhlllleeeEELLL--EELLLllLLL
Query srTSKDFATVAR--------rDIELIHEQLADAGetgwegftaIFTAP--EEIAApgXPV  123
ident   |  |                       |   |           |
Sbjct -aTCGDVDVLIThpdgrshrgIFSRLLDSLRQEG---------FLTDDlvSQEEN--GQQ  222
DSSP  -lEELLEEEEEElllllllllHHHHHHHHHHHHLL---------LEEEEeeEEEEE--LLE


DSSP  LLEEEEEEEEEllEEEEEEEEEEEEL-LLLLllllleeeeelhhhhllllllleeeeellhH
Query KPRRFTAKLSYrgRAFATVPIEVSSV-EAGNadqfdtltsdalglvgvpaavavpcxtiP  182
ident         |                |
Sbjct QKYLGVCRLPG--PGRRHRRLDIIVVpYSEF---------------------------aC  253
DSSP  EEEEEEEELLL--LLLLEEEEEEEEELlLLLH---------------------------hH


DSSP  HHHhhhhhhhhllllllllllllHHHHHHH-------------------------hhhhh
Query WQIaqklhavtavleepkandraHDLVDLQ-------------------------llegl  217
ident 
Sbjct ALL----------------yftgSAHFNRSmralaktkgmslsehalstavvrnthgckv  297
DSSP  HHH----------------hhhlLHHHHHHhhhhhllllleeellllleeelleellllllee


DSSP  llhHHHHhhHHHHHhhhhhhllllLLLLLLllllhhhhhhhhhlllllllllllhhhhhh
Query lldADLXptRSACIaifearaqhpWPPRVAtlphwpliyagalegldhlelartvdaaaq  277
ident      |
Sbjct gpgRVLP--TPTEK-------dvfRLLGLP------------------------------  318
DSSP  ellLLLL--LLLHH-------hhhHHLLLL------------------------------


DSSP  hhhhhhhhhhhllll
Query avqrfvaridratkr  292
ident 
Sbjct ------yrepaerdw  327
DSSP  ------lllhhhlll
```

No 388: Query=mol1A Sbjct=4xrhB Z-score=6.2

back to top

```
DSSP  --------------------------lllllllllhhhhhhhhhhhhhhllllhHHHHH
Query --------------------------aapysspptnlrslrdrltqvaerqgvvfGRLQR   34
ident 
Sbjct sesvpvlelfsniwgagtktaqmwyqqgfrsledirsqaslttqqaiglkhysdfLERMP   60
DSSP  llhhhhhhhhhllllllhhhhhhhhhlllllhhhhhhhhllllhhhhhhhhhlhhhhLLLEE
```

```
Query [illegible]
ident         |             |    |              |  |
Sbjct REEATEIEQTVQKAaQAFNSGLLCVACGSYRRGK-------aTCGDVDVLIThpdgrshr  113
DSSP  HHHHHHHHHHHHHHHhHLLLLLLEEEELHHHHLLL-------lEELLEEEEEEllllllll


DSSP  lLHHHHHHHHHHHHhhllllleeEEELLLEELLLllLLLLLLEEEEEEEEEllEEEEEEEEE
Query rDIELIHEQLADAGetgwegftAIFTAPEEIAApgXPVKPRRFTAKLSYrgRAFATVPIE  145
ident          |   |                                |
Sbjct gIFSRLLDSLRQEG--------FLTDDLVSQEE-nGQQQKYLGVCRLPG--PGRRHRRRLD  162
DSSP  lLHHHHHHHHHHHLL--------LEEEEEEEEEE-eLLEEEEEEEEELLLL--LLLLLEEEE


DSSP  EEEL-LLLLlllleeeeelhhhhlllllleeeeellhHHHHhhhhhhhhllllllllll
Query VSSV-EAGNadqfdtltsdalglvgvpaavavpcxtiPWQIaqklhavtavleepkandr  204
ident    |
Sbjct IIVVpYSEF---------------------------aCALL----------------yft  179
DSSP  EEELlHHHH---------------------------hHHHH----------------hhh


DSSP  lHHHHHHH-------------------------hhhhhllhHHHHhhHHHHHhhhhhhll
Query aHDLVDLQ-------------------------llegllldADLXptRSACIaifearaq  239
ident                                            |
Sbjct gSAHFNRSmralaktkgmslsehalstavvrnthgckvgpgRVLP--TPTEK-------d  230
DSSP  lLHHHHHHhhhhhhhlleeellllleeelleelllllleeellEEEL--LLLHH-------h


DSSP  llLLLLLLllllhhhhhhhhhlllllllllllhhhhhhhhhhhhhhhhhllll
Query hpWPPRVAtlphwpliyagalegldhlelartvdaaaqavqrfvaridratkr  292
ident
Sbjct vfRLLGLP------------------------------------yrepaerdw  247
DSSP  hhHHLLLL------------------------------------lllhhhhll
```

No 389: Query=mol1A Sbjct=5ddmB Z-score=6.2

back to top

```
DSSP  --------------------------llllllllllhhhhhhhhhhhhhhllllhHHHHH
Query --------------------------aapyssspptnlrslrdrltqvaerqgvvfGRLQR   34
ident
Sbjct sesvpvlelfsniwgagtktaqmwyqqgfrsledirsqaslttqqaiglkhysdfLERMP   60
DSSP  llhhhhhhhhhlllllllhhhhhhhhhlllllhhhhhhhllllhhhhhhhhlhhhhHLLEE


DSSP  HHHHHHHHHHHHHLL-LLLLLLLLEEEEHHHHHHHhhlllllLLLLEEEEEL--------
Query HVAXIVVAQFAATL-TDDTGAPLLLVKGGSSLELrrgipdsrTSKDFATVAR--------   85
ident         |             |    |              |  |
Sbjct REEATEIEQTVQKAaQAFNSGLLCVACGSYRRGK-------aTCGDVDVLIThpdgrshr  113
DSSP  HHHHHHHHHHHHHHHhHLLLLLLEEEEEELHHHHLL-------lEELLEEEEEEllllllll


DSSP  lLHHHHHHHHHHHHhhllllleeEEELLLEELlLLLLLLLLEEEEEEEEEllEEEEEEEEE
Query rDIELIHEQLADAGetgwegftAIFTAPEEIaAPGXPVKPRRFTAKLSYrgRAFATVPIE  145
ident          |   |                                |
Sbjct gIFSRLLDSLRQEG--------FLTDDLVSQ-EENGQQQKYLGVCRLPG--PGRRHRRRLD  162
DSSP  lHHHHHHHHHHHHHL--------LEEEEEEEE-EEELLEEEEEEEEELLLL--LLLLLEEEE


DSSP  EEEL-LLLLlllleeeeelhhhhlllllleeeeellhHHHHhhhhhhhhllllllllll
Query VSSV-EAGNadqfdtltsdalglvgvpaavavpcxtiPWQIaqklhavtavleepkandr  204
ident    |
Sbjct IIVVpYSEF---------------------------aCALL----------------yft  179
DSSP  EEELlHHHH---------------------------hHHHH----------------hhh


DSSP  lHHHHHHH-------------------------hhhhhllhHHHHhhHHHHHhhhhhhll
Query aHDLVDLQ-------------------------llegllldADLXptRSACIaifearaq  239
ident                                            |
Sbjct gSAHFNRSmralaktkgmslsehalstavvrnthgckvgpgRVLP--TPTEK-------d  230
DSSP  lLHHHHHHhhhhhhhlleeellllleeelleelllllleeellEEEL--LLLHH-------h


DSSP  llLLLLLlllllhhhhhhhhhlllllllllllhhhhhhhhhhhhhhhhhllll
Query hpWPPRVatlphwpliyagalegldhlelartvdaaaqavqrfvaridratkr  292
ident
Sbjct vfRLLGL------------------------------------pyrepaerdw  247
```

No 390: Query=mol1A Sbjct=4qzfA Z-score=6.2

back to top

```
DSSP  ------------------------------------------------------------
Query ------------------------------------------------------------    0
ident 
Sbjct vkkisqyacqrrttlnnynqlftdaldilaendelrenegsclafmrassvlkslpfpit   60
DSSP  lllllllhhhlllllllllhhhhhhhhhhhhhhhlllhhhhhhhhhhhhhhhhlllllll


DSSP  ------------------------------------------------------------
Query ------------------------------------------------------------    0
ident 
Sbjct smkdtegipclgdkvksiiegiiedgesseakavlnderyksfklftsvfgvglktaekw  120
DSSP  lhhhhlllllllhhhhhhhhhhhhhlllhhhhhhhhlhhhhhhhhhhllllllhhhhhhh


DSSP  ----lllllllllhhhhhhhhhhhhhllllhHHHHHHHHHHHHHHHHHL-LLLLlLLL
Query ----aapysspptnlrslrdrltqvaerqgvvfGRLQRHVAXIVVAQFAAT-LTDDtGAP   55
ident                                             |
Sbjct frmgfrtlskiqsdkslrftqmqkagflyyedlVSCVNRPEAEAVSMLVKEaVVTFlPDA  180
DSSP  hhllllhhhhhhllllllhhhhhhhhlhhhhHLLEEHHHHHHHHHHHHHHhHHHHlLLL


DSSP  LEEEEHHHHHHHhhllllllLLLLEEEEEL----------lLHHHHHHHHHHHHhhllll
Query LLLVKGGSSLELrrgipdsrTSKDFATVAR----------rDIELIHEQLADAGetgweg  105
ident |    ||             |  |                             |
Sbjct LVTMTGGFRRGK-------mTGHDVDFLITspeatedeeqqLLHKVTDFWKQQG------  227
DSSP  EEEELHHHHLLL-------lEELLEEEEEEllllhhhhhhHHHHHHHHHHHHLL------


DSSP  eeeEELLLEELlllllllLLEEEEEEEEEL---LEEEEEEEEEEEEL-LLLLllllleeee
Query ftaIFTAPEEIaapgxpvKPRRFTAKLSYR---GRAFATVPIEVSSV-EAGNadqfdtlt  161
ident                          ||      |
Sbjct ---LLLYCDIL-------QKCFLILKLDHGrvhGKGWKAIRVDLVMCpYDRR--------  269
DSSP  ---LEEEEEEL-------LEEEEEEEEEHHhllLLLEEEEEEEEEEELlHHHH--------


DSSP  elhhhhllllllleeeeellhHHHHhhhhhhhhllllllllllllHHHHHHH---------
Query sdalglvgvpaavavpcxtiPWQIaqklhavtavleepkandraHDLVDLQ---------  212
ident 
Sbjct -------------------aFALL----------------gwtgSRQFERDlrryather  294
DSSP  -------------------hHHHH----------------hhhlLHHHHHHhhhhhhhh


DSSP  -----hhhhhllhhhhhhhHHHHhhhhhhhllllLLLLLLllllhhhhhhhhhlllllll
Query -----llegllldadlxptRSACiaifearaqhpWPPRVAtlphwpliyagalegldhle  267
ident 
Sbjct kmmldnhalydrtkrvfleAESE-------eeifAHLGLD--------------------  327
DSSP  leeellllleeellllleellLLLH-------hhhhHHHLLL--------------------


DSSP  llllhhhhhhhhhhhhhhhhhllll
Query lartvdaaaqavqrfvaridratkr  292
ident 
Sbjct ----------------yiepwerna  336
DSSP  ----------------lllhhhlll
```

No 391: Query=mol1A Sbjct=1kejA Z-score=6.1

back to top

```
DSSP  ------------------------------------------------------------
Query ------------------------------------------------------------    0
ident 
Sbjct kkisqyacqrrttlnnynqlftdaldilaendelrenegsclafmrassvlkslpfpits   60
DSSP  llllllhhhllllllllllhhhhhhhhhhhhhhhhllllllhhhhhhhhhhhhhlllllll


DSSP  ------------------------------------------------------------
Query ------------------------------------------------------------    0
```

```
Sbjct [illegible]
DSSP  llllllllllhhhhhhhhhhhllllllhhhhhhlllhhhhhhhhhlllllllllhhhhhh


DSSP  ---llllllllllhhhhhhhhhhhhhhllllhhhhhHHHHHHHHHHHHHL-LLLLLLLLL
Query ---aapyssspptnlrslrdrltqvaerqgvvfgrlqRHVAXIVVAQFAAT-LTDDTGAPL   56
ident                                            |               |
Sbjct rmgfrtlskiqsdkslrftqmqkagflyyedlvscvNRPEAEAVSMLVKEaVVTFLPDAL  180
DSSP  hhllllhhhllllllllllhhhhhhhhlhhhhhlleEHHHHHHHHHHHHHHhHHHHLLLLE


DSSP  EEEEHHHHHHHHhhllllllLLLLEEEEEL----------lLHHHHHHHHHHHHHhhlllle
Query LLVKGGSSLELrrgipdsrTSKDFATVAR----------rDIELIHEQLADAGetgwegf  106
ident     ||             |  |                             |
Sbjct VTMTGGFRRGK-------mTGHDVDFLITspeatedeeqqLLHKVTDFWKQQG-------  226
DSSP  EEEELHHHHLL-------lEELLLEEEEEllllllllhhhHHHHHHHHHHHLLL-------


DSSP  eeEELLLEELLLLL----------LLLLLEEEEEEEEEEL--------lEEEEEEEEEEEE
Query taIFTAPEEIAAPG----------XPVKPRRFTAKLSYR--------gRAFATVPIEVSS  148
ident                                   ||
Sbjct --LLLYCDILESTFeqpsrkvdalDHFQKCFLILKLDHGrvhseksegKGWKAIRVDLVM  284
DSSP  --LEEEEEEELLLLllllllllllLLLEEEEEEEEELLHHhlllllllllLLLLEEEEEEEEE


DSSP  L-LLLLllllleeeeelhhhhllllllleeeeellhHHHHhhhhhhhhllllllllllllHH
Query V-EAGNadqfdtltsdalglvgvpaavavpcxtiPWQIaqklhavtavleepkandraHD  207
ident
Sbjct CpYDRR---------------------------aFALL----------------gwtgSR  301
DSSP  LlHHHH---------------------------hHHHH----------------hhhlLH


DSSP  HHHHHH-------------hhhhllhhhhhhhHHHHhhhhhhhllllLLLLlllllllhhh
Query LVDLQL-------------leglllldadlxptRSACiaifearaqhpWPPRvatlphwpl  254
ident      |
Sbjct QFERDLrryatherkmmldnhalydrtkrvflEAES------eeeifAHLG---------  346
DSSP  HHHHHHhhhhhhhhhleeellllleeellllllllLLLL------hhhhhHHHL---------


DSSP  hhhhhhlllllllllllhhhhhhhhHHHHhhhhhllll
Query iyagalegldhlelartvdaaaqavQRFVaridratkr  292
ident
Sbjct -------------------------LDYI--epwerna  357
DSSP  -------------------------LLLL--lhhhlll
```

No 392: Query=mol1A Sbjct=1rztM Z-score=6.1

back to top

```
DSSP  ------------------------------------------------------------
Query ------------------------------------------------------------    0
ident
Sbjct atnhnlhiteklevlakaysvqgdkwralgyakainalksfhkpvtsyqeacsipgigkr   60
DSSP  llllllhhhhhhhhhhhhhhhhhllllhhhhhhhhhhhhhhhhhllllllllhhhhhllllllhh


DSSP  ----------------------------------------------llllllllllhhhh
Query ----------------------------------------------aapyssspptnlrsl   14
ident
Sbjct maekiieilesghlrkldhisesvpvlelfsniwgagtktaqmwyqqgfrsledirsqas  120
DSSP  hhhhhhhhhhhlllhhhhlllllhhhhhhhhlllllhhhhhhhhhlllllhhhhhhhll


DSSP  hhhhhhhhhhllllhHHHHHHHHHHHHHHHHHHHLL-LLLLLLLLEEEEHHHHHHHHhhllll
Query rdrltqvaerqgvvfGRLQRHVAXIVVAQFAATL-TDDTGAPLLLVKGGSSLELrrgipd   73
ident                             |             |    |
Sbjct lttqqaiglkhysdfLERMPREEATEIEQTVQKAaQAFNSGLLCVACGSYRRGK------  174
DSSP  llhhhhhhhhlhhhhHLLEEHHHHHHHHHHHHHHHHHhHLLLLLLLEEEELHHHHLLL------


DSSP  llLLLLLEEEEEL--------lLHHHHHHHHHHHHHhhlllleeEEELLLE-ELLLllLLLL
Query srTSKDFATVAR--------rDIELIHEQLADAGetgwegftAIFTAPE-EIAApgXPVK  124
ident   |  |                       |   |
Sbjct -aTCGDVDVLIThpdgrshrgIFSRLLDSLRQEG--------FLTDDLVsQEEN--GQQQ  223
DSSP  -lEELLEEEEEEllllllllLHHHHHHHHHHHLL--------LEEEEEEeEEEE--LLEE
```

```
DSSP  [illegible]
Query PRRFTAKLSYrgRAFATVPIEVSSV-EAGNadqfdtltsdalglvgvpaavavpcxtiPW  183
ident        |                |
Sbjct KYLGVCRLPG--PGRRHRRLDIIVVpYSEF---------------------------aCA  254
DSSP  EEEEEELLLL--LLLLLEEEEEEEL1HHHH---------------------------hHH


DSSP  HHhhhhhhhhlllllllllllHHHHHHH-----------------------hhhhhllh
Query QIaqklhavtavleepkandraHDLVDLQ-----------------------lleglllld  220
ident
Sbjct LL----------------yftgSAHFNRSmralaktkgmslsehalstavvrnthgckvg  298
DSSP  HH----------------hhhlLHHHHHHhhhhhhhlleeellllleeelleelllllleee


DSSP  hhhhHHHHHHHhhhhhhllllLLLLLLLllllhhhhhhhhhlllllllllllhhhhhhhh
Query adlxPTRSACIaifearaqhpWPPRVAtlphwpliyagalegldhlelartvdaaaqavq  280
ident
Sbjct pgrvLPTPTEK-------dvfRLLGLP---------------------------------  318
DSSP  llleELLLLHH-------hhhHHLLLL---------------------------------


DSSP  hhhhhhhhllll
Query rfvaridratkr  292
ident
Sbjct ---yrepaerdw  327
DSSP  ---lllhhhlll
```

No 393: Query=mol1A Sbjct=4p4mA Z-score=6.1

back to top

```
DSSP  -------------------------------lllllllllhhhhhhhhhhhhhhllllh
Query -------------------------------aapysspptnlrslrdrltqvaerqgvvf   29
ident
Sbjct kpklkaiqeltqvhgfgpraaaalfdregiftvdellqkadsipsltdqqrvgikyfydi   60
DSSP  lhhhhhhhhhlllllllhhhhhhhhhhhllllhhhhhhhhhhllllllhhhhhhhhlhhhh


DSSP  hhhhHHHHHHHHHHHHHH-LLLL-lLLLLLEEEEHHHHHHhhhllllLLLLLLEEEEEL--
Query grlqRHVAXIVVAQFAA-TLTD-dTGAPLLLVKGGSSLElrrgipdSRTSKDFATVAR--   85
ident                               |  |               | |
Sbjct nekiPMQESVLHENYLReKCMEvlGKDFSILICGSYRRR-------HPFSGDVDAILSrt  113
DSSP  llleEHHHHHHHHHHHHHhHHHHhhLLLEEEEEELHHHHLL-------LLEELLEEEEEEel


DSSP  -------------lLHHHHHHHHHHHhhhllllleeEEEL-LLEElllllllLLLEEEEEE
Query -------------rDIELIHEQLADAgetgwegftAIFT-APEEiaapgxpVKPRRFTAK  131
ident                     | |
Sbjct ldapplsepvaatgVLGHFVEFLESL---------KYLEaTMAQ------gPLKYMGMGR  158
DSSP  lllllllllhhhhlHHHHHHHHHHHHL---------LLEEeEEEE------lLLEEEEEEL


DSSP  EEEL--LEEEEEEEEEEEEL-LLLLllllleeeeelhhhhllllllleeeeellhHHHHhhh
Query LSYR--GRAFATVPIEVSSV-EAGNadqfdtltsdalglvgvpaavavpcxtiPWQIaqk  188
ident |  |
Sbjct LPPRinTKVYKARRVDIRLIeTKSV---------------------------pTAML---  188
DSSP  LLLLllLLLLLLEEEEEEEEElHHHH---------------------------hHHHH---


DSSP  hhhhhllllllllllllHHHH-------------------------------------hhh
Query lhavtavleepkandraHDLV-------------------------------------dlq  212
ident
Sbjct -------------tftgSKNFnvimrqaaiskgyllneyglfklgtpeeargknageelg  235
DSSP  -------------hhhlLHHHhhhhhhhhhhlleeellllleeellllhhhhlllllleell


DSSP  hhhhhllhhHHHHhhHHHHhhhhhhlllLLLLLllllllhhhhhhhhhlllllllllh
Query llegllldaDLXPtrSACIaifearaqhPWPPRvatlphwpliyagalegldhlelartv  272
ident
Sbjct vpkdeledkRVEV--RSEQ-----dvfdVLGMP---------------------------  261
DSSP  eelhhhhllLLLL--LLHH-----hhhhHLLLL---------------------------


DSSP  hhhhhhhhhhhhhhhhllll
Query daaaqavqrfvaridratkr  292
ident
```

```
DSSP             lllhhhlll
```

No 394: Query=mol1A Sbjct=4m0aA Z-score=6.1

back to top

```
DSSP  ------------------------------------------------------------
Query ------------------------------------------------------------     0
ident 
Sbjct mpayacqrptplthhntglsealeilaeaagfegsegrlltfcraasvlkalpspvttls    60
DSSP  llllhhhllllllllllhhhhhhhhhhhhhhhhlllhhhhhhhhhhhhhhhllllllllhh


DSSP  ------------------------------------------------------------
Query ------------------------------------------------------------     0
ident 
Sbjct qlqglphfgehssrvvqellehgvceevervrrseryqtmklftqifgvgvktadrwyre   120
DSSP  hhllllllllhhhhhhhhhhhhhlllhhhhhhhhlhhhhhhhhhhlllllllhhhhhhhhhl


DSSP  lllllllllhhhhhhhhhhhhhhllllhHHHHHHHHHHHHHHHHHL-LLLLLLLLLLEEE
Query aapysspptnlrslrdrltqvaerqgvvfGRLQRHVAXIVVAQFAAT-LTDDTGAPLLLV    59
ident                                           |
Sbjct glrtlddlreqpqkltqqqkaglqhhqdlSTPVLRSDVDALQQVVEEaVGQALPGATVTL   180
DSSP  llllhhhhhllhhhllhhhhhhhhlhhhhLLLEEHHHHHHHHHHHHHHhHHHHLLLLEEEE


DSSP  EHHHHHHHhhllllllLLLLEEEEEL--------lLHHHHHHHHHHHHHhhllllleeeEEL
Query KGGSSLELrrgipdsrTSKDFATVAR--------rDIELIHEQLADAGetgwegftaIFT   111
ident  ||                |                       | | |
Sbjct TGGFRRGK-------lQGHDVDFLIThpkegqeagLLPRVMCRLQDQG---------LIL   224
DSSP  LHHHHLLL-------lEELLEEEEEEllllllllHHHHHHHHHHHLL---------LEE


DSSP  LLEellllllLLLLEEEEEEEEEELLEeEEEEEEEEEEL-LLLLllllleeeeelhhhhlll
Query APEeiaapgxpVKPRRFTAKLSYRGRaFATVPIEVSSV-EAGNadqfdtltsdalglvgv   170
ident                     |   |     |
Sbjct YHQ--------FERSFCIFRLPQPGS-WKAVRVDLVVApVSQF-----------------   258
DSSP  ELL--------LLEEEEEEEEEELLE-EEEEEEEEEELlHHHH-----------------


DSSP  llleeeeellhHHHHhhhhhhhhllllllllllllHHHHHHH----------------hh
Query paavavpcxtiPWQIaqklhavtavleepkandraHDLVDLQ----------------ll   214
ident                                      |
Sbjct ----------pFALL----------------gwtgSKLFQRElrrfsrkekglwlnshgl   292
DSSP  ----------hHHHH----------------hhhlLHHHHHHhhhhhhhhhlleellllle


DSSP  hhhllhHHHHhhHHHHHhhhhhhllllLLLLLLllllhhhhhhhhhllllllllllhhh
Query eglllldADLXptRSACIaifearaqhpWPPRVAtlphwpliyagalegldhlelartvda   274
ident 
Sbjct fdpeqkTFFQ--AASEE-------difRHLGLE---------------------------   316
DSSP  eellllEELL--LLLHH-------hhhHHLLLL---------------------------


DSSP  hhhhhhhhhhhhhhllll
Query aaqavqrfvaridratkr  292
ident 
Sbjct ---------ylppeqrna  325
DSSP  ---------lllhhhlll
```

No 395: Query=mol1A Sbjct=4fhyA Z-score=6.0

back to top

```
DSSP  llllllllllhhhhhhhhhhhhhhlLLLHHHHHHHHHHHHHHHHHHHHLLLlllllLLEEEE
Query aapysspptnlrslrdrltqvaerqGVVFGRLQRHVAXIVVAQFAATLTddtgaPLLLVK    60
ident                                     |                 |
Sbjct --------shkeftkfcyevyneikISDKEFKEKRAALDTLRLCLKRIS-----PDAELV    47
DSSP  --------llhhhhhhhhhhhhhhlLLHHHHHHHHHHHHHHHHHHHHHHHL-----LLLEEE


DSSP  HHhhhhhhhllllllllLLEEEEEL--LLHHHHHHHHHHHHHHHL111eEEEELLLEELll
```

```
ident                   |                |  |
Sbjct AF--gslesglalknsdMDLCVLMDsrVQSDTIALQFYEELIAE----GFEGKFLQRA--    99
DSSP  EE--lhhhhlllllllEEEEEELLlLLHHHHHHHHHHHHHHL----LEEEEEEEEL--


DSSP  lllllLLEEEEEEEEEEllеEEEEEEEEEEEelllllllleeeeelhhhhllllllеeeee
Query pgxpvKPRRFTAKLSYrgrAFATVPIEVSsveagnadqfdtltsdalglvgvpaavavpc   178
ident                    |     |
Sbjct -----RIPIIKLTSDT---ASFQCDIGFN-------------------------------   120
DSSP  -----LEEEEEEEEEL---LLLLEEEEEL-------------------------------


DSSP  llhhhhhhHHHHHHhlllllllLLLLhhhhhhhhhhhhllhhhhhhhhhhhhhhhhhhl
Query xtipwqiaQKLHAVtavleepkaNDRAhdlvdlqllegllldadlxptrsaciaifeara   238
ident
Sbjct ----nrlaIHNTLL---------LSSY---------------------------------   134
DSSP  ----lhhhHHHHHH---------HHHH---------------------------------


DSSP  lllllllllllLHHHhHHHHHLL-LLLL-----------------llllLHHHHHH---
Query qhpwpprvatlpHWPLiYAGALEG-LDHL-----------------elarTVDAAAQ---   277
ident
Sbjct ----------tkLDAR-LKPMVLLvKHWAkrkqinspyfgtlssygyvlmVLYYLIHvik   183
DSSP  ----------hhHLLL-HHHHHHHhHHHHhhllllllhhhllllhhhhhhhHHHHHHHlll


DSSP  ------------------------------------------------------------
Query ------------------------------------------------------------   277
ident
Sbjct ppvfpnlllsplkqekivdgfdvgfddkledippsqnysslgsllhgffrfyaykfepre   243
DSSP  llllllllllllllleelleelllllhhhllllllllhhhhhhhhhhhhhhllllll


DSSP  --hhHHHH---hhhhHLLLL----------------------------------------
Query --avQRFV---aridRATKR----------------------------------------   292
ident      |
Sbjct kvvtFRRPdgyltkqEKGWTryilaiedpfeishnvgrtvsssglyrirgefmaasrlln   303
DSSP  llllLLLLllllhhHHLLLllllllllllllllllllllhhhhhhhhhhhhhhhhhhh


DSSP  ---------------
Query ---------------  292
ident
Sbjct srsypipydslfeea  318
DSSP  llllllhhhhhlll
```

No 396: Query=mol1A Sbjct=4k4iI Z-score=6.0

back to top

```
DSSP  ------------------------------------------------------------
Query ------------------------------------------------------------     0
ident
Sbjct tnhnlhiteklevlakaysvqgdkwralgyakainalksfhkpvtsyqeacsipgigkrm    60
DSSP  llllhhhhhhhhhhhhhhhhlllhhhhhhhhhhhhhhhhhllllllllhhhhhhlllllhhh


DSSP  ---------------------------------------------llllllllllhhhhh
Query ---------------------------------------------aapyssppptnlrslr    15
ident
Sbjct aekiieilesghlrkldhisesvpvlelfsniwgagtktaqmwyqqgfrsledirsqasl   120
DSSP  hhhhhhhhhllllhhhhhlllllhhhhhhhhllllllhhhhhhhhhlllllhhhhhhhlll


DSSP  hhhhhhhhhllllhhhhhHHHHHHHHHHHHH-LLLLllLLLLEEEEHHHHHHhhhllllL
Query drltqvaerqgvvfgrlqRHVAXIVVAQFAA-TLTDdtGAPLLLVKGGSSLElrrgipdS    74
ident                            |             |    |
Sbjct ttqqaiglkhysdflermPREEATEIEQTVQkAAQAfnSGLLCVACGSYRRG-------K   173
DSSP  lhhhhhhhlhhhhhlleEHHHHHHHHHHHHHhHHHHhlLLLEEEELHHHHLL-------L


DSSP  LLLLLEEEEEL--------lLHHHHHHHHHHHHhhllllееEEELLLE-ELLLllLLLLL
Query RTSKDFATVAR--------rDIELIHEQLADAGetgwegftAIFTAPE-EIAApgXPVKP   125
ident  |  |                       |   |
Sbjct ATCGDVDVLIThpdgrshrgIFSRLLDSLRQEG--------FLTDDLVsQEEN--GQQQK   223
DSSP  LEELLEEEEEElllllllllHHHHHHHHHHHHLL--------LEEEEEEeEEEE--LLEEE
```

```
DSSP  EEEEEEEEEllEEEEEEEEEEEEL-LLLLllleeeeelhhhhllllllleeeeellhHHH
Query RRFTAKLSYrgRAFATVPIEVSSV-EAGNadqfdtltsdalglvgvpaavavpcxtiPWQ  184
ident       |                |
Sbjct YLGVCRLPG--PGRRHRRLDIIVVpYSEF---------------------------aCAL  254
DSSP  EEEEEELLL--LLLLEEEEEEEELlHHHH---------------------------hHHH


DSSP  HhhhhhhhhlllllllllllHHHH-------------------hhhhhhhhllhhHHHH
Query IaqklhavtavleepkandraHDLV-------------------dlqlleglllldaDLXP  225
ident                                                          |
Sbjct L----------------yftgSAHFnrsmralaktkgmslsehalstavvrntpgrVLPT  298
DSSP  H----------------hhhlLHHHhhhhhhhhhllleeellllleeelllllllllEELL


DSSP  hhHHHHhhhhhhlllLLLLLlllllllhhhhhhhhhllllllllllllhhhHHHHhhhhhhh
Query trSACIaifearaqhPWPPRvatlphwpliyagalegldhlelartvdaAAQAvqrfvar  285
ident
Sbjct --PTEK-----dvfrLLGLP-------------------yrepaerdwlEHHH-------  325
DSSP  --LLHH-----hhhhLLLLL-------------------lllhhhllllLLLL-------


DSSP  hhhllll
Query idratkr  292
ident
Sbjct -------  325
DSSP  -------
```

No 397: Query=mol1A Sbjct=3c5gA Z-score=6.0

back to top

```
DSSP  ------------------------------------------------------------
Query ------------------------------------------------------------    0
ident
Sbjct atnhnlhiteklevlakaysvqgdkwralgyakainalksfhkpvtsyqeacsipgigkr   60
DSSP  llllhhhhhhhhhhhhhhhhllhhhhhhhhhhhhhhhllllllllhhhhhlllllllhh


DSSP  ---------------------------------------------lllllllllhhhh
Query ---------------------------------------------aapysspptnlrsl   14
ident
Sbjct maekiieilesghlrkldhisesvpvlelfsniwgagtktaqmwyqqgfrsledirsqas  120
DSSP  hhhhhhhhhhhlllhhhhhlllhhhhhhhhhllllllhhhhhhhhhlllllhhhhhhhll


DSSP  hhhhhhhhhlllllhHHHHHHHHHHHHHHHHHHLL-LLLLLLLEEEEHHHHHHHhhllll
Query rdrltqvaerqgvvfGRLQRHVAXIVVAQFAATL-TDDTGAPLLLVKGGSSLELrrgipd   73
ident                             |             |    |
Sbjct lttqqaiglkhysdfLERMPREEATEIEQTVQKAaQAFNSGLLCVACGSYRRGK------  174
DSSP  llhhhhhhhlhhhhHLLEEHHHHHHHHHHHHHHHhHLLLLLLEEEELHHHHLLL------


DSSP  llLLLLEEEEEL--------lLHHHHHHHHHHHHhhllllleeeEELLLEELLllllLLLL
Query srTSKDFATVAR--------rDIELIHEQLADAGetgwegftaIFTAPEEIAapgxPVKP  125
ident   |  |                       |   |           |
Sbjct -aTCGDVDVLIThpdgrshrgIFSRLLDSLRQEG---------FLTDDLVSQeengQQQK  224
DSSP  -lEELLEEEEEEllllllllHHHHHHHHHHHHLL---------LEEEEEELLllllLLLE


DSSP  EEEEEEEEEllEEEEEEEEEEEEELLLLLllleeeeelhhhhlllllleeeeellhHHHH
Query RRFTAKLSYrgRAFATVPIEVSSVEAGNadqfdtltsdalglvgvpaavavpcxtiPWQI  185
ident       |                |
Sbjct YLGVCRLPG--PGRRHRRLDIIVVPYSE--------------------------faCALL  256
DSSP  EEEEELLLL--LLLLLEEEEEEEELLHHH--------------------------hhHHHH


DSSP  hhhhhhhhllllllllllllHHHHHHH---------------------hhhhhllhHHH
Query aqklhavtavleepkandraHDLVDLQ---------------------lleglllldADL  223
ident                                                            |
Sbjct ----------------yftgSAHFNRSmkalaktkgmslsehalstavvhgckvgpgRVL  300
DSSP  ----------------hhhlLHHHHHHhhhhhhhlleeellllleeellllllllllLEE


DSSP  HhhHHHHHhhhhhhllllLLLLLLllllhhhhhhhhhllllllllllllhhhhhhhhhhh
Query XptRSACIaifearaqhpWPPRVAtlphwpliyagalegldhlelartvdaaaqavqrfv  283
```

```
Sbjct [illegible]
DSSP  L--LLLHH-------hhhHHLLLL------------------------------------


DSSP  hhhhhllll
Query aridratkr  292
ident
Sbjct yrepaerdw  324
DSSP  lllhhhlll
```

No 398: Query=mol1A Sbjct=2be3B Z-score=6.0

back to top

```
DSSP  lllllllllhhhhhhhhhhhhhHLLLLHHHHHHHHHHHHHHHHHHHLL--LLLL--LLLL
Query aapysspptnlrslrdrltqvaeRQGVVFGRLQRHVAXIVVAQFAATL--TDDT--GAPL   56
ident                                     |
Sbjct -----------------------TLEWEEFLDPYIQAVGELKIKLRGIrkQYRKqnKHSP   37
DSSP  -----------------------LHHHHHHHHHHHHHHHHHHHHHHHHhhHHHHllLLLL


DSSP  -EEEEhhHHHHhhhlLLLLL---------lLLLEEEEELL--LHHHHHHHHHHHHhhlll
Query -LLVKggSSLElrrgIPDSR---------tSKDFATVARR--DIELIHEQLADAGetgwe  104
ident    |              |                       |       |
Sbjct iEFVT--GRVK----PIESIkekxahdlqdIAGLRVXVQFvdDVKEVVDILHKRQ-----   86
DSSP  eEEEE--EEEL----LHHHHhhllllllllLEEEEEEELLllHHHHHHHHHHLLL-----


DSSP  leEEEELLLEEL---LLLLLllLLEEEEEEEEEL---lEEEEEEEEEEEELLllllllle
Query gfTAIFTAPEEI---AAPGXpvKPRRFTAKLSYR---gRAFATVPIEVSSVEagnadqfd  158
ident                                              |
Sbjct --DXRIIQERDYithRKASG-yRSYHVVVEYTVDtingAKTILAEIQIRTLA--------  135
DSSP  --LEEEEEEEELlllLLLLL-lLLEEEEEEEEELllllLEEEEEEEEEEEHH--------


DSSP  eeeelhhhhlllllleeeeellhhhhhHHHHHHHHLllllllllllhhhhhhhhhhhhhl
Query tltsdalglvgvpaavavpcxtipwqiAQKLHAVTAvleepkandrahdlvdlqllegll  218
ident
Sbjct ------------------------xnfWATIEHSLN------------------------  147
DSSP  ------------------------hhhHHHHHHHHH------------------------


DSSP  lhhhhhhhhhhhhhhHHHHllllllllllllllhhhhhhhhhllllllllllhhhhhhhh
Query ldadlxptrsaciaiFEARaqhpwpprvatlphwpliyagalegldhlelartvdaaaqa  278
ident                 |
Sbjct --ykyqdfpdeikkrLEIT------------------------------ariahqldeex  175
DSSP  --hhlllllhhhhhhHHHH------------------------------hhhhhhhhhhh


DSSP  hhhhhhhhhhllll
Query vqrfvaridratkr  292
ident
Sbjct geirddiqeaqalf  189
DSSP  hhhhhhhhhhhhll
```

No 399: Query=mol1A Sbjct=4qzeA Z-score=6.0

back to top

```
DSSP  ------------------------------------------------------------
Query ------------------------------------------------------------    0
ident
Sbjct kkisqyacqrrttlnnynqlftdaldilaendelrenegsclafmrassvlkslpfpits   60
DSSP  llllllhhhlllllllllhhhhhhhhhhhhhhhhlllhhhhhhhhhhhhhhhhhlllllll


DSSP  ------------------------------------------------------------
Query ------------------------------------------------------------    0
ident
Sbjct mkdtegipclgdkvksiiegiiedgesseakavlnderyksfklftsvfgvglktaekwf  120
DSSP  hhhhlllllllhhhhhhhhhhhhhlllhhhhhhhhllhhhhhhhhhhlllllhhhhhhhh
```

```
Query [illegible]
ident                                            |               |
Sbjct rmgfrtlskiqsdkslrftqmqkagflyyedlvscvNRPEAEAVSMLVKEaVVTFlPDAL  180
DSSP  hllllhhhhhhllllllhhhhhhhhlhhhhhlleEHHHHHHHHHHHHHhHHHHlLLLE


DSSP  EEEEHHHHHHHhhllllllLLLLEEEEEL----------lLHHHHHHHHHHHHhhlllle
Query LLVKGGSSLELrrgipdsrTSKDFATVAR----------rDIELIHEQLADAGetgwegf  106
ident     ||             |  |                             |
Sbjct VTMTGGFRRGK-------mTGHDVDFLITspeatedeeqqLLHKVTDFWKQQG-------  226
DSSP  EEELHHHHLLL-------lEELLEEEEEElllllhhhhhhHHHHHHHHHHHHL-------


DSSP  eeEELLLEELlllllllLLEEEEEEEEEL---lEEEEEEEEEEEEL-LLLLllllleeeee
Query taIFTAPEEIaapgxpvKPRRFTAKLSYR---gRAFATVPIEVSSV-EAGNadqfdtlts  162
ident                         ||
Sbjct --LLLYCDIL-------QKCFLILKLDHGrvhgKGWKAIRVDLVMCpYDRR---------  268
DSSP  --LEEEEEEL-------LEEEEEEEEEHHhlllLLEEEEEEEEEEELlHHHH---------


DSSP  lhhhhllllllleeeeellhHHHHhhhhhhhhllllllllllllHHHHHHH----------
Query dalglvgvpaavavpcxtiPWQIaqklhavtavleepkandraHDLVDLQ----------  212
ident
Sbjct ------------------aFALL----------------gwtgSRQFERDlrryatherk  294
DSSP  ------------------hHHHH----------------hhhlLHHHHHHhhhhhhhhhl


DSSP  ---hhhhhllhhhhhhhHHHHhhhhhhhlllLLLLLLlllllhhhhhhhhhllllllll
Query ---lleglllldadlxptRSACiaifearaqhPWPPRVatlphwpliyagalegldhlela  269
ident
Sbjct mmldnhalydrtkrvflEAES-----eeeifAHLGLD-----------------------  326
DSSP  eeellllleeellllleeeLLLL-----hhhhhHHHLLL-----------------------


DSSP  llhhhhhhhhhhhhhhhhhllll
Query rtvdaaaqavqrfvaridratkr  292
ident
Sbjct --------------yiepwerna  335
DSSP  --------------lllhhhlll
```

No 400: Query=mol1A Sbjct=4e80A Z-score=5.9

back to top

```
DSSP  llllllllllhhhhhhhhhhhhhhllLLHHHHHHHHHHHHHHHHHHHHHLLLlllllLLEEEE
Query aapysspptnlrslrdrltqvaerqgVVFGRLQRHVAXIVVAQFAATLTddtgaPLLLVK   60
ident                                     |                 |
Sbjct ---------hkeftkfcyevyneikiSDKEFKEKRAALDTLRLCLKRIS-----PDAELV   46
DSSP  ---------lhhhhhhhhhhhhhhllLHHHHHHHHHHHHHHHHHHHHHHLLL-----LLLEEE


DSSP  HHhhhhhhhlllLLLL----lLLEEEEEELLlhhHHHHHHHHHHHHHLllleEEEELLLEEl
Query GGsslelrrgipDSRT----sKDFATVARRdieLIHEQLADAGETGwegfTAIFTAPEEi  116
ident                       |           |  |
Sbjct AF------gsleSGLAlknsdMDLCVLMDD---TIALQFYEELIAE----GFEGKFLQR-   92
DSSP  EE------lhhhHLLLlllllEEEEEELLL---LHHHHHHHHHHHH----LLEEEEEEE-


DSSP  llllllLLLEEEEEEEEEEEllеEEEEEEEEEEEellllllllleeeeelhhhhlllllleee
Query aapgxpVKPRRFTAKLSYrgrAFATVPIEVSsveagnadqfdtltsdalglvgvpaavav  176
ident                      |     |
Sbjct ------ARIPIIKLTSDG---ASFQCDIGFN-----------------------------  114
DSSP  ------LLEEEEEEEELL---LLLLEEEEEL-----------------------------


DSSP  eellhhhhhhhhhhhhhllllllllLLLLhhhhhhhhhhhhllhhhhhhhhhhhhhhhhhh
Query pcxtipwqiaqklhavtavleepkaNDRAhdlvdlqlleglllldadlxptrsaciaifea  236
ident
Sbjct ----------------nrlaihntlLLSS-------------------------------  127
DSSP  ----------------lhhhhhhhhHHHH-------------------------------


DSSP  hllllllllllllLHHHhHHHH-HLLLLL-------------------llLLLLhhHHH
Query raqhpwpprvatlpHWPLiYAGA-LEGLDH-------------------leLARTvdAAA  276
ident                              |                     |
Sbjct ------------ytKLDA-RLKPmVLLVKHwakrkqinspyfgtlssygyvLMVL-yYLI  173
```

```
DSSP  HH----------------------------------------------------------
Query QA----------------------------------------------------------  278
ident
Sbjct HVikppvfpnlllsplkqekivdgfdvgfddkledippsqnysslgsllhgffrfyaykf  233
DSSP  HLlllllllhhhllllllleelleelllllhhhllllllllhhhhhhhhhhhhhll
```

```
DSSP  ----------hhHHHHHHHHLLL-------------------------------------
Query ----------vqRFVARIDRATK-------------------------------------  291
ident
Sbjct eprekvvtfrrpDGYLTKQEKGWtskdryilaiedpfeishnvgrtvsssglyrirgefm  293
DSSP  lllllllllllLLLLLHHHHLLllllllllllllllllllllllllhhhhhhhhhhh
```

```
DSSP  -----------------------l
Query -----------------------r  292
ident
Sbjct aasrllnsrsypipydslfeeapi  317
DSSP  hhhhhhlllllllhhhhhlllll
```

No 401: Query=mol1A Sbjct=1jmsA Z-score=5.9

back to top

```
DSSP  ------------------------------------------------------------
Query ------------------------------------------------------------    0
ident
Sbjct kkisqyacqrrttlnnynqlftdaldilaendelrenegsclafmrassvlkslpfpits   60
DSSP  lllllhhhllllllllllhhhhhhhhhhhhhhhlllhhhhhhhhhhhhhhhhllllllll
```

```
DSSP  ------------------------------------------------------------
Query ------------------------------------------------------------    0
ident
Sbjct mkdtegipclgdkvksiiegiiedgesseakavlnderyksfklftsvfgvglktaekwf  120
DSSP  hhhhlllllllhhhhhhhhhhhhhlllhhhhhhhhlhhhhhhhhhhlllllllhhhhhhhh
```

```
DSSP  ---llllllllllhhhhhhhhhhhhhhllllhhhhhHHHHHHHHHHHHHL-LLLLlLLLL
Query ---aapyssppptnlrslrdrltqvaerqgvvfgrlqRHVAXIVVAQFAAT-LTDDtGAPL   56
ident                                            |               |
Sbjct rmgfrtlskiqsdkslrftqmqkagflyyedlvscvNRPEAEAVSMLVKEaVVTFlPDAL  180
DSSP  hllllhhhhhhllllllhhhhhhhhlhhhhhlllLHHHHHHHHHHHHHHhHHHHlLLLE
```

```
DSSP  EEEEHHHHHHHhhllllllLLLLEEEEEL----------lLHHHHHHHHHHHHhhllle
Query LLVKGGSSLELrrgipdsrTSKDFATVAR----------rDIELIHEQLADAGetgwegf  106
ident     ||             |  |                             |
Sbjct VTMTGGFRRGK-------mTGHDVDFLITspeatedeeqqLLHKVTDFWKQQG-------  226
DSSP  EEELHHHHLLL-------lLLLLEEEEEElllllhhhhhhHHHHHHHHHHHHLL-------
```

```
DSSP  eeEELLLEELLLLL-------------lllLLEEEEEEEEEL--------lEEEEEEEEE
Query taIFTAPEEIAAPG-------------xpvKPRRFTAKLSYR--------gRAFATVPIE  145
ident                                      ||
Sbjct --LLYCDILESTFekfkqpsrkvdaldhfQKCFLILKLDHGrvhseksegKGWKAIRVD  284
DSSP  --LEEEEEEELLLLlllllllllllllllEEEEEEEEEEEHHhllllllllLLEEEEEEE
```

```
DSSP  EEEL-LLLLllllleeeeelhhhhlllllleeeeellhHHHHhhhhhhhhllllllllll
Query VSSV-EAGNadqfdtltsdalglvgvpaavavpcxtiPWQIaqklhavtavleepkandr  204
ident
Sbjct LVMCpYDRR---------------------------aFALL----------------gwt  301
DSSP  EEELlHHHH---------------------------hHHHH----------------hhh
```

```
DSSP  lHHHHHHH--------------hhhhhllhhhhhhhHHHHHhhhhhhllllLLLLlllll
Query aHDLVDLQ--------------llegllldadlxptRSACIaifearaqhpWPPRvatlp  250
ident
Sbjct gSRQFERDlrryatherkmmldnhalydrtkrvfleAESEE-------eifAHLG-----  349
DSSP  lLHHHHHHhhhhhhhhhhleeellllleeellllleellLLLHH-------hhhHHHL-----
```

```
DSSP  lhhhhhhhhhlllllllllllhhhhhhhhhhhhhhhhhhllll
```

```
ident
Sbjct -------------------------------ldyiepwerna  360
DSSP  -------------------------------lllllhhhlll
```

No 402: Query=mol1A Sbjct=3mghA Z-score=5.9

back to top

```
DSSP  ------------------------------------------------------------
Query ------------------------------------------------------------    0
ident
Sbjct atnhnlhiteklevlakaysvqgdkwralgyakainalksfhkpvtsyqeacsipgigkr   60
DSSP  llllhhhhhhhhhhhhhhhhlllhhhhhhhhhhhhhhlllllllhhhhhhllllllhh


DSSP  ----------------------------------------------llllllllllhhhh
Query ----------------------------------------------aapysspptnlrsl   14
ident
Sbjct maekiieilesghlrkldhisesvpvlelfsniwgagtktaqmwyqqgfrsledirsqas  120
DSSP  hhhhhhhhhhlllhhhhhllllhhhhhhhllllllhhhhhhhhhlllllhhhhhhhll


DSSP  hhhhhhhhhllllhHHHHHHHHHHHHHHHHH-LLLLllLLLLEEEEHHHHHHHhhllll
Query rdrltqvaerqgvvfGRLQRHVAXIVVAQFAA-TLTDdtGAPLLLVKGGSSLELrrgipd   73
ident                             |             |    |
Sbjct lttqqaiglkhysdfLERMPREEATEIEQTVQkAAQAfnSGLLCVACGSYRRGK------  174
DSSP  llhhhhhhhlhhhhHLLEEHHHHHHHHHHHHHhHHHLllLLLEEEELHHHHLLL------


DSSP  llLLLLEEEEEL--------lLHHHHHHHHHHHHhhlllleeEEELLLEELllllllLLL
Query srTSKDFATVAR--------rDIELIHEQLADAGetgwegftAIFTAPEEIaapgxpVKP  125
ident   |  |                       |   |
Sbjct -aTCGDVDVLIThpdgrshrgIFSRLLDSLRQEG--------FLTDDLVKG------ETK  219
DSSP  -lEELLEEEEEEllllllllLHHHHHHHHHHHL--------LEEEEEEEL------LLE


DSSP  EEEEEEEEEllEEEEEEEEEEEEELLLLLLllleeeeelhhhhllllleeeeellhHHHH
Query RRFTAKLSYrgRAFATVPIEVSSVEAGNAdqfdtltsdalglvgvpaavavpcxtiPWQI  185
ident       |                |
Sbjct YLGVCRLPG--PGRRHRRLDIIVVPYSEF--------------------------aCALL  251
DSSP  EEEEELLLL--LLLLLEEEEEEEELLHHHH--------------------------hHHHH


DSSP  hhhhhhhhllllllllllllHHHHHHHH----------------------hhhhllhHHH
Query aqklhavtavleepkandraHDLVDLQL----------------------leglllldADL  223
ident                                                            |
Sbjct ----------------yftgSAHFNRSMralaktkgmslsehalstavvrnckvgpgRVL  295
DSSP  ----------------hhhlLHHHHHHHhhhhhhlleeellllleeellllllllllLEE


DSSP  HhhHHHHHhhhhhhllllLLLLLlllllhhhhhhhhhllllllllllhhhhhhhhhhhh
Query XptRSACIaifearaqhpWPPRVatlphwpliyagalegldhlelartvdaaaqavqrfv  283
ident
Sbjct P--TPTEK-------dvfRLLGL-------------------------------------p  310
DSSP  L--LLLHH-------hhhHHLLL-------------------------------------l


DSSP  hhhhhllll
Query aridratkr  292
ident
Sbjct yrepaerdw  319
DSSP  lllhhhlll
```

No 403: Query=mol1A Sbjct=4k4gA Z-score=5.9

back to top

```
DSSP  ------------------------------------------------------------
Query ------------------------------------------------------------    0
ident
Sbjct tnhnlhiteklevlakaysvqgdkwralgyakainalksfhkpvtsyqeacsipgigkrm   60
DSSP  llllhhhhhhhhhhhhhhhhlllhhhhhhhhhhhhhhlllllllhhhhhhlllllhhh
```

```
DSSP                                                lllllllllllhhhh
Query ---------------------------------------------aapyssppptnlrslr   15
ident 
Sbjct aekiieilesghlrkldhisesvpvlelfsniwgagtktaqmwyqqgfrsledirsqasl  120
DSSP  hhhhhhhhhhlllhhhhlllllhhhhhhhhlllllhhhhhhhhhlllllhhhhhhhhlll


DSSP  hhhhhhhhhllllhHHHHHHHHHHHHHHHHHLL-LLLLLLLLEEEEHHHHHHHHhhlllll
Query drltqvaerqgvvfGRLQRHVAXIVVAQFAATL-TDDTGAPLLLVKGGSSLELrrgipds   74
ident                            |             |    |
Sbjct ttqqaiglkhysdfLERMPREEATEIEQTVQKAaQAFNSGLLCVACGSYRRGK-------  173
DSSP  lhhhhhhhhlhhhhHLLEEHHHHHHHHHHHHHHHhHLLLLLLLEEEELHHHHLLL-------


DSSP  lLLLLEEEEEL--------lLHHHHHHHHHHHHHhhlllleeEEELLLEELlllllLLLLE
Query rTSKDFATVAR--------rDIELIHEQLADAGetgwegftAIFTAPEEIaapgxPVKPR  126
ident  |  |                       |   |
Sbjct aTCGDVDVLIThpdgrshrgIFSRLLDSLRQEG--------FLTDDLVSQ-eengQQQKY  224
DSSP  lEELLEEEEEEllllllllHHHHHHHHHHHLL--------LEEEEEELL-llllLLLEE


DSSP  EEEEEEEEllEEEEEEEEEEEELLLLLllllleeeeelhhhhllllllleeeeellhHHHHh
Query RFTAKLSYrgRAFATVPIEVSSVEAGNadqfdtltsdalglvgvpaavavpcxtiPWQIa  186
ident      |                |
Sbjct LGVCRLPG--PGRRHRRLDIIVVPYSE--------------------------faCALL-  255
DSSP  EEEEELLL--LLLLEEEEEEEEELLHHH--------------------------hhHHHH-


DSSP  hhhhhhhllllllllllllHHHHHHHH----------------hhhhllhHHHHhhHHHH
Query qklhavtavleepkandraHDLVDLQL----------------leglllldADLXptRSAC  230
ident                                                     |
Sbjct ---------------yftgSAHFNRSMralaktkgmslsehalstavvpgRVLP--TPTE  298
DSSP  ---------------hhhlLHHHHHHHhhhhhhllleellleelllllllLLLL--LLLH


DSSP  HhhhhhhllllLLLLLlllllhhhhhhhhhllllllLLLLHhhhhhhhhhhhhhhhhll
Query IaifearaqhpWPPRVatlphwpliyagalegldhleLARTVdaaaqavqrfvaridrat  290
ident                                      |
Sbjct K-------dvfRLLGL-----------pyrepaerdwLEHHH------------------  322
DSSP  H-------hhhHHLLL-----------llllhhhlllLLLLL------------------


DSSP  ll
Query kr  292
ident 
Sbjct --  322
DSSP  --
```

No 404: Query=mol1A Sbjct=4qzhA Z-score=5.9

back to top

```
DSSP  ------------------------------------------------------------
Query ------------------------------------------------------------    0
ident 
Sbjct kisqyacqrrttlnnynqlftdaldilaendelrenegsclafmrassvlkslpfpitsm   60
DSSP  llllhhhllllllllllhhhhhhhhhhhhhhhhhlllhhhhhhhhhhhhhhhllllllllh


DSSP  ------------------------------------------------------------
Query ------------------------------------------------------------    0
ident 
Sbjct kdtegipclgdkvksiiegiiedgesseakavlnderyksfklftsvfgvglktaekwfr  120
DSSP  hhhlllllllhhhhhhhhhhhhhlllhhhhhhhhlhhhhhhhhhhlllllllhhhhhhhhh


DSSP  --lllllllllhhhhhhhhhhhhhhhllllhhhhhHHHHHHHHHHHHHHL-LLLLlLLLLE
Query --aapyssppptnlrslrdrltqvaerqgvvfgrlqRHVAXIVVAQFAAT-LTDDtGAPLL   57
ident                                           |               |
Sbjct mgfrtlskiqsdkslrftqmqkagflyyedlvscvNRPEAEAVSMLVKEaVVTFlPDALV  180
DSSP  lllllhhhhhhlllllllhhhhhhhhlhhhhhlleEHHHHHHHHHHHHHhHHHHlLLLEE


DSSP  EEEHHHHHHHHhhlllllLLLLEEEEEL----------lLHHHHHHHHHHHHhhllllee
Query LVKGGSSLELrrgipdsrTSKDFATVAR----------rDIELIHEQLADAGetgwegft  107
ident    ||             |  |                             |
```

```
[illegible]


DSSP  eEELLLEELLlllllLLLEEEEEEEEEL----lEEEEEEEEEEEEL-LLLLllllleeeee
Query aIFTAPEEIAapgxpVKPRRFTAKLSYR----gRAFATVPIEVSSV-EAGNadqfdtlts  162
ident                        ||
Sbjct -LLLYCDILE-----SQKCFLILKLDHGrvhsgKGWKAIRVDLVMCpYDRR---------  270
DSSP  -LEEEEEEEL-----LLEEEEEEEEEHHhllllLLEEEEEEEEEELlHHHH---------


DSSP  lhhhhllllllleeeeellhHHHHhhhhhhhllllllllllHHHHHHH----------
Query dalglvgvpaavavpcxtiPWQIaqklhavtavleepkandraHDLVDLQ----------  212
ident
Sbjct -------------------aFALL----------------gwtgSRQFERDlrryatherk  296
DSSP  -------------------hHHHH----------------hhhlLHHHHHHhhhhhhhhhl


DSSP  ---hhhhhllhhhhhhhHHHHhhhhhhhlllLLLLLLlllllhhhhhhhhhlllllllll
Query ---llegllldadlxptRSACiaifearaqhPWPPRVatlphwpliyagalegldhlela  269
ident
Sbjct mmldnhalydrtkrvflEAES-----eeeifAHLGLD-----------------------  328
DSSP  eeellllleeellllleeeLLLL-----hhhhhHHHLLL-----------------------


DSSP  llhhhhhhhhhhhhhhhhhllll
Query rtvdaaaqavqrfvaridratkr  292
ident
Sbjct --------------yiepwerna  337
DSSP  --------------lllhhhlll
```

No 405: Query=mol1A Sbjct=5dedG Z-score=5.9

back to top

```
DSSP  llllllllllhhhhhhhhhhhhhHLLLLHHHHHHHHHHHHHHHHHHHLLL--LLLL---LL
Query aapyssspptnlrslrdrltqvaeRQGVVFGRLQRHVAXIVVAQFAATLT--DDTG---AP   55
ident                                     |                      |
Sbjct -----------------------DKQWERFLVPYRQAVEELKVKLKGIRtlYEYEddhSP   37
DSSP  -----------------------LHHHHHHLHHHHHHHHHHHHHHHHHHHHhlLLLLlllLL


DSSP  LEEEEhhHHHHhhhlLLLLL------------------lLLLEEEEELL--LHHHHHHHH
Query LLLVKggSSLElrrgIPDSR------------------tSKDFATVARR--DIELIHEQL   95
ident    |              |                                ||    | |
Sbjct IEFVT--GRVK----PVASIlekarrksiplheietmqdIAGLRIMCQFvdDIQIVKEML   91
DSSP  LLEEE--EEEL----LHHHHhhhhllllllhhhhhhlllLEEEEEEELLhhHHHHHHHHH


DSSP  HHHHhhlllleEEEELLLEEL--LLLLlllLLEEEEEEEEEL---lEEEEEEEEEEEELL
Query ADAGetgwegfTAIFTAPEEI--AAPGxpvKPRRFTAKLSYR---gRAFATVPIEVSSVE  150
ident                                                    | |
Sbjct FARK-------DFTVVDQRDYiaEHKEsgyRSYHLVVLYPLQtvsgEKHVLVEIQIRTLA  144
DSSP  HHLL-------LLEEEEEEELllLLLLlllLLEEEEEEEEEEllllEEEEEEEEEEEEEHH


DSSP  llllllleeeeelhhhhlllllleeeeellhhhhhHHHHHHHHLLllllllllllhhhhh
Query agnadqfdtltsdalglvgvpaavavpcxtipwqiAQKLHAVTAVleepkandrahdlvd  210
ident
Sbjct --------------------------------mnfWATIEHSLYK---------------  157
DSSP  --------------------------------hhhHHHHHHHHLLL---------------


DSSP  hhhhhhhllhhhhhhhhhhhhhhHHHHllllllllllllllhhhhhhhhhlllllllll
Query lqllegllldadlxptrsaciaiFEARaqhpwpprvatlphwpliyagalegldhlelar  270
ident
Sbjct ---------------ipekvklrLQRA---------------------------------  169
DSSP  ---------------llhhhhhhHHHH---------------------------------


DSSP  lhhhhhhhhhhhhhhhhhhllll
Query tvdaaaqavqrfvaridratkr  292
ident
Sbjct -seaasrldeemseirgevqea  190
DSSP  -hhhhhhhhhhhhhhhhhhhll
```

back to top

```
DSSP  llllllllllhhhhhhhhhhhhhHLLLLHHHHHHHHHHHHHHHHHHLLL--LLLL---LL
Query aapysspptnlrslrdrltqvaeRQGVVFGRLQRHVAXIVVAQFAATLT--DDTG---AP   55
ident                                     |                      |
Sbjct -----------------------DKQWERFLVPYRQAVEELKVKLKGIRtlYEYEddhSP   37
DSSP  -----------------------LHHHHHHHHHHHHHHHHHHHHHHHHHhlLLLLlllLL


DSSP  LEEEEhhHHHHhhhlLLLLL------------------lLLLEEEEELL--LHHHHHHHH
Query LLLVKggSSLElrrgIPDSR------------------tSKDFATVARR--DIELIHEQL   95
ident    |              |                                ||    | |
Sbjct IEFVT--GRVK----PVASIlekarrksiplheietmqdIAGLRIMCQFvdDIQIVKEML   91
DSSP  LLEEE--EEEL----LHHHHhhhhhhllllhhhhhhlllLEEEEEEELLhhHHHHHHHHH


DSSP  HHHHhhlllleEEEELLLEEL---LLLLlllLLEEEEEEEEEL---lEEEEEEEEEEEEL
Query ADAGetgwegfTAIFTAPEEI---AAPGxpvKPRRFTAKLSYR---gRAFATVPIEVSSV  149
ident                                                     | |
Sbjct FARK-------DFTVVDQRDYiaeHKES-gyRSYHLVVLYPLQtvsgEKHVLVEIQIRTL  143
DSSP  HHLL-------LLEEEEEEELlllLLLL-llLLEEEEEEEEEEllllEEEEEEEEEEEEH


DSSP  LllllllleeeeelhhhhlllllleeeeellhhhhhHHHHHHHHLlllllllllllhhhh
Query EagnadqfdtltsdalglvgvpaavavpcxtipwqiAQKLHAVTAvleepkandrahdlv  209
ident
Sbjct A--------------------------------mnfWATIEHSLN---------------  156
DSSP  H--------------------------------hhhHHHHHHHHH---------------


DSSP  hhhhhhhllhhhhhhhhhhhhhhhhhhlllllllllLLLLLlhhhhhhhhhlllllllll
Query dlqllegllldadlxptrsaciaifearaqhpwpprVATLPhwpliyagalegldhlela  269
ident
Sbjct -----------------------------------yKYSGN--------ipekvklrlqr  173
DSSP  -----------------------------------hHLLLL--------llhhhhhhhhh


DSSP  llhhhhhhhhhhhhhhhhhllll
Query rtvdaaaqavqrfvaridratkr  292
ident
Sbjct aseaasrldeemseirgevqeaa  196
DSSP  hhhhhhhhhhhhhhhhhhhllll
```

No 407: Query=mol1A Sbjct=4ig8A Z-score=5.8

back to top

```
DSSP  llllllllllhhhhhhhhhhhhHHLL-LLHHHHHHHHHHHHHHHHHH-LLLL-LLLLLLE
Query aapysspptnlrslrdrltqvaERQG-VVFGRLQRHVAXIVVAQFAA-TLTD-DTGAPLL   57
ident                                | |   |      |
Sbjct -------dlrntpaksldkfieDYLLpDTCFRMQINHAIDIICGFLKeRCFRgSSYPVCV   53
DSSP  -------lllllhhhhhhhhhhHHLLlLHHHHHHHHHHHHHHHHHHHhLLLLlLLLLLLE


DSSP  -EEEHHHhhHHHHLLLlllllLLEEEEELL---------LHHHHHHHHHHHHHHLL--LL
Query -LVKGGSslELRRGIPdsrtsKDFATVARR---------DIELIHEQLADAGETGW--EG  105
ident   |  |                |                             |
Sbjct sKVVKGG-sSGKGTTLrgrsdADLVVFLSPlttfqdqlnRRGEFIQEIRRQLEACQreRA  112
DSSP  eEEEEEH-hHHHLLLLlllllEEEEEEELLlllhhhhhhHHHHHHHHHHHHHHHHHhlLL


DSSP  EEEEELlleellllllllllEEEEEEEEEL-lEEEEEEEEEEEELLLLLlllleeeeelh
Query FTAIFTapeeiaapgxpvkpRRFTAKLSYR-gRAFATVPIEVSSVEAGNadqfdtltsda  164
ident |   |               |     ||                   |
Sbjct FSVKFE-----------gnpRALSFVLSSLqlGEGVEFDVLPAFDALGQ-----------  150
DSSP  LEEELL-----------lllLLEEEEEELLllLLLEEEEEEEEELLLLL-----------


DSSP  hhhlllllleeeeellhhhhhhhhhhhhhllllllllllllhhhhhhhhhhhllhhhhhh
Query lglvgvpaavavpcxtipwqiaqklhavtavleepkandrahdlvdlqllegllldadlx  224
ident
Sbjct -----------------------------------------------------------l  151
DSSP  -----------------------------------------------------------l
```

```
DSSP  [illegible]
Query PTRSacIAIFEARAQ---------------------------------------------  239
ident 
Sbjct TGGY--KPNPQIYVKlieectdlqkegefstcftelqrdflkqrptklkslirlvkhwyq  209
DSSP  LLLL--LLLHHHHHHhhhhhhllllllhhhhhhhhhlllllllhhhhhhhhhhhhhh


DSSP  lllllllllllhHHHHH-----------------hhhllllllllllhHHHH------
Query hpwpprvatlphwPLIYA-----------------galegldhlelartvDAAA------  276
ident               |
Sbjct nckkklgklppqyALELLtvyawergsmkthfntaqgfrtvlelvinyqqLCIYwtkyyd  269
DSSP  hhhhhhlllllhhHHHHHhhhhhhhhlllllllhhhhhhhhhhhhlhhhLEELllllll


DSSP  -----------------------------------------------------hhhhhhh
Query -----------------------------------------------------qavqrfv  283
ident 
Sbjct fknpiiekylrrqltkprpvildpadptgnlgggdpkgwrqlaqeaeawlnypcfknwdg  329
DSSP  lllhhhhhhhhhhllllleeeelleeeeeellllhhhhhhhhhhhhhhlllhhhlllll


DSSP  hhhhhllll
Query aridratkr  292
ident 
Sbjct spvsswill  338
DSSP  lllllllll
```

No 408: Query=mol1A Sbjct=3nybA Z-score=5.8

back to top

```
DSSP  --------llllllllllhhhhhhhhhhhhhhlLLLHHHHHHHHHHHHHHHHHHHLLLlll
Query --------aapysspptnlrslrdrltqvaerqGVVFGRLQRHVAXIVVAQFAATLTddt   52
ident                                          |             |
Sbjct gsypwilnhdhskqkeisdwltfeikdfvayisPSREEIEIRNQTISTIREAVKQLW---   57
DSSP  lllhhhlllllllllhhhhhhhhhhhhhhhllLLHHHHHHHHHHHHHHHHHHHHLLL---


DSSP  llLLEEEEHHhhhhhhhlllLLLL----llLEEEEELL---LHHHHHHHHHHHHHHLlll
Query gaPLLLVKGGsslelrrgipDSRT----skDFATVARR---DIELIHEQLADAGETGweg  105
ident   |                           |                  ||
Sbjct --PDADLHVF------gsysTDLYlpgsdiDCVVTSELggkESRNNLYSLASHLKKK---  106
DSSP  --LLLLEEEE------llllLLLLllllleEEEELLLLlhhHHHHHHHHHHHHHHL---


DSSP  EEEE-ELLLEELllllllLLEEEEEEEEEEllleeEEEEEEEEEelllllllllleeeeelh
Query FTAI-FTAPEEIaapgxpvKPRRFTAKLSYrgraFATVPIEVSsveagnadqfdtltsda  164
ident   |
Sbjct NLATeVEVVAKA-------RVPIIKFVEPH---sGIHIAVSFE-----------------  139
DSSP  LLLLlLEEEELL-------LLEEEEEEELL---lLEEEEEELL-----------------


DSSP  hhhlllllleeeeellhhhhhhHHHHHHHlllllllLLLLhhhhhhhhhhhllhhhhh
Query lglvgvpaavavpcxtipwqiaQKLHAVTavleepkaNDRAhdlvdlqlleglllldadlx  224
ident                           |
Sbjct -------------------rtnGIEAAKL--------IREW-------------------  153
DSSP  -------------------lllLLHHHHH--------HHHH-------------------


DSSP  hhhhhhhhhhhhhllllllllllllLHHHhHHHH-HLLLL------------------l
Query ptrsaciaifearaqhpwpprvatlpHWPLiYAGA-LEGLD------------------h  265
ident 
Sbjct -------------------------lDDTP-GLRElVLIVKqflharrlnnvhtgglggf  187
DSSP  -------------------------hHLLL-LHHHhHHHHHhhhhhlllllhhhllllhh


DSSP  lllllLHHHHHH-----------------------------------------hhhhHHHH
Query lelarTVDAAAQ-----------------------------------------avqrFVAR  285
ident 
Sbjct siiclVFSFLHMhpriitneidpkdnlgvllieffelygknfgyddvalgssdgypVYFP  247
DSSP  hhhhhHHHHHHLlhhhhllllllhhhlhhhhhhhhhhhhhhlllllleeeeellllleEEEE


DSSP  hhHLLLL-----------------------------------------------------
Query idRATKR-----------------------------------------------------  292
ident 
```

```
DSSP  [illegible]


DSSP  ----------------
Query ----------------  292
ident
Sbjct fkdrlgksilgnviky  323
DSSP  hhhhllllllllllll
```

No 409: Query=mol1A Sbjct=5decA Z-score=5.8

back to top

```
DSSP  lllllllllhhhhhhhhhhhhHLLLLHHHHHHHHHHHHHHHHHHHLLL--LLLLLL-LE
Query aapysspptnlrslrdrltqvaeRQGVVFGRLQRHVAXIVVAQFAATLT--DDTGAP-LL   57
ident                                     |
Sbjct -----------------------DDKWERFLVPYRQAVEELKVKLKGIRtlYEDDHSpIE   37
DSSP  -----------------------LHHHHHHHHHHHHHHHHHHHHHHHHHHhhHLLLLLlLL


DSSP  EEEhhHHHHhhhlLLLLL------------------lLLLEEEEELL--LHHHHHHHHH
Query LVKggSSLElrrgIPDSR------------------tSKDFATVARR--DIELIHEQLAD   97
ident  |              |                                ||    | |
Sbjct FVT--GRVK----PVASIlekarrksiplheietmqdIAGLRIMCQFvdDIQIVKEMLFA   91
DSSP  EEE--EEEL----LHHHHhhhhhhllllhhhhhhlllLEEEEEEELLhhHHHHHHHHHHH


DSSP  HHhhlllleEEEELLLEELLLLLllllLLEEEEEEEEEEL---lEEEEEEEEEEEEELLllll
Query AGetgwegfTAIFTAPEEIAAPgxpvKPRRFTAKLSYR---gRAFATVPIEVSSVEagna  154
ident                     |                          | |
Sbjct RK-------DFTVVDQRDYIAG---yRSYHLVVLYPLQtvsgEKHVLVEIQIRTLA----  137
DSSP  LL-------LLEEEEEEELLLL---lLLEEEEEEEEEEelleEEEEEEEEEEEEHH----


DSSP  llleeeeelhhhhllllllleeeeellhhhhhHHHHHHHHLllllllllllhhhhhhhh
Query dqfdtltsdalglvgvpaavavpcxtipwqiAQKLHAVTAvleepkandrahdlvdlqll  214
ident
Sbjct ----------------------------mnfWATIEHSLN--------------------  149
DSSP  ----------------------------hhhHHHHHHHHH--------------------


DSSP  hhhllhhhhhhhhhhhhhhhhhhlllllllLLLLLLHhhhhhhhllllllllllhhh
Query eglllDadlxptrsaciaifearaqhpwpprVATLPHWpliyagalegldhlelartvda  274
ident
Sbjct ---------------------------ykysGNIPEKV------------klrlqrasea  170
DSSP  ---------------------------hhllLLLLHHH------------hhhhhhhhhh


DSSP  hhhhhhhhhhhhhhllll
Query aaqavqrfvaridratkr  292
ident
Sbjct asrldeemseirgevqea  188
DSSP  hhhhhhhhhhhhhhhhll
```

No 410: Query=mol1A Sbjct=4ud5A Z-score=5.8

back to top

```
DSSP  lllllllllhhhhhhhhhhhhhllLLHHHHHHHHHHHHHHHHHHHHLLLlllllLLEEEE
Query aapysspptnlrslrdrltqvaerqgVVFGRLQRHVAXIVVAQFAATLTddtgaPLLLVK   60
ident                                     |                 |
Sbjct ---------hkeftkfcyevyneikiSDKEFKEKRAALDTLRLCLKRIS-----PDAELV   46
DSSP  ---------lhhhhhhhhhhhhhhllLHHHHHHHHHHHHHHHHHHHHHHL-----LLLEEE


DSSP  HHhhhhhhhlllLLLL----lLLEEEEEELLLhHHHHHHHHHHHHHHLllleEEEELLlEEL
Query GGsslelrrgipDSRT----sKDFATVARRDiELIHEQLADAGETGwegfTAIFTApEEI  116
ident                       |           |  |
Sbjct AF------gsleSGLAlknsdMDLCVLMDSS-DTIALQFYEELIAE----GFEGAF-LQA   94
DSSP  EE------lhhhHLLLllllllEEEEEEELLLL-LLHHHHHHHHHHHL----LEEEEE-EEE


DSSP  LllllllLLEEEEEEEEEEL--lEEEEEEEEEEEellllllllleeeeelhhhhllllllle
```

```
ident |                      |     |
Sbjct A------RIPIIKLTSDTKngfGASFQCDIGFN---------------------------  121
DSSP  L------LEEEEEEEEELLlllLLLLLEEEEEL---------------------------


DSSP  eeeellhhhhhhhhhhhhhllllllllLLLLhhhhhhhhhhhllhhhhhhhhhhhhhh
Query avpcxtipwqiaqklhavtavleepkaNDRAhdlvdlqllegllldadlxptrsaciaif  234
ident 
Sbjct ------------------nrlaihntlLLSS-----------------------------  134
DSSP  ------------------lhhhhhhhhHHHH-----------------------------


DSSP  hhhlllllllllllllLHHHhHHHH-HLLLLL-------------------llLLLLhhH
Query earaqhpwpprvatlpHWPLiYAGA-LEGLDH-------------------leLARTvdA  274
ident                                |                     |
Sbjct --------------ytKLDA-RLKPmVLLVKHwakrkqinspyfgtlssygyvLMVL-yY  178
DSSP  --------------hhHHLL-LHHHHhHHHHHHhhhhlllllhhhllllhhhhhHHHH-hH


DSSP  HHHH--------------------------------------------------------
Query AAQA--------------------------------------------------------  278
ident 
Sbjct LIHVikppvfpnlllsplkqekivdgfdvgfddkledippsqnysslgsllhgffafyay  238
DSSP  HHHLllllllllhhhllllllleelleellllllhhhllllllllllhhhhhhhhhhhhhl


DSSP  ------------hhHHHHHHHHLLL-----------------------------------
Query ------------vqRFVARIDRATK-----------------------------------  291
ident 
Sbjct afeprekvvtfrrpDGYLTKQEKGWtsatehtgsadqiikdryilaiedpfeishnvgrt  298
DSSP  llllllllllllllLLLLLHHHHLLllleeeellllleeeelllllllllllllllllll


DSSP  ---------------------------------------l
Query ---------------------------------------r  292
ident 
Sbjct vsssglyrirgefmaasrllnsrsypipydslfeeapipp  338
DSSP  llhhhhhhhhhhhhhhhhhhhlllllllhhhhhlllllll
```

No 411: Query=mol1A Sbjct=4ud5B Z-score=5.8

back to top

```
DSSP  llllllllllhhhhhhhhhhhhhhlLLLHHHHHHHHHHHHHHHHHHLLLlllllLLEEEE
Query aapysspptnlrslrdrltqvaerqGVVFGRLQRHVAXIVVAQFAATLTddtgaPLLLVK   60
ident                                     |                 |
Sbjct ---------hkeftkfcyevyneikISDKEFKEKRAALDTLRLCLKRIS-----PDAELV   46
DSSP  ---------lhhhhhhhhhhhhhhlLLHHHHHHHHHHHHHHHHHHHHHL-----LLLEEE


DSSP  hhHHHHhhhlllLLLL----lLLEEEEEL---LLHHhHHHHHHHHHHHLllleEEEELLL
Query ggSSLElrrgipDSRT----sKDFATVAR---RDIElIHEQLADAGETGwegfTAIFTAP  113
ident                       |              |  |
Sbjct --AFGS----leSGLAlknsdMDLCVLMDsrvQSDT-IALQFYEELIAE----GFEGAFL   95
DSSP  --EELH----hhHLLLlllllEEEEEELLlhhHHHH-HHHHHHHHHHHL----LEEEEEE


DSSP  EELlllllllLLEEEEEEEEEL--lEEEEEEEEEEEellllllllleeeeelhhhhllll
Query EEIaapgxpvKPRRFTAKLSYR--gRAFATVPIEVSsveagnadqfdtltsdalglvgvp  171
ident                           |     |
Sbjct QAA-------RIPIIKLTSDTKngfGASFQCDIGFN------------------------  124
DSSP  EEL-------LEEEEEEEEELLlllLLLLLEEEEEL------------------------


DSSP  lleeeeellhhhhhhhhhhhhhllllllllllllhhhhhhhhhhhhhllhhhhhhhhhhh
Query aavavpcxtipwqiaqklhavtavleepkandrahdlvdlqllegllldadlxptrsaci  231
ident 
Sbjct ------------------------------------------------------------  124
DSSP  ------------------------------------------------------------


DSSP  hhhhhhlllllllllllllLHHHhHHHH-HLLLLL-------------------llLLLL
Query aifearaqhpwpprvatlpHWPLiYAGA-LEGLDH-------------------leLART  271
ident                                   |                     |
Sbjct ----nrlaihntlllssytKLDA-RLKPmVLLVKHwakrkqinspyfgtlssygyvLMVL  179
DSSP  ----lhhhhhhhhhhhhhhHHLL-LHHHHhHHHHHHhhhhlllllhhhllllhhhhhHHHH
```

```
DSSP  hhHHHHH-----------------------------------------------------
Query vdAAAQA-----------------------------------------------------  278
ident
Sbjct -yYLIHVikppvfpnlllsplkqekivdgfdvgfddkledippsqnysslgsllhgffaf  238
DSSP  -hHHHHLllllllllllllllllleelleellllllhhhllllllllllhhhhhhhhhhh


DSSP  ---------------hhHHHHHhhHLLL--------------------------------
Query ---------------vqRFVARidRATK--------------------------------  291
ident
Sbjct yayafeprekvvtfrrpDGYLTkqEKGWtsatehtgsadqiikdryilaiedpfeishnv  298
DSSP  hhllllllllllllllLLLLLhhHHLLllleeeelllllleeeellllllllllllllll


DSSP  -------------------------------------------l
Query -------------------------------------------r  292
ident
Sbjct grtvsssglyrirgefmaasrllnsrsypipydslfeeapippr  342
DSSP  lllllhhhhhhhhhhhhhhhhhhhlllllllhhhhhlllllllll
```

No 412: Query=mol1A Sbjct=4k4gE Z-score=5.8

back to top

```
DSSP  ------------------------------------------------------------
Query ------------------------------------------------------------    0
ident
Sbjct atnhnlhiteklevlakaysvqgdkwralgyakainalksfhkpvtsyqeacsipgigkr   60
DSSP  llllllhhhhhhhhhhhhhhhhllllhhhhhhhhhhhhhhhhlllllllhhhhhllllllhh


DSSP  ----------------------------------------------llllllllllhhhh
Query ----------------------------------------------aapyssspptnlrsl   14
ident
Sbjct maekiieilesghlrkldhisesvpvlelfsniwgagtktaqmwyqqgfrsledirsqas  120
DSSP  hhhhhhhhhhhlllhhhhhllllhhhhhhhhlllllllhhhhhhhhhllllllhhhhhhhll


DSSP  hhhhhhhhhhllllhhhhhHHHHHHHHHHHHH-LLLLllLLLLEEEEHHHHHHhhhllll
Query rdrltqvaerqgvvfgrlqRHVAXIVVAQFAA-TLTDdtGAPLLLVKGGSSLElrrgipd   73
ident                             |             |    |
Sbjct lttqqaiglkhysdflermPREEATEIEQTVQkAAQAfnSGLLCVACGSYRRG-------  173
DSSP  llhhhhhhhhhlhhhhhhlleEHHHHHHHHHHHHHHhHHHLllLLLEEEEELHHHHLL-------


DSSP  LLLLLLEEEEEL--------lLHHHHHHHHHHHHHhhllllleeEEELLLEElllllllLLL
Query SRTSKDFATVAR--------rDIELIHEQLADAGetgwegftAIFTAPEEiaapgxpVKP  125
ident   |  |                       |   |
Sbjct KATCGDVDVLIThpdgrshrgIFSRLLDSLRQEG--------FLTDDLVS------qQQK  219
DSSP  LLEELLEEEEEEllllllllLHHHHHHHHHHHLL--------LEEEEEEL------lLLE


DSSP  EEEEEEEEEllEEEEEEEEEEEEL-LLLLllllleeeeelhhhhlllllleeeeellhHHH
Query RRFTAKLSYrgRAFATVPIEVSSV-EAGNadqfdtltsdalglvgvpaavavpcxtiPWQ  184
ident       |                |
Sbjct YLGVCRLPG--PGRRHRRLDIIVVpYSEF---------------------------aCAL  250
DSSP  EEEEELLLL--LLLLLLEEEEEEEEL1HHHH---------------------------hHHH


DSSP  HhhhhhhhhlllllllllllHHHH-------------------------hhhhhhhhll
Query Iaqklhavtavleepkandra HDLV-------------------------dlqlleglll  219
ident
Sbjct L----------------yftgSAHFnrsmralaktkgmslsehalstavvrnthgckvgp  294
DSSP  H----------------hhhlLHHHhhhhhhhhhhllleellllleellllllllllllll


DSSP  hHHHHhhHHHHHhhhhhhlllLLLLLLlllllhhhhhhhhhllllllllllhhhhhhhh
Query dADLXptRSACIaifearaqhPWPPRVatlphwpliyagalegldhlelartvdaaaqav  279
ident    |
Sbjct gRVLP--TPTEK-----dvfrLLGLPY----------------------------------  314
DSSP  lLEEL--LLLHH-----hhhhHHLLLL----------------------------------


DSSP  hhhhhhhhhllll
Query qrfvaridratkr  292
```

```
[illegible]
DSSP  ----llhhhllll
```

No 413: Query=mol1A Sbjct=4m04A Z-score=5.8

back to top

```
DSSP  ------------------------------------------------------------
Query ------------------------------------------------------------    0
ident
Sbjct wmpayacqrptplthhntglsealeilaeaagfegsegrlltfcraasvlkalpspvttl   60
DSSP  lllllhhhllllllllllhhhhhhhhhhhhhhhhllllhhhhhhhhhhhhhhhlllllllh


DSSP  ------------------------------------------------------------
Query ------------------------------------------------------------    0
ident
Sbjct sqlqglphfgehssrvvqellehgvceevervrrseryqtmklftqifgvgvktadrwyr  120
DSSP  hhhlllllllhhhhhhhhhhhhhlllhhhhhhhhlhhhhhhhhhhhllllllhhhhhhhhh


DSSP  -lllllllllhhhhhhhhhhhhhllllhhhhhHHHHHHHHHHHHHL-LLLLlLLLLEE
Query -aapyssspptnlrslrdrltqvaerqgvvfgrlqRHVAXIVVAQFAAT-LTDDtGAPLLL   58
ident                                            |
Sbjct eglrtlddlreqpqkltqqqkaglqhhqdlstpvLRSDVDALQQVVEEaVGQAlPGATVT  180
DSSP  llllhhhhhhlhhhllhhhhhhhhlhhhhllleEHHHHHHHHHHHHHhHHHHlLLLEEE


DSSP  EEHHHHHHHhhllllllLLLLEEEEEL--------lLHHHHHHHHHHHHhhlllleeeEE
Query VKGGSSLELrrgipdsrTSKDFATVAR--------rDIELIHEQLADAGetgwegftaIF  110
ident   ||                |                       | | |
Sbjct LTGGFRRGK-------lQGHDVDFLIThpkegqeagLLPRVMCRLQDQG---------LI  224
DSSP  ELHHHHLLL-------lEELLEEEEEEllllllllHHHHHHHHHHHHLL---------LE


DSSP  LLLEellllllLLLEEEEEEEEELLEeEEEEEEEEEEL-LLLLllllleeeeelhhhhll
Query TAPEeiaapgxpVKPRRFTAKLSYRGRaFATVPIEVSSV-EAGNadqfdtltsdalglvg  169
ident                      |   |     |
Sbjct LYHQ-------aFERSFCIFRLPQPGS-WKAVRVDLVVApVSQF----------------  260
DSSP  EELL-------lLLEEEEEEEEEELLE-EEEEEEEEEELlHHHH----------------


DSSP  llllleeeeellhHHHHhhhhhhhhllllllllllllHHHHHHH----------------h
Query vpaavavpcxtiPWQIaqklhavtavleepkandraHDLVDLQ----------------l  213
ident                                       |
Sbjct -----------pFALL----------------gwtgSKLFQRElrrfsrkekglwlnshg  293
DSSP  -----------hHHHH----------------hhhlLHHHHHHhhhhhhhhhhlleellll


DSSP  hhhhllhHHHHhhHHHHHhhhhhhllllLLLLLLllllhhhhhhhhhlllllllllllhh
Query legllldADLXptRSACIaifearaqhpWPPRVAtlphwpliyagalegldhlelartvd  273
ident
Sbjct lfdpeqkTFFQ--AASEE-------difRHLGLE--------------------------  318
DSSP  eeellllEELL--LLLHH-------hhhHHLLLL--------------------------


DSSP  hhhhhhhhhhhhhhhllll
Query aaaqavqrfvaridratkr  292
ident
Sbjct ----------ylppeqrna  327
DSSP  ----------lllhhhlll
```

No 414: Query=mol1A Sbjct=5dedC Z-score=5.8

back to top

```
DSSP  llllllllllhhhhhhhhhhhhHLLLLHHHHHHHHHHHHHHHHHHHHLLL--LLLL---LL
Query aapyssspptnlrslrdrltqvaeRQGVVFGRLQRHVAXIVVAQFAATLT--DDTG---AP   55
ident                                     |                      |
Sbjct -----------------------DKQWERFLVPYRQAVEELKVKLKGIRtlYEYEddhSP   37
DSSP  -----------------------LLLLLLLLHHHHHHHHHHHHHHHHHHHhhHHLLlllLL
```

```
Query [illegible]
ident    |              |                                ||    | |
Sbjct IEFVT--GRVK----PVASIlekarrksiplheietmqdIAGLRIMCQFvdDIQIVKEML   91
DSSP  LLEEE--EEEL----LHHHHhhhhhhllllhhhhhhlllLEEEEEEELLhhHHHHHHHHH


DSSP  HHHHhhlllleEEEELLLEEL---LLLLLllLLEEEEEEEEEL---lEEEEEEEEEEEEL
Query ADAGetgwegfTAIFTAPEEI---AAPGXpvKPRRFTAKLSYR---gRAFATVPIEVSSV  149
ident                                                     | |
Sbjct FARK-------DFTVVDQRDYiaeHKESG-yRSYHLVVLYPLQtvsgEKHVLVEIQIRTL  143
DSSP  HHLL-------LLEEEEEEELlllLLLLL-lLLEEEEEEEEEEllllEEEEEEEEEEEEH


DSSP  LllllllleeeeelhhhhlllllleeeeellhhhhhHHHHHHHHLlllllllllllhhhh
Query EagnadqfdtltsdalglvgvpaavavpcxtipwqiAQKLHAVTAvleepkandrahdlv  209
ident
Sbjct A--------------------------------mnfWATIEHSLN---------------  156
DSSP  H--------------------------------hhhHHHHHHHHH---------------


DSSP  hhhhhhhhllhhhhhhhhhhhhhhhhhhhllllllLLLLLLLHhhhhhhhhlllllllll
Query dlqllegllldadlxptrsaciaifearaqhpwppRVATLPHWpliyagalegldhlela  269
ident
Sbjct --------------------------------ykySGNIPEKV-------------klrl  171
DSSP  --------------------------------hhhLLLLLHHH-------------hhhh


DSSP  llhhhhhhhhhhhhhhhhhllll
Query rtvdaaaqavqrfvaridratkr  292
ident
Sbjct qraseaasrldeemseirgevqe  194
DSSP  hhhhhhhhhhhhhhhhhhhhhll
```

No 415: Query=mol1A Sbjct=4fhpA Z-score=5.7

back to top

```
DSSP  lllllllllhhhhhhhhhhhhhhllLLHHHHHHHHHHHHHHHHHHHLLLlllllLLEEEE
Query aapyssppptnlrslrdrltqvaerqgVVFGRLQRHVAXIVVAQFAATLTddtgaPLLLVK   60
ident                                     |                 |
Sbjct --------shkeftkfcyevyneikiSDKEFKEKRAALDTLRLCLKRIS-----PDAELV   47
DSSP  --------llhhhhhhhhhhhhhhllLHHHHHHHHHHHHHHHHHHHLLL-----LLLEEE


DSSP  HHhhhhhhhlllLLLL---lLLEEEEEL--LLHHHHHHHHHHHHHHLllleEEEELLLEE
Query GGsslelrrgipDSRT---sKDFATVAR--RDIELIHEQLADAGETGwegfTAIFTAPEE  115
ident                      |             |  |
Sbjct AF-----gslesGLALknsdMDLCVLMDsrVQSDTIALQFYEELIAE----GFEGKFLQR   98
DSSP  EE-----lhhhhLLLLllllEEEEEELLllLLHHHHHHHHHHHHHHL----LEEEEEEEE


DSSP  LlllllllLLEEEEEEEEELLE--EEEEEEEEEEelllllllllleeeeelhhhhllllll
Query IaapgxpvKPRRFTAKLSYRGR--AFATVPIEVSsveagnadqfdtltsdalglvgvpaa  173
ident                         |     |
Sbjct A-------RIPIIKLTSDTKNGfgASFQCDIGFN--------------------------  125
DSSP  L-------LEEEEEEEEELLLLllLLLLEEEEEL--------------------------


DSSP  eeeeellhhhhhhHHHHHHhllllllllLLLLhhhhhhhhhhhhllhhhhhhhhhhhhhh
Query vavpcxtipwqiaQKLHAVtavleepkaNDRAhdlvdlqllegllldadlxptrsaciai  233
ident
Sbjct ---------nrlaIHNTLL---------LSSY----------------------------  139
DSSP  ---------lhhhHHHHHH---------HHHH----------------------------


DSSP  hhhhlllllllllllllLHHHhHHHHHLL-LLLL-----------------llllLHHHH
Query fearaqhpwpprvatlpHWPLiYAGALEG-LDHL-----------------elarTVDAA  275
ident
Sbjct ---------------tkLDAR-LKPMVLLvKHWAkrkqinspyfgtlssygyvlmVLYYL  183
DSSP  ---------------hlLLLL-HHHHHHHhHHHHhhlllllhhhllllhhhhhhhHHHHH


DSSP  HH----------------------------------------------------------
Query AQ----------------------------------------------------------  277
ident
Sbjct IHvikppvfpnlllsplkqekivdgfdvgfddkledippsqnysslgsllhgffrfyayk  243
```

```
DSSP  -------hhHHHH---hhhhHLLLL-----------------------------------
Query -------avQRFV---aridRATKR-----------------------------------  292
ident           |
Sbjct feprekvvtFRRPdgyltkqEKGWTryilaiedpfeishnvgrtvsssglyrirgefmaa  303
DSSP  lllllllllLLLLlllllhhHHLLLllllllllllllllllllllhhhhhhhhhhhhh


DSSP  --------------------
Query --------------------  292
ident
Sbjct srllnsrsypipydslfeea  323
DSSP  hhhhhlllllllhhhhhlll
```

No 416: Query=mol1A Sbjct=5bpcA Z-score=5.7

back to top

```
DSSP  ------------------------------------------------------------
Query ------------------------------------------------------------    0
ident
Sbjct tlnggitdmltelanfeknvsqaihkynayrkaasviakyphkiksgaeakklpgvgtki   60
DSSP  lllhhhhhhhhhhhhhhhhhllllhhhhhhhhhhhhhhhhhlllllllhhhhhlllllhhh


DSSP  ------------------------------------------------llllllllllhh
Query ------------------------------------------------aapysspptnlr   12
ident
Sbjct aekideflatgklrklekirqddtsssinfltrvsgigpsaarkfvdegiktledlrkne  120
DSSP  hhhhhhhhhhlllhhhhhhhhlhhhhhhhhhlllllllhhhhhhhhhlllllhhhhhhlh


DSSP  hhhhhhhhhhhhllllhhhhhHHHHHHHHHHHHHH-LLLLllLLLLEEEEHHHHHHHhhll
Query slrdrltqvaerqgvvfgrlqRHVAXIVVAQFAA-TLTDdtGAPLLLVKGGSSLELrrgi   71
ident                                                |  |
Sbjct dklnhhqriglkyfgdfekriPREEMLQMQDIVLnEVKKvdSEYIATVCGSFRRGA----  176
DSSP  hhllhhhhhhhhlhhhhhlleEHHHHHHHHHHHHHhHHHHhlLLLEEEEELHHHHLLL----


DSSP  llllLLLLEEEEEL--------lLHHHHHHHHHHHHhhlllleeEEELLLEElllllllL
Query pdsrTSKDFATVAR--------rDIELIHEQLADAGetgwegftAIFTAPEEiaapgxpV  123
ident      | |                     |||             |
Sbjct ---eSSGDMDVLLThpsftseskLLHQVVEQLQKVH--------FITDTLSK------gE  219
DSSP  ---lEELLEEEEEEllllllllLHHHHHHHHHHHLL--------LEEEEEEE------lL


DSSP  LLEEEEEEEEElLEEEEEEEEEEEEL-LLLLllllleeeeelhhhhllllllleeeeellhH
Query KPRRFTAKLSYrGRAFATVPIEVSSV-EAGNadqfdtltsdalglvgvpaavavpcxtiP  182
ident         |           |
Sbjct TKFMGVCQLPS-EKEYPHRRIDIRLIpKDQY---------------------------yC  251
DSSP  LEEEEEELLLL-LLLLLLLEEEEEEEELlHHHH---------------------------hH


DSSP  HHHhhhhhhhhhllllllllllllHHHH-------------hhhhhhhhllhHHHHHHhhHH
Query WQIaqklhavtavleepkandraHDLV-------------dlqllegllldADLXPtrSA  229
ident                         |                            |
Sbjct GVL----------------yftgSDIFnknmrahalekgftineytirpagEPLPV--DS  293
DSSP  HHH----------------hhhlLHHHhhhhhhhhhllllleellllleellLLLLL--LL


DSSP  HHhhhhhhlllLLLLLLlllllhhhhhhhhhlllllllllllhhhhhhhhhhhhhhhhl
Query CIaifearaqhPWPPRVatlphwpliyagalegldhlelartvdaaaqavqrfvaridra  289
ident
Sbjct EK-----difdYIQWKY--------------------------------------repkd  310
DSSP  HH-----hhhhHLLLLL--------------------------------------llhhh


DSSP  lll
Query tkr  292
ident
Sbjct rse  313
DSSP  lll
```

back to top

```
DSSP  ------------------------------------------------------------
Query ------------------------------------------------------------    0
ident 
Sbjct tlnggitdmltelanfeknvsqaihkynayrkaasviakyphkiksgaeakklpgvgtki   60
DSSP  lllhhhhhhhhhhhhhhhllllhhhhhhhhhhhhhhhllllllllhhhhhlllllhhh


DSSP  ------------------------------------------------llllllllllhh
Query ------------------------------------------------aapysspptnlr   12
ident 
Sbjct aekideflatgklrklekirqddtsssinfltrvsgigpsaarkfvdegiktledlrkne  120
DSSP  hhhhhhhhhhlllhhhhhhhhlhhhhhhhhhlllllllhhhhhhhhhlllllhhhhhllh


DSSP  hhhhhhhhhhhhllllhHHHHHHHHHHHHHHHHHL-LLLLLLLLLEEEEHHHHHHHHhhll
Query slrdrltqvaerqgvvfGRLQRHVAXIVVAQFAAT-LTDDTGAPLLLVKGGSSLELrrgi   71
ident                                                |  |
Sbjct dklnhhqriglkyfgdfEKRIPREEMLQMQDIVLNeVKKVDSEYIATVCGSFRRGA----  176
DSSP  hhllhhhhhhhhlhhhhLLLEEHHHHHHHHHHHHHHhHHHHLLLLEEEEELHHHHLLL----


DSSP  llllLLLLEEEEEL--------lLHHHHHHHHHHHHhhlllleeEEELLLEELllllllL
Query pdsrTSKDFATVAR--------rDIELIHEQLADAGetgwegftAIFTAPEEIaapgxpV  123
ident      | |                     |||           |
Sbjct ---eSSGDMDVLLThpsftseskLLHQVVEQLQKVH--------FITDTLSKG------E  219
DSSP  ---lEELLEEEEEEllllllllHHHHHHHHHHHLL--------LEEEEEEEL------L


DSSP  LLEEEEEEEEEElleEEEEEEEEEEEL-LLLLllleeeeelhhhhllllllleeeeellhH
Query KPRRFTAKLSYrgrAFATVPIEVSSV-EAGNadqfdtltsdalglvgvpaavavpcxtiP  182
ident         |           |
Sbjct TKFMGVCQLPS---EYPHRRIDIRLIpKDQY---------------------------yC  249
DSSP  LEEEEEELLLL---LLLLEEEEEEELlHHHH---------------------------hH


DSSP  HHHhhhhhhhhllllllllllllHHHHHHHH-------hhhhllhHHHHHhhHHHHHhhh
Query WQIaqklhavtavleepkandraHDLVDLQL-------leglllldADLXPtrSACIAife  235
ident                         |                      |
Sbjct GVL----------------yftgSDIFNKNMrahtinkytirplgEPLPV--DSEKD---  288
DSSP  HHH----------------hhhlLHHHHHHHhhlllllllllllLLLLL--LLHHH---


DSSP  hhllllLLLLllllllhhhhhhhhhllllllllllllhhhhhhhhhhhhhhhhllll
Query araqhpWPPRvatlphwpliyagalegldhlelartvdaaaqavqrfvaridratkr  292
ident 
Sbjct ----ifDYRE-----------------------------------------pkdrse  300
DSSP  ----hhHLLL-----------------------------------------hhhlll
```

No 418: Query=mol1A Sbjct=5dedB Z-score=5.7

back to top

```
DSSP  llllllllllhhhhhhhhhhhhhLLLLHHHHHHHHHHHHHHHHHHHHLLL--LLLL---LL
Query aapysspptnlrslrdrltqvaerQGVVFGRLQRHVAXIVVAQFAATLT--DDTG---AP   55
ident                                     |                      |
Sbjct -----------------------dKQWERFLVPYRQAVEELKVKLKGIRtlYEYEddhSP   37
DSSP  -----------------------lHHHHHHHHHHHHHHHHHHHHHHHHHHHhhHHHLlllLL


DSSP  LEEEEhhHHHHhhhlLLLLL------------------lLLLEEEEELL--LHHHHHHHH
Query LLLVKggSSLElrrgIPDSR------------------tSKDFATVARR--DIELIHEQL   95
ident    |              |                                ||    | |
Sbjct IEFVT--GRVK----PVASIlekarrksiplheietmqdIAGLRIMCQFvdDIQIVKEML   91
DSSP  LLEEE--EEEL----LHHHHhhhhhllllllllllllllLEEEEEEEELLhhHHHHHHHHH


DSSP  HHHHhhlllleEEEELLLEEL---LLLLLllLLEEEEEEEEEL---lEEEEEEEEEEEEL
Query ADAGetgwegfTAIFTAPEEI---AAPGXpvKPRRFTAKLSYR---gRAFATVPIEVSSV  149
ident                                                     | |
Sbjct FARK-------DFTVVDQRDYiaeHKESG-yRSYHLVVLYPLQtvsgEKHVLVEIQIRTL  143
DSSP  HHLL-------LLEEEEEEELlllLLLLL-lLLEEEEEEEEEEllllEEEEEEEEEEEEH
```

```
DSSP  [illegible]
Query Eagnadqfdtltsdalglvgvpaavavpcxtipwqi AQKLHAVTAvleepkandrahdlv  209
ident
Sbjct A--------------------------------mnfWATIEHSLN---------------  156
DSSP  H--------------------------------hhhHHHHHHHHH---------------
```

```
DSSP  hhhhhhhhllhhhhhhhhhhhhhhHHHHllllllllllllllhhhhhhhhhlllllllll
Query dlqlleglllldadlxptrsaciaiFEARaqhpwpprvatlphwpliyagalegldhlela  269
ident
Sbjct -------------yksgnekvklrLQRA--------------------------------  171
DSSP  -------------hllllllhhhhHHHH--------------------------------
```

```
DSSP  llhhhhhhhhhhhhhhhhhllll
Query rtvdaaaqavqrfvaridratkr  292
ident
Sbjct ---seaasrldeemseirgevaa  191
DSSP  ---hhhhhhhhhhhhhhhhhlll
```

No 419: Query=mol1A Sbjct=4qziA Z-score=5.7

back to top

```
DSSP  ------------------------------------------------------------
Query ------------------------------------------------------------    0
ident
Sbjct kkisqyacqrrttlnnynqlftdaldilaendelrenegsclafmrassvlkslpfpits   60
DSSP  llllllhhhllllllllllhhhhhhhhhhhhhhhhllllhhhhhhhhhhhhhhhhlllllll
```

```
DSSP  ------------------------------------------------------------
Query ------------------------------------------------------------    0
ident
Sbjct mkdtegipclgdkvksiiegiiedgesseakavlnderyksfklftsvfgvglktaekwf  120
DSSP  hhhhllllllllhhhhhhhhhhhhhhllllhhhhhhhhhlhhhhhhhhhhhhllllllhhhhhhhh
```

```
DSSP  ---lllllllllhhhhhhhhhhhhhhllllhHHHHHHHHHHHHHHHHHHLL-LLLLLLLL
Query ---aapyssspptnlrslrdrltqvaerqgvvfGRLQRHVAXIVVAQFAATL-TDDTGAPL   56
ident                                            |               |
Sbjct rmgfrtlskiqsdkslrftqmqkagflyyedlVSCVNRPEAEAVSMLVKEAvVTFLPDAL  180
DSSP  hlllllhhhhhhllllllllhhhhhhhhlhhhhHLLEEHHHHHHHHHHHHHHHhHHHLLLLE
```

```
DSSP  EEEEHHHHHHHhhllllllLLLLEEEEEL----------lLHHHHHHHHHHHHhhlllle
Query LLVKGGSSLELrrgipdsrTSKDFATVAR----------rDIELIHEQLADAGetgwegf  106
ident     ||             |  |                             |
Sbjct VTMTGGFRRGK-------mTGHDVDFLITspeatedeeqqLLHKVTDFWKQQG-------  226
DSSP  EEELHHHHLLL-------lEELLEEEEEElllllhhhhhhHHHHHHHHHHHHLL-------
```

```
DSSP  eeEELLLEELLlllllLLLEEEEEEEEEEL--lEEEEEEEEEEEEEL-LLLLllllleeeeel
Query taIFTAPEEIAapgxpVKPRRFTAKLSYR--gRAFATVPIEVSSV-EAGNadqfdtltsd  163
ident                         ||
Sbjct --LLLYCDILE-----AQKCFLILKLDHGrvgKGWKAIRVDLVMCpYDRR----------  269
DSSP  --LEEEEEEEL-----LEEEEEEEEEEHHhllLLEEEEEEEEEEELlHHHH----------
```

```
DSSP  hhhhllllllleeeeellhHHHHhhhhhhhhllllllllllllHHHHHHH-----------
Query alglvgvpaavavpcxtiPWQIaqklhavtavleepkandraHDLVDLQ-----------  212
ident
Sbjct -----------------aFALL----------------gwtgSRQFERDlrryatherkm  296
DSSP  -----------------hHHHH----------------hhhlLHHHHHHhhhhhhhhhle
```

```
DSSP  ---hhhhhllhhhhhhhHHHHHhhhhhhllllLLLLLLllllhhhhhhhhhlllllllll
Query ---lleglllldadlxptRSACIaifearaqhpWPPRVAtlphwpliyagalegldhlela  269
ident
Sbjct mldnhalydrtkrvfleAESEE-------eifAHLGLD----------------------  327
DSSP  eellllleeellllleeelLLLHH-------hhhHHHLLL----------------------
```

```
DSSP  llhhhhhhhhhhhhhhhhhllll
Query rtvdaaaqavqrfvaridratkr  292
ident
```

```
DSSP  [illegible]
```

No 420: Query=mol1A Sbjct=3hiyB Z-score=5.6

back to top

```
DSSP  lllllllllhhhhhhhhhhhhhhllLLHHHHHHHHHHHHHHHHHHHLLllllllLLEEEE
Query aapysspptnlrslrdrltqvaerqgVVFGRLQRHVAXIVVAQFAATLtddtgaPLLLVK   60
ident                                   |     |                 |
Sbjct -------vakrefirgxxahyraslpPPEHSVVIHELQKRVLDIGXLA-----vNKAHVE   48
DSSP  -------lhhhhhhhhhhhhhhllllLLLHHHHHHHHHHHHHHHHHHH-----lLLLEEE


DSSP  HHhhhhhhhllllllllLLEEEEEL-------------lLHHHHHHHHHHHHHHLlllEE
Query GGsslelrrgipdsrtsKDFATVAR-------------rDIELIHEQLADAGETGwegFT  107
ident                   |     |
Sbjct LF--gshvsgfctphsdADISLTYRnfspwlqgxervdeQNNKRXTRFGKEASAX---GX  103
DSSP  EE--lhhhhllllllllEEEEEELLlllhhhlllhhhhhHHHHHHHHHHHHHHHH---LL


DSSP  EEELLLEELllllllllLEEEEEEEEElleEEEEEEEEEEellllllllleeeeelhhhh
Query AIFTAPEEIaapgxpvkPRRFTAKLSYrgrAFATVPIEVSsveagnadqfdtltsdalgl  167
ident
Sbjct EDVRYIRAR--------IPVVQFTDGV---TGIHCDVSIG--------------------  132
DSSP  EEEEEELLL--------LLEEEEELLL---LLLEEEEEEL--------------------


DSSP  llllleeeeellhhhhhhhHHHHHHhllllllllLLLLhhhhhhhhhhhhllhhhhhhhh
Query vgvpaavavpcxtipwqiaQKLHAVtavleepkaNDRAhdlvdlqllegllldadlxptr  227
ident
Sbjct --------------niggvENSKIL---------CAIR----------------------  147
DSSP  --------------lhhhhHHHHHH---------HHHH----------------------


DSSP  hhhhhhhhhllllllllllllllLHHHHHHHHHL-lLLLL-----------------lll
Query saciaifearaqhpwpprvatlpHWPLIYAGALE-gLDHL-----------------ela  269
ident                          |  |
Sbjct ----------------------qVFPDFYGAYIHlvKAWGkareviaperstfnsftvtt  185
DSSP  ----------------------hLLHHHHHHHHHhhHHHHhhlllllhhhllllhhhhhh


DSSP  lLHHHHHH----------------------------------------------------
Query rTVDAAAQ----------------------------------------------------  277
ident
Sbjct xALXVLQElgllpvfskptgefgeltvadaexllqefklppiydslhdddeklgeavffc  245
DSSP  hHHHHHHHllllllllllllllllllhhhhhhhhhhllllhhhhhllllhhhhhhhhhhh


DSSP  ------------------hHHHHHHHhhHLLL----------------------------
Query ------------------aVQRFVARidRATK----------------------------  291
ident
Sbjct lqrfaeyyakydfsagtvsLIHPRRHrtVYERvvrrhlellgsrkrlewekhiaehkedg  305
DSSP  hhhhhhhhhhlllllllllLLLLLLLhhHHHHhhhhhhhhhhhhhhhhhhhhhhhlhhhl


DSSP  ------------------------------------------------------------
Query ------------------------------------------------------------  291
ident
Sbjct pldendfsasxqnettqrpsnspyvvedfvnyvncgrrvqasrvrhiqqefnrlrexlid  365
DSSP  lllhhhhhhhhhhhhhllllllllllllllllllllllllhhhhhhhhhhhhhhhhhhll


DSSP  ----------------l
Query ----------------r  292
ident
Sbjct keselkfdevfresdtv  382
DSSP  lhhhllhhhhlllllll
```

No 421: Query=mol1A Sbjct=4nktA Z-score=5.6

back to top

```
DSSP  lllllllllhhhhhhhhhhhhhhllLLHHHHHHHHHHHHHHHHHHHLLllllllLLEEEE
```

```
ident                                     |                 |
Sbjct ------dmshkeftkfcyevyneikiSDKEFKEKRAALDTLRLCLKRIS-----PDAELV   49
DSSP  ------llllhhhhhhhhhhhhhhllLHHHHHHHHHHHHHHHHHHHLLL-----LLLEEE


DSSP  HHhhhhhhhllllllllLLEEEEElllhhHHHHHHHHHHHHLllleEEEELLLEelllll
Query GGsslelrrgipdsrtsKDFATVArrdieLIHEQLADAGETGwegfTAIFTAPEeiaapg  120
ident                   |           |  |
Sbjct AF--gslesglalknsdMDLCVLM-----TIALQFYEELIAE----GFEGKFLQ------   92
DSSP  EE--lhhhhllllllllEEEEEEL-----LLHHHHHHHHHHL----LEEEEELL------


DSSP  lllLLEEEEEEEEELl--lEEEEEEEEEEEellllllllleeeeelhhhhlllllleeeee
Query xpvKPRRFTAKLSYR--gRAFATVPIEVSsveagnadqfdtltsdalglvgvpaavavpc  178
ident                    |     |
Sbjct -raRIPIIKLTSDTKngfGASFQCAIGFN-------------------------------  120
DSSP  -llLLLLEEEEEELLlllLLLLLEEEEEL-------------------------------


DSSP  llhhhhhhHHHHHHhllllllllLLLLhhhhhhhhhhhhllhhhhhhhhhhhhhhhhhhl
Query xtipwqiaQKLHAVtavleepkaNDRAhdlvdlqlleglllldadlxptrsaciaifeara  238
ident
Sbjct ----nrlaIHNTLL---------LSSY---------------------------------  134
DSSP  ----lhhhHHHHHH---------HHHH---------------------------------


DSSP  llllllllllllLHHHhHHHHHLL-LLLL-----------------llllLHHHHHH---
Query qhpwpprvatlpHWPLiYAGALEG-LDHL-----------------elarTVDAAAQ---  277
ident
Sbjct ----------tkLDAR-LKPMVLLvKHWAkrkqinspyfgtlssygyvlmVLYYLIHvik  183
DSSP  ----------hhHLLL-HHHHHHHhHHHHhhlllllhhhllllhhhhhhhHHHHHHHlll


DSSP  ------------------------------------------------------------
Query ------------------------------------------------------------  277
ident
Sbjct ppvfpnlllsplkqekivdgfdvgfddkledippsqnysslgsllhgffrfyaykfepre  243
DSSP  llllllllllllllllee lleelllllllhhhllllllllllhhhhhhhhhhhhhhllllll


DSSP  --hhHHHH---hhhhHLLLL----------------------------------------
Query --avQRFV---aridRATKR----------------------------------------  292
ident      |
Sbjct kvvtFRRPdgyltkqEKGWTsryilaiedpfeishnvgrtvsssglyrirgefmaasrll  303
DSSP  llllLLLLlllllhhHHLLLllllllllllllllllllllllhhhhhhhhhhhhhhhhh


DSSP  ----------------
Query ----------------  292
ident
Sbjct nsrsypipydslfeea  319
DSSP  llllllllhhhhhlll
```

No 422: Query=mol1A Sbjct=4nkuA Z-score=5.6

back to top

```
DSSP  llllllllllhhhhhhhhhhhhhhllLLHHHHHHHHHHHHHHHHHHLLLlllllLLEEEE
Query aapysspptnlrslrdrltqvaerqgVVFGRLQRHVAXIVVAQFAATLTddtgaPLLLVK   60
ident                                     |                 |
Sbjct ------dmshkeftkfcyevyneikiSDKEFKEKRAALDTLRLCLKRIS-----PDAELV   49
DSSP  ------llllhhhhhhhhhhhhhhllLHHHHHHHHHHHHHHHHHHHLLL-----LLLEEE


DSSP  HHhhhhhhhlllLLLL---lLLEEEEELllhhHHHHHHHHHHHHLllleEEEELLLEEll
Query GGsslelrrgipDSRT---sKDFATVARrdieLIHEQLADAGETGwegfTAIFTAPEEia  117
ident                      |           |  |
Sbjct AF-----gslesGLALknsdMDLCVLMD----TIALQFYEELIAE----GFEGKFLQR--   94
DSSP  EE-----lhhhhLLLLllllEEEEEELL----LHHHHHHHHHHHL----LEEEEELLL--


DSSP  llllllLLEEEEEEEEELLE--EEEEEEEEEEellllllllleeeeelhhhhllllllee
Query apgxpvKPRRFTAKLSYRGR--AFATVPIEVSsveagnadqfdtltsdalglvgvpaava  175
ident                       |     |
Sbjct -----aRIPIIKLTSDTKNGfgASFQCAIGFN----------------------------  121
DSSP  -----lLLLLEEEEEELLLLllLLLLEEEEEL----------------------------
```

```
DSSP  eeellhhhhhhHHHHHHhlllllllLLLLhhhhhhhhhhhhllhhhhhhhhhhhhhhhhh
Query vpcxtipwqiaQKLHAVtavleepkaNDRAhdlvdlqllegllldadlxptrsaciaife  235
ident 
Sbjct -------nrlaIHNTLL---------LSSY------------------------------  135
DSSP  -------lhhhHHHHHH---------HHHH------------------------------


DSSP  hhllllllllllllllHHHHhHHHHLLL-LLLL------------------lllLHHHHH
Query araqhpwpprvatlphWPLIyAGALEGL-DHLE------------------larTVDAAA  276
ident                            |  |
Sbjct --------------tkLDAR-LKPMVLLvKHWAkrkqinspyfgtlssygyvlmVLYYLI  180
DSSP  --------------hhHLLL-HHHHHHHhHHHHhhlllllhhhllllhhhhhhhHHHHHH


DSSP  H-----------------------------------------------------------
Query Q-----------------------------------------------------------  277
ident 
Sbjct Hvikppvfpnlllsplkqekivdgfdvgfddkledippsqnysslgsllhgffrfyaykf  240
DSSP  Hlllllllllllllllllleelleellllllhhhlllllllllhhhhhhhhhhhhhhhhll


DSSP  ------hhHHHH----hhhhHLLLL-----------------------------------
Query ------avQRFV----aridRATKR-----------------------------------  292
ident          |
Sbjct eprekvvtFRRPdgyltkqeKGWTSryilaiedpfeishnvgrtvsssglyrirgefmaa  300
DSSP  llllllllLLLLlllllhhhHLLLLlllllllllllllllllllllhhhhhhhhhhhhhh


DSSP  --------------------
Query --------------------  292
ident 
Sbjct srllnsrsypipydslfeea  320
DSSP  hhhhhlllllllhhhhhlll
```

No 423: Query=mol1A Sbjct=3wfpH Z-score=5.6

back to top

```
DSSP  llllllllllhhhhhhhhhhhhhhllllhhhhhhhhhhHHHHHHHHLLLLllLLLLEEEE
Query aapysspptnlrslrdrltqvaerqgvvfgrlqrhvaxIVVAQFAATLTDdtGAPLLLVK   60
ident                                              |
Sbjct ----------------------------------lnfyLSYFDDVAKVLP--REHYCFIV   24
DSSP  ----------------------------------llllLLHHHHHHHHLL--LLLLEEEE


DSSP  HHHHHHHHHLLLlllllLLEEEEEELLLHHHHHHHHHHHhhlllleeeEELLLEELllll
Query GGSSLELRRGIPdsrtsKDFATVARRDIELIHEQLADAGetgwegftaIFTAPEEIaapg  120
ident ||       | |      |       |        |
Sbjct GGWVRDRILGEP-vgynIDVDFLTTADPVELAKNFAKRI---------GGHFFVFE----   70
DSSP  LHHHHHHHLLLL-llleEEEEEEELLLHHHHHHHHHHHH---------LLEEEEEL----


DSSP  llLLLEEEEEEEeelleEEEEEEEEEEEL-----LLLL---------------llllEEE
Query xpVKPRRFTAKLsyrgrAFATVPIEVSSV-----EAGN---------------adqfDTL  160
ident            |              |       |                      | |
Sbjct --PTIASVVLHL-----PPYRYRFDFSPLkgkdlEKALiedlkerdftanaiavnldDVL  123
DSSP  --LLEEEEEEEL-----LLLEEEEEEEEELllllhHHHHhhhhlllllhhhlleeehhHHH


DSSP  EelhhhhlllllleeeeelLHHH-----------hhhhhhhHHHLlllllllLLLLHhhh
Query TsdalglvgvpaavavpcxTIPW-----------qiaqklhAVTAvleepkaNDRAHdlv  209
ident                                                       |
Sbjct S-----------igakqtiVYDPtggikdleqgllrpvsieNLKR------dPVRVL---  163
DSSP  L-----------lllllllEELLllhhhhhhlleellllhhHHHH------lLLHHH---


DSSP  hhhhhhhllhhhhhhhhhhhhhhhhhhhllllllllllllllhhhHHHHHHLLLLlllll
Query dlqllegllldadlxptrsaciaifearaqhpwpprvatlphwplIYAGALEGLDhlela  269
ident 
Sbjct ---------------------------------------------RGFRIAIEKN----l  174
DSSP  ---------------------------------------------HHHHHHHHLL----l


DSSP  LLHHHHHHHHHH------------------------------------------------
Query RTVDAAAQAVQR------------------------------------------------  281
```

```
Sbjct [illegible]
DSSP  EELHHHHHHHHHlhhhhhhllhhhhhhhhhhhhhlllhhhhhhhhhhllhhhhhlhhhhh


DSSP  ------------------------------------------------------------
Query ------------------------------------------------------------  281
ident
Sbjct lrevkdpldehtlktleyleqviedrakylsaellenfgkkrvlgeftdvellkwgalfh  294
DSSP  hhllllhhhhhhhhhhhhhhhhlhhhhllhhhhhllllleellleehhhhhhhhhhll


DSSP  ------------------------------------------------------------
Query ------------------------------------------------------------  281
ident
Sbjct digkpqtfvtfyehdkvgaqivreigerlrwgdeatefvaklvrhhlrpfflreafkkge  354
DSSP  llllllllllllhhhhhhhhhhhhhhhllllhhhhhhhhhhhhlllhhhhhhhhhhlll


DSSP  ----------------------------------------------hhhhhhhllll
Query ----------------------------------------------fvaridratkr  292
ident
Sbjct lkrrgmanfwrecgdiaphlfllsiadamasgdeeedikalmetiaelesfnrnemk  411
DSSP  llhhhhhhhhhhllllhhhhhhhhhhhhhhllllhhhhhhhhhhhhhhhhhhhhllll
```

No 424: Query=mol1A Sbjct=4e7xD Z-score=5.6

back to top

```
DSSP  llllllllllhhhhhhhhhhhhhhllLLHHHHHHHHHHHHHHHHHHHLLLlllllLLEEEE
Query aapysspptnlrslrdrltqvaerqgVVFGRLQRHVAXIVVAQFAATLTddtgaPLLLVK   60
ident                                     |                 |
Sbjct ---------hkeftkfcyevyneikiSDKEFKEKRAALDTLRLCLKRIS-----PDAELV   46
DSSP  ---------lhhhhhhhhhhhhhhllLHHHHHHHHHHHHHHHHHHHHHHHL-----LLLEEE


DSSP  hhHHHHhhhlllLLLL----lLLEEEEELLlhhHHHHHHHHHHHHHLllleEEEELLLEEl
Query ggSSLElrrgipDSRT----sKDFATVARRdieLIHEQLADAGETGwegfTAIFTAPEEi  116
ident                       |           |  |
Sbjct --AFGS----leSGLAlknsdMDLCVLMDD---TIALQFYEELIAE----GFEGKFLQR-   92
DSSP  --EELH----hhHLLLlllllEEEEEELLL---LHHHHHHHHHHHL----LLEEEEEEE-


DSSP  llllllLLLEEEEEEEEEElleeEEEEEEEEEellllllllleeeeelhhhhllllllleee
Query aapgxpVKPRRFTAKLSYrgraFATVPIEVSsveagnadqfdtltsdalglvgvpaavav  176
ident                            |
Sbjct ------ARIPIIKLTSDT----SFQCDIGFN-----------------------------  113
DSSP  ------LLEEEEEEEELL----LLLEEEEEL-----------------------------


DSSP  eellhhhhhhhhhhhhhlllllllllllhHHHhhhhhhllhhhhhhhhhhhhhhhh
Query pcxtipwqiaqklhavtavleepkandrahDLVDlqlleglllldadlxptrsaciaifea  236
ident                                |
Sbjct ----------------------nrlaihntLLLS--------------------------  125
DSSP  ----------------------lhhhhhhhHHHH--------------------------


DSSP  hlllllllllllllHHHHhHHHHLL--LLLL-----------------lLLLLHHHHHH
Query raqhpwpprvatlphWPLIyAGALEG--LDHL-----------------eLARTVDAAAQ  277
ident
Sbjct -----------sytkLDAR-LKPMVLlvKHWAkrkqinspyfgtlssygyVLMVLYYLIH  173
DSSP  -----------hhhhHLLL-HHHHHHhhHHHHhhllllllhhhllllhhhhHHHHHHHHHH


DSSP  ------------------------------------------------------------
Query ------------------------------------------------------------  277
ident
Sbjct vikppvfpnlllsplkqekivdgfdvgfddkledippsqnysslgsllhgffrfyaykfe  233
DSSP  llllllllllllllllllleelleellllllhhhlllllllllhhhhhhhhhhhhhhlll


DSSP  -----hhHHHH----hhhHHLLLL------------------------------------
Query -----avQRFV----ariDRATKR------------------------------------  292
ident         |
Sbjct prekvvtFRRPdgyltkqEKGWTSatehdryilaiedpfeishnvgrtvsssglyrirge  293
DSSP  llllleelLLLLlllllhhHHLLLLllllllllleellllllllllllllhhhhhhhhh
```

```
Query ---------------------------  292
ident
Sbjct fmaasrllnsrsypipydslfeeapip  320
DSSP  hhhhhhhhllllllllhhhhhllllll
```

No 425: Query=mol1A Sbjct=5dedH Z-score=5.5

back to top

```
DSSP  lllllllllhhhhhhhhhhhhhHLLLLHHHHHHHHHHHHHHHHHHHLLL----lLLLLL-
Query aapysspptnlrslrdrltqvaeRQGVVFGRLQRHVAXIVVAQFAATLT----dDTGAP-   55
ident                                     |
Sbjct -----------------------DKQWERFLVPYRQAVEELKVKLKGIRtlyeyEDDHSp   37
DSSP  -----------------------LHHHHHHHHHHHHHHHHHHHHHHHHHHhhlllLLLLLl

DSSP  LEEEEhhHHHHhhhlLLLLL------------------lLLLEEEEELL--LHHHHHHHH
Query LLLVKggSSLElrrgIPDSR------------------tSKDFATVARR--DIELIHEQL   95
ident    |              |                                ||    | |
Sbjct IEFVT--GRVK----PVASIlekarrksiplheietmqdIAGLRIMCQFvdDIQIVKEML   91
DSSP  LLEEE--EEEL----LHHHHhhhhhhllllhhhhhhlllLEEEEEEEELLhhHHHHHHHHH

DSSP  HHHHhhlllleEEEELLLEEL--LLLLlllLLEEEEEEEEEL---lEEEEEEEEEEEELL
Query ADAGetgwegfTAIFTAPEEI--AAPGxpvKPRRFTAKLSYR---gRAFATVPIEVSSVE  150
ident                                                    | |
Sbjct FARK-------DFTVVDQRDYiaEHKEsgyRSYHLVVLYPLQtvsgEKHVLVEIQIRTLA  144
DSSP  HHLL-------LLEEEEEEELllLLLLlllLLEEEEEEEEELllllLEEEEEEEEEEEHH

DSSP  lllllleeeeelhhhhlllllleeeeellhhhhhHHHHHHHHLllllllllllhhhhh
Query agnadqfdtltsdalglvgvpaavavpcxtipwqiAQKLHAVTAvleepkandrahdlvd  210
ident
Sbjct --------------------------------mnfWATIEHSLN----------------  156
DSSP  --------------------------------hhhHHHHHHHHH----------------

DSSP  hhhhhhhllhhhhhhhhhhhhhhhhhlllllllLLLLLLHhhhhhhhhlllllllll
Query lqllegllldadlxptrsaciaifearaqhpwpprVATLPHWpliyagalegldhlelar  270
ident
Sbjct -------------------------------ykysGNIPEKV-------------klrlq  172
DSSP  -------------------------------hhllLLLLHHH-------------hhhhh

DSSP  lhhhhhhhhhhhhhhhhhllll
Query tvdaaaqavqrfvaridratkr  292
ident
Sbjct raseaasrldeemseirgevaa  194
DSSP  hhhhhhhhhhhhhhhhhhhlll
```

No 426: Query=mol1A Sbjct=4e8fA Z-score=5.5

back to top

```
DSSP  lllllllllhhhhhhhhhhhhhhlLLLHHHHHHHHHHHHHHHHHHHHLLLlllllLLEEEE
Query aapysspptnlrslrdrltqvaerqGVVFGRLQRHVAXIVVAQFAATLTddtgaPLLLVK   60
ident                                     |                 |
Sbjct ------pnshkeftkfcyevyneikISDKEFKEKRAALDTLRLCLKRIS-----PDAELV   49
DSSP  ------llllhhhhhhhhhhhhhhhlLLHHHHHHHHHHHHHHHHHHHHHHL-----LLLEEE

DSSP  HHhhhhhhhllllllllLLEEEEELLlHHHHHHHHHHHHHHLllleEEEELLLEEllllll
Query GGsslelrrgipdsrtsKDFATVARRdIELIHEQLADAGETGwegfTAIFTAPEEiaapg  120
ident                   |           |  |
Sbjct AF--gslesglalknsdMDLCVLMDS-SDTIALQFYEELIAE----GFEGKFLQR-----   97
DSSP  EE--lhhhhllllllllEEEEEELLL-LLLHHHHHHHHHHL----LEEEEEEEE-----

DSSP  lllLLEEEEEEEEEELL--EEEEEEEEEEEellllllllleeeeelhhhhlllllleeeee
Query xpvKPRRFTAKLSYRG--RAFATVPIEVSsveagnadqfdtltsdalglvgvpaavavpc  178
ident                    |     |
Sbjct --aRIPIIKLTSDTKNgfGASFQCDIGFN-------------------------------  124
```

```
DSSP  llhhhhhhHHHHHHhlllllllLLLLhhhhhhhhhhhhllhhhhhhhhhhhhhhhhhhl
Query xtipwqiaQKLHAVtavleepkaNDRAhdlvdlqlleglllddadlxptrsaciaifeara  238
ident
Sbjct ----nrlaIHNTLL---------LSSY---------------------------------  138
DSSP  ----lhhhHHHHHH---------HHHH---------------------------------


DSSP  lllllllllllLHHHhHHHHHLL-LLLL-----------------llllLHHHHHH---
Query qhpwpprvatlpHWPLiYAGALEG-LDHL-----------------elarTVDAAAQ---  277
ident
Sbjct ----------tkLDAR-LKPMVLLvKHWAkrkqinspyfgtlssygyvlmVLYYLIHvik  187
DSSP  ----------hhHLLL-HHHHHHHhHHHHhhllllllllllllhhhhhhHHHHHHHlll


DSSP  ------------------------------------------------------------
Query ------------------------------------------------------------  277
ident
Sbjct ppvfpnlllsplkqekivdgfdvgfddkledippsqnysslgsllhgffrfyaykfepre  247
DSSP  lllllllllllllllleelleellllllhhhlllllllllhhhhhhhhhhhhhhllllll


DSSP  --hhHHHH---hhhhHLLLL----------------------------------------
Query --avQRFV---aridRATKR----------------------------------------  292
ident      |
Sbjct kvvtFRRPdgyltkqEKGWTsdryilaiedpfeishnvgrtvsssglyrirgefmaasrl  307
DSSP  leelLLLLlllllhhHHLLLllllllleellllllllllllllllhhhhhhhhhhhhhhh


DSSP  --------------------
Query --------------------  292
ident
Sbjct lnsrsypipydslfeeapip  327
DSSP  hlllllllhhhhhllllll
```

No 427: Query=mol1A Sbjct=4e7xA Z-score=5.5

back to top

```
DSSP  llllllllllhhhhhhhhhhhhhhllLLHHHHHHHHHHHHHHHHHHHHLLLllllllLLEEEE
Query aapysspptnlrslrdrltqvaerqgVVFGRLQRHVAXIVVAQFAATLTddtgaPLLLVK   60
ident                                     |                 |
Sbjct ---------hkeftkfcyevyneikiSDKEFKEKRAALDTLRLCLKRIS-----PDAELV   46
DSSP  ---------llhhhhhhhhhhhhhhllLHHHHHHHHHHHHHHHHHHHHHHHHL-----LLLEEE


DSSP  HHhhhhhhhlllLLLL----lLLEEEEEELllhHHHHHHHHHHHHHHLllleEEEELLLEEl
Query GGsslelrrgipDSRT----sKDFATVARrdiELIHEQLADAGETGwegfTAIFTAPEEi  116
ident                       |           |  |
Sbjct AF------gsleSGLAlknsdMDLCVLMD---DTIALQFYEELIAE----GFEGKFLQR-   92
DSSP  EE------lhhhHLLLlllllEEEEEELL---LLHHHHHHHHHHHL----LEEEEEEEE-


DSSP  llllllLLLEEEEEEEEEelLEEE--EEEEEEEEellllllllleeeeelhhhhllllllle
Query aapgxpVKPRRFTAKLSyrGRAF--ATVPIEVSsveagnadqfdtltsdalglvgvpaav  174
ident                              |
Sbjct ------ARIPIIKLTSD--TGFGasFQCDIGFN---------------------------  117
DSSP  ------LLEEEEEEEEE--LLLLllLLEEEEEL---------------------------


DSSP  eeeellhhhhhhhhhhhhhllllllllLLLLhhhhhhhhhhhhllhhhhhhhhhhhhhhh
Query avpcxtipwqiaqklhavtavleepkaNDRAhdlvdlqlleglllddadlxptrsaciaif  234
ident
Sbjct -----------------nrlaihntllLSSY-----------------------------  131
DSSP  -----------------lhhhhhhhhhHHHH-----------------------------


DSSP  hhhllllllllllllLHHHhHHHHHL-lLLLL-----------------lLLLLHHHHH
Query earaqhpwpprvatlpHWPLiYAGALE-gLDHL-----------------eLARTVDAAA  276
ident
Sbjct --------------tkLDAR-LKPMVLlvKHWAkrkqinspyfgtlssygyVLMVLYYLI  176
DSSP  --------------hhHLLL-HHHHHHhhHHHHhhlllllhhhllllhhhhHHHHHHHHH


DSSP  H-----------------------------------------------------------
```

```
Sbjct Hvikppvfpnlllsplkqekivdgfdvgfddkledippsqnysslgsllhgffrfyaykf  236
DSSP  Hllllllllllllllllllleelleellllllhhhllllllllllhhhhhhhhhhhhhhll


DSSP  ------hhHHHH----hhhhHLLLL-----------------------------------
Query ------avQRFV----aridRATKR-----------------------------------  292
ident          |
Sbjct eprekvvtFRRPdgyltkqeKGWTSdryilaiedpfeishnvgrtvsssglyrirgefma  296
DSSP  llllleelLLLLlllllhhhHLLLLllllleellllllllllllllllhhhhhhhhhhhh


DSSP  -----------------------
Query -----------------------  292
ident
Sbjct asrllnsrsypipydslfeeapi  319
DSSP  hhhhhllllllllhhhhhlllll
```

No 428: Query=mol1A Sbjct=4e80B Z-score=5.5

back to top

```
DSSP  lllllllllhhhhhhhhhhhhhhhlLLLHHHHHHHHHHHHHHHHHHLLLlllllLLEEEE
Query aapyssspptnlrslrdrltqvaerqGVVFGRLQRHVAXIVVAQFAATLTddtgaPLLLVK   60
ident                                     |                 |
Sbjct ---------hkeftkfcyevyneikISDKEFKEKRAALDTLRLCLKRIS-----PDAELV   46
DSSP  ---------lhhhhhhhhhhhhhhlLLHHHHHHHHHHHHHHHHHHLLLL-----LLLEEE


DSSP  HHhhhhhhlllllllllLLEEEEELllhHHHHHHHHHHHHHLllleEEEELLLEElllll
Query GGsslelrrgipdsrtsKDFATVARrdiELIHEQLADAGETGwegfTAIFTAPEEiaapg  120
ident                   |           |  |
Sbjct AF--gslesglalknsdMDLCVLMD---DTIALQFYEELIAE----GFEGKFLQR-----   92
DSSP  EE--lhhhhllllllllEEEEEELL---LHHHHHHHHHHHHH----LLLEEEEEE-----


DSSP  lllLLEEEEEEEeelleeeEEEEEEEEelllllllllleeeeelhhhhlllllleeeeell
Query xpvKPRRFTAKLsyrgrafATVPIEVSsveagnadqfdtltsdalglvgvpaavavpcxt  180
ident                        |
Sbjct --aRIPIIKLTS-------FQCDIGFN---------------------------------  110
DSSP  --lLEEEEEEEL-------LEEEEEEL---------------------------------


DSSP  hhhhhhHHHHHHhllllllllLLLLhhhhhhhhhhhhllhhhhhhhhhhhhhhhhhhlll
Query ipwqiaQKLHAVtavleepkaNDRAhdlvdlqllegllldadlxptrsaciaifearaqh  240
ident
Sbjct --nrlaIHNTLL---------LSSY-----------------------------------  124
DSSP  --lhhhHHHHHH---------HHHH-----------------------------------


DSSP  lllllllllllHHHHhHHHHLLL-LLLL------------------lllLHHHHHH----
Query pwpprvatlphWPLIyAGALEGL-DHLE------------------larTVDAAAQ----  277
ident                       |  |
Sbjct ---------tkLDAR-LKPMVLLvKHWAkrkqinspyfgtlssygyvlmVLYYLIHvikp  174
DSSP  ---------hhHLLL-HHHHHHHhHHHHhhlllllhhhllllhhhhhhhHHHHHHHllll


DSSP  ------------------------------------------------------------
Query ------------------------------------------------------------  277
ident
Sbjct pvfpnlllsplkqekivdgfdvgfddkledippsqnysslgsllhgffrfyaykfeprek  234
DSSP  llllhhhlllllllleelleellllllhhhllllllllllhhhhhhhhhhhhhhlllllll


DSSP  -hhHHHH---hhhhHLLLL-----------------------------------------
Query -avQRFV---aridRATKR-----------------------------------------  292
ident     |
Sbjct vvtFRRPdgyltkqEKGWTsryilaiedpfeishnvgrtvsssglyrirgefmaasrlln  294
DSSP  lllLLLLlllllhhHHLLLlllllllllllllllllllllllhhhhhhhhhhhhhhhhhh


DSSP  -----------------
Query -----------------  292
ident
Sbjct srsypipydslfeeapi  311
DSSP  lllllllhhhhhlllll
```

No 429: Query=mol1A Sbjct=4lzdA Z-score=5.5

back to top

```
DSSP  ------------------------------------------------------------
Query ------------------------------------------------------------   0
ident 
Sbjct wmpayacqrptplthhntglsealeilaeaagfegsegrlltfcraasvlkalpspvttl  60
DSSP  lllllhhhlllllllllhhhhhhhhhhhhhhhhlllhhhhhhhhhhhhhhhhlllllllh


DSSP  ------------------------------------------------------------
Query ------------------------------------------------------------   0
ident 
Sbjct sqlqglphfgehssrvvqellehgvceevervrrseryqtmklftqifgvgvktadrwyr 120
DSSP  hhhllllllllhhhhhhhhhhhhhlllhhhhhhhhlhhhhhhhhhhhlllllhhhhhhhhh


DSSP  -llllllllllhhhhhhhhhhhhhhllllhhhhhHHHHHHHHHHHHHL-LLLLlLLLLEE
Query -aapysspptnlrslrdrltqvaerqgvvfgrlqRHVAXIVVAQFAAT-LTDDtGAPLLL  58
ident                                            |
Sbjct eglrtlddlreqpqkltqqqkaglqhhqdlstpvLRSDVDALQQVVEEaVGQAlPGATVT 180
DSSP  llllhhhhhllhhhllhhhhhhhhlhhhhhlleEHHHHHHHHHHHHHhHHHHlLLLEEE


DSSP  EEHHHHHHHhhlllllLLLLEEEEEL--------lLHHHHHHHHHHHHhhlllleeeEE
Query VKGGSSLELrrgipdsrTSKDFATVAR--------rDIELIHEQLADAGetgwegftaIF 110
ident   ||                |                       | | |
Sbjct LTGGFRRGK-------lQGHDVDFLIThpkegqeagLLPRVMCRLQDQG---------LI 224
DSSP  ELHHHHLLL-------lEELLEEEEEEllllllllHHHHHHHHHHHHLL---------LE


DSSP  LLLEELLLL-lLLLLLEEEEEEEEEELLEeEEEEEEEEEEL-LLLLllllleeeeelhhhhl
Query TAPEEIAAP-gXPVKPRRFTAKLSYRGRaFATVPIEVSSV-EAGNadqfdtltsdalglv 168
ident                       |   |     |
Sbjct LYHQHQHSChmDAFERSFCIFRLPQPGS-WKAVRVDLVVApVSQF--------------- 268
DSSP  EEEEEELLLllLLLEEEEEEEEEEEELLE-EEEEEEEEEELlHHHH---------------


DSSP  lllllleeeeellhHHHHhhhhhhhhlllllllllllHHHHHHHhhhhhllhhhhhhhhh
Query gvpaavavpcxtiPWQIaqklhavtavleepkandraHDLVDLQllegllldadlxptrs 228
ident                                        |
Sbjct ------------pFALL----------------gwtgSKLFQRE-lrrfsrkekglwlns 299
DSSP  ------------hHHHH----------------hhhlLHHHHHH-hhhhhhhhhlleell


DSSP  HHHH------hHHHH--lllllLLLLLllllhhhhhhhhhllllllllllhhhhhhhhh
Query ACIA------iFEAR--aqhpwPPRVAtlphwpliyagalegldhlelartvdaaaqavq 280
ident            | |
Sbjct HGLFdpeqktfFQAAseedifrHLGLE--------------------------------- 326
DSSP  LLEEelllleeLLLLlhhhhhhHLLLL---------------------------------


DSSP  hhhhhhhhllll
Query rfvaridratkr  292
ident 
Sbjct ---ylppeqrna  335
DSSP  ---lllhhhlll
```

No 430: Query=mol1A Sbjct=3wfpB Z-score=5.5

back to top

```
DSSP  llllllllllhhhhhhhhhhhhhhllllhhhhhhhhhhHHHHHHHHLLLLllLLLLEEEE
Query aapysspptnlrslrdrltqvaerqgvvfgrlqrhvaxIVVAQFAATLTDdtGAPLLLVK  60
ident                                              |
Sbjct ----------------------------------lnfyLSYFDDVAKVLP--REHYCFIV  24
DSSP  ----------------------------------llllLLHHHHHHHHHLL--LLLLEEEE


DSSP  HHHHHHHHHLLLlllllLLEEEEEELLLHHHHHHHHHHHHHhhlllleeeEELLLEELllll
Query GGSSLELRRGIPdsrtsKDFATVARRDIELIHEQLADAGetgwegftaIFTAPEEIaapg 120
ident ||       | |      |       |        |
```

```
DSSP  llLLLEEEEEEEeelleEEEEEEEEEEEL-----LLLL---------------llllEEE
Query xpVKPRRFTAKLsyrgrAFATVPIEVSSV-----EAGN---------------adqfDTL  160
ident            |              |       |                      | |
Sbjct --PTIASVVLHL-----PPYRYRFDFSPLkgkdlEKALiedlkerdftanaiavnldDVL  123
DSSP  --LLEEEEEEEL-----LLLEEEEEEEEELllllhHHHHhhhhlllllhhhlleeehhHHH


DSSP  EelhhhhllllllleeeeelLHHH-----------hhhhhhhHHHLlllllllLLLLHhhh
Query TsdalglvgvpaavavpcxTIPW-----------qiaqklhAVTAvleepkaNDRAHdlv  209
ident                                                       |
Sbjct S-----------igakqtiVYDPtggikdleqgllrpvsieNLKR------dPVRVL---  163
DSSP  L-----------lllllllEELLllhhhhhhlleellllhhHHHH------lLLHHH---


DSSP  hhhhhhhhllhhhhhhhhhhhhhhhhhhhllllllllllllllhhhHHHHHHLLLLlllll
Query dlqllegllldadlxptrsaciaifearaqhpwpprvatlphwplIYAGALEGLDhlela  269
ident
Sbjct ---------------------------------------------RGFRIAIEKN----l  174
DSSP  ---------------------------------------------HHHHHHHHLL----l


DSSP  LLHHHHHHHHHH------------------------------------------------
Query RTVDAAAQAVQR------------------------------------------------  281
ident          |
Sbjct QLTEDFYEFVKEdprivlksaverithelfkimkektahkvirelyeygvleaiipeigr  234
DSSP  EELHHHHHHHHHlhhhhhhllhhhhhhhhhhhhhlllhhhhhhhhhhllhhhhhlhhhhh


DSSP  ------------------------------------------------------------
Query ------------------------------------------------------------  281
ident
Sbjct lrevkdpldehtlktleyleqviedrakylsaellenfgkkrvlgeftdvellkwgalfh  294
DSSP  hhlllllhhhhhhhhhhhhhhhhhlhhhhllhhhhhlllllleellleehhhhhhhhhll


DSSP  ------------------------------------------------------------
Query ------------------------------------------------------------  281
ident
Sbjct digkpqtfvtfyehdkvgaqivreigerlrwgdeatefvaklvrhhlrpfflreafkkge  354
DSSP  llllllllllllhhhhhhhhhhhhhhhhllllhhhhhhhhhhhhlllhhhhhhhhhhlll


DSSP  ------------------------------------------------------------
Query ------------------------------------------------------------  281
ident
Sbjct lkrrgmanfwrecgdiaphlfllsiadamasgdeeedikalmetiaelesfnrnemkeex  414
DSSP  llhhhhhhhhhhllllhhhhhhhhhhhhhhllllhhhhhhhhhhhhhhhhhhhhhllllll


DSSP  -------------------------hhhhhhhhllll
Query -------------------------fvaridratkr  292
ident
Sbjct xxxxxxxxxxxxxxxxxxxxxxxxxxxxxxxxxxxx  450
DSSP  hhhhlllhhhhhhhhhhhhhhllhhhhhhhhhhhhl
```

No 431: Query=mol1A Sbjct=4rwoA Z-score=5.4

back to top

```
DSSP  llllllllllhhhhhhhhhhhhHHLL--LLHHHHHHHHHHHHHHHHHH--LLLLLLLLLL
Query aapysspptnlrslrdrltqvaERQG--VVFGRLQRHVAXIVVAQFAA--TLTDDTGAPL   56
ident                       |         | |   |   |  |
Sbjct -------melrhtpardldkfiEDHLlpNTCFRTQVKEAIDIVCRFLKerCFQGTADPVR   53
DSSP  -------llhhhllhhhhhhhhHHHLllLHHHHHHHHHHHHHHHHHHHHhlLLLLLLLLL


DSSP  E-EEEHHHhhHHHHLLLlllLLLLEEEEELL---------LHHHHHHHHHHHHHHLL--L
Query L-LVKGGSslELRRGIPdsrTSKDFATVARR---------DIELIHEQLADAGETGW--E  104
ident    |  |                |                             |
Sbjct VsKVVKGG-sSGKGTTLrgrSDADLVVFLTKltsfedqlrRRGEFIQEIRRQLEACQreQ  112
DSSP  EeEEEEEH-hHHHLLLLlllLLEEEEEEEELLlllhhhhhhHHHHHHHHHHHHHHHHHllL
```

```
Query [illegible]
ident  |   |               |     ||                   |
Sbjct KFKVTFEV-----qsprrenpRALSFVLSSPqlqqEVEFDVRPAFDALGQ----------  157
DSSP  LLEEELLL-----lllllllLLEEEEEEELLllllLEEEEEEEEELLLLLL----------


DSSP  hhhhllllleeeeellhhhhhhhhhhhhhlllllllllllhhhhhhhhhhhhllhhhh
Query alglvgvpaavavpcxtipwqiaqklhavtavleepkandrahdlvdlqlleglllldadl  223
ident
Sbjct ------------------------------------------------------------  157
DSSP  ------------------------------------------------------------


DSSP  hHHHHhhHHHHHHHLL--------------------------------------------
Query xPTRSacIAIFEARAQ--------------------------------------------  239
ident            |   |
Sbjct wTPGY--KPNPEIYVQlikecksrgkegefstcftelqrdflrnrptklkslirlvkhwy  215
DSSP  lLLLL--LLLHHHHHHhhhhhhhhlllllhhhhhhhhhhhhhlllhhhhhhhhhhhhhh


DSSP  --lllllllllllhHHHHHH-----------------hhllllllllllhHHHH----
Query --hpwpprvatlphwPLIYAG-----------------alegldhlelartvDAAA----  276
ident                 |
Sbjct qtckkthgnklppqyALELLTvyaweqgsrktdfstaqgfqtvlelvlkhqkLCIFweay  275
DSSP  hhhhhhhllllllhhHHHHHHhhhhhhhllllllhhhhhhhhhhhhhlhhhLEELllll


DSSP  --------------------------------------------------------hhhh
Query --------------------------------------------------------qavq  280
ident
Sbjct ydftnpvvgrcmlqqlkkprpvildpadptgnvgggdthswqrlaqearvwlgypccknl  335
DSSP  llllhhhhhhhhhllllleellllllllllllhhhhhhhhhhhhhlllhhhlll


DSSP  hhhhhhhhllll
Query rfvaridratkr  292
ident
Sbjct dgslvgawtmlq  347
DSSP  llllllllllll
```

No 432: Query=mol1A Sbjct=5f2vP Z-score=5.4

back to top

```
DSSP  llllllllllhhhhhhhhhhhhhHLLLLHHHHHHHHHHHHHHHHHHHLL--LLLL--LLLL
Query aapysspptnlrslrdrltqvaeRQGVVFGRLQRHVAXIVVAQFAATL--TDDT--GAPL   56
ident                                     |
Sbjct -----------------------DKQWERFLVPYRQAVEELKVKLKGIrtLYEYedDHSP   37
DSSP  -----------------------LLLHHHHLHHHHHHHHHHHHHHHHHHhhHHLLllLLLL


DSSP  -EEEEhhHHHHhhhlLLLLL------------------lLLLEEEEELL--LHHHHHHHH
Query -LLVKggSSLElrrgIPDSR------------------tSKDFATVARR--DIELIHEQL   95
ident    |              |                                ||    | |
Sbjct iEFVT--GRVK----PVASIlekarrksiplheietmqdIAGLRIMCQFvdDIQIVKEML   91
DSSP  lLEEE--EEEL----LHHHHhhhhhhllllhhhhhhlllLEEEEEEEELLhhHHHHHHHHH


DSSP  HHHHhhlllleEEEELLLeellllllllLLEEEEEEEEEL---lEEEEEEEEEEEEELLll
Query ADAGetgwegfTAIFTAPeeiaapgxpvKPRRFTAKLSYR---gRAFATVPIEVSSVEag  152
ident                                                  | |
Sbjct FARK-------DFTVVDQ----------RSYHLVVLYPLQtvsgEKHVLVEIQIRTLA--  132
DSSP  HLLL-------LLEELLL----------LEEEEEEEEEEEelleEEEEEEEEEEEEEHH--


DSSP  llllleeeeelhhhhllllllleeeeellhhhhhHHHHHHHHLllllllllllhhhhhhh
Query nadqfdtltsdalglvgvpaavavpcxtipwqiAQKLHAVTAvleepkandrahdlvdlq  212
ident
Sbjct ------------------------------mnfWATIEHSLN------------------  144
DSSP  ------------------------------hhhHHHHHHHHH------------------


DSSP  hhhhhllhhhhhhhhhhhhhhhhhhhlllllllLLLLLLHhhhhhhhhllllllllllh
Query lleglllldadlxptrsaciaifearaqhpwpprVATLPHWpliyagalegldhlelartv  272
ident
Sbjct -----------------------------ykysGNIPEKV------------klrlqras  163
```

```
DSSP  hhhhhhhhhhhhhhhhllll
Query daaaqavqrfvaridratkr  292
ident
Sbjct eaasrldeemseirgevqea  183
DSSP  hhhhhhhhhhhhhhhhhhll
```

No 433: Query=mol1A Sbjct=4ud4B Z-score=5.4

back to top

```
DSSP  lllllllllhhhhhhhhhhhhhhllLLHHHHHHHHHHHHHHHHHHLLLlllllLLEEEE
Query aapyssppptnlrslrdrltqvaerqgVVFGRLQRHVAXIVVAQFAATLTddtgaPLLLVK   60
ident                                           |                 |
Sbjct ---------hkeftkfcyevyneikiSDKEFKEKRAALDTLRLCLKRIS-----PDAELV   46
DSSP  ---------lhhhhhhhhhhhhhhllLHHHHHHHHHHHHHHHHHHHHHL-----LLLEEE


DSSP  HHhhhhhhhlllLLLL----lLLEEEEEL---LLHHhHHHHHHHHHHHHLllleEEEELLL
Query GGsslelrrgipDSRT----sKDFATVAR---RDIElIHEQLADAGETGwegfTAIFTAP  113
ident                      |               |  |
Sbjct AF------gsleSGLAlknsdMDLCVLMDsrvQSDT-IALQFYEELIAE----GFEGAFL   95
DSSP  EL------llhhHLLLlllllEEEEEEELlhhHHHH-HHHHHHHHHHL----LLLEEEE


DSSP  EElllllllLLEEEEEEeeelleEEEEEEEEEEELLlllllllleeeeelhhhhllllll
Query EEiaapgxpvKPRRFTAKlsyrgrAFATVPIEVSSVEagnadqfdtltsdalglvgvpaa  173
ident                         |      |
Sbjct QA-------aRIPIIKLT------ASFQCDIGFNNRL-----------------------  119
DSSP  EE-------lLEEEEEEL------LLLLEEEEEEELL-----------------------


DSSP  eeeeellhhhhhhhhhhhhhlllllllLLLLhhhhhhhhhhhllhhhhhhhhhhhhh
Query vavpcxtipwqiaqklhavtavleepkaNDRAhdlvdlqllegllldadlxptrsaciai  233
ident
Sbjct ---------------------aihntllLSSY---------------------------  130
DSSP  ---------------------hhhhhhhHHHH---------------------------


DSSP  hhhhlllllllllllllHHHHhHHHHLLLL-------------------llllllLHHH
Query fearaqhpwpprvatlphWPLIyAGALEGLD-------------------hlelarTVDA  274
ident                                  |
Sbjct ----------------tkLDAR-LKPMVLLVkhwakrkqinspyfgtlssygyvlmVLYY  173
DSSP  ----------------hhHLLL-HHHHHHHHhhhhhhlllllhhhllllhhhhhhhHHHH


DSSP  HHHH---------------------------------------------------------
Query AAQA---------------------------------------------------------  278
ident
Sbjct LIHVikppvfpnlllsplkqekivdgfdvgfddkledippsqnysslgsllhgffafyay  233
DSSP  HHHLlllllllllllllllleelleelllllhhhllllllllllhhhhhhhhhhhhh


DSSP  ------------hhHHHHHHHHLLL-----------------------------------
Query ------------vqRFVARIDRATK-----------------------------------  291
ident
Sbjct afeprekvvtfrrpDGYLTKQEKGWtryilaiedpfeishnvgrtvsssglyrirgefma  293
DSSP  lllllllllllllLLLLLHHHHLLllllllllllllllllllllllhhhhhhhhhhhh


DSSP  -----------------------l
Query -----------------------r  292
ident
Sbjct asrllnsrsypipydslfeeapip  317
DSSP  hhhhhllllllllhhhhhllllll
```

No 434: Query=mol1A Sbjct=4ep7A Z-score=5.4

back to top

```
DSSP  lllllllllhhhhhhhhhhhhhhllLLHHHHHHHHHHHHHHHHHHLLLlllllLLEEEE
Query aapyssppptnlrslrdrltqvaerqgVVFGRLQRHVAXIVVAQFAATLTddtgaPLLLVK   60
```

```
[illegible]
DSSP  ------lllhhhhhhhhhhhhhhllLHHHHHHHHHHHHHHHHHHHHLLL-----LLLEEE


DSSP  HHhhhhhhhlllLLLL----lLLEEEEElllhhHHHHHHHHHHHHLllleEEEELLLEel
Query GGsslelrrgipDSRT----sKDFATVArrdieLIHEQLADAGETGwegfTAIFTAPEei  116
ident                       |           |  |
Sbjct AF------gsleSGLAlknsdMDLCVLM-----TIALQFYEELIAE----GFEGKFLQ--   92
DSSP  EE------lhhhHLLLlllllEEEEEEL-----LLHHHHHHHHHHL----LEEEELLL--


DSSP  llllllLLEEEEEEEEEL--lEEEEEEEEEEEellllllllleeeeelhhhhlllllle
Query aapgxpvKPRRFTAKLSYR--gRAFATVPIEVSsveagnadqfdtltsdalglvgvpaav  174
ident                        |     |
Sbjct -----raRIPIIKLTSDTKngfGASFQCDIGFN---------------------------  120
DSSP  -----llLLLLEEEEEELLlllLLLLLEEEEEL---------------------------


DSSP  eeeellhhhhhhhhhhhhhhllllllllLLLLhhhhhhhhhhhhllhhhhhhhhhhhhhhh
Query avpcxtipwqiaqklhavtavleepkaNDRAhdlvdlqlleglllldadlxptrsaciaif  234
ident
Sbjct ------------------nrlaihntlLLSS-----------------------------  133
DSSP  ------------------lhhhhhhhhHHHH-----------------------------


DSSP  hhhllllllllllllLHHHhHHHH-HLLLLL-------------------lLLLLhhH
Query earaqhpwpprvatlpHWPLiYAGA-LEGLDH-------------------leLARTvdA  274
ident                                |                     |
Sbjct --------------ytKLDA-RLKPmVLLVKHwakrkqinspyfgtlssygyvLMVL-yY  177
DSSP  --------------hhHHLL-LHHHhHHHHHHhhhhlllllhhhllllhhhhhHHHH-hH


DSSP  HHH---------------------------------------------------------
Query AAQ---------------------------------------------------------  277
ident
Sbjct LIHvikppvfpnlllsplkqekivdgfdvgfddkledippsqnysslgsllhgffrfyay  237
DSSP  HHHllllllllllllllllleelleellllllhhhllllllllllhhhhhhhhhhhhh


DSSP  --------hhHHHH----hhhhHLLLL---------------------------------
Query --------avQRFV----aridRATKR---------------------------------  292
ident            |
Sbjct kfeprekvvtFRRPdgyltkqeKGWTSryilaiedpfeishnvgrtvsssglyrirgefm  297
DSSP  lllllleelLLLLllllllllLLLLLlllleelllllllllllllllhhhhhhhhhhh


DSSP  ----------------------
Query ----------------------  292
ident
Sbjct aasrllnsrsypipydslfeea  319
DSSP  hhhhhhllllllllhhhhhlll
```

No 435: Query=mol1A Sbjct=2gwsA Z-score=5.4

back to top

```
DSSP  ------------------------------------------------------------
Query ------------------------------------------------------------    0
ident
Sbjct atnhnlhiteklevlakaysvqgdkwralgyakainalksfhkpvtsyqeacsipgigkr   60
DSSP  llllhhhhhhhhhhhhhhhhhlllhhhhhhhhhhhhhhhhlllllllllhhhhhhlllllhh


DSSP  ----------------------------------------------llllllllllhhhh
Query ----------------------------------------------aapysspptnlrsl   14
ident
Sbjct maekiieilesghlrkldhisesvpvlelfsniwgagtktaqmwyqqgfrsledirsqas  120
DSSP  hhhhhhhhhhllllhhhhhllllhhhhhhhhllllllhhhhhhhhhllllllhhhhhhhll


DSSP  hhhhhhhhhhllllhHHHHHHHHHHHHHHHHHHLL-LLLLLLLLEEEEHHHHHHHHhhllll
Query rdrltqvaerqgvvfGRLQRHVAXIVVAQFAATL-TDDTGAPLLLVKGGSSLELrrgipd   73
ident                             |             |    |
Sbjct lttqqaiglkhysdfLERMPREEATEIEQTVQKAaQAFNSGLLCVACGSYRRGK------  174
DSSP  llhhhhhhhhlhhhhHLLEEHHHHHHHHHHHHHHHhHLLLLLLLEEEELHHHHLLL------
```

```
[illegible]
Query srTSKDFATVAR--------rDIELIHEQLADAGetgwegftAIFTAPE-EIAApgXPVK  124
ident   |  |                       |   |
Sbjct -aTCGDVDVLIThpdgrshrgIFSRLLDSLRQEG--------FLTDDLVsQEEN--GQQQ  223
DSSP  -lEELLEEEEEElllllllllLHHHHHHHHHHLL--------LEEEEEEeEEEE--LLEE


DSSP  LEEEEEEEEEllEEEEEEEEEEEEL-LLLLllllleeeeelhhhhlllllleeeeellhHH
Query PRRFTAKLSYrgRAFATVPIEVSSV-EAGNadqfdtltsdalglvgvpaavavpcxtiPW  183
ident        |                |
Sbjct KYLGVCRLPG--PGRRHRRLDIIVVpYSEF---------------------------aCA  254
DSSP  EEEEEELLLL--LLLLLEEEEEEEELlHHHH---------------------------hHH


DSSP  HHhhhhhhhhllllllllllllHHHHHHH----------------------hhhhhllh
Query QIaqklhavtavleepkandraHDLVDLQ----------------------lleglllld  220
ident
Sbjct LL----------------yftgSAHFNRSmralaktkgmslsehalstavvrnthgckvg  298
DSSP  HH----------------hhhlLHHHHHHhhhhhhhlleeellllleeelleellllleee


DSSP  hhhhhhHHHHhhhhhhllllLLLLLlllllhhhhhhhhhllllllllllhhhhhhhhh
Query adlxptRSACIaifearaqhpWPPRVatlphwpliyagaleglldhlelartvdaaaqavq  280
ident
Sbjct pgrvlpTPTEK-------dvfRLLGL----------------------------------  317
DSSP  llleelLLLHH-------hhhHHLLL----------------------------------


DSSP  hhhhhhhhllll
Query rfvaridratkr  292
ident
Sbjct --pyrepaerdw  327
DSSP  --llllhhhlll
```

No 436: Query=mol1A Sbjct=2gwsE Z-score=5.4

back to top

```
DSSP  ------------------------------------------------------------
Query ------------------------------------------------------------    0
ident
Sbjct tnhnlhiteklevlakaysvqgdkwralgyakainalksfhkpvtsyqeacsipgigkrm   60
DSSP  llllhhhhhhhhhhhhhhhhlllhhhhhhhhhhhhhhhhlllllllllhhhhhlllllhhh


DSSP  ---------------------------------------------lllllllllhhhhh
Query ---------------------------------------------aapysspptnlrslr   15
ident
Sbjct aekiieilesghlrkldhisesvpvlelfsniwgagtktaqmwyqqgfrsledirsqasl  120
DSSP  hhhhhhhhhhllllllllllhhhhhhhhhllllllhhhhhhhhhlllllhhhhhhhlll


DSSP  hhhhhhhhhllllhHHHHHHHHHHHHHHHHHHLL-LLLLLLLLEEEEHHHHHHHhhlllll
Query drltqvaerqgvvfGRLQRHVAXIVVAQFAATL-TDDTGAPLLLVKGGSSLELrrgipds   74
ident                                |             |    |
Sbjct ttqqaiglkhysdfLERMPREEATEIEQTVQKAaQAFNSGLLCVACGSYRRGK-------  173
DSSP  lhhhhhhhhlllhhHLLEEHHHHHHHHHHHHHHHHhHLLLLLLLEEEELHHHHLLL-------


DSSP  lLLLLEEEEEL--------lLHHHHHHHHHHHHHhhllllleeEEELLLE-ELLLllLLLLL
Query rTSKDFATVAR--------rDIELIHEQLADAGetgwegftAIFTAPE-EIAApgXPVKP  125
ident  |  |                       |   |
Sbjct aTCGDVDVLIThpdgrshrgIFSRLLDSLRQEG--------FLTDDLVsQEEN--GQQQK  223
DSSP  lEELLEEEEEElllllllllLHHHHHHHHHHLL--------LEEEEEEeEEEE--LLEEE


DSSP  EEEEEEEEEllEEEEEEEEEEEEL-LLLLllllleeeeelhhhhlllllleeeeellhHHH
Query RRFTAKLSYrgRAFATVPIEVSSV-EAGNadqfdtltsdalglvgvpaavavpcxtiPWQ  184
ident       |                |
Sbjct YLGVCRLPG--PGRRHRRLDIIVVpYSEF---------------------------aCAL  254
DSSP  EEEEELLLL--LLLLLEEEEEEEELlHHHH---------------------------hHHH


DSSP  Hhhhhhhhhllllllllllll HHHHHHH----------------------hhhhhllhh
Query IaqklhavtavleepkandraHDLVDLQ----------------------lleglllda  221
ident
```

```
[illegible]

DSSP  hhhhhHHHHHhhhhhhllllLLLLLllllhhhhhhhhhllllllllllhhhhhhhhhh
Query dlxptRSACIaifearaqhpWPPRVatlphwpliyagalegldhlelartvdaaaqavqr  281
ident
Sbjct grvlpTPTEK-------dvfRLLGL-----------------------------------  316
DSSP  lllllLLLHH-------hhhHHHLL-----------------------------------


DSSP  hhhhhhhllll
Query fvaridratkr  292
ident
Sbjct -pyrepaerdw  326
DSSP  -llllhhhlll
```

No 437: Query=mol1A Sbjct=1rztI Z-score=5.4

back to top

```
DSSP  ------------------------------------------------------------
Query ------------------------------------------------------------    0
ident
Sbjct tnhnlhiteklevlakaysvqgdkwralgyakainalksfhkpvtsyqeacsipgigkrm   60
DSSP  llllhhhhhhhhhhhhhhhhlllhhhhhhhhhhhhhhhllllllllhhhhhhllllhhh


DSSP  ---------------------------------------------lllllllllhhhhh
Query ---------------------------------------------aapysssppptnlrslr   15
ident
Sbjct aekiieilesghlrkldhisesvpvlelfsniwgagtktaqmwyqqgfrsledirsqasl  120
DSSP  hhhhhhhhlllllhhhllllllhhhhhhhhllllllhhhhhhhhhlllllhhhhhhhlll


DSSP  hhhhhhhhhllllhhHHHHHHHHHHHHHHHHHHLL-LLLLLLLLEEEEHHHHHHHhhlllll
Query drltqvaerqgvvfgRLQRHVAXIVVAQFAATL-TDDTGAPLLLVKGGSSLELrrgipds   74
ident                            |             |    |
Sbjct ttqqaiglkhysdflERMPREEATEIEQTVQKAaQAFNSGLLCVACGSYRRGK-------  173
DSSP  lhhhhhhhhlhhhhhLLEEHHHHHHHHHHHHHHHHhHLLLLLLLEEEELHHHHLLL-------


DSSP  lLLLLEEEEEL--------lLHHHHHHHHHHHHHhhlllleeeEELLL-EELLLLLlLLLL
Query rTSKDFATVAR--------rDIELIHEQLADAGetgwegftaIFTAP-EEIAAPGxPVKP  125
ident  |  |                       |   |           |         |
Sbjct aTCGDVDVLIThpdgrshrgIFSRLLDSLRQEG---------FLTDDlVSQEENG-QQQK  223
DSSP  lEELLEEEEEEllllllllHHHHHHHHHLLLL---------LEEEEeEEEEEEL-LEEE


DSSP  EEEEEEEEEllEEEEEEEEEEEEL-LLLLllllleeeeelhhhhllllllleeeeellhHHH
Query RRFTAKLSYrgRAFATVPIEVSSV-EAGNadqfdtltsdalglvgvpaavavpcxtiPWQ  184
ident       |                |
Sbjct YLGVCRLPG--PGRRHRRLDIIVVpYSEF---------------------------aCAL  254
DSSP  EEEEELLLL--LLLLLEEEEEEEELlHHHH---------------------------hHHH


DSSP  Hhhhhhhhhlllllllllll1HHHHHHH----------------------hhhhhllhh
Query IaqklhavtavleepkandraHDLVDLQ----------------------lleglllda  221
ident
Sbjct L----------------yftgSAHFNRSmralaktkgmslsehalstavvrnthgckvgp  298
DSSP  H----------------hhhlLHHHHHHhhhhhhhlleeellllleeelleellllleeel


DSSP  hhhhhHHHHHhhhhhhllllLLLLLllllhhhhhhhhhllllllllllhhhhhhhhhh
Query dlxptRSACIaifearaqhpWPPRVatlphwpliyagalegldhlelartvdaaaqavqr  281
ident
Sbjct grvlpTPTEK-------dvfRLLGL-----------------------------------  316
DSSP  lleelLLLHH-------hhhHHLLL-----------------------------------


DSSP  hhhhhhhllll
Query fvaridratkr  292
ident
Sbjct -pyrepaerdw  326
DSSP  -llllhhhlll
```

back to top

```
DSSP  ------------------------------------------------llllllLLLL--
Query ------------------------------------------------aapyssPPTN--   10
ident
Sbjct nitlkiietylgrvpsvneyhmlksqarniqkitvfnkdifvslvkknkkrffsDVNTsa   60
DSSP  lhhhhhhhhhhlllllhhhhhhhhllhhhhhhhhlllhhhhhhhhhhhhhhhllLLLLlh


DSSP  -----------------------------------------------hhhhhhhhhhhhh
Query -----------------------------------------------lrslrdrltqvae   23
ident
Sbjct seikdrilsyfskqtqtynigklftiielqsvlvttytdilgvltinvtsmeelardmln  120
DSSP  hhhhhhhhhhllhhhllllhhhhhhhhhhhhhhhhhhhhhhlhhhllllhhhhhhhhhhh


DSSP  hllllhhhhhHHHHHHHHHHHHHHLLllllllLLEEEEHHHHHHHHHLllLLLLlLLEEEE
Query rqgvvfgrlqRHVAXIVVAQFAATLtddtgaPLLLVKGGSSLELRRGipDSRTsKDFATV   83
ident                                       |   || |         |    |
Sbjct smnvavvsslVKNVNKLMEEYLRRH-----nKSCICYGSYSLYLINP--NIRY-GDIDIL  172
DSSP  hhllllllhHHHHHHHHHHHHHHHL-----lLLLEEEHHHHHHLLLL--LLLL-LLLEEE


DSSP  ELLlHHHHHHHHHHHHHHHLllleeeEELLlEELLLlllllLLEEEEEEeeelleeeEEEE
Query ARRdIELIHEQLADAGETGwegftaIFTApEEIAApgxpvKPRRFTAKlsyrgrafATVP  143
ident            ||            |
Sbjct QTN-SRTFLIDLAFLIKFI--tgnnIILS-KIPYL-----RNYMVIKD-----endNHII  218
DSSP  ELL-HHHHHHHHHHHHHHH--llllLEEE-ELLLL-----LLEEEEEL-----lllLLLE


DSSP  EEEEEL---LLLLllLLEEEEelhhhhllllleEEEELLHHHHHHHHHHHHHHLLLllll
Query IEVSSV---EAGNadQFDTLTsdalglvgvpaavAVPCXTIPWQIAQKLHAVTAVLeepk  200
ident                                              |
Sbjct DSFNIRqdtMNVV--PKIFID-------------NIYIVDPTFQLLNMIKMFSQID---r  260
DSSP  EEEELLhhhHHHL--LEEELL-------------LLEEELHHHHHHHHHHHHLLLHH---h


DSSP  LLLL----LHHHHHHHHHHHHL--LHHH--------------------------------
Query ANDR----AHDLVDLQLLEGLL--LDAD--------------------------------  222
ident    |
Sbjct LEDLskdpEKFNARMATMLEYVVryTHGIvfdgkrnnmpmkciidennrivtvttkdyfsf  320
DSSP  HLLLllllLHHHHHHHHHHHHHHhhHLLLlllllllllllllllllllllleehhhllll


DSSP  ------------------------------hhhhhhhhhhhhHHLLLL---------l
Query ------------------------------lxptrsaciaifeARAQHP---------w  242
ident
Sbjct kkclvyldenvlssdildlnadtscdfesvtnsvylihdnimytYFSNTIllsdkgkvhe  380
DSSP  leeeeellhhhhhhhhhhllllllleelhhhhlleellhhhleeeELLLLLleeelleelh


DSSP  LLLLlllllhhhhhhhhhllllllllllhhhhhhhhhhHHHHH--------hHLLL---
Query PPRVatlphwpliyagaleglsdhlelartvdaaaqavqrFVARI--------dRATK---  291
ident
Sbjct ISAR---------------------------------glCAHILlyqmltsgeYKQClsd  407
DSSP  HHHH---------------------------------hhHHHHHhhhhhllllLHHHhhh


DSSP  -------------------------------------l
Query -------------------------------------r  292
ident
Sbjct llnsmmnrdkipiyshterdkkpgrhgfiniekdiivf  445
DSSP  hhhlllllllllllllllllllllleeeellllleeel
```

No 439: Query=mol1A Sbjct=3ercD Z-score=5.4

back to top

```
DSSP  ------------------------------------------------llllllLLLL--
Query ------------------------------------------------aapyssPPTN--   10
ident
Sbjct nitlkiietylgrvpsvneyhmlksqarniqkitvfnkdifvslvkknkkrffsDVNTsa   60
DSSP  lhhhhhhhhhhlllllhhhhhhhhllhhhhhhhhlllhhhhhhhhhhhhhhhllLLLLlh
```

```
- - - -                                                   -------------------------
Query -----------------------------------------------lrslrdrltqvae   23
ident
Sbjct seikdrilsyfskqtqtynigklftiielqsvlvttytdilgvltinvtsmeelardmln  120
DSSP  hhhhhhhhhhllhhhllllhhhhhhhhhhhhhhhhhhhhhlhhhllllhhhhhhhhhhh


DSSP  hllllhhhhhHHHHHHHHHHHHHHLLllllllLLEEEEHHHHHHHHHLllLLLLlLLEEEE
Query rqgvvfgrlqRHVAXIVVAQFAATLtddtgaPLLLVKGGSSLELRRGipDSRTsKDFATV   83
ident                                       |   || |        |   |
Sbjct smnvavvsslVKNVNKLMEEYLRRH-----nKSCICYGSYSLYLINP--NIRY-GDIDIL  172
DSSP  hhllllllhHHHHHHHHHHHHHHL-----lLLLEEEHHHHHHLLLL--LLLL-LLLEEE


DSSP  ELLlHHHHHHHHHHHHHHLlllleeeEELLlEELLLlllllLLEEEEEEeeelleeeEEEE
Query ARRdIELIHEQLADAGETGwegftaIFTApEEIAApgxpvKPRRFTAKlsyrgrafATVP  143
ident            ||             |
Sbjct QTN-SRTFLIDLAFLIKFI--tgnnIILS-KIPYL-----RNYMVIKD-----endNHII  218
DSSP  ELL-HHHHHHHHHHHHHHH--llllLEEE-ELLLL-----LLEEEEEL-----lllLLLE


DSSP  EEEEEL---LLLLllLLEEEEelhhhhllllllleEEEELLHHHHHHHHHHHHHLLLllll
Query IEVSSV---EAGNadQFDTLTsdalglvgvpaavAVPCXTIPWQIAQKLHAVTAVLeepk  200
ident                                         |
Sbjct DSFNIRqdtMNVV--PKIFID-------------NIYIVDPTFQLLNMIKMFSQID---r  260
DSSP  EEEELLhhhHHHL--LEEELL-------------LLEEELHHHHHHHHHHHLLLHH---h


DSSP  LLLL----LHHHHHHHHHHHHL--LHHH--------------------------------
Query ANDR----AHDLVDLQLLEGLL--LDAD--------------------------------  222
ident    |
Sbjct LEDLskdpEKFNARMATMLEYVryTHGIvfdgkrnnmpmkciidennrivtvttkdyfsf  320
DSSP  HLLLllllLHHHHHHHHHHHHHhhHLLLlllllllllllllllllllllleehhhllll


DSSP  -------------------------------hhhhhhhhhhhhhHLLLL---------l
Query -------------------------------lxptrsaciaifeaRAQHP---------w  242
ident
Sbjct kkclvyldenvlssdildlnadtscdfesvtnsvylihdnimytyFSNTIllsdkgkvhe  380
DSSP  leeeeellhhhhhhhhhhllllllleelhhhhlleellhhhleeeeLLLLLleeelleelh


DSSP  LLLLllllhhhhhhhhlllllllllllhhhhhhhhhhHHHHH--------hHLLL---
Query PPRVatlphwpliyagalegldhlelartvdaaaqavqrFVARI--------dRATK---  291
ident
Sbjct ISAR---------------------------------glCAHILlyqmltsgeYKQClsd  407
DSSP  HHHH---------------------------------hhHHHHHhhhhhllllLHHHhhh


DSSP  -------------------------------------l
Query -------------------------------------r  292
ident
Sbjct llnsmmnrdkipiyshterdkkpgrhgfiniekdiivf  445
DSSP  hhhlllllllllllllllllllllleeeellllleeel
```

No 440: Query=mol1A Sbjct=4xq8A Z-score=5.4

back to top

```
DSSP  -----------------------llllllllllhhhhhhhhhhhhhhllllhHHHHHHHH
Query -----------------------aapysspptnlrslrdrltqvaerqgvvfGRLQRHVA   37
ident
Sbjct vpvlelfsniwgagtktaqmwyqqgfrsledirsqaslttqqaiglkhysdfLERMPREE   60
DSSP  lhhhhhhhllllllhhhhhhhhhllllhhhhhhhllllhhhhhhhhlhhhhLLLEEHHH


DSSP  HHHHHHHHHHLL-LLLLLLLLEEEEHHHHHHHHhhllllllLLLLEEEEEL--------lLH
Query XIVVAQFAATL-TDDTGAPLLLVKGGSSLELrrgipdsrTSKDFATVAR--------rDI   88
ident       |              |     |                 |  |
Sbjct ATEIEQTVQKAaQAFNSGLLCVACGSYRRGK-------aTCGDVDVLIThpdgrshrgIF  113
DSSP  HHHHHHHHHHHhHLLLLLLLEEEEELLLLLL-------lEELEEEEEEEllllllllLH


DSSP  HHHHHHHHHHHhhlllleeeEELLL-EELLLlLLLLLLEEEEEEEEEllEEEEEEEEEEE
Query ELIHEQLADAGetgwegftaIFTAP-EEIAApGXPVKPRRFTAKLSYrgRAFATVPIEVS  147
ident        |    |             |                    |
```

```
DSSP  [illegible]

DSSP  EL-LLLLlllleeeeelhhhhlllllleeeeellhHHHHhhhhhhhhllllllllllllH
Query SV-EAGNadqfdtltsdalglvgvpaavavpcxtiPWQIaqklhavtavleepkandraH  206
ident  |
Sbjct VVpYSEF---------------------------aCALL----------------yftgS  178
DSSP  ELlHHHH---------------------------hHHHH----------------hhhlL

DSSP  HHHHHH-------------------------hhhhhllhHHHHhhHHHHHhhhhhhllll
Query DLVDLQ-------------------------llegllldADLXptRSACIaifearaqhp  241
ident                                          |
Sbjct AHFNRSmralaktkgmslsehalstavvrnthgckvgpgRVLP--TPTEK-------dvf  229
DSSP  HHHHHHhhhhhhhlleeelllleeelleelllllleeellLLLL--LLLHH-------hhh

DSSP  LLLLLllllllhhhhhhhhhllllllllllhhhhhhhhhhhhhhhhhhllll
Query WPPRVatlphwpliyagalegldhlelartvdaaaqavqrfvaridratkr  292
ident
Sbjct RLLGL------------------------------------pyrepaerdw  244
DSSP  HHHLL------------------------------------llllhhhlll
```

No 441: Query=mol1A Sbjct=5cj7B Z-score=5.4

back to top

```
DSSP  ----------------------llllllllllhhhhhhhhhhhhhhllllhHHHHHHHHH
Query ----------------------aapyssspptnlrslrdrltqvaerqgvvfGRLQRHVAX   38
ident
Sbjct pvlelfsniwgagtktaqmwyqqgfrsledirsqaslttqqaiglkhysdfLERMPREEA   60
DSSP  lhhhhhhlllllhhhhhhllllllllhhhhhhlllllhhhhhhhhhlllhhHLLEEHHHH

DSSP  HHHHHHHHLL-LLLLLLLEEEEHHHHHHHHhhllllllLLLLEEEEEL--------lLHH
Query IVVAQFAATL-TDDTGAPLLLVKGGSSLELrrgipdsrTSKDFATVAR--------rDIE   89
ident     |             |    |              |  |
Sbjct TEIEQTVQKAaQAFNSGLLCVACGSYRRGK-------aTCGDVDVAIThpdgrshrgIFS  113
DSSP  HHHHHHHHHHhHHHLLLLEEEELHHHHLLL-------lEELLEEEEEElllllllllLHH

DSSP  HHHHHHHHHHhhlllleeEEELLLE-ELLLllLLLLLEEEEEEEEEllEEEEEEEEEEEE
Query LIHEQLADAGetgwegftAIFTAPE-EIAApgXPVKPRRFTAKLSYrgRAFATVPIEVSS  148
ident      |   |                                 |
Sbjct RLLDSLRQEG--------FLTDDLVsQEEN--GQQQKYLGVCRLPG--PGRRHRRLDIIV  161
DSSP  HHHHHHHHHL--------LEEEEEEeEEEE--LLEEEEEEEELLLL--LLLLLEEEEEEE

DSSP  L-LLLLlllleeeeelhhhhlllllleeeeellhHHHHhhhhhhhhllllllllllllHH
Query V-EAGNadqfdtltsdalglvgvpaavavpcxtiPWQIaqklhavtavleepkandraHD  207
ident |
Sbjct VpYSEF---------------------------aCALL----------------yftgSA  178
DSSP  LlHHHH---------------------------hHHHH----------------hhhlLH

DSSP  HHHHH-------------------------hhhhhllhHHHHhhHHHHHhhhhhhllllL
Query LVDLQ-------------------------llegllldADLXptRSACIaifearaqhpW  242
ident                                         |
Sbjct HFNRSmralaktkgmslsehalstavvrnthgckvgpgRVLP--TPTEK-------dvfR  229
DSSP  HHHHHhhhhhhhlleeelllleeeleeelllllleeeelLEEL--LLLHH-------hhhH

DSSP  LLLLllllllhhhhhhhhhllllllllllhhhhhhhhhhhhhhhhhhllll
Query PPRVatlphwpliyagalegldhlelartvdaaaqavqrfvaridratkr  292
ident
Sbjct LLGL------------------------------------pyrepaerdw  243
DSSP  HHLL------------------------------------llllhhhlll
```

No 442: Query=mol1A Sbjct=5chgB Z-score=5.4

back to top

```
DSSP  ----------------------llllllllllhhhhhhhhhhhhhhllllhHHHHHHHHH
```

```
Sbjct pvlelfsniwgagtktaqmwyqqgfrsledirsqaslttqqaiglkhysdfLERMPREEA   60
DSSP  lhhhhhhlllllllhhhhhhlllllllhhhhhhhlllllhhhhhhhhlllhhHLLEEHHHH


DSSP  HHHHHHHHHLL-LLLLLLLLEEEEHHHHHHHhhllllllLLLLEEEEEL--------lLHH
Query IVVAQFAATL-TDDTGAPLLLVKGGSSLELrrgipdsrTSKDFATVAR--------rDIE   89
ident     |             |    |              |  |
Sbjct TEIEQTVQKAaQAFNSGLLCVACGSYRRGK-------aTCGDVDVAIThpdgrshrgIFS  113
DSSP  HHHHHHHHHHhHHHLLLLEEEEEELLLLLL-------lEELLEEEEEElllllllllLHH


DSSP  HHHHHHHHHHhhllllleeEEELLLE-ELLLllLLLLLEEEEEEEEEllEEEEEEEEEEEE
Query LIHEQLADAGetgwegftAIFTAPE-EIAApgXPVKPRRFTAKLSYrgRAFATVPIEVSS  148
ident      |   |                                 |
Sbjct RLLDSLRQEG--------FLTDDLVsQEEN--GQQQKYLGVCRLPG--PGRRHRRLDIIV  161
DSSP  HHHHHHHHLL--------LEEEEEElLLEE--LLEEEEEEEEELLLL--LLLLLEEEEEEE


DSSP  L-LLLLllllleeeeelhhhhllllllleeeeellhHHHHhhhhhhhhllllllllllllHH
Query V-EAGNadqfdtltsdalglvgvpaavavpcxtiPWQIaqklhavtavleepkandraHD  207
ident |
Sbjct VpYSEF---------------------------aCALL----------------yftgSA  178
DSSP  LlHHHH---------------------------hHHHH----------------hhhlLH


DSSP  HHHHH-------------------------hhhhhllhhHHHHhhHHHHhhhhhhllllL
Query LVDLQ-------------------------llegllldaDLXPtrSACIaifearaqhpW  242
ident                                         |
Sbjct HFNRSmralaktkgmslsehalstavvrnthgckvgpgrVLPT--PTEK-------dvfR  229
DSSP  HHHHHhhhhhhhlleeellllleeeleeellllllleeeellEELL--LLHH-------hhhH


DSSP  LLLLlllllhhhhhhhhhllllllllllllhhhhhhhhhhhhhhhhhllll
Query PPRVatlphwpliyagalegldhlelartvdaaaqavqrfvaridratkr  292
ident
Sbjct LLGL------------------------------------pyrepaerdw  243
DSSP  HLLL------------------------------------llllhhhlll
```

No 443: Query=mol1A Sbjct=4xrhA Z-score=5.4

back to top

```
DSSP  ----------------------llllllllllhhhhhhhhhhhhhhllllhHHHHHHHHH
Query ----------------------aapysspptnlrslrdrltqvaerqgvvfGRLQRHVAX   38
ident
Sbjct pvlelfsniwgagtktaqmwyqqgfrsledirsqasltqqaiglkhysdfLERMPREEA   60
DSSP  lhhhhhhlllllllhhhhhhhhllllllhhhhhhhlllllhhhhhhhhlllhhHLLEEHHHH


DSSP  HHHHHHHHHLL-LLLLLLLLEEEEHHHHHHHhhllllllLLLLEEEEEL--------lLHH
Query IVVAQFAATL-TDDTGAPLLLVKGGSSLELrrgipdsrTSKDFATVAR--------rDIE   89
ident     |             |    |              |  |
Sbjct TEIEQTVQKAaQAFNSGLLCVACGSYRRGK-------aTCGDVDVLIThpdgrshrgIFS  113
DSSP  HHHHHHHHHHhHHHLLLLEEEEEELLLLLL-------lEELLEEEEEElllllllllLHH


DSSP  HHHHHHHHHHhhllllleeEEELLLE-ELLLllLLLLLEEEEEEEEEllEEEEEEEEEEEE
Query LIHEQLADAGetgwegftAIFTAPE-EIAApgXPVKPRRFTAKLSYrgRAFATVPIEVSS  148
ident      |   |                                 |
Sbjct RLLDSLRQEG--------FLTDDLVsQEEN--GQQQKYLGVCRLPG--PGRRHRRLDIIV  161
DSSP  HHHHHHHHLL--------LEEEEEEeEEEE--LLEEEEEEEEELLLL--LLLLLEEEEEEE


DSSP  L-LLLLllllleeeeelhhhhllllllleeeeellhHHHHhhhhhhhhllllllllllllHH
Query V-EAGNadqfdtltsdalglvgvpaavavpcxtiPWQIaqklhavtavleepkandraHD  207
ident |
Sbjct VpYSEF---------------------------aCALL----------------yftgSA  178
DSSP  LlHHHH---------------------------hHHHH----------------hhhlLH


DSSP  HHHHH-------------------------hhhhhllhHHHHhhHHHHHhhhhhhllllL
Query LVDLQ-------------------------llegllldADLXptRSACIaifearaqhpW  242
ident                                         |
Sbjct HFNRSmralaktkgmslsehalstavvrnthgckvgpgRVLP--TPTEK------dvfrL  230
DSSP  HHHHHhhhhhhhlleeellllleeelleellllllleeellLEEL--LLLHH------hhhhH
```

```
DSSP  LLLLlllllhhhhhhhhhllllllllllhhhhhhhhhhhhhhhhhhllll
Query PPRVatlphwpliyagalegldhlelartvdaaaqavqrfvaridratkr  292
ident
Sbjct LGLP-------------------------------------yrepaerdw  243
DSSP  HLLL-------------------------------------lllhhhlll
```

No 444: Query=mol1A Sbjct=5deeT Z-score=5.4

back to top

```
DSSP  lllllllllhhhhhhhhhhhhhLLLLHHHHHHHHHHHHHHHHHHHLLL--LLLL---LL
Query aapyssspptnlrslrdrltqvaerQGVVFGRLQRHVAXIVVAQFAATLT--DDTG---AP   55
ident                                     |                      |
Sbjct -----------------------dKQWERFLVPYRQAVEELKVKLKGIRtlYEYEddhSP   37
DSSP  -----------------------lLHHHHHHHHHHHHHHHHHHHHHHHHHHhhHHLLlllLL


DSSP  LEEEEhhHHHHhhhlLLLLL------------------lLLLEEEEELL--LHHHHHHHH
Query LLLVKggSSLElrrgIPDSR------------------tSKDFATVARR--DIELIHEQL   95
ident    |              |                                ||    | |
Sbjct IEFVT--GRVK----PVASIlekarrksiplheietmqdIAGLRIMCQFvdDIQIVKEML   91
DSSP  LLEEE--EEEL----LHHHHhhhhhhhlllhhhhhhlllLEEEEEEEELLhhHHHHHHHHH


DSSP  HHHHhhllllleEEEELL-leELLL-LLLLLLLEEEEEEEEEL---lEEEEEEEEEEEELL
Query ADAGetgwegfTAIFTA-peEIAA-PGXPVKPRRFTAKLSYR---gRAFATVPIEVSSVE  150
ident                      ||                            | |
Sbjct FARK-------DFTVVDqrdYIAEhKESGYRSYHLVLYPLQtvsgEKHVLVEIQIRTLA  144
DSSP  HHLL-------LLEEELlllLHHHhHHLLLLEEEEEEEEEEEelleEEEEEEEEEEEEHH


DSSP  llllllleeeeelhhhhllllllleeeeellhhhhhHHHHHHHHLllllllllllhhhhh
Query agnadqfdtltsdalglvgvpaavavpcxtipwqiAQKLHAVTAvleepkandrahdlvd  210
ident
Sbjct --------------------------------mnfWATIEHSLN----------------  156
DSSP  --------------------------------hhhHHHHHHHHH----------------


DSSP  hhhhhhhllhhhhhhhhhhhhhhhhhlllllllLLLLLLLHhhhhhhhhlllllllll
Query lqlleglllddadlxptrsaciaifearaqhpwppRVATLPHWpliyagalegldhlelar  270
ident
Sbjct -------------------------------ykySGNIPEKV------------klrlqr  173
DSSP  -------------------------------hhlLLLLLHHH------------hhhhhh


DSSP  lhhhhhhhhhhhhhhhhhllll
Query tvdaaaqavqrfvaridratkr  292
ident
Sbjct aseaasrldeemseirgevqea  195
DSSP  hhhhhhhhhhhhhhhhhhhhll
```

No 445: Query=mol1A Sbjct=5f2vT Z-score=5.4

back to top

```
DSSP  lllllllllhhhhhhhhhhhhHLLLLHHHHHHHHHHHHHHHHHHHLLL--LLLL---LL
Query aapyssspptnlrslrdrltqvaeRQGVVFGRLQRHVAXIVVAQFAATLT--DDTG---AP   55
ident                                     |                      |
Sbjct -----------------------DKQWERFLVPYRQAVEELKVKLKGIRtlYEYEddhSP   37
DSSP  -----------------------LLHHHHHHHHHHHHHHHHHHHHHHHHHHhhHLLLlllLL


DSSP  LEEEEhhHHHHhhhlLLLLL------------------lLLLEEEEELL--LHHHHHHHH
Query LLLVKggSSLElrrgIPDSR------------------tSKDFATVARR--DIELIHEQL   95
ident    |              |                                ||    | |
Sbjct IEFVT--GRVK----PVASIlekarrksiplheietmqdIAGLRIMCQFvdDIQIVKEML   91
DSSP  LLEEE--EEEL----LHHHHhhhhhhhlllhhhhhhlllLEEEEEEEELLhhHHHHHHHHH


DSSP  HHHHhhllllleEEEELL-leELLL-LLLLLLLEEEEEEEEEL---lEEEEEEEEEEEELL
Query ADAGetgwegfTAIFTA-peEIAA-PGXPVKPRRFTAKLSYR---gRAFATVPIEVSSVE  150
ident                      ||                            | |
```

```
DSSP  [illegible]
```

```
DSSP  llllllleeeeelhhhhlllllleeeeellhhhhhHHHHHHHHLlllllllllllhhhhh
Query agnadqfdtltsdalglvgvpaavavpcxtipwqiAQKLHAVTAvleepkandrahdlvd  210
ident
Sbjct --------------------------------mnfWATIEHSLN----------------  156
DSSP  --------------------------------hhhHHHHHHHHH----------------
```

```
DSSP  hhhhhhllhhhhhhhhhhhhhhhhhhhlllllllLLLLLLLHhhhhhhhhhlllllllll
Query lqlleglllddadlxptrsaciaifearaqhpwppRVATLPHWpliyagalegldhlelar  270
ident
Sbjct -------------------------------ykySGNIPEKV------------klrlqr  173
DSSP  -------------------------------hhlLLLLLHHH------------hhhhhh
```

```
DSSP  lhhhhhhhhhhhhhhhhhllll
Query tvdaaaqavqrfvaridratkr  292
ident
Sbjct aseaasrldeemseirgevqea  195
DSSP  hhhhhhhhhhhhhhhhhhhlll
```

No 446: Query=mol1A Sbjct=5decC Z-score=5.4

back to top

```
DSSP  llllllllllhhhhhhhhhhhhhhLLLLHHHHHHHHHHHHHHHHHHLLL--LLLL---LL
Query aapysspptnlrslrdrltqvaerQGVVFGRLQRHVAXIVVAQFAATLT--DDTG---AP   55
ident                                     |                      |
Sbjct -----------------------dDKWERFLVPYRQAVEELKVKLKGIRtlYEYEddhSP   37
DSSP  -----------------------lHHHHHHHHHHHHHHHHHHHHHHHHHhhHHLLlllLL
```

```
DSSP  LEEEEhhHHHHhhhlLLLLL------------------lLLLEEEEELL--LHHHHHHHH
Query LLLVKggSSLElrrgIPDSR------------------tSKDFATVARR--DIELIHEQL   95
ident    |              |                                ||    | |
Sbjct IEFVT--GRVK----PVASIlekarrksiplheietmqdIAGLRIMCQFvdDIQIVKEML   91
DSSP  LLEEE--EEEL----LHHHHhhhhhhlllllllhhhlllLEEEEEEELLhhHHHHHHHHH
```

```
DSSP  HHHHhhlllleEEEELLLEelllllllllLEEEEEEEEEL---lEEEEEEEEEEEELLll
Query ADAGetgwegfTAIFTAPEeiaapgxpvkPRRFTAKLSYR---gRAFATVPIEVSSVEag  152
ident                                                  | |
Sbjct FARK-------DFTVVDQR----------SYHLVVLYPLQtvsgEKHVLVEIQIRTLA--  132
DSSP  HHLL-------LLEEEEEL----------LEEEEEEEEEEelleEEEEEEEEEEEEHH--
```

```
DSSP  llllleeeeelhhhhlllllleeeeellhhhhhHHHHHHHHHLlllllllllllhhhhhhh
Query nadqfdtltsdalglvgvpaavavpcxtipwqiAQKLHAVTAvleepkandrahdlvdlq  212
ident
Sbjct ------------------------------mnfWATIEHSLN------------------  144
DSSP  ------------------------------hhhHHHHHHHHH------------------
```

```
DSSP  hhhhllhhhhhhhhhhhhhhhhhhhlllllllLLLLLLLHhhhhhhhhhllllllllllh
Query llegllldadlxptrsaciaifearaqhpwppRVATLPHWpliyagalegldhlelartv  272
ident
Sbjct -----------------------------ykySGNIPEKV------------klrlqras  163
DSSP  -----------------------------hhlLLLLLHHH------------hhhhhhhh
```

```
DSSP  hhhhhhhhhhhhhhhhllll
Query daaaqavqrfvaridratkr  292
ident
Sbjct eaasrldeemseirgevqea  183
DSSP  hhhhhhhhhhhhhhhhhhll
```

No 447: Query=mol1A Sbjct=3wfpF Z-score=5.4

back to top

```
DSSP  lllllllllhhhhhhhhhhhhhhhlllhhhhhhhhhhhHHHHHHHHL-LLLLlllLLEEE
```

```
Sbjct ----------------------------------lnfyLSYFDDVAKvLPRE---HYCFI   23
DSSP  ----------------------------------llllLLHHHHHHHhLLLL---LLEEE


DSSP  EHHHHHHHHHLLLlllllLLEEEEEELLLHHHHHHHHHHHhhlllleeeEELLLEELlll
Query KGGSSLELRRGIPdsrtsKDFATVARRDIELIHEQLADAGetgwegftaIFTAPEEIaap  119
ident  ||       | |      |       |        |
Sbjct VGGWVRDRILGEP-vgynIDVDFLTTADPVELAKNFAKRI---------GGHFFVFE---   70
DSSP  ELHHHHHHLLLLL-llleEEEEEEEELLLHHHHHHHHHHH---------LLLEEELL---


DSSP  lllLLLEEEEEEEeelleEEEEEEEEEEEL-----LLLL---------------llllEE
Query gxpVKPRRFTAKLsyrgrAFATVPIEVSSV-----EAGN---------------adqfDT  159
ident             |              |       |                      |
Sbjct ---PTIASVVLHL-----PPYRYRFDFSPLkgkdlEKALiedlkerdftanaiavnldDV  122
DSSP  ---LLLEEEEEEL-----LLLEEEEEEEELllllhHHHHhhhhlllllhhhlleeehhHH


DSSP  EEelhhhhlllllleeeeelLHHH-----------hhhhhhhHHHLlllllllLLLLHhh
Query LTsdalglvgvpaavavpcxTIPW-----------qiaqklhAVTAvleepkaNDRAHdl  208
ident |                                                      |
Sbjct LS-----------igakqtiVYDPtggikdleqgllrpvsieNLKR------dPVRVL--  163
DSSP  HL-----------lllllllEELLllhhhhhhlleellllhhHHHH------lLLHHH--


DSSP  hhhhhhhhhllhhhhhhhhhhhhhhhhhhllllllllllllllhhhHHHHHHLLLLllll
Query vdlqllegllldadlxptrsaciaifearaqhpwpprvatlphwplIYAGALEGLDhlel  268
ident
Sbjct ----------------------------------------------RGFRIAIEKN----  173
DSSP  ----------------------------------------------HHHHHHHHLL----


DSSP  lLLHHHHHHHHHH-----------------------------------------------
Query aRTVDAAAQAVQR-----------------------------------------------  281
ident           |
Sbjct lQLTEDFYEFVKEdprivlksaverithelfkimkektahkvirelyeygvleaiipeig  233
DSSP  lEELHHHHHHHHHlhhhhhhhllhhhhhhhhhhhhlllhhhhhhhhhhllhhhhhlhhhh


DSSP  ------------------------------------------------------------
Query ------------------------------------------------------------  281
ident
Sbjct rlrevkdpldehtlktleyleqviedrakylsaellenfgkkrvlgeftdvellkwgalf  293
DSSP  hllllllhhhhhhhhhhhhhhhhhlhhhhllhhhhhlllllleellleehhhhhhhhhhl


DSSP  ------------------------------------------------------------
Query ------------------------------------------------------------  281
ident
Sbjct hdigkpqtfvtfyehdkvgaqivreigerlrwgdeatefvaklvrhhlrpfflreafkkg  353
DSSP  lllllllllllllhhhhhhhhhhhhhhhllllhhhhhhhhhhhhlllhhhhhhhhhhhll


DSSP  -----------------------------------------------hhhhhhhllll
Query -----------------------------------------------fvaridratkr  292
ident
Sbjct elkrrgmanfwrecgdiaphlfllsiadamasgdeeedikalmetiaelesfnrnemk  411
DSSP  lllhhhhhhhhhhllllhhhhhhhhhhhhhhhllllhhhhhhhhhhhhhhhhhhllll
```

No 448: Query=mol1A Sbjct=4xq7A Z-score=5.3

back to top

```
DSSP  lllllllllhhhhhhhhhhhhhhhLLLLHHHHHHHHHHHHHHHHHH-LLLLllLLLLEE-
Query aapyssptnlrslrdrltqvaerQGVVFGRLQRHVAXIVVAQFAA-TLTDdtGAPLLL-   58
ident                         |           |   |  |              |
Sbjct -----qelystpasrldsfvaqwlQPHREWKEEVLDAVRTVEEFLRqEHFQ-gQDVRVLk   54
DSSP  -----llhhhllhhhhhhhhhhhlLLLHHHHHHHHHHHHHHHHHHHhLLEE-lLEELEEe


DSSP  EEHHhhhHHHHLLLlllllLLEEEEELL---------LHHHHHHHHHHHHHHLLL--LEE
Query VKGGsslELRRGIPdsrtsKDFATVARR---------DIELIHEQLADAGETGWE--GFT  107
ident |
Sbjct VVKV-gsFGNGTVLrstreVELVAFLSCfhsfqeaakHHKDVLRLIWKTMWQSQDllDLG  113
DSSP  EEEE-hhHHHLLLLlllleEEEEEEELLlllhhhhhhHHHHHHHHHHHHHHLLLHHhLLL
```

```
DSSP  EE-ELLLEELllllllllLEEEEEEEE-ellEEEEEEEEEEEELLLLllllleeeeelhh
Query AI-FTAPEEIaapgxpvkPRRFTAKLS-yrgRAFATVPIEVSSVEAGnadqfdtltsdal  165
ident                   |                || |       |
Sbjct LEdLRMEQRV--------PDALVFTIQtrgtAEPITVTIVPAYRALG-------------  152
DSSP  LEeEEEELLL--------LLEEEEEEEllllLLEEEEEEEEELLLLL-------------


DSSP  hhllllleeeeellhhhhhhhhhhhhhhhllllllllllhhhhhhhhhhhhhllhhhhh
Query glvgvpaavavpcxtipwqiaqklhavtavleepkandrahdlvdlqllegllldadlxp  225
ident
Sbjct -------------------------------------------------------pslpn  157
DSSP  -------------------------------------------------------lllll


DSSP  HHHHHHHHHHHHL----------------------------------------------l
Query TRSACIAIFEARA----------------------------------------------q  239
ident
Sbjct SQPPPEVYVSLIKacfxpsfselqrnfvkhrptklksllrlvkhwyqqyvkarspranlp  217
DSSP  LLLLHHHHHHHHHhlllhhhhhhhhhhhhlllhhhhhhhhhhhhhhhhhhlhhhllllll


DSSP  llllllllLLLLHHhhhhhhhllllllllllLHHH------------------hhhhhHH
Query hpwpprvaTLPHWPliyagalegldhlelarTVDA------------------aaqavQR  281
ident         |   |                    |
Sbjct plyalellTIYAWE--------------mgtEEDEnfmldegfttvmdllleyeviciYW  263
DSSP  lhhhhhhhHHHHHH--------------hhhLLLLlllhhhhhhhhhhhhhlhhhleeLL


DSSP  HH----------------------------------------------------------
Query FV----------------------------------------------------------  283
ident
Sbjct TKyytlhnaiiedcvrkqlkkerpiildpadptlnvaegyrwdivaqrasqclkqdccyd  323
DSSP  LLllllllhhhhhhhhhhhhllllleellllllllllllhhhhhhhhhhhhlllhhhll


DSSP  --hhhhhllll
Query --aridratkr  292
ident
Sbjct nrenpisswnv  334
DSSP  lllllllllll
```

No 449: Query=mol1A Sbjct=5decD Z-score=5.3

back to top

```
DSSP  llllllllllhhhhhhhhhhhhhHLLLLHHHHHHHHHHHHHHHHHHLLL--LLLLLL-LE
Query aapysspptnlrslrdrltqvaeRQGVVFGRLQRHVAXIVVAQFAATLT--DDTGAP-LL   57
ident                                     |
Sbjct -----------------------DDKWERFLVPYRQAVEELKVKLKGIRtlYEDDHSpIE   37
DSSP  -----------------------LHHHHHHHHHHHHHHHHHHHHHHHHHhhHLLLLLlLL


DSSP  EEEhhHHHHhhhlLLLLL------------------lLLLEEEEELL--LHHHHHHHHHH
Query LVKggSSLElrrgIPDSR------------------tSKDFATVARR--DIELIHEQLAD   97
ident  |              |                                ||     | |
Sbjct FVT--GRVK----PVASIlekarrksiplheietmqdIAGLRIMCQFvdDIQIVKEMLFA   91
DSSP  EEE--EEEL----LHHHHhhhhllllllhhhhhhlllLEEEEEEELLhhHHHHHHHHHHH


DSSP  HHhhlllleEEEELLLEELLLLLLllLLEEEEEEEEEL---lEEEEEEEEEEEELLllll
Query AGetgwegfTAIFTAPEEIAAPGXpvKPRRFTAKLSYR---gRAFATVPIEVSSVEagna  154
ident                     | |                        | |
Sbjct RK-------DFTVVDQRDYIASGY--RSYHLVVLYPLQtvsgEKHVLVEIQIRTLA----  138
DSSP  LL-------LLEEEEEEELLLLLL--LLEEEEEEEEEEelleEEEEEEEEEEEEHH----


DSSP  llleeeeelhhhhlllllleeeeellhhhhhHHHHHHHHLllllllllllhhhhhhhhhh
Query dqfdtltsdalglvgvpaavavpcxtipwqiAQKLHAVTAvleepkandrahdlvdlqll  214
ident
Sbjct ----------------------------mnfWATIEHSLN--------------------  150
DSSP  ----------------------------hhhHHHHHHHHH--------------------


DSSP  hhhllhhhhhhhhhhhhhhhhhhllLLLLlllllllhhhhhhhhlllllllllllllhhh
Query egllldadlxptrsaciaifearaqHPWPprvatlphwpliyagalegldhlelartvda  274
```

```
DSSP  ---------------hhlllllllhhHHHH------------------------hhhhhhh


DSSP  hhhhhhhhhhhhhhllll
Query aaqavqrfvaridratkr  292
ident
Sbjct asrldeemseirgevqea  189
DSSP  hhhhhhhhhhhhhhhlll
```

No 450: Query=mol1A Sbjct=4at9A Z-score=5.3

back to top

```
DSSP  --------------lllllllllhhhhhhhhhhhhhlLLLHHHHHHHHHHHHHHHHHH
Query --------------aapysspptnlrslrdrltqvaerqGVVFGRLQRHVAXIVVAQFAA   46
ident
Sbjct gshipfdfylcemafprvkpapdetsfseallkrnqdlaPNSAEQASILSLVTKINNVID   60
DSSP  lllllllhhhlhhhllllllllllhhhhhhhhhhhhhlLLHHHHHHHHHHHHHHHHHHHH


DSSP  LLL--LLLL-lLLEEEEHHhhhhhhhlllLLLL-----lLLEEEEEL--LLHHHHHHHHH
Query TLT--DDTG-aPLLLVKGGsslelrrgipDSRT-----sKDFATVAR--RDIELIHEQLA   96
ident  |     |       |                        |           |
Sbjct NLIvaPGTFevQIEEVRQV------gsykKGTMttghnvADLVVILKilPTLEAVAALGN  114
DSSP  HHHhlLLLLllLEEEEEEE------hhhhHLLLllllleEEEEEEELllLLHHHHHHHHH


DSSP  HHHHH-LLLL--eeEEELLLEellllllllllEEEEEEEeelleEEEEEEEEEEELLLLL
Query DAGET-GWEG--ftAIFTAPEeiaapgxpvkpRRFTAKLsyrgrAFATVPIEVSSVEAGN  153
ident    |                |             |           ||| |    |
Sbjct KVVESlRAQDpsevLTMLTNE-----------TGFEISS-----SDATVKILITTVPPNL  158
DSSP  HHHHHhHHHLllllLEEEELL-----------LEEEEEL-----LLLEEEEEEEEELHHHL


DSSP  llllleeeeelhhhhllllllleeeeellhhhhhhHHHHHHhllllllllLLLLhhhhhhhh
Query adqfdtltsdalglvgvpaavavpcxtipwqiaQKLHAVtavleepkaNDRAhdlvdlql  213
ident
Sbjct -------------rkldpelhldikvlqsalaaIRHARW-------feENAS--------  190
DSSP  -------------lllllllllhhhhhhhhhHHHHHH-------hhHHLL--------


DSSP  hhhhllhhhhhhhhhhhhhhhhhhllllllllllllhhHHHHHHHLLL-LLLL-----
Query leglllddadlxptrsaciaifearaqhpwpprvatlphwpLIYAGALEGL-DHLE-----  267
ident                                               |  |   |
Sbjct ----------------------------------------QSTVKVLIRLlKDLRirfpg  210
DSSP  ----------------------------------------LHHHHHHHHHhHHHHhhlhh


DSSP  ------------lllLHHH----------------------hhhhhHHHH------HHHH
Query ------------larTVDA----------------------aaqavQRFV------ARID  287
ident                 |                                        |
Sbjct fepltpwildllghyAVMNnrqplalnvyrrclqilaaglflpgsvGITDpcesgnFRVH  270
DSSP  hllllhhhhhhhhhhHHHLllllllllllhhhhhhhhllllllllLLLLllllllLLHH


DSSP  HLLLL-------------------------------------------------------
Query RATKR-------------------------------------------------------  292
ident
Sbjct TVMTLeqqdmvcytaqtlvrilshggfrkilgqegdasylaseistwdgvivtpsekaye  330
DSSP  HHLLHhhhhhhhhhhhhhhhhhlllhhhhllllllhhhhllleeelleeellllllll


DSSP  --
Query --  292
ident
Sbjct kp  332
DSSP  ll
```

No 451: Query=mol1A Sbjct=5a2zB Z-score=5.3

back to top

```
ident                                                   |       |
Sbjct ktftevqterleqadrsvlikcpsklnEKKLLqylsshgkidnyffFENRGIH--ALIEf   58
DSSP  llhhhhhhhhhhhhhleeeeellllllHHHHHhhhhhhlleeeeeeEELLLEE--EEEEe


DSSP  ------------------------------------------------------------
Query ------------------------------------------------------------   20
ident
Sbjct sekssvaslqavtgipkhvvpyksrlftftlknpgsqaaeerpvkispqshipvnelipk  118
DSSP  lllhhhhhhhhhlllllllllllleeeeeellllllllllllllllllllhhhhhhh


DSSP  --------------hhhhllLLHHHHHHHHHHHHHHHHHHLLLlllllLLEEEEHHhhhh
Query --------------vaerqgVVFGRLQRHVAXIVVAQFAATLTddtgaPLLLVKGGssle   66
ident                            |  |   |  ||         |   ||
Sbjct lchadsissqmyillneyqlTEENIKLRYLACSLVRDFARAYF-----PDSTVKPF--gs  171
DSSP  hhllllhhhhhhhhhhhllLHHHHHHHHHHHHHHHHHHHHLLL-----LLEEEEEE--hh


DSSP  hhhlllllllLLEEEEEL-----------------lLHHHHHHHHHHHHHHHLLllEEEE
Query lrrgipdsrtsKDFATVAR-----------------rDIELIHEQLADAGETGWegFTAI  109
ident             |                            |     |
Sbjct svntfgklgcdVDMFLDFHkgpfemeyqmkrlpserlATQKILSIIGDCLDNFG--PGYS  229
DSSP  hlllllllllEEEEEEELllleeeeellllllhhhHHHHHHHHHHHHHHHLL--LLEE


DSSP  -ELLLEELlllllllLLEEEEEEEEEEllee EEEEEEEEEel llllllllleeeeelhhhhl
Query -FTAPEEIaapgxpvKPRRFTAKLSYrgraFATVPIEVSsveagnadqfdtltsdalglv  168
ident                                      ||
Sbjct sVQKILNA-------RCPLVKFSHQP---tGFQCDLSVS---------------------  258
DSSP  eEEEELLL-------LLLEEEEEELL---lLEEEEEEEL---------------------


DSSP  lllllleeeeellhhhhhhHHHHHHhllllllllLLLLhhhhhhhhhhhhllhhhhhhhhh
Query gvpaavavpcxtipwqiaQKLHAVtavleepkaNDRAhdlvdlqlleglllldadlxptrs  228
ident
Sbjct --------------nsiaIRCSEL---------LYIY-----------------------  272
DSSP  --------------lhhhHHHHHH---------HHHH-----------------------


DSSP  hhhhhhhhhllllllllllllLHHHhHHHHHL-lLLLL------------------lll
Query aciaifearaqhpwpprvatlpHWPLiYAGALE-gLDHL------------------ela  269
ident                         |
Sbjct --------------------gcLDPR-VRALVFslRCWArvhgltnsvpgtwitnfsltm  311
DSSP  --------------------hhHLHH-HHHHHHHhhHHHHhhllllllllllllhhhhhh


DSSP  lLHHHHHHH---------------------------------------------------
Query rTVDAAAQA---------------------------------------------------  278
ident
Sbjct mIMFFLQKRsppiiptldqlkeladekdkhviggydcsfvsdlskikptkntetldellc  371
DSSP  hHHHHHHLLlllllllhhhhhhhllhhhlllllllllllllllllllllllllhhhhhh


DSSP  ---------------hhhhhhhhhHLLLL-------------------------------
Query ---------------vqrfvaridRATKR-------------------------------  292
ident
Sbjct dffqyfgnfdfrknslnlrkgkevNKPESsplyiwnpfeqdlnisknvnqpqlekfvama  431
DSSP  hhhhhhhlllllleeeellllleeeELLLLlllllllllllllllllllhhhhhhhhhhh


DSSP  -------------------------------
Query -------------------------------  292
ident
Sbjct resawilqkedktqqminkepwglaavlipf  462
DSSP  hhhhhhhhlllhhhhhhhllllllhhhhllll
```

No 452: Query=mol1A Sbjct=5deeV Z-score=5.3

back to top

```
DSSP  lllllllllhhhhhhhhhhhhhHLLLLHHHHHHHHHHHHHHHHHHHLLL---LLLLLL-L
Query aapyssppptnlrslrdrltqvaeRQGVVFGRLQRHVAXIVVAQFAATLT---DDTGAP-L   56
ident                                     |
Sbjct -----------------------DKQWERFLVPYRQAVEELKVKLKGIRtlyEYEDHSpI   37
```

```
DSSP  EEEEhhHHHHhhhlLLLLL------------------lLLLEEEEELL--LHHHHHHHH
Query LLVKggSSLElrrgIPDSR------------------tSKDFATVARR--DIELIHEQLA   96
ident   |              |                                ||    | |
Sbjct EFVT--GRVK----PVASIlekarrksiplheietmqdIAGLRIMCQFvdDIQIVKEMLF   91
DSSP  LEEE--EEEL----LHHHHhhhhhhllllhhhhhhlllLEEEEEEELLhhHHHHHHHHHH


DSSP  HHHhhllllеEEEELlleellllllllLLEEEEEEEEEL---lEEEEEEEEEEEEELLlll
Query DAGetgwegfTAIFTapeeiaapgxpvKPRRFTAKLSYR---gRAFATVPIEVSSVEagn  153
ident                                                 | |
Sbjct ARK-------DFTVV------------RSYHLVVLYPLQtvsgEKHVLVEIQIRTLA---  129
DSSP  HLL-------LLEEL------------LEEEEEEEEEEEelleEEEEEEEEEEEEHH---


DSSP  lllleeeeelhhhhllllllеeeeellhhhhhHHHHHHHHLllllllllllhhhhhhh
Query adqfdtltsdalglvgvpaavavpcxtipwqiAQKLHAVTAvleepkandrahdlvdlql  213
ident
Sbjct -----------------------------mnfWATIEHSLN-------------------  141
DSSP  -----------------------------hhhHHHHHHHHH-------------------


DSSP  hhhhllhhhhhhhhhhhhhhhhhhlllllllLLLLLLHhhhhhhhhllllllllllhh
Query leglllddadlxptrsaciaifearaqhpwpprVATLPHWpliyagalegldhlelartvd  273
ident
Sbjct ----------------------------ykysGNIPEKV------------klrlqrase  161
DSSP  ----------------------------hhllLLLLHHH------------hhhhhhhhh


DSSP  hhhhhhhhhhhhhhhhllll
Query aaaqavqrfvaridratkr  292
ident
Sbjct aasrldeemseirgevqea  180
DSSP  hhhhhhhhhhhhhhhhlll
```

No 453: Query=mol1A Sbjct=5f2vQ Z-score=5.3

back to top

```
DSSP  llllllllllhhhhhhhhhhhhhhLLLLHHHHHHHHHHHHHHHHHHHHLL--LLLL--LLLL
Query aapyssppptnlrslrdrltqvaerQGVVFGRLQRHVAXIVVAQFAATL--TDDT--GAPL   56
ident                                     |
Sbjct ------------------------KQWERFLVPYRQAVEELKVKLKGIrtLYEYedDHSP   36
DSSP  ------------------------LLHHHHLHHHHHHHHHHHHHHHHHHhhHHLLllLLLL


DSSP  -EEEEhhHHHHhhhlLLLLL------------------lLLLEEEEELL--LHHHHHHHH
Query -LLVKggSSLElrrgIPDSR------------------tSKDFATVARR--DIELIHEQL   95
ident    |              |                                ||    | |
Sbjct iEFVT--GRVK----PVASIlekarrksiplheietmqdIAGLRIMCQFvdDIQIVKEML   90
DSSP  lLEEE--EEEL----LLHHHhhhhhhllllllhhhlllLEEEEEEEELLhhHHHHHHHHH


DSSP  HHHHhhllllеEEEElllееllllllllLLEEEEEEEEEL---lEEEEEEEEEEEEELLll
Query ADAGetgwegfTAIFtapeeiaapgxpvKPRRFTAKLSYR---gRAFATVPIEVSSVEag  152
ident                                                  | |
Sbjct FARK-------DFTV-------------RSYHLVVLYPLQtvsgEKHVLVEIQIRTLA--  128
DSSP  HHLL-------LLLL-------------LEEEEEEEEEEElllIEEEEEEEEEEEEHH--


DSSP  llllleeeeelhhhhllllllеeeeellhhhhhHHHHHHHHLllllllllllhhhhhhh
Query nadqfdtltsdalglvgvpaavavpcxtipwqiAQKLHAVTAvleepkandrahdlvdlq  212
ident
Sbjct ------------------------------mnfWATIEHSLN------------------  140
DSSP  ------------------------------hhhHHHHHHHHH------------------


DSSP  hhhhhllhhhhhhhhhhhhhhhhhhlllllllLLLLLLHhhhhhhhhllllllllllh
Query lleglllddadlxptrsaciaifearaqhpwpprVATLPHWpliyagalegldhlelartv  272
ident
Sbjct -----------------------------ykysGNIPEKV------------klrlqras  159
DSSP  -----------------------------hhllLLLLHHH------------hhhhhhhh


DSSP  hhhhhhhhhhhhhhhhllll
```

```
Sbjct eaasrldeemseirgevqea  179
DSSP  hhhhhhhhhhhhhhhhhlll
```

No 454: Query=mol1A Sbjct=4ep7B Z-score=5.3

back to top

```
DSSP  lllllllllhhhhhhhhhhhhhhllLLHHHHHHHHHHHHHHHHHHLLLlllllLLEEEE
Query aapyssspptnlrslrdrltqvaerqgVVFGRLQRHVAXIVVAQFAATLTddtgaPLLLVK   60
ident                                     |                 |
Sbjct -------mshkeftkfcyevyneikiSDKEFKEKRAALDTLRLCLKRIS-----PDAELV   48
DSSP  -------lllhhhhhhhhhhhhhllLHHHHHHHHHHHHHHHHHHHHHHL-----LLLEEE


DSSP  HHhhhhhhhllllllllLLEEEEElllhhHHHHHHHHHHHHHLllleEEEELLLEEllll
Query GGsslelrrgipdsrtsKDFATVArrdieLIHEQLADAGETGwegfTAIFTAPEEiaapg  120
ident                   |           |  |
Sbjct AF--gslesglalknsdMDLCVLM-----TIALQFYEELIAE----GFEGKFLQR-----   92
DSSP  EE--lhhhhllllllllEEEEEEL-----LHHHHHHHHHHHL----LEEEEEELL-----


DSSP  lllllEEEEEEEEEELLEEE--EEEEEEEEellllllllleeeeelhhhhlllllleeeee
Query xpvkpRRFTAKLSYRGRAF--ATVPIEVSsveagnadqfdtltsdalglvgvpaavavpc  178
ident                          |
Sbjct -----PIIKLTSDTKNGFGasFQCDIGFN-------------------------------  116
DSSP  -----LEEEEEEELLLLLLllLLEEEEEL-------------------------------


DSSP  llhhhhhhHHHHHHhlllllllLLLLhhhhhhhhhhhllhhhhhhhhhhhhhhhhhl
Query xtipwqiaQKLHAVtavleepkaNDRAhdlvdlqlleglllldadlxptrsaciaifeara  238
ident
Sbjct ----nrlaIHNTLL---------LSSY---------------------------------  130
DSSP  ----lhhhHHHHHH---------HHHH---------------------------------


DSSP  llllllllllllLHHHhHHHHHLL-LLLL-----------------llllLHHHHHH---
Query qhpwpprvatlpHWPLiYAGALEG-LDHL-----------------elarTVDAAAQ---  277
ident
Sbjct ----------tkLDAR-LKPMVLLvKHWAkrkqinspyfgtlssygyvlmVLYYLIHvik  179
DSSP  ----------hhHLLL-HHHHHHHhHHHHhhllllllhhhllllhhhhhhhHHHHHHHlll


DSSP  ------------------------------------------------------------
Query ------------------------------------------------------------  277
ident
Sbjct ppvfpnlllsplkqekivdgfdvgfddkledippsqnysslgsllhgffrfyaykfepre  239
DSSP  llllllhhhllllllleelleellllllhhhllllllllllhhhhhhhhhhhhhhllllll


DSSP  --hhHHHH---hhhhHLLLL----------------------------------------
Query --avQRFV---aridRATKR----------------------------------------  292
ident      |
Sbjct kvvtFRRPdgyltkqEKGWTsryilaiedpfeishnvgrtvsssglyrirgefmaasrll  299
DSSP  leelLLLLlllllhhHHLLLllllleellllllllllllllhhhhhhhhhhhhhhhhhh


DSSP  ----------------
Query ----------------  292
ident
Sbjct nsrsypipydslfeea  315
DSSP  llllllllhhhhllll
```

No 455: Query=mol1A Sbjct=4z6cA Z-score=5.3

back to top

```
DSSP  ------------------------------------------------------------
Query ------------------------------------------------------------    0
ident
Sbjct tlnggitdmltelanfeknvsqaihkynayrkaasviakyphkiksgaeakklpgvgtki   60
DSSP  lllhhhhhhhhhhhhhhhhhllllhhhhhhhhhhhhhhhllllllllhhhhhlllllllhhh
```

```
Query ------------------------------------------------aapysspptnlr   12
ident
Sbjct aekideflatgklrklekirqddtsssinfltrvsgigpsaarkfvdegiktledlrkne  120
DSSP  hhhhhhhhhhlllhhhhhhhllhhhhhhhhhlllllllhhhhhhhhhlllllhhhhhllh


DSSP  hhhhhhhhhhhhllllhhhhhHHHHHHHHHHHHHH-LLLLLLLLLLEEEEHHHHHHHhhhll
Query slrdrltqvaerqgvvfgrlqRHVAXIVVAQFAA-TLTDDTGAPLLLVKGGSSLElrrgi   71
ident                                                | |
Sbjct dklnhhqriglkyfgdfekriPREEMLQMQDIVLnEVKKVDSEYIATVCGSFRRG-----  175
DSSP  hhllhhhhhhhhlhhhhllleEHHHHHHHHHHHHhHHHHHLLLLEEEEELLLLL-----


DSSP  llLLLLLLEEEEEL------------lLHHHHHHHHHHHHhhllllleeEEELLLEEllll
Query pdSRTSKDFATVAR------------rDIELIHEQLADAGetgwegftAIFTAPEEiaap  119
ident      | |                         |||             |
Sbjct --AESSGDMDVLLThpsftsestkqpkLLHQVVEQLQKVH--------FITDTLSK----  221
DSSP  --LLEELLEEEEEElllleelleellllHHHHHHHHHHHHLL--------LEEEEEEE----


DSSP  lllLLLEEEEEEEEEL-LEEEEEEEEEEEL-LLLLllllleeeeelhhhhlllllleeee
Query gxpVKPRRFTAKLSYR-GRAFATVPIEVSSV-EAGNadqfdtltsdalglvgvpaavavp  177
ident             |            |
Sbjct --gETKFMGVCQLPSKnEKEYPHRRIDIRLIpKDQY------------------------  255
DSSP  --lLLEEEEEELLLLLlLLLLLLEEEEEEELlHHHH------------------------


DSSP  ellhHHHH---hhHHHHH-HLLLllllllllllhhhhhhhhhhhllhhHHHHhhHHHHhh
Query cxtiPWQI---aqKLHAV-TAVLeepkandrahdlvdlqlleglllldaDLXPtrSACIai  233
ident                 |                                |
Sbjct ---yCGVLyftgsDIFAKnMRAH-alekgftineytirplgvtgvagePLPV--DSEK--  307
DSSP  ---hHHHHhhhllHHHHHhHHHH-hhllleeellllleeellllllllLLLL--LLHH--


DSSP  hhhhlllLLLLLllllllhhhhhhhhhllllllllllhhhhhhhhhhhhhhhhhllll
Query fearaqhPWPPRvatlphwpliyagalegldhlelartvdaaaqavqrfvaridratkr  292
ident
Sbjct ---difdYIQWK--------------------------------------yrepkdrse  325
DSSP  ---hhhhHLLLL--------------------------------------lllhhhlll
```

No 456: Query=mol1A Sbjct=2ga9D Z-score=5.3

back to top

```
DSSP  ------------------------------------------------llllllLLLL--
Query ------------------------------------------------aapyssPPTN--   10
ident
Sbjct nitlkiietylgrvpsvneyhmlksqarniqkitvfnkdifvslvkknkkrffsDVNTsa   60
DSSP  lhhhhhhhhhhlllllhhhhhhhhhhhhhhhhhhhhllhhhhhhhhhhhhhhhllLLLLlh


DSSP  -----------------------------------------------hhhhhhhhhhhhh
Query -----------------------------------------------lrslrdrltqvae   23
ident
Sbjct seikdrilsyfskqtqtynigklftiielqsvlvttytdilgvltinvtsmeelardmln  120
DSSP  hhhhhhhhhhllhhhhlllhhhhhhhhhhhhhhhhhhhhhhllhhhllhhhlhhhhhhhhh


DSSP  hllllhhhhhHHHHHHHHHHHHHHHLLllllllLLEEEEHHHHHHHHHHLllLLLLlLLEEEE
Query rqgvvfgrlqRHVAXIVVAQFAATLtddtgaPLLLVKGGSSLELRRGipDSRTsKDFATV   83
ident                                      |  || |       |   |
Sbjct smnvavvsslVKNVNKLMEEYLRRH-----nKSCICYGSYSLYLINP--NIRY-GDIDIL  172
DSSP  hhlllllllhHHHHHHHHHHHHHHHL-----lLLEEEEELHHHHHLLLL--LLLL-LLLEEE


DSSP  ELLlHHHHHHHHHHHHHHHLlllleeeEELLlEELLLlllllLLEEEEEEeeelleeeEEEE
Query ARRdIELIHEQLADAGETGwegftaIFTApEEIAApgxpvKPRRFTAKlsyrgrafATVP  143
ident            ||            |
Sbjct QTN-SRTFLIDLAFLIKFI--tgnnIILS-KIPYL-----RNYMVIKD-----endNHII  218
DSSP  ELL-HHHHHHHHHHHHHHH--hlllLEEE-ELLLL-----LLEEEEEL-----lllLEEE


DSSP  EEEEE---LLLLLllLLEEEEelhhhhlllllleEEEELLHHHHHHHHHHHHHLLLllll
Query IEVSS---VEAGNadQFDTLTsdalglvgvpaavAVPCXTIPWQIAQKLHAVTAVLeepk  200
ident                                            |
```

```
DSSP  LLLL----LHHHHHHHHHHHHL--LHHH--------------------------------
Query ANDR----AHDLVDLQLLEGLL--LDAD--------------------------------  222
ident   |
Sbjct LEDLskdpEKFNARMATMLEYVryTHGIvfdgkrnnmpmkciidennrivtvttkdyfsf  320
DSSP  HHHHhhlhHHHHHHHHHHHHHHhhHHLLlllllllllllleeeellllleeeeellllllll


DSSP  -------------------------------hhhhhhhhhhhhhHLLLL---------l
Query -------------------------------lxptrsaciaifeaRAQHP---------w  242
ident
Sbjct kkclvyldenvlssdildlnadtscdfesvtnsvylihdnimytyFSNTIllsdkgkvhe  380
DSSP  leeeeellhhhhhhhhhhlllllleelhhhllleeeeelleeeeeLLLLLeeeelleelh


DSSP  LLLLllllhhhhhhhhhllllllllllhhhhhhhhhhHHHHH--------hHLLL---
Query PPRVatlphwpliyagalegldhlelartvdaaaqavqrFVARI--------dRATK---  291
ident
Sbjct ISAR---------------------------------glCAHILlyqmltsgeYKQClsd  407
DSSP  HHHH---------------------------------hhHHHHHhhhhhllllLHHHhhh


DSSP  -------------------------------------l
Query -------------------------------------r  292
ident
Sbjct llnsmmnrdkipiyshterdkkpgrhgfiniekdiivf  445
DSSP  hhhlllllllllleelllllllllleeeellllleeel
```

No 457: Query=mol1A Sbjct=5deeR Z-score=5.3

back to top

```
DSSP  lllllllllhhhhhhhhhhhhhHLLLLHHHHHHHHHHHHHHHHHHHHLLL--LLLL---LL
Query aapysspptnlrslrdrltqvaeRQGVVFGRLQRHVAXIVVAQFAATLT--DDTG---AP   55
ident                                     |                      |
Sbjct -----------------------DKQWERFLVPYRQAVEELKVKLKGIRtlYEYEddhSP   37
DSSP  -----------------------LHHHHHHHHHHHHHHHHHHHHHHHHHHHhhLLLLlllLL


DSSP  LEEEEhhHHHHhhhlLLLLL------------------lLLLEEEEELL--LHHHHHHHH
Query LLLVKggSSLElrrgIPDSR------------------tSKDFATVARR--DIELIHEQL   95
ident    |              |                                ||    | |
Sbjct IEFVT--GRVK----PVASIlekarrksiplheietmqdIAGLRIMCQFvdDIQIVKEML   91
DSSP  LLEEE--EEEL----LHHHHhhhhhhlllhhhhhhlllLEEEEEEEELLhhHHHHHHHHH


DSSP  HHHhhhlllleEEEELLleellllllLLLLEEEEEEEEEL---lEEEEEEEEEEEEELLll
Query ADAgetgwegfTAIFTApeeiaapgxPVKPRRFTAKLSYR---gRAFATVPIEVSSVEag  152
ident                                                  | |
Sbjct FAR-------kDFTVVD---------GYRSYHLVVLYPLQtvsgEKHVLVEIQIRTLA--  133
DSSP  HLL-------lLLEEEL---------LLLEEEEEEEEEEEelleEEEEEEEEEEEEEHH--


DSSP  lllleeeeelhhhhllllleeeeellhhhhhHHHHHHHHLllllllllllhhhhhhh
Query nadqfdtltsdalglvgvpaavavpcxtipwqiAQKLHAVTAvleepkandrahdlvdlq  212
ident
Sbjct ------------------------------mnfWATIEHSLN------------------  145
DSSP  ------------------------------hhhHHHHHHHLHH------------------


DSSP  hhhhhllhhhhhhhhhhhhhhhhhhlllllllLLLLLLLHhhhhhhhhlllllllllllh
Query llegllldadlxptrsaciaifearaqhpwppRVATLPHWpliyagalegldhlelartv  272
ident
Sbjct ------------------------------ykySGNIPEKV------------klrlqras  164
DSSP  ------------------------------hhlLLLLLHHH------------hhhhhhhh


DSSP  hhhhhhhhhhhhhhhhllll
Query daaaqavqrfvaridratkr  292
ident
Sbjct eaasrldeemseirgevqea  184
DSSP  hhhhhhhhhhhhhhhhhlll
```

back to top

```
DSSP  llllllllllhhhhhhhhhhhhhHLLLLHHHHHHHHHHHHHHHHHHLLL--LLLL---LL
Query aapysspptnlrslrdrltqvaeRQGVVFGRLQRHVAXIVVAQFAATLT--DDTG---AP   55
ident                                     |                      |
Sbjct -----------------------DKQWERFLVPYRQAVEELKVKLKGIRtlYEYEddhSP   37
DSSP  -----------------------LHHHHHHHHHHHHHHHHHHHHHHHHHhhLLLLlllLL


DSSP  LEEEEhhHHHHhhhlLLLLL------------------lLLLEEEEELL--LHHHHHHHH
Query LLLVKggSSLElrrgIPDSR------------------tSKDFATVARR--DIELIHEQL   95
ident    |              |                                ||    | |
Sbjct IEFVT--GRVK----PVASIlekarrksiplheietmqdIAGLRIMCQFvdDIQIVKEML   91
DSSP  LLEEE--EEEL----LHHHHhhhhhhhlllhhhhhhlllLEEEEEEEELLhhHHHHHHHH


DSSP  HHHhhhlllleEEEELLleellllllLLLLEEEEEEEEEL---lEEEEEEEEEEEELLll
Query ADAgetgwegfTAIFTApeeiaapgxPVKPRRFTAKLSYR---gRAFATVPIEVSSVEag  152
ident                                                  | |
Sbjct FAR-------kDFTVVD---------GYRSYHLVVLYPLQtvsgEKHVLVEIQIRTLA--  133
DSSP  HLL-------lLLEEEL---------LLLLLEEEEEEEEEelleEEEEEEEEEEEEHH--


DSSP  llllleeeeelhhhhlllllleeeeellhhhhhHHHHHHHHLllllllllllhhhhhhh
Query nadqfdtltsdalglvgvpaavavpcxtipwqiAQKLHAVTAvleepkandrahdlvdlq  212
ident
Sbjct ------------------------------mnfWATIEHSLN------------------  145
DSSP  ------------------------------hhhHHHHHHHHH------------------


DSSP  hhhhhllhhhhhhhhhhhhhhhhhhlllllllLLLLLLLHhhhhhhhhllllllllllllh
Query llegllldadlxptrsaciaifearaqhpwppRVATLPHWpliyagalegldhlelartv  272
ident
Sbjct -----------------------------ykySGNIPEKV------------klrlqras  164
DSSP  -----------------------------lllLLLLLHHH------------hhhhhhhh


DSSP  hhhhhhhhhhhhhhhhllll
Query daaaqavqrfvaridratkr  292
ident
Sbjct eaasrldeemseirgevqea  184
DSSP  hhhhhhhhhhhhhhhhhlll
```

No 459: Query=mol1A Sbjct=5f2vY Z-score=5.3

back to top

```
DSSP  llllllllllhhhhhhhhhhhhhHLLLLHHHHHHHHHHHHHHHHHHLLL--LLLL---LL
Query aapysspptnlrslrdrltqvaeRQGVVFGRLQRHVAXIVVAQFAATLT--DDTG---AP   55
ident                                     |                      |
Sbjct -----------------------DKQWERFLVPYRQAVEELKVKLKGIRtlYEYEddhSP   37
DSSP  -----------------------LHHHHHHHHHHHHHHHHHHHHHHHHHhhHLLLlllLL


DSSP  LEEEEhhHHHHhhhlLLLLL------------------lLLLEEEEELL--LHHHHHHHH
Query LLLVKggSSLElrrgIPDSR------------------tSKDFATVARR--DIELIHEQL   95
ident    |              |                                ||    | |
Sbjct IEFVT--GRVK----PVASIlekarrksiplheietmqdIAGLRIMCQFvdDIQIVKEML   91
DSSP  EEEEE--EEEL----LHHHHhhhhhhllllhhhhhhlllLEEEEEEEELLhhHHHHHHHH


DSSP  HHHHhhlllleEEEELLLeelllllllllLEEEEEEEEEL---lEEEEEEEEEEEELLll
Query ADAGetgwegfTAIFTAPeeiaapgxpvkPRRFTAKLSYR---gRAFATVPIEVSSVEag  152
ident                                                  | |
Sbjct FARK-------DFTVVDQ-----------SYHLVVLYPLQtvsgEKHVLVEIQIRTLA--  131
DSSP  HHLL-------LLEELLL-----------LEEEEEEEEEEelleEEEEEEEEEEEEHH--


DSSP  llllleeeeelhhhhlllllleeeeellhhhhhHHHHHHHHLLLlllllllllhhhhhhh
Query nadqfdtltsdalglvgvpaavavpcxtipwqiAQKLHAVTAVLeepkandrahdlvdlq  212
ident
Sbjct ------------------------------mnfWATIEHSLNYK----------------  145
DSSP  ------------------------------hhhHHHHHHHHHHH----------------
```

```
Query lleglllddadlxptrsaciaifearaqhpwpprVATLPHWpliyagalegldhlelartv  272
ident
Sbjct -------------------------------ysGNIPEKV------------klrlqras  162
DSSP  -------------------------------llLLLLHHH------------hhhhhhhh


DSSP  hhhhhhhhhhhhhhhllll
Query daaaqavqrfvaridratkr  292
ident
Sbjct eaasrldeemseirgevqea  182
DSSP  hhhhhhhhhhhhhhhhhlll
```

No 460: Query=mol1A Sbjct=5deeP Z-score=5.3

back to top

```
DSSP  llllllllllhhhhhhhhhhhhhHLLLLHHHHHHHHHHHHHHHHHHHLL--LLLL--LLLL
Query aapyssspptnlrslrdrltqvaeRQGVVFGRLQRHVAXIVVAQFAATL--TDDT--GAPL   56
ident                                     |
Sbjct -----------------------DKQWERFLVPYRQAVEELKVKLKGIrtLYEYedDHSP   37
DSSP  -----------------------LLLHHHHLHHHHHHHHHHHHHHHHHHhhHHLLllLLLL


DSSP  -EEEEhhHHHHhhhlLLLLL------------------lLLLEEEEELL--LHHHHHHHH
Query -LLVKggSSLElrrgIPDSR------------------tSKDFATVARR--DIELIHEQL   95
ident    |              |                                ||    | |
Sbjct iEFVT--GRVK----PVASIlekarrksiplheietmqdIAGLRIMCQFvdDIQIVKEML   91
DSSP  lLEEE--EEEL----LHHHHhhhhhhllllhhhhhhlllLEEEEEEEELLhhHHHHHHHHH


DSSP  HHHHhhllllеEEEELLLeellllllllLLEEEEEEEEEL---lEEEEEEEEEEEELLll
Query ADAGetgwegfTAIFTAPeeiaapgxpvKPRRFTAKLSYR---gRAFATVPIEVSSVEag  152
ident                                                  | |
Sbjct FARK-------DFTVVDQ----------RSYHLVVLYPLQtvsgEKHVLVEIQIRTLA--  132
DSSP  HLLL-------LLEELLL----------LEEEEEEEEEEEelleEEEEEEEEEEEEHH--


DSSP  llllleeeeelhhhhllllllleeeeellhhhhhHHHHHHHHLLLlllllllllhhhhhhh
Query nadqfdtltsdalglvgvpaavavpcxtipwqiAQKLHAVTAVLeepkandrahdlvdlq  212
ident
Sbjct ------------------------------mnfWATIEHSLNYK----------------  146
DSSP  ------------------------------hhhHHHHHHHHHHH----------------


DSSP  hhhhhllhhhhhhhhhhhhhhhhhhllllllllLLLLLLHhhhhhhhhllllllllllh
Query lleglllddadlxptrsaciaifearaqhpwpprVATLPHWpliyagalegldhlelartv  272
ident
Sbjct -------------------------------ysGNIPEKV------------klrlqras  163
DSSP  -------------------------------llLLLLHHH------------hhhhhhhh


DSSP  hhhhhhhhhhhhhhhllll
Query daaaqavqrfvaridratkr  292
ident
Sbjct eaasrldeemseirgevqea  183
DSSP  hhhhhhhhhhhhhhhhhhll
```

No 461: Query=mol1A Sbjct=5f2vV Z-score=5.3

back to top

```
DSSP  llllllllllhhhhhhhhhhhhhHLLLLHHHHHHHHHHHHHHHHHHHLL--LLLL-LLLL-
Query aapyssspptnlrslrdrltqvaeRQGVVFGRLQRHVAXIVVAQFAATL--TDDT-GAPL-   56
ident                                     |
Sbjct -----------------------DKQWERFLVPYRQAVEELKVKLKGIrtLYEYeDHSPi   37
DSSP  -----------------------LHHHHHHHHHHHHHHHHHHHHHHHHHHhhHHLLlLLLLl


DSSP  EEEEhhHHHHhhhlLLLLL------------------lLLLEEEEELL--LHHHHHHHH
Query LLVKggSSLElrrgIPDSR------------------tSKDFATVARR--DIELIHEQLA   96
ident   |              |                                ||    | |
Sbjct EFVT--GRVK----PVASIlekarrksiplheietmqdIAGLRIMCQFvdDIQIVKEMLF   91
```

```
DSSP  HHHhhlllleEEEELlleellllllllLLEEEEEEEEEL---lEEEEEEEEEEEELLlll
Query DAGetgwegfTAIFTapeeiaapgxpvKPRRFTAKLSYR---gRAFATVPIEVSSVEagn  153
ident                                                 | |
Sbjct ARK-------DFTVV------------RSYHLVVLYPLQtvsgEKHVLVEIQIRTLA---  129
DSSP  HLL-------LLEEL------------LEEEEEEEEEEelleEEEEEEEEEEEEEHH---


DSSP  lllleeeeelhhhhllllllleeeeellhhhhhHHHHHHHHLllllllllllhhhhhhhh
Query adqfdtltsdalglvgvpaavavpcxtipwqiAQKLHAVTAvleepkandrahdlvdlql  213
ident
Sbjct -----------------------------mnfWATIEHSLN-------------------  141
DSSP  -----------------------------hhhHHHHHHHHH-------------------


DSSP  hhhhllhhhhhhhhhhhhhhhhhhlllllllLLLLLLLHhhhhhhhhllllllllllhh
Query legllldadlxptrsaciaifearaqhpwppRVATLPHWpliyagalegldhlelartvd  273
ident
Sbjct ----------------------------ykySGNIPEKV------------klrlqrase  161
DSSP  ----------------------------hhlLLLLLHHH------------hhhhhhhhh


DSSP  hhhhhhhhhhhhhhhllll
Query aaaqavqrfvaridratkr  292
ident
Sbjct aasrldeemseirgevqea  180
DSSP  hhhhhhhhhhhhhhhhlll
```

No 462: Query=mol1A Sbjct=4yd1A Z-score=5.3

back to top

```
DSSP  ------------------------------------------------------------
Query ------------------------------------------------------------    0
ident
Sbjct mpayacqrptplthhntglsealeilaeaagfegsegrlltfcraasvlkalpspvttls   60
DSSP  llllhhhlllllllllhhhhhhhhhhhhhhhhlllhhhhhhhhhhhhhhhhllllllllhh


DSSP  ------------------------------------------------------------
Query ------------------------------------------------------------    0
ident
Sbjct qlqglphfgehssrvvqellehgvceevervrrseryqtmklftqifgvgvktadrwyre  120
DSSP  hhlllllllhhhhhhhhhhhhhlllhhhhhhhhlhhhhhhhhhhllllllhhhhhhhhhl


DSSP  llllllllllhhhhhhhhhhhhhhllllhhhhhHHHHHHHHHHHHHHL-LLLLlLLLLEEE
Query aapysspptnlrslrdrltqvaerqgvvfgrlqRHVAXIVVAQFAAT-LTDDtGAPLLLV   59
ident                                           |
Sbjct glrtlddlreqpqkltqqqkaglqhhqdlstpvLRSDVDALQQVVEEaVGQAlPGATVTL  180
DSSP  llllhhhhhllhhhllhhhhhhhhlhhhhllleEHHHHHHHHHHHHHHhHHHHlLLLEEEE


DSSP  EHHHHHHHhhllllllLLLLEEEEEL--------lLHHHHHHHHHHHHHhhlllleeeEEL
Query KGGSSLELrrgipdsrTSKDFATVAR--------rDIELIHEQLADAGetgwegftaIFT  111
ident  ||                |                       | | |
Sbjct TGGFRRGK-------lQGHDVDFLIThpkegqeagLLPRVMCRLQDQG---------LIL  224
DSSP  LHHHHLLL-------lEELEEEEEEEllllllllLHHHHHHHHHLLL---------LEE


DSSP  LLEELL-llllLLLLEEEEEEEEEELLEeEEEEEEEEEEL-LLLLllllleeeeelhhhhll
Query APEEIA-apgxPVKPRRFTAKLSYRGRaFATVPIEVSSV-EAGNadqfdtltsdalglvg  169
ident                      |   |     |
Sbjct YHQHQHschmdAFERSFCIFRLPQPGS-WKAVRVDLVVApVSQF----------------  267
DSSP  EEEEELllllLLEEEEEEEEEEEEELLE-EEEEEEEEEELlHHHH----------------


DSSP  llllеeeeellhHHHHhhhhhhhhllllllllllllHHHHHHH--------------hhh
Query vpaavavpcxtiPWQIaqklhavtavleepkandraHDLVDLQ--------------lle  215
ident                                       |
Sbjct -----------pFALL----------------gwtgSKLFQRElrrfsrkekglwlnshg  300
DSSP  -----------hHHHH----------------hhhlLHHHHHHhhhhhhhhhlleeelle


DSSP  hhllhhhhhhhHHHHHhhhhhhllllLLLLLlllllhhhhhhhhhllllllllllhhhh
```

```
Sbjct lfdpeqktffqAASEE-------difRHLGL-----------------------------  324
DSSP  eeelllleellLLLHH-------hhhHHLLL-----------------------------


DSSP  hhhhhhhhhhhhhllll
Query aqavqrfvaridratkr  292
ident
Sbjct --------ylppeqrna  333
DSSP  --------lllhhhlll
```

No 463: Query=mol1A Sbjct=5cj7A Z-score=5.3

back to top

```
DSSP  -------------------------llllllllllhhhhhhhhhhhhhhllllhHHHHHH
Query -------------------------aapysspptnlrslrdrltqvaerqgvvfGRLQRH   35
ident
Sbjct esvpvlelfsniwgagtktaqmwyqqgfrsledirsqasltttqqaiglkhysdfLERMPR   60
DSSP  lhhhhhhhhhllllllhhhhhhhhhlllllhhhhhhhllllhhhhhhhhlhhhhHLLEEH


DSSP  HHHHHHHHHHHLL-LLLLLLLLEEEEHHHHHHHhhllllllLLLLEEEEEL--------l
Query VAXIVVAQFAATL-TDDTGAPLLLVKGGSSLELrrgipdsrTSKDFATVAR--------r   86
ident        |             |    |              |  |
Sbjct EEATEIEQTVQKAaQAFNSGLLCVACGSYRRGK-------aTCGDVDVAIThpdgrshrg  113
DSSP  HHHHHHHHHHHHHhHHHLLLLEEEEEELLLLLL-------lEELEEEEEEElllllllll


DSSP  LHHHHHHHHHHHHhhlllleeEEELLLEELLLllLLLLLEEEEEEEEEllEEEEEEEEEE
Query DIELIHEQLADAGetgwegftAIFTAPEEIAApgXPVKPRRFTAKLSYrgRAFATVPIEV  146
ident         |   |                                |
Sbjct IFSRLLDSLRQEG--------FLTDDLVSQEE-nGQQQKYLGVCRLPG--PGRRHRRLDI  162
DSSP  LHHHHHHHHHHLL--------LEEEEEEEEEE-eLLEEEEEEEELLLL--LLLLLEEEEE


DSSP  EEL-LLLLlllleeeeelhhhhlllllleeeeellhHHHHhhhhhhhhllllllllllll
Query SSV-EAGNadqfdtltsdalglvgvpaavavpcxtiPWQIaqklhavtavleepkandra  205
ident   |
Sbjct IVVpYSEF---------------------------aCALL----------------yftg  179
DSSP  EELlLLLH---------------------------hHHHH----------------hhhl


DSSP  HHHHHHH-------------------------hhhhhllhHHHHhhHHHHHhhhhhhlll
Query HDLVDLQ-------------------------llegllldADLXptRSACIaifearaqh  240
ident                                           |
Sbjct SAHFNRSmralaktkgmslsehalstavvrnthgckvgpgRVLP--TPTEK-------dv  230
DSSP  LHHHHHHhhhhhhhllleelllleellleellllleeellLEEL--LLLHH-------hh


DSSP  lLLLLLLllllhhhhhhhhhlllllllllllhhhhhhhhhhhhhhhhhllll
Query pWPPRVAtlphwpliyagalegldhlelartvdaaaqavqrfvaridratkr  292
ident
Sbjct fRLLGLP------------------------------------yrepaerdw  246
DSSP  hHHLLLL------------------------------------lllhhhhll
```

No 464: Query=mol1A Sbjct=5cp2A Z-score=5.3

back to top

```
DSSP  -------------------llllllllllhhhhhhhhhhhhhhllllhHHHHHHHHHHHH
Query -------------------aapysspptnlrslrdrltqvaerqgvvfGRLQRHVAXIVV   41
ident
Sbjct elfsniwgagtktaqmwyqqgfrsledirsqasltttqqaiglkhysdfLERMPREEATEI   60
DSSP  lllllllllhhhhlllllllllllhhhhhhhllllhhhhhhhhlllhhHLLEEHHHHHHH


DSSP  HHHHHLL-LLLLLLLLEEEEHHHHHHHhhllllllLLLLEEEEEL--------lLHHHHH
Query AQFAATL-TDDTGAPLLLVKGGSSLELrrgipdsrTSKDFATVAR--------rDIELIH   92
ident  |             |    |              |  |
Sbjct EQTVQKAaQAFNSGLLCVACGSYRRGK-------aTCGDVDVAIThpdgrshrgIFSRLL  113
DSSP  HHHHHHHhHLLLLLLEEEELHHHHLLL-------lEELLEEEEEElllllllllLHHHHH
```

```
Query EQLADAGetgwegftAIFTAPE-EIAApgXPVKPRRFTAKLSYrgRAFATVPIEVSSV-E  150
ident   |   |                                 |                |
Sbjct DSLRQEG--------FLTDDLVsQEEN--GQQQKYLGVCRLPG--PGRRHRRLDIIVVpY  161
DSSP  HHHHHLL--------LEEEEEEeEEEE--LLEEEEEEEELLLL--LLLLLEEEEEEELlH
```

```
DSSP  LLLllllleeeeelhhhhllllllleeeeellhHHHHhhhhhhhhlllllllllllHHHHH
Query AGNadqfdtltsdalglvgvpaavavpcxtiPWQIaqklhavtavleepkandraHDLVD  210
ident
Sbjct SEF---------------------------aCALL----------------yftgSAHFN  178
DSSP  HHH---------------------------hHHHH----------------hhhlLHHHH
```

```
DSSP  HH-------------------------hhhhhllhHHHHhhHHHHHhhhhhhllllLLLL
Query LQ-------------------------llegllldADLXptRSACIaifearaqhpWPPR  245
ident                                      |
Sbjct RSmralaktkgmslsehalstavvrnthgckvgpgRVLP--TPTEK-------dvfRLLG  229
DSSP  HHhhhhhhhhlleeellllleeelleellllleeellLEEL--LLLHH-------hhhHHHL
```

```
DSSP  Llllllhhhhhhhhllllllllllhhhhhhhhhhhhhhhhllll
Query Vatlphwpliyagalegldhlelartvdaaaqavqrfvaridratkr  292
ident
Sbjct L-------------------------------------pyrepaerdw  240
DSSP  L-------------------------------------llllhhhlll
```

No 465: Query=mol1A Sbjct=5ca7A Z-score=5.3

back to top

```
DSSP  ----------------------llllllllllhhhhhhhhhhhhhhllllhHHHHHHHHH
Query ----------------------aapysspptnlrslrdrltqvaerqgvvfGRLQRHVAX   38
ident
Sbjct pvlelfsniwgagtktaqmwyqqgfrsledirsqaslttqqaiglkhysdfLERMPREEA   60
DSSP  llhhhhhllllllllhhhhhhhllllllhhhhhhhllllhhhhhhhhllllhhHLLEEHHHH
```

```
DSSP  HHHHHHHHLL-LLLLLLLLEEEEHHHHHHHhhllllllLLLLEEEEEL--------lLHH
Query IVVAQFAATL-TDDTGAPLLLVKGGSSLELrrgipdsrTSKDFATVAR--------rDIE   89
ident     |             |    |              |  |
Sbjct TEIEQTVQKAaQAFNSGLLCVACGSYRRGK-------aTCGDVDVLIThpdgrshrgIFS  113
DSSP  HHHHHHHHHHHhHLLLLLLLEEEELHHHHLLL-------lEELLEEEEEEllllllllHHH
```

```
DSSP  HHHHHHHHHHHhhllllleeEEELLLE-ELLLllLLLLLEEEEEEEEEEllEEEEEEEEEEEE
Query LIHEQLADAGetgwegftAIFTAPE-EIAApgXPVKPRRFTAKLSYrgRAFATVPIEVSS  148
ident      |   |                                 |
Sbjct RLLDSLRQEG--------FLTDDLVsQEEN--GQQQKYLGVCRLPG--PGRRHRRLDIIV  161
DSSP  HHHHHHHHLL--------LEEEEEEeEEEE--LLEEEEEEEELLLL--LLLLLEEEEEEE
```

```
DSSP  L-LLLLllllleeeeelhhhhllllllleeeeellhHHHHhhhhhhhhlllllllllllHH
Query V-EAGNadqfdtltsdalglvgvpaavavpcxtiPWQIaqklhavtavleepkandraHD  207
ident |
Sbjct VpYSEF---------------------------aCALL----------------yftgSA  178
DSSP  LlHHHH---------------------------hHHHH----------------hhhlLH
```

```
DSSP  HHHHH-------------------------hhhhhllhHHHHhhHHHHHhhhhhhllllL
Query LVDLQ-------------------------llegllldADLXptRSACIaifearaqhpW  242
ident                                         |
Sbjct HFNRSmralaktkgmslsehalstavvrnthgckvgpgRVLP--TPTEK-------dvfR  229
DSSP  HHHHHhhhhhhhhlleeellllleeelleellllleeellLEEL--LLLHH-------hhhH
```

```
DSSP  LLLLllllllhhhhhhhhllllllllllhhhhhhhhhhhhhhhhllll
Query PPRVatlphwpliyagalegldhlelartvdaaaqavqrfvaridratkr  292
ident
Sbjct LLGL-------------------------------------pyrepaerdw  243
DSSP  HLLL-------------------------------------llllhhhlll
```

No 466: Query=mol1A Sbjct=4k4iM Z-score=5.3

```
DSSP  ------------------------------------------------------------
Query ------------------------------------------------------------   0
ident
Sbjct atnhnlhiteklevlakaysvqgdkwralgyakainalksfhkpvtsyqeacsipgigkr  60
DSSP  llllhhhhhhhhhhhhhhhhhllhhhhhhhhhhhhhhhlllllllhhhhhlllllhh


DSSP  ----------------------------------------------llllllllllhhhh
Query ----------------------------------------------aapyssspptnlrsl  14
ident
Sbjct maekiieilesghlrkldhisesvpvlelfsniwgagtktaqmwyqqgfrsledirsqas  120
DSSP  hhhhhhhhhhhlllhhhhhllllhhhhhhhhhlllllhhhhhhhhhhllllhhhhhhhll


DSSP  hhhhhhhhhhllllhHHHHHHHHHHHHHHHHHHLL-LLLLLLLLEEEEHHHHHHHhhllll
Query rdrltqvaerqgvvfGRLQRHVAXIVVAQFAATL-TDDTGAPLLLVKGGSSLELrrgipd   73
ident                             |             |    |
Sbjct lttqqaiglkhysdfLERMPREEATEIEQTVQKAaQFANSGLLCVACGSYRRGK------  174
DSSP  llhhhhhhhhlhhhhHLLEEHHHHHHHHHHHHHHHhHLLLLLLEEEELHHHHLLL------


DSSP  llLLLLLEEEEEL--------lLHHHHHHHHHHHHhhlllleeeEELLL--EELLLllLLL
Query srTSKDFATVAR--------rDIELIHEQLADAGetgwegftaIFTAP--EEIAApgXPV  123
ident   |  |                       |   |           |
Sbjct -aTCGDVDVLIThpdgrshrgIFSRLLDSLRQEG---------FLTDDlvSQEEN--GQQ  222
DSSP  -lEELLEEEEEEllllllllHHHHHHHHHHHHLL---------LEEEEeeEEEEE--LLE


DSSP  LLEEEEEEEEEllEEEEEEEEEEEEL-LLLLllllleeeeelhhhhllllllleeeeellhH
Query KPRRFTAKLSYrgRAFATVPIEVSSV-EAGNadqfdtltsdalglvgvpaavavpcxtiP  182
ident         |                |
Sbjct QKYLGVCRLPG--PGRRHRRLDIIVVpYSEF---------------------------aC  253
DSSP  EEEEEEEELLL--LLLLEEEEEEEEELlHHHH---------------------------hH


DSSP  HHHhhhhhhhhlllllllllllHHHHHHH-------------------------hhhhh
Query WQIaqklhavtavleepkandraHDLVDLQ-------------------------llegl  217
ident
Sbjct ALL----------------yftgSAHFNRSmralaktkgmslsehalstavvrnthgckv  297
DSSP  HHH----------------hhhlLHHHHHHhhhhhhhlleeellllleeellllllllll


DSSP  llhHHHHhhHHHHHhhhhhhllllLLLLLlllllhhhhhhhhlllllllllllhhhhhh
Query lldADLXptRSACIaifearaqhpWPPRVatlphwpliyagalegldhlelartvdaaaq  277
ident      |
Sbjct gpgRVLP--TPTEK-------dvfRLLGL-------------------------------  317
DSSP  lllLEEL--LLLHH-------hhhHHHLL-------------------------------


DSSP  hhhhhhhhhhhllll
Query avqrfvaridratkr  292
ident
Sbjct ---pyrepaerdwle  329
DSSP  ---llllhhhlllll
```

No 467: Query=mol1A Sbjct=5decB Z-score=5.3

back to top

```
DSSP  lllllllllhhhhhhhhhhhhhHLLLLHHHHHHHHHHHHHHHHHHHHLLL--LLLLLLLEE
Query aapyssspptnlrslrdrltqvaeRQGVVFGRLQRHVAXIVVAQFAATLT--DDTGAPLLL   58
ident                                     |                   |
Sbjct -----------------------DDKWERFLVPYRQAVEELKVKLKGIRtlYEDHSPIEF   37
DSSP  -----------------------LHHHHHHHHHHHHHHHHHHHHHHHHHHHhhHLLLLLLE


DSSP  EEhhHHHHhhhlLLLLL------------------lLLLEEEEELL--LHHHHHHHHHHH
Query VKggSSLElrrgIPDSR------------------tSKDFATVARR--DIELIHEQLADA   98
ident |              |                                ||     | |
Sbjct VT--GRVK----PVASIlekarrksiplheietmqdIAGLRIMCQFvdDIQIVKEMLFAR   91
DSSP  EE--EEEL----LHHHHhhhhhhlllllllhhhlllLEEEEEEELLhhHHHHHHHHHHHL


DSSP  HhhlllleEEEELLLEEL-LLLLlllLLEEEEEEEEEL---lEEEEEEEEEEEELLllll
Query GetgwegfTAIFTAPEEI-AAPGxpvKPRRFTAKLSYR---gRAFATVPIEVSSVEagna  154
```

```
DSSP  L-------LLEEEEEEEELlLLLLlllLLEEEEEEEEEEEelleEEEEEEEEEEEEEEHH----


DSSP  llleeeeelhhhhllllllleeeeellhhhhhHHHHHHHHLllllllllllhhhhhhhhh
Query dqfdtltsdalglvgvpaavavpcxtipwqiAQKLHAVTAvleepkandrahdlvdlqll  214
ident
Sbjct ----------------------------mnfWATIEHSLN--------------------  152
DSSP  ----------------------------hhhHHHHHHHHH--------------------


DSSP  hhhllhhhhhhhhhhhhhhhhhhlllllllLLLLLLLHhhhhhhhhllllllllllhhh
Query eglllddadlxptrsaciaifearaqhpwppRVATLPHWpliyagalegldhlelartvda  274
ident
Sbjct ---------------------------ykySGNIPEKV------------klrlqrasea  173
DSSP  ---------------------------hhlLLLLLLHHH------------hhhhhhhhhh


DSSP  hhhhhhhhhhhhhhllll
Query aaqavqrfvaridratkr  292
ident
Sbjct asrldeemseirgevqea  191
DSSP  hhhhhhhhhhhhhhhhll
```

No 468: Query=mol1A Sbjct=5deeS Z-score=5.3

back to top

```
DSSP  lllllllllhhhhhhhhhhhhhHLLLLHHHHHHHHHHHHHHHHHHHLLL--LLLL---LL
Query aapysspptnlrslrdrltqvaeRQGVVFGRLQRHVAXIVVAQFAATLT--DDTG---AP   55
ident                                     |                      |
Sbjct -----------------------DKQWERFLVPYRQAVEELKVKLKGIRtlYEYEddhSP   37
DSSP  -----------------------LLLHHHHLHHHHHHHHHHHHHHHHHHHhhHHLLlllLL


DSSP  LEEEEhhHHHHhhhlLLLLL------------------lLLLEEEEELL--LHHHHHHHH
Query LLLVKggSSLElrrgIPDSR------------------tSKDFATVARR--DIELIHEQL   95
ident    |              |                                ||    | |
Sbjct IEFVT--GRVK----PVASIlekarrksiplheietmqdIAGLRIMCQFvdDIQIVKEML   91
DSSP  LLEEE--EEEL----LHHHHhhhhhhllllhhhhhhlllLEEEEEEEELLhhHHHHHHHHH


DSSP  HHHHhhllllleEEEELLleellllllllLLEEEEEEEEEL---lEEEEEEEEEEEELLll
Query ADAGetgwegfTAIFTApeeiaapgxpvKPRRFTAKLSYR---gRAFATVPIEVSSVEag  152
ident                                                  | |
Sbjct FARK-------DFTVVD-----------RSYHLVVLYPLQtvsgEKHVLVEIQIRTLA--  131
DSSP  HHLL-------LLEELL-----------LEEEEEEEEEEEllllEEEEEEEEEEEEEEHH--


DSSP  llllleeeeelhhhhllllllleeeeellhhhhhHHHHHHHHLllllllllllhhhhhhh
Query nadqfdtltsdalglvgvpaavavpcxtipwqiAQKLHAVTAvleepkandrahdlvdlq  212
ident
Sbjct ------------------------------mnfWATIEHSLN------------------  143
DSSP  ------------------------------hhhHHHHHHHHH------------------


DSSP  hhhhhllhhhhhhhhhhhhhhhhhhlllllllLLLLLLLHhhhhhhhhllllllllllh
Query lleglllddadlxptrsaciaifearaqhpwppRVATLPHWpliyagalegldhlelartv  272
ident
Sbjct -----------------------------ykySGNIPEKV------------klrlqras  162
DSSP  -----------------------------hhlLLLLLLHHH------------hhhhhhhh


DSSP  hhhhhhhhhhhhhhhhllll
Query daaaqavqrfvaridratkr  292
ident
Sbjct eaasrldeemseirgevqea  182
DSSP  hhhhhhhhhhhhhhhhhlll
```

No 469: Query=mol1A Sbjct=5deeQ Z-score=5.3

back to top

```
[illegible]
ident                                     |                      |
Sbjct ------------------------KQWERFLVPYRQAVEELKVKLKGIRtlYEYEddhSP   36
DSSP  ------------------------LLHHHHLHHHHHHHHHHHHHHHHHHhhHLLLlllLL


DSSP  LEEEEhhHHHHhhhlLLLLL------------------lLLLEEEEELL--LHHHHHHHH
Query LLLVKggSSLElrrgIPDSR------------------tSKDFATVARR--DIELIHEQL   95
ident    |              |                                ||    | |
Sbjct IEFVT--GRVK----PVASIlekarrksiplheietmqdIAGLRIMCQFvdDIQIVKEML   90
DSSP  LLEEE--EEEL----LLHHHhhhhhhlllllllhhhlllLEEEEEEELLhhHHHHHHHHH


DSSP  HHHHhhlllleEEEEllleellllllllLLEEEEEEEEEL---lEEEEEEEEEEEELLll
Query ADAGetgwegfTAIFtapeeiaapgxpvKPRRFTAKLSYR---gRAFATVPIEVSSVEag  152
ident                                                  | |
Sbjct FARK-------DFTV-------------RSYHLVVLYPLQtvsgEKHVLVEIQIRTLA--  128
DSSP  HHLL-------LLLL-------------LEEEEEEEEEEEllllEEEEEEEEEEEEHH--


DSSP  llllleeeeelhhhhlllllleeeeellhhhhhHHHHHHHHLllllllllllllhhhhhh
Query nadqfdtltsdalglvgvpaavavpcxtipwqiAQKLHAVTAvleepkandrahdlvdlq  212
ident
Sbjct ------------------------------mnfWATIEHSLN------------------  140
DSSP  ------------------------------hhhHHHHHHHHH------------------


DSSP  hhhhhllhhhhhhhhhhhhhhhhhhhllllllLLLLLLLHhhhhhhhllllllllllllh
Query llegllldadlxptrsaciaifearaqhpwppRVATLPHWpliyagalegldhlelartv  272
ident
Sbjct -----------------------------ykySGNIPEKV------------klrlqras  159
DSSP  -----------------------------hhlLLLLLHHH------------hhhhhhhh


DSSP  hhhhhhhhhhhhhhhhllll
Query daaaqavqrfvaridratkr  292
ident
Sbjct eaasrldeemseirgevqea  179
DSSP  hhhhhhhhhhhhhhhhhlll
```

No 470: Query=mol1A Sbjct=5f2vZ Z-score=5.3

back to top

```
DSSP  lllllllllhhhhhhhhhhhhhhhlLLLHHHHHHHHHHHHHHHHHHLLL--LLLL---LL
Query aapysspptnlrslrdrltqvaerqGVVFGRLQRHVAXIVVAQFAATLT--DDTG---AP   55
ident                                     |                      |
Sbjct -------------------------QWERFLVPYRQAVEELKVKLKGIRtlYEYEddhSP   35
DSSP  -------------------------LHHHHLHHHHHHHHHHHHHHHHHHhhHHHLlllLL


DSSP  LEEEEhhHHHHhhhlLLLLL------------------lLLLEEEEELL--LHHHHHHHH
Query LLLVKggSSLElrrgIPDSR------------------tSKDFATVARR--DIELIHEQL   95
ident    |              |                                ||    | |
Sbjct IEFVT--GRVK----PVASIlekarrksiplheietmqdIAGLRIMCQFvdDIQIVKEML   89
DSSP  LLEEE--EEEL----LHHHHhhhhhhlllllllhhhlllLEEEEEEELLhhHHHHHHHHH


DSSP  HHHHhhlllleEEEELlleellllllllLLEEEEEEEEEL---lEEEEEEEEEEEELLll
Query ADAGetgwegfTAIFTapeeiaapgxpvKPRRFTAKLSYR---gRAFATVPIEVSSVEag  152
ident                                                  | |
Sbjct FARK-------DFTVV------------RSYHLVVLYPLQtvsgEKHVLVEIQIRTLA--  128
DSSP  HLLL-------LLEEL------------LLLEEEEEEEEEelleEEEEEEEEEEEEHH--


DSSP  llllleeeeelhhhhlllllleeeeellhhhhhHHHHHHHHLllllllllllllhhhhhh
Query nadqfdtltsdalglvgvpaavavpcxtipwqiAQKLHAVTAvleepkandrahdlvdlq  212
ident
Sbjct ------------------------------mnfWATIEHSLN------------------  140
DSSP  ------------------------------hhhHHHHHHHHH------------------


DSSP  hhhhhllhhhhhhhhhhhhhhhhhhhllllllLLLLLLLHhhhhhhhllllllllllllh
Query llegllldadlxptrsaciaifearaqhpwppRVATLPHWpliyagalegldhlelartv  272
ident
Sbjct -----------------------------ykySGNIPEKV------------klrlqras  159
```

```
DSSP  hhhhhhhhhhhhhhhhllll
Query daaaqavqrfvaridratkr  292
ident
Sbjct eaasrldeemseirgevqea  179
DSSP  hhhhhhhhhhhhhhhhhhll
```

No 471: Query=mol1A Sbjct=3er9B Z-score=5.3

back to top

```
DSSP  ------------------------------------------------llllllLLLL--
Query ------------------------------------------------aapyssPPTN--    10
ident
Sbjct nitlkiietylgrvpsvneyhmlksqarniqkitvfnkdifvslvkknkkrffsDVNTsa    60
DSSP  lhhhhhhhhhhlllllhhhhhhhhhhhhhhhhhhhhllhhhhhhhhhhhhhhllLLLLlh


DSSP  -----------------------------------------------hhhhhhhhhhhhh
Query -----------------------------------------------lrslrdrltqvae    23
ident
Sbjct seikdrilsyfskqtqtynigklftiielqsvlvttytdilgvltinvtsmeelardmln   120
DSSP  hhhhhhhhhhllhhhhlllhhhhhhhhhhhhhhhhhhhhhhlhhhhllhhhhhhhhhhhh


DSSP  hllllhhhhhHHHHHHHHHHHHHHLLllllllLLEEEEHHHHHHHHHLllLLLLLlLEEEE
Query rqgvvfgrlqRHVAXIVVAQFAATLtddtgaPLLLVKGGSSLELRRGipDSRTSkDFATV    83
ident                                      |  || |       |   |
Sbjct smnvavvsslVKNVNKLMEEYLRRH-----nKSCICYGSYSLYLINP--NIRYG-DIDIL   172
DSSP  hhllllllllHHHHHHHHHHHHHHL-----lLLEEEELHHHHHLLLL--LLLLL-LLEEE


DSSP  ELLlHHHHHHHHHHHHHHLllleeeEELLlEELLLlllllLLEEEEEEeeelleeeEEEE
Query ARRdIELIHEQLADAGETGwegftaIFTApEEIAApgxpvKPRRFTAKlsyrgrafATVP   143
ident            ||            |
Sbjct QTN-SRTFLIDLAFLIKFI--tgnnIILS-KIPYL-----RNYMVIKD-----endNHII   218
DSSP  ELL-HHHHHHHHHHHHHHH--hlllLEEE-ELLLL-----LLEEEEEL-----lllLEEE


DSSP  EEEEE---LLLLLllLLEEEEelhhhhlllllleEEEELLHHHHHHHHHHHHHLLLllll
Query IEVSS---VEAGNadQFDTLTsdalglvgvpaavAVPCXTIPWQIAQKLHAVTAVLeepk   200
ident                                            |
Sbjct DSFNIrqdTMNVV--PKIFID-------------NIYIVDPTFQLLNMIKMFSQID---r   260
DSSP  EEEELlhhHHHHL--LEEEEL-------------LEEEELHHHHHHHHHHHLLLHH---h


DSSP  LLLL----LHHHHHHHHHHHHL--LHHH--------------------------------
Query ANDR----AHDLVDLQLLEGLL--LDAD--------------------------------   222
ident   |
Sbjct LEDLskdpEKFNARMATMLEYVryTHGIvfdgkrnnmpmkciidennrivtvttkdyfsf   320
DSSP  HHHHhhlhHHHHHHHHHHHHHHhhHHLLlllllllllllleeeelllleeeeelllllll


DSSP  -------------------------------hhhhhhhhhhhhhhHLLLL---------l
Query -------------------------------lxptrsaciaifeaRAQHP---------w   242
ident
Sbjct kkclvyldenvlssdildlnadtscdfesvtnsvylihdnimytyFSNTIllsdkgkvhe   380
DSSP  leeeeellhhhhhhhhhhhlllllleelhhhlleeeeelleeeeeLLLLLeeeelleelh


DSSP  LLLLlllllhhhhhhhhhllllllllllllhhhhhhhhhhHHHHH--------hHLLL---
Query PPRVatlphwpliyagalegldhlelartvdaaaqavqrFVARI--------dRATK---   291
ident
Sbjct ISAR---------------------------------glCAHILlyqmltsgeYKQClsd   407
DSSP  HHHH---------------------------------hhHHHHHhhhhhllllLHHHhhh


DSSP  -------------------------------------l
Query -------------------------------------r  292
ident
Sbjct llnsmmnrdkipiyshterdkkpgrhgfiniekdiivf  445
DSSP  hhhllllllllllleellllllllllleeeellllleeel
```

back to top

```
DSSP  lllllllllhhhhhhhhhhhhhhllllhhhhhhhhhhHHHHHHHHL-LLLLlllLLEEE
Query aapysspptnlrslrdrltqvaerqgvvfgrlqrhvaxIVVAQFAAT-LTDDtgaPLLLV   59
ident                                              |  |
Sbjct ----------------------------------lnfyLSYFDDVAKvLPRE---HYCFI   23
DSSP  ----------------------------------llllLLLHHHHHHhLLLL---LLEEE


DSSP  EHHHHHHHHHLLLlllllLLEEEEEELLLHHHHHHHHHHHHhhllllleeeEELLLEEL-LL
Query KGGSSLELRRGIPdsrtsKDFATVARRDIELIHEQLADAGetgwegftaIFTAPEEI-AA  118
ident  ||       | |      |       |        |
Sbjct VGGWVRDRILGEP-vgynIDVDFLTTADPVELAKNFAKRI---------GGHFFVFEkRG   73
DSSP  ELHHHHHHHLLLL-llleEEEEEEELLLHHHHHHHHHHHH---------LLEEELLLlLL


DSSP  LL-lLLLLLEEEEEEEEeelleEEEEEEEEEEEL-----LLLL---------------llll
Query PG-xPVKPRRFTAKLsyrgrAFATVPIEVSSV-----EAGN---------------adqf  157
ident               |              |       |
Sbjct FLikRPTIASVVLHL-----PPYRYRFDFSPLkgkdlEKALiedlkerdftanaiavnld  128
DSSP  LLllLLLLLEEEEEEL-----LLLEEEEEEEEELllllhHHHHhhhhlllllhhhlleeehh


DSSP  EEEEelhhhhlllllleeeeelLHHH-----------hhhhhhhHHHLlllllllLLLLH
Query DTLTsdalglvgvpaavavpcxTIPW-----------qiaqklhAVTAvleepkaNDRAH  206
ident | |                                                      |
Sbjct DVLS-----------igakqtiVYDPtggikdleqgllrpvsieNLKR------dPVRVL  171
DSSP  HHHL-----------lllllllEELLllhhhhhhlleellllhhHHHH------lLLHHH


DSSP  hhhhhhhhhhhllhhhhhhhhhhhhhhhhhhlllllllllllllhhhHHHHHHHLLLLll
Query dlvdlqllegllldadlxptrsaciaifearaqhpwpprvatlphwplIYAGALEGLDhl  266
ident
Sbjct ------------------------------------------------RGFRIAIEKN--  181
DSSP  ------------------------------------------------HHHHHHHHLL--


DSSP  lllLLHHHHHHHHHH---------------------------------------------
Query elaRTVDAAAQAVQR---------------------------------------------  281
ident             |
Sbjct --lQLTEDFYEFVKEdprivlksaverithelfkimkektahkvirelyeygvleaiipe  239
DSSP  --lEELHHHHHHHHHlhhhhhhllhhhhhhhhhhhhhlllhhhhhhhhhhllhhhhhlhh


DSSP  ------------------------------------------------------------
Query ------------------------------------------------------------  281
ident
Sbjct igrlrevkdpldehtlktleyleqviedrakylsaellenfgkkrvlgeftdvellkwga  299
DSSP  hhhhhllllhhhhhhhhhhhhhhhhhhlhhhhllhhhhhlllllleellleehhhhhhhhh


DSSP  ------------------------------------------------------------
Query ------------------------------------------------------------  281
ident
Sbjct lfhdigkpqtfvtfyehdkvgaqivreigerlrwgdeatefvaklvrhhlrpfflreafk  359
DSSP  hlllllllllllllllhhhhhhhhhhhhhhhhllllhhhhhhhhhhhhhlllhhhhhhhhhh


DSSP  -------------------------------------------------hhhhhhhllll
Query -------------------------------------------------fvaridratkr  292
ident
Sbjct kgelkrrgmanfwrecgdiaphlfllsiadamasgdeeedikalmetiaelesfnrnemk  419
DSSP  llllhhhhhhhhhhllllhhhhhhhhhhhhhhllllhhhhhhhhhhhhhhhhhhhhhllll
```

No 473: Query=mol1A Sbjct=5hc9B Z-score=5.3

back to top

```
DSSP  lllllllllhhhhhhhhhhhhhhllllhhhhhhHHHHHHHHHHHHHLLLLlllLLLEEEE
Query aapysspptnlrslrdrltqvaerqgvvfgrlqrHVAXIVVAQFAATLTDdtgAPLLLVK   60
ident                                                  |        |
Sbjct -------------------mqifrdvskllvervDPKILNLFRLLGKFGD-evNMPVYVV   40
DSSP  -------------------llleeelhhhhhhhlLHHHHHHHHHHHHHHHH-hhLLLEEEE
```

```
Query GGSSLELRRGIpdsrTSKDFATVARRDIELIHEQLADAGetgwegftAIFTAPEEiaapg  120
ident ||    |  ||       |   |         |
Sbjct GGFVRDLLLGI----KNLDIDIVVEGNALEFAEYAKRFL--------PGKLVKHD-----   83
DSSP  LHHHHHHHHLL----LLLLEEEEELLLHHHHHHHHHHHL--------LEEEEEEL-----


DSSP  lLLLLEeEEEEEeelleeeEEEEEEEEELLlLLLL-----------------llEEEEel
Query xPVKPRrFTAKLsyrgrafATVPIEVSSVEaGNAD-----------------qfDTLTsd  163
ident                        |
Sbjct -KFMTA-SLFLK-------GGLRIDIATAR-STIKkdlyrrdftinamaiklnpKDFG--  131
DSSP  -LLLEE-EEEEL-------LLLEEEEEELL-LLLHhhhhllllhhhlleeellLLLL--


DSSP  hhhhllllleeeeelLHHH---------hhhhhhhhHHLLllllllLLLLhhhhhhhhh
Query alglvgvpaavavpcxTIPW---------qiaqklhaVTAVleepkaNDRAhdlvdlqll  214
ident
Sbjct --------------llIDFFggyrdlkegvirvlhtlSFVD------DPTR---------  162
DSSP  --------------eeELLLlhhhhhhlleellllllHHHH------LLLH---------


DSSP  hhhllhhhhhhhhhhhhhhhhhhhllllllllllllhhHHHHHHHHLLLLlllllLLHHH
Query eglllddadlxptrsaciaifearaqhpwpprvatlphwpLIYAGALEGLDhlelaRTVDA  274
ident                                        |         |     |
Sbjct ---------------------------------------iLRAIRFEQRFD----fRIEET  180
DSSP  ---------------------------------------hHHHHHHHHHHL----lEELHH


DSSP  HHHH--------------------------------------------------------
Query AAQA--------------------------------------------------------  278
ident
Sbjct TERLlkqaveegylerttgprlrqelekileeknplksirrmaqfdvikhlfpktyytps  240
DSSP  HHHHhhhhhhllhhhhllhhhhhhhhhhhhllllhhhhhhhhhhllhhhhhllllllllhh


DSSP  ------------------------------------------------------------
Query ------------------------------------------------------------  278
ident
Sbjct mdekmenlfrnipwveenfgevdrfyavlhvflefyddeswkevrdryslrrnlineirh  300
DSSP  hhhhhhhhhhhhhhhhhhhhllllhhhhhhhhhlllllhhhhhhhhhhllllllhhhhhhh


DSSP  ------------------------------------------------------------
Query ------------------------------------------------------------  278
ident
Sbjct veksapallemlservpasfvyplvkgvsnetichflaylsgekeglfksyllkikntkl  360
DSSP  hhhhhhhhhhhhhllllhhhhhhhhllllhhhhhhhhllllhhhhhhhhhhhhhhhllll


DSSP  ------------------------------------hhhhhhhhhhllll
Query ------------------------------------vqrfvaridratkr  292
ident
Sbjct ekingeylirkgitsgkiigevlekilmkkldgdtrdeeeileevlasle  410
DSSP  lllhhhhhhllllllhhhhhhhhhhhhhhhllllllhhhhhhhhhhhhll
```

No 474: Query=mol1A Sbjct=4rwnA Z-score=5.2

back to top

```
DSSP  lllllllllhhhhhhhhhhhhHHLL--LLHHHHHHHHHHHHHHHHHHH--LLLLLLLLLL
Query aapysspptnlrslrdrltqvaERQG--VVFGRLQRHVAXIVVAQFAA--TLTDDTGAPL   56
ident                       |         | |   |   |  |
Sbjct -------melrhtpardldkfiEDHLlpNTCFRTQVKEAIDIVCRFLKerCFQGTADPVR   53
DSSP  -------llhhhllhhhhhhhhHHHLllLHHHHHHHHHHHHHHHHHHHHhlLLLLLLLLL


DSSP  E-EEEHHHhhHHHHLLLlllLLLLEEEEELL---------LHHHHHHHHHHHHHHLL--L
Query L-LVKGGSslELRRGIPdsrTSKDFATVARR---------DIELIHEQLADAGETGW--E  104
ident    |  |                |                             |
Sbjct VsKVVKGG-sSGKGTTLrgrSDADLVVFLTKltsfedqlrRRGEFIQEIRRQLEACQreQ  112
DSSP  EeEEEEEH-hHHHLLLLlllLLEEEEEEEELLlllhhhhhhHHHHHHHHHHHHHHHHhlL


DSSP  LEEEEELlleellllllllllEEEEEEEEEL-leeEEEEEEEEEELLLLLllllleeeeel
Query GFTAIFTapeeiaapgxpvkpRRFTAKLSYR-graFATVPIEVSSVEAGNadqfdtltsd  163
ident  |   |               |     ||                   |
```

```
DSSP  hhhhllllleeeeellhhhhhhhhhhhhhhhllllllllllhhhhhhhhhhhhhllhhhh
Query alglvgvpaavavpcxtipwqiaqklhavtavleepkandrahdlvdlqllegllldadl  223
ident 
Sbjct ------------------------------------------------------------  157
DSSP  ------------------------------------------------------------


DSSP  hHHHHhhHHHHHHHLL--------------------------------------------
Query xPTRSacIAIFEARAQ--------------------------------------------  239
ident            |   |
Sbjct wTPGY--KPNPEIYVQlikecksrgkegefstcftelqrdflrnrptklkslirlvkhwy  215
DSSP  lLLLL--LLLHHHHHHhhhhhhhhlllllhhhhhhhhhhhhhhhlllhhhhhhhhhhhhh


DSSP  --llllllllllllhHHHHHH-----------------hhlllllllllllhHHHH----
Query --hpwpprvatlphwPLIYAG-----------------alegldhlelartvDAAA----  276
ident                 |
Sbjct qtckkthgnklppqyALELLTvyaweqgsrktdfstaqgfqtvlelvlkhqkLCIFweay  275
DSSP  hhhhhhlllllllhhHHHHHHhhhhhhhllllllhhhhhhhhhhhhhhlhhhLEELllll


DSSP  ----------------------------------------------------------hh
Query ----------------------------------------------------------qa  278
ident 
Sbjct ydftnpvvgrcmlqqlkkprpvildpadptgnvgggdthswqrlaqearvwlgypccknl  335
DSSP  llllhhhhhhhhhhhllllllee lllllllllllllhhhhhhhhhhhhhhhlllhhhlll


DSSP  hhhhhhhhhhllll
Query vqrfvaridratkr  292
ident 
Sbjct dgslvgawtmlqki  349
DSSP  llllllllllllll
```

No 475: Query=mol1A Sbjct=4at8A Z-score=5.2

back to top

```
DSSP  -------------llllllllllhhhhhhhhhhhhhhllLLHHHHHHHHHHHHHHHHHHL
Query -------------aapysspptnlrslrdrltqvaerqgVVFGRLQRHVAXIVVAQFAAT   47
ident 
Sbjct shipfdfylcemafprvkpapdetsfseallkrnqdlapNSAEQASILSLVTKINNVIDN   60
DSSP  lllllhhhlllllllllllllllhhhhhhhhhhhhhhllLHHHHHHHHHHHHHHHHHHHH


DSSP  L-LLLLL-LLLE-EEEHHhhhhhhhlllLLLL-----lLLEEEEEL--LLHHHHHHHHHH
Query L-TDDTG-APLL-LVKGGsslelrrgipDSRT-----sKDFATVAR--RDIELIHEQLAD   97
ident |             |                        |           |
Sbjct LiVAPGTfEVQIeEVRQV------gsykKGTMttghnvADLVVILKilPTLEAVAALGNK  114
DSSP  HhLLLLLlLLLEeEEEEE------lhhhHLLLllllleEEEEEEELllLLHHHHHHHHHH


DSSP  HHHH-LLLL--eeEEELLLEellllllllllEEEEEEEeelleEEEEEEEEEEELLLLLl
Query AGET-GWEG--ftAIFTAPEeiaapgxpvkpRRFTAKLsyrgrAFATVPIEVSSVEAGNa  154
ident   |                |             |           ||| |    |
Sbjct VVESlRAQDpsevLTMLTNE-----------TGFEISS-----SDATVKILITTVPPNL-  157
DSSP  HHHHhHHHLllllLEEEELL-----------LEEEEEL-----LLLEEEEEEEELHHHH-


DSSP  llleeeeelhhhhlllllleeeeellhhhhhhHHHHHHhllllllllLLLLhhhhhhhhh
Query dqfdtltsdalglvgvpaavavpcxtipwqiaQKLHAVtavleepkaNDRAhdlvdlqll  214
ident 
Sbjct ------------rkldpelhldikvlqsalaaIRHARW-------feENAS---------  189
DSSP  ------------llllllllllhhhhhhhhhhHHHHHH-------hhHHLL---------


DSSP  hhhllhhhhhhhhhhhhhhhhhhllllllllllllllHHHHHHHHHLlLLLL--------
Query egllldadlxptrsaciaifearaqhpwpprvatlphWPLIYAGALEgLDHL--------  266
ident                                                  |
Sbjct -------------------------------------QSTVKVLIRLlKDLRirfpgfep  212
DSSP  -------------------------------------LHHHHHHHHHhHHHHhhlhhhll
```

```
ident              |
Sbjct ltpwildllghyAVMNnptrqplalnvayrrclqilaaglflpgsvgITDP-cESGNfrv  271
DSSP  llhhhhhhhhhhHHHLllllllllhhhhhhhhhhhhhllllllllllLLLL-lLLLLllh


DSSP  -----------------------------------------------------------l
Query -----------------------------------------------------------t  290
ident
Sbjct htvmtleqqdmvcytaqtlvrilshggfrkilgqegdasylaseistwdgvivtpsekay  331
DSSP  hhhllhhhhhhhhhhhhhhhhhhlllhhhhhllllllhhhhllleeelleeellllllll


DSSP  ll
Query kr  292
ident
Sbjct ek  333
DSSP  ll
```

No 476: Query=mol1A Sbjct=4at7A Z-score=5.2

back to top

```
DSSP  -------------lllllllllhhhhhhhhhhhhhlLLLHHHHHHHHHHHHHHHHHHL
Query -------------aapysspptnlrslrdrltqvaerqGVVFGRLQRHVAXIVVAQFAAT   47
ident
Sbjct shipfdfylcemafprvkpapdetsfseallkrnqdlaPNSAEQASILSLVTKINNVIDN   60
DSSP  llllllhhhlhhhllllllllllhhhhhhhhhhhhhlLLHHHHHHHHHHHHHHHHHHHH


DSSP  L--LLLLL-lLLEEEEHHhhhhhhhlllLLLL-----lLLEEEEEL--LLHHHHHHHHHH
Query L--TDDTG-aPLLLVKGGsslelrrgipDSRT-----sKDFATVAR--RDIELIHEQLAD   97
ident |     |       |                        |           |
Sbjct LivAPGTFevQIEEVRQV------gsykKGTMttghnvADLVVILKilPTLEAVAALGNK  114
DSSP  HhhLLLLLllLEEEEEEE------hhhhHLLLllllleEEEEEEELllLLHHHHHHHHHH


DSSP  HHHH-LLLL--eeEEELLLEelllllllllEEEEEEEeelleEEEEEEEEEEELLLLLl
Query AGET-GWEG--ftAIFTAPEeiaapgxpvkpRRFTAKLsyrgrAFATVPIEVSSVEAGna  154
ident   |                |             |           ||| |    |
Sbjct VVESlRAQDpsevLTMLTNE-----------TGFEISS-----SDATVKILITTVPPN--  156
DSSP  HHHHhHHHLllllLEEEEEL-----------LEEEEEL-----LLLEEEEEEEELHHH--


DSSP  llleeeeelhhhhllllleeeeellhhhhhhHHHHHHhlllllllLLLLhhhhhhhh
Query dqfdtltsdalglvgvpaavavpcxtipwqiaQKLHAVtavleepkaNDRAhdlvdlqll  214
ident
Sbjct ----------lrkldpelhldikvlqsalaaiRHARWF--------eENAS---------  189
DSSP  ----------hhllllllllhhhhhhhhhhHHHHHH--------hHHLL---------


DSSP  hhhllhhhhhhhhhhhhhhhhhllllllllllllhhHHHHHHHHLLL-LLLL------
Query eglllldadlxptrsaciaifearaqhpwpprvatlphwpLIYAGALEGL-DHLE------  267
ident                                              |  |   |
Sbjct ---------------------------------------QSTVKVLIRLlKDLRirfpgf  210
DSSP  ---------------------------------------LHHHHHHHHHhHHHHhhlhhh


DSSP  -----------lllLHHH-------------------------hhhhhHHHH------HH
Query -----------larTVDA-------------------------aaqavQRFV------AR  285
ident                |                                           |
Sbjct epltpwildllghyAVMNnptrqplalnvayrrclqilaaglflpgsvGITDpcesgnFR  270
DSSP  llllhhhhhhhhhhHHHLllllllllhhhhhhhhhhhhhlllllllllLLLLllllllLL


DSSP  HHHLLLL-----------------------------------------------------
Query IDRATKR-----------------------------------------------------  292
ident
Sbjct VHTVMTLeqqdmvcytaqtlvrilshggfrkilgqegdasylaseistwdgvivtpseka  330
DSSP  HHHHLLHhhhhhhhhhhhhhhhhhhlllhhhhhllllllhhhhllleeelleeellllll


DSSP  ----
Query ----  292
ident
Sbjct yekp  334
```

No 477: Query=mol1A Sbjct=5dee0 Z-score=5.2

back to top

```
DSSP  lllllllllhhhhhhhhhhhhhHLLLLHHHHHHHHHHHHHHHHHHHLLL--LLLL---LL
Query aapysspptnlrslrdrltqvaeRQGVVFGRLQRHVAXIVVAQFAATLT--DDTG---AP   55
ident                                     |                      |
Sbjct -----------------------DKQWERFLVPYRQAVEELKVKLKGIRtlYEYEddhSP   37
DSSP  -----------------------LLLHHHHLHHHHHHHHHHHHHHHHHHhhHHHLlllLL


DSSP  LEEEEhhHHHHhhhlLLLLL------------------lLLLEEEEELL--LHHHHHHHH
Query LLLVKggSSLElrrgIPDSR------------------tSKDFATVARR--DIELIHEQL   95
ident    |              |                                ||    | |
Sbjct IEFVT--GRVK----PVASIlekarrksiplheietmqdIAGLRIMCQFvdDIQIVKEML   91
DSSP  LLEEE--EEEL----LHHHHhhhhhhllllhhhhhhlllLEEEEEEELLhhHHHHHHHHH


DSSP  HHHHhhlllleEEEELLleellllllLLLLEEEEEEEEEL---lEEEEEEEEEEEELLll
Query ADAGetgwegfTAIFTApeeiaapgxPVKPRRFTAKLSYR---gRAFATVPIEVSSVEag  152
ident                                                  | |
Sbjct FARK-------DFTVVD---------GYRSYHLVVLYPLQtvsgEKHVLVEIQIRTLA--  133
DSSP  HLLL-------LLEEEL---------LLLEEEEEEEEEEEelleEEEEEEEEEEEEHH--


DSSP  llllleeeeelhhhhllllllleeeeellhhhhhHHHHHHHHLllllllllllhhhhhhh
Query nadqfdtltsdalglvgvpaavavpcxtipwqiAQKLHAVTAvleepkandrahdlvdlq  212
ident
Sbjct ------------------------------mnfWATIEHSLN------------------  145
DSSP  ------------------------------hhhHHHHHHHHH------------------


DSSP  hhhhhllhhhhhhhhhhhhhhhhhlllllllLLLLLLHhhhhhhhlllllllllllh
Query lleglllldadlxptrsaciaifearaqhpwpprVATLPHWpliyagalegldhlelartv  272
ident
Sbjct -----------------------------ykysGNIPEKV------------klrlqras  164
DSSP  -----------------------------hhllLLLLHHH------------hhhhhhhh


DSSP  hhhhhhhhhhhhhhhhllll
Query daaaqavqrfvaridratkr  292
ident
Sbjct eaasrldeemseirgevqea  184
DSSP  hhhhhhhhhhhhhhhhhhll
```

No 478: Query=mol1A Sbjct=5a30B Z-score=5.2

back to top

```
DSSP  -----------------------------LLLL-----------lllLLLHHHHhhHHHH
Query -----------------------------AAPY-----------sspPTNLRSLrdRLTQ   20
ident                                                  |       |
Sbjct kktftevqterleqadrsvlikcpsklneKKLLqylsshgkidnyffFENRGIH--ALIE   58
DSSP  lllhhhhhhhhhhhhhleeeeellllllhHHHHhhhhllllleeeeeEELLLEE--EEEE


DSSP  ------------------------------------------------------------
Query ------------------------------------------------------------   20
ident
Sbjct fsekssvaslqavtgipkhvvpyksrlftftlknpgsqaaeerpvkispqshipvnelip  118
DSSP  elllhhhhhhhhllllllllllllleeeeeelllllllllllllllllllllllhhhhhh


DSSP  ---------------hhhhllLLHHHHHHHHHHHHHHHHHHHHLLLlllllLLEEEEHHhhh
Query ---------------vaerqgVVFGRLQRHVAXIVVAQFAATLTddtgaPLLLVKGGssl   65
ident                             |  |   |  ||         |   ||
Sbjct klchadsissqmyillneyqlTEENIKLRYLACSLVRDFARAYF-----PDSTVKPF--g  171
DSSP  hhhllllhhhhhhhhhhhhllLHHHHHHHHHHHHHHHHHHHHLLLL-----LLEEEEEE--h


DSSP  hhhhllllllllLLEEEEEL------------------lLHHHHHHHHHHHHHHHLLllEE
Query elrrgipdsrtsKDFATVAR------------------rDIELIHEQLADAGETGWegFT  107
```

```
DSSP  hhllllllllllEEEEEEELlllleeeeellllllhhhHHHHHHHHHHHHHHHLL--LL


DSSP  EE-ELLLEELlllllllLLEEEEEEEEEEllleeEEEEEEEEEEellllllllleeeeelhhh
Query AI-FTAPEEIaapgxpvKPRRFTAKLSYrgraFATVPIEVSsveagnadqfdtltsdalg  166
ident                                        ||
Sbjct YSsVQKILNA-------RCPLVKFSHQP---tGFQCDLSVS-------------------  260
DSSP  EEeEEEELLL-------LLLEEEEEEELL---lLEEEEEEEL-------------------


DSSP  hllllleeeeellhhhhhhHHHHHHhllllllllLLLLhhhhhhhhhhhllhhhhhhh
Query lvgvpaavavpcxtipwqiaQKLHAVtavleepkaNDRAhdlvdlqllegllldadlxpt  226
ident
Sbjct ----------------nsiaIRCSEL---------LYIY---------------------  274
DSSP  ----------------lhhhHHHHHH---------HHHH---------------------


DSSP  hhhhhhhhhhlllllllllllLHHHhHHHHHL-lLLLL------------------l
Query rsaciaifearaqhpwpprvatlpHWPLiYAGALE-gLDHL------------------e  267
ident
Sbjct -----------------------gCLDPrVRALVFslRCWArvhgltnsvpgtwitnfsl  311
DSSP  -----------------------hHHLHhHHHHHHhhHHHHhhlllllllllllllhhhh


DSSP  lllLHHHHHHH-------------------------------------------------
Query larTVDAAAQA-------------------------------------------------  278
ident
Sbjct tmmIMFFLQKRsppiiptldqlkeladekdkhviggydcsfvsdlskikptkntetldel  371
DSSP  hhhHHHHHHLLlllllllhhhhhhllllllleelleellllllllllllllllllhhhh


DSSP  -----------------hhhhhhhhhHLLLL-----------------------------
Query -----------------vqrfvaridRATKR-----------------------------  292
ident
Sbjct lcdffqyfgnfdfrknslnlrkgkevNKPESsplyiwdpfeqdlnisknvnqpqlekfva  431
DSSP  hhhhhhhhhlllllleeeellllleeeELLLLllllllllllllllllllhhhhhhhhh


DSSP  ---------------------------------
Query ---------------------------------  292
ident
Sbjct maresawilqkedktqqminkepwglaavlipf  464
DSSP  hhhhhhhhhhhllhhhhhhlllllhhhhllll
```

No 479: Query=mol1A Sbjct=5f2vS Z-score=5.2

back to top

```
DSSP  lllllllllhhhhhhhhhhhhHLLLLHHHHHHHHHHHHHHHHHHLLL--LLLL---LL
Query aapyssppptnlrslrdrltqvaeRQGVVFGRLQRHVAXIVVAQFAATLT--DDTG---AP   55
ident                                      |                      |
Sbjct -----------------------DKQWERFLVPYRQAVEELKVKLKGIRtlYEYEddhSP   37
DSSP  -----------------------LLLHHHHLHHHHHHHHHHHHHHHHHHHhhHHLLlllLL


DSSP  LEEEEhhHHHHhhhlLLLLL-------------------lLLLEEEEELL--LHHHHHHHH
Query LLLVKggSSLElrrgIPDSR-------------------tSKDFATVARR--DIELIHEQL   95
ident    |              |                                ||    | |
Sbjct IEFVT--GRVK----PVASIlekarrksiplheietmqdIAGLRIMCQFvdDIQIVKEML   91
DSSP  LLEEE--EEEL----LHHHHhhhhhhllllhhhhhhlllLEEEEEEEELLhhHHHHHHHHH


DSSP  HHHHhhlllleEEEELLleellllllllLLEEEEEEEEEL---lEEEEEEEEEEEEELLll
Query ADAGetgwegfTAIFTApeeiaapgxpvKPRRFTAKLSYR---gRAFATVPIEVSSVEag  152
ident                                                  | |
Sbjct FARK-------DFTVVD-----------RSYHLVVLYPLQtvsgEKHVLVEIQIRTLA--  131
DSSP  HHLL-------LLEELL-----------LEEEEEEEEEEEllllEEEEEEEEEEEEEHH--


DSSP  lllllleeeeelhhhhllllllleeeeellhhhhhHHHHHHHHLlllllllllllhhhhhhh
Query nadqfdtltsdalglvgvpaavavpcxtipwqiAQKLHAVTAvleepkandrahdlvdlq  212
ident
Sbjct ------------------------------mnfWATIEHSLN------------------  143
DSSP  ------------------------------hhhHHHHHHHHH------------------
```

```
Query llegllldadlxptrsaciaifearaqhpwpprVATLPHWpliyagalegldhlelartv  272
ident
Sbjct -----------------------------ykysGNIPEKV------------klrlqras  162
DSSP  -----------------------------hhllLLLLHHH------------hhhhhhhh


DSSP  hhhhhhhhhhhhhhhhllll
Query daaaqavqrfvaridratkr  292
ident
Sbjct eaasrldeemseirgevqea  182
DSSP  hhhhhhhhhhhhhhhhhlll
```

No 480: Query=mol1A Sbjct=5deeX Z-score=5.2

back to top

```
DSSP  lllllllllhhhhhhhhhhhhhHLLLLHHHHHHHHHHHHHHHHHHHLLL--LLLL---ll
Query aapysspptnlrslrdrltqvaeRQGVVFGRLQRHVAXIVVAQFAATLT--DDTG---ap   55
ident                                     |
Sbjct -----------------------DKQWERFLVPYRQAVEELKVKLKGIRtlYEYEddhsp   37
DSSP  -----------------------LLLLLLLLHHHHHHHHHHHHHHHHHHHhhHLLLlllll


DSSP  LEEEEhhHHHHhhhlLLLLL------------------lLLLEEEEELL--LHHHHHHHH
Query LLLVKggSSLElrrgIPDSR------------------tSKDFATVARR--DIELIHEQL   95
ident    |              |                                ||    | |
Sbjct IEFVT--GRVK----PVASIlekarrksiplheietmqdIAGLRIMCQFvdDIQIVKEML   91
DSSP  LLEEE--EEEL----LHHHHhhhhhhllllhhhhhhlllLEEEEEEELLhhHHHHHHHHH


DSSP  HHHHhhllllеEEEELlleellllllllLLEEEEEEEEEL---lEEEEEEEEEEEELLll
Query ADAGetgwegfTAIFTapeeiaapgxpvKPRRFTAKLSYR---gRAFATVPIEVSSVEag  152
ident                                                  | |
Sbjct FARK-------DFTVV------------RSYHLVVLYPLQtvsgEKHVLVEIQIRTLA--  130
DSSP  HHLL-------LLEEL------------LEEEEEEEEEEEelleEEEEEEEEEEEEHH--


DSSP  llllleeeeelhhhhllllleeeeellhhhhhHHHHHHHHLllllllllllhhhhhhh
Query nadqfdtltsdalglvgvpaavavpcxtipwqiAQKLHAVTAvleepkandrahdlvdlq  212
ident
Sbjct ------------------------------mnfWATIEHSLN------------------  142
DSSP  ------------------------------hhhHHHHHHHHH------------------


DSSP  hhhhhllhhhhhhhhhhhhhhhhhllllllllLLLLLLHhhhhhhhhlllllllllllh
Query lleglllldadlxptrsaciaifearaqhpwpprVATLPHWpliyagalegldhlelartv  272
ident
Sbjct -----------------------------ykysGNIPEKV------------klrlqras  161
DSSP  -----------------------------hhllLLLLLLLH------------hhhhhhhh


DSSP  hhhhhhhhhhhhhhhhllll
Query daaaqavqrfvaridratkr  292
ident
Sbjct eaasrldeemseirgevqea  181
DSSP  hhhhhhhhhhhhhhhhhlll
```

No 481: Query=mol1A Sbjct=4e7xC Z-score=5.2

back to top

```
DSSP  lllllllllhhhhhhhhhhhhhllLLHHHHHHHHHHHHHHHHHHHHLLLlllllLLEEEE
Query aapysspptnlrslrdrltqvaerqgVVFGRLQRHVAXIVVAQFAATLTddtgaPLLLVK   60
ident                                     |                 |
Sbjct ---------hkeftkfcyevyneikiSDKEFKEKRAALDTLRLCLKRIS-----PDAELV   46
DSSP  ---------llhhhhhhhhhhhhhllLHHHHHHHHHHHHHHHHHHHHHL-----LLLEEE


DSSP  HHhhhhhhhlllLLLL----lLLEEEEEELLlhhHHHHHHHHHHHHHLllleEEEELLLEEl
Query GGsslelrrgipDSRT----sKDFATVARRdieLIHEQLADAGETGwegfTAIFTAPEEi  116
ident                       |           |  |
Sbjct AF------gsleSGLAlknsdMDLCVLMDD---TIALQFYEELIAE----GFEGKFLQR-   92
```

```
DSSP  lllllllLLEEEEEEEEeelleeEEEEEEEEEellllllllleeeeelhhhhllllllleee
Query aapgxpvKPRRFTAKLsyrgraFATVPIEVSsveagnadqfdtltsdalglvgvpaavav  176
ident                            |
Sbjct ------aRIPIIKLTS------SFQCDIGFN-----------------------------  111
DSSP  ------lLEEEEEELL------LLLEEEEEL-----------------------------


DSSP  eellhhhhhhHHHHhllllllllllllhhhhhhhhhhhllhhhhhhhhhhhhhhhhhhhh
Query pcxtipwqiaqKLHAVtavleepkandrahdlvdlqllegllldadlxptrsaciaifea  236
ident
Sbjct ------nrlaiHNTLL--------------------------------------------  121
DSSP  ------lhhhhHHHHH--------------------------------------------


DSSP  hllllllllllllllHHHHhHHHHLL--LLLL-----------------llllLHHHHHH
Query raqhpwpprvatlphWPLIyAGALEG--LDHL-----------------elarTVDAAAQ  277
ident
Sbjct ---------lssytkLDAR-LKPMVLlvKHWAkrkqinspyfgtlssygyvlmVLYYLIH  171
DSSP  ---------hhhhhhHLLL-HHHHHHhhHHHHhhlllllhhhlllhhhhhhhhHHHHHHH


DSSP  ------------------------------------------------------------
Query ------------------------------------------------------------  277
ident
Sbjct vikppvfpnlllsplkqekivdgfdvgfddkledippsqnysslgsllhgffrfyaykfe  231
DSSP  llllllllllllllllleelleellllllhhhllllllllllhhhhhhhhhhhhhhhlll


DSSP  -----hhHHHH---hhhhHLLLL-------------------------------------
Query -----avQRFV---aridRATKR-------------------------------------  292
ident         |
Sbjct prekvvtFRRPdgyltkqEKGWTsatedryilaiedpfeishnvgrtvsssglyrirgef  291
DSSP  lllleelLLLLlllllhhHHLLLlllllllleellllllllllllllllhhhhhhhhhh


DSSP  -------------------------
Query -------------------------  292
ident
Sbjct maasrllnsrsypipydslfeeapi  316
DSSP  hhhhhhllllllllhhhhhllllll
```

No 482: Query=mol1A Sbjct=4e80C Z-score=5.2

back to top

```
DSSP  lllllllllhhhhhhhhhhhhhhhllLLHHHHHHHHHHHHHHHHHHLLLlllllLLEEEE
Query aapysspptnlrslrdrltqvaerqgVVFGRLQRHVAXIVVAQFAATLTddtgaPLLLVK   60
ident                                     |                 |
Sbjct ----------keftkfcyevyneikiSDKEFKEKRAALDTLRLCLKRIS-----PDAELV   45
DSSP  ----------llhhhhhhhhhhhhllLHHHHHHHHHHHHHHHHHHHLLL-----LLLEEE


DSSP  HHhhhhhhhllllLLLL---------LLEEEEELllhHHHHHHHHHHHHHLllleEEEEL
Query GGsslelrrgipdSRTS---------KDFATVARrdiELIHEQLADAGETGwegfTAIFT  111
ident                            |           |  |
Sbjct AF-----------GSLEsglalknsdMDLCVLMDsrsDTIALQFYEELIAE----GFEGK   90
DSSP  EE-----------LHHHhllllllllEEEEEELLlllLLHHHHHHHHHHHH----LLEEE


DSSP  LLEEllllllLLLEEEEEEEEelleeeEEEEEEEEelllllllllleeeeelhhhhllll
Query APEEiaapgxpVKPRRFTAKLSyrgrafATVPIEVSsveagnadqfdtltsdalglvgvp  171
ident                                 |
Sbjct FLQR-------ARIPIIKLTSD------FQCDIGFN------------------------  113
DSSP  EEEE-------LLEEEEEEEEL------LLEEEEEL------------------------


DSSP  lleeeeellhhhhhhhhhhhhhhlllllllllLLHHhhhhhhhhhhllhhhhhhhhhhhh
Query aavavpcxtipwqiaqklhavtavleepkandRAHDlvdlqllegllldadlxptrsaci  231
ident
Sbjct ------------------------nrlaihntLLLS------------------------  125
DSSP  ------------------------lhhhhhhhHHHH------------------------


DSSP  hhhhhllllllllllllllLHHHhHHHHHL-lLLLL------------------lLLLLh
```

```
Sbjct ---------------sytkLDAR-LKPMVLlvKHWAkrkqinspyfgtlssygyvLMVL-  168
DSSP  ---------------hhhhHLLL-HHHHHHhhHHHHhhlllllhhhllllhhhhhHHHH-


DSSP  hHHHH-------------------------------------------------------
Query dAAAQ-------------------------------------------------------  277
ident
Sbjct yYLIHvikppvfpnlllsplkqekivdgfdvgfddkledippsqnysslgsllhgffrfy  228
DSSP  hHHHHlllllllllhhhllllllleelleelllllhhhllllllllllhhhhhhhhhhhh


DSSP  --------hhhHHHH-----hhhHLLLL--------------------------------
Query --------avqRFVA-----ridRATKR--------------------------------  292
ident             |
Sbjct aykfeprekvvTFRRpdgyltkqEKGWTsatehtgsadqiikdryilaiedpfeishnvg  288
DSSP  hhlllllllllLLLLllllllhhHHLLLlleeeellllleeeelllllllllllllllll


DSSP  ------------------------------------------
Query ------------------------------------------  292
ident
Sbjct rtvsssglyrirgefmaasrllnsrsypipydslfeeapipp  330
DSSP  llllhhhhhhhhhhhhhhhhhllllllllhhhhhllllllll
```

No 483: Query=mol1A Sbjct=3mdcA Z-score=5.2

back to top

```
DSSP  ------------------------------------------------------------
Query ------------------------------------------------------------    0
ident
Sbjct lhiteklevlakaysvqgdkwralgyakainalksfhkpvtsyqeacsipgigkrmaeki   60
DSSP  llhhhhhhhhhhhhhhlllhhhhhhhhhhhhhhhlllllllhhhhhlllllllhhhhhhh


DSSP  -----------------------------------------lllllllllhhhhhhhhhh
Query -----------------------------------------aapysspptnlrslrdrlt   19
ident
Sbjct ieilesghlrkldhisesvpvlelfsniwgagtktaqmwyqqgfrsledirsqaslttqq  120
DSSP  hhhhhhlllhhhhhllllhhhhhhhllllllhhhhhhhhhlllllhhhhhhhhllllhhh


DSSP  hhhhhllllhhhhHHHHHHHHHHHHHHL-LLLLLLLLLEEEEHHHHHHHHhhlllllLLL
Query qvaerqgvvfgrlQRHVAXIVVAQFAAT-LTDDTGAPLLLVKGGSSLELrrgipdsrTSK   78
ident                        |             |    |              |
Sbjct aiglkhysdflerMPREEATEIEQTVQKaAQAFNSGLLCVACGSYRRGK-------aTCG  173
DSSP  hhhhhlllhhhllEEHHHHHHHHHHHHHhHHHHLLLLEEEELHHHHLLL-------lEEL


DSSP  LEEEEEL--------lLHHHHHHHHHHHHhhlllleeEEELLLEELlllllLLLLEEEEE
Query DFATVAR--------rDIELIHEQLADAGetgwegftAIFTAPEEIaapgxPVKPRRFTA  130
ident |                       |   |
Sbjct DVDVLIThpdgrshrgIFSRLLDSLRQEG--------FLTDDLVSQ-eengQQQKYLGVC  224
DSSP  LEEEEEElllllllllHHHHHHHHHHHLL--------LEEEEEELL-llllLLLEEEEEE


DSSP  EEEEllEEEEEEEEEEEEL-LLLLlllleeeeelhhhhlllllleeeeellhHHHHhhhh
Query KLSYrgRAFATVPIEVSSV-EAGNadqfdtltsdalglvgvpaavavpcxtiPWQIaqkl  189
ident  |                |
Sbjct RLPG--PGRRHRRLDIIVVpYSEF---------------------------aCALL----  251
DSSP  LLLL--LLLLLEEEEEEELlHHHH---------------------------hHHHH----


DSSP  hhhhllllllllllllHHHHHHH---------------hhhhhllhHHHHhhHHHHHhhh
Query havtavleepkandraHDLVDLQ---------------llegllldADLXptRSACIaif  234
ident                                                 |
Sbjct ------------yftgSAHFNRSmralaktkgmslsehalstagpgRVLP--TPTEK---  294
DSSP  ------------hhhlLHHHHHHhhhhhhhlleeellllleeellllLLLL--LLLHH---


DSSP  hhhlllLLLLLLlllllhhhhhhhhhllllllllllllhhhhhhhhhhhhhhhhhllll
Query earaqhPWPPRVatlphwpliyagalegldhlelartvdaaaqavqrfvaridratkr  292
ident
Sbjct ---dvfRLLGLP-------------------------------------yrepaerdw  312
DSSP  ---hhhHHLLLL-------------------------------------lllhhhhll
```

No 484: Query=mol1A Sbjct=3upqA Z-score=5.2

back to top

```
DSSP  ------------------------------------------------------------
Query ------------------------------------------------------------    0
ident 
Sbjct hnlhiteklevlakaysvqgdkwralgyakainalksfhkpvtsyqeacsipgigkrmae   60
DSSP  llllhhhhhhhhhhhhhhlllhhhhhhhhhhhhhhllllllllhhhhhlllllhhhhh


DSSP  -------------------------------------------lllllllllhhhhhhh
Query -------------------------------------------aapysspptnlrslrdr   17
ident 
Sbjct kiieilesghlrkldhisesvpvlelfsniwgagtktaqmwyqqgfrsledirsqasltt  120
DSSP  hhhhhhhhlllhhhhhllllhhhhhhhhlllllllhhhhhhhhhlllllhhhhhhhllllh


DSSP  hhhhhhhllllhhhhHHHHHHHHHHHHHH-LLLLllLLLLEEEEHHHHHHHhhllllllL
Query ltqvaerqgvvfgrlQRHVAXIVVAQFAA-TLTDdtGAPLLLVKGGSSLELrrgipdsrT   76
ident                          |             |    |              |
Sbjct qqaiglkhysdflerMPREEATEIEQTVQkAAQAfnSGLLCVACGSYRRGK-------aT  173
DSSP  hhhhhhhlhhhhhllEEHHHHHHHHHHHHHhHHLLllLLLLEEEELHHHHLLL-------lE


DSSP  LLLEEEEEL--------lLHHHHHHHHHHHHhhllllleeEEELLLEELllllllLLLEEE
Query SKDFATVAR--------rDIELIHEQLADAGetgwegftAIFTAPEEIaapgxpVKPRRF  128
ident   |                       |   |
Sbjct CGDVDVLIThpdgrshrgIFSRLLDSLRQEG--------FLTDDLVKG------ETKYLG  219
DSSP  ELEEEEEEEllllllllLHHHHHHHHHHLL--------LEEEEEEEL------LLEEEE


DSSP  EEEEEEllEEEEEEEEEEEEL-LLLLllllleeeeelhhhhllllleeeeellhHHHHhh
Query TAKLSYrgRAFATVPIEVSSV-EAGNadqfdtltsdalglvgvpaavavpcxtiPWQIaq  187
ident    |                |
Sbjct VCRLPG--PGRRHRRLDIIVVpYSEF---------------------------aCALL--  248
DSSP  EELLLL--LLLLLEEEEEEEELlHHHH---------------------------hHHHH--


DSSP  hhhhhhllllllllllHHHHHHH--------------hhhhhllhHHHHhhHHHHHhh
Query klhavtavleepkandraHDLVDLQ--------------lleglllddADLXptRSACIai  233
ident                                                  |
Sbjct --------------yftgSAHFNRSmralaktkgmslsehalstavgRVLP--TPTEK--  290
DSSP  --------------hhhlLHHHHHHhhhhhhlleeellllleeelllLEEL--LLLHH--


DSSP  hhhhllllLLLLLlllllhhhhhhhhhllllllllllhhhhhhhhhhhhhhhhllll
Query fearaqhpWPPRVatlphwpliyagalegldhlelartvdaaaqavqrfvaridratkr  292
ident 
Sbjct -----dvfRLLGL------------------------------------pyrepaerdw  308
DSSP  -----hhhHHLLL------------------------------------llllhhhhll
```

No 485: Query=mol1A Sbjct=1xslE Z-score=5.2

back to top

```
DSSP  ------------------------------------------------------------
Query ------------------------------------------------------------    0
ident 
Sbjct nhnlhiteklevlakaysvqgdkwralgyakainalksfhkpvtsyqeacsipgigkrma   60
DSSP  lllhhhhhhhhhhhhhhhhlllhhhhhhhhhhhhhhhllllllllhhhhhlllllhhhh


DSSP  --------------------------------------------llllllllllhhhhhh
Query --------------------------------------------aapysspptnlrslrd   16
ident 
Sbjct ekiieilesghlrkldhisesvpvlelfsniwgagtktaqmwyqqgfrsledirsqaslt  120
DSSP  hhhhhhhhhlllhhhhhllllhhhhhhhhllllllhhhhhhhhhlllllhhhhhhhhllll


DSSP  hhhhhhhhllllhHHHHHHHHHHHHHHHHHHLL-LLLLLLLLEEEEHHHHHHHhhllllll
Query rltqvaerqgvvfGRLQRHVAXIVVAQFAATL-TDDTGAPLLLVKGGSSLELrrgipdsr   75
ident                           |             |    |
```

```
DSSP  LLLLEEEEEL--------lLHHHHHHHHHHHHhhlllleeEEELLLE-ELLLllLLLLLE
Query TSKDFATVAR--------rDIELIHEQLADAGetgwegftAIFTAPE-EIAApgXPVKPR  126
ident |  |                       |   |
Sbjct TCGDVDVLIThpdgrshrgIFSRLLDSLRQEG--------FLTDDLVsQEEN--GQQQKY  223
DSSP  EELLEEEEEElllllllllLHHHHHHHHHHLL--------LEEEEEEeEEEE--LLEEEE


DSSP  EEEEEEEEllEEEEEEEEEEEEL-LLLLlllleeeeelhhhhlllllleeeeellhHHHH
Query RFTAKLSYrgRAFATVPIEVSSV-EAGNadqfdtltsdalglvgvpaavavpcxtiPWQI  185
ident      |                |
Sbjct LGVCRLPG--PGRRHRRLDIIVVpYSEF---------------------------aCALL  254
DSSP  EEEELLLL--LLLLLEEEEEEELlHHHH---------------------------hHHHH


DSSP  hhhhhhhlllllllllllllHHHHHHH-------------------hhhhhllhhhhhhh
Query aqklhavtavleepkandraHDLVDLQ-------------------llegllldadlxpt  226
ident
Sbjct ----------------yftgSAHFNRSmralaktkgmslsehalstavvrnthgckvgpg  298
DSSP  ----------------hhhlLHHHHHHhhhhhhhlleeellleeelllllllllllllll


DSSP  hhhHHHHhhhhllllLLLLLLllllhhhhhhhhhlllllllllllhhhhhhhhhhhhhhh
Query rsaCIAIfearaqhpWPPRVAtlphwpliyagalegldhlelartvdaaaqavqrfvari  286
ident
Sbjct rvlPTPT---ekdvfRLLGLP------------------------------------yre  319
DSSP  leeLLLL---hhhhhHHHLLL------------------------------------lll


DSSP  hhllll
Query dratkr  292
ident
Sbjct paerdw  325
DSSP  hhhlll
```

No 486: Query=mol1A Sbjct=5deeY Z-score=5.2

back to top

```
DSSP  lllllllllllllllllllllllHLLLLHHHHHHHHHHHHHHHHHHLLL--LLLL---LL
Query aapysspptnlrslrdrltqvaeRQGVVFGRLQRHVAXIVVAQFAATLT--DDTG---AP   55
ident                                     |                      |
Sbjct -----------------------DKQWERFLVPYRQAVEELKVKLKGIRtlYEYEddhSP   37
DSSP  -----------------------LHHHHHHHHHHHHHHHHHHHHHHHHHhhHHHHlllLL


DSSP  LEEEEhhHHHHhhhlLLLLL------------------lLLLEEEEELL--LHHHHHHHH
Query LLLVKggSSLElrrgIPDSR------------------tSKDFATVARR--DIELIHEQL   95
ident    |              |                                ||    | |
Sbjct IEFVT--GRVK----PVASIlekarrksiplheietmqdIAGLRIMCQFvdDIQIVKEML   91
DSSP  LLEEE--EEEL----LHHHHhhhhhhllllhhhhhhlllLEEEEEEELLhhHHHHHHHHH


DSSP  HHHHhhlllleEEEELLLeellllllllLEEEEEEEEEL---lEEEEEEEEEEEELLll
Query ADAGetgwegfTAIFTAPeeiaapgxpvkPRRFTAKLSYR---gRAFATVPIEVSSVEag  152
ident                                                  | |
Sbjct FARK-------DFTVVDQ-----------SYHLVVLYPLQtvsgEKHVLVEIQIRTLA--  131
DSSP  HHLL-------LLEELLL-----------LEEEEEEEEEEllllEEEEEEEEEEEEHH--


DSSP  llllleeeeelhhhhlllllleeeeellhhhhhHHHHHHHHLLllllllllllhhhhhhh
Query nadqfdtltsdalglvgvpaavavpcxtipwqiAQKLHAVTAVleepkandrahdlvdlq  212
ident
Sbjct ------------------------------mnfWATIEHSLNY-----------------  144
DSSP  ------------------------------hhhHHHHHHHHHH-----------------


DSSP  hhhhhllhhhhhhhhhhhhhhhhhhhllllllLLLLLLLHhhhhhhhhlllllllllllh
Query llegllldadlxptrsaciaifearaqhpwppRVATLPHWpliyagalegldhlelartv  272
ident
Sbjct ------------------------------kySGNIPEKV------------klrlqras  162
DSSP  ------------------------------hlLLLLLHHH------------hhhhhhhh
```

```
ident
Sbjct eaasrldeemseirgevqea  182
DSSP  hhhhhhhhhhhhhhhhhlll
```

No 487: Query=mol1A Sbjct=2gafD Z-score=5.2

back to top

```
DSSP  ------------------------------------------------llllllLLLL--
Query ------------------------------------------------aapyssPPTN--   10
ident
Sbjct nitlkiietylgrvpsvneyhmlksqarniqkitvfnkdifvslvkknkkrffsDVNTsa   60
DSSP  lhhhhhhhhhhlllllhhhhhhhhhhhhhhhhhhhhllhhhhhhhhhhhhhhhllLLLLll

DSSP  -------------------------------------------------hhhhhhhhhhh
Query -------------------------------------------------lrslrdrltqv   21
ident
Sbjct seikdrilsyfskqtqtynigklftiielqsvlvttytdilgvltikaptsmeelardml  120
DSSP  hhhhhhhhhhllhhhhlllhhhhhhhhhhhhhhhhhhhhhhlllllllllllhhhhhhhh

DSSP  hhhllllhhhhhHHHHHHHHHHHHHHLLllllllLLEEEEHHHHHHHHHHLlLLLLlLLEE
Query aerqgvvfgrlqRHVAXIVVAQFAATLtddtgaPLLLVKGGSSLELRRGipDSRTsKDFA   81
ident                                        |  || |       |   |
Sbjct nsmnvavivsslVKNVNKLMEEYLRRH-----nKSCICYGSYSLYLINP--NIRY-GDID  172
DSSP  hhhlllllllllHHHHHHHHHHHHHHL-----lLLEEEELHHHHHLLLL--LLLL-LLLE

DSSP  EEELLlHHHHHHHHHHHHHHLlllleeeEELLlEELLLlllllLLEEEEEEeeelleeeEE
Query TVARRdIELIHEQLADAGETGwegftaIFTApEEIAApgxpvKPRRRFTAKlsyrgrafAT  141
ident              ||            |
Sbjct ILQTN-SRTFLIDLAFLIKFI--tgnnIILS-KIPYL-----RNYMVIKD-----endNH  218
DSSP  EEELL-HHHHHHHHHHHHHHH--llllEEEE-ELLLL-----LLEEEEEE-----lllLE

DSSP  EEEEEEEL---LLLLllLLEEEEelhhhhllllllleEEEELLHHHHHHHHHHHHHHLLLll
Query VPIEVSSV---EAGNadQFDTLTsdalglvgvpaavAVPCXTIPWQIAQKLHAVTAVLee  198
ident                                              |
Sbjct IIDSFNIRqdtMNVV--PKIFID-------------NIYIVDPTFQLLNMIKMFSQID--  261
DSSP  EEEEEEELLhhhHHHL--LEEEEL-------------LEEEELHHHHHHHHHHHHLLLHH--

DSSP  llLLLL----LHHHHHHHHHHHHHL--LHHH------------------------------
Query pkANDR----AHDLVDLQLLEGLL--LDAD------------------------------  222
ident     |
Sbjct -rLEDLskdpEKFNARMATMLEYVryTHGIvfdgkrnnmpmkciidennrivtvttkdyf  320
DSSP  -hHHHHhhllHHHHHHHHHHHHHHhhHHLLlllllllllllleeeellllleeeeellllll

DSSP  ---------------------------------hhhhhhhhhhhhhhHLLLL--------
Query ---------------------------------lxptrsaciaifeaRAQHP--------  241
ident
Sbjct sfkkclvyldenvlssdildlnadtscdfesvtnsvylihdnimytyFSNTIllsdkgkv  380
DSSP  llleeeeellhhhhhhhhhhlllllleelhhhllleeeellllleeeeLLLLLLeellllll

DSSP  -lLLLLlllllhhhhhhhhhlllllllllllhhhhhhhhhhHHHHH-------hHLLL--
Query -wPPRVatlphwpliyagalegldhlelartvdaaaqavqrFVARI-------dRATK--  291
ident
Sbjct heISAR---------------------------------glCAHILlyqmltsgEYKQcl  407
DSSP  lhHHHH---------------------------------hhHHHHHHhhhhhlllLLLHhh

DSSP  ---------------------------------------l
Query ---------------------------------------r  292
ident
Sbjct sdllnsmmnrdkipiyshterdkkpgrhgfiniekdiivf  447
DSSP  hhhhhllllllllllleellllllllllleeeeellllleeel
```

No 488: Query=mol1A Sbjct=3owgA Z-score=5.2

```
DSSP  ------------------------------------------------llllllLLLL--
Query ------------------------------------------------aapyssPPTN--   10
ident
Sbjct nitlkiietylgrvpsvneyhmlksqarniqkitvfnkdifvslvkknkkrffsDVNTsa   60
DSSP  lhhhhhhhhhhlllllhhhhhhhhhhhhhhhhhhhhllhhhhhhhhhhhhhhhllLLLLll


DSSP  -----------------------------------------------------------h
Query -----------------------------------------------------------l   11
ident
Sbjct seikdrilsyfskqtqtynigklftiielqsvlvttytdilgvltikapnvisskisynv  120
DSSP  lhhhhhhhhhllllllllhhhhhhhhhhhhhhhhhhhhhlhhhhllllllllllllllll


DSSP  hhhhhhhhhhhhhllllhhhhhHHHHHHHHHHHHHLLllllllLLEEEEHHHHHHHHHLl
Query rslrdrltqvaerqgvvfgrlqRHVAXIVVAQFAATLtddtgaPLLLVKGGSSLELRRGi   71
ident                                                  |  || |
Sbjct tsmeelardmlnsmnvavvsslVKNVNKLMEEYLRRH-----nKSCICYGSYSLYLINP-  174
DSSP  lhhhhhhhhhhhhhllllllllHHHHHHHHHHHHHHL-----lLLEEEELHHHHHLLLL-


DSSP  lLLLLlLLEEEEELLlHHHHHHHHHHHHHHLllleeeEELLlEELLLlllllLLEEEEEE
Query pDSRTsKDFATVARRdIELIHEQLADAGETGwegftaIFTApEEIAApgxpvKPRRFTAK  131
ident    |   |               ||            |
Sbjct -NIRY-GDIDILQTN-SRTFLIDLAFLIKFI--tgnnIILS-KIPYL-----RNYMVIKD  223
DSSP  -LLLL-LLLEEEELL-HHHHHHHHHHHHHHH--hlllEEEE-EELLL-----LLEEEEEE


DSSP  eeelleeeeEEEEEEEEL---LLLLllLLEEEEelhhhhlllllleEEEELLHHHHHHHH
Query lsyrgrafaTVPIEVSSV---EAGNadQFDTLTsdalglvgvpaavAVPCXTIPWQIAQK  188
ident                                                        |
Sbjct -----endnHIIDSFNIRqdtMNVV--PKIFID-------------NIYIVDPTFQLLNM  263
DSSP  -----llllEEEEEEELLhhhHHHL--LEEEEL-------------LEEEELHHHHHHHH


DSSP  HHHHHLLLllllLLLL----LHHHHHHHHHHHHL--LHHH--------------------
Query LHAVTAVLeepkANDR----AHDLVDLQLLEGLL--LDAD--------------------  222
ident               |
Sbjct IKMFSQID---rLEDLskdpEKFNARMATMLEYVryTHGIvfdgkrnnmpmkciidennr  320
DSSP  HHLLLLHH---hHHHHhhlhHHHHHHHHHHHHHHhhHHLLllllllllllllllleellll


DSSP  -------------------------------------------hhhhhhhhhhhhhhHLL
Query -------------------------------------------lxptrsaciaifeaRAQ  239
ident
Sbjct ivtvttkdyfsfkkclvyldenvlssdildlnadtscdfesvtnsvylihdnimytyFSN  380
DSSP  leeeellllllleeeeellhhhhhhhhhlllllllleelhhhhlleeeeelleeeeeLLL


DSSP  LL---------lLLLLlllllhhhhhhhhhlllllllllllhhhhhhhhhhHHHHH----
Query HP---------wPPRVatlphwpliyagalegldhlelartvdaaaqavqrFVARI----  286
ident
Sbjct TIllsdkgkvheISAR---------------------------------glCAHILlyqm  407
DSSP  LLeeeelleelhHHHH---------------------------------hhHHHHHhhhh


DSSP  ----hHLLL----------------------------------------l
Query ----dRATK----------------------------------------r  292
ident
Sbjct ltsgeYKQClsdllnsmmnrdkipiyshterdkkpgrhgfiniekdiivf  457
DSSP  hhlllLHHHhhhhhhllllllllllleellllllllllleeellllllll
```

No 489: Query=mol1A Sbjct=3er8C Z-score=5.2

back to top

```
DSSP  ------------------------------------------------llllllLLLL--
Query ------------------------------------------------aapyssPPTN--   10
ident
Sbjct nitlkiietylgrvpsvneyhmlksqarniqkitvfnkdifvslvkknkkrffsDVNTsa   60
DSSP  lhhhhhhhhhhlllllhhhhhhhhhhhhhhhhhhlllllhhhhhhhhhhhhhhllLLLLll


DSSP  -----------------------------------------------hhhhhhhhhhhhh
Query -----------------------------------------------lrslrdrltqvae   23
```

```
DSSP  lhhhhhhhhhllhhhhlllhhhhhhhhhhhhhhhhhhhhhhlllllllhhhhhhhhhhhh


DSSP  hllllhhhhhHHHHHHHHHHHHHHLLlllllllLLEEEEHHHHHHHHHLlLLLLlLLEEEE
Query rqgvvfgrlqRHVAXIVVAQFAATLtddtgaPLLLVKGGSSLELRRGipDSRTsKDFATV   83
ident                                      |  || |       |   |
Sbjct smnvavvsslVKNVNKLMEEYLRRH-----nKSCICYGSYSLYLINP--NIRY-GDIDIL  172
DSSP  hhllllllllhHHHHHHHHHHHHHHL-----lLLLEEELHHHHHLLLL--LLLL-LLLEEE


DSSP  ELLlHHHHHHHHHHHHHHLlllleeeEELLlEELLLlllllLLEEEEEEeeelleeeEEEE
Query ARRdIELIHEQLADAGETGwegftaIFTApEEIAApgxpvKPRRFTAKlsyrgrafATVP  143
ident            ||            |
Sbjct QTN-SRTFLIDLAFLIKFI--tgnnIILS-KIPYL-----RNYMVIKD-----endNHII  218
DSSP  ELL-HHHHHHHHHHHHHHH--hlllLEEE-ELLLL-----LLEEEEEL-----lllLEEE


DSSP  EeEEEL----LLLLllLLEEEEelhhhhllllllleEEEELLHHHHHHHHHHHHHLLLlll
Query IeVSSV----EAGNadQFDTLTsdalglvgvpaavAVPCXTIPWQIAQKLHAVTAVLeep  199
ident                                             |
Sbjct D-SFNIrqdtMNVV--PKIFID-------------NIYIVDPTFQLLNMIKMFSQID---  259
DSSP  E-EEELlhhhHHHL--LEEELL-------------LLEEELHHHHHHHHHLLLLLHH---


DSSP  lLLLL----LHHHHHHHHHHHHL--LHHH--------------------------------
Query kANDR----AHDLVDLQLLEGLL--LDAD--------------------------------  222
ident    |
Sbjct rLEDLskdpEKFNARMATMLEYVryTHGIvfdgkrnnmpmkciidennrivtvttkdyfs  319
DSSP  hHHHHhlllLHHHHHHHHHHHHHhhHLLLllllllllllllleeellllleeeeellllll


DSSP  --------------------------------hhhhhhhhhhhhhHLLLL---------
Query --------------------------------lxptrsaciaifeaRAQHP---------  241
ident
Sbjct fkkclvyldenvlssdildlnadtscdfesvtnsvylihdnimytyFSNTIllsdkgkvh  379
DSSP  lleeeeellhhhhhhhhhhlllllleelhhhllleeeeelleeeeeLLLLLeeeelleel


DSSP  lLLLLlllllhhhhhhhhhllllllllllhhhhhhhhhhHHHHH--------hHLLL--
Query wPPRVatlphwpliyagaleg1dhlelartvdaaaqavqrFVARI--------dRATK--  291
ident
Sbjct eISAR---------------------------------glCAHILlyqmltsgeYKQCls  406
DSSP  hHHHH---------------------------------hhHHHHHhhhhhhlllLHHHhh


DSSP  --------------------------------------l
Query --------------------------------------r  292
ident
Sbjct dllnsmmnrdkipiyshterdkkpgrhgfiniekdiivf  445
DSSP  hhhhhlllllllllleellllllllllleeeellllleeel
```

No 490: Query=mol1A Sbjct=5iioM Z-score=5.2

back to top

```
DSSP  ------------------------------------------------------------
Query ------------------------------------------------------------    0
ident
Sbjct tnhnlhiteklevlakaysvqgdkwralgyakainalksfhkpvtsyqeacsipgigkrm   60
DSSP  llllhhhhhhhhhhhhhhhhhllhhhhhhhhhhhhhhhhllllllhhhhhllllllhhh


DSSP  ---------------------------------------------llllllllllhhhhh
Query ---------------------------------------------aapysspptnlrslr   15
ident
Sbjct aekiieilesghlrkldhisesvpvlelfsniwgagtktaqmwyqqgfrsledirsqasl  120
DSSP  hhhhhhhhhhlllhhhhlllllhhhhhhhhlllllhhhhhhhhhlllllhhhhhhhlll


DSSP  hhhhhhhhhllllhHHHHHHHHHHHHHHHHHHHLL-LLLLLLLLEEEEHHHHHHHhhlllll
Query drltqvaerqgvvfGRLQRHVAXIVVAQFAATL-TDDTGAPLLLVKGGSSLELrrgipds   74
ident                            |             |    |
Sbjct ttqqaiglkhysdfLERMPREEATEIEQTVQKAaQAFNSGLLCVACGSYRRGK-------  173
DSSP  lhhhhhhhhlhhhhHLLEEHHHHHHHHHHHHHHHHhHLLLLLLLEEEELHHHHLLL-------
```

```
Query rTSKDFATVAR--------rDIELIHEQLADAGetgwegftAIFTAPE-EIAApgXPVKP  125
ident  |  |                       |   |
Sbjct aTCGDVDVLIThpdgrshrgIFSRLLDSLRQEG--------FLTDDLVsQEEN--GQQQK  223
DSSP  lEELLEEEEEEllllllllLHHHHHHHHHHHLL--------LEEEEEEeEEEE--LLEEE


DSSP  EEEEEEEEEllEEEEEEEEEEEEL-LLLLllllleeeeelhhhhllllllleeeeellhHHH
Query RRFTAKLSYrgRAFATVPIEVSSV-EAGNadqfdtltsdalglvgvpaavavpcxtiPWQ  184
ident       |                |
Sbjct YLGVCRLPG--PGRRHRRLDIIVVpYSEF---------------------------aCAL  254
DSSP  EEEEELLLL--LLLLLEEEEEEEELlHHHH---------------------------hHHH


DSSP  HhhhhhhhhlllllllllllHHHHHHH-------------------------hhhhhll
Query IaqklhavtavleepkandraHDLVDLQ-------------------------lleglll  219
ident
Sbjct L----------------yftgSAHFNRSmralaktkgmslsehalstavvrnthgckvgp  298
DSSP  H----------------hhhlLHHHHHHhhhhhhhlleeellllleeelllllllllll


DSSP  hHHHHhhHHHHHhhhhhhllllLLLLLlllllhhhhhhhhhllllllllllhhhhhhhh
Query dADLXptRSACIaifearaqhpWPPRVatlphwpliyagalegldhlelartvdaaaqav  279
ident    |
Sbjct gRVLP--TPTEK-------dvfRLLGL---------------------------------  316
DSSP  lLEEL--LLLHH-------hhhHHLLL---------------------------------


DSSP  hhhhhhhhhllll
Query qrfvaridratkr  292
ident
Sbjct ---pyrepaerdw  326
DSSP  ---llllhhhlll
```

No 491: Query=mol1A Sbjct=5iioI Z-score=5.2

back to top

```
DSSP  ------------------------------------------------------------
Query ------------------------------------------------------------    0
ident
Sbjct tnhnlhiteklevlakaysvqgdkwralgyakainalksfhkpvtsyqeacsipgigkrm   60
DSSP  llllhhhhhhhhhhhhhhhhlllhhhhhhhhhhhhhhhlllllllllhhhhhlllllhhh


DSSP  ---------------------------------------------lllllllllhhhhh
Query ---------------------------------------------aapysspptnlrslr   15
ident
Sbjct aekiieilesghlrkldhisesvpvlelfsniwgagtktaqmwyqqgfrsledirsqasl  120
DSSP  hhhhhhhhhhlllhhhhlllllhhhhhhhhllllllhhhhhhhhhlllllhhhhhhhlll


DSSP  hhhhhhhhhllllhHHHHHHHHHHHHHHHHHHLL-LLLLLLLLEEEEHHHHHHHhhlllll
Query drltqvaerqgvvfGRLQRHVAXIVVAQFAATL-TDDTGAPLLLVKGGSSLELrrgipds   74
ident                            |             |    |
Sbjct ttqqaiglkhysdfLERMPREEATEIEQTVQKAaQAFNSGLLCVACGSYRRGK-------  173
DSSP  lhhhhhhhhlhhhhHLLEEHHHHHHHHHHHHHHHhHHHLLLLEEEELHHHHLLL-------


DSSP  lLLLLLEEEEEL--------lLHHHHHHHHHHHHHhhllllleeEEELLLE-ELLLllLLLLL
Query rTSKDFATVAR--------rDIELIHEQLADAGetgwegftAIFTAPE-EIAApgXPVKP  125
ident  |  |                       |   |
Sbjct aTCGDVDVLIThpdgrshrgIFSRLLDSLRQEG--------FLTDDLVsQEEN--GQQQK  223
DSSP  lEELLEEEEEEllllllllHHHHHHHHHHHHLL--------LEEEEEEeEEEE--LLEEE


DSSP  EEEEEEEEEllEEEEEEEEEEEEL-LLLLllllleeeeelhhhhllllllleeeeellhHHH
Query RRFTAKLSYrgRAFATVPIEVSSV-EAGNadqfdtltsdalglvgvpaavavpcxtiPWQ  184
ident       |                |
Sbjct YLGVCRLPG--PGRRHRRLDIIVVpYSEF---------------------------aCAL  254
DSSP  EEEEELLLL--LLLLLEEEEEEEELlHHHH---------------------------hHHH


DSSP  HhhhhhhhhlllllllllllHHHHHHH-----------------------hhhhhllhh
Query IaqklhavtavleepkandraHDLVDLQ-----------------------lleglllda  221
ident
```

```
DSSP  hhhhhHHHHHhhhhhhllllLLLLLlllllhhhhhhhhhllllllllllhhhhhhhhhh
Query dlxptRSACIaifearaqhpWPPRVatlphwpliyagalegldhlelartvdaaaqavqr  281
ident 
Sbjct grvlpTPTEK-------dvfRLLGL-----------------------------------  316
DSSP  lleelLLLHH-------hhhHHLLL-----------------------------------


DSSP  hhhhhhhllll
Query fvaridratkr  292
ident 
Sbjct -pyrepaerdw  326
DSSP  -llllhhhlll
```

No 492: Query=mol1A Sbjct=5iijA Z-score=5.2

back to top

```
DSSP  ------------------------------------------------------------
Query ------------------------------------------------------------    0
ident 
Sbjct hnlhiteklevlakaysvqgdkwralgyakainalksfhkpvtsyqeacsipgigkrmae   60
DSSP  llllhhhhhhhhhhhhhlllhhhhhhhhhhhhhhhhlllllllllhhhhlllllhhhhh


DSSP  -------------------------------------------llllllllllhhhhhhh
Query -------------------------------------------aapysspptnlrslrdr   17
ident 
Sbjct kiieilesghlrkldhisesvpvlelfsniwgagtktaqmwyqqgfrsledirsqasltt  120
DSSP  hhhhhhhhlllhhhhhllllhhhhhhhhlllllllhhhhhhhhhlllllhhhhhhhlllh


DSSP  hhhhhhhllllhhHHHHHHHHHHHHHHHHHLL-LLLLLLLLEEEEHHHHHHHhhllllllL
Query ltqvaerqgvvfgRLQRHVAXIVVAQFAATL-TDDTGAPLLLVKGGSSLELrrgipdsrT   76
ident                          |             |    |              |
Sbjct qqaiglkhysdflERMPREEATEIEQTVQKAaQAFNSGLLCVACGSYRRGK-------aT  173
DSSP  hhhhhhhlhhhhhLLEEHHHHHHHHHHHHHHHHhHLLLLLLLEEEELHHHHLLL-------lE


DSSP  LLLEEEEEL--------lLHHHHHHHHHHHHHhhlllleeEEELLLEELlllllLLLLEEE
Query SKDFATVAR--------rDIELIHEQLADAGetgwegftAIFTAPEEIaapgxPVKPRRF  128
ident   |                       |   |
Sbjct CGDVDVLIThpdgrshrgIFSRLLDSLRQEG--------FLTDDLVSQ-eengQQQKYLG  224
DSSP  ELLEEEEEEllllllllLHHHHHHHHHHHLL--------LEEEEEELL-llllLLLEEEE


DSSP  EEEEEEllEEEEEEEEEEEEELLLLLLlllleeeeelhhhhllllllleeeeellhHHHHhhh
Query TAKLSYrgRAFATVPIEVSSVEAGNAdqfdtltsdalglvgvpaavavpcxtiPWQIaqk  188
ident    |                |
Sbjct VCRLPG--PGRRHRRLDIIVVPYSEF--------------------------aCALL---  253
DSSP  EELLLL--LLLLLEEEEEEEELLHHHH--------------------------hHHHH---


DSSP  hhhhhllllllllllllHHHHHHH---------------hhhhhllhHHHHhhHHHHHhh
Query lhavtavleepkandraHDLVDLQ---------------lleglldADLXptRSACIai  233
ident                                                  |
Sbjct -------------yftgSAHFNRSmralaktkgmslsehalstavpgRVLP--TPTEK--  296
DSSP  -------------hhhlLHHHHHHhhhhhhhlleeelllleeellllLLLL--LLLHH--


DSSP  hhhhlllLLLLLLlllllhhhhhhhhhlllllllllllhhhhhhhhhhhhhhhhhllll
Query fearaqhPWPPRVatlphwpliyagalegldhlelartvdaaaqavqrfvaridratkr  292
ident 
Sbjct ---dvfrLLGLPY--------------------------------------repaerdw  314
DSSP  ---hhhhHLLLLL--------------------------------------llhhhhll
```

No 493: Query=mol1A Sbjct=5cwrB Z-score=5.2

back to top

```
DSSP  ------------------------------------------------------------
```

```
Sbjct tnhnlhiteklevlakaysvqgdkwralgyakainalksfhkpvtsyqeacsipgigkrm   60
DSSP  lllhhhhhhhhhhhhhhhhhhllhhhhhhhhhhhhhhhhllllllhhhhhllllllhhh


DSSP  ---------------------------------------------llllllllllhhhhh
Query ---------------------------------------------aapysspptnlrslr   15
ident
Sbjct aekiieilesghlrkldhisesvpvlelfsniwgagtktaqmwyqqgfrsledirsqasl  120
DSSP  hhhhhhhhhhlllhhhhhllllhhhhhhhhllllllhhhhhhhhhlllllhhhhhhhlll


DSSP  hhhhhhhhhllllhHHHHHHHHHHHHHHHHHHLL-LLLLLLLLEEEEHHHHHHHhhlllll
Query drltqvaerqgvvfGRLQRHVAXIVVAQFAATL-TDDTGAPLLLVKGGSSLELrrgipds   74
ident                            |             |    |
Sbjct ttqqaiglkhysdfLERMPREEATEIEQTVQKAaQAFNSGLLCVACGSYRRGK-------  173
DSSP  lhhhhhhhhlhhhhHLLEEHHHHHHHHHHHHHHHhHLLLLLLEEEELHHHHLLL-------


DSSP  lLLLLEEEEEL--------lLHHHHHHHHHHHHhhllllleeEEELLLEELllllLLLLLE
Query rTSKDFATVAR--------rDIELIHEQLADAGetgwegftAIFTAPEEIaapgXPVKPR  126
ident  |  |                       |   |
Sbjct aTCGDVDVAIThpdgrshrgIFSRLLDSLRQEG--------FLTDDLVSQ----GQQQKY  221
DSSP  lEELLEEEEEEllllllllHHHHHHHHHHHHLL--------LEEEEEELL----LLLEEE


DSSP  EEEEEEEEllEEEEEEEEEEEEEL-LLLLlllleeeeelhhhhllllllleeeeellhHHHH
Query RFTAKLSYrgRAFATVPIEVSSV-EAGNadqfdtltsdalglvgvpaavavpcxtiPWQI  185
ident      |                |
Sbjct LGVCRLPG--PGRRHRRLDIIVVpYSEF---------------------------aCALL  252
DSSP  EEEELLLL--LLLLLEEEEEEEELlHHHH---------------------------hHHHH


DSSP  hhhhhhhhllllllllllllHHHHHHH-------------------------hhhhhllh
Query aqklhavtavleepkandraHDLVDLQ-------------------------llegllld  220
ident
Sbjct ----------------yftgSAHFNRSmralaktkgmslsehalstavvrnthgckvgpg  296
DSSP  ----------------hhhlLHHHHHHhhhhhhhlleeellllleeelleellllleeell


DSSP  HHHHhhHHHHHhhhhhhlllLLLLLLlllllhhhhhhhhhllllllllllhhhhhhhhh
Query ADLXptRSACIaifearaqhPWPPRVatlphwpliyagalegldhlelartvdaaaqavq  280
ident   |
Sbjct RVLP--TPTEK-----dvfrLLGLPY----------------------------------  315
DSSP  LEEL--LLLHH-----hhhhHLLLLL----------------------------------


DSSP  hhhhhhhhllll
Query rfvaridratkr  292
ident
Sbjct ----repaerdw  323
DSSP  ----llhhhhll
```

No 494: Query=mol1A Sbjct=5f2vU Z-score=5.2

back to top

```
DSSP  llllllllllhhhhhhhhhhhhhHLLLLHHHHHHHHHHHHHHHHHHLLL--LLLL---LL
Query aapysspptnlrslrdrltqvaeRQGVVFGRLQRHVAXIVVAQFAATLT--DDTG---AP   55
ident                                     |                      |
Sbjct -----------------------DKQWERFLVPYRQAVEELKVKLKGIRtlYEYEddhSP   37
DSSP  -----------------------LHHHHHHLHHHHHHHHHHHHHHHHHHHhhHHHHlllLL


DSSP  LEEEEhhHHHHhhhlLLLLL------------------lLLLEEEEELL--LHHHHHHHH
Query LLLVKggSSLElrrgIPDSR------------------tSKDFATVARR--DIELIHEQL   95
ident    |              |                                ||    | |
Sbjct IEFVT--GRVK----PVASIlekarrksiplheietmqdIAGLRIMCQFvdDIQIVKEML   91
DSSP  LLEEE--EEEL----LHHHHhhhhhhllllhhhhhhlllLEEEEEEELLhhHHHHHHHHH


DSSP  HHHHhhllllleEEEELLleellllLLLLLLEEEEEEEEEEL---lEEEEEEEEEEEELLll
Query ADAGetgwegfTAIFTApeeiaapGXPVKPRRFTAKLSYR---gRAFATVPIEVSSVEag  152
ident                                                  | |
Sbjct FARK-------DFTVVD-------ESGYRSYHLVVLYPLQtvsgEKHVLVEIQIRTLA--  135
DSSP  HHLL-------LLEEEL-------LLHHHEEEEEEEEEEEEelleEEEEEEEEEEEEEHH--
```

```
DSSP  lllllleeeeelhhhhllllllleeeeellhhhhhHHHHHHHHLllllllllllhhhhhhh
Query nadqfdtltsdalglvgvpaavavpcxtipwqiAQKLHAVTAvleepkandrahdlvdlq  212
ident
Sbjct ------------------------------mnfWATIEHSLN------------------  147
DSSP  ------------------------------hhhHHHHHHHHH------------------


DSSP  hhhhhllhhhhhhhhhhhhhhhhhhlllllllLLLLLLLHhhhhhhhhllllllllllh
Query lleglllldadlxptrsaciaifearaqhpwppRVATLPHWpliyagalegldhlelartv  272
ident
Sbjct -----------------------------ykySGNIPEKV------------klrlqras  166
DSSP  -----------------------------hhlLLLLLLHHH------------hhhhhhhh


DSSP  hhhhhhhhhhhhhhhhllll
Query daaaqavqrfvaridratkr  292
ident
Sbjct eaasrldeemseirgevqea  186
DSSP  hhhhhhhhhhhhhhhhhlll
```

No 495: Query=mol1A Sbjct=5deeU Z-score=5.2

back to top

```
DSSP  llllllllllhhhhhhhhhhhhhHLLLLHHHHHHHHHHHHHHHHHHHLL--LLLL--LLLL
Query aapysspptnlrslrdrltqvaeRQGVVFGRLQRHVAXIVVAQFAATL--TDDT--GAPL   56
ident                                          |
Sbjct -----------------------DKQWERFLVPYRQAVEELKVKLKGIrtLYEYedDHSP   37
DSSP  -----------------------LHHHHHHLHHHHHHHHHHHHHHHHHHhhHHHHhlLLLL


DSSP  -EEEEhhHHHHhhhlLLLLL------------------lLLLEEEEELL--LHHHHHHHH
Query -LLVKggSSLElrrgIPDSR------------------tSKDFATVARR--DIELIHEQL   95
ident    |              |                                 ||     | |
Sbjct iEFVT--GRVK----PVASIlekarrksiplheietmqdIAGLRIMCQFvdDIQIVKEML   91
DSSP  lLEEE--EEEL----LHHHHhhhhhhllllhhhhhhlllLEEEEEEEELLhhHHHHHHHHH


DSSP  HHHHhhlllleEEEELLleellllLLLLLLEEEEEEEEEL---lEEEEEEEEEEEEELLll
Query ADAGetgwegfTAIFTApeeiaapGXPVKPRRFTAKLSYR---gRAFATVPIEVSSVEag  152
ident                                                | |
Sbjct FARK-------DFTVVD-------ESGYRSYHLVVLYPLQtvsgEKHVLVEIQIRTLA--  135
DSSP  HHLL-------LLEEEL-------LLLLLEEEEEEEEEEEellеEEEEEEEEEEEEEHH--


DSSP  lllllleeeeelhhhhllllllleeeeellhhhhhHHHHHHHHLllllllllllhhhhhhh
Query nadqfdtltsdalglvgvpaavavpcxtipwqiAQKLHAVTAvleepkandrahdlvdlq  212
ident
Sbjct ------------------------------mnfWATIEHSLN------------------  147
DSSP  ------------------------------hhhHHHHHHHHH------------------


DSSP  hhhhhllhhhhhhhhhhhhhhhhhhlllllllLLLLLLLHhhhhhhhhllllllllllh
Query lleglllldadlxptrsaciaifearaqhpwppRVATLPHWpliyagalegldhlelartv  272
ident
Sbjct -----------------------------ykySGNIPEKV------------klrlqras  166
DSSP  -----------------------------hhlLLLLLLHHH------------hhhhhhhh


DSSP  hhhhhhhhhhhhhhhhllll
Query daaaqavqrfvaridratkr  292
ident
Sbjct eaasrldeemseirgevqea  186
DSSP  hhhhhhhhhhhhhhhhhlll
```

No 496: Query=mol1A Sbjct=5f2vX Z-score=5.2

back to top

```
DSSP  llllllllllhhhhhhhhhhhhhHLLLLHHHHHHHHHHHHHHHHHHHLL--LLLL--LLLL
Query aapysspptnlrslrdrltqvaeRQGVVFGRLQRHVAXIVVAQFAATL--TDDT--GAPL   56
ident                                          |
```

```
DSSP  -EEEEhhHHHHhhhlLLLLL------------------lLLLLEEEEELL--LHHHHHHHH
Query -LLVKggSSLElrrgIPDSR------------------tSKDFATVARR--DIELIHEQL   95
ident    |              |                                ||    | |
Sbjct iEFVT--GRVK----PVASIlekarrksiplheietmqdIAGLRIMCQFvdDIQIVKEML   91
DSSP  lLEEE--EEEL----LHHHHhhhhhhllllhhhhhhlllLEEEEEEEELLhhHHHHHHHHH


DSSP  HHHHhhllllеEEEELlleellllllllLLEEEEEEEEEL---lEEEEEEEEEEEELLll
Query ADAGetgwegfTAIFTapeeiaapgxpvKPRRFTAKLSYR---gRAFATVPIEVSSVEag  152
ident                                                  | |
Sbjct FARK-------DFTVV------------RSYHLVVLYPLQtvsgEKHVLVEIQIRTLA--  130
DSSP  HHLL-------LLEEL------------LEEEEEEEEEEEelleEEEEEEEEEEEEHH--


DSSP  llllleeeeelhhhhlllllleeeeellhhhhhHHHHHHHHLllllllllllhhhhhhh
Query nadqfdtltsdalglvgvpaavavpcxtipwqiAQKLHAVTAvleepkandrahdlvdlq  212
ident
Sbjct ------------------------------mnfWATIEHSLN------------------  142
DSSP  ------------------------------hhhHHHHHHHHH------------------


DSSP  hhhhhllhhhhhhhhhhhhhhhhhllllllLLLLLLLHhhhhhhhhlllllllllh
Query llegllldadlxptrsaciaifearaqhpwppRVATLPHWpliyagalegldhlelartv  272
ident
Sbjct -----------------------------ykySGNIPEKV------------klrlqras  161
DSSP  -----------------------------lllLLLLLLLH------------hhhhhhh


DSSP  hhhhhhhhhhhhhhhhllll
Query daaaqavqrfvaridratkr  292
ident
Sbjct eaasrldeemseirgevqea  181
DSSP  hhhhhhhhhhhhhhhhhlll
```

No 497: Query=mol1A Sbjct=3ouxA Z-score=5.2

back to top

```
DSSP  -------------------------------------------LLLL----llLLLLHHH
Query -------------------------------------------AAPY----ssPPTNLRS   13
ident
Sbjct raipeltkllndedqvvvnkaavmvhqlskkeasrhaimrspqMVSAivrtmqNTNDVET   60
DSSP  llhhhhhhhhllllllhhhhhhhhhhllllllllllllhhHHHHhhhhhHLLLHHH


DSSP  HHHHHHHHHHHLL---------llhHHHHHHHHHHH--------------------hhhh
Query LRDRLTQVAERQG---------vvfGRLQRHVAXIV--------------------vaqf   44
ident  |                       |
Sbjct ARCTAGTLHNLSHhregllaifksgGIPALVKMLGSpvdsvlfyaittlhnlllhqegak  120
DSSP  HHHHHHHHHHHLLlllhhhhhhhllHHHHHHHHHHLllhhhhhhhhhhhhhhhhlllhh


DSSP  hhlllllllllеEEEH--------HHHHHHHHLllllllllleeeeellllhhhhhhhh
Query aatltddtgapllLVKG--------GSSLELRRGipdsrtskdfatvarrdielihеqla   96
ident                              |
Sbjct mavrlagglqkmvALLNktnvkflaITTDCLQIL--------------------------  154
DSSP  hhhhhhlhhhhhhHHHHlllhhhhhHHHHHHHHH--------------------------


DSSP  hhhhhllllеeeeelllеellllllllllеeeeeeeeelleeeeeeeeeeeellllllll
Query dagetgwegftaiftapeeiaapgxpvkprrftaklsyrgrafatvpievssveagnadq  156
ident
Sbjct ------------------------------------------------------------  154
DSSP  ------------------------------------------------------------


DSSP  leeeeelhhhhllllllеeeeeLLHHHHHHHHHHHHHhlllllllllLLHHHHHHHHHH
Query fdtltsdalglvgvpaavavpcXTIPWQIAQKLHAVtavleepkandRAHDLVDLQLLEG  216
ident                          |                          |
Sbjct ---------aygnqeskliilaSGGPQALVNIMRTY----------tYEKLLWTTSRVLK  195
DSSP  ---------hlllhhhhhhhhhLLHHHHHHHHHHHL----------lLHHHHHHHHHHHH
```

```
ident  |            |                  |
Sbjct VLsvCSSNKPAIVEAG--------gmqalGLHLT---------------------dPSQR  226
DSSP  HHhlLLLHHHHHHHLL--------hhhhhHLLLL---------------------lLLHH


DSSP  HHHHHHHHHHHHHHLLLL------------------------------------------
Query AAQAVQRFVARIDRATKR------------------------------------------  292
ident   |           |
Sbjct LVQNCLWTLRNLSDAATKqegmegllgtlvqllgsddinvvtcaagilsnltcnnyknkm  286
DSSP  HHHHHHHHHHHHHHHLLLllllhhhhhhhhhhlllllhhhhhhhhhhhhhhllllhhhhh


DSSP  ------------------------------------------------------------
Query ------------------------------------------------------------  292
ident 
Sbjct mvcqvggiealvrtvlragdreditepaicalrhltsrhqeaemaqnavrlhyglpvvvk  346
DSSP  hhhhllhhhhhhhhhhhhlllhhhhhhhhhhhhhhlllllhhhhhhhhhhhllhhhhhhh


DSSP  ------------------------------------------------------------
Query ------------------------------------------------------------  292
ident 
Sbjct llhppshwplikatvglirnlalcpanhaplreqgaiprlvqllvrahqdtqregvrmee  406
DSSP  llllllhhhhhhhhhhhhhhhllhhhhhhhhhhllhhhhhhhhhhhhhhhhllllllhhh


DSSP  ------------------------------------------------------------
Query ------------------------------------------------------------  292
ident 
Sbjct ivegctgalhilardvhnrivirglntiplfvqllyspieniqrvaagvlcelaqdkeaa  466
DSSP  hhhhhhhhhhhhlllhhhhhhhhhhlllhhhhhhhllllhhhhhhhhhhhhhhhllhhhh


DSSP  -------------------------------------
Query -------------------------------------  292
ident 
Sbjct eaieaegatapltellhsrnegvatyaaavlfrmsed  503
DSSP  hhhhlllhhhhhhhlllllhhhhhhhhhhhhhhhlll
```

No 498: Query=mol1A Sbjct=5dedD Z-score=5.1

back to top

```
DSSP  llllllllllhhhhhhhhhhhhhHLLLLHHHHHHHHHHHHHHHHHHLLLLLLL-----LL
Query aapyssspptnlrslrdrltqvaeRQGVVFGRLQRHVAXIVVAQFAATLTDDTG-----AP   55
ident                                     |                      |
Sbjct -----------------------DKQWERFLVPYRQAVEELKVKLKGIRTLYEyeddhSP   37
DSSP  -----------------------LHHHHHHLHHHHHHHHHHHHHHHHHHHHHHhllllLL


DSSP  LEEEEhhHHHHhhhlLLLLL------------------lLLLEEEEELL--LHHHHHHHH
Query LLLVKggSSLElrrgIPDSR------------------tSKDFATVARR--DIELIHEQL   95
ident    |              |                                ||    | |
Sbjct IEFVT--GRVK----PVASIlekarrksiplheietmqdIAGLRIMCQFvdDIQIVKEML   91
DSSP  LLEEE--EEEL----LHHHHhhhhhhllllhhhhhhlllLEEEEEEELLllHHHHHHHHH


DSSP  HHHHhhlllleEEEELLLEEL---LLLLLllLLEEEEEEEEEL---lEEEEEEEEEEEEL
Query ADAGetgwegfTAIFTAPEEI---AAPGXpvKPRRFTAKLSYR---gRAFATVPIEVSSV  149
ident                                                     | |
Sbjct FARK-------DFTVVDQRDYiaeHKESG-yRSYHLVVLYPLQtvsgEKHVLVEIQIRTL  143
DSSP  HHLL-------LLEEEEEEELlllLLLLL-lLLEEEEEEEEEEllllEEEEEEEEEEEEH


DSSP  LllllllleeeeelhhhhllllllleeeeellhhhhhHHHHHHHHLLllllllllllhhhh
Query EagnadqfdtltsdalglvgvpaavavpcxtipwqiAQKLHAVTAVleepkandrahdlv  209
ident 
Sbjct A--------------------------------mnfWATIEHSLYK--------------  157
DSSP  H--------------------------------hhhHHHHHHHLLL--------------


DSSP  hhhhhhhllhhhhhhhhhhhhhhhhHHHLLlllllllllllhhhhhhhhhllllllllll
Query dlqlleglllldadlxptrsaciaifEARAQhpwpprvatlphwpliyagalegldhlela  269
ident 
Sbjct -------------------pekvklRLQRA------------------------------  168
```

```
DSSP  llhhhhhhhhhhhhhhhhllll
Query rtvdaaaqavqrfvaridratkr  292
ident
Sbjct ---seaasrldeemseirgvqea  188
DSSP  ---hhhhhhhhhhhhhhllllll
```

No 499: Query=mol1A Sbjct=2be3A Z-score=5.1

back to top

```
DSSP  llllllllllhhhhhhhhhhhhHHLLLLHHHHHHHHHHHHHHHHHHHLL--LLLL--LLLL
Query aapysspptnlrslrdrltqvaERQGVVFGRLQRHVAXIVVAQFAATL--TDDT--GAPL   56
ident                                     |
Sbjct ----------------------XTLEWEEFLDPYIQAVGELKIKLRGIrkQYRKqnKHSP   38
DSSP  ----------------------LLHHHHHHHHHHHHHHHHHHHHHHHHHHhhHHHHllLLLL


DSSP  -EEEEhhHHHHhhhlLLLLL-------------------lLLLEEEEELL--LHHHHHHH
Query -LLVKggSSLElrrgIPDSR-------------------tSKDFATVARR--DIELIHEQ   94
ident    |              |                                 |
Sbjct iEFVT--GRVK----PIESIkekxarrgityatlehdlqdIAGLRVXVQFvdDVKEVVDI   92
DSSP  eEEEE--EEEL----LHHHHhhhhhhhllllllhhhhlllLEEEEEEEELLhhHHHHHHHH


DSSP  HHHHHhhllleEEEELLLEEL--LLLLlllLLEEEEEEEEEL---lEEEEEEEEEEEEL
Query LADAGetgwegfTAIFTAPEEI--AAPGxpvKPRRFTAKLSYR---gRAFATVPIEVSSV  149
ident |                                                     |
Sbjct LHKRQ-------DXRIIQERDYitHRKAsgyRSYHVVVEYTVDtingAKTILAEIQIRTL  145
DSSP  HHLLL-------LEEEEEEEELllLLLLlllLLEEEEEEEEELllllLEEEEEEEEEEEH


DSSP  LllllllleeeeelhhhhllllllleeeeellhhhhhHHHHHHHHLlllllllllllhhhh
Query EagnadqfdtltsdalglvgvpaavavpcxtipwqiAQKLHAVTAvleepkandrahdlv  209
ident
Sbjct A-------------------------------xnfWATIEHSLN---------------  158
DSSP  H-------------------------------hhhHHHHHHHHH---------------


DSSP  hhhhhhhhllhhhhhhhhhhhhhhhhhhhhlllllllllLLLLLLHhhhhhhhhlllllllll
Query dlqllegllldadlxptrsaciaifearaqhpwpprVATLPHWpliyagalegldhlela  269
ident
Sbjct --------------------------------ykyqGDFPDEI------kkrleitaria  180
DSSP  --------------------------------hhhlLLLLHHH------hhhhhhhhhhh


DSSP  llhhhhhhhhhhhhhhhhllll
Query rtvdaaaqavqrfvaridratkr  292
ident
Sbjct hqldeexgeirddiqeaqalfdp  203
DSSP  hhhhhhhhllhhhhhhhhhhhlll
```

No 500: Query=mol1A Sbjct=5dedF Z-score=5.1

back to top

```
DSSP  llllllllllhhhhhhhhhhhhHLLLLHHHHHHHHHHHHHHHHHHHHLLL--LLLL---LL
Query aapysspptnlrslrdrltqvaeRQGVVFGRLQRHVAXIVVAQFAATLT--DDTG---AP   55
ident                                     |                      |
Sbjct -----------------------DKQWERFLVPYRQAVEELKVKLKGIRtlYEYEddhSP   37
DSSP  -----------------------LHHHHHHHHHHHHHHHHHHHHHHHHHHhlLLLLlllLL


DSSP  LEEEEhhHHHHhhhlLLLLL------------------lLLLEEEEELL--LHHHHHHH
Query LLLVKggSSLElrrgIPDSR------------------tSKDFATVARR--DIELIHEQL   95
ident    |              |                                ||     | |
Sbjct IEFVT--GRVK----PVASIlekarrksiplheietmqdIAGLRIMCQFvdDIQIVKEML   91
DSSP  LLEEE--EEEL----LHHHHhhhhhhllllhhhhhhlllLEEEEEEEELLhhHHHHHHHHH


DSSP  HHHHhhlllleEEEELLLEEL---LLLLLllLLEEEEEEEEEL---lEEEEEEEEEEEEL
Query ADAGetgwegfTAIFTAPEEI---AAPGXpvKPRRFTAKLSYR---gRAFATVPIEVSSV  149
```

```
DSSP  HHLL-------LLEEEEEEELlllLLLLL-lLLEEEEEEEELLllllLLEEEEEEEEEEH


DSSP  LllllllleeeeelhhhhlllllleeeeellhhhhhHHHHHHHHLlllllllllllhhhh
Query EagnadqfdtltsdalglvgvpaavavpcxtipwqiAQKLHAVTAvleepkandrahdlv  209
ident
Sbjct A--------------------------------mnfWATIEHSLN---------------  156
DSSP  H--------------------------------hhhHHHHHHHHL---------------


DSSP  hhhhhhhhllhhhhhhhhhhhhhhhhhhhlllllllLLLLllhhhhhhhhhhllllllll
Query dlqllegllldadlxptrsaciaifearaqhpwpprVATLphwpliyagalegldhlela  269
ident
Sbjct -----------------------------------yKSGN------------ipekvklr  169
DSSP  -----------------------------------lLLLL------------llllllll


DSSP  llhhhhhhhhhhhhhhhhhllll
Query rtvdaaaqavqrfvaridratkr  292
ident
Sbjct lqraseaasrldeemseirgeva  192
DSSP  lhhhhhhhhhhhhhhhhhhhlll
```

No 501: Query=mol1A Sbjct=4atbA Z-score=5.1

back to top

```
DSSP  -------------llllllllllhhhhhhhhhhhhhhlLLLHHHHHHHHHHHHHHHHHHL
Query -------------aapysspptnlrslrdrltqvaerqGVVFGRLQRHVAXIVVAQFAAT   47
ident
Sbjct shipfdfylcemafprvkpapdetsfseallkrnqdlaPNSAEQASILSLVTKINNVIDN   60
DSSP  lllllhhhlhhhlllllllllllhhhhhhhhhhhhhhlLLHHHHHHHHHHHHHHHHHHHH


DSSP  LL--LLLL-lLLEEEEHHhhhhhhhlllLLLL-----lLLEEEEEL--LLHHHHHHHHHH
Query LT--DDTG-aPLLLVKGGsslelrrgipDSRT-----sKDFATVAR--RDIELIHEQLAD   97
ident |     |       |                        |           |
Sbjct LIvaPGTFevQIEEVRQV------gsykKGTMttghnvADLVVILKilPTLEAVAALGNK  114
DSSP  HHhlLLLLllLEEEEEEE------hhhhHLLLllllleEEEEEEELllLLHHHHHHHHHH


DSSP  HHHH-LLLL--eeEEELLLEellllllllllEEEEEEEeelleEEEEEEEEEEELLLLll
Query AGET-GWEG--ftAIFTAPEeiaapgxpvkpRRFTAKLsyrgrAFATVPIEVSSVEAGna  154
ident   |                |             |           ||| |    |
Sbjct VVESlRAQDpsevLTMLTNE-----------TGFEISS-----SDATVKILITTVPPN--  156
DSSP  HHHHhHHHLllllLEEEELL-----------LEEEEEL-----LLLEEEEEEEELHHH--


DSSP  llleeeeelhhhhlllllleeeeellhhhhhhHHHHHHhllllllllLLLLhhhhhhhhh
Query dqfdtltsdalglvgvpaavavpcxtipwqiaQKLHAVtavleepkaNDRAhdlvdlqll  214
ident
Sbjct ----------lrkldpelhldikvlqsalaaiRHARWF--------eENAS---------  189
DSSP  ----------hllllllllllhhhhhhhhhhhHHHHHH--------hHHLL---------


DSSP  hhhllhhhhhhhhhhhhhhhhhhhlllllllllllllhhHHHHHHHLLL-LLLL------
Query egllldadlxptrsaciaifearaqhpwpprvatlphwpLIYAGALEGL-DHLE------  267
ident                                              |  |   |
Sbjct ---------------------------------------QSTVKVLIRLlKDLRirfpgf  210
DSSP  ---------------------------------------LHHHHHHHHHhHHHHhhlhhh


DSSP  -----------lllLHHH-------------------------hhhhhHHHH-------H
Query -----------larTVDA-------------------------aaqavQRFV-------A  284
ident                |
Sbjct epltpwildllghyAVMNnptrqplalnvayrrclqilaaglflpgsvGITDpcesgnfR  270
DSSP  llllhhhhhhhhhhHHHLllllllllhhhhhhhhhhhhhlllllllllLLLLlllllllL


DSSP  HHHHLLLL----------------------------------------------------
Query RIDRATKR----------------------------------------------------  292
ident      |
Sbjct VHTVMTLEqqdmvcytaqtlvrilshggfrkilgqegdasylaseistwdgvivtpseka  330
DSSP  HHHHLLHHhhhhhhhhhhhhhhhhhhlllhhhhhlllllhhhhllleeelleeellllll
```

```
Query ----  292
ident
Sbjct yekp  334
DSSP  llll
```

No 502: Query=mol1A Sbjct=4atbC Z-score=5.1

back to top

```
DSSP  ------------lllllllllhhhhhhhhhhhhhlLLLHHHHHHHHHHHHHHHHHHLL
Query ------------aapysspptnlrslrdrltqvaerqGVVFGRLQRHVAXIVVAQFAATL   48
ident                                                            |
Sbjct hipfdfylcemafprvkpapdetsfseallkrnqdlaPNSAEQASILSLVTKINNVIDNL   60
DSSP  lllllhhhlllllllllllllhhhhhhhhhhhhhlLLHHHHHHHHHHHHHHHHHHHHHHH


DSSP  LlLLLL-LLEEEEEHHhhhhhhhlllLLLL-----lLLEEEEEL--LLHHHHHHHHHHHHH
Query TdDTGA-PLLLVKGGsslelrrgipDSRT-----sKDFATVAR--RDIELIHEQLADAGE  100
ident     |      |                        |           |          |
Sbjct IvAPGTvQIEEVRQV------gsykKGTMttghnvADLVVILKilPTLEAVAALGNKVVE  114
DSSP  HhLLLLlLEEEEEEE------hhhhHLLLllllleEEEEEEELllLLHHHHHHHHHHHHH


DSSP  H-LLLL--eeEEELLLEellllllllllEEEEEEEeelleEEEEEEEEEEEELLLLLllll
Query T-GWEG--ftAIFTAPEeiaapgxpvkpRRFTAKLsyrgrAFATVPIEVSSVEAGNadqf  157
ident                 |             |           ||| |    |
Sbjct SlRAQDpsevLTMLTNE-----------TGFEISS-----SDATVKILITTVPPNL----  154
DSSP  HhHHHLllllLEEEELL-----------LLEEEEL-----LLLEEEEEEEELHHHH----


DSSP  eeeeelhhhhllllllleeeeellhhhhhhHHHHHHhlllllllLLLLhhhhhhhhhhhh
Query dtltsdalglvgvpaavavpcxtipwqiaQKLHAVtavleepkaNDRAhdlvdlqllegl  217
ident
Sbjct ---------rkldpelhldikvlqsalaaIRHARW-------feENAS------------  186
DSSP  ---------lllllllllhhhhhhhhhhHHHHHH-------hhHHLL------------


DSSP  llhhhhhhhhhhhhhhhhhhlllllllllllllhhHHHHHHHLLL-LLLL---------
Query lldadlxptrsaciaifearaqhpwpprvatlphwpLIYAGALEGL-DHLE---------  267
ident                                           |  |   |
Sbjct ------------------------------------QSTVKVLIRLlKDLRirfpgfepl  210
DSSP  ------------------------------------LHHHHHHHHhHHHHhhlhhhlll


DSSP  --------lllLHHH-------------------------hhhhhHHHH------HHHHH
Query --------larTVDA-------------------------aaqavQRFV------ARIDR  288
ident             |                                           |
Sbjct tpwildllghyAVMNnptrqplalnvayrrclqilaaglflpgsvGITDpcesgnFRVHT  270
DSSP  lhhhhhhhhhHHLLllllllllhhhhhhhhhhhhlllllllllLLLLllllllLLHHH


DSSP  LLLL--------------------------------------------------------
Query ATKR--------------------------------------------------------  292
ident
Sbjct VMTLeqqdmvcytaqtlvrilshggfrkilgqegdasylaseistwdgvivtpsekayek  330
DSSP  HLLHhhhhhhhhhhhhhhhhhhlllhhhhhlllllllhhhhllleeelleeellllllll


DSSP  -
Query -  292
ident
Sbjct p  331
DSSP  l
```

No 503: Query=mol1A Sbjct=5a2vA Z-score=5.1

back to top

```
DSSP  ----------------------------LLLLL-----------lllLHHHHhhHHHH-
Query ----------------------------AAPYS-----------spptNLRSLrdRLTQ-   20
ident                                                 |       |
Sbjct ktftevqterleqadrsvlikcpsklneKKLLQylsshgkidnyfffeNRGIH--ALIEf   58
```

```
DSSP  ------------------------------------------------------------
Query ------------------------------------------------------------   20
ident
Sbjct sekssvaslqavtgipkaaehhvvpyksrlftftlknpgsqaaeerpvkispqshipvne  118
DSSP  lllhhhhhhhhlllllllllllllllleeeeeellhhhllllllllllllllllllhhh


DSSP  ------------------hhhhllLLHHHHHHHHHHHHHHHHHHLLLlllllLLEEEEHH
Query ------------------vaerqgVVFGRLQRHVAXIVVAQFAATLTddtgaPLLLVKGG   62
ident                                |  |   |  ||         |   ||
Sbjct lipklchadsissqmyillneyqlTEENIKLRYLACSLVRDFARAYF-----PDSTVKPF  173
DSSP  hhhhhhlllhhhhhhhhhhhhhllLHHHHHHHHHHHHHHHHHHHLLL-----LLLEEEEE


DSSP  hhhhhhhlllLLLL---lLLEEEEEL---------------------lLHHHHHHHHHHH
Query sslelrrgipDSRT---sKDFATVAR---------------------rDIELIHEQLADA   98
ident                    |                                |     |
Sbjct -----gssvnTFGKlgcdVDMFLDFHdimkkgpfemeyqmkrlpserlATQKILSIIGDC  228
DSSP  -----hhhllLLLLllllEEEEEELLlllllllleeeelllllllhhhHHHHHHHHHHHH


DSSP  HHHLLlLEEEEELLLEELlllllllLLEEEEEEEEEEllееEEEEEEEEEellllllllle
Query GETGWeGFTAIFTAPEEIaapgxpvKPRRFTAKLSYrgraFATVPIEVSsveagnadqfd  158
ident                                                ||
Sbjct LDNFG-PGYSSVQKILNA-------RCPLVKFSHQP---tGFQCDLSVS-----------  266
DSSP  HHHLL-LLLLLLEEELLL-------LLLEEEEEELL---lLEEEEEEEL-----------


DSSP  eeeelhhhhlllllleeeeellhhhhhhHHHHHHhllllllllllllhhhhhhhhhhhhl
Query tltsdalglvgvpaavavpcxtipwqiaQKLHAVtavleepkandrahdlvdlqllegll  218
ident
Sbjct ------------------------nsiaIRCSEL--------------------------  276
DSSP  ------------------------lhhhHHHHHH--------------------------


DSSP  lhhhhhhhhhhhhhhhhhhhlllllllllllllhHHHHHHHHLLL-LLLL----------
Query ldadlxptrsaciaifearaqhpwpprvatlphwPLIYAGALEGL-DHLE----------  267
ident                                         ||
Sbjct ----------------------------lyiygcLDPRVRALVFSlRCWArvhgltnsvp  308
DSSP  ----------------------------hhhhhhHLHHHHHHHHHhHHHHhhllllllll


DSSP  ---------lllLHHHHHHH----------------------------------------
Query ---------larTVDAAAQA----------------------------------------  278
ident
Sbjct gtwitnfsltmmIMFFLQKRsppiiptldqlkeladekdkhviggydcsfvsdlskikpt  368
DSSP  lllllhhhhhhhHHHHHHLLlllllllhhhhhhlllhhhlleelleellllllhhhllll


DSSP  --------------------------hhhhhhhhHHLLLL--------------------
Query --------------------------vqrfvariDRATKR--------------------  292
ident
Sbjct kntetldellcdffqyfgnfdfrknslnlrkgkeVNKPESsplyiwnpfeqdlnisknvn  428
DSSP  lllllhhhhhhhhhhhhhllllleeeellllleeEELLLLllllllllllllllllllll


DSSP  ------------------------------------------
Query ------------------------------------------  292
ident
Sbjct qpqlekfvamaresawilqkedktqqminkepwglaavlipf  470
DSSP  hhhhhhhhhhhhhhhhhhhhllhhhhhhhlllllhhhhllll
```

No 504: Query=mol1A Sbjct=2m2uA Z-score=5.1

back to top

```
DSSP  lllllllllhhhhhhhhhhhhhllllhhhhhHHHHHHHHHHHHHHHLL--------llll
Query aapysspptnlrslrdrltqvaerqgvvfgrlqRHVAXIVVAQFAATL--------tddt   52
ident
Sbjct -------------------------------mlTLIQGKKIVNHLRSRlafeyngqliki   29
DSSP  -------------------------------leEHHHHHHHHHHHHHLeeeeelleeeee


DSSP  llLLEEEEHHHHHHhhhllllLLLLLLEEEEEL--------lLHHHHHhhhHHHHhhlll
```

```
Sbjct lsKNIVAVGSLRRE-------EKMLNDVDLLIIvpekkllkhVLPNIR---IKGL-----   74
DSSP  llLLEEEELHHHHL-------LLEELLEEEEEEellhhhhhlLLLEEE---ELLL-----


DSSP  leeeEELLlEELLLlllllLLEEEEEEEEelleeEEEEEEEEEEELLLLLlllleeeeelh
Query gftaIFTApEEIAApgxpvKPRRFTAKLSyrgraFATVPIEVSSVEAGNadqfdtltsda  164
ident      |                              |         |
Sbjct ----SFSV-KVCGE-----RKCVLFIEWE-----KKTYQLDLFTALAEE-----------  108
DSSP  ----EEEE-EEELL-----LEEEEEEEEL-----LEEEEEEEEEEEHHH-----------


DSSP  hhhllllleeeeellhHHHHhhhhhHHHLLllllllllllhhhhhhhhhhhhllhhHHH
Query lglvgvpaavavpcxtiPWQIaqklhAVTAVleepkandrahdlvdlqlleglllldaDLX  224
ident                                                           |
Sbjct ---------------kpYAIFhftgpVSYLI-riraalkkknyklnqyglfknqtlvPLK  152
DSSP  ---------------hhHHHHhhhllHHHHH-hhhhhhhlleeeelleeeelleeeLLL


DSSP  HhhHHHHhhhhhhllllLLLLllllllhhhhhhhhhlllllllllllhhhhhhhhhhhh
Query PtrSACIaifearaqhpWPPRvatlphwpliyagalegldhlelartvdaaaqavqrfva  284
ident
Sbjct I--TTEK-----elikeLGFT---------------------------------------  166
DSSP  L--LLHH-----hhhhhHLLL---------------------------------------


DSSP  hhhhllll
Query ridratkr  292
ident
Sbjct yripkkrl  174
DSSP  lllhhhll
```

No 505: Query=mol1A Sbjct=5cwrA Z-score=5.1

back to top

```
DSSP  ------------------------------------------------------------
Query ------------------------------------------------------------    0
ident
Sbjct tnhnlhiteklevlakaysvqgdkwralgyakainalksfhkpvtsyqeacsipgigkrm   60
DSSP  llllhhhhhhhhhhhhhhhhllhhhhhhhhhhhhhhhhlllllllhhhhhllllllhhh


DSSP  ---------------------------------------------lllllllllhhhhh
Query ---------------------------------------------aapysspptnlrslr   15
ident
Sbjct aekiieilesghlrkldhisesvpvlelfsniwgagtktaqmwyqqgfrsledirsqasl  120
DSSP  hhhhhhhhhlllhhhhhllllhhhhhhhhlllllhhhhhhhhhlllllhhhhhhhlll


DSSP  hhhhhhhhllllhHHHHHHHHHHHHHHHHHHL-LLLLLLLLLEEEEHHHHHHHHhhlllll
Query drltqvaerqgvvfGRLQRHVAXIVVAQFAAT-LTDDTGAPLLLVKGGSSLELrrgipds   74
ident                            |             |    |
Sbjct ttqqaiglkhysdfLERMPREEATEIEQTVQKaAQAFNSGLLCVACGSYRRGK-------  173
DSSP  lhhhhhhhlhhhhHLLEEHHHHHHHHHHHHHHhHHLLLLLLEEEELHHHHLLL-------


DSSP  lLLLLEEEEEL--------lLHHHHHHHHHHHHhhlllleeEEELLLEELllllllLLLE
Query rTSKDFATVAR--------rDIELIHEQLADAGetgwegftAIFTAPEEIaapgxpVKPR  126
ident  |  |                       |   |
Sbjct aTCGDVDVAIThpdgrshrgIFSRLLDSLRQEG--------FLTDDLVSQ--eengQQKY  223
DSSP  lEELLEEEEEEllllllllHHHHHHHHHHHHLL--------LEEEEEELL--llllLLEE


DSSP  EEEEEEEEllEEEEEEEEEEEEEL-LLLLlllleeeeelhhhhllllllleeeeellhHHHH
Query RFTAKLSYrgRAFATVPIEVSSV-EAGNadqfdtltsdalglvgvpaavavpcxtiPWQI  185
ident      |                |
Sbjct LGVCRLPG--PGRRHRRLDIIVVpYSEF---------------------------aCALL  254
DSSP  EEEELLLL--LLLLLEEEEEEEELlHHHH---------------------------hHHHH


DSSP  hhhhhhhlllllllllllHHHHHHHhhhhhllhhhhhhhhhHHHH-------------
Query aqklhavtavleepkandraHDLVDLQlleglllldadlxptrsACIA------------  232
ident
Sbjct ----------------yftgSAHFNRS--mralaktkgmslseHALStavvrnthgckvg  296
DSSP  ----------------hhhlLHHHHHHH--hhhhhhhlleeellLLEEelleellllleee
```

```
DSSP  ---hHHHH--lllllLLLLLLlllhhhhhhhhhlllllllllllhhhhhhhhhhhhhhhh
Query ---iFEAR--aqhpwPPRVATlphwpliyagalegldhlelartvdaaaqavqrfvarid  287
ident 
Sbjct pgrvLPTPtekdvfrLLGLPY------------------------------------rep  320
DSSP  llleELLLlhhhhhhHLLLLL------------------------------------llh


DSSP  hllll
Query ratkr  292
ident 
Sbjct aerdw  325
DSSP  hhlll
```

No 506: Query=mol1A Sbjct=3hx0K Z-score=5.1

back to top

```
DSSP  ------------------------------------------------------------
Query ------------------------------------------------------------    0
ident 
Sbjct atnhnlhiteklevlakaysvqgdkwraagyakainalksfhkpvtsyqeacsipgigkr   60
DSSP  llllllhhhhhhhhhhhhhhhhllhhhhhhhhhhhhhhhlllllllllhhhhllllllllhh


DSSP  ----------------------------------------------llllllllllhhhh
Query ----------------------------------------------aapysspptnlrsl   14
ident 
Sbjct maekiieilesghlrkldhisesvpvlelfsniwgagtktaqmwyqqgfrsledirsqas  120
DSSP  hhhhhhhhhlllllhhhhllllhhhhhhhhhlllllhhhhhhhhllllllhhhhhllll


DSSP  hhhhhhhhhhllllhHHHHHHHHHHHHHHHHHHHLL-LLLLLLLLEEEEHHHHHHHhhllll
Query rdrltqvaerqgvvfGRLQRHVAXIVVAQFAATL-TDDTGAPLLLVKGGSSLELrrgipd   73
ident                             |             |    |
Sbjct lttqqaiglkhysdfLERMPREEATEIEQTVQKAaQAFNSGLLCVACGSYRRGK------  174
DSSP  llhhhhhhhhlhhhhHLLEEHHHHHHHHHHHHHHHhHLLLLLLEEEEEHHHHLLL------


DSSP  llLLLLEEEEEL--------lLHHHHHHHHHHHHHhhlllleeEEELLLE-ELLLllLLLL
Query srTSKDFATVAR--------rDIELIHEQLADAGetgwegftAIFTAPE-EIAApgXPVK  124
ident   |  |                     |   |
Sbjct -aTCGDVDVLIThpdgrshrgIFSRLLDSLRQEG--------FLTDDLVsQEEN--GQQQ  223
DSSP  -lEELEEEEEEElllllllLHHHHHHHHHHHLL--------LEEEEEEeEEEE--LLEE


DSSP  LEEEEEEEEEllEEEEEEEEEEEEL-LLLLlllleeeeelhhhhllllllleeeeellhHH
Query PRRFTAKLSYrgRAFATVPIEVSSV-EAGNadqfdtltsdalglvgvpaavavpcxtiPW  183
ident        |                |
Sbjct KYLGVCRLPG--PGRRHRRLDIIVVpYSEF---------------------------aCA  254
DSSP  EEEEEELLLL--LLLLEEEEEEEELlHHHH---------------------------hHH


DSSP  HHhhhhhhhhllllllllllllHHHHHHH------------------------hhhhhl
Query QIaqklhavtavleepkandraHDLVDLQ------------------------llegll  218
ident 
Sbjct LL----------------yftgSAAFNASmralaktkgmslsehalstavvrnthgakvg  298
DSSP  HH----------------hhhlLHHHHHHhhhhhhllleeellllleeelllllllllll


DSSP  lhHHHHhhHHHHHhhhhhhllllLLLLLLllllhhhhhhhhhlllllllllllhhhhhhh
Query ldADLXptRSACIaifearaqhpWPPRVAtlphwpliyagalegldhlelartvdaaaqa  278
ident     |
Sbjct pgRVLP--TPTEK-------dvfRLLGLP-------------------------------  318
DSSP  llLEEL--LLLHH-------hhhHHHLLL-------------------------------


DSSP  hhhhhhhhhhllll
Query vqrfvaridratkr  292
ident 
Sbjct -----yrepaerdw  327
DSSP  -----lllhhhlll
```

back to top

```
DSSP  ------------------------------------------------------------
Query ------------------------------------------------------------   0
ident 
Sbjct tnhnlhiteklevlakaysvqgdkwralgyakainalksfhkpvtsyqeacsipgigkrm  60
DSSP  llllhhhhhhhhhhhhhhhhlllhhhhhhhhhhhhhhhlllllllhhhhhhlllllhhh


DSSP  ---------------------------------------------llllllllllhhhhh
Query ---------------------------------------------aapysspptnlrslr  15
ident 
Sbjct aekiieilesghlrkldhisesvpvlelfsniwgagtktaqmwyqqgfrsledirsqasl 120
DSSP  hhhhhhhhlllllhhhhlllllhhhhhhhhllllllhhhhhhhhhlllllhhhhhhllll


DSSP  hhhhhhhhhllllhHHHHHHHHHHHHHHHHHHHLL-LLLLLLLLEEEEHHHHHHHHhhlllll
Query drltqvaerqgvvfGRLQRHVAXIVVAQFAATL-TDDTGAPLLLVKGGSSLELrrgipds  74
ident                            |             |    |
Sbjct ttqqaiglkhysdfLERMPREEATEIEQTVQKAaQAFNSGLLCVACGSYRRGK------- 173
DSSP  lhhhhhhhhlhhhhHLLEEHHHHHHHHHHHHHHHHhHHHLLLLEEEELHHHHLLL-------


DSSP  lLLLLEEEEEL--------lLHHHHHHHHHHHHHhhllllleeeEELLL-EELLLLLlLLLL
Query rTSKDFATVAR--------rDIELIHEQLADAGetgwegftaIFTAP-EEIAAPGxPVKP 125
ident  |  |                       |   |           |         |
Sbjct aTCGDVDVLIThpdgrshrgIFSRLLDSLRQEG---------FLTDDlVSQEENG-QQQK 223
DSSP  lEELLEEEEEEllllllllHHHHHHHHHHHHLL---------LEEEEeEEEEEEL-LEEE


DSSP  EEEEEEEEEllEEEEEEEEEEEEL-LLLLlllleeeeelhhhhllllllleeeeellhHHH
Query RRFTAKLSYrgRAFATVPIEVSSV-EAGNadqfdtltsdalglvgvpaavavpcxtiPWQ 184
ident       |                 |
Sbjct YLGVCRLPG--PGRRHRRLDIIVVpYSEF---------------------------aCAL 254
DSSP  EEEEELLLL--LLLLLEEEEEEEELlHHHH---------------------------hHHH


DSSP  HhhhhhhhhlllllllllllHHHHHHH----------------------hhhhhllhh
Query IaqklhavtavleepkandraHDLVDLQ----------------------lleglllda 221
ident 
Sbjct L----------------yftgSAHFNRSmralaktkgmslsehalstavvrnthgckvgp 298
DSSP  H----------------hhhlLHHHHHHhhhhhhhlleeellllleeellllllllllll


DSSP  hhhhhHHHHHhhhhhhllllLLLLLlllllhhhhhhhhhllllllllllhhhhhhhhh
Query dlxptRSACIaifearaqhpWPPRVatlphwpliyagalegldhlelartvdaaaqavqr 281
ident 
Sbjct grvlpTPTEK-------dvfRLLGL----------------------------------- 316
DSSP  lleelLLLHH-------hhhHHLLL-----------------------------------


DSSP  hhhhhhhllll
Query fvaridratkr  292
ident 
Sbjct -pyrepaerdw  326
DSSP  -llllhhhlll
```

No 508: Query=mol1A Sbjct=3er8D Z-score=5.1

back to top

```
DSSP  ------------------------------------------------llllllLLLL--
Query ------------------------------------------------aapyssPPTN--  10
ident 
Sbjct nitlkiietylgrvpsvneyhmlksqarniqkitvfnkdifvslvkknkkrffsDVNTsa  60
DSSP  lhhhhhhhhhhllllhhhhhhhhhhhhhhhhhhlllllhhhhhhhhhhhhllLLLLll


DSSP  -----------------------------------------------hhhhhhhhhhhh
Query -----------------------------------------------lrslrdrltqvae  23
ident 
Sbjct seikdrilsyfskqtqtynigklftiielqsvlvttytdilgvltinvtsmeelardmln 120
DSSP  lhhhhhhhhhllhhhhlllhhhhhhhhhhhhhhhhhhhhhlhhhllllhhhhhhhhhhh
```

```
Query rqgvvfgrlqRHVAXIVVAQFAATLtddtgaPLLLVKGGSSLELRRGipDSRTsKDFATV   83
ident                                      |  || |       |   |
Sbjct smnvavvsslVKNVNKLMEEYLRRH-----nKSCICYGSYSLYLINP--NIRY-GDIDIL  172
DSSP  hhlllllllhHHHHHHHHHHHHHHL-----lLLLEEELHHHHHLLLL--LLLL-LLLEEE


DSSP  ELLlHHHHHHHHHHHHHHLlllleeeEELLlEELLLlllllLLEEEEEEeeelleeeEEEE
Query ARRdIELIHEQLADAGETGwegftaIFTApEEIAApgxpvKPRRFTAKlsyrgrafATVP  143
ident            ||            |
Sbjct QTN-SRTFLIDLAFLIKFI--tgnnIILS-KIPYL-----RNYMVIKD-----endNHII  218
DSSP  ELL-HHHHHHHHHHHHHHH--hlllLEEE-ELLLL-----LLEEEEEL-----lllLEEE


DSSP  EeEEELllLLLL--LLEEEEelhhhhllllleEEEEELLHHHHHHHHHHHHLLLllllL
Query IeVSSVeaGNAD--QFDTLTsdalglvgvpaavAVPCXTIPWQIAQKLHAVTAVLeepkA  201
ident                                           |
Sbjct D-SFNIrqDTMNvvPKIFID-------------NIYIVDPTFQLLNMIKMFSQID---rL  261
DSSP  E-EEELlhHHHHhlLEEELL-------------LLEEELHHHHHHHHHLLLLHH---hH


DSSP  LLL----LHHHHHHHHHHHL--LHHH---------------------------------
Query NDR----AHDLVDLQLLEGLL--LDAD---------------------------------  222
ident  |
Sbjct EDLskdpEKFNARMATMLEYVryTHGIvfdgkrnnmpmkciidennrivtvttkdyfsfk  321
DSSP  HHHhlllLHHHHHHHHHHHHHhhHLLLlllllllllllleeellllleeeeelllllll


DSSP  ------------------------------hhhhhhhhhhhhHHLLLL---------lL
Query ------------------------------lxptrsaciaifeARAQHP---------wP  243
ident
Sbjct kclvyldenvlssdildlnadtscdfesvtnsvylihdnimytYFSNTIllsdkgkvheI  381
DSSP  eeeeellhhhhhhhhhhlllllleelhhhllleeeeelleeeeELLLLLeeeelleelhH


DSSP  LLLlllllhhhhhhhhhlllllllllllhhhhhhhhhHHHHH-------hHLLL-----
Query PRVatlphwpliyagalegldhlelartvdaaaqavqrFVARI-------dRATK-----  291
ident
Sbjct SAR---------------------------------glCAHILlyqmltsgEYKQclsdl  408
DSSP  HHH---------------------------------hhHHHHHhhhhhllLLHHhhhhh


DSSP  -----------------------------------l
Query -----------------------------------r  292
ident
Sbjct lnsmmnrdkipiyshterdkkpgrhgfiniekdiivf  445
DSSP  hhhllllllllleellllllllleeeellllleeel
```

No 509: Query=mol1A Sbjct=3hx0P Z-score=5.1

[back to top](#)

```
DSSP  ------------------------------------------------------------
Query ------------------------------------------------------------    0
ident
Sbjct atnhnlhiteklevlakaysvqgdkwraagyakainalksfhkpvtsyqeacsipgigkr   60
DSSP  lllllhhhhhhhhhhhhhhhllllhhhhhhhhhhhhhhhlllllllllhhhhhhllllhh


DSSP  ----------------------------------------------llllllllllhhhh
Query ----------------------------------------------aapysspptnlrsl   14
ident
Sbjct maekiieilesghlrkldhisesvpvlelfsniwgagtktaqmwyqqgfrsledirsqas  120
DSSP  hhhhhhhhhllllhhhhllllhhhhhhhhlllllhhhhhhhhhllllhhhhhhhll


DSSP  hhhhhhhhhhllllhHHHHHHHHHHHHHHHHHHHLL-LLLLLLLLEEEEHHHHHHHhhllll
Query rdrltqvaerqgvvfGRLQRHVAXIVVAQFAATL-TDDTGAPLLLVKGGSSLELrrgipd   73
ident                             |             |    |
Sbjct lttqqaiglkhysdfLERMPREEATEIEQTVQKAaQAFNSGLLCVACGSYRRGK------  174
DSSP  llhhhhhhhhlhhhhHLLEEHHHHHHHHHHHHHHHHhHLLLLLLLEEEELHHHHLLL------


DSSP  llLLLLLEEEEEL--------lLHHHHHHHHHHHHhhllllleeEEELLLE-ELLLllLLLL
Query srTSKDFATVAR--------rDIELIHEQLADAGetgwegftAIFTAPE-EIAApgXPVK  124
ident   |  |                       |   |
```

```
DSSP  LEEEEEEEEEllEEEEEEEEEEEEL-LLLLlllleeeeelhhhhllllleeeeellhHH
Query PRRFTAKLSYrgRAFATVPIEVSSV-EAGNadqfdtltsdalglvgvpaavavpcxtiPW  183
ident        |                |
Sbjct KYLGVCRLPG--PGRRHRRLDIIVVpYSEF---------------------------aCA  254
DSSP  EEEEEELLLL--LLLLLEEEEEEEELlLLLH---------------------------hHH


DSSP  HHhhhhhhhhllllllllllHHHHHHH-------------------------hhhhhl
Query QIaqklhavtavleepkandraHDLVDLQ-------------------------llegll  218
ident
Sbjct LL----------------yftgSAAFNASmralaktkgmslsehalstavvrnthgakvg  298
DSSP  HH----------------hhhlLHHHHHHhhhhhhhhleellllleellllllllllll


DSSP  lhHHHHhhHHHHHhhhhhhllllLLLLLlllllhhhhhhhhhlllllllllllhhhhhhh
Query ldADLXptRSACIaifearaqhpWPPRVatlphwpliyagalegldhlelartvdaaaqa  278
ident     |
Sbjct pgRVLP--TPTEK------dvfrLLGLP--------------------------------  318
DSSP  llLLLL--LLLHH------hhhhHHLLL--------------------------------


DSSP  hhhhhhhhhhllll
Query vqrfvaridratkr  292
ident
Sbjct -----yrepaerdw  327
DSSP  -----lllllllll
```

No 510: Query=mol1A Sbjct=3owgB Z-score=5.1

back to top

```
DSSP  ------------------------------------------------llllllLLLL--
Query ------------------------------------------------aapyssPPTN--   10
ident
Sbjct nitlkiietylgrvpsvneyhmlksqarniqkitvfnkdifvslvkknkkrffsDVNTsa   60
DSSP  lhhhhhhhhhhllllllhhhhhhhhhhhhhhhhhhhhhllhhhhhhhhhhhhhhhllLLLLll


DSSP  -----------------------------------------------------------h
Query -----------------------------------------------------------l   11
ident
Sbjct seikdrilsyfskqtqtynigklftiielqsvlvttytdilgvltikapnvisskisynv  120
DSSP  lhhhhhhhhhlllllllllhhhhhhhhhhhhhhhhhhhhhhlhhhhlllllllllllllll


DSSP  hhhhhhhhhhhhhllllhhhhhHHHHHHHHHHHHHHLLllllllLLEEEEHHHHHHHHHLl
Query rslrdrltqvaerqgvvfgrlqRHVAXIVVAQFAATLtddtgaPLLLVKGGSSLELRRGi   71
ident                                                  |  || |
Sbjct tsmeelardmlnsmnvavvsslVKNVNKLMEEYLRRH-----nKSCICYGSYSLYLINP-  174
DSSP  lhhhhhhhhhhhhhllllllllHHHHHHHHHHHHHHHL-----lLLEEEEHHHHHHLLLL-


DSSP  lLLLLlLLEEEEEELLlHHHHHHHHHHHHHHHLllleeeEELLlEELLLlllllLLEEEEEE
Query pDSRTsKDFATVARRdIELIHEQLADAGETGwegftaIFTApEEIAApgxpvKPRRFTAK  131
ident    |   |               ||            |
Sbjct -NIRY-GDIDILQTN-SRTFLIDLAFLIKFI--tgnnIILS-KIPYL-----RNYMVIKD  223
DSSP  -LLLL-LLLEEEEELL-HHHHHHHHHHHHHHHH--hlllEEEE-LLLLL-----LLEEEEEE


DSSP  eeelleeeeEEEEEEEEL---LLLLllLLEEEEelhhhhllllleEEEELLHHHHHHHH
Query lsyrgrafaTVPIEVSSV---EAGNadQFDTLTsdalglvgvpaavAVPCXTIPWQIAQK  188
ident                                                        |
Sbjct -----endnHIIDSFNIRqdtMNVV--PKIFID-------------NIYIVDPTFQLLNM  263
DSSP  -----llllEEEEEEEELLhhhHHHL--LEEEEL-------------LEEEELHHHHHHHH


DSSP  HHHHHLLLllllLLLL----LHHHHHHHHHHHHHL--LHHH--------------------
Query LHAVTAVLeepkANDR----AHDLVDLQLLEGLL--LDAD--------------------  222
ident               |
Sbjct IKMFSQID---rLEDLskdpEKFNARMATMLEYVryTHGIvfdgkrnnmpmkciidennr  320
DSSP  HHHLLLHH---hHHHHhhlhHHHHHHHHHHHHHHHHhhHHLLlllllllllllleeeellll
```

```
ident
Sbjct ivtvttkdyfsfkkclvyldenvlssdildlnadtscdfesvtnsvylihdnimytyFSN  380
DSSP  eeeeellllllleeeeellllhhhhhhhllllllllleelhhhhlleeeeelleeeeeLLL


DSSP  LL---------lLLLLlllllhhhhhhhhhlllllllllllhhhhhhhhhhHHHHH----
Query HP---------wPPRVatlphwpliyagalegldhlelartvdaaaqavqrFVARI----  286
ident
Sbjct TIllsdkgkvheISAR---------------------------------glCAHILlyqm  407
DSSP  LLeeeelleelhHHHH---------------------------------hhHHHHHhhhh


DSSP  ----hHLLL----------------------------------------l
Query ----dRATK----------------------------------------r  292
ident
Sbjct ltsgeYKQClsdllnsmmnrdkipiyshterdkkpgrhgfiniekdiivf  457
DSSP  hhlllLHHHhhhhhhllllllllllleellllllllllleeellllleell
```

No 511: Query=mol1A Sbjct=4qzgA Z-score=5.1

[back to top]

```
DSSP  ------------------------------------------------------------
Query ------------------------------------------------------------    0
ident
Sbjct kkisqyacqrrttlnnynqlftdaldilaendelrenegsclafmrassvlkslpfpits   60
DSSP  llllllhhhlllllllllhhhhhhhhhhhhhhhhlllhhhhhhhhhhhhhhhlllllll


DSSP  ------------------------------------------------------------
Query ------------------------------------------------------------    0
ident
Sbjct mkdtegipclgdkvksiiegiiedgesseakavlnderyksfklftsvfgvglktaekwf  120
DSSP  hhhhlllllllhhhhhhhhhhhhhlllhhhhhhhhlhhhhhhhhhhlllllhhhhhhhh


DSSP  ---lllllllllhhhhhhhhhhhhhhhllllhHHHHHHHHHHHHHHHHHL-LLLLlLLLL
Query ---aapysspptnlrslrdrltqvaerqgvvfGRLQRHVAXIVVAQFAAT-LTDDtGAPL   56
ident                                            |               |
Sbjct rmgfrtlskiqsdkslrftqmqkagflyyedlVSCVNRPEAEAVSMLVKEaVVTFlPDAL  180
DSSP  hlllllhhhhhhlllllllhhhhhhhhlhhhhHLLEEHHHHHHHHHHHHHhHHHHlLLLE


DSSP  EEEEHHHHHHHhhllllllLLLLEEEEEL----------lLHHHHHHHHHHHHhhlllle
Query LLVKGGSSLELrrgipdsrTSKDFATVAR----------rDIELIHEQLADAGetgwegf  106
ident     ||             |  |                             |
Sbjct VTMTGGFRRGK-------mTGHDVDFLITspeatedeeqqLLHKVTDFWKQQG-------  226
DSSP  EEELHHHHLLL-------lEELLEEEEEElllllhhhhhhHHHHHHHHHHHLL-------


DSSP  eeEELLLEELLLllLLLLLEEEEEEEEEL---lEEEEEEEEEEEEL-LLLLlllleeeee
Query taIFTAPEEIAApgXPVKPRRFTAKLSYR---gRAFATVPIEVSSV-EAGNadqfdtlts  162
ident                         ||
Sbjct --LLLYCDILES--DHAQKCFLILKLDHGrvhgKGWKAIRVDLVMCpYDRR---------  273
DSSP  --LEEEEEEELL--LLLLEEEEEEEEEHHhlllLEEEEEEEEEEELlHHHH---------


DSSP  lhhhhlllllleeeeellhHHHHhhhhhhhhllllllllllllHHHHHHH----------
Query dalglvgvpaavavpcxtiPWQIaqklhavtavleepkandraHDLVDLQ----------  212
ident
Sbjct ------------------aFALL----------------gwtgSRQFERDlrryatherk  299
DSSP  ------------------hHHHH----------------hhhlLHHHHHHhhhhhhhhhl


DSSP  ----hhhhhllhhhhhhhHHHHHhhhhhhllllLLLLLLllllhhhhhhhhhllllllll
Query ----llegllldadlxptRSACIaifearaqhpWPPRVAtlphwpliyagalegldhlel  268
ident
Sbjct mmldnhalydrtkrvfleAESEE-------eifAHLGLD---------------------  331
DSSP  eeellllleeellllleeelLLLHH-------hhhHHHLLL---------------------


DSSP  lllhhhhhhhhhhhhhhhhhllll
Query artvdaaaqavqrfvaridratkr  292
ident
Sbjct ---------------yiepwerna  340
```

No 512: Query=mol1A Sbjct=4k4gM Z-score=5.1

back to top

```
DSSP  ------------------------------------------------------------
Query ------------------------------------------------------------    0
ident 
Sbjct atnhnlhiteklevlakaysvqgdkwralgyakainalksfhkpvtsyqeacsipgigkr   60
DSSP  lllllhhhhhhhhhhhhhhhhhllhhhhhhhhhhhhhhhhllllllhhhhhlllllllhh


DSSP  ----------------------------------------------llllllllllhhhh
Query ----------------------------------------------aapyssppptnlrsl   14
ident 
Sbjct maekiieilesghlrkldhisesvpvlelfsniwgagtktaqmwyqqgfrsledirsqas  120
DSSP  hhhhhhhhhhhlllhhhhlllllhhhhhhhhllllllhhhhhhhhhlllllhhhhhhhll


DSSP  hhhhhhhhhhllllhHHHHHHHHHHHHHHHHHLL-LLLLLLLEEEEHHHHHHHHhhllll
Query rdrltqvaerqgvvfGRLQRHVAXIVVAQFAATL-TDDTGAPLLLVKGGSSLELrrgipd   73
ident                             |             |    |
Sbjct lttqqaiglkhysdfLERMPREEATEIEQTVQKAaQAFNSGLLCVACGSYRRGK------  174
DSSP  llhhhhhhhhlhhhhLLLEEHHHHHHHHHHHHHHhHLLLLLLEEEELHHHHLLL------


DSSP  llLLLLEEEEEL--------lLHHHHHHHHHHHHhhlllleeEEELLLE-ELLLllLLLL
Query srTSKDFATVAR--------rDIELIHEQLADAGetgwegftAIFTAPE-EIAApgXPVK  124
ident   |  |                       |   |
Sbjct -aTCGDVDVLIThpdgrshrgIFSRLLDSLRQEG--------FLTDDLVsQEEN--GQQQ  223
DSSP  -lEELLEEEEEEllllllllHHHHHHHHHHHHLL--------LEEEEEEeEEEE--LLEE


DSSP  LEEEEEEEEEllEEEEEEEEEEEEL-LLLLlllleeeeelhhhhlllllleeeeellhHH
Query PRRFTAKLSYrgRAFATVPIEVSSV-EAGNadqfdtltsdalglvgvpaavavpcxtiPW  183
ident        |                |
Sbjct KYLGVCRLPG--PGRRHRRLDIIVVpYSEF---------------------------aCA  254
DSSP  EEEEEELLLL--LLLLLEEEEEEELlHHHH---------------------------hHH


DSSP  HHhhhhhhhlllllllllllllHHHHHHHH--------------------hhhhllhhhh
Query QIaqklhavtavleepkandraHDLVDLQL--------------------legllldadl  223
ident 
Sbjct LL----------------yftgSAHFNRSMralaktkgmslsehalstavvrhgckvgpg  298
DSSP  HH----------------hhhlLHHHHHHhhhhhhlleeellllleeellllllllllll


DSSP  hhhHHHHhhhhhhhllllLLLLLlllllhhhhhhhhhllllllLLLLlhhhhhhhhhhhh
Query xptRSACiaifearaqhpWPPRVatlphwpliyagalegldhlELARtvdaaaqavqrfv  283
ident                                             |
Sbjct rvlPTPT------ekdvfRLLGL-----------pyrepaerdWLEH-------------  328
DSSP  leeLLLL------hhhhhHHLLL-----------llllhhhllLLLL-------------


DSSP  hhhhhllll
Query aridratkr  292
ident 
Sbjct ---------  328
DSSP  ---------
```

No 513: Query=mol1A Sbjct=4k4hM Z-score=5.1

back to top

```
DSSP  ------------------------------------------------------------
Query ------------------------------------------------------------    0
ident 
Sbjct atnhnlhiteklevlakaysvqgdkwralgyakainalksfhkpvtsyqeacsipgigkr   60
DSSP  lllllhhhhhhhhhhhhhhhhhllhhhhhhhhhhhhhhllllllllhhhhhlllllllhh


DSSP  ----------------------------------------------llllllllllhhhh
Query ----------------------------------------------aapyssppptnlrsl   14
```

```
DSSP  hhhhhhhhhhhlllhhhhlllllhhhhhhhhlllllhhhhhhhhlllllhhhhhhhll


DSSP  hhhhhhhhhhllllhHHHHHHHHHHHHHHHHHHHLL-LLLLLLLLEEEEHHHHHHHHhhllll
Query rdrltqvaerqgvvfGRLQRHVAXIVVAQFAATL-TDDTGAPLLLVKGGSSLELrrgipd   73
ident                             |             |    |
Sbjct lttqqaiglkhysdfLERMPREEATEIEQTVQKAaQAFNSGLLCVACGSYRRGK------  174
DSSP  llhhhhhhhhlhhhhHLLEEHHHHHHHHHHHHHHHHhHLLLLLLEEEELHHHHLLL------


DSSP  llLLLLLEEEEEL--------lLHHHHHHHHHHHHHhhllllleeeEELLL--EELLLllLLL
Query srTSKDFATVAR--------rDIELIHEQLADAGetgwegftaIFTAP--EEIAApgXPV  123
ident   |  |                       |   |           |
Sbjct -aTCGDVDVLIThpdgrshrgIFSRLLDSLRQEG---------FLTDDlvSQEEN--GQQ  222
DSSP  -lEELLEEEEEEllllllllHHHHHHHHHHHHLL---------LEEEEeeEEEEE--LLE


DSSP  LLEEEEEEEEEllEEEEEEEEEEEEL-LLLLllleeeeelhhhhllllllleeeeellhH
Query KPRRFTAKLSYrgRAFATVPIEVSSV-EAGNadqfdtltsdalglvgvpaavavpcxtiP  182
ident         |                |
Sbjct QKYLGVCRLPG--PGRRHRRLDIIVVpYSEF---------------------------aC  253
DSSP  EEEEEEEEELLL--LLLLEEEEEEEEELlHHHH---------------------------hH


DSSP  HHHhhhhhhhhlllllllllllHHHHHHH---------------------hhhhhllhH
Query WQIaqklhavtavleepkandraHDLVDLQ---------------------llegllldA  221
ident
Sbjct ALL----------------yftgSAHFNRSmralaktkgmslsehalstavvrckvgpgR  297
DSSP  HHH----------------hhhlLHHHHHHhhhhhhhlleeellleeellllllllllL


DSSP  HHHhhHHHHHhhhhhhllllLLLLLlllllhhhhhhhhhlllllllllllhhhhhhhhhh
Query DLXptRSACIaifearaqhpWPPRVatlphwpliyagalegldhlelartvdaaaqavqr  281
ident  |
Sbjct VLP--TPTEK-------dvfRLLGL----------------------------------p  314
DSSP  EEL--LLLHH-------hhhHHHLL----------------------------------l


DSSP  hhhhhhhllll
Query fvaridratkr  292
ident
Sbjct yrepaerdwle  325
DSSP  lllhhhlllll
```

No 514: Query=mol1A Sbjct=5iioE Z-score=5.1

back to top

```
DSSP  ------------------------------------------------------------
Query ------------------------------------------------------------    0
ident
Sbjct nhnlhiteklevlakaysvqgdkwralgyakainalksfhkpvtsyqeacsipgigkrma   60
DSSP  lllhhhhhhhhhhhhhhhhlllhhhhhhhhhhhhhhhlllllllhhhhhllllllhhhh


DSSP  --------------------------------------------llllllllllhhhhhh
Query --------------------------------------------aapysspptnlrslrd   16
ident
Sbjct ekiieilesghlrkldhisesvpvlelfsniwgagtktaqmwyqqgfrsledirsqaslt  120
DSSP  hhhhhhhhhlllhhhhllllllhhhhhhhhlllllhhhhhhhhhlllllhhhhhhhhllll


DSSP  hhhhhhhhllllhHHHHHHHHHHHHHHHHHHHLL-LLLLLLLLEEEEHHHHHHHHhhllllll
Query rltqvaerqgvvfGRLQRHVAXIVVAQFAATL-TDDTGAPLLLVKGGSSLELrrgipdsr   75
ident                           |             |    |
Sbjct tqqaiglkhysdfLERMPREEATEIEQTVQKAaQAFNSGLLCVACGSYRRGK-------a  173
DSSP  hhhhhhhhlhhhhLLLEEHHHHHHHHHHHHHHHHhHLLLLLLEEEELHHHHLLL-------l


DSSP  LLLLLEEEEEL--------lLHHHHHHHHHHHHHhhllllleeeEELLL--EELLLllLLLLL
Query TSKDFATVAR--------rDIELIHEQLADAGetgwegftaIFTAP--EEIAApgXPVKP  125
ident |  |                       |   |           |
Sbjct TCGDVDVLIThpdgrshrgIFSRLLDSLRQEG---------FLTDDlvSQEEN--GQQQK  222
DSSP  EELLEEEEEEllllllllHHHHHHHHHHHHLL---------LEEEEeeEEEEE--LLEEE
```

```
Query RRFTAKLSYrgRAFATVPIEVSSV-EAGNadqfdtltsdalglvgvpaavavpcxtiPWQ  184
ident       |                |
Sbjct YLGVCRLPG--PGRRHRRLDIIVVpYSEF---------------------------aCAL  253
DSSP  EEEEELLLL--LLLLLEEEEEEEELlHHHH---------------------------hHHH


DSSP  HhhhhhhhhllllllllllllHHHHHHH-----------------------hhhhhllhh
Query IaqklhavtavleepkandraHDLVDLQ-----------------------llegllda  221
ident
Sbjct L----------------yftgSAHFNRSmralaktkgmslsehalstavvrnthgckvgp  297
DSSP  H----------------hhhlLHHHHHhhhhhhlleeellllleeellllllllllll


DSSP  hhhhhHHHHHhhhhhhllllLLLLLlllllhhhhhhhhhllllllllllhhhhhhhhhh
Query dlxptRSACIaifearaqhpWPPRVatlphwpliyagalegldhlelartvdaaaqavqr  281
ident
Sbjct grvlpTPTEK-------dvfRLLGL-----------------------------------  315
DSSP  lleelLLLHH-------hhhHHHLL-----------------------------------


DSSP  hhhhhhhllll
Query fvaridratkr  292
ident
Sbjct -pyrepaerdw  325
DSSP  -llllhhhlll
```

No 515: Query=mol1A Sbjct=4k4hE Z-score=5.1

back to top

```
DSSP  ------------------------------------------------------------
Query ------------------------------------------------------------    0
ident
Sbjct atnhnlhiteklevlakaysvqgdkwralgyakainalksfhkpvtsyqeacsipgigkr   60
DSSP  lllllhhhhhhhhhhhhhhhhlllhhhhhhhhhhhhhhhhlllllllhhhhhlllllllhh


DSSP  ----------------------------------------------llllllllllhhhh
Query ----------------------------------------------aapysspptnlrsl   14
ident
Sbjct maekiieilesghlrkldhisesvpvlelfsniwgagtktaqmwyqqgfrsledirsqas  120
DSSP  hhhhhhhhhhhlllhhhhhllllhhhhhhhhllllllhhhhhhhhhlllllhhhhhhhll


DSSP  hhhhhhhhhhllllhHHHHHHHHHHHHHHHHHHHLL-LLLLLLLEEEEHHHHHHHHhhllll
Query rdrltqvaerqgvvfGRLQRHVAXIVVAQFAATL-TDDTGAPLLLVKGGSSLELrrgipd   73
ident                             |             |    |
Sbjct lttqqaiglkhysdfLERMPREEATEIEQTVQKAaQAFNSGLLCVACGSYRRGK------  174
DSSP  llhhhhhhhhlhhhhHLLEEHHHHHHHHHHHHHHHHhHLLLLLLLEEEELHHHHLLL------


DSSP  llLLLLEEEEEL--------lLHHHHHHHHHHHHhhlllleeEEELLLEELllllLLLLL
Query srTSKDFATVAR--------rDIELIHEQLADAGetgwegfftAIFTAPEEIaapgXPVKP  125
ident   |  |                       |   |
Sbjct -aTCGDVDVLIThpdgrshrgIFSRLLDSLRQEG--------FLTDDLVSQ---eGQQQK  222
DSSP  -lEELLEEEEEElllllllllLHHHHHHHHHHHLL--------LEEEEEEEE---lLLLEE


DSSP  EEEEEEEEEllEEEEEEEEEEEEEL-LLLLllllleeeeelhhhhllllllleeeeellhHHH
Query RRFTAKLSYrgRAFATVPIEVSSV-EAGNadqfdtltsdalglvgvpaavavpcxtiPWQ  184
ident       |                |
Sbjct YLGVCRLPG--PGRRHRRLDIIVVpYSEF---------------------------aCAL  253
DSSP  EEEEELLLL--LLLLLEEEEEEEELlHHHH---------------------------hHHH


DSSP  HhhhhhhhhllllllllllllHHHHHHH-------------------------hhhhhll
Query IaqklhavtavleepkandraHDLVDLQ-------------------------llegll  219
ident
Sbjct L----------------yftgSAHFNRSmralaktkgmslsehalstavvrnthgckvgp  297
DSSP  H----------------hhhlLHHHHHhhhhhhllleellllleellllllllllllll


DSSP  hHHHhhHHHHHhhhhhhllllLLLLLlllllhhhhhhhhhlllllllllllhhhhhhhh
Query dADLXptRSACIaifearaqhpWPPRVatlphwpliyagalegldhlelartvdaaaqav  279
ident    |
```

```
DSSP  hhhhhhhhhllll
Query qrfvaridratkr  292
ident
Sbjct --pyrepaerdwl  326
DSSP  --llllhhhllll
```

No 516: Query=mol1A Sbjct=5iiiA Z-score=5.1

back to top

```
DSSP  ------------------------------------------------------------
Query ------------------------------------------------------------    0
ident
Sbjct hnlhiteklevlakaysvqgdkwralgyakainalksfhkpvtsyqeacsipgigkrmae   60
DSSP  llllhhhhhhhhhhhhhhlllhhhhhhhhhhhhhhhllllllllhhhhhhlllllhhhhh


DSSP  -------------------------------------------llllllllllhhhhhhh
Query -------------------------------------------aapysspptnlrslrdr   17
ident
Sbjct kiieilesghlrkldhisesvpvlelfsniwgagtktaqmwyqqgfrsledirsqasltt  120
DSSP  hhhhhhlllllhhhhhllllhhhhhhhhlllllhhhhhhhhhlllllhhhhhhhhllllh


DSSP  hhhhhhhllllhhHHHHHHHHHHHHHHHHHLL-LLLLLLLLEEEEHHHHHHHhhllllllL
Query ltqvaerqgvvfgRLQRHVAXIVVAQFAATL-TDDTGAPLLLVKGGSSLELrrgipdsrT   76
ident                             |              |    |              |
Sbjct qqaiglkhysdflERMPREEATEIEQTVQKAaQAFNSGLLCVACGSYRRGK-------aT  173
DSSP  hhhhhhhlhhhhhLLEEHHHHHHHHHHHHHHHhHHHLLLLEEEELHHHHLLL-------lE


DSSP  LLLEEEEEL--------lLHHHHHHHHHHHHhhlllleeeEELLLEELLllllLLLLEEE
Query SKDFATVAR--------rDIELIHEQLADAGetgwegftaIFTAPEEIAapgxPVKPRRF  128
ident   |                        |   |           |
Sbjct CGDVDVLIThpdgrshrgIFSRLLDSLRQEG---------FLTDDLVSQeengQQQKYLG  224
DSSP  ELLEEEEEEllllllllLHHHHHHHHHHLL---------LEEEEEELLllllLLLEEEE


DSSP  EEEEEEllEEEEEEEEEEEEELLLLLLllleeeeelhhhhllllleeeeellhHHHHhhh
Query TAKLSYrgRAFATVPIEVSSVEAGNAdqfdtltsdalglvgvpaavavpcxtiPWQIaqk  188
ident     |                |
Sbjct VCRLPG--PGRRHRRLDIIVVPYSEF--------------------------aCALL---  253
DSSP  EELLLL--LLLLLEEEEEEEELLHHHH--------------------------hHHHH---


DSSP  hhhhhllllllllllllHHHHHHH---------------hhhhhllhHHHHhhHHHHHhh
Query lhavtavleepkandraHDLVDLQ---------------llegllldADLXptRSACIai  233
ident                                                   |
Sbjct -------------yftgSAHFNRSmralaktkgmslsehalstavpgRVLP--TPTEK--  296
DSSP  -------------hhhlLHHHHHHhhhhhhllleellllleelllllLLLL--LLLHH--


DSSP  hhhhllllLLLLLlllllhhhhhhhhhllllllllllhhhhhhhhhhhhhhhhhllll
Query fearaqhpWPPRVatlphwpliyagalegldhlelartvdaaaqavqrfvaridratkr  292
ident
Sbjct -----dvfRLLGL------------------------------------pyrepaerdw  314
DSSP  -----hhhHHLLL------------------------------------llllhhhhll
```

No 517: Query=mol1A Sbjct=5dkwB Z-score=5.1

back to top

```
DSSP  ------------------------------------------------------------
Query ------------------------------------------------------------    0
ident
Sbjct atnhnlhiteklevlakaysvqgdkwralgyakainalksfhkpvtsyqeacsipgigkr   60
DSSP  llllhhhhhhhhhhhhhhhhlllhhhhhhhhhhhhhhhllllllllhhhhhllllllhh


DSSP  ----------------------------------------------llllllllllhhhh
```

```
Sbjct maekiieilesghlrkldhisesvpvlelfsniwgagtktaqmwyqqgfrsledirsqas  120
DSSP  hhhhhhhhhhllllhhhhlllllhhhhhhhllllllhhhhhhhhhlllllhhhhhhhll


DSSP  hhhhhhhhhhllllhHHHHHHHHHHHHHHHHHHLL-LLLLLLLLEEEEHHHHHHHhhllll
Query rdrltqvaerqgvvfGRLQRHVAXIVVAQFAATL-TDDTGAPLLLVKGGSSLELrrgipd   73
ident                             |             |    |
Sbjct lttqqaiglkhysdfLERMPREEATEIEQTVQKAaQAFNSGLLCVACGSYRRGK------  174
DSSP  llhhhhhhhhlhhhhHLLEEHHHHHHHHHHHHHHHhHLLLLLLEEEEEEHHHLLLL------


DSSP  llLLLLLEEEEEL--------lLHHHHHHHHHHHHHhhllllleeeEELLL-EELLLlLLLLL
Query srTSKDFATVAR--------rDIELIHEQLADAGetgwegftaIFTAP-EEIAApGXPVK  124
ident   |  |                       |   |           |
Sbjct -aTCGDVDVLIThpdgrshrgIFSRLLDSLRQEG---------FLTDDlVSQEE-NGQQQ  223
DSSP  -lEELLEEEEEEllllllllLHHHHHHHHHHLL---------LEEEEeEEEEE-ELLEE


DSSP  LEEEEEEEEEllEEEEEEEEEEEEL-LLLLllllleeeeelhhhhllllllleeeeellhHH
Query PRRFTAKLSYrgRAFATVPIEVSSV-EAGNadqfdtltsdalglvgvpaavavpcxtiPW  183
ident        |                |
Sbjct KYLGVCRLPG--PGRRHRRLDIIVVpYSEF---------------------------aCA  254
DSSP  EEEEEELLLL--LLLLLEEEEEEEELlHHHH---------------------------hHH


DSSP  HHhhhhhhhhllllllllllllHHHHHHH-----------------hhhhhllhHHHHhh
Query QIaqklhavtavleepkandraHDLVDLQ-----------------lleglldADLXpt  226
ident                                                         |
Sbjct LL----------------yftgSAHFNRSmralaktkgmslsehalstavvgpgRVLP--  296
DSSP  HH----------------hhhlLHHHHHHhhhhhhhlleeellllleeellllllLEEL--


DSSP  HHHHHhhhhhhllllLLLLLLLllllhhhhhhhhhlllllllllllhhhhhhhhhhhhhh
Query RSACIaifearaqhpWPPRVAtlphwpliyagalegldhlelartvdaaaqavqrfvari  286
ident
Sbjct TPTEK-------dvfRLLGLP-------------------------------------yre  313
DSSP  LLLHH-------hhhHLLLLL-------------------------------------lll


DSSP  hhllll
Query dratkr  292
ident
Sbjct paerdw  319
DSSP  hhhlll
```

No 518: Query=mol1A Sbjct=4xa5A Z-score=5.1

back to top

```
DSSP  ------------------------------------------------------------
Query ------------------------------------------------------------    0
ident
Sbjct hnlhiteklevlakaysvqgdkwralgyakainalksfhkpvtsyqeaxsipgigkrmae   60
DSSP  llllhhhhhhhhhhhhhhlllhhhhhhhhhhhhhhhllllllllhhhhhhlllllhhhhh


DSSP  -------------------------------------------llllllllllhhhhhhh
Query -------------------------------------------aapysspptnlrslrdr   17
ident
Sbjct kiieilesghlrkldhisesvpvlelfsniwgagtktaqmwyqqgfrsledirsqasltt  120
DSSP  hhhhhhhllllhhhhhllllhhhhhhhhhllllllhhhhhhhhhlllllhhhhhhhhllllh


DSSP  hhhhhhhllllhhHHHHHHHHHHHHHHHHHHLL-LLLLLLLLEEEEHHHHHHHhhllllllL
Query ltqvaerqgvvfgRLQRHVAXIVVAQFAATL-TDDTGAPLLLVKGGSSLELrrgipdsrT   76
ident                          |             |    |              |
Sbjct qqaiglkhysdflERMPREEATEIEQTVQKAaQAFNSGLLCVACGSYRRGK-------aT  173
DSSP  hhhhhhhlhhhhhLLEEHHHHHHHHHHHHHHHHhHHHLLLLEEEELHHHHLLL-------lE


DSSP  LLLLEEEEEL--------lLHHHHHHHHHHHHHhhllllleeeEELLLEELLllllLLLLEEE
Query SKDFATVAR--------rDIELIHEQLADAGetgwegftaIFTAPEEIAapgxPVKPRRF  128
ident   |                       |   |           |
Sbjct CGDVDVLIThpdgrshrgIFSRLLDSLRQEG---------FLTDDLVSQeengQQQKYLG  224
DSSP  ELLEEEEEEllllllllLHHHHHHHHHHLL---------LEEEEEEELLllllLLLEEEE
```

```
DSSP  EEEEEEllEEEEEEEEEEEEELLLLLLllleeeeelhhhhllllllleeeeellhHHHHhhh
Query TAKLSYrgRAFATVPIEVSSVEAGNAdqfdtltsdalglvgvpaavavpcxtiPWQIaqk  188
ident    |                |
Sbjct VCRLPG--PGRRHRRLDIIVVPYSEF--------------------------aCALL---  253
DSSP  EELLLL--LLLLLEEEEEEEELLHHHH--------------------------hHHHH---


DSSP  hhhhhllllllllllllHHHHHHH---------------hhhhhllhHHHHhhHHHHHhh
Query lhavtavleepkandraHDLVDLQ---------------llegllldADLXptRSACIai  233
ident                                                  |
Sbjct -------------yftgSAHFNRSmralaktkgmslsehalstavpgRVLP--TPTEK--  296
DSSP  -------------hhhlLHHHHHHhhhhhhhlleeellllleeellllLLLL--LLLHH--


DSSP  hhhhllllLLLLLllllllhhhhhhhhhllllllllllhhhhhhhhhhhhhhhhhllll
Query fearaqhpWPPRVatlphwpliyagalegldhlelartvdaaaqavqrfvaridratkr  292
ident
Sbjct -----dvfRLLGL------------------------------------pyrepaerdw  314
DSSP  -----hhhHHLLL------------------------------------llllhhhhll
```

No 519: Query=mol1A Sbjct=5dedE Z-score=5.1

back to top

```
DSSP  lllllllllhhhhhhhhhhhhhhLLLLHHHHHHHHHHHHHHHHHHHHLLL--LLLL---LL
Query aapysspptnlrslrdrltqvaerQGVVFGRLQRHVAXIVVAQFAATLT--DDTG---AP   55
ident                                     |                      |
Sbjct -----------------------dKQWERFLVPYRQAVEELKVKLKGIRtlYEYEddhSP   37
DSSP  -----------------------lHHHHHHHHHHHHHHHHHHHHHHHHHHhlLHHHlllLL


DSSP  LEEEEhhHHHHhhhlLLLLL------------------lLLLEEEEELL--LHHHHHHHH
Query LLLVKggSSLElrrgIPDSR------------------tSKDFATVARR--DIELIHEQL   95
ident    |              |                                ||    | |
Sbjct IEFVT--GRVK----PVASIlekarrksiplheietmqdIAGLRIMCQFvdDIQIVKEML   91
DSSP  LLEEE--EEEL----LHHHHhhhhhhllllhhhhhhlllLEEEEEEEELLhhHHHHHHHHH


DSSP  HHHHhhlllleEEEELLLEEL---LLLLLllLLEEEEEEEEEL---lEEEEEEEEEEEEL
Query ADAGetgwegfTAIFTAPEEI---AAPGXpvKPRRFTAKLSYR---gRAFATVPIEVSSV  149
ident                                                     | |
Sbjct FARK-------DFTVVDQRDYiaeHKESG-yRSYHLVVLYPLQtvsgEKHVLVEIQIRTL  143
DSSP  HHLL-------LLEEEEEEELlllLLLLL-lLLEEEEEEEEELLllllLLEEEEEEEEEEH


DSSP  LllllllleeeeelhhhhlllllleeeeellhhhhhHHHHHHHHLlllllllllllhhhh
Query EagnadqfdtltsdalglvgvpaavavpcxtipwqiAQKLHAVTAvleepkandrahdlv  209
ident
Sbjct A---------------------------------mnfWATIEHSLN---------------  156
DSSP  H---------------------------------hhhHHHHHHHHH---------------


DSSP  hhhhhhhhllhhhhhhhhhhhhhhHHHHllllllllllllllhhhhhhhhhlllllllll
Query dlqlleglllldadlxptrsaciaiFEARaqhpwpprvatlphwpliyagalegldhlela  269
ident
Sbjct --------------ykipekvklrLQRA--------------------------------  170
DSSP  --------------hlllhhhhhhHHHH--------------------------------


DSSP  llhhhhhhhhhhhhhhhhhllll
Query rtvdaaaqavqrfvaridratkr  292
ident
Sbjct ---seaasrldeemseirgevqe  190
DSSP  ---hhhhhhhhhhhhhhhhhhll
```

No 520: Query=mol1A Sbjct=3l9dB Z-score=5.1

back to top

```
DSSP  lllllllllhhhhhhhhhhhhhhlllLHHHHHHHHHHHHHHHHHHHHLLL--LLLL---ll
Query aapysspptnlrslrdrltqvaerqgvVFGRLQRHVAXIVVAQFAATLT--DDTG---ap   55
ident                                     |
```

```
DSSP  LEEEEhhHHHHhhhlLLLLL-------------------lLLLEEEEELL--LHHHHHHH
Query LLLVKggSSLElrrgIPDSR-------------------tSKDFATVARR--DIELIHEQ   94
ident    |              |                                 |     |
Sbjct IEFVT--GRVK----PIESIkekmvlrgikkenltqdmqdIAGLRIMVQFvdDVNDVLEL   87
DSSP  LLEEE--EEEL----LHHHHhhhhhhlllllllllllllLEEEEEEELLllHHHHHHHH


DSSP  HHHHHhhllllеEEEELLLEEL-LLLLLllLLEEEEEEEEEL---lEEEEEEEEEEEEELL
Query LADAGetgwegfTAIFTAPEEI-AAPGXpvKPRRFTAKLSYR---gRAFATVPIEVSSVE  150
ident |                         |                          |
Sbjct LRQRK-------DMKVIQERDYiNNSGY--RSYHVIVEYPVDtisgQRIIMAEIQIRTLA  138
DSSP  HHLLL-------LLEEEEEEEElLLLLL--LEEEEEEEEEEEelleEEEEEEEEEEEEEHH


DSSP  lllllleeeeelhhhhllllleeeeellhhhhhHHHHHHHHLllllllllllhhhhh
Query agnadqfdtltsdalglvgvpaavavpcxtipwqiAQKLHAVTAvleepkandrahdlvd  210
ident
Sbjct --------------------------------mnfWATIEHSLN----------------  150
DSSP  --------------------------------hhhHHHHHHHHH----------------


DSSP  hhhhhhhllhhhhhhhhhhhhhhhhhhllllllLLLLLLLHhhhhhhhhllllllllll
Query lqlleglllddadlxptrsaciaifearaqhpwppRVATLPHWpliyagaleglldhlelar  270
ident
Sbjct -------------------------------ykyHGEFPEDI--------krrleltski  171
DSSP  -------------------------------hhlLLLLLLHHH--------hhhhhhhhhh


DSSP  lhhhhhhhhhhhhhhhhhllll
Query tvdaaaqavqrfvaridratkr  292
ident
Sbjct afqldeemrqirddikeaqllf  193
DSSP  hhhhhhhhhhlhhhhhhhhhll
```

No 521: Query=mol1A Sbjct=1g3jA Z-score=5.1

back to top

```
DSSP  ------------------------------------------------------------
Query ------------------------------------------------------------    0
ident
Sbjct havvnlinyqddaelatraipeltkllndedqvvvnkaavmvhqlskkeasrhaimrspq   60
DSSP  lhhhhhhhhhhhhhhhhlhhhhhhhhhhllllhhhhhhhhhhhhhhhhllhhhhhhhhllhh


DSSP  LLLL----llLLLLLHHHHHHHHHHHHHHHLL---------llhHHHHHHHHHHHHH------
Query AAPY----ssPPTNLRSLRDRLTQVAERQG---------vvfGRLQRHVAXIVV------   41
ident                   |                       |
Sbjct MVSAivrtmqNTNDVETARCTAGTLHNLSHhregllaifksgGIPALVKMLGSPvdsvlf  120
DSSP  HHHHhhhhhhHLLLHHHHHHHHHHHHHHHHLLlhhhhhhhhhllHHHHHHHHHHHLLlhhhhh


DSSP  --------------hhhhhllllllllllеEEEH--------HHHHHHHHLllllllll
Query --------------aqfaatltddtgapllLVKG--------GSSLELRRGipdsrtskd   79
ident                                                |
Sbjct yaittlhnlllhqegakmavrlagglqkmvALLNktnvkflaITTDCLQIL---------  171
DSSP  hhhhhhhhhhhhlllhhhhhhhllhhhhhhHHHHlllhhhhhhHHHHHHHHH---------


DSSP  eeeeelllhhhhhhhhhhhhhhllllееeeellleellllllllllеееееееееlleee
Query fatvarrdieliheqladagetgwegftaiftapeeiaapgxpvkprrftaklsyrgraf  139
ident
Sbjct ------------------------------------------------------------  171
DSSP  ------------------------------------------------------------


DSSP  eeeeeeeeellllllllleeeeelhhhhllllleeeeeLLHHHHHHHHHHHHhllllll
Query atvpievssveagnadqfdtltsdalglvgvpaavavpcXTIPWQIAQKLHAVtavleep  199
ident                                           |
Sbjct --------------------------aygnqeskliilaSGGPQALVNIMRTY-------  198
DSSP  --------------------------hlllhhhhhhhhhLLHHHHHHHHHHLLL-------
```

```
ident         |         |            |                  |
Sbjct ---tYEKLLWTTSRVLKVLsvCSSNKPAIVEAG--------gmqalGLHLT---------  238
DSSP  ---lLHHHHHHHHHHHHHHllLLLHHHHHHHLL--------hhhhhHLLLL---------


DSSP  hhhlllllllllLHHHHHHHHHHHHHHHHHHLLLL-------------------------
Query galegldhlelarTVDAAAQAVQRFVARIDRATKR-------------------------  292
ident                    |           |
Sbjct ------------dPSQRLVQNCLWTLRNLSDAATKqegmegllgtlvqllgsddinvvtc  286
DSSP  ------------lLLHHHHHHHHHHHHHHHHHLLLLLllllhhhhhhhhhhlllllhhhhhh


DSSP  ------------------------------------------------------------
Query ------------------------------------------------------------  292
ident
Sbjct aagilsnltcnnyknkmmvcqvggiealvrtvlragdreditepaicalrhltsrhqeae  346
DSSP  hhhhhhhhhlllhhhhhhhhhllhhhhhhhhhhhhlllhhhhhhhhhhhhhhlllllllhh


DSSP  ------------------------------------------------------------
Query ------------------------------------------------------------  292
ident
Sbjct maqnavrlhyglpvvvkllhppshwplikatvglirnlalcpanhaplreqgaiprlvql  406
DSSP  hhhhhhhhlllhhhhhhllllllhhhhhhhhhhhhhhlllhhhhhhhhhllhhhhhhhh


DSSP  ------------------------------------------------------------
Query ------------------------------------------------------------  292
ident
Sbjct lvrahqdtqrrfvegvrmeeivegctgalhilardvhnrivirglntiplfvqllyspie  466
DSSP  hhhhhhllllllllllhhhhhhhhhhhhhhhhlllhhhhhhhhhlllhhhhhhhhhlllh


DSSP  --------------------------------------------------------
Query --------------------------------------------------------  292
ident
Sbjct niqrvaagvlcelaqdkeaaeaieaegatapltellhsrnegvatyaaavlfrmse  522
DSSP  hhhhhhhhhhhhhlllhhhhhhhhhlllhhhhhhhlllllhhhhhhhhhhhhhhhl
```

No 522: Query=mol1A Sbjct=1v18A Z-score=5.1

back to top

```
DSSP  -------------------------------------------LLLL----llLLLLHH
Query -------------------------------------------AAPY----ssPPTNLR   12
ident
Sbjct traipeltkllndedqvvvnkaavmvhqlskkeasrhaimrspqMVSAivrtmqNTNDVE   60
DSSP  llhhhhhhhhhllllhhhhhhhhhhhhhhhhllhhhhhhhhllhhHHHHhhhhhlLLLLHH


DSSP  HHHHHHHHHHHHLL---------llhHHHHHHHHHHHH--------------------hh
Query SLRDRLTQVAERQG---------vvfGRLQRHVAXIVV--------------------aq   43
ident   |                       |
Sbjct TARCTAGTLHNLSHhregllaifksgGIPALVKMLGSPvdsvlfyaittlhnlllhqega  120
DSSP  HHHHHHHHHHHHLLlhhhhhhhhhllHHHHHHHHLLLLlhhhhhhhhhhhhhhhhhhlllh


DSSP  hhhlllllllllleEEEH--------HHHHHHHHLlllllllleeeeelllhhhhhhhh
Query faatltddtgapllLVKG--------GSSLELRRGipdsrtskdfatvarrdieliheql   95
ident                                |
Sbjct kmavrlagglqkmvALLNktnvkflaITTDCLQIL-------------------------  155
DSSP  hhhhhhllhhhhhhHHHHlllhhhhhHHHHHHHHH-------------------------


DSSP  hhhhhhllllleeeeellleellllllllllleeeeeeeeelleeeeeeeeeeeellllll
Query adagetgwegftaiftapeeiaapgxpvkprrftaklsyrgrafatvpievssveagnad  155
ident
Sbjct ------------------------------------------------------------  155
DSSP  ------------------------------------------------------------


DSSP  lleeeeelhhhhllllleeeeeLLHHHHHHHHHHHHhlllllllllLLHHHHHHHHH
Query qfdtltsdalglvgvpaavavpcXTIPWQIAQKLHAVtavleepkandRAHDLVDLQLLE  215
ident                           |                         |
Sbjct ----------aygnqesklriilaSGGPQALVNIMRTY----------tYEKLLWTTSRVL  195
```

```
DSSP  HHL--LHHHHHHHHHHHhhhhhhhllllllLLLLLlllhhhhhhhhhlllllllllLHH
Query GLL--LDADLXPTRSACiaifearaqhpwpPRVATlphwpliyagalegldhlelarTVD  273
ident   |            |                  |
Sbjct KVLsvCSSNKPAIVEAG--------gmqalGLHLT---------------------dPSQ  226
DSSP  HHHllLLLHHHHHHHLL--------hhhhhHLLLL---------------------lLLH


DSSP  HHHHHHHHHHHHHHHLLLL-----------------------------------------
Query AAAQAVQRFVARIDRATKR-----------------------------------------  292
ident    |           |
Sbjct RLVQNCLWTLRNLSDAATKqegmegllgtlvqllgsddinvvtcaagilsnltcnnyknk  286
DSSP  HHHHHHHHHHHHHHLLLLLllllhhhhhhhhhhllllhhhhhhhhhhhhhhhllllhhhh


DSSP  ------------------------------------------------------------
Query ------------------------------------------------------------  292
ident
Sbjct mmvcqvggiealvrtvlragdreditepaicalrhltsrhqeaemaqnavrlhyglpvvv  346
DSSP  hhhhhllhhhhhhhhhhhhlllhhhhhhhhhhhhhhlllllhhhhhhhhhhlllhhhhh


DSSP  ------------------------------------------------------------
Query ------------------------------------------------------------  292
ident
Sbjct kllhppshwplikatvglirnlalcpanhaplreqgaiprlvqllvrahqdtqrrqfveg  406
DSSP  hllllllhhhhhhhhhhhhhhlllhhhhhhhhhllhhhhhhhhhhhhhhhhhlllllll


DSSP  ------------------------------------------------------------
Query ------------------------------------------------------------  292
ident
Sbjct vrmeeivegctgalhilardvhnrivirglntiplfvqllyspieniqrvaagvlcelaq  466
DSSP  llhhhhhhhhhhhhhhhlllhhhhhhhhhlllhhhhhhhlllllhhhhhhhhhhhhhhll


DSSP  -----------------------------------------
Query -----------------------------------------  292
ident
Sbjct dkeaaeaieaegatapltellhsrnegvatyaaavlfrmse  507
DSSP  lhhhhhhhhhlllhhhhhhhllllhhhhhhhhhhhhhhll
```

No 523: Query=mol1A Sbjct=1t08A Z-score=5.1

back to top

```
DSSP  ------------------------------------------------LLLL----llLL
Query ------------------------------------------------AAPY----ssPP    8
ident
Sbjct aelatraipeltkllndedqvvvnkaavmvhqlskkeasrhaimrspqMVSAivrtmqNT   60
DSSP  lhhhhlhhhhhhhhlllllhhhhhhhhhhhhhhhllhhhhhhhhllhhHHHHhhhhhlLL


DSSP  LLHHHHHHHHHHHHHHLL---------llhHHHHHHHHHHH-------------------
Query TNLRSLRDRLTQVAERQG---------vvfGRLQRHVAXIV-------------------   40
ident       |                       |
Sbjct NDVETARCTAGTLHNLSHhregllaifksgGIPALVKMLGSpvdsvlfyaittlhnlllh  120
DSSP  LLHHHHHHHHHHHHHHLLlhhhhhhhhhllHHHHHHHHHHLllhhhhhhhhhhhhhhhhh


DSSP  -hhhhhhlllllllllleEEEH--------HHHHHHHHLlllllllleeeeelllhhhh
Query -vaqfaatltddtgapllLVKG--------GSSLELRRGipdsrtskdfatvarrdieli   91
ident                                    |
Sbjct qegakmavrlagglqkmvALLNktnvkflaITTDCLQIL---------------------  159
DSSP  lllhhhhhhhllhhhhhhHHLLlllhhhhhHHHHHHHHH---------------------


DSSP  hhhhhhhhhhllllleeeeeeellleellllllllllleeeeeeeeelleeeeeeeeeeeeelll
Query heqladagetgwegftaiftapeeiaapgxpvkprrftaklsyrgrafatvpievssvea  151
ident
Sbjct ------------------------------------------------------------  159
DSSP  ------------------------------------------------------------


DSSP  lllllleeeeelhhhhllllllleeeeeLLHHHHHHHHHHHHHhllllllllllLLHHHHHH
```

```
Sbjct --------------aygnqeskliilaSGGPQALVNIMRTY----------tYEKLLWTT  195
DSSP  --------------hlllhhhhhhhhhLLHHHHHHHHHHHL----------lLHHHHHHH


DSSP  HHHHHHL--LHHHHHHHHHHHhhhhhhlllllllLLLLLlllhhhhhhhhhlllllllll
Query QLLEGLL--LDADLXPTRSACiaifearaqhpwpPRVATlphwpliyagalegldhlela  269
ident       |            |                  |
Sbjct SRVLKVLsvCSSNKPAIVEAG--------gmqalGLHLT---------------------  226
DSSP  HHHHHHHllLLLHHHHHHHLL--------hhhhhHLLLL---------------------


DSSP  lLHHHHHHHHHHHHHHHHHLLLL-------------------------------------
Query rTVDAAAQAVQRFVARIDRATKR-------------------------------------  292
ident        |           |
Sbjct dPSQRLVQNCLWTLRNLSDAATKqegmegllgtlvqllgsddinvvtcaagilsnltcnn  286
DSSP  lLLHHHHHHHHHHHHHHHLLLLLllllhhhhhhhhhhlllllhhhhhhhhhhhhhhllll


DSSP  ------------------------------------------------------------
Query ------------------------------------------------------------  292
ident
Sbjct yknkmmvcqvggiealvrtvlragdreditepaicalrhltsrhqeaemaqnavrlhygl  346
DSSP  hhhhhhhhhhllhhhhhhhhhhhhlllhhhhhhhhhhhhhhlllllhhhhhhhhhhlllh


DSSP  ------------------------------------------------------------
Query ------------------------------------------------------------  292
ident
Sbjct pvvvkllhppshwplikatvglirnlalcpanhaplreqgaiprlvqllvrahqdtqrqf  406
DSSP  hhhhllllllllhhhhhhhhhhhhhhhlllhhhhhhhhllhhhhhhhhhhhhhhhhhlll


DSSP  ------------------------------------------------------------
Query ------------------------------------------------------------  292
ident
Sbjct vegvrmeeivegctgalhilardvhnrivirglntiplfvqllyspieniqrvaagvlce  466
DSSP  lllllhhhhhhhhhhhhhhhhlllhhhhhhhhhlllhhhhhhhllllllhhhhhhhhhhhh


DSSP  --------------------------------------------
Query --------------------------------------------  292
ident
Sbjct laqdkeaaeaieaegatapltellhsrnegvatyaaavlfrmse  510
DSSP  hlllhhhhhhhhhhhlllhhhhhhhhllllhhhhhhhhhhhhhl
```

No 524: Query=mol1A Sbjct=4rwpA Z-score=5.0

back to top

```
DSSP  lllllllllhhhhhhhhhhhhhHHLL-LLHHHHHHHHHHHHHHHHHH--LLLLLLLLLLE
Query aapyssspptnlrslrdrltqvaERQG-VVFGRLQRHVAXIVVAQFAA--TLTDDTGAPLL   57
ident                                |  |   |   |  |
Sbjct ------melrhtpardldkfieDHLLpNTCFRTQVKEAIDIVCRFLKerCFQGTADPVRV   54
DSSP  ------llhhhllhhhhhhhhhHHLLlLHHHHHHHHHHHHHHHHHHHhlLLLLLLLLLLE


DSSP  -EEEHHHhHHHHHLLLlllllLLEEEEELL---------LHHHHHHHHHHHHHHLL--LL
Query -LVKGGSsLELRRGIPdsrtsKDFATVARR---------DIELIHEQLADAGETGW--EG  105
ident   |  |                |                             |
Sbjct sKVVKGG-SSGKGTTLrgrsdADLVVFLTKltsfedqlrRRGEFIQEIRRQLEACQreQK  113
DSSP  eEEEELH-HHHHLLLLlllleEEEEEEELLlllhhhhhhHHHHHHHHHHHHHHHHHhlLL


DSSP  EEEEELlleellllllllllEEEEEEEEEL-leeEEEEEEEEEELLLLllllleeeeelh
Query FTAIFTapeeiaapgxpvkpRRFTAKLSYR-graFATVPIEVSSVEAGnadqfdtltsda  164
ident |   |               |     ||                   |
Sbjct FKVTFE-----vqsprrenpRALSFVLSSPqlqqEVEFDVLPAFDALG------------  156
DSSP  LEEEEL-----lllllllllLLEEEEEELLllllLEEEEEEEEELLLL------------


DSSP  hhhlllllleeeeelLHHHhhhhhhhhhhllllllllllllhhhhhhhhhhhhllhhhhh
Query lglvgvpaavavpcxTIPWqiaqklhavtavleepkandrahdlvdlqlleglllldadlx  224
ident
Sbjct ---------------QWTP----------------------------------------g  161
DSSP  ---------------LLLL----------------------------------------l
```

```
DSSP  HHHHhhHHHHHHHLL---------------------------------------llllll
Query PTRSacIAIFEARAQ---------------------------------------hpwppr  245
ident         |
Sbjct YKPN--PEIYVQLIKecksrgkegefstcftelqrdflrnrptklkslirlvkhwyqtck  219
DSSP  LLLL--LHHHHHHHHhhllllllllhhhhhhhhhhhhhhlllhhhhhhhhhhhhhhhhhh


DSSP  llllllhhhhhHHHH--------------------lllllllllllhHHHHhhhHHHH--
Query vatlphwpliyAGAL--------------------egldhlelartvDAAAqavQRFV--  283
ident            |  |
Sbjct kthgnklppqyALELltvyaweqgsrktdfstaqgfqtvlelvlkhqKLCI---FWEAyy  276
DSSP  hhhllllllhhHHHHhhhhhhhhhhllllllhhhhhhhhhhhhhlhhHLEE---LLLLll


DSSP  ------------------------------------------------------------
Query ------------------------------------------------------------  283
ident
Sbjct dftnpvvgrcmlqqlkkprpvildpadptgnvgggdthswqrlaqearvwlgypccknld  336
DSSP  llllhhhhhhhhhhlllllleellllllllllllllhhhhhhhhhhhhhhlllhhhllll


DSSP  -hhhhhllll
Query -aridratkr  292
ident
Sbjct gslvgawtml  346
DSSP  llllllllll
```

No 525: Query=mol1A Sbjct=3l9dA Z-score=5.0

back to top

```
DSSP  llllllllllhhhhhhhhhhhhhhLLLLHHHHHHHHHHHHHHHHHHLL--LLLL--LLLL
Query aapysspptnlrslrdrltqvaerQGVVFGRLQRHVAXIVVAQFAATL--TDDT--GAPL   56
ident                                     |
Sbjct ------------------------MNWEEFLDPYIQAVGELKIKFRGIrkQFRKqkRHSP   36
DSSP  ------------------------LLHHHHLHHHHHHHHHHHHHHHHHhhHHHHllLLLL


DSSP  -EEEEhhHHHHhhhlLLLLL-------------------lLLLEEEEELL--LHHHHHHH
Query -LLVKggSSLElrrgIPDSR-------------------tSKDFATVARR--DIELIHEQ   94
ident    |              |                                 |     |
Sbjct iEFVT--GRVK----PIESIkekmvlrgikkenltqdmqdIAGLRIMVQFvdDVNDVLEL   90
DSSP  lLEEE--EEEL----LHHHHhhhhhhhlllhhhhhhhlllLEEEEEEEELLlHHHHHHHH


DSSP  HHHHHhhlllleEEEELLLEEL-LLLLlllLLEEEEEEEEEL---lEEEEEEEEEEEELL
Query LADAGetgwegfTAIFTAPEEI-AAPGxpvKPRRFTAKLSYR---gRAFATVPIEVSSVE  150
ident |                                                    |
Sbjct LRQRK-------DMKVIQERDYiNNKPsgyRSYHVIVEYPVDtisgQRIIMAEIQIRTLA  143
DSSP  HHLLL-------LLEEEEEEEElLLLLlllLEEEEEEEEEEEelleEEEEEEEEEEEEHH


DSSP  llllllleeeeelhhhhlllllleeeeellhhhhhHHHHHHHHLlllllllllllhhhhh
Query agnadqfdtltsdalglvgvpaavavpcxtipwqiAQKLHAVTAvleepkandrahdlvd  210
ident
Sbjct --------------------------------mnfWATIEHSLN----------------  155
DSSP  --------------------------------hhhHHHHHHHHH----------------


DSSP  hhhhhhllhhhhhhhhhhhhhhhhhhhllllllllLLLLLLHhhhhhhhlllllllllll
Query lqllegllldadlxptrsaciaifearaqhpwpprVATLPHWpliyagalegldhlelar  270
ident
Sbjct -------------------------------ykyhGEFPEDI--------krrleltski  176
DSSP  -------------------------------hhhlLLLLHHH--------hhhhhhhhhh


DSSP  lhhhhhhhhhhhhhhhhhllll
Query tvdaaaqavqrfvaridratkr  292
ident
Sbjct afqldeemrqirddikeaqllf  198
DSSP  hhhhhhhhhhlhhhhhhhhlll
```

back to top

```
DSSP  lllllllllhhhhhhhhhhhhhHLLLLHHHHHHHHHHHHHHHHHHHLLL--LLLL---LL
Query aapysspptnlrslrdrltqvaeRQGVVFGRLQRHVAXIVVAQFAATLT--DDTG---AP   55
ident                                     |                      |
Sbjct -----------------------DKQWERFLVPYRQAVEELKVKLKGIRtlYEYEddhSP   37
DSSP  -----------------------LLHHHHHHHHHHHHHHHHHHHHHHHHHHhhHLLLlllLL


DSSP  LEEEEhhHHHHhhhlLLLLL------------------lLLLEEEEELL--LHHHHHHHH
Query LLLVKggSSLElrrgIPDSR------------------tSKDFATVARR--DIELIHEQL   95
ident    |              |                                ||    | |
Sbjct IEFVT--GRVK----PVASIlekarrksiplheietmqdIAGLRIMCQFvdDIQIVKEML   91
DSSP  LLLEE--EEEL----LHHHHhhhhhhllllhhhhhhlllLEEEEEEEELLhhHHHHHHHHH


DSSP  HHHHhhlllleEEEELLLEELlllllllLEEEEEEEEEL---lEEEEEEEEEEEEELLll
Query ADAGetgwegfTAIFTAPEEIaapgxpvkPRRFTAKLSYR---gRAFATVPIEVSSVEag  152
ident                                                  | |
Sbjct FARK-------DFTVVDQRDY------iaSYHLVVLYPLQtvsgEKHVLVEIQIRTLA--  136
DSSP  HHLL-------LLEEEEELLL------llLEEEEEEEEEEllllEEEEEEEEEEEEEHH--


DSSP  llllleeeeelhhhhllllllleeeeellhhhhhHHHHHHHHLllllllllllhhhhhhh
Query nadqfdtltsdalglvgvpaavavpcxtipwqiAQKLHAVTAvleepkandrahdlvdlq  212
ident
Sbjct ------------------------------mnfWATIEHSLN------------------  148
DSSP  ------------------------------hhhHHHHHHHHH------------------


DSSP  hhhhhllhhhhhhhhhhhhhhhhhhlllllllLLLLLLHhhhhhhhhlllllllllllh
Query llegllldadlxptrsaciaifearaqhpwpprVATLPHWpliyagalegldhlelartv  272
ident
Sbjct -----------------------------ykysGNIPEKV------------klrlqras  167
DSSP  -----------------------------hhllLLLLHHH------------hhhhhhhh


DSSP  hhhhhhhhhhhhhhhhllll
Query daaaqavqrfvaridratkr  292
ident
Sbjct eaasrldeemseirgevqea  187
DSSP  hhhhhhhhhhhhhhhhhlll
```

No 527: Query=mol1A Sbjct=3hiyA Z-score=5.0

back to top

```
DSSP  lllllllllhhhhhhhhhhhhhllLLHHHHHHHHHHHHHHHHHHHLLllllllLLEEEE
Query aapysspptnlrslrdrltqvaerqgVVFGRLQRHVAXIVVAQFAATLtddtgaPLLLVK   60
ident                                   |     |                 |
Sbjct -------vakrefirgxxahyraslpPPEHSVVIHELQKRVLDIGXLA-----vNKAHVE   48
DSSP  -------lhhhhhhhhhhhhhhllllLLLHHHHHHHHHHHHHHHHHHH-----lLLLEEE


DSSP  HHhhhhhhhllllllllLLEEEEEL-------------lLHHHHHHHHHHHHHHHLlllEE
Query GGsslelrrgipdsrtsKDFATVAR-------------rDIELIHEQLADAGETGwegFT  107
ident                   |     |
Sbjct LF--gshvsgfctphsdADISLTYRnfspwlqgxervdeQNNKRXTRFGKEASAX---GX  103
DSSP  EE--lhhhhllllllllEEEEEEELLlllhhhlllhhhhhHHHHHHHHHHHHHHHH---LL


DSSP  EEELLLEELllllllllLEEEEEEEEEEllеEEEEEEEEEEellllllllleeeeelhhhh
Query AIFTAPEEIaapgxpvkPRRFTAKLSYrgrAFATVPIEVSsveagnadqfdtltsdalgl  167
ident
Sbjct EDVRYIRAR--------IPVVQFTDGV---TGIHCDVSIG--------------------  132
DSSP  EEEEEELLL--------LLEEEEELLL---LLLEEEEEEL--------------------


DSSP  llllllеeeeellhhhhhhhhhhhhhllllllllLLLLhhhhhhhhhhhhllhhhhhhhh
Query vgvpaavavpcxtipwqiaqklhavtavleepkaNDRAhdlvdlqllegllldadlxptr  227
ident
Sbjct ------------------------niggvenskiLCAI----------------------  146
DSSP  ------------------------lhhhhhhhhhHHHH----------------------
```

```
Query saciaifearaqhpwpprvatlpHWPLiYAGA-LEGL-DHLE-----------------l  268
ident                               ||
Sbjct ---------------------rQVFPdFYGAyIHLVkAWGKareviaperstfnsftvt  184
DSSP  ---------------------hHLLHhHHHHhHHHHhHHHHhlllllhhhllllhhhhh


DSSP  llLHHHHHH---------------------------------------------------
Query arTVDAAAQ---------------------------------------------------  277
ident
Sbjct txALXVLQElgllpvfskptgefgeltvadaexllqefklppiydslhdddeklgeavff  244
DSSP  hhHHHHHHHllllllllllllllllhhhhhhhhhhllllhhhhhhllllhhhhhhhhhh


DSSP  --------------------hHHHHHHHhhHLLL--------------------------
Query --------------------aVQRFVARidRATK--------------------------  291
ident
Sbjct clqrfaeyyakydfsagtvsLIHPRRHrtVYERvvrrhlellgsrkrlewekhiaehked  304
DSSP  hhhhhhhhhhhlllllllllLLLLLLLhhHHHHhhhhhhhhhhhhhhhhhhhhhhhlhhh


DSSP  ------------------------------------------------------------
Query ------------------------------------------------------------  291
ident
Sbjct gpldendfsasxqnettqrpsnspyvvedfvnyvncgrrvqasrvrhiqqefnrlrexli  364
DSSP  llllhhhhhhhhhhhhhllllllllllllllllllllllllhhhhhhhhhhhhhhhhhl


DSSP  -----------------l
Query -----------------r  292
ident
Sbjct dkeselkfdevfresdtv  382
DSSP  llhhhllhhhhlllllll
```

No 528: Query=mol1A Sbjct=4e7xB Z-score=5.0

back to top

```
DSSP  lllllllllhhhhhhhhhhhhhhllLLHHHHHHHHHHHHHHHHHHHHLLLlllllLLEEEE
Query aapysspptnlrslrdrltqvaerqgVVFGRLQRHVAXIVVAQFAATLTddtgaPLLLVK   60
ident                                      |                  |
Sbjct ---------hkeftkfcyevyneikiSDKEFKEKRAALDTLRLCLKRIS-----PDAELV   46
DSSP  ---------lhhhhhhhhhhhhhhllLHHHHHHHHHHHHHHHHHHHHLLLL-----LLLEEE


DSSP  HHhhhhhhhllllllLLLLEEEEELllhHHHHHHHHHHHHHHLlllEEEEELLLEEllll
Query GGsslelrrgipdsrTSKDFATVARrdiELIHEQLADAGETGwegfTAIFTAPEEiaapg  120
ident                   |           |  |
Sbjct AF--gslesglalknSDMDLCVLMD---DTIALQFYEELIAE----GFEGKFLQR-----   92
DSSP  EE--lhhhhllllllLLEEEEEELL---LLHHHHHHHHHHHH----LLLEEEEEE-----


DSSP  llLLLEEEEEEEeelleeeeEEEEEEEellllllllleeeeelhhhhllllllleeeeell
Query xpVKPRRFTAKLsyrgrafaTVPIEVSsveagnadqfdtltsdalglvgvpaavavpcxt  180
ident                        |
Sbjct --ARIPIIKLTS--------QCDIGFN---------------------------------  109
DSSP  --LLEEEEEELL--------LEEEEEL---------------------------------


DSSP  hhhhhhHHHHHHhllllllllLLLLhhhhhhhhhhhhllhhhhhhhhhhhhhhhhhhlll
Query ipwqiaQKLHAVtavleepkaNDRAhdlvdlqllegllldadlxptrsaciaifearaqh  240
ident
Sbjct --nrlaIHNTLL---------LSSY-----------------------------------  123
DSSP  --lhhhHHHHHH---------HHHH-----------------------------------


DSSP  llllllllLHHHhHHHHHL-lLLLL-----------------llllLHHHHHH-----
Query pwpprvatlpHWPLiYAGALE-gLDHL-----------------elarTVDAAAQ-----  277
ident
Sbjct --------tkLDAR-LKPMVLlvKHWAkrkqinspyfgtlssygyvlmVLYYLIHvikpp  174
DSSP  --------hhHLLL-HHHHHHhhHHHHhhlllllhhhlllllhhhhhhhHHHHHHHlllll


DSSP  ------------------------------------------------------------
Query ------------------------------------------------------------  277
ident
```

```
DSSP  hhHHHH----hhhhHLLLL-----------------------------------------
Query avQRFV----aridRATKR-----------------------------------------  292
ident    |
Sbjct vtFRRPdgyltkqeKGWTSdryilaiedpfeishnvgrtvsssglyrirgefmaasrlln  294
DSSP  elLLLLlllllhhhHLLLLllllleellllllllllhhhhllhhhhhhhhhhhhhhhhhhl


DSSP  ------------------
Query ------------------  292
ident
Sbjct ssypipydslfeeapipp  312
DSSP  llllllhhhhhlllllll
```

No 529: Query=mol1A Sbjct=4nktB Z-score=5.0

back to top

```
DSSP  llllllllllhhhhhhhhhhhhhhllLLHHHHHHHHHHHHHHHHHHLLLlllllLLEEEE
Query aapysspptnlrslrdrltqvaerqgVVFGRLQRHVAXIVVAQFAATLTddtgaPLLLVK   60
ident                                     |                 |
Sbjct ------dmshkeftkfcyevyneikiSDKEFKEKRAALDTLRLCLKRIS-----PDAELV   49
DSSP  ------llllhhhhhhhhhhhhhhllLHHHHHHHHHHHHHHHHHHHHHL-----LLLEEE


DSSP  HHhhhhhhhllllllllLLEEEEElllhhHHHHHHHHHHHHLllleEEEELLLEElllll
Query GGsslelrrgipdsrtsKDFATVArrdieLIHEQLADAGETGwegfTAIFTAPEEiaapg  120
ident                   |           |  |
Sbjct AF--gslesglalknsdMDLCVLM-----TIALQFYEELIAE----GFEGKFLQI-----   93
DSSP  EE--lhhhhllllllllEEEEEEL-----LHHHHHHHHHHLL----LLLEEEELL-----


DSSP  lllllEEEEEEEeelleeEEEEEEEEEellllllllleeeeelhhhhlllllleeeeell
Query xpvkpRRFTAKLsyrgraFATVPIEVSsveagnadqfdtltsdalglvgvpaavavpcxt  180
ident                        |
Sbjct -----PIIKLTS------SFQCAIGFN---------------------------------  109
DSSP  -----LEEEELL------LLLEEEEEL---------------------------------


DSSP  hhhhhhHHHHHHhllllllllLLLLhhhhhhhhhhhhllhhhhhhhhhhhhhhhhhhlll
Query ipwqiaQKLHAVtavleepkaNDRAhdlvdlqllegllldadlxptrsaciaifearaqh  240
ident
Sbjct --nrlaIHNTLL---------LSSY-----------------------------------  123
DSSP  --lhhhHHHHHH---------HHHH-----------------------------------


DSSP  llllllllllLHHHhHHHHHLL-LLLL-----------------llllLHHHHHH-----
Query pwpprvatlpHWPLiYAGALEG-LDHL-----------------elarTVDAAAQ-----  277
ident
Sbjct --------tkLDAR-LKPMVLLvKHWAkrkqinspyfgtlssygyvlmVLYYLIHvikpp  174
DSSP  --------hhHLLL-HHHHHHHhHHHHhhlllllhhhllllhhhhhhhHHHHHHHlllll


DSSP  ------------------------------------------------------------
Query ------------------------------------------------------------  277
ident
Sbjct vfpnlllsplkqekivdgfdvgfddkledippsqnysslgsllhgffrfyaykfeprekv  234
DSSP  llllhhhllllllleelleellllllhhhllllllllllhhhhhhhhhhhhhhlllllllll


DSSP  hhHHHH---hhhhHLLLL-------------------------------------------
Query avQRFV---aridRATKR-------------------------------------------  292
ident    |
Sbjct vtFRRPdgyltkqEKGWTsryilaiedpfeishnvgrtvsssglyrirgefmaasrllns  294
DSSP  llLLLLlllllhhHHLLLllllllllllllllllhhhhllhhhhhhhhhhhhhhhhhhhll


DSSP  --------------
Query --------------  292
ident
Sbjct rsypipydslfeea  308
DSSP  llllllhhhhhlll
```

back to top

```
DSSP  lllllllllhhhhhhhhhhhhhhhlLLLHHHHHHHHHHHHHHHHHH-LLLLLLL-lLLEE
Query aapyssspptnlrslrdrltqvaerqGVVFGRLQRHVAXIVVAQFAA-TLTDDTG-aPLLL   58
ident                                         |
Sbjct ---------------klkkvldklrLKRKDISEAAETVNKVVERLLrRMQKRESefKGVE   45
DSSP  ---------------lhhhhhhhhlLLHHHHHHHHHHHHHHHHHHHhHLLLLLLllLLLE


DSSP  EEHHhhhhhhhlllLLLL-----lLLEEEEEL----------------------------
Query VKGGsslelrrgipDSRT-----sKDFATVAR----------------------------   85
ident                          |
Sbjct QLNT------gsyyEHVKisapneFDVMFKLEvprielqeyyetgafylvkfkprgnpls   99
DSSP  EEEE------llhhHLLLllllleEEEEEEEElllleeeeellllllleeeeeellllllh


DSSP  -----------lLHHHHHHHHHHHHHHLlLLEEEEELLLEellllllllllEEEEEEEEe
Query -----------rDIELIHEQLADAGETGwEGFTAIFTAPEeiaapgxpvkpRRFTAKLSy  134
ident                                                       |
Sbjct hflegevlsatkMLSKFRKIIKEEVKEI-KDIDVSVEKEK--------pgsPAVTLLIR-  149
DSSP  hheelleelhhhHHHHHHHHHHHHHLLL-LLLEEEELLLL--------lllLEEEEEEE-


DSSP  llEEEEEEEEEEEELLLL---------------------llllleeeeelhhhhllllllee
Query rgRAFATVPIEVSSVEAG---------------------nadqfdtltsdalglvgvpaava  175
ident        | |       |
Sbjct -nPEEISVDIILALESKGswpistkeglpiqgwlgtkvrtnlrrepfylvpknakdgnsf  208
DSSP  -lLLEEEEEEEEEEEEELLlllhhhllllllllllllhhhhhhhlllleeeeellllllll


DSSP  eeellhHHHHhHHHHHHhllllllllllllhhhhhhhhhhhhllhhhhhhhhhhhhhhhhh
Query vpcxtiPWQIaQKLHAVtavleepkandrahdlvdlqlleglllldadlxptrsaciaife  235
ident
Sbjct qgetwrLSFS-HTEKYI-------------------------------------------  224
DSSP  lllleeEELH-HHHHHH-------------------------------------------


DSSP  hhllllllllllLLLL-------hhHHHHHHHL-lLLLL----------------lllll
Query araqhpwpprvaTLPH-------wpLIYAGALE-gLDHL----------------elart  271
ident                                |      |
Sbjct --------lnnhGIEKtccessgakCCRKECLKlmKYLLeqlkkefqeldafcsyhvkta  276
DSSP  --------hhllLLLLlllllllllLLHHHHHHhhHHHHhhhhhhlhhhllllhhhhhhh


DSSP  hhHHHH----------------------------------hhhhhhhHHHH---------
Query vdAAAQ----------------------------------avqrfvaRIDR---------  288
ident
Sbjct ifHMWTqdpqdsqwdprnlsscfdkllafflecl rtekldhyfipkfNLFSqelidrksk  336
DSSP  hhHHHHhlllhhhhlhhhhhhhhhhhhhhhhhhhhhlllllllllllLLLLlllllhhhh


DSSP  ---------------llll
Query ---------------atkr  292
ident
Sbjct eflskkieyernngfpifd  355
DSSP  hhhhhhhhhhhhlllhhhl
```

No 531: Query=mol1A Sbjct=2pfoA Z-score=5.0

back to top

```
DSSP  ------------------------------------------------------------
Query ------------------------------------------------------------    0
ident
Sbjct nlhiteklevlakaysvqgdkwralgyakainalksfhkpvtsyqeacsipgigkrmaek   60
DSSP  llllhhhhhhhhhhhhhhllhhhhhhhhhhhhhhlllllllllhhhhhlllllhhhhhh


DSSP  ------------------------------------------lllllllllhhhhhhhhh
Query ------------------------------------------aapyssspptnlrslrdrl   18
ident
Sbjct iieilesghlrkldhisesvpvlelfsniwgagtktaqmwyqqgfrsledirsqasltt q  120
DSSP  hhhhhhllllhhhhlllllhhhhhhhhllllllhhhhhhhhhlllllhhhhhhhllllhh
```

```
Query tqvaerqgvvfgrlqRHVAXIVVAQFAATL-TDDTGAPLLLVKGGSSLELrrgipdsrTS   77
ident                         |             |    |              |
Sbjct qaiglkhysdflermPREEATEIEQTVQKAaQAFNSGLLCVACGSYRRGK-------aTC  173
DSSP  hhhhhhlllhhhlleEHHHHHHHHHHHHHHhHLLLLLLEEEELHHHHLLL-------lEE


DSSP  LLEEEEEL--------lLHHHHHHHHHHHHhhlllleeeEELLLEELlllllLLLLEEEE
Query KDFATVAR--------rDIELIHEQLADAGetgwegftaIFTAPEEIaapgxPVKPRRFT  129
ident  |                       |   |           |
Sbjct GDVDVLIThpdgrshrgIFSRLLDSLRQEG---------FLTDDLVSqeengQQQKYLGV  224
DSSP  LEEEEEEEllllllllLHHHHHHHHHHLL---------LEEEEEELlllllLLLEEEEE


DSSP  EEEEEllEEEEEEEEEEEEL-LLLLllllleeeeelhhhhllllllleeeeellhHHHHhhh
Query AKLSYrgRAFATVPIEVSSV-EAGNadqfdtltsdalglvgvpaavavpcxtiPWQIaqk  188
ident   |                |
Sbjct CRLPG--PGRRHRRLDIIVVpYSEF---------------------------aCALL---  252
DSSP  ELLLL--LLLLLEEEEEEEELlHHHH---------------------------hHHHH---


DSSP  hhhhhlllllllllllHHHHHHH-------------------------hhhhhllhHHH
Query lhavtavleepkandraHDLVDLQ-------------------------lleglllldADL  223
ident                                                            |
Sbjct -------------yftgSAHFNRSmralaktkgmslsehalstavvrnthgakvgpgRVL  299
DSSP  -------------hhhlLHHHHHHhhhhhhhlleeellllleeellllllllllllllEEL


DSSP  HhhHHHHHhhhhhhllllLLLLLlllllhhhhhhhhhllllllllllhhhhhhhhhhh
Query XptRSACIaifearaqhpWPPRVatlphwpliyagaleglidhlelartvdaaaqavqrfv  283
ident
Sbjct P--TPTEK-------dvfRLLGL------------------------------------p  314
DSSP  L--LLLHH-------hhhHHLLL------------------------------------l


DSSP  hhhhhllll
Query aridratkr  292
ident
Sbjct yrepaerdw  323
DSSP  lllhhhhll
```

No 532: Query=mol1A Sbjct=1rztE Z-score=5.0

back to top

```
DSSP  ------------------------------------------------------------
Query ------------------------------------------------------------    0
ident
Sbjct tnhnlhiteklevlakaysvqgdkwralgyakainalksfhkpvtsyqeacsipgigkrm   60
DSSP  llllhhhhhhhhhhhhhhhhhllhhhhhhhhhhhhhhhllllllllhhhhhlllllhhh


DSSP  ---------------------------------------------llllllllllhhhhh
Query ---------------------------------------------aapyssspptnlrslr   15
ident
Sbjct aekiieilesghlrkldhisesvpvlelfsniwgagtktaqmwyqqgfrsledirsqasl  120
DSSP  hhhhhhhhhhlllhhhhhllllhhhhhhhhlllllllhhhhhhhhhhlllllhhhhhhhlll


DSSP  hhhhhhhhhllllhHHHHHHHHHHHHHHHHHHLL-LLLLLLLLEEEEHHHHHHHhhlllll
Query drltqvaerqgvvfGRLQRHVAXIVVAQFAATL-TDDTGAPLLLVKGGSSLELrrgipds   74
ident                            |             |    |
Sbjct ttqqaiglkhysdfLERMPREEATEIEQTVQKAaQAFNSGLLCVACGSYRRGK-------  173
DSSP  lhhhhhhhhlhhhhHLLEEHHHHHHHHHHHHHHHhHLLLLLLEEEELHHHHLLL-------


DSSP  lLLLLEEEEEL--------lLHHHHHHHHHHHHhhlllleeeEELLL-EELLLLLlLLLL
Query rTSKDFATVAR--------rDIELIHEQLADAGetgwegftaIFTAP-EEIAAPGxPVKP  125
ident  |  |                       |   |           |         |
Sbjct aTCGDVDVLIThpdgrshrgIFSRLLDSLRQEG---------FLTDDlVSQEENG-QQQK  223
DSSP  lEELLEEEEEEllllllllLHHHHHHHHHHLLL---------LEEEEeEEEEEEL-LEEE


DSSP  EEEEEEEEEllEEEEEEEEEEEEL-LLLLllllleeeeelhhhhllllllleeeeellhHHH
Query RRFTAKLSYrgRAFATVPIEVSSV-EAGNadqfdtltsdalglvgvpaavavpcxtiPWQ  184
ident       |                |
```

```
DSSP  Hhhhhhhhhlllllllllllllllllll
```

```
DSSP  HhhhhhhhhlllllllllllHHHHHHH-----------------------hhhhhllhh
Query IaqklhavtavleepkandraHDLVDLQ-----------------------lleglllda  221
ident
Sbjct L-----------------yftgSAHFNRSmralaktkgmslsehalstavvrnthgckvgp  298
DSSP  H-----------------hhhlLHHHHHHhhhhhhhlleeellllleeellllllllllll
```

```
DSSP  hhhhhHHHHHhhhhhhllllLLLLLlllllhhhhhhhhhllllllllllhhhhhhhhhh
Query dlxptRSACIaifearaqhpWPPRVatlphwpliyagalegldhlelartvdaaaqavqr  281
ident
Sbjct grvlpTPTEK-------dvfRLLGL-----------------------------------  316
DSSP  lllllLLLHH-------hhhHHHLL-----------------------------------
```

```
DSSP  hhhhhhhllll
Query fvaridratkr  292
ident
Sbjct -pyrepaerdw  326
DSSP  -llllhhhlll
```

No 533: Query=mol1A Sbjct=5f2v0 Z-score=5.0

back to top

```
DSSP  llllllllllhhhhhhhhhhhhhHLLLLHHHHHHHHHHHHHHHHHHHHLL--LLLL--LLLL
Query aapysspptnlrslrdrltqvaeRQGVVFGRLQRHVAXIVVAQFAATL--TDDT--GAPL   56
ident                                     |
Sbjct -----------------------DKQWERFLVPYRQAVEELKVKLKGIrtLYEYedDHSP   37
DSSP  -----------------------LLLHHHHHHHHHHHHHHHHHHHHHHHHHhhHHHHllLLLL
```

```
DSSP  -EEEEhhHHHHhhhlLLLLL-----------------lLLLEEEEELL--LHHHHHHHH
Query -LLVKggSSLElrrgIPDSR-----------------tSKDFATVARR--DIELIHEQL   95
ident    |              |                                ||    | |
Sbjct iEFVT--GRVK----PVASIlekarrksiplheietmqdIAGLRIMCQFvdDIQIVKEML   91
DSSP  lLEEE--EEEL----LHHHHhhhhhhllllhhhllllllLEEEEEEELLhhHHHHHHHHH
```

```
DSSP  HHHHhhllllеEEEELLleellllllLLLLEEEEEEEEEL---lEEEEEEEEEEEEELLll
Query ADAGetgwegfTAIFTApeeiaapgxPVKPRRFTAKLSYR---gRAFATVPIEVSSVEag  152
ident                                                  | |
Sbjct FARK-------DFTVVD---------GYRSYHLVVLYPLQtvsgEKHVLVEIQIRTLA--  133
DSSP  HHLL-------LLEEEL---------LLLEEEEEEEEEEEelleEEEEEEEEEEEEEHH--
```

```
DSSP  llllleeeeelhhhhllllllleeeeellhhhhhHHHHHHHHLlllllllllllhhhhhhh
Query nadqfdtltsdalglvgvpaavavpcxtipwqiAQKLHAVTAvleepkandrahdlvdlq  212
ident
Sbjct ------------------------------mnfWATIEHSLN------------------  145
DSSP  ------------------------------hhhHHHHHHHHHH------------------
```

```
DSSP  hhhhhllhhhhhhhhhhhhhhhhhhhllllllLLLLLLLHhhhhhhhhlllllllllllh
Query lleglllddadlxptrsaciaifearaqhpwppRVATLPHWpliyagalegldhlelartv  272
ident
Sbjct -----------------------------ykySGNIPEKV------------klrlqras  164
DSSP  -----------------------------hhlLLLLLLHHH------------hhhhhhhh
```

```
DSSP  hhhhhhhhhhhhhhhhhllll
Query daaaqavqrfvaridratkr  292
ident
Sbjct eaasrldeemseirgevqea  184
DSSP  hhhhhhhhhhhhhhhhhhll
```

No 534: Query=mol1A Sbjct=5d46A Z-score=5.0

back to top

```
DSSP  ------------------------------------------------------------
```

```
Sbjct kisqyacqrrttlnnynqlftdaldilaendelrenegsclafmrassvlkslpfpitsm   60
DSSP  lllllhhhlllllllllhhhhhhhhhhhhhhhhlllhhhhhhhhhhhhhhllllllllh


DSSP  ------------------------------------------------------------
Query ------------------------------------------------------------    0
ident
Sbjct kdtegipclgdkvksiiegiiedgesseakavlnderyksfklftsvfgvglktaekwfr  120
DSSP  hhhlllllllhhhhhhhhhhhhhlllhhhhhhlllhhhhhhhhhhllllllhhhhhhhhh


DSSP  --llllllllllhhhhhhhhhhhhhhllllhhhhHHHHHHHHHHHHHHL-LLLLlLLLLE
Query --aapysspptnlrslrdrltqvaerqgvvfgrlQRHVAXIVVAQFAAT-LTDDtGAPLL   57
ident                                           |               |
Sbjct mgfrtlskiqsdkslrftqmqkagflyyedlvscVNRPEAEAVSMLVKEaVVTFlPDALV  180
DSSP  lllllhhhhhllllllllhhhhhhhhlhhhhhllEEHHHHHHHHHHHHHhHHHHlLLLEE


DSSP  EEEHHHHHHHhhllllllLLLLEEEEEL---------lLHHHHHHHHHHHHhhlllleee
Query LVKGGSSLELrrgipdsrTSKDFATVAR---------rDIELIHEQLADAGetgwegfta  108
ident    ||             |  |                            |
Sbjct TMTGGFRRGK-------mTGHDVDFLITspatedeeqqLLHKVTDFWKQQG---------  224
DSSP  EEELLLLLLL-------lEELLEEEEEEllllhhhhhhHHHHHHHHHHHLL---------


DSSP  EELLLEELLL------lLLLLLLEEEEEEEEEL---lEEEEEEEEEEEEL-LLLLlllle
Query IFTAPEEIAA------pGXPVKPRRFTAKLSYR---gRAFATVPIEVSSV-EAGNadqfd  158
ident                             ||
Sbjct LLLYCDILESpsrkvdaLDHFQKCFLILKLDHGrvhgKGWKAIRVDLVMCpYDRR-----  279
DSSP  LEEELLEELLlllllllLLLLEEEEEEEELLHHhlllLLLLEEEEEEEELlHHHH-----


DSSP  eeeelhhhhlllllleeeeellhHHHHhhhhhhhhllllllllllllHHHHHHH------
Query tltsdalglvgvpaavavpcxtiPWQIaqklhavtavleepkandraHDLVDLQ------  212
ident
Sbjct ----------------------aFALL----------------gwtgSRQFERDlrryat  301
DSSP  ----------------------hHHHH----------------hhhlLHHHHHHhhhhhh


DSSP  --------hhhhllhhhhhhhhHHHHHhhhhhhllllLLLLLLllllhhhhhhhhhllll
Query --------llegllldadlxptRSACIaifearaqhpWPPRVAtlphwpliyagalegld  264
ident
Sbjct herkmmldnhalydrtkrvfleAESEE-------eifAHLGLD-----------------  337
DSSP  hhhleeelllleeelllleeelLLLHH-------hhhHHHLLL-----------------


DSSP  llllllhhhhhhhhhhhhhhhhhhllll
Query hlelartvdaaaqavqrfvaridratkr  292
ident
Sbjct -------------------yiepwerna  346
DSSP  -------------------lllhhhlll
```

No 535: Query=mol1A Sbjct=5iioA Z-score=5.0

back to top

```
DSSP  ------------------------------------------------------------
Query ------------------------------------------------------------    0
ident
Sbjct atnhnlhiteklevlakaysvqgdkwralgyakainalksfhkpvtsyqeacsipgigkr   60
DSSP  llllhhhhhhhhhhhhhhhhlllhhhhhhhhhhhhhhhlllllllllhhhhhhlllllhh


DSSP  ----------------------------------------------llllllllllhhhh
Query ----------------------------------------------aapysspptnlrsl   14
ident
Sbjct maekiieilesghlrkldhisesvpvlelfsniwgagtktaqmwyqqgfrsledirsqas  120
DSSP  hhhhhhhhhhlllhhhhhllllhhhhhhhhllllllhhhhhhhhhlllllhhhhhhhhll


DSSP  hhhhhhhhhhllllhHHHHHHHHHHHHHHHHHLL-LLLLLLLEEEEHHHHHHHHhhllll
Query rdrltqvaerqgvvfGRLQRHVAXIVVAQFAATL-TDDTGAPLLLVKGGSSLELrrgipd   73
ident                             |             |    |
Sbjct lttqqaiglkhysdfLERMPREEATEIEQTVQKAaQAFNSGLLCVACGSYRRGK------  174
DSSP  llhhhhhhhlhhhhhHLLEEHHHHHHHHHHHHHHhHLLLLLLEEEELHHHHLLL------
```

```
DSSP  llLLLLEEEEEL--------lLHHHHHHHHHHHHhhlllleeeEELLL-EELLLLLlLLL
Query srTSKDFATVAR--------rDIELIHEQLADAGetgwegftaIFTAP-EEIAAPGxPVK  124
ident   |  |                       |   |           |         |
Sbjct -aTCGDVDVLIThpdgrshrgIFSRLLDSLRQEG---------FLTDDlVSQEENG-QQQ  223
DSSP  -lEELLEEEEEElllllllllHHHHHHHHHHHLL---------LEEEEeEEEEEEL-LEE


DSSP  LEEEEEEEEEllEEEEEEEEEEEEL-LLLLllllleeeeelhhhhllllllleeeeellhHH
Query PRRFTAKLSYrgRAFATVPIEVSSV-EAGNadqfdtltsdalglvgvpaavavpcxtiPW  183
ident        |                |
Sbjct KYLGVCRLPG--PGRRHRRLDIIVVpYSEF---------------------------aCA  254
DSSP  EEEEEELLLL--LLLLLEEEEEEEELlHHHH---------------------------hHH


DSSP  HHhhhhhhhhllllllllllllHHHHHHH-------------------------hhhhhl
Query QIaqklhavtavleepkandraHDLVDLQ-------------------------llegll  218
ident
Sbjct LL----------------yftgSAHFNRSmralaktkgmslsehalstavvrnthgckvg  298
DSSP  HH----------------hhhlLHHHHHHhhhhhhhlleeellllleeelleellllleee


DSSP  lhHHHHhhHHHHHhhhhhhllllLLLLLlllllhhhhhhhhhllllllllllhhhhhhh
Query ldADLXptRSACIaifearaqhpWPPRVatlphwpliyagalegldhlelartvdaaaqa  278
ident     |
Sbjct pgRVLP--TPTEK-------dvfRLLGL--------------------------------  317
DSSP  llLEEL--LLLHH-------hhhHHLLL--------------------------------


DSSP  hhhhhhhhhhllll
Query vqrfvaridratkr  292
ident
Sbjct ----pyrepaerdw  327
DSSP  ----llllhhhlll
```

No 536: Query=mol1A Sbjct=4x4tC Z-score=5.0

back to top

```
DSSP  lllllllllhhhhhhhhhhhhhhllLLHHHHHHHHHHHHHHHHHHHHLLLlllllLLEEEE
Query aapysspptnlrslrdrltqvaerqgVVFGRLQRHVAXIVVAQFAATLTddtgaPLLLVK   60
ident                                     |          |
Sbjct -----------kveeilekalelvipDEEEVRKGREAEEELRRRLDELG-----VEYVFV   44
DSSP  -----------lhhhhhhhhhhllllLHHHHHHHHHHHHHHHHHHHHHHLL-----LLEEEE


DSSP  HHHHHHhhhlllLLLL-lLLEEEEEEL-----------lLHHHHHHHHhhhhhhllllleee
Query GGSSLElrrgipDSRT-sKDFATVAR-----------rDIELIHEQLadagetgwegfta  108
ident |                                         |
Sbjct GSYARN------TWLKgsLEIDVFLLfpeefskeelreRGLEIGKAV-------------   85
DSSP  HHHHHL------LLLLllLLEEEEEEellllllhhhhHHHHHHHHLL-------------


DSSP  eELLLEELLLllllLLLEEEEEEEeeelleeeeEEEEEEEEELLLLlllllleeeeelhhhhl
Query iFTAPEEIAApgxpVKPRRFTAKlsyrgrafaTVPIEVSSVEAGnadqfdtltsdalglv  168
ident         |             |          |   |
Sbjct lDSYEIRYAE----HPYVHGVVK---------GVEVDVVPCYKL--------------ke  118
DSSP  lLEEEEEELL----LEEEEEEEL---------LEEEEEEEEEL--------------ll


DSSP  llllleeeeeLLHHHHHHH---------------hhhhhhllllLLLLLLLLHHHHHHHH
Query gvpaavavpcXTIPWQIAQ---------------klhavtavleePKANDRAHDLVDLQL  213
ident                                               |       |  |
Sbjct pkniksavdrTPFHHKWLEgrikgkenevrllkgflkangiygaeYKVRGFSGYLCELLI  178
DSSP  lllllllllHHHHHHHHLllllllhhhhhhhhhhhhhlllllllLLLLLLLHHHHHHHH


DSSP  HHhhllhhhhhhhhhhhhhhhhhhllllllllllllhhhhhhhhhllllllLLLLLHH
Query LEglllldadlxptrsaciaifearaqhpwpprvatlphwpliyagalegldhleLARTVD  273
ident
Sbjct VF------------------------------------------------ygSFLETV  188
DSSP  HH------------------------------------------------hlLHHHHH


DSSP  H-----hhhhhhHHHHH-----------------------------------------
Query A-----aaqavqRFVAR-----------------------------------------  285
```

```
DSSP  Hhhllllllleee ELLLLeeeellllleeeelleeeeellllllhhhhhhhhhhhhhhhhll
```

```
DSSP  ------------------------------------------------------------
Query ------------------------------------------------------------  285
ident
Sbjct slgffkpkhpleieperlrkiveergtavfavkfrkpdivddnlypqlerasrkifefle  308
DSSP  lhhhhllllllllllhhhhhhhhhhhlleeeeeeeeelllllhhhhhhhhhhhhhhhhhhh
```

```
DSSP  ------------------------------------------------------------
Query ------------------------------------------------------------  285
ident
Sbjct renfmplrsafkaseefcyllfecqikeisrvfrrmgpqfedernvkkflsrnrafrpfi  368
DSSP  hllllleeeeeeeeellleeeeeeeeelllllllleeeeeeeeelllhhhhhhhhhlllllllee
```

```
DSSP  ------------------------------------------------------------
Query ------------------------------------------------------------  285
ident
Sbjct engrwwafemrkfttpeegvrsyasthwhtlgknvgesireyfeiisgeklfkepvtael  428
DSSP  elleeeeeeelllllhhhhhhhhhhhlhhhllhhhhhhhhhlleeeehhhhhhlllhhhh
```

```
DSSP  -hhhllll
Query -idratkr  292
ident
Sbjct cemmgvkd  436
DSSP  hhhhllll
```

No 537: Query=mol1A Sbjct=3ouyA Z-score=5.0

back to top

```
DSSP  lllllllllhhhhhhhhhhhhhhllLLHHHHHHHHH-HHHHHHHHHLLLlllllLLEEE
Query aapysspptnlrslrdrltqvaerqgVVFGRLQRHVA-XIVVAQFAATLTddtgaPLLLV   59
ident                                                 |
Sbjct -----------mkveeilekalelviPDEEEVRKGREaEEELRRRLDELG-----VEYVF   44
DSSP  -----------llhhhhhhhhhlllLLHHHHHHHHHhHHHHHHHHHHHLL-----LLEEE
```

```
DSSP  EHHHHHhhhhlllLLLL--lLLEEEEEL-----------lLHHHHHHHHhhhhhhlllle
Query KGGSSLelrrgipDSRT--sKDFATVAR-----------rDIELIHEQLadagetgwegf  106
ident  |                                          |
Sbjct VGSYAR-------NTWLkgsLEIDVFLLfpeefskeelreRGLEIGKAV-----------   86
DSSP  ELLHHH-------LLLLlllLEEEEEEEelllllhhhhhhHHHHHHHHH-----------
```

```
DSSP  eeeELLLEELLLllllLLLEEEEEEeeelleeeeEEEEEEEEELLLLlllllleeeeelhhh
Query taiFTAPEEIAApgxpVKPRRFTAKlsyrgrafaTVPIEVSSVEAGnadqfdtltsdalg  166
ident           |             |          |   |
Sbjct --lDSYEIRYAE----HPYVHGVVK---------GVEVDVVPCYKL--------------  117
DSSP  --lLEEELLLLL----LLLEEEEEL---------LEEEEEEEEELL--------------
```

```
DSSP  hllllllleeeeeLLHHHHHH-------------------hhhhhhHLLLllllLLLLLHH
Query lvgvpaavavpcXTIPWQIA-------------------qklhavTAVLeepkANDRAHD  207
ident
Sbjct kepkniksavdrTPFHHKWLegrikgkenevrllkgflkangiygAEYK----VRGFSGY  173
DSSP  llllllllllllHHHHHHHHhlllllhhhhhhhhhhhhllllllLLLL----LLLLLHH
```

```
DSSP  HHHHHhhhhllhhhhhhhhhhhhhhhhhhllllllllllllllhhhhhhhhhllllllL
Query LVDLQLleglllldadlxptrsaciaifearaqhpwpprvatlphwpliyagalegldhlE  267
ident |  |
Sbjct LCELLI--------------------------------------------------vfyG  183
DSSP  HHHHHH--------------------------------------------------hhhL
```

```
DSSP  LLL--LHHH---hhhhhHHHHHH-------------------------------------
Query LAR--TVDA---aaqavQRFVAR-------------------------------------  285
ident         |
Sbjct SFLetVKNArrwtrrtvIDVAKGevrkgeeffvvdpvdekrnvaanlsldnlarfvhlcr  243
DSSP  LHHhhHHHHllllllleeEELLLLeeeellllleeeelleeeeellllllhhhhhhhhhhh
```

```
Query ------------------------------------------------------------  285
ident
Sbjct efmeapslgffkpkhpleieperlrkiveergtavfavkfrkpdivddnlypqlerasrk  303
DSSP  hhhhlllhhhhllleellllhhhhhhhhhhhhlleeeeeeeellllhhhhhhhhhhhhhh


DSSP  ------------------------------------------------------------
Query ------------------------------------------------------------  285
ident
Sbjct ifeflerenfmplrsafkaseefcyllfecqikeisrvfrrmgpqfedernvkkflsrnr  363
DSSP  hhhhhhhlllleeeeeeeellleeeeeeeelllllleeeeeeeeelllhhhhhhhlllll


DSSP  ------------------------------------------------------------
Query ------------------------------------------------------------  285
ident
Sbjct afrpfiengrwwafemrkfttpeegvrsyasthwhtlgknvgesireyfeiisgeklfke  423
DSSP  llllleeelleeeeeeeellllhhhhhhhhhhhhhhhhlhhhhhhhhhhlleeeehhhhll


DSSP  -----------hhhllll
Query -----------idratkr  292
ident
Sbjct pvtaelcemmgvkdcccm  441
DSSP  llhhhhhhhhllllleel
```

No 538: Query=mol1A Sbjct=4wc1A Z-score=5.0

back to top

```
DSSP  llllllllllhhhhhhhhhhhhhllllhhhhhhhhhhhhhhhhHHHLLLLLllLLLEEEE
Query aapysspptnlrslrdrltqvaerqgvvfgrlqrhvaxivvaqFAATLTDDtgAPLLLVK   60
ident
Sbjct -------------------------------------------MVGQIAKE-mGLRAYIV   16
DSSP  -------------------------------------------LHHHHHHH-lLLEEEEE


DSSP  HHHHHHHHHLLlllllLLLEEEEEELLLHHHHHHHHHHHHhlllleeeEELLLEElllll
Query GGSSLELRRGIpdsrtSKDFATVARRDIELIHEQLADAGEtgwegftaIFTAPEEiaapg  120
ident ||       |        |   |           ||                |
Sbjct GGVVRDILLGK----eVWDVDFVVEGNAIELAKELARRHG--------VNVHPFP-----   59
DSSP  LHHHHHHHLLL----lLLEEEEEEEELLHHHHHHHHHHHL--------LLEEEEL-----


DSSP  llLLLEEEEEEeeelleeeeEEEEEEEELLL--LLLL--------------------lLE
Query xpVKPRRFTAKlsyrgrafaTVPIEVSSVEA--GNAD--------------------qFD  158
ident                         |
Sbjct -eFGTAHLKIG---------KLKLEFATARRepASLKedlirrdftinamaisvnledYG  109
DSSP  -lLLEEEEEEL---------LEEEEEEEELLllLLHHhhhhllllhhhlleeelllllLL


DSSP  EEeelhhhhllllllleeeeelLHHH---------hhhhhhhhHHLLllllllLLLLhhhh
Query TLtsdalglvgvpaavavpcxTIPW---------qiaqklhaVTAVleepkaNDRAhdlv  209
ident ||                                                    |
Sbjct TL-------------------IDYFgglrdlkdkvirvlhpvSFIE-----dPVRI----  141
DSSP  LE-------------------ELLLlllhhhhlleellllllHHHH-----lLLHH----


DSSP  hhhhhhhllhhhhhhhhhhhhhhhhhhhhlllllllllllllhhhHHHHHHLLLLlllll
Query dlqllegllldadlxptrsaciaifearaqhpwpprvatlphwplIYAGALEGLDhlela  269
ident                                                      |
Sbjct --------------------------------------------lRALRFAGRLN----f  153
DSSP  --------------------------------------------hHHHHHHHHLL----l


DSSP  LLHHHHHHH---------------------------------------------------
Query RTVDAAAQA---------------------------------------------------  278
ident
Sbjct KLSRSTEKLlkqavnlgllkeaprgrlineiklalredrfleilelyrkyrvleeiiegf  213
DSSP  EELHHHHHHhhhhhhllhhhhllhhhhhhhhhhhllllhhhhhhhhhhhhlhhhhlllll


DSSP  ------------------------------------------------------------
Query ------------------------------------------------------------  278
ident
```

```
DSSP  ------------------------------------------------------------
Query ------------------------------------------------------------  278
ident
Sbjct vretykfmkfklgslkeelkkakenyevyrllkplhtsvllllmleeelkekiklylekl  333
DSSP  hhhhhhhhhllhhhhhhhhhllllhhhhhhhhllllhhhhhhhlllhhhhhhhhhhhhlh


DSSP  -----------------hhhhhhhhhhllll
Query -----------------vqrfvaridratkr  292
ident
Sbjct rkvklpkekieelkkelgerieelkreimnk  364
DSSP  hhlllllhhhhhhhllhhhhhhhhhhhhhll
```

No 539: Query=mol1A Sbjct=3ifqA Z-score=5.0

back to top

```
DSSP  -----------------------------------------------------------L
Query -----------------------------------------------------------A    1
ident
Sbjct aivhlinyqddaelatralpeltkllndedpvvvtkaamivnqlskkeasrralmgspqL   60
DSSP  lhhhhhlhhhhhhhhhhlhhhhhhhhllllhhhhhhhhhhhhhhlllhhhhhhhhllhhH


DSSP  LLL----llLLLLHHHHHHHHHHHHHHLL---------llhHHHHHHHHHHHH-------
Query APY----ssPPTNLRSLRDRLTQVAERQG---------vvfGRLQRHVAXIVV-------   41
ident              |   |                       |
Sbjct VAAvvrtmqNTSDLDTARCTTSILHNLSHhregllaifksgGIPALVRMLSSPvesvlfy  120
DSSP  HHHhhhhhlLLLLHHHHHHHHHHHHHHLLlhhhhhhhhhllHHHHHHHHHHLLlhhhhhh


DSSP  -------------hhhhhllllllllllleEEEH--------HHHHHHHHLllllllllle
Query -------------aqfaatltddtgapllLVKG--------GSSLELRRGipdsrtskdf   80
ident                                               |
Sbjct aittlhnlllyqegakmavrladglqkmvPLLNknnpkflaITTDCLQLL----------  170
DSSP  hhhhhhhhhhhlllhhhhhhhllhhhhlhHHHHlllhhhhhHHHHHHHHH----------


DSSP  eeeelllhhhhhhhhhhhhhhlllleeeeellleelllllllllleeeeeeeeelleeee
Query atvarrdieliheqladagetgwegftaiftapeeiaapgxpvkprrftaklsyrgrafa  140
ident
Sbjct ------------------------------------------------------------  170
DSSP  ------------------------------------------------------------


DSSP  eeeeeeeellllllllleeeeelhhhhllllllleeeeeLLHHHHHHHHHHHHhllllll
Query tvpievssveagnadqfdtltsdalglvgvpaavavpcXTIPWQIAQKLHAVtavleepk  200
ident                                          |    |
Sbjct -------------------------aygnqeskliilaNGGPQALVQIMRNY--------  197
DSSP  -------------------------hlllhhhhhhhhhLLHHHHHHHHHHHL--------


DSSP  lllLLHHHHHHHHHHHHL--LHHHHHHHHHHHhhhhhhhllllllLLLLLlllhhhhhhh
Query andRAHDLVDLQLLEGLL--LDADLXPTRSACiaifearaqhpwpPRVATlphwpliyag  258
ident        |         |            |                  |
Sbjct --sYEKLLWTTSRVLKVLsvCPSNKPAIVEAG--------gmqalGKHLT----------  237
DSSP  --lLHHHHHHHHHHHHHHllLLLHHHHHHHLL--------hhhhhHHHHH----------


DSSP  hhllllllllllLHHHHHHHHHHHHHHHHHLLLL--------------------------
Query alegldhlelarTVDAAAQAVQRFVARIDRATKR--------------------------  292
ident                   |
Sbjct -----------sNSPRLVQNCLWTLRNLSDVATKqeglesvlkilvnqlsvddvnvltca  286
DSSP  -----------lLLHHHHHHHHHHHHHHHHLLLLllllhhhhhhhhllllllllhhhhhhh


DSSP  ------------------------------------------------------------
Query ------------------------------------------------------------  292
ident
Sbjct tgtlsnltcnnsknktlvtqnsgvealihailragdkdditepavcalrhltsrhpeaem  346
DSSP  hhhhhhhllllhhhhhhhllllhhhhhhhhhhhhlllhhhhhhhhhhhhhhllllllhhh
```

```
ident
Sbjct aqnsvrlnygipaivkllnqpnqwplvkatiglirnlalcpanhaplqeaaviprlvqll  406
DSSP  hhhhhhlllhhhhhhhhhhllllhhhhhhhhhhhhhhlllhhhhhhhhhllhhhhhhhhh


DSSP  ------------------------------------------------------------
Query ------------------------------------------------------------  292
ident
Sbjct vkahqdaqrhvaagtqqpytdgvrmeeivegctgalhilardpmnrmeifrlntiplfvq  466
DSSP  hhhhhhhhhhhllllllllllhhhhhhhhhhhhhhlllhhhhhhhhhlllhhhhhh


DSSP  ------------------------------------------------------------
Query ------------------------------------------------------------  292
ident
Sbjct llyssveniqrvaagvlcelaqdkeaadaidaegasaplmellhsrnegtatyaaavlfr  526
DSSP  hllllhhhhhhhhhhhhhhlllhhhhhhhhhlllhhhhhhhlllllhhhhhhhhhhhh


DSSP  --------------------
Query --------------------  292
ident
Sbjct isedknprkrvsveltnslf  546
DSSP  llllllllhhhhhhllllll
```

No 540: Query=mol1A Sbjct=4at8C Z-score=4.9

back to top

```
DSSP  ------------llllllllllhhhhhhhhhhhhhhlLLLHHHHHHHHHHHHHHHHHHLL
Query ------------aapysspptnlrslrdrltqvaerqGVVFGRLQRHVAXIVVAQFAATL   48
ident                                                            |
Sbjct hipfdfylcemafprvkpapdetsfseallkrnqdlaPNSAEQASILSLVTKINNVIDNL   60
DSSP  lllllhhhlllllllllllllhhhhhhhhhhhhhhlLLHHHHHHHHHHHHHHHHHHHHHH


DSSP  L--LLLLlLLEEEEHHhhhhhhhlllLLLL-----lLLEEEEEL--LLHHHHHHHHHHHH
Query T--DDTGaPLLLVKGGsslelrrgipDSRT-----sKDFATVAR--RDIELIHEQLADAG   99
ident      |      |                        |           |
Sbjct IvaPGTFvQIEEVRQV------gsykKGTMttghnvADLVVILKilPTLEAVAALGNKVV  114
DSSP  HhlLLLLlLEEEEEEE------lhhhHLLLlllleEEEEEEELllLLHHHHHHHHHHH


DSSP  HH-LLLL--eeEEELLLEellllllllllEEEEEEEeelleEEEEEEEEEEELLLLLlll
Query ET-GWEG--ftAIFTAPEeiaapgxpvkpRRFTAKLsyrgrAFATVPIEVSSVEAGNadq  156
ident |                |             |           ||| |    |
Sbjct ESlRAQDpsevLTMLTNE-----------TGFEISS-----SDATVKILITTVPPNL---  155
DSSP  HHhHHHLllllLEEEELL-----------LEEEEEL-----LLLEEEEEEEEELHHHH---


DSSP  leeeeelhhhhllllllleeeeellhhhhhhHHHHHHhlllllllLLLLLhhhhhhhhhh
Query fdtltsdalglvgvpaavavpcxtipwqiaQKLHAVtavleepkaNDRAhdlvdlqlleg  216
ident
Sbjct ----------rkldpelhldikvlqsalaaIRHARW-------feENAS-----------  187
DSSP  ----------llllllllllhhhhhhhhhhHHHHHH-------hhHHLL-----------


DSSP  hllhhhhhhhhhhhhhhhhhhllllllllllllhhHHHHHHHLLL-LLLL--------
Query llldadlxptrsaciaifearaqhpwpprvatlphwpLIYAGALEGL-DHLE--------  267
ident                                            |  |   |
Sbjct -------------------------------------QSTVKVLIRLlKDLRirfpgfep  210
DSSP  -------------------------------------LHHHHHHHHHhHHHHhhlhhhll


DSSP  ---------lllLHHH--------------------------hhhhhHHHHhhHHHL---
Query ---------larTVDA--------------------------aaqavQRFVarIDRA---  289
ident              |
Sbjct ltpwildllghyAVMNnptrqplalnvayrrclqilaaglflpgsvgITDP-cESGNfrv  269
DSSP  llhhhhhhhhhHHHLllllllllhhhhhhhhhhhhhllllllllllLLLL-lLLLLllh


DSSP  ------------------------------------------------------------
Query ------------------------------------------------------------  289
ident
Sbjct htvmtleqqdmvcytaqtlvrilshggfrkilgqegdasylaseistwdgvivtpsekay  329
```

```
DSSP  lll
Query tkr  292
ident
Sbjct ekp  332
DSSP  lll
```

No 541: Query=mol1A Sbjct=5deeZ Z-score=4.9

back to top

```
DSSP  llllllllllhhhhhhhhhhhhhhlLLLHHHHHHHHHHHHHHHHHHHLL--LLLL--LLLL
Query aapyssspptnlrslrdrltqvaerqGVVFGRLQRHVAXIVVAQFAATL--TDDT--GAPL   56
ident                                     |
Sbjct -------------------------QWERFLVPYRQAVEELKVKLKGIrtLYEYedDHSP   35
DSSP  -------------------------LHHHHLHHHHHHHHHHHHHHHHHHHhhHHLLllLLLL


DSSP  -EEEEhhHHHHhhhlLLLLL------------------lLLLEEEEELL--LHHHHHHHH
Query -LLVKggSSLElrrgIPDSR------------------tSKDFATVARR--DIELIHEQL   95
ident    |              |                                ||    | |
Sbjct iEFVT--GRVK----PVASIlekarrksiplheietmqdIAGLRIMCQFvdDIQIVKEML   89
DSSP  lLEEE--EEEL----LHHHHhhhhhhlllllllhhhlllLEEEEEEEELLhhHHHHHHHHH


DSSP  HHHHhhlllleEEEELlleellllllllLLEEEEEEEEEL---lEEEEEEEEEEEEELLll
Query ADAGetgwegfTAIFTapeeiaapgxpvKPRRFTAKLSYR---gRAFATVPIEVSSVEag  152
ident                                                  | |
Sbjct FARK-------DFTVV------------RSYHLVVLYPLQtvsgEKHVLVEIQIRTLA--  128
DSSP  HLLL-------LLEEL------------LEEEEEEEEEEEelleEEEEEEEEEEEEEHH--


DSSP  llllleeeeelhhhhllllleeeeellhhhhhHHHHHHHHLllllllllllhhhhhhh
Query nadqfdtltsdalglvgvpaavavpcxtipwqiAQKLHAVTAvleepkandrahdlvdlq  212
ident
Sbjct ------------------------------mnfWATIEHSLN------------------  140
DSSP  ------------------------------hhhHHHHHHHHH------------------


DSSP  hhhhhllhhhhhhhhhhhhhhhhhhlllllllLLLLLLHhhhhhhhhlllllllllllh
Query lleglllldadlxptrsaciaifearaqhpwpprVATLPHWpliyagalegldhlelartv  272
ident
Sbjct -----------------------------ykysGNIPEKV------------klrlqras  159
DSSP  -----------------------------hhllLLLLHHH------------hhhhhhhh


DSSP  hhhhhhhhhhhhhhhhllll
Query daaaqavqrfvaridratkr  292
ident
Sbjct eaasrldeemseirgevqea  179
DSSP  hhhhhhhhhhhhhhhhhhll
```

No 542: Query=mol1A Sbjct=3hj1A Z-score=4.9

back to top

```
DSSP  llllllllllhhhhhhhhhhhhhhllLLHHHHHHHHHHHHHHHHHHHHHLLllllllLLEEEE
Query aapyssspptnlrslrdrltqvaerqgVVFGRLQRHVAXIVVAQFAATLtddtgaPLLLVK   60
ident                                   |     |                 |
Sbjct ---ghmnvakrefirgmmahyraslpPPEHSVVIHELQKRVLDIGMLA-----vNKAHVE   52
DSSP  ---llllhhhhhhhhhhhhhhhhhllLLLHHHHHHHHHHHHHHHHHHH-----lLLLEEE


DSSP  HHhhhhhhhllllllllLLEEEEEL-------------lLHHHHHHHHHHHHHHLlllEE
Query GGsslelrrgipdsrtsKDFATVAR-------------rDIELIHEQLADAGETGwegFT  107
ident                   |     |
Sbjct LF--gshvsgfctphsdADISLTYRnfspwlqgmervdeQNNKRMTRFGKEASAM---GM  107
DSSP  EE--lhhhhllllllllEEEEEELLlllhhhlllhhhhhHHHHHHHHHHHHHHHH---LL


DSSP  EEELLLEEllllllllLLEEEEEEEEEElleEEEEEEEEEEEellllllllleeeeelhhhh
Query AIFTAPEEiaapgxpvKPRRFTAKLSYrgrAFATVPIEVSsveagnadqfdtltsdalgl  167
```

```
DSSP  EEEEEELL--------LLLEEEEELLL---LLLEEEEEEL--------------------


DSSP  llllleeeeellhhhhhhhhhhhhhhllllllllLLLLhhhhhhhhhhhhllhhhhhhhh
Query vgvpaavavpcxtipwqiaqklhavtavleepkaNDRAhdlvdlqllegllldadlxptr  227
ident
Sbjct ------------------------niggvenskiLCAI----------------------  150
DSSP  ------------------------lhhhhhhhhhHHHH----------------------


DSSP  hhhhhhhhhllllllllllllllLHHHhHHHH-HLLLLLLL------------------l
Query saciaifearaqhpwpprvatlpHWPLiYAGA-LEGLDHLE------------------l  268
ident                               ||
Sbjct ----------------------rQVFPdFYGAyIHLVKAWGkareviaperstfnsftvt  188
DSSP  ----------------------hHLLHhHHHHhHHHHHHHHhhlllllhhhllllhhhhh


DSSP  llLHHHHHH---------------------------------------------------
Query arTVDAAAQ---------------------------------------------------  277
ident
Sbjct tmALMVLQElgllpvfskptgefgeltvadaemllqefklppiydslhdddeklgeavff  248
DSSP  hhHHHHHHHhllllllllllllllllhhhhhhhhhhllllhhhhhllllhhhhhhhhhh


DSSP  -------------------hHHHHHHHhhHLLL---------------------------
Query -------------------aVQRFVARidRATK---------------------------  291
ident
Sbjct clqrfaeyyakydfsagtvsLIHPRRHrtVYERvvrrhlellgsrkrlewekhiaehked  308
DSSP  hhhhhhhhhhhlllllllllLLLLLLLhhHHHHhhhhhhhhhhhhhhhhhhhhhhhlhhh


DSSP  ------------------------------------------------------------
Query ------------------------------------------------------------  291
ident
Sbjct gpldendfsasmqnettqrpsnspyvvedfvnyvncgrrvqasrvrhiqqefnrlremli  368
DSSP  llllhhhhhhhhhhhhhlllllllllllllllllllllllhhhhhhhhhhhhhhhhhhhl


DSSP  ---------------l
Query ---------------r  292
ident
Sbjct dkeselkfdevfresd  384
DSSP  llhhhllhhhhlllll
```

No 543: Query=mol1A Sbjct=4k99A Z-score=4.9

back to top

```
DSSP  llllllllllhhhhhhhhhhhhhhlLLLHHHHHHHHHHHHHHHHHHLLLLLLL-lLLEEE
Query aapysspptnlrslrdrltqvaerqGVVFGRLQRHVAXIVVAQFAATLTDDTG-aPLLLV   59
ident                                        ||
Sbjct --------------klkkvldklrlKRKDISEAAETVNKVVERLLRRMQKRESefKGVEQ   46
DSSP  --------------lhhhhhhhhllLHHHHHHHHHHHHHHHHHHHHHHHLLLLllLLLEE


DSSP  EHHhhhhhhhllllllllLLEEEEEL----------------------------------
Query KGGsslelrrgipdsrtsKDFATVAR----------------------------------   85
ident                    |
Sbjct LNT-gsyyehvkisapneFDVMFKLEvprielqeyyetgafylvkfkgnplshflegevl  105
DSSP  EEE-hhhhhllllllllleEEEEEEEElleeeeeelllllleeeeeelllhhhhhheellee


DSSP  ---lLHHHHHHHHHHHHHHLlLLEEEEELLLEellllllllllEEEEEEEEellEEEEEE
Query ---rDIELIHEQLADAGETGwEGFTAIFTAPEeiaapgxpvkpRRFTAKLSyrgRAFATV  142
ident                                               |            |
Sbjct satkMLSKFRKIIKEEVKEI-KDIDVSVEKEK--------pgsPAVTLLIR--nPEEISV  154
DSSP  lhhhHHHHHHHHHHHHHHHL-LLLEEEEELLL--------lllLEEEEEEE--lLLEEEE


DSSP  EEEEEELLLL-------------------llllleeeeelhhhhllllleeeeeellhHH
Query PIEVSSVEAG-------------------nadqfdtltsdalglvgvpaavavpcxtiPW  183
ident  |       |
Sbjct DIILALESKGswpistkeglpiqgwlgtkvrtnlrrepfylvpknakdgnsfqgetwrLS  214
DSSP  EEEEEEEELLlllhhhllllllllllllhhhhhhhllleeeellllleelleellllleeEE
```

```
Query QIaQKLHAVtavleepkaNDRAhdlvdlqlleglllddadlxptrsaciaifearaqhpwp  243
ident
Sbjct FS-HTEKYI---------LNNH--------------------------------------  226
DSSP  LH-HHHHHH---------HHLL--------------------------------------
```

```
DSSP  llllLLLL-----hHHHHHHHHL-lLLLL---------------llllllhhHHHH-----
Query prvaTLPH-----wPLIYAGALE-gLDHL---------------elartvdAAAQ-----  277
ident
Sbjct -gieKTCCessgakCCRKECLKLmkYLLEqlkkefqeldafcsyhvktaifHMWTqdpqd  285
DSSP  -lllLLLLllllllLLHHHHHHHhhHHHHhhhhhlhhhllllhhhhhhhhhHHHHhlllh
```

```
DSSP  -----------------------------hhhhhhhHHHH--------------------
Query -----------------------------avqrfvaRIDR--------------------  288
ident
Sbjct sqwdprnlsscfdkllaffleclrtekldhyfipkfNLFSqelidrkskeflskkieyer  345
DSSP  hhhlhhhhhhhhhhhhhhhhhhhhhllllllllllllLLLLllllllhhhhhhhhhhhhhh
```

```
DSSP  -----llll
Query -----atkr  292
ident
Sbjct nngfpifdk  354
DSSP  hlllhhhhl
```

No 544: Query=mol1A Sbjct=5deeW Z-score=4.9

back to top

```
DSSP  llllllllllhhhhhhhhhhhhhHLLLLHHHHHHHHHHHHHHHHHHHHLL--LLLL--LLLL
Query aapyssspptnlrslrdrltqvaeRQGVVFGRLQRHVAXIVVAQFAATL--TDDT--GAPL   56
ident                                     |
Sbjct -----------------------DKQWERFLVPYRQAVEELKVKLKGIrtLYEYedDHSP   37
DSSP  -----------------------LLHHHHHHHHHHHHHHHHHHHHHHHHHhhHHLLlLLLL
```

```
DSSP  -EEEEhhHHHHhhhlLLLLL------------------lLLLEEEEELL--LHHHHHHHH
Query -LLVKggSSLElrrgIPDSR------------------tSKDFATVARR--DIELIHEQL   95
ident    |              |                                ||    | |
Sbjct iEFVT--GRVK----PVASIlekarrksiplheietmqdIAGLRIMCQFvdDIQIVKEML   91
DSSP  lLLEE--EEEL----LHHHHhhhhhllllhhhhhhlllLEEEEEEELLhhHHHHHHHHH
```

```
DSSP  HHHHhhlllleEEEELLLEELllllllllLEEEEEEEEEL---lEEEEEEEEEEEEELLll
Query ADAGetgwegfTAIFTAPEEIaapgxpvkPRRFTAKLSYR---gRAFATVPIEVSSVEag  152
ident                                                  | |
Sbjct FARK-------DFTVVDQRDY------iaSYHLVVLYPLQtvsgEKHVLVEIQIRTLA--  136
DSSP  HHLL-------LLEEEEELLL------llLEEEEEEEEEEelleEEEEEEEEEEEEEHH--
```

```
DSSP  llllleeeeelhhhhllllllleeeeellhhhhhHHHHHHHHLLllllllllllhhhhhhh
Query nadqfdtltsdalglvgvpaavavpcxtipwqiAQKLHAVTAVleepkandrahdlvdlq  212
ident
Sbjct ------------------------------mnfWATIEHSLNY-----------------  149
DSSP  ------------------------------hhhHHHHHHHHHH-----------------
```

```
DSSP  hhhhhllhhhhhhhhhhhhhhhhhhllllllLLLLLLLHhhhhhhhhlllllllllllh
Query lleglllddadlxptrsaciaifearaqhpwppRVATLPHWpliyagalegldhlelartv  272
ident
Sbjct ------------------------------kySGNIPEKV------------klrlqras  167
DSSP  ------------------------------hlLLLLLLHHH------------hhhhhhhh
```

```
DSSP  hhhhhhhhhhhhhhhhllll
Query daaaqavqrfvaridratkr  292
ident
Sbjct eaasrldeemseirgevqea  187
DSSP  hhhhhhhhhhhhhhhhhlll
```

No 545: Query=mol1A Sbjct=4i27A Z-score=4.9

```
DSSP  ------------------------------------------------------------
Query ------------------------------------------------------------   0
ident
Sbjct avkkisqyacqrrttlnnynqlftdaldilaendelrenegsclafmrassvlkslpfpi  60
DSSP  lllllllhhhlllllllllhhhhhhhhhhhhhhhhlllhhhhhhhhhhhhhhhhllllll


DSSP  ------------------------------------------------------------
Query ------------------------------------------------------------   0
ident
Sbjct tsmkdtegipclgdkvksiiegiiedgesseakavlnderyksfklftsvfgvglktaek 120
DSSP  llhhhhllllllhhhhhhhhhhhhhlllhhhhhhhhllhhhhhhhhhhllllllhhhhhh


DSSP  -----llllllllllhhhhhhhhhhhhhhllllhhhhhHHHHHHHHHHHHHL-LLLLlLL
Query -----aapysspptnlrslrdrltqvaerqgvvfgrlqRHVAXIVVAQFAAT-LTDDtGA  54
ident                                              |
Sbjct wfrmgfrtlskiqsdkslrftqmqkagflyyedlvscvNRPEAEAVSMLVKEaVVTFlPD 180
DSSP  hhhlllllhhhhhhlllllllhhhhhhhhlhhhhhlleEHHHHHHHHHHHHHhHHHHlLL


DSSP  LLEEEEHHHHHHHhhllllllLLLLEEEEEL----------lLHHHHHHHHHHHHhhlll
Query PLLLVKGGSSLELrrgipdsrTSKDFATVAR----------rDIELIHEQLADAGetgwe 104
ident  |    ||             |  |                             |
Sbjct ALVTMTGGFRRGK-------mTGHDVDFLITspeatedeeqqLLHKVTDFWKQQG----- 228
DSSP  LEEEELHHHHLLL-------lEELLEEEEEElllllhhhhhhHHHHHHHHHHHLL-----


DSSP  leeeEELLLLEELLLL---------lLLLLLEEEEEEEE-----------LLEEEEEEEE
Query gftaIFTAPEEIAAPG---------xPVKPRRFTAKLSY-----------RGRAFATVPI 144
ident                                    ||              |
Sbjct ----LLLYCDILESTFekfkqpsaldHFQKCFLILKLDHgrvhseksgqqEGKGWKAIRV 284
DSSP  ----LEEEEEEEELLLllllllllllLLEEEEEEEEEEEHhhlllllllllLLLLEEEEEE


DSSP  EEEEL-LLLLlllleeeeelhhhhlllllleeeeellhHHHHhhhhhhhhllllllllll
Query EVSSV-EAGNadqfdtltsdalglvgvpaavavpcxtiPWQIaqklhavtavleepkand 203
ident
Sbjct DLVMCpYDRR---------------------------aFALL----------------gw 301
DSSP  EEEELlHHHH---------------------------hHHHH----------------hh


DSSP  llHHHHHHH-------------hhhhhllhhhhhhhHHHHhhhhhhhllllLLLLLLlll
Query raHDLVDLQ-------------llegllldadlxptRSACiaifearaqhpWPPRVAtlp 250
ident
Sbjct tgSRQFERDlrryatherkmmldnhalydrtkrvflEAES------eeeifAHLGLD--- 352
DSSP  hlLHHHHHHhhhhhhhhhleeelllleeelllleeeLLLL------hhhhhHHLLLL---


DSSP  lhhhhhhhhhllllllllllhhhhhhhhhhhhhhhhhhllll
Query hwpliyagalegldhlelartvdaaaqavqrfvaridratkr  292
ident
Sbjct ---------------------------------yiepwerna  361
DSSP  ---------------------------------lllhhhlll
```

No 546: Query=mol1A Sbjct=3wfrF Z-score=4.9

back to top

```
DSSP  llllllllllhhhhhhhhhhhhhhllllhhhhhhhhhhHHHHHHHHLLLLllLLLLEEEE
Query aapysspptnlrslrdrltqvaerqgvvfgrlqrhvaxIVVAQFAATLTDdtGAPLLLVK  60
ident                                              |
Sbjct -------------------------------xxxlnfyLSYFDDVAKVLP--REHYCFIV  27
DSSP  -------------------------------lllllllLLLHHHHHHHHL--LLLLEEEE


DSSP  HHHHHHHHHLLLlllllLLEEEEEELLLHHHHHHHHHHHhhlllleeeEELLLEELllll
Query GGSSLELRRGIPdsrtsKDFATVARRDIELIHEQLADAGetgwegftaIFTAPEEIaapg 120
ident ||       | |      |       |        |
Sbjct GGWVRDRILGEP-vgynIDVDFLTTADPVELAKNFAKRI---------GGHFFVFE----  73
DSSP  LHHHHHHHHLLL-llleEEEEEEEELLLHHHHHHHHHHH---------LLEEEEEL----


DSSP  llLLLLEEEEEEeelleEEEEEEEEEEEL-----LLLL---------------lllLEEE
Query xpVKPRRFTAKLsyrgrAFATVPIEVSSV-----EAGN---------------adqFDTL 160
```

```
DSSP  --LLEEEEEEEL-----LLLEEEEEEEEELllllhHHHHhhhhhllllllllleeehHHHL


DSSP  eelhhhhlllllleeeeelLHHH-----------hhhhhhhHHHLlllllllLLLLHhhh
Query tsdalglvgvpaavavpcxTIPW-----------qiaqklhAVTAvleepkaNDRAHdlv  209
ident                                                  |
Sbjct -----------------tiVYDPtggikdleqgllrpvsieNLKR------dPVRVL---  160
DSSP  -----------------llEELLllhhhhhllleellllhhHHHH------lLLHHH---


DSSP  hhhhhhhhllhhhhhhhhhhhhhhhhhhllllllllllllhhhHHHHHHLLLLlLLLL
Query dlqlleglllldadlxptrsaciaifearaqhpwpprvatlphwplIYAGALEGLDhLELA  269
ident                                                        | |
Sbjct ---------------------------------------------RGFRIAIEKN-LQLT  174
DSSP  ---------------------------------------------HHHHHHHHLL-LEEL


DSSP  llhHHHHHHHHH------------------------------------------------
Query rtvDAAAQAVQR------------------------------------------------  281
ident          |
Sbjct ---EDFYEFVKEdprivlksaverithelfkimkektahkvirelyeygvleaiipeigr  231
DSSP  ---HHHHHHHHHlhhhhhhllhhhhhhhhhhhhhlllhhhhhhhhhhllhhhhhlhhhhh


DSSP  ------------------------------------------------------------
Query ------------------------------------------------------------  281
ident
Sbjct lrevkdpldehtlktleyleqviedrakylsaellenfgkkrvlgeftdvellkwgalfh  291
DSSP  hllllllhhhhhhhhhhhhhhhhhhlhhhhllllllllllllllllhhhhhhhhhhll


DSSP  ------------------------------------------------------------
Query ------------------------------------------------------------  281
ident
Sbjct digkpqtfavtfyehdkvgaqivreigerlrwgdeatefvaklvrhhlrpfflreafkkg  351
DSSP  lllllllllllllhhhhhhhhhhhhhhhllllhhhhhhhhhhhhlllhhhhhhhhhhll


DSSP  ------------------------------------------------------------
Query ------------------------------------------------------------  281
ident
Sbjct elkrrgmanfwrecgdiaphlfllsiadamasgdeeedikalmetiaelesfnrnemkxx  411
DSSP  lllhhhhhhhhhllllhhhhhhhhhhhhhhllllhhhhhhhhhhhhhhhhhhhhllllll


DSSP  -----------------hhhhhhhllll
Query -----------------fvaridratkr  292
ident
Sbjct xxxxxxxxxxxxxxxxxxxxxxxxxxxx  439
DSSP  lhhhhhhhhhhhhlllhhhhhhhhhhhl
```

No 547: Query=mol1A Sbjct=1qz7A Z-score=4.9

back to top

```
DSSP  ----------------------------------------------------LLLL----
Query ----------------------------------------------------AAPY----    4
ident
Sbjct yqddaelatraipeltkllndedqvvvnkaavmvhqlskkeasrhaimrspqMVSAivrt   60
DSSP  lllhhhhhhhhhhhhhhllllllllhhhhhhhhhhllllhhhhhhhhllhhHHHHhhhh


DSSP  llLLLLHHHHHHHHHHHHHHLL---------llhHHHHHHHHHHH---------------
Query ssPPTNLRSLRDRLTQVAERQG---------vvfGRLQRHVAXIV---------------   40
ident           |                       |
Sbjct mqNTNDVETARCTAGTLHNLSHhregllaifksgGIPALVKMLGSpvdsvlfyaittlhn  120
DSSP  hhHLLLLLHHHHHHHHHHHHHLLlhhhhhhhhhllHHHHHHHHHHLllhhhhhhhhhhhh


DSSP  -----hhhhhhlllllllllleEEEH--------HHHHHHHHLllllllllleeeeelll
Query -----vaqfaatltddtgapllLVKG--------GSSLELRRGipdsrtskdfatvarrd   87
ident                                      |
Sbjct lllhqegakmavrlagglqkmvALLNktnvkflaITTDCLQIL-----------------  163
DSSP  hhhhlllhhhhhhhhlhhhhhhHHHHlllhhhhhHHHHHHHHH-----------------
```

```
Query ieliheqladagetgwegftaiftapeeiaapgxpvkprrftaklsyrgrafatvpievs  147
ident
Sbjct ------------------------------------------------------------  163
DSSP  ------------------------------------------------------------


DSSP  elllllllleeeeelhhhhllllllleeeeeLLHHHHHHHHHHHHhllllllllllLLHH
Query sveagnadqfdtltsdalglvgvpaavavpcXTIPWQIAQKLHAVtavleepkandRAHD  207
ident                                   |
Sbjct ------------------aygnqeskliilaSGGPQALVNIMRTY----------tYEKL  195
DSSP  ------------------hlllhhhhhhhhhLLHHHHHHHHHLLL----------lLHHH


DSSP  HHHHHHHHHHL--LHHHHHHHHHHHhhhhhhhllllllLLLLLlllhhhhhhhhhlllll
Query LVDLQLLEGLL--LDADLXPTRSACiaifearaqhpwpPRVATlphwpliyagalegldh  265
ident |         |            |                  |
Sbjct LWTTSRVLKVLsvCSSNKPAIVEAG--------gmqalGLHLT-----------------  230
DSSP  HHHHHHHHHHHhlLLLHHHHHHHHL--------hhhhhHHHLL-----------------


DSSP  lllllLHHHHHHHHHHHHHHHHHLLLL---------------------------------
Query lelarTVDAAAQAVQRFVARIDRATKR---------------------------------  292
ident            |           |
Sbjct ----dPSQRLVQNCLWTLRNLSDAATKqegmegllgtlvqllgsddinvvtcaagilsnl  286
DSSP  ----lLLHHHHHHHHHHHHHHHHHLLLllllhhhhhhhhhhlllllhhhhhhhhhhhhhh


DSSP  ------------------------------------------------------------
Query ------------------------------------------------------------  292
ident
Sbjct tcnnyknkmmvcqvggiealvrtvlragdreditepaicalrhltsrhqeaemaqnavrl  346
DSSP  llllhhhhhhhhhllhhhhhhhhhhhhlllhhhhhhhhhhhhhhhlllllhhhhhhhhhh


DSSP  ------------------------------------------------------------
Query ------------------------------------------------------------  292
ident
Sbjct hyglpvvvkllhppshwplikatvglirnlalcpanhaplreqgaiprlvqllvrahqdt  406
DSSP  lllhhhhhhlllllllhhhhhhhhhhhhhhlllhhhhhhhhhllhhhhhhhhhhhhhhhh


DSSP  ------------------------------------------------------------
Query ------------------------------------------------------------  292
ident
Sbjct qrrtsmggtqqqfvegvrmeeivegctgalhilardvhnrivirglntiplfvqllyspi  466
DSSP  hllllllllllllllllhhhhhhhhhhhhhhhlllhhhhhhhhhlllhhhhhhhhhlll


DSSP  ----------------------------------------------------------
Query ----------------------------------------------------------  292
ident
Sbjct eniqrvaagvlcelaqdkeaaeaieaegatapltellhsrnegvatyaaavlfrmsed  524
DSSP  hhhhhhhhhhhhhhlllhhhhhhhhhlllhhhhhhhlllllhhhhhhhhhhhhhhhll
```

No 548: Query=mol1A Sbjct=1th1B Z-score=4.9

back to top

```
DSSP  ------------------------------------------------LLLL----llLL
Query ------------------------------------------------AAPY----ssPP    8
ident
Sbjct aelatraipeltkllndedqvvvnkaavmvhqlskkeasrhaimrspqMVSAivrtmqNT   60
DSSP  lllhhhhhhhhhhhllllhhhhhhhhhhhhhhhllllhhhhhhhhlhhHHHHhhhhhlLL


DSSP  LLHHHHHHHHHHHHHHLL---------llhHHHHHHHHHHHH------------------
Query TNLRSLRDRLTQVAERQG---------vvfGRLQRHVAXIVV------------------   41
ident       |                       |
Sbjct NDVETARCTAGTLHNLSHhregllaifksgGIPALVKMLGSPvdsvlfyaittlhnlllh  120
DSSP  LLHHHHHHHHHHHHHHLLlhhhhhhhhhllHHHHHHHHHHLLlhhhhhhhhhhhhhhhhh


DSSP  --hhhhhllllllllllleEEEH--------HHHHHHHHLllllllllleeeeelllhhhh
Query --aqfaatltddtgapllLVKG--------GSSLELRRGipdsrtskdfatvarrdieli   91
ident                                    |
```

```
DSSP  hhhhhhhhhhlllleeeeellleellllllllllleeeeeeeeelleeeeeeeeeeeelll
Query heqladagetgwegftaiftapeeiaapgxpvkprrftaklsyrgrafatvpievssvea  151
ident
Sbjct ------------------------------------------------------------  159
DSSP  ------------------------------------------------------------


DSSP  lllllleeeeelhhhhlllllleeeeeLLHHHHHHHHHHHHhlllllllllLLLHHHHHH
Query gnadqfdtltsdalglvgvpaavavpcXTIPWQIAQKLHAVtavleepkanDRAHDLVDL  211
ident                               |                         |
Sbjct --------------aygnqeskliilaSGGPQALVNIMRTY----------TYEKLLWTT  195
DSSP  --------------hlllhhhhhhhhhLLHHHHHHHHHHHL----------LLHHHHHHH


DSSP  HHHHHHL--LHHHHHHHHHHHhhhhhhhhlllllLLLLLlllhhhhhhhhhlllllllll
Query QLLEGLL--LDADLXPTRSACiaifearaqhpwpPRVATlphwpliyagalegldhlela  269
ident       |            |                  |
Sbjct SRVLKVLsvCSSNKPAIVEAG--------gmqalGLHLT---------------------  226
DSSP  HHHHHHHllLLLHHHHHHHLL--------hhhhhHLLLL---------------------


DSSP  lLHHHHHHHHHHHHHHHHHLLLL-------------------------------------
Query rTVDAAAQAVQRFVARIDRATKR-------------------------------------  292
ident        |           |
Sbjct dPSQRLVQNCLWTLRNLSDAATKqegmegllgtlvqllgsddinvvtcaagilsnltcnn  286
DSSP  lLLHHHHHHHHHHHHHHHHHLLLllllhhhhhhhhhhlllllhhhhhhhhhhhhhhhlll


DSSP  ------------------------------------------------------------
Query ------------------------------------------------------------  292
ident
Sbjct yknkmmvcqvggiealvrtvlragdreditepaicalrhltsrhqeaemaqnavrlhygl  346
DSSP  hhhhhhhhllhhhhhhhhhhhhlllhhhhhhhhhhhhhllllllhhhhhhhhhhhhlllh


DSSP  ------------------------------------------------------------
Query ------------------------------------------------------------  292
ident
Sbjct pvvvkllhppshwplikatvglirnlalcpanhaplreqgaiprlvqllvrahqdtqrrt  406
DSSP  hhhhllllllhhhhhhhhhhhhhhlllhhhhhhhhhllhhhhhhhhhhhhhhhhhhhlll


DSSP  ------------------------------------------------------------
Query ------------------------------------------------------------  292
ident
Sbjct sqfvegvrmeeivegctgalhilardvhnrivirglntiplfvqllyspieniqrvaagv  466
DSSP  lllllllhhhhhhhhhhhhhhhhlllhhhhhhhhhhlllhhhhhhllllllhhhhhhhhhh


DSSP  -----------------------------------------------
Query -----------------------------------------------  292
ident
Sbjct lcelaqdkeaaeaieaegatapltellhsrnegvatyaaavlfrmse  513
DSSP  hhhhllhhhhhhhhllllhhhhhhlllllhhhhhhhhhhhhhhhhhl
```

No 549: Query=mol1A Sbjct=2ii2A Z-score=4.8

back to top

```
DSSP  ------------------------------------------------------------
Query ------------------------------------------------------------    0
ident
Sbjct ygdaipevkaileakneeelvtftsrwsaeerkelrtqfqdttglefiaflkkcikngpy   60
DSSP  lllhhhhhhhhhhlllhhhhhhhhllllhhhhhhhhhhhhhhhlllhhhhhhhhllllhh


DSSP  ------------------------------llllllllllhhhhhhhhHHHHHHLLLlHH
Query ------------------------------aapysspptnlrslrdrlTQVAERQGVvFG   30
ident                                                     |  |
Sbjct edvmalgwdcnisarvnvikkamknvndfraihdvvliatpderlklaQAYKEKTGN-DL  119
DSSP  hhhhhhhhllhhhhhhhhhhhhhhllllhhhhhhhhhlllhhhhhhhhHHHHHHHLL-LH
```

```
ident
Sbjct LQDFVDQ-----------------------------------------------------  126
DSSP  HHHHHHH-----------------------------------------------------


DSSP  hhhhhhhhhhhllllleeeeellleellllllllllleeeeeeeeeelleeeeeeeeeeeeell
Query ihеqladagetgwegftaiftapeeiaapgxpvkprrftaklsyrgrafatvpievssve  150
ident
Sbjct ------------------------------------------------------------  126
DSSP  ------------------------------------------------------------


DSSP  lllllleeeeelhhhhllllllleeeeeLLHHHHHHHHHHHHHHLlllllllLLLLHHhHH
Query agnadqfdtltsdalglvgvpaavavpcXTIPWQIAQKLHAVTAvleepkaNDRAHDlVD  210
ident                                    |  |             |
Sbjct -------ipltsaasylchlairenrtpRGSVASDAEVLKHNLI------dADEPDH-EA  172
DSSP  -------llllllhhhhhhhhhhhllllLLLHHHHHHHHHHHHHL------lLLLLLH-HH


DSSP  HHHHHHHLLHHHHHHHHHHHHHHHHHhhhlllLLLL--LLLLlllhhhhhhhhhlllllll
Query LQLLEGLLLDADLXPTRSACIAIFearaqhPWPP--RVATlphwpliyagalegldhlel  268
ident    |                                   |
Sbjct VVRLIITSTADEYKEINHRFEVLT------GKSVqeAIET--------------------  206
DSSP  HHHHHHHLLHHHHHHHHHHHHHHH------LLLHhhHHHH--------------------


DSSP  llLHHHHHHHHHHHHHHHH-----------------------------------------
Query arTVDAAAQAVQRFVARID-----------------------------------------  287
ident        |
Sbjct ryADKENARGLCIAHYYNLaparavayafhsavetqnddmayeqaaritglfhdlhkfaw  266
DSSP  hlLLHHHHHHHHHHHHHHHlhhhhhhhhhhhhhllllhhhhhhhhhhhhhhhllllllhh


DSSP  ---------------------------------hllll
Query ---------------------------------ratkr  292
ident
Sbjct vhyacwgvmrddilsrfqskeankvnfrdaclmfwkla  304
DSSP  hhhllllhhhhhhhhlllllllllhhhhhhhhhlll
```

No 550: Query=mol1A Sbjct=5a2wB Z-score=4.8

back to top

```
DSSP  ----------------------------LLLL-----------lllLLLHHHHhHHHH-
Query ----------------------------AAPY-----------sspPTNLRSLrdRLTQ-   20
ident                                                 |       |
Sbjct ktftevqterleqadrsvlikcpsklneKKLLqylsshgkidnyffFENRGIH--ALIEf   58
DSSP  llhhhhhhhhhhhhhleeeeellllllhHHHHhhhhlllleeeeeeEELLLEE--EEEEe


DSSP  ------------------------------------------------------------
Query ------------------------------------------------------------   20
ident
Sbjct sekssvaslqavtgipkaaehhvvpyksrlftftlknpgsqaaeerpvkispqshipvne  118
DSSP  lllhhhhhhhhlllllllllllllllleeeeeellhhhllllllllllllllllhhh


DSSP  ------------------hhhhllLLHHHHHHHHHHHHHHHHHHHLLLlllllLLEEEEHH
Query ------------------vaerqgVVFGRLQRHVAXIVVAQFAATLTddtgaPLLLVKGG   62
ident                                |  |   |  ||         |   ||
Sbjct lipklchadsissqmyillneyqlTEENIKLRYLACSLVRDFARAYF-----PDSTVKPF  173
DSSP  hhhhhhllllhhhhhhhhhhhhllLHHHHHHHHHHHHHHHHHHHLLLL-----LLEEEEEE


DSSP  hhhhhhhlllllllLLEEEEEL------------------lLHHHHHHHHHHHHHHHLLl
Query sslelrrgipdsrtsKDFATVAR------------------rDIELIHEQLADAGETGWe  104
ident                 |                             |     |
Sbjct --gssvntfgklgcdVDMFLDFHkkgpfemeyqmkrlpserlATQKILSIIGDCLDNFG-  230
DSSP  --hhhllllllllllEEEEEEELllllleeeeellllllhhhHHHHHHHHHHHHHHHHLL-


DSSP  lEEEE-ELLLEELllllllLLEEEEEEEEEEeeEEEEEEEEEelllllllllleeeeel
Query gFTAI-FTAPEEIaapgxpvKPRRFTAKLSYrgraFATVPIEVSsveagnadqfdtltsd  163
ident                                           ||
Sbjct -PGYSsVQKILNA-------RCPLVKFSHQP---tGFQCDLSVS----------------  263
```

```
DSSP  hhhhlllllleeeeellhhhhhhHHHHHHhllllllllLLLLhhhhhhhhhhhhllhhhh
Query alglvgvpaavavpcxtipwqiaQKLHAVtavleepkaNDRAhdlvdlqllegllldadl  223
ident
Sbjct -------------------nsiaIRCSEL---------LYIY------------------  277
DSSP  -------------------lhhhHHHHHH---------HHHH------------------


DSSP  hhhhhhhhhhhhhllllllllllllllLHHHhHHHHHL-lLLLL----------------
Query xptrsaciaifearaqhpwpprvatlpHWPLiYAGALE-gLDHL----------------  266
ident
Sbjct --------------------------gCLDPrVRALVFslRCWArvhgltnsvpgtwitn  311
DSSP  --------------------------hHHLHhHHHHHHhhHHHHhhlllllllllllllh


DSSP  --llllLHHHHHHH----------------------------------------------
Query --elarTVDAAAQA----------------------------------------------  278
ident
Sbjct fsltmmIMFFLQKRsppiiptldqlkeladekdkhviggydcsfvsdlskikptkntetl  371
DSSP  hhhhhhHHHHHHLLlllllllhhhhhhlllllllleelleeelllllllllllllllllh


DSSP  --------------------hhhhhhhhhHLLLL--------------------------
Query --------------------vqrfvaridRATKR--------------------------  292
ident
Sbjct dellcdffqyfgnfdfrknslnlrkgkevNKPESsplyiwnpfeqdlnisknvnqpqlek  431
DSSP  hhhhhhhhhhhhhlllllleeellllleeeLLLLLllllllllllllllllllllhhhhhh


DSSP  ------------------------------------
Query ------------------------------------  292
ident
Sbjct fvamaresawilqkedktqqminkepwglaavlipf  467
DSSP  hhhhhhhhhhhhhhhllhhhhhhlllllhhhhllll
```

No 551: Query=mol1A Sbjct=4at8B Z-score=4.8

back to top

```
DSSP  llllllllllhhhhhhhhhhhhhhlLLLHHHHHHHHHHHHHHHHHHLL---LLLL-lLLL
Query aapysspptnlrslrdrltqvaerqGVVFGRLQRHVAXIVVAQFAATL---TDDT-gAPL   56
ident                                                     |      |
Sbjct -------rifvnddrhvmakhssvyPTQEELEAVQNMVSHTERALKAVsdwIDEQekRTL   53
DSSP  -------lllhhhhhhhhhhhhhhlLLHHHHHHHHHHHHHHHHHHHHHhhhHHLLllLLE


DSSP  EEEEhhHHHHhhhlllLLLL-----lLLEEEEEL--LLHHHHHHHHHHHHHHLL--LLEE
Query LLVKggSSLElrrgipDSRT-----sKDFATVAR--RDIELIHEQLADAGETGW--EGFT  107
ident   |                                     |
Sbjct RGVM--RVGL----vaKGLLlkgdldLELVLLCKekPTTALLDKVADNLAIQLTtvTEDK  107
DSSP  EEEE--ELHH----hhHLLLllllleEEEEEEELllLLHHHHHHHHHHHHHHHHhlLLLL


DSSP  EEELLLEELlllllllllEEEEEEEEelleeEEEEEEEEEELLLLllllleeeeelhhhh
Query AIFTAPEEIaapgxpvkpRRFTAKLSyrgraFATVPIEVSSVEAGnadqfdtltsdalgl  167
ident                        |            |   |
Sbjct YEILQSVDD---------AAIVIKNT--kepPLSLTIHLTSPVVR-------eemekvag  149
DSSP  LEEEEELLL---------LEEEEELL--lllLLEEEEEEELHHHH-------hhhhhlll


DSSP  llllllleeeeellhhhhhhHHHHHHhlllllllllLLLHhhhhhhhhhhhllhhhhhhhh
Query vgvpaavavpcxtipwqiaQKLHAVtavleepkanDRAHdlvdlqllegllldadlxptr  227
ident                                     ||
Sbjct edppdvldrqkclaalaslRHAKWF---------qARAN---------------------  179
DSSP  lllllllhhhhhhhhhhhhHHHHHH---------hHHLL---------------------


DSSP  hhhhhhhhhlllllllllllllllHHHHHHHHHLlLLLL----------------llllL
Query saciaifearaqhpwpprvatlphWPLIYAGALEgLDHL----------------elarT  271
ident                                     |
Sbjct -----------------------gLKSCVIVIRVlRDLCtrvptwgplrgwplellcekS  216
DSSP  -----------------------lLLLHHHHHHHhHHHHhhlhhhhhhllhhhhhhhhhH


DSSP  HHH-----------------------hhhhhHHHH------HHHHHLLLL----------
```

```
Sbjct IGTanrpmgagealrrvleclasgivmpdgsGIYDpcekeaTDAIGHLDRqqreditqsa  276
DSSP  HHLllllllhhhhhhhhhhhhhllllllllLLLLllllllLLLLLLLLHhhhhhhhhhh


DSSP  ----------------------------------------
Query ----------------------------------------  292
ident 
Sbjct qhalrlaafgqlhkvlgmdplpdytvqippsttyaitprp  316
DSSP  hhhhhhhhlllhhhhllllllllllllllllllllllll
```

No 552: Query=mol1A Sbjct=4k97A Z-score=4.8

back to top

```
DSSP  lllllllllhhhhhhhhhhhhhhlLLLHHHHHHHHHHHHHHHHHHH-LLLLLLLL-LLEE
Query aapysspptnlrslrdrltqvaerqGVVFGRLQRHVAXIVVAQFAA-TLTDDTGA-PLLL   58
ident                                         |
Sbjct ---------------klkkvldklrLKRKDISEAAETVNKVVERLLrRMQKRESEfKGVE   45
DSSP  ---------------lhhhhhhhhlLLHHHHHHHHHHHHHHHHHHHHhHHLLLLLLlLLLE


DSSP  EEHHhhhhhhhlllLLLL----lLLEEEEEL-----------------------------
Query VKGGsslelrrgipDSRT----sKDFATVAR-----------------------------   85
ident                         |
Sbjct QLNT-----gsyyeHVKIsapneFDVMFKLEvprielqeyyetgafylvkfkplshfleg  100
DSSP  EEEE-----lllllLLLLlllleEEEEEEEEllleeeeellllleeeeeellllllleel


DSSP  ------lLHHHHHHHHHHHHHHLlLLEEEEEELLLEellllllllllEEEEEEEEEllEEE
Query ------rDIELIHEQLADAGETGwEGFTAIFTAPEeiaapgxpvkpRRFTAKLSYrgRAF  139
ident                                                  |
Sbjct evlsatkMLSKFRKIIKEEVKEI-KDIDVSVEKEK--------pgsPAVTLLIRN--PEE  149
DSSP  leelhhhHHHHHHHHHHHHHLLL-LLLEEEELLLL--------lllLEEEEEEEL--LLE


DSSP  EEEEEEEEELLLL-------------------lllllleeeeelhhhhlllllleeeeell
Query ATVPIEVSSVEAG-------------------nadqfdtltsdalglvgvpaavavpcxt  180
ident   | |       |
Sbjct ISVDIILALESKGswpistkeglpiqgwlgtkvrtnlrrepfylvpknakdgnsfqgetw  209
DSSP  EEEEEEEEEEELLlllhhhllllllllllhhhhhhhllllleeeellllllllllllle


DSSP  hHHHHhHHHHHHhllllllllllllhhhhhhhhhhhhllhhhhhhhhhhhhhhhhhhlll
Query iPWQIaQKLHAVtavleepkandrahdlvdlqllegllldadlxptrsaciaifearaqh  240
ident 
Sbjct rLSFS-HTEKYI------------------------------------------------  220
DSSP  eEELH-HHHHHH------------------------------------------------


DSSP  lllllllLLLL--------hHHHHHHHHL-lLLLL---------------llllllhhHHH
Query pwpprvaTLPH--------wPLIYAGALE-gLDHL---------------elartvdAAA  276
ident 
Sbjct ---lnnhGIEKtccessgakCCRKECLKLmkYLLEqlkkefqeldafcsyhvktaifHMW  277
DSSP  ---hhllLLLLlllllllllLLHHHHHHHhhHHHHhhhhhlhhhllllhhhhhhhhhHHH


DSSP  H-----------------------------------hhhhhhhHHHH-------------
Query Q-----------------------------------avqrfvaRIDR-------------  288
ident 
Sbjct TqdpqdsqwdprnlsscfdkllaffleclrtekldhyfipkfNLFSqelidrkskeflsk  337
DSSP  HhlllhhhhlhhhhhhhhhhhhhhhhhhhhhhhlllllllllllLLLLlllllhhhhhhhhh


DSSP  ----------llll
Query ----------atkr  292
ident 
Sbjct kieyernngfpifd  351
DSSP  hhhhhhllllhhhl
```

No 553: Query=mol1A Sbjct=4k98A Z-score=4.8

```
DSSP  lllllllllhhhhhhhhhhhhhhhlLLLHHHHHHHHHHHHHHHHHH-LLLLLLL-lLLEE
Query aapysspptnlrslrdrltqvaerqGVVFGRLQRHVAXIVVAQFAA-TLTDDTG-aPLLL   58
ident                                            |
Sbjct ---------------klkkvldklrLKRKDISEAAETVNKVVERLLrRMQKRESefKGVE   45
DSSP  ---------------lhhhhhhhhlLLHHHHHHHHHHHHHHHHHHHHhHHLLLLLllLLLE


DSSP  EEHHhhhhhhhlllLLLL----lLLEEEEEL----------------------------
Query VKGGsslelrrgipDSRT----sKDFATVAR----------------------------   85
ident                           |
Sbjct QLNT-----gsyyeHVKIsapneFDVMFKLEvprielqeyyetgafylvkfkgnplshfl  100
DSSP  EEEE-----hhhhhLLLLlllleEEEEEEEElllleeeeelllllleeeeeelllhhhhhe


DSSP  --------lLHHHHHHHHHHHHHHHLlLLEEEEELLLEelllllllllEEEEEEEEEllE
Query --------rDIELIHEQLADAGETGwEGFTAIFTAPEeiaapgxpvkpRRFTAKLSYrgR  137
ident                                                    |
Sbjct egevlsatkMLSKFRKIIKEEVKEI-KDIDVSVEKEK--------pgsPAVTLLIRN--P  149
DSSP  elleelhhhHHHHHHHHHHHHHHHHLL-LLLEEEELLLL--------lllLEEEEEEEL--L


DSSP  EEEEEEEEEEELLL----------------------------LLLLlleeeeelhhhhll
Query AFATVPIEVSSVEA----------------------------GNADqfdtltsdalglvg  169
ident     | |
Sbjct EEISVDIILALESKgswpistkeglpiqgwlgtkvrtnlrrePFYL--------vpknak  201
DSSP  LEEEEEEEEEEEELllllhhhlllllllllllhhhhhhhhllLEEE--------elllee


DSSP  llleeeeellhhHHHHHHHHHHhlllllllllllhhhhhhhhhhhllhhhhhhhhhh
Query vpaavavpcxtipWQIAQKLHAVtavleepkandrahdlvdlqlleglllldadlxptrsa  229
ident                  |
Sbjct dgnsfqgetwrlsFSHTEKYILN-------------------------------------  224
DSSP  elleeellleeeeLHHHHHHHHH-------------------------------------


DSSP  hhhhhhhhlllllllllLLLL-------lhHHHHHHHHL-lLLLL---------------
Query ciaifearaqhpwpprvATLP-------hwPLIYAGALE-gLDHL---------------  266
ident
Sbjct ---------------nhGIEKtccessgakCCRKECLKLmkYLLEqlkkefqeldafcsy  269
DSSP  ---------------llLLLLlllllllllLLHHHHHHHhHHHHhhhhhlhhhllllhh


DSSP  lllllhhHHHH-------------------------------------------------
Query elartvdAAAQ-------------------------------------------------  277
ident
Sbjct hvktaifHMWTqdpqdsqwdprnlsscfdkllafflecl rtekldhyfipkfnlfsqeli  329
DSSP  hhhhhhhHHHHhlllhhhhlhhhhhhhhhhhhhhhhhhhhhllllllllllllllll


DSSP  ----------hhhhhhhhhhhllll
Query ----------avqrfvaridratkr  292
ident
Sbjct drkskeflskkieyernngfpifdk  354
DSSP  lhhhhhhhhhhhhhhhhhlllhhhhhl
```

No 554: Query=mol1A Sbjct=3au2A Z-score=4.8

back to top

```
DSSP  ------------------------------------------------------------
Query ------------------------------------------------------------    0
ident
Sbjct mrnqelarifeeiglmseflgdnpfrvrayhqaartlydldtpieeiaekgkealmelpg   60
DSSP  llhhhhhhhhhhhhhhhhhhllllhhhhhhhhhhhhhhhhllllhhhhhlllhhhhlllll


DSSP  ------------------------------------------------------------
Query ------------------------------------------------------------    0
ident
Sbjct vgpdlaekileflrtgkvrkheelsrkvprgvlevmevpgvgpktarllyeglgidslek  120
DSSP  llhhhhhhhhhhhhhlllhhhhhhhhhllhhhhhhhllllllhhhhhhhhhhhllllhhh


DSSP  ------llllllllllhhhhhhhhhhhhhhllllhHHHHHHHHHHHHHHHHHHLLLLllll
Query ------aapysspptnlrslrdrltqvaerqgvvfGRLQRHVAXIVVAQFAATLTDdtga   54
```

```
DSSP  hhhhhhhlhhhhllllhhhhhhhhhhhhhhhhllLLEEHHHHHHHHHHHHHHHHHLllll
```

```
DSSP  lLEEEEHHHHHHhhhllllLLLLLLEEEEEL-----lLHHHHHHHHHhhhhhlllleeee
Query pLLLVKGGSSLElrrgipdSRTSKDFATVAR-----rDIELIHEQLAdagetgwegftai  109
ident       |              |  |              |
Sbjct eRAELCGSARRY-------KDTVGDLDFLVAsregerAVEGFVRLPQ------------v  221
DSSP  lEEEELHHHHLL-------LLEELLEEEEEElllhhhHHHHHHLLLL------------e
```

```
DSSP  ELLLEEllllllLLLEEEEEEEeelleeeeEEEEEEEEL-LLLLllllleeeeelhhhhl
Query FTAPEEiaapgxpVKPRRFTAKLsyrgrafaTVPIEVSSV-EAGNadqfdtltsdalglv  168
ident                      |                 |
Sbjct KEVYAK------gKERATVFLKN--------GLQVDLRVVpPESY---------------  252
DSSP  EEEEEE------lLLEEEEEELL--------LLEEEEEEElHHHH---------------
```

```
DSSP  llllleeeeellhHHHH----hhhHHHHHLLllllllllllhhhhhhhhhhhhllhhhhh
Query gvpaavavpcxtiPWQI----aqkLHAVTAVleepkandrahdlvdlqlleglllldadlx  224
ident
Sbjct ------------gAGLQyltgskaHSIRLRA---------laqekglklseygvfrgekr  291
DSSP  ------------hHHHHhhhllhhHHHHHHH---------hhhhllleeelleeeellee
```

```
DSSP  hhhhHHHHhhhhhlllLLLLlllllllhhhhhhhhhllllllllllhhHHHHHHHH---
Query ptrsACIAifearaqhPWPPrvatlphwpliyagalegldhlelartvdAAAQAVQR---  281
ident                                                      |
Sbjct iageTEEE-----vyaALGL------------------pwipppplredqGEVEAALEgrl  328
DSSP  eellLHHH-----hhhHLLL------------------llllhhhllllLHHHHHHLlll
```

```
DSSP  ------------------------------------------------------------
Query ------------------------------------------------------------  281
ident
Sbjct pkllelpqvkgdlqvhstysdgqntleelweaaktmgyrylavtdhspavrvaggpspee  388
DSSP  llllhhhlleeeeellllllllllhhhhhhhhhhhllleeeeeeelhhhhlllllllhhh
```

```
DSSP  ------------------------------------------------------------
Query ------------------------------------------------------------  281
ident
Sbjct alkrvgeirrfnethgppyllagaevdihpdgtldypdwvlreldlvlvsvhsrfnlpka  448
DSSP  hhhhhhhhhhhhhhhllleeeeeeeeellllllllllhhhhllllleeeeelllllllllhh
```

```
DSSP  ------------------------------------------------------------
Query ------------------------------------------------------------  281
ident
Sbjct dqtkrllkalenpfvhvlahptarllgrrapieadweavfqkakekgvaveidgyydrmd  508
DSSP  hhhhhhhhhhlllllleellllllllllllllllhhhhhhhhhhhhlleeeeeellllll
```

```
DSSP  --------------------------------------------------------hhhh
Query --------------------------------------------------------fvar  285
ident
Sbjct lpddlarmaygmglwislstdahqtdhlrfmelavgtaqrawigpervlntldyedllsw  568
DSSP  llhhhhhhhhhhlllleeeeellllhhhhhhhhhhhhhhhhhhlllllllllhhhllhhhhhhh
```

```
DSSP  hhhllll
Query idratkr  292
ident
Sbjct lkarrgv  575
DSSP  hhlllll
```

No 555: Query=mol1A Sbjct=4lzgA Z-score=4.8

back to top

```
DSSP  ------------------------------------------------------------
Query ------------------------------------------------------------    0
ident
Sbjct mpayacqrptplthhntglsealeilaeaagfegsegrlltfcraasvlkalpspvttls   60
DSSP  llllhhhllllllllllhhhhhhhhhhhhhhhhhlllhhhhhhhhhhhhhhhhhllllllllhh
```

```
Query ------------------------------------------------------------    0
ident
Sbjct qlqglphfgehssrvvqellehgvceevervrrseryqtmklftqifgvgvktadrwyre  120
DSSP  hhllllllllhhhhhhhhhhhhhlllhhhhhhhhlhhhhhhhhhhlllllllhhhhhhhhhl


DSSP  llllllllllhhhhhhhhhhhhhhllllhhhhhHHHHHHHHHHHHHLLLL-llLLLLEEE
Query aapysspptnlrslrdrltqvaerqgvvfgrlqRHVAXIVVAQFAATLTD-dtGAPLLLV   59
ident                                           |
Sbjct glrtlddlreqpqkltqqqkaglqhhqdlstpvLRSDVDALQQVVEEAVGqalPGATVTL  180
DSSP  llllhhhhhllhhhllhhhhhhhhlhhhhllleEHHHHHHHHHHHHHHHHHhhlLLLEEE


DSSP  EHHHHHHHhhllllllLLLLEEEEEL--------lLHHHHHHHHHHHHhhlllleeeEEL
Query KGGSSLELrrgipdsrTSKDFATVAR--------rDIELIHEQLADAGetgwegftaIFT  111
ident  ||                |                        | | |
Sbjct TGGFRRGK-------lQGHDVDFLIThpkegqeagLLPRVMCRLQDQG---------LIL  224
DSSP  LHHHHLLL-------lEELLEEEEEEllllllllHHHHHHHHHHHHLL---------LEE


DSSP  LLEellllllLLLEEEEEEEEEELLEeEEEEEEEEEEL-LLLLllllleeeeelhhhhlll
Query APEeiaapgxpVKPRRFTAKLSYRGRaFATVPIEVSSV-EAGNadqfdtltsdalglvgv  170
ident                     |   |      |
Sbjct YHQ--------FERSFCIFRLPQPGS-WKAVRVDLVVApVSQF-----------------  258
DSSP  ELL--------LLEEEEEEEEEELLE-EEEEEEEEEELlHHHH-----------------


DSSP  llleeeeellhHHHHhhhhhhhhllllllllllHHHHHHH----------------hh
Query paavavpcxtiPWQIaqklhavtavleepkandraHDLVDLQ----------------ll  214
ident                                       |
Sbjct ----------pFALL----------------gwtgSKLFQRElrrfsrkekglwlnshgl  292
DSSP  ----------hHHHH----------------hhhlLHHHHHHhhhhhhhhhlleellle


DSSP  hhhllhHHHHhhHHHHHhhhhhhllllLLLLLLllllhhhhhhhhhlllllllllllhhh
Query eglllldADLXptRSACIaifearaqhpWPPRVAtlphwpliyagalegldhlelartvda  274
ident
Sbjct fdpeqkTFFQ--AASEE-------difRHLGLE---------------------------  316
DSSP  eelllllEELL--LLLHH-------hhhHHLLLL---------------------------


DSSP  hhhhhhhhhhhhhhllll
Query aaqavqrfvaridratkr  292
ident
Sbjct ---------ylppeqrna  325
DSSP  ---------lllhhhlll
```

No 556: Query=mol1A Sbjct=3b0xA Z-score=4.8

back to top

```
DSSP  ------------------------------------------------------------
Query ------------------------------------------------------------    0
ident
Sbjct mrnqelarifeeiglmseflgdnpfrvrayhqaartlydldtpieeiaekgkealmelpg   60
DSSP  llhhhhhhhhhhhhhhhhhhllllhhhhhhhhhhhhhhhhllllhhhhhlllhhhhhllll


DSSP  ------------------------------------------------------------
Query ------------------------------------------------------------    0
ident
Sbjct vgpdlaekileflrtgkvrkheelsrkvprgvlevmevpgvgpktarllyeglgidslek  120
DSSP  llhhhhhhhhhhhhhlllhhhhhhhhhllhhhhhhhllllllhhhhhhhhhllllllhhh


DSSP  ------llllllllllhhhhhhhhhhhhhhllllhhHHHHHHHHHHHHHHHHHLLLLllll
Query ------aapysspptnlrslrdrltqvaerqgvvfgRLQRHVAXIVVAQFAATLTDdtga   54
ident                                   |
Sbjct lkaaldrgdltrlkgfgpkraerireglalaqaagkRRPLGAVLSLARSLLEAIRAlpgv  180
DSSP  hhhhhhhlhhhhlllllhhhhhhhhhhhhhhhhhllLEEHHHHHHHHHHHHHHHHHLllll


DSSP  lLEEEEHHHHHHHhhllllllLLLLEEEEEL-----lLHHHHHHHHHhhhhhllllleeee
Query pLLLVKGGSSLELrrgipdsrTSKDFATVAR-----rDIELIHEQLAdagetgwegftai  109
ident       |                |  |               |
```

```
DSSP  ELLLEELllllllLLLEEEEEEEeelleeeeEEEEEEEEL-LLLLllllleeeeelhhhhl
Query FTAPEEIaapgxpVKPRRFTAKLsyrgrafaTVPIEVSSV-EAGNadqfdtltsdalglv  168
ident                      |                 |
Sbjct KEVYAKG------KERATVFLKN--------GLQVDLRVVpPESY---------------  252
DSSP  EEEEEEL------LLEEEEEELL--------LLEEEEEEEElHHHH---------------


DSSP  lllllleeeeellhHHHHhhhhhhhhllllllllllllHHHHHHH------------hhhh
Query gvpaavavpcxtiPWQIaqklhavtavleepkandraHDLVDLQ------------lleg  216
ident
Sbjct ------------gAGLQ----------------yltgSAAHSIRlralaqekglklseyg  284
DSSP  ------------hHHHH----------------hhhlLHHHHHHhhhhhhhllleeelle


DSSP  hllhhhhhhhhhHHHHhhhhhlllLLLLlllllllhhhhhhhhhllllllllllhhhhHH
Query llldadlxptrsACIAifearaqhPWPPrvatlphwpliyagalegldhlelartvdaAA  276
ident
Sbjct vfrgekriagetEEEV------yaALGL---------------------pwippplreDQ  317
DSSP  eeelleeeelllHHHH------hhHLLL---------------------llllhhhllLL


DSSP  HHHHHH------------------------------------------------------
Query QAVQRF------------------------------------------------------  282
ident   |
Sbjct GEVEAAlegrlpkllelpqvkgdlqvhstysdgqntleelweaaktmgyrylavtdhspa  377
DSSP  LHHHHHhllllllllhhhlleeeeellllllllllhhhhhhhhhhlllleeeeeeelll


DSSP  ------------------------------------------------------------
Query ------------------------------------------------------------  282
ident
Sbjct vrvaggpspeealkrvgeirrfnethgppyllagaevdihpdgtldypdwvlreldlvlv  437
DSSP  llllllllhhhhhhhhhhhhhhhhhhhllleeeeeeeeeellllllllllhhhhlllleeee


DSSP  ------------------------------------------------------------
Query ------------------------------------------------------------  282
ident
Sbjct svhsrfnlpkadqtkrllkalenpfvhvlahptarllgrrapieadweavfqkakekgva  497
DSSP  ellllllllhhhhhhhhhhhlllllllleellllllllllllllllhhhhhhhhhhhhlle


DSSP  ------------------------------------------------------------
Query ------------------------------------------------------------  282
ident
Sbjct veidgyydrmdlpddlarmaygmglwislstdahqtdhlrfmelavgtaqrawigpervl  557
DSSP  eeeellllllllllhhhhhhhhhllllleeeellllllhhhhhhhhhhhhhhhhhlllllllh


DSSP  --------hhhhhhllll
Query --------varidratkr  292
ident
Sbjct ntldyedllswlkarrgv  575
DSSP  hhllhhhhhhhhhlllll
```

No 557: Query=mol1A Sbjct=3b0yA Z-score=4.8

back to top

```
DSSP  ------------------------------------------------------------
Query ------------------------------------------------------------    0
ident
Sbjct mrnqelarifeeiglmseflgdnpfrvrayhqaartlydldtpieeiaekgkealmelpg   60
DSSP  llhhhhhhhhhhhhhhhhhhllllhhhhhhhhhhhhhhhhhlllllhhhhhlllhhhhlllll


DSSP  ------------------------------------------------------------
Query ------------------------------------------------------------    0
ident
Sbjct vgpdlaekileflrtgkvrkheelsrkvprgvlevmevpgvgpktarllyeglgidslek  120
DSSP  llhhhhhhhhhhhhhlllhhhhhhhhhllhhhhhhhhlllllhhhhhhhhhhhlllllhh
```

```
ident                                     |
Sbjct lkaaldrgdltrlkgfgpkraerireglalaqaagkRRPLGAVLSLARSLLEAIRAlpgv  180
DSSP  hhhhhhlhhhllllllhhhhhhhhhhhhhhhllLEEHHHHHHHHHHHHHHHHHLllll


DSSP  lLEEEEHHHHHHHhhllllllLLLLEEEEEL-----lLHHHHHHHHHhhhhhlllleeee
Query pLLLVKGGSSLELrrgipdsrTSKDFATVAR-----rDIELIHEQLAdagetgwegftai  109
ident       |              |  |              |
Sbjct eRAELCGSARRYK-------dTVGDLDFLVAsregerAVEGFVRLPQ------------v  221
DSSP  lEEEELHHHHLLL-------lEELLEEEEEElllhhhHHHHHHLLLL------------e


DSSP  ELLLEELllllllLLLEEEEEEEeelleeeeEEEEEEEEL-LLLLllllleeeeelhhhhl
Query FTAPEEIaapgxpVKPRRFTAKLsyrgrafaTVPIEVSSV-EAGNadqfdtltsdalglv  168
ident                      |                 |
Sbjct KEVYAKG------KERATVFLKN--------GLQVDLRVVpPESY---------------  252
DSSP  EEEEEEL------LLEEEEEELL--------LLEEEEEEElHHHH---------------


DSSP  llllleeeeellhHHHHhhhhhhhhllllllllllllHHHHHHH------------hhhh
Query gvpaavavpcxtiPWQIaqklhavtavleepkandraHDLVDLQ------------lleg  216
ident                                       |
Sbjct ------------gAGLQ----------------yltgSDAHSIRlralaqekglklseyg  284
DSSP  ------------hHHHH----------------hhhlLHHHHHHhhhhhhhllleeelle


DSSP  hllhhhhhhhhhHHHHhhhhhlllLLLLlllllllhhhhhhhhhlllllllllllhhhHH
Query llldadlxptrsACIAifearaqhPWPPrvatlphwpliyagalegldhlelartvdaAA  276
ident
Sbjct vfrgekriagetEEEV------yaALGL---------------------pwippplreDQ  317
DSSP  eeelleeeelllHHHH------hhHLLL---------------------llllhhhllLL


DSSP  HHHHHH------------------------------------------------------
Query QAVQRF------------------------------------------------------  282
ident   |
Sbjct GEVEAAlegrlpkllelpqvkgdlqvhstysdgqntleelweaaktmgyrylavtdhspa  377
DSSP  LHHHHHhllllllllhhhlleeeeelllllllllhhhhhhhhhhlllleeeeeeelll


DSSP  ------------------------------------------------------------
Query ------------------------------------------------------------  282
ident
Sbjct vrvaggpspeealkrvgeirrfnethgppyllagaevdihpdgtldypdwvlreldlvlv  437
DSSP  lllllllhhhhhhhhhhhhhhhhhhhllleeeeeeeeeelllllllllhhhhlllleeee


DSSP  ------------------------------------------------------------
Query ------------------------------------------------------------  282
ident
Sbjct svhsrfnlpkadqtkrllkalenpfvhvlahptarllgrrapieadweavfqkakekgva  497
DSSP  elllllllhhhhhhhhhhhhhllllleellllllllllllllllhhhhhhhhhhhhlle


DSSP  ------------------------------------------------------------
Query ------------------------------------------------------------  282
ident
Sbjct veidgyydrmdlpddlarmaygmglwislstdahqtdhlrfmelavgtaqrawigpervl  557
DSSP  eeeelllllllllhhhhhhhhhllllleeeellllhhhhhhhhhhhhhhhhhlllllllh


DSSP  --------hhhhhhllll
Query --------varidratkr  292
ident
Sbjct ntldyedllswlkarrgv  575
DSSP  hhllhhhhhhhhhlllll
```

No 558: Query=mol1A Sbjct=1jqrA Z-score=4.8

back to top

```
DSSP  llllllllllhhhhhhhhhhhhhhhllllhhhhhHHHHHHHHHHHHHHLL-------llLLL
Query aapysspptnlrslrdrltqvaerqgvvfgrlqRHVAXIVVAQFAATL-------tdDTG   53
ident
Sbjct -------------------------------mlTLIQGKKIVNHLRSRlafeyngqlIKI   29
```

```
DSSP  LL-LEEEEHHHHHHHhhllllllLLLLEEEEEL--------lLHHHHHhhhHHHHhhlll
Query AP-LLLVKGGSSLELrrgipdsrTSKDFATVAR--------rDIELIHeqlADAGetgwe  104
ident         |    |            |                   |
Sbjct LSkNIVAVGSLRREE-------kMLNDVDLLIIvpekkllkhVLPNIR---IKGL-----   74
DSSP  LHhHEEELHHHHLLL-------lEELLEEEEEEelllllhhhHLLLEE---ELLL-----


DSSP  leeeEELLLEELllllllLLLEEEEEEEEelleeEEEEEEEEEEELLLLLllleeeeelh
Query gftaIFTAPEEIaapgxpVKPRRFTAKLSyrgraFATVPIEVSSVEAGNAdqfdtltsda  164
ident      |                              |         |
Sbjct ----SFSVKVCG------ERKCVLFIEWE-----KKTYQLDLFTALAEEK----------  109
DSSP  ----EEEEEEEL------LLEEEEEEEEL-----LEEEEEEEEEEELLHH----------


DSSP  hhhlllllleeeeellhHHHHHHhhhhhhllllllllllllhHHHHHH------------
Query lglvgvpaavavpcxtiPWQIAQklhavtavleepkandrahDLVDLQ------------  212
ident                                               |
Sbjct ----------------pYAIFHF-----------------tgPVSYLIriraalkkknyk  136
DSSP  ----------------hHHHHHH-----------------hlLHHHHHhhhhhhhlllee


DSSP  -hhhhhllhHHHHhhhhhhhhhhhhhllllLLLLLlllllhhhhhhhhhlllllllllll
Query -llegllldADLXptrsaciaifearaqhpWPPRVatlphwpliyagalegldhlelart  271
ident            |
Sbjct lnqyglfknQTLV----plkittekelikeLGFTY-------------------------  167
DSSP  eelleeeelLEEL----lllllllhhhhhhHLLLL-------------------------


DSSP  hhhhhhhhhhhhhhhhhllll
Query vdaaaqavqrfvaridratkr  292
ident
Sbjct --------------ripkkrl  174
DSSP  --------------llhhhll
```

No 559: Query=mol1A Sbjct=3wfrE Z-score=4.8

back to top

```
DSSP  llllllllllhhhhhhhhhhhhhhllllhhhhhhhhhhHHHHHHHHLLLLllLLLLEEEE
Query aapysspptnlrslrdrltqvaerqgvvfgrlqrhvaxIVVAQFAATLTDdtGAPLLLVK   60
ident                                              |
Sbjct ------------------------------xxxxlnfyLSYFDDVAKVLP--REHYCFIV   28
DSSP  ------------------------------llllllllLLLHHHHHHHLL--LLLLEEEE


DSSP  HHHHHHHHHLLLlllllLLEEEEEELLLHHHHHHHHHHHhhlllleeeEELLLEELllll
Query GGSSLELRRGIPdsrtsKDFATVARRDIELIHEQLADAGetgwegftaIFTAPEEIaapg  120
ident ||       | |      |       |        |
Sbjct GGWVRDRILGEP-vgynIDVDFLTTADPVELAKNFAKRI---------GGHFFVFE----   74
DSSP  LHHHHHHHHLLL-llleEEEEEEEELLLHHHHHHHHHHH---------LLEEEEEL----


DSSP  llLLLEEEEEEEeelleEEEEEEEEEEEL-----LLLL---------------llllEEE
Query xpVKPRRFTAKLsyrgrAFATVPIEVSSV-----EAGN---------------adqfDTL  160
ident            |              |       |                      | |
Sbjct --PTIASVVLHL-----PPYRYRFDFSPLkgkdlEKALiedlkerdftanaiavnldDVL  127
DSSP  --LLEEEEEEEL-----LLLEEEEEEEELllllhHHHHhhhhllllllhhhleeehhHHL


DSSP  EelhhhhllllleeeeeelLHHH-----------hhhhhhhHHHLlllllllLLLLHhhh
Query TsdalglvgvpaavavpcxTIPW-----------qiaqklhAVTAvleepkaNDRAHdlv  209
ident                                                       |
Sbjct S----------------tiVYDPtggikdleqgllrpvsieNLKR------dPVRVL---  162
DSSP  L----------------llEELLllhhhhhhlleellllhhHHHH------lLLHHH---


DSSP  hhhhhhhhllhhhhhhhhhhhhhhhhhhllllllllllllllhhhHHHHHHLLLLlllll
Query dlqllegllldadlxptrsaciaifearaqhpwpprvatlphwplIYAGALEGLDhlela  269
ident
Sbjct ---------------------------------------------RGFRIAIEKN----l  173
DSSP  ---------------------------------------------HHHHHHHHLL----l


DSSP  LLHHHHHHHHHH------------------------------------------------
```

```
Sbjct QLTEDFYEFVKEdprivlksaverithelfkimkektahkvirelyeygvleaiipeigr  233
DSSP  EELHHHHHHHHHlhhhhhhllhhhhhhhhhhhhhlllhhhhhhhhhhllhhhhhlhhhhh


DSSP  ------------------------------------------------------------
Query ------------------------------------------------------------  281
ident
Sbjct lrevkdpldehtlktleyleqviedrakylsaellenfgkkrvlgeftdvellkwgalfh  293
DSSP  hhlllllhhhhhhhhhhhhhhhhhlhhhhllhhhhhllllllllllllhhhhhhhhhhll


DSSP  ------------------------------------------------------------
Query ------------------------------------------------------------  281
ident
Sbjct digkpqtfafyehdkvgaqivreigerlrwgdeatefvaklvrhhlrpfflreafkkgel  353
DSSP  llllllllllllhhhhhhhhhhhhhhhllllhhhhhhhhhhhhlllhhhhhhhhhhlllll


DSSP  ------------------------------------------------------------
Query ------------------------------------------------------------  281
ident
Sbjct krrgmanfwrecgdiaphlfllsiadamasgdeeedikalmetiaelesfnrnemkxxxx  413
DSSP  lhhhhhhhhhhllllhhhhhhhhhhhhhhhlllhhhhhhhhhhhhhhhhhhhlllllllhh


DSSP  ---------------hhhhhhhllll
Query ---------------fvaridratkr  292
ident
Sbjct xxxxxxxxxxxxxxxxxxxxxxxxxx  439
DSSP  hhhhhhhhhhhllhhhhhhhhhhhhl
```

No 560: Query=mol1A Sbjct=3wfqG Z-score=4.8

back to top

```
DSSP  lllllllllhhhhhhhhhhhhhhllllhhhhhhhhhhhhHHHHHHHHLLLLllLLLLEEEE
Query aapysspptnlrslrdrltqvaerqgvvfgrlqrhvaxIVVAQFAATLTDdtGAPLLLVK   60
ident                                              |
Sbjct -------------------------------xxxlnfyLSYFDDVAKVLP--REHYCFIV   27
DSSP  -------------------------------lllllllLLLHHHHHHHLL--LLLLEEEE


DSSP  HHHHHHHHHLLLlllllLLEEEEEELLLHHHHHHHHHHHHHhllllleeeEELLLEElllll
Query GGSSLELRRGIPdsrtsKDFATVARRDIELIHEQLADAGEtgwegftaIFTAPEEiaapg  120
ident ||       | |      |       |        |                  |
Sbjct GGWVRDRILGEP-vgynIDVDFLTTADPVELAKNFAKRIG--------GHFFVFE-----   73
DSSP  LHHHHHHHHLLL-llleEEEEEEELLLHHHHHHHHHHHHL--------LEEEEEL-----


DSSP  llLLLEeEEEEEEelleEEEEEEEEEEEL-----LLLL--------------llllleEEE
Query xpVKPRrFTAKLSyrgrAFATVPIEVSSV-----EAGN--------------adqfdTLT  161
ident                           |       |
Sbjct --PTIA-SVVLHL----PPYRYRFDFSPLkgkdlEKALiedlkerdftanaiavnldDVL  126
DSSP  --LLEE-EEEEEL----LLLEEEEEEEELllllhHHHHhhhhllllhhhlleeehhHHL


DSSP  ElhhhhllllllleeeeelLHHH-----------hhhhhhHHHLlllllllLLLLHhhhh
Query SdalglvgvpaavavpcxTIPW-----------qiaqklhAVTAvleepkaNDRAHdlvd  210
ident |                                                    |
Sbjct S---------------tiVYDPtggikdleqgllrpvsieNLKR------dPVRVL----  161
DSSP  L---------------llEELLllhhhhhhlleellllhhHHHH------lLLHHH----


DSSP  hhhhhhllhhhhhhhhhhhhhhhhhllllllllllllllllhhhHHHHHHLLLLlllllL
Query lqllegllldadlxptrsaciaifearaqhpwpprvatlphwplIYAGALEGLDhlelaR  270
ident
Sbjct --------------------------------------------RGFRIAIEKN----lQ  173
DSSP  --------------------------------------------HHHHHHHHLL----lE


DSSP  LHHHHHHHHHH-------------------------------------------------
Query TVDAAAQAVQR-------------------------------------------------  281
ident         |
Sbjct LTEDFYEFVKEdprivlksaverithelfkimkektahkvirelyeygvleaiipeigrl  233
DSSP  ELHHHHHHHHHlhhhhhhllhhhhhhhhhhhhhlllhhhhhhhhhhllhhhhhlhhhhll
```

```
DSSP  ------------------------------------------------------------
Query ------------------------------------------------------------  281
ident 
Sbjct revkdpldehtlktleyleqviedrakylsaellenfgkkrvlgeftdvellkwgalfhd  293
DSSP  llllllhhhhhhhhhhhhhhhhhhllllllhhhhhllllllllllllhhhhhhhhhhlll


DSSP  ------------------------------------------------------------
Query ------------------------------------------------------------  281
ident 
Sbjct igkpqtfafyehdkvgaqivreigerlrwgdeatefvaklvrhhlrpfflreafkkgelk  353
DSSP  lllllllllllhhhhhhhhhhhhhhllllhhhhhhhhhhhhhlllhhhhhhhhhhlllll


DSSP  ------------------------------------------------------------
Query ------------------------------------------------------------  281
ident 
Sbjct rrgmanfwrecgdiaphlfllsiadamasgdeeedikalmetiaelesfnrnemkxxxxx  413
DSSP  hhhhhhhhhhllllhhhhhhhhhhhhhhllllhhhhhhhhhhhhhhhhhhhhllllllhh


DSSP  ---------------hhhhhhhllll
Query ---------------fvaridratkr  292
ident 
Sbjct xxxxxxxxxxxxxxxxxxxxxxxxxx  439
DSSP  hhhhhhhhhhhllhhhhhhhhhhhhl
```

No 561: Query=mol1A Sbjct=1lujA Z-score=4.8

back to top

```
DSSP  --------------------------------------------LLLL----llLLLLHH
Query --------------------------------------------AAPY----ssPPTNLR   12
ident 
Sbjct traipeltkllndedqvvvnkaavmvhqlskkeasrhaimrspqMVSAivrtmqNTNDVE   60
DSSP  lllhhhhhhhhllllhhhhhhhhhhhhhhhlllhhhhhhlllhhHHHHhhhhhhHLLLHH


DSSP  HHHHHHHHHHHHLL---------llhHHHHHHHHHHH--------------------hhh
Query SLRDRLTQVAERQG---------vvfGRLQRHVAXIV--------------------vaq   43
ident   |                       |
Sbjct TARCTAGTLHNLSHhregllaifksgGIPALVKMLGSpvdsvlfyaittlhnlllhqega  120
DSSP  HHHHHHHHHHHHLLlhhhhhhhhhllHHHHHHHHLLLllllhhhhhhhhhhhhhhhlllh


DSSP  hhhllllllllllleEEEH--------HHHHHHHHLlllllllleeeeellllhhhhhhhh
Query faatltddtgapllLVKG--------GSSLELRRGipdsrtskdfatvarrdieliheql   95
ident                                |
Sbjct kmavrlagglqkmvALLNktnvkflaITTDCLQIL-------------------------  155
DSSP  hhhhhhllhhhhhhHHHHlllhhhhhHHHHHHHHH-------------------------


DSSP  hhhhhhllleeeeellleellllllllllleeeeeeeeelleeeeeeeeeeeeellllll
Query adagetgwegftaiftapeeiaapgxpvkprrftaklsyrgrafatvpievssveagnad  155
ident 
Sbjct ------------------------------------------------------------  155
DSSP  ------------------------------------------------------------


DSSP  lleeeeelhhhhlllllleeeeeLLHHHHHHHHHHHHhllllllllllLLHHHHHHHHHH
Query qfdtltsdalglvgvpaavavpcXTIPWQIAQKLHAVtavleepkandRAHDLVDLQLLE  215
ident                           |                         |
Sbjct ----------aygnqeskliilaSGGPQALVNIMRTY----------tYEKLLWTTSRVL  195
DSSP  ----------hlllhhhhhhhhhLLHHHHHHHHHHHL----------lLHHHHHHHHHHH


DSSP  HHL--LHHHHHHHHHHHhhhhhhhllllllLLLLLlllhhhhhhhhhllllllllllLHH
Query GLL--LDADLXPTRSACiaifearaqhpwpPRVATlphwpliyagalegldhlelarTVD  273
ident   |            |                  |
Sbjct KVLsvCSSNKPAIVEAG--------gmqalGLHLT---------------------dPSQ  226
DSSP  HHHhlLLLHHHHHHHLL--------hhhhhHLLLL---------------------lLLH


DSSP  HHHHHHHHHHHHHHHLLLL-----------------------------------------
Query AAAQAVQRFVARIDRATKR-----------------------------------------  292
```

```
DSSP  HHHHHHHHHHHHHHHLLLLLllllhhhhhhhhhhllllhhhhhhhhhhhhhhllllhhhh


DSSP  ------------------------------------------------------------
Query ------------------------------------------------------------  292
ident
Sbjct mmvcqvggiealvrtvlragdreditepaicalrhltsrhqeaemaqnavrlhyglpvvv  346
DSSP  hhhhllhhhhhhhhhhhhlllhhhhhhhhhhhhhhlllllhhhhhhhhhhlllhhhhh


DSSP  ------------------------------------------------------------
Query ------------------------------------------------------------  292
ident
Sbjct kllhppshwplikatvglirnlalcpanhaplreqgaiprlvqllvrahqdtqrgvrmee  406
DSSP  hllllllhhhhhhhhhhhhhhlllhhhhhhhhhllhhhhhhhhhhhhhhhhlllllhhh


DSSP  ------------------------------------------------------------
Query ------------------------------------------------------------  292
ident
Sbjct ivegctgalhilardvhnrivirglntiplfvqllyspieniqrvaagvlcelaqdkeaa  466
DSSP  hhhhhhhhhhhlllhhhhhhhhhlllhhhhhhhhhlllhhhhhhhhhhhhhhlllhhhh


DSSP  -----------------------------------
Query -----------------------------------  292
ident
Sbjct eaieaegatapltellhsrnegvatyaaavlfrms  501
DSSP  hhhhhlllhhhhhllllllhhhhhhhhhhhhhll
```

No 562: Query=mol1A Sbjct=3wfrH Z-score=4.7

back to top

```
DSSP  lllllllllhhhhhhhhhhhhhhllllhhhhhhhhhhHHHHHHHHLLLLlLLLLlEEEE
Query aapysspptnlrslrdrltqvaerqgvvfgrlqrhvaxIVVAQFAATLTDdTGAPlLLVK   60
ident                                              |
Sbjct -------------------------------xxxlnfyLSYFDDVAKVLP-REHY-CFIV   27
DSSP  -------------------------------lllllllLLHHHHHHHHHLL-LLLL-EEEE


DSSP  HHHHHHHHHLLLlllllLLEEEEEELLLHHHHHHHHHHHHHhllllleeeEELLLEELLLLL
Query GGSSLELRRGIPdsrtsKDFATVARRDIELIHEQLADAGEtgwegftaIFTAPEEIAAPG  120
ident ||       | |      |       |        |                  |
Sbjct GGWVRDRILGEP-vgynIDVDFLTTADPVELAKNFAKRIG--------GHFFVFEKRGFL   78
DSSP  LHHHHHHHHLLL-llleEEEEEEEELLLHHHHHHHHHHHHHL--------LEELLEELLLLL


DSSP  LLLLLEeEEEEEEelleEEEEEEEEEEEL-----LLLL---------------llllEEE
Query XPVKPRrFTAKLSyrgrAFATVPIEVSSV-----EAGN---------------adqfDTL  160
ident                           |       |                      | |
Sbjct IKRPTIaSVVLHL----PPYRYRFDFSPLkgkdlEKALiedlkerdftanaiavnldDVL  134
DSSP  LLLLEEeEEEEEL----LLLEEEEEEEEEELllllhHHHhhhhllllhhhlleeehhHHL


DSSP  EElhhhhlllllleeeeelLHHH-----------hhhhhhhHHHLlllllllLLLLHhhh
Query TSdalglvgvpaavavpcxTIPW-----------qiaqklhAVTAvleepkaNDRAHdlv  209
ident                                                       |
Sbjct ST----------------iVYDPtggikdleqgllrpvsieNLKR------dPVRVL---  169
DSSP  LL----------------lLLLLllhhhhhhlleellllhhHHHH------lLLHHH---


DSSP  hhhhhhhllhhhhhhhhhhhhhhhhhhlllllllllllllhhhhHHHHHLLLllllll
Query dlqlleglllldadlxptrsaciaifearaqhpwpprvatlphwpliYAGALEGLdhlela  269
ident
Sbjct ---------------------------------------------rGFRIAIEK-----n  179
DSSP  ---------------------------------------------hHHHHHHHL-----l


DSSP  llHHHHHHHH--------------------------------------------------
Query rtVDAAAQAV--------------------------------------------------  279
ident
Sbjct lqLTEDFYEFvkedprivlksaverithelfkimkektahkvirelyeygvleaiipeig  239
DSSP  leELHHHHHHhhhlhhhhhhllhhhhhhhhhhhhhlllhhhhhhhhhhllhhhhhlhhhh
```

```
Query ------------------------------------------------------------  279
ident
Sbjct rlrevkdpldehtlktleyleqviedrakylsaellenfgkkrvlgeftdvellkwgalf  299
DSSP  hhhlllllhhhhhhhhhhhhhhhhhlhhhhllhhhhhlllllllllllhhhhhhhhhhl


DSSP  ------------------------------------------------------------
Query ------------------------------------------------------------  279
ident
Sbjct hdigkpqtfafyehdkvgaqivreigerlrwgdeatefvaklvrhhlrpfflreafkkge  359
DSSP  lllllllllllllhhhhhhhhhhhhhhhhhlllhhhhhhhhhhhhlllhhhhhhhhhhlll


DSSP  ------------------------------------------------------------
Query ------------------------------------------------------------  279
ident
Sbjct lkrrgmanfwrecgdiaphlfllsiadamasgdeeedikalmetiaelesfnrnemkeei  419
DSSP  llhhhhhhhhhhllllhhhhhhhhhhhhhhhllllhhhhhhhhhhhhhhhhhhhhlllhhhh


DSSP  -------------------------------hhhhhhhhhllll
Query -------------------------------qrfvaridratkr  292
ident
Sbjct qkxxxxxxxxxxxxxxxxxxxxxxxxxxxxxxxxxxxxxxxxxx  463
DSSP  hllhhhhhhhlllllhhhhhhhhhhhlllllhhhhhhhhhhhhl
```

No 563: Query=mol1A Sbjct=1jajA Z-score=4.7

back to top

```
DSSP  lllllllllhhhhhhhhhhhhhhlllLHHHHHHHHHHHHHHHHHhLLLLLLLLL---lE
Query aapysspptnlrslrdrltqvaerqgvVFGRLQRHVAXIVVAQFAaTLTDDTGAP---lL   57
ident                                 |
Sbjct ---------------------------MLTLIQGKKIVNHLRSRL-AFEYNGQLIkilsK   32
DSSP  ---------------------------LEEHHHHHHHHHHHHHHLE-EEEELLEEEeellL


DSSP  EEEhhHHHHHHHLLLlllllLLEEEEELL--LHHHHHHHHhhhhhHLLLlEEEEELLLEE
Query LVKggSSLELRRGIPdsrtsKDFATVARR--DIELIHEQLadageTGWEgFTAIFTAPEE  115
ident          |||         |                                     |
Sbjct NIV--AVGSLRREEK-mlndVDLLIIVPEkkLLKHVLPNI-----RIKG-LSFSVKVCGE   83
DSSP  LEE--EEEHHHHLLL-eellEEEEEEELLhhHHHLLLLEE-----EELL-LEEEEEEELL


DSSP  lllllllllEEEEEEEEELlEEEEEEEEEEEELLllllllleeeeelhhhhllllllee
Query iaapgxpvkpRRFTAKLSYRgRAFATVPIEVSSVEagnadqfdtltsdalglvgvpaava  175
ident           |                       |
Sbjct ----------RKCVLFIEWE-KKTYQLDLFTALAE-------------------------  107
DSSP  ----------LEEEEEEELL-LLLEEEEEEEEELL-------------------------


DSSP  eeellhhhhhhhhhhhhhhlllllllLLLLhhhhhhhhhhhhllhhhhhhhhhhhHHHH-
Query vpcxtipwqiaqklhavtavleepkaNDRAhdlvdlqlleglllldadlxptrsacIAIF-  234
ident
Sbjct -------------ekpyaifhftgpvSYLI-riraalkkknyklnqyglfknqtlVPLKi  153
DSSP  -------------lhhhhhhhhllhHHHH-hhhhhhhlleeeelleeeelleeELLLl


DSSP  -hhhllllLLLLLlllllhhhhhhhhhlllllllllllhhhhhhhhhhhhhhhhhllll
Query -earaqhpWPPRVatlphwpliyagalegldhlelartvdaaaqavqrfvaridratkr  292
ident
Sbjct ttekelikELGFT--------------------------------------yripkkrl  174
DSSP  llhhhhhhHHLLL--------------------------------------lllhhhll
```

No 564: Query=mol1A Sbjct=2z6gA Z-score=4.7

back to top

```
DSSP  ------------------------------------------------------------
Query ------------------------------------------------------------    0
ident
Sbjct epsqmlkhavvnlinyqddaelatraipeltkllndedqvvvnkaavmvhqlskkeasrh   60
```

```
DSSP  -------LLLL----llLLLLHHHHHHHHHHHHHHLL---------llhhHHHHHHHHHH
Query -------AAPY----ssPPTNLRSLRDRLTQVAERQG---------vvfgRLQRHVAXIV   40
ident                          |
Sbjct aimrspqMVSAivrtmqNTNDVETARCTSGTLHNLSHhregllaifksggIPALVNMLGS  120
DSSP  hhlllhhHHHHhhhhhhHLLLHHHHHHHHHHHHHHHLlhhhhhhhhhllhHHHHHHHLLL


DSSP  H--------------------hhhhhlllllllllleEEEH-------HHHHhHHHLlll
Query V--------------------aqfaatltddtgapllLVKG-------GSSLeLRRGipd   73
ident
Sbjct PvdsvlfhaittlhnlllhqegakmavrlagglqkmvALLNktnvkflAITTdCLQI---  177
DSSP  LlhhhhhhhhhhhhhhhhhhlllhhhhhhhllhhhhhHHHHlllhhhhHHHHhHHHH---


DSSP  llllleeeeelllhhhhhhhhhhhhhhhhlllleeeeellleelllllllllleeeeeeee
Query srtskdfatvarrdieliheqladagetgwegftaiftapeeiaapgxpvkprrftakls  133
ident
Sbjct ------------------------------------------------------------  177
DSSP  ------------------------------------------------------------


DSSP  elleeeeeeeeeeeeelllllllllleeeeelhhhhllllleeeeeLLHHHHHHHHHHHHh
Query yrgrafatvpievssveagnadqfdtltsdalglvgvpaavavpcXTIPWQIAQKLHAVt  193
ident                                                 |
Sbjct -------------------------------laygnqeskliilaSGGPQALVNIMRTY-  205
DSSP  -------------------------------hhlllhhhhhhhhlLLHHHHHHHHHLLL-


DSSP  llllllllllLLHHHHHHHHHHHHL--LHHHHHHHHHHHhhhhhhhllllllLLLLLLll
Query avleepkandRAHDLVDLQLLEGLL--LDADLXPTRSACiaifearaqhpwpPRVATLph  251
ident               |         |            |                  |
Sbjct ---------tYEKLLWTTSRVLKVLsvCSSNKPAIVEAG--------gmqalGLHLTD--  246
DSSP  ---------lLHHHHHHHHHHHHHHhlLLLHHHHHHHLL--------hhhhhHHHLLL--


DSSP  hhhhhhhhlllllllllllLHHHHHHHHHHHHHHHHHLLLL-------------------
Query wpliyagalegldhlelarTVDAAAQAVQRFVARIDRATKR-------------------  292
ident                          |           |
Sbjct -------------------PSQRLVQNCLWTLRNLSDAATKqegmegllgtlvqllgsdd  287
DSSP  -------------------LLHHHHHHHHHHHHHHHLLLLLllllhhhhhhhhhhlllll


DSSP  ------------------------------------------------------------
Query ------------------------------------------------------------  292
ident
Sbjct invvtcaagilsnltcnnyknkmmvcqvggiealvrtvlragdreditepaicalrhlts  347
DSSP  hhhhhhhhhhhhhhllllhhhhhhhhhlllhhhhhhhhhhhhhlllhhhhhhhhhhhlll


DSSP  ------------------------------------------------------------
Query ------------------------------------------------------------  292
ident
Sbjct rhqdaemaqnavrlhyglpvvvkllhppshwplikatvglirnlalcpanhaplreqgai  407
DSSP  lllhhhhhhhhhhlllhhhhhhllllllhhhhhhhhhhhhhhhhllhhhhhhhhhhllhh


DSSP  ------------------------------------------------------------
Query ------------------------------------------------------------  292
ident
Sbjct prlvqllvrahqdtqrrtsqfvegvrmeeiveactgalhilardihnrivirglntiplf  467
DSSP  hhhhhhhhhhhhhhhllllllllllhhhhhhhhhhhhhhhlllhhhhhhhhhhlllhhhh


DSSP  ------------------------------------------------------------
Query ------------------------------------------------------------  292
ident
Sbjct vqllyspieniqrvaagvlcelaqdkeaaeaieaegataplteTllhsrnegvatyaaavl  527
DSSP  hhhhlllhhhhhhhhhhhhhhhllhhhhhhhhhhlllhhhhhhhhhhlllhhhhhhhhhh


DSSP  -----------------------
Query -----------------------  292
ident
Sbjct frmsedkpqdykkrlsveltssl  550
DSSP  hhhhllllllhhhhhhhhhhhhl
```

back to top

```
DSSP  ----------------------------LLLL-----------lllLLLHHHHhhHHHH-
Query ----------------------------AAPY-----------sspPTNLRSLrdRLTQ-   20
ident                                                 |       |
Sbjct ktftevqterleqadrsvlikcpsklneKKLLqylsshgkidnyffFENRGIH--ALIEf   58
DSSP  llhhhhhhhhhhhhhleeeeellllllhHHHHhhhhlllleeeeeeEELLLEE--EEEEe


DSSP  ------------------------------------------------------------
Query ------------------------------------------------------------   20
ident
Sbjct sekssvaslqavtgipkhhvvpyksrlftftlknpgsqaeerpvkispqshipvnelipk  118
DSSP  lllhhhhhhhhllllllllllllllleeeeeelllllllllllllllllllllhhhhhhh


DSSP  --------------hhhhllLLHHHHHHHHHHHHHHHHHHLLLlllllLLEEEEHHhhhh
Query --------------vaerqgVVFGRLQRHVAXIVVAQFAATLTddtgaPLLLVKGGssle   66
ident                            |  |   |  ||         |   ||
Sbjct lchadsissqmyillneyqlTEENIKLRYLACSLVRDFARAYF-----PDSTVKPF--gs  171
DSSP  hhllllhhhhhhhhhhhhllLHHHHHHHHHHHHHHHHHHLLLL-----LLEEEEEE--hh


DSSP  hhhllllllllLLEEEEEL-------------------lLHHHHHHHHHHHHHHLLllEE
Query lrrgipdsrtsKDFATVAR-------------------rDIELIHEQLADAGETGWegFT  107
ident             |                              |     |
Sbjct svntfgklgcdVDMFLDFHdkkgpfemeyqmkrlpserlATQKILSIIGDCLDNFG--PG  229
DSSP  hllllllllllEEEEEEELllllleeeeelllllllhhhHHHHHHHHHHHHHHHLL--LL


DSSP  EE-ELLLEELlllllllLLEEEEEEEEEllleeEEEEEEEEEellllllllleeeeelhhh
Query AI-FTAPEEIaapgxpvKPRRFTAKLSYrgraFATVPIEVSsveagnadqfdtltsdalg  166
ident                                        ||
Sbjct YSsVQKILNA-------RCPLVKFSHQP---tGFQCDLSVS-------------------  260
DSSP  EEeEEEELLL-------LLLEEEEEELL---lLEEEEEEEL-------------------


DSSP  hlllllleeeeellhhhhhHHHHHHHHllllllllLLLLhhhhhhhhhhhhllhhhhhhh
Query lvgvpaavavpcxtipwqiAQKLHAVTavleepkaNDRAhdlvdlqlleglllldadlxpt  226
ident
Sbjct -----------------nsIAIRCSEL--------LYIY---------------------  274
DSSP  -----------------lhHHHHHHHH--------HHHH---------------------


DSSP  hhhhhhhhhhllllllllllllllLHHHhHHHHHL-lLLLL------------------l
Query rsaciaifearaqhpwpprvatlpHWPLiYAGALE-gLDHL------------------e  267
ident
Sbjct -----------------------gCLDPrVRALVFslRCWArvhgltnsvpgtwitnfsl  311
DSSP  -----------------------hHHLHhHHHHHHhhHHHHhhlllllllllllllhhhh


DSSP  lllLHHHHHHH-------------------------------------------------
Query larTVDAAAQA-------------------------------------------------  278
ident
Sbjct tmmIMFFLQKRsppiiptldqlkeladekdkhviggydcsfvsdlskikptkntetldel  371
DSSP  hhhHHHHHHLLlllllllhhhhhhllllllleelleelllllllhhhllllllllllhhhh


DSSP  ------------------hhhhhhhhhHLLLL----------------------------
Query ------------------vqrfvaridRATKR----------------------------  292
ident
Sbjct lcdffqyfgnfdfrknslnlrkgkevnKPESSplyiwnpfeqdlnisknvnqpqlekfva  431
DSSP  hhhhhhhhlllllleeeellllleeeeLLLLLlllllllllllllllllllhhhhhhhhh


DSSP  ---------------------------------
Query ---------------------------------  292
ident
Sbjct maresawilqkedktqqminkepwglaavlipf  464
DSSP  hhhhhhhhhhlllhhhhhhllllllhhhhllll
```

No 566: Query=mol1A Sbjct=5a2xA Z-score=4.7

back to top

```
ident                                                  |       |
Sbjct kktftevqterleqadrsvlikcpsklneKKLLqylsshgkidnyffFENRGIH--ALIE   58
DSSP  lllhhhhhhhhhhhhhleeeeellllllhHHHHhhhhlllleeeeeeEELLLEE--EEEE


DSSP  ------------------------------------------------------------
Query ------------------------------------------------------------   20
ident
Sbjct fsekssvaslqavtgipkaaehhvvpyksrlftftlknpgsqaaeerpvkispqshipvn  118
DSSP  elllhhhhhhhhlllllhhhllllllllllleeeeeellllllllllllllllllllhh


DSSP  -------------------hhhhllLLHHHHHHHHHHHHHHHHHHLLLlllllLLEEEEH
Query -------------------vaerqgVVFGRLQRHVAXIVVAQFAATLTddtgaPLLLVKG   61
ident                                 |  |   |  ||         |   ||
Sbjct elipklchadsissqmyillneyqlTEENIKLRYLACSLVRDFARAYF-----PDSTVKP  173
DSSP  hhhhhhhllllhhhhhhhhhhhhllLHHHHHHHHHHHHHHHHHHHHLLL-----LLLEEEE


DSSP  HhhhhhhhlllllllLLEEEEEL------------------lLHHHHHHHHHHHHHHHLL
Query GsslelrrgipdsrtsKDFATVAR------------------rDIELIHEQLADAGETGW  103
ident                  |                             |     |
Sbjct F--gssvntfgklgcdVDMFLDFHdkgpfemeyqmkrlpserlATQKILSIIGDCLDNFG  231
DSSP  E--hhhllllllllllEEEEEELLllllleeeeellllllhhhHHHHHHHHHHHHHHHLL


DSSP  llEEEE-ELLLEELlllllllLLEEEEEEEEEElleeEEEEEEEEEelllllllleeeee
Query egFTAI-FTAPEEIaapgxpvKPRRFTAKLSYrgraFATVPIEVSsveagnadqfdtlts  162
ident                                            ||
Sbjct --PGYSsVQKILNA-------RCPLVKFSHQP---tGFQCDLSVS---------------  264
DSSP  --LLEEeEEEELLL-------LLLEEEEEELL---lLLEEEEEEL---------------


DSSP  lhhhhlllllleeeeellhhhhhHHHHHHHHlllllllLLLLhhhhhhhhhhhhllhhh
Query dalglvgvpaavavpcxtipwqiAQKLHAVTavleepkaNDRAhdlvdlqllegllldad  222
ident
Sbjct ---------------------nsIAIRCSEL--------LYIY-----------------  278
DSSP  ---------------------lhHHHHHHHH--------HHHH-----------------


DSSP  hhhhhhhhhhhhhhhlllllllllllLHHHhHHHHHL-lLLLL---------------
Query lxptrsaciaifearaqhpwpprvatlpHWPLiYAGALE-gLDHL---------------  266
ident
Sbjct ---------------------------gCLDPrVRALVFslRCWArvhgltnsvpgtwit  311
DSSP  ---------------------------hHHLHhHHHHHHhhHHHHhhllllllllllll


DSSP  ---llllLHHHHHHH---------------------------------------------
Query ---elarTVDAAAQA---------------------------------------------  278
ident
Sbjct nfsltmmIMFFLQKRsppiiptldqlkeladekdkhviggydcsfvsdlskikptkntet  371
DSSP  hhhhhhhHHHHHHLLlllllllhhhhhhhllhhhlleelleellllllhhhllllllll


DSSP  ---------------------hhhhhhhhhHLLLL-------------------------
Query ---------------------vqrfvaridRATKR-------------------------  292
ident
Sbjct ldellcdffqyfgnfdfrknslnlrkgkevNKPESsplyiwnpfeqdlnisknvnqpqle  431
DSSP  hhhhhhhhhhhhhlllllleeellllleeeLLLLLllleelllllllllllllhhhhh


DSSP  -------------------------------------
Query -------------------------------------  292
ident
Sbjct kfvamaresawilqkedktqqminkepwglaavlipf  468
DSSP  hhhhhhhhhhhhhhllhhhhhhllllllhhhhllll
```

No 567: Query=mol1A Sbjct=5a2xB Z-score=4.7

back to top

```
DSSP  ------------------------------LLLLL-----------llllLHHHhhHHHH
Query ------------------------------AAPYS-----------spptNLRSlrDRLT   19
ident                                                   |       |
Sbjct rkktftevqterleqadrsvlikcpsklneKKLLQylsshgkidnyfffeNRGI--HALI   58
```

```
DSSP  ------------------------------------------------------------
Query ------------------------------------------------------------   19
ident                                                               
Sbjct efsekssvaslqavtgipkhvvpyksrlftftlknpgsqaeerpvkispqshipvnelip  118
DSSP  eelllhhhhhhhhlllllllllllleeeeeellhhhllllllllllllllllhhhhhh


DSSP  --------------hhhhhllLLHHHHHHHHHHHHHHHHHHLLLlllllLLEEEEHHhhh
Query --------------qvaerqgVVFGRLQRHVAXIVVAQFAATLTddtgaPLLLVKGGssl   65
ident                             |  |   |  ||         |   ||      
Sbjct klchadsissqmyillneyqlTEENIKLRYLACSLVRDFARAYF-----PDSTVKPF--g  171
DSSP  hhhllllhhhhhhhhhhhhllLHHHHHHHHHHHHHHHHHHHHLLL-----LLEEEEEE--h


DSSP  hhhhlllllllllLLEEEEEL--------------------lLHHHHHHHHHHHHHHLLll
Query elrrgipdsrtsKDFATVAR--------------------rDIELIHEQLADAGETGWeg  105
ident              |                               |     |         
Sbjct ssvntfgklgcdVDMFLDFHdmkkgpfemeyqmkrlpserlATQKILSIIGDCLDNFG--  229
DSSP  hhllllllllllEEEEEEELllllllleeeeellllllllhhhHHHHHHHHHHHHHHHLL--


DSSP  EEEE-ELLLEELlllllllLLEEEEEEEEEElleeEEEEEEEEEellllllllleeeeelh
Query FTAI-FTAPEEIaapgxpvKPRRFTAKLSYrgraFATVPIEVSsveagnadqfdtltsda  164
ident                                          ||                  
Sbjct PGYSsVQKILNA-------RCPLVKFSHQP---tGFQCDLSVS-----------------  262
DSSP  LLEEeEEEELLL-------LLLEEEEEELL---lLLEEEEEEL-----------------


DSSP  hhhllllllleeeeellhhhhhHHHHHHHHllllllllLLLLhhhhhhhhhhhhllhhhhh
Query lglvgvpaavavpcxtipwqiAQKLHAVTavleepkaNDRAhdlvdlqllegllldadlx  224
ident                                                               
Sbjct -------------------nsIAIRCSEL--------LYIY-------------------  276
DSSP  -------------------lhHHHHHHHH--------HHHH-------------------


DSSP  hhhhhhhhhhhhhllllllllllllLHHHhHHHHHL-lLLLL-----------------
Query ptrsaciaifearaqhpwpprvatlpHWPLiYAGALE-gLDHL-----------------  266
ident                                                               
Sbjct -------------------------gCLDPrVRALVFslRCWArvhgltnsvpgtwitnf  311
DSSP  -------------------------hHHLHhHHHHHHhhHHHHhhllllllllllllhh


DSSP  -llllLHHHHHHH-----------------------------------------------
Query -elarTVDAAAQA-----------------------------------------------  278
ident                                                               
Sbjct sltmmIMFFLQKRsppiiptldqlkeladekdkhviggydcsfvsdlskikptkntetld  371
DSSP  hhhhhHHHHHHHLLllllllllhhhhhhhllhhhlleelleelllllhhhlllllllllhh


DSSP  -------------------hhhhhhhhhHLLLL---------------------------
Query -------------------vqrfvaridRATKR---------------------------  292
ident                                                               
Sbjct ellcdffqyfgnfdfrknslnlrkgkevNKPESsplyiwnpfeqdlnisknvnqpqlekf  431
DSSP  hhhhhhhhhhhllllllleeellllleeeLLLLLlllleellllllllllllllhhhhhhh


DSSP  ------------------------------------
Query ------------------------------------  292
ident                                     
Sbjct vamaresawilqkedktqqminkepwglaavlipfg  467
DSSP  hhhhhhhhhhhhlllhhhhhhhlllllhhhhlllll
```

No 568: Query=mol1A Sbjct=4e80D Z-score=4.7

back to top

```
DSSP  llllllllllhhhhhhhhhhhhhhllLLHHHHHHHHHHHHHHHHHHLLLlllllLLEEEE
Query aapysspptnlrslrdrltqvaerqgVVFGRLQRHVAXIVVAQFAATLTddtgaPLLLVK   60
ident                                     |                 |     
Sbjct ---------hkeftkfcyevyneikiSDKEFKEKRAALDTLRLCLKRIS-----PDAELV   46
DSSP  ---------lhhhhhhhhhhhhhhhllLHHHHHHHHHHHHHHHHHHHHHHL-----LLLEEE


DSSP  HHhhhhhhhllllllllLLEEEEEELllhHHHHHHHHHHHHHHLllleEEEELLLEEllll
```

```
Sbjct AF--gslesglalknsdMDLCVLMD---DTIALQFYEELIAE----GFEGKFLQR-----   92
DSSP  EE--lhhhhlllllllEEEEEELL---LLHHHHHHHHHHHH----LLLEEEEEE-----


DSSP  lllLLEEEEEEEeelleeEEEEEEEEEelllllllleeeeelhhhhllllllleeeeell
Query xpvKPRRFTAKLsyrgraFATVPIEVSsveagnadqfdtltsdalglvgvpaavavpcxt  180
ident                        |
Sbjct --aRIPIIKLTS------SFQCDIGFN---------------------------------  111
DSSP  --lLEEEEEELL------LLLEEEEEL---------------------------------


DSSP  hhhhhhHHHHHHhllllllllLLLLhhhhhhhhhhhhllhhhhhhhhhhhhhhhhhhlll
Query ipwqiaQKLHAVtavleepkaNDRAhdlvdlqlleglllldadlxptrsaciaifearaqh  240
ident
Sbjct --nrlaIHNTLL---------LSSY-----------------------------------  125
DSSP  --lhhhHHHHHH---------HHHH-----------------------------------


DSSP  llllllllllLHHHhHHHHHL-lLLLL-----------------llllLHHHHHH-----
Query pwpprvatlpHWPLiYAGALE-gLDHL-----------------elarTVDAAAQ-----  277
ident
Sbjct --------tkLDAR-LKPMVLlvKHWAkrkqinspyfgtlssygyvlmVLYYLIHvikpp  176
DSSP  --------hhHLLL-HHHHHHhhHHHHhhllllllhhhllllhhhhhhhHHHHHHHlllll


DSSP  ------------------------------------------------------------
Query ------------------------------------------------------------  277
ident
Sbjct vfpnlllsplkqekivdgfdvgfddkledippsqnysslgsllhgffrfyaykfeprekv  236
DSSP  llllllllllllleelleelllllllhhhlllllllllllhhhhhhhhhhhhhhlllllle


DSSP  hhHHHH----hhhhHLLLL-----------------------------------------
Query avQRFV----aridRATKR-----------------------------------------  292
ident    |
Sbjct vtFRRPdgyltkqeKGWTSatehtgsadkdryilaiedpfeishnvgrtvsssglyrirg  296
DSSP  elLLLLlllllhhhHLLLLllllllllllllllleelllllllllllllllhhhhhhhhh


DSSP  ----------------------------
Query ----------------------------  292
ident
Sbjct efmaasrllnsrsypipydslfeeapip  324
DSSP  hhhhhhhhhlllllllllhhhhhllllll
```

No 569: Query=mol1A Sbjct=4nkuB Z-score=4.7

back to top

```
DSSP  llllllllllhhhhhhhhhhhhhhhllLLHHHHHHHHHHHHHHHHHHHHLLLllllllLLEEEE
Query aapysspptnlrslrdrltqvaerqgVVFGRLQRHVAXIVVAQFAATLTddtgaPLLLVK   60
ident                                     |                 |
Sbjct -------mshkeftkfcyevyneikiSDKEFKEKRAALDTLRLCLKRIS-----PDAELV   48
DSSP  -------lllhhhhhhhhhhhhhhhllLHHHHHHHHHHHHHHHHHHHHHHLLL-----LLLEEE


DSSP  HHhhhhhhhlllllllllLLEEEEEELllhhHHHHHHHHHHHHHLllleEEEELLLEElllll
Query GGsslelrrgipdsrtsKDFATVARrdieLIHEQLADAGETGwegfTAIFTAPEEiaapg  120
ident                   |           |  |
Sbjct AF--gslesglalknsdMDLCVLMD----TIALQFYEELIAE----GFEGKFLQI-----   93
DSSP  EE--hhhhhllllllllEEEEEELL----LHHHHHHHHHHHHL----LLLEEEELL-----


DSSP  lllllEEEEEEEEeelleeeEEEEEEEEelllllllleeeeelhhhhllllllleeeeell
Query xpvkpRRFTAKLsyrgrafATVPIEVSsveagnadqfdtltsdalglvgvpaavavpcxt  180
ident                        |
Sbjct -----PIIKLTS-------FQCAIGFN---------------------------------  108
DSSP  -----LEEEELL-------LLEEEEEL---------------------------------


DSSP  hhhhhhHHHHHHhllllllllLLLLhhhhhhhhhhhhllhhhhhhhhhhhhhhhhhhlll
Query ipwqiaQKLHAVtavleepkaNDRAhdlvdlqlleglllldadlxptrsaciaifearaqh  240
ident
Sbjct --nrlaIHNTLL---------LSSY-----------------------------------  122
DSSP  --lhhhHHHHHH---------HHHH-----------------------------------
```

```
DSSP  llllllllllLHHHhHHHHHLL-LLLL-----------------llllLHHHHHH-----
Query pwpprvatlpHWPLiYAGALEG-LDHL-----------------elarTVDAAAQ-----  277
ident
Sbjct --------tkLDAR-LKPMVLLvKHWAkrkqinspyfgtlssygyvlmVLYYLIHvikpp  173
DSSP  --------hhHLLL-HHHHHHHhHHHHhhlllllhhhllllhhhhhhhHHHHHHHlllll


DSSP  ------------------------------------------------------------
Query ------------------------------------------------------------  277
ident
Sbjct vfpnlllsplkqekivdgfdvgfddkledippsqnysslgsllhgffrfyaykfeprekv  233
DSSP  llllhhhllllllleelleellllllhhhlllllllllhhhhhhhhhhhhhhllllllle


DSSP  hhHHHH---hhhhHLLLL------------------------------------------
Query avQRFV---aridRATKR------------------------------------------  292
ident     |
Sbjct vtFRRPdgyltkqEKGWTsatdryilaiedpfeishnvgrtvsssglyrirgefmaasrl  293
DSSP  elLLLLlllllhhHHLLLllllllllleelllllllllhhhhllhhhhhhhhhhhhhhhhh


DSSP  -----------------
Query -----------------  292
ident
Sbjct lnsrsypipydslfeea  310
DSSP  hllllllllhhhhhlll
```

No 570: Query=mol1A Sbjct=5a2vB Z-score=4.7

back to top

```
DSSP  ----------------------------LLLL-----------lllLLLHHHHhhHHHH-
Query ----------------------------AAPY-----------sspPTNLRSLrdRLTQ-   20
ident                                                 |       |
Sbjct ktftevqterleqadrsvlikcpsklneKKLLqylsshgkidnyffFENRGIH--ALIEf   58
DSSP  llhhhhhhhhhhhhhleeeeeelllllhHHHHhhhhllleeeeeeeEELLLEE--EEEEe


DSSP  ------------------------------------------------------------
Query ------------------------------------------------------------   20
ident
Sbjct sekssvaslqavtgipkhvvpyksrlftftlknpgsqaaeerpvkispqshipvnelipk  118
DSSP  lllhhhhhhhhllllllllllllleeeeellllhhhllllllllllllllllllhhhhhhh


DSSP  --------------hhhhllLLHHHHHHHHHHHHHHHHHHLLLlllllLLEEEEhhHHHH
Query --------------vaerqgVVFGRLQRHVAXIVVAQFAATLTddtgaPLLLVKggSSLE   66
ident                            |  |   |  ||         |   ||
Sbjct lchadsissqmyillneyqlTEENIKLRYLACSLVRDFARAYF-----PDSTVK--PFGS  171
DSSP  hhllllhhhhhhhhhhhhllLHHHHHHHHHHHHHHHHHHHLLL-----LLEEEE--EEHH


DSSP  hhhlllLLLL----lLLEEEEEL---------------------------lLHHHHHHHH
Query lrrgipDSRT----sKDFATVAR---------------------------rDIELIHEQL   95
ident                 |                                      |
Sbjct ----svNTFGklgcdVDMFLDFHdiqkhatkmkkgpfemeyqmkrlpserlATQKILSII  227
DSSP  ----hlLLLLlllllEEEEEEELlllllllllllleeeeelllllllhhhHHHHHHHHH


DSSP  HHHH-HHLLllEEEEELLLEELlllllllLLEEEEEEEEEllaaEEEEEEEEEellllll
Query ADAG-ETGWegFTAIFTAPEEIaapgxpvKPRRFTAKLSYrgraFATVPIEVSsveagna  154
ident  |     |                                           ||
Sbjct GDCLdNFGP--GYSSVQKILNA-------RCPLVKFSHQP---tGFQCDLSVS-------  268
DSSP  HHHHhHHLL--LEEEEEEELLL-------LLLEEEEEELL---lLEEEEEEEL-------


DSSP  llleeeeelhhhhllllleeeeellhhhhhhhhhhhhhlllllllllllllhHHHHhhhh
Query dqfdtltsdalglvgvpaavavpcxtipwqiaqklhavtavleepkandrahDLVDlqll  214
ident                                                      |
Sbjct --------------------------------------------nsiaircsELLY----  280
DSSP  --------------------------------------------lhhhhhhhHHHH----


DSSP  hhhllhhhhhhhhhhhhhhhhhhhllllllllllllHHHHHHHHHL-lLLLL------
Query eglllldadlxptrsaciaifearaqhpwpprvatlphWPLIYAGALE-gLDHL------  266
```

```
DSSP  ---------------------------------hhhhHLHHHHHHHHhhHHHHhhlllll


DSSP  -----------lLLLLHHHHHH--------------------------------------
Query -----------eLARTVDAAAQ--------------------------------------  277
ident
Sbjct svpgtwitnfslTMMIMFFLQKrsppiiptldqlkeladekdkhviggydcsfvsdlski  367
DSSP  llllllllhhhhHHHHHHHHHLllllllllhhhhhhlllhhhlleelleellllllhhhl


DSSP  -------------------------------HHHHHHHhhHHLLL---------------
Query -------------------------------AVQRFVAriDRATK---------------  291
ident
Sbjct kptkntetldellcdffqyfgnfdfrknslnLRKGKEV--NKPESsplyiwnpfeqdlni  425
DSSP  llllllllhhhhhhhhhhhhhhllllleeeeLLLLEEE--ELLLLlllllllllllllll


DSSP  ----------------------------------------------l
Query ----------------------------------------------r  292
ident
Sbjct sknvnqpqlekfvamaresawilqkedktqqminkepwglaavlipf  472
DSSP  lllllhhhhhhhhhhhhhhhhhhhllhhhhhhlllllllhhhhllll
```

No 571: Query=mol1A Sbjct=4i2iA Z-score=4.7

back to top

```
DSSP  ------------------------------------------------------------
Query ------------------------------------------------------------    0
ident
Sbjct kkisqyacqrrttlnnynqlftdaldilaendelrenegsclafmrassvlkslpfpits   60
DSSP  llllllhhhllllllllllhhhhhhhhhhhhllllllllllhhhhhhhhhhhhhhllllll


DSSP  ------------------------------------------------------------
Query ------------------------------------------------------------    0
ident
Sbjct mkdtegipclgdkvksiiegiiedgesseakavlnderyksfklftsvfgvglktaekwf  120
DSSP  hhhhlllllllhhhhhhhhhhhhhhlllhhhhhhllhhhhhhhhhhllllllhhhhhhhh


DSSP  ---llllllllllhhhhhhhhhhhhhhllllhHHHHHHHHHHHHHHHHHL-LLLLlLLLL
Query ---aapysspptnlrslrdrltqvaerqgvvfGRLQRHVAXIVVAQFAAT-LTDDtGAPL   56
ident                                            |               |
Sbjct rmgfrtlskiqsdkslrftqmqkagflyyedlVSCVNRPEAEAVSMLVKEaVVTFlPDAL  180
DSSP  hlllllhhhhhllllllllhhhhhhhhlhhhhHLLEEHHHHHHHHHHHHHhHHHHlLLLE


DSSP  EEEEHHHHHHHhhllllllLLLLEEEEEL----------lLHHHHHHHHHHHHhhlllle
Query LLVKGGSSLELrrgipdsrTSKDFATVAR----------rDIELIHEQLADAGetgwegf  106
ident     ||             |  |                             |
Sbjct VTMTGGFRRGK-------mTGHDVDFLITspeatedeeqqLLHKVTDFWKQQG-------  226
DSSP  EEELHHHHLLL-------lEELLEEEEEElllllhhhhhhHHHHHHHHHHHHL-------


DSSP  eeEELLLEEllllllLLLLEEEEEEEEEL-----lEEEEEEEEEEEEL-LLLLlllleee
Query taIFTAPEEiaapgxPVKPRRFTAKLSYR-----gRAFATVPIEVSSV-EAGNadqfdtl  160
ident                         ||
Sbjct --LLLYCDI------HFQKCFLILKLDHGrvhsekKGWKAIRVDLVMCpYDRR-------  271
DSSP  --LEEEEEL------LLEEEEEEEEEEHHhlllllLLEEEEEEEEEELlHHHH-------


DSSP  eelhhhhlllllleeeeellhHHHHhhhhhhhhllllllllllllHHHHHHH--------
Query tsdalglvgvpaavavpcxtiPWQIaqklhavtavleepkandraHDLVDLQ--------  212
ident
Sbjct --------------------aFALL----------------gwtgSRQFERDlrryathe  295
DSSP  --------------------hHHHH----------------hhhlLHHHHHHhhhhhhhh


DSSP  --------hhhhhllhhHHHHhhHHHHhhhhhhlllLLLLLLlllllhhhhhhhhhllll
Query --------llegllldaDLXPtrSACIaifearaqhPWPPRVatlphwpliyagalegld  264
ident                   |
Sbjct rkmmldnhalydrtkrvFLEA--ESEE-----eifaHLGLDY------------------  330
DSSP  hleeellleeellllleEELL--LLHH-----hhhhHLLLLL------------------
```

```
Query hlelartvdaaaqavqrfvaridratkr  292
ident
Sbjct --------------------iepwerna  338
DSSP  --------------------llhhhlll
```

No 572: Query=mol1A Sbjct=2m2vA Z-score=4.7

back to top

```
DSSP  lllllllllhhhhhhhhhhhhhhllllhhhhhHHHHHHHHHHHHHLL------llLLLL
Query aapysspptnlrslrdrltqvaerqgvvfgrlqRHVAXIVVAQFAATL------tdDTGA   54
ident
Sbjct -------------------------------mlTLIQGKKIVNHLRSRlafeyngqLIKI   29
DSSP  -------------------------------leEHHHHHHHHHHHHHHleeeelleEEEL


DSSP  L--LEEEEHHHHHHHhhllllllLLLLEEEEEL--------lLHHHHHhhhHHHHhhlll
Query P--LLLVKGGSSLELrrgipdsrTSKDFATVAR--------rDIELIHeqlADAGetgwe  104
ident         |    |            |                   |
Sbjct LskNIVAVGSLRREE-------kMLNDVDLLIIvpekkllkhVLPNIR---IKGL-----   74
DSSP  LhhHEEEELHHHHLL-------lEELLEEEEEEellllhhhhLLLLEE---LLLL-----


DSSP  leeeEELLlEELLLlllllLLEEEEEEEEellee EEEEEEEEEEELLLLLllllleeeeelh
Query gftaIFTApEEIAApgxpvKPRRFTAKLSyrgraFATVPIEVSSVEAGNadqfdtltsda  164
ident      |                              |         |
Sbjct ----SFSV-KVCGE-----RKCVLFIEWE-----KKTYQLDLFTALAEE-----------  108
DSSP  ----LLLE-EEELL-----LEEEEEEEEL-----LEEEEEEEEEEEHHH-----------


DSSP  hhhllllllleeeeellhHHHHHHHHhhhhllllllllllllhHHHHHH-----------
Query lglvgvpaavavpcxtiPWQIAQKLhavtavleepkandrahDLVDLQ------------  212
ident                                               |
Sbjct ---------------kpYAIFHFTG-----------------PVSYLIriraalkkknyk  136
DSSP  ---------------hhHHHHHHHL-----------------LHHHHHhhhhhhhlllle


DSSP  ----hhhhhllhHHHHHhhHHHHhhhhhhllllLLLLLlllllhhhhhhhhhlllllll
Query ----llegllldADLXPtrSACIaifearaqhpWPPRVatlphwpliyagalegldhlel  268
ident               |
Sbjct lnqyglfknqtlVPLKI--TTEK-----elikeLGFTY----------------------  167
DSSP  eelleeeelleeELLLL--LLHH-----hhhhhHLLLL----------------------


DSSP  lllhhhhhhhhhhhhhhhhhllll
Query artvdaaaqavqrfvaridratkr  292
ident
Sbjct -----------------ripkkrl  174
DSSP  -----------------lllllll
```

No 573: Query=mol1A Sbjct=3wfqE Z-score=4.7

back to top

```
DSSP  lllllllllhhhhhhhhhhhhhhllllhhhhhhhhhhHHHHHHHHHLLLLllLLLLEEEE
Query aapysspptnlrslrdrltqvaerqgvvfgrlqrhvaxIVVAQFAATLTDdtGAPLLLVK   60
ident                                              |
Sbjct ------------------------------xxxglnfyLSYFDDVAKVLP--REHYCFIV   28
DSSP  ------------------------------llllllllLLLHHHHHHHLL--LLLLEEEE


DSSP  HHHHHHHHHLLLlllllLLEEEEEELLLHHHHHHHHHHHHhhllllleeeEELLLEELllll
Query GGSSLELRRGIPdsrtsKDFATVARRDIELIHEQLADAGetgwegftaIFTAPEEIaapg  120
ident ||       | |      |       |        |
Sbjct GGWVRDRILGEP-vgynIDVDFLTTADPVELAKNFAKRI---------GGHFFVFE----   74
DSSP  LHHHHHHHHLLL-lllleEEEEEEELLLHHHHHHHHHHHH---------LLEEEEEL----


DSSP  llLLLEEEEEEEeelleEEEEEEEEEEEL-----LLLL---------------lllLEEE
Query xpVKPRRFTAKLsyrgrAFATVPIEVSSV-----EAGN---------------adqFDTL  160
ident            |              |       |                      | |
Sbjct --PTIASVVLHL-----PPYRYRFDFSPLkgkdlEKALiedlkerdftanaiavnlDDVL  127
```

```
DSSP  eelhhhhllllllleeeeelLHHH-----------hhhhhhhHHHLllllllLLLLhhhh
Query tsdalglvgvpaavavpcxTIPW-----------qiaqklhAVTAvleepkaNDRAhdlv  209
ident                                                       |
Sbjct -----------------tiVYDPtggikdleqgllrpvsieNLKR------dPVRV----  160
DSSP  -----------------llEELLllhhhhhhhleellllhhHHHH------lLLHH----


DSSP  hhhhhhhhllhhhhhhhhhhhhhhhhhhllllllllllllllhhhHHHHHHLLLLlllll
Query dlqllegllldadlxptrsaciaifearaqhpwpprvatlphwplIYAGALEGLDhlela  269
ident
Sbjct --------------------------------------------lRGFRIAIEKN----l  172
DSSP  --------------------------------------------hHHHHHHHHLL----l


DSSP  LLHHHHHHHHHH------------------------------------------------
Query RTVDAAAQAVQR------------------------------------------------  281
ident          |
Sbjct QLTEDFYEFVKEdprivlksaverithelfkimkektahkvirelyeygvleaiipeigr  232
DSSP  EELHHHHHHHHHlhhhhhhllhhhhhhhhhhhhhllllhhhhhhhhhhllhhhhhlhhhhh


DSSP  ------------------------------------------------------------
Query ------------------------------------------------------------  281
ident
Sbjct lrevkdpldehtlktleyleqviedrakylsaellenfgkkrvlgeftdvellkwgalfh  292
DSSP  hhhllllhhhhhhhhhhhhhhhhhhlhhhlllhhhhlllllleellleehhhhhhhhhhll


DSSP  ------------------------------------------------------------
Query ------------------------------------------------------------  281
ident
Sbjct digkpqtfafyehdkvgaqivreigerlrwgdeatefvaklvrhhlrpfflreafkkgel  352
DSSP  lllllllllllhhhhhhhhhhhhhhhllllhhhhhhhhhhhhlllhhhhhhhhhhllll


DSSP  ------------------------------------------------------------
Query ------------------------------------------------------------  281
ident
Sbjct krrgmanfwrecgdiaphlfllsiadamasgdeeedikalmetiaelesfnrnemkxxxx  412
DSSP  lhhhhhhhhhhllllhhhhhhhhhhhhhhllllhhhhhhhhhhhhhhhhhhhllllllhhh


DSSP  ----------------hhhhhhhllll
Query ----------------fvaridratkr  292
ident
Sbjct xxxxxxxxxxxxxxxxxxxxxxxxxxx  439
DSSP  hhhhhhhhhhhhllhhhhhhhhhhhhl
```

No 574: Query=mol1A Sbjct=1q78A Z-score=4.6

back to top

```
DSSP  ---------llllllllllhhhhhhhhhhhhhhlLLLHHHHHHHHHHHHHHhHHHHLLLL-
Query ---------aapysspptnlrslrdrltqvaerqGVVFGRLQRHVAXIVVaQFAATLTD-   50
ident                                           |
Sbjct hygitspislaapketdxlltqklvetlkpfgvfEEEEELQRRILILGKLnNLVKEWIRe   60
DSSP  llllllllllllllhhhhhhhhhhhhhhhhhlllLLHHHHHHHHHHHHHHHhHHHHHHHHHh


DSSP  -----------llLLLLeEEEHHhhhhhhhllllllllLLEEEEEEL--LLHHHHHHHHHH
Query -----------dtGAPLlLVKGGsslelrrgipdsrtsKDFATVAR--RDIELIHEQLAD   97
ident                                        |   ||    |         |
Sbjct isesknlpqsvieNVGG-KIFTF--gsyrlgvhtkgadIDALCVAPrhVDRSDFFTSFYD  117
DSSP  hhhhllllhhhhlLLLL-EEEEE--hhhhhllllllllEEEEEEELllLLHHHHLLHHHH


DSSP  HHHHLllLEEEEEELLLEELLlllllllLEEEEEEEEelleeEEEEEEEEEEELLLllLLLL
Query AGETGweGFTAIFTAPEEIAapgxpvkPRRFTAKLSyrgraFATVPIEVSSVEAgnADQF  157
ident               | ||                            |
Sbjct KLKLQ--EEVKDLRAVEEAF-------VPVIKLCFD-----GIEIDILFARLALqtIPED  163
DSSP  HHHLL--LLEEEEEEEELLLL-------LLEEEEEEL-----LEEEEEEEEEELLLllLLLL


DSSP  EeeeelhhhhllllllleeeeellhhhhhhhhhhhhhllllllllllllhhhhHHHHHHhh
```

```
Sbjct L------------------------DLRD--dsllknldirxirslngcrvtDEILHL--  195
DSSP  L------------------------LLLL--hhhhlllhhhhhhhhhhhhhhHHHLLL--
```

```
DSSP  llhhhhhhhhhhhhhhhhhhlllllllllllllhhhHHHHHHL-lLLLL----------
Query lldadlxptrsaciaifearaqhpwpprvatlphwplIYAGALE-gLDHL----------  266
ident                                           |
Sbjct --------------------------------vpnidNFRLTLRaiKLWAkrhniysnil  223
DSSP  --------------------------------lllhhHHHHHHHhhHHHHhhllllllll
```

```
DSSP  -------lllLLHHHHHH------------------------hhhhHHHHhhHLLLL---
Query -------elaRTVDAAAQ------------------------avqrFVARidRATKR---  292
ident
Sbjct gflggvswamLVARTCQLypnaiastlvhkfflvfskwewpnpvllKQPE--ECNLNlpv  281
DSSP  llllhhhhhhHHHHHHHLlllllhhhhhhhhhhhhlllllllllllLLLL--LLLLLlll
```

```
DSSP  ------------------------------------------------------------
Query ------------------------------------------------------------  292
ident
Sbjct wdprvnpsdryhlmpiitpaypqqnstynvsvstrmvmveefkqglaitdeillskaews  341
DSSP  llllllhhhhhllllllllllllllllllllhhhhhhhhhhhhhhhhhhhhhhhhlllhh
```

```
DSSP  ------------------------------------------------------------
Query ------------------------------------------------------------  292
ident
Sbjct klfeapnffqkykhyivllasaptekqrlewvglveskirilvgsleknefitlahvnpq  401
DSSP  hhhllllhhhllleeeeeeeeellhhhhhhhhhhhhhhhhhhhhhhhllleeeeeeelll
```

```
DSSP  ------------------------------------------------------------
Query ------------------------------------------------------------  292
ident
Sbjct sfpapkenpdkeefrtmwviglvfkdltydiqsftdtvyrqainskmfevdmkiaamhvk  461
DSSP  leelllllllllleeeeeeeeeeelllhhhhhhhhhhhhhhhhlllllllllleeeeeeeee
```

```
DSSP  ---------
Query ---------  292
ident
Sbjct rkqlhqllp  470
DSSP  hhhhlllll
```

No 575: Query=mol1A Sbjct=5a2yA Z-score=4.6

back to top

```
DSSP  ----------------------------LLLL-----------lllLLLHHHHhhHHHH-
Query ----------------------------AAPY-----------sspPTNLRSLrdRLTQ-   20
ident                                                 |       |
Sbjct ktftevqterleqadrsvlikcpsklneKKLLqylsshgkidnyffFENRGIH--ALIEf   58
DSSP  llhhhhhhhhhhhhhleeeeellllllhHHHHhhhhhhlleeeeeeEELLLEE--EEEEe
```

```
DSSP  ------------------------------------------------------------
Query ------------------------------------------------------------   20
ident
Sbjct sekssvaslqavtgipkaaehhvvpyksrlftftlknpgsqaaeerpvkispqshipvne  118
DSSP  lllhhhhhhhhhllllhhhllllllllllleeeeeellllllllllllllllllllllhhh
```

```
DSSP  ------------------hhhhllLLHHHHHHHHHHHHHHHHHHLLLlllllLLEEEEHH
Query ------------------vaerqgVVFGRLQRHVAXIVVAQFAATLTddtgaPLLLVKGG   62
ident                                |  |   |  ||         |   ||
Sbjct lipklchadsissqmyillneyqlTEENIKLRYLACSLVRDFARAYF-----PDSTVKPF  173
DSSP  hhhhhhllllhhhhhhhhhhhhllLHHHHHHHHHHHHHHHHHHLLLL-----LLLEEEEE
```

```
DSSP  hhhhhhhllllllllLLEEEEEEL-------------------lLHHHHHHHHHHHH-HHL
Query sslelrrgipdsrtsKDFATVAR-------------------rDIELIHEQLADAG-ETG  102
ident                 |                              |     |     |
Sbjct --gssvntfgklgcdVDMFLDFHdkkgpfemeyqmkrlpserlATQKILSIIGDCLdNFG  231
DSSP  --hhhllllllllllEEEEEELLlllllleeeeellllllhhhHHHHHHHHHHHHHhHHL
```

```
DSSP  LllEEEEEELLLEELlllllllLLEEEEEEEEEllleeEEEEEEEEEEelllllllllleeeee
Query WegFTAIFTAPEEIaapgxpvKPRRFTAKLSYrgraFATVPIEVSsveagnadqfdtlts  162
ident                                            ||
Sbjct P--GYSSVQKILNA-------RCPLVKFSHQP---tGFQCDLSVS---------------  264
DSSP  L--LEEEEEEELLL-------LLLEEEEEELL---lLEEEEEEEL---------------


DSSP  lhhhhlllllleeeeellhhhhhHHHHHHHHlllllllLLLLhhhhhhhhhhhhllhhh
Query dalglvgvpaavavpcxtipwqiAQKLHAVTavleepkaNDRAhdlvdlqlleglllldad  222
ident
Sbjct ---------------------nsIAIRCSEL--------LYIY-----------------  278
DSSP  ---------------------lhHHHHHHHH--------HHHH-----------------


DSSP  hhhhhhhhhhhhhhhhllllllllllllLHHHhHHHHHL-lLLLL---------------
Query lxptrsaciaifearaqhpwpprvatlpHWPLiYAGALE-gLDHL---------------  266
ident
Sbjct ---------------------------gCLDPrVRALVFslRCWArvhgltnsvpgtwit  311
DSSP  ---------------------------hHHLHhHHHHHHhhHHHHhhlllllllllllll


DSSP  ---llllLHHHHHHH---------------------------------------------
Query ---elarTVDAAAQA---------------------------------------------  278
ident
Sbjct nfsltmmIMFFLQKRsppiiptldqlkeladekdkhviggydcsfvsdlskikptkntet  371
DSSP  hhhhhhhHHHHHHLLlllllllhhhhhhlllllllleelleelllllhhhlllllllll


DSSP  ----------------------hhhhhhhhhHLLLL------------------------
Query ----------------------vqrfvaridRATKR------------------------  292
ident
Sbjct ldellcdffqyfgnfdfrknslnlrkgkevnKPESSplyiwnpfeqdlnisknvnqpqle  431
DSSP  hhhhhhhhhhhhhhllllleeeellllleeeeLLLLLllllllllllllllllllhhhhh


DSSP  -------------------------------------
Query -------------------------------------  292
ident
Sbjct kfvamaresawilqkedktqqminkepwglaavlipf  468
DSSP  hhhhhhhhhhhhhhlllhhhhhhlllllllhhhhllll
```

No 576: Query=mol1A Sbjct=3hj4A Z-score=4.6

back to top

```
DSSP  llllllllllhhhhhhhhhhhhhhllLLHHHHHHHHHHHHHHHHHHHHLLllllllLLEEEE
Query aapyssptnlrslrdrltqvaerqgVVFGRLQRHVAXIVVAQFAATLtddtgaPLLLVK   60
ident                                   |     |                 |
Sbjct -------vakrefirgxxahyraslpPPEHSVVIHELQKRVLDIGXLA-----vNKAHVE   48
DSSP  -------lhhhhhhhhhhhhhhhhhllLLLHHHHHHHHHHHHHHHHHHHH-----lLLLEEE


DSSP  HHhhhhhhhllllllllLLEEEEEL-------------lLHHHHHHHHHHHHHHHLlllEE
Query GGsslelrrgipdsrtsKDFATVAR-------------rDIELIHEQLADAGETGwegFT  107
ident                   |     |
Sbjct LF--gshvsgfctphsdADISLTYRnfspwlqgxervdeQNNKRXTRFGKEASAX---GX  103
DSSP  EE--lhhhhllllllllEEEEEELLlllhhhlllhhhhhHHHHHHHHHHHHHHHH---LL


DSSP  EEELLLEEllllllllLLEEEEEEEEEllleEEEEEEEEEEellllllllleeeeelhhhh
Query AIFTAPEEiaapgxpvKPRRFTAKLSYrgrAFATVPIEVSsveagnadqfdtltsdalgl  167
ident
Sbjct EDVRYIRA--------RIPVVQFTDGV---TGIHCDVSIG--------------------  132
DSSP  EEEEEELL--------LLLEEEEELLL---LLLEEEEEEL--------------------


DSSP  llllleeeeellhhhhhhHHHHHHhllllllllLLLLhhhhhhhhhhhhllhhhhhhhh
Query vgvpaavavpcxtipwqiaQKLHAVtavleepkaNDRAhdlvdlqlleglllldadlxptr  227
ident
Sbjct --------------niggvENSKIL---------CAIR----------------------  147
DSSP  --------------lhhhhHHHHHH---------HHHH----------------------


DSSP  hhhhhhhhhhllllllllllllLHHHHHHHHHL-lLLLL-------------lllllhH
Query saciaifearaqhpwpprvatlpHWPLIYAGALE-gLDHL-------------elartvD  273
```

```
DSSP  ---------------------hlLHHHHHHHHHHHhhHHHHhlllllhhhllllhhhhhhH


DSSP  HHHH--------------------------------------------------------
Query AAAQ--------------------------------------------------------  277
ident |
Sbjct ALXVlqelgllpvfskptgefgeltvadaexllqefklppiydslhdddeklgeavffcl  246
DSSP  HHHHhhhhllllllllllllllllhhhhhhhhlllllhhhhhllllhhhhhhhhhhh


DSSP  -----------------hHHHHHHHhhHLLL-----------------------------
Query -----------------aVQRFVARidRATK-----------------------------  291
ident
Sbjct qrfaeyyakydfsagtvsLIHPRRHrtVYERvvrrhlellgsrkrlewekhiaehkedgp  306
DSSP  hhhhhhhhhlllllllllLLLLLLLhhHHHHhhhhhhhhhhhhhhhhhhhhhhhlhhhll


DSSP  ------------------------------------------------------------
Query ------------------------------------------------------------  291
ident
Sbjct ldendfsasxqnettqrpsnspyvvedfvnyvncgrrvqasrvrhiqqefnrlrexlidk  366
DSSP  llhhhhhhhhhhhhhllllllllllllllllllllllhhhhhhhhhhhhhhhhhhlll


DSSP  --------------l
Query --------------r  292
ident
Sbjct eselkfdevfresdt  381
DSSP  hhhllhhhhllllll
```

No 577: Query=mol1A Sbjct=3hj4B Z-score=4.6

[back to top]

```
DSSP  lllllllllhhhhhhhhhhhhhhlLLLHHHHHHHHHHHHHHHHHHHLLllllllLLEEEE
Query aapysspptnlrslrdrltqvaerqGVVFGRLQRHVAXIVVAQFAATLtddtgaPLLLVK   60
ident                                   |     |                 |
Sbjct -------vakrefirgxxahyraslPPPEHSVVIHELQKRVLDIGXLA-----vNKAHVE   48
DSSP  -------lhhhhhhhhhhhhhhhhlLLLLHHHHHHHHHHHHHHHHHHH-----lLLLEEE


DSSP  HHhhhhhhhlllllllLLEEEEEL-------------lLHHHHHHHHHHHHHHLlllEE
Query GGsslelrrgipdsrtsKDFATVAR-------------rDIELIHEQLADAGETGwegFT  107
ident                   |     |
Sbjct LF--gshvsgfctphsdADISLTYRnfspwlqgxervdeQNNKRXTRFGKEASAX---GX  103
DSSP  EE--lhhhhllllllllEEEEEELLllllllllhhhhhHHHHHHHHHHHHHHHH---LL


DSSP  EEELLLEEllllllllLLEEEEEEEEEElleEEEEEEEEEEellllllllleeeeelhhhh
Query AIFTAPEEiaapgxpvKPRRFTAKLSYrgrAFATVPIEVSsveagnadqfdtltsdalgl  167
ident
Sbjct EDVRYIRA--------RIPVVQFTDGV---TGIHCDVSIG--------------------  132
DSSP  EEEEEELL--------LLLEEEEELLL---LLLEEEEEEL--------------------


DSSP  llllllleeeeellhhhhhhhhhhhhhlllllllLLLLhhhhhhhhhhhllhhhhhhhh
Query vgvpaavavpcxtipwqiaqklhavtavleepkaNDRAhdlvdlqllegllldadlxptr  227
ident                                      |
Sbjct -------------------------niggvenskILCA----------------------  145
DSSP  -------------------------lhhhhhhhhHHHH----------------------


DSSP  hhhhhhhhhhllllllllllllHHHH-hHHHHLLL-LLLL------------------
Query saciaifearaqhpwpprvatlphWPLI-yAGALEGL-DHLE------------------  267
ident                                ||   |
Sbjct ----------------------irQVFPdfYGAYIHLvKAWGkareviaperstfnsftv  183
DSSP  ----------------------hhHLLHhhHHHHHHHhHHHHhhlllllhhhllllhhhh


DSSP  lllLHHHHHH--------------------------------------------------
Query larTVDAAAQ--------------------------------------------------  277
ident
Sbjct ttxALXVLQElgllpvfskptgefgeltvadaexllqefklppiydslhdddeklgeavf  243
DSSP  hhhHHHHHHHhlllllllllllllllhhhhhhhhhhhllllhhhhhllllhhhhhhhhh
```

```
Query --------------------aVQRFVARidRATK--------------------------  291
ident
Sbjct fclqrfaeyyakydfsagtvsLIHPRRHrtVYERvvrrhlellgsrkrlewekhiaehke  303
DSSP  hhhhhhhhhhhlllllllllLLLLLLLhhHHHhhhhhhhhhhhhhhhhhhhhhhhhlhh


DSSP  ------------------------------------------------------------
Query ------------------------------------------------------------  291
ident
Sbjct dgpldenfsasxqnettqrpsnspyvvedfvnyvncgrrvqasrvrhiqqefnrlrexli  363
DSSP  hllllllhhhhhhhhhllllllllllllllllllllllhhhhhhhhhhhhhhhhhhh


DSSP  ------------------l
Query ------------------r  292
ident
Sbjct dkeselkfdevfresdtvp  382
DSSP  llhhhllhhhhllllllll
```

No 578: Query=mol1A Sbjct=4e8fB Z-score=4.6

back to top

```
DSSP  lllllllllhhhhhhhhhhhhhhllllhhhhhHHHHhHHHHHHHHHLLLlllllLLEEEE
Query aapysspptnlrslrdrltqvaerqgvvfgrlqRHVAxIVVAQFAATLTddtgaPLLLVK   60
ident                                  |                    |
Sbjct ------------pnshkeftkfcyevyneikikRAAL-DTLRLCLKRIS-----PDAELV   42
DSSP  ------------llllhhhhhhhhhhhhhhlllLLLL-LLHHHHHHHHL-----LLLEEE


DSSP  hhHHHHhhhlllllllLLEEEEEELLLHhHHHHHHHHHHHHHLllleEEEELLLEELllll
Query ggSSLElrrgipdsrtsKDFATVARRDIeLIHEQLADAGETGwegfTAIFTAPEEIaapg  120
ident                   |           |  |
Sbjct --AFSL-esglalknsdMDLCVLMDSRD-TIALQFYEELIAE----GFEGKFLQRA----   90
DSSP  --ELLL-lllllllllLLEEEEELLLL-HHHHHHHHHHHHL----LEEEEEEEEL----


DSSP  lllLLEEEEEEEEEELL--EEEEEEEEEEEELLlllllleeeeelhhhhlllllleeeee
Query xpvKPRRFTAKLSYRG--RAFATVPIEVSSVEagnadqfdtltsdalglvgvpaavavpc  178
ident                    |     |
Sbjct ---RIPIIKLTSDTKNgfGASFQCDIGFNNRL----------------------------  119
DSSP  ---LEEEEEEEEELLLllLLLLLEEEEEEELH----------------------------


DSSP  llhhhhhhHHHHHHHllllllllllllhhhhhhhhhhhllhhhhhhhhhhhhhhhhhl
Query xtipwqiaQKLHAVTavleepkandrahdlvdlqlleglllddadlxptrsaciaifeara  238
ident           |
Sbjct -----aihNTLLLSS---------------------------------------------  129
DSSP  -----hhhHHHHHHHH--------------------------------------------


DSSP  llllllllllllLHHHhHHHHHLL-LLLL-----------------llllLHHHHHHH--
Query qhpwpprvatlpHWPLiYAGALEG-LDHL-----------------elarTVDAAAQA--  278
ident
Sbjct ---------ytkLDAR-LKPMVLLvKHWAkrkqinspyfgtlssygyvlmVLYYLIHVik  179
DSSP  ---------hhhHLLL-HHHHHHHhHHHHhhllllllllllllhhhhhhhHHHHHHHLll


DSSP  ------------------------------------------------------------
Query ------------------------------------------------------------  278
ident
Sbjct ppvfpnlllsplkqekivdgfdvgfddkledippsqnysslgsllhgffrfyaykfepre  239
DSSP  lllllllllllllllleelleellllllhhhllllllllllhhhhhhhhhhhhhhllllll


DSSP  ------hhHHHHhhhHLLLL----------------------------------------
Query ------vqRFVAridRATKR----------------------------------------  292
ident
Sbjct kvvtfrrpDGYLtkqEKGWTsdryilaiedpfeishnvgrtvsssglyrirgefmaasrl  299
DSSP  leellllllLLLLlhhHHLLLllllllleellllllllllllllllhhhhhhhhhhhhhhhh


DSSP  --------------------
Query --------------------  292
ident
```

No 579: Query=mol1A Sbjct=4atbB Z-score=4.6

back to top

```
DSSP  lllllllllhhhhhhhhhhhhhhhlLLLHHHHHHHHHHHHHHHHHHLL---LLLLL--LL
Query aapysspptnlrslrdrltqvaerqGVVFGRLQRHVAXIVVAQFAATL---TDDTG--AP   55
ident                                                     |
Sbjct --------ifvnddrhvmakhssvyPTQEELEAVQNMVSHTERALKAVsdwIDEQEkgRT   52
DSSP  --------llhhhhhhhhhhhhhhlLLHHHHHHHHHHHHHHHHHHHHHhhhHHHHHllLL


DSSP  LEEEEHHhhhhhhhlllLLLL-----lLLEEEEEL--LLHHHHHHHHHHHHHHLL--LLE
Query LLLVKGGsslelrrgipDSRT-----sKDFATVAR--RDIELIHEQLADAGETGW--EGF  106
ident |  |                                     |
Sbjct LRGVMRV------glvaKGLLlkgdldLELVLLCKekPTTALLDKVADNLAIQLTtvTED  106
DSSP  EEEEEEL------hhhhHLLLllllleEEEEEEELllLLHHHHHHHHHHHHHHHHhlLLL


DSSP  EEELLLEELllllllllllEEEEEEEEelleeEEEEEEEEEELLLLllllleeeeelhhh
Query TAIFTAPEEIaapgxpvkpRRFTAKLSyrgraFATVPIEVSSVEAGnadqfdtltsdalg  166
ident                         |            |   |
Sbjct KYEILQSVDD---------AAIVIKNT--kepPLSLTIHLTSPVVR-----eemekvlag  150
DSSP  LLEEEEELLL---------LEEEEELL--lllLLEEEEEEELHHHH-----hhhhhhhll


DSSP  hllllleeeeellhhhhhhhHHHHHHhlllllllllLLLHhhhhhhhhhhhllhhhhhhh
Query lvgvpaavavpcxtipwqiaQKLHAVtavleepkanDRAHdlvdlqllegllldadlxpt  226
ident                                      ||
Sbjct etlppdvldrqkclaalaslRHAKWF---------qARAN--------------------  181
DSSP  lllllllllhhhhhhhhhhhHHHHHH---------hHHLL--------------------


DSSP  hhhhhhhhhhllllllllllllllLHHHhHHHHHLL-LLLL-------------llllLH
Query rsaciaifearaqhpwpprvatlpHWPLiYAGALEG-LDHL-------------elarTV  272
ident                                       |
Sbjct ------------------------GLKS-CVIVIRVlRDLCtrvptwgplrgwplellCE  216
DSSP  ------------------------LLLL-HHHHHHHhHHHHhhlhhhhhhllhhhhhhHH


DSSP  HH--------------------------hhhhhHHHH------HHHHHLLLL--------
Query DA--------------------------aaqavQRFV------ARIDRATKR--------  292
ident                                                    |
Sbjct KSigtanrpmgagealrrvleclasgivmpdgsGIYDpcekeaTDAIGHLDRqqreditq  276
DSSP  HHhhlllllllhhhhhhhhhhhhhlllllllllLLLLllllllLLLLLLLLHhhhhhhhh


DSSP  -------------------------------------------
Query -------------------------------------------  292
ident
Sbjct saqhalrlaafgqlhkvlgmdplpdytvqippsttyapmkrpm  319
DSSP  hhhhhhhhhhlllhhhhhlllllllllllllllllllllllll
```

No 580: Query=mol1A Sbjct=3v7lA Z-score=4.6

back to top

```
DSSP  ------------------------------------------------------------
Query ------------------------------------------------------------    0
ident
Sbjct tlnggitdmlvelanfeknvsqaihkynayrkaasviakyphkiksgaeakklpgvgtki   60
DSSP  llllllhhhhhhhhhhhhlllllllhhhhhhhhhhhhhhllllllllllllllllllhhh


DSSP  -------------------------------------------------llllllllllh
Query -------------------------------------------------aapysspptnl   11
ident
Sbjct aeeideflatgklrklekirqddtsssinfltrvtgigpsaarklvdegiktledlrkne  120
DSSP  hhhhhhlllllllhhhhhhhlllllllhhhhllllllllllllllhhhllllllllllll


DSSP  hhhhhhhhhhhhhllllhHHHHhHHHHHHHHHHHHL-LLLLLLLLEEEEhhhHHHHHHHL
```

```
Sbjct dklnhhqriglkyfedfeKKIP-KEEMLQMQDIVLNevKKLDPEYIAIVC---GSFKRRGA  176
DSSP  lllllllllllllllllLLEE-HHHHHHHHHHHHHhHHHHLLLLEEEEL---HHHHHLL


DSSP  LllllLLLLEEEEEL------------lLHHHHHHHHHHHHhhlllleeEEELLLEELll
Query IpdsrTSKDFATVAR------------rDIELIHEQLADAGetgwegftAIFTAPEEIaa  118
ident       | |                         |||             |
Sbjct E----SSGDMDVLLThpnftsesskqpkLLHRVVEQLQKVR--------FITDTLSKG--  222
DSSP  L----EELLEEEEEElllllllllllhHHHHHHHHHHHHLL--------LEEEEEELL--


DSSP  llllLLLEEEEEEEEELL--eEEEEEEEEEEEL-LLLLllllleeeeelhhhhllllllee
Query pgxpVKPRRFTAKLSYRG--rAFATVPIEVSSV-EAGNadqfdtltsdalglvgvpaava  175
ident              |             |
Sbjct ----ETKFMGVCQLPSENdenEYPHRRIDIRLIpKDQY----------------------  256
DSSP  ----LLEEEEEELLLLLLlllLLLLLLEEEEEL1HHHL----------------------


DSSP  eeellhHHHHHHHhhhhhllllllllllllhhhhHHHHH---------------------
Query vpcxtiPWQIAQKlhavtavleepkandrahdlvDLQLL---------------------  214
ident
Sbjct -----yCGVLYFT--------------------gSDIFNknmrahalekgftineytirp  291
DSSP  -----hHHHHHHL--------------------lLHHHHhhhhhhhhhhleeellllleee


DSSP  -hhhllhhhhhhhhHHHHhhhhhhlllLLLLLLlllllhhhhhhhhllllllllllhh
Query -eglllldadlxptrSACIaifearaqhPWPPRVatlphwpliyagalegldhlelartvd  273
ident               |
Sbjct lgvtgvageplpvdSEQD-------ifDYIQWR---------------------------  317
DSSP  llllllllllllllLHHH-------hhHHLLLL---------------------------


DSSP  hhhhhhhhhhhhhhhllll
Query aaaqavqrfvaridratkr  292
ident
Sbjct -----------yrepkdrs  325
DSSP  -----------lllhhhll
```

No 581: Query=mol1A Sbjct=3auoA Z-score=4.6

back to top

```
DSSP  ------------------------------------------------------------
Query ------------------------------------------------------------    0
ident
Sbjct mrnqelarifeeiglmseflgdnpfrvrayhqaartlydldtpieeiaekgkealmelpg   60
DSSP  llhhhhhhhhhhhhhhhhhhllllhhhhhhhhhhhhhhhhllllhhhhhlllhhhllllll


DSSP  ------------------------------------------------------------
Query ------------------------------------------------------------    0
ident
Sbjct vgpdlaekileflrtgkvrkheelsrkvprgvlevmevpgvgpktarllyeglgidslek  120
DSSP  lllhhhhhhhhhhhhlllhhhhhhhhhhllhhhhhhlllllllhhhhhhhhhhhhlllllhhh


DSSP  -----llllllllllhhhhhhhhhhhhhhllllhhhhHHHHHHHHHHHHH-HLLLLllll
Query -----aapysspptnlrslrdrltqvaerqgvvfgrlQRHVAXIVVAQFA-ATLTDdtga   54
ident                                          |    |
Sbjct lkaaldrgdltrlkgfgpkraerireglalaqaagkrRPLGAVLSLARSLlEAIRAlpgv  180
DSSP  hhhhhhlllllllllllhhhhhhhhhhhhhhhhhllllEEHHHHHHHHHHHHhHHHHLllll


DSSP  lLEEEEHHHHHHHhhllllllLLLLEEEEEL-----lLHHHHHHHhhhhhhhlllleeeE
Query pLLLVKGGSSLELrrgipdsrTSKDFATVAR-----rDIELIHEQladagetgwegftaI  109
ident       |              |  |              |
Sbjct eRAELCGSARRYK-------dTVGDLDFLVAsregerAVEGFVRL-------------pQ  220
DSSP  lEEEEEHHHHLLL-------lEELLEEEEEElllhhhHHHHHHLL-------------lL


DSSP  ELL-LEELlllllllLLLEEEEEEEEeelleeeeEEEEEEEELLLLLllllleeeeelhhhhl
Query FTA-PEEIaapgxpVKPRRFTAKLsyrgrafaTVPIEVSSVEAGNadqfdtltsdalglv  168
ident                       |                 |
Sbjct VKEvYAKG------KERATVFLKN--------GLQVDLRVVPPES---------------  251
DSSP  EEEeEEEL------LLEEEEEELL--------LLEEEEEEELHHH---------------
```

```
DSSP  llllleeeeeellhHHHHhhhhhhhhhlllllllllllHHHHHHH-----------hhhhh
Query gvpaavavpcxtiPWQIaqklhavtavleepkandraHDLVDLQ-----------llegl  217
ident
Sbjct -----------ygAGLQ----------------yltgSKAHSIRlralaqekglklseyg  284
DSSP  -----------hhHHHH----------------hhhlLHHHHHHhhhhhhhllleellll


DSSP  llhhhhhhhhhHHHHhhhhhllllLLLLLlllllhhhhhhhhhlllllllllllhhHHHH
Query lldadlxptrsACIAifearaqhpWPPRVatlphwpliyagalegldhlelartvdAAAQ  277
ident
Sbjct vfrgekriageTEEE-------vyAALGL----------------pwippplredqGEVE  321
DSSP  eeelleeeellLHHH-------hhHHLLL----------------llllhhhllllLHHH


DSSP  HHHH--------------------------------------------------------
Query AVQR--------------------------------------------------------  281
ident |
Sbjct AALEgrlpkllelpqvkgdlqvhstysdgqntleelweaaktmgyrylavtdhspavrva  381
DSSP  HHHLllllllllhhhlleeeeellllllllllhhhhhhhhhhllleeeeeeelhhhlll


DSSP  ------------------------------------------------------------
Query ------------------------------------------------------------  281
ident
Sbjct ggpspeealkrvgeirrfnethgppyllagaevdihpdgtldypdwvlreldlvlvsvhs  441
DSSP  llllllhhhhhhhhhhhhhhhllleeeeeeeeeelllllllllhhhhhllleeeeelll


DSSP  ------------------------------------------------------------
Query ------------------------------------------------------------  281
ident
Sbjct rfnlpkadqtkrllkalenpfvhvlahptarllgrrapieadweavfqkakekgvaveid  501
DSSP  lllllhhhhhhhhhhhhhllllleellllllllllllllllhhhhhhhhhhllleeeee


DSSP  ------------------------------------------------------------
Query ------------------------------------------------------------  281
ident
Sbjct gyydrmdlpddlarmaygmglwislstdahqtdhlrfmelavgtaqrawigpervlntld  561
DSSP  lllllllllhhhhhhhhhlllleeeellllllhhhhhhhhhhhhhhhhlllllllllllll
```

```
DSSP  hhhhhhhllll
Query fvaridratkr  292
ident
Sbjct yedllswlkar  572
DSSP  hhhhhhhhhll
```

No 582: Query=mol1A Sbjct=2ihmB Z-score=4.6

back to top

```
DSSP  ------------------------------------------------------------
Query ------------------------------------------------------------    0
ident
Sbjct smpayacqrpsplthhntllsealetlaeaagfeanegrllsfsraasvlkslpcpvasl   60
DSSP  lllllhhhllllllllllhhhhhhhhhhhhhhhhlllhhhhhhhhhhhhhhhllllllllh


DSSP  ------------------------------------------------------------
Query ------------------------------------------------------------    0
ident
Sbjct sqlhglpyfgehstrviqellehgtceevkqvrcseryqtmklftqvfgvgvktanrwyq  120
DSSP  hhlllllllllhhhhhhhhhhhhhlllhhhhhhhhlhhhhhhhhhhllllllhhhhhhhhh


DSSP  -llllllllllhhhhhhhhhhhhhhllllhhhhhHHHHHHHHHHHHHLLLL-llLLLLEE
Query -aapyssppptnlrslrdrltqvaerqgvvfgrlqRHVAXIVVAQFAATLTD-dtGAPLLL   58
ident                                    |         |
Sbjct eglrtldelreqpqrltqqqkaglqyyqdlstpvRRADAEALQQLIEAAVRqtlPGATVT  180
DSSP  hllllhhhllhhhllhhhhhhhhlhhhhllleEHHHHHHHHHHHHHHHHllLLLEEE


DSSP  EEHHHHHHHhhllllllLLLLEEEEEL--------LLHHHHHHHHHHHHhhllllleeeEE
Query VKGGSSLELrrgipdsrTSKDFATVAR--------RDIELIHEQLADAGetgwegftaIF  110
```

```
DSSP   ELHHHHLLL-------LEELLEEEEEELLLLLLLLHHHHHHHHHHLLL---------LE


DSSP   LLLEelllllllLLLEEEEEEEEEELL--------EEEEEEEEEEEEL-LLLLllllleeee
Query  TAPEeiaapgxpVKPRRFTAKLSYRG--------RAFATVPIEVSSV-EAGNadqfdtlt  161
ident                        |                 |
Sbjct  LYHQ-------yFERSFCILGLPQPQqagalppcPTWKAVRVDLVVTpSSQF--------  269
DSSP   EELL-------lLLEEEEEEEEEELLLlllllllLLEEEEEEEEEEELlHHHH--------


DSSP   elhhhhllllleeeeellhHHHHhhhhhhhhllllllllllllHHHHHHH---------
Query  sdalglvgvpaavavpcxtiPWQIaqklhavtavleepkandraHDLVDLQ---------  212
ident
Sbjct  --------------------pFALL----------------gwtgSQFFEREIrrfsrqek  294
DSSP   --------------------hHHHH----------------hhhlLHHHHHHhhhhhhhll


DSSP   -----hhhhhllhhhhhhhHHHHHhhhhhlllLLLLLLlllllhhhhhhhhhllllll
Query  -----lleglllddadlxptRSACIaifearaqhPWPPRVatlphwpliyagalegldhle  267
ident
Sbjct  glwlnshglfdpeqkrvfhATSEE------dvfRLLGLK---------------------  327
DSSP   lleellllleellllllllLLLHH------hhhHHHLLL---------------------


DSSP   llllhhhhhhhhhhhhhhhhhllll
Query  lartvdaaaqavqrfvaridratkr  292
ident
Sbjct  ----------------ylppeqrna  336
DSSP   ----------------lllhhhlll
```

No 583: Query=mol1A Sbjct=4k8vA Z-score=4.6

back to top

```
DSSP   lllllllllhhhhhhhhhhhhhhLLLLHHHHHHHHHHHHHHHHHHHLL--LLLLllLLEE
Query  aapysspptnlrslrdrltqvaerQGVVFGRLQRHVAXIVVAQFAATL--TDDTgaPLLL   58
ident                                             ||
Sbjct  -----------spdklkkvldklrLKRKDISEAAETVNKVVERLLRRMqkRESE-fKGVE   48
DSSP   -----------lhhhhhhhhhhhhHHHHHLHHHHHHHHHHHHHHHHHHHhhLLLL-lLLLE


DSSP   EEHhhHHHHhhllllLLLL------LLEEEEEL---------------------------
Query  VKGgsSLELrrgipdSRTS------KDFATVAR---------------------------   85
ident                           |
Sbjct  QLN--TGSY-----yEHVKisapneFDVMFKLEvprielqeyyetgafylvkfkriprgn  101
DSSP   EEL--LLLL-----lLLLLllllleEEEEEEEEllleeeeelllllllleeeeelllllll


DSSP   --------------lLHHHHHHHHHHHHHHHLlLLEEEEELLLEELlllllllllEEEEEE
Query  --------------rDIELIHEQLADAGETGwEGFTAIFTAPEEIaapgxpvkpRRFTAK  131
ident                                                         |
Sbjct  plshflegevlsatkMLSKFRKIIKEEVKEI-KDIDVSVEKEKPG--------sPAVTLL  152
DSSP   llhhheelleelhhhHHHHHHHHHHHHHHLLL-LLLEEEEELLLLLL--------lLLEEEE


DSSP   EEEllEEEEEEEEEEEEELLLL-------------------llllleeeeelhhhhlllll
Query  LSYrgRAFATVPIEVSSVEAG-------------------nadqfdtltsdalglvgvpa  172
ident           | |       |
Sbjct  IRN--PEEISVDIILALESKGswpistkeglpiqgwlgtkvrtnlrrepfylvpknakdg  210
DSSP   EEL--LLEEEEEEEEEEEELLlllhhhllllllllllhhhhhhhhllleeeeelllllll


DSSP   leeeeellhHHHHhHHHHHHhlllllllllllhhhhhhhhhhhllhhhhhhhhhhhhh
Query  avavpcxtiPWQIaQKLHAVtavleepkandrahdlvdlqlleglllddadlxptrsacia  232
ident
Sbjct  nsfqgetwrLSFS-HTEKYI----------------------------------------  229
DSSP   lllllllleeEELH-HHHHHH----------------------------------------


DSSP   hhhhhlllllllllLLLL--------hHHHHHHHHL-LLLLL----------------l
Query  ifearaqhpwpprvaTLPH--------wPLIYAGALE-GLDHL----------------e  267
ident
Sbjct  -----------lnnhGIEKtccessgakCCRKECLKLmKYLLEqlkkefqeldafcsyhv  278
DSSP   -----------hhllLLLLllllllllLLHHHHHHHhHHHHHhhhhhlhhhllllhhhh
```

```
Query iartvdAAAQ-----------------------------avQRFV---------------  283
ident          |
Sbjct ktaifhMWTQdpqdsqwdprnlsscfdkllafflecl rtekLDHYfipkfnlfsqelidr  338
DSSP  hhhhhhHHHHlllhhhhlhhhhhhhhhhhhhhhhhhhhhllLLLLllllllllllllllh


DSSP  ---------------hhhhhllll
Query ---------------aridratkr  292
ident
Sbjct kskeflskkieyernngfpifdkl  362
DSSP  hhhhhhhhhhhhhhhlllhhhhll
```

No 584: Query=mol1A Sbjct=1jpwA Z-score=4.6

back to top

```
DSSP  -------------------------------------------LLLL----llLLLLHHH
Query -------------------------------------------AAPY----ssPPTNLRS   13
ident
Sbjct raipeltkllndedqvvvnkaavmvhqlskkeasrhaimrspqMVSAivrtmqNTNDVET   60
DSSP  lhhhhhhhhhllllhhhhhhhhhhhhhhlllhhhhhhhhllhhHHHHhhhhhhHLLLLLH


DSSP  HHHHHHHHHHHLL---------llhHHHHHHHHHHHH--------------------hhh
Query LRDRLTQVAERQG---------vvfGRLQRHVAXIVV--------------------aqf   44
ident  |                       |
Sbjct ARCTAGTLHNLSHhregllaifksgGIPALVKMLGSPvdsvlfyaittlhnlllhqegak  120
DSSP  HHHHHHHHHHHLLlhhhhhhhhhllHHHHHHHHLLLLlhhhhhhhhhhhhhhhhhlllhh


DSSP  hhllllllllllleEEEH--------HHHHHHHHLlllllllleeeeellllhhhhhhhhh
Query aatltddtgapllLVKG--------GSSLELRRGipdsrtskdfatvarrdieliheqla   96
ident                               |
Sbjct mavrlagglqkmvALLNktnvkflaITTDCLQIL--------------------------  154
DSSP  hhhhhllhhhhhhHHHHlllhhhhhHHHHHHHHH--------------------------


DSSP  hhhhhlllleeeeellleellllllllllleeeeeeeeeelleeeeeeeeeeellllllll
Query dagetgwegftaiftapeeiaapgxpvkprrftaklsyrgrafatvpievssveagnadq  156
ident
Sbjct ------------------------------------------------------------  154
DSSP  ------------------------------------------------------------


DSSP  leeeeelhhhhlllllleeeeeLLHHHHHHHHHHHHhllllllllLLLHHHHHHHHHHH
Query fdtltsdalglvgvpaavavpcXTIPWQIAQKLHAVtavleepkanDRAHDLVDLQLLEG  216
ident                          |                         |
Sbjct ---------aygnqeskliilaSGGPQALVNIMRTY----------TYEKLLWTTSRVLK  195
DSSP  ---------hlllhhhhhhhhhLLHHHHHHHHHHHL----------LLHHHHHHHHHHHH


DSSP  HL--LHHHHHHHHHHHhhhhhhhllllllLLLLLlllhhhhhhhhhllllllllllLHHH
Query LL--LDADLXPTRSACiaifearaqhpwpPRVATlphwpliyagalegldhlelarTVDA  274
ident  |            |                  |
Sbjct VLsvCSSNKPAIVEAG--------gmqalGLHLT---------------------dPSQR  226
DSSP  HHllLLLHHHHHHHLL--------hhhhhHHHLL---------------------lLLHH


DSSP  HHHHHHHHHHHHHHLLLL------------------------------------------
Query AAQAVQRFVARIDRATKR------------------------------------------  292
ident   |           |
Sbjct LVQNCLWTLRNLSDAATKqegmegllgtlvqllgsddinvvtcaagilsnltcnnyknkm  286
DSSP  HHHHHHHHHHHHHLLLLLllllhhhhhhhhhhllllhhhhhhhhhhhhhhhllllhhhhh


DSSP  ------------------------------------------------------------
Query ------------------------------------------------------------  292
ident
Sbjct mvcqvggiealvrtvlragdreditepaicalrhltsrhqeaemaqnavrlhyglpvvvk  346
DSSP  hhhhllhhhhhhhhhhhhlllhhhhhhhhhhhhhhllllllhhhhhhhhhhlllhhhhhh


DSSP  ------------------------------------------------------------
Query ------------------------------------------------------------  292
ident
```

```
DSSP  ------------------------------------------------------------
Query ------------------------------------------------------------  292
ident
Sbjct eeivegctgalhilardvhnrivirglntiplfvqllyspieniqrvaagvlcelaqdke  466
DSSP  hhhhhhhhhhhhhhllllhhhhhhhhhllllhhhhhhhhhllllhhhhhhhhhhhhhhhllhh


DSSP  ------------------------------------
Query ------------------------------------  292
ident
Sbjct aaeaieaegatapltellhsrnegvatyaaavlfrm  502
DSSP  hhhhhhllllhhhhhhhllllllhhhhhhhhhhhhhl
```

No 585: Query=mol1A Sbjct=4at8D Z-score=4.5

back to top

```
DSSP  lllllllllhhhhhhhhhhhhhhlLLLHHHHHHHHHHHHHHHHHHHLL---LLLLL--LL
Query aapyssspptnlrslrdrltqvaerqGVVFGRLQRHVAXIVVAQFAATL---TDDTG--AP   55
ident                                                     |
Sbjct --------ifvnddrhvmakhssvyPTQEELEAVQNMVSHTERALKAVsdwIDEQEkgRT   52
DSSP  --------llhhhhhhhhhhhhhhlLLHHHHHHHHHHHHHHHHHHHHHHHhhhHHHHHllLL


DSSP  LEEEEHHhhhhhhhlllLLLL-----lLLEEEEELL-LHHHHHHHHHHHHHH---lLLLE
Query LLLVKGGsslelrrgipDSRT-----sKDFATVARR-DIELIHEQLADAGET---gWEGF  106
ident |  |                                          ||
Sbjct LRGVMRV------glvaKGLLlkgdldLELVLLCKEkPTTALLDKVADNLAIqlttVTED  106
DSSP  EEEEEEL------hhhhHLLLllllleEEEEEEELLlLLHHHHHHHHHHHHHHhhhhLLLL


DSSP  EEEELLLEELlllllllllEEEEEEEEelleeEEEEEEEEEEL-LLLLllllleeeeelhh
Query TAIFTAPEEIaapgxpvkpRRFTAKLSyrgraFATVPIEVSSV-EAGNadqfdtltsdal  165
ident                         |            |   |
Sbjct KYEILQSVDD---------AAIVIKNT--kepPLSLTIHLTSPvVREE--------mekv  147
DSSP  LLEEEEELLL---------LEEEEELL--lllLLEEEEEEELHhHHHH--------hhhh


DSSP  hhllllllleeeeellhhhhhhHHHHHHhlllllllLLLLhhhhhhhhhhhllhhhhhh
Query glvgvpaavavpcxtipwqiaQKLHAVtavleepkaNDRAhdlvdlqllegllldadlxp  225
ident                                       ||
Sbjct lagetlpdvldrqkclaalasLRHAKW--------fQARA--------------------  179
DSSP  hllllllllllhhhhhhhhhhHHHHHH--------hHHHL--------------------


DSSP  hhhhhhhhhhhhllllllllllllLHHHhHHHHHLL-LLLL-------------llllL
Query trsaciaifearaqhpwpprvatlpHWPLiYAGALEG-LDHL-------------elarT  271
ident                                        |
Sbjct ------------------------nGLKS-CVIVIRVlRDLCtrvptwgplrgwplellC  214
DSSP  ------------------------lLLLL-HHHHHHHhHHHHhhlhhhhhhllhhhhhhH


DSSP  HHHHHH---------------------------hhHHHHhhHHHL---------------
Query VDAAAQ---------------------------avQRFVarIDRA---------------  289
ident                                             |
Sbjct EKSIGTanrpmgagealrrvleclasgivmpdgsgIYDP-cEKEAtdaighldrqqredi  273
DSSP  HHHHHLllllllhhhhhhhhhhhhhlllllllllLLLL-lLLLLllllllllhhhhhhh


DSSP  ----------------------------------------lll
Query ----------------------------------------tkr  292
ident
Sbjct tqsaqhalrlaafgqlhkvlgmdplpdytvqippsttyapmrp  316
DSSP  hhhhhhhhhhhhlllhhhhllllllllllllllllllllll
```

No 586: Query=mol1A Sbjct=2w9mA Z-score=4.5

back to top

```
DSSP  ------------------------------------------------------------
```

```
Sbjct psrnrivnalertadiidiidrksrayrsaarsleeinitgipkvgkgiaaeisdiarsg   60
DSSP  llhhhhhhhhhhhhhhhhhhllllhhhhhhhhhhhhllllllllllhhhhhhhhhhhhhl


DSSP  ----LLLL-LLLLllHHHHHHH--------------------------------------
Query ----AAPY-SSPPtnLRSLRDR--------------------------------------   17
ident             |     |
Sbjct tfapLEAAaGQLP--PGLLDLLgvrglgpkkirslwlagidslerlreaaesgelaglkg  118
DSSP  llhhHHHHhHHLL--HHHHHHLllllllhhhhhhhhhlllllhhhhhhhhhhllllllll


DSSP  -------hhhhhhhllllhHHHHHHHHHHHHHHHHHHLLlllllLLLEEEEHHHHHHHhhl
Query -------ltqvaerqgvvfGRLQRHVAXIVVAQFAATLtddtgAPLLLVKGGSSLELrrg   70
ident                       |   |   ||   |              |     |
Sbjct fgaksaatilenvvflfeaRQRQSLRAGLAVAEELAGA---ltDLSPAPAGDVRRGL---  172
DSSP  llhhhhhhhhhhhhhhhhhLLLEEHHHHHHHHHHHHHH---lhHHLLEELHHHHHLL---


DSSP  lllllLLLLEEEEEL---lLHHHHHHhhhhhhhllllleeeeelLLEELllllllllLEE
Query ipdsrTSKDFATVAR---rDIELIHEqladagetgwegftaiftAPEEIaapgxpvkPRR  127
ident      |             |
Sbjct ----eTVRAAELTVTgtpdDVLARLP------------------ELTVQ--------VLS  202
DSSP  ----lEELLEEEEEEllhhHHHHHLL------------------LLEEL--------LEE


DSSP  EEEEeeelleeeeEEEEEEEELLLLLllllleeeeelhhhhlllllleeeeellhHHHHhh
Query FTAKlsyrgrafaTVPIEVSSVEAGNadqfdtltsdalglvgvpaavavpcxtiPWQIaq  187
ident              || |     |
Sbjct GDYE---------GVPVEIACAPAEA---------------------------rgALDL--  225
DSSP  EEEL---------LEEEEEEEELLLL---------------------------hhHHHH--


DSSP  hhhhhhlllllllllllhhhHHHHHHHHH-------------hllhhhhhhhhHHHHhhh
Query klhavtavleepkandrahdlVDLQLLEG-------------llldadlxptrSACIaif  234
ident
Sbjct ----------------lrsgeHFAGQVQAaaqargftltagglsrgdevlptpTEAV---  266
DSSP  ----------------hlllhHHHHHHHHhhhllllleeelleeeelleeellllLHHH---


DSSP  hhhllllLLLLLlllllhhhhhhhHHLLllllllllLLHHH--------------------
Query earaqhpWPPRVatlphwpliyagALEGldhlelaRTVDA--------------------  274
ident                           |
Sbjct ----vfhALDLP-----frpaeyrEPEH------dDLWQTlpdpaelvtvgdlrgmihth  311
DSSP  ----hhhHLLLL-----lllhhhlLHHH------lLLLLLlllhhhlllhhhlleeeeel


DSSP  ------------------------------------------------------------
Query ------------------------------------------------------------  274
ident
Sbjct stwsdggasiremaeatltlgheflgtadhsraayyangltierlreqlkeirelqragl  371
DSSP  llllllllhhhhhhhhhhllllleeeeleellllhhhllllhhhhhhhhhhhhhhhhlll


DSSP  ------------------------------------------------------------
Query ------------------------------------------------------------  274
ident
Sbjct pivagsevdilddgsldfpddvlgeldyvvvsvhsnftldaarqterliravshplvtvl  431
DSSP  leeleeeeellllllllllhhhhllllleeeeellllllllhhhhhhhhhhhhlllllllee


DSSP  ------------------------------------------------------------
Query ------------------------------------------------------------  274
ident
Sbjct ghatgrlllrrpgyaldldavlgaceangtvveinanaarldldwrealrwrerlkfain  491
DSSP  lllllllllllllllhhhhhhhhhhhlleeeeellllllllhhhhhhhlllllleeee


DSSP  --------------------------------hhhhhhhhhhhhhhllll
Query --------------------------------aaqavqrfvaridratkr  292
ident
Sbjct tdahvpgglrdarygvmqarkagltpahvvnslgraefldfvarqraarg  541
DSSP  lllllhhhhhhhhhhhhhhhhhllllhhhlhhhllhhhhhhhhhhhhhlll
```

No 587: Query=mol1A Sbjct=3auoB Z-score=4.5

```
DSSP  ------------------------------------------------------------
Query ------------------------------------------------------------   0
ident
Sbjct mrnqelarifeeiglmseflgdnpfrvrayhqaartlydldtpieeiaekgkealmelpg  60
DSSP  llhhhhhhhhhhhhhhhhhhlllhhhhhhhhhhhhhhhhllllhhhhhlllhhhhlllll


DSSP  ------------------------------------------------------------
Query ------------------------------------------------------------   0
ident
Sbjct vgpdlaekileflrtgkvrkheelsrkvprgvlevmevpgvgpktarllyeglgidslek 120
DSSP  llhhhhhhhhhhhhhlllhhhhhhhhhlllhhhhhlllllhhhhhhhhhhhlllllhhh


DSSP  -----lllllllllhhhhhhhhhhhhhhllllhhhhHHHHHHHHHHHHHHLL-llllll
Query -----aapyssppptnlrslrdrltqvaerqgvvfgrlQRHVAXIVVAQFAATL-tddtga  54
ident                                          |    |
Sbjct lkaaldrgdltrlkgfgpkraerireglalaqaagkrRPLGAVLSLARSLLEAiralpgv 180
DSSP  hhhhhhlhhhlllllhhhhhhhhhhhhhhhlllllEEHHHHHHHHHHHHHHHhhhllll


DSSP  lLEEEEHHHHHHHhhllllllLLLLEEEEEL-----lLHHHHHHHhhhhhhhlllleeee
Query pLLLVKGGSSLELrrgipdsrTSKDFATVAR-----rDIELIHEQladagetgwegftai 109
ident       |              |  |              |
Sbjct eRAELCGSARRYK-------dTVGDLDFLVAsregerAVEGFVRL------------pqv 221
DSSP  lEEEEEHHHHLLL-------lEELLEEEEEElllhhhHHHHHHLL------------lle


DSSP  ELLLEELllllllLLLEEEEEEEeelleeeeEEEEEEEEL-LLLLllllleeeeelhhhhl
Query FTAPEEIaapgxpVKPRRFTAKLsyrgrafaTVPIEVSSV-EAGNadqfdtltsdalglv 168
ident                      |                 |
Sbjct KEVYAKG------KERATVFLKN--------GLQVDLRVVpPESY--------------- 252
DSSP  EEEEEEL------LLEEEEEELL--------LLEEEEEEElHHHH---------------


DSSP  llllleeeeellhHHHHhhhhhhhhlllllllllllHHHHHHH-----------hhhhh
Query gvpaavavpcxtiPWQIaqklhavtavleepkandraHDLVDLQ-----------llegl 217
ident
Sbjct ------------gAGLQ----------------yltgSKAHSIRlralaqekglklseyg 284
DSSP  ------------hHHHH----------------hhhlLHHHHHHhhhhhhhllleeelle


DSSP  llhhhhhhhhhHHHHhhhhhllllLLLLLllllllhhhhhhhhhllllllllllllhhHHHH
Query lldadlxptrsACIAifearaqhpWPPRVatlphwpliyagalegldhlelartvdAAAQ 277
ident
Sbjct vfrgekriageTEEE-------vyAALGL----------------pwippplredqGEVE 321
DSSP  eeelleeeellLHHH-------hhHLLLL----------------llllhhhllllLHHH


DSSP  HHHH--------------------------------------------------------
Query AVQR-------------------------------------------------------- 281
ident |
Sbjct AALEgrlpkllelpqvkgdlqvhstysdgqntleelweaaktmgyrylavtdhspavrva 381
DSSP  HHHLlllllllllhhhlleeeeelllllllllllhhhhhhhhhhlllleeeeeeellllll


DSSP  ------------------------------------------------------------
Query ------------------------------------------------------------ 281
ident
Sbjct ggpspeealkrvgeirrfnethgppyllagaevdihpdgtldypdwvlreldlvlvsvhs 441
DSSP  llllhhhhhhhhhhhhhhhhhhhllleeeeeeeeeellllllllllhhhhhhlleeeeelll


DSSP  ------------------------------------------------------------
Query ------------------------------------------------------------ 281
ident
Sbjct rfnlpkadqtkrllkalenpfvhvlahptarllgrrapieadweavfqkakekgvaveid 501
DSSP  lllllhhhhhhhhhhhhllllllleellllllllllllllllhhhhhhhhhhhllleeeee


DSSP  ------------------------------------------------------------
Query ------------------------------------------------------------ 281
ident
Sbjct gyydrmdlpddlarmaygmglwislstdahqtdhlrfmelavgtaqrawigpervlntld 561
DSSP  lllllllllhhhhhhhhhhllllleeeelllllhhhhhhhhhhhhhhhhhhlllllllllhhhll


DSSP  hhhhhhhllll
```

```
Sbjct yedllswlkar  572
DSSP  hhhhhhhhhll
```

No 588: Query=mol1A Sbjct=2m2tA Z-score=4.5

back to top

```
DSSP  lllllllllhhhhhhhhhhhhhhllllhhhHHHHHHHHHHH-HHHHLL-------llll
Query aapysspptnlrslrdrltqvaerqgvvfgrLQRHVAXIVVA-QFAATL-------tddt   52
ident                                                 |
Sbjct -------------------------------MLTLIQGKKIVnHLRSRLafeyngqliki   29
DSSP  -------------------------------LEEHHHHHHHHhHHHHLEeeeelleeeee


DSSP  llLLEEEEHHHHHHHhhllllllLLLLEEEEEL--------lLHHHhhHHHHhhhhhlll
Query gaPLLLVKGGSSLELrrgipdsrTSKDFATVAR--------rDIELihEQLAdagetgwe  104
ident         |    |            |
Sbjct lsKNIVAVGSLRREE-------kMLNDVDLLIIvpekkllkhVLPN--IRIK--------   72
DSSP  llLLEEEEEHHHHLL-------lEELEEEEEEEellhhhhhlLLLE--EEEL--------


DSSP  leEEEELlLEELLlllllLLLEEEEEEEeelleEEEEEEEEEEEELLLLLllllleeeeelh
Query gfTAIFTaPEEIAapgxpVKPRRFTAKLsyrgrAFATVPIEVSSVEAGNadqfdtltsda  164
ident      |                              |         |
Sbjct --GLSFS-VKVCG-----ERKCVLFIEW-----EKKTYQLDLFTALAEE-----------  108
DSSP  --LLEEE-EEEEL-----LLEEEEEEEE-----LLEEEEEEEEEEELLL-----------


DSSP  hhhllllllleeeeellhHHHHhhhhhhhhhlllllllllLLHHH------hhhhhhhhhl
Query lglvgvpaavavpcxtiPWQIaqklhavtavleepkandRAHDL------vdlqllegll  218
ident 
Sbjct ---------------kpYAIF-------------hftgpVSYLIriraalkkknyklnqy  140
DSSP  ---------------hhHHHH-------------hhhllLHHHHhhhhhhhllleeeell


DSSP  lhhhhhhhhhHHHHhhhhllllLLLLLlllllhhhhhhhhhlllllllllllhhhhhhh
Query ldadlxptrsaCIAIfearaqhpWPPRVatlphwpliyagalegldhlelartvdaaaqa  278
ident             |
Sbjct glfknqtlvplKITT---ekeliKELGF--------------------------------  165
DSSP  eeeelleeellLLLL---hhhhhHHHLL--------------------------------


DSSP  hhhhhhhhhhllll
Query vqrfvaridratkr  292
ident 
Sbjct -----tyripkkrl  174
DSSP  -----lllllllll
```

No 589: Query=mol1A Sbjct=2m2wA Z-score=4.5

back to top

```
DSSP  lllllllllhhhhhhhhhhhhhhllllhhhhHHHHHHHHHHHHHHHLL--------llll
Query aapysspptnlrslrdrltqvaerqgvvfgrlQRHVAXIVVAQFAATL--------tddt   52
ident 
Sbjct -------------------------------mLTLIQGKKIVNHLRSRlafeyngqliki   29
DSSP  -------------------------------lLLHHHHHHHHHHHHHHHeeeeelleeeee


DSSP  llLLEEEEHHHHHHhhhllllLLLLLLEEEEEL--------lLHHHHHhhhHHHHhhlll
Query gaPLLLVKGGSSLElrrgipdSRTSKDFATVAR--------rDIELIHeqlADAGetgwe  104
ident         |    |            |                   |
Sbjct lsKNIVAVGSLRRE-------EKMLNDVDLLIIvpekkllkhVLPNIR---IKGL-----   74
DSSP  lhHHLEEEEHHHHL-------LLLLLLEEEEEEellllhhhlLLLLEE---ELLL-----


DSSP  leeeEELLlEELLLlllllLLEEEEEEEEelleeEEEEEEEEEEL-LLLLllllleeeeel
Query gftaIFTApEEIAApgxpvKPRRFTAKLSyrgraFATVPIEVSSV-EAGNadqfdtltsd  163
ident      |                              |
Sbjct ----SFSV-KVCGE-----RKCVLFIEWE-----KKTYQLDLFTAlAEEK----------  109
DSSP  ----EEEE-EEELL-----LEEEEEEEEL-----LEEEEEEELEEeLLLH----------
```

```
Query alglvgvpaavavpcxtIPWQIAQKlhavtavleepkandrahdLVDLQ-----------  212
ident                                                |
Sbjct -----------------pYAIFHFT-----------------gPVSYLIriraalkkkny  135
DSSP  -----------------hHHHHHHH-----------------lLHHHHHhhhhhhllll


DSSP  -----hhhhhllhHHHHHhhHHHHhhhhhhllllLLLLLlllllhhhhhhhhhllllll
Query -----llegllldADLXPtrSACIaifearaqhpWPPRVatlphwpliyagalegldhle  267
ident                 |
Sbjct klnqyglfknqtlVPLKI--TTEK-----elikeLGFTY---------------------  167
DSSP  eeelleeeelleeELLLL--LLHH-----hhhhhHLLLL---------------------


DSSP  llllhhhhhhhhhhhhhhhhllll
Query lartvdaaaqavqrfvaridratkr  292
ident
Sbjct ------------------ripkkrl  174
DSSP  ------------------llhhhll
```

No 590: Query=mol1A Sbjct=4wc7A Z-score=4.5

back to top

```
DSSP  lllllllllhhhhhhhhhhhhhhllllhhhhhhhhhhhhhhhHHHLLLLLllLLLEEEE
Query aapysspptnlrslrdrltqvaerqgvvfgrlqrhvaxivvaqFAATLTDDtgAPLLLVK   60
ident
Sbjct -------------------------------------------MVGQIAKE-mGLRAYIV   16
DSSP  -------------------------------------------LHHHHHHH-lLLEEEEE


DSSP  HHHHHHHHHLLlllllLLLEEEEEELLLHHHHHHHHHHHHHhllllleeeeELLLEElllll
Query GGSSLELRRGIpdsrtSKDFATVARRDIELIHEQLADAGEtgwegftaiFTAPEEiaapg  120
ident ||       |        |   |           ||
Sbjct GGVVRDILLGK----eVWDVDFVVEGNAIELAKELARRHG---------VNVHPF-----   58
DSSP  LHHHHHHHLLL----lLLEEEEEEELLHHHHHHHHHHHLL---------LLLEEE-----


DSSP  llLLLEEEEEEeeelleeeeEEEEEEEELLL----LLLL--------------------l
Query xpVKPRRFTAKlsyrgrafaTVPIEVSSVEA----GNAD--------------------q  156
ident                         |
Sbjct peFGTAHLKIG---------KLKLEFATARRetepASLKedlirrdftinamaisvnled  109
DSSP  llLLEEEEEEL---------LEEEEEEELLLllllLLHHhhhhllllhhhlleeelllll


DSSP  LEEEeelhhhhllllleeeeeelLHHH---------hhhhhhhHHLLlllllLLLLhh
Query FDTLtsdalglvgvpaavavpcxTIPW---------qiaqklhaVTAVleepkaNDRAhd  207
ident   ||                                                    |
Sbjct YGTL-------------------IDYFgglrdlkdkvirvlhpvSFIE-----dPVRI--  143
DSSP  LLLE-------------------ELLLlhhhhhhlleellllllHHHH-----lLLHH--


DSSP  hhhhhhhhhllhhhhhhhhhhhhhhhhhhlllllllllllllhhhHHHHHHLLLLlll
Query lvdlqllegllldadlxptrsaciaifearaqhpwpprvatlphwplIYAGALEGLDhle  267
ident                                                        |
Sbjct -----------------------------------------------lRALRFAGRLN---  154
DSSP  -----------------------------------------------hHHHHHHHHLL---


DSSP  llLLHHHHHHH-------------------------------------------------
Query laRTVDAAAQA-------------------------------------------------  278
ident
Sbjct -fKLSRSTEKLlkqavnlgllkeaprgrlineiklalredrfleilelyrkyrvleeiie  213
DSSP  -lEELHHHHHHhhhhhhllhhhhllhhhhhhhhhhhhhllllhhhhhhhhhhlllhhhhll


DSSP  ------------------------------------------------------------
Query ------------------------------------------------------------  278
ident
Sbjct gfqwnekvlqklyalrkvvdwhalefseeridygwlyllilisnldyergkhfleemsap  273
DSSP  lllllhhhhhhhhhhhhhhhhhhhhhlhhhllllhhhhhhhhhllllhhhhhhhhhhllll


DSSP  ------------------------------------------------------------
Query ------------------------------------------------------------  278
ident
```

```
DSSP  --------------------hhhhhhhhhhllll
Query --------------------vqrfvaridratkr  292
ident
Sbjct klrkvklpkekieelkkgkelgerieelkreimn  367
DSSP  lllllllhhhhhhhlllhhhhhhhhhhhhhhl
```

No 591: Query=mol1A Sbjct=1vj7A Z-score=4.4

[back to top](#)

```
DSSP  ------------------------------------------------------------
Query ------------------------------------------------------------    0
ident
Sbjct inltgeevvalaakymnetdaafvkkaldyataahfyqvrksgepyivhpiqvagiladl   60
DSSP  llllhhhhhhhhhhhllhhhhhhhhhhhhhhhhhllllllllllllhhhhhhhhhhhl


DSSP  ------------------------------------------------------------
Query ------------------------------------------------------------    0
ident
Sbjct hldavtvacgflhdvvedtditldniefdfgkdvrdivdgvtklghrkmlmamskdirvi  120
DSSP  lllhhhhhhhhhllhhhhllllhhhhhhhhlhhhhhhhhhhhhhllllllllllllhhhh


DSSP  ------------------------------------llllllllllhhhhhhhhhhhhh
Query ------------------------------------aapysspptnlrslrdrltqvaer   24
ident
Sbjct lvkladrlhnmrtlkqerisretmeiyaplahrlgisrikweledlafrylnetefykis  180
DSSP  hhhhhhhhhhhhlllhhhhhhhhhhlhhhhhhhlllhhhhhhhhhhhhhhhlhhhhhhhh


DSSP  LLLLHHHHHHHHHHHHHHHHHHH-LLLLllLLLLLEEEehhHHHHhhhllLLLL--------
Query QGVVFGRLQRHVAXIVVAQFAA-TLTDdtGAPLLLVkggSSLElrrgiPDSR--------   75
ident       |  |               |         |              |
Sbjct HMMNEKRREREALVDDIVTKIKsYTTE--QGLFGDV--yGRPK----hIYSIyrkmrdkk  232
DSSP  HHHHHLHHHHHHHHHHHHHHHHHhHHHL--LLLLLEE--eELLL----lHHHHhhhhhhh


DSSP  -------lLLLEEEEEL--LLHHHHHHHHHHHhhhllllleeEEEL--LLEEL--LLLLll
Query -------tSKDFATVAR--RDIELIHEQLADAgetgwegftAIFT--APEEI--AAPGxp  122
ident               |     |                                 |
Sbjct krfdqifdLIAIRCVMEtqSDVYAMVGYIHEL---------WRPMpgRFKDYiaAPKAng  283
DSSP  hhllllhhHLEEEEEELlhHHHHHHHHHHHHH---------LLLLllLLEELllLLLLll


DSSP  lLLEEEEEEEEelleeEEEEEEEEEELLlllllllleeeeelhhhhllllllleeeeellhh
Query vKPRRFTAKLSyrgraFATVPIEVSSVEagnadqfdtltsdalglvgvpaavavpcxtip  182
ident       |              |     |
Sbjct yQSIHTTVYGP-----KGPIEIQIRTKE--------------------------------  306
DSSP  lLLEEEEEELL-----LLEEEEEEEEHH--------------------------------


DSSP  hhhHHHHhHHHLlllllllllllhhhhhhhhhhhllhhhhhhhhhhhhhhhhhhlllll
Query wqiAQKLhAVTAvleepkandrahdlvdlqlleglllldadlxptrsaciaifearaqhpw  242
ident          | |
Sbjct mhqVAEY-GVAA------------------------------------------------  317
DSSP  hhhHHHH-LLLL------------------------------------------------


DSSP  lllllLLLLHHhhhhhhhllllllllllhhhhhhhhhhhhhhhhhllll
Query pprvaTLPHWPliyagalegldhlelartvdaaaqavqrfvaridratkr  292
ident
Sbjct -----NWIKEL------------------------------------vel  326
DSSP  -----LHHHHH------------------------------------hhl
```

No 592: Query=mol1A Sbjct=1zelA Z-score=4.4

[back to top](#)

```
DSSP  llllllLLLL--------------------------------------------------
```

```
Sbjct --amvvSPAGadrriptwasrvvsglardrpvvvtkedltqrlteagcgrdpdsairelr   58
DSSP  --leeeLLLLllllllhhhhhhhhhhhhhhllllleehhhhhhhhhhhlllllllhhhhhhhhh


DSSP  --------------hhhHHHHhhhhhhhllllhhhhhhhHHHHHHHHHHHLlllllLLLL
Query --------------lrsLRDRltqvaerqgvvfgrlqrhVAXIVVAQFAATltddtGAPL   56
ident 
Sbjct rigwlvqlpvkgtwafiPPGE---------------aaiSDPYLPLRSWLA---rdQNAG  100
DSSP  hhlleeellllleeeelLLLL---------------leeELLLHHHHHHHH---hlLLLL


DSSP  EEEHHHHHHHHHllllLLLLL-LEEEEELLLHhHHHHHHhhhhhhlllleeeeellllee
Query LLVKGGSSLELRRgipdSRTSK-DFATVARRDIeLIHEQLadagetgwegftaiftapee  115
ident     | |           |                    |
Sbjct FMLAGASAAWHLG--ylDRQPDgRIPIWLPPAK-RLPDGL--------------------  137
DSSP  LEELHHHHHHHHL--llLLLLLlLEEEELLLLL-LLLHHH--------------------


DSSP  llllllllleeeeeeeeeelleeeeeeEEEEEELLLL--lLLLLE---------------
Query iaapgxpvkprrftaklsyrgrafatvPIEVSSVEAG--nADQFD---------------  158
ident                               |
Sbjct -------------------------asYVSVVRIPWNaadTALLAprpallvrrrldlva  172
DSSP  -------------------------llLEEEELLLLLlllHHHHLllhhhhhhlllllll


DSSP  EEEElhhhhllllllleeEEELLHHHHHHHHHHHHHhllllllllllHHHHHHHHHHHHL
Query TLTSdalglvgvpaavaVPCXTIPWQIAQKLHAVtavleepkandraHDLVDLQLLEGLL  218
ident   |               |         |                          |  |
Sbjct WATG-------------LPALGPEALLVQIATRP---------asfgPWADLVPHLDDLV  210
DSSP  LLLL-------------LLLLLHHHHHHHHHHHLH---------hhllLHHHHHHHHHHHH


DSSP  LhhHHHHHHHHHHhHHHHhhlLLLLLL---------------------------------
Query LdaDLXPTRSACiAIFEaraQHPWPP---------------------------------  244
ident                        |
Sbjct AdcSDERLERLL-SGRP---TSAWQRasylldsggepargqallakrhtevmpvtrftta  266
DSSP  HllLHHHHHHHH-LLLL---HHHHHHhhhhhhlllhhhhhhhhllllllllleeelll


DSSP  ------------LLLLlllhhhhhhhhhlllllllLLLHHhhhhhhhhhhhhhhhhllll
Query ------------RVATlphwpliyagalegldhlelARTVDaaaqavqrfvaridratkr  292
ident              |                       |
Sbjct hsgesvwapeyqLVDE---------------lvvplLRVIG------------------k  293
DSSP  llllleeehhhlEEEL---------------lhhhhHHHHH------------------l
```

No 593: Query=mol1A Sbjct=5cwuA Z-score=4.3

back to top

```
DSSP  lllllllllhhhhhhhhhhhhhhhllllhhhhhhhhhhhhhhHHHHllllllllllEEEE
Query aapysspptnlrslrdrltqvaerqgvvfgrlqrhvaxivvaQFAAtltddtgaplLLVK   60
ident 
Sbjct ------------------------------------------PRDV--thllalvsSAIH   16
DSSP  ------------------------------------------LLLL--lhhhhhhhHHHH


DSSP  -------------HHHHHHHHHLlllllllllleeeeellllhhhhhhhhhhhhhhlllllee
Query -------------GGSSLELRRGipdsrtskdfatvarrdieliheqladagetgwegft  107
ident                  |
Sbjct avenpfgandlpyFRTLLKILFV-------------------------------------   39
DSSP  llllllllllhhhHHHHHHHHHH-------------------------------------


DSSP  eeellleellllllllllleeeeeeeeelleeeeeeeeeeeellllllLLLEeeeelhhhh
Query aiftapeeiaapgxpvkprrftaklsyrgrafatvpievssveagnaDQFDtltsdalgl  167
ident                                                 |
Sbjct ---------------------------------------vlraakqiTQQV---------   51
DSSP  ---------------------------------------hhhlllllHHHH---------


DSSP  llllleeeeelLHHH-HHHHHHHHHHLLLllllLLLLLHHHHHHHHHHHHHL------lh
Query vgvpaavavpcxTIPW-QIAQKLHAVTAVLeepkANDRAHDLVDLQLLEGLL------ld  220
ident             ||     |    |  |            ||
Sbjct -----------lTILDrVVARCFRALAALV----TTTAPEDLALITAILQAClsvpgieq   96
DSSP  -----------hHHHHhLHHHHHHHHLLLLL----LLLLLLHHHHHHHHHHHHHhllllllhh
```

```
DSSP  HHHHHhHHHHHHHHhhhllllLLLL--LLLLllhhhhhhhhhlllllllllLLHHHHHHH
Query ADLXPtRSACIAIFearaqhpWPPR--VATLphwpliyagalegldhlelaRTVDAAAQA  278
ident            |                                             |
Sbjct CQVQV-LNIXAAHD------vFQVAvaLFSW---------------adsgdPVYGELALL  134
DSSP  HHHHH-HHHHHHLL------hHHHHhhHHHL---------------lllllLHHHHHHHH


DSSP  HHHHHHH--hHHLL----------------------------------------------
Query VQRFVAR--iDRAT----------------------------------------------  290
ident
Sbjct FLLELSAlpaLAEHlacdgllghlaaarlagyxrrtnvgpfaenagaarcyaiwakcllp  194
DSSP  HHHHHLLlhhHHHHhhhhlhhhhhhlllhhhhhllllllllllhhhhhhhhhhhhlhhh


DSSP  ------------------------------------------------------------
Query ------------------------------------------------------------  290
ident
Sbjct lllnilaalgstvapevawvlnqfpnllqssverislleiseihslalltrvlaacdvpe  254
DSSP  hhhhhhlllhhhhhhhhhhhhhhllhhhhhhhhhhlllhhhhhhhhhhhhhhllhhhhlllll


DSSP  ------------------------------------------ll
Query ------------------------------------------kr  292
ident
Sbjct vtwdgakvlecveywlrgrkvlrernrleekavgllvgvrevle  298
DSSP  lllllhhhhhhhlllllllllllllhhhhlhhhhhhhhhhhhll
```

No 594: Query=mol1A Sbjct=1dk5A Z-score=4.3

back to top

```
DSSP  ---------LLLLLLL--LLLHHHhhhhhhhhhhhhllllhhhhhhhhhhhhhhhhhlll
Query ---------AAPYSSP--PTNLRSlrdrltqvaerqgvvfgrlqrhvaxivvaqfaatlt   49
ident
Sbjct hhhhmasltVPAHVPSaaEDCEQL------------------------------------   24
DSSP  lllllllllLLLLLLLhhHHHHHH------------------------------------


DSSP  llllllleeeehhhhhhhhhlllllllllleeeeelllhhhhhhhhhhhhhhlLLLEeee
Query ddtgaplllvkggsslelrrgipdsrtskdfatvarrdieliheqladagetgWEGFtai  109
ident
Sbjct ---------------------------rsafkgwgtnekliisilahrtaaqrKLIR---   54
DSSP  ---------------------------hllllllllllhhhhhhhhllllhhhhHHHH---


DSSP  ellleellllllllllleeeeeeeeELLE--------------------------------
Query ftapeeiaapgxpvkprrftaklsYRGR--------------------------------  137
ident
Sbjct -----------------------qTYAEtfgedllkeldrelthdfeklvlvwtldpser   91
DSSP  -----------------------hHHHHhhlllhhhhllllllhhhhhhhhhhllhhhh


DSSP  ------------------------------------eeeeeeeeeeelllllllllleeee
Query ------------------------------------afatvpievssveagnadqfdtlt  161
ident
Sbjct dahlakeatkrwtksnfvlvelactrspkelvlareayharykksleedvayhttgdhrk  151
DSSP  hhhhhhhhllhhhllhhhhhhhhhhlllhhhhhhhhhhhhhhhhlllhhhhhhhhllhhhhh


DSSP  elhhhhllllleeeeelLHHHHHHHHHHHHHHLlllllllllLLHHHH-HHHHHHHHHLLH
Query sdalglvgvpaavavpcxTIPWQIAQKLHAVTAvleepkandRAHDLV-DLQLLEGLLLD  220
ident                            ||
Sbjct llvplvssyryggeevdlRLAKAESKILHEKIS---------DKAYSDdEVIRILATRSK  202
DSSP  hhhhhhllllllllllhHHHHHHHHHHHHHHHH---------LLLLLLhHHHHHHHHHLLH


DSSP  HHHHHHHHHHHHHHHhhlllLLLL-LLLLlllhhhhhhhhhlllllllllllhHHHHHHH
Query ADLXPTRSACIAIFEaraqhPWPP-RVATlphwpliyagalegldhlelartvDAAAQAV  279
ident | |  |                                               |
Sbjct AQLNATLNHYKDEHG-----EDILkQLED----------------------gDEFVALL  234
DSSP  HHHHHHHHHHHHHLL-----LLHHhHLLL----------------------lLHHHHHH


DSSP  HHHHHHHHH---------------------------------------------------
Query QRFVARIDR---------------------------------------------------  288
```

```
DSSP  HHHHHHHHLhhhhhhhhhhhhhlllllllhhhhhhhhhhhhhlllhhhhhhhhhhhhhlllhh


DSSP  ------------------llll
Query ------------------atkr  292
ident
Sbjct raiakdtrgdyesmllallgqe  316
DSSP  hhhhhlllhhhhhhhhhhllll
```

No 595: Query=mol1A Sbjct=4k96B Z-score=4.3

back to top

```
DSSP  lllllllllhhhhhhhhhhhhhlLLLHHHHHHHHHHHHHHHHHHHLLLLLLLL-LLEEE
Query aapyssppptnlrslrdrltqvaerqGVVFGRLQRHVAXIVVAQFAATLTDDTGA-PLLLV   59
ident                                        ||
Sbjct -----------spdklkkvldklrlKRKDISEAAETVNKVVERLLRRMQKRESEfKGVEQ   49
DSSP  -----------lhhhhhhhhhhhllLHHHHHHHHHHHHHHHHHHHHHHHHLLLLLLlLLLEE


DSSP  EHHhhhhhhhlllLLLL-----lLLEEEEEL-----------------------------
Query KGGsslelrrgipDSRT-----sKDFATVAR-----------------------------   85
ident                         |
Sbjct LNT------gsyyEHVKisapneFDVMFKLEvprielqeyyetgafylvkfkprgnplsh  103
DSSP  EEE------lhhhHLLLllllleEEEEEEEElleeeeellllleeeeeellllllhh


DSSP  ---------lLHHHHHHHH-HHHHHHLlLLEEEEELLLEELlllllllllEEEEEEEEEl
Query ---------rDIELIHEQL-ADAGETGwEGFTAIFTAPEEIaapgxpvkpRRFTAKLSYr  135
ident                                                      |
Sbjct flegevlsatKMLSKFRKIiKEEVKEI-KDIDVSVEKEKPG--------sPAVTLLIRN-  153
DSSP  heelleelhhHHHHHHHHHHhHHHHLLL-LLLEEEELLLLLL--------lLEEEEEEEL-


DSSP  leeEEEEEEEEEELLLL-----llllleeeeelhhhhllllllleeeeellhhhhhhhhhh
Query graFATVPIEVSSVEAG-----nadqfdtltsdalglvgvpaavavpcxtipwqiaqklh  190
ident       | |       |
Sbjct -peEISVDIILALESKGswpistkeglpiqgwlgtkvrtnlrrepfylvpknakdgnsfq  212
DSSP  -llEEEEEEEEEEEELLlllhhhllllllllllhhhhhhhllllleeeeellllllllll


DSSP  hhhllllllllLLLHhhhhhhhhhhhllhhhhhhhhhhhhhhhhhhlllllllllll
Query avtavleepkanDRAHdlvdlqllegllldadlxptrsaciaifearaqhpwpprvatlp  250
ident
Sbjct getwrlsfshteKYIL------------------------------nnhgiektccess  241
DSSP  lleeeeelhhhhHHHH------------------------------hlllllllllll


DSSP  lhhhhHHHHHL-lLLLL-----------------llllLHHHHHHH-------------
Query hwpliYAGALE-gLDHL-----------------elarTVDAAAQA--------------  278
ident          |      |                           |
Sbjct gakccRKECLKlmKYLLeqlkkefqeldafcsyhvktaIFHMWTQDpqdsqwdprnlssc  301
DSSP  lllllHHHHHHhhHHHHhhhhhhlhhhllllhhhhhhhHHHHHHHLllhhhhlhhhhhhh


DSSP  -----------------hhhhHHHHH--------------------------hllll
Query -----------------vqrfVARID--------------------------ratkr  292
ident
Sbjct fdkllaffleclrtekldhyfIPKFNlfsqelidrkskeflskkieyernngfpifd  358
DSSP  hhhhhhhhhhhhlllllllLLLLLlllllllllhhhhhhhhhhhhhhhhlllhhhl
```

No 596: Query=mol1A Sbjct=1r8bA Z-score=4.3

back to top

```
DSSP  llllllllllhhhhhhhhhhhhhhllLLHHHHHHHHHHHHHHHHHHHHLLLlllllLLEEEE
Query aapyssppptnlrslrdrltqvaerqgVVFGRLQRHVAXIVVAQFAATLTddtgaPLLLVK   60
ident                                     |          |
Sbjct ----------mkveeilekalelvipDEEEVRKGREAEEELRRRLDELG-----VEYVFV   45
DSSP  ----------llhhhhhhhhhhhhllLHHHHHHHHHHHHHHHHHHHHHHL-----LLEEEE
```

```
ident |              |                        |
Sbjct GSYARN-----twLKGSLEIDVFLLFpeefskeelreRGLEIGKAV-------------l   87
DSSP  HHHHHL-----llLLLLLEEEEEEEEllllhhhhhhHHHHHHHHH-------------l


DSSP  ELLLEELLLllllLLLEEEEEEEeeelleeeeEEEEEEEELLLLllllleeeeelhhhhll
Query FTAPEEIAApgxpVKPRRFTAKlsyrgrafaTVPIEVSSVEAGnadqfdtltsdalglvg  169
ident        |             |          |   |
Sbjct DSYEIRYAE----HPYVHGVVK---------GVEVDVVPCYKL----------kepknik  124
DSSP  LEEEEEELL----LEEEEEEEL---------LEEEEEEEEELL----------lllllll


DSSP  llllleeeeeLLHHH-hhhhhhhhhhllllllllLLLHH------HHHHHHHHHHLLHhh
Query vpaavavpcXTIPW-qiaqklhavtavleepkanDRAHD------LVDLQLLEGLLLDad  222
ident
Sbjct savdrtpfhHKWLEgrikgkenevrllkgflkanGIYGAeykvrgFSGYLCELLIVFY--  182
DSSP  lllllhhhhHHHHLllllllhhhhhhhhhhhhhlLLLLLllllllLLHHHHHHHHHHH--


DSSP  hhhhhhhhhhhhhhhlllllllllllllhhhhhhhhhllllllllllhhhhhhhhHHH
Query lxptrsaciaifearaqhpwpprvatlphwpliyagalegldhlelartvdaaaqavQRF  282
ident
Sbjct --------------------------gsfletvknarrwtrrtvidvakgevrkgeeFFV  216
DSSP  --------------------------llhhhhhhhllllllleeeehhhleeeellllLEE


DSSP  H-----------------------------------------------------------
Query V-----------------------------------------------------------  283
ident |
Sbjct Vdpvdekrnvaanlsldnlarfvhlcrefmeapslgffkpkhpleieperlrkiveergt  276
DSSP  Eelleeeeellllllhhhhhhhhhhhhhhhhhlllhhhhllllllllllhhhhhhhhhhhll


DSSP  ------------------------------------------------------------
Query ------------------------------------------------------------  283
ident
Sbjct avfavkfrkpdivddnlypqlerasrkifeflerenfmplrsafkaseefcyllfecqik  336
DSSP  eeeeeeeelllllhhhhhhhhhhhhhhhhhhhhhllllleeeeeeeellleeeeeeeelll


DSSP  ------------------------------------------------------------
Query ------------------------------------------------------------  283
ident
Sbjct eisrvfrrmgpqfedernvkkflsrnrafrpfiengrwwafemrkfttpeegvrsyasth  396
DSSP  lllleeeeeeeelllhhhhhhhhhlllllllleeelleeeeeeelllllhhhhhhhhhhhl


DSSP  --------------------------------hhhhhllll
Query --------------------------------aridratkr  292
ident
Sbjct whtlgknvgesireyfeiisgeklfkepvtaelcemmgvkd  437
DSSP  hhhllhhhhhhhhhlleeeehhhhllllllhhhhhhhlllll
```

No 597: Query=mol1A Sbjct=3au6A Z-score=4.3

back to top

```
DSSP  ---------llLLLL---------------llLLHHHHH---------------------
Query ---------aaPYSS---------------ppTNLRSLR---------------------   15
ident
Sbjct mrnqelarifeEIGLmseflgdnpfrvrayhqAARTLYDldtpieeiaekgkealmelpg   60
DSSP  llhhhhhhhhHHHHhhhhhlllhhhhhhhhhHHHHHLLllllhhhhhlllllllllll


DSSP  ------------------------------------------------------------
Query ------------------------------------------------------------   15
ident
Sbjct vgpdlaekileflrtgkvrkheelsrkvprgvlevmevpgvgpktarllyeglgidslek  120
DSSP  llhhhhhhhhhhhlllllhhhhhhhhhllhhhhhhhlllllhhhhhhhhhhhllllhhh


DSSP  ------------hhhhhhhHHLL--LLHHHhHHHHHHHHHHHHHH-LLLLlllllLEEEE
Query ------------drltqvaERQG--VVFGRlQRHVAXIVVAQFAA-TLTDdtgapLLLVK   60
ident                              |      |     |
Sbjct lkaaldrgfgpkraerireGLALaqAAGKR-RPLGAVLSLARSLLeAIRAlpgveRAELC  179
```

```
DSSP  HHHHHHHhhlllllLLLLEEEEEL-----lLHHHHHHHhhhhhhhlllleeEEELLLEE
Query GGSSLELrrgipdsrTSKDFATVAR-----rDIELIHEQladagetgwegftAIFTAPEE  115
ident |              |  |              |
Sbjct GSARRYK-------dTVGDLDFLVAsregerAVEGFVRL------------pQVKEVYAK  220
DSSP  HHHHLLL-------lEELLEEEEELLllllllHHHHHLLL------------lLEEEEEEL


DSSP  LllllllLLLEEEEEEEEeelleeeeEEEEEEEEELLLLLllllleeeeelhhhhllllllee
Query IaapgxpVKPRRFTAKLsyrgrafaTVPIEVSSVEAGNadqfdtltsdalglvgvpaava  175
ident                |                 |
Sbjct G------KERATVFLKN--------GLQVDLRVVPPES----------------------  244
DSSP  L------LLEEEEEELL--------LLEEEEELLLHHH----------------------


DSSP  eeellhHHHHhhhhhhhhlllllllllllHHHHHHH-----------hhhhhllhHHHH
Query vpcxtiPWQIaqklhavtavleepkandraHDLVDLQ-----------lleglldADLX  224
ident
Sbjct ----ygAGLQ----------------yltgSKAHSIRlralaqekglklseygvfrGEKR  284
DSSP  ----hhHHHH----------------hhhlLHHHHHHhhhhhhhllleellllleeeLLEE


DSSP  hhHHHHhhhhhhhlllllLLLLLllllhhhhhhhhhlllllllllLLHHHHHH-------
Query ptRSACiaifearaqhpwPPRVAtlphwpliyagalegldhlelaRTVDAAAQ-------  277
ident                                                   ||
Sbjct --IAGE-----teeevyaALGLP-------------wipppplredQGEVEAALegrlpkl  324
DSSP  --EELL-----lhhhhhhHLLLL-------------lllhhhlllLLHHHHHLlllllll


DSSP  ------------------------------------------------------------
Query ------------------------------------------------------------  277
ident
Sbjct lelpqvkgdlqvhstysdgqntleelweaaktmgyrylavtdhspavrvgpspeealkrv  384
DSSP  llhhhlleellllllllllllhhhhhhhhhhhllleeelleellllllllllhhhhhhhh


DSSP  ------------------------------------------------------------
Query ------------------------------------------------------------  277
ident
Sbjct geirrfnethgppyllagaevdihpdgtldypdwvlreldlvlvsvhsrfnlpkadqtkr  444
DSSP  hhhhhhhlllllleeeleeeellllllllllhhhhlllleeeelllllllllhhhhhhh


DSSP  ------------------------------------------------------------
Query ------------------------------------------------------------  277
ident
Sbjct llkalenpfvhvlahptarllgrrapieadweavfqkakekgvaveidgyydrmdlpddl  504
DSSP  hhhhlllllllleellllllllllllllllhhhhhhhhhllllleeeeellllllllhhh


DSSP  -------------------------------------------hhhhhhhhhhhllll
Query -------------------------------------------avqrfvaridratkr  292
ident
Sbjct armaygmglwislstdahqtdhlrfmelavgtaqrawigpervlntldyedllswlka  562
DSSP  hhhhhllllleeeellllllhhhhhhhhhhhhhhhhhllllllllhhhllhhhhhhhhhl
```

No 598: Query=mol1A Sbjct=4wc6A Z-score=4.3

back to top

```
DSSP  lllllllllhhhhhhhhhhhhhllllhhhhhhhhhhhhhhhHHHLLLLLlLLLEEEE
Query aapyssspptnlrslrdrltqvaerqgvvfgrlqrhvaxivvaqFAATLTDDtgAPLLLVK   60
ident
Sbjct -------------------------------------------MVGQIAKE-mGLRAYIV   16
DSSP  -------------------------------------------LHHHHHHH-lLLEEEEE


DSSP  HHHHHHHHHLLlllllLLLEEEEELLLHHHHHHHHHHHHHhllleeeeELLLEEllll
Query GGSSLELRRGIpdsrtSKDFATVARRDIELIHEQLADAGEtgwegftaiFTAPEEiaapg  120
ident ||       |        |   |           ||
Sbjct GGVVRDILLGK----eVWDVDFVVEGNAIELAKELARRHG---------VNVHPF-----   58
DSSP  LHHHHHHHLLL----lLLEEEEEEELLHHHHHHHHHHHLL---------LLLEEE-----


DSSP  llLLLEEEEEEeeelleeeeEEEEEEEEELLL----LLLL--------------------
```

```
Sbjct perGIAHLKIG---------KLKLEFAIAKketepASLKedIirrdftinamaisvnled  109
DSSP  llLLEEEEEEL---------LEEEEEEELLLllllLLHHhhhllllhhhlleeellllll


DSSP  LLEEeeelhhhhlllllleeeeelLHHH---------hhhhhhhhHHLLllllllLLLLh
Query QFDTltsdalglvgvpaavavpcxTIPW---------qiaqklhaVTAVleepkaNDRAh  206
ident                                                          |
Sbjct YGTL--------------------IDYFgglrdlkdkvirvlhpvSFIE-----dPVRI-  143
DSSP  LLLE--------------------ELLLlhhhhhhlleellllllHHHH-----lLLHH-


DSSP  hhhhhhhhhhllhhhhhhhhhhhhhhhhhhlllllllllllllllhhhHHHHHHLLLLll
Query dlvdlqllegllldadlxptrsaciaifearaqhpwpprvatlphwplIYAGALEGLDhl  266
ident                                                         |
Sbjct -----------------------------------------------lRALRFAGRLN--  154
DSSP  -----------------------------------------------hHHHHHHHHLL--


DSSP  lllLLHHHHHHH------------------------------------------------
Query elaRTVDAAAQA------------------------------------------------  278
ident
Sbjct --fKLSRSTEKLlkqavnlgllkeaprgrlineiklalredrfleilelyrkyrvleeii  212
DSSP  --lEELHHHHHHhhhhhhllhhhhllhhhhhhhhhhhhllllhhhhhhhhhhllhhhhhl


DSSP  ------------------------------------------------------------
Query ------------------------------------------------------------  278
ident
Sbjct egfqwnekvlqklyalrkvvdwhalefseeridygwlyllilisnldyergkhfleemsa  272
DSSP  lllllhhhhhhhhhhhhhhhhhhhhlhhhllllhhhhhhhhhlllllhhhhhhhhhhlll


DSSP  ------------------------------------------------------------
Query ------------------------------------------------------------  278
ident
Sbjct pswvretykfmkfklgslkeelkkakenyevyrllkplhtsvllllmleeelkekiklyl  332
DSSP  lhhhhhhhhhhlhhhhhhhhhhllllhhhhhhhhllllhhhhhhhlllhhhhhhhhhhhh


DSSP  ---------------------hhhhhhhhhhllll
Query ---------------------vqrfvaridratkr  292
ident
Sbjct eklrkvklpkekieelkkgkelgerieelkreimn  367
DSSP  hllllllllhhhhhhhhllhhhhhhhhhhhhhhhl
```

No 599: Query=mol1A Sbjct=3c2gA Z-score=4.2

back to top

```
DSSP  ----------------------------------------------------LLLL--lL
Query ----------------------------------------------------AAPY--sS    6
ident
Sbjct xnitqaaeqairlwfntpdpxqrlhxaktirtwirqdkfaqvdqanxpncvqQILNiiyD   60
DSSP  llhhhhhhhhhhhhhhlllhhhhhhhhhhhhhhhhhhhlllllhhhhhhhhhHHHHhhhH


DSSP  LLL----------LHHHHHHHHHHHHHHLL------------------------------
Query PPT----------NLRSLRDRLTQVAERQG------------------------------   26
ident                      |
Sbjct GLKpqpvqlpisyYAQLWYNLLDILRRFTFlpiispyihqvvqxfcprengpqdfrelic  120
DSSP  HHLllllllhhhHHHHHHHHHHHHHHHHHHlhhhhhhhhhhhhllllllllllllhhhhhh


DSSP  -------------llhHHHHHHH---------------hhhhhHHHHHllllllllllEE
Query -------------vvfGRLQRHV---------------axivvAQFAAtltddtgaplLL   58
ident                                              |
Sbjct nlislnwqkdphxkhcANQVFQIfnciixgvkneklrtefaqhLKFEK--------lvGT  172
DSSP  hhllhhhhhllllhhhHHHHHHHhhhhhhhlllhhhhhhhhhhHLLHH--------hhHH


DSSP  EE------------hHHHHHHHHLllllllllleeeeelllhhhhhhhhhhhhhhlllle
Query VK------------gGSSLELRRGipdsrtskdfatvarrdieliheqladagetgwegf  106
ident                       |
Sbjct LSeyfnpqvhpgxinPAIFIIFRF------------------------------------  196
DSSP  HHhhhlllllhhhllLHHHHHHHH------------------------------------
```

```
DSSP  eeeellleellllllllllleeeeeeeeellleeeeeeeeeeeelllLLLLLleeeeelhhh
Query taiftapeeiaapgxpvkprrftaklsyrgrafatvpievssveaGNADQfdtltsdalg  166
ident
Sbjct ------------------------iiskdtrlkdyfiwnnnphdqPPPPT----------  222
DSSP  ------------------------hhlllhhhhhhhhlllllllLLLLL----------


DSSP  hllllllleeeeelLHHHHHHHHHHHHHL--LLLL-------lllllLLHHHHHHHHHHHH
Query lvgvpaavavpcxTIPWQIAQKLHAVTA--VLEE-------pkandRAHDLVDLQLLEGL  217
ident                                                         | ||
Sbjct -------------GLIIKLNAVXIGSYRliAGQNpetlpqnpelahLIQVIIRTFDLLGL  269
DSSP  -------------LHHHHHHHHHHHHHHhhLLLLhhhhhhlhhhhhHHHHHHHHHHHHHH


DSSP  LLHH--HHHHHHHHHhhhhhhhllllLLLL--LLLLllhhhhhhhhhlllllllllllHH
Query LLDA--DLXPTRSACiaifearaqhpWPPR--VATLphwpliyagalegldhlelartVD  273
ident ||                              |
Sbjct LLHDsdAIDGFVRSD---------gvGAITtvVQYP----------------------NN  298
DSSP  HLLLhhHHHHHHHHLL---------hhHHHHhhLLLL----------------------LH


DSSP  HHHHHHHHHHHHHHHLLL------------------------------------------
Query AAAQAVQRFVARIDRATK------------------------------------------  291
ident     |          |
Sbjct DLIRAGCKLLLQVSDAKAlaktplenilpfllrlieihpddeviysgtgflsnvvahkqh  358
DSSP  HHHHHHHHHHHHHHLLLHHhhllllllhhhhhhhhhhhlllhhhhhhhhhhhhhhhllllhh


DSSP  ------------------------------------------------------------
Query ------------------------------------------------------------  291
ident
Sbjct vkdiairsnaifllhtiiskyprldeltdapkrnrvceiicnclrtlnnflxxwiptpng  418
DSSP  hhhhhhhllhhhhhhhhhhhllllhhhlllhhhhhhhhhhhhhhhhhhhhhhhhhllllll


DSSP  ------------------------------------------------------------
Query ------------------------------------------------------------  291
ident
Sbjct etktagpnekqqvckfieidilkklxsclscexdtpgllelrstilrsfilllrtpfvpk  478
DSSP  llllllhhhhhhhhhhhlhhhhhhhhhhhhlllllllhhhhhhhhhhhhhhhhhllllllll


DSSP  ------------------------------------------------------------
Query ------------------------------------------------------------  291
ident
Sbjct dgvlnvidenrkenlighicaayswvfrqpnntrtqstkqqlvertisllllvlxeqcgae  538
DSSP  llhhhllllllllllhhhhhhhhhhhhhhlllllllhhhhhhhhhhhhhhhhhhhhhhlllh


DSSP  ------------------------------------------------------------
Query ------------------------------------------------------------  291
ident
Sbjct kevaqysysidcplnllngnqvkptfihnvlvvcdkilehcptradiwtidrpxlegltn  598
DSSP  hhhhhllllllllhhhhhllllllhhhhhhhhhhhhhhhhhhhlllhhhhhllllhhhhhhhhl


DSSP  -------------------l
Query -------------------r  292
ident
Sbjct hrnsdiakaansllsrfpen  618
DSSP  lllhhhhhhhhhhhllllll
```

No 600: Query=mol1A Sbjct=4x4pG Z-score=4.2

back to top

```
DSSP  lllllllllhhhhhhhhhhhhhhllLLHHHHHHHHHHHHHHHHHHHHLLLlllllLLEEEE
Query aapysspptnlrslrdrltqvaerqgVVFGRLQRHVAXIVVAQFAATLTddtgaPLLLVK   60
ident                                     |          |
Sbjct -----------kveeilekalelvipDEEEVRKGREAEEELRRRLDELG-----VEYVFV   44
DSSP  -----------lhhhhhhhhhhhhhllLHHHHHHHHHHHHHHHHHHHHLLLL-----LLEEEE


DSSP  HHHHHHhhhlllLLLL-LLLEEEEELL-----------LHHHHHHHHhhhhhhllllleee
Query GGSSLElrrgipDSRT-SKDFATVARR-----------DIELIHEQLadagetgwegfta  108
```

```
DSSP  HHHHLL------LLLLlLLLEEEEEEEElllllhhhhhhhhhhhhLL-------------


DSSP  eELLLEELLLllllLLLEEEEEEEeeelleeeeEEEEEEEEL----LLLLllllleeeeelh
Query iFTAPEEIAApgxpVKPRRFTAKlsyrgrafaTVPIEVSSV----EAGNadqfdtltsda  164
ident         |             |          |   |
Sbjct lDSYEIRYAE----HPYVHGVVK---------GVEVDVVPCyksaVDRT-----------  121
DSSP  lLEEEEEELL----LEEEEEEEL---------LEEEEEEEEelllLLHH-----------


DSSP  hhhlllllleeeeeLLHH----------------hhhhhhhhHHLLllllllllllhhH
Query lglvgvpaavavpcXTIP----------------wqiaqklhaVTAVleepkandrahdL  208
ident
Sbjct -----------pfhHKWLegrikgkenevrllkgflkangiygAEYK---------vrgF  161
DSSP  -----------hhhHHHHhhhllllhhhhhhhhhhhhhhllllLLLL---------lllL


DSSP  HHHHHHHHHLL-------------------hHHHH----------hhHHHHHhhhhhll
Query VDLQLLEGLLL-------------------dADLX----------ptRSACIaifearaq  239
ident
Sbjct SGYLCELLIVFygsfletvknarrwtrrtviDVAKgevrkgeeffvvDPVDE-------k  214
DSSP  LHHHHHHHHHHhllhhhhhhhhllllllleeeELLLleeeellllleeeLLLLL-------l


DSSP  llllLLLLLLllhhhhhhhhhllllllllllLHHH-------------------------
Query hpwpPRVATLphwpliyagalegldhlelarTVDA-------------------------  274
ident
Sbjct rnvaANLSLD-------------------nlARFVhlcrefmeapslgffkpkhpleiep  255
DSSP  llllLLLLHH-------------------hhHHHHhhhhhhhhlllhhhhlllllllllh


DSSP  ------------------------------------------------------------
Query ------------------------------------------------------------  274
ident
Sbjct erlrkiveergtavfavkfrkpdivddnlypqlerasrkifeflerenfmplrsafkase  315
DSSP  hhhhhhhhhhlleeeeeeeeelllllhhhhhhhhhhhhhhhhhhhhllllleeeeeeeell


DSSP  ------------------------------------------------------------
Query ------------------------------------------------------------  274
ident
Sbjct efcyllfecqikeisrvfrrmgpqfedernvkkflsrnrafrpfiengrwwafemrkftt  375
DSSP  leeeeeeeelllllleeeeeeeeelllhhhhhhhhllllllleeelleeeeeeeelllll


DSSP  -----------------------------------hhhhhhhhhhhhhhllll
Query -----------------------------------aaqavqrfvaridratkr  292
ident
Sbjct peegvrsyasthwhtlgknvgesireyfeiisgeklfkepvtaelcemmgvkd  428
DSSP  hhhhhhhhhhlhhhhlhhhhhhhhhhlleeeehhhllllllhhhhhhhhllll
```

No 601: Query=mol1A Sbjct=4wbzB Z-score=4.2

back to top

```
DSSP  lllllllllhhhhhhhhhhhhhhhllllhhhhhhhhhhhhhhhHHHLLLLLllLLLEEEE
Query aapysspptnlrslrdrltqvaerqgvvfgrlqrhvaxivvaqFAATLTDDtgAPLLLVK   60
ident
Sbjct -------------------------------------------MVGQIAKE-mGLRAYIV   16
DSSP  -------------------------------------------LHHHHHHH-lLLEEEEE


DSSP  HHHHHHHHHLLlllllLLLEEEEELLLHHHHHHHHHHHHHhlllleeeEELLLEElllll
Query GGSSLELRRGIpdsrtSKDFATVARRDIELIHEQLADAGEtgwegftaIFTAPEEiaapg  120
ident ||       |        |   |           ||                |
Sbjct GGVVRDILLGK----eVWDVDFVVEGNAIELAKELARRHG--------VNVHPFP-----   59
DSSP  LHHHHHHHLLL----lLLEEEEEEEELLHHHHHHHHHHHL--------LLEEEEL-----


DSSP  llLLLEEEEEEeeelleeeeEEEEEEEELLL--LLLL-lleeeeelhhhhlllllleeee
Query xpVKPRRFTAKlsyrgrafaTVPIEVSSVEA--GNAD-qfdtltsdalglvgvpaavavp  177
ident                         |
Sbjct -eFGTAHLKIG---------KLKLEFATARRepASLKedlirrdftinamaisvnledyg  109
DSSP  -lLLEEEEEEL---------LEEEEEEEELLLllLLHHhhhhllllhhhlleeellllll
```

```
Query cxTIPW---------qiaqklhaVTAVleepkaNDRAhdlvdlqllegllldadlxptrs  228
ident                                    |
Sbjct tlIDYFgglrdlkdkvirvlhpvSFIE-----dPVRI-----------------------  141
DSSP  leELLLlhhhhhhlleellllllHHHH-----lLHHH-----------------------


DSSP  hhhhhhhhhllllllllllllllhhhHHHHHHLLLLlllllLLHHHHHHH----------
Query aciaifearaqhpwpprvatlphwplIYAGALEGLDhlelaRTVDAAAQA----------  278
ident                                   |
Sbjct -------------------------lRALRFAGRLN----fKLSRSTEKLlkqavnlgll  172
DSSP  -------------------------hHHHHHHHHHL----lEELHHHHHHhhhhhhllll


DSSP  ------------------------------------------------------------
Query ------------------------------------------------------------  278
ident
Sbjct keaprgrlineiklalredrfleilelyrkyrvleeiiegfqwnekvlqklyalrkvvdw  232
DSSP  llllhhhhhhhhhhhhllllhhhhhhhhhhllhhhhhlllllllhhhhhhhhhhhhhhhh


DSSP  ------------------------------------------------------------
Query ------------------------------------------------------------  278
ident
Sbjct halefseeridygwlyllilisnldyergkhfleemsapswvretykfmkfklgslkeel  292
DSSP  hhhhlllllllllhhhhhhhhlllllhhhhhhhhhhllllhhhhhhhhhhlllhhhhhhhh


DSSP  --------------------------------------------hhhhhhhhhhllll
Query --------------------------------------------vqrfvaridratkr  292
ident
Sbjct kkakenyevyrllkplhtsvllllmleeelkekiklyleklrkvkerieelkreimnk  350
DSSP  hllllhhhhhhhhllllhhhhhhlllllllhhhhhhhhhlhhhlllhhhhhhhhhhll
```

No 602: Query=mol1A Sbjct=4wc0B Z-score=4.2

back to top

```
DSSP  llllllllllhhhhhhhhhhhhhhhllllhhhhhhhhhhhhhhhHHHLLLLLLlLLLLEEEE
Query aapysspptnlrslrdrltqvaerqgvvfgrlqrhvaxivvaqFAATLTDDtGAPLLLVK   60
ident
Sbjct -------------------------------------------MVGQIAKE-MGLRAYIV   16
DSSP  -------------------------------------------LHHHHHHH-HLLEEEEE


DSSP  HHHHHHHHHLLlllllLLLEEEEEELLLHHHHHHHHHHHHHHhllllleeeeELLLEElllll
Query GGSSLELRRGIpdsrtSKDFATVARRDIELIHEQLADAGEtgwegftaiFTAPEEiaapg  120
ident ||       |        |   |           ||
Sbjct GGVVRDILLGK----eVWDVDFVVEGNAIELAKELARRHG---------VNVHPF-----   58
DSSP  LHHHHHHHHLLL----lLLEEEEEEEELLHHHHHHHHHHHHHL---------LLLEEE-----


DSSP  llLLLEEEEEEeeelleeeeEEEEEEEEELLL--LLLL-lleeeeelhhhhlllllleeee
Query xpVKPRRFTAKlsyrgrafaTVPIEVSSVEA--GNAD-qfdtltsdalglvgvpaavavp  177
ident                         |
Sbjct peFGTAHLKIG---------KLKLEFATARRepASLKedlirrdftinamaisvnledyg  109
DSSP  llLLEEEEEEL---------LEEEEEEELLLllLLHHhhhhllllhhhlleeellllllll


DSSP  elLHHH---------hhhhhhhhHHLLlllllLLLLhhhhhhhhhhhhllhhhhhhhhh
Query cxTIPW---------qiaqklhaVTAVleepkaNDRAhdlvdlqllegllldadlxptrs  228
ident                                    |
Sbjct tlIDYFgglrdlkdkvirvlhpvSFIE-----dPVRI-----------------------  141
DSSP  leELLLlhhhhhhlleellllllHHHH-----lLHHH-----------------------


DSSP  hhhhhhhhhllllllllllllllhhhHHHHHHLLLLlllllLLHHHHHHH----------
Query aciaifearaqhpwpprvatlphwplIYAGALEGLDhlelaRTVDAAAQA----------  278
ident                                   |
Sbjct -------------------------lRALRFAGRLN----fKLSRSTEKLlkqavnlgll  172
DSSP  -------------------------hHHHHHHHHHL----lEELHHHHHHhhhhhhllll


DSSP  ------------------------------------------------------------
Query ------------------------------------------------------------  278
ident
```

```
DSSP  ------------------------------------------------------------
Query ------------------------------------------------------------  278
ident 
Sbjct halefseeridygwlyllilisnldyergkhfleemsapswvretykfmkfklgslkeel  292
DSSP  hhhhllllllllhhhhhhhhlllllhhhhhhhhhhllllhhhhhhhhhhlllhhhhhhhh


DSSP  --------------------------------------------hhhhhhhhhhllll
Query --------------------------------------------vqrfvaridratkr  292
ident 
Sbjct kkakenyevyrllkplhtsvllllmleeelkekiklyleklrkvkerieelkreimnk  350
DSSP  hllllhhhhhhhhllllhhhhhhllllllhhhhhhhhhlhhhlllhhhhhhhhhhll
```

No 603: Query=mol1A Sbjct=4woyA Z-score=4.1

back to top

```
DSSP  lllllllllhhhhhhhhhhhhhhLLLLhHHHHHHHHHHHHH-HHHHLLL---LLLL---
Query aapysspptnlrslrdrltqvaerQGVVfGRLQRHVAXIVVA-QFAATLT---DDTG---   53
ident                            |             |   |  |
Sbjct --------------tfqerllafeRKHViTPEAHVTLAKQLAgDIALELQaylRSKFpel   46
DSSP  --------------lhhhhhhhhhHHHLlLLHHHHHHHHHHHHhHHHHHHHHhhhHHHLlll


DSSP  -llLEEEEHHHHHhhHHLLlllllLLLEEEEEEL---------------------------
Query -apLLLVKGGSSLelRRGIpdsrtSKDFATVAR---------------------------   85
ident     |   |                      |
Sbjct pfgALVPGGPLYD--GLQA---gtAEHVRLLAPlelepglwslvpgvdtvaaeprcwavr  101
DSSP  lllLLEEELLLLL--LLLL---llLLLEEEEEEllllllleeeeehhhllllllleeeee


DSSP  ------------------------------lLHHHH--hHHHHHHHHHHLllleEEEELLL
Query ------------------------------rDIELI--hEQLADAGETGwegfTAIFTAP  113
ident                                              |
Sbjct rtqlefhprgcspwdrflvggylssrvllelLRKALsasVNWPAIGSLL----GCLIWPD  157
DSSP  elllllllllllhhhhheelleelhhhhhhhHHHHHhhlLLHHHHHHHH----LLEEEEL


DSSP  EELlllllllllEEEEEEEEEllEEEEEEEEEEEELLLLllllleeeeelhhhhllllll
Query EEIaapgxpvkpRRFTAKLSYrgrAFATVPIEVSSVEAGnadqfdtltsdalglvgvpaa  173
ident                  |              |  |  |
Sbjct VAS---------EELLLKVQH---ECLEFTLAVLMVVPG------------astddrlll  193
DSSP  LLL---------LEEEEELLL---LLLEEEEEEEEEEELL------------llllllleee


DSSP  eeeeellhhhhhhHHHHhhhllllllllllllhHHHHHHHHHHHHLlhhhhhhhhhhhhhh
Query vavpcxtipwqiaQKLHavtavleepkandrahDLVDLQLLEGLLldadlxptrsaciai  233
ident              | |                       |
Sbjct awpleglasnlwlQDLY----------------PVETARLRALDD---------------  222
DSSP  lllllllllllleeEELH----------------HHHHHHHHHHHH---------------


DSSP  hhhhllllllllllllllhhhhhhhHHLLL-----------------llllLLLLHHH--
Query fearaqhpwpprvatlphwpliyagALEGL-----------------dhleLARTVDA--  274
ident                           |  |
Sbjct -----------------qdagtrrrLLLLcgicrghpalvrlgwshltqvVLHLGEEev  265
DSSP  -----------------hhlllhhhHHHHHhhhhhhlllllllhhhhhhhHHHHHLLll


DSSP  -----------------------hhhhhhhhHHHHH-----------------------h
Query -----------------------aaqavqrfVARID-----------------------r  288
ident 
Sbjct awteealgerflqaleflvgsleqaslpchfNPSVNllgnfreeeiddigyvlysglqvp  325
DSSP  lllhhhhhhhhhhhhhhhhhhhhllllleellEEEEElllllllhhhhhhhhhhhhllllh


DSSP  llll
Query atkr  292
ident 
Sbjct eslf  329
DSSP  hhll
```

back to top

```
DSSP  lllllllllhhhhhhhhhhhhhhllLLHHHHHHHHHHHHHHHHHHHHLLllllllLLEEEE
Query aapysspptnlrslrdrltqvaerqgVVFGRLQRHVAXIVVAQFAATLtddtgaPLLLVK   60
ident                                   |     |                 |
Sbjct ---ghmnvakrefirgmmahyraslpPPEHSVVIHELQKRVLDIGMLA-----vNKAHVE   52
DSSP  ---lllllhhhhhhhhhhhhhhhhllLLLHHHHHHHHHHHHHHHHHHHH-----lLLLEEE


DSSP  HHhhhhhhhlllllllLLEEEEEEL-------------lLHHHHHHHHHHHHHHHLlllEE
Query GGsslelrrgipdsrtsKDFATVAR-------------rDIELIHEQLADAGETGwegFT  107
ident                   |     |
Sbjct LF--gshvsgfctphsdADISLTYRnfspwlqgmervdeQNNKRMTRFGKEASAM---GM  107
DSSP  EE--lhhhhllllllllEEEEEEELLllllllllhhhhhHHHHHHHHHHHHHHHH---LL


DSSP  EEELLLEElllllllLLEEEEEEEEEEllеEEEEEEEEEEelllllllleeeeelhhhh
Query AIFTAPEEiaapgxpvKPRRFTAKLSYrgrAFATVPIEVSsveagnadqfdtltsdalgl  167
ident
Sbjct EDVRYIRA--------RIPVVQFTDGV---TGIHCDVSIG--------------------  136
DSSP  EEEEEELL--------LLLEEEEELLL---LLLEEEEEEL--------------------


DSSP  llllleeeeellhhhhhhhhhhhhhlllllllLLLLhhhhhhhhhhhllhhhhhhhh
Query vgvpaavavpcxtipwqiaqklhavtavleepkaNDRAhdlvdlqllegllldadlxptr  227
ident
Sbjct ------------------------niggvenskiLCAI----------------------  150
DSSP  ------------------------lhhhhhhhhhHHHH----------------------


DSSP  hhhhhhhhhhlllllllllllLHHHHHHHH-HLLLLLLL------------------l
Query saciaifearaqhpwpprvatlpHWPLIYAGA-LEGLDHLE------------------l  268
ident                               ||
Sbjct ----------------------rQVFPDFYGAyIHLVKAWGkareviaperstfnsftvt  188
DSSP  ----------------------hHLLHHHHHHhHHHHHHHHHhhlllllhhhllllhhhhh


DSSP  llLHHHHHH---------------------------------------------------
Query arTVDAAAQ---------------------------------------------------  277
ident
Sbjct tmALMVLQElgllpvfskptgefgeltvadaemllqefklppiydslhdddeklgeavff  248
DSSP  hhHHHHHHHhllllllllllllllllhhhhhhhhhhllllhhhhhhllllhhhhhhhhhh


DSSP  -------------------hHHHHHHHHHHHLLL---------------------------
Query -------------------aVQRFVARIDRATK---------------------------  291
ident
Sbjct clqrfaeyyakydfsagtvsLIHPRRHRTVYERvvrrhlellgsrkrlewekhiaehked  308
DSSP  hhhhhhhhhhhlllllllllLLLLLLLHHHHHHhhhhhhhhhhhhhhhhhhhhhhhlhhh


DSSP  ------------------------------------------------------------
Query ------------------------------------------------------------  291
ident
Sbjct gpldendfsasmqnettqrpsnspyvvedfvnyvncgrrvqasrvrhiqqefnrlremli  368
DSSP  llllhhhhhhhhhhhhhllllllllllllllllllllllllhhhhhhhhhhhhhhhhhhh


DSSP  ------------------l
Query ------------------r  292
ident
Sbjct dkeselkfdevfresdtvp  387
DSSP  llhhhllhhhhllllllll
```

No 605: Query=mol1A Sbjct=4x4pC Z-score=4.1

back to top

```
DSSP  lllllllllhhhhhhhhhhhhhhlLLLHHHHHHHHHHHHHHHHHHHHHLLLlllllllEEEE
Query aapysspptnlrslrdrltqvaerqGVVFGRLQRHVAXIVVAQFAATLTddtgaplLLVK   60
ident                                     |          |
Sbjct ------------veeilekalelviPDEEEVRKGREAEEELRRLDELG-------VEYV   41
DSSP  ------------lhhhhhhhhhhhlLLHHHHHHHHHHHHHHHHHHHHLLLL-------LLEE
```

```
Query GGSslelrrgipdsrtsKDFATVAR------RDIELIHEQLADAGetgwegFTAIFTAPE  114
ident                   |               |   |
Sbjct FV-gsyarntwlkgsleIDVFLLFPeefskeELRERGLEIGKAVL--------DSYEIRY   92
DSSP  EE-hhhhllllllllllEEEEEEELllllhhHHHHHHHHHHHLLL--------LEEEEEL


DSSP  ELlllllllllEEEEEEEEelleeEEEEEEEEEEELLLLllllleeeeelhhhhllllle
Query EIaapgxpvkpRRFTAKLSyrgraFATVPIEVSSVEAGnadqfdtltsdalglvgvpaav  174
ident                            |
Sbjct AH---------PYVHGVVK-----GVEVDVVPCYKLKE----------------------  116
DSSP  LL---------LEEEEEEL-----LEEEEEEEEELLLL----------------------


DSSP  eeeellhhhhhhHHHHHHhllllllllLLLHhhhhhhhhhhhllhhhhhhhhhhhhhh
Query avpcxtipwqiaQKLHAVtavleepkanDRAHdlvdlqlleglllddadlxptrsaciaif  234
ident
Sbjct pkniksavdrtpFHHKWL----------EGRI----------------------------  138
DSSP  llllllllhhhHHHHHH----------HHHL----------------------------


DSSP  hhhlllllllllllllhhhHHHHHHLLL-LLLL--------------------lllLHH
Query earaqhpwpprvatlphwplIYAGALEGL-DHLE--------------------larTVD  273
ident                             |                             |
Sbjct --------------------KGKENEVRLlKGFLkangiygaeykvrgfsgylcellIVF  178
DSSP  --------------------LLLHHHHHHhHHHHhhlllllllllllllhhhhhhhHHH


DSSP  HH----------------hhHHHH--------------hhHHHH----------------
Query AA----------------aqAVQR--------------fvARID----------------  287
ident                      |
Sbjct YGsfletvknarrwtrrtviDVAKgevrkgeeffvvdpvdEKRNvaanlsldnlarfvhl  238
DSSP  HLlhhhhhhhhlllllleeeELLLleeeellllleeellllLLLLllllllhhhhhhhhh


DSSP  ------------------------------------------------------------
Query ------------------------------------------------------------  287
ident
Sbjct crefmeapslgffkpkhpleieperlrkiveergtavfavkfrkpdivddnlypqleras  298
DSSP  hhhhhhlllhhhhllllllllhhhhhhhhhhlleeeeeeeelllllhhhhhhhhhhh


DSSP  ------------------------------------------------------------
Query ------------------------------------------------------------  287
ident
Sbjct rkifeflerenfmplrsafkaseefcyllfecqikeisrvfrrmgpqfedernvkkflsr  358
DSSP  hhhhhhhhhlllleeeeeeeellleeeeeeeellllllleeeeeeeeelllhhhhhhhlll


DSSP  ------------------------------------------------------------
Query ------------------------------------------------------------  287
ident
Sbjct nrafrpfiengrwwafemrkfttpeegvrsyasthwhtlgknvgesireyfeiisgeklf  418
DSSP  llllllleeelleeeeeeelllllhhhhhhhhhhlhhhhlhhhhhhhhhlleeeehhhll


DSSP  -----------hllll
Query -----------ratkr  292
ident
Sbjct kepvtaelcemmgvkd  434
DSSP  llllhhhhhhhhllll
```

No 606: Query=mol1A Sbjct=3upsA Z-score=4.1

[back to top]

```
DSSP  llllllllllhhhhhhhhhhhhhhllllhhhhHHHHHHHHHHHHHHHLLlllllllLEEEE
Query aapysspptnlrslrdrltqvaerqgvvfgrlQRHVAXIVVAQFAATLtddtgapLLLVK   60
ident                                         |                |
Sbjct -------------------------------fDPEXLLKLVTDSLDDD-------QALEI   22
DSSP  -------------------------------lLHHHHHHHHHHHHHHL-------LLEEE


DSSP  HHHhhhhhhlllLLLL---lLLEEEEELL--LHHHHHHHHHHHHHHHLlllEEEEEELLLEE
Query GGSslelrrgipDSRT---sKDFATVARR--DIELIHEQLADAGETGwegFTAIFTAPEE  115
ident                            |           |||
```

```
DSSP  lllllllLLEEEEEEEeeelleeeeEEEEEEEELLlllllleeeeelhhhhllllllee
Query iaapgxpvKPRRFTAKlsyrgrafaTVPIEVSSVEagnadqfdtltsdalglvgvpaava  175
ident               |             |     |
Sbjct -----paaDWVLLDAG---------DIIIHLFRPE-------------------------   93
DSSP  -----lllLEEEEELL---------LEEEEEELHH-------------------------


DSSP  eeellhhhhhhhhhhhhhlllllllllllhhhhhhhhhhhllhhhhhhhhhhhhhhhh
Query vpcxtipwqiaqklhavtavleepkandrahdlvdlqlleglllldadlxptrsaciaife  235
ident
Sbjct -------------------------------------------------------vrsfy   98
DSSP  -------------------------------------------------------hhhhh


DSSP  HHLLLLllllllllllhhhhhhhhhllllllllllhhhhhhhhhhhhhhhhhllll
Query ARAQHPwpprvatlphwpliyagalegldhlelartvdaaaqavqrfvaridratkr  292
ident
Sbjct NLERXW-----------------------------------------------gfgd  108
DSSP  HHHHHL-----------------------------------------------llll
```

No 607: Query=mol1A Sbjct=5hzdA Z-score=4.1

back to top

```
DSSP  --------------------------------------------------llllllllll
Query --------------------------------------------------aapysspptn   10
ident
Sbjct vrlyscdacphavftthaallahaeehhadllpdharlrriaqklnpvwnralnarrnti   60
DSSP  llleellllllleellhhhhhhhhhhhhhhhhlllhhhhhhhhhhhhhhhhhhhhhlhhhh


DSSP  hhhhhhhhhhhhhhlLLLHHHHHHHHHHHHHHHHHHHLLLlllllLLEEEEhhHHHHhhhl
Query lrslrdrltqvaerqGVVFGRLQRHVAXIVVAQFAATLTddtgaPLLLVKggSSLElrrg   70
ident                           | |                        |
Sbjct tswgkkifhvaaqrdAGESKMQEAHRARAQLECVVRRWH-----DKARVF--IFGS----  109
DSSP  hhhhhhhhhhhhhhlLLHHHHHHHHHHHHHHHHHHHHHHL-----LLLEEE--EELH----


DSSP  llLLLL-----lLLEEEEE---------LLLHHHHHHHHHHHHHHHLLlleeeEELLLEEL
Query ipDSRT-----sKDFATVA---------RRDIELIHEQLADAGETGWegftaIFTAPEEI  116
ident                  ||| |
Sbjct svAMGVwdgtadIDFAVVDvdamergswPPLEKNAVRSITELLRRVG-fsfvNLEPISHA  168
DSSP  hhHLLLllllllEEEEEELhhhhhllllLLLHHHHHHHHHHHHHHHLL-llllLEEEELLL


DSSP  lllllllLLEEEEEEEE--------------------------------------------
Query aapgxpvKPRRFTAKL--------------------------------------------  132
ident
Sbjct -------RVPIIKHHAsspdvvarsirfilngpatredrlllegsvrdavgptgvqqvww  221
DSSP  -------LLLEEEEELLlllllhhheeeeeelllllhhhhhhhhhhhhhhhllllleeeeee


DSSP  -------------------------------------eeLLEE----EEEEEEEEEell
Query -------------------------------------syRGRA----FATVPIEVSsve  150
ident
Sbjct nrtsdmmsatlesttaavraamcspalasaslrtkvqpahDECRpelyNIDFDLSFR---  278
DSSP  llllleeeeeellhhhhhhhhllllllllllleeeeellLLLLlhhhHLLEEEELL---


DSSP  lllllllleeeeelhhhhllllllleeeeellhhhhhhhhhhhhlllllllllllhhhhH
Query agnadqfdtltsdalglvgvpaavavpcxtipwqiaqklhavtavleepkandrahdlvD  210
ident
Sbjct ----------------------------------------------------afgirnsT  286
DSSP  ----------------------------------------------------lhhhhhhH


DSSP  HHHhhhhllhhhhhhhhhhhhhhhhhhlllllllllllllHHHHHHHHHLLLL------
Query LQLlegllldadlxptrsaciaifearaqhpwpprvatlphWPLIYAGALEGLD------  264
ident |                                                    |
Sbjct LLR----------------------------------kyllSHPCARPGAIVLKdwskts  312
DSSP  HHH----------------------------------hhhlLLLLHHHHHHHHHhhhhhh
```

```
ident                         |
Sbjct gvnnsvngyftsyaINIMWIYYLVQkgyvpyvdpleipeslvnytdfdprytpmidpeit  372
DSSP  llllhhhllllhhhHHHHHHHHHHHllllllllhhhllllllllllllllllllllllll


DSSP  ----------------------------------hhHHHHHhhhHLLLL-----------
Query ----------------------------------avQRFVAridRATKR-----------  292
ident
Sbjct ntereelykaagdmlvgffyfysfefdwghnvislnRPGITtkrMLGWHvedvrhptrye  432
DSSP  hhhhhhhhhhhhhhhhhhhhhhhhhllllleelllllLLLLEehhHHLLLllllllllll


DSSP  ----------------------------------------------
Query ----------------------------------------------  292
ident
Sbjct lciedpyeenlnlgrhigvtkslrvrtelyrgllsllkecvfaa  476
DSSP  llllllllllllllllhhhhhhhhhhhhhhhhhlllllllll
```

No 608: Query=mol1A Sbjct=4r10A Z-score=4.1

back to top

```
DSSP  -----------------------------------------------LLLL---llLLLLH
Query -----------------------------------------------AAPY---ssPPTNL   11
ident                                                           |
Sbjct glslpdlvklmcdhdesvvaravhraymlsredpnffnapgfdhrsFVEAlmaasKSSNV   60
DSSP  lllhhhhhhhhllllhhhhhhhhhhhhhhhhhlhhhllllllllhhhHHHHhhhhlLLLLH


DSSP  HHHHHHHHHHHHHHLL---------llhHHHHHHHHHHH--------------------hh
Query RSLRDRLTQVAERQG---------vvfGRLQRHVAXIV--------------------va   42
ident    |                       |
Sbjct NVRRNAIGALSHMSEqrggpllifrsgGLAEIIRMLYDslesvvhyavttlrnllmhvsd  120
DSSP  HHHHHHHHHHHHHHLLlllhhhhhhhllLHHHHHHHHHLllhhhhhhhhhhhhhhhhllh


DSSP  hhhhlllllllllleEEEH--------HHHHHHHHLllllllllleeeeelllhhhhhhh
Query qfaatltddtgapllLVKG--------GSSLELRRGipdsrtskdfatvarrdieliheq   94
ident                                 |
Sbjct sraqaralnavealtPHLHktnpkllaQVADGLYFL------------------------  156
DSSP  hhhhhhhllhhhhhhHHHHlllhhhhhHHHHHHHHH------------------------


DSSP  hhhhhhhllllleeeeellleelllllllllleeeeeeeeelleeeeeeeeeeeelllll
Query ladagetgwegftaiftapeeiaapgxpvkprrftaklsyrgrafatvpievssveagna  154
ident
Sbjct ------------------------------------------------------------  156
DSSP  ------------------------------------------------------------


DSSP  llleeeeelhhhhllllllleeeeeLLHHHHHHHHHHHHHHllllllllLLLHHHHHHHHH
Query dqfdtltsdalglvgvpaavavpcXTIPWQIAQKLHAVTavleepkanDRAHDLVDLQLL  214
ident                            |      |             |
Sbjct -----------liddapskitflsLLGPQILVSILREYS---------DHRKLIYTVVRC  196
DSSP  -----------hlllhhhhhhhhhLLHHHHHHHHHHHHHLL---------LLHHHHHHHHHH


DSSP  HHHL--LHHHHHHHHHHHHhhhhhhhllllllLLLLLlllhhhhhhhhhllllllllllLH
Query EGLL--LDADLXPTRSACiaifearaqhpwpPRVATlphwpliyagalegldhlelarTV  272
ident    |           |                   |
Sbjct IRSLsvCPSNKPALISLG-------clpalyVELCT---------------------aKD  228
DSSP  HHHHllLLLHHHHHHHHLL-------hhhhhhHHHLL---------------------lLL


DSSP  HHHHHHHHHHHHHHHHHHLLLL----------------------------------------
Query DAAAQAVQRFVARIDRATKR----------------------------------------  292
ident      |
Sbjct ERSQTAILVAMRNLSDSATNeenltqliiklleiirvandgmtacacgtlsnltcnntrn  288
DSSP  HHHHHHHHHHHHHHHHHHHLLLllllhhhhhhhhhhhhhllhhhhhhhhhhhhhhllllhhh


DSSP  ------------------------------------------------------------
Query ------------------------------------------------------------  292
ident
Sbjct kqtvcshggidalvtairrlpeveevtepalcalrhctarhslaeeaqselrfcqafpvi  348
```

```
DSSP  ------------------------------------------------------------
Query ------------------------------------------------------------  292
ident 
Sbjct ldqletlrtpvikaalgvirnsallqtnlieltqeqtanghtavsltmdilrraitaiee  408
DSSP  hhhhllllhhhhhhhhhhhhhhhhllhhhhhhhhllllllllhhhhhhhhhhhhhhhhhh


DSSP  ------------------------------------------------------------
Query ------------------------------------------------------------  292
ident 
Sbjct npdiavdgvpmwgviegavsalhqlanhpavaaaccddigqvgnpecppfldllhrllah  468
DSSP  lllleelleehhhhhhhhhhhhhhlllhhhhhhhhhhlllllllllllhhhhhhhhhhhl


DSSP  ------------------------------------------------------------
Query ------------------------------------------------------------  292
ident 
Sbjct prlgsmddevlereilgllyqlskrpdgaravestgvsallmesrgsqyksvvtyangvl  528
DSSP  hhhhlllllhhhhhhhhhhhhhhlllhhhhhhhllllhhhhhhhhllllhhhhhhhhhhh


DSSP  ---------
Query ---------  292
ident 
Sbjct snlkrgdsa  537
DSSP  hhhhhhhll
```

No 609: Query=mol1A Sbjct=4lt6A Z-score=4.0

back to top

```
DSSP  ----------llllllllllhhhhhhhhhhhhhhlLLLHHHHHHHHHHHHHHhhhhhLLLL
Query ----------aapysspptnlrslrdrltqvaerqGVVFGRLQRHVAXIVVaqfaaTLTD   50
ident                                            | |
Sbjct khygitspislaspkeidhiytqklidamkpfgvfEDEEELNHRLVVLGKLnnlvkEWIS   60
DSSP  llllllllllllllhhhhhhhhhhhhhhhhhlllLLHHHHHHHHHHHHHHHhhhhhHHHH


DSSP  ------------llLLLLeEEEHHhhhhhhhlllllLLLL----------LEEEEEL--L
Query ------------dtGAPLlLVKGGsslelrrgipdsRTSK----------DFATVAR--R   86
ident                                                   |   ||
Sbjct dvsesknlppsvvaTVGG-KIFTF------------GSYRlgvhtkgadiDALCVAPrhV  107
DSSP  hhlllllllhhhhlLLLL-EEEEE------------HHHHhllllllllleEEEEEELllL


DSSP  LHHHHHHHHHHHHHHLLllEEEEEELLLEELLllllllLEEEEEEEEelleeEEEEEEEE
Query DIELIHEQLADAGETGWegFTAIFTAPEEIAapgxpvkPRRFTAKLSyrgraFATVPIEV  146
ident                          | |
Sbjct ERSDFFQSFFEKLKHQD--GIRNLRAVEDAF-------VPVIKFEFD-----GIEIDLVF  153
DSSP  LHHHHHHHHHHHHHHLL--LEEEEEEELLLL-------LLEEEEEEL-----LEEEEEEE


DSSP  EELLLllLLLLEeeeelhhhhllllleeeeellhhHHHHhhhhhhhlllllllllllllh
Query SSVEAgnADQFDtltsdalglvgvpaavavpcxtipWQIAqklhavtavleepkandrah  206
ident 
Sbjct ARLAI-qTISDN-----------------------lDLRD--dsrlrsldircirslngc  187
DSSP  EELLL-lLLLLL-----------------------lLLLL--hhhhllllhhhhhhhhhh


DSSP  hhhHHHHhhhllhhhhhhhhhhhhhhhhhhhllllllllllllllhhHHHHHHHL-lLLL
Query dlvDLQLLeglllldadlxptrsaciaifearaqhpwpprvatlphwpLIYAGALE-gLDH  265
ident    |  |                                              |
Sbjct rvtDEILH----------------------------------lvpnkETFRLTLRavKLW  213
DSSP  hhhHHHHH----------------------------------llllhHHHHHHHHhhHHH


DSSP  L-----------------llLLLHHHHHH-----------------------hhhHHHHH
Query L-----------------elARTVDAAAQ-----------------------avqRFVAR  285
ident 
Sbjct AkrrgiysnmlgflggvswaMLVARTCQLypnaaastlvhkfflvfskwewpnpvLLKQP  273
DSSP  HhhhlllllllllllhhhhhHHHHHHHHHlllllhhhhhhhhhhhhhhlllllllLLLLL


DSSP  hhHLLLL-----------------------------------------------------
```

```
[illegible]
DSSP  --LLLLLlllllllllllhhhllllllllllllllllllhhhhhhhhhhhhhhhhh


DSSP  ------------------------------------------------------------
Query ------------------------------------------------------------  292
ident
Sbjct deilqgksdwsklleppnffqkyrhyivltasasteenhlewvglveskirvlvgnlern  391
DSSP  hhhllllllhhhhhllllllllllleeeeeeeeellhhhhhhhhhhhhllhhhhhhhhhhl


DSSP  ------------------------------------------------------------
Query ------------------------------------------------------------  292
ident
Sbjct efitlahvnpqsfpgnyvsmwflgiifrdltydiqsftdtvyrqanninmlkegmkieat  451
DSSP  lleeeeeeeeeeellleeeeeeeeeeeellllhhhhhhhhhhhhhhhhhllllllllleeeee


DSSP  ----------------
Query ----------------  292
ident
Sbjct hvkkkqlhhylpaeil  467
DSSP  eeellllhhhlllllll
```

No 610: Query=mol1A Sbjct=4r0zA Z-score=4.0

back to top

```
DSSP  lllllllllhhhhhhhhhhhhhhllllhhHHHHHHHHHHHHHH--HHLLllllllllee
Query aapyssppptnlrslrdrltqvaerqgvvfgRLQRHVAXIVVAQF--AATLtddtgaplll   58
ident                                 |
Sbjct ------------------------------TQQLKQSVMDLLTYegSNDM----------   20
DSSP  ------------------------------LHHHHHHHHHHHHHhlLLLL----------


DSSP  eehhhhhhhhhlllllllllleeeeelllhhhhhhhhhhhhHHLLLLEEeeellleelll
Query vkggsslelrrgipdsrtskdfatvarrdieliheqladagETGWEGFTaiftapeeiaa  118
ident
Sbjct -------------------------------------sglsLPDLVKLM-----------   32
DSSP  -------------------------------------llllHHHHHHHH-----------


DSSP  lllllleeeeeeeeelleeeeEEEE-------------------------------eee
Query pgxpvkprrftaklsyrgrafaTVPI-------------------------------evs  147
ident                        |
Sbjct ----------------cdhdesVVARavhraymlsredpnffnapgfdhrsfvealmaas   76
DSSP  ----------------hhllhhHHHHhhhhhhhhhhhhlhhhhhllllllhhhhhhhhhhl


DSSP  elllllllleeeeelhhhhllllllleeeeELLHhHHHHHHHHHhhlllllllllLLLHH
Query sveagnadqfdtltsdalglvgvpaavavpCXTIpWQIAQKLHAvtavleepkanDRAHD  207
ident                                      |   |
Sbjct kssnvnvrrnaigalshmseqrggpllifrSGGL-AEIIRMLYD-----------SLESV  124
DSSP  llllhhhhhhhhhhhhhlllllhhhhhhhHLLH-HHHHHHHHL-----------LLHHH


DSSP  HHHHHHHHHHL---LHHHHHHHHHHHhhhhhhlllllLLLLLlllhhhhhhhhhllll
Query LVDLQLLEGLL---LDADLXPTRSACiaifearaqhpwpPRVATlphwpliyagalegld  264
ident           |           |
Sbjct VHYAVTTLRNLlmhVSDSRAQARALN--------avealTPHLH----------------  160
DSSP  HHHHHHHHHHHhhhLLLHHHHHHHHLL--------hhhhhHHHHH----------------


DSSP  llllllLHHHHHHHHHHHHHHHHHH------------------------------------
Query hlelarTVDAAAQAVQRFVARIDR------------------------------------  288
ident       |       |
Sbjct -----kTNPKLLAQVADGLYFLLIddapskitflsllgpqilvsilreysdhrkliytvv  215
DSSP  -----lLLHHHHHHHHHHHHHHHHLllhhhhhhhhhllhhhhhhhhhhhllllhhhhhhhh


DSSP  ------------------------------------------------------------
Query ------------------------------------------------------------  288
ident
Sbjct rcirslsvcpsnkpalislgclpalyvelctakdersqtailvamrnlsdsatneenltq  275
DSSP  hhhhhhlllllhhhhhhhllhhhhhhhhhhhlllhhhhhhhhhhhhhhhhhhlllllllhh
```

```
DSSP  ------------------------------------------------------------
Query ------------------------------------------------------------  288
ident
Sbjct liiklleiirvandgmtacacgtlsnltcnntrnkqtvcshggidalvtairrlpeveev  335
DSSP  hhhhhhhhlllllhhhhhhhhhhhhhhhlllhhhhhhhhhllhhhhhhhhhhhlllllhhh


DSSP  ------------------------------------------------------------
Query ------------------------------------------------------------  288
ident
Sbjct tepalcalrhctarhslaeeaqselrfcqafpvildqletlrtpvikaalgvirnsallq  395
DSSP  hhhhhhhhhhhlllllhhhhhhhhhhlllhhhhhhhhhllllhhhhhhhhhhhhhhhllll


DSSP  ------------------------------------------------------------
Query ------------------------------------------------------------  288
ident
Sbjct tnlieltqeqtanghtavsltmdilrraitaieenpdiavdgvpmwgviegavsalhqla  455
DSSP  lhhhhhhhlllllllhhhhhhhhhhhhhhhhhhhlllleelleehhhhhhhhhhhhhhhl


DSSP  ------------------------------------------------------------
Query ------------------------------------------------------------  288
ident
Sbjct nhpavaaaccddigqvgnpecppfldllhrllahprlgsmddevlereilgllyqlskrp  515
DSSP  llhhhhhhhhhllllllllllllhhhhhhhhhllhhhhlllllhhhhhhhhhhhhhlllh


DSSP  --------------------------------------llll
Query --------------------------------------atkr  292
ident
Sbjct dgaravestgvsallmesrgsqyksvvtyangvlsnlkrgds  557
DSSP  hhhhhhhllllhhhhhhhllllllhhhhhhhhhhhhhhhhhhl
```

No 611: Query=mol1A Sbjct=4txzA Z-score=4.0

back to top

```
DSSP  lllllllllhhhhhhhhhhhhhhhlLLLHHHHHHHHHHHHHHHHHHLL---LLLL-----
Query aapysspptnlrslrdrltqvaerqGVVFGRLQRHVAXIVVAQFAATL---TDDT-----   52
ident
Sbjct -----twnfhqyytnrndglmgklvLTDEEKNNLKALRKIIRLRTRDVfeeAKGIakavk   55
DSSP  -----leelhhhhhlllllhhhhhlLLHHHHHHHHHHHHHHHHHHHHHhhhHHHHhhhhh


DSSP  --------------------------------------------LLLLEEEEHHhhhhhh
Query --------------------------------------------GAPLLLVKGGsslelr   68
ident
Sbjct ksaltfeiiqekvsttqikhlsdseqrevakliyemdddardefLGLTPRFWTQ------  109
DSSP  hllllhhhhhhhhlllhhhhllhhhhhhhhhhhhlllhhhhhhhLLLLLEEEEE------


DSSP  hlllLLLL----------lLLEEEEEL----lLHHHHHHHHHHHHHH-LLLLeeeEELLL
Query rgipDSRT----------sKDFATVAR----rDIELIHEQLADAGET-GWEGftaIFTAP  113
ident                     |  |           |              |     | |
Sbjct gsfqYDTLnrpfqpgqemdIDDGTYMPmpikiGHSLLILLVDASLKSlVAENhgwKFEAK  169
DSSP  lhhhHLLLllllllllleeEEEEEEEEellllLHHHHHHHHHHHHHHhHLLLlleEEEEL


DSSP  EellllllllllEEEEEEEeellEEEEEEEEEEEEL-LLLLlllleeeeelhhhhlllll
Query EeiaapgxpvkpRRFTAKLsyrgRAFATVPIEVSSV-EAGNadqfdtltsdalglvgvpa  172
ident                  |
Sbjct Q-----------TCGRIKI---eAEKTHIDVPMYAIpKDEF-------------------  196
DSSP  L-----------LEEEEEE---hHHLEEEEEEEEEEeLLLL-------------------


DSSP  leeeeelLHHH----------------hhhHHHHHHHllllllllLLLLhhhhhhhhhhhh
Query avavpcxTIPW----------------qiaQKLHAVTavleepkaNDRAhdlvdlqllegl  217
ident                               |             ||
Sbjct -------ELDSenvnlalregdrkwinsdPKIVEDW-------fNDSC------------  230
DSSP  -------LLLHhhleeellllllleeellHHHHHHH-------hHHHH------------


DSSP  llhhhhhhhhhhhhhhhhhhhllllllllllllLHHHHHHHHHL-lLLLL----------
Query lldadlxptrsaciaifearaqhpwpprvatlpHWPLIYAGALE-gLDHL----------  266
```

```
DSSP  --------------------------------[illegible]


DSSP  ---llllLHHH-----------------------------hhhhhhhhhHHHHH------
Query ---elarTVDA-----------------------------aaqavqrfvARIDR------  288
ident        ||
Sbjct sislmaaTVNIldsvahdasdlgetmkiiakhlpsefargvespdstdeKPLFPpsykhg  318
DSSP  hhhhhhhHHHHhhhlllllllhhhhhhhhhhhhhhhhhlllllllllllLLLLLlhhhll


DSSP  ------------------------------------------------llll
Query ------------------------------------------------atkr  292
ident
Sbjct premdimsklerlpeilssaesadsksealkkinmafgnrvtnselivlaka  370
DSSP  hhhhhhhhhhhhhhhhhhhhhhllllhhhhhhhhhhhhlllllhhhleelll
```

No 612: Query=mol1A Sbjct=5a2zA Z-score=3.9

back to top

```
DSSP  ---------------------------LLLLL------------llLLLHHHHhhHHHH-
Query ---------------------------AAPYS------------spPTNLRSLrdRLTQ-   20
ident                                                 |       |
Sbjct ktftevqterleqadrsvlikcpsklnEKKLLqylsshgkidnyffFENRGIH--ALIEf   58
DSSP  llhhhhhhhhhhhhleeeeeellllllHHHHHhhhhhhhlleeeeeEELLLEE--EEEEe


DSSP  ------------------------------------------------------------
Query ------------------------------------------------------------   20
ident
Sbjct sekssvaslqavtgipkaaehhvvpyksrlftftlknpgsqaaeerpvkispqshipvne  118
DSSP  lllhhhhhhhhhllllhhhlllllllllleeeeeelllllllllllllllllllllllhhh


DSSP  ------------------hhhhllLLHHHHHHHHHHHHHHHHHHLLLlllllLLEEEEHH
Query ------------------vaerqgVVFGRLQRHVAXIVVAQFAATLTddtgaPLLLVKGG   62
ident                                |  |   |  ||         |   ||
Sbjct lipklchadsissqmyillneyqlTEENIKLRYLACSLVRDFARAYF-----PDSTVKPF  173
DSSP  hhhhhhllllhhhhhhhhhhhhllLHHHHHHHHHHHHHHHHHHHLLL-----LLLEEEEE


DSSP  hhhhhhhllllllllLLEEEEEL-----------------lLHHHHHHHHHHHHHHLLll
Query sslelrrgipdsrtsKDFATVAR-----------------rDIELIHEQLADAGETGWeg  105
ident                 |                            |     |
Sbjct --gssvntfgklgcdVDMFLDFHkgpfemeyqmkrlpserlATQKILSIIGDCLDNFG--  229
DSSP  --hhhllllllllllEEEEEELLllleeeeellllllhhhHHHHHHHHHHHHHHHHLL--


DSSP  EEEE-ELLLEELlllllllLLEEEEEEEEEElleeEEEEEEEEEellllllllleeeeelh
Query FTAI-FTAPEEIaapgxpvKPRRFTAKLSYrgraFATVPIEVSsveagnadqfdtltsda  164
ident                                          ||
Sbjct PGYSsVQKILNA-------RCPLVKFSHQP---tGFQCDLSVS-----------------  262
DSSP  LLEEeEEEELLL-------LLLEEEEEELL---lLEEEEEEEL-----------------


DSSP  hhhlllllleeeeellhhhhhHHHHHHHHllllllllLLLLhhhhhhhhhhhhllhhhhh
Query lglvgvpaavavpcxtipwqiAQKLHAVTavleepkaNDRAhdlvdlqllegllldadlx  224
ident
Sbjct -------------------nsIAIRCSEL--------LYIY-------------------  276
DSSP  -------------------lhHHHHHHHH--------HHHH-------------------


DSSP  hhhhhhhhhhhhhlllllllllllllLHHHhHHHHHL-lLLLL-----------------
Query ptrsaciaifearaqhpwpprvatlpHWPLiYAGALE-gLDHL-----------------  266
ident
Sbjct -------------------------gCLDPrVRALVFslRCWArvhgltnsvpgtwitnf  311
DSSP  -------------------------hHHLHhHHHHHHhhHHHHhhlllllllllllllhh


DSSP  -llllLHHHHHHH-----------------------------------------------
Query -elarTVDAAAQA-----------------------------------------------  278
ident
Sbjct sltmmIMFFLQKRsppiiptldqlkeladekdkhviggydcsfvsdlskikptkntetld  371
DSSP  hhhhhHHHHHHLLlllllllhhhhhhhllhhhlleelleellllllllllllllllllhh
```

```
Query                    vqrfvaridRATKR                             292
ident
Sbjct ellcdffqyfgnfdfrknslnlrkgkevNKPESsplyiwnpfeqdlnisknvnqpqlekf  431
DSSP  hhhhhhhhhhlllllleeeelllllleeeELLLLllllllllllllllllllllhhhhhhh


DSSP  -----------------------------------
Query -----------------------------------  292
ident
Sbjct vamaresawilqkedktqqminkepwglaavlipf  466
DSSP  hhhhhhhhhhhhlllhhhhhhllllllhhhhllll
```

No 613: Query=mol1A Sbjct=4r11E Z-score=3.9

back to top

```
DSSP  -----------------------------LLLL---llLLLLHHHHHHHHHHHHHHLL--
Query -----------------------------AAPY---ssPPTNLRSLRDRLTQVAERQG--   26
ident                                          |    |
Sbjct dlvklmcdhdesvvaravhraymlsrhrsFVEAlmaasKSSNVNVRRNAIGALSHMSEqr   60
DSSP  lhhhhhllllhhhhhhhhhhhhhhhllhhHHHHhhhhhHLLLHHHHHHHHHHHHHHLLll


DSSP  -------llhHHHHHHHHHHH--------------------hhhhhhlllllllllleEE
Query -------vvfGRLQRHVAXIV--------------------vaqfaatltddtgapllLV   59
ident           |
Sbjct ggpllifrsgGLAEIIRMLYDslesvvhyavttlrnllmhvsdsraqaralnavealtPH  120
DSSP  lhhhhhhhhlLHHHHHHLLLLllhhhhhhhhhhhhhhhhhlhhhhhhhhhhllhhhhhHH


DSSP  EH--------HHHHHHHHLllllllllleeeeelllhhhhhhhhhhhhhhlllleeeeel
Query KG--------GSSLELRRGipdsrtskdfatvarrdieliheqladagetgwegftaift  111
ident                |
Sbjct LHktnpkllaQVADGLYFL-----------------------------------------  139
DSSP  HHlllhhhhhHHHHHHHHH-----------------------------------------


DSSP  lleelllllllllleeeeeeeeelleeeeeeeeeeelllllllllleeeeelhhhhllll
Query apeeiaapgxpvkprrftaklsyrgrafatvpievssveagnadqfdtltsdalglvgvp  171
ident
Sbjct ------------------------------------------------------liddap  145
DSSP  ------------------------------------------------------hlllhh


DSSP  lleeeeeLLHHHHHHHHHHHHhlllllllllLLLHHHHHHHHHHHHL--LHHHHHHHHHH
Query aavavpcXTIPWQIAQKLHAVtavleepkanDRAHDLVDLQLLEGLL--LDADLXPTRSA  229
ident           |      |             |              |           |
Sbjct skitflsLLGPQILVSILREY---------sDHRKLIYTVVRCIRSLsvCPSNKPALISL  196
DSSP  hhhhhhhLLHHHHHHHHHHHL---------lLLHHHHHHHHHHHHHHllLLLHHHHHHHL


DSSP  HhhhhhhhllllLLLL--LLLLllhhhhhhhhhllllllllllLHHHHHHHHHHHHHHHH
Query CiaifearaqhpWPPR--VATLphwpliyagalegldhlelarTVDAAAQAVQRFVARID  287
ident                     |                             |
Sbjct G---------clPALYveLCTA---------------------KDERSQTAILVAMRNLS  226
DSSP  L---------hhHHHHhhHHHL---------------------LLHHHHHHHHHHHHHHH


DSSP  HLLLL-------------------------------------------------------
Query RATKR-------------------------------------------------------  292
ident
Sbjct DSATNeenltqliiklleiirvandgmtacacgtlsnltcnntrnkqtvcshggidalvt  286
DSSP  HHLLLllllhhhhhhhhhhhhhhllhhhhhhhhhhhhhllllhhhhhhhhhhllhhhhhh


DSSP  ------------------------------------------------------------
Query ------------------------------------------------------------  292
ident
Sbjct airrlpeveevtepalcalrhctarhslaeeaqselrfcqafpvildqletlrtpvikaa  346
DSSP  hhhhllllhhhhhhhhhhhhhhllllllhhhhhhhhhhlllhhhhhhhhhhlllhhhhhh


DSSP  ------------------------------------------------------------
Query ------------------------------------------------------------  292
ident
```

```
DSSP  ------------------------------------------------------------
Query ------------------------------------------------------------  292
ident 
Sbjct egavsalhqlanhpavaaaccddcppfldllhrllahprlgsmddevlereilgllyqls  466
DSSP  hhhhhhhhhhlllhhhhhhhhlllllhhhhhhhhhllhhhhlllllhhhhhhhhhhhhh
```

```
DSSP  ------------------------------------------
Query ------------------------------------------  292
ident 
Sbjct krpdgaravestgvsallmesrgsqyksvvtyangvlsnlkr  508
DSSP  hlhhhhhhhhhllllhhhhhhhlllllhhhhhhhhhhhhhhl
```

No 614: Query=mol1A Sbjct=4k9bA Z-score=3.8

back to top

```
DSSP  lllllllllhhhhhhhhhhhhhhlLLLHHHHHHHHHHHHHHHHHHHLLLLLLLL-LLEEE
Query aapyssppptnlrslrdrltqvaerqGVVFGRLQRHVAXIVVAQFAATLTDDTGA-PLLLV   59
ident                                        ||
Sbjct --------------klkkvldklrlKRKDISEAAETVNKVVERLLRRMQKRESEfKGVEQ   46
DSSP  --------------lhhhhhhhhllLHHHHHHHHHHHHHHHHHHHHHHHHHLLLLLLlLLLEE

DSSP  EHHhhhhhhhlllllllLLEEEEE-----------------------------------
Query KGGsslelrrgipdsrtsKDFATVA-----------------------------------   84
ident                    |
Sbjct LNT-gsyyehvkisapneFDVMFKLevprielqeyyetgafylvkfkrgnplshflegev  105
DSSP  EEE-llhhhllllllllleEEEEEEEellleeeeellllllleeeeeellllllllleelle

DSSP  --llLHHHHHHHH-HHHHHHLlLLEEEEEELLLEellllllllllEEEEEEEEElleeEEE
Query --rrDIELIHEQL-ADAGETGwEGFTAIFTAPEeiaapgxpvkpRRFTAKLSYrgraFAT  141
ident                                                |
Sbjct lsatKMLSKFRKIiKEEVKEI-KDIDVSVEKEK--------pgsPAVTLLIRN--peEIS  154
DSSP  elhhHHHHHHHHHhHHHHHLL-LLLEEEELLLL--------lllLEEEEEEEL--llEEE

DSSP  EEEEEEELLLLllllleeeeelhhhhllllleeeeellHHHH-----------------
Query VPIEVSSVEAGnadqfdtltsdalglvgvpaavavpcxtIPWQ-----------------  184
ident | |       |
Sbjct VDIILALESKG---swpistkeglpiqgwlgtkvrtnlrREPFylvpknakdgnsfqget  211
DSSP  EEEEEEEEELL---lllhhhlllllllllllhhhhhhhhLLLEeeellleeelleeelll

DSSP  ---hhHHHHHHHlllllllLLLLhhhhhhhhhhhhllhhhhhhhhhhhhhhhhhhhllll
Query ---iaQKLHAVTavleepkaNDRAhdlvdlqlleglllladlxptrsaciaifearaqhp  241
ident 
Sbjct wrlsfSHTEKYI--------LNNH-----------------------------------g  228
DSSP  eeeelHHHHHHH--------HHLL-----------------------------------l

DSSP  llllllllllhhhhHHHHHL-lLLLL-----------------llllLHHHHHHH-----
Query wpprvatlphwpliYAGALE-gLDHL-----------------elarTVDAAAQA-----  278
ident                   |      |                           |
Sbjct iektccessgakccRKECLKlmKYLLeqlkkefqeldafcsyhvktaIFHMWTQDpqdsq  288
DSSP  llllllllllllllHHHHHHhhHHHHhhhhhhhlhhhllllhhhhhhhHHHHHHHLllhhh

DSSP  -----------------------hhhhhhhHHHL--------------------------
Query -----------------------vqrfvarIDRA--------------------------  289
ident                               |
Sbjct wdprnlsscfdkllafflecl rtekldhyfIPKFnlfsqelidrkskeflskkieyernn  348
DSSP  hlhhhhhhhhhhhhhhhhhhhhhlllllllLLLLlllllllllhhhhhhhhhhhhhhhl

DSSP  ----lll
Query ----tkr  292
ident 
Sbjct gfpifdk  355
DSSP  llhhhhl
```

back to top

```
DSSP  lllllllllhhhhhhhhhhhhhlLLLHHHHHHHHHHHHHHHHHHLL---LLLL-----
Query aapyssspptnlrslrdrltqvaerqGVVFGRLQRHVAXIVVAQFAATL---TDDT-----   52
ident 
Sbjct -----twnfhqyytnrndglmgklvLTDEEKNNLKALRKIIRLRTRDVfeeAKGIakavk   55
DSSP  -----leelhhhhhlllllhhhhhlLLHHHHHHHHHHHHHHHHHHHHHhhhHHHHhhhhh


DSSP  ---------------------------------------------LLLLEEEEHHhhhhh
Query ---------------------------------------------GAPLLLVKGGsslelr   68
ident 
Sbjct ksaltfeiiqekvsttqikhlsdseqrevakliyemdddardefLGLTPRFWTQ------  109
DSSP  hllllhhhhhhhhlllhhhhllhhhhhhhhhhhlllhhhhhhhLLLLLEEEEE------


DSSP  hlllLLLL----------lLLEEEEEL--lLHHHHHHHHHHHHHH-LLLLeeeEELLLEe
Query rgipDSRT----------sKDFATVAR--rDIELIHEQLADAGET-GWEGftaIFTAPEe  115
ident                     |  |         |              |     | |
Sbjct gsfqYDTLnrpfqpgqemdIDDGTYMPmpiGHSLLILLVDASLKSlVAENhgwKFEAKQ-  168
DSSP  lhhhHLLLlllllllleeEEEEEEEEellLHHHHHHHHHHHHHHhHLLLlllEEEEEL-


DSSP  lllllllllEEEEEEEeellEEEEEEEEEEEELL------LLLLllleeeeelhhhhll
Query iaapgxpvkpRRFTAKLsyrgRAFATVPIEVSSVE------AGNAdqfdtltsdalglvg  169
ident                |
Sbjct ----------TCGRIKI---eAEKTHIDVPMYAIPkdefdsENVN---------------  200
DSSP  ----------LEEEEEE---hHHLEEEEELEEEEElllllhHHLE---------------


DSSP  llleeeeellhHHHHhHHHHHHhlllllllLLLLhhhhhhhhhhhllhhhhhhhhhh
Query vpaavavpcxtiPWQIaQKLHAVtavleepkaNDRAhdlvdlqlleglllldadlxptrsa  229
ident                                 ||
Sbjct lalregdrkwinSDPK-IVEDWF---------NDSC------------------------  226
DSSP  eellllllleeeLLHH-HHHHHH---------HHHH------------------------


DSSP  hhhhhhhhllllllllllllLHHHHHHHHHL-lLLLL------------llllLHHH--
Query ciaifearaqhpwpprvatlpHWPLIYAGALE-gLDHL------------elarTVDA--  274
ident                                                       ||
Sbjct -------------------irIGKHLRKVCRFmkAWRDaqwdvggpssislmaaTVNIld  267
DSSP  -------------------hhHLLLHHHHHHHhhHHHHhhlllllllhhhhhhhHHHHhh


DSSP  ---------------------------hhhhhhhhhHHHHH-------------------
Query ---------------------------aaqavqrfvARIDR-------------------  288
ident 
Sbjct svahdasdlgetmkiiakhlpsefargvespdstdeKPLFPpsykhgpremdimsklerl  327
DSSP  hlllllllhhhhhhhhhhhhhhhhhhhllllllllllLLLLLlhhhllhhhhhhhhhhllh


DSSP  ---------------------------------llll
Query ---------------------------------atkr  292
ident 
Sbjct peilssaesadsksealkkinmafgnrvtnselivla  364
DSSP  hhhhhhhhllllhhhhhhhhhhhhllllllhhhleel
```

No 616: Query=mol1A Sbjct=4x4nC Z-score=3.8

back to top

```
DSSP  lllllllllhhhhhhhhhhhhhlLLLHHHHHHHHHHHHHHHHHHHLLLllllllEEEE
Query aapyssspptnlrslrdrltqvaerqGVVFGRLQRHVAXIVVAQFAATLTddtgaplLLVK   60
ident                                     |          |
Sbjct -----------kveeilekalelviPDEEEVRKGREAEEELRRRLDELG-------VEYV   42
DSSP  -----------lhhhhhhhhhhhhlLLHHHHHHHHHHHHHHHHHHHHHHL-------LLEE


DSSP  HHhhhhhhhlllLLLL-----lLLEEEEEL------lLHHHHHHHHHHHHHhhlllleeEE
Query GGsslelrrgipDSRT-----sKDFATVAR------rDIELIHEQLADAGetgwegftAI  109
ident                        |               |   |
Sbjct FV------gsyaRNTWlkgsleIDVFLLFPeefskeeLRERGLEIGKAVL--------DS   88
DSSP  EE------hhhhLLLLllllllEEEEEEELlllllhhHHHHHHHHHHLLL--------LL
```

```
Query [illegible]
ident                                 |
Sbjct YEIRYAE--------hPYVHGVVK-----GVEVDVVPCYKLKE-----------------  118
DSSP  LEEEELL--------lEEEEEEEL-----LEEEEEEEEELLLL-----------------


DSSP  lllleeeeellhhHHHHHHHhhhhlllllllllLLLHhhhhhhhhhhhllhhhhhhhhhh
Query vpaavavpcxtipWQIAQKLhavtavleepkanDRAHdlvdlqlleglllddadlxptrsa  229
ident                    |
Sbjct ---pkniksavdrTPFHHKW------------lEGRI-----------------------  140
DSSP  ---llllllllllHHHHHHH------------hHHHL-----------------------


DSSP  hhhhhhhllllllllllllllhhhHHHHHHLLL-LLLL--------------------l
Query ciaifearaqhpwpprvatlphwplIYAGALEGL-DHLE--------------------l  268
ident                                  |
Sbjct -------------------------KGKENEVRLlKGFLkangiygaeykvrgfsgylce  175
DSSP  -------------------------LLLHHHHHHhHHHHhhllllllllllllllhhhhh


DSSP  llLHHHH----------------hhHHHH--------------hHHHHH-----------
Query arTVDAA----------------aqAVQR--------------fVARID-----------  287
ident    |                      |
Sbjct llIVFYGsfletvknarrwtrrtviDVAKgevrkgeeffvvdpvDEKRNvaanlsldnla  235
DSSP  hhHHHHLlhhhhhhhhllllllleeeELLLleeeellllleeellLLLLLllllllhhhhh


DSSP  ------------------------------------------------------------
Query ------------------------------------------------------------  287
ident
Sbjct rfvhlcrefmeapslgffkpkhpleieperlrkiveergtavfavkfrkpdivddnlypq  295
DSSP  hhhhhhhhhhhhlllhhhhllllllllhhhhhhhhhhhlleeeeeeeellllhhhhhhh


DSSP  ------------------------------------------------------------
Query ------------------------------------------------------------  287
ident
Sbjct lerasrkifeflerenfmplrsafkaseefcyllfecqikeisrvfrrmgpqfedernvk  355
DSSP  hhhhhhhhhhhhhhlllleeeeeeeeellleeeeeeeellllllleeeeeeeelllhhhhh


DSSP  ------------------------------------------------------------
Query ------------------------------------------------------------  287
ident
Sbjct kflsrnrafrpfiengrwwafemrkfttpeegvrsyasthwhtlgknvgesireyfeiis  415
DSSP  hhhhllllllleellleeeeeeellllhhhhhhhhhhlhhhllhhhhhhhhhhlleeee


DSSP  ----------------hllll
Query ----------------ratkr  292
ident
Sbjct geklfkepvtaelcemmgvkd  436
DSSP  hhhhlllllhhhhhhhhllll
```

No 617: Query=mol1A Sbjct=1n44A Z-score=3.8

back to top

```
DSSP  ------------------------------------------------------------
Query ------------------------------------------------------------    0
ident
Sbjct alrgtvtdfsgfdgradaevlekamkglgtdedsilnlltarsnaqrqqiaeefktlfgr   60
DSSP  llllllllllllhhhhhhhhhhhhllllllhhhhhhhhllllhhhhhhhhhhhhhhhhll


DSSP  ---------------------LLLL------------------------llllllhhhhh
Query ---------------------AAPY------------------------sspptnlrslr   15
ident                        |
Sbjct dlvndmkseltgkfeklivalMKPSrlydayelkhalkgagtdekvlteiiasrtpeelr  120
DSSP  lhhhhhhhhlllhhhhhhhhhHLLHhhhhhhhhhhhhllllllhhhhhhhhhhllhhhhh


DSSP  hhhHHHHHHHLLLLHHHHHHHhhhhhhhhhhllllllllleeeehhhhhhhhhhllllll
Query drlTQVAERQGVVFGRLQRHVaxivvaqfaatltddtgaplllvkggsslelrrgipdsr   75
ident        |  |
```

```
DSSP  llllleeeeellllhhhhhhhhhhhhhhllllleeeeeellleellllllllllleeeeeeeeeel
Query tskdfatvarrdieliheqladagetgwegftaiftapeeiaapgxpvkprrftaklsyr  135
ident
Sbjct ------------------------------------------------------------  141
DSSP  ------------------------------------------------------------


DSSP  leeeeeeeeeeeelllllllllleeeeeelhhhhllllllleeeeeLLHHHHHHHHHHHHHLl
Query grafatvpievssveagnadqfdtltsdalglvgvpaavavpcXTIPWQIAQKLHAVTAv  195
ident                                                   || |
Sbjct -------------------tsgyyqrmlvvllqanrdpdtaidDAQVELDAQALFQAGE-  181
DSSP  -------------------llhhhhhhhhhhhlllllllllllHHHHHHHHHHHHHHHLL-


DSSP  llllllLLLLhhHHHHHHHHHHHLLHHHHHHHHHHHHHHHHhhhlllLLLL--LLLLlllhh
Query leepkaNDRAhdLVDLQLLEGLLLDADLXPTRSACIAIFearaqhPWPP--RVATlphwp  253
ident                     |      |         |
Sbjct ----lkWGTD--EEKFITILGTRSVSHLRRVFDKYMTIS------GFQIeeTIDR-----  224
DSSP  ----llLLLL--HHHHHHHHHHLLHHHHHHHHHHHHHHH------LLLHhhHHHH-----


DSSP  hhhhhhhllllllllllHHHHHHHHHHHHHHHHH-------------------------
Query liyagalegldhlelartVDAAAQAVQRFVARIDR-------------------------  288
ident                              |  |
Sbjct ----------------etSGNLENLLLAVVKSIRSipaylaetlyyamkgagtddhtlir  268
DSSP  ----------------hlLHHHHHHHHHHHHHHHHLhhhhhhhhhhhhhllllllhhhhhh


DSSP  ----------------------------------------------llll
Query ----------------------------------------------atkr  292
ident
Sbjct vivsrseidlfnirkefrknfatslysmikgdtsgdykkallllcggedd  318
DSSP  hhhhhllllhhhhhhhhhhhhlllhhhhhhhhllhhhhhhhhhhhlllll
```

No 618: Query=mol1A Sbjct=4evpA Z-score=3.8

back to top

```
DSSP  --------------------------------LLLL----llLLLLLHHHHHHHHHHHHHHH
Query --------------------------------AAPY----ssPPTNLRSLRDRLTQVAER   24
ident                                                   |
Sbjct dedqvvvnkaavmvhqlskkeasrhaimrspqMVSAivrtmqNTNDVETARCTAGTLHNL   60
DSSP  lllllllhhhhhhhhhhlllhhhhhhhlllllHHHHhhhhhhHLLLHHHHHHHHHHHHHHH


DSSP  LL---------llhHHHHHHHHHHH--------------------hhhhhhllllllll
Query QG---------vvfGRLQRHVAXIV--------------------vaqfaatltddtgap   55
ident               |
Sbjct SHhregllaifksgGIPALVKMLGSpvdsvlfyaittlhnlllhqegakmavrlagglqk  120
DSSP  LLlhhhhhhhhhllHHHHHHHHHHLllhhhhhhhhhhhhhhhhhlllhhhhhhhllhhhh


DSSP  leEEEH--------HHHHHHHHLllllllllleeeeelllhhhhhhhhhhhhhhlllllee
Query llLVKG--------GSSLELRRGipdsrtskdfatvarrdieliheqladagetgwegft  107
ident                    |
Sbjct mvALLNktnvkflaITTDCLQIL-------------------------------------  143
DSSP  hhHHHHlllhhhhhHHHHHHHHH-------------------------------------


DSSP  eeellleellllllllllleeeeeeeeelleeeeeeeeeeeeellllllllleeeeelhhhh
Query aiftapeeiaapgxpvkprrftaklsyrgrafatvpievssveagnadqfdtltsdalgl  167
ident
Sbjct ----------------------------------------------------------ay  145
DSSP  ----------------------------------------------------------hl


DSSP  llllllleeeeeLLHHHHHHHHHHHHHhllllllllllLLHHHHHHHHHHHHL--LHHHHHH
Query vgvpaavavpcXTIPWQIAQKLHAVtavleepkandRAHDLVDLQLLEGLL--LDADLXP  225
ident               |                         |         |
Sbjct gnqeskliilaSGGPQALVNIMRTY----------tYEKLLWTTSRVLKVLsvCSSNKPA  195
DSSP  llhhhhhhhhhLLHHHHHHHHHHHHL----------lLHHHHHHHHHHHHHHllLLLHHHH
```

```
ident    |                      |                            |
Sbjct IVEAG--------gmqalGLHLT---------------------dPSQRLVQNCLWTLRN  226
DSSP  HHHLL--------hhhhhHLLLL---------------------lLLHHHHHHHHHHHHH


DSSP  HHHLLLL-----------------------------------------------------
Query IDRATKR-----------------------------------------------------  292
ident    |
Sbjct LSDAATKqegmegllgtlvqllgsddinvvtcaagilsnltcnnyknkmmvcqvggieal  286
DSSP  HHLLLLLllllhhhhhhhhhhlllllhhhhhhhhhhhhhhhlllhhhhhhhhhllhhhhh


DSSP  ------------------------------------------------------------
Query ------------------------------------------------------------  292
ident 
Sbjct vrtvlragdreditepaicalrhltsrhqeaemaqnavrlhyglpvvvkllhppshwpli  346
DSSP  hhhhhhlllhhhhhhhhhhhhhhlllllhhhhhhhhhhlllhhhhhhlllllllhhhh


DSSP  ------------------------------------------------------------
Query ------------------------------------------------------------  292
ident 
Sbjct katvglirnlalcpanhaplreqgaiprlvqllvrahqdtqregvrmeeivegctgalhi  406
DSSP  hhhhhhhhhlllhhhhhhhhhhllhhhhhhhhhhhhhhhhhlllllhhhhhhhhhhhhhh


DSSP  ------------------------------------------------------------
Query ------------------------------------------------------------  292
ident 
Sbjct lardvhnrivirglntiplfvqllyspieniqrvaagvlcelaqdkeaaeaieaegatap  466
DSSP  hlllhhhhhhhhhlllhhhhhhhlllllhhhhhhhhhhhhhhhlllhhhhhhhhhhlllhhh


DSSP  -----------------------
Query -----------------------  292
ident 
Sbjct ltellhsrnegvatyaaavlfrm  489
DSSP  hhhhlllllhhhhhhhhhhhhhl
```

No 619: Query=mol1A Sbjct=4ev9A Z-score=3.8

back to top

```
DSSP  -----------------------------------LLLL----llLLLLHHHHHHHHHHH
Query -----------------------------------AAPY----ssPPTNLRSLRDRLTQV   21
ident                                                      |
Sbjct llndedqvvvnkaavmvhqlskkeasrhaimrspqMVSAivrtmqNTNDVETARCTAGTL   60
DSSP  lllllllllllllhhhhhhhhllhhhhhhhlllhhHHHHhhhhhhHLLLHHHHHHHHHHHH


DSSP  HHHLL---------llhHHHHHHHHHHH--------------------hhhhhhllllll
Query AERQG---------vvfGRLQRHVAXIV--------------------vaqfaatltddt   52
ident                  |
Sbjct HNLSHhregllaifksgGIPALVKMLGSpvdsvlfyaittlhnlllhqegakmavrlagg  120
DSSP  HHHLLlhhhhhhhhhllHHHHHHHHHHLllhhhhhhhhhhhhhhhhllllhhhhhhhhlh


DSSP  llllеEEEH--------HHHHHHHHLllllllllleeeeelllhhhhhhhhhhhhhhlll
Query gapllLVKG--------GSSLELRRGipdsrtskdfatvarrdieliheqladagetgwe  104
ident                       |
Sbjct lqkmvALLNktnvkflaITTDCLQIL----------------------------------  146
DSSP  hhhhhHHHHlllhhhhhHHHHHHHHH----------------------------------


DSSP  leeeeellleellllllllllleeeeeeeeeelleeeeeeeeeeeelllllllllleeeeelh
Query gftaiftapeeiaapgxpvkprrftaklsyrgrafatvpievssveagnadqfdtltsda  164
ident 
Sbjct ------------------------------------------------------------  146
DSSP  ------------------------------------------------------------


DSSP  hhhllllllleeeeeLLHHHHHHHHHHHHHhllllllllllLLHHHHHHHHHHHHHL--LHHH
Query lglvgvpaavavpcXTIPWQIAQKLHAVtavleepkandRAHDLVDLQLLEGLL--LDAD  222
ident                  |                         |         |
Sbjct -aygnqeskliilaSGGPQALVNIMRTY----------tYEKLLWTTSRVLKVLsvCSSN  195
```

```
DSSP  HHHHHHHHhhhhhhhhlllllLLLLLlllhhhhhhhhhllllllllllLHHHHHHHHHHH
Query LXPTRSACiaifearaqhpwpPRVATlphwpliyagalegldhlelarTVDAAAQAVQRF  282
ident       |                  |                            |
Sbjct KPAIVEAG--------gmqalGLHLT---------------------dPSQRLVQNCLWT  226
DSSP  HHHHHHLL--------hhhhhHLLLL---------------------lLLHHHHHHHHHH


DSSP  HHHHHHLLLL--------------------------------------------------
Query VARIDRATKR--------------------------------------------------  292
ident       |
Sbjct LRNLSDAATKqegmegllgtlvqllgsddinvvtcaagilsnltcnnyknkmmvcqvggi  286
DSSP  HHHHLLLLLLllllhhhhhhhhhhllllhhhhhhhhhhhhhhhlllhhhhhhhhhhllhh


DSSP  ------------------------------------------------------------
Query ------------------------------------------------------------  292
ident
Sbjct ealvrtvlragdreditepaicalrhltsrhqeaemaqnavrlhyglpvvvkllhppshw  346
DSSP  hhhhhhhhhhlllhhhhhhhhhhhhhhlllllhhhhhhhhhhhlllhhhhhhllllllllh


DSSP  ------------------------------------------------------------
Query ------------------------------------------------------------  292
ident
Sbjct plikatvglirnlalcpanhaplreqgaiprlvqllvrahqdtqregvrmeeivegctga  406
DSSP  hhhhhhhhhhhhlllhhhhhhhhhllhhhhhhhhhhhhhhhhhlllllhhhhhhhhhhhh


DSSP  ------------------------------------------------------------
Query ------------------------------------------------------------  292
ident
Sbjct lhilardvhnrivirglntiplfvqllyspieniqrvaagvlcelaqdkeaaeaieaega  466
DSSP  hhhhlllhhhhhhhhhhlllhhhhhhlllllhhhhhhhhhhhhhhhlllhhhhhhhhlll


DSSP  --------------------------
Query --------------------------  292
ident
Sbjct tapltellhsrnegvatyaaavlfrm  492
DSSP  hhhhhhhlllllhhhhhhhhhhhhhl
```

No 620: Query=mol1A Sbjct=4evtA Z-score=3.8

back to top

```
DSSP  ---------------------------------LLLL----llLLLLLHHHHHHHHHHHH
Query ---------------------------------AAPY----ssPPTNLRSLRDRLTQVAE   23
ident                                                    |
Sbjct ndedqvvvnkaavmvhqlskkeasrhaimrspqMVSAivrtmqNTNDVETARCTAGTLHN   60
DSSP  lllllllllhhhhhhhhhhhlhhhhhhhhllllLHHHhhhhhhHLLLHHHHHHHHHHHHH


DSSP  HLL---------llhHHHHHHHHHHH--------------------hhhhhhllllllll
Query RQG---------vvfGRLQRHVAXIV--------------------vaqfaatltddtga   54
ident                |
Sbjct LSHhregllaifksgGIPALVKMLGSpvdsvlfyaittlhnlllhqegakmavrlagglq  120
DSSP  HLLlhhhhhhhhhllHHHHHHHHHHLlllhhhhhhhhhhhhhhhllllhhhhhhhllhhh


DSSP  lleEEEH--------HHHHHHHHLlllllllleeeeelllhhhhhhhhhhhhhhhlllle
Query pllLVKG--------GSSLELRRGipdsrtskdfatvarrdielihegladagetgwegf  106
ident                     |
Sbjct kmvALLNktnvkflaITTDCLQIL------------------------------------  144
DSSP  hhhHHHHlllhhhhhHHHHHHHHH------------------------------------


DSSP  eeeellleelllllllllleeeeeeeeeelleeeeeeeeeeeelllllllllleeeeelhhh
Query taiftapeeiaapgxpvkprrftaklsyrgrafatvpievssveagnadqfdtltsdalg  166
ident
Sbjct -----------------------------------------------------------a  145
DSSP  -----------------------------------------------------------h


DSSP  hlllllleeeeeLLHHHHHHHHHHHHhllllllllllLLHHHHHHHHHHHHL--LHHHHH
```

```
Sbjct ygnqeskiiiiaSGGrQALVNIMKTI[illegible]tIEKLLWTTSKVLKVLSVCSSNKP  195
DSSP  lllhhhhhhhhhLLHHHHHHHHHHHHL----------lLHHHHHHHHHHHHHHHhlLLLHHH


DSSP  HHHHHHhhhhhhhllllllLLLLLlllhhhhhhhhhlllllllllLHHHHHHHHHHHHH
Query PTRSACiaifearaqhpwpPRVATlphwpliyagalegldhlelarTVDAAAQAVQRFVA  284
ident     |                  |                            |
Sbjct AIVEAG--------gmqalGLHLT---------------------dPSQRLVQNCLWTLR  226
DSSP  HHHHLL--------hhhhhHLLLL---------------------lLLHHHHHHHHHHHH


DSSP  HHHHLLLL----------------------------------------------------
Query RIDRATKR----------------------------------------------------  292
ident     |
Sbjct NLSDAATKqegmegllgtlvqllgsddinvvtcaagilsnltcnnyknkmmvcqvggiea  286
DSSP  HHHLLLLLllllhhhhhhhhhhlllllhhhhhhhhhhhhhhhlllhhhhhhhhhllhhhh


DSSP  ------------------------------------------------------------
Query ------------------------------------------------------------  292
ident
Sbjct lvrtvlragdreditepaicalrhltsrhqeaemaqnavrlhyglpvvvkllhppshwpl  346
DSSP  hhhhhhhhlllhhhhhhhhhhhhhhlllllhhhhhhhhhhlllhhhhhhlllllllhhh


DSSP  ------------------------------------------------------------
Query ------------------------------------------------------------  292
ident
Sbjct ikatvglirnlalcpanhaplreqgaiprlvqllvrahqdtqregvrmeeivegctgalh  406
DSSP  hhhhhhhhhhhlllhhhhhhhhhllhhhhhhhhhhhhhhhhhllllhhhhhhhhhhhhh


DSSP  ------------------------------------------------------------
Query ------------------------------------------------------------  292
ident
Sbjct ilardvhnrivirglntiplfvqllyspieniqrvaagvlcelaqdkeaaeaieaegata  466
DSSP  hhlllhhhhhhhhhlllhhhhhhhhhlllhhhhhhhhhhhhhhlllhhhhhhhhhlllhh


DSSP  ------------------------
Query ------------------------  292
ident
Sbjct pltellhsrnegvatyaaavlfrm  490
DSSP  hhhhhhhlllhhhhhhhhhhhhhl
```

No 621: Query=mol1A Sbjct=4evaA Z-score=3.8

back to top

```
DSSP  ----------------------------------LLLL----llLLLLHHHHHHHHHHHH
Query ----------------------------------AAPY----ssPPTNLRSLRDRLTQVA   22
ident                                                     |
Sbjct lndedqvvvnkaavmvhqlskkeasrhaimrspqMVSAivrtmqNTNDVETARCTAGTLH   60
DSSP  llllllllllhhhhhhhhhhhlhhhhhhhhllhhHHHHhhhhhhHLLLHHHHHHHHHHHH


DSSP  HHLL---------llhHHHHHHHHHHH--------------------hhhhhhlllllll
Query ERQG---------vvfGRLQRHVAXIV--------------------vaqfaatltddtg   53
ident                 |
Sbjct NLSHhregllaifksgGIPALVKMLGSpvdsvlfyaittlhnlllhqegakmavrlaggl  120
DSSP  HHLLlhhhhhhhhhllHHHHHHHHHHLllhhhhhhhhhhhhhhhhhlllhhhhhhhllhh


DSSP  llleEEEH--------HHHHHHHHLlllllllleeeeelllhhhhhhhhhhhhhhllll
Query apllLVKG--------GSSLELRRGipdsrtskdfatvarrdieliheqladagetgweg  105
ident                      |
Sbjct qkmvALLNktnvkflaITTDCLQIL-----------------------------------  145
DSSP  hhhhHHHHlllhhhhhHHHHHHHHHH-----------------------------------


DSSP  eeeeellleellllllllleeeeeeeeelleeeeeeeeeeeellllllllleeeeelhh
Query ftaiftapeeiaapgxpvkprrftaklsyrgrafatvpievssveagnadqfdtltsdal  165
ident
Sbjct ------------------------------------------------------------  145
DSSP  ------------------------------------------------------------
```

```
DSSP  [illegible]
Query glvgvpaavavpcXTIPWQIAQKLHAVtavleepkandRAHDLVDLQLLEGLL--LDADL  223
ident                 |                         |         |
Sbjct aygnqeskliilaSGGPQALVNIMRTY----------tYEKLLWTTSRVLKVLsvCSSNK  195
DSSP  hlllhhhhhhhhhLLHHHHHHHHHHHHL----------lLHHHHHHHHHHHHHHllLLLHH


DSSP  HHHHHHHhhhhhhllllllLLLLLlllhhhhhhhhhlllllllllLHHHHHHHHHHHH
Query XPTRSACiaifearaqhpwpPRVATlphwpliyagalegldhlelarTVDAAAQAVQRFV  283
ident      |                  |                            |
Sbjct PAIVEAG--------gmqalGLHLT---------------------dPSQRLVQNCLWTL  226
DSSP  HHHHHLL--------hhhhhHLLLL---------------------lLLHHHHHHHHHHH


DSSP  HHHHHLLLL---------------------------------------------------
Query ARIDRATKR---------------------------------------------------  292
ident      |
Sbjct RNLSDAATKqegmegllgtlvqllgsddinvvtcaagilsnltcnnyknkmmvcqvggie  286
DSSP  HHHLLLLLLllllhhhhhhhhhhlllllhhhhhhhhhhhhhhhlllhhhhhhhhhllhhh


DSSP  ------------------------------------------------------------
Query ------------------------------------------------------------  292
ident
Sbjct alvrtvlragdreditepaicalrhltsrhqeaemaqnavrlhyglpvvvkllhppshwp  346
DSSP  hhhhhhhhhlllhhhhhhhhhhhhhhhlllllhhhhhhhhhhlllhhhhhhlllllllhh


DSSP  ------------------------------------------------------------
Query ------------------------------------------------------------  292
ident
Sbjct likatvglirnlalcpanhaplreqgaiprlvqllvrahqdtqregvrmeeivegctgal  406
DSSP  hhhhhhhhhhhhlllhhhhhhhhhllhhhhhhhhhhhhhhhhhlllllhhhhhhhhhhhh


DSSP  ------------------------------------------------------------
Query ------------------------------------------------------------  292
ident
Sbjct hilardvhnrivirglntiplfvqllyspieniqrvaagvlcelaqdkeaaeaieaegat  466
DSSP  hhhlllhhhhhhhhhlllhhhhhhhlllllhhhhhhhhhhhhhhhlllhhhhhhhhhlllh


DSSP  -------------------------
Query -------------------------  292
ident
Sbjct apltellhsrnegvatyaaavlfrm  491
DSSP  hhhhhhlllllhhhhhhhhhhhhhl
```

No 622: Query=mol1A Sbjct=4ev8A Z-score=3.8

back to top

```
DSSP  -----------------------------------LLLL----llLLLLLHHHHHHHHHH
Query -----------------------------------AAPY----ssPPTNLRSLRDRLTQV   21
ident                                                      |
Sbjct llndedqvvvnkaavmvhqlskkeasrhaimrspqMVSAivrtmqNTNDVETARCTAGTL   60
DSSP  lllllllllllllhhhhhhhlllhhhhhhhhllllLHHHhhhhhhHLLLLHHHHHHHHHHH


DSSP  HHHLL---------llhHHHHHHHHHHH--------------------hhhhhhllllll
Query AERQG---------vvfGRLQRHVAXIV--------------------vaqfaatltddt   52
ident                  |
Sbjct HNLSHhregllaifksgGIPALVKMLGSpvdsvlfyaittlhnlllhqegakmavrlagg  120
DSSP  HHHLLlhhhhhhhhhllHHHHHHHHHHLllhhhhhhhhhhhhhhhhhlllhhhhhhhllh


DSSP  lllleEEEH--------HHHHHHHHLlllllllleeeeellllhhhhhhhhhhhhhhlll
Query gapllLVKG--------GSSLELRRGipdsrtskdfatvarrdielihe qladagetgwe  104
ident                       |
Sbjct lqkmvALLNktnvkflaITTDCLQIL----------------------------------  146
DSSP  hhhhhHHHHlllhhhhhHHHHHHHHH----------------------------------


DSSP  leeeeellleellllllllleeeeeeeeelleeeeeeeeeeeelllllllllleeeeelh
Query gftaiftapeeiaapgxpvkprrftaklsyrgrafatvpievssveagnadqfdtltsda  164
```

```
DSSP

DSSP  hhhllllleeeeeLLHHHHHHHHHHHHHhllllllllllLLHHHHHHHHHHHHL--LHHH
Query lglvgvpaavavpcXTIPWQIAQKLHAVtavleepkandRAHDLVDLQLLEGLL--LDAD  222
ident                  |                         |         |
Sbjct -aygnqeskliilaSGGPQALVNIMRTY----------tYEKLLWTTSRVLKVLsvCSSN  195
DSSP  -hlllhhhhhhhhhLLHHHHHHHHHHHL----------lLHHHHHHHHHHHHHHllLLLH


DSSP  HHHHHHHHhhhhhhhllllllLLLLLlllhhhhhhhhhllllllllllLHHHHHHHHHHH
Query LXPTRSACiaifearaqhpwpPRVATlphwpliyagalegldhlelarTVDAAAQAVQRF  282
ident       |                  |                            |
Sbjct KPAIVEAG--------gmqalGLHLT---------------------dPSQRLVQNCLWT  226
DSSP  HHHHHHLL--------hhhhhHLLLL---------------------lLLHHHHHHHHHH


DSSP  HHHHHHLLLL--------------------------------------------------
Query VARIDRATKR--------------------------------------------------  292
ident       |
Sbjct LRNLSDAATKqegmegllgtlvqllgsddinvvtcaagilsnltcnnyknkmmvcqvggi  286
DSSP  HHHHHLLLLLllllhhhhhhhhhhlllllhhhhhhhhhhhhhhhlllhhhhhhhhhllhh


DSSP  ------------------------------------------------------------
Query ------------------------------------------------------------  292
ident
Sbjct ealvrtvlragdreditepaicalrhltsrhqeaemaqnavrlhyglpvvvkllhppshw  346
DSSP  hhhhhhhhhhlllhhhhhhhhhhhhhhlllllhhhhhhhhhhhlllhhhhhhlllllllh


DSSP  ------------------------------------------------------------
Query ------------------------------------------------------------  292
ident
Sbjct plikatvglirnlalcpanhaplreqgaiprlvqllvrahqdtqregvrmeeivegctga  406
DSSP  hhhhhhhhhhhhhlllhhhhhhhhhllhhhhhhhhhhhhhhhhlllllhhhhhhhhhhhh


DSSP  ------------------------------------------------------------
Query ------------------------------------------------------------  292
ident
Sbjct lhilardvhnrivirglntiplfvqllyspieniqrvaagvlcelaqdkeaaeaieaega  466
DSSP  hhhhlllhhhhhhhhhlllhhhhhhhlllllhhhhhhhhhhhhhlllhhhhhhhhhhlll


DSSP  --------------------------
Query --------------------------  292
ident
Sbjct tapltellhsrnegvatyaaavlfrm  492
DSSP  hhhhhhhlllllhhhhhhhhhhhhhl
```

No 623: Query=mol1A Sbjct=4evaC Z-score=3.8

back to top

```
DSSP  -------------------------------LLLL----llLLLLHHHHHHHHHHHHHHL
Query -------------------------------AAPY----ssPPTNLRSLRDRLTQVAERQ   25
ident                                                  |
Sbjct edqvvvnkaavmvhqlskkeasrhaimrspqMVSAivrtmqNTNDVETARCTAGTLHNLS   60
DSSP  lllllllhhhhhhhhhhhhlhhhhlllllhhHHHHhhhhhhHLLLHHHHHHHHHHHHHHL


DSSP  L---------llhHHHHHHHHHHH--------------------hhhhhhllllllllll
Query G---------vvfGRLQRHVAXIV--------------------vaqfaatltddtgapl   56
ident              |
Sbjct HhregllaifksgGIPALVKMLGSpvdsvlfyaittlhnlllhqegakmavrlagglqkm  120
DSSP  LlhhhhhhhhhllHHHHHHHHHHLllhhhhhhhhhhhhhhhhhlllhhhhhhhllhhhhh


DSSP  eEEEH--------HHHHHHHHLllllllllleeeeelllhhhhhhhhhhhhhhlllleee
Query lLVKG--------GSSLELRRGipdsrtskdfatvarrdieliheqladagetgwegfta  108
ident                   |
Sbjct vALLNktnvkflaITTDCLQIL--------------------------------------  142
DSSP  hHHHHlllhhhhhHHHHHHHHH--------------------------------------
```

```
Query iftapeeiaapgxpvkprrftaklsyrgrafatvpievssveaghadqrdtrtsdaigrv  168
ident
Sbjct ---------------------------------------------------------ayg  145
DSSP  ---------------------------------------------------------hll


DSSP  llllleeeeeLLHHHHHHHHHHHHhllllllllllLLHHHHHHHHHHHHL--LHHHHHHH
Query gvpaavavpcXTIPWQIAQKLHAVtavleepkandRAHDLVDLQLLEGLL--LDADLXPT  226
ident              |                         |         |
Sbjct nqeskliilaSGGPQALVNIMRTY----------tYEKLLWTTSRVLKVLsvCSSNKPAI  195
DSSP  lhhhhhhhhhLLHHHHHHHHHHHL----------lLHHHHHHHHHHHHHHllLLLHHHHH


DSSP  HHHHhhhhhhhhlllllLLLLLlllhhhhhhhhhllllllllllLHHHHHHHHHHHHHHH
Query RSACiaifearaqhpwpPRVATlphwpliyagalegldhlelarTVDAAAQAVQRFVARI  286
ident   |                  |                            |
Sbjct VEAG--------gmqalGLHLT---------------------dPSQRLVQNCLWTLRNL  226
DSSP  HHLL--------hhhhhHLLLL---------------------lLLHHHHHHHHHHHHHH


DSSP  HHLLLL------------------------------------------------------
Query DRATKR------------------------------------------------------  292
ident   |
Sbjct SDAATKqegmegllgtlvqllgsddinvvtcaagilsnltcnnyknkmmvcqvggiealv  286
DSSP  LLLLLLllllhhhhhhhhhhlllllhhhhhhhhhhhhhhhlllhhhhhhhhhllhhhhhh


DSSP  ------------------------------------------------------------
Query ------------------------------------------------------------  292
ident
Sbjct rtvlragdreditepaicalrhltsrhqeaemaqnavrlhyglpvvvkllhppshwplik  346
DSSP  hhhhhhlllhhhhhhhhhhhhhhhlllllhhhhhhhhhhlllhhhhhhlllllllhhhhh


DSSP  ------------------------------------------------------------
Query ------------------------------------------------------------  292
ident
Sbjct atvglirnlalcpanhaplreqgaiprlvqllvrahqdtqregvrmeeivegctgalhil  406
DSSP  hhhhhhhhhlllhhhhhhhhhllhhhhhhhhhhhhhhhhhhlllllhhhhhhhhhhhhhh


DSSP  ------------------------------------------------------------
Query ------------------------------------------------------------  292
ident
Sbjct ardvhnrivirglntiplfvqllyspieniqrvaagvlcelaqdkeaaeaieaegatapl  466
DSSP  lllhhhhhhhhhlllhhhhhhhhhlllhhhhhhhhhhhhhhlllhhhhhhhhhlllhhhh


DSSP  ----------------------
Query ----------------------  292
ident
Sbjct tellhsrnegvatyaaavlfrm  488
DSSP  hhhhhlllhhhhhhhhhhhhhl
```

No 624: Query=mol1A Sbjct=1g3jC Z-score=3.8

back to top

```
DSSP  llllllllllhhhhhhHHHHHHHHLlllhhhHHHHHHHHHHhhHHHL-------------
Query aapysspptnlrslrdRLTQVAERQgvvfgrLQRHVAXIVVaqFAAT-------------   47
ident                  |                 |
Sbjct ------------hhreGLLAIFKSG-----gIPALVKMLGS--PVDSvlfyaittlhnll   41
DSSP  ------------llhhHHHHHHHLL-----lHHHHHHLLLL--LLHHhhhhhhhhhhhhh


DSSP  ----------lllllllllleEEEH--------HHHHHHHHLllllllllleeeeelllhh
Query ----------ltddtgapllLVKG--------GSSLELRRGipdsrtskdfatvarrdie   89
ident                                      |
Sbjct lhqegakmavrlagglqkmvALLNktnvkflaITTDCLQIL-------------------   82
DSSP  hllllhhhhhhhllhhhhhhHHHHlllhhhhhHHHHHHHHH-------------------


DSSP  hhhhhhhhhhhhlllleeeeellleellllllllllleeeeeeeeelleeeeeeeeeeeel
Query liheqladagetgwegftaiftapeeiaapgxpvkprrftaklsyrgrafatvpievssv  149
ident
```

```
DSSP  lllllllleeeeelhhhhlllllleeeeeLLHHHHHHHHHHHHhllllllllllLLHHHH
Query eagnadqfdtltsdalglvgvpaavavpcXTIPWQIAQKLHAVtavleepkandRAHDLV  209
ident                                 |                         |
Sbjct ----------------aygnqeskliilaSGGPQALVNIMRTY----------tYEKLLW  116
DSSP  ----------------hlllhhhhhhhhhLLHHHHHHHHHHHL----------lLHHHHH


DSSP  HHHHHHHHL--LHHHHHHHHHHHhhhhhhhllllllLLLLLlllhhhhhhhhhlllllll
Query DLQLLEGLL--LDADLXPTRSACiaifearaqhpwpPRVATlphwpliyagalegldhle  267
ident         |            |                  |
Sbjct TTSRVLKVLsvCSSNKPAIVEAG--------gmqalGLHLT-------------------  149
DSSP  HHHHHHHHHllLLLHHHHHHHLL--------hhhhhHLLLL-------------------


DSSP  lllLHHHHHHHHHHHHHHHHHLLLL-----------------------------------
Query larTVDAAAQAVQRFVARIDRATKR-----------------------------------  292
ident          |           |
Sbjct --dPSQRLVQNCLWTLRNLSDAATKqegmegllgtlvqllgsddinvvtcaagilsnltc  207
DSSP  --lLLHHHHHHHHHHHHHHHHHLLLllllhhhhhhhhhhlllllhhhhhhhhhhhhhhll


DSSP  ------------------------------------------------------------
Query ------------------------------------------------------------  292
ident
Sbjct nnyknkmmvcqvggiealvrtvlragdreditepaicalrhltsrhqeaemaqnavrlhy  267
DSSP  llhhhhhhhhhllhhhhhhhhhhhhlllhhhhhhhhhhhhhhlllllhhhhhhhhhhll


DSSP  ------------------------------------------------------------
Query ------------------------------------------------------------  292
ident
Sbjct glpvvvkllhppshwplikatvglirnlalcpanhaplreqgaiprlvqllvrahqdtqr  327
DSSP  lhhhhhhllllllhhhhhhhhhhhhhhlllhhhhhhhhhllhhhhhhhhhhhhhhhhh


DSSP  ------------------------------------------------------------
Query ------------------------------------------------------------  292
ident
Sbjct rtsmgqqfvegvrmeeivegctgalhilardvhnrivirglntiplfvqllyspieniqr  387
DSSP  hhhhlllllllllhhhhhhhhhhhhhhhlllhhhhhhhhhlllhhhhhhhhhlllhhhhh


DSSP  ----------------------------------------------------
Query ----------------------------------------------------  292
ident
Sbjct vaagvlcelaqdkeaaeaieaegatapltellhsrnegvatyaaavlfrmse  439
DSSP  hhhhhhhhhlllhhhhhhhhhlllhhhhhhhlllllhhhhhhhhhhhhhhll
```

No 625: Query=mol1A Sbjct=4amqA Z-score=3.7

back to top

```
DSSP  llllllllllhhhhhHHHHhhhhhllllHHHHHHHHHH-HHHHHHHhllllllllllleEE
Query aapysspptnlrslrDRLTqvaerqgvvFGRLQRHVAX-IVVAQFAatltddtgapllLV   59
ident                  |          |
Sbjct ---------------RMLI---------FTYKLERYIKnKILPKIL------vvpdrdKY   30
DSSP  ---------------LLLL---------LLHHHHHHHHhHLHHHHL------llllhhHE


DSSP  EHHhhhhhhhlllllLLLL---------LEEEEEL-----lLHHHHHHHHHHHHHHLLll
Query KGGsslelrrgipdsRTSK---------DFATVAR-----rDIELIHEQLADAGETGWeg  105
ident                             |             |      |
Sbjct QIK------------GSFRrripyitdiDIVNNVHpeyddtNIYQRIVDLINSFTNDN--   76
DSSP  EEE------------HHHHlllllllleeEEEEELLllllllLHHHHHHHHHHHHLLLL--


DSSP  eEEEELLLEELL------------------------------------------------
Query fTAIFTAPEEIA------------------------------------------------  117
ident
Sbjct -QIKLIYVICGTddrfllteysdeeiekikillnptelvelnnvnkkvfyineiiwdlyk  135
DSSP  -LEEEEEEEEEElhhhlllllhhhhhhhhllllllhhhhllllllllhhhhhhhhhhhe
```

```
ident                                                   | |
Sbjct lrwtssevlagkkilrggievsfqdvvKNNSILLLQYFVKI-eYYPIGFDIAVRYKP---  191
DSSP  eeelhhhhhhleeelhhhleeehhhhhHLLLEEEEEEEEEL-lLLEEEEEEEEELLL---


DSSP  lllleeeeelhhhhlllllleeeeellhhhhhhhhhhhhhlllllLLLLllhhhhhhhh
Query adqfdtltsdalglvgvpaavavpcxtipwqiaqklhavtavleepKANDrahdlvdlql  213
ident
Sbjct ----------------------------------------------FYQL----------  195
DSSP  ----------------------------------------------LLLL----------


DSSP  hhhhllhhhhhhhhhhhhhhhhhhlllllllLLLL-LLLHHHHHHHhhlllllllLLLL-
Query leglllldadlxptrsaciaifearaqhpwppRVAT-LPHWPLIYAGalegldhleLART-  271
ident                                            |
Sbjct -------------------------klanysKEYYfMLFPLRFYFK-------ndPTISk  223
DSSP  -------------------------lhhhllLLLLlLHHHHHHHLL-------llHHHHh


DSSP  ------------------------------------------------------------
Query ------------------------------------------------------------  271
ident
Sbjct qleyiietkfglykqllvridsyrtiyesgnldldtaksiiisiikdirklngidmniid  283
DSSP  hhhhhhhlllhhhhhhhhhhhhhhhhhhhllllhhhhhhhhhhhhhhhllllllhhhhh


DSSP  -------------------------------------hhhhhhhhhhhhhhhhhllll
Query -------------------------------------vdaaaqavqrfvaridratkr  292
ident
Sbjct kiqevsnnsagqdkiiawntlltqlytninksvnkqskkyftryiniipkedrklccl  341
DSSP  hhhhhhhhllhhhhhhhhhhhhhhhhhhhhhhhhhhhhhhhhhhhhhhllllhhhhlllll
```

No 626: Query=mol1A Sbjct=3ovsB Z-score=3.7

back to top

```
DSSP  lllllllllhhhhhhhhhhhhhhlLLLHHHHHHHHHHHHHHHHHHHLLLlllllllEEEE
Query aapysspptnlrslrdrltqvaerqGVVFGRLQRHVAXIVVAQFAATLTddtgaplLLVK   60
ident                                      |          |
Sbjct ----------mkveeilekalelviPDEEEVRKGREAEEELRRRLDELG-------VEYV   43
DSSP  ----------llllhhhhhhhhhhlLLHHHHHHHHHHHHHHHHHHHHHLL-------LLEE


DSSP  HHhhhhhhhlllLLLL-----lLLEEEEEL------lLHHHHHHHHHHHHhhllllleeEE
Query GGsslelrrgipDSRT-----sKDFATVAR------rDIELIHEQLADAGetgwegftAI  109
ident                        |               |   |
Sbjct FV------gsyaRNTWlkgsleIDVFLLFPeefskeeLRERGLEIGKAVL--------DS   89
DSSP  EE------llhhHLLLlllllleEEEEEEELllllhhhHHHHHHHHHHHLLL--------LE


DSSP  ELLLEELlllllllLEEEEEEEEelleeEEEEEEEEEEELLLLlllllleeeeelhhhhll
Query FTAPEEIaapgxpvkPRRFTAKLSyrgraFATVPIEVSSVEAGnadqfdtltsdalglvg  169
ident                                 |
Sbjct YEIRYAE--------HPYVHGVVK-----GVEVDVVPCYKLKE-----------------  119
DSSP  EEELLLL--------LLLEEEEEL-----LEEEEEEEEELLLL-----------------


DSSP  llllleeeeellhhhhhhHHHHHHhllllllllLLLLhhhhhhhhhhhhllhhhhhhhhhh
Query vpaavavpcxtipwqiaQKLHAVtavleepkaNDRAhdlvdlqllegllldadlxptrsa  229
ident                                   |
Sbjct -----pkniksavdrtpFHHKWL---------EGRI------------------------  141
DSSP  -----lllllllllhhhHHHHHH---------LLLL------------------------


DSSP  hhhhhhhhllllllllllllllhhhHHHHHHHLL-LLLL-------------------lll
Query ciaifearaqhpwpprvatlphwplIYAGALEG-LDHL-------------------ela  269
ident                                      |
Sbjct ------------------------kGKENEVRLlKGFLkangiygaeykvrgfsgylcel  177
DSSP  ------------------------lLLLHHHHHhHHHHhhllllllllllllllhhhhhh


DSSP  LLHHHH----------------hhHHHHHH-------------------------------
Query RTVDAA----------------aqAVQRF-------------------------------  282
ident    |                      |
Sbjct LIVFYGsfletvknarrwtrrtviDVAKGevrkgeeffvvdpvdekrnvaanlsldnlar  237
```

```
DSSP  ------------------------------------------------------------
Query ------------------------------------------------------------  282
ident
Sbjct fvhlcrefmeapslgffkpkhpleieperlrkiveergtavfavkfrkpdivddnlypql  297
DSSP  hhhhhhhhhlllhhhllllllllllhhhhhhhhhhhlleeeeeeeelllllhhhhhhhh


DSSP  ------------------------------------------------------------
Query ------------------------------------------------------------  282
ident
Sbjct erasrkifeflerenfmplrsafkaseefcyllfecqikeisrvfrrmgpqfedernvkk  357
DSSP  hhhhhhhhhhhhhllllleeeeeeeeellleeeeeeeeellllllleeeeeeeeellhhhhhh


DSSP  ------------------------------------------------------------
Query ------------------------------------------------------------  282
ident
Sbjct flsrnrafrpfiengrwwafemrkfttpeegvrsyasthwhtlgknvgesireyfeiisg  417
DSSP  hhllllllleeelleeeeeeelllllhhhhhhhhhhlhhhllhhhhhhhhhlleeeeh


DSSP  ----------hhhhhhllll
Query ----------varidratkr  292
ident
Sbjct eklfkepvtaelcemmgvkd  437
DSSP  hhhhhlllhhhhhhhhllll
```

No 627: Query=mol1A Sbjct=4x4uF Z-score=3.7

back to top

```
DSSP  lllllllllhhhhhhhhhhhhhhhlLLLHHHHHHHHHHHHHHHHHHLLLlllllllEEEE
Query aapysspptnlrslrdrltqvaerqGVVFGRLQRHVAXIVVAQFAATLTddtgaplLLVK   60
ident                                     |          |
Sbjct -----------kveeilekalelviPDEEEVRKGREAEEELRRRLDELG-------VEYV   42
DSSP  -----------lhhhhhhhhhhlllLLHHHHHHHHHHHHHHHHHHHLLL-------LLEE


DSSP  HHhhhhhhlllllllllLLEEEEEL------LLHHHHHHHHHHHHhhllleeEEELLLE
Query GGsslelrrgipdsrtsKDFATVAR------RDIELIHEQLADAGetgwegftAIFTAPE  114
ident                   |               |   |
Sbjct FV-gsyarntwlkgsleIDVFLLFPeefskeELRERGLEIGKAVL--------DSYEIRY   93
DSSP  EE-hhhhhllllllllleEEEEEEELllllhhHHHHHHHHHHHHHL--------EEEEEEE


DSSP  ELllllllllLEEEEEEEEelleeEEEEEEEEEELLLLllllleeeeelhhhhlllllle
Query EIaapgxpvkPRRFTAKLSyrgraFATVPIEVSSVEAGnadqfdtltsdalglvgvpaav  174
ident                            |
Sbjct AE--------HPYVHGVVK-----GVEVDVVPCYKLKE----------------------  118
DSSP  LL--------LEEEEEEEL-----LEEEEEEEEELLLL----------------------


DSSP  eeeellhhhhhhHHHHHHhlllllllllLLLHhhhhhhhhhhhllhhhhhhhhhhhhhhh
Query avpcxtipwqiaQKLHAVtavleepkanDRAHdlvdlqlleglllldadlxptrsaciaif  234
ident
Sbjct pkniksavdrtpFHHKWL----------EGRI----------------------------  140
DSSP  hhhllllhhhhhHHHHHH----------LLLL----------------------------


DSSP  hhhllllllllllllllhhhHHHHHHLL-LLLL-------------------llllLHHH
Query earaqhpwpprvatlphwplIYAGALEG-LDHL-------------------elarTVDA  274
ident                                 |                        |
Sbjct -------------------kGKENEVRLlKGFLkangiygaeykvrgfsgylcellIVFY  181
DSSP  -------------------lLLHHHHHHhHHHHhhllllllllllllllhhhhhhhHHHH


DSSP  ----------------hhHHHHH--------------hhHHHH-----------------
Query ----------------aaQAVQR--------------fvARID-----------------  287
ident                     |
Sbjct gsfletvknarrwtrrtvIDVAKgevrkgeeffvvdpvdEKRNvaanlsldnlarfvhlc  241
DSSP  llhhhhhhhhlllllleeEELLLleeeellllleeeelleEEEElllllhhhhhhhhhhhh


DSSP  ------------------------------------------------------------
```

```
Sbjct [illegible]   301
DSSP  hhhhhlllhhhhlllllllllhhhhhhhhhhhlleeeeeeeelllllhhhhhhhhhhhhh


DSSP  ------------------------------------------------------------
Query ------------------------------------------------------------  287
ident
Sbjct kifeflerenfmplrsafkaseefcyllfecqikeisrvfrrmgpqfedernvkkflsrn  361
DSSP  hhhhhhhhlllleeeeeeeellleeeeeeeelllllleeeeeeeelllhhhhhhhhhll


DSSP  ------------------------------------------------------------
Query ------------------------------------------------------------  287
ident
Sbjct rafrpfiengrwwafemrkfttpeegvrsyasthwhtlgknvgesireyfeiisgeklfk  421
DSSP  llllleeelleeeeeeellllhhhhhhhhhhhlhhhllhhhhhhhhhlleeeehhhlll


DSSP  ----------hllll
Query ----------ratkr  292
ident
Sbjct epvtaelcemmgvkd  436
DSSP  lllhhhhhhhhllll
```

No 628: Query=mol1A Sbjct=4x4uA Z-score=3.7

back to top

```
DSSP  lllllllllhhhhhhhhhhhhhhlLLLHHHHHHHHHHHHHHHHHHHLLLlllllllEEEE
Query aapysspptnlrslrdrltqvaerqGVVFGRLQRHVAXIVVAQFAATLTddtgaplLLVK   60
ident                                     |          |
Sbjct ----------mkveeilekalelviPDEEEVRKGREAEEELRRRLDELG-------VEYV   43
DSSP  ----------llhhhhhhhhlllllLLHHHHHHHHHHHHHHHHHHHHHL-------LLEE


DSSP  HHhhhhhhhhlllLLLL-----lLLEEEEEL------lLHHHHHHHHHHHHhhllllleeEE
Query GGsslelrrgipDSRT-----sKDFATVAR------rDIELIHEQLADAGetgwegftAI  109
ident                        |               |   |
Sbjct FV------gsyaRNTWlkgsleIDVFLLFPeefskeeLRERGLEIGKAVL--------DS   89
DSSP  EE------llllLLLLllllllEEEEEEELlllllllLHHHHHHHHHHHL--------LL


DSSP  ELLLEEllllllllllEEEEEEEEelleeEEEEEEEEEELLLLlllllleeeeelhhhhll
Query FTAPEEiaapgxpvkpRRFTAKLSyrgraFATVPIEVSSVEAGnadqfdtltsdalglvg  169
ident                                 |
Sbjct YEIRYH----------PYVHGVVK-----GVEVDVVPCYKLKE-----------------  117
DSSP  LEEELL----------LEEEEEEL-----LEEEEEEEEEELLLL-----------------


DSSP  llllleeeeellhhHHHHHHHHhhhhlllllllllLLLHhhhhhhhhhhhllhhhhhhhhhh
Query vpaavavpcxtipWQIAQKLhavtavleepkanDRAHdlvdlqllegllldadlxptrsa  229
ident                   |
Sbjct ---pkniksavdrTPFHHKW------------lEGRI-----------------------  139
DSSP  ---llllllllllHHHHHHH------------hLLLL-----------------------


DSSP  hhhhhhhhlllllllllllllhhhHHHHHHLLL-LLLL--------------------l
Query ciaifearaqhpwpprvatlphwplIYAGALEGL-DHLE--------------------l  268
ident                                  |
Sbjct -------------------------KGKENEVRLlKGFLkangiygaeykvrgfsgylce  174
DSSP  -------------------------LLLHHHHHHhHHHHhlllllllllllllllhhhhh


DSSP  llLHHHH----------------hhHHHH--------------hHHHHH-----------
Query arTVDAA----------------aqAVQR--------------fVARID-----------  287
ident    |                      |
Sbjct llIVFYGsfletvknarrwtrrtviDVAKgevrkgeeffvvdpvDEKRNvaanlsldnla  234
DSSP  hhHHHHLlhhhhhhhhllllllleeeELLLleeeellllleeeellEEEEElllllhhhhh


DSSP  ------------------------------------------------------------
Query ------------------------------------------------------------  287
ident
Sbjct rfvhlcrefmeapslgffkpkhpleieperlrkiveergtavfavkfrkpdivddnlypq  294
DSSP  hhhhhhhhhhhlllhhhhllllllllllhhhhhhhhhhhlleeeeeeeellllllhhhhhhh
```

```
DSSP
Query ------------------------------------------------------------  287
ident
Sbjct lerasrkifeflerenfmplrsafkaseefcyllfecqikeisrvfrrmgpqfedernvk  354
DSSP  hhhhhhhhhhhhhhlllleeeeeeeellleeeeeeeelllllllleeeeeeeelllhhhhh


DSSP  ------------------------------------------------------------
Query ------------------------------------------------------------  287
ident
Sbjct kflsrnrafrpfiengrwwafemrkfttpeegvrsyasthwhtlgknvgesireyfeiis  414
DSSP  hhhhllllllleeelleeeeeeeelllllhhhhhhhhhhlhhhllhhhhhhhhhlleeee


DSSP  ----------------hllll
Query ----------------ratkr  292
ident
Sbjct geklfkepvtaelcemmgvkd  435
DSSP  hhhhlllllhhhhhhhhllll
```

No 629: Query=mol1A Sbjct=1kdhA Z-score=3.7

back to top

```
DSSP  ------------------------------------------------------------
Query ------------------------------------------------------------    0
ident
Sbjct kkisqyacqrrttlnnynqlftdaldilaendelrenegsclafmrassvlkslpfpits   60
DSSP  lllllhhhllllllllllhhhhhhhhhhhhhhhhlllhhhhhhhhhhhhhhhhlllllll


DSSP  ------------------------------------------------------------
Query ------------------------------------------------------------    0
ident
Sbjct mkdtegipclgdkvksiiegiiedgesseakavlnderyksfklftsvfgvglktaekwf  120
DSSP  hhhhlllllllhhhhhhhhhhhhhhlllhhhhhhhhlhhhhhhhhhhllllllhhhhhhh


DSSP  ---llllllllllhhhhhhhhhhhhhhllllhHHHHHHHHHHHHHHHHHL-LLLLLLLLL
Query ---aapysspptnlrslrdrltqvaerqgvvfGRLQRHVAXIVVAQFAAT-LTDDTGAPL   56
ident                                            |               |
Sbjct rmgfrtlskiqsdkslrftqmqkagflyyedlVSCVNRPEAEAVSMLVKEaVVTFLPDAL  180
DSSP  hllllhhhhhhlllllllhhhhhhhhlhhhhhHLLLLHHHHHHHHHHHHHhHHHHLLLLE


DSSP  EEEEHHHHHHHhhllllllLLLLEEEEEL----------lLHHHHHHHHHHHHhhllllle
Query LLVKGGSSLELrrgipdsrTSKDFATVAR----------rDIELIHEQLADAGetgwegf  106
ident     ||             |  |                             |
Sbjct VTMTGGFRRGK-------mTGHDVDFLITspeatedeeqqLLHKVTDFWKQQG-------  226
DSSP  EEEELLHHHLL-------lLLLEEEEEEEellllhhhhhhHHHHHHHHHHLLL-------


DSSP  eeEELLLEELLLLL---------LLLLLEEEEEEEEE--------LLEEEEEEEEEEEEL
Query taIFTAPEEIAAPG---------XPVKPRRFTAKLSY--------RGRAFATVPIEVSSV  149
ident                                  ||           |
Sbjct --LLLYCDILESTFekfkqpsrlDHFQKCFLILKLDHgrvhseksEGKGWKAIRVDLVMC  284
DSSP  --LEEEEELLLLLLlllllllllLLLLEEEEEEEEEHhhllllllLLLLEEEEEEEEEEE


DSSP  -LLLLlllleeeeelhhhhlllllleeeeellhHHHHhhhhhhhhhlllllllllllHHH
Query -EAGNadqfdtltsdalglvgvpaavavpcxtiPWQIaqklhavtavleepkandraHDL  208
ident
Sbjct pYDRR---------------------------aFALL----------------gwtgSRQ  301
DSSP  eLLLH---------------------------hHHHH----------------hhllLHH


DSSP  HHHH--------------hhhhhllhhhhhhhHHHHHhhhhhhllllLLLLllllllhhh
Query VDLQ--------------llegllldadlxptRSACIaifearaqhpWPPRvatlphwpl  254
ident
Sbjct FERDlrryatherkmmldnhalydrtkrvfleAESEE-------eifAHLG---------  345
DSSP  HHHHhhhhhhhhhhleeelllleeellleeelLLLHH-------hhhHHHL---------


DSSP  hhhhhhlllllllllllhhhhhhhhhHHHHhhhhhllll
Query iyagalegldhlelartvdaaaqavQRFVaridratkr  292
```

```
DSSP                                LLLL  lhhhlll
```

No 630: Query=mol1A Sbjct=5jnbA Z-score=3.6

back to top

```
DSSP  lllllllllhhhhhhhhhhhhhhLLLL-hhHHHHHHHHHHHHHHHHH-LLLLllLLLLLEE
Query aapyssppptnlrslrdrltqvaerQGVV-fgRLQRHVAXIVVAQFAA-TLTDdtGAPLLL   58
ident                                                           |
Sbjct ------------gamdvlsekiwdYHNKvsqTDEMLQRKLHLRDMLYtAISPvfPLSGLY   48
DSSP  ------------llhhhhhhhhhHHHHhllLHHHHHHHHHHHHHHHHhHHLLlLLLLEEE


DSSP  EEHHHHHHhhhllllLLLL-LLEEEEEEL-------------lLHHHHHHHHhHHHHhlll
Query VKGGSSLElrrgipdSRTS-KDFATVAR-------------rDIELIHEQLaDAGEtgwe  104
ident | | |                |                       ||   |
Sbjct VVGSSLNG-------FGNNsSDMDLCLMitnkdldqkndavvVLNLILSTL-QYEK----   96
DSSP  EEHHHLLL-------LLLLlLLEEEEEEllllllllllhhhhHHHHHHHHHH-LLLL----


DSSP  leEEEEELLLEELLllllllLLEEEEEEEeelleEEEE-EEEEEEELLllllllleeeeel
Query gfTAIFTAPEEIAapgxpvKPRRFTAKLsyrgrAFAT-VPIEVSSVEagnadqfdtltsd  163
ident                     |            |
Sbjct --FVESQKLILAK------VPILRINFA-----APFDdITVALNANN------------  130
DSSP  --LEEEEEEEELL------LEEEEEEEL-----LLLLlLLEEEEEELL------------


DSSP  hhhhllllllleeeeeLLHHHHHHH------------------hhhhhhhllllllLLLL--
Query alglvgvpaavavpcXTIPWQIA------------------qklhavtavleepKAND--  203
ident
Sbjct -------------svAIRNTHLLcyyssydwrvrplvsvvkewakrkgindankSSFTsy  177
DSSP  -------------lhHHHHHHHHHhhhhhhlllhhhhhhhhhhhhhlllllllLLLLhh


DSSP  LLHHHhhhhhhhhhllhhhhhhhhhhhhhhhhhhlllllllllllllhhhhhhhhhlll
Query RAHDLvdlqllegllldadlxptrsaciaifearaqhpwpprvatlphwpliyagalegl  263
ident
Sbjct SLVLM-------------------------------------------------------  182
DSSP  HHHHH-------------------------------------------------------


DSSP  lllllllLHHHhhHHHHhHHHHHHHL----------------------------------
Query dhlelarTVDAaaQAVQrFVARIDRA----------------------------------  289
ident
Sbjct vihflqcGPTK--VLPN-LQQSYPNRfsnkvdvrtlnvtmaleevaddidqslsekttlg  239
DSSP  hhhhhhhLLLL--LLLL-HHHHLLLLllllllhhhllllllllllllllllllhhhllhh


DSSP  ------------------------------------------------------------
Query ------------------------------------------------------------  289
ident
Sbjct elligfldyyanefnydrdaisirqgrrveraphfwrsqwrcvcieepftahsiydemvf  299
DSSP  hhhhhhhhhhhhlllllleeeellllleeeellllleeeellleeeellllllllllhhhhh


DSSP  ---------------------------lll
Query ---------------------------tkr  292
ident
Sbjct eaikkafreahgelqhnhdldklmecepik  329
DSSP  hhhhhhhhhhhhhhhhhhhllhhhhhlllllll
```

No 631: Query=mol1A Sbjct=4xj3A Z-score=3.6

back to top

```
DSSP  lllllllllhhhhhhhhhhhhhhlLLLHHHHHHHHHHHHHHHHHHHLL---LLLL-----
Query aapyssppptnlrslrdrltqvaerqGVVFGRLQRHVAXIVVAQFAATL---TDDT-----   52
ident
Sbjct ---rmtwnfhqyytnrndglmgklvLTDEEKNNLKALRKIIRLRTRDVfeeAKGIakavk   57
DSSP  ---leeeelhhhhhlllllhhhhhlLLHHHHHHHHHHHHHHHHHHHHHHHhhhHHHHhhhhh
```

```
ident
Sbjct ksaltfeiiqekvsttqikhlsdseqrevakliyemdddardefLGLTPRFWTQ------  111
DSSP  hllllhhhhhhhhhllhhhhllhhhhhhhhhhhhhllhhhhhhhHLLLLEEEEE------


DSSP  hllllLLLL---------------llEEEEEL-----LLHHHHHHHHHHHHHHLLLLE-e
Query rgipdSRTS---------------kdFATVAR-----RDIELIHEQLADAGETGWEGF-t  107
ident                             |            |
Sbjct -----GSFQydtlnrpfqpgqemdidDGTYMPmpifkIGHSLLILLVDASLKSLVAENhg  166
DSSP  -----LLLLllllllllllllleeeeEEEEEEellllLLHHHHHHHHHHHHHHHHHHLll


DSSP  eeeLLLEellllllllllEEEEEEeeellEEEEEEEEEEEELLLLllllleeeeelhhhh
Query aifTAPEeiaapgxpvkpRRFTAKlsyrgRAFATVPIEVSSVEAGnadqfdtltsdalgl  167
ident     |                  |
Sbjct wkfEAKQ-----------TCGRIK---ieAEKTHIDVPMYAIPKD--------------g  198
DSSP  eeeEELL-----------LLEEEE---ehHHLEEEEEELEEEEELL--------------l


DSSP  llllleeeeellhhhhhhHHHHHHhllllllllLLLLhhhhhhhhhhhhllhhhhhhhh
Query vgvpaavavpcxtipwqiaQKLHAVtavleepkaNDRAhdlvdlqlleglllldadlxptr  227
ident                     |
Sbjct senvnlalregdrkwinsdPKIVED---------WFND----------------------  227
DSSP  hhhleeellllllleeellHHHHHH---------HHHH----------------------


DSSP  hhhhhhhhhlllllllllllLLLHHHHHHHHHHLlLLLL-------------llllLHHH
Query saciaifearaqhpwpprvatLPHWPLIYAGALEgLDHL-------------elarTVDA  274
ident                                                         ||
Sbjct -------------------scIRIGKHLRKVCRFmKAWRdaqwdvggpssislmaaTVNI  268
DSSP  -------------------hhHHHLLHHHHHHHHHhHHHHhhhlllllllhhhhhhhHHHH


DSSP  HHHH--------------------------------------------------------
Query AAQA--------------------------------------------------------  278
ident
Sbjct LDSVahdasdlgetmkiiakhlpsefargvespdstdekplfppsykhgpremdimskle  328
DSSP  HHHLllllllhhhhhhhhhhhhhhhhhhlllllllllllllllllhhhllhhhhhhhhhhh


DSSP  ---------------------------hhhhhhhhhllll
Query ---------------------------vqrfvaridratkr  292
ident
Sbjct rlpeilssaesadsksealkkinmafgnrvtnselivlaka  369
DSSP  hhhhhhhhhhllllhhhhhhhhhhhhhllllllhhhleeeel
```

No 632: Query=mol1A Sbjct=4wcwC Z-score=3.6

back to top

```
DSSP  lllllllllhhhhhhhhhhhhhhllllhhHHHHHHHHHHHHHHHHHLLllllllLLEEEE
Query aapysspptnlrslrdrltqvaerqgvvfgRLQRHVAXIVVAQFAATLtddtgaPLLLVK   60
ident                                        | |  ||            |
Sbjct ----------------------------taNREAIDMARVAAGAAAAK------LADDVV   26
DSSP  ----------------------------llLHHHHHHHHHHHHHHHHL------LLEEEE


DSSP  hhhhhhhhlllllllLLLEEEEELLL--HHHHHHHHHHHHHHLlllEEEEEELLlEELLL
Query ggsslelrrgipdsrtSKDFATVARRD--IELIHEQLADAGETGwegFTAIFTApEEIAA  118
ident                                 |                      |
Sbjct -----vidvsgqlvitDCFVIASGSNErqVNAIVDEVEEKMRQA---GYRPARR-EGARE   77
DSSP  -----eeellllllllLEEEEEEELLHhhHHHHHHHHHHHHHHL---LLLLLEE-ELLLL


DSSP  llllLLLEEEEEeeeelleeeeEEEEEEEELLllllllleeeeelhhhhlllllleeeee
Query pgxpVKPRRFTAklsyrgrafaTVPIEVSSVEagnadqfdtltsdalglvgvpaavavpc  178
ident
Sbjct ----GRWTLLDY---------rDIVVHIQHQD----------------------------   96
DSSP  ----LLEEEEEL---------lLEEEEEEELL----------------------------


DSSP  llhhHHHHHHHhhhhlllllllllllhHHHHhhhhhhllhhhhhhhhhhhhhhhhhl
Query xtipWQIAQKLhavtavleepkandrahDLVDlqlleglllldadlxptrsaciaifeara  238
ident        |
Sbjct --drNFAALDR----------------lWGDC----------------------------  110
```

```
DSSP  lllllllllllllhhhhhhhhhllllllllllhhhhhhhhhhhhhhhhhhllll
Query qhpwpprvatlphwpliyagalegldhlelartvdaaaqavqrfvaridratkr  292
ident
Sbjct -----------------------------------------------pvvpvdl  117
DSSP  -----------------------------------------------lllllll
```

No 633: Query=mol1A Sbjct=1px5B Z-score=3.6

back to top

```
DSSP  llllllllllhhhhhhhhhhhhhhllLLHHHHHHHHHHHHHHHHHH-LLLL-LLLLLLEE
Query aapysspptnlrslrdrltqvaerqgVVFGRLQRHVAXIVVAQFAA-TLTD-DTGAPLLL   58
ident                               | |   |   |  |
Sbjct -----melrhtpardldkfiedhllpNTXFRTQVKEAIDIVXRFLKeRCFQgTADPVRVS   55
DSSP  -----llhhhllhhhhhhhhhhhlllLHHHHHHHHHHHHHHHHHHHhHLLLlLLLLLLEE


DSSP  -EEHHhhhhhhhllllllllLLEEEEEL---------lLHHHHHHHHHHHHHHLLLLE--
Query -VKGGsslelrrgipdsrtsKDFATVAR---------rDIELIHEQLADAGETGWEGF--  106
ident  |  |                |                             |
Sbjct kVVKG----gssgkgrgrsdADLVVFLTkltsfedqlrRRGEFIQEIRRQLEACQREQkf  111
DSSP  eEEEE----eellllllleeeEEEEEEELllllllhhhhLHHHHHHHHHHHHHHHHHHLll


DSSP  --eeEELLLEelllllllllleEEEEEE-eelleeEEEEEEEEEELLllllllleeeeel
Query --taIFTAPEeiaapgxpvkprRFTAKL-syrgraFATVPIEVSSVEagnadqfdtltsd  163
ident         |                  |
Sbjct kvtfEVQSPR------------ALSFVLsspqlqqEVEFDVLPAFDA-------------  146
DSSP  lleeEELLLL------------LLEEEEeelllleEEEEEEEEEELL-------------


DSSP  hhhhlllllleeeeeLLHH---------------------------hhhhHHHHHHhlll
Query alglvgvpaavavpcXTIP---------------------------wqiaQKLHAVtavl  196
ident
Sbjct --------------lGQWTpgykpnpeiyvqlikecksrgkegefstcftELQRDF----  188
DSSP  --------------lLLLLllllllhhhhhhhhhhhhhhhlllllhhhhhHHHHHH----


DSSP  lllllLLLLhhhhhhhhhhhhllhhhhhhhhhhhhhhhhhhhlllllllllllllhhHHH
Query eepkaNDRAhdlvdlqllegllldadlxptrsaciaifearaqhpwpprvatlphwpLIY  256
ident
Sbjct -----LRNR-----------------------------------------------pTKL  196
DSSP  -----HHLL-----------------------------------------------lHHH


DSSP  HHHHL-lLLLL----------------llllLHHHHH-----------------------
Query AGALE-gLDHL----------------elarTVDAAA-----------------------  276
ident                                || |
Sbjct KSLIRlvKHWYqtckkthgnklppqyalellTVYAWEqgsrktdfstaqgfqtvlelvlk  256
DSSP  HHHHHhhHHHHhhhhhhhllllllhhhhhhhHHHHHHhhlllllllhhhhhhhhhhhhhl


DSSP  --hhhHHHH-------------------------------hhHHHLLLL-----------
Query --qavQRFV-------------------------------arIDRATKR-----------  292
ident
Sbjct hqklcIFWEayydftnpvvgrcmlqqlkkprpvildpadptgNVGGGDThswqrlaqear  316
DSSP  hhhleELLLlllllllhhhhhhhhhhhlllllleellllllllLLLLLLHhhhhhhhhhh


DSSP  -------------------------
Query -------------------------  292
ident
Sbjct vwlgypccknldgslvgawtmlqki  341
DSSP  hhlllhhhlllllllllllhhhlll
```

No 634: Query=mol1A Sbjct=4x4nF Z-score=3.6

back to top

```
DSSP  llllllllllhhhhhhhhhhhhhhlLLLHHHHHHHHHHHHHHHHHHLLLlllllllEEEE
Query aapysspptnlrslrdrltqvaerqGVVFGRLQRHVAXIVVAQFAATLTddtgaplLLVK   60
```

```
[illegible]

DSSP  HHhhhhhhhllllllllLLEEEEEL------LLHHHHHHHHHHHHhhlllleeEEELLLE
Query GGsslelrrgipdsrtsKDFATVAR------RDIELIHEQLADAGetgwegftAIFTAPE  114
ident                   |               |   |
Sbjct FV-gsyarntwlkgsleIDVFLLFPeefskeELRERGLEIGKAVL--------DSYEIRY   94
DSSP  EE-hhhhhlllllllleEEEEEEELllllhhHHHHHHHHHHHLLL--------LEEEEEE


DSSP  ELlllllllllEEEEEEEEelleeEEEEEEEEEELLLLllllleeeeelhhhhlllllle
Query EIaapgxpvkpRRFTAKLSyrgraFATVPIEVSSVEAGnadqfdtltsdalglvgvpaav  174
ident                            |
Sbjct AE--------hPYVHGVVK-----GVEVDVVPCYKLKE----------------------  119
DSSP  LL--------lEEEEEEEL-----LEEEEEEEEELLLL----------------------


DSSP  eeeellhhhhhhHHHHHHhlllllllllLLLHhhhhhhhhhhhllhhhhhhhhhhhhhhh
Query avpcxtipwqiaQKLHAVtavleepkanDRAHdlvdlqlleglllldadlxptrsaciaif  234
ident
Sbjct pkniksavdrtpFHHKWL----------EGRI----------------------------  141
DSSP  llllllllllhhHHHHHH----------HHHL----------------------------


DSSP  hhhlllllllllllllhhhHHHHHHHLL-LLLL-------------------llllLHHH
Query earaqhpwpprvatlphwplIYAGALEG-LDHL-------------------elarTVDA  274
ident                                 |                        |
Sbjct -------------------kGKENEVRLlKGFLkangiygaeykvrgfsgylcellIVFY  182
DSSP  -------------------lLLHHHHHHhHHHHhhllllllllllllllhhhhhhhHHHH


DSSP  H----------------hhHHHH--------------hhHHHH-----------------
Query A----------------aqAVQR--------------fvARID-----------------  287
ident                     |
Sbjct GsfletvknarrwtrrtviDVAKgevrkgeeffvvdpvdEKRNvaanlsldnlarfvhlc  242
DSSP  LlhhhhhhhhlllllleeeELLLleeeellllleeellllLLLLllllllhhhhhhhhhhh


DSSP  ------------------------------------------------------------
Query ------------------------------------------------------------  287
ident
Sbjct refmeapslgffkpkhpleieperlrkiveergtavfavkfrkpdivddnlypqlerasr  302
DSSP  hhhhhlllhhhhllllllllllhhhhhhhhhhhlleeeeeeeelllllhhhhhhhhhhhhh


DSSP  ------------------------------------------------------------
Query ------------------------------------------------------------  287
ident
Sbjct kifeflerenfmplrsafkaseefcyllfecqikeisrvfrrmgpqfedernvkkflsrn  362
DSSP  hhhhhhhhllllleeeeeeeelllleeeeeeeelllllllleeeeeeeelllhhhhhhhhhll


DSSP  ------------------------------------------------------------
Query ------------------------------------------------------------  287
ident
Sbjct rafrpfiengrwwafemrkfttpeegvrsyasthwhtlgknvgesireyfeiisgeklfk  422
DSSP  lllllleelllleeeeeeelllllhhhhhhhhhhhlhhhllhhhhhhhhhleeeeeehhhhll


DSSP  ----------hllll
Query ----------ratkr  292
ident
Sbjct epvtaelcemmgvkd  437
DSSP  lllhhhhhhhhllll
```

No 635: Query=mol1A Sbjct=4x4sC Z-score=3.6

back to top

```
DSSP  llllllllllhhhhhhhhhhhhhllLLLHHHHHHHHHHHHHHHHHHLLLlllllllEEEE
Query aapysspptnlrslrdrltqvaerqGVVFGRLQRHVAXIVVAQFAATLTddtgaplLLVK   60
ident                                     |          |
Sbjct ----------mkveeilekalelviPDEEEVRKGREAEEELRRRLDELG-------VEYV   43
DSSP  ----------lllhhhhhhhhhhhlLLHHHHHHHHHHHHHHHHHHHHHL-------LLEE
```

```
Query GGSSlelrrgipDSRT-----SKDFATVAR------RDIELIHEQLADAGetgwegftAI  109
ident                        |               |   |
Sbjct FV------gsyaRNTWlkgsleIDVFLLFPeefskeELRERGLEIGKAVL--------DS   89
DSSP  EE------llllLLLLllllleEEEEEEELllllhhHHHHHHHHHHHHHL--------LE


DSSP  ELLLEELllllllllLEEEEEEEEelleeEEEEEEEEEELLLLllllleeeeelhhhhll
Query FTAPEEIaapgxpvkPRRFTAKLSyrgraFATVPIEVSSVEAGnadqfdtltsdalglvg  169
ident                                 |
Sbjct YEIRYAE--------HPYVHGVVK-----GVEVDVVPCYKLKE-----------------  119
DSSP  EELLLLL--------LLLEEEEEL-----LEEEEEEEEELLLL-----------------


DSSP  llleeeeellhhhhhhhHHHHHHhlllllllllLLLHhhhhhhhhhhhllhhhhhhhhhh
Query vpaavavpcxtipwqiaQKLHAVtavleepkanDRAHdlvdlqlleglllddadlxptrsa  229
ident
Sbjct -----pkniksavdrtpFHHKWL----------EGRI-----------------------  141
DSSP  -----llllllllllhhHHHHHH----------HHHH-----------------------


DSSP  hhhhhhhlllllllllllllllhhhHHHHHHLL-LLLL-------------------lll
Query ciaifearaqhpwpprvatlphwplIYAGALEG-LDHL-------------------ela  269
ident                                      |
Sbjct ------------------------kGKENEVRLlKGFLkangiygaeykvrgfsgylcel  177
DSSP  ------------------------lLLHHHHHHhHHHHhlllllllllllllllhhhhhh


DSSP  lLHHHH----------------hhHHHH--------------hhHHHH------------
Query rTVDAA----------------aqAVQR--------------fvARID------------  287
ident   |                      |
Sbjct lIVFYGsfletvknarrwtrrtviDVAKgevrkgeeffvvdpvdEKRNvaanlsldnlar  237
DSSP  hHHHHLlhhhhhhhhhllllleeeEHHHleeeelllleeeelleEEEEllllllhhhhhh


DSSP  ------------------------------------------------------------
Query ------------------------------------------------------------  287
ident
Sbjct fvhlcrefmeapslgffkpkhpleieperlrkiveergtavfavkfrkpdivddnlypql  297
DSSP  hhhhhhhhhhlllhhhhlllllllllhhhhhhhhhhlleeeeeeeeelllllhhhhhhhh


DSSP  ------------------------------------------------------------
Query ------------------------------------------------------------  287
ident
Sbjct erasrkifeflerenfmplrsafkaseefcyllfecqikeisrvfrrmgpqfedernvkk  357
DSSP  hhhhhhhhhhhhhlllleeeeeeeellleeeeeeeelllllllleeeeeeeelllhhhhhh


DSSP  ------------------------------------------------------------
Query ------------------------------------------------------------  287
ident
Sbjct flsrnrafrpfiengrwwafemrkfttpeegvrsyasthwhtlgknvgesireyfeiisg  417
DSSP  hhhllllllleeelleeeeeeelllllhhhhhhhhhhhlhhhllhhhhhhhhhlleeeel


DSSP  ---------------hllll
Query ---------------ratkr  292
ident
Sbjct eklfkepvtaelcemmgvkd  437
DSSP  hhhhllllhhhhhhhhllll
```

No 636: Query=mol1A Sbjct=4yjlC Z-score=3.6

back to top

```
DSSP  llllllllllhhhhhhhhhhhhhhllllhhhhhhhhhhhhhhhhhhllllllllllleeee
Query aapysspptnlrslrdrltqvaerqgvvfgrlqrhvaxivvaqfaatltddtgaplllvk   60
ident
Sbjct ------------------------------------------------------------    0
DSSP  ------------------------------------------------------------


DSSP  hhhhhhhhhlllllllllleeeeeellllhhhhhhhhhhhhhhllllleeeeelllleellll
Query ggsslelrrgipdsrtskdfatvarrdielihеqladagetgwegftaiftapeeiaapg  120
ident
```

```
DSSP  lllleeeeeeeeeelleeeeeeeeeeeeellllllLLLEeeeelhhhhllllllleeeeeelL
Query xpvkprrftaklsyrgrafatvpievssveagnaDQFDtltsdalglvgvpaavavpcxT  180
ident 
Sbjct ----------------------------------HHHH---------------------H    5
DSSP  ----------------------------------LLLL---------------------H


DSSP  HHHHHHHHHHHHHL--LLLL-----------LLLLLLL--HHHHHHHHHHHHL-LHHHHH
Query IPWQIAQKLHAVTA--VLEE-----------PKANDRA--HDLVDLQLLEGLL-LDADLX  224
ident           |                                     |  |
Sbjct HMLHLLEQIRAYCEtcWEWQeahepgmdqdkNPMPAPVehQICPAVCVLMKLSfDEEHRH   65
DSSP  HHHHHHHHHHHHHHhhHHHHhllllllllllLLLLLHHhhLHHHHHHHHHHHLlLHHHHH


DSSP  HHHHHHhhhhhhllllllLLLL--LLLLLhhhhhhhhhlllllllllLLHHHHHHHHHHH
Query PTRSACiaifearaqhpwPPRV--ATLPHwpliyagalegldhlelaRTVDAAAQAVQRF  282
ident 
Sbjct AMNELG-------glqaiAELLqvDCEMY--------------gltnDHYSITLRRYAGM  104
DSSP  HHHHLL-------hhhhhHHHHhhHHHHH--------------llllLHHHHHHHHHHHH


DSSP  HHHHHHLLLL--------------------------------------------------
Query VARIDRATKR--------------------------------------------------  292
ident 
Sbjct ALTNLTFGDVankatlcsmkgcmralvaqlksesedlqqviasvlrnlswradvnskktl  164
DSSP  HHHHHHLLLHhhhhhhhhlhhhhhhhhhhhhlllhhhhhhhhhhhhhhhllllhhhhhhh


DSSP  ------------------------------------------------------------
Query ------------------------------------------------------------  292
ident 
Sbjct revgsvkalmecalevkkestlksvlsalwnlsahctenkadicavdgalaflvgtltyr  224
DSSP  hhllhhhhhhhhhhhlllhhhhhhhhhhhhhhhlllhhhhhhhhhlllhhhhhhhhllll


DSSP  ------------------------------------------------------------
Query ------------------------------------------------------------  292
ident 
Sbjct sqtntlaiiesgggilrnvssliatnedhrqilrennclqtllqhlkshsltivsnacgt  284
DSSP  llllllhhhhhhhhhhhhhhhhhhhlllhhhhhhhhhllhhhhhhhhllllllhhhhhhhhhh


DSSP  ------------------------------------------------------
Query ------------------------------------------------------  292
ident 
Sbjct lwnlsarnpkdqealwdmgavsmlknlihskhkmiamgsaaalrnlmanrpaky  338
DSSP  hhhhllllhhhhhhhhhllhhhhhhlllllllhhhhhhhhhhhhhhhlllllll
```

No 637: Query=mol1A Sbjct=5eogF Z-score=3.5

back to top

```
DSSP  lllllllllhhhhhhhhhhhhhLLLLHHHHHHHHHHHHHHHHHHHHLLLLlllLLLEeeE
Query aapysspptnlrslrdrltqvaerQGVVFGRLQRHVAXIVVAQFAATLTDdtgAPLLlvK   60
ident                                        ||
Sbjct gamdiaaqaklvyhlnkyynekcqARKAAIAKTIREVCKVVSDVLKEVEV-qePRFI-yE   58
DSSP  llhhhhhhhhhhhhhhhhhhhlhhHHHHHHHHHHHHHHHHHHHHHHHHHH-hlLLLL-lL


DSSP  HHHhhhhhhlllllllLLEEEEEE----------------------------------ll
Query GGSslelrrgipdsrtsKDFATVA----------------------------------rr   86
ident |
Sbjct GLE--------vispteFEVVLYLfvddgslpgcklsdgrkrsmlwvefitasgylsark  110
DSSP  LEE--------eeelleEEEEEELlllllllllllllllllllhhhllllllllhhh


DSSP  LHHHHHHHHHHHHHH-lLLLEEEEeLLLEellllllllleEEEEEEEelleEEEEEEEE
Query DIELIHEQLADAGET-gWEGFTAIfTAPEeiaapgxpvkprRFTAKLSyrgrAFATVPIE  145
ident          | |                                            | |
Sbjct IRSRFQTLVAQAVDKcsYRDVVKMvADTS------------EVKLRIR----DRYVVQIT  154
DSSP  HHHHHHHHHHHHHHLLlLLLLEEElLLLL------------LLEEEEL----LLEEEEEE
```

```
ident
Sbjct PAF-----------------------------------------kCTGIWPRSAA-----  168
DSSP  EEE-----------------------------------------eELLLLLLHHH-----


DSSP  hhhhhhhhhhhhllhhhhhhhhhhhhhhhhhhhhlllllllLLLL---------------
Query hdlvdlqllegllldadlxptrsaciaifearaqhpwpprvATLP---------------  250
ident                                          |
Sbjct --------------------------hwplphipwpgpnrvAEVKaegfnllskecdawv  202
DSSP  --------------------------hllllllllllhhhhHHHHhlleeeellllllle


DSSP  -------lHHHH-hHHHH-LLLL------------------lllLLLL------------
Query -------hWPLI-yAGAL-EGLD------------------hleLART------------  271
ident
Sbjct lqfaeaenRLQMggCRKKcLSILktlrdrhlelpgqplnnyhmkTLVSyecekhpresdw  262
DSSP  eelhhhhhHHLLllLHHHhHHHHhhhhhhhlllllllllhhhhhHHHHhhhhhllllhhh


DSSP  -----------------------------------------------hhhhhhhhhhhhh
Query -----------------------------------------------vdaaaqavqrfva  284
ident
Sbjct desclgdrlngillqlisclqcrrcphyflpnldlfqgkphsalenaakqtwrlareilt  322
DSSP  lhhhhhhhhhhhhhhhhhhhhhllllllllllllllllllhhhhhhhhhhhhhhhhhhhh


DSSP  hhhhllll
Query ridratkr  292
ident
Sbjct npkslekl  330
DSSP  lllhhhhl
```

No 638: Query=mol1A Sbjct=1mivA Z-score=3.5

back to top

```
DSSP  llllllllllhhhhhhhhhhhhhhllllhhHHHHHHHHHHHHHHLLLllllllLEEE-
Query aapysspptnlrslrdrltqvaerqgvvfgRLQRHVAXIVVAQFAATLTddtgapLLLV-   59
ident                                           |
Sbjct ------------------------------XKPPFQEALGIIQQLKQHG------YDAYf   24
DSSP  ------------------------------LLHHHHHHHHHHHHHHHHHLL------LLEEe


DSSP  EHHHHHhhhhLLLLLLLLLLEEEEEllLHHHHHHHHHhhhhhlllleeeEELLLEEllll
Query KGGSSLelrrGIPDSRTSKDFATVArrDIELIHEQLAdagetgwegftaIFTAPEEiaap  119
ident  ||                          |
Sbjct VGGAVR---dLLLGRPIGDVDIATS-aLPEDVXAIFP-----------KTIDVGS----   64
DSSP  ELHHHH---hHHHLLLLLLLEEEEL-lLHHHHHHHLL-----------LEEEEEL----


DSSP  lllLLLEEEEEEEeelleeeEEEEEEEEELL----LLLLllleeeeelhhhhllllllee
Query gxpVKPRRFTAKLsyrgrafATVPIEVSSVE----AGNAdqfdtltsdalglvgvpaava  175
ident
Sbjct ---KHGTVVVVHK-------GKAYEVTTFKTdgsvTFVR---------------------   93
DSSP  ---LLLEEEEEEL-------LEEEEEEELEEelleEELL---------------------


DSSP  eeelLHHHHhHHHH----------------------------------hhhHLLLlllLL
Query vpcxTIPWQiAQKL----------------------------------havTAVLeepKA  201
ident                                                            |
Sbjct ---sLEEDL-KRRDftxnaiaxdeygtiidpfggreairrriirtvgeaekRFRE---DA  146
DSSP  ---lHHHHH-LLLLlhhhlleelllllleellllhhhhhhhleellllhhhHHHH---LL


DSSP  LLllhhhhhhhhhhhhllhhhhhhhhhhhhhhhhhhhhllllllllllllHHHHHHHHhl
Query NDrahdlvdlqllegllldadlxptrsaciaifearaqhpwpprvatlphWPLIYAGAle  261
ident
Sbjct LR---------------------------------------------xxrAVRFVSEL--  159
DSSP  HH---------------------------------------------hhhHHHHHHHH--


DSSP  llllllllllhhHHHHHH------------------------------------------
Query gldhlelartvdAAAQAV------------------------------------------  279
ident             | |
Sbjct ----------gfALAPDTeqaivqnapllahisverxtxexekllggpfaaralpllaet  209
```

```
DSSP  ------------------------------------------------------------
Query ------------------------------------------------------------  279
ident
Sbjct glnaylpglagkekqlrlaaayrwpwlaareerwallchalgvqesrpflrawklpnkvv  269
DSSP  lllllllllllhhhhhhhllllhhhlllhhhhhhhhhhlllllhhhhhhhllllhhhh


DSSP  ------------------------------------------------------------
Query ------------------------------------------------------------  279
ident
Sbjct deagailtaladiprpeawtneqlfsagleralsvetvraaftgappgpwheklrrrfas  329
DSSP  hhhhhhhhhhhhlllhhhllhhhhhhhhhhhhhhhhhhhhhhhhllllhhhhhhhhhhll


DSSP  -----------------------------------------------------hhhhhhh
Query -----------------------------------------------------qrfvari  286
ident
Sbjct lpiktkgelavngkdviewvgkpagpwvkealdaiwravvngevenekeriyawlxernr  389
DSSP  llllhhhllllhhhhhllllllllhhhhhhhhhhhhhlllllllhhhhhhhhhhhhl


DSSP  hhllll
Query dratkr  292
ident
Sbjct treknc  395
DSSP  llllll
```

No 639: Query=mol1A Sbjct=2q0cB Z-score=3.5

back to top

```
DSSP  llllllllllhhhhhhhhhhhhhhlLLLHhHHHHHHHHHHHHHHHHLLLllllllEEEE
Query aapysspptnlrslrdrltqvaerqGVVFgRLQRHVAXIVVAQFAATLTddtgaplLLVK   60
ident                                         |    |
Sbjct ------pspavvgrslvnsfkqfvsKDLH-TRHVDATYRLVLDCVAAVD-------MRLY   46
DSSP  ------llhhhhhhhhhhllhhhhhLLLL-LHHHHHHHHHHHHHHHLLLL-------LEEE


DSSP  HHhhhhhhhlllllllLLEEEEE---llLHHHHHHHHHHHHHHHLLlleeEEELLLEell
Query GGsslelrrgipdsrtsKDFATVA---rrDIELIHEQLADAGETGWegftAIFTAPEeia  117
ident                   ||             |   ||
Sbjct TF-gstvvygvhekgsdVDFVVLNkvakgLQADILAKLARVIRQKH--lsWNVEEVR---  100
DSSP  EE-lhhhhhlllllllEEEEEELllllhHHHLLLLLLHHHHHHHL--llLLLLLLL---


DSSP  llllllllEEEEEEEEelleEEEEEEEEEEELLlllllleeeeelhhhhlllllleeee
Query apgxpvkpRRFTAKLSyrgrAFATVPIEVSSVEagnadqfdtltsdalglvgvpaavavp  177
ident              |            |
Sbjct --------PVVRVKGG----GAVDFDITAYRRN---------------------------  121
DSSP  --------LLEEELLL----LLLLEEEEELLLH---------------------------


DSSP  ellhhhhhhhhhhhhhlllllllLLLLhhhhhhhhhhhllhhhhhhhhhhhhhhhhh
Query cxtipwqiaqklhavtavleepkaNDRAhdlvdlqlleglllddadlxptrsaciaifear  237
ident
Sbjct -----------------gvrnsalLRAY--------------------------------  132
DSSP  -----------------hhhhhhhHHHH--------------------------------


DSSP  llllllllllllLHHHhHHHHHL--LLLL-----------------lllllLHHHHHHH
Query aqhpwpprvatlpHWPLiYAGALE--GLDH-----------------lelarTVDAAAQA  278
ident                |                                     |    |
Sbjct -----------feQNPP-CRWLSMsiKRWSkqtglnasviggsitsygfnlmVVYYLLQR  180
DSSP  -----------hhHLHH-HHHHHHhhHHHHhhllllllllllllhhhhhhhHHHHHHHL


DSSP  ------------------------------------------------------------
Query ------------------------------------------------------------  278
ident
Sbjct nhlqfvppstidvsrveplpphlpleepadeglelgtqvldflhfflhefdsdkqvisln  240
DSSP  lllllllhhhllllllllllllllllllllhhhhhhhhhhhhhhhhllllllleellll


DSSP  hHHHHhhHHHLLL-----------------------------------------------
```

```
Sbjct [illegible]
DSSP  lLLLE--EHHHHLllhhhhhhhhhllllllllllllllllllllllllhhhhhhhhhh


DSSP  ------------l
Query ------------r  292
ident
Sbjct lekardtalltiv  311
DSSP  hhhhhhlllllll
```

No 640: Query=mol1A Sbjct=2pbeA Z-score=3.5

back to top

```
DSSP  lllllllllhhhhhhhhhhhhhhllllhhhHHHHHHHHHHHHHHHLLllllllLLEE-E
Query aapyssppptnlrslrdrltqvaerqgvvfgrLQRHVAXIVVAQFAATLtddtgaPLLL-V   59
ident                                      |     ||              |
Sbjct ------------------------------lRSEQEXXDIFLDFALND------ERIRlV   24
DSSP  ------------------------------lLLHHHHHHHHHHHHHHL------LLEEeE


DSSP  EHHhhhhhhhllllllllLLEEEEELL--LHHHhhHHHHHHHHHhlllleeeeELLLE---
Query KGGsslelrrgipdsrtsKDFATVARR--DIELihEQLADAGEtgwegftaiFTAPE---  114
ident                    |                |                  ||
Sbjct TLE------------fqdYDISYFVTDveSFKE-nDQWLEIFG-----krixXQKPEdxe   66
DSSP  EEL------------lllEEEEEEELLhhHHHL-lLHHHHHHL-----leeeEELLLlll


DSSP  ---ELLLlllllLLEEEEEEEEelleEEEEEEEEEEELLllllllleeeeelhhhhllll
Query ---EIAApgxpvKPRRFTAKLSyrgrAFATVPIEVSSVEagnadqfdtltsdalglvgvp  171
ident
Sbjct lfpPELG-----NWFSYIILFE----DGNKLDLTLIPIR---------------eaedyf  102
DSSP  lllLLLL-----LLEEEEEEEL----LLLEEEEEEEEEHH---------------hhhhhh


DSSP  lleeeEELLHHHhhhhhhhhhhhllllllllllllhhhhHHHHHHhhllhhhhhhhhhhh
Query aavavPCXTIPWqiaqklhavtavleepkandrahdlvDLQLLEglllddadlxptrsaci  231
ident                                       | |
Sbjct ankvlLDKDSFI---------------------nykvnDRQYWI----------------  125
DSSP  hlleeEELLLLL---------------------lllllHHHLEE----------------


DSSP  hhhhhhllllllLLLL-----LLLLhhhhhhhhhlllllllllLLHHHHHHHHHHHHHH--
Query aifearaqhpwpPRVA-----TLPHwpliyagalegldhlelaRTVDAAAQAVQRFVA--  284
ident
Sbjct ----krptarefDDCCnefwxVSTY-vvkglarneilfaidhlNEIVRPNLLRXXAWHia  180
DSSP  ----llllhhhhHHHHhhhhhHHHH-hhhhhhlllhhhhhhhhHHLHHHHHHHHHHHHhh


DSSP  ---------------------------------------------------------hhh
Query ---------------------------------------------------------rid  287
ident
Sbjct sqkgysfsxgknykfxkrylsnkeweelxstysvngyqexwkslftcyalfrkyskavse  240
DSSP  hhhlleeellhhhllhhhlllhhhhhhhhlllllllhhhhhhhhhhhhhhhhhhhhhhhh


DSSP  HLLL------------------l
Query RATK------------------r  292
ident
Sbjct GLAYkypdydegitkytegiycs  263
DSSP  HHLLllllhhhhhhhhhhhhhhl
```

No 641: Query=mol1A Sbjct=4s3nA Z-score=3.5

back to top

```
DSSP  lllllllllhhhhhhhhhhhhHHLL-LLHHHHHHHHHHHHHHHHHHHHLLLlllllLLEEE
Query aapyssppptnlrslrdrltqvaERQG-VVFGRLQRHVAXIVVAQFAATLTddtgaPLLLV   59
ident                                      |    |              |
Sbjct ------mdlystpaaaldrfvaRKLQpRKEFVEKARRALGALAAALRERP-----RVLKT   49
DSSP  ------lhhhhllhhhhhhhhhHHLLlLHHHHHHHHHHHHHHHHHHHHHHLLL-----LEEEE
```

```
Query [illegible]
ident          |                               |        |  |
Sbjct V--KGGSsGRGTALkggcdSELVIFLDCfksyvdqraRRAEILSEMRASLESWWQNPvpg  107
DSSP  E--EEHHhHHLLLLlllleeEEEEEEELLlllllhhhhLLHHHHHHHHHHHHHHLLLLlll


DSSP  ---eeeeLLLEellllllllllEEEEEEE-eelleeeEEEEEEEEELLLLLllllleeeee
Query ---taifTAPEeiaapgxpvkpRRFTAKL-syrgrafATVPIEVSSVEAGNadqfdtlts  162
ident                             |          |         |
Sbjct lrltfpeQSVP-----------GALQFRLtsvdledwMDVSLVPAFNVLGQ---------  147
DSSP  eeeelllLLLL-----------LLEEEEEeellllleEEEEEEEELLLLLL---------


DSSP  lhhhhllllllleeeeellhhhhhhhhhhhhhllllllllllllhhhhhhhhhhhllhhh
Query dalglvgvpaavavpcxtipwqiaqklhavtavleepkandrahdlvdlqlleglllldad  222
ident
Sbjct ------------------------------------------------------------  147
DSSP  ------------------------------------------------------------


DSSP  hhHHHHhhHHHHHHHHLL-----------------------------llllllllllllhh
Query lxPTRSacIAIFEARAQ-----------------------------hpwpprvatlphwp  253
ident
Sbjct --VKPK--PQVYSTLLNsgcqggehaacftelrrnfvnirpaklknlillvkhwyhqvcl  203
DSSP  --LLLL--LHHHHHHHHllllllllhhhhhhhhhhhhhhlllhhhhhhhhhhhhhhhhhhh


DSSP  hhhhhhhllllllLLLL-------------------------lhHHHHhhhHHHHhhHHH
Query liyagalegldhlELAR-------------------------tvDAAAqavQRFVarIDR  288
ident                                                       |
Sbjct qetlppvyalellTIFAweqgckkdafslaeglrtvlgliqqhqHLCV---FWTV-nYGF  259
DSSP  llllllhhhhhhhHHHHhhhhllllllllhhhhhhhhhhhhhhlhhHLEE---LLLL-lLLL


DSSP  L-----------------------------------------------------------
Query A-----------------------------------------------------------  289
ident
Sbjct Edpavgqflqrqlkrprpvildpadptwdlgngaawhwdllaqeaascydhpcflrgmgd  319
DSSP  Llhhhhhhhhhhhllllleellllllllllllllllllhhhhhhhhhhhllllhhheellle


DSSP  ---------------------lll
Query ---------------------tkr  292
ident
Sbjct pvqswkgpglpragcsglghpiql  343
DSSP  elllllllllllllllllllllll
```

No 642: Query=mol1A Sbjct=3nmxB Z-score=3.5

back to top

```
DSSP  -LLLL--------------------llllLLHHhHHHHHHHHHHHHLL---------llhH
Query -AAPY--------------------ssppTNLRsLRDRLTQVAERQG---------vvfG   30
ident                                                            |
Sbjct hHHMLhlleqiraycetcwewqeapmpapVEHQ-ICPAVCVLMKLSFdeehrhamnelgG   59
DSSP  lHHHHhhhhhhhhhhhhhhhhhhhlllllllHHHL-HHHHHHHHHHHLLlhhhhhhhhhllH


DSSP  HHHHHHHHHHHHHHHHH--------------------------------llllllllleE
Query RLQRHVAXIVVAQFAA--------------------------------tltddtgapllL   58
ident          |
Sbjct LQAIAELLQVDCEMYGltndhysitlrryagmaltnltfgdvankatlcsmkgcmralvA  119
DSSP  HHHHHHHHHHHHHHHHLllllhhhhhhhhhhhhhhhhhhhhlllhhhhhhhhhlhhhhhhhhH


DSSP  EEH-------HHHHHHHhllllllllllleeeeelllhhhhhhhhhhhhhhllllleeeeel
Query VKG-------GSSLELRrgipdsrtskdfatvarrdieliheqladagetgwegftaift  111
ident
Sbjct QLKsesedlqQVIASVL-------------------------------------------  136
DSSP  HHHlllhhhhHHHHHHH-------------------------------------------


DSSP  lleellllllllllleeeeeeeeeelleeeeeeeeeeeeelllllLLLEeeeelhhhhllll
Query apeeiaapgxpvkprrftaklsyrgrafatvpievssveagnaDQFDtltsdalglvgvp  171
ident
```

```
DSSP  lleeeeelLHHHHHHHHHHHHhllllllllllLLHHHHHHHHHHHHHL--LHHH-HHHHHH
Query aavavpcxTIPWQIAQKLHAVtavleepkandRAHDLVDLQLLEGLL--LDAD-LXPTRS  228
ident                     |               |         |
Sbjct --------GSVKALMECALEV----------kKESTLKSVLSALWNLsaHCTEnKADICA  196
DSSP  --------LHHHHHHHHHLLL----------lLHHHHHHHHHHHHHHhlLLHHhHHHHHH


DSSP  hhHHHHhhhllllLLLL--LLLLllHHHHhhhhhllllllllllHHHHHHHHHHHHHHH
Query acIAIFearaqhpWPPR--VATLphWPLIyagalegldhlelartVDAAAQAVQRFVARI  286
ident                                                |
Sbjct --VDGA------lAFLVgtLTYR--SQTN----------------TLAIIESGGGILRNV  230
DSSP  --LLLH------hHHHHhhHHLL--LLLL----------------LLHHHHHHHHHHHHH


DSSP  HHLLLL------------------------------------------------------
Query DRATKR------------------------------------------------------  292
ident
Sbjct SSLIATnedhrqilrennclqtllqhlkshsltivsnacgtlwnlsarnpkdqealwdmg  290
DSSP  HHHHLLlhhhhhhhhhllhhhhhhhhlllllhhhhhhhhhhhhhhllllhhhhhhhhhll


DSSP  -----------------------------------
Query -----------------------------------  292
ident
Sbjct avsmlknlihskhkmiamgsaaalrnlmanrpaky  325
DSSP  hhhhhhllllllhhhhhhhhhhhhhhhhhhlllll
```

No 643: Query=mol1A Sbjct=3nmzA Z-score=3.5

back to top

```
DSSP  ------------------------------------------------------------
Query ------------------------------------------------------------    0
ident
Sbjct dkddmsrtllamsssqdscismrqsgclplliqllhgndkdsvlsrgskeararasaalh   60
DSSP  llhhhhhhhhhhhllllhhhhhhhhllhhhhhhhhlllllllllllllhhhhhhhhhhhh


DSSP  ------------------------------------------------------------
Query ------------------------------------------------------------    0
ident
Sbjct niihsqpddkrgrreirvlhlleqiraycetcwewqeahepgmdqdknpapvehqicpav  120
DSSP  hhhhhllllhhhhhhhhhhhhhhhhhhhhhhhhhhhllllllllllllllllllllhhhh


DSSP  ------------------------------------------------------------
Query ------------------------------------------------------------    0
ident
Sbjct cvlmklsfdeehrhamnelgglqaiaellqvdcemygltndhysitlrryagmaltnltf  180
DSSP  hhhhhhlllhhhhhhhhhllhhhhhhhhhhhhhhhhhhlllllhhhhhhhhhhhhhhhhl


DSSP  --------------LLLL---llLLLLHHHHHHHHHHHHHHLL-----------llhHHH
Query --------------AAPY---ssPPTNLRSLRDRLTQVAERQG-----------vvfGRL   32
ident
Sbjct gdvankatlcsmkgCMRAlvaqlKSESEDLQQVIASVLRNLSWradvnskktlrevgSVK  240
DSSP  llhhhhhhhhhlhhHHHHhhhhhHLLLHHHHHHHHHHHHHHLLlllhhhhhhhhhllHHH


DSSP  HHHHHHHH---------------------hhhhhhllllllllllleEEEH----------
Query QRHVAXIV---------------------vaqfaatltddtgapllLVKG----------   61
ident
Sbjct ALMECALEvkkestlksvlsalwnlsahctenkadicavdgalaflVGTLtyrsqtntla  300
DSSP  HHHHHHLLlllhhhhhhhhhhhhhhhhlhhhhhhhhhhlllhhhhhHHHLllllllllh


DSSP  ---HHHHHHHHLllllllllleeeeelllhhhhhhhhhhhhhhlllleeeeellleelll
Query ---GSSLELRRGipdsrtskdfatvarrdieliheqladagetgwegftaiftapeeiaa  118
ident         ||
Sbjct iieSGGGILRNV------------------------------------------------  312
DSSP  hhhHHHHHHHHH------------------------------------------------
```

```
ident
Sbjct -------------------------ssliatnedhrQILR------------------eN  329
DSSP  -------------------------hhhhlllhhhhHHHH------------------hL


DSSP  LLHhHHHHHHHHHhhlllllllllLLLH-HHHHHHHHHHHL--LHHHHHHHHHHHhhhhh
Query XTIpWQIAQKLHAvtavleepkanDRAH-DLVDLQLLEGLL--LDADLXPTRSACiaife  235
ident         | |                         |  |      |
Sbjct NCL-QTLLQHLKS-----------HSLTiVSNACGTLWNLSarNPKDQEALWDMG-----  372
DSSP  LHH-HHHHHHLLL-----------LLHHhHHHHHHHHHHHHllLHHHHHHHHHHL-----


DSSP  hhllllLLLL--LLLLllhhhhhhhhhlllllllllllHHHHHHHHHHHHHHHHHLLLL-
Query araqhpWPPR--VATLphwpliyagalegldhlelartVDAAAQAVQRFVARIDRATKR-  292
ident                                           |
Sbjct ----avSMLKnlIHSK----------------------HKMIAMGSAAALRNLMANRPAk  406
DSSP  ----hhHHHHllLLLL----------------------LHHHHHHHHHHHHHHHHLLLLl
```

No 644: Query=mol1A Sbjct=4atbD Z-score=3.4

back to top

```
DSSP  llllllllllhhhhhhhhhhhhhhLLLLHHHHHHHHHHHHHHHHHHLLLLllLLLLE-EE
Query aapysspptnlrslrdrltqvaerQGVVFGRLQRHVAXIVVAQFAATLTDdtGAPLL-LV   59
ident                         |                        |      |  |
Sbjct -----ifvnddrhvmakhssvyptQEELEAVQNMVSHTERALKAVSDWID--EQRTLrGV   53
DSSP  -----llhhhhhhhhhhhhhhlllHHHHHHHHHHHHHHHHHHHHHHHHHH--LLLLEeEE


DSSP  EHHhhhhhhhlllLLLL-----lLLEEEEELL-LHHHHHHHHHHHHHHL-LLLE--eEEE
Query KGGsslelrrgipDSRT-----sKDFATVARR-DIELIHEQLADAGETG-WEGF--tAIF  110
ident                                           ||
Sbjct MRV------glvaKGLLlkgdldLELVLLCKEkPTTALLDKVADNLAIQlTTVTedkYEI  107
DSSP  EEL------hhhhHLLLllllleEEEEEEEELLlLLHHHHHHHHHHHHHhHHLLlllLEE


DSSP  LLLEELlllllllllEEEEEEeeelleeEEEEEEEEEELLLLllllleeeeelhhhhlll
Query TAPEEIaapgxpvkpRRFTAKlsyrgraFATVPIEVSSVEAGnadqfdtltsdalglvgv  170
ident                     |            |   |
Sbjct LQSVDD---------AAIVIK--ntkepPLSLTIHLTSPVVR-----------eemekva  145
DSSP  EEELLL---------LEEEEE--lllllLLEEEEEEELHHHH-----------hhhhhll


DSSP  llleeeeellhhhhhhHHHHHHhllllllllLLLLhhhhhhhhhhhhllhhhhhhhhhhh
Query paavavpcxtipwqiaQKLHAVtavleepkaNDRAhdlvdlqlleglllddadlxptrsac  230
ident                                  ||
Sbjct gpdvldrqkclaalasLRHAKW--------fQARA-------------------------  172
DSSP  lllllhhhhhhhhhhhHHHHHH--------hHHHL-------------------------


DSSP  hhhhhhhlllllllllllllLHHHhHHHHHL-lLLLL-------------llllLHHHHH
Query iaifearaqhpwpprvatlpHWPLiYAGALE-gLDHL-------------elarTVDAAA  276
ident                                   |
Sbjct -------------------nGLKS-CVIVIRvlRDLCtrvptwgplrgwplellCEKSIG  212
DSSP  -------------------lLLLL-HHHHHHhhHHHHhhlhhhhhhllhhhhhhHHHHHH


DSSP  H---------------------------hhHHHHhhHHHL--------------------
Query Q---------------------------avQRFVarIDRA--------------------  289
ident                                        |
Sbjct TanrpmgagealrrvleclasgivmpdgsgIYDP-cEKEAtdaighldrqqreditqsaq  271
DSSP  LllllllhhhhhhhhhhhhhllllllllllLLLL-lLLLLlllllllhhhhhhhhhhhhh


DSSP  --------------------------------lll
Query --------------------------------tkr  292
ident
Sbjct halrlaafgqlhkvlgmdplpsdytvqippsttya  306
DSSP  hhhhhhhlllhhhhlllllllllllllllllllll
```

No 645: Query=mol1A Sbjct=4x4uE Z-score=3.4

```
DSSP  [illegible]
Query aapyssspptnlrslrdrltqvaerqGVVFGRLQRHVAXIVVAQFAATLTddtgaplLLVK   60
ident                                     |          |
Sbjct ----------mkveeilekalelviPDEEEVRKGREAEEELRRRLDELG-------VEYV   43
DSSP  ----------lhhhhhhhhhhhhllLLHHHHHHHHHHHHHHHHHHHHHHLL-------LLEE


DSSP  HHhhhhhhhlllLLLL-----lLLEEEEEL------LLHHHHHHHHHHHHhhllllleeEE
Query GGsslelrrgipDSRT-----sKDFATVAR------RDIELIHEQLADAGetgwegftAI  109
ident                        |               |   |
Sbjct FV------gsyaRNTWlkgsleIDVFLLFPeefskeELRERGLEIGKAVL--------DS   89
DSSP  EE------hhhhHLLLllllleEEEEEEELllllhhHHHHHHHHHHHHHL--------EE


DSSP  ELLLEELlllllllllEEEEEEEEelleeEEEEEEEEEELLLLllllleeeeelhhhhll
Query FTAPEEIaapgxpvkpRRFTAKLSyrgraFATVPIEVSSVEAGnadqfdtltsdalglvg  169
ident                                 |
Sbjct YEIRYAE--------hPYVHGVVK-----GVEVDVVPCYKLKE-----------------  119
DSSP  EEEEELL--------lEEEEEEEL-----LEEEEEEEEELLLL-----------------


DSSP  lllleeeeellhhHHHHHHHhhhhlllllllllLLLHhhhhhhhhhhhllhhhhhhhhhh
Query vpaavavpcxtipWQIAQKLhavtavleepkanDRAHdlvdlqllegllldadlxptrsa  229
ident                   |
Sbjct ---pkniksavdrTPFHHKW------------lEGRI-----------------------  141
DSSP  ---hhhllllhhhHHHHHHH------------hHHHL-----------------------


DSSP  hhhhhhhhlllllllllllllhhhHHHHHHHLLL-LLLL--------------------l
Query ciaifearaqhpwpprvatlphwplIYAGALEGL-DHLE--------------------l  268
ident                                  |
Sbjct -------------------------KGKENEVRLlKGFLkangiygaeykvrgfsgylce  176
DSSP  -------------------------LLLHHHHHHhHHHHhhllllllllllllllhhhhh


DSSP  llLHHHH----------------hhHHHH---------------hhHHHH-----------
Query arTVDAA----------------aqAVQR---------------fvARID-----------  287
ident    |                      |
Sbjct llIVFYGsfletvknarrwtrrtviDVAKgevrkgeeffvvdpvdEKRNvaanlsldnla  236
DSSP  hhHHHHLlhhhhhhhhllllleeeELLLLleeeellllleeeelleEEEElllllhhhhh


DSSP  ------------------------------------------------------------
Query ------------------------------------------------------------  287
ident
Sbjct rfvhlcrefmeapslgffkpkhpleieperlrkiveergtavfavkfrkpdivddnlypq  296
DSSP  hhhhhhhhhhhlllhhhhlllllllllhhhhhhhhhhlleeeeeeeeelllllhhhhhhh


DSSP  ------------------------------------------------------------
Query ------------------------------------------------------------  287
ident
Sbjct lerasrkifeflerenfmplrsafkaseefcyllfecqikeisrvfrrmgpqfedernvk  356
DSSP  hhhhhhhhhhhhhhllllleeeeeeeellleeeeeeeeelllllllleeeeeeeellhhhhh


DSSP  ------------------------------------------------------------
Query ------------------------------------------------------------  287
ident
Sbjct kflsrnrafrpfiengrwwafemrkfttpeegvrsyasthwhtlgknvgesireyfeiis  416
DSSP  hhhhlllllllleeelleeeeeeelllllhhhhhhhhhhhlhhhllhhhhhhhhhlleeel


DSSP  ----------------hllll
Query ----------------ratkr  292
ident
Sbjct geklfkepvtaelcemmgvkd  437
DSSP  hhhhllllllhhhhhhhhllll
```

No 646: Query=mol1A Sbjct=4x4tE Z-score=3.4

back to top

```
DSSP  lllllllllhhhhhhhhhhhhhhlLLLHHHHHHHHHHHHHHHHHHHLLLlllllllEEEE
Query aapyssspptnlrslrdrltqvaerqGVVFGRLQRHVAXIVVAQFAATLTddtgaplLLVK   60
```

```
DSSP  [illegible]

DSSP  HHhhhhhhhlllLLLL-----lLLEEEEEL------LLHHHHHHHHHHHHhhlllleeEE
Query GGsslelrrgipDSRT-----sKDFATVAR------RDIELIHEQLADAGetgwegftAI  109
ident                        |               |   |
Sbjct FV------gsyaRNTWlkgsleIDVFLLFPeefskeELRERGLEIGKAVL--------DS   89
DSSP  EE------hhhhHLLLllllllEEEEEEELllllhhHHHHHHHHHHHHHL--------EE

DSSP  ELLLEELlllllllllEEEEEEEEelleeEEEEEEEEEELLLLllllleeeeelhhhhll
Query FTAPEEIaapgxpvkpRRFTAKLSyrgraFATVPIEVSSVEAGnadqfdtltsdalglvg  169
ident                                 |
Sbjct YEIRYAE--------hPYVHGVVK-----GVEVDVVPCYKLKE-----------------  119
DSSP  EEEEELL--------lEEEEEEEL-----LEEEEEEEEELLLL-----------------

DSSP  lllleeeeellhhhhhhHHHHHHhlllllllllLLLHhhhhhhhhhhhllhhhhhhhhhh
Query vpaavavpcxtipwqiaQKLHAVtavleepkanDRAHdlvdlqllegllldadlxptrsa  229
ident
Sbjct -----pkniksavdrtpFHHKWL----------EGRI-----------------------  141
DSSP  -----hhhlllhhhhhhHHHHHH----------HHHL-----------------------

DSSP  hhhhhhhhllllllllllllllhhhHHHHHHLLL-LLLL--------------------l
Query ciaifearaqhpwpprvatlphwplIYAGALEGL-DHLE--------------------l  268
ident                                  |
Sbjct -------------------------KGKENEVRLlKGFLkangiygaeykvrgfsgylce  176
DSSP  -------------------------LLLHHHHHHhHHHHhhllllllllllllllhhhhh

DSSP  llLHHHH----------------hhHHHH--------------hhHHHH-----------
Query arTVDAA----------------aqAVQR--------------fvARID-----------  287
ident    |                      |
Sbjct llIVFYGsfletvknarrwtrrtviDVAKgevrkgeeffvvdpvdEKRNvaanlsldnla  236
DSSP  hhHHHHLlhhhhhhhhlllllleeeELLLleeeelllleeeelleEEEEllllllhhhhh

DSSP  ------------------------------------------------------------
Query ------------------------------------------------------------  287
ident
Sbjct rfvhlcrefmeapslgffkpkhpleieperlrkiveergtavfavkfrkpdivddnlypq  296
DSSP  hhhhhhhhhhhlllhhhhlllllllllhhhhhhhhhhhlleeeeeeeelllllhhhhhhh

DSSP  ------------------------------------------------------------
Query ------------------------------------------------------------  287
ident
Sbjct lerasrkifeflerenfmplrsafkaseefcyllfecqikeisrvfrrmgpqfedernvk  356
DSSP  hhhhhhhhhhhhhhlllleeeeeeeeellleeeeeeeelllllleeeeeeeelllhhhhh

DSSP  ------------------------------------------------------------
Query ------------------------------------------------------------  287
ident
Sbjct kflsrnrafrpfiengrwwafemrkfttpeegvrsyasthwhtlgknvgesireyfeiis  416
DSSP  hhhhllllllleeelleeeeeeeellllhhhhhhhhhhhlhhhllhhhhhhhhhleeeee

DSSP  ----------------hllll
Query ----------------ratkr  292
ident
Sbjct geklfkepvtaelcemmgvkd  437
DSSP  hhhhllllllhhhhhhhhllll
```

No 647: Query=mol1A Sbjct=4x4nA Z-score=3.4

back to top

```
DSSP  llllllllllhhhhhhhhhhhhhhlLLLHHHHHHHHHHHHHHHHHHLLLlllllllEEEE
Query aapyssppptnlrslrdrltqvaerqGVVFGRLQRHVAXIVVAQFAATLTddtgaplLLVK   60
ident                                     |          |
Sbjct -----------kveeilekalelviPDEEEVRKGREAEEELRRRLDELG-------VEYV   42
DSSP  -----------lhhhhhhhhlllllLLHHHHHHHHHHHHHHHHHHHHHL-------LLEE
```

```
Query GGsslelrrgipdsrtsKDFATVAR------RDIELIHEQLADAGetgwegftAIFTAPE  114
ident                   |               |   |
Sbjct FV-gsyarntwlkgsleIDVFLLFPeefskeeLRERGLEIGKAVL--------DSYEIRY   93
DSSP  EE-hhhhllllllllllEEEEEEELllllllhHHHHHHHHHHHLLL--------LEELLEE


DSSP  ELllllllllEEEEEEEEelleeEEEEEEEEEELLLLlllllleeeeelhhhhllllllle
Query EIaapgxpvkpRRFTAKLSyrgraFATVPIEVSSVEAGnadqfdtltsdalglvgvpaav  174
ident                            |
Sbjct AE--------hPYVHGVVK-----GVEVDVVPCYKLKE----------------------  118
DSSP  LL--------lEEEEEEEL-----LEEEEEEEEELLLL----------------------


DSSP  eeeellhhhhhhHHHHHHhllllllllLLLHhhhhhhhhhhllhhhhhhhhhhhhhh
Query avpcxtipwqiaQKLHAVtavleepkanDRAHdlvdlqlleglllddadlxptrsaciaif  234
ident
Sbjct pkniksavdrtpFHHKWL----------EGRI----------------------------  140
DSSP  llllllhhhlhhHHHHHH----------HHHL----------------------------


DSSP  hhhllllllllllllllhhhHHHHHHHLL-LLLL-------------------llllLHHH
Query earaqhpwpprvatlphwplIYAGALEG-LDHL-------------------elarTVDA  274
ident                                 |                        |
Sbjct -------------------kGKENEVRLlKGFLkangiygaeykvrgfsgylcellIVFY  181
DSSP  -------------------lLLHHHHHHHhHHHHhllllllllllllllhhhhhhhHHHH


DSSP  H----------------hhHHHH--------------hhHHHH-----------------
Query A----------------aqAVQR--------------fvARID-----------------  287
ident                     |
Sbjct GsfletvknarrwtrrtviDVAKgevrkgeeffvvdpvdEKRNvaanlsldnlarfvhlc  241
DSSP  LlhhhhhhhhhllllllleeeELLLleeeellllleeellllLLLLllllllhhhhhhhhhh


DSSP  ------------------------------------------------------------
Query ------------------------------------------------------------  287
ident
Sbjct refmeapslgffkpkhpleieperlrkiveergtavfavkfrkpdivddnlypqlerasr  301
DSSP  hhhhhlllhhhhllllllllhhhhhhhhhhlleeeeeeeellllhhhhhhhhhhhh


DSSP  ------------------------------------------------------------
Query ------------------------------------------------------------  287
ident
Sbjct kifeflerenfmplrsafkaseefcyllfecqikeisrvfrrmgpqfedernvkkflsrn  361
DSSP  hhhhhhhhllllleeeeeeeeellleeeeeeeeellllllleeeeeeeeelllhhhhhhhllll


DSSP  ------------------------------------------------------------
Query ------------------------------------------------------------  287
ident
Sbjct rafrpfiengrwwafemrkfttpeegvrsyasthwhtlgknvgesireyfeiisgeklfk  421
DSSP  llllleeelleeeeeeelllllhhhhhhhhhhhlhhhllhhhhhhhhhlleeeehhhhll


DSSP  ----------hllll
Query ----------ratkr  292
ident
Sbjct epvtaelcemmgvkd  436
DSSP  lllhhhhhhhlllll
```

No 648: Query=mol1A Sbjct=4x4oC Z-score=3.4

back to top

```
DSSP  llllllllllhhhhhhhhhhhhhhlLLLHHHHHHHHHHHHHHHHHHHHLLLlllllllEEEE
Query aapysspptnlrslrdrltqvaerqGVVFGRLQRHVAXIVVAQFAATLTddtgaplLLVK   60
ident                                     |          |
Sbjct -----------kveeilekalelviPDEEEVRKGREAEEELRRRLDELG-------VEYV   42
DSSP  -----------lhhhhhhhhhhhhlLLHHHHHHHHHHHHHHHHHHHHHHHL-------LLEE


DSSP  HHhhhhhhhllllllllLLEEEEEL------LLHHHHHHHHHHHHhhllllleeEEELLLE
Query GGsslelrrgipdsrtsKDFATVAR------RDIELIHEQLADAGetgwegftAIFTAPE  114
ident                   |               |   |
```

```
DSSP  ELlllllllllEEEEEEEEelleeEEEEEEEEEELLLLllllleeeeelhhhhllllllle
Query EIaapgxpvkpRRFTAKLSyrgraFATVPIEVSSVEAGnadqfdtltsdalglvgvpaav  174
ident                            |
Sbjct AE--------hPYVHGVVK-----GVEVDVVPCYKLKE----------------------  118
DSSP  LL--------lEEEEEEEL-----LEEEEEEEEELLLL----------------------


DSSP  eeeellhhhhhhHHHHHHhlllllllllLLLHhhhhhhhhhhhllhhhhhhhhhhhhhhh
Query avpcxtipwqiaQKLHAVtavleepkanDRAHdlvdlqlleglllldadlxptrsaciaif  234
ident
Sbjct pkniksavdrtpFHHKWL----------EGRI----------------------------  140
DSSP  llllllhhhhhhHHHHHH----------HHHL----------------------------


DSSP  hhhllllllllllllllhhhHHHHHHLL-LLLL-------------------llllLHHH
Query earaqhpwpprvatlphwplIYAGALEG-LDHL-------------------elarTVDA  274
ident                                 |                        |
Sbjct -------------------kGKENEVRLlKGFLkangiygaeykvrgfsgylcellIVFY  181
DSSP  -------------------lLLHHHHHHhHHHHhhlllllllllllllllhhhhhhHHHH


DSSP  H----------------hhHHHH--------------hhHHHH-----------------
Query A----------------aqAVQR--------------fvARID-----------------  287
ident                     |
Sbjct GsfletvknarrwtrrtviDVAKgevrkgeeffvvdpvdEKRNvaanlsldnlarfvhlc  241
DSSP  LlhhhhhhhllllllleeeELLLleeeelllleeellllLLLLllllllhhhhhhhhhhh


DSSP  ------------------------------------------------------------
Query ------------------------------------------------------------  287
ident
Sbjct refmeapslgffkpkhpleieperlrkiveergtavfavkfrkpdivddnlypqlerasr  301
DSSP  hhhhlllhhhhllleellllhhhhhhhhhhhhlleeeeeeeeellllhhhhhhhhhhhhh


DSSP  ------------------------------------------------------------
Query ------------------------------------------------------------  287
ident
Sbjct kifeflerenfmplrsafkaseefcyllfecqikeisrvfrrmgpqfedernvkkflsrn  361
DSSP  hhhhhhhhlllleeeeeeeeellleeeeeeeellllllleeeeeeeeelllhhhhhhhhhll


DSSP  ------------------------------------------------------------
Query ------------------------------------------------------------  287
ident
Sbjct rafrpfiengrwwafemrkfttpeegvrsyasthwhtlgknvgesireyfeiisgeklfk  421
DSSP  lllleeellleeeeeeelllllhhhhhhhhhhhlhhhllhhhhhhhhhlleeeelhhhhl


DSSP  ----------------hllll
Query ----------------ratkr  292
ident
Sbjct epvtaelcemmgvkdsnsssv  442
DSSP  lllhhhhhhhhllllleelll
```

No 649: Query=mol1A Sbjct=4x4pA Z-score=3.4

back to top

```
DSSP  llllllllllhhhhhhhhhhhhhhlLLLHHHHHHHHHHHHHHHHHHLLLlllllllEEEE
Query aapysspptnlrslrdrltqvaerqGVVFGRLQRHVAXIVVAQFAATLTddtgaplLLVK   60
ident                                     |          |
Sbjct ----------mkveeilekalelviPDEEEVRKGREAEEELRRRLDELG-------VEYV   43
DSSP  ----------llhhhhhhhhhhhhlLLHHHHHHHHHHHHHHHHHHHHHL-------LLEE


DSSP  HHhhhhhhhllllllllLLEEEEEL------LLHHHHHHHHHHHHhhlllleeEEELLLE
Query GGsslelrrgipdsrtsKDFATVAR------RDIELIHEQLADAGetgwegftAIFTAPE  114
ident                   |               |   |
Sbjct FV-gsyarntwlkgsleIDVFLLFPeefskeELRERGLEIGKAVL--------DSYEIRY   94
DSSP  EE-hhhhllllllllllEEEEEEELllllhhHHHHHHHHHHHHHL--------LEEEEEL
```

```
ident                              |
Sbjct AE--------hPYVHGVVK-----GVEVDVVPCYKLKE----------------------  119
DSSP  LL--------lLEEEEEEL-----LEEEEEEEEELLLL----------------------


DSSP  eeeellhhhhhhHHHHHHhlllllllllLLLHhhhhhhhhhhhllhhhhhhhhhhhhhhh
Query avpcxtipwqiaQKLHAVtavleepkanDRAHdlvdlqllegllldadlxptrsaciaif  234
ident
Sbjct pkniksavdrtpFHHKWL----------EGRI----------------------------  141
DSSP  lllllllllhhhHHHHHH----------HHHL----------------------------


DSSP  hhhllllllllllllllhhhHHHHHHLLL-LLLL--------------------lllLHH
Query earaqhpwpprvatlphwplIYAGALEGL-DHLE--------------------larTVD  273
ident                             |                             |
Sbjct --------------------KGKENEVRLlKGFLkangiygaeykvrgfsgylcellIVF  181
DSSP  --------------------LLLHHHHHHhHHHHhhllllllllllllllhhhhhhhHHH


DSSP  HH----------------hhHHHH--------------hhHHHH----------------
Query AA----------------aqAVQR--------------fvARID----------------  287
ident                      |
Sbjct YGsfletvknarrwtrrtviDVAKgevrkgeeffvvdpvdEKRNvaanlsldnlarfvhl  241
DSSP  HLlhhhhhhhhlllllleeeELLLleeeelllleeellllLLLLllllllhhhhhhhhhh


DSSP  ------------------------------------------------------------
Query ------------------------------------------------------------  287
ident
Sbjct crefmeapslgffkpkhpleieperlrkiveergtavfavkfrkpdivddnlypqleras  301
DSSP  hhhhhhlllhhhhlllllllllhhhhhhhhhhhlleeeeeeeelllllhhhhhhhhhhhh


DSSP  ------------------------------------------------------------
Query ------------------------------------------------------------  287
ident
Sbjct rkifeflerenfmplrsafkaseefcyllfecqikeisrvfrrmgpqfedernvkkflsr  361
DSSP  hhhhhhhhhlllleeeeeeeellleeeeeeeelllllleeeeeeeeelllhhhhhhhlll


DSSP  ------------------------------------------------------------
Query ------------------------------------------------------------  287
ident
Sbjct nrafrpfiengrwwafemrkfttpeegvrsyasthwhtlgknvgesireyfeiisgeklf  421
DSSP  lllllleeelleeeeeeelllllhhhhhhhhhhhhhhhllhhhhhhhhhlleeeelhhhh


DSSP  -----------hllll
Query -----------ratkr  292
ident
Sbjct kepvtaelcemmgvkd  437
DSSP  llllhhhhhhhhllll
```

No 650: Query=mol1A Sbjct=4evfA Z-score=3.3

back to top

```
DSSP  ------------------------------------------------------------
Query ------------------------------------------------------------    0
ident
Sbjct pkvtdianelkqaidakdevqiafiaseysaesrekiakayvasygkelpddikkalkgg   60
DSSP  llhhhhhhhhhhhhhlllhhhhhhhhhlllhhhhhhhhhhhhhhhlllhhhhhhhhllll


DSSP  ------------------------------lllllllllhhhhhhhhhhhhhhhLLLLHH
Query ------------------------------aapysspptnlrslrdrltqvaerQGVVFG   30
ident
Sbjct seesllmdlfsdrhevraqhirdalsgrndhmaffdtvilctpedwhetvaaytRMFKKP  120
DSSP  hhhhhhhhhlllhhhhhhhhhhhhhhhllllhhhhhhhhhllllhhhhhhhhhhHHHLLL


DSSP  HHHHHHH-------------hhHHHHhhHLLLLLLLllleeeehhhhhhhhhllllllll
Query RLQRHVA-------------xiVVAQfaATLTDDTGaplllvkggsslelrrgipdsrts   77
ident
Sbjct LVEDFMKdvgrkedwcllmekwMAHE--RVSRPGSP-------------------edeaq  159
```

```
DSSP  lleeeeelllHHHHHH-----------hhhhhhhhllllleeeeellLEELllllllllLLE
Query kdfatvarrdIELIHE-----------qladagetgwegftaiftaPEEIaapgxpvKPR  126
ident                                                          |
Sbjct rldqafdqknTAYLIDffgtvpsaeyrpiaeafkaqngksieqaiaTIYT-------KTD  212
DSSP  hhhhhhhlllHHHHHHhhhhllhhhhhhhhhhhhhhhllllhhhhhhHHLL-------HHH


DSSP  EeeeeeeelleeeeeeeeEEEElllllllllleeeeelhhhhllllllleeeeELLHHHHHH
Query RftaklsyrgrafatvpiEVSSveagnadqfdtltsdalglvgvpaavavpCXTIPWQIA  186
ident                                                            |
Sbjct Y-----------------YTFY------------------------cahfaLLGMHRLAA  231
DSSP  H-----------------HHHH------------------------hhhhhHHLHHHHHH


DSSP  HHHHHHHLLllllllllLLHHHHHHHH-HHHHLlhhhhhHHHHHHHHhhhhhlllLLLL-
Query QKLHAVTAVleepkandRAHDLVDLQL-LEGLLldadlxPTRSACIAifearaqhPWPP-  244
ident                                            |
Sbjct YLINCACND------kgDEKRMRRITGmMVDKC-----lGAKHAYKI--------YGDMg  272
DSSP  HHHHHHHHL------llLHHHHHHHHHhHHHHL-----lLHHHHHHH--------LLLHh


DSSP  -LLLLlllhhhhhhhhhlllllllllllHHHHHHHHHHHHHHhhhllll
Query -RVATlphwpliyagalegldhlelartVDAAAQAVQRFVARidratkr  292
ident                                 |
Sbjct tDIER---------------------cfDKRMAPILRTLWRV------k  294
DSSP  hHHHH---------------------hlLLLHHHHHHHHHLL------l
```

No 651: Query=mol1A Sbjct=4txaA Z-score=3.3

back to top

```
DSSP  lllllllllhhhhhhhhhhhhhhllllhhhhhhhhhhhhhhhhhhlllllllllleeee
Query aapysspptnlrslrdrltqvaerqgvvfgrlqrhvaxivvaqfaatltddtgaplllvk   60
ident
Sbjct ------------------------------------------------------------    0
DSSP  ------------------------------------------------------------


DSSP  hhhhhhhhhllllllllleeeeelllhhhhhhhhhhhhhhllllleeeeellleellLLL
Query ggsslelrrgipdsrtskdfatvarrdieliheqladagetgwegftaiftapeeiaAPG  120
ident
Sbjct -----------------------------------------------------pqwtDFL    7
DSSP  -----------------------------------------------------llllLLL


DSSP  LLL-----------------------------------llEEEEEeeeelleeeeeeeeee
Query XPV-----------------------------------kpRRFTAklsyrgrafatvpiev  146
ident                                           |
Sbjct SCPictqtfdetirkpislgcghtvckmclnklhrkacpFDQTT---intdiellpvnsa   64
DSSP  LLLllllllllllleeelllllleeehhhhhhhlllllLLLLL---llllllllllhh


DSSP  eellllllllleeeeelhhhhllllllleeeeellhHHHHHHHHHHHHL-lllllllLLLL
Query ssveagnadqfdtltsdalglvgvpaavavpcxtiPWQIAQKLHAVTA-vleepkaNDRA  205
ident                                          ||               ||
Sbjct llqlvglcsgvedtkhyeeakkcveelalylkpvlSRPMQRKLVTLVHcqlveeegRIRA  124
DSSP  hhllllllllhhhhhhhhhhhhhhhhhhhhhhllllLHHHHHHHHHHHLllllllhhhHHHH


DSSP  -HHHHHHHHHHHHL-LHHH-----------------------------------------
Query -HDLVDLQLLEGLL-LDAD-----------------------------------------  222
ident       |
Sbjct mRAARSLGERTVTElILQHqnpqqlssnlwaavrargcqflgparqeealklvllaledg  184
DSSP  hHHHHHHHHHHHHHHhHLLLlllllhhhhhhhhhhlllllllllhhhhhhhhhhhhhhhlll


DSSP  ------------------------------------------------------------
Query ------------------------------------------------------------  222
ident
Sbjct salsrkvlvlfvvqrleprfpqasktsighvvqllyrascfkvtkrdsslmqlkeefrty  244
DSSP  llllhhhhhhhhhhllllllllhhhhhhhhhhhhhllleeeeellleeeeeelhhhllh


DSSP  ---------hhhhhhhhhhhhhhllllllllllllllhhhhhhhhhlLLLLLL------
```

```
Sbjct [illegible]
DSSP  hhhhhhhhhhhhhhhhhlllllhhhhhhhhhlllllhhhhhhhhhhlLLLLHHhhhhhh


DSSP  ------------------------LLLL------------HHHHHHHHHHHHHHHHH-LL
Query ------------------------LART------------VDAAAQAVQRFVARIDR-AT  290
ident                                               ||   |
Sbjct ltialqrtgdpanlnrlrphlellANIDpspdappptweqLENGLVAVRTVVHGLVDyIQ  364
DSSP  hhhhhhlllllhhhhhhhhhhhHLLLllllllllhhhHHHHHHHHHHHHHHHHHHhHH


DSSP  LL-
Query KR-  292
ident
Sbjct NHs  367
DSSP  HLl
```

No 652: Query=mol1A Sbjct=4at9B Z-score=3.3

back to top

```
DSSP  lllllllllhhhhhhhhhhhhhLLLLHHHHHHHHHHHHHHHHHHHLLLLLLL-lLLEE-
Query aapysspptnlrslrdrltqvaerQGVVFGRLQRHVAXIVVAQFAATLTDDTG-aPLLL-   58
ident                         |                        |       |
Sbjct -----ifvnddrhvmakhssvyptQEELEAVQNMVSHTERALKAVSDWIDEQEkgRTLRg   55
DSSP  -----llhhhhhhhhhhhhhhlllHHHHHHHHHHHHHHHHHHHHHHHHHHHHHhlLLEEe


DSSP  EEHHhhhhhhhlllllllllLLEEEEELL-LHHHHHHHHHHHHHHL-LLLE---eeEELLL
Query VKGGsslelrrgipdsrtsKDFATVARR-DIELIHEQLADAGETG-WEGF---taIFTAP  113
ident |                                     ||               |
Sbjct VMRV-glvakglllkgdldLELVLLCKEkPTTALLDKVADNLAIQlTTVTedkyeILQSV  114
DSSP  EEEL-hhhhhlllllllleEEEEEEELLlLLHHHHHHHHHHHHHHhLLLLllleeEEEEL


DSSP  EEllllllllllEEEEEEeeelleeeEEEEEEEEEL-LLLLllllleeeeelhhhhlllll
Query EEiaapgxpvkpRRFTAKlsyrgrafATVPIEVSSV-EAGNadqfdtltsdalglvgvpa  172
ident                  |            |   |
Sbjct DD----------AAIVIK--ntkeppLSLTIHLTSPvVREE-----------meknddppd  151
DSSP  LL----------LEEEEE--elllllLEEEEEEELLhHHHH-----------hhllllll


DSSP  leeeeellhhhhhhHHHHHHhllllllllLLLLhhhhhhhhhhhhllhhhhhhhhhhhh
Query avavpcxtipwqiaQKLHAVtavleepkaNDRAhdlvdlqlleglllldadlxptrsacia  232
ident                                ||
Sbjct vldrqkclaalaslRHAKWF---------QARA----------------------------  175
DSSP  lllhhhhhhhhhhhHHHHHH---------HHHL----------------------------


DSSP  hhhhhlllllllllllllLHHHhHHHHHL-lLLLLL-------------lllLHHHHHH-
Query ifearaqhpwpprvatlpHWPLiYAGALE-gLDHLE-------------larTVDAAAQ-  277
ident                                 |
Sbjct -----------------nGLKS-CVIVIRvlRDLCTrvptwgplrgwplellCEKSIGTa  217
DSSP  -----------------lLLLL-HHHHHHhhHHHHHhllllhhhllhhhhhhHHHHHHLl


DSSP  ----------------------------------hhhhHHHH------------------
Query ----------------------------------avqrFVAR------------------  285
ident
Sbjct nrpmgagealrrvleclasgivmpdgsgiydpcekeatDAIGhldrqqreditqsaqhal  277
DSSP  llllhhhhhhhhhhhhlllllllllllllllllllllLLLLlllhhhhhhhhhhhhhhh


DSSP  ------------------------hhhllll
Query ------------------------idratkr  292
ident
Sbjct rlaafgqlhkvlgmdplpvdytvqippstty  308
DSSP  hhhhlllhhhhllllllllllllllllllll
```

No 653: Query=mol1A Sbjct=4r11C Z-score=3.3

```
DSSP                                                       LLLL   HHLLLLHH
Query ---------------------------------------------AAPY---ssPPTNLR   12
ident                                                          |
Sbjct lslpdlvklmcdhdesvvaravhraymlsredpnffnapgfdhrsFVEAlmaasKSSNVN   60
DSSP  llhhhhhhhhllllhhhhhhhhhhhhhhhhhhlllllllllllhhhHHHHhhhhlLLLLHH

DSSP  HHHHHHHHHHHHHLL---------llhHHHHHHHHHHH--------------------hhh
Query SLRDRLTQVAERQG---------vvfGRLQRHVAXIV--------------------vaq   43
ident   |                       |
Sbjct VRRNAIGALSHMSEqrggpllifrsgGLAEIIRMLYDslesvvhyavttlrnllmhvsds  120
DSSP  HHHHHHHHHHHHHHLlllhhhhhhhllHHHHHHHHHHLllhhhhhhhhhhhhhhhhhlhhh

DSSP  hhhlllllllllleEEEH--------HHHHHHHHLlllllllleeeeeellhhhhhhhh
Query faatltddtgapllLVKG--------GSSLELRRGipdsrtskdfatvarrdieliheql   95
ident                                |
Sbjct raqaralnavealtPHLHktnpkllaQVADGLYFL-------------------------  155
DSSP  hhhhhhllhhhhhhHHHHlllhhhhhHHHHHHHHH-------------------------

DSSP  hhhhhhllllleeeeellleellllllllllleeeeeeeeelleeeeeeeeeeeelllllll
Query adagetgwegftaiftapeeiaapgxpvkprrftaklsyrgrafatvpievssveagnad  155
ident
Sbjct ------------------------------------------------------------  155
DSSP  ------------------------------------------------------------

DSSP  lleeeeelhhhhllllllleeeeeLLHHHHHHHHHHHHHHllllllllLLLHHHHHHHHHH
Query qfdtltsdalglvgvpaavavpcXTIPWQIAQKLHAVTavleepkanDRAHDLVDLQLLE  215
ident                           |    |                |
Sbjct ----------liddapskitflsLLGPQILVSILREYS---------DHRKLIYTVVRCI  196
DSSP  ----------hlllhhhhhhhhhLLHHHHHHHHHHHHLL---------LLHHHHHHHHHH

DSSP  HHL--LHHHHHHHHHHHhhhhhhhlllllLLLLLlllhhhhhhhhhllllllllllLHH
Query GLL--LDADLXPTRSACiaifearaqhpwpPRVATlphwpliyagalegldhlelarTVD  273
ident   |           |                   |
Sbjct RSLsvCPSNKPALISLG-------clpalyVELCT---------------------aKDE  228
DSSP  HHHllLLLHHHHHHHLL-------hhhhhhHHHLL---------------------lLLH

DSSP  HHHHHHHHHHHHHHHLLLL-----------------------------------------
Query AAAQAVQRFVARIDRATKR-----------------------------------------  292
ident     |
Sbjct RSQTAILVAMRNLSDSATNeenltqliiklleiirvandgmtacacgtlsnltcnntrnk  288
DSSP  HHHHHHHHHHHHHHHLLLLLllllhhhhhhhhhhhlllllhhhhhhhhhhhhhhllllhhhh

DSSP  ------------------------------------------------------------
Query ------------------------------------------------------------  292
ident
Sbjct qtvcshggidalvtairrlpeveevtepalcalrhctarhslaeeaqselrfcqafpvil  348
DSSP  hhhhlllhhhhhhhhhhllllhhhhhhhhhhhhhhlllllhhhhhhhhhhlllhhhhh

DSSP  ------------------------------------------------------------
Query ------------------------------------------------------------  292
ident
Sbjct dqletlrtpvikaalgvirnsallqtnlieltqeqtanghtavsltmdilrraitaieen  408
DSSP  hhhhlllhhhhhhhhhhhhhhlllhhhhhhhhhlllllllhhhhhhhhhhhhhhhhlll

DSSP  ------------------------------------------------------------
Query ------------------------------------------------------------  292
ident
Sbjct pdiavdgvpmwgviegavsalhqlanhpavaaaccddigqvgnpecppfldllhrllahp  468
DSSP  llleelleehhhhhhhhhhhhhhhlllhhhhhhhhhllllllllllllhhhhhhhhhllh

DSSP  ------------------------------------------------------------
Query ------------------------------------------------------------  292
ident
Sbjct rlgsmddevlereilgllyqlskrpdgaravestgvsallmesrgsqyksvvtyangvls  528
DSSP  hhhlllllhhhhhhhhhhhhhlllhhhhhhhhhlllhhhhhhhlllllhhhhhhhhhhh

DSSP  --------
```

```
Sbjct [illegible]
DSSP  hhhhhhll
```

No 654: Query=mol1A Sbjct=5jnbC Z-score=3.2

back to top

```
DSSP  llllllllllhhhhhhhhhhhhhhllLLHHHHHHHHHHHHHHHHHHHLLLlllllLLEEEE
Query aapysspptnlrslrdrltqvaerqgVVFGRLQRHVAXIVVAQFAATLTddtgaPLLLVK   60
ident                                                       ||
Sbjct -------gamdvlsekiwdyhnkvsqTDEMLQRKLHLRDMLYTAISPVF-----PLSGLY   48
DSSP  -------llhhhhhhhhhhhhhhhllLHHHHHHHHHHHHHHHHHHHLLLL-----LLLEEE


DSSP  HHhhhhhhhllllLLLL---------lLEEEEEL-------lLHHHHHHHHHHHHHHLll
Query GGsslelrrgipdSRTS---------kDFATVAR-------rDIELIHEQLADAGETGwe  104
ident                            |              |
Sbjct VV-----------GSSLngfgnnssdmDLCLMITnkdldqknDAVVVLNLILSTLQYE--   95
DSSP  EE-----------HHHLllllllllleEEEEELLlllllllHHHHHHHHHHHHHHLLL--


DSSP  LEEEEELLLEELllllllllLEEEEEEEEelLEEE-EEEEEEEEEllllllllleeeeel
Query GFTAIFTAPEEIaapgxpvkPRRFTAKLSyrGRAF-ATVPIEVSSveagnadqfdtltsd  163
ident  |                                   ||
Sbjct KFVESQKLILAK--------VPILRINFA--APFDdITVALNANN---------------  130
DSSP  LLEEEEEEEELL--------LLEEEEEEL--LLLLlLEEEEEEELL---------------


DSSP  hhhhllllleeeeellhhhhhhhhhhhhhlllllllllLLHHHHhhhhhhhhllhhhh
Query alglvgvpaavavpcxtipwqiaqklhavtavleepkandRAHDLVdlqlleglllldadl  223
ident
Sbjct ---------------------------------svairntHLLCYY--------------  143
DSSP  ---------------------------------lhhhhhhHHHHHH--------------


DSSP  hhhhhhhhhhhhhhlllllllllllllhhhHHHHhHLLL-LLLL---------------
Query xptrsaciaifearaqhpwpprvatlphwplIYAGaLEGL-DHLE---------------  267
ident                                 |      |
Sbjct ------------------------------sSYDWrVRPLvSVVKewakrkgiftsyslv  173
DSSP  ------------------------------hLLLLlHHHHhHHHHhhhhhllllllhhhhh


DSSP  --------------------------------llllhhhHHHH-----------------
Query --------------------------------lartvdaAAQA-----------------  278
ident                                        |
Sbjct lmvihflqcgptkvlpnlqqsypnrfsnkvdvrtlnvtmALESlsekttlgelligfldy  233
DSSP  hhhhhhhhhllllllllhhhhlllllllllllhhhlllllLLLLhhhhllhhhhhhhhhhh


DSSP  ---------hhhhhhhhHHLLLL---------------------------------
Query ---------vqrfvariDRATKR---------------------------------  292
ident                   |
Sbjct yanefnydrdaisirqgRRVERAwrcvcieepfkkafreahgelqhnhdldklmec  289
DSSP  hhhllllllleeeellllEEEELLllllleellllhhhhhhhhhhhhhllhhhhlll
```

No 655: Query=mol1A Sbjct=4amsA Z-score=3.2

back to top

```
DSSP  llllllllllhhhhhhhhhhhhhhlllLHHHHHHHHHHHHHHHHHHHhllllllllllleEEE
Query aapysspptnlrslrdrltqvaerqgvVFGRLQRHVAXIVVAQFAatltddtgapllLVK   60
ident
Sbjct -----------shhhhhhslevlfgslIYTYKLEKYVRTKIFPKI-----llipdknRYI   44
DSSP  -----------lllllllllllllllLLLHHHHHHHHHHHLHHHH-----llllllhhHEE


DSSP  HHhhhhhhhLLLLllllLLEEEEEL-----lLHHHHHHHHHHHHHHHLLlleEEEEELLLEE
Query GGsslelrrGIPDsrtsKDFATVAR-----rDIELIHEQLADAGETGWegfTAIFTAPEE  115
ident                   |             |      |             |
Sbjct IK---gsfrRRVPfvtdIDVVNNVYpeisreNIYDEIIKLVNNIQSDP---NIILAYLSC   98
DSSP  EE---hhhhLLLLllleEEEEEELLlllllLHHHHHHHHHHHHHHLLL---LEEEEEEEE
```

```
Query [illegible]
ident
Sbjct GTderfkistgsskelsniqsllpdneknefqlvlqkklfflneliwdhyklrwkpedvl  158
DSSP  EElhhhllllllhhhhhhlllllllhhhhhhhlllllhhhhhhhhhhhhheeeelhhhhh


DSSP  ---------------llllLLLLEEEEEEEEellEEEEEEEEEEEEELLlllllllleeeee
Query ---------------apgxPVKPRRFTAKLSyrgRAFATVPIEVSSVEagnadqfdtlts  162
ident
Sbjct igsmnlannvsvnfretveNNSTILLQYYVK-lgSYPVGIDVVINYQK------------  205
DSSP  hleeeellleeeehhhhhhLLLEEEEEEEEE-llLLEEEEEEEEELLL------------


DSSP  lhhhhllllllleeeeellhhhhhHHHHHHHHLllllllLLLLlhhhhhhhhhhhhllhhh
Query dalglvgvpaavavpcxtipwqiAQKLHAVTAvleepkANDRahdlvdlqllegllldad  222
ident                                |
Sbjct ----------------------iDLTPAYKNA-----aLYQL------------------  220
DSSP  ----------------------lLLHHHHHHH-----hHHHH------------------


DSSP  hhhhhhhhhhhhhhhllllllllllllLHHHHHHH-----hhllLLLL-----------
Query lxptrsaciaifearaqhpwpprvatlpHWPLIYAG-----alegLDHL-----------  266
ident                                  |
Sbjct ---------------qlanysreyyymlFPLRYYFKnnqdisqrlENIIekkyglykqlm  265
DSSP  ---------------hhhhhlllhhhhhHHHHHHLLllhhhhhhhhHHHHhlllhhhhhhh


DSSP  ------------------------------------------------------------
Query ------------------------------------------------------------  266
ident
Sbjct vriddyhtlyksgnlkidmatnivigilrdieklpgfesdtiyqikkvatnnspsikiee  325
DSSP  hhhhhhhhhhhllllhhhhhhhhhhhhhhhhlllllllllhhhhhhhhhlllllhhhhhhh


DSSP  ----------------llllhhhhhhhhhhhhhhhhhhllll
Query ----------------elartvdaaaqavqrfvaridratkr  292
ident
Sbjct wdillkvlyqeintavnnksrkyfyryiamvppqdrsknyis  367
DSSP  hhhhhhhhhhhhhhhhhhhhhhhhhhhhhhllllhhhhllllll
```

No 656: Query=mol1A Sbjct=1axnA Z-score=3.2

back to top

```
DSSP  llllllllllhhhhhhhhhhhhhhhllllhhhhhhhHHHHHHHHHHH-----------llL
Query aapysspptnlrslrdrltqvaerqgvvfgrlqrhVAXIVVAQFAA-----------tlT   49
ident
Sbjct sasiwvghrgtvrdypdfspsvdaeaiqkairgigTDEKMLISILTersnaqrqlivkeY   60
DSSP  llllllllllllllllllhhhhhhhhhhhhllllLLHHHHHHHHLlllhhhhhhhhhhH


DSSP  LLLL-LLLEeeehhhhhhhhhhlllllllllLLEEEeellllhhhhhhhhhhhhhhllllleee
Query DDTG-APLLlvkggsslelrrgipdsrtsKDFATvarrdieliheqladagetgwegfta  108
ident        |
Sbjct QAAYgKELK-----------ddlkgdlsgHFEHL--------------------------   83
DSSP  HHHHlLLHH-----------hhhhhhllhHHHHH--------------------------


DSSP  eellleellllllllllleeeeeeeeeelleeeeeeeeeeeeelllllllllleeeeeelhhhhl
Query iftapeeiaapgxpvkprrftaklsyrgrafatvpievssveagnadqfdtltsdalglv  168
ident
Sbjct ------------------------------------------------------------   83
DSSP  ------------------------------------------------------------


DSSP  lllllleeeeELLHHHHHHHHHHHHHHHHLLlllllllLLLLhhHHHHHHHHHHHLLHHHHHHHHH
Query gvpaavavpCXTIPWQIAQKLHAVTAVleepkaNDRAhdLVDLQLLEGLLLDADLXPTRS  228
ident                  |  |                     |
Sbjct -----mvalVTPPAVFDAKQLKKSMKG------AGTN--EDALIEILTTRTSRQMKDISQ  130
DSSP  -----hhhhHLLHHHHHHHHHHHHHHLL------LLLL--HHHHHHHHHHLLHHHHHHHHH


DSSP  HHHHHHHhhllllllLLLLllllhhhhhhhhhllllllllllllHHHHHHhhHHHHHH---
Query ACIAIFEaraqhpwpPRVAtlphwpliyagalegldhlelartVDAAAQavQRFVAR---  285
ident |
```

```
DSSP  ------------------------------------------------------------
Query ------------------------------------------------------------  285
ident 
Sbjct eslkvdehlakqdaqilykagenrwgtdedkfteilclrsfpqlkltfdeyrnisqkdiv  225
DSSP  llllllhhhhhhhhhhhhhhlllllllhhhhhhhhhhllhhhhhhhhhhhhhhlllhh


DSSP  ------------------------------------------------------------
Query ------------------------------------------------------------  285
ident 
Sbjct dsikgelsghfedlllaivncvrntpaflaerlhralkgigtdeftlnrimvsrseidll  285
DSSP  hhhhhhllhhhhhhhhhhhhhhhhhhhhhhhhhhhhhhlllllhhhhhhhhhhllllhh


DSSP  -------------------------------hhhllll
Query -------------------------------idratkr  292
ident 
Sbjct dirtefkkhygyslysaiksdtsgdyeitllkicggdd  323
DSSP  hhhhhhhhhhlllhhhhhhhhllhhhhhhhhhhhllll
```

No 657: Query=mol1A Sbjct=2nrkA Z-score=3.2

back to top

```
DSSP  lllllllllhhhhhhhhhhhhhHLLLLHHHHHHHHHhhhhhHHHHHlllllllllLEEEE
Query aapysspptnlrslrdrltqvaeRQGVVFGRLQRHVaxivvAQFAAtltddtgapLLLVK   60
ident                                 |         |                |
Sbjct ---------------------ivTEYQPAWVEQFEEeaqalKQILK---------ENCLK   30
DSSP  ---------------------llLLLLLHHHHHHHHhhhhhHHHHH---------HHEEE


DSSP  hhhhhhhhhLLLL----lllllLEEEEElllHHHHHHhHHHHHHHLllleEEEELLLEEL
Query ggsslelrrGIPD----srtskDFATVArrdIELIHEqLADAGETGwegfTAIFTAPEEI  116
ident            |                   ||     |    |
Sbjct --vehigstSVPNlaakpiidfLVIVEE---IEKVDL-LQWEFERI----GYEYXGEFGL   80
DSSP  --eeeelhhHLLLllllllleeeEEEELL---LHHHHH-HHHHHHHL----LLEELLLLL


DSSP  lllllllllEEEEEEEEElLEEEEEEEEEEEElllllllllleeeeelhhhhllllllleee
Query aapgxpvkpRRFTAKLSYrGRAFATVPIEVSSveagnadqfdtltsdalglvgvpaavav  176
ident           |              | |
Sbjct --------sGRRYLRKGP-IKRTHHVHIYQFD---ntqeilrhlafrnylrenpaiatty  128
DSSP  --------lLEEEEEELL-LLLLEEEEEEELL---lhhhhhhhhhhhhhhhlhhhhhhh


DSSP  eellhhhhhhhhhhhhhlllLLLLL--LLLLHhhhhhhhhhhhllhhhhhhhhhhhhhhh
Query pcxtipwqiaqklhavtavlEEPKA--NDRAHdlvdlqllegllldadlxptrsaciaif  234
ident                               |
Sbjct gtlkkqlaqahpdsidkyxkDAFIKkiEKEAL----------------------------  160
DSSP  hhhhhhhhlllllhhhhllHHHHHHhhHHHHH----------------------------


DSSP  hhhlllllllllllllhhhHHHHhhllllllllllhhhhhhhhhhhhhhhhllll
Query earaqhpwpprvatlphwplIYAGalegldhlelartvdaaaqavqrfvaridratkr  292
ident                      |
Sbjct -------------------kKYWE----------------------------------  165
DSSP  -------------------hHHLL----------------------------------
```

No 658: Query=mol1A Sbjct=4ud4A Z-score=3.2

back to top

```
DSSP  lllllllllhhhhhhhhhhhhhhlLLLHHHHHHHHHHHHHHHHHHHLLLlllllLLEEEE
Query aapysspptnlrslrdrltqvaerqGVVFGRLQRHVAXIVVAQFAATLTddtgaPLLLVK   60
ident                                     |                 |
Sbjct --------shkeftkfcyevyneikISDKEFKEKRAALDTLRLCLKRIS-----PDAELV   47
DSSP  --------llhhhhhhhhhhhhhhlLLHHHHHHHHHHHHHHHHHHHHHHL-----LLLEEE


DSSP  HHhhhhhhhllllllllLLEEEEEL--LLHHHHHHHHHHHHHHHLllleEEEELLLEELll
```

```
Sbjct [illegible]
DSSP  EE--lhhhhlllllllEEEEEELLlLLLHHHHHHHHHHHHHHHL----LLEEEEEEEL--


DSSP  lllllLLEEEEEEEEellEEEEEEEEEEEellllllllleeeeelhhhhllllllleeeee
Query pgxpvKPRRFTAKLSyrgRAFATVPIEVSsveagnadqfdtltsdalglvgvpaavavpc  178
ident                    |     |
Sbjct -----RIPIIKLTSD-gfGASFQCDIGFN-------------------------------  122
DSSP  -----LEEEEEEEEL-llLLLLLEEEEEL-------------------------------


DSSP  llhhhhhhHHHHHHhllllllllLLLLhhhhhhhhhhhhllhhhhhhhhhhhhhhhhhhl
Query xtipwqiaQKLHAVtavleepkaNDRAhdlvdlqllegllldadlxptrsaciaifeara  238
ident
Sbjct ----nrlaIHNTLL---------LSSY---------------------------------  136
DSSP  ----lhhhHHHHHH---------HHHH---------------------------------


DSSP  llllllllllllLHHHhHHHHHLL-LLLL-----------------llllLHHHHHH---
Query qhpwpprvatlpHWPLiYAGALEG-LDHL-----------------elarTVDAAAQ---  277
ident
Sbjct ----------tkLDAR-LKPMVLLvKHWAkrkqinspyfgtlssygyvlmVLYYLIHvik  185
DSSP  ----------hhHLLL-HHHHHHHhHHHHhhlllllhhhllllhhhhhhhHHHHHHHlll


DSSP  ------------------------------------------------------------
Query ------------------------------------------------------------  277
ident
Sbjct ppvfpnlllsplkqekivdgfdvgfddkledippsqnysslgsllhgffafyayafepre  245
DSSP  lllllllllllllllleelleellllllhhhllllllllllhhhhhhhhhhhhhlllllll


DSSP  --hhHHHH---hhhhHLLLL----------------------------------------
Query --avQRFV---aridRATKR----------------------------------------  292
ident      |
Sbjct kvvtFRRPdgyltkqEKGWTkdryilaiedpfeishnvgrtvsssglyrirgefmaasrl  305
DSSP  llllLLLLlllllhhHHLLLlllllllllllllllhhhhllhhhhhhhhhhhhhhhhh


DSSP  -------------------
Query -------------------  292
ident
Sbjct lnsrsypipydslfeeapi  324
DSSP  hllllllllhhhhhlllll
```

No 659: Query=mol1A Sbjct=4xj6A Z-score=3.1

back to top

```
DSSP  llllllllllhhhhhhhhhhhhhhllLLHHHHHHHHHHHHHHHHHH-LLLL---------
Query aapysspptnlrslrdrltqvaerqgVVFGRLQRHVAXIVVAQFAA-TLTD---------   50
ident                                         |
Sbjct -----pwdfnnyyshnmdglisklklSKTESDKLKALRQIVRERTRdVFQEarqvaidvr   55
DSSP  -----leelhhhhhlllllhhhhhllLHHHHHHHHHHHHHHHHHHHhHHHHhhhhhhhhh


DSSP  ------------------------------------------llLLLLEEEEHHhhhhhh
Query ------------------------------------------dtGAPLLLVKGGsslelr   68
ident
Sbjct rqaltlesvrlklektnvrylspeeradlarlifemedearddfIKFQPRFWTQ------  109
DSSP  hlllhhhhhhhhlllhhhhllhhhhhhhhhhhhlllhhhhhhhHHLLLEEEEE------


DSSP  hlllLLLL----------lLLEEEEEL---------LLHHHHHHHHHHHHHHL-LLLEeE
Query rgipDSRT----------sKDFATVAR---------RDIELIHEQLADAGETG-WEGFtA  108
ident                     |  |                |              |
Sbjct gsfqYDTLnrpfhpgqemdIDDGTYMPmtvfesepsIGHTLLLLLVDTSLKSLeAENDgW  169
DSSP  hhhlLLLLllllllllleeEEEEEEEEellllllllLLHHHHHHHHHHHHHHhHHLLlE


DSSP  EELLLEellllllllllEEEEEEEEellEEEEEEEEEEEELLLLllllleeeeelhhhhl
Query IFTAPEeiaapgxpvkpRRFTAKLSyrgRAFATVPIEVSSVEAGnadqfdtltsdalglv  168
ident  |                    |     |
Sbjct VFEEKN-----------TCGRIKIY---REKTHIDVPMYAIPKE----------------  199
DSSP  EEEELL-----------LLEEEEEH---HHLEEEEELEEEEELL----------------
```

```
DSSP  [illegible]
Query gvpaavavpcxTIPW--------------qiaqKLHAVtavleepkaNDRAhdlvdlqll  214
ident                                  |
Sbjct -----------VESDkvnlalregvrrwsvsdpKIVED---------WFNE---------  230
DSSP  -----------LLHHhleeellllllleeellhHHHHH---------HHHH---------


DSSP  hhhllhhhhhhhhhhhhhhhhhhllllllllllLLLHhHHHHHHHL-lLLLL-------
Query eglllldadlxptrsaciaifearaqhpwpprvatLPHWpLIYAGALE-gLDHL-------  266
ident
Sbjct ---------------------------------scKRIG-GHLRSVCRfmKAWRdaqwevg  257
DSSP  ---------------------------------hhHHHL-LHHHHHHHhhHHHHhhhllll


DSSP  ------lllLLHHHHHHH------------------------------------------
Query ------elaRTVDAAAQA------------------------------------------  278
ident            |
Sbjct gpssislmtAVVNILDREshngsdltgtmkliarllpeefnrgvespddtdekplfpaes  317
DSSP  lllhhhhhhHHHHHHHHLllllllhhhhhhhhhhhhhhhhhhlllllllllllllllhh


DSSP  ---------------------------------------hhhhhhhhhhllll
Query ---------------------------------------vqrfvaridratkr  292
ident
Sbjct nhnvhhraivetmeglygillaaeqsesreealrkineafgkrvtnallitss  370
DSSP  hllhhhhhhhhhhhhhhhhhhhhhhllllhhhhhhhhhhhhlllllhhhleel
```

No 660: Query=mol1A Sbjct=2zocA Z-score=3.1

back to top

```
DSSP  llllllllllhhhhhhhhhhhhhhhllllhhhhhHHHHHHHHHHH---------hhlLLLL
Query aapyssspptnlrslrdrltqvaerqgvvfgrlqRHVAXIVVAQF---------aatLTDD   51
ident                                     | | |
Sbjct -matkggtvkaasgfnamedaqtlrkamkglgtDEDAIISVLAYrntaqrqeirtaYKST   59
DSSP  -llllllllllllllhhhhhhhhhhhllllllLHHHHHHHHLLllhhhhhhhhhhHHHH


DSSP  LLLLLEeeehhhhhhhhhllllllllLLEEeeelllhhhhhhhhhhhhhhlllleeeeel
Query TGAPLLlvkggsslelrrgipdsrtsKDFAtvarrdieliheqladagetgwegftaift  111
ident  |  |
Sbjct IGRDLI-----------ddlkselsgNFEQ------------------------------   78
DSSP  LLLLHH-----------hhhhhhllhHHHH------------------------------


DSSP  lleellllllllleeeeeeeeelleeeeeeeeeeeellllllllleeeeelhhhhllll
Query apeeiaapgxpvkprrftaklsyrgrafatvpievssveagnadqfdtltsdalglvgvp  171
ident
Sbjct ------------------------------------------------------------   78
DSSP  ------------------------------------------------------------


DSSP  lleeeeELLHHHHHHHHHHHHHHHLLLllllllllllhHHHHHHHHHHHLLHHHHHHHHHHHH
Query aavavpCXTIPWQIAQKLHAVTAVLeepkandrahDLVDLQLLEGLLLDADLXPTRSACI  231
ident                |  |                 |   |
Sbjct -vivgmMTPTVLYDVQELRRAMKGA--------gtDEGCLIEILASRTPEEIRRISQTYQ  129
DSSP  -hhhhhHLLHHHHHHHHHHHHHHLLL--------llLHHHHHHHHHHLLHHHHHHHHHHHH


DSSP  HHHHhhllllLLLL--LLLL----------------------------------------
Query AIFEaraqhpWPPR--VATL----------------------------------------  249
ident
Sbjct QQYG---rslEDDIrsDTSFmfqrvlvslsaggrdegnylddalvrqdaqdlyeagekkw  186
DSSP  HHHL---llhHHHHhhHLLHhhhhhhhhhllllllllllllhhhhhhhhhhhhhhlllll


DSSP  ---------------------------------------------------------llh
Query ---------------------------------------------------------phw  252
ident
Sbjct gtdevkfltvlcsrnrnhllhvfdeykrisqkdieqsiksetsgsfedallaivkcmrnk  246
DSSP  lllhhhhhhhhhllhhhhhhhhhhhhhhhlllhhhhhhhhllhhhhhhhhhhhhhhhhlh


DSSP  hhhhhhHHLLLLLL---------------------------------llllllhhhhhhhh
Query pliyagALEGLDH---------------------------------lelartvdaaaqav  279
```

[illegible]

```
DSSP  hhhhhhhhhllll
Query qrfvaridratkr  292
ident
Sbjct yrkvllvlcggdd  319
DSSP  hhhhhhhhhllll
```

No 661: Query=mol1A Sbjct=4z31B Z-score=3.1

back to top

```
DSSP  lllllllllhhhhhhhhhhhhhhllllhhhhhhhhhhhhhhhhhhlllllllllleeee
Query aapysspptnlrslrdrltqvaerqgvvfgrlqrhvaxivvaqfaatltddtgaplllvk   60
ident
Sbjct ------------------------------------------------------------    0
DSSP  ------------------------------------------------------------


DSSP  hhhhhhhhhllllllllllleeeeelllhhhhhhhhhhhhhhllllleeeeellleellll
Query ggsslelrrgipdsrtskdfatvarrdielihеqladagetgwegftaiftapeeiaapg  120
ident
Sbjct ------------------------------------------------------------    0
DSSP  ------------------------------------------------------------


DSSP  llllleeeeeeeeeelleeeeeeeeeeeellllllllleeeeelhhhhllllllleeeeell
Query xpvkprrftaklsyrgrafatvpievssveagnadqfdtltsdalglvgvpaavavpcxt  180
ident
Sbjct ------------------------------------nlgenkhyevakkcvedlalylka   24
DSSP  ------------------------------------lllllhhhhhhhhhhhhhhlllll


DSSP  hHHHHHHHHHHHHHLlllllLLLL--LLHHHHHHHHHHHHHL-LHHH--------------
Query iPWQIAQKLHAVTAvleepKAND--RAHDLVDLQLLEGLL-LDAD--------------  222
ident        ||                       |
Sbjct lSRPMQRKLVTLVNcqlveEEGRvrAMRAARSLGERTVTElILQHqnpqqlsanlwaavr   84
DSSP  lLHHHHHHHHHHHHLlllllHHHHhhHHHHHHHHHHHHHHHHHhHHHLlllllhhhhhhhhhh


DSSP  ------------------------------------------------------------
Query ------------------------------------------------------------  222
ident
Sbjct argcqflgpamqeealklvllaledgsalsrkvlvlfvvqrleprfpqasktsighvvql  144
DSSP  hlllllllhhhhhhhhhhhhhhhlllllleehhhhhhhhhhhhhllllllllhhhhhhhhhh


DSSP  --------------------------------------hhhhhhhhhhhhhhhllllllll
Query --------------------------------------lxptrsaciaifearaqhpwpp  244
ident
Sbjct lyrascfkvtkrdedsslmqlkeefrsyealrrehdaqivhiameaglrispeqwsslly  204
DSSP  hhhllleeeeeelleeeeeeelhhhllhhhhhhhhhhhhhhhhhhhlllllllhhhhhhhhh


DSSP  lllllllhhhhhhhhhlLLLLLL-----------------------------LLLL----
Query rvatlphwpliyagaleGLDHLE-----------------------------LART----  271
ident
Sbjct gdlahkshmqsiidklqSPESFAksvqeltivlqrtgdpanlnrlrphlellANIDpnpd  264
DSSP  llllllhhhhhhhhhhhlLLLHHHhhhhhhhhhhhhhhllllllhhhhhhhhhhhHLLLllll


DSSP  --------HHHHHHHHHHHHHHHHH-LLLL-
Query --------VDAAAQAVQRFVARIDR-ATKR-  292
ident            |  ||   |
Sbjct avsptweqLENAMVAVKTVVHGLVDfIQNYs  295
DSSP  llllllhhhHHHHHHHHHHHHHHHHHHhHHHLl
```

No 662: Query=mol1A Sbjct=4rwqB Z-score=3.1

back to top

```
Sbjct -----melrhtpardldkfiedhlLPNTCFRTQVKEAIDIVCRFLKeRCFQgTADPVRVs   55
DSSP  -----llhhhlllllhhhhhhhllLLLHHHHHHHHHHHHHHHHHHHhHLLLlLLLLLLEe


DSSP  EEEHHhhhhhhhlllllllLLEEEEEL---------lLHHHHHHHHHHHHHHLLLLE--
Query LVKGGsslelrrgipdsrtsKDFATVAR---------rDIELIHEQLADAGETGWEGF--  106
ident  |  |                |                             |
Sbjct KVVKG-gssgkgttlrgrsdADLVVFLTkltsfedqlrRRGEFIQEIRRQLEACQREQkf  114
DSSP  EEEEL-lllllllllllllLEEEEEELllllllhhhhLHHHHHHHHHHHHHHHHHHHLll


DSSP  --eeEELLleellllllllleEEEEEE-eelleeEEEEEEEEEELLllLLLLleeeeel
Query --taIFTApeeiaapgxpvkprRFTAKL-syrgraFATVPIEVSSVEagNADQfdtltsd  163
ident                            |
Sbjct kvtfEVQS-------prrenprALSFVLsspqlqqEVEFDVLPAFDA--LGQW-------  158
DSSP  lleeEELL-------lllllllLLEEEEellllllEEEEEEEEEELL--LLLL-------


DSSP  hhhhllllleeeeellhhhhhhhhhhhhllllllllllllhhhhhhhhhhhllhhhhH
Query alglvgvpaavavpcxtipwqiaqklhavtavleepkandrahdlvdlqlleglllldadL  223
ident
Sbjct ---------------------------------------------------------tpG  161
DSSP  ---------------------------------------------------------llL


DSSP  HHHhhHHHHHHHHHLLL-------------------------------------------
Query XPTrsACIAIFEARAQH-------------------------------------------  240
ident
Sbjct YKP--NPEIYVQLIKECksrgkegefstcftelqrdflrnrptklkslirlvkhwyqtck  219
DSSP  LLL--LHHHHHHHHHHHhhhllllllhhhhhhhhhhhhllllhhhhhhhhhhhhhhhhhh


DSSP  --------lllllllLLLLHHhhhhhhhlllllllllLHHH------------------
Query --------pwpprvaTLPHWPliyagalegldhlelarTVDA------------------  274
ident
Sbjct kthgnklppqyalelLTVYAW--------------eqgSRKTdfstaqgfqtvlelvlkh  265
DSSP  hhhllllllhhhhhhHHHHHH--------------hllLLLLlllhhhhhhhhhhhhhlh


DSSP  hhhhhHHHHhhHHHL---------------------------------------------
Query aaqavQRFVarIDRA---------------------------------------------  289
ident             |
Sbjct qklciFWEA-yYDFTnpvvgrcmlqqlkkprpvildpadptgnvgggdthswqrlaqear  324
DSSP  hhleeLLLL-lLLLLlhhhhhhhhhhhlllllleellllllllllllllhhhhhhhhhhh


DSSP  -------------------lll
Query -------------------tkr  292
ident
Sbjct vwlgypccknldgslvgawtml  346
DSSP  hhlllhhhhlllllllllllll
```

No 663: Query=mol1A Sbjct=4x4vC Z-score=3.1

back to top

```
DSSP  llllllllllhhhhhhhhhhhhhhlLLLHHHHHHHHHHHHHHHHHHHHHLLLlllllllEEEE
Query aapysspptnlrslrdrltqvaerqGVVFGRLQRHVAXIVVAQFAATLTddtgaplLLVK   60
ident                                     |          |
Sbjct ------------veeilekalelviPDEEEVRKGREAEEELRRRLDELG-------VEYV   41
DSSP  ------------lhhhhhhhhhhhlLLHHHHHHHHHHHHHHHHHHHHHHHLL-------LLEE


DSSP  HHhhhhhhhlllllllLLEEEEEL------LLHHHHHHHHHHHHhhllllleeEEELLLE
Query GGsslelrrgipdsrtsKDFATVAR------RDIELIHEQLADAGetgwegftAIFTAPE  114
ident                   |               |   |
Sbjct FV-gsyarntwlkgsleIDVFLLFPeefskeELRERGLEIGKAVL--------DSYEIRY   92
DSSP  EE-llhhhllllllllleEEEEEEELllllhhHHHHHHHHHHHHHHL--------LEEELLL


DSSP  ELlllllllllEEEEEEEEelleeEEEEEEEEEELLLLllllleeeeelhhhhllllllle
Query EIaapgxpvkpRRFTAKLSyrgraFATVPIEVSSVEAGnadqfdtltsdalglvgvpaav  174
ident                            |
Sbjct AE--------hPYVHGVVK-----GVEVDVVPCYKLKE----------------------  117
```

```
DSSP  eeeellhhhhhhHHHHHHhllllllllLLLHhhhhhhhhhhhllhhhhhhhhhhhhhh
Query avpcxtipwqiaQKLHAVtavleepkanDRAHdlvdlqllegllldadlxptrsaciaif  234
ident
Sbjct pkniksavdrtpFHHKWL----------EGRI----------------------------  139
DSSP  lllllllllhhhHHHHHH----------HHHL----------------------------


DSSP  hhhllllllllllllllhhhHHHHHHLL-LLLL-------------------llllLHHH
Query earaqhpwpprvatlphwplIYAGALEG-LDHL-------------------elarTVDA  274
ident                                 |                        |
Sbjct -------------------kGKENEVRLlKGFLkangiygaeykvrgfsgylcellIVFY  180
DSSP  -------------------lLLHHHHHHHhHHHHhhlllllllllllllhhhhhhhHHHH


DSSP  H----------------hhHHHH--------------hhHHHH-----------------
Query A----------------aqAVQR--------------fvARID-----------------  287
ident                     |
Sbjct GsfletvknarrwtrrtviDVAKgevrkgeeffvvdpvdEKRNvaanlsldnlarfvhlc  240
DSSP  LlhhhhhhhhlllllleeeELLLleeeellllleeeelleEEEEllllllhhhhhhhhhhh


DSSP  ------------------------------------------------------------
Query ------------------------------------------------------------  287
ident
Sbjct refmeapslgffkpkieperlrkiveergtavfavkfrkpdivddnlypqlerasrkife  300
DSSP  hhhhhlllhhhhlllllhhhhhhhhhhhlleeeeeeeelllllhhhhhhhhhhhhhhhhh


DSSP  ------------------------------------------------------------
Query ------------------------------------------------------------  287
ident
Sbjct flerenfmplrsafkaseefcyllfecqikeisrvfrrmgpqfedernvkkflsrnrafr  360
DSSP  hhhhllllleeeeeeeellleeeeeeeellllllleeeeeeeellllhhhhhhhhhllllll


DSSP  ------------------------------------------------------------
Query ------------------------------------------------------------  287
ident
Sbjct pfiengrwwafemrkfttpeegvrsyasthwhtlgknvgesireyfeiisgeklfkepvt  420
DSSP  leeelleeeeeeellllllhhhhhhhhhhhlhhhllhhhhhhhhhlleeeeelhhhhllllh


DSSP  ------hllll
Query ------ratkr  292
ident
Sbjct aelcemmgvkd  431
DSSP  hhhhhhhllll
```

No 664: Query=mol1A Sbjct=3wfqH Z-score=3.0

back to top

```
DSSP  llllllllllhhhhhhhhhhhhhhllllhhhhhhhhhhHHHHHHHHHLLllllLLLEEEE
Query aapysspptnlrslrdrltqvaerqgvvfgrlqrhvaxIVVAQFAATLtddtgAPLLLVK   60
ident                                              |
Sbjct -----------------------------xxxxglnfyLSYFDDVAKV---lpREHYCFI   28
DSSP  -----------------------------lllllllllLLLHHHHHHLL---llLLLLEEE


DSSP  HHHHHHHHHHLLL-llllllLEEEEELlLHHHHHHHHHHHHHHhllllleeeEELLLEElllll
Query GGSSLELRRGIP-dsrtskDFATVARrDIELIHEQLADAGEtgwegftaIFTAPEEiaap  119
ident  |     |                   |        |                  |
Sbjct VGGWVRDRILGEpvgynidVDFLTTA-DPVELAKNFAKRIG--------GHFFVFE----   75
DSSP  ELHHHHHHHHHLLllllleeeEEEEEELL-LHHHHHHHHHHHHHL--------LEEEEEL----


DSSP  llllLLEEEEEEEEeelLEEEEEEEEEEEeLLLLLllllleeeeelhhhhllllllleeeeeel
Query gxpvKPRRFTAKLsyrGRAFATVPIEVSsVEAGNadqfdtltsdalglvgvpaavavpcx  179
ident
Sbjct ----PTIASVVLH---LPPYRYRFDFSPlKGKDL--------------------------  102
DSSP  ----LLEEEEEEE---LLLLEEEEEEEEElLLLLH--------------------------


DSSP  lhHHHHHHHHHhhhhlllllllllllllhHHHH-----------------HHHHHhhllhh
```

```
Sbjct [illegible]
DSSP  -hHHHHHHHH---------------llLLHHhlleeehhhhllllleellLLHHH------


DSSP  hhhhhhhhhhhhhhhhllllllllLLLL-----llhhhhhhhhhLLLLLllllLLHH---
Query dlxptrsaciaifearaqhpwpprVATL-----phwpliyagalEGLDHlelaRTVD---  273
ident                                             |         | |
Sbjct --------------dleqgllrpvSIENlkrdpvrvlrgfriaiEKNLQ----LTEDfye  182
DSSP  --------------hhhhleellllLHHHhhhlllhhhhhhhhhhHLLLE----ELHHhhh


DSSP  ------------------------------------------------------------
Query ------------------------------------------------------------  273
ident
Sbjct fvkedprivlksaverithelfkimkektahkvirelyeygvleaiipeigrlrevkdpl  242
DSSP  hhhhlhhhhhlllhhhhhhhhhhhhhlllhhhhhhhhhhllhhhhhlhhhhllllllllh


DSSP  ------------------------------------------------------------
Query ------------------------------------------------------------  273
ident
Sbjct dehtlktleyleqviedrakylsaellenfgkkrvlgeftdvellkwgalfhdigkpqtf  302
DSSP  hhhhhhhhhhhhhhhhlhhhlllhhhhlllllllllllllhhhhhhhhhhllllllllll


DSSP  ------------------------------------------------------------
Query ------------------------------------------------------------  273
ident
Sbjct afyehdkvgaqivreigerlrwgdeatefvaklvrhhlrpfflreafkkgelkrrgmanf  362
DSSP  llllhhhhhhhhhhhhhhhllllhhhhhhhhhhhhhhhhhhhhhhhhhhlllllhhhhhhh


DSSP  ------------------------------------------------------------
Query ------------------------------------------------------------  273
ident
Sbjct wrecgdiaphlfllsiadamasgdeeedikalmetiaelesfnrnemkxxxxxxxxxxxx  422
DSSP  hhhllllhhhhhhhhhhhhhhllllhhhhhhhhhhhhhhhhhhhhllllllhhhhhhhhhh


DSSP  hhhhhhhhhhhhhhhhllll
Query aaaqavqrfvaridratkr  292
ident
Sbjct xxxxxxxxxxxxxxxxxxx  441
DSSP  hhhhllhhhhhhhhhhhhl
```

No 665: Query=mol1A Sbjct=3m62A Z-score=3.0

back to top

```
DSSP  ------------------------------------------------------------
Query ------------------------------------------------------------    0
ident
Sbjct spefrsmtaiedilqittdpsdtrgysllkseevpqgstlgvdfidtlllyqltenekld   60
DSSP  llhhhhhhhhhhhhleellllllllleellllllllllllhhhhhhhhhhhhhhllllll


DSSP  ------------------------------------------------------------
Query ------------------------------------------------------------    0
ident
Sbjct kpfeylndcfrrnqqqkritknkpnaeslhstfqeidrlvigygvvalqienfcmngafi  120
DSSP  lhhhhhhhhhhhhhhhhhhhhhllllhhhhhhhhhhhhhhhhhhhhhhhllllllllllhh


DSSP  ---------------------------------------------------------LLL
Query ---------------------------------------------------------AAP    3
ident
Sbjct nyitgivsnvnsytdflsqiiqrailegtaldllnavfptlleycnkhvshfdlnesVIY  180
DSSP  hhhhhhhlhhhlhhhhhhhhhhhhhhllhhhhhhhhhhhhhhhhhhhllllllllhHHH


DSSP  LLLL-----------------------------------llLHHHHHHHHHhHHHHL---
Query YSSP-----------------------------------ptNLRSLRDRLTqVAERQ---   25
ident                                           |
Sbjct NNVLtifelfvtfkpiaeiftkidgffadysckpqdferktILGPILSLSP-IEAAVair  239
DSSP  HHHHhhhhhhhllhhhhhhhhhllllllllllhhhhhhhlLLHHHHLLLL-LLHHHhhh
```

```
DSSP
Query ------------------------------------------------------------   25
ident
Sbjct nygdnllrskqqtamiheslqaehkvvidrlffivdklvrgslnsrtdmisyfahiankn  299
DSSP  hlllllllhhhhhhhhhhhhhhhhhhhhhhhhhhhhhhhhhlhhhhhhhhhhhhhhhhll


DSSP  ----------lllhhHHHHHHHHHHHHHHHHLLllllLLLLeeeehhhhhhhhhllllll
Query ----------gvvfgRLQRHVAXIVVAQFAATLtddtGAPLllvkggsslelrrgipdsr   75
ident                             |
Sbjct hlrradhppfkelssNGFMSNITLLLVRFSQPF---lDISY-------------------  337
DSSP  hhhhlllllhhhlllHHHHHHHHHHHHHHHHHH---hLLLL-------------------


DSSP  lllleeeeeLLLHHhhhhhhhhhhhhlllleeeeellLEEL-------------------
Query tskdfatvaRRDIEliheqladagetgwegftaiftaPEEI-------------------  116
ident             |
Sbjct -------kkIDKID---------------------anYFNNpslfidlsgetrlnsdfke  369
DSSP  -------llHHHLL---------------------llLLLLllllllllllllllllhhh


DSSP  -----------llllllllleeEEEEeeelleeeeEEEE---------------------
Query -----------aapgxpvkprrFTAKlsyrgrafaTVPI---------------------  144
ident                       |            |
Sbjct adafydknrktadskpnfisdcFFLT--------lTYLHyglggtlsfeekmgseikalk  421
DSSP  hhhhhhhhlllllllllhhhhhHHHH--------hHHHHhlhhhhhhhhhlhhhhhhhhh


DSSP  ------------------------------------------------------------
Query ------------------------------------------------------------  144
ident
Sbjct eeiekvkkiaanhdvfarfitaqlskmekalktteslrfalqgffahrslqlevfdficg  481
DSSP  hhhhhllllllllhhhhhhhhhhhhhhhhhhhhhhhhhhhhhhhhhhllhhhhhhhhhhhh


DSSP  ----eeEELL--------------------------------------------------
Query ----evSSVE--------------------------------------------------  150
ident         |
Sbjct astfliRVVDpehefpfkqiklplipdqivdnadflrahapvpfkyypefvvegpvnysl  541
DSSP  hhhhhhHHHLllllllllllllllllllllllhhhhhhlllllhhhlllhhhhhhhhhhh


DSSP  --------llllLLLEeeeelhhhhlllllleeeeeLLHHHHHHHHHHHHhllllllllL
Query --------agnaDQFDtltsdalglvgvpaavavpcXTIPWQIAQKLHAVtavleepkaN  202
ident                                               |
Sbjct yiskyqtspifrNPRL--------------------GSFVEFTTMVLRCP------elvS  575
DSSP  hhllllllllllLLLH--------------------HHHHHHHHHHHHLL------lllL


DSSP  LLLHHHHHHHHHHHHL----------LHHHHHHhhHHHHHHhhhhllllllLLLL--LLL
Query DRAHDLVDLQLLEGLL----------LDADLXPtrSACIAIfearaqhpwpPRVA--TLP  250
ident          |||
Sbjct NPHLKGKLVQLLSVGAmpltdnspgfMMDIFEH-dELVNKN-----llyalLDFYviVEK  629
DSSP  LHHHHHHHHHHHHHHHllllllllllLHHHHHH-lHHHHHH-----hhhhhHHHHhhHHH


DSSP  LhhHHHHhhhllllllllllLHHHHHHHHHHHHHHHH-HLLL------------------
Query HwpLIYAgalegldhlelarTVDAAAQAVQRFVARID-RATK------------------  291
ident
Sbjct T-gSSSQ-----------fyDKFNSRYSISIILEELYyKIPSyknqliwqsqnnadffvr  677
DSSP  L-lLLLL-----------llLHHHHHHHHHHHHHHHHhHLHHhhhhhhhhhhhlhhhhhh


DSSP  ------------------------------------------------------------
Query ------------------------------------------------------------  291
ident
Sbjct fvarmlndltflldeglsnlaevhniqneldnrargapreeedkelqtrlasasrqakss  737
DSSP  hhhhhhhhhhhhhhhhhhhhhhhhhhhhhhhlllllllllllhhhhhhhhhhhhhhhhhh


DSSP  ------------------------------------------------------------
Query ------------------------------------------------------------  291
ident
Sbjct cgladksmklfeiyskdipaafvtpeivyrlasmlnynleslvgpkcgelkvkdpqsysf  797
DSSP  hhhhhhhhhhhhhhhhhlhhhhllhhhhhhhhhhhhhhhhhlhhhhllllllllhhhhll


DSSP  ------------------------------------------------------------
```

```
Sbjct [illegible]
DSSP  lhhhhhhhhhhhhhhhlllhhhhhhhhhhlllllhhhhhhhhhhhlllllllhhhhhhhh


DSSP  ------------------------------------------------------------
Query ------------------------------------------------------------  291
ident
Sbjct lnfankaeeqrkadeeedleygdvpdefldplmytimkdpvilpaskmnidrstikahll  917
DSSP  hhhhhhhhhhhhhhhhhhhhhhllllhhhllllllllllleeelllllleeehhhhhhhl


DSSP  -------------------------------------l
Query -------------------------------------r  292
ident
Sbjct sdstdpfnrmplkledvtpneelrqkilcfkkqkkeea  955
DSSP  llllllllllllhhhleelhhhhhhhhhhhhhhhhlll
```

No 666: Query=mol1A Sbjct=2h0kA Z-score=3.0

back to top

```
DSSP  llllllllllhhhhhhhhhhhhhhllllhhhhhhhhHHHHHHHHHHLLLllllLLLEeee
Query aapysspptnlrslrdrltqvaerqgvvfgrlqrhvAXIVVAQFAATLTddtgAPLLlvk   60
ident                                                      |
Sbjct ------alrgtvtdfsgfdgeadaevlekameglgtDEDSILNLLTARS---nAQRQ---   48
DSSP  ------lllllllllllllhhhhhhhhhhlllllllLHHHHHHHHLLLL---hHHHH---


DSSP  hhhhhhhhllllllllllleeeeELLL----hHHHHhhhhhhhhhllllleeeeellleel
Query ggsslelrrgipdsrtskdfatvARRD----iELIHeqladagetgwegftaiftapeei  116
ident                        |
Sbjct ---------------------qiAEEFetlfgRDLV------------------------   63
DSSP  ---------------------hhHHHHhhhhlLLHH------------------------


DSSP  llllllllleEEEEeeeelleeeeeeEEEEeellllllllleeeeelhhhhllllllleee
Query aapgxpvkprRFTAklsyrgrafatvPIEVssveagnadqfdtltsdalglvgvpaavav  176
ident
Sbjct -------ndmKSEL---------tgkFEKL---------------------------iva   80
DSSP  -------hhhHHHL---------lhhHHHH---------------------------hhh


DSSP  eellhhHHHHHHHHHHHHLllllllLLLLhHHHHHHHHHHHHLLHHHHHHHHHHHHHHHh
Query pcxtipWQIAQKLHAVTAvleepkaNDRAhDLVDLQLLEGLLLDADLXPTRSACIAIFEa  236
ident          |  |                 |   |           |     |
Sbjct lmkpsrLYDAYELKHALK-------GAGT-DEKVLTEIIASRTPEELRAIKQAYEEEYG-  131
DSSP  hhllhhHHHHHHHHHHHL-------LLLL-LHHHHHHHHHHLLHHHHHHHHHHHHHHHL-


DSSP  hlllLLLL--LLLLlllhhhhhhhhhllllllllllhhhhhHHHHHHHHHHhHLLL---
Query raqhPWPP--RVATlphwpliyagalegldhlelartvdaaaQAVQRFVARIdRATK---  291
ident              |                                  |     |
Sbjct ---sNLEDdvVGDT--------------------------sgYYQRMLVVLL-QANRdpd  161
DSSP  ---lLHHHhhHHHL--------------------------lhHHHHHHHHHH-LLLLlll


DSSP  ------------------------------------------------------------
Query ------------------------------------------------------------  291
ident
Sbjct taiddaqveldaqalfqagelkwgtdeeefitilgtrsvshlrrvfdkymtisgfqieet  221
DSSP  llllhhhhhhhhhhhhhhllllllllhhhhhhhhhhllhhhhhhhhhhhhhhhhlllhhhh


DSSP  ------------------------------------------------------------
Query ------------------------------------------------------------  291
ident
Sbjct idretsgnlenlllavvksirsipaylaetlyyamkgagtddhtlirvivsrseidlfni  281
DSSP  hhhhllhhhhhhhhhhhhhhhhlhhhhhhhhhhhhhhlllllhhhhhhhhhhhllllhhhh


DSSP  ------------------------------------l
Query ------------------------------------r  292
ident
Sbjct rkefrknfatslysmikgdtsgdykkallllsggedd  318
DSSP  hhhhhhhhlllhhhhhhhhllhhhhhhhhhhhlllll
```

No 667: Query=mol1A Sbjct=1ycnA Z-score=3.0

back to top

```
DSSP  -----LLLLLLL--LLLHHHhhhhhhhhhhhhlllllhhhhhhhhhhhhhhhhhhhlllllll
Query -----AAPYSSP--PTNLRSlrdrltqvaerqgvvfgrlqrhvaxivvaqfaatltddtg   53
ident 
Sbjct satlkVSDSVPApsDDAEQL----------------------------------------   20
DSSP  lllllLLLLLLLLhhHHHHHH----------------------------------------


DSSP  llleeeehhhhhhhhhllllllllleeeeellhhhhhhhhhhhhhhlLLLEeeeelll
Query aplllvkggsslelrrgipdsrtskdfatvarrdieliheqladagetgWEGFtaiftap  113
ident 
Sbjct ----------------------------rtafeedliisilahrsaeqrKVIR-------   45
DSSP  ----------------------------hhhhllhhhhhhhllllhhhhHHHH-------


DSSP  eellllllllllleeeeeeeeELLE------------------------------------
Query eeiaapgxpvkprrftaklsYRGR------------------------------------  137
ident 
Sbjct ------------------qAYHEtygedllktldkelsndferaillwtlepgerdall   86
DSSP  ------------------hHHHHhhlllllllllllllhhhhhhhhhhllhhhhhhhh


DSSP  --------------------------------eeeeeeeeeeelllllllllleeeeelhh
Query --------------------------------afatvpievssveagnadqfdtltsdal  165
ident 
Sbjct aneatkrwtssnqvlmevactrtstqllharqayharykksleedvahhttgdfrkllvs  146
DSSP  hhhhhhllllllhhhhhhhlllhhhhhhhhhhhhhhlllhhhhhhhhllhhhhhhhhh


DSSP  hhlllllleeeeelLHHHHHHHHHHHHHLlllllllllLLHHHH-HHHHHHHHLLHHHHH
Query glvgvpaavavpcxTIPWQIAQKLHAVTAvleepkandRAHDLV-DLQLLEGLLLDADLX  224
ident               |   | |   |               |    |          |
Sbjct lvtsyryegdevnmTLAKQEAKLVHEKIK---------DKHYNDeDVIRILSTRSKAQIN  197
DSSP  hhhllllllllllhHHHHHHHHHHHHHHHH---------LLLLLLhHHHHHHHHLLHHHHH


DSSP  HHHHHHHHHHHHhhlllLLLL--LLLLLllhhhhhhhhhlllllllllllhHHHHHHHHHH
Query PTRSACIAIFEaraqhPWPP--RVATLphwpliyagalegldhlelartvDAAAQAVQRF  282
ident  |                                                |
Sbjct ATFNRYQDDHG-----EEILksLEEGD--------------------ddDKFLALLRST  231
DSSP  HHHHHHHHHHL-----LLHHhhHLLLL--------------------llLHHHHHHHHH


DSSP  HHHHHH------------------------------------------------------
Query VARIDR------------------------------------------------------  288
ident      |
Sbjct IQCLTRpelyfvdvlrsainktgtdegaltrivttraeidlkvigeeyqrrnsiplekai  291
DSSP  HHHHHLhhhhhhhhhhhhhllllllllhhhhhhhhhllllhhhhhhhhhhhhlllhhhhh


DSSP  -----------------llll
Query -----------------atkr  292
ident 
Sbjct tkdtrgdyekmlvallgedda  312
DSSP  llllllhhhhhhhhhlllllll
```

No 668: Query=mol1A Sbjct=3jyyB Z-score=3.0

back to top

```
DSSP  llllllllllhhhhhhhhhhhhhhllllhhhHHHHHHHHHHHHHHHHLLLlllllLLEEEE
Query aapysspptnlrslrdrltqvaerqgvvfgrLQRHVAXIVVAQFAATLTddtgaPLLLVK   60
ident                                |        |
Sbjct -------------------------------LKQKELIANVKNLTESDE-----RITACX   24
DSSP  -------------------------------LHHHHHHHHHHHHHHHHLL-----LEEEEE


DSSP  HHhhhhhhhlllLLLL----lLLEEEEEL----LLHHhhHHHHHHHHhhhllllEEEE-EL
Query GGsslelrrgipDSRT----sKDFATVAR----RDIEliHEQLADAgetgwegFTAI-FT  111
ident                        |
```

```
DSSP  LLEEllllllllllEEEEEEEEelleEEEEEEEEEEEL--LLLLllllleeeeelhhhhll
Query APEEiaapgxpvkpRRFTAKLSyrgrAFATVPIEVSSV--EAGNadqfdtltsdalglvg  169
ident    |                                |
Sbjct KNEY----------GTEVVIFD----NLIRGEFHFLSEkdXNII------psfkdsgyip  110
DSSP  ELLL----------LLEEEEEL----LLLEEEEEEEEEHhhHHHH------hhhhhlllll


DSSP  llllleeEEEL---LHHHHHHhhHHHHhllllllllllllhhhhhhhhhhhhllhhhhhhh
Query vpaavaVPCX---TIPWQIAqkLHAVtavleepkandrahdlvdlqllegllldadlxpt  226
ident
Sbjct dtkaxlIYDEtgqLENYLSE--ISGA----------------------------------  134
DSSP  llhhheEEELllhHHHHHHH--HLLL----------------------------------


DSSP  hhhhhhhhhhhllllllllLLLLL-----------------------------------
Query rsaciaifearaqhpwpprVATLP-----------------------------------  250
ident
Sbjct -------------------RPNRLteenanfllcnfsnlwlxginvlkrgeyarslells  175
DSSP  -------------------LLLLLlhhhhhhhhhhhhhhhhhhhhhhhhhhllhhhhhhhhh


DSSP  ---------------LHHHHHHHHHL----------------------------------
Query ---------------HWPLIYAGALE----------------------------------  261
ident
Sbjct qlqkntlqlirxaekNADNWLNXSKNlekeislenykkfakttarldkvelfeayknsll  235
DSSP  hhhhhhhhhhhhhhllLLLLLLLLLLLhhhhllhhhhhhhhlllllllhhhhhhhhhhhh


DSSP  llllllllllhhhhhhhhhhhhhhhhhhllll
Query gldhlelartvdaaaqavqrfvaridratkr  292
ident
Sbjct lvxdlqshlieqynlkvthdilerllnyise  266
DSSP  hhhhhhhhhlllllllllhhhhhhhhhhlll
```

No 669: Query=mol1A Sbjct=4rwqA Z-score=3.0

back to top

```
DSSP  llllllllllhhhhhhhhhhhhhhLLLLHHHHHHHHHHHHHHHHHHH-LLLL-LLLLLLE-
Query aapysspptnlrslrdrltqvaerQGVVFGRLQRHVAXIVVAQFAA-TLTD-DTGAPLL-   57
ident                               | |    |    |   |
Sbjct -----melrhtpardldkfiedhlLPNTCFRTQVKEAIDIVCRFLKeRCFQgTADPVRVs   55
DSSP  -----lllllllllllhhhhhhllLLLHHHHHHHHHHHHHHHHHHHHhHLLLlLLLLLLEe


DSSP  EEEHHhhhhhhhlllllllLLEEEEEEL---------lLHHHHHHHHHHHHHH--LLLL-
Query LVKGGsslelrrgipdsrtsKDFATVAR---------rDIELIHEQLADAGET--GWEG-  105
ident  |  |                 |                            |
Sbjct KVVKG-gssgkgttlrgrsdADLVVFLTkltsfedqlrRRGEFIQEIRRQLEAcqREQKf  114
DSSP  EEEEE-eeeeelllllleeeEEEEEEELllllllhhhhLHHHHHHHHHHHHHHhhHHLLl


DSSP  -eeeEELLleellllllllleEEEEEE-eelleeEEEEEEEEEELLllLLLLleeeeel
Query -ftaIFTApeeiaapgxpvkprRFTAKL-syrgraFATVPIEVSSVEagNADQfdtltsd  163
ident                               |
Sbjct kvtfEVQS-------prrenprALSFVLsspqlqqEVEFDVLPAFDA--LGQW-------  158
DSSP  lleeEELL-------lllllllLLLEEEeellllleEEEEEEELLEELL--LLLL-------


DSSP  hhhhllllllleeeeellhhhhhhhhhhhhhlllllllllllhhhhhhhhhhhllhhhH
Query alglvgvpaavavpcxtipwqiaqklhavtavleepkandrahdlvdlqllegllldadL  223
ident
Sbjct ---------------------------------------------------------tpG  161
DSSP  ---------------------------------------------------------llL


DSSP  HHHhhHHHHHHHHHHLLL------------------------------------------
Query XPTrsACIAIFEARAQH------------------------------------------  240
ident
Sbjct YKP--NPEIYVQLIKECksrgkegefstcftelqrdflrnrptklkslirlvkhwyqtck  219
DSSP  LLL--LHHHHHHHHHHHhhlllllllhhhhhhhhhhhhhlllhhhhhhhhhhhhhhhhh
```

```
ident                 |   |
Sbjct kthgnklppqyalellTVYAWE---------------qgSRKTdfstaqgfqtvlelvlk  264
DSSP  hhhhhhlllhhhhhhhHHHHHH---------------llLLLLlllhhhhhhhhhhhhhl


DSSP  -hhhhhHHHHhhHHHL---------------------------------------------
Query -aaqavQRFVarIDRA---------------------------------------------  289
ident              |
Sbjct hqklciFWEA-yYDFTnpvvgrcmlqqlkkprpvildpadptgnvgggdthswqrlaqea  323
DSSP  hhhleeLLLL-lLLLLlhhhhhhhhhhhlllllleeellllllleellllhhhhhhhhhh


DSSP  --------------------lll
Query --------------------tkr  292
ident
Sbjct rvwlgypccknldgslvgawtml  346
DSSP  hhllllhhhlllllllllllll
```

No 670: Query=mol1A Sbjct=3zymC Z-score=3.0

back to top

```
DSSP  lllllllllhhhhhhhhhhhhhhllllhhhhhhhhhhhhhhhhhhlllllllllleeee
Query aapyssppptnlrslrdrltqvaerqgvvfgrlqrhvaxivvaqfaatltddtgaplllvk   60
ident
Sbjct -----------------------------------sltdritaaqhsvtgsavsktvcka   25
DSSP  -----------------------------------lhhhhhhhhhlllhhhhhhhhhhh


DSSP  hhhhhhhhhlllllllllleeeeelLLHHHHHHHHHHHHhHHLL--lleeeeellleelll
Query ggsslelrrgipdsrtskdfatvarRDIELIHEQLADAgETGW--egftaiftapeeiaa  118
ident                              |
Sbjct ttheimgpkkkhldyliqctnemnvNIPQLADSLFERT-TNSSwvvvfkslitthhlmvy   84
DSSP  llllllllhhhhhhhhhhhllllLHHHHHHHHHHHL-LLLLhhhhhhhhhhhhhhhhh


DSSP  llLLLL---LEEE-EEEE---------eelleeeeeeeeeeeelllllllllleeeeelhh
Query pgXPVK---PRRF-TAKL---------syrgrafatvpievssveagnadqfdtltsdal  165
ident            |     |
Sbjct gnERFIqylASRNtLFNLsnfldksglqgydmstfirrysrylnekavsyrqvafdftkv  144
DSSP  llHHHHhhhHHLLlLLLLlllllllllhhhhhhhhhhhhhhhhhhhhhhhhhhhhhllllll


DSSP  hhllllllleeeeeLLHHHHHHHHHHHHHHL--LLLL---------------lllllLLHHH
Query glvgvpaavavpcXTIPWQIAQKLHAVTA--VLEE---------------pkandRAHDL  208
ident                             |
Sbjct krgadgvmrtmntEKLLKTVPIIQNQMDAllDFNVnsneltngvinaafmllfkdAIRLF  204
DSSP  llllllhhhhllhHHHHHHHHHHHHHHHHHhhLLLLlhhhlllhhhhhhhhhhhhHHHHH


DSSP  HHHHHHHHH--LLHH-----hhhHHHHhhhhhhhhlllllLLLL--LLLLlHHHHhhhh
Query VDLQLLEGL--LLDA-----dlxPTRSaciaifearaqhpwPPRV--ATLPhWPLIyaga  259
ident                                            |
Sbjct AAYNEGIINllEKYFdmkknqckEGLD---------iykkfLTRMtrISEF-LKVA----  250
DSSP  HHHHHHHHHHhhHHHHhllhhhhhHHHH---------hhhhhHHLHhhHHHH-HHHH----


DSSP  hlllllllllLLHHHHHHHHHHHHHHHHHHLLll
Query legldhlelaRTVDAAAQAVQRFVARIDRATkr  292
ident             |          | ||
Sbjct --eqvgrnlqSEVEGVKNIMTQNVERILARG-e  280
DSSP  --hhlllhhhHHHHHHHHHHHHHHHHHHHHHL-l
```

No 671: Query=mol1A Sbjct=1y1uA Z-score=3.0

back to top

```
DSSP  lllllllllhhhhhhhhhhhhhhllllhhhhhhhhhhhhhhhhhhllllllLLLLeeee
Query aapyssppptnlrslrdrltqvaerqgvvfgrlqrhvaxivvaqfaatltddtGAPLllvk   60
ident
Sbjct -----------------------------------------------------SQKH----    4
DSSP  -----------------------------------------------------LLLL----
```

```
Query ggsslelrrgipdsrtskdfatvarrdieliheqladagetgwegftaiftapeeiaapg  120
ident
Sbjct ------------------------------------------------------------    4
DSSP  ------------------------------------------------------------


DSSP  lllleeeeeeeeeelleeeeeeeeeeeeellllllLLLEeeeelhhhhlllllleeeeeLL
Query xpvkprrftaklsyrgrafatvpievssveagnaDQFDtltsdalglvgvpaavavpcXT  180
ident
Sbjct --------------------------------lqINQR-------------------fEE   13
DSSP  --------------------------------llHHHH-------------------hHH


DSSP  HHHHHHHHHHHH-hLLLL-LLLL-------------------------------------
Query IPWQIAQKLHAV-tAVLE-EPKA-------------------------------------  201
ident                    |
Sbjct LRLITQDTENELkkLQQTqEYFIiqyqeslriqaqfaqlgqlnpqermsretalqqkqvs   73
DSSP  HHHHHHHHHHHHhhHHHHhHHHHhhhhhhhhlllllllllllllllllhhhhhhhhhhhh


DSSP  -------------llLLHHHHHHHHHHHHL------------------------------
Query -------------ndRAHDLVDLQLLEGLL------------------------------  218
ident                |       |    ||
Sbjct letwlqreaqtlqqyRVELAEKHQKTLQLLrkqqtiilddeliqwkrrqqlagnggppeg  133
DSSP  hhhhhhhhhhhhhhhHHHHHHHHHHHHHHHhhhhhhhhhlhhhhhhhhhhhhhlllllll


DSSP  -------lhHHHHHHHhhhhhhhhhhlllllllLLLL--LLLHHHhhhhhhlllllllll
Query -------ldADLXPTRsaciaifearaqhpwppRVAT--LPHWPLiyagalegldhlela  269
ident            |                     | |       |
Sbjct sldvlqswcEKLAEII----------wqnrqqiRRAEhlCQQLPI--------------p  169
DSSP  llhhhhhhhHHHHHHH----------hhhhhhhHHHHhhHLLLLL--------------l


DSSP  LLHHHHHHHHHHHHHHHHH-LLLL------------------------------------
Query RTVDAAAQAVQRFVARIDR-ATKR------------------------------------  292
ident   |      |      |
Sbjct GPVEEMLAEVNATITDIISaLVTStfiiekqppqvlktqtkfaatvrllvggklnvhmnp  229
DSSP  LLHHHHHHHHHHHHHHHHHhHHHHleeeeelllllleeelllllleeeeeeellllllllllll


DSSP  ------------------------------------------------------------
Query ------------------------------------------------------------  292
ident
Sbjct pqvkatiiseqqaksllknentrnecsgeilnnccvmeyhqatgtlsahfrnmslkresv  289
DSSP  lleeeeeeehhhhhhhhhlllllllllllllllllleeellllleeeleeeeelllllllh


DSSP  ------------------------------------------------------------
Query ------------------------------------------------------------  292
ident
Sbjct teekftvlfesqfsvgsnelvfqvktlslpvvvivhgsqdhnatatvlwdnafaepgrvp  349
DSSP  hhleeeeeeeeeeellllllleeeeeeelllleeeellllllhhhhhhhhhhhhhhlllllll


DSSP  ------------------------------------------------------------
Query ------------------------------------------------------------  292
ident
Sbjct favpdkvlwpqlcealnmkfkaevqsnrgltkenlvflaqklfnissnhledynsmsvsw  409
DSSP  lllleeehhhhhhhhhhhhhhhhhlllllllhhhhhhhhhhhllllllhhhhhhhleeeh


DSSP  ------------------------------------------------------------
Query ------------------------------------------------------------  292
ident
Sbjct sqfnrenlpgwnytfwqwfdgvmevlkkhhkphwndgailgfvnkqqahdllinkpdgtf  469
DSSP  hhhhllllllllllhhhhhhhhhhhhhhhlhhhhhhllllllllhhhhhhhhhhllllle


DSSP  ------------------------------------------------------------
Query ------------------------------------------------------------  292
ident
Sbjct llrfsdseiggitiawkfdspdrnlwnlkpfttrdfsirsladrlgdlnyliyvfpdrpk  529
DSSP  eeeeellllleeeeeeellllllllleeeeelhhhhhllhhhhhhhlllllllllllllh


DSSP  ---------------
```

```
Sbjct [illegible]
DSSP  hhhhllllllllll
```

No 672: Query=mol1A Sbjct=5tgqA Z-score=2.9

back to top

```
DSSP  lllllllllhhhhhhhhhhhhhhlllLHHHHHHHHHHHHHHHHHHLL--LLLLllllee
Query aapysspptnlrslrdrltqvaerqgvVFGRLQRHVAXIVVAQFAATL--TDDTgaplll   58
ident 
Sbjct ---------------------------MNFKKYEENLVASIEEVIQRIidDKHR------   27
DSSP  ---------------------------LLHHHHHHHHHHHHHHHHHHHhhLLLL------


DSSP  eehhhhhhhhhlllllllllleeeeelLLHHHHHHHHHHHHH-HLLLlEEEEELLLE--E
Query vkggsslelrrgipdsrtskdfatvarRDIELIHEQLADAGE-TGWEgFTAIFTAPE--E  115
ident 
Sbjct ----------------pniigktrvgaEVSDYLEDEFVKYISsGKSS-SLYDAQGAPkeK   70
DSSP  ----------------llllllllhhhHHHHHHHHHHHHHHHHlLLLL-LEEEEEELLllL


DSSP  LLLlllllllleEEEEEEEEllEEEEEEEEEEEEL-LLLL---------------------
Query IAApgxpvkprRFTAKLSYrgRAFATVPIEVSSV-EAGN---------------------  153
ident                |            |
Sbjct TKN------pwDARCKFKF-mDREEEIWIDFKAFkITNMdsnpdigtpnkivkfihegnf  123
DSSP  LLL------llLEEEEEEE-lLEEEEEEEEEEEEeHHHLllllleeelhhhhhhhhhllll


DSSP  ------------------llLLEEeeelhhhhllllllleeeeellhhhhhhhhhhhhhll
Query ------------------adQFDTltsdalglvgvpaavavpcxtipwqiaqklhavtav  195
ident 
Sbjct ylvfvlvyyeskqdgvefvkYNND-------------------------------ykkvy  152
DSSP  eeeeeeeeeeeelleeeeelLLLL-------------------------------leeee


DSSP  llllllLLLLHHhhhhhhhhhhllhhhhhhhhhhhhhhhhhhllllllllllllllHHHH
Query leepkaNDRAHDlvdlqllegllldadlxptrsaciaifearaqhpwpprvatlphWPLI  255
ident 
Sbjct llkdvnESFRIN--------------------pkpqmqvniaaeptyrtreefihfFVKK  192
DSSP  ehhhllLLLEEE--------------------llleeellllllllllhhhhhhhHHHH


DSSP  HHHhhlllllllllllhhhhhhhhhhhhhhhhhllll
Query YAGalegldhlelartvdaaaqavqrfvaridratkr  292
ident 
Sbjct WKE---sferqikslekkeimlkdledklknsndnsi  226
DSSP  HHH---hhhhhhhhhhhhhhhhhhhhhhhhhhhhhhl
```

No 673: Query=mol1A Sbjct=4x4wA Z-score=2.9

back to top

```
DSSP  lllllllllhhhhhhhhhhhhHLLLLHHHHHHHH-HHHHHHHHHHLLlllllllLEEE
Query aapysspptnlrslrdrltqvaeRQGVVFGRLQRHV-AXIVVAQFAATLtddtgapLLLV   59
ident 
Sbjct --------------------mftMKLQSPEFQSLFTeGLKSLTELFVKE-------NHEL   33
DSSP  --------------------lllLLLLLLHHHHLLLLhHHHHHHHHHHHL-------LLLE


DSSP  EHHhhhhhhhlLLLLLLL-LLEEEEEllllHHHHHHHHHHHHHhhllllleeEEELLleelll
Query KGGsslelrrgIPDSRTS-KDFATVArrdIELIHEQLADAGetgwegftAIFTApeeiaa  118
ident                     |||| |        |    ||
Sbjct RIA-ggavrdlLNGVKPQdIDFATTA--tPTQMKEMFQSAG--------IRMIN------   76
DSSP  EEE-lhhhhhhHLLLLLLlLEEEELL--lHHHHHHHHHHHL--------LEEEL------


DSSP  lllllllEEEEEEEEelleeEEEEEEEEEElllllllllleeeeelhhhhllllllleeeee
Query pgxpvkpRRFTAKLSyrgraFATVPIEVSsveagnadqfdtltsdalglvgvpaavavpc  178
ident           || |           |
Sbjct -------GTITARLH-----EENFEITTL--------------------ridvttdgaev  104
DSSP  -------LEEEEEEL-----LEEEEEEEL--------------------eeeellllllee
```

```
Query [illegible]
ident
Sbjct efttdwqkDAERRdltinsmflgfdgtlfdyfngyedlknkkvrfvghakQRIQeDYLR-  163
DSSP  eelllhhhHHHHLllhhhlleellllleellllhhhhhhlllllllllhhHHHHhLLLH-


DSSP  lllhhhhhhhhhhhhllhhhhhhhhhhhhhhhhhhlllllllllllLLLHHHHHHhhhll
Query drahdlvdlqllegllldadlxptrsaciaifearaqhpwpprvatLPHWPLIYAgaleg  262
ident
Sbjct -------------------------------------------ilrYFRFYGRIV-----  175
DSSP  -------------------------------------------hhhHHHHHHHHL-----


DSSP  lllllllllhHHHH----------------------------------------------
Query ldhlelartvDAAA----------------------------------------------  276
ident
Sbjct --------dkPGDHdpetleaiaenakglagisgeriwvelkkilvgnhvnhlihliydl  227
DSSP  --------llLLLLlhhhhhhhhhhhhhhhhllhhhhhhhhhhhhhhlllhhhhhhhhhl


DSSP  ------------------------------------------------------------
Query ------------------------------------------------------------  276
ident
Sbjct dvapyiglpanasleefdkvsknvdgfspkpvtllaslfkvqddvtkldlrlkiakeekn  287
DSSP  llhhhhlllllllhhhhhhhhhhhlllllhhhhhhhhllllhhhhhhhhhhhlllhhhhh


DSSP  ------------------------------------------------------------
Query ------------------------------------------------------------  276
ident
Sbjct lglfivknrkdlikatdssdplkpyqdfiidsdattrvcellkyqgehcllkemqqwsip  347
DSSP  hhhhhhhlllllllllllllhhhhhhhhlllhhhhhhhhhhhlllhhhhhhhhlllll


DSSP  -----------------------------------hhhhhhhhhhhhllll
Query -----------------------------------qavqrfvaridratkr  292
ident
Sbjct pfpvsghdirkvgissgkeigallqqlreqwkksgyqmekdellsyikktl  398
DSSP  lllllhhhhhhlllllhhhhhhhhhhhhhhhhhlllllhhhhhhhhhhhhl
```

No 674: Query=mol1A Sbjct=4ebjA Z-score=2.9

back to top

```
DSSP  llllllllllhhhhhhhhhhhhhhllLLHHHHHHHHHHHHHHHHHHLLllllllLLEE-E
Query aapyssspptnlrslrdrltqvaerqgVVFGRLQRHVAXIVVAQFAATLtddtgaPLLL-V   59
ident
Sbjct --------------------------ENLYFQGVQHTIARWVDRLREE-----yADAVaI   29
DSSP  --------------------------LHHHHHHHHHHHHHHHHHHHHH-----lLLEEeE


DSSP  EHHhhhhhhhlllLLLL----lLLEEEEELLLhhhhhhhhhhhhhhlllleeeeeellee
Query KGGsslelrrgipDSRT----sKDFATVARRDieliheqladagetgwegftaiftapee  115
ident                         ||
Sbjct LLK------gsyaRGDAatwsdIDFDVLVSTQ----------------------------   55
DSSP  EEE------hhhhHLLLlllllEEEEEEELLL----------------------------


DSSP  lllllllLLLEEEEEEEEellEEEEEEEEEEEELLllllllleeeeelhhhhllllllee
Query iaapgxpVKPRRFTAKLSyrgRAFATVPIEVSSVEagnadqfdtltsdalglvgvpaava  175
ident              |                |  |
Sbjct -------DVEDYRTWIEP-vgDRLVHISAAVEWVT-gwerdtvdpsswsyglptqettrl  106
DSSP  -------LLEEEEEEEEE-elLEEEEEEEEEEEHH-hhhhhhhllllllllllleeeeeee


DSSP  EEEL----LHHHHHhhHHHHhhLLLLLL--------------------------------
Query VPCX----TIPWQIaqKLHAvtAVLEEP--------------------------------  199
ident                           ||
Sbjct XWAIndetRRRLDR--PYKT--HPAAEPevedtvealgkirnaiargddlgvyqsaqtva  162
DSSP  EEELlhhhHHHHLL--LEEE--ELLLLLlhhhhhhhhhhhhhhhhhhllhhhhhhhhhhh


DSSP  ------------llLLLLhhhhhhhhhhhhllhhhhhhhhhhhhhhhhhhllllllllll
Query ------------kaNDRAhdlvdlqllegllldadlxptrsaciaifearaqhpwpprva  247
ident
```

```
DSSP  lllLHHHH----------------------------------hhhhhlllllllllllhh
Query tlpHWPLI----------------------------------yagalegldhlelartvd  273
ident
Sbjct -arQAIEAilafprvpvgfaadwltclglveersarstaaaaerxvrgvlexlptdpdll  239
DSSP  -hhHHHHHhhllllllllhhhhhhhhhlllllllhhhhhhhhhhhhhhhhllllllhhhh


DSSP  hhhhhhhhhhhhhhhllll
Query aaaqavqrfvaridratkr  292
ident
Sbjct gediarlxnagllekyvqq  258
DSSP  lhhhhhhhhhlhhhhhhhl
```

No 675: Query=mol1A Sbjct=1aeiA Z-score=2.9

back to top

```
DSSP  llllllllllhhhhhhhhhhhhhhhllllhhhhhhhhHHHHHHHHHHLLlllllLLLEeee
Query aapyssppptnlrslrdrltqvaerqgvvfgrlqrhvAXIVVAQFAATLtddtgAPLLlvk   60
ident                                              ||      |
Sbjct ------vvqgtvkphasfnsredaetlrkamkgigtDEKSITHILATR---snAQRQ---   48
DSSP  ------llllllllllllhhhhhhhhhhhlllllLHHHHHHHHHLL---lhHHHH---


DSSP  hhhhhhhhhllllllllleeeeELLLH---HHHHhhhhhhhhhllllleeeeellleell
Query ggsslelrrgipdsrtskdfatvARRDI---ELIHeqladagetgwegftaiftapeeia  117
ident
Sbjct ---------------------qiKTDYTtlfGKHL-------------------------   62
DSSP  ---------------------hhHHHHHhhhLLLH-------------------------


DSSP  lllllllleEEEEeeeelleeeeeeEEEEeellllllllleeeeelhhhhllllllleeee
Query apgxpvkprRFTAklsyrgrafatvPIEVssveagnadqfdtltsdalglvgvpaavavp  177
ident
Sbjct -----edelKSEL---------sgnYEAA---------------------------alal   81
DSSP  -----hhhhHHHL---------lhhHHHH---------------------------hhhh


DSSP  ELLHHHHHHHHHHHHHHLlllllllLLLLhHHHHHHHHHHHLLHHHHHHHHHHHHHHHHhh
Query CXTIPWQIAQKLHAVTAvleepkaNDRAhDLVDLQLLEGLLLDADLXPTRSACIAIFEar  237
ident         |  |||               |   |         |       |
Sbjct LRKPDEFLAEQLHAAMK-------GLGT-DENALIDILCTQSNAQIHAIKAAFKLLYK--  131
DSSP  HLLHHHHHHHHHHHHHL-------LLLL-LHHHHHHHHLLLLHHHHHHHHHHHHHHHL--


DSSP  lllLLLL--LLLLlllhhhhhhhhhlllllllllllhhhhhhhhhHHHHHhHHLLL----
Query aqhPWPP--RVATlphwpliyagalegldhlelartvdaaaqavqRFVARiDRATK----  291
ident             |                                  |
Sbjct --eDLEKeiISET--------------------------sgnfqrLLVSM-LQGGRkede  162
DSSP  --lLHHHhhHHHL--------------------------lhhhhhHHHHH-HLLLLllll


DSSP  ------------------------------------------------------------
Query ------------------------------------------------------------  291
ident
Sbjct pvnaahaaedaaaiyqagegqigtdesrfnavlatrsypqlhqifheyskisnktilqai  222
DSSP  lllhhhhhhhhhhhhhhlllllllllhhhhhhhhhllhhhhhhhhhhhhhhllllhhhhh


DSSP  ------------------------------------------------------------
Query ------------------------------------------------------------  291
ident
Sbjct enefsgdikngllaivksvenrfayfaerlhhamkglgtsdktlirilvsrseidlanik  282
DSSP  hhhllhhhhhhhhhhhhhhhhhhhhhhhhhhhhhhhlllllllhhhhhhhhhhhllllhhhhh


DSSP  --------------------------------l
Query --------------------------------r  292
ident
Sbjct etfqamygkslyefiaddcsgdykdlllqitgh  315
DSSP  hhhhhhhlllhhhhhhhhllhhhhhhhhhhhll
```

back to top

```
DSSP  ------------------------------------------------------------
Query ------------------------------------------------------------    0
ident
Sbjct prahtahlrtarleltpldpaadarhlhhaygdeevmrwwtrpacadpaeteryltscaa   60
DSSP  lllllllleellllleeeellhhhhhhhhhhhlllllllllllllllhhhhhhhhhhhh


DSSP  ------------------------------------------------------------
Query ------------------------------------------------------------    0
ident
Sbjct apgarlwtirapdgtvpgmagllggtdvpgltwllrrdswghgyateaaaavvghaledg  120
DSSP  llleeeeeellllllleeeeellllllllleeeelhhhllllhhhhhhhhhhhhhhlll


DSSP  --------------------------------llllllllllhhhhhhhhhhhhhhllll
Query --------------------------------aapysspptnlrslrdrltqvaerqgvv   28
ident
Sbjct gldrveawieagnrrslavaarvglterarlaqhyphrpgphemvvlgkaraeeplttla  180
DSSP  llleeeeeeelllhhhhhhhhhhlleeeeeeeellllllleeeeeeeeellllllleeee


DSSP  hHHHH----hhhhhhHHHHHHHlllllllllleeeehhhhhHHHHLL------------l
Query fGRLQ----rhvaxiVVAQFAAtltddtgaplllvkggsslELRRGI------------p   72
ident                 |
Sbjct vITELpvrdvaatlrLVEAALG-artafaigdppefaeaalTPWSAGprfrlaavpgpgp  239
DSSP  eEEEEeellhhhhhhHHHHHLL-leeeeeelllllleeeeelLLLLLLleeeeeelllll


DSSP  lllllLLEEEEEllLHHHHHHHHHHHHhhlllleeEEEL-LLEELLLlllllLLEEEEEE
Query dsrtsKDFATVArrDIELIHEQLADAGetgwegftAIFT-APEEIAApgxpvKPRRFTAK  131
ident                    |    |||        |     |              |
Sbjct vepvrLHLDAAG--TADSLHRRAVDAG--------ARVDgPPVRRPW-----GRSEFVIT  284
DSSP  lllleEEEEEEL--LHHHHHHHHHHLL--------LLLLlLLEELLL-----LLEEEEEE


DSSP  EEelleEEEEEEEEEEElllllllllleeeeelhhhhlllllleeeeellhhhhhhhhhhh
Query LSyrgrAFATVPIEVSSveagnadqfdtltsdalglvgvpaavavpcxtipwqiaqklha  191
ident |
Sbjct LP----EGHELTVSAPV-------------------------------------------  297
DSSP  LL----LLLEEEEEELL-------------------------------------------


DSSP  hhllllllllllllhhhhhhhhhhhllhhhhhhhhhhhhhhhhhhhllllllllllllll
Query vtavleepkandrahdlvdlqllegllldadlxptrsaciaifearaqhpwpprvatlph  251
ident
Sbjct ------------------------------------------------------------  297
DSSP  ------------------------------------------------------------


DSSP  hhhhhhhhlllllllllllhhhhhhhhhhhhhhhhhhllll
Query wpliyagalegldhlelartvdaaaqavqrfvaridratkr  292
ident
Sbjct -----------------------------------------  297
DSSP  -----------------------------------------
```

No 677: Query=mol1A Sbjct=3tjzB Z-score=2.9

back to top

```
DSSP  llllllllllhhhhhhhhhhhhhhllllhhhhhhhhhhhhhhhhhllllllllllleeee
Query aapysspptnlrslrdrltqvaerqgvvfgrlqrhvaxivvaqfaatltddtgaplllvk   60
ident
Sbjct ------------------------------------------------------------    0
DSSP  ------------------------------------------------------------


DSSP  hhhhhhhhllllllllllleeeeellhhhhhhhhhhhhhhhllllleeeeellleelllll
Query ggsslelrrgipdsrtskdfatvarrdieliheqladagetgwegftaiftapeeiaapg  120
ident
Sbjct ------------------------------------------------------------    0
DSSP  ------------------------------------------------------------
```

```
Query [illegible]
ident
Sbjct --------------------------------------------------------qHLE    4
DSSP  --------------------------------------------------------lLLL


DSSP  HHHHHHHHhHHHHLLllllllLLLL-HHHHHHHHHHHHL-LHHHHHhhhhhhhhhhhhhl
Query IPWQIAQKlHAVTAVleepkaNDRA-HDLVDLQLLEGLL-LDADLXptrsaciaifeara  238
ident                                 |     |      |
Sbjct KSAVLQEA-RVFNET------PINPrKCAHILTKILYLInQGEHLG-----------tte   46
DSSP  HHHHHHHH-HHLLLL------LLLHhHHHHHHHHHHHHHhHLLLLL-----------hhh


DSSP  lllllLLLL--LLLLHHhhhhhhhlllllllllLHHHHHHHHHHHHHHHHHH--------
Query qhpwpPRVA--TLPHWPliyagalegldhlelarTVDAAAQAVQRFVARIDR--------  288
ident                  |                 |              |
Sbjct ateafFAMTklFQSNDP-----------------TLRRMCYLTIKEMSCIAEdviivtss   89
DSSP  hhhhhHHHHhhHHLLLH-----------------HHHHHHHHHHHHHLLLLLlhhhhhhh


DSSP  ------------------------------------------------------------
Query ------------------------------------------------------------  288
ident
Sbjct ltkdmtgkedsyrgpavralcqitdstmlqaierymkqaivdkvpsvsssalvsslhllk  149
DSSP  hhhhhhlllhhhhhhhhhhhhhhhlllllhhhhhhhhhhhhllllhhhhhhhhhhhhhll


DSSP  ------------------------------------------------------------
Query ------------------------------------------------------------  288
ident
Sbjct csfdvvkrwvneaqeaassdnimvqyhalgllyhvrkndrlavskmiskftrhglkspfa  209
DSSP  llhhhhhllhhhhhhhlllllhhhhhhhhhhhhhhhlllhhhhhhhhhhhhlllllhhh


DSSP  ---------------------------------------------------llll
Query ---------------------------------------------------atkr  292
ident
Sbjct ycmmirvasrqsclrnkhemvvyeaasaivnavsvlqlfcsspkaalryaavrtl  264
DSSP  hhhhhhhhlllllllllhhhhhhhhhhhllllllhhhhhhllllllhhhhhhhll
```

No 678: Query=mol1A Sbjct=3ov7B Z-score=2.9

back to top

```
DSSP  llllllllllhhhhhhhhhhhhhhlLLLHHHHHHHHHHHHHHHHHHLLLlllllllEEEE
Query aapysspptnlrslrdrltqvaerqGVVFGRLQRHVAXIVVAQFAATLTddtgaplLLVK   60
ident                                      |          |
Sbjct ----------mkveeilekalelviPDEEEVRKGREAEEELRRRLDELG-------VEYV   43
DSSP  ----------llllhhhhhhhhhhlLLHHHHHHHHHHHHHHHHHHHHHL-------LLEE


DSSP  HHhhhhhhhllllllllLLEEEEEL------LLHHHHHHHHHHHHhhlllleeEEELLLE
Query GGsslelrrgipdsrtsKDFATVAR------RDIELIHEQLADAGetgwegftAIFTAPE  114
ident                    |               |   |
Sbjct FV-gsyarntwlkgsleIDVFLLFPeefskeELRERGLEIGKAVL--------DSYEIRY   94
DSSP  EE-hhhhllllllllllEEEEEEELllllhhHHHHHHHHHHHHHL--------LLEELLL


DSSP  ELllllllllLEEEEEEEEEelleeEEEEEEEEEELLLLllllleeeeelhhhhlllllle
Query EIaapgxpvkPRRFTAKLSyrgraFATVPIEVSSVEAGnadqfdtltsdalglvgvpaav  174
ident                            |
Sbjct AE--------HPYVHGVVK-----GVEVDVVPCYKLKE----------------------  119
DSSP  LL--------LLLEEEEEL-----LEEEEEEEEELLLL----------------------


DSSP  eeeellhhhhhhHHHHHHhllllllllLLLLhhhhhhhhhhhhllhhhhhhhhhhhhhhh
Query avpcxtipwqiaQKLHAVtavleepkaNDRAhdlvdlqllegllldadlxptrsaciaif  234
ident                               |
Sbjct pkniksavdrtpFHHKWL---------EGRI-----------------------------  141
DSSP  lllllllllhhhHHHHHH---------HHHL-----------------------------


DSSP  hhhllllllllllllllhhhHHHHHHLL-LLLL-------------------llllLHHH
Query earaqhpwpprvatlphwplIYAGALEG-LDHL-------------------elarTVDA  274
ident                                  |                        |
```

```
DSSP  H----------------hhhHHHHhhhhhHLLL---------------------------
Query A----------------aqaVQRFvaridRATK---------------------------  291
ident                     |
Sbjct GsfletvknarrwtrrtvidVAKG-evrkGEEFfvvdpvdekrnvaanlsldnlarfvhl  241
DSSP  LlhhhhhhhhllllllleeeLLLL-eeelLLLLeelllllllllllllllhhhhhhhhhh


DSSP  ------------------------------------------------------------
Query ------------------------------------------------------------  291
ident
Sbjct crefmeapslgffkpkhpleieperlrkiveergtavfavkfrkpdivddnlypqleras  301
DSSP  hhhhhhlllhhhllllllllllhhhhhhhhhhhlleeeeeeeelllllhhhhhhhhhhhh


DSSP  ------------------------------------------------------------
Query ------------------------------------------------------------  291
ident
Sbjct rkifeflerenfmplrsafkaseefcyllfecqikeisrvfrrmgpqfedernvkkflsr  361
DSSP  hhhhhhhhhlllleeeeeeeellleeeeeeeellllllleeeeeeeeelllhhhhhhhhl


DSSP  ------------------------------------------------------------
Query ------------------------------------------------------------  291
ident
Sbjct nrafrpfiengrwwafemrkfttpeegvrsyasthwhtlgknvgesireyfeiisgeklf  421
DSSP  lllllleeelleeeeeeellllllhhhhhhhhhhhhhhhllhhhhhhhhhlleeeelhhhh


DSSP  ---------------l
Query ---------------r  292
ident
Sbjct kepvtaelcemmgvkd  437
DSSP  llllhhhhhhhhllll
```

No 679: Query=mol1A Sbjct=2ia7A Z-score=2.9

back to top

```
DSSP  llllllllllhhhhhhhhhhhhhHLLLLHHHHHHHHHHHHHHH-----------hHHLLL
Query aapysspptnlrslrdrltqvaeRQGVVFGRLQRHVAXIVVAQ-----------fAATLT   49
ident                                       |
Sbjct ----------------------aXVLSSAEEDIAESIRIILGTargervxrpdfgCGIHD   38
DSSP  ----------------------lLLLLLHHHHHHHHHHHHHLLllllllllllllLHHHH


DSSP  llllllleeeehhhhhhhhhhllllllllleeeeellLHHHHHHHHHHHHHHLLLLEEE-
Query ddtgaplllvkggsslelrrgipdsrtskdfatvarrDIELIHEQLADAGETGWEGFTA-  108
ident                                         ||      |
Sbjct ----------------------------rvfsvinttTLGLIENEVKEALILWEPRIELl   70
DSSP  ----------------------------lllllllhhHHHHHHHHHHHHHHHHLLLEEEe


DSSP  EELLLE-ELLLlllllLLEEEEEEEEEL-lEEEEEEEEEEEELLLLllllleeeeelhhh
Query IFTAPE-EIAApgxpvKPRRFTAKLSYR-gRAFATVPIEVSSVEAGnadqfdtltsdalg  166
ident   ||   | |                 |               |
Sbjct SVTASPrEAAE-----GRLLIDIEYRVRstNTRFNLVYPFYLKESA--------------  111
DSSP  EEEEELlLHHH-----LEEEEEEEEEELllLEEEEEEEEEELLLLL--------------


DSSP  hllllllleeeeellhhhhhhhhhhhhhhlllllllllllhhhhhhhhhhhllhhhhhhhh
Query lvgvpaavavpcxtipwqiaqklhavtavleepkandrahdlvdlqllegllldadlxpt  226
ident
Sbjct ------------------------------------------------------------  111
DSSP  ------------------------------------------------------------


DSSP  hhhhhhhhhhllllllllllllllhhhhhhhhhhhllllllllllhhhhhhhhhhhhhhh
Query rsaciaifearaqhpwpprvatlphwpliyagalegldhlelartvdaaaqavqrfvari  286
ident
Sbjct ------------------------------------------------------------  111
DSSP  ------------------------------------------------------------
```

```
Sbjct ------  111
DSSP  ------
```

No 680: Query=mol1A Sbjct=3hpvA Z-score=2.9

back to top

```
DSSP  llllllllllhhhhhhhhhhhhhhllllhhHHHHHHHHHH-HHHH---------------
Query aapysspptnlrslrdrltqvaerqgvvfgRLQRHVAXIV-VAQF---------------   44
ident                                |         |
Sbjct --------------amtgvlrpghaqvrvlNLEEGIHFYRnVLGLvetgrddqgrvyfkc   46
DSSP  --------------llleeeeeeeeeeeelLHHHHHHHHHhLLLLeeeeelllllleeeel


DSSP  ------------------------------------------------------------
Query ------------------------------------------------------------   44
ident
Sbjct wderdhscyiireadtagidffgfkvldkatlekldadlqaygltttripagemletger  106
DSSP  lllllllleeeeellllleeeeeeeeellhhhhhhhhhhhhhhhlllleeellllllllle


DSSP  -------------------------hhllllLLLLL-----lEEEEH-------------
Query -------------------------aatltdDTGAP-----lLLVKG-------------   61
ident
Sbjct vrfelpsghlielyaektcvgngisevnpapWNAQRehgiapIQLDHcllygpniaevqk  166
DSSP  eeeelllllleeeeellllllllllllllllLLHHHhlllllLEEEEeeeeellhhhhhh


DSSP  -------------------------------hhhhhhhlllllLLLLLEEEEEELL--LH
Query -------------------------------gsslelrrgipdsRTSKDFATVARR--DI   88
ident
Sbjct iftevlgfylvervlspdgdsmgiwlscshkvhdiafveypekGKLHHCSFLLESweQV  226
DSSP  hhhhllllleeeeeeeelllllleeeeeeeelllllleeeeellllLEEEEEEEELLLhhHH


DSSP  HHHHHHHHHHHhhlllleeeEELLLEELLLLLLLLLLEEEEEEeeelleeeEEEEEEEEE
Query ELIHEQLADAGetgwegftaIFTAPEEIAAPGXPVKPRRFTAKlsyrgrafATVPIEVSS  148
ident                                                         |
Sbjct LRAGDIMSMNE---------VNVDIGPTRHGVTRGCTIYAWDP--------SGNRFETFM  269
DSSP  HHHHHHHHHLL---------LLEEEEEEELLLLLLEEEEEEELL--------LLLEEEEEE


DSSP  LLLLllllleeeeelhhhhllllllleeeeellhhhhhhhhhhhhhlllllllllllhhh
Query VEAGnadqfdtltsdalglvgvpaavavpcxtipwqiaqklhavtavleepkandrahdl  208
ident
Sbjct GGYH--------------------------------------------------------  273
DSSP  LLLL--------------------------------------------------------


DSSP  hhhhhhhhllhhhhhHHHHhhhhhhhhhLLLLlllllllllhhhhhhhhhllllllll
Query vdlqllegllldadlxPTRSaciaifearAQHPwpprvatlphwpliyagalegldhlel  268
ident
Sbjct ---------------pYPDY------eplSWTY---------------------------  285
DSSP  ---------------lLLLL------lleEELH---------------------------


DSSP  lllhhhhhhhhhhhhhhhhhllll
Query artvdaaaqavqrfvaridratkr  292
ident
Sbjct ------------dnfaqgldypqr  297
DSSP  ------------hhhhhlllllll
```

No 681: Query=mol1A Sbjct=2iicA Z-score=2.9

back to top

```
DSSP  ------------------------------------------------------------
Query ------------------------------------------------------------    0
ident
Sbjct daipevkaileakneeelvtftsrwsaeerkelrtqfqdttglefiaflkkcikngpyed   60
DSSP  llhhhhhhhhhlllhhhhhhhhhhllhhhhhhhhhhhhhhhhlllhhhhhllllllhhhh
```

```
[illegible]
Query ------AAPY----------------------sspptnlrslrdrlTQVAERQGVVFGRL   32
ident                                                   |  |
Sbjct vmalgwDCNIsarvnvikkamknvndfraihdvvliatpderlklaQAYKEKTGNDLLQD  120
DSSP  hhhhhlLLHHhhhhhhhhhhhlllllhhhhhhhhhlllhhhhhhhhHHHHLLLLLLHHHH


DSSP  HHHHhhhhhhhhhhhlllllllllleeeehhhhhhhhhlllllllllleeeeeelllhhh
Query QRHVaxivvaqfaatltddtgaplllvkggsslelrrgipdsrtskdfatvarrdielih   92
ident
Sbjct FVDQ--------------------------------------------------------  124
DSSP  HHHH--------------------------------------------------------


DSSP  hhhhhhhhhllllleeeeellleellllllllleeeeeeeeelleeeeeeeeeeeellll
Query eqladagetgwegftaiftapeeiaapgxpvkprrftaklsyrgrafatvpievssveag  152
ident
Sbjct ------------------------------------------------------------  124
DSSP  ------------------------------------------------------------


DSSP  llllleeeeelhhhhlllllleeeeeLLHHHHHHHHHHHHHLlllllllLLLLHhhHHHH
Query nadqfdtltsdalglvgvpaavavpcXTIPWQIAQKLHAVTAvleepkaNDRAHdlVDLQ  212
ident                                  |  |                |
Sbjct -----ipltsaasylchlairenrtpRGSVASDAEVLKHNLI-----daDEPDH--EAVV  172
DSSP  -----lllllhhhhhhhhllllllllLLLHHHHHHHHHHHLL-----llLLLLH--HHHH


DSSP  HHHHHLLHHHHHHHHHHHHHHHHhhlllLLLL--LLLLlllhhhhhhhhhllllllllll
Query LLEGLLLDADLXPTRSACIAIFEaraqhPWPP--RVATlphwpliyagalegldhlelar  270
ident  |
Sbjct RLIITSTADEYKEINHRFEVLTG-----KSVQeaIETR---------------------y  206
DSSP  HHHHLLLHHHHHHHHHHHHHHHL-----LLHHhhHHHH---------------------l


DSSP  lHHHHHHHHHHHHHHHHH------------------------------------------
Query tVDAAAQAVQRFVARIDR------------------------------------------  288
ident      |
Sbjct aDKENARGLCIAHYYNLAparavayafhsavetqnddmayeqaaritglfhdlhkfawvh  266
DSSP  lLHHHHHHHHHHHHHLLLhhhhhhhhhhhhhllllhhhhhhhhhhhhhhhllllllhhhh


DSSP  --------------------------------llll
Query --------------------------------atkr  292
ident
Sbjct yacwgvmrddilsrfqskeankvnfrdaclmfwkla  302
DSSP  hhhlllhhhhhhhhlllllhhhllhhhhhhhhllll
```

No 682: Query=mol1A Sbjct=2iicB Z-score=2.9

back to top

```
DSSP  ------------------------------------------------------------
Query ------------------------------------------------------------    0
ident
Sbjct daipevkaileakneeelvtftsrwsaeerkelrtqfqdttglefiaflkkcikngpyed   60
DSSP  llhhhhhhhhlllhhhhhhhhhllhhhhhhhhhhhhhhhlllhhhhhlllllllhhhh


DSSP  ------LLLL----------------------llllllhhhhhhhhHHHHHHLLLLHHHH
Query ------AAPY----------------------sspptnlrslrdrlTQVAERQGVVFGRL   32
ident                                                   |  |
Sbjct vmalgwDCNIsarvnvikkamknvndfraihdvvliatpderlklaQAYKEKTGNDLLQD  120
DSSP  hhhhhlLLHHhhhhhhhhhhhlllllhhhhhhhhhlllhhhhhhhhHHHHLLLLLLHHHH


DSSP  HHHHhhhhhhhhhhhlllllllllleeeehhhhhhhhhlllllllllleeeeeelllhhh
Query QRHVaxivvaqfaatltddtgaplllvkggsslelrrgipdsrtskdfatvarrdielih   92
ident
Sbjct FVDQ--------------------------------------------------------  124
DSSP  HHHH--------------------------------------------------------


DSSP  hhhhhhhhhllllleeeeellleellllllllleeeeeeeeelleeeeeeeeeeeellll
Query eqladagetgwegftaiftapeeiaapgxpvkprrftaklsyrgrafatvpievssveag  152
```

```
DSSP  llllleeeeelhhhhllllllleeeeeLLHHHHHHHHHHHHHLlllllllLLLLHhhHHHH
Query nadqfdtltsdalglvgvpaavavpcXTIPWQIAQKLHAVTAvleepkaNDRAHdlVDLQ  212
ident                                  |  |                |
Sbjct -----ipltsaasylchlairenrtpRGSVASDAEVLKHNLI-----daDEPDH--EAVV  172
DSSP  -----llllhhhhhhhhlllllllLLLHHHHHHHHHHHLL-----llLLLLH--HHHH


DSSP  HHHHHLLHHHHHHHHHHHHHHHHhhlllLLLL--LLLLlllhhhhhhhhhllllllll
Query LLEGLLLDADLXPTRSACIAIFEaraqhPWPP--RVATlphwpliyagalegldhlelar  270
ident  |
Sbjct RLIITSTADEYKEINHRFEVLTG-----KSVQeaIETR--------------------y  206
DSSP  HHHHLLLHHHHHHHHHHHHHHHL-----LLHHhhHHHH--------------------l


DSSP  lHHHHHHHHHHHHHHHHH----------------------------------------
Query tVDAAAQAVQRFVARIDR----------------------------------------  288
ident      |
Sbjct aDKENARGLCIAHYYNLAparavayafhsavetqnddmayeqaaritglfhdlhkfawvh  266
DSSP  lLHHHHHHHHHHHHHLLLhhhhhhhhhhhhllllhhhhhhhhhhhhhhhlllllllhhhh


DSSP  --------------------------------llll
Query --------------------------------atkr  292
ident
Sbjct yacwgvmrddilsrfqskeankvnfrdaclmfwkla  302
DSSP  hhhlllhhhhhhhhlllllhhhllhhhhhhhhllll
```

No 683: Query=mol1A Sbjct=2iicC Z-score=2.9

back to top

```
DSSP  ------------------------------------------------------------
Query ------------------------------------------------------------    0
ident
Sbjct daipevkaileakneeelvtftsrwsaeerkelrtqfqdttglefiaflkkcikngpyed   60
DSSP  llhhhhhhhhhlllhhhhhhhhhhllhhhhhhhhhhhhhhhlllhhhhhhllllllhhhh


DSSP  ------LLLL----------------------llllllhhhhhhhhHHHHHHLLLLHHHH
Query ------AAPY----------------------sspptnlrslrdrlTQVAERQGVVFGRL   32
ident                                                   |  |
Sbjct vmalgwDCNIsarvnvikkamknvndfraihdvvliatpderlklaQAYKEKTGNDLLQD  120
DSSP  hhhhhlLLHHhhhhhhhhhhhhlllllhhhhhhhhhlllhhhhhhhhHHHHLLLLLLHHHH


DSSP  HHHHhhhhhhhhhhllllllllleeeehhhhhhhhhllllllllleeeeelllhhhhh
Query QRHVaxivvaqfaatltddtgaplllvkggsslelrrgipdsrtskdfatvarrdielih   92
ident
Sbjct FVDQ--------------------------------------------------------  124
DSSP  HHHH--------------------------------------------------------


DSSP  hhhhhhhhhllllleeeeellleellllllllleeeeeeeeelleeeeeeeeeeeellll
Query eqladagetgwegftaiftapeeiaapgxpvkprrftaklsyrgrafatvpievssveag  152
ident
Sbjct ------------------------------------------------------------  124
DSSP  ------------------------------------------------------------


DSSP  llllleeeeelhhhhllllllleeeeeLLHHHHHHHHHHHHHLlllllllLLLLHHhHHHH
Query nadqfdtltsdalglvgvpaavavpcXTIPWQIAQKLHAVTAvleepkaNDRAHDlVDLQ  212
ident                                  |  |             |
Sbjct -----ipltsaasylchlairenrtpRGSVASDAEVLKHNLI------dADEPDH-EAVV  172
DSSP  -----llllhhhhhhhhlllllllLLLHHHHHHHHHHHLL------lLLLLLH-HHHH


DSSP  HHHHHLLHHHHHHHHHHHHHHHHhhlllLLLL--LLLLlllhhhhhhhhhllllllll
Query LLEGLLLDADLXPTRSACIAIFEaraqhPWPP--RVATlphwpliyagalegldhlelar  270
ident  |
Sbjct RLIITSTADEYKEINHRFEVLTG-----KSVQeaIETR--------------------y  206
DSSP  HHHHLLLHHHHHHHHHHHHHHHL-----LLHHhhHHHH--------------------l
```

```
Query [illegible]
ident      |
Sbjct aDKENARGLCIAHYYNLAparavayafhsavetqnddmayeqaaritglfhdlhkfawvh  266
DSSP  lLHHHHHHHHHHHHHLLLhhhhhhhhhhhhhllllhhhhhhhhhhhhhhhllllllhhhh


DSSP  --------------------------------llll
Query --------------------------------atkr  292
ident
Sbjct yacwgvmrddilsrfqskeankvnfrdaclmfwkla  302
DSSP  hhhlllhhhhhhhhlllllhhhllhhhhhhhhllll
```

No 684: Query=mol1A Sbjct=2w3cA Z-score=2.9

[back to top]

```
DSSP  --------------------------------------------------LLLL---llL
Query --------------------------------------------------AAPY---ssP    7
ident
Sbjct levgiqamehlihvlqtdrsdseiigyaldtlyniisnsqfteifikqqeNVTLllsllE   60
DSSP  lllllllhhhhhhhhhllllhhhhhhhhhhhhhhhhllhhhhhhhhllhhHHHHhhhhlL


DSSP  LLLHHHHHHHHHHHHHHLL-------------llhhhhHHHHhHHHH-------------
Query PTNLRSLRDRLTQVAERQG-------------vvfgrlQRHVaXIVV-------------   41
ident
Sbjct EFDFHVRWPGVKLLTSLLKqlgpqvqqiilvspmgvsrLMDL-LADSrevirndgvlllq  119
DSSP  LLLHHHHHHHHHHHHHHHHhlhhhhhhhhhhlllhhhhHHHH-HHLLlhhhhhhhhhhhh


DSSP  -------hhhhhllllllllllleEEEH-----------HHHHHHHHLllllllllleeee
Query -------aqfaatltddtgapllLVKG-----------GSSLELRRGipdsrtskdfatv   83
ident                                            | |
Sbjct altrsngaiqkivafenaferllDIISeegnsdggivvEDCLILLQN-------------  166
DSSP  hhhlllhhhhhhhhhllhhhhhhHHHHhllhhhllhhhHHHHHHHHH-------------


DSSP  elllhhhhhhhhhhhhhhlllleeeeellleellllllllllleeeeeeeeelleeeeeee
Query arrdieliheqladagetgwegftaiftapeeiaapgxpvkprrftaklsyrgrafatvp  143
ident
Sbjct ------------------------------------------------------------  166
DSSP  ------------------------------------------------------------


DSSP  eeeeelllllllllleeeeelhhhhllllleeeeeelLHHHHhHHHHHHhhlllllllLLL
Query ievssveagnadqfdtltsdalglvgvpaavavpcxTIPWQiAQKLHAvtavleepkAND  203
ident                                      |
Sbjct --------------------llknnnsnqnffkegsYIQRM-KPWFEV---------GWS  196
DSSP  --------------------hhlllhhhhhhhhhllLHHHH-LHHHLL---------LLL


DSSP  L--LHHHHHHHHHHHHL---------LHHHHHHHHHHHhhhhhhhlllllLLLLLLLllh
Query R--AHDLVDLQLLEGLL---------LDADLXPTRSACiaifearaqhpwPPRVATLphw  252
ident       |     |   |
Sbjct AqkVTNLHLMLQLVRVLvsptnppgaTSSCQKAMFQCG-------llqqlCTILMAT---  246
DSSP  HhhHHHHHHHHHHHHHHHlllllhhhHHHHHHHHHHLL-------hhhhhHHHHHLL---


DSSP  hhhhhhhhllllllllllllHHHHHHHHHHHHHHHHHLLLL--------------------
Query pliyagalegldhlelartVDAAAQAVQRFVARIDRATKR--------------------  292
ident                               |    |
Sbjct -----------------gvPADILTETINTVSEVIRGCQVnqdyfasvnapspprpaivv  289
DSSP  -----------------llLHHHHHHHHHHHHHHHLLLHHhhhhhhlleellllleehhhh


DSSP  ------------------------------------------------------------
Query ------------------------------------------------------------  292
ident
Sbjct llmsmvnerqpfvlrcavlycfqcflyknqkgqgeivstllpstidatgnsvsagqllcg  349
DSSP  hhhhhllllllhhhhhhhhhhhhhhhhlllhhhhhhhhhllllllllllllllhhhhhhh


DSSP  ------------------------------------------------------------
Query ------------------------------------------------------------  292
ident
```

```
DSSP  ------------------------------------------------------------
Query ------------------------------------------------------------  292
ident
Sbjct iqtrvgllmllctwlsncpiavthflhnsanvpfltgqiaenlgeeeqlvqglcalllgi  469
DSSP  hhhhhhhhhhhhhhlllhhhhhhhhllllhhhhhhhhhhllllllhhhhhhhhhhhhh
```

```
DSSP  ------------------------------------------------------------
Query ------------------------------------------------------------  292
ident
Sbjct siyfndnslesymkeklkqliekrigkenfieklgfiskhelysrasqkpqpnfpspeym  529
DSSP  hhhllllllllllhhhhhhhhhhllhhhhhhhhhlllllllhhhhllllllllllhhhl
```

```
DSSP  ----------------------
Query ----------------------  292
ident
Sbjct ifdheftklvkelegvitkaiy  551
DSSP  lllhhhhhhhhhhhhhhhhhll
```

No 685: Query=mol1A Sbjct=2iicD Z-score=2.9

back to top

```
DSSP  ------------------------------------------------------------
Query ------------------------------------------------------------    0
ident
Sbjct daipevkaileakneeelvtftsrwsaeerkelrtqfqdttglefiaflkkcikngpyed   60
DSSP  llhhhhhhhhlllhhhhhhhhhhllhhhhhhhhhhhhhhhlllhhhhhllllllhhhh
```

```
DSSP  ------LLLL----------------------llllllhhhhhhhHHHHHHLLLLHHHH
Query ------AAPY----------------------sspptnlrslrdrlTQVAERQGVVFGRL   32
ident                                                   |  |
Sbjct vmalgwDCNIsarvnvikkamknvndfraihdvvliatpderlklaQAYKEKTGNDLLQD  120
DSSP  hhhhhlLLHHhhhhhhhhhhlllllhhhhhhhhhlllhhhhhhhhHHHHLLLLLLHHHH
```

```
DSSP  HHHHhhhhhhhhhhlllllllllleeeehhhhhhhhhllllllllleeeeellhhhhh
Query QRHVaxivvaqfaatltddtgaplllvkggsslelrrgipdsrtskdfatvarrdielih   92
ident
Sbjct FVDQ--------------------------------------------------------  124
DSSP  HHHH--------------------------------------------------------
```

```
DSSP  hhhhhhhhlllleeeeellleellllllllllleeeeeeeeelleeeeeeeeeeeeellll
Query eqladagetgwegftaiftapeeiaapgxpvkprrftaklsyrgrafatvpievssveag  152
ident
Sbjct ------------------------------------------------------------  124
DSSP  ------------------------------------------------------------
```

```
DSSP  lllllleeeeelhhhhllllllleeeeeLLHHHHHHHHHHHHHHLllllllLLLLHHhHHHH
Query nadqfdtltsdalglvgvpaavavpcXTIPWQIAQKLHAVTAvleepkaNDRAHDlVDLQ  212
ident                                  |  |             |
Sbjct -----ipltsaasylchlairenrtpRGSVASDAEVLKHNLI------dADEPDH-EAVV  172
DSSP  -----lllllhhhhhhhhlllllllLLLHHHHHHHHHHHHLL------lLLLLLH-HHHH
```

```
DSSP  HHHHHLLHHHHHHHHHHHHHHHHHhhlllLLLL--LLLLlllhhhhhhhhlllllllll
Query LLEGLLLDADLXPTRSACIAIFEaraqhPWPP--RVATlphwpliyagalegldhlelar  270
ident  |
Sbjct RLIITSTADEYKEINHRFEVLTG-----KSVQeaIETR---------------------y  206
DSSP  HHHHLLLHHHHHHHHHHHHHHHHL-----LLHHhhHHHH---------------------l
```

```
DSSP  lHHHHHHHHHHHHHHHHH------------------------------------------
Query tVDAAAQAVQRFVARIDR------------------------------------------  288
ident      |
Sbjct aDKENARGLCIAHYYNLAparavayafhsavetqnddmayeqaaritglfhdlhkfawvh  266
DSSP  lLHHHHHHHHHHHHHLLLhhhhhhhhhhhhhllllhhhhhhhhhhhhhhhhlllllhhhh
```

```
Sbjct yacwgvmrddilsrfqskeankvnfrdaclmfwkla  302
DSSP  hhhlllhhhhhhhhlllllhhhllhhhhhhhhllll
```

No 686: Query=mol1A Sbjct=4u03A Z-score=2.8

back to top

```
DSSP  llllllllllhhhhhhhhhhhhhhllLLHHHHHHHHHHHHHHHHHH-LLLL---------
Query aapysspptnlrslrdrltqvaerqgVVFGRLQRHVAXIVVAQFAA-TLTD---------   50
ident 
Sbjct ----mtwnfhqyytnrndglmgklvlTDEEKNNLKALRKIIRLRTRdVFEEakgiakavk   56
DSSP  ----lleelhhhhhlllllhhhhhllLHHHHHHHHHHHHHHHHHHHhHHHHhhhhhhhhh


DSSP  -------------------------------------------lLLLLlEEEEHH----h
Query -------------------------------------------dTGAPlLLVKGG----s   63
ident                                              |
Sbjct ksaltfeiiqekvsttqikhlsdseqrevakliyemdddardefLGLT-PRFWTQgsfqy  115
DSSP  hllllhhhhhhhhhhlhhhhllhhhhhhhhhhhhhllhhhhhhhHLLL-LEEEEElhhhh


DSSP  hhhhhhllllllllLLEEEEEL---------lLHHHHHHHHHHHHHHLLLLE-eeEELLL
Query slelrrgipdsrtsKDFATVAR---------rDIELIHEQLADAGETGWEGF-taIFTAP  113
ident                |  |                |                    | |
Sbjct dtlnrpfqpgqemdIDDGTYMPmpifesepkiGHSLLILLVDASLKSLVAENhgwKFEAK  175
DSSP  llllllllllllleeEEEEEEEEellllllllLHHHHHHHHHHHHHHHHLLLlleEEEEL


DSSP  EellllllllllEEEEEEEeellEEEEEEEEEEEEELLLLLLlleeeeelhhhhllllll
Query EeiaapgxpvkpRRFTAKLsyrgRAFATVPIEVSSVEAGNADqfdtltsdalglvgvpaa  173
ident                  |
Sbjct Q-----------TCGRIKI---eAEKTHIDVPMYAIPKDEFQ--------------kkqi  207
DSSP  L-----------LLEEEEE---hHHLEEEEELEEEEEHHHHH--------------hhhh


DSSP  eeeeellhhHHHH------------------hHHHHhhllllllllLLLLhhhhhhhhhh
Query vavpcxtipWQIA------------------qKLHAvtavleepkaNDRAhdlvdlqlle  215
ident                                 |
Sbjct aleanrsfyELDSenvnlalregdrkwinsdpKIVE---------dWFND----------  248
DSSP  hhhhlllllLLLHhhleeelllllllleeellhHHHH---------hHHHH----------


DSSP  hhllhhhhhhhhhhhhhhhhhhhllllllllllLLLHHHHHHHHHLLLLLL---------
Query gllldadlxptrsaciaifearaqhpwpprvatLPHWPLIYAGALEGLDHL---------  266
ident 
Sbjct -------------------------------scIRIGKHLRKVCRFMKAWRdaqwdvggp  277
DSSP  -------------------------------hhHHHLLHHHHHHHHHHHHHhhhllllll


DSSP  ----llllLHHHHHH---------------------------------------------
Query ----elarTVDAAAQ---------------------------------------------  277
ident         ||
Sbjct ssislmaaTVNILDSvahdasdlgetmkiiakhlpsefargvespdstdekplfppsykh  337
DSSP  lhhhhhhhHHHHHHHllllllhhhhhhhhhhhhhhhhhhlllllllllllllllllhhhl


DSSP  -------------------------------------hhhhhhhhhhhllll
Query -------------------------------------avqrfvaridratkr  292
ident 
Sbjct gpremdimsklerlpeilssaesadksealkkinmafgnrvtnselivlakx  390
DSSP  lhhhhhhhhhhhhhhhhhhhhllllhhhhhhhhhhhhllllllhhhleelll
```

No 687: Query=mol1A Sbjct=4kf8B Z-score=2.8

back to top

```
DSSP  llllllllllhhhhhhhhhhhhhhllllhhhhhhhhhhhhhhhHHHLLllllllllEEEE
Query aapysspptnlrslrdrltqvaerqgvvfgrlqrhvaxivvaqFAATLtddtgaplLLVK   60
ident                                                          ||
Sbjct -------------------------------------------LLPKD---isqllSLVS   14
DSSP  -------------------------------------------LLLLL---hhhhhHHHH
```

```
Query -----------------gGSSLELRRGipdsrtskdfatvarrdieliheqladagetgw  103
ident                      |
Sbjct atingvdnpwskdqisyfRTLLKVLFV---------------------------------   41
DSSP  hhhlllllllllllhhhhHHHHHHHHH---------------------------------


DSSP  lleeeeellleellllllllllleeeeeeeeelleeeeeeeeeeeelllllLLLLeeeeel
Query egftaiftapeeiaapgxpvkprrftaklsyrgrafatvpievssveagnADQFdtltsd  163
ident                                                     |
Sbjct -----------------------------------------vlrgtkhsaVTQL------   54
DSSP  -----------------------------------------hhhllllllHHHH------


DSSP  hhhhllllleeeeeelLHHH-HHHHHHHHHHLLLllllLLLLLHHHHHHHHHHHHL----
Query alglvgvpaavavpcxTIPW-QIAQKLHAVTAVLeepkANDRAHDLVDLQLLEGLL----  218
ident                 |      |       |            ||
Sbjct --------------vlTTLDrVVARSFRNLAALV----HATTPEDLALITAILQAClsvp   96
DSSP  --------------hhHHHHhLHHHHHHHHLLLL----LLLLHHHHHHHHHHHHHHlllh


DSSP  -LHHHHHHHHHHHHHHHhhhllllLLLL--LLLLllhhhhhhhhllllllllllLLHHHH
Query -LDADLXPTRSACIAIFearaqhpWPPR--VATLphwpliyagalegldhlelaRTVDAA  275
ident
Sbjct gIEQCQLQVLNIXSSHN------vLQVAtsLFSW---------------sdrgdPIYGEL  135
DSSP  hHHHLHHHHHHHHHHLL------hHHHHhhHHHH---------------lllllLHHHHH


DSSP  HHHHHHHHHHhHHLL---------------------------------------------
Query AQAVQRFVARiDRAT---------------------------------------------  290
ident |            |
Sbjct ALLLLLELSA-LPALaeqlacdgllghltsanlagfxrrannagaarcyaiwakgilpll  194
DSSP  HHHHHHHHLL-LHHHhhhhhhhlhhhhhhhllhhhhhhlllllllhhhhhhhhhlhhhhh


DSSP  ------------------------------------------------------------
Query ------------------------------------------------------------  290
ident
Sbjct lnilgaltiapevafvlnqfpnllrssvdralvalsevhslalltrvlaakavgllegir  254
DSSP  hhhhlllllhhhhhhhhhhllhhhhhlllllllhhhhhhhhhhhhhhhllhhhhhhhhhh


DSSP  --ll
Query --kr  292
ident
Sbjct dvla  258
DSSP  hhhl
```

No 688: Query=mol1A Sbjct=1n00A Z-score=2.8

back to top

```
DSSP  --------LLLLLLL--------------------------lllhhHHHHhhhhhhhhll
Query --------AAPYSSP--------------------------ptnlrSLRDrltqvaerqg   26
ident              |
Sbjct hhhatltvPTTVPSVsedceqlrkafsgwgtnegliidilghrnaeQRNL----------   50
DSSP  llllllllLLLLLLHhhhhhhhhhhlllllllhhhhhhhhllllhhHHHH----------


DSSP  llhhhhhhhhhhhhhhhhhhlllllllllleeeehhhhhhhhhlllllllllleeeeell
Query vvfgrlqrhvaxivvaqfaatltddtgaplllvkggsslelrrgipdsrtskdfatvarr   86
ident
Sbjct ------------------------------------------------------------   50
DSSP  ------------------------------------------------------------


DSSP  lhhhhhhhhhhhhhhhllleeeeellleellllllllllleeeeeeeEELLE---------
Query dieliheqladagetgwegftaiftapeeiaapgxpvkprrftaklSYRGR---------  137
ident
Sbjct -----------------------------------irktyaetygeDLLKAldkelsndf   75
DSSP  -----------------------------------hhhhhhhhhllLHHHhhhhllhhh


DSSP  --------------------------------------------------eeeeeeeeee
Query --------------------------------------------------afatvpievs  147
```

```
DSSP  ellllllllleeeeelhhhhlllllleeeeelLHHHHHHHHHHHHHLlllllllllLLHH
Query sveagnadqfdtltsdalglvgvpaavavpcxTIPWQIAQKLHAVTAvleepkandRAHD  207
ident                                 |     |  ||
Sbjct eedvahhttgdfhklllplvssyryegeevnmTLAKTEAKLLHEKIS---------NKAY  186
DSSP  hhhhhhllhhhhhhhhhhhhhllllllllllhHHHHHHHHHHHHHHH---------LLLL


DSSP  HH-HHHHHHHHLLHHHHHHHHHHHHHHHHHhhllllLLLL--LLLLllhhhhhhhhllll
Query LV-DLQLLEGLLLDADLXPTRSACIAIFEaraqhpWPPR--VATLphwpliyagalegld  264
ident    |          |    |
Sbjct SDdDVIRVLATRSKAQINATLNHYKNEYG------NDINkdLKAD---------------  225
DSSP  LLhHHHHHHHHLLHHHHHHHHHHHHHHHL------LLLLhhHLLL---------------


DSSP  lllllllhHHHHHHHHHHHHHHHH------------------------------------
Query hlelartvDAAAQAVQRFVARIDR------------------------------------  288
ident         |         |
Sbjct ------pkDEFLALLRSTVKCLVYpekyfekvlrlainrrgtdegaltrvvctraevdlk  279
DSSP  ------llLHHHHHHHHHHHHHHLhhhhhhhhhhhhhllllllllhhhhhhhhhllllhh


DSSP  -----------------------------------llll
Query -----------------------------------atkr  292
ident
Sbjct viadeyqrrnsvpltraivkdthgdyeklllvlaghven  318
DSSP  hhhhhhhhhhlllhhhhllllllhhhhhhhhhhllllll
```

No 689: Query=mol1A Sbjct=3qmlD Z-score=2.8

back to top

```
DSSP  lllllllllhhhhhhhhhhhhhhllllhhhhhhhhHHHHHHHhhhhlllllllllleeee
Query aapysspptnlrslrdrltqvaerqgvvfgrlqrhVAXIVVAqfaatltddtgaplllvk   60
ident
Sbjct ---------------------------------efSSDFKEM------------------    9
DSSP  ---------------------------------llHHHHHHH------------------


DSSP  hhhhhhhhhlllllllllleeeeelllhhhhHHHHhhhhhhhhlllleeeeellleellll
Query ggsslelrrgipdsrtskdfatvarrdielIHEQladagetgwegftaiftapeeiaapg  120
ident
Sbjct --------------------------rniiDSNP--------------------------   17
DSSP  --------------------------hhllLLLL--------------------------


DSSP  llllleEEEEeeeelleeeeeeeeeeeelllllllllleEEEElhhhhlllllleEEEE--
Query xpvkprRFTAklsyrgrafatvpievssveagnadqfdTLTSdalglvgvpaavAVPC--  178
ident                                         |
Sbjct -tlssqDIAR--------ledsfdrimefahdykhgykIITH------------EFALla   56
DSSP  -lllhhHHHH--------hhhhhhhhhhhhllhhhhhhHHHH------------HHHHhh


DSSP  ---------------------------------------lLHHHHHHHHhHHHHLLllll
Query ---------------------------------------xTIPWQIAQKlHAVTAVleep  199
ident                                               |
Sbjct nlslnenlpltlrelstrvitsclrnnppvvefinesfpnFKSKIMAAL-SNLNDS----  111
DSSP  hhhhllllhhhhhhhhhhhhhhllllhhhhhhhhlllHHHHHHHHH-HHHHLL----


DSSP  lllLLLHHHHHHHHHHHHLLHH-hHHHHHhhhhhhhhhhlllllLLLL--LLLLlhhhhh
Query kanDRAHDLVDLQLLEGLLLDA-dLXPTRsaciaifearaqhpwPPRV--ATLPhwpliy  256
ident                   |       |                    |
Sbjct ---SSNILIKRYLSILNELPVTseDLPIY-----------stvvLQNVyeRNNK------  151
DSSP  ---LLHHHHHHHHHHHHHLLLLhhHHHHL-----------lhhhHHHHhhLLLL------


DSSP  hhhhlllllllllllHHHHHHHHHHH----------hhhhhhLLLL--------------
Query agalegldhlelartVDAAAQAVQRF----------varidrATKR--------------  292
ident                       |
Sbjct ---------------DKQLQIKVLELiskilkadmnlilfkrNAENwssnlqewanefqe  196
DSSP  ---------------LHHHHHHHHHHhhhhhlllllhhhhhhHHHLlllllhhhhhhhhh
```

```
ident
Sbjct mvqnksidelhtrtffdtlynlkkifksditinkgflnwlaqqckarqsnldnglqerdt  256
DSSP  hllllllhhhhhhhhhhhhhhhhhhhhhhllllllhhhhhhhhhhhhhhhhhllllllll


DSSP  -----------------
Query -----------------  292
ident
Sbjct eqdsfdkklidsrhlif  273
DSSP  hhhhhhhhhhhhhhhll
```

No 690: Query=mol1A Sbjct=3ovbB Z-score=2.8

back to top

```
DSSP  lllllllllhhhhhhhhhhhhhhlLLLHHHHHHHHHHHHHHHHHHLLLlllllllEEEE
Query aapysspptnlrslrdrltqvaerqGVVFGRLQRHVAXIVVAQFAATLTddtgaplLLVK   60
ident                                     |          |
Sbjct ----------mkveeilekalelviPDEEEVRKGREAEEELRRRLDELG-------VEYV   43
DSSP  ----------llhhhhhhhlhhhhlLLHHHHHHHHHHHHHHHHHHHLLLL-------LLEE


DSSP  HHhhhhhhhllllllllLLEEEEEEL------LLHHHHHHHHHHHHHhhlllleeEEELLLE
Query GGsslelrrgipdsrtsKDFATVAR------RDIELIHEQLADAGetgwegftAIFTAPE  114
ident                   |               |   |
Sbjct FV-gsyarntwlkgsleIDVFLLFPeefskeELRERGLEIGKAVL--------DSYEIRY   94
DSSP  EE-hhhhhllllllllleEEEEEEELllllhhHHHHHHHHHHHHHL--------LEEELLL


DSSP  ellllllllLLEEEEEEEEellee EEEEEEEEEEELLLLLllll eeeeelhhhhlllllle
Query eiaapgxpvKPRRFTAKLSyrgraFATVPIEVSSVEAGNadqfdtltsdalglvgvpaav  174
ident                            |
Sbjct --------aEHPYVHGVVK-----GVEVDVVPCYKLKEP---------------------  120
DSSP  --------lLLLLEEEEEL-----LEEEEEEEEELLLLL---------------------


DSSP  eeeellhhhhhhHHHHHHhllllllllLLLLhhhhhhhhhhhhllhhhhhhhhhhhhhh
Query avpcxtipwqiaQKLHAVtavleepkaNDRAhdlvdlqlleglllldadlxptrsaciaif  234
ident                              |
Sbjct -kniksavdrtpFHHKWL---------EGRI-----------------------------  141
DSSP  -lllllllllhhHHHHHH---------HHHL-----------------------------


DSSP  hhhlllllllllllllhhhHHHHHHLL-LLLL-------------------llllLHHH
Query earaqhpwpprvatlphwplIYAGALEG-LDHL-------------------elarTVDA  274
ident                                 |                        |
Sbjct -------------------kGKENEVRLlKGFLkangiygaeykvrgfsgylcellIVFY  182
DSSP  -------------------lLLHHHHHHhHHHHhhlllllllllllllhhhhhhhHHHH


DSSP  H----------------hhHHHHH------------------------------------
Query A----------------aqAVQRF------------------------------------  282
ident                     |
Sbjct GsfletvknarrwtrrtviDVAKGevrkgeeffvvdpvdekrnvaanlsldnlarfvhlc  242
DSSP  LlhhhhhhhhllllllleeeELLLLeeeellllleeeelleeeeellllllhhhhhhhhhh


DSSP  ------------------------------------------------------------
Query ------------------------------------------------------------  282
ident
Sbjct refmeapslgffkpkhpleieperlrkiveergtavfavkfrkpdivddnlypqlerasr  302
DSSP  hhhhhlllhhhllllllllllhhhhhhhhhhhlleeeeeeeelllllhhhhhhhhhhhh


DSSP  ------------------------------------------------------------
Query ------------------------------------------------------------  282
ident
Sbjct kifeflerenfmplrsafkaseefcyllfecqikeisrvfrrmgpqfedernvkkflsrn  362
DSSP  hhhhhhhhllllleeeeeeeellleeeeeeeelllllllleeeeeeeellllhhhhhhhhll


DSSP  ------------------------------------------------------------
Query ------------------------------------------------------------  282
ident
```

```
DSSP  -----hhhhhhllll
Query -----varidratkr  292
ident
Sbjct epvtaelcemmgvkd  437
DSSP  lllhhhhhhhhllll
```

No 691: Query=mol1A Sbjct=2b51A Z-score=2.8

back to top

```
DSSP  llllllllllhhhhhhhhhhhhhhllLLHHHHHHHHHHHHHHHHHHHLLLlllllLLEEEE
Query aapyssspptnlrslrdrltqvaerqggVVFGRLQRHVAXIVVAQFAATLTddtgaPLLLVK   60
ident                                     |       |   |      |   |
Sbjct ---npspdhyavwgkaimaennrrvgPEHMFRTAIRAQQQLQGLADKWT-----PDAKVY   52
DSSP  ---lllhhhhhhhhhhhhhhhhhhllLHHHHHHHHHHHHHHHHHHHHHL-----LLLEEE


DSSP  HHhhhhhhhllllllllLLEEEEEL------lLHHHHHHHHHHHHHHHLLL-leeeEELLL
Query GGsslelrrgipdsrtsKDFATVAR------rDIELIHEQLADAGETGWE-gftaIFTAP  113
ident                   | |                   |
Sbjct CC-gsmvtygqmergsdLDLACMFDdpypsheVQAKRTDKLRTVIKRYVPhylrnNLLGL  111
DSSP  EE-lhhhhhlllllllEEEEEELLlllllhhHHHHHHHHHHHHHHHLLLLhhhhlLEEEL


DSSP  EELlllllllLLEEEEEEE------------------------------------------
Query EEIaapgxpvKPRRFTAK------------------------------------------  131
ident  |
Sbjct TEA-------RTPVVKLRfandekvararytplseeedrkartalldvrnqcvgdndvey  164
DSSP  LLL-------LLLEEEELlllhhhhhhhhhllllhhhhhhhleeeeeeelllllhhhhhh


DSSP  ----------------------------------------------------------ee
Query ----------------------------------------------------------ls  133
ident
Sbjct iaekmgrdnvegirvdrttygcriaiqctskeqmieaigffpdgkimtrgmredytrdvl  224
DSSP  hhhhhlhhheeeeeeelllleeeeeeeellhhhhhhhhllllllllllhhhhhheeeeel


DSSP  eLLEE----EEEEEEEEEellllllllleeeeelhhhhlllllleeeeellhhhhhhHHH
Query yRGRA----FATVPIEVSsveagnadqfdtltsdalglvgvpaavavpcxtipwqiaQKL  189
ident               |
Sbjct dVRFVpemfMYRWDISFV-----------------------------------gygvKNS  249
DSSP  lLLLLlhhhLLLEEEELL-----------------------------------lhhhHHH


DSSP  HHHhlllllllllLLLHhhhhhhhhhhhllhhhhhhhhhhhhhhhhhlllllllllll
Query HAVtavleepkanDRAHdlvdlqllegllldadlxptrsaciaifearaqhpwpprvatl  249
ident
Sbjct YLI----------RHYL-------------------------------------------  256
DSSP  HHH----------HHHH-------------------------------------------


DSSP  lLHHHhHHHHHLL-LLLL-----------------llllLHHHHHH-------------
Query pHWPLiYAGALEG-LDHL-----------------elarTVDAAAQ-------------  277
ident    |
Sbjct hNGPVaARHTAMAvKAWGkatnvgagsgamltsyavtvmFIYYLLVtrqvlwvdpwslph  316
DSSP  hLLLLlHHHHHHHhHHHHhllllllllllllhhhhhhhHHHHHHHllllllllhhhlll


DSSP  -------------------------------------------hHHHH-hhhhhHLLLL-
Query -------------------------------------------aVQRF-varidRATKR-  292
ident                                               |
Sbjct pahlprypdfsplydcdptelgrllhgffifyahhfdyerevvsLNRNrrsyrsDIGWNf  376
DSSP  hhhlllllllllllllhhhhhhhhhhhhhhhhhhllllllleeeeLLLLlleehhHHLLLl


DSSP  ------------------------------------------------------------
Query ------------------------------------------------------------  292
ident
Sbjct pqnkkgtfsynfciedpyedvgtgglnlvrhlhpakfqlvkqeflraaqcmerflptnap  436
DSSP  hhheelleellllleellllllllllllllllhhhhhhhhhhhhhhhhhhhhhlllllll
```

```
Sbjct eksilgvk  444
DSSP  llhhhlll
```

No 692: Query=mol1A Sbjct=4dvgB Z-score=2.7

back to top

```
DSSP  lllllllllhhhhhhhhhhhhhhllllhhhhhhhhhhhhhhhhhhllllllllLEEEe
Query aapysspptnlrslrdrltqvaerqgvvfgrlqrhvaxivvaqfaatltddtgapLLLVk   60
ident                                                        |
Sbjct ----------evvqqkfaivakexkidnpelitipnqwklvqeyekkqkkdiriqLNAQ-   49
DSSP  ----------llllhhhhhhhhhllllllhhhllhhhhhhhhhhhhhhhhhhhhhhHHHH-


DSSP  hhhhhhhhhlllllllllEEEEelllhhhhhhhhhhhhhhllllleeeeellleelllll
Query ggsslelrrgipdsrtskdFATVarrdieliheqladagetgwegftaiftapeeiaapg  120
ident
Sbjct -------------------KTGN-------------------------------------   53
DSSP  -------------------HHLL-------------------------------------


DSSP  lllllleeeeeeeeelleeeeeeeeeeellllllllleeeeelhhhhllllllleeeeELL
Query xpvkprrftaklsyrgrafatvpievssveagnadqfdtltsdalglvgvpaavavpCXT  180
ident
Sbjct -------------------------------------------------------wrNAI   58
DSSP  -------------------------------------------------------hhHHL


DSSP  H-HHHHHHHHHHHhllllllllllllhHHHHHHHHHHHL-LHHHhHHHHHHHHHHHHhhhl
Query I-PWQIAQKLHAVtavleepkandrahDLVDLQLLEGLL-LDADlXPTRSACIAIFeara  238
ident   |   |  |                 |  ||
Sbjct TdPKYLADLLKTR--------------DDXDLLNEXVVVfRSSS-VSFIKTFVSVG----   99
DSSP  LlHHHHHHHLLLL--------------LLHHHHHHHHHHhHHLL-HHHHHHHHHLL----


DSSP  lllllLLLL---LLLLHHhhhhhhhlllllllllLLHHHHHHHHHHHHHHHHHHLLL----
Query qhpwpPRVA---TLPHWPliyagalegldhlelaRTVDAAAQAVQRFVARIDRATK----  291
ident                                    |
Sbjct glanlXAIYkkkIEAENS----------------NTAIDEERKCCEVLRYVFAEEDatva  143
DSSP  lhhhhHHHHhhhHHLLLL----------------HHHHHHHHHHHHHHHHHHLLLHHhhhh


DSSP  ------------------------------------------------------------
Query ------------------------------------------------------------  291
ident
Sbjct lieidggvelllkgxnskritpdnqldilleitltssxvehpsqeglylggdvcvxnafs  203
DSSP  hhllllhhhhhhhhhllllllhhhhhhhhhhhhhhhhllllllllllllhhhhhhhhh


DSSP  ------------------------------------------------------------
Query ------------------------------------------------------------  291
ident
Sbjct nlvsegvdxkkflsffslfsksksekfkhaslvlinnlidqpelehrxdvrnsfieiglv  263
DSSP  hhhhlllllllhhhhhhhhhlllhhhhhhhhhhhhhhhllllhhhhhhhhhhhhlllhh


DSSP  -----------------------------------------l
Query -----------------------------------------r  292
ident
Sbjct nelenxkntewxkidkikdsindffdsweedkkevesrfddl  305
DSSP  hhhhhhhllhhhhlhhhhhhhhhhhhhhhhhhhhhhhhhlll
```

No 693: Query=mol1A Sbjct=3ovbA Z-score=2.7

back to top

```
DSSP  lllllllllhhhhhhhhhhhhhhlLLLHHHHHHHHHHHHHHHHHHHHLLLlllllllEEEE
Query aapysspptnlrslrdrltqvaerqGVVFGRLQRHVAXIVVAQFAATLTddtgaplLLVK   60
ident                                     |          |
Sbjct ----------mkveeilekalelviPDEEEVRKGREAEEELRRRLDELG-------VEYV   43
DSSP  ----------llhhhhhhhhhhhhlLLHHHHHHHHHHHHHHHHHHHHHHL-------LLEE
```

```
Query GGsslelrrgipdsrtsKDFATVAR------RDIELIHEQLADAGetgwegftAIFTAPE  114
ident                   |               |   |
Sbjct FV-gsyarntwlkgsleIDVFLLFPeefskeELRERGLEIGKAVL--------DSYEIRY   94
DSSP  EE-hhhhhlllllllleEEEEEEELllllhhHHHHHHHHHHHHHL--------LEEELLL


DSSP  ellllllllLLEEEEEEEEelleeEEEEEEEEEELLLLLllllleeeeelhhhhllllle
Query eiaapgxpvKPRRFTAKLSyrgraFATVPIEVSSVEAGNadqfdtltsdalglvgvpaav  174
ident                            |
Sbjct --------aEHPYVHGVVK-----GVEVDVVPCYKLKEP---------------------  120
DSSP  --------lLLLLEEEEEL-----LEEEEEEEEELLLLL---------------------


DSSP  eeeellhhhhhhHHHHHHhllllllllLLLLhhhhhhhhhhhhllhhhhhhhhhhhhhhh
Query avpcxtipwqiaQKLHAVtavleepkaNDRAhdlvdlqlleglllddadlxptrsaciaif  234
ident                              |
Sbjct -kniksavdrtpFHHKWL---------EGRI-----------------------------  141
DSSP  -lllllllllhhHHHHHH---------HLLL-----------------------------


DSSP  hhhllllllllllllllhhhHHHHHHLL-LLLL-------------------llllLHHH
Query earaqhpwpprvatlphwplIYAGALEG-LDHL-------------------elarTVDA  274
ident                                 |                       |
Sbjct -------------------kGKENEVRLlKGFLkangiygaeykvrgfsgylcellIVFY  182
DSSP  -------------------lLLHHHHHHhHHHHhhllllllllllllllhhhhhhhHHHH


DSSP  H----------------hhHHHHH------------------------------------
Query A----------------aqAVQRF------------------------------------  282
ident                    |
Sbjct GsfletvknarrwtrrtviDVAKGevrkgeeffvvdpvdekrnvaanlsldnlarfvhlc  242
DSSP  LlhhhhhhhhlllllleeeEHHHLeeeellllleeeelleeeeelllllhhhhhhhhhhh


DSSP  ------------------------------------------------------------
Query ------------------------------------------------------------  282
ident
Sbjct refmeapslgffkpkhpleieperlrkiveergtavfavkfrkpdivddnlypqlerasr  302
DSSP  hhhhhlllhhhhllleellllhhhhhhhhhhhhlleeeeeeeelllllhhhhhhhhhhhhh


DSSP  ------------------------------------------------------------
Query ------------------------------------------------------------  282
ident
Sbjct kifeflerenfmplrsafkaseefcyllfecqikeisrvfrrmgpqfedernvkkflsrn  362
DSSP  hhhhhhhhlllleeeeeeeeellleeeeeeeeellllllleeeeeeeeelllhhhhhhhhhll


DSSP  ------------------------------------------------------------
Query ------------------------------------------------------------  282
ident
Sbjct rafrpfiengrwwafemrkfttpeegvrsyasthwhtlgknvgesireyfeiisgeklfk  422
DSSP  llllleeelleeeeeeeelllllhhhhhhhhhhhlhhhllhhhhhhhhhlleeeehhhhhl


DSSP  ---------hhhhhhllll
Query ---------varidratkr  292
ident
Sbjct epvtaelcemmgvkdcccq  441
DSSP  lllhhhhhhhhllllleel
```

No 694: Query=mol1A Sbjct=3evhA Z-score=2.7

back to top

```
DSSP  llllllllllhhhhhhhhhhhhhhllllhhhhhhhhhhhhhhhhhllllllllllleeee
Query aapysspptnlrslrdrltqvaerqgvvfgrlqrhvaxivvaqfaatltddtgaplllvk   60
ident
Sbjct -------------gshpfdqavvkdptasyvdvkarrtflqsgqlddrlkaalpkeydct   47
DSSP  -------------lllllllllllllllllllhhhhhhhhhhllllhhhhhhhllllllhh


DSSP  hHHHHhhhhlllllllllleeeeELLLHHHHHhhhhhhhhhllllleeeeellleelllll
Query gGSSLelrrgipdsrtskdfatvARRDIELIHeqladagetgwegftaiftapeeiaapg  120
```

```
DSSP  llllleeeeeeeeeelleeeeeeeeeeeelllllLLLLeeeeelhhhhlllllleeeeELL
Query xpvkprrftaklsyrgrafatvpievssveagnADQFdtltsdalglvgvpaavavpCXT  180
ident
Sbjct ----------------------------pvnpdYEPV--------------------VTL   83
DSSP  ----------------------------lllllHHHH--------------------HHH


DSSP  HhHHHHHHHHHHHLLLLllLLLL--LLHHHHHHHHHHHH-LLHH------hhHHHHHHHH
Query IpWQIAQKLHAVTAVLEepKAND--RAHDLVDLQLLEGL-LLDA------dlXPTRSACI  231
ident                              |  |        |
Sbjct Y-RDFEKISATLGNLYV-aTGKPvyATCLLNMLDKWAKAdALLNydpksqswYQVEWSAA  141
DSSP  H-HHHHHHHHHHHHHHH-hHLLHhhHHHHHHHHHHHHHLlLLLLlllllhhhHHHHHHHH


DSSP  hhhhhhlllllLLLL---LLLLLHhhhhhhhhlllllllllllHHHHHHHHHHHHHHHHH
Query aifearaqhpwPPRV---ATLPHWpliyagalegldhlelartVDAAAQAVQRFVARIDR  288
ident                      |                        |    |     |  |
Sbjct ---------taAFALstmMAEPNV-------------------DTAQRERVVKWLNRVAR  173
DSSP  ---------hhHHHHhllLLLLLL-------------------LHHHHHHHHHHHHHHHH


DSSP  -LLLL-------------------------------------------------------
Query -ATKR-------------------------------------------------------  292
ident   |
Sbjct hQTSFpggdtsccnnasywrgqeatiigviskddelfrwglgryvqamglinedgsfvhe  233
DSSP  hHLLLlllllllllhhhhhhhhhhhhhhhhhhllhhhhhhhhhhhhhhhllllllllllh


DSSP  ------------------------------------------------------------
Query ------------------------------------------------------------  292
ident
Sbjct mtrheqslhyqnyamlpltmiaetasrqgidlyaykengrdihsarkfvfaavknpdlik  293
DSSP  hhlhhhhhhhhhhhhhhhhhhhhhhhlllllhhhllllllhhhhhhhhhhhhhhllhhhh


DSSP  ------------------------------------------------------------
Query ------------------------------------------------------------  292
ident
Sbjct kyasepqdtrafkpgrgdlnwieyqrarfgfadelgfmtvpifdprtggsatllaykpqg  353
DSSP  llllllllllllllllhhhhhhhhhhhhhlllllllllllleehhhleehhhhlllllll
```

No 695: Query=mol1A Sbjct=5hc9A Z-score=2.6

back to top

```
DSSP  llllllllllhhhhhhhhhhhhhhllllhHHHHhhHHHHHHHHHHHLLlllllLLLEEEE
Query aapysspptnlrslrdrltqvaerqgvvfGRLQrhVAXIVVAQFAATLtddtgAPLLLVK   60
ident                               |                           |
Sbjct -----------------mqifrdvskllvERVD--PKILNLFRLLGKF---gdEVNMPVY   38
DSSP  -----------------llleeelhhhhhHHLL--HHHHHHHHHHHHH---hhHLLLLEE


DSSP  hhHHHHHHHLLLL--llllLLEEEEEllLHHHHHHHHHHHHhhlllleEEEELLLEELll
Query ggSSLELRRGIPD--srtsKDFATVArrDIELIHEQLADAGetgwegfTAIFTAPEEIaa  118
ident         |           |             |
Sbjct --VVGGFVRDLLLgiknldIDIVVEG--NALEFAEYAKRFL-------PGKLVKHDKF--   85
DSSP  --EELHHHHHHHHllllllEEEEELL--LHHHHHHHHHHHL-------LEEEEEELLL--


DSSP  lllllllEEEEEEEEelleEEEEEEEEEEE------lLLLLlllleeeeelhhhhlllll
Query pgxpvkpRRFTAKLSyrgrAFATVPIEVSS------vEAGNadqfdtltsdalglvgvpa  172
ident              |           |
Sbjct -------MTASLFLK----GGLRIDIATARleyyespAKLP-------------------  115
DSSP  -------LEEEEEEL----LLLEEEEEELLlllllllLLLL-------------------


DSSP  leeeeellHHHHhHHHHhhhhllllllllllLLHH----------------hhhHHHHhh
Query avavpcxtIPWQiAQKLhavtavleepkandRAHD----------------lvdLQLLeg  216
ident                |               |
Sbjct -------dVEMS-TIKK----------dlyrRDFTinamaiklnpkdfgllidfFGGY--  155
DSSP  -------lHHHH-HHHH----------hhllLLLHhhlleeellllllleeellLLHH--
```

```
ident
Sbjct ---------------------rdlkegvirvlHTLSFVDdptrilrairfeqRFDFRIEe  194
DSSP  ---------------------hhhhlleellLLLHHHHlllhhhhhhhhhhHLLLEELh


DSSP  ------------------------------------------------------------
Query ------------------------------------------------------------  271
ident
Sbjct tterllkqaveegylerttgprlrqelekileeknplksirrmaqfdvikhlfpktyytp  254
DSSP  hhhhhhhhhhllhhhhllhhhhhhhhhhhhllllhhhhhhhhhhllhhhhhlllllllh


DSSP  ------------------------------------------------------------
Query ------------------------------------------------------------  271
ident
Sbjct smdekmenlfrnipwveenfgevdrfyavlhvflefyddeswkevrdryslrrnlineir  314
DSSP  hhhhhhhhhhhhhhhhhhhhlllllhhhhhhhhhlllllhhhhhhhhhhllllllhhhhhh


DSSP  ------------------------------------------------------------
Query ------------------------------------------------------------  271
ident
Sbjct hveksapallemlservpasfvyplvkgvsnetichflaylsgekeglfksyllkikntk  374
DSSP  hhhhhhhhhhhhhhhllllhhhhhhhhhllllhhhhhhhhhllllhhhhhhhhhhhhhhhhlll


DSSP  ------------------------------hhhhhhhhhhhhhhhhhllll
Query ------------------------------vdaaaqavqrfvaridratkr  292
ident
Sbjct lekingeylirkgitsgkiigevlekilmkkldgdtrdeeeileevlasle  425
DSSP  llllhhhhhhhllllllhhhhhhhhhhhhhhhhlllllhhhhhhhhhhhhl
```

No 696: Query=mol1A Sbjct=3gq2A Z-score=2.6

back to top

```
DSSP  lllllllllhhhhhhhhhhhhhhllllHHHHHHHHHHHHH-------hhHHHLLlllll
Query aapyssspptnlrslrdrltqvaerqgvvFGRLQRHVAXIVV-------aqFAATLtddtg   53
ident                                  |  |
Sbjct -----------hteaetiqklcdrvassTLLDDRRNAVRALkslskkyrlEVGIQ-----   44
DSSP  -----------llllhhhhhhhhhhhhlLLHHHHHHHHHHHHhhllllllhhHHHHH-----


DSSP  lllEEEE------------HHHHHHHHhllllllllllleeeeelllhhhhhhhhhhhhhh
Query aplLLVK------------GGSSLELRrgipdsrtskdfatvarrdieliheqladaget  101
ident                     |  |
Sbjct -amEHLIhvlqtdrsdseiIGYALDTL---------------------------------   70
DSSP  -hhHHHHhhhllllllhhhHHHHHHHH---------------------------------


DSSP  llleeeeellleellllllllllleeeeeeeeeelleeeeeeeeeeeellllLLLLeeee
Query gwegftaiftapeeiaapgxpvkprrftaklsyrgrafatvpievssveagnADQFdtlt  161
ident
Sbjct --------------------------------yniisndeeeedlgsqfteiFIKQ----   94
DSSP  --------------------------------hhhhllllllllhhhhhhhhHHLL----


DSSP  elhhhhlllllleeeeelLHHHHHHHHHhhhhllllllllLLLHHHHHHHHHHhHLLHH
Query sdalglvgvpaavavpcxTIPWQIAQKLhavtavleepkanDRAHDLVDLQLLEgLLLDA  221
ident                            |                |       |   ||
Sbjct -----------------qENVTLLLSLL-----------eeFDFHVRWPGVKLLtSLLKQ  126
DSSP  -----------------hHHHHLHHHHL-----------llLLHHHHHHHHHHHhHHHHH


DSSP  HHHHHHHHHHHHHHhhlllLLLL--LLLLlllhhhhhhhhllllllllllLHHHHHHHHH
Query DLXPTRSACIAIFEaraqhPWPP--RVATlphwpliyagalegldhlelarTVDAAAQAV  279
ident
Sbjct LGPQVQQIILVSPM-----GVSRlmDLLA---------------------dSREVIRNDG  160
DSSP  LHHHHHHHHHHLLL-----HHHHhhHHHH---------------------lLLHHHHHHH


DSSP  HHHHHHHHHLLLL-----------------------------------------------
Query QRFVARIDRATKR-----------------------------------------------  292
ident         |
```

```
DSSP  ------------------------------------------------------------
Query ------------------------------------------------------------  292
ident 
Sbjct qnffkegsyiqrmkpwfevgdensgwsaqkvtnlhlmlqlvrvlvspnnppgatsscqka  280
DSSP  hhhhhhlllhhhlhhhhlllllllllhhhhhhhhhhhhhhhhhhlllllhhhhhhhhhh


DSSP  ------------------------------------------------------------
Query ------------------------------------------------------------  292
ident 
Sbjct mfqcgllqqlctilmatgvpadiltetintvsevirgcqvnqdyfasvnapsnpprpaiv  340
DSSP  hhhllhhhhhhhhhhlllllhhhhhhhhhhhhhhhlllhhhhhhhhhllllllllllhhh


DSSP  ------------------------------------------------------------
Query ------------------------------------------------------------  292
ident 
Sbjct vllmsmvnerqpfvlrcavlycfqcflyknqkgqgeivstllpstidatgntvsagqllc  400
DSSP  hhhhhhlllllhhhhhhhhhhhhhhhlllhhhhhhhhhhhllllllllllllhhhhhh


DSSP  ------------------------------------------------------------
Query ------------------------------------------------------------  292
ident 
Sbjct gglfstdslsnwcaavalahalqenatqkeqllrvqlatsignppvsllqqctnilsqgs  460
DSSP  hhhhlllhhhhhhhhhhhhhhhlllhhhhhhhllleellllllllleehhhhhhhhhllll


DSSP  ------------------------------------------------------------
Query ------------------------------------------------------------  292
ident 
Sbjct kiqtrvgllmllctwlsncpiavthflhnsanvpfltgqiaenlgeeeqlvqglcalllg  520
DSSP  lhhhhhhhhhhhhhhhhlllhhhhhhhhhlllhhhhhhhhhhllllhhhhhhhhhhhhhh


DSSP  ------------------------------------------------------------
Query ------------------------------------------------------------  292
ident 
Sbjct isiyfndnsletymkeklkqliekrigkenfieklgfiskhelysrasqkpqpnfpspey  580
DSSP  hhhhllllllhhhlhhhhhhhhhhllhhhhhhhhhhhhhlllhhhhllllllllllhhh


DSSP  ----------------------------------
Query ----------------------------------  292
ident 
Sbjct mifdheftklvkelegvitkaiyksseedkkeee  614
DSSP  llllhhhhhhhhhhhhhhhhhhhllllhhhhhhhll
```

No 697: Query=mol1A Sbjct=3hm0A Z-score=2.6

back to top

```
DSSP  llllllllllhhhhhhhHHHHHhhllllhhhHHHHhHHHHHHHHHHHLlllllLLLLeeee
Query aapysspptnlrslrdrLTQVAerqgvvfgrLQRHvAXIVVAQFAATltddtGAPLllvk   60
ident                                                     |
Sbjct ---nafhdfqarvyvadTDFSG-------vvYHAR-YLEFFERGRSEflrdtGFNN----   45
DSSP  ---llleeeeeellhhhLLLLL-------llLLLH-HHHHHHHHHHHhhhllLLLH----


DSSP  hhhhhhhhhlllllllllleEEEElllhhhhhhhhhhhhhhllLLEEEEEELLLEELLlll
Query ggsslelrrgipdsrtskdfATVArrdieliheqladagetgwEGFTAIFTAPEEIAapg  120
ident                                               |
Sbjct ------tllasgvegeklffVVRH-------meinfsrpaqidNLLTIKTRISRLQG---   89
DSSP  ------hhhhhllllllleeeEEEE-------eeeeelllllllLEEEEEEEEEEELL---


DSSP  lllLLEEEEEEEEEELLEEEEEEEEEEEEELLLLLlllleeeeelhhhhllllllleeeeell
Query xpvKPRRFTAKLSYRGRAFATVPIEVSSVEAGNadqfdtltsdalglvgvpaavavpcxt  180
ident                     |   |
Sbjct ---ARFFMEQYILHGESMLVTAKVEIALINEEG---------------------------  119
DSSP  ---LEEEEEEEEEELLEEEEEEEEEEEEEEELLL---------------------------
```

```
Sbjct ------------------------------------------------------------  119
DSSP  ------------------------------------------------------------


DSSP  lllllllllllhhhhhhhhhlllllllllllhhhhhhhhhhhhhhhhhllll
Query pwpprvatlphwpliyagalegldhlelartvdaaaqavqrfvaridratkr  292
ident
Sbjct ---------------------------------------------kprrlpk  126
DSSP  ---------------------------------------------lllllll
```

No 698: Query=mol1A Sbjct=3bg3A Z-score=2.6

back to top

```
DSSP  lllllllllhhhhhhhhhhhhhllllhHHHHHHHHHHHHHHHHHLL--lLLLLLL---
Query aapyssppptnlrslrdrltqvaerqgvvfGRLQRHVAXIVVAQFAATL--tDDTGAP---   55
ident                                                  |
Sbjct -----------------------------QNRAQKLLHYLGHVMVNGPttpIPVKASpsp   31
DSSP  -----------------------------LHHHHHHHHHHHHHHHHHLLlllLLLLLLlll


DSSP  ------------------------------------------------------------
Query ------------------------------------------------------------   55
ident
Sbjct tdpvvpavpigpppagfrdillregpegfaravrnhpglllmdttfrdahqsllatrvrt   91
DSSP  llllllllllllllhhhhhhhhhhhhhhhhhhhllllleeeellllhhhhhhllllllh


DSSP  ------------------------------------------------------------
Query ------------------------------------------------------------   55
ident
Sbjct hdlkkiapyvahnfsklfsmenwggatfdvamrflyecpwrrlqelrelipnipfqmllr  151
DSSP  hhhhhhhhhhhhhllllleeeeeeellhhhhhhhlllllhhhhhhhhhhhlllllleeeeel


DSSP  ------------------------------------------------------------
Query ------------------------------------------------------------   55
ident
Sbjct ganavgytnypdnvvfkfcevakengmdvfrvfdslnylpnmllgmeaagsaggvveaai  211
DSSP  hhhllllllllhhhhhhhhhhhhhhllleeeeeellllhhhhhhhhhhhhllllleeeeee


DSSP  ------------------------------------------------------------
Query ------------------------------------------------------------   55
ident
Sbjct sytgdvadpsrtkyslqyymglaeelvragthilcixdmagllkptactmlvsslrdrfp  271
DSSP  elllllllllllllhhhhhhhhhhhhhhllleeeeeelllllllhhhhhhhhhhhhhhll


DSSP  ------------------------------------------------------------
Query ------------------------------------------------------------   55
ident
Sbjct dlplhihthdtsgagvaamlacaqagadvvdvaadsmsgmtsqpsmgalvactrgtpldt  331
DSSP  llleeeellllllhhhhhhhhhhllllleeeellhhhlllllllllhhhhhhhhlllllll


DSSP  ------------------------------------------------------------
Query ------------------------------------------------------------   55
ident
Sbjct evpmervfdyseywegarglyaafdctatmksgnsdvyeneipggqytnlhfqahsmglg  391
DSSP  lllhhhhhhhhhhhhhhhhhhlhhhlhhhllllllllhhhhlllhhhhhhhhhhhllllll


DSSP  ------------------------------------------------------------
Query ------------------------------------------------------------   55
ident
Sbjct skfkevkkayveanqmlgdlikvtpsskivgdlaqfmvqnglsraeaeaqaeelsfprsv  451
DSSP  lhhhhhhhhhhhhhhhlllllllllhhhhhhhhhhhhhhllllhhhhhhhllllllllhhh


DSSP  ------------------------------------------------------------
Query ------------------------------------------------------------   55
ident
Sbjct veflqgyigvphggfpepfrskvlkdlprvegrpgaslppldlqalekelvdrhgeevtp  511
```

```
DSSP   --------leeeehhhhHHHHhlllllllllleeeeelllhhhhhhhhhhHHHHLLL---
Query  --------lllvkggssLELRrgipdsrtskdfatvarrdieliheqladAGETGWE---  104
ident                                                          |
Sbjct  edvlsaamypdvfahfkDFTA------------tfgpldslntrlflqgpKIAEEFEvel  559
DSSP   hhhhhhhlhhhhhhhhhHHHH------------hllllllllhhhhhhllLLLLEEEeel


DSSP   ------LEEE-EELLLEELlllllllllEEEEEEEEELLEEeEEEEEE---EEELlllll
Query  ------GFTA-IFTAPEEIaapgxpvkpRRFTAKLSYRGRAfATVPIE---VSSVeagna  154
ident           |                            |
Sbjct  ergktlHIKAlAVSDLNRA---------GQRQVFFELNGQL-RSILVKdtqAMKE-----  604
DSSP   lllleeEEEEeEEEEELLL---------LEEEEEEELLLLE-EEEEEElhhHLLL-----


DSSP   llleeeeelhhhhlllllleeeeellhhhhhhhhhhhhhllllllllllllhhhhhhhhh
Query  dqfdtltsdalglvgvpaavavpcxtipwqiaqklhavtavleepkandrahdlvdlqll  214
ident
Sbjct  ------------------------------------------------------------  604
DSSP   ------------------------------------------------------------


DSSP   hhhllhhhhhhhhhhhhhhhhhhhlllLLLLLLlllllhhhhhhhhhllllllllllhhh
Query  eglllddadlxptrsaciaifearaqhPWPPRVatlphwpliyagalegldhlelartvda  274
ident                                 |
Sbjct  --------------------------KALKDV----------------------------  610
DSSP   --------------------------LLLLLL----------------------------


DSSP   hhhhhhhhhhhhHHLLLL------------------------------------------
Query  aaqavqrfvariDRATKR------------------------------------------  292
ident
Sbjct  -------kgqigAPMPGKvidikvvagakvakgqplcvlsamkmetvvtspmegtvrkvh  663
DSSP   -------llleeLLLLEEeeeellllllllllllleeeelllleeeellllllllll


DSSP   -----------------
Query  -----------------  292
ident
Sbjct  vtkdmtlegddlileie  680
DSSP   llleeeelllleeelll
```

No 699: Query=mol1A Sbjct=2pbiA Z-score=2.6

back to top

```
DSSP   lllllllllhhhhhhhhhhhhhhlllhhhhhhhhhhhhhhhhhhlllllLLLLLleeee
Query  aapysspptnlrslrdrltqvaerqgvvfgrlqrhvaxivvaqfaatltddTGAPlllvk   60
ident
Sbjct  --------------------------------------------------gQQYR-----    5
DSSP   --------------------------------------------------lLLLL-----


DSSP   hhhhhhhhhlllllllllleeeeeelllhhhHHHHHHH------hhhhllllleeeeellle
Query  ggsslelrrgipdsrtskdfatvarrdielIHEQLAD------agetgwegftaiftape  114
ident                                     |
Sbjct  -------------------------prmafLQKIEALvkdmqnpetgvrmhnqrvlvtsv   40
DSSP   -------------------------llllhHHHHHHHhhhhllllllllllllllllll


DSSP   ellllllllleeeeeeeeeLLEEEE----eEEEE-----------------------EE
Query  eiaapgxpvkprrftaklsyRGRAFA----tVPIE-----------------------VS  147
ident                         |       |
Sbjct  phamtggdvlqwitqrlwisNLEAQNlgnfiVKYGyiyplqdpknlilkpdsslyrfqTP  100
DSSP   llleehhhhhhhhhhhhlllHHHHHhhhhhHHLLleeellllllllllllleeeelLH


DSSP   ELlllllllleeeeelhhhhlllllleEEEELLHHHHHHHHHHHHHHLLllllllllLLHH
Query  SVeagnadqfdtltsdalglvgvpaavAVPCXTIPWQIAQKLHAVTAVleepkandRAHD  207
ident
Sbjct  YF----------------------wptQQWPAEDTDYAIYLAKRNIKK----kgilEEYE  134
DSSP   HH----------------------lllLLLLLLHHHHHHHHHHHHHHLL----llllLHHH


DSSP   HHHHHHHHHLLHhHHHHHHHHHHHHHHHHHL---llllllllllllhhhhhhHHLL--
```

```
DSSP  HHHHHHHHHHLHH-HHHHHHHHHHHHHHHHHLlllhhhhhhhhhhhhhhhhhhhllLLLLll


DSSP  ------------------------------------------------------------
Query ------------------------------------------------------------  262
ident
Sbjct vldygldrvtnpnevkkqtvtavrkeimyyqqalmrstvkssvslggivkyseqfssnda  253
DSSP  llllllllllllllllllhhhhhhhhhhhhhhhhlllllhhhhhhhhhhhhhhhhhhlh


DSSP  ------------------------------------------------------------
Query ------------------------------------------------------------  262
ident
Sbjct imsgclpsnpwitddtqfwdlnaklveiptkmrverwafnfselirdpkgrqsfqyflkk  313
DSSP  hhhllllllhhhhlllhhhhhlllllllllhhhhhhllllhhhhhhlhhhhhhhhhhhhh


DSSP  ------------------------------------------------------------
Query ------------------------------------------------------------  262
ident
Sbjct efsgenlgfweacedlkygdqskvkekaeeiyklflapgarrwinidgktmditvkglrh  373
DSSP  llllhhhhhhhhhhhhhlllllhhhhhhhhhhhhllllllllllllhhhhhhhhhhllll


DSSP  ------------lllllllllhhhhhhhhhhhhhhhhhllll
Query ------------ldhlelartvdaaaqavqrfvaridratkr  292
ident
Sbjct phryvldaaqthiymlmkkdsyarylkspiykemlakaiepq  415
DSSP  llllllhhhhhhhhhhhhhlhhhhhhllhhhhhhhhhlllll
```

No 700: Query=mol1A Sbjct=4mduB Z-score=2.6

back to top

```
DSSP  lllllllllhhhhhhhhhhHHHHLlllhhhhhhhhhhhhhhhhhhllllllllleeee
Query aapyssppptnlrslrdrltqVAERQgvvfgrlqrhvaxivvaqfaatltddtgaplllvk   60
ident
Sbjct ------isefgitrslihsfDPHGK-----------------------------------   19
DSSP  ------lllllllllhhhllLLLLL-----------------------------------


DSSP  hhhhhhhhhlllllllllleeeeelllhhhhhhhhhhhhhhlllleeeeeellleelllll
Query ggsslelrrgipdsrtskdfatvarrdieliheqladagetgwegftaiftapeeiaapg  120
ident
Sbjct ------------------------------------------------------------   19
DSSP  ------------------------------------------------------------


DSSP  lllllleeeeeeeeelleeeeeeeeeeeelllllllllleeeeelhhhhllllllleeeeELL
Query xpvkprrftaklsyrgrafatvpievssveagnadqfdtltsdalglvgvpaavavpCXT  180
ident
Sbjct ------------------------------------------------hyrptikptTGF   31
DSSP  ------------------------------------------------lllllllllLLL


DSSP  HHHHHHHHHHHHHHLlllllllLLLLhHHHHHHHHHHHHLLHHHHHHHHHHHHHHHHHhhlll
Query IPWQIAQKLHAVTAvleepkaNDRAhDLVDLQLLEGLLLDADLXPTRSACIAIFEaraqh  240
ident      |  ||
Sbjct SASADAERLHRSMK-------GPGT-NELAIINILARRTNYERQEICQSYKSLYK----q   79
DSSP  LHHHHHHHHHHHHHL-------LLLL-LHHHHHHHHLLLLHHHHHHHHHHHHHHHHHL----l


DSSP  LLLL--LLLLlllhhhhhhhhhllllllllllhhhhhHHHHHhhHHHHH----------
Query PWPP--RVATlphwpliyagalegldhlelartvdaaaQAVQRfvARIDR----------  288
ident          |                                     |
Sbjct DLKDdlKSDT--------------------------sgDFRKVlcQLIVDtpymlaksly  113
DSSP  LHHHhhHHHL--------------------------lhHHHHHhhHHHLLhhhhhhhhhh


DSSP  ------------------------------------------------------------
Query ------------------------------------------------------------  288
ident
Sbjct yamkglgtndrvlieifttlwndemkavadaykqvlkdkgseesersl vtdmkketcgdy  173
DSSP  hhhhleellhhhhhhhhhlllhhhhhhhhhhhhhhhhhhhllllllllllhhhhhllhhhhlhh
```

```
Query ------------------------------------------------------------  288
ident 
Sbjct eyallslvqaerddipilqlkaipdkgvnsiinhelaeadakdlyasgagrvgtserrit  233
DSSP  hhhhhhhhlllllllhhhhhlhhhhlhhhhllhhhhhhhhhhhhhlllllllllllhhhh


DSSP  ------------------------------------------------------------
Query ------------------------------------------------------------  288
ident 
Sbjct rvicnrtpyqlyltseiyfkmygktllehiesetsgdyrkllavlryaidrpsliaewl  293
DSSP  hhhhllhhhhhhhhhhhhhhlllhhhhhhhhllhhhhhhhhhhhhhhhlhhhhhhhhh


DSSP  ------------------------------------------------------------
Query ------------------------------------------------------------  288
ident 
Sbjct hdsmaglgtkdyalmrllitrseidlqdimdayesiygksllnavkddtsgdyrrtlcvl  353
DSSP  hhhllllhhhllhhhhhhhhhhlllhhhhhhhhhhhhlllhhhhhhhhllhhhhhhhhhh


DSSP  ---llll
Query ---atkr  292
ident 
Sbjct mgeiynq  360
DSSP  lllllll
```

No 701: Query=mol1A Sbjct=3n0fA Z-score=2.6

back to top

```
DSSP  -------lllllllllLHHHhhhhhhhhhhllllhhhhhhhhhhhhhhhhhhlllllll
Query -------aapysspptNLRSLrdrltqvaerqgvvfgrlqrhvaxivvaqfaatltddtg   53
ident 
Sbjct sadyepnswdydfllsSIEVY---------------------------------------   21
DSSP  llllllllllhhhhllLLHHH---------------------------------------


DSSP  llleeeehhhhhhhhllllllllleeeeellhhhhhhhhhhhhhhllllleeeeelll
Query aplllvkggsslelrrgipdsrtskdfatvarrdieliheqladagetgwegftaiftap  113
ident 
Sbjct -------------------------------------------kdkakkleaevrreinn   38
DSSP  -------------------------------------------hhhhhhhhhhhhhhhhl


DSSP  EELL-----------------------LLLLlllleeeeeeeeeelleeeeeeeEEEEell
Query EEIA-----------------------APGXpvkprrftaklsyrgrafatvpIEVSsve  150
ident |
Sbjct EKAEfltllelidnvqrlglgyrfesdIRRA-----------------ldrfvSSGG---   78
DSSP  LLLLhhhhhhhhhhhhhlllhhhlhhhHHHH-----------------hhhhhHLLH---


DSSP  llllllleeeeelhhhhlllllleeeeellhhhHHHHHHHHHHLllllllLLLLLhhHHH
Query agnadqfdtltsdalglvgvpaavavpcxtipwQIAQKLHAVTAvleepkANDRAhdLVD  210
ident 
Sbjct --------------------------fdgvtktSLHATALSFRL--lrqhGFEVS--QEA  108
DSSP  --------------------------hhhhlllLHHHHHHHHHH--hhhlLLLLL--HHH


DSSP  HHHHH----HHLLHhHHHHhHHHHHhhhhhhlllllLLLLLlllhhhhhhhhhllllll
Query LQLLE----GLLLDaDLXPtRSACIaifearaqhpwpPRVATlphwpliyagalegldhl  266
ident            |          |
Sbjct FSGFKdqngNFLEN-LKED-TKAIL---------slyEASFL------------------  139
DSSP  HHHHLllllLLLHH-HHHL-HHHHH---------hhhHHHLL------------------


DSSP  lllLLHHhHHHHHHHHHHHHHHHLL-----------------------------------
Query elaRTVDaAAQAVQRFVARIDRAT-----------------------------------  290
ident                |
Sbjct -alEGEN-ILDEARVFAISHLKELseekigkelaeqvnhalelplhrrtqrleavwsiea  197
DSSP  -llLLLH-HHHHHHHHHHHHHHLLlhhhhlhhhhhhhhhhhhlllllllhhhhhhhhhh


DSSP  ------------------------------------------------------------
Query ------------------------------------------------------------  290
```

```
DSSP  ------------------------------------------------------------
Query ------------------------------------------------------------  290
ident
Sbjct vgvafepqysdcrnsvakmfsfvtiiddiydvygtldelelftdaverwdvnaindlpdy  317
DSSP  hhhlllhhhhhhhhhhhhhhhhhhhhhhhhllllhhhhhhhhhhhhhllhhhhhhllhh


DSSP  ------------------------------------------------------------
Query ------------------------------------------------------------  290
ident
Sbjct mklcflalyntineiaydnlkdkgenilpyltkawadlcnaflqeakwlynkstptfddy  377
DSSP  hhhhhhhhhhhhhhhhhhhhhhhhhllllhhhhhhhhhhhhhhhhhhhhhhhhllllllhhhh


DSSP  ------------------------------------------------------------
Query ------------------------------------------------------------  290
ident
Sbjct fgnawksssgplqlifayfavvqnikkeeienlqkyhdiisrpshifrlcndlasasaei  437
DSSP  hhhhhhllhhhhhhhhhhhhlllllhhhhhhhhlllhhhhhhhhhhhhhhhhhhhhhhh


DSSP  ------------------------------------------------------------
Query ------------------------------------------------------------  290
ident
Sbjct argetansvscymrtkgiseelatesvmnlidetwkkmnkeklggslfakpfvetainla  497
DSSP  llllllhhhhhhhhhlllhhhhhhhhhhhhhhhhhhhhhhhhhhllllllhhhhhhhhhhh


DSSP  --------------------------------ll
Query --------------------------------kr  292
ident
Sbjct rqshctyhngtspdeltrkrvlsvitepilpfer  531
DSSP  hhhhhhllllllhhhhhhhhhhhhhllllllllll
```

No 702: Query=mol1A Sbjct=3p3qB Z-score=2.6

back to top

```
DSSP  lllllllllhhhhhhhhhhhhhhlllhhhhhhhhhhhhhhhhhhllllllllllleeee
Query aapysspptnlrslrdrltqvaerqgvvfgrlqrhvaxivvaqfaatltddtgaplllvk   60
ident
Sbjct ------------------------------------------------------------    0
DSSP  ------------------------------------------------------------


DSSP  hhhhhhhhlllllllllleeeeeelllhhhhhhhhhhhhhhlllleeeeellleellllll
Query ggsslelrrgipdsrtskdfatvarrdieliheqladagetgwegftaiftapeeiaapg  120
ident
Sbjct ------------------------------------------------------------    0
DSSP  ------------------------------------------------------------


DSSP  lllleeeeeeeeeelleeeeeeeeeeeelllllllleeeeelhhhhllllleeeeeelL
Query xpvkprrftaklsyrgrafatvpievssveagnadqfdtltsdalglvgvpaavavpcxT  180
ident
Sbjct -------------------------------------------------xlcdslptasR   11
DSSP  -------------------------------------------------llllllleehH


DSSP  HHHHHHHHHHHHHlllllllllllhHHHHHHHHHHHLLHhhhhhhhhhhhhhhhhhlll
Query IPWQIAQKLHAVTavleepkandrahDLVDLQLLEGLLLDadlxptrsaciaifearaqh  240
ident     |                         |  |
Sbjct TAAAILNLAQSED------------vTAEALAQLIQTDPA--------------------   39
DSSP  HHHHHHHHHHHLL------------lLHHHHHHHHLLLHH--------------------


DSSP  lllllllllllhhhhhhhhhlLLLLL--LLLLLhHHHHHHHHHHHHHHHH-LLLL-----
Query pwpprvatlphwpliyagaleGLDHL--ELARTvDAAAQAVQRFVARIDR-ATKR-----  292
ident                                          |         |
Sbjct ----ltgrilrfanapaqgtrRPVASviDAIDL-VGLPAVRQFALSLSLIdAHREgrcea   94
DSSP  ----hhhhhhhhhhlhhhlllLLLLLhhHHHHH-HLHHHHHHHHHHHHHHlEELLlllll
```

```
[illegible]
ident
Sbjct fdyaaywqkslaravalqsitaqastvapkeaftlglladvgrlalatawpeeyseclrk  154
DSSP  llhhhhhhhhhhhhhhhhhhhhhhhllllhhhhhhhhhhllhhhhhhhhhlhhhhhhhhh


DSSP  ------------------------------------------------------------
Query ------------------------------------------------------------  292
ident
Sbjct adgealialererfatdhdeltrxlltdwgfpqvfidalqlsqqdeirdegrtgrfarql  214
DSSP  llhhhhhhhhhhhlllhhhhhhhhhhhllllhhhhhhhhhhllllllllhhhhhhhh


DSSP  ---------------------------------------
Query ---------------------------------------  292
ident
Sbjct tlaqhiadhrlaaalspllraearrcglgdedlarllad  253
DSSP  hhhhhhhhhhlllllhhhhhhhhhllllllhhhhhhll
```

No 703: Query=mol1A Sbjct=3rtrC Z-score=2.6

back to top

```
DSSP  lllllllllhhhhhhhhhhhhhhllllhhhhhhhhhhhhhhhhhhllllllllleeee
Query aapyssppptnlrslrdrltqvaerqgvvfgrlqrhvaxivvaqfaatltddtgaplllvk   60
ident
Sbjct ------------------------------------------------------------    0
DSSP  ------------------------------------------------------------


DSSP  hhhhhhhhlllllllllleeeeellllhhhhhhhhhhhhhhllllleeeeellleelllll
Query ggsslelrrgipdsrtskdfatvarrdieliheqladagetgwegftaiftapeeiaapg  120
ident
Sbjct ------------------------------------------------------------    0
DSSP  ------------------------------------------------------------


DSSP  llllleeeeeeeeelleeeeeeeeeeeelllllllleeeeelhhhhllllleeeeeelL
Query xpvkprrftaklsyrgrafatvpievssveagnadqfdtltsdalglvgvpaavavpcxT  180
ident
Sbjct ---------------------------------------------------------peE    3
DSSP  ---------------------------------------------------------lhH


DSSP  HHHHHHHHHHhhhllllllllllllHHHHHHHHHHHHL-----LHHHHHHHHHHHHHHHH
Query IPWQIAQKLHavtavleepkandraHDLVDLQLLEGLL-----LDADLXPTRSACIAIFE  235
ident         |                     |             |
Sbjct LARYCDSLLK--------------kELEDTLNQVMEKFkkiedKDVFQKFYAKMLAKRLV   49
DSSP  HHHHHHHHHL--------------lLHHHHHLHHHHLLlllllHHHHHHHHHHHHHHHHH


DSSP  hhlLLLLlLLLLllllhhhhhhhhllllllllllllhhhhhhhhHHHHH---hhhHLLL-
Query araQHPWpPRVAtlphwpliyagalegldhlelartvdaaaqavQRFVA---ridRATK-  291
ident                                                           |
Sbjct ---HQNS-ASDD------------------------------aeASMISklkqaeYTSKl   75
DSSP  ---LLLL-LLHH------------------------------hhHHHHHhhllllLLHHh


DSSP  ------------------------------------------------------------
Query ------------------------------------------------------------  291
ident
Sbjct qrmfqdigvskdlneqfkkhltnsepldldfsiqvlssgswpfqqsctfalpselersyq  135
DSSP  hhhhhhhhhhhhhhhhhhhlllllllllleeeelleelllllllllllllllhhhhhhhh


DSSP  ------------------------------------------------------------
Query ------------------------------------------------------------  291
ident
Sbjct rftafyasrhsgrkltwlyqlskgelastfqmaillqyytvqqltdstqikmdilaqvlq  195
DSSP  hhhhhhhhhllleeeeelllllleeellllhhhhhhllllhhhhhhhllllhhhhhhhhh


DSSP  ------------------------------------------------------------
Query ------------------------------------------------------------  291
ident
```

```
DSSP  ------------------------------------------------------------
Query ------------------------------------------------------------  291
ident
Sbjct ivrimkmrkvlkhqqllgevltqlssrfkprvpvikkcidiliekeylervdgekdtysy  315
DSSP  hhhhhhhlleeehhhhhhhhhhhhhlllllhhhhhhhhhhhhhllleeellllllleeee


DSSP  -l
Query -r  292
ident
Sbjct la  317
DSSP  ll
```

No 704: Query=mol1A Sbjct=1bg1A Z-score=2.6

back to top

```
DSSP  lllllllllhhhhhhhhhhhhhhllllhhhhhhhhhhhhhhhhhhlllllLLLLLLeeee
Query aapyssspptnlrslrdrltqvaerqgvvfgrlqrhvaxivvaqfaatltddTGAPLllvk   60
ident
Sbjct ---------------------------------------------------VVTEK----    5
DSSP  ---------------------------------------------------LLLHH----


DSSP  hhhhhhhhhllllllllleeeeeelllhhhhhhhhhhhhhhlllleeeeellleelllll
Query ggsslelrrgipdsrtskdfatvarrdielihеqladagetgwegftaiftapeeiaapg  120
ident
Sbjct ------------------------------------------------------------    5
DSSP  ------------------------------------------------------------


DSSP  lllllleeeeeeeeeelleeeeeeeeeeeelllllllLLLEeeeelhhhhllllllleeeeeLL
Query xpvkprrftaklsyrgrafatvpievssveagnaDQFDtltsdalglvgvpaavavpcXT  180
ident
Sbjct -------------------------------qqmLEQH-------------------lQD   15
DSSP  -------------------------------hhhHHHH-------------------hHH


DSSP  HHHHHHHHHHHH---hlLLLLLL----------------------------lllLLHHHH
Query IPWQIAQKLHAV---taVLEEPK----------------------------andRAHDLV  209
ident                                                         |
Sbjct VRKRVQDLEQKMkvvenLQDDFDfnyktlksqgdsvtrqkmqqleqmltaldqmRRSIVS   75
DSSP  HHHHHHHHHHHHhhhhhHHHHHHhhhhhhhlllllllllhhhhhhhhhhhhhhhHHHHHH


DSSP  HHHHHHHHL-------------------------------------lhHHHHHHHhhhhh
Query DLQLLEGLL-------------------------------------ldADLXPTRsacia  232
ident  |  |                                            |
Sbjct ELAGLLSAMeyvqktltdeeladwkrrqqiaciggppnicldrlenwiTSLAESQ-----  130
DSSP  HHHHHHHHHhhhhhhhhhlhhhhhhhhhhhhhlllllllllhhhhhhhHHHHHHH-----


DSSP  hhhhhlllllllLLLL--LLLLHHHhhhhhhlllllllllLHHHHHHHHHHHHHHHHH-L
Query ifearaqhpwppRVAT--LPHWPLiyagalegldhlelarTVDAAAQAVQRFVARIDR-A  289
ident                                                         |
Sbjct -----lqtrqqiKKLEelQQKVSY-------------kgdPIVQHRPMLEERIVELFRnL  172
DSSP  -----hhhhhhhHHHHhhHHHLLL-------------lllLLLLLLHHHHHHHHHHHHhH


DSSP  LLL---------------------------------------------------------
Query TKR---------------------------------------------------------  292
ident  |
Sbjct MKSafvverqpcmpmhpdrplviktgvqfttkvrllvkfpelnyqlkikvcidkdsgdva  232
DSSP  HHHheeeeeeeellllllllleeellllеeeeeeellllhhhlllleeeeeeellhhhlll


DSSP  ------------------------------------------------------------
Query ------------------------------------------------------------  292
ident
Sbjct alrgsrkfnilgtntkvmnmeesnngslsaefkhltlreqrcgnggrancdaslivteel  292
DSSP  llllllleeeelllееellllllllllеeeeeeeeeeeеllllllllllhhhlllhhhll
```

```
Sbjct hlitfetevyhqglkidlethslpvvvisnicqmpnawasilwynmltnnpknvnfftkp  352
DSSP  lleeeeeeeeelleeeeeeeeellllееellhhhhhhhhhhhhhhhhllllllllhhhll


DSSP  ------------------------------------------------------------
Query ------------------------------------------------------------  292
ident
Sbjct pigtwdqvaevlswqfssttkrglsieqlttlaekllgpgvnysgcqitwakfckenmag  412
DSSP  lleehhhhhhhhhhhhhhhllllllhhhhhhhhhhhhlllllllllleelhhhhllllll


DSSP  ------------------------------------------------------------
Query ------------------------------------------------------------  292
ident
Sbjct kgfsfwvwldniidlvkkyilalwnegyimgfiskererailstkppgtfllrfsesske  472
DSSP  llllhhhhhhhhhhhhhhlllhhhhllllllllllhhhllllllllllllleeellllll


DSSP  ------------------------------------------------------------
Query ------------------------------------------------------------  292
ident
Sbjct ggvtftwvekdisgstqiqsvepytkqqlnnmsfaeiimgykimdatnilvsplvylypd  532
DSSP  lleeelllllllllllllllllhhhhllllhhhhllllllllllllllllllllllllll


DSSP  ---------------------------
Query ---------------------------  292
ident
Sbjct ipkeeafgkycraapxlktkficvtpf  559
DSSP  lllllllhhhllllllllllllllll
```

No 705: Query=mol1A Sbjct=2o5aA Z-score=2.6

back to top

```
DSSP  llllllllllhhhhhhhhhhhhhhllllhhhhHHHHHHHHHHHHHHLLllllllLLEEEE
Query aapysspptnlrslrdrltqvaerqgvvfgrlQRHVAXIVVAQFAATLtddtgaPLLLVK   60
ident                                                           |
Sbjct --------------------------------SNQELLQLAVNAVDDK------KAEQVV   22
DSSP  --------------------------------LLHHHHHHHHHHHHHL------LLEEEE


DSSP  hhHHHHhhhlllLLLLlLLEEEEElLLHHHHHHHHHHHHH-hLLLLEeeeeLLLEellll
Query ggSSLElrrgipDSRTsKDFATVArRDIELIHEQLADAGE-tGWEGFtaifTAPEeiaap  119
ident                                              |
Sbjct --ALNX-----kGISLdFFLICHG-NSEKQVQAIAHELKKvaQEQGIeikrLEGY-----   69
DSSP  --EEEL-----lLLLLlEEEEEEE-LLHHHHHHHHHHHHHhhHHLLLllleEELL-----


DSSP  lllllLEEEEEEeeelleeeEEEEEEEEELlllllllleeeeelhhhhllllllеeeeel
Query gxpvkPRRFTAKlsyrgrafATVPIEVSSVeagnadqfdtltsdalglvgvpaavavpcx  179
ident                       |   |
Sbjct --eqaRWVLIDL--------GDVVVHVFHK------------------------------   89
DSSP  --lllLEEEEEL--------LLEEEEEEEL------------------------------


DSSP  lhhhhhHHHHhhhhlllllllllllhhhhhhhhhhhhllhhhhhhhhhhhhhhhhhhhll
Query tipwqiAQKLhavtavleepkandrahdlvdlqllegllldadlxptrsaciaifearaq  239
ident
Sbjct derayyNLEK--------------------------------------------------   99
DSSP  llhhhlLLLL--------------------------------------------------


DSSP  llllllllllllhhhhhhhhhlllllllllllhhhhhhhhhhhhhhhhhllll
Query hpwpprvatlphwpliyagalegldhlelartvdaaaqavqrfvaridratkr  292
ident
Sbjct --------------------------------------------lwptveleg  108
DSSP  --------------------------------------------lllllllll
```

No 706: Query=mol1A Sbjct=3innC Z-score=2.6

```
Query ------------------------------------------------------------    0
ident
Sbjct smqiihtieelrqalaparqqgkkigfvptmgylhkghlelvrrarvendvtlvsifvnp   60
DSSP  lleeellhhhhhhhhhhhhhllllleeeeeeellllhhhhhhhhhhhhhhlleeeeeeelllh


DSSP  ------------------------------------------------------------
Query ------------------------------------------------------------    0
ident
Sbjct lqfganedlgryprdlerdagllhdaqvdylfaptvsdmyprpmqtvvdvpplgnqiege  120
DSSP  hhllllllllllllhhhhhhhhhhllllleeellllhhhhllllllleeelhhhhlllhhh


DSSP  ------------------------------------------------------llllll
Query ------------------------------------------------------aapyss    6
ident
Sbjct arpghfagvatvvsklfnivgpdaayfgekdfqqlviirrmvddmaipvrivgvetvred  180
DSSP  hlllhhhhhhhhhhhhhhhhhllleeeeeelllhhhhhhhhhhhhhlllllleeeeellllll


DSSP  llllhhHHHhhhhhhhhllllHHHHhhhhhHHHHHHhllllllllleeeehhhhhh
Query pptnlrSLRDrltqvaerqgvvFGRLQrhvaxIVVAQFAatltddtgaplllvkggssle   66
ident       |                         | | |
Sbjct dglacsSRNV----------ylTPEQR--raaIIVPQAL---------------------  207
DSSP  llllllHHHH----------hlLHHHH--hhlLHHHHHH---------------------


DSSP  hhhllllllllleeeeeLLLHHHHHHHHHHHHHHHlLLLEEE-EELLLEELL---lllll
Query lrrgipdsrtskdfatvaRRDIELIHEQLADAGETgWEGFTA-IFTAPEEIA---apgxp  122
ident                     |
Sbjct --------deadrlyrsgMDDPDALEAAIRTFIGR-QPLAVPeVIAIRDPETlerlpalq  258
DSSP  --------hhhhhhhhllLLLHHHHHHHHHHHHHL-LLLEEEeEEEEELLLLllllllll


DSSP  LLLEEEEEEEEEllEEEEEEEEEEEellllllllleeeeelhhhhllllleeeeeellhh
Query VKPRRFTAKLSYrgRAFATVPIEVSsveagnadqfdtltsdalglvgvpaavavpcxtip  182
ident   |
Sbjct GRPILVALFVRV-gATRLLDNRVIG-----------------------------------  282
DSSP  LLLEEEEEEEEE-lLEEEEEEEEEL-----------------------------------


DSSP  hhhhhhhhhhlllllllllllhhhhhhhhhhhllhhhhhhhhhhhhhhhhhhhhhlllll
Query wqiaqklhavtavleepkandrahdlvdlqllegllldadlxptrsaciaifearaqhpw  242
ident
Sbjct ------------------------------------------------------------  282
DSSP  ------------------------------------------------------------


DSSP  llllllllhhhhhhhhhllllllllllhhhhhhhhhhhhhhhhhhhllll
Query pprvatlphwpliyagalegldhlelartvdaaaqavqrfvaridratkr  292
ident
Sbjct ------------------------------------------------ha  284
DSSP  ------------------------------------------------ll
```

No 707: Query=mol1A Sbjct=3ebbC Z-score=2.6

back to top

```
DSSP  ------------------------------------------------------------
Query ------------------------------------------------------------    0
ident
Sbjct txniyfpkkeavtfdqanptqilgklkelngtapeekklteddlillekilslicnssse   60
DSSP  lllllllllllllllllhhhhhhhhhhhhllllllllllhhhhhhhhhhhhhhhhlllll


DSSP  -----LLLL---llLLLLHHhHHHHHHHHHHHLL---------llhhhhHHHHHHHHHHH
Query -----AAPY---ssPPTNLRsLRDRLTQVAERQG---------vvfgrlQRHVAXIVVAQ   43
ident                          |
Sbjct kptvqQLQIlwkaiNCPEDI-VFPALDILRLSIKhpsvnenfcnkegaqFSSHLINLLNP  119
DSSP  lllhhHHHHhhhhlLLLHHH-LHHHHHHHHHHLLlhhhhhhhhlllhhhHHHHHHHHHLL


DSSP  -------------------hhhllllllllllEEEE----------------hHHHHhhh
Query -------------------faatltddtgaplLLVK----------------gGSSLelr   68
```

```
DSSP  hllllllllllleeeeelllhhhhhhhhhhhhhhlllleeeeellleellllllllllleee
Query rgipdsrtskdfatvarrdieliheqladagetgwegftaiftapeeiaapgxpvkprrf  128
ident
Sbjct ------------------------------------------------------------  176
DSSP  ------------------------------------------------------------


DSSP  eeeeeelleeeeeeeeeeeeellllllLLLEeeeelhhhhllllleeeeelLHHHHHHHH
Query taklsyrgrafatvpievssveagnaDQFDtltsdalglvgvpaavavpcxTIPWQIAQK  188
ident                                                         |
Sbjct ---------------lnysvcfhkdhNIEG-------------------kaQCLSLISTI  202
DSSP  ---------------hhhhhhhhhhlLHHH-------------------hhHHHHHHHHH


DSSP  HHHHhllllllllllLLHHHHHHHHHHHHL---LHHHHHHHHHHHhhhhhhlllllLLLL
Query LHAVtavleepkandRAHDLVDLQLLEGLL---LDADLXPTRSACiaifearaqhpWPPR  245
ident |  |                  |    | |            |
Sbjct LEVV----------qDLEATFRLLVALGTLisdDSNAVQLAKSLG---------vdSQIK  243
DSSP  HLLL----------lLHHHHHHHHHHHHHHhllLHHHHHHHHHLL---------hhHHHH


DSSP  --LLLLllhhhhhhhhhllllllllllLHHHHHHHHHHHHHHHhhllll
Query --VATLphwpliyagalegldhlelarTVDAAAQAVQRFVARIdratkr  292
ident
Sbjct kySSVS---------------------EPAKVSECCRFILNLL------  265
DSSP  hhHHLL---------------------LLHHHHHHHHHHHHHL------
```

No 708: Query=mol1A Sbjct=3pq1A Z-score=2.5

back to top

```
DSSP  ------------------------LLLL-----------lllllLHHHhhHHHH------
Query ------------------------AAPY-----------sspptNLRSlrDRLT------   19
ident
Sbjct rfsexqnerreqaqrtvlihcpekNKFLkylsqfgpinnhffyeSFGL--YAVVefcsig   58
DSSP  llhhhhhhhhhhhleeeeeeelllLHHHhhhhhhllllleeeelLLLE--EEEEellllh


DSSP  -----------------------------------hhhhhllLLHHHHHHHHHHHHHHHH
Query -----------------------------------qvaerqgVVFGRLQRHVAXIVVAQF   44
ident                                                  |
Sbjct slqngthtxxxxxxxnkqlfellcyaesiddqlntllkefqlTEENTKLRYLTCSLIEDX  118
DSSP  hhhlllllllllllllhhhhhhllllllhhhhhhhhhhhhllLHHHHHHHHHHHHHHHHH


DSSP  HHLLLllllllleeeehhhhhhhhhllllLLLL---------lLEEEEEL----------
Query AATLTddtgaplllvkggsslelrrgipdSRTS---------kDFATVAR----------   85
ident ||                                         |
Sbjct AAAYF----------------pdcivrpfGSSVntfgklgcdlDXFLDLDsahkisgxef  162
DSSP  HLLLL----------------llleeeeeHHHLllllllllleEEEEELLlllleellee


DSSP  ------lLHHHHHHHHHHHHHHL---LLLEeeEELL-LEELllllllllleeEEEEEEEl
Query ------rDIELIHEQLADAGETG---WEGFtaIFTA-PEEIaapgxpvkprrFTAKLSYr  135
ident                |   ||       |
Sbjct qvknvpsERIATQKILSVLGECLdhfGPGCvgVQKIlNARC----------pLVRFSHQ-  211
DSSP  lllllllHHHHHHHHHHHHHHHHhhlLLLEeeEEEElLLLL----------lEEEEEEL-


DSSP  leeEEEEEEEEEElllllllllleeeeelhhhhllllleeeeellhhhhhhhhhhhhhhll
Query graFATVPIEVSSveagnadqfdtltsdalglvgvpaavavpcxtipwqiaqklhavtav  195
ident
Sbjct -asGFQCALTTNN-----------------------------------------------  223
DSSP  -llLLEEEEELLL-----------------------------------------------


DSSP  lllllllLLHHhhhhhhhhhllhhhhhhhhhhhhhhhhhhhhhhhhllllllllllhhhH
Query leepkandRAHDlvdlqllegllldadlxptrsaciaifearaqhpwpprvatlphwplI  255
ident                                                            |
Sbjct -rialtssELLY-----------------------------------------------I  235
DSSP  -llhhhhhHHHH-----------------------------------------------H
```

```
ident |                                             |
Sbjct YGAldsrvralvFSVRcwarahsltssgawitnfsltxXVIFFLQRRSdslktladaesq  295
DSSP  HHLllhhhhhhhHHHHhhhhhlllllllllllhhhhhhHHHHHHHLLLlllhhhhlllll


DSSP  --------------------------------------------------hhhhhhllll
Query --------------------------------------------------varidratkr  292
ident
Sbjct ntetlelllkeffeyfgnfxxxxxxxxxxsqsqlqkfvdlaresawilqqepwglvslll  355
DSSP  llllhhhhhhhhhhhhhhllllllllllllhhhhhhhhhhhhhhhhhhlllllllhhhll
```

No 709: Query=mol1A Sbjct=1hm6A Z-score=2.5

back to top

```
DSSP  llllllllllhhhhhhhhhhhhhhllllhhhhhhhhhhhhhhhhhhllllllllLLEE--
Query aapyssppptnlrslrdrltqvaerqgvvfgrlqrhvaxivvaqfaatltddtgaPLLL--   58
ident                                                        |
Sbjct -------------------------------------------------amvseFLKQaw   11
DSSP  -------------------------------------------------lhhhhHHHHhh


DSSP  ------------------------------------------------------------
Query ------------------------------------------------------------   58
ident
Sbjct fidneeqeyiktvkgskggpgsavspyptfnpssdvealhkaitvkgvdeatiieiltkr   71
DSSP  hhhhlhhhhhhhhhhhlllllllllllllllhhhhhhhhhhhhllllllhhhhhhhhhll


DSSP  ---------------------------------------------------------eeh
Query ---------------------------------------------------------vkg   61
ident
Sbjct tnaqrqqikaaylqekgkpldealkkaltghleevalallktpaqfdadelraamkglgt  131
DSSP  lhhhhhhhhhhhhhhhlllhhhhhhhhhllhhhhhhhhhhllhhhhhhhhhhhhhlllll


DSSP  HHHHHHHHL---------------------------------------------------
Query GSSLELRRG---------------------------------------------------   70
ident
Sbjct DEDTLNEILasrtnreireinrvykeelkrdlakditsdtsgdyqkallslakgdrsedl  191
DSSP  LHHHHHHHhhllhhhhhhhhhhhhhhlllllhhhhhhhhhllhhhhhhhhhhhlllllll


DSSP  ----------------------------------------llllllllleeeeelLLHHh
Query ----------------------------------------ipdsrtskdfatvarRDIEl   90
ident
Sbjct ainddladtdaralyeagerrkgtdlnvfitilttrsyphlrrvfqkyskyskhdMNKV-  250
DSSP  lllhhhhhhhhhhhhhhllllllllhhhhhhhhhhllhhhhhhhhhhhhllllllLLLL-


DSSP  hhHHHHhhhhhlllleeeeeelleellllllllllleeeeeeeelleeeeeEEEEeeeell
Query ihEQLAdagetgwegftaiftapeeiaapgxpvkprrftaklsyrgrafaTVPIevssve  150
ident     |                                                |
Sbjct --LDLE-------------------------------------------lKGDI----en  261
DSSP  --LLLL-------------------------------------------lLHHH----hh


DSSP  lllllLLEEeeelhhhhlllllleeeeelLHHHHHHHHHHhhhllllllllLLLLhHHHH
Query agnadQFDTltsdalglvgvpaavavpcxTIPWQIAQKLHavtavleepkaNDRAhDLVD  210
ident                                     |
Sbjct cltvvVKCA---------------tskpmFFAEKLHQAMK-----------GIGT-RHKT  294
DSSP  hhhhhHHHH---------------hlhhhHHHHHHHHHHL-----------LLLL-LHHH


DSSP  HHHHHHHLLHHHHHHHHHHHHHHHHhhlllllLLLLLllllhhhhhhhhhllllllllll
Query LQLLEGLLLDADLXPTRSACIAIFEaraqhpwPPRVAtlphwpliyagalegldhlelar  270
ident |          |
Sbjct LIRIMVSRSEIDMNDIKACYQKLYG---islcQAILD----------------------e  329
DSSP  HHHHHHHLLLLLHHHHHHHHHHHHL---llhhHHHHH----------------------h


DSSP  lHHHHHHHHHHHHhhhhhllll
Query tVDAAAQAVQRFVaridratkr  292
ident
```

No 710: Query=mol1A Sbjct=4bwrA Z-score=2.5

back to top

```
DSSP  ------------------------------------------------------------
Query ------------------------------------------------------------    0
ident 
Sbjct nvnleqlkqkaesgeakaqlelgyryfqgnettkdltqaxdwfrraaeqgytpaeyvlgl   60
DSSP  lllhhhhhhhhhlllhhhhhhhhhhhhhllllllllhhhhhhhhhhhhhlllhhhhhhhhh


DSSP  ------------------------------------------------------------
Query ------------------------------------------------------------    0
ident 
Sbjct ryxngegvpqdyaqaviwykkaalkglpqaqqnlgvxyhegngvkvdkaesvkwfrlaae  120
DSSP  hhhhllllllllhhhhhhhhhhhhhlllhhhhhhhhhhhhhllllllllhhhhhhhhhhhh


DSSP  ------------------------------------------------------------
Query ------------------------------------------------------------    0
ident 
Sbjct qgrdsgqqsxgdayfegdgvtrdyvxarewyskaaeqgnvwscnqlgyxysrglgvernd  180
DSSP  lllhhhhhhhhhhhhhlllllllhhhhhhhhhhhhhlllhhhhhhhhhhhhhlllllllh


DSSP  ------------------------------------------------------------
Query ------------------------------------------------------------    0
ident 
Sbjct aisaqwyrksatsgdelgqlhladxyyfgigvtqdytqsrvlfsqsaeqgnsiaqfrlgy  240
DSSP  hhhhhhhhhhhhlllhhhhhhhhhhhhhllllllllhhhhhhhhhhhhllllhhhhhhhhh


DSSP  ---------------------------------------lllLLLLlllhhhhhhhhhhh
Query ---------------------------------------aapYSSPptnlrslrdrltqv   21
ident 
Sbjct ileqglagakeplkalewyrksaeqgnsdgqyylahlydkgaEGVA--------------  286
DSSP  hhhhllllllllhhhhhhhhhhhhhlllhhhhhhhhhhhhhhllLLLL--------------


DSSP  hhhllllhhhhhhhhhhhhhhhhhhhllllllllllleeeehhhhhhhhhllllllllllee
Query aerqgvvfgrlqrhvaxivvaqfaatltddtgaplllvkggsslelrrgipdsrtskdfa   81
ident 
Sbjct ---------------------------------------------knreqaiswytksae  301
DSSP  ---------------------------------------------llhhhhhhhhhhhhh


DSSP  eeelllhhhHHHHHHHHHHhlllleEEEE---LLLE----ELLLlllllLLEE------e
Query tvarrdielIHEQLADAGEtgwegfTAIF---TAPE----EIAApgxpvKPRR------f  128
ident                  |
Sbjct qgdataqanLGAIYFRLGS-----eEEHKkavEWFRkaaaKGEK---aaQFNLgnallqg  353
DSSP  lllhhhhhhHHHHHHHHLLL-----hHHHHhhhHHHHhhhhLLLH---hhHHHHhhhhhhl


DSSP  EEEEeelleeeeeeeeeEEEL-----------lllllllLEEEeelhhhhlllllleeeE
Query TAKLsyrgrafatvpieVSSV-----------eagnadqFDTLtsdalglvgvpaavavP  177
ident 
Sbjct KGVK-----kdeqqaaiWXRKaaeqglsaaqvqlgeiyyYGLG----------------V  392
DSSP  LLLL-----llhhhhhhHHHHhhhlllhhhhhhhhhhhhHLLL----------------L


DSSP  ELLHHHHHHHHHHHHHHLLlllllllllLLHHHHHHHHHHHHHLLHHHHHHHHHHHHHHHHHH
Query CXTIPWQIAQKLHAVTAVleepkandRAHDLVDLQLLEGLLLDADLXPTRSACIAIFEAR  237
ident         |    | |                     |  |    |           |
Sbjct ERDYVQAWAWFDTASTND-------xNLFGTENRNITEKKLTAKQLQQAELLSQQYIEKY  445
DSSP  LLLHHHHHHHHHHHHHHHH-------lLHHHHHHHHHHHHLLLLHHHHHHHHHHHHHHHHHHH


DSSP  -LLLLllllllllllhhhhhhhhhllllllllllllhhhhhhhhhhhhhhhhhllll
Query -AQHPwpprvatlphwpliyagalegldhlelartvdaaaqavqrfvaridratkr  292
ident 
Sbjct aPEAW-----------------------------------arxqklkaqsavktgnk  467
DSSP  lHHHH-----------------------------------hhhhhhllllllllll
```

back to top

```
DSSP  ---------------------llllllLLLLHHHHHHHHHHHHHHLLLLHHHHHHHHHHH
Query ---------------------aapyssPPTNLRSLRDRLTQVAERQGVVFGRLQRHVAXI   39
ident                                   |
Sbjct ghhhhhhxddikvfqnevlgtlqrgkeENISCDNLVLEINSLKYAYNISLKEVXQVLSHV   60
DSSP  lhhhhhhhhhhhhhhhhhhhhhhhhhhHLLLHHHHHHHHHHHHHHLLLLHHHHHHHHHHH


DSSP  HHHHHHHLLLLlllllleeeehhhhhhhhhlllllllllleeeeelllhhhHHHHHHHHH
Query VVAQFAATLTDdtgaplllvkggsslelrrgipdsrtskdfatvarrdielIHEQLADAG   99
ident |
Sbjct VLEFPLQQXDS--------------------------------------plDSSRYCALL   82
DSSP  HHHHHHHHLLL--------------------------------------llLHHHHHHHH


DSSP  HHLLLLEeeeellleelllllllllleeeeeeeeellEEEE-----------------ee
Query ETGWEGFtaiftapeeiaapgxpvkprrftaklsyrgRAFA-----------------tv  142
ident
Sbjct LPLLKAW------------------------------SPVFrnyikraadhlealaaied  112
DSSP  HHHHHHH------------------------------HHHHhhhlllhhhhhhhhhhhhh


DSSP  EEEEeelllllllllleeeeelhhhhlllllleeeeellhhhhhhhhhhhhhhllllllll
Query PIEVssveagnadqfdtltsdalglvgvpaavavpcxtipwqiaqklhavtavleepkan  202
ident
Sbjct FFLE--------------------------healgisxakvlxafyqleilagetilswf  146
DSSP  HHHH--------------------------lhhhhhhhhhhhhhhhhllllhhhhhhhhh


DSSP  LLLHhhhhhhhhhhhllhhhhhhhhhhhhhhhhhlllllllllllllhhhhhhhhhhll
Query DRAHdlvdlqlleglllldadlxptrsaciaifearaqhpwpprvatlphwpliyagaleg  262
ident
Sbjct SQRD--------------------------------------------------------  150
DSSP  LLLL--------------------------------------------------------


DSSP  llllllllhhhhhhhhhhhhhhhhhhllll
Query ldhlelartvdaaaqavqrfvaridratkr  292
ident
Sbjct ------dkgqqlrknqqlqrfiqwlkeaee  174
DSSP  ------lhhhhhlllhhhhhhhhhhhhhhl
```

No 712: Query=mol1A Sbjct=4l7mA Z-score=2.5

back to top

```
DSSP  llllllllllhhhhhhhhhhhhhhllllhhhhhhhhhhhhhhhhhhllllllllLLEEEE
Query aapysspptnlrslrdrltqvaerqgvvfgrlqrhvaxivvaqfaatltddtgaPLLLVK   60
ident                                                            |
Sbjct ------------------------------------------------------NNPISK    6
DSSP  ------------------------------------------------------LLLLLH


DSSP  --HHHHH--hHHHLlllllllleeeeelllhhhhhhhhhhhhhhlllleeeeeellleel
Query --GGSSL--eLRRGipdsrtskdfatvarrdieliheqladagetgwegftaiftapeei  116
ident
Sbjct teLRELVlswQILD----------------------------------------------   20
DSSP  hhHHHHHhllLHHH----------------------------------------------


DSSP  lllllllllleeeeeeeeeelleeeeeeeeeeellllllLLLEeeeelhhhhlllllleee
Query aapgxpvkprrftaklsyrgrafatvpievssveagnaDQFDtltsdalglvgvpaavav  176
ident
Sbjct --------------------------------avslalEDKR------------------   30
DSSP  --------------------------------hhhhhhHLHH------------------


DSSP  eellhhHHHHHHHHHhhlllllllllLLLH-HHHHHHHHHHH--LLHH-HHHHHHHHHHh
Query pcxtipWQIAQKLHAvtavleepkanDRAH-DLVDLQLLEGL--LLDA-DLXPTRSACIa  232
ident         |                 |     |     |       |    |
Sbjct ----alFLILELAGE-----------DDETtRLRAFVALGEIlkRADSdLRXXVLERHL-   74
DSSP  ----hhHHHHHHLLL-----------LLHHhHHHHHHHHHHHhlLLLHhHHHHHHHHHH-
```

```
ident                                                 |
Sbjct -------dvfINALSQE----------------------NEKVTIKALRALGYLVKDVPX  105
DSSP  -------hhhHHLLLLL----------------------LHHHHHHHHHHHHHHHLLLLL


DSSP  ------------------------------------------------------------
Query ------------------------------------------------------------  292
ident 
Sbjct gsktflkaaktlvsllespddxxrietidvlsklqpledsklvrtyinelvvspdlytkv  165
DSSP  lhhhhhhhhhhhhhhlllllhhhhhhhhhhhlllllllllhhhhhhhhhhhhlllhhhhh


DSSP  ------------------------------------------------------------
Query ------------------------------------------------------------  292
ident 
Sbjct agfclflnxlnssadsghltlildeipsllqndnefivelaldvlekalsfpllenvkie  225
DSSP  hhhhhhhhhhhhlllllhhhllllhhhhhllllhhhhhhhhhhhhhhhlllllllllhhh


DSSP  ------------------------------------
Query ------------------------------------  292
ident 
Sbjct llkisrivdglvyregapiirlkakkvsdlidsvis  261
DSSP  hhhhhhhhhhhllllllhhhhhhhhhhhhhhhhhll
```

No 713: Query=mol1A Sbjct=5byuA Z-score=2.5

back to top

```
DSSP  llllllllllhhhhhHHHHhhhHHLLllHHHHHHHHHHHHHHHHHHLLlllllllLEEEE
Query aapysspptnlrslrDRLTqvaERQGvvFGRLQRHVAXIVVAQFAATLtddtgapLLLVK   60
ident                          |                     |
Sbjct -einkiihkktfdiaWGDM---DALGhvNNARYFDYFQEARIDWLREL-------DIKMT   49
DSSP  -llleeeeeeeeellHHHL---LLLLllLHHHHHHHHHHHHHHHHHHL-------LLLLL


DSSP  hhhhhhhhllllllllllleeeeelllhhhhhhhhhhhhhhlllLEEEEELLLEElllll
Query ggsslelrrgipdsrtskdfatvarrdieliheqladagetgweGFTAIFTAPEEiaapg  120
ident 
Sbjct --------------------gqtgpvvihvactflkpivypatvTIHSKVNSLGN-----   84
DSSP  --------------------lleeeeeeeeeeeellllllllееEEEEEEEEELL-----


DSSP  lllllEEEEEEEEEL----LEEEEEEEEEEEelllllllleeeeeelhhhhlllllleee
Query xpvkpRRFTAKLSYR----GRAFATVPIEVSsveagnadqfdtltsdalglvgvpaavav  176
ident                      |     |
Sbjct -----SSMIMDHDLYqeetLMAQGVSKIVWI-----------------------------  110
DSSP  -----LEEEEEEEEEelleEEEEEEEEEEEE-----------------------------


DSSP  eellhhhHHHHhhhhhhlllllllllllhhhhhhhhhhhhhhllhhhhhhhhhhhhhhhh
Query pcxtipwQIAQklhavtavleepkandrahdlvdlqllegllldadlxptrsaciaifea  236
ident 
Sbjct ------dYTQN-------------------------------------------------  115
DSSP  ------eLLLL-------------------------------------------------


DSSP  hllllllllllllllhhhhhhhhhllllllllllhhhhhhhhhhhhhhhhhhllll
Query raqhpwpprvatlphwpliyagalegldhlelartvdaaaqavqrfvaridratkr  292
ident 
Sbjct -------------------------------------------ksvplpdiirnlv  128
DSSP  -------------------------------------------eellllhhhhlll
```

No 714: Query=mol1A Sbjct=1yvlB Z-score=2.5

back to top

```
DSSP  llllllllllhhhhhhhhhhhhhhllllhhhhhhhhhhhhhhhhhhlllllllllleeee
Query aapysspptnlrslrdrltqvaerqgvvfgrlqrhvaxivvaqfaatltddtgaplllvk   60
ident 
Sbjct ------------------------------------------------------------    0
```

```
DSSP  hhhhhhhhllllllllllleeeeelllhhhhhhhhhhhhhHLLLLEeeeellleelllll
Query ggsslelrrgipdsrtskdfatvarrdieliheqladageTGWEGFtaiftapeeiaapg  120
ident                                            |
Sbjct ---------------------------------------sQWYELQ--------------    7
DSSP  ---------------------------------------lHHHHHH--------------


DSSP  llllleeeeeeeeeLLEEE-----------------------------------------
Query xpvkprrftaklsyRGRAF-----------------------------------------  139
ident                   |
Sbjct -------------qLDSKFleqvhqlyddsfpmeirqylaqwlekqdwehaandvsfati   54
DSSP  -------------lLLLLLhhhhhllllllllhhhhhhlhhhhhhllhhhhlllhhhhhh


DSSP  -----------------------------------------------------------e
Query -----------------------------------------------------------a  140
ident
Sbjct rfhdllsqlddqysrfslennfllqhnirkskrnlqdnfqedpiqmsmiiysclkeerki  114
DSSP  hhhhhhhhhhhhhhhhhhhllhhhhhhhhhhhhhhhhhhlllhhhhhhhhhhhhhhhhhh


DSSP  eeeeeeeellllllLLLEeeeelhhhhlllllleeeeeLLHHHHHHHHHHHH---hllLL
Query tvpievssveagnaDQFDtltsdalglvgvpaavavpcXTIPWQIAQKLHAV---tavLE  197
ident                                                  |
Sbjct lenaqrfnmldkqkELDS------------------kvRNVKDKVMCIEHEIksledlQD  156
DSSP  hhlllllllhhhhhHHHH------------------hhHHHHHHHHHHHHHHhhhhhhHH


DSSP  LLL---------------------------llllLHHHHHHHHHHHH--LLHH-------
Query EPK---------------------------andrAHDLVDLQLLEGL--LLDA-------  221
ident |                                          |     |
Sbjct EYDfkcktlqnrehdqkqeqlllkkmylmldnkrKEVVHKIIELLNVteLTQNalindel  216
DSSP  HHHhhhhhllllllllllllllllhhhhhhhhhhHHHHHHHHHHHHHhhHHHHhhhhlhh


DSSP  -------------------------hhhHHHhhhhhhhhhhhllllLLLL--LLLL--LH
Query -------------------------dlxPTRSaciaifearaqhpwPPRV--ATLP--HW  252
ident
Sbjct vewkrrqqsaciggppnacldqlqnwftIVAE---------slqqvRQQLkkLEELeqKY  267
DSSP  hhhhhhhhhhhhllllllllhhhhhhhhHHHH---------hhhhhHHHHhhHHHHhhHL


DSSP  HHhhhhhhllllllllllLHHHHHHHHHHHHHHHHH-LLLL-------------------
Query PLiyagalegldhlelarTVDAAAQAVQRFVARIDR-ATKR-------------------  292
ident                         |
Sbjct TY-------------ehdPITKNKQVLWDRTFSLFQqLIQSsfvverqpcmpthpqrplv  314
DSSP  LL-------------lllHHHHHHHHHHHHHHHHHHhHHHHleeeeeeeeelllllllle


DSSP  ------------------------------------------------------------
Query ------------------------------------------------------------  292
ident
Sbjct lktgvqftvklrllvklqelnynlkvkvlfdkdvnerntvkgfrkfnilgthtkvmnmee  374
DSSP  eelllleeeeeeellllhhhlllleeeeeellllllllllllllleeelllleeeleell


DSSP  ------------------------------------------------------------
Query ------------------------------------------------------------  292
ident
Sbjct stngslaaefrhlqlkeqgplivteelhslsfetqlcqpglvidlettslpvvvisnvsq  434
DSSP  llllleeeeeeeeleeelllllllleeleeeeeeeeeelleeeeeeeelllleeeellhhh


DSSP  ------------------------------------------------------------
Query ------------------------------------------------------------  292
ident
Sbjct lpsgwasilwynmlvaeprnlsffltppcarwaqlsevlswqfssvtkrglnvdqlnmlg  494
DSSP  hhhhhhhhhhhhhhlllllllllllllleelhhhhhhhhhhhhlllllllllhhhhhhhh


DSSP  ------------------------------------------------------------
Query ------------------------------------------------------------  292
ident
Sbjct ekllgpnaspdglipwtrfckenindknfpfwlwiesilelikkhllplwndgcimgfis  554
DSSP  hhhlllllllllleehhhhhllllllllllhhhhhhhhhhhhhhhhlhhhhhllllllll
```

```
Sbjct kererallkdqqpgtfllrfsessregaitftwversnggepdfhavepytkkelsavtf  614
DSSP  hhhhhhhllllllleeeeellllllleeeeelllllllllleellllllhhhhllllh


DSSP  ---------------------------------------
Query ---------------------------------------  292
ident 
Sbjct pdiirnykvmaaenipenplkylypnidkdhafgkyysr  653
DSSP  hhhhhhlleelllllleellllllllllhhhhhhhhlll
```

No 715: Query=mol1A Sbjct=2a4wA Z-score=2.5

back to top

```
DSSP  lllllllllhhhhhhhhhhhhhhllllhhHHHH--hHHHHHHHHHHHlllllllllee
Query aapyssppptnlrslrdrltqvaerqgvvfgRLQR--hVAXIVVAQFAAtltddtgaplll   58
ident 
Sbjct -----------------------sarislfAVVVedmAKSLEFYRKLG------veipae   31
DSSP  -----------------------lleeeeeEEEEllhHHHHHHHHLLL------llllhh


DSSP  eehhhhhHHHHlllLLLLL----------------------LLEEEEEL--LLHHHHHHH
Query vkggsslELRRgipDSRTS----------------------KDFATVAR--RDIELIHEQ   94
ident        |        |                           |
Sbjct adsaphtEAVL-dgGIRLAwdtvetvrsydpewqaptgghrFAIAFEFPdtASVDKKYAE   90
DSSP  hhhlleeEEEL-hhHLEEEeeehhhhhlllllllllllllEEEEEEELLlhHHHHHHHHH


DSSP  HHHHHhhllllleeeEELLLEELLLLLLlLLLEEEEEEEeeelleeeEEEEEEEEELLllll
Query LADAGetgwegftaIFTAPEEIAAPGXpVKPRRFTAKlsyrgrafATVPIEVSSVEagna  154
ident | |||                  |
Sbjct LVDAG---------YEGHLKPWNAVWG-QRYAIVKDP--------DGNVVDLFAPL----  128
DSSP  HHHLL---------LLEEEEEEELLLL-EEEEEEELL--------LLLEEEEEEEL----


DSSP  llleeeeelhhhhllllllleeeeellhhhhhhhhhhhhhlllllllllllhhhhhhhhh
Query dqfdtltsdalglvgvpaavavpcxtipwqiaqklhavtavleepkandrahdlvdlqll  214
ident 
Sbjct ------------------------------------------------------------  128
DSSP  ------------------------------------------------------------


DSSP  hhhllhhhhhhhhhhhhhhhhhhhllllllllllllllhhhhhhhhhllllllllllhhh
Query eglllddadlxptrsaciaifearaqhpwpprvatlphwpliyagalegldhlelartvda  274
ident 
Sbjct ------------------------------------------------------------  128
DSSP  ------------------------------------------------------------


DSSP  hhhhhhhhhhhhhhllll
Query aaqavqrfvaridratkr  292
ident 
Sbjct ------------------  128
DSSP  ------------------
```

No 716: Query=mol1A Sbjct=5fu7A Z-score=2.4

back to top

```
DSSP  lllllllllhhhhhhhhhhhhhhllllhhhhhhhhhhhhhhhhhhhlllllllLLEE--
Query aapyssppptnlrslrdrltqvaerqgvvfgrlqrhvaxivvaqfaatltddtgaPLLL--   58
ident                                                       |  |
Sbjct ------------------------------------------------aseyddPPGLre   12
DSSP  ------------------------------------------------llllllLLLHhh


DSSP  EEHHHHHHhhhlllLLLLllleeeeelllhhhhhhhhhhhhhhllllleeeeellleelll
Query VKGGSSLElrrgipDSRTskdfatvarrdieliheqladagetgwegftaiftapeeiaa  118
ident        |
Sbjct KAEYLLRE---wvnLYHS------------------------------------------   27
DSSP  HHHHHHHH---hhhHHHL------------------------------------------
```

```
Query pgxpvkprrftaklsyrgrafatvpievssveagnadqfdTLTSdalglvgvpaavAVPC  178
ident
Sbjct ------------------------aaagrdstkafsafvgQMHQ------------QGIL   51
DSSP  ------------------------llllllhhhhhhhhhhHHHH------------LLLL


DSSP  -------lLHHHHHHHHHHHHH--LLLL--LLLL---lLLLHHHHHHHHHHHHL------
Query -------xTIPWQIAQKLHAVT--AVLE--EPKA---nDRAHDLVDLQLLEGLL------  218
ident                                | |     ||     |     |
Sbjct ktddlitrFFRLCTEMCVEISYraQAEQqhNPAAnptmIRAKCYHNLDAFVRLIallvkh  111
DSSP  llhhhhhhHHHHHHHHHHHHHHhhHHHHhlLLLLlhhhHHHHHHHHHHHHHHHHhhhhhl


DSSP  -------------lHHHHHHHHHHHHHHHHHHHLLLLLLlllllllhhhhhhhhhlllll
Query -------------lDADLXPTRSACIAIFEARAQHPWPPrvatlphwpliyagalegldh  265
ident                  |             |
Sbjct sgeatntvtkinllNKVLGIVVGVLLQDHDVRQSEFQQL---------------------  150
DSSP  lllllllhhhhhhhHHHHHHHHHHHHHHHHHHHHHLLLH---------------------


DSSP  llllllhhhhhhhhHHHHH------------------hhhhLLLL---------------
Query lelartvdaaaqavQRFVA------------------ridrATKR---------------  292
ident                |
Sbjct ------------pyHRIFImlllelnaletinfqtltafcnTFHIlrptkapgfvyawle  198
DSSP  ------------hhHHHHhhhhhhllllhhhhhhhhhhhhhHHHHllllllhhhhhhhhh


DSSP  ------------------------------------------------------------
Query ------------------------------------------------------------  292
ident
Sbjct lishrifiarmlahtpqqkgwpmyaqllidlfkylapflrnveltkpmqilykgtlrvll  258
DSSP  hhllhhhhhhhhlllhhhlhhhhhhhhhhhhhhhhhhhhhlllllhhhhhhhhhhhhhhh


DSSP  ------------------------------------------------------------
Query ------------------------------------------------------------  292
ident
Sbjct vllhdfpeflcdyhygfcdvippnciqlrnlilsafprnmrlpdpftpnlkvdmlseini  318
DSSP  hhhhhlhhhhhhlhhhhhhhlllllhhhhhhhhhlllllllllllllllllllllhhhhl


DSSP  ------------------------------------------------------------
Query ------------------------------------------------------------  292
ident
Sbjct apriltnftgvmppqfkkdldsylktrspvtflsdlrsnlqvsnepgnrynlqlinalvl  378
DSSP  lllllllhhhhllhhhhhhhhhhhhhllllhhhhhhhhhleelllllleelhhhhhhhhh


DSSP  ------------------------------------------------------------
Query ------------------------------------------------------------  292
ident
Sbjct yvgtqaiahihnkgstpsmstithsahmdifqnlavdldtegrylflnaianqlrypnsh  438
DSSP  hhhhhhhhhhhhlllllllllllllhhhhhhhhhhhhhllhhhhhhhhhhhlllllllhh


DSSP  ------------------------------------------------------------
Query ------------------------------------------------------------  292
ident
Sbjct thyfsctmlylfaeanteaiqeqitrvllerlivnrphpwgllitfieliknpafkfwnh  498
DSSP  hhhhhhhhhhhhllllhhhhhhhhhhhhhhhhhllllllhhhhhhhhhhhlllhhhhhhh


DSSP  ---------------------
Query ---------------------  292
ident
Sbjct efveeepeieklfqsvaqccm  519
DSSP  hhhhlllhhhhhhhhhhhhll
```

No 717: Query=mol1A Sbjct=5i6eA Z-score=2.4

back to top

```
DSSP  llllllllllhhhhhhhhhhhhhhhllllhhhhhhhhhHHHHH-------hhhlllllll
Query aapysspptnlrslrdrltqvaerqgvvfgrlqrhvaxIVVAQ-------faatltddtg   53
```

```
DSSP  llleeeehHHHHHHH---------------------------------------------
Query aplllvkgGSSLELR---------------------------------------------   68
ident            |
Sbjct nqvimnasLQQLIEVlrnpklpysewklhisalhsrlpakldeqmeelvarslrrgavfp   88
DSSP  llllhhhhHHHHHHHhllllhhhhhhhhhhhhhlllllhhhhhhhhhhhhhhhhhlllll


DSSP  -----hllllllllllleeeeellLHHHHHHhhhhhhhhlllleeeeellleellllllll
Query -----rgipdsrtskdfatvarrDIELIHEqladagetgwegftaiftapeeiaapgxpv  123
ident
Sbjct arqlsklidmavknpeynpdkllGAVVEPL------------------------------  118
DSSP  hhhhhhhhhhhhhlllllllllhHHHLHHH------------------------------


DSSP  lleeeeeeeeelleeeeeeeeeeeelllllLLLLEeeeelhhhhlllllleeeeeLLHHH
Query kprrftaklsyrgrafatvpievssveagnADQFDtltsdalglvgvpaavavpcXTIPW  183
ident
Sbjct -----------------------adiahkySNGLE--------------------AHEHS  135
DSSP  -----------------------hhhhhhlLLLHH--------------------HHHHH


DSSP  HHHHHHHHHHLL--------------------------lLLLLL-------lLLLHHHHH
Query QIAQKLHAVTAV--------------------------lEEPKA-------nDRAHDLVD  210
ident      |     |
Sbjct IFVHFLEEYYEVeklfngpnvreeniilklrdenpkdldKVALTvlshskvsAKNNLILA  195
DSSP  HHHHHHHHHHHHhhhhllllllhhhhhhhhhhhlllllhHHHHHhhhhhlhhHHHHHHHH


DSSP  HHHHHHHL---LHHHHHHHHhhhhhhhhhhlllllLLLL-LLLLlhhhhhhhhhllllll
Query LQLLEGLL---LDADLXPTRsaciaifearaqhpwPPRV-ATLPhwpliyagalegldhl  266
ident        |                              |
Sbjct ILKHYQPLcklSSKVSAIFS------------tplQHIVeLESK----------------  227
DSSP  HHHHHHHHhhhLHHHHHHHH------------hhhHHHHlLLLL----------------


DSSP  lllllHHHHHHHHHHHHHHH----------------------------------------
Query elartVDAAAQAVQRFVARI----------------------------------------  286
ident          |
Sbjct ----aTAKVALQAREILIQGalpsvkerteqiehilkssvvkvaygssnpkrsepdlnil  283
DSSP  ----lLHHHHHHHHHHHHLLlhhhhhhhhhhhhhhhhhhhllllllllllllllllhhhh


DSSP  ------------------------------------------------------------
Query ------------------------------------------------------------  286
ident
Sbjct kdlidsnyvvfdvllqflthqdpvvtaaaaqvyirrayraytigdirvhegvtvpivewk  343
DSSP  hhhhllllllhhhhhhllllllhhhhhhhhhhhhhhlllleeeeeeeeelllllleeeee


DSSP  ------------------------------------------------------------
Query ------------------------------------------------------------  286
ident
Sbjct fqlpsaafstfptvmgmnravxvsdlsyvansqssplregilmavdhlddvdeilsqsle  403
DSSP  eelllhhhllllllllllllllllllllllllllllleeeeeeeeeehhhhhhhhhhhll


DSSP  ----------------------------------------------------------hh
Query ----------------------------------------------------------dr  288
ident
Sbjct viprhqsaslsnvanvcvastegfeseeeilvrlreildlnkqelinasirritfmfgfk  463
DSSP  llllllllllleeeeeeeeeelllllhhhhhhhhhhhhhhlhhhhlllleeeeeeeelll


DSSP  LLLL--------------------------------------------------------
Query ATKR--------------------------------------------------------  292
ident
Sbjct DGSYpkyytfngpnynenetirhiepalafqlelgrlsnfnikpiftdnrnihvyeavsk  523
DSSP  LLLLleeeeeelllleelhhhllllhhhhhhlllllllleeeeeellllllleeeeeeeeel


DSSP  ------------------------------------------------------------
Query ------------------------------------------------------------  292
ident
Sbjct tspldkrfftrgiirtghirddisiqeyltseanrlmsdildnlevtdtsnsdlnhifin  583
DSSP  llllleeeeeeeeeelllllllllhhhhhhhhhhhhhhhhhhhhhhlllllllleeeeee
```

```
Query ------------------------------------------------------------  292
ident
Sbjct fiavfdispedveaafggflerfgkrllrlrvssaeiriiikdpqtgapvplralinnvs  643
DSSP  eeeeelllhhhhhhhlllllllllhhhhhhlleeeeeeeeeeelllllleeeeeeeeelll


DSSP  --------------------------------------------
Query --------------------------------------------  292
ident
Sbjct gyviktemytevknakgewvfkslgkpgsmhlrpiatpypvkew  687
DSSP  lllleeeeeeleellllleellllllllllllllllllllll
```

No 718: Query=mol1A Sbjct=3omsA Z-score=2.4

back to top

```
DSSP  lllllllllhhhhhhhhhhhhhhllllhhhhhhhhhhhhhhhhhhlllllllllleeee
Query aapysspptnlrslrdrltqvaerqgvvfgrlqrhvaxivvaqfaatltddtgaplllvk   60
ident
Sbjct ------------------------------------------------------------    0
DSSP  ------------------------------------------------------------


DSSP  hhhhhhhhhlllllllLLLEEEEEELLLHHHHHHHHHHHHhhllllleeEEELLLEELLL--
Query ggsslelrrgipdsrtSKDFATVARRDIELIHEQLADAGetgwegftAIFTAPEEIAA--  118
ident                             |
Sbjct ---------------qKITTFLXFEGKAEEAXNFYTSLF--------DQSEIVSISRYde   37
DSSP  ---------------lLLLEEEEELLLHHHHHHHHHLLL--------LLLLEEEEEELll


DSSP  -LLLL---lLLEEEEEEEeeelleeeeEEEEEEEEL--LLLLLllLEEE------------
Query -PGXP---vKPRRFTAKlsyrgrafaTVPIEVSSV--EAGNAdqFDTL------------  160
ident              ||                             |
Sbjct nGPGKegtvIHATFTLN---------GQEFXCIDSyvNHNFT--FTPAxslyvtceteee   86
DSSP  lLLLLllleEEEEEEEL---------LEEEEEEELllLLLLL--LLLLlleeeeellhhh


DSSP  ---------------eelhhHHLLLLLLEEEeellhhhhhhhhhhhhhllllllllll
Query ---------------tsdalGLVGVPAAVAVpcxtipwqiaqklhavtavleepkandra  205
ident
Sbjct idtvfhklaqdgailxplgsYPFSKKFGWLN-----------------------------  117
DSSP  hhhhhhhhhllleeeeeeeeELLEEEEEEEE-----------------------------


DSSP  hhhhhhhhhhhllhhhhhhhhhhhhhhhhhllllllllllllllhhhhhhhhhlllll
Query hdlvdlqllegllldadlxptrsaciaifearaqhpwpprvatlphwpliyagalegldh  265
ident
Sbjct ------------------------------------------------------------  117
DSSP  ------------------------------------------------------------


DSSP  llllllhhhhhhhhhhhhhhhhhllll
Query lelartvdaaaqavqrfvaridratkr  292
ident
Sbjct ---------------dkygvswqltla  129
DSSP  ---------------llllleeeeeel
```

No 719: Query=mol1A Sbjct=2i52F Z-score=2.4

back to top

```
DSSP  llllLLLL-----llhHHHHhhhhhhhhhllllhhhhhhHHHHHHHHHHHHHlllllllll
Query aapySSPP-----tnlRSLRdrltqvaerqgvvfgrlqrHVAXIVVAQFAAtltddtgap   55
ident     |                                    | |
Sbjct ----SLYDpaekyfncTDIQ----------------rafFEAGIKLGAIFH---------   31
DSSP  ----LLLLllhhhlllLHHH----------------hhhHHHHHHHHHHHH---------


DSSP  leeeehhhhhhhhhllllllllleeEEELL---LHHHHHHHHHHHHHHHLlLLEEEEELL
Query lllvkggsslelrrgipdsrtskdfaTVARR---DIELIHEQLADAGETGwEGFTAIFTA  112
ident                           |             |
```

```
DSSP  LEEllllLLLL----lLEEEEEEEEEL-lEEEEEEEeeeeellllllllleeeeelhhhh
Query PEEiaapGXPV----kPRRFTAKLSYR-gRAFATVPievssveagnadqfdtltsdalgl  167
ident                        |          |
Sbjct INNvkrsTYSYsslneKMLHAEVLINYngKKVLGVL------------------------  102
DSSP  ELLllllLLLLeelllLLEEEEEEEEEllEEEEEEE------------------------


DSSP  llllllleeeeellhhhhhhhhhhhhhlllllllllllhhhhhhhhhhhllhhhhhhhh
Query vgvpaavavpcxtipwqiaqklhavtavleepkandrahdlvdlqlleglllddadlxptr  227
ident
Sbjct ------------------------------------------------------------  102
DSSP  ------------------------------------------------------------


DSSP  hhhhhhhhhhllllllllllllhhhhhhhhhllllllllllhhhhhhhhhhhhhhh
Query saciaifearaqhpwpprvatlphwpliyagalegldhlelartvdaaaqavqrfvarid  287
ident
Sbjct ------------------------------------------------nydegldypvmy  114
DSSP  ------------------------------------------------eeellllleeeee


DSSP  hllll
Query ratkr  292
ident
Sbjct akevl  119
DSSP  eeeel
```

No 720: Query=mol1A Sbjct=1kw2A Z-score=2.4

back to top

```
DSSP  --------------------------------------------llllllllLLHHHhhh
Query --------------------------------------------aapyssppTNLRSlrd   16
ident
Sbjct grdyeknkvckefshlgkedftslslvlysrkfpsgtfeqvsqlvkevvsltEACCA---   57
DSSP  lllhhhhhhhhhhhhhlhhhhhhhhhhhhhhhhlllllhhhhhhhhhhhhhhHHHHL---


DSSP  hhhhhhhhllllhhhhhhhhhhhhhhhhhhllllllllleeeehhhhhhhhhlllllll
Query rltqvaerqgvvfgrlqrhvaxivvaqfaatltddtgaplllvkggsslelrrgipdsrt   76
ident
Sbjct ------------------------------------------------------------   57
DSSP  ------------------------------------------------------------


DSSP  llleeeeeelllhhhhhhhhhhhhhhhlllleeeEELL---------------------LEE
Query skdfatvarrdieliheqladagetgwegftaIFTA---------------------PEE  115
ident
Sbjct -------egadpdcydtrtsalsakscesnspFPVHpgtaecctkeglerklcmaalKHQ  110
DSSP  -------llllllhhhhhhhhhhhhlllllllLLLLllhhhhllllhhhhhhhhhhlLLL


DSSP  L-----------------------------------------------------------
Query I-----------------------------------------------------------  116
ident
Sbjct Pqefptyveptndeiceafrkdpkeyanqfmweystnygqaplsllvsytksylsmvgsc  170
DSSP  Lllllllllllhhhhhhhhhhlhhhhhhhhhhhhhhhlllllhhhhhhhhhhhhhhhhhh


DSSP  ---------------------------------lLLLLllllleeeeeeeeelleeeeeee
Query ---------------------------------aAPGXpvkprrftaklsyrgrafatvp  143
ident                                   |
Sbjct ctsasptvcflkerlqlkhlsllttlsnrvcsqyAAYG----------------------  208
DSSP  llllhhhhhhhhhhhhhhhhhhhhhhhhhhhhhHHHH----------------------


DSSP  eeeeellllllllleeeeelhhhhllllleeeeeLLHHHHHHHHHHHHHL--LLLLllL
Query ievssveagnadqfdtltsdalglvgvpaavavpcXTIPWQIAQKLHAVTA--VLEEpkA  201
ident                                           |
Sbjct ---------------ekksrlsnliklaqkvptadLEDVLPLAEDITNILSkcCESA-sE  252
DSSP  ---------------hhhhhhhhhhhhhhhlllllHHHHHHHHHHHHHHHHhhLLLL-lL
```

```
Sbjct DCMAKELPEHTVKLCDNlSTKN--------------------------------------  274
DSSP  LHHHHHHHHHHHHHHHHhLLLL--------------------------------------


DSSP  lllllllllllhHHHHHHHHHHHHHhhHLLLL----------------------------
Query egldhlelartvDAAAQAVQRFVARidRATKR----------------------------  292
ident               |             |
Sbjct ---skfedccqeKTAMDVFVCTYFM--PAAQLpelpdvelptnkdvcdpgntkvmdkytf  329
DSSP  ---hhhhhhlllLLHHHHHHHHHHL--LLLLLlllllllllllhhhllllllhhhhhhhh


DSSP  ------------------------------------------------------------
Query ------------------------------------------------------------  292
ident
Sbjct elsrrthlpevflskvleptlkslgeccdvedsttcfnakgpllkkelssfidkgqelca  389
DSSP  hhhhllllhhhhhhhhhhhhhhhlhhhlllllhhhhhhhhhhhhhhhhhhhhhhhhhhll


DSSP  ------------------------------------------------------------
Query ------------------------------------------------------------  292
ident
Sbjct dysentfteykkklaerlkaklpdatpkelaklvnkrsdfasnccsinspplycdseida  449
DSSP  llllllhhhhhhhhhhhhhhhlllllhhhhhhhhhhhhhhhhhhllllllhhhhhhhhhh


DSSP  ------
Query ------  292
ident
Sbjct elknil  455
DSSP  hhllll
```

No 721: Query=mol1A Sbjct=3ct8A Z-score=2.4

back to top

```
DSSP  llllllllllhhhhhhhhhhhhhhllLLHHHHHHhhhhhhhhhhhllllllllllleeee
Query aapysspptnlrslrdrltqvaerqgVVFGRLQRhvaxivvaqfaatltddtgaplllvk   60
ident
Sbjct --------------------------NLYFQGXL--------------------------    8
DSSP  --------------------------LLLLLLLL--------------------------


DSSP  hhhhhhhhhllllllllLLEEEEELLlHHHHHHHHHHHHHHLllleEEEELLLEElllll
Query ggsslelrrgipdsrtsKDFATVARRdIELIHEQLADAGETGwegfTAIFTAPEEiaapg  120
ident                             |
Sbjct -----------------HHVEINVDH-LEESIAFWDWLLGEL----GYEDYQSWS-----   41
DSSP  -----------------LEEEEEELL-HHHHHHHHHHHHHHL----LLEEEEEEL-----


DSSP  llllleeEEEEEeelleeEEEEEEeEEELLLLLLLLLEE---------------------
Query xpvkprrFTAKLsyrgraFATVPIeVSSVEAGNADQFDT---------------------  159
ident           |         |    |          |
Sbjct -----rgKSYKH------GKTYLVfVQTEDRFQTPTFHRkrtglnhlafhaasrekvdel   90
DSSP  -----leEEEEE------LLEEEEeEELLHHHLLLLLLLlllllleeeeelllhhhhhhh


DSSP  ---------------eeelhhhhlLLLLLEeeeellhhhhhhhhhhhhhlllllllllll
Query ---------------ltsdalglvGVPAAVavpcxtipwqiaqklhavtavleepkandr  204
ident
Sbjct tqklkergdpilyedrhpfaggpnHYAVFC------------------------------  120
DSSP  hhhhhhllllllllllllllllllLLEEEE------------------------------


DSSP  lhhhhhhhhhhhhllhhhhhhhhhhhhhhhhhhlllllllllllllllhhhhhhhhhllll
Query ahdlvdlqlleglllldadlxptrsaciaifearaqhpwpprvatlphwpliyagalegld  264
ident
Sbjct ------------------------------------------------------------  120
DSSP  ------------------------------------------------------------


DSSP  lllllllhhhhhhhhhhhhhhhhhllll
Query hlelartvdaaaqavqrfvaridratkr  292
ident
Sbjct ---------------edpnrikveivap  133
```

No 722: Query=mol1A Sbjct=5dhhB Z-score=2.4

[back to top]

```
DSSP  lllllllllhhhhhhhhhhhhhhllllhhhhhhhhhhhhhhhhhhlllllllllleeee
Query aapysspptnlrslrdrltqvaerqgvvfgrlqrhvaxivvaqfaatltddtgaplllvk   60
ident
Sbjct ------------------------------------------------------------    0
DSSP  ------------------------------------------------------------


DSSP  hhhhhhhhhlllllllllleeeeelllhhhhhhhhhhhhhhllllleeeeellleelllll
Query ggsslelrrgipdsrtskdfatvarrdielihеqladagetgwegftaiftapeeiaapg  120
ident
Sbjct ------------------------------------------------------------    0
DSSP  ------------------------------------------------------------


DSSP  lllllеeeeeeeeelleeeeeeeeeeeellllllllleeeeelhhhhllllllleeeeeLL
Query xpvkprrfftaklsyrgrafatvpievssveagnadqfdtltsdalglvgvpaavavpcXT  180
ident
Sbjct ---------------------------------------------------------lED    3
DSSP  ---------------------------------------------------------lHH


DSSP  HHHHHHHHHHHHHHLllllllLLLL-LHHHHHHHHHHHHHLL---------hhhhhhhhhh
Query IPWQIAQKLHAVTAvleepkANDR-AHDLVDLQLLEGLLL---------dadlxptrsac  230
ident          |                       |
Sbjct NWETLNDNLKVIEK----adNAAQvKDALTKMRAAALDAQkatppkledkspdspemkdf   59
DSSP  HHHHHHHHHHHHHH----llLHHHhHHHHHHHHHHHHHHHllllllllllllllhhhhhh


DSSP  hhhhhhhllllllllllLLLLHhhhhhhhhllllllllllhhhhHHHHHHHHHHHHH-L
Query iaifearaqhpwpprvaTLPHWpliyagalegldhlelartvdaaAQAVQRFVARIDR-A  289
ident                                              |
Sbjct rhgfdilvgqiddalklANEGK------------------vkeaQAAAEQLKTTRNAyI  100
DSSP  hhhhhhhhhhhhhhhhhHHLLL------------------lhhhHHHHHHHHHHHHHhH


DSSP  LLL---------------------------------------------------------
Query TKR---------------------------------------------------------  292
ident  |
Sbjct QKYlaflplglkvtivglylavcvgglgnclvmyvilrhtkmktatniyifnlaladtl  160
DSSP  HLLlllllhhhhhhhhhhhhhhhhhhhhhhhhhhhhhhhhllllllhhhhhhhhhhhhhh


DSSP  ------------------------------------------------------------
Query ------------------------------------------------------------  292
ident
Sbjct vlltlpfqgtdillgfwpfgnalcktviaidyynmftstftltamsvdryvaichpiral  220
DSSP  hhhhhhhhhhhhhhlllllhhhhhhhhhhhhhhhhhhhhhhhhhhhhhhhhhhlhhhhh


DSSP  ------------------------------------------------------------
Query ------------------------------------------------------------  292
ident
Sbjct dvrtsskaqavnvaiwalasvvgvpvaimgsaqveeieclveiptpqdywgpvfaicifl  280
DSSP  hhllhhhhhhhhhhhhhhhhhhhhhhhhhhheellllllllееllllllhhhhhhhhhhhhh


DSSP  ------------------------------------------------------------
Query ------------------------------------------------------------  292
ident
Sbjct fsfivpvlvisvcyslmirrlrgsrekdrnlrritrlvlvvvavfvgcwtpvqvfvlaqg  340
DSSP  hhlhhhhhhhhhhhhhhhhhllllllllhhhhhhhhhhhhhhhhhhhhhhhhhhhhhhhhh


DSSP  -----------------------------------------------
Query -----------------------------------------------  292
ident
Sbjct lgvqpssetavailrfctalgyvnsclnpilyafldenfkacfrkfc  387
DSSP  lllllllhhhhhhhhhhhhhhhhhhhhhhhhhhhhhlhhhhhhhhhhl
```

back to top

```
DSSP  llllllllllhhhhhhhhhhhhhhllllhhhhhhhhhhhhhhhhhhlllllllllleeee
Query aapysspptnlrslrdrltqvaerqgvvfgrlqrhvaxivvaqfaatltddtgaplllvk   60
ident
Sbjct ------------------------------------------------------------    0
DSSP  ------------------------------------------------------------


DSSP  hhhhhhhhllllllllllleeeeelllhhhhhhhhhhhhhlllleeeeellleellllll
Query ggsslelrrgipdsrtskdfatvarrdieliheqladagetgwegftaiftapeeiaapg  120
ident
Sbjct ------------------------------------------------------------    0
DSSP  ------------------------------------------------------------


DSSP  llllleeeeeeeeelleeeeeeeeeeeelllllllleeeeelhhhhlllllleeeeeeLL
Query xpvkprrftaklsyrgrafatvpievssveagnadqfdtltsdalglvgvpaavavpcXT  180
ident                                                           |
Sbjct ----------------------------------------------------------XN    2
DSSP  ----------------------------------------------------------LH


DSSP  HHHHHHHHHHHHhlllllllllLLLH-HHHHHHHHHHHL--LHHHHHHHHHHhhhhhhhh
Query IPWQIAQKLHAVtavleepkanDRAH-DLVDLQLLEGLL--LDADLXPTRSAciaifear  237
ident         |                         |
Sbjct RLEKFFTTLNTA----------QSKVlKGYTTDELVSQIkeYVDFTPYILKQ--------   44
DSSP  HHHHHHHHHHHL----------LLHHhHHHHHHHHHHLLllHHHHHHHHHHH--------


DSSP  lllllllLLLLlllhhhhhhhhhlllllllllllhhhhHHHHHHHHHH------------
Query aqhpwppRVATlphwpliyagalegldhlelartvdaaAQAVQRFVAR------------  285
ident                                                |
Sbjct --tyrllCGQA-------------------------seDRRNGARILRslxfqfklvtdf   77
DSSP  --hhhhhLLLL-------------------------hhHHHHHHHHHHllllleelllll


DSSP  ----------------------hhhLLLL-------------------------------
Query ----------------------idrATKR-------------------------------  292
ident
Sbjct kieykesssiylsstgeqfnderpiENVLdffeqisdnllsyewykrhgaflafaaxfse  137
DSSP  lllllllllllllllllllllllllLLHHhhhhhhhhhhhhlllhhhhhhhhhhhhhlll


DSSP  ------------------------------------------------------------
Query ------------------------------------------------------------  292
ident
Sbjct idnggdiqirvdsklfskiyeilvtdkfndfvddrtvapvrdaaayllsriypligpndi  197
DSSP  llllleellllllhhhhhhhhhhhhllllllllllllhhhhhhhhhhhhllllllllllh


DSSP  ------------------------------------------------------------
Query ------------------------------------------------------------  292
ident
Sbjct ieqlvgfldsgdwqvqfsglialgylkefvedkdglcrklvsllsspdedikllsaellc  257
DSSP  hhhhhhhhhlllhhhhhhhhhhhlllllllllhhhhhhhhhhllllllhhhhhhhhhhhl


DSSP  ------------------------------------------------------------
Query ------------------------------------------------------------  292
ident
Sbjct hfpitdsldlvlekcwknieseelisvsktsnlslltkiyrenpelsipperlkdifpcf  317
DSSP  lllllllhhhhhhhhhhhhhllllllllhhhhhhhhhhhhhhllllllllllhhhlhhhh


DSSP  ------------------------------------------------------------
Query ------------------------------------------------------------  292
ident
Sbjct tspvpevrtsilnxvknlseesidflvaevvlieekdeirexaikllkklpknlilhfxn  377
DSSP  hlllhhhhhhhhhhllllllhhhhhhhhhhhhllllhhhhhhhhhhhllllhhhhhhhhh


DSSP  ------------------------------------------------------------
Query ------------------------------------------------------------  292
ident
Sbjct viggslyepyseddfvsyedlyftksginvvgkdeilknrcllfecixksglpdlqstit  437
DSSP  hhllllllllllllllllllllllhhhhhhhhhhhhhhhhhhhhhhhlllllllllllll
```

```
Query -------------------------------------------------------------  292
ident
Sbjct tsrtfislyrsvqalvpanieeleyyfdrckdlkxaplkefkkklsapgirsihpxvdpl  497
DSSP  llhhhhhhhhhhhhhllllllllhhhhhllhhhhllhhhhhhhhhlllllllllhhhh


DSSP  -------------------------------------------------------------
Query -------------------------------------------------------------  292
ident
Sbjct ysdytrxvasiefpgleratalfevetckqflhlfskxiteyydaekisidnfllkayeg  557
DSSP  hhhhhhhhhhhlllllllhhhhhhhllllhhhhhhhhhhhhhhhhlllllllhhhhhhhhh


DSSP  -------------------------------------------------------------
Query -------------------------------------------------------------  292
ident
Sbjct lasgkdgflsffevfntrllahsffhkigslenrldffsktihiytktsqiqkigfvfdd  617
DSSP  hhhllhhhhhhhhhlllhhhhllhhhhhlllllhhhhlllllllllllhhhhllhhhhhh


DSSP  -------------------------------------------------------------
Query -------------------------------------------------------------  292
ident
Sbjct alreknitvingfxrslefnekfvrkaledldvelldavlxsgdhsfnplfvkpllrnis  677
DSSP  hhhhllhhhhhhhlllhhhhhhhhhhhhhlllhhhhhhhlllllllhhhlhhhhhhhhh


DSSP  ---------------------------------------------
Query ---------------------------------------------  292
ident
Sbjct gnidreasskvlskviptlgfstntkiskdllexierekkslesl  722
DSSP  llllhhhhhhhhhhhlllllllllllllhhhhhhhhhhhhhhlll
```

No 724: Query=mol1A Sbjct=3cwgB Z-score=2.4

back to top

```
DSSP  llllllllllhhhhhhhhhhhhhhllllhhhhhhhhhhhhhhhhhhlllllLLLLLeeee
Query aapysspptnlrslrdrltqvaerqgvvfgrlqrhvaxivvaqfaatltddTGAPLllvk   60
ident
Sbjct ---------------------------------------------------VVTEK----    5
DSSP  ---------------------------------------------------LLLLH----


DSSP  hhhhhhhhhlllllllllleeeeelllhhhhhhhhhhhhhhllllleeeeellleelllll
Query ggsslelrrgipdsrtskdfatvarrdielihеqladagetgwegftaiftapeeiaapg  120
ident
Sbjct -------------------------------------------------------------    5
DSSP  -------------------------------------------------------------


DSSP  lllllеeeeeeeeelleeeeeeeeeeeelllllllLLLEeeeelhhhhlllllleeeeeLL
Query xpvkprrftaklsyrgrafatvpievssveagnaDQFDtltsdalglvgvpaavavpcXT  180
ident
Sbjct -------------------------------qqmLEQH------------------lQD   15
DSSP  -------------------------------hhhHHHH------------------hHH


DSSP  HHHHHHHHHHHH-hLLLL------------------------------llllllLLHHHHH
Query IPWQIAQKLHAV-tAVLE------------------------------epkandRAHDLVD  210
ident                                                         |
Sbjct VRKRVQDLEQKMkvVENLqddfdfnyktlksqgdvtrqkmqqleqmltaldqmRRSIVSE   75
DSSP  HHHHHHHHHHHHhhHHHHhhhhhhhhhhhlllllllllllhhhhhhhhhhhhhHHHHHHH


DSSP  HHHHHHHL-------------------------------------lhHHHHHHHhhhhhh
Query LQLLEGLL-------------------------------------ldADLXPTRsaciai  233
ident |  |                                             |
Sbjct LAGLLSAMeyvqktltdeeladwkrrqqiaciggppnicldrlenwiTSLAESQ------  129
DSSP  HHHHHHHHhhhhhhhhhhhhhhhhhhhhhhhhhhhhlllllllllhhhhhhhHHHHHHH------


DSSP  hhhhllllllLLLLL--LLHHHHhhhhhhlllllllllLHHHHHHHHHHHHHHHHH-LL
Query fearaqhpwppRVATL--PHWPLiyagalegldhlelarTVDAAAQAVQRFVARIDR-AT  290
```

```
DSSP  LL----------------------------------------------------------
Query KR----------------------------------------------------------  292
ident |
Sbjct KSafvverqpcmpmhpdrplviktgvqfttkvrllvkfpelnyqlkikvcidkrgsrkfn  232
DSSP  HHleeeeeeeellllllllleeelllleeeeeeelllllllllleeeellllllllllee


DSSP  ------------------------------------------------------------
Query ------------------------------------------------------------  292
ident
Sbjct ilgtntkvmnmeengslsaefkhltlreqrcivteelhlitfetevyhqglkidlethsl  292
DSSP  eellleeeeeeelllleeeeeeeeeeeellllhhhleelleeeeeeellllleeeeeeell


DSSP  ------------------------------------------------------------
Query ------------------------------------------------------------  292
ident
Sbjct pvvvisnicqmpnawasilwynmltnnpknvnfftkppigtwdqvaevlswqfssttkrg  352
DSSP  leeeellllhhhhhhhhhhhhhhlllllllllllllllllhhhhhhhhhhhhhhhllll


DSSP  ------------------------------------------------------------
Query ------------------------------------------------------------  292
ident
Sbjct lsieqlttlaekllgpgvnysgcqitwakfckenmagkgfsfwvwldniidlvkkyilal  412
DSSP  llllllllllhhhhllllllllllllhhhhhllllllllllhhhhhhhhhhhhhhlllll


DSSP  ------------------------------------------------------------
Query ------------------------------------------------------------  292
ident
Sbjct wnegyimgfiskererailstkppgtfllrfsesskeggvtftwktqiqsvepytkqqln  472
DSSP  lllllllllllllhhhllllllllllllllllllllllllllllllllllllllllllll


DSSP  -----------------------------------
Query -----------------------------------  292
ident
Sbjct faeiimgyktnilvsplvylypdipkeeafgkycr  507
DSSP  lllllllllllllllllllllllllllllllllll
```

No 725: Query=mol1A Sbjct=3ebbA Z-score=2.4

back to top

```
DSSP  ------------------------------------------------------------
Query ------------------------------------------------------------    0
ident
Sbjct xniyfpkkeavtfdqanptqilgklkelngtapeekklteddlillekilslicnsssek   60
DSSP  lllllllllllllllllhhhhhhhhhhhhllllhhhlllhhhhhhhhhhhhhhhllllll


DSSP  ----LLLL---llLLLLHHhHHHHHHHHHHHLLL---------lhhhhhHHHHHHHHHH-
Query ----AAPY---ssPPTNLRsLRDRLTQVAERQGV---------vfgrlqRHVAXIVVAQ-   43
ident                         |                             |
Sbjct ptvqQLQIlwkaiNCPEDI-VFPALDILRLSIKHpsvnenfcnekegaqFSSHLINLLNp  119
DSSP  llhhHHHHhhhhhLLLLLL-LHHHHHHHHHHLLLhhhhhhhhllllhhhHHHHHHHLLLl


DSSP  -------------------hhhllllllllllEEEE----------------hHHHHhhh
Query -------------------faatltddtgaplLLVK----------------gGSSLelr   68
ident                                  |
Sbjct kgkpanqllalrtfcncfvgqagqklxxsqreSLXShaielksgsnknihialATLA---  176
DSSP  lllhhhhhhhhhhhhhhhhhlhhhhhhhhlhhHHHHhhhhhhllllhhhhhhhHHHH---


DSSP  hllllllllllleeeeelllhhhhhhhhhhllllleeeeellleellllllllllllleee
Query rgipdsrtskdfatvarrdieliheqladagetgwegftaiftapeeiaapgxpvkprrf  128
ident
Sbjct ------------------------------------------------------------  176
DSSP  ------------------------------------------------------------
```

```
ident                                                          |
Sbjct ---------------lnysvcfhkdhNIEG-------------------kaQCLSLISTI  202
DSSP  ---------------hhhhhhhhhllLHHH-------------------hhHHHHHHHHH


DSSP  HHHHhllllllllllLLHH-HHHHHHHHHHL--LHHHHHHHHHHHhhhhhhhllllLLLL
Query LHAVtavleepkandRAHD-LVDLQLLEGLL--LDADLXPTRSACiaifearaqhpWPPR  245
ident |  |                   |  |  |            |
Sbjct LEVV----------qDLEAtFRLLVALGTLIsdDSNAVQLAKSLG---------vdSQIK  243
DSSP  HLLL----------lLHHHhHHHHHHHHHHHllLHHHHHHHHHHLL---------hhHHHH


DSSP  --LLLLllhhhhhhhhhlllllllllLHHHHHHHHHHHHHHHhhllll
Query --VATLphwpliyagalegldhlelarTVDAAAQAVQRFVARIdratkr  292
ident
Sbjct kySSVS---------------------EPAKVSECCRFILNLL------  265
DSSP  hhHHLL---------------------LLHHHHHHHHHHHLLL------
```

No 726: Query=mol1A Sbjct=3nmwB Z-score=2.4

back to top

```
DSSP  lllllllllhhhhhhhhhhhhhhlllLHHHHHHHHHHHHHHH-hhhllLLLL-------
Query aapysspptnlrslrdrltqvaerqgvVFGRLQRHVAXIVVAQ-faatlTDDT-------   52
ident                                |
Sbjct ---------------------------HHHXLHLLEQIRAYCEtcwewqEAHEdknpxpa   33
DSSP  ---------------------------LHHHHHHHHHHHHHHHHhhhhhhHLLLlllllll


DSSP  -------------------------------lllleEEEH-------------------H
Query -------------------------------gapllLVKG-------------------G   62
ident
Sbjct pvehqicpavcvlxklsfdeehrhaxnelgglqaiaELLQvdcexygltndhysitlrrY   93
DSSP  hhhhlhhhhhhhhhhhlllhhhhhhhhhllhhhhhhHHHHhhhhhhlllllhhhhhhhhH


DSSP  HHHHHHHLllllllllleeeeelllhhhhhhhhhhhhhllllleeeeellleellllllll
Query SSLELRRGipdsrtskdfatvarrdielihеqladagetgwegftaiftapeeiaapgxp  122
ident     |
Sbjct AGXALTNL----------------------------------------------------  101
DSSP  HHHHHHHH----------------------------------------------------


DSSP  llleeeeeeeeelleeeeeeeeeeeelllllllllleeeeelhhhhllllllleeeeeLLHH
Query vkprrftaklsyrgrafatvpievssveagnadqfdtltsdalglvgvpaavavpcXTIP  182
ident
Sbjct ----------------------------------------tfgdvankatlcsxkGCXR  120
DSSP  ----------------------------------------llllhhhhhhhhhlhHHHH


DSSP  HHHhHHHHHhhlllllllllLLLHHHHHHHHHHHHL----lHHHHHHHHHHHhhhhhhhl
Query WQIaQKLHAvtavleepkanDRAHDLVDLQLLEGLL----lDADLXPTRSACiaifeara  238
ident       |                            |            |
Sbjct ALV-AQLKS-----------ESEDLQQVIASVLRNLswradVNSKKTLREVG-------s  161
DSSP  HHH-HHHHL-----------LLHHHHHHHHHHHHHHHhllllHHHHHHHHHHLL-------h


DSSP  lllllLLLLLlllhhhhhhhhhlllllllllLHHHHHHHHHHHHHHHHHHLLLL------
Query qhpwpPRVATlphwpliyagalegldhlelarTVDAAAQAVQRFVARIDRATKR------  292
ident                                         |
Sbjct vkalxECALE---------------------vKKESTLKSVLSALWNLSAHCTEnkadic  200
DSSP  hhhhhHHHHH---------------------lLLHHHHHHHHHHHHHHHHLLLHHhhhhhh


DSSP  ------------------------------------------------------------
Query ------------------------------------------------------------  292
ident
Sbjct avdgalaflvgtltyrsqtntlaiiesgggilrnvssliatnedhrqilrennclqtllq  260
DSSP  hlllhhhhhhhhlllllllllhhhhhhhhhhhhhhhhlllhhhhhhhhlllhhhhhhh


DSSP  ------------------------------------------------------------
Query ------------------------------------------------------------  292
ident
```

```
DSSP  -----------
Query -----------  292
ident
Sbjct nlxanrpakyk  331
DSSP  hhhllllhhhl
```

No 727: Query=mol1A Sbjct=2q0eA Z-score=2.3

back to top

```
DSSP  llllllllllhhhhhhhhhhhhHHLLLLHHHhhhhhhHHHHHHHHH-LLLLllLLLLEEE
Query aapysspptnlrslrdrltqvaERQGVVFGRlqrhvaXIVVAQFAA-TLTDdtGAPLLLV   59
ident                         | |                              |
Sbjct ---------pspavvgrslvnsFKQFVSKDL--htrhVDATYRLVLdCVAAvdPLMRLYT   49
DSSP  ---------llhhhhhhhhhhhLLLLLLLLL--lhhhHHHHHHHHHhHHHHhlLLLEEEE


DSSP  EHHHHHHhhhllllLLLL--LLEEEEEL-------------------lLHHHHHHHHHHH
Query KGGSSLElrrgipdSRTS--KDFATVAR-------------------rDIELIHEQLADA   98
ident  |                   |   |                          |   ||
Sbjct FGSTVVY-------GVHEkgSDVDFVVLnktdvedgkggdaatqvakgLQADILAKLARV  102
DSSP  ELHHHHH-------LLLLllLLEEEEEElhhhhhllllllllhhhhhHHHHHHHHHHHH


DSSP  HHHLLllEEEEELLEELlllllllLLEEEEEEEeelleEEEEEEEEEEELLllllllle
Query GETGWegFTAIFTAPEEIaapgxpvKPRRFTAKLsyrgrAFATVPIEVSSVEagnadqfd  158
ident                                 |        |
Sbjct IRQKH--LSWNVEEVRRT-------RVPVVRVKG-----GGAVDFDITAYRR--------  140
DSSP  HHHHL--LLLEEEEELLL-------LLLEEEEEL-----LLLLEEEEEELLL--------


DSSP  eeeelhhhhllllllleeeeellhHHHHHHHH--------------------hhhhllllL
Query tltsdalglvgvpaavavpcxtiPWQIAQKL--------------------havtavleE  198
ident
Sbjct --------------------ngvRNSALLRAyfeqnppcrwlsmsikrwskqtglnasvI  180
DSSP  --------------------hhhHHHHHHHHHhhhhlhhhhhhhhhhhhhhhhlllllllL


DSSP  LLLLL--LLHHhhhhhhhhhllhhhhhhhhhhhhhhhhhlllllllllllllhhhhh
Query PKAND--RAHDlvdlqllegllldadlxptrsaciaifearaqhpwpprvatlphwpliy  256
ident
Sbjct GGSITsyGFNL-------------------------------------------------  191
DSSP  LLLLLhhHHHH-------------------------------------------------


DSSP  hhhhllllllllllhHHHHhhhHHHH---------------------------------
Query agaleglddhlelartvDAAAqavQRFV---------------------------------  283
ident                        |
Sbjct --------mvvyyllqRNHL---QFVPpstidvsrveplpphlpleepadeglelgtqvl  240
DSSP  --------hhhhhhhhLLLL---LLLLhhhllhhhlllllllllllllllllhhhhhhhh


DSSP  ------------------------------------------------------------
Query ------------------------------------------------------------  283
ident
Sbjct dflhfflhefdsdkqvislnrpgittkeeldwtksaedfarmngekvhyqwciedpyeln  300
DSSP  hhhhhhhhllllleellllllllleehhhhlllhhhhhhleelleellllllllllllll


DSSP  ----------------------hhhhhllll
Query ----------------------aridratkr  292
ident
Sbjct lnvgrnvtplkrdflrrhlekardtalltiv  331
DSSP  lllllllllhhhhhhhhhhhhhhhhlllllll
```

No 728: Query=mol1A Sbjct=2i4gA Z-score=2.3

back to top

```
DSSP  llllllllllhhhhhhhhhhhhhhhllllhhhhhhhhhhhhhhhhhhlllllLLLL-----
```

```
DSSP  lllllleehhhhhhhhhhhhhhhhhhhhhhhhhllllllllllllllhhhhHHLLlllll
```

```
DSSP  ---------leeeehhhhhHHHH-llLLLL----------------------llLLEEEE
Query ---------lllvkggsslELRR-giPDSR----------------------tsKDFATV   83
ident                                                            |
Sbjct pydatrvklsnvcsdyinaSYIPgnnFRREyivtqgplpgtkddfwkmvweqnvHNIVMV  120
DSSP  llhhheeelllllleeeeEEELlllLLLLeeeelllllllhhhhhhhhhlllLEEEEL
```

```
DSSP  ELL---lhhhhhHHHHHH-hHHLLLLEEEEELLLEELLllllllLLEEEEEEEEEL--le
Query ARR---dielihEQLADA-gETGWEGFTAIFTAPEEIAapgxpvKPRRFTAKLSYR--gr  137
ident                                                    |
Sbjct TQCvekgrvkcdHYWPADqdSLYYGDLILQMLSESVLP------EWTIREFKICGEeqld  174
DSSP  LLLeelleellLLLLLLllLEEELLEEEEEEEEEEELL------LEEEEEEEEELLllll
```

```
DSSP  EEEEEEEEEEE-LLLLL---------llllleeeeelhhhhllllllleeeEELLhHHHHHH
Query AFATVPIEVSS-VEAGN---------adqfdtltsdalglvgvpaavavPCXTiPWQIAQ  187
ident |
Sbjct AHRLIRHFHYTvWPDHGvpettqsliqfvrtvrdyinrspgagptvvhcSAGV-GRTGTF  233
DSSP  LLEEEEEEEELlLLLLLllllhhhhhhhhhhhhhhhhlllllleeeelLLLL-HHHHHH
```

```
DSSP  HHHHHHLLllllllllllhhHHHHHHHHHHLlhhhhhhHHHHHHHhhhhhllllLLLLll
Query KLHAVTAVleepkandrahdLVDLQLLEGLLldadlxpTRSACIAifearaqhpWPPRva  247
ident                           |           |
Sbjct IALDRILQ--qldskdsvdiYGAVHDLRLHR--vhmvqTECQYVY--------lHQCV--  279
DSSP  HHHHHHHH--hhhhlleelhHHHHHHHHHLLL--lllllLHHHHHH--------hHHHH--
```

```
DSSP  llllhhhhhhhhhllllllllllhhhhhhhhhhhhhhhhhllll
Query tlphwpliyagalegldhlelartvdaaaqavqrfvaridratkr  292
ident
Sbjct -----------------------------------rdvlrarklr  289
DSSP  -----------------------------------hhhhhhhhhl
```

No 729: Query=mol1A Sbjct=2p25A Z-score=2.3

back to top

```
DSSP  lllllllllhhhhhhhhhhhhhhlLLLHhhHHHHhhhhhhhhhhhlllllllllleeee
Query aapysspptnlrslrdrltqvaerqGVVFgrLQRHvaxivvaqfaatltddtgapllllvk   60
ident                                   |
Sbjct -------------------------XFFK--EIHH-------------------------    8
DSSP  -------------------------LLLL--LLLL-------------------------
```

```
DSSP  hhhhhhhhlllllllllLEEEEelllhhhHHHHHHHH-HHHLllleEEEELLLEELLLl
Query ggsslelrrgipdsrtskDFATVarrdielIHEQLADA-GETGwegfTAIFTAPEEIAAp  119
ident                                         |
Sbjct -----------------vAINAS-------NYQATKNFyVEKL----GFEVLRENHRPE-   39
DSSP  -----------------eEEEEL-------LHHHHHHHhLLLL----LLEEEEEEEEHH-
```

```
DSSP  lllLLLEEEEEEEEeelleEEEEEEEEEEEL-lllllllLEEEeelhhhhLLLL-------
Query gxpVKPRRFTAKLsyrgrAFATVPIEVSSV-eagnadqFDTLtsdalglVGVP-------  171
ident            ||           |  |                     |
Sbjct ---KNDIKLDLKL-----GSQELEIFISDQfparpsypEALGlrhlafkVEHIeeviafl   91
DSSP  ---HLEEEEEEEE-----LLEEEEEEELLLllllllllLLLLllleeeeLLLHhhhhhhh
```

```
DSSP  -------------------LLEEEeellhhhhhhhhhhhhhlllllllllllhhhhhhh
Query -------------------AAVAVpcxtipwqiaqklhavtavleepkandrahdlvdlq  212
ident
Sbjct neqgieteplrvddftgkkXTFFF------------------------------------  115
DSSP  hhllllllleellllllleEEEEE------------------------------------
```

```
DSSP  hhhhllhhhhhhhhhhhhhhhhhhlllllllllllllhhhhhhhhhlllllllllh
Query lleglllldadlxptrsaciaifearaqhpwpprvatlphwpliyagalegldhlelartv  272
ident
Sbjct ------------------------------------------------------------  115
DSSP  ------------------------------------------------------------
```

```
Query daaaqavqrfvaridratkr  292
ident
Sbjct ---------dpdglplelhe  126
DSSP  ---------llllleeeeel
```

No 730: Query=mol1A Sbjct=4ol0B Z-score=2.3

back to top

```
DSSP  llllllllllhhhhhhhhhhhhhhlllhhhhhhhhhhhhhhhhhhlllllllllllEEEE
Query aapysspptnlrslrdrltqvaerqgvvfgrlqrhvaxivvaqfaatltddtgaplLLVK   60
ident
Sbjct --------------------------------------------------------AKPT    4
DSSP  --------------------------------------------------------LLLL


DSSP  HHHH-------------hhhhhLLLLLllllleeeeelllhhhhhhhhhhhhhhlllleee
Query GGSS-------------lelrrGIPDSrtskdfatvarrdieliheqladagetgwegft  107
ident
Sbjct LQLVyqavqalyhdpdpsgkerASFWL---------------------------------   31
DSSP  HHHHhhhhhhhhhhhlllhhhhHHHHH---------------------------------


DSSP  eeellleellllllllleeeeeeeeelleeeeeeeeeeeeelllllllllleeeeelhhhh
Query aiftapeeiaapgxpvkprrftaklsyrgrafatvpievssveagnadqfdtltsdalgl  167
ident
Sbjct ------------------------------------------------------------   31
DSSP  ------------------------------------------------------------


DSSP  llllleeeeelLHHHHHHHHHHHHhllllllllllLLLHHHHHHHHHHHHL---LHHHhh
Query vgvpaavavpcxTIPWQIAQKLHAVtavleepkanDRAHDLVDLQLLEGLL---LDADlx  224
ident                    | |
Sbjct -----gelqrsvHAWEISDQLLQIR----------QDVESCYFAAQTMKMKiqtSFYE--   74
DSSP  -----hhhllllLHHHHHHHHHHLL----------LLHHHHHHHHHHHHHHhhhLLLL--


DSSP  hhhhhhhhhhhhhllllLLLL----LLLLllhhhhhhhhhlllllllllllHHHHHHHHH
Query ptrsaciaifearaqhpWPPR----VATLphwpliyagalegldhlelartVDAAAQAVQ  280
ident
Sbjct -------lptdshaslrDSLLthiqNLKD---------------------lSPVIVTQLA  106
DSSP  -------llhhhhhhhhHHHHhhhhHLLL---------------------lLHHHHHHHH


DSSP  HHHHHHHHLLLL------------------------------------------------
Query RFVARIDRATKR------------------------------------------------  292
ident    |
Sbjct LAIADLALQMPSwkgcvqtlvekysndvtslpflleiltvlpeevhsrslriganrrtei  166
DSSP  HHHHHHHHLLLLlllhhhhhhhhhlllhhhhhhhhhhhhhlllllllllllllhhhhhhh


DSSP  ------------------------------------------------------------
Query ------------------------------------------------------------  292
ident
Sbjct iedlafysstvvsllmtcvekagtdekmlmkvfrclgswfnlgvldnnkllallfevlqq  226
DSSP  hhhhhhhhhhhhhhhhhhllllllhhhhhhhhhhhhhhllhhhlllllhhhhhhhhhhhl


DSSP  ------------------------------------------------------------
Query ------------------------------------------------------------  292
ident
Sbjct dktssnlheaasdcvcsalyaienvetnlplamqlfqgvltletayhmavaredldkvln  286
DSSP  llllhhhhhhhhhhhhhhhhhhhhhhhllllhhhhhhhhhhllhhhhhhhhhlllhhhhh


DSSP  ------------------------------------------------------------
Query ------------------------------------------------------------  292
ident
Sbjct ycriftelcetflekivctpgqglgdlrtlelllicaghpqyevveisfnfwyrlgehly  346
DSSP  hhhhhhhhhhhlhhhhhhllllhhhllhhhhhhhhhhllllhhhhhllhhhhhhhhhhhh


DSSP  ------------------------------------------------------------
Query ------------------------------------------------------------  292
```

```
DSSP  ------------------------------------------------------------
Query ------------------------------------------------------------  292
ident
Sbjct gsmecfaqlystlkegnppwevteavlfimaaiaksvdpennptlvevlegvvrlpetvh  466
DSSP  lhhhhhhhhhhhhhlllllhhhhhhhhhhhhllhhhllhhhhhhhhhhhhhhhlllllll


DSSP  ------------------------------------------------------------
Query ------------------------------------------------------------  292
ident
Sbjct tavrytsielvgemsevvdrnpqfldpvlgylmkglcekplasaaakaihnicsvcrdhm  526
DSSP  hhhhhhhhhhhhllhhhhhhlhhhhhhhhhhhhhhhhlhhhhhhhhhhhhhhhhhlllhh


DSSP  ------------------------------------------------------------
Query ------------------------------------------------------------  292
ident
Sbjct aqhfnglleiarsldsfllspeaavgllkgtalvlarlpldkiteclselcsvqvmalkk  586
DSSP  hllhhhhhhhhhllllllllhhhhhhhhhhhhhhhllllhhhhhhhhhhhhhhhhhhhhh


DSSP  ------------------------------------------------------------
Query ------------------------------------------------------------  292
ident
Sbjct llssdptvfldrlavifrhtnpivengqthpcqkviqeiwpvlsetlnkhradnriverc  646
DSSP  llllllhhhhhhhhhhhhllllllllllllhhhhlhhhhhhhhhhhhhhhllllhhhhhhh


DSSP  ------------------------------------------------------------
Query ------------------------------------------------------------  292
ident
Sbjct crclrfavrcvgkgsaallqplvtqmvnvyhvhqhscflylgsilvdeygmeegcrqgll  706
DSSP  hhhhhhhhhhhllllhhhhhhhhhhhhhhhhllllllhhhhhhhhhhhhhhlllllhhhhh


DSSP  ------------------------------------------------------------
Query ------------------------------------------------------------  292
ident
Sbjct dmlqalciptfqlleqqnglqnhpdtvddlfrlatrfiqrspvtllrsqvvipilqwaia  766
DSSP  hhhhhhhhhhhhhhlllllllllhhhhhhhhhhhhhhhhhlhhhhhllllhhhhhhhhhh


DSSP  ------------------------------------------------------------
Query ------------------------------------------------------------  292
ident
Sbjct sttldhrdancsvmrflrdlihtgvandheedfelrkeligqvmnqlgqqlvsqllhtcc  826
DSSP  hlllllhhhhhhhhhhhhhhhhhlllllllllhhhhhhhhhhhhhllhhhhhhhhhhhhh


DSSP  ------------------------------------------------------------
Query ------------------------------------------------------------  292
ident
Sbjct fclppytlpdvaevlweimqvdrptfcrwlenslkglpkettvthkqltdfhkqvtsaee  886
DSSP  hlllhhhhhhhhhhhhhhhhhhlhhhhhhhhhhhhhllllllllllllhhhhhhhhhllll


DSSP  ----------------
Query ----------------  292
ident
Sbjct ckqvcwalrdftrlfr  902
DSSP  hhhhhhhhhhhhhhll
```

No 731: Query=mol1A Sbjct=2kjzA Z-score=2.3

back to top

```
DSSP  llllllllllhhhhhhhhhhhhhhllllhhhhhhhhhhhhhhhhhhhllllllllllleeee
Query aapyssppptnlrslrdrltqvaerqgvvfgrlqrhvaxivvaqfaatltddtgaplllvk   60
ident
Sbjct ------------------------------------------------------------    0
DSSP  ------------------------------------------------------------
```

```
ident
Sbjct -------------MTHPDFTILYVD-NPPASTQFYKALL--------GVDPVESS-----   33
DSSP  -------------LLLLLEEEEEEL-LHHHHHHHHHHHH--------LLLLLEEE-----


DSSP  llLLLEEEEEEeeelleeeEEEEEEEEELL-----LLLLLLleeeeelhhhhLLLLLL--
Query xpVKPRRFTAKlsyrgrafATVPIEVSSVE-----AGNADQfdtltsdalglVGVPAA--  173
ident        |                   |       |
Sbjct --PTFSLFVLA--------NGMKLGLWSRHtvepkASVTGG---ggelafrvENDAQVde   80
DSSP  --LLEEEEELL--------LLLEEEEEELLlllllLLLLLL---lleeeeelLLHHHHhh


DSSP  ------------------------EEEEellhhhhhhhhhhhhhllllllllllllhhhh
Query ------------------------VAVPcxtipwqiaqklhavtavleepkandrahdlv  209
ident
Sbjct tfagwkasgvamlqqpakmefgytFTAA--------------------------------  108
DSSP  hhhhhhhllllllllleeelleeeEEEL--------------------------------


DSSP  hhhhhhhhllhhhhhhhhhhhhhhhhhhllllllllllllllhhhhhhhhhlllllllll
Query dlqllegllldadlxptrsaciaifearaqhpwpprvatlphwpliyagalegldhlela  269
ident
Sbjct ------------------------------------------------------------  108
DSSP  ------------------------------------------------------------


DSSP  llhhhhhhhhhhhhhhhhhllll
Query rtvdaaaqavqrfvaridratkr  292
ident
Sbjct ---------dpdshrlrvyafag  122
DSSP  ---------llllleeeeeeell
```

No 732: Query=mol1A Sbjct=2j5cB Z-score=2.3

[back to top]

```
DSSP  llllllllllhhhhhhhhhhhhhhllllhhhhhhhhhhhhhhhhhlllllllLLLEE--
Query aapysspptnlrslrdrltqvaerqgvvfgrlqrhvaxivvaqfaatltddtgAPLLL--   58
ident
Sbjct ---------------------------------------raagmidqvkmmlqEEVDSir   21
DSSP  ---------------------------------------lhhhhhhhhhhhhhHLLLHhh


DSSP  ----------eehhhhhhhhhlllllllllLEEEeelllhhhhhhhhhhhhhhlllleee
Query ----------vkggsslelrrgipdsrtskDFATvarrdieliheqladagetgwegfta  108
ident
Sbjct rleliddlrrlgischfereiveilnskyyTNNE--------------------------   55
DSSP  hhhhhhhhhhllhhhhhhhhhhhhhhhhllLLLL--------------------------


DSSP  eellleellllllllllleeeeeeeeelleeeeeeeeeeeelllllllllleeeeelhhhhl
Query iftapeeiaapgxpvkprrftaklsyrgrafatvpievssveagnadqfdtltsdalglv  168
ident
Sbjct ------------------------------------------------------------   55
DSSP  ------------------------------------------------------------


DSSP  llllleeeeellhhhHHHHHHHHHHLllllllLLLLLhhHHHHHHHHH---hllhhHHHH
Query gvpaavavpcxtipwQIAQKLHAVTAvleepkANDRAhdLVDLQLLEG---llldaDLXP  225
ident                                                          |
Sbjct -----------iderDLYSTALRFRL--lrqyDFSVS--QEVFDCFKNakgtdfkpSLVD  100
DSSP  -----------llllLHHHHHHHHHH--hhhlLLLLL--HHHHHHHLLllllllllhHHHH


DSSP  hHHHHhhhhhhhllllllLLLLLlllhhhhhhhhhllllllllllLHHHHHHHHHHHHHH
Query tRSACiaifearaqhpwpPRVATlphwpliyagalegldhlelarTVDAAAQAVQRFVAR  285
ident                                                         |
Sbjct -DTRG--------llqlyEASFL--------------------saQGEETLRLARDFATK  131
DSSP  -LHHH--------hhhhhHHHHH--------------------llLLLHHHHHHHHHHHH


DSSP  HHHLLLL-----------------------------------------------------
Query IDRATKR-----------------------------------------------------  292
ident
```

```
DSSP  ------------------------------------------------------------
Query ------------------------------------------------------------  292
ident 
Sbjct vqaqfqqelkeasrwwnstglvhelpfvrdrivecyywttgvverrqhgyerimltkina  251
DSSP  hhhhhhhhhhhhhhhhhhhhlhhhhllllllhhhhhhhhhhhllllllhhhhhhhhhhhh


DSSP  ------------------------------------------------------------
Query ------------------------------------------------------------  292
ident 
Sbjct lvttiddvfdiygtleelqlfttaiqrwdiesmkqlppymqicylalfnfvnemaydtlr  311
DSSP  hhhhhhhhhhllllhhhhhhhhhhhhhlllhhhhhllhhhhhhhhhhhhhhhhhhhhhhh


DSSP  ------------------------------------------------------------
Query ------------------------------------------------------------  292
ident 
Sbjct dkgfdstpylrkvwvgliesylieakwyykghkpsleeymknswisiggipilshlffrl  371
DSSP  hhllllhhhhhhhhhhhhhhhhhhhhhhhhllllhhhhhhhhhhhhlllhhhhhhhhhhh


DSSP  ------------------------------------------------------------
Query ------------------------------------------------------------  292
ident 
Sbjct tdsieeeaaesmhkyhdivrasctilrladdmgvpksvqcymneknaseeearehvrsli  431
DSSP  lllllhhhhhhhllllhhhhhhhhhhhhhhhlllllhhhhhhhhllllhhhhhhhhhhhh


DSSP  ------------------------------------------------------------
Query ------------------------------------------------------------  292
ident 
Sbjct dqtwkmmnkemmtssfskyfvevsanlarmaqwiyqhesdgfqhslvnkmlrdllfhrye  491
DSSP  hhhhhhhhhhhhhllllhhhhhhhhhhhhhhhhhlhhhlllllllhhhhhhhhhhlllll
```

No 733: Query=mol1A Sbjct=3a6pA Z-score=2.3

back to top

```
DSSP  llllllllllhhhhhhhhhhhhhhllllhhhhhhhhhhhhhhhhhhhlllllllllleeee
Query aapysspptnlrslrdrltqvaerqgvvfgrlqrhvaxivvaqfaatltddtgaplllvk   60
ident 
Sbjct ------------------------------------------------------------    0
DSSP  ------------------------------------------------------------


DSSP  hhhhhhhhhlllllllllleeeeelllhhhhhhhhhhhhhlllleeeeeellleelllll
Query ggsslelrrgipdsrtskdfatvarrdieliheqladagetgwegftaiftapeeiaapg  120
ident 
Sbjct ------------------------------------------------------------    0
DSSP  ------------------------------------------------------------


DSSP  llllleeeeeeeeelleeeeeeeeeeeellllllllleeeeeelhhhhlllllleeeeeLL
Query xpvkprrftaklsyrgrafatvpievssveagnadqfdtltsdalglvgvpaavavpcXT  180
ident 
Sbjct -----------------------------------------------------amdqvNA    7
DSSP  -----------------------------------------------------llllhHH


DSSP  HHHHHHHHHHHHHLLLllllLLLL-LHHHHHHHHHHHHLLhhHHHHHHHHHHHhhHHHLl
Query IPWQIAQKLHAVTAVLeepkANDR-AHDLVDLQLLEGLLLdaDLXPTRSACIAifEARAq  239
ident    |                        |                |            |
Sbjct LCEQLVKAVTVMMDPN---sTQRYrLEALKFCEEFKEKCP--ICVPCGLRLAE-kTQVA-   60
DSSP  HHHHHHHHHHHHHLLL---lLHHHhHHHHHHHHHHHHHLL--LHHHHHHHHLL-lLLLH-


DSSP  lllllllllllhhhhhhhhhhlllllllllllhhhhhhhhhhhhHHHHH-----------
Query hpwpprvatlphwpliyagalegldhlelartvdaaaqavqrfvARIDR-----------  288
ident                                               |
Sbjct --------------------------------------ivrhfgLQILEhvvkfrwngms   82
DSSP  --------------------------------------hhhhhhHHHHHhhhhhlhhhll
```

```
Sbjct rlekvylknsvmeliangtlnileeenhikdalsrivvemikrewpqhwpdmlieldtls  142
DSSP  hhhhhhhhhhhhhhhhhlllllllllhhhhhhhhhhhhhhhhhhlllllllhhhhhhhhh


DSSP  ------------------------------------------------------------
Query ------------------------------------------------------------  288
ident
Sbjct kqgetqtelvmfillrlaedvvtfqtlppqrrrdiqqtltqnmerifsfllntlqenvnk  202
DSSP  hllhhhhhhhhhhhhhhhhhhhhhhhhllllllhhhhhhhhlhhhhhhhhhhhhhhhhhh


DSSP  ------------------------------------------------------------
Query ------------------------------------------------------------  288
ident
Sbjct yqqvktdtsqeskaqancrvgvaalntlagyidwvsmshitaencklleilclllneqel  262
DSSP  hhhhllllllhhhhhhhhhhhhhhhhhhhlllllllhhhhhllllhhhhhhhhhhhllll


DSSP  ------------------------------------------------------------
Query ------------------------------------------------------------  288
ident
Sbjct qlgaaeclliavsrkgkledrkplmvlfgdvamhyilsaaqtadggglvekhyvflkrlc  322
DSSP  hhhhhhhhhhhhlllllhhhhhhhhhhhlhhhhhhhhhhhllllllllhhhhhhhhhhhh


DSSP  ------------------------------------------------------------
Query ------------------------------------------------------------  288
ident
Sbjct qvlcalgnqlcallgadsdvetpsnfgkylesflaftthpsqflrsstqmtwgalfrhei  382
DSSP  hhhhhhhhhhhhhhhlllllllllhhhhhhhhhhhhllllhhhhhhhhhhhhhhhhllll


DSSP  ------------------------------------------------------------
Query ------------------------------------------------------------  288
ident
Sbjct lsrdplllaiipkylrasmtnlvkmgfpsktdspsceysrfdfdsdedfnaffnssraqq  442
DSSP  llllhhhhhhhhhhhhhhhhhhllllllllllllhhhhhhhlllhhhhhhhhhhhhhhhhh


DSSP  ------------------------------------------------------------
Query ------------------------------------------------------------  288
ident
Sbjct gevmrlacrldpktsfqmagewlkyqlstfslcsvfspsfvqweamtlflesvitqmfrt  502
DSSP  hhhhhhhhhlhhhhhhhhhhhhhhhhhlllllllllhhhhhhhhhhhhhhhhhhhhhhhh


DSSP  ------------------------------------------------------------
Query ------------------------------------------------------------  288
ident
Sbjct lnreeipvndgiellqmvlnfdtkdplilscvltnvsalfpfvtyrpeflpqvfsklfss  562
DSSP  lllllllhhhhhhhhhhhhlllllhhhhhhhhhhhhhhhhhhhllhhhhhhhhhhhhhhh


DSSP  ------------------------------------------------------------
Query ------------------------------------------------------------  288
ident
Sbjct vtfetveeskaprtravrnvrrhacssiikmcrdypqlvlpnfdmlynhvkqllsnelll  622
DSSP  hhllllllllllllhhhhhhhhhhhhhhhhhhhlhhhhhhhhhhhhhhhhhhhhhlllll


DSSP  ------------------------------------------------------------
Query ------------------------------------------------------------  288
ident
Sbjct tqmekcalmealvlisnqfknyerqkvfleelmapvasiwlsqdmhrvlsdvdafiayvg  682
DSSP  lhhhhhhhhhhhhhhhhhhllhhhhhhhhhhhhhhhhhhhllhhhhhhhhlhhhhhhhhl


DSSP  ------------------------------------------------------------
Query ------------------------------------------------------------  288
ident
Sbjct tdqkdpgledpcglnrarmsfcvysilgvvkrtcwptdleeakaggfvvgytssgnpifr  742
DSSP  llllllllllhhhhhhhhhhhhhhhhhhhhhhllllllhhhhhhllleeeellllleeel


DSSP  ------------------------------------------------------------
Query ------------------------------------------------------------  288
ident
```

```
DSSP  ------------------------------------------------------------
Query ------------------------------------------------------------  288
ident
Sbjct lndspvfktvlermqrffstlyencfhilgkagpsmqqdfytvedlatqllssafvnlnn  862
DSSP  lllllllllhhhhhhhhhhhhhhhhhhhhhhhhhhhhlllllllllhhhhhhhhlllllll


DSSP  ------------------------------------------------------------
Query ------------------------------------------------------------  288
ident
Sbjct ipdyrlrpmlrvfvkplvlfcppehyealvspilgplftylhmrlsqkwqvinqresqem  922
DSSP  llhhhhhhhhhhlhhhhhhlllhhhllllhhhhhhhhhhhhhhhhhhhhhhhhhlllhhhh


DSSP  ------------------------------------------------------------
Query ------------------------------------------------------------  288
ident
Sbjct leeqlvrmltrevmdlitvccvseltdlgkclmkhedvctallitafnslawkdtlscqr  982
DSSP  hhhhhhhhhhhhhhhhhhhhhhllllhhhhhhhllhhhhhhhhhhhhhhlllllhhhhhh


DSSP  ------------------------------------------------------------
Query ------------------------------------------------------------  288
ident
Sbjct ttsqlcwpllkqvlsgtlladavtwlftsvlkglqmhgqhdgcmaslvhlafqiyealrp 1042
DSSP  hhhhlhhhhhllllllllhhhhhhhhhhhhhhhhhhllllhhhhhhhhhhhhhhhhhlll


DSSP  --------------------------llll
Query --------------------------atkr  292
ident
Sbjct ryleiravmeqipeiqkdsldqfdckllnp 1072
DSSP  llllhhhhhhllllllllhhhhhhhhhhllll
```

No 734: Query=mol1A Sbjct=1xm9A Z-score=2.3

back to top

```
DSSP  lllllllllhhhhhhhhhhhhhhlllhhhhhhhhhhhhhhhhhhhhllllllllleeee
Query aapyssppptnlrslrdrltqvaerqgvvfgrlqrhvaxivvaqfaatltddtgaplllvk   60
ident
Sbjct ------------------------------------------------------------    0
DSSP  ------------------------------------------------------------


DSSP  hhhhhhhhhlllllllllleeeeeelllhhhhhhhhhhhhhhllllleeeeellleellll
Query ggsslelrrgipdsrtskdfatvarrdielihe qladagetgwegftaiftapeeiaapg  120
ident
Sbjct ------------------------------------------------------------    0
DSSP  ------------------------------------------------------------


DSSP  llllleeeeeeeeelleeeeeeeeeeeelllllllllleeeeelhhhhllllllleeeeELL
Query xpvkprrftaklsyrgrafatvpievssveagnadqfdtltsdalglvgvpaavavpCXT  180
ident                                                            |
Sbjct ---------------------------------------------------------GLT    3
DSSP  ---------------------------------------------------------LLL


DSSP  HhHHHHHHHhhhhlllllllllLLLHHHHHHhhhhhhllHHHHhHHHHHHHHHHHhhhlll
Query IpWQIAQKLhavtavleepkanDRAHDLVDLqlleglllDADLxPTRSACIAIFearaqh  240
ident |     | |                                |
Sbjct I-PKAVQYL-----------ssQDEKYQAIGayyiqhtcFQDE-SAKQQVYQLG------   44
DSSP  H-HHHHHHH-----------hlLLLHHHHHHhhhhhhhlLLLL-LHHHHHHHLL------


DSSP  lLLLL---LLLLllhhhhhhhhhllllllllllHHHHHHHHHHHHHHHHHHLLL------
Query pWPPR---VATLphwpliyagalegldhlelartVDAAAQAVQRFVARIDRATK------  291
ident                                        ||
Sbjct gICKLvdlLRSP----------------------NQNVQQAAAGALRNLVFRSTtnklet   82
DSSP  hHHHHhhhLLLL----------------------LHHHHHHHHHHHHHHHHLLLHhhhhh
```

```
Sbjct rrqngireavsllrrtgnaeiqkqltgllwnlsstdelkeeliadalpvladrviipfsg  142
DSSP  hhlllhhhhhhhhllllhhhhhhhhhhhhhhhlllllhhhhhhhhhhhhhhhhllhhhhl


DSSP  ------------------------------------------------------------
Query ------------------------------------------------------------  291
ident
Sbjct wcvvdpevffnatgclrnlssadagrqtmrnysglidslmayvqncvaasrcddksvenc  202
DSSP  llllhhhhhhhhhhhhhhlllhhhhhhhlllllhhhhhhhhhhhhhhhlllllllhhhh


DSSP  ------------------------------------------------------------
Query ------------------------------------------------------------  291
ident
Sbjct mcvlhnlsyrldaevptryrqleynalpeeetnpkgsgwlyhsdairtylnlmgkskkda  262
DSSP  hhhhhhlllhhhhlllhhhhhhhllllllllllhhhhhhlhhhhhhhhhhhhhhlllhh


DSSP  ------------------------------------------------------------
Query ------------------------------------------------------------  291
ident
Sbjct tleacagalqnltaskglmssgmsqliglkekglpqiarllqsgnsdvvrsgasllsnms  322
DSSP  hhhhhhhhhhhlllllhhhhhhhhhhhlllllhhhhhhhlllllhhhhhhhhhhhhhhh


DSSP  ------------------------------------------------------------
Query ------------------------------------------------------------  291
ident
Sbjct rhpllhrvmgnqvfpevtrlltshtgntsnsedilssacytvrnlmasqpqlakqyfsss  382
DSSP  llhhhhhhhhhhlhhhhhhllllllllllhhhhhhhhhhhhhhhhhllllhhhhhhllhh


DSSP  -------------------------------------l
Query -------------------------------------r  292
ident
Sbjct mlnniinlcrssaspkaaeaarlllsdmwsskelqgvl  420
DSSP  hhhhhhhhhllllhhhhhhhhhhhhlllllllllllll
```

No 735: Query=mol1A Sbjct=4yc7B Z-score=2.2

back to top

```
DSSP  lllLLLL----------------------llLHHHHHHHHhhhhhhllllhhhhhhhhhh
Query aapYSSP----------------------ptNLRSLRDRLtqvaerqgvvfgrlqrhvax   38
ident
Sbjct eerFAIVlnamnlppdkarllrqydnekkweLICDQERFQ----------------vknp   44
DSSP  lllLLLLlhhhlllhhhhhhhhlllhhhhhhHHHHHHHLL----------------llll


DSSP  HHHHHHHHLL--LLLL--------------------------------------llllEE
Query IVVAQFAATL--TDDT--------------------------------------gaplLL   58
ident
Sbjct PHTYIQKLKGylDPAVtrkkfrrrvqestqvlreleislrtnhigwvreflneenkglDV  104
DSSP  HHHHHHHHHHhhLLLLlhhhhhhhhhhhhhhhhhhhhhhhhhhhllhhhhhhhhlllllhhHH


DSSP  EE--------------------------------HHHHHHHHHLLLllllllleeeeell
Query VK--------------------------------GGSSLELRRGIPdsrtskdfatvarr   86
ident
Sbjct LVeylsfaqyavtfsrrtlknsrlvskkddvhvcIMCLRAIMNYQY--------------  150
DSSP  HHhhhhhhhlllllhhhhhhhhhhhlllhhhhhHHHHHHHHHLLHH--------------


DSSP  lhhhhhhhhhhhhhhhlllleeeeellleelllllllllleeeeeeeeelleeeeeeeeee
Query dieliheqladagetgwegftaiftapeeiaapgxpvkprrftaklsyrgrafatvpiev  146
ident
Sbjct ------------------------------------------------------------  150
DSSP  ------------------------------------------------------------


DSSP  eellllllllllleeeeelhhhhllllleeeeeLLHHHHHhHHHHHhhlllllllllLLLH
Query ssveagnadqfdtltsdalglvgvpaavavpcXTIPWQIaQKLHAvtavleepkanDRAH  206
ident                                           |
Sbjct -----------------------gfnmvmshpHAVNEIA-LSLNN-----------KNPR  175
```

```
DSSP  -HHHHHHHHHHHL----LHHHHHHHHHHHHHHHHHhhllLLLLL---LLLL-LLLHHhhhh
Query -DLVDLQLLEGLL----LDADLXPTRSACIAIFEaraqHPWPP---RVAT-LPHWPliya  257
ident      | ||
Sbjct tKALVLELLAAVClvrgGHEIILSAFDNFKEVCG---eKQRFEklmEHFRnEDNNI----  228
DSSP  hHHHHHHHHHHHHhlllHHHHHHHHHHHHHHHHHL---lLLLLHhhhHHHHhLLLLH----


DSSP  hhhllllllllllLHHHHHHHHHHHHHH--------------------------------
Query galegldhlelarTVDAAAQAVQRFVAR--------------------------------  285
ident                  |       |
Sbjct -------------DFMVASMQFINIVVHsvedmnfrvhlqyeftklgldeyldklkhtes  275
DSSP  -------------HHHHHHHHHHHHHHHllllhhhhhhhhhhhhhllhhhhhhhhlllll


DSSP  ---------hhhllll
Query ---------idratkr  292
ident
Sbjct dklqvqiqayldnvfd  291
DSSP  hhhhhhhhhhhhhlll
```

No 736: Query=mol1A Sbjct=1z1wA Z-score=2.2

back to top

```
DSSP  ------------------------------------------------------------
Query ------------------------------------------------------------    0
ident
Sbjct mevekydltldfdiqkrtfngtetitadagdivldavglqinwmkvngrdtaftydgqtv   60
DSSP  lleeeeeeeeeellllleeeeeeeeeelleeeeeellllleeeeeeelllleelleellllee


DSSP  ------------------------------------------------------------
Query ------------------------------------------------------------    0
ident
Sbjct rapgdsqpqkieisfagkvsdslsgiyyagrengmitthfeatdarrmfpcvdhpaykav  120
DSSP  eeeeellllleeeeeeeeelllllleeeeellllleeeeellllhhhlllllllllllle


DSSP  ------------------------------------------------------------
Query ------------------------------------------------------------    0
ident
Sbjct faitvvidkdydaisnmppkrievserkvvefqdtprmstyllyvgigkfryeyekyrdi  180
DSSP  eeeeeeellllleeeellleeeeeelllleeeeellllllhhhlleeeelleeeeeeelle


DSSP  ------------------------------------------------------------
Query ------------------------------------------------------------    0
ident
Sbjct dlilaslkdirskypldmarksvefyenyfgipyalpkmhlisvpefgagamenwgaitf  240
DSSP  eeeeeelllllllhhhhhhhhhhhhhhhhhhlllllllleeeeeeellllllleellllleeee


DSSP  ------------------------------------------------------------
Query ------------------------------------------------------------    0
ident
Sbjct reiymdiaensavtvkrnsanviaheiahqwfgdlvtmkwwndlwlnesfatfmsyktmd  300
DSSP  ehhhhllllllllhhhhhhhhhhhhhhhhhhllllllleelllllllhhhhhhhhhhhhhhhh


DSSP  ------------------------------------------------------------
Query ------------------------------------------------------------    0
ident
Sbjct tlfpewsfwgdffvsrtsgalrsdslknthpievdvrdpdeisqifdeisygkgasilrm  360
DSSP  hhlhhhlhhhhhhhhlhhhhhhhllllllllllllllllllllllllllllhhhhhhhhh


DSSP  --------------------------------llllllllLLHHHHHHHHHHhhhhllll
Query --------------------------------aapysspppTNLRSLRDRLTQvaerqgvv   28
ident
Sbjct iedyagyeefrkgiskylndhkfgnaegsdlwtaiedvsgKPVKRVMEYWIK--------  412
DSSP  hhhhhlhhhhhhhhhhhhhhhllleellhhhhhhhhhhhhhlLLHHHHHHHHHH--------


DSSP  HHHHhhhhhHHHHhhhhhllllllllllleeeehhhhhhhhhllllllllllleeeeellllh
```

```
DSSP  LLLL-----EEEE-----------------------------------------------


DSSP  hhHHHHHH--------------------------hhhhhllLLEEEEEL-LLEElllll
Query elIHEQLA--------------------------dagetgwEGFTAIFT-APEEiaapgx  121
ident                                              |
Sbjct lkRNGRKItmyqtrfllngeeegrwpvpvnikkkdgverilLEDEASIEaDGLI------  474
DSSP  eeELLLEEeeeeeeellllleeellllleeeeeellllleeeeeELLLEEEElLLLL------


DSSP  llllleeeeeeeeelleeeeeeeeEEEE---------------lllllllLEEEeelhhh
Query pvkprrftaklsyrgrafatvpiEVSS---------------veagnadqFDTLtsdalg  166
ident                          |
Sbjct ------kinadsagfyrvlyddaTFSDvmghyrdlspldriglvddlfafLLSG------  522
DSSP  ------eelhhhllllleeeelhhHHHHhhhlhhhllhhhhhhhhhhhhhhHHHL------


DSSP  hlllllleeEEEL--LHHHHhHHHHHHHhlllllllllLLHHHHHHHHHHHHHLL--HHH
Query lvgvpaavaVPCX--TIPWQiAQKLHAVtavleepkandRAHDLVDLQLLEGLLL--DAD  222
ident                                                      |
Sbjct ---------HIDPetYRQRI-RNFFDDE-----------DHNVITAIVGQMEYLRmlTHA  561
DSSP  ---------LLLHhhHHHHH-HLLLLLL-----------LHHHHHHHHHHHHHHHLllLLL


DSSP  HHHHhhhhhhhhhhhhlllllLLLL--LLLLLHHhhhhhhhllllllllllLHHHHHHHHH
Query LXPTrsaciaifearaqhpwPPRV--ATLPHWPliyagalegldhlelarTVDAAAQAVQ  280
ident                       |    |                          |   |
Sbjct FDDD-----------arafcRSRMqfLTGKQDE-----------------NLKIALGRVS  593
DSSP  LHHH-----------hhhhhHHHHhhHLLLLLH-----------------HHHHHHHHHH


DSSP  HHHHHHHH----------------------------------------------------
Query RFVARIDR----------------------------------------------------  288
ident |     |
Sbjct RLYVMVDEsyaeemsklfkdfdsaepemrssiatayalvtgdlkgllekfrsvdrdedrv  653
DSSP  HHHHHHLHhhhhhhhhhhhhllllllhhhhhhhhhhhhhhhlllhhhhhhhhhhlllllhhhh


DSSP  ------------------------------------------------------------
Query ------------------------------------------------------------  288
ident
Sbjct riisafgklksntdlstvygmvekteikkqdmisffssaletlpgrefifanldriirlv  713
DSSP  hhhhhhhhllllllllllllllllllllhhhlhhhhhhhlllhhhhhhhhhhhhhhlhhhh


DSSP  ------------------------------------------------------------
Query ------------------------------------------------------------  288
ident
Sbjct iryftgnrtasrtvemmipvigldhpdaedivrnigsknismglakgiemlavnrklver  773
DSSP  hhhllllllhhhhhhhhhhhhhhllllllhhhhhhllllllhhhhhhhhhhhhhhhhhhhhh


DSSP  ---llll
Query ---atkr  292
ident
Sbjct irqtavk  780
DSSP  hllllll
```

No 737: Query=mol1A Sbjct=3j2mA Z-score=2.2

back to top

```
DSSP  llllllllllhhhhhhhhhhhhhhlLLLHhHHHHHHHHHHHHHHHHHHLLLLL--------L
Query aapysspptnlrslrdrltqvaerqGVVFgRLQRHVAXIVVAQFAATLTDD--------T   52
ident                          |
Sbjct -------------------------GYFY-NSSFRRYATLMGDLFSNIQIKrqlesgdkF   34
DSSP  -------------------------LLLL-LLHHHHHHHHHHHHHHLLLLEEEeellllleeE


DSSP  LLLlEEEEHH-------------------------------------------hhhhh
Query GAPlLLVKGG-------------------------------------------sslel   67
ident
Sbjct IRV-PITYASkehfmmklnkwtsinsqedvakvetilprinlhlvdfsynapvvsqynps   93
DSSP  EEL-LEEELLhhhhhhhhhhhhlllllhhhhhhhhhhllleeeeeeeeeeeellllllllle
```

```
Query rrgipdsrtskdfatvarrdiELIHEQLADagETGWEG-------------FTAIFTAPE  114
ident 
Sbjct pikmiyelsiftryeddmfqiVEQILPYFQ-pHFNTTMyeqfgndipfkrdIKIVLMSAA  152
DSSP  eeeeeeeeeeeellhhhhhhhHHHHHHHLL-lEEEEEEeeeellllleeeeEEEEEEEEE


DSSP  ELLLLL-lLLLLEEEEEEEEEElleeeeeeeeeeeellllllllleeeeelhhhHLLL---
Query EIAAPG-xPVKPRRFTAKLSYrgrafatvpievssveagnadqfdtltsdalgLVGV---  170
ident    |           |
Sbjct IDEAIDgrRRIEWSLTFEVNG--------------------wmyppvddaeglIRTTytd  192
DSSP  EELLLLllLLEEEEEEEEEEEE--------------------eellllllhhhllEEEEeel


DSSP  ----LLLE----EEEELLHhhhhhhhhhhhhlllllllllllhhhhhhhhhhhhllhhh
Query ----PAAV----AVPCXTIpwqiaqklhavtavleepkandrahdlvdlqllegllldad  222
ident              |
Sbjct fhanTRDLpdgeGVFESVD-----------------------------------------  211
DSSP  llllEEELllhhHHLLLLL-----------------------------------------


DSSP  hhhhhhhhhhhhhhhllllllllllllllhhhhhhhhhllllllllllllhhhhhhhhhhh
Query lxptrsaciaifearaqhpwpprvatlphwpliyagalegldhlelartvdaaaqavqrf  282
ident 
Sbjct ------------------------------------------------------------  211
DSSP  ------------------------------------------------------------


DSSP  hhhhhhllll
Query varidratkr  292
ident 
Sbjct ----------  211
DSSP  ----------
```

No 738: Query=mol1A Sbjct=1s5uE Z-score=2.2

back to top

```
DSSP  llllllllllhhhhhHHHHHhhhhllllHHHHHHHHHHHHHHHHHHHHLLllllllLLEEee
Query aapyssspptnlrslrDRLTQvaerqggvvFGRLQRHVAXIVVAQFAATLtddtgaPLLLvk   60
ident                   |
Sbjct ghmnttlfrwpvrvyYEDTD---aggvvYHASYVAFYERARTEMLRHH------HFSQ--   49
DSSP  lllllllleeeeeellHHHLL---lllllLHHHHHHHHHHHHHHHHHHHL------LLLH--


DSSP  hhhhhhhhhllllllllleeeeELLL------hhhhhhhhhhhhhhllLLEEEEEELLLE
Query ggsslelrrgipdsrtskdfatvARRD------ieliheqladagetgwEGFTAIFTAPE  114
ident                                                         |
Sbjct ----------------------qaLMAErvafvvrkmtveyyaparlddmLEIQTEITSMR   88
DSSP  ----------------------hhHHHLlleeeeeeeeeeellllllleEEEEEEEEEEL


DSSP  ElllllllllllEEEEEEEEEL-----LEEEEEEEEEEEellllllllleeeeelhhhhll
Query EiaapgxpvkpRRFTAKLSYR-----GRAFATVPIEVSsveagnadqfdtltsdalglvg  169
ident                               | |
Sbjct G----------TSLVFTQRIVnaentLLNEAEVLVVCV----------------------  116
DSSP  L----------LEEEEEEEEElllllEEEEEEEEEEEE----------------------


DSSP  llllleeeeellhhhHHHHhhhhhhllllllllllllhhhhhhhhhhhhllhhhhhhhhhh
Query vpaavavpcxtipwQIAQklhavtavleepkandrahdlvdlqllegllldadlxptrsa  229
ident 
Sbjct -------------dPLKM------------------------------------------  121
DSSP  -------------eLLLL------------------------------------------


DSSP  hhhhhhhllllllllllllllhhhhhhhhhlllllllllllhhhhhhhhhhhhhhhhhl
Query ciaifearaqhpwpprvatlphwpliyagalegldhlelartvdaaaqavqrfvaridra  289
ident 
Sbjct ------------------------------------------------kpralpksivae  133
DSSP  ------------------------------------------------eellllhhhhhh


DSSP  lll
Query tkr  292
```

No 739: Query=mol1A Sbjct=3zhpA Z-score=2.2

back to top

```
DSSP  lllllllllhhhhhhhhhhhhhhhllllhhhhhhhhhhHHHHHHHhlllllllllleeee
Query aapyssptnlrslrdrltqvaerqgvvfgrlqrhvaxIVVAQFAatltddtgaplllvk   60
ident 
Sbjct --------------------------------------AEIVKIL---------------    7
DSSP  --------------------------------------LHHHHHH---------------


DSSP  hhhhhhhhhlllllllllleeeeelllhhhhhhhhhhhhhhllllleeeeellLEELllll
Query ggsslelrrgipdsrtskdfatvarrdieliheqladagetgwegftaiftaPEEIaapg  120
ident                                                      |
Sbjct ----------------------------------------------kdnlaiLEKQ----   17
DSSP  ----------------------------------------------hhhhhhHHLL----


DSSP  llllleEEEEeeeelleeeeEEEEeeeelllllllllleeeeELHHhhlllllleEEEEL-
Query xpvkprRFTAklsyrgrafaTVPIevssveagnadqfdtltSDALglvgvpaavAVPCX-  179
ident          |                                  |
Sbjct ---dkkTDKA---------sEEVS-kslqamkeilcppteaVAQL----aqelySSGLLv   60
DSSP  ---lhhHHLH---------hHHHH-hhhhhhhhhhllllllHHHH----hhhhhHHLHHh


DSSP  ----------------------------------------lhHHHHHHHHHHHHLlllll
Query ----------------------------------------tiPWQIAQKLHAVTAvleep  199
ident                                           |      |    |
Sbjct tliadlqlidfegkkdvtqifnnilrrqigtrsptveyisahPHILFMLLKGYEA-----  115
DSSP  hhhhllllllhhhhhhhhhhhhhhhllllllllhhhhhhhhlLHHHHHHHHHHHL-----


DSSP  llLLLL-HHHHHHHHHHHHHLLhhhhhHHHHhhhhhhhhhhllllLLLL--LLLLLlhhhhh
Query kaNDRA-HDLVDLQLLEGLLLdadlxPTRSaciaifearaqhpWPPR--VATLPhwpliy  256
ident      |      |                                       |
Sbjct --PQIAlRCGIMLRECIRHEP--lakIILF----------snqFRDFfkYVELS------  155
DSSP  --LLLHhHHHHHHHHHHLLLHH--hhhHHHL----------lhhHHHHhhHLLLL------


DSSP  hhhhlllllllllllLHHHHHHHHHHHHHHH-----hhhLLLL-------------------
Query agalegldhlelarTVDAAAQAVQRFVAR-----idrATKR-------------------  292
ident                    | |
Sbjct ------------tfDIASDAFATFKDLLTrhkvlvadFLEQnydtifedyekllqsenyv  203
DSSP  ------------lhHHHHHHHHHHHHHHHllhhhhhhHHHHlhhhhhhhhhhhhllllhh


DSSP  ------------------------------------------------------------
Query ------------------------------------------------------------  292
ident 
Sbjct tkrqslkllgelildrhnfaimtkyiskpenlklmmnllrdkspniqfeafhvfkvfvas  263
DSSP  hhhhhhhhhhhhhhlhhhhhhhhhhhlllhhhhhhhhhhlllllhhhhhhhhhhhhhhhhl


DSSP  ----------------------------------------------------
Query ----------------------------------------------------  292
ident 
Sbjct phktqpiveillknqpklieflssfqkertddeqfadeknylikqirdlkkt  315
DSSP  llllhhhhhhhhhlhhhhhhhhhhlllllllllhhhhhhhhhhhhhhlllll
```

No 740: Query=mol1A Sbjct=2mxzA Z-score=2.2

back to top

```
DSSP  lllllllllhhhhhhhhhhhhhhhllllhhhhhHHHHhhHHHHHHHHLLLLlllllLEEEE
Query aapyssptnlrslrdrltqvaerqgvvfgrlqRHVAxiVVAQFAATLTDdtgapLLLVK   60
ident                                             |
Sbjct -------------------msfkfgknsekqlaTVKP--ELQKVARRALE-lspyDFTIV   38
DSSP  -------------------llllllhhhhhhhhLLLL--LHHHHHHHHHH-hlllLEEEE
```

```
Sbjct Q-GIRT-----VAQSaqniangtsflkdpskskhitgdaIDFAPYingkidwndleAFWA   92
DSSP  E-LLLL-----LLHHhhhhhlllllllllllllhhhlleEEEEEEelleellllhhHHHH


DSSP  HHHHHHHHHHHLLlleEEEELLL---------EELLLllllLLLEE-EEEEEeelleeee
Query IHEQLADAGETGWegfTAIFTAP---------EEIAApgxpVKPRR-FTAKLsyrgrafa  140
ident        ||                        ||
Sbjct VKKAFEQAGKELG---IKLRFGAdwnasgdyhDEIKR----GTYDGgHVELV--------  137
DSSP  HHHHHHHHHHHHL---LLLLLLLhhhhlllllLLLLL----LLLLLlLEELL--------


DSSP  eeeeeeeellllllllleeeeelhhhhllllllleeeeellhhhhhhhhhhhhhlllllll
Query tvpievssveagnadqfdtltsdalglvgvpaavavpcxtipwqiaqklhavtavleepk  200
ident
Sbjct ------------------------------------------------------------  137
DSSP  ------------------------------------------------------------


DSSP  lllllhhhhhhhhhhhhllhhhhhhhhhhhhhhhhhhhllllllllllllllhhhhhhhhh
Query andrahdlvdlqlleglldadlxptrsaciaifearaqhpwpprvatlphwpliyagal  260
ident
Sbjct ------------------------------------------------------------  137
DSSP  ------------------------------------------------------------


DSSP  llllllllllhhhhhhhhhhhhhhhhhllll
Query egldhlelartvdaaaqavqrfvaridratkr  292
ident
Sbjct --------------------------------  137
DSSP  --------------------------------
```

No 741: Query=mol1A Sbjct=1gxbD Z-score=2.2

back to top

```
DSSP  llllllllllhhhhhhhhhhhhhhhllllhhhhhhhhhhhhhhhhhhllllllllllleeEE
Query aapysspptnlrslrdrltqvaerqgvvfgrlqrhvaxivvaqfaatltddtgaplllVK   60
ident
Sbjct ----------------------------------------------------------MN    2
DSSP  ----------------------------------------------------------LL


DSSP  HHHHHHHHHLlllllllllleeeeelllhhhhhhhhhhhhhhllllleeeeellleelllll
Query GGSSLELRRGipdsrtskdfatvarrdieliheqladagetgwegftaiftapeeiaapg  120
ident     |
Sbjct INEILKKLIN--------------------------------------------------   12
DSSP  HHHHHHHHHL--------------------------------------------------


DSSP  llllleeeeeeeeeelleeeeeeeeeeeeellllllllleeeeelhhhhllllllleeeeelL
Query xpvkprrftaklsyrgrafatvpievssveagnadqfdtltsdalglvgvpaavavpcxT  180
ident
Sbjct -----------------------------------------------------ksdleiN   19
DSSP  -----------------------------------------------------lllllhH


DSSP  HHHHHHHHHHHHHlllllllllLLHH-HHHHHHHHHHLlhhhHHHHHHHHHHHHHHHHHLL
Query IPWQIAQKLHAVTavleepkandRAHD-LVDLQLLEGLLldadLXPTRSACIAIFEARAQ  239
ident      |                         |  |                       |
Sbjct EAEELAKAIIRGE---------vPEILvSAILVALRMKG---eSKNEIVGFARAMRELAI   67
DSSP  HHHHHHHHHHLLL---------lLHHHhHHHHHHHHHHHL---lLHHHHHHHHHHHHHHHLL


DSSP  ------------------------------------------------------------
Query ------------------------------------------------------------  239
ident
Sbjct kidvpnaidtagtggdglgtvnvstasaillslvnpvakhgnravsgksgsadvlealgy  127
DSSP  llllllleeeeelllllllllllhhhhhhhhhhllllleeeeellllllllllhhhhhhhhll


DSSP  --------------------------lllllLLLLlllhhhhhhhhhllllllllllllhH
Query --------------------------hpwppRVATlphwpliyagalegldhlelartvD  273
ident                                 ||
Sbjct niivpperakelvnktnfvflfaqyyhpamkNVAN-----------------------vrK  165
```

```
DSSP  HHHHHhhHHHHHH---------------------hhLLLL--------------------
Query AAAQAvqRFVARI---------------------drATKR--------------------  292
ident
Sbjct TLGIR--TIFNILgpltnpanakyqlmgvfskdhldLLSKsayeldfnkiilvygepgid  223
DSSP  HHLLL--LHHHHLhhhllllllleeeeelllhhhhhHHHHhhlllllleeeeeeelllll


DSSP  ------------------------------------------------------------
Query ------------------------------------------------------------  292
ident
Sbjct evspigntfmkivskrgieevklnvtdfgispipieklivnsaedsaikivraflgkdeh  283
DSSP  lllllleeeeeeeelleeeeeeeehhhhlllllllhhhhllllhhhhhhhhhhhhhlllhh


DSSP  ------------------------------------------------------------
Query ------------------------------------------------------------  292
ident
Sbjct vaefikintavalfaldrvgdfregyeyadhliekslddklneiismngdvtklktivvks  343
DSSP  hhhhhhhhhhhhhhhhlllllhhhhhhhhhhhhhhhhhhhhhhhhhllllhhhhhhhhhhl


DSSP  --
Query --  292
ident
Sbjct sg  345
DSSP  ll
```

No 742: Query=mol1A Sbjct=4c00A Z-score=2.2

back to top

```
DSSP  ------------------------------------------------------------
Query ------------------------------------------------------------    0
ident
Sbjct rlqveglsgqleknvraqlstiesdevtpdrrfrarvddaireglkalgyyqptiefdlv   60
DSSP  leeeellllhhhhhhhhhlllllllllllllhhhhhhhhhhhhhlllleeeeelleell


DSSP  ------------------------------------------------------------
Query ------------------------------------------------------------    0
ident
Sbjct liakvtpgvpvliggtdvvlrggartdkdylklldtrpaigtvlnqgdyenfkksltsia  120
DSSP  eeeleeeeeleeeeeeeeeeehhhhlhhhhhhhhhlllllllllhhhhhhhhhhhhhhh


DSSP  ------------llllllllllhhhhhhhhhhhhhhllllhhhhhhhHHHHHHHHHHhll
Query ------------aapysspptnlrslrdrltqvaerqgvvfgrlqrhVAXIVVAQFAatl   48
ident
Sbjct lrkgyfdseftkaqlgialglhkafwdidynsgeryrfghvtfegsqIRDEYLQNLV---  177
DSSP  hhhllllleeeeeeeeeellllleeeeeeeeeeelllleeeeeeeeelllLLHHHHHLLL---


DSSP  lllllllleeeehhhhhhhhhllllllllllleeEEELLLhHHHHHHHHHHHHHHlLLLE-E
Query tddtgaplllvkggsslelrrgipdsrtskdfaTVARRDiELIHEQLADAGETgWEGF-T  107
ident                                               |          |
Sbjct ------------------------------pfkEGDEYE-SKDLAELNRRLSA-TGWFnS  205
DSSP  ------------------------------lllLLLLLL-HHHHHHHHHHHHH-LLLEeE


DSSP  EEELLLE-ELLLllllLLLEEEEEEEEEELL--eeeeeeeeeeeellllllllleeeeelH
Query AIFTAPE-EIAApgxpVKPRRFTAKLSYRG--rafatvpievssveagnadqfdtltsdA  164
ident                  |    |   | |
Sbjct VVVAPQFdKARE----TKVLPLTGVVSPRTentietgvgystdvgprvkatwkkpwmnsY  261
DSSP  EEEEELHhHHHH----HLEEEEEEEEEELLlleeeeeeeeellleeeeeeeeeellllL


DSSP  HHH----------------lllLLLEEEeellhhhhhhhhhhhhhllllllllllllhhh
Query LGL----------------vgvPAAVAVpcxtipwqiaqklhavtavleepkandrahdl  208
ident
Sbjct GHSlttstsisapeqtldfsykMPLLKNpleqyylvqggfkrtdlndtesdsttlvasry  321
DSSP  LLEeeeeeeeelleeeeeeeeeEELLLLllleeeeeeeeeeeeeelleeeeeeeeeeee


DSSP  hhhhhhhhhllhhhhhhhhhhhhhhhhhhlllllllllllllhhhhhhhhhllllLLLL
```

```
DSSP  eellllleeeeeeeeeeeeeeeeelleeeeeeeeeeeeeeeeeeeeellllllleeeeeeEEEE


DSSP  LLL---------------------------------------------------------
Query ART---------------------------------------------------------  271
ident
Sbjct YSNtawgsdvdfsvfqaqnvwirtlydrhrfvtrgtlgwietgdfdkvppdlrffaggdr  441
DSSP  EELhhhllllleeeeeeeeeeeeeeellleeeeeeeeeeeeeeellhhhllhhhllllllll


DSSP  ------------------------------------------------------------
Query ------------------------------------------------------------  271
ident
Sbjct sirgykyksiapkyangdlkgasklitgsleyqynvtgkwwgavfvdsgeavsdirrsdf  501
DSSP  llllllllllllllllllllllleeeeeeeeeeeeeelleeeeeeeeeeeeeellllllll


DSSP  ----------------------hhhhhhhhhhhhhhhhhllll
Query ----------------------vdaaaqavqrfvaridratkr  292
ident
Sbjct ktgtgvgvrwespvgpikldfavpvadkdehglqfyiglgpel  544
DSSP  eeeeeeeeeeeelleeeeeeeeeelllllllllleeeeellllll
```

No 743: Query=mol1A Sbjct=3jbrA Z-score=2.2

back to top

```
DSSP  ------------------------------------------------------------
Query ------------------------------------------------------------    0
ident
Sbjct fcltlqnplrkacisivewkpfetiilltifancvalavylpmpeddnnslnlglekley   60
DSSP  llllllllhhhhhhhhhlllhhhhhhhhhhhhhhhhhhhhhhlllllllllllhhhhhhhhh


DSSP  ----------------------LLLL----------------------------------
Query ----------------------AAPY----------------------------------    4
ident                          |
Sbjct ffltvfsieaamkiiaygflfhQDAYlrsgwnvldfiivflgvftailvkalrafrvlrp  120
DSSP  hhhhhhhhhhhhhhhllllllhhHLHHhhhhhhhhhhhhhhhhhhhhhhllhhhhhhhhhll


DSSP  ---------lLLLL----------------------------------------------
Query ---------sSPPT----------------------------------------------    9
ident
Sbjct lrlvsgvpslQVVLnsifkamlplfhiallvlfmviiyaiiglelfkgkmhktcyyigtd  180
DSSP  hhhhhllllhHHHHhhhhhhhlllhhhhhhhhhhhhhhhhhhhllhhhhhhhhhlllllll


DSSP  ------------------------------------------------------------
Query ------------------------------------------------------------    9
ident
Sbjct ivatvenekpspcartgsgrpctingsecrggwpgpnhgithfdnfgfsmltvyqcitme  240
DSSP  lllllllllllllllllllllllllllllllllllllllllllllhhhhhhhhhhhhlll


DSSP  ------------------------------------------------------------
Query ------------------------------------------------------------    9
ident
Sbjct gwtdvlywvndaignewpwiyfvtlillgsffilnlvlgvlsgeftkerekaqqleedlr  300
DSSP  lhhhhhhhhhhllllllhhhhhhhhhhhhhhhhhhhhhhhhhhhhhhhhhhhhhllhhhhhhh


DSSP  ------------------------------------------------------------
Query ------------------------------------------------------------    9
ident
Sbjct gymswitqchdlvksrvfywlvilivalntlsiasehhnqplwlthlqdianrvllslft  360
DSSP  hhhhhhhllhhhhhhllllllhhhhhhhhhhhhhhhhhhhhhhhhlllllllllllllllhhh


DSSP  ------------------------------------------------------------
Query ------------------------------------------------------------    9
ident
Sbjct iemllkmyglglrqyfmsifnrfdcfvvcsgilelllvesgamtplgisvlrcirllrlf  420
DSSP  hhhhhhhhhhhhhhhhhhhhhlhhhhhlhhhhhhhhhhhhhhlllllllllhhhhhhhhhhh
```

```
Query ---------------------nlrslrdrltqvaerqgvvfgrlqrhvaxivvaqfaatl   48
ident
Sbjct kitkywtslsnlvasllnsirsiaslllllflfiiifallgmqlfggrydfedtevrrsn  480
DSSP  hhhhllllhhhhhhhhhhhhhhhhhhhhhhhhhhhhhhhhhhhhhlllllllllllllll


DSSP  lllllllleeeehhhhhhhhhlllllllllleeeeelLLHHHhHHHHHHHHhhlllleee
Query tddtgaplllvkggsslelrrgipdsrtskdfatvarRDIELiHEQLADAGetgwegfta  108
ident
Sbjct fdnfpqalisvfqvltgedwnsvmyngimayggpsypGVLVC-IYFIILFV---------  530
DSSP  lllhhhhhhhhhhhhhlllhhhhhhhhhhhhllllllLHHHH-HHHHHHHH---------


DSSP  eellleelllllllllleeeeeeeeELLE-------------------------------
Query iftapeeiaapgxpvkprrftaklsYRGR-------------------------------  137
ident
Sbjct ------------------cgnyillNVFLaiavdnlaeaesatwftnfillfillssaal  572
DSSP  ------------------hhhhhhhHHHHhhhhhhhhhhhllhhhhhhhhllhhhhhhhh


DSSP  ------------------------------------------------------------
Query ------------------------------------------------------------  137
ident
Sbjct aaedpiraesvrnqilgyfdiaftsvftveivlkmttygaflhkgsfcrnyfnildllvv  632
DSSP  hhhhhhhhhhhhlllhhhhhlllhhhhhhhhhhhhhhhhhhhhhhhlllllhhhhhlhhhh


DSSP  ------------------------------------------------------------
Query ------------------------------------------------------------  137
ident
Sbjct avslismglesstisvvkilrvlrvlrplrainrakglkhvvqcvfvairtignivlvtt  692
DSSP  hhhhhhhhllllllllllhhhhhhlllhhhhhhllhhhhhhhhhhhhhhhhlhhhhhhhh


DSSP  ------------------------------------------------------------
Query ------------------------------------------------------------  137
ident
Sbjct llqfmfacigvqlfkgkffscndlskmteeecrgyyyvykdgdptqmelrprqwihndfh  752
DSSP  hhhhhhhhhhhhhhllllllllllllllllllllllleellllllleellllllllllll


DSSP  ------------------------------------------------------------
Query ------------------------------------------------------------  137
ident
Sbjct fdnvlsammslftvstfegwpqllyraidsneedmgpvynnrvemaiffiiyiiliaffm  812
DSSP  lllhhhhhhhhhhhhllllhhhhhhhhhllllllllllllllhhhhhhhhhhhhhhhhhh


DSSP  -----------------eeeeeeeeeeelllllLLLEeeeelhhhhllllllleeeeeLL
Query -----------------afatvpievssveagnaDQFDtltsdalglvgvpaavavpcXT  180
ident
Sbjct mnifvgfvivtfqeqgeteypknpyqyqvwyvvtSSYF-------------------eYL  853
DSSP  hhhhhhhhhhhhhhhhhhhhllllhhhhhhhhhhhLLHH-------------------hHH


DSSP  HHHHHHHHHHHHHLLL-LLLLLLL--LLHHHHHHHHHHHHLLhhhhhhhhhhhhhhhhhh
Query IPWQIAQKLHAVTAVL-EEPKAND--RAHDLVDLQLLEGLLLdadlxptrsaciaifear  237
ident     |                          |       |
Sbjct MFALIMLNTICLGMQHyHQSEEMNhiSDILNVAFTIIFTLEM------------------  895
DSSP  HHHHHHHHHHHHHLLLlLLLLLHHhhHHHHHHHHHHHHHHHH------------------


DSSP  llllllllllllllhhhhhhhhhllllllLLLLL--------HHHHHHHHHHHHhHHHH-
Query aqhpwpprvatlphwpliyagalegldhlELART--------VDAAAQAVQRFVaRIDR-  288
ident
Sbjct ilkllafkargyfgdpwnvfdflivigsiIDVILseidtrisSAFFRLFRVMRL-IKLLs  954
DSSP  hhhhllllllllllllhhhhhhhhhhhhhHHHHHhhlllllhHHHHHHHHHHHH-HHHHh


DSSP  LLLL--------------------------------------------------------
Query ATKR--------------------------------------------------------  292
ident
Sbjct RAEGvrtllwtfiksfqalpyvallivmlffiyavigmqmfgkialvdgtnrnnnfqtfp 1014
DSSP  HLLLlhhhhhhhhhhhhlllhhhhhhhhhhhhhhhhhhllllllllllllllllllllhh


DSSP  ------------------------------------------------------------
```

```
DSSP  hhhhhhhhhhhlllhhhhhhhhlllllllllllllllllllllllllhhhhhhhhhhhhh


DSSP  ------------------------------------------------------------
Query ------------------------------------------------------------  292
ident 
Sbjct liinlfvavimdnfdyltrdwsilgxxxxxxxxxxxxxxxxxxxxxxxxxxxxxxxxxxx 1134
DSSP  hhhhhhhhhhhhlhhhhhhhhhhhlllllhhhhhhhhhhlllllhhhhhhlhhhhhhh


DSSP  ------------------------------------------------------------
Query ------------------------------------------------------------  292
ident 
Sbjct xxxxxxxxxxxxxxxxxxxxxxxxxxxxxxxxxxxxxxxxxxxxxxxxxxxxxxxxxxxx 1194
DSSP  hhhlllllllhhhhhhhhhhhhhlllllhhhhhhhhhhhhhhhlllllhhhhllllhhh


DSSP  ------------------------------
Query ------------------------------  292
ident 
Sbjct xxxxxxxxxxxxxxxxxxxxxxxxxxxxxx 1224
DSSP  hhhhhhhlllllllhhhhhhhhhhhhhhhl
```

No 744: Query=mol1A Sbjct=5i6xA Z-score=2.2

back to top

```
DSSP  LLLLLlllllhhhhhhhhhhhhhhhllllhhhhhhhhhhhhhhhhhhlllllllllleeee
Query AAPYSspptnlrslrdrltqvaerqgvvfgrlqrhvaxivvaqfaatltddtgaplllvk   60
ident 
Sbjct GSQGE-------------------------------------------------------    5
DSSP  LLLLL-------------------------------------------------------


DSSP  hhhhhhhhlllllllllleeeeelllhhhhhhhhhhhhhhllllleeeeellleellll
Query ggsslelrrgipdsrtskdfatvarrdieliheqladagetgwegftaiftapeeiaapg  120
ident 
Sbjct ------------------------------------------------------------    5
DSSP  ------------------------------------------------------------


DSSP  llllleeeeeeeeeelleeeeeeeeeeeellllllllleeeeelhhhhlLLLLLE------
Query xpvkprrftaklsyrgrafatvpievssveagnadqfdtltsdalglvGVPAAV------  174
ident 
Sbjct ------------------------------------------------RETWGKkvdfll   17
DSSP  ------------------------------------------------LLLLLLhhhhhh


DSSP  ---eeeeLLHH-HHHHHHHHHHHL------------------------------------
Query ---avpcXTIP-WQIAQKLHAVTA------------------------------------  194
ident             |
Sbjct svigyavDLGNvWRFPYICAQNGGgafllpytimaifggiplfymelalgqyhrngcisi   77
DSSP  hhhhhhlLHHHhHHHHHHHHHHLLHhhhhhhhhhhhhhlhhhhhhhhhhhhhhhlllllhh


DSSP  ------------------------------------------------------------
Query ------------------------------------------------------------  194
ident 
Sbjct wrkicpifkgigyaiciiafyiasyyntimawalyylissftdqlpwtscknswntgnct  137
DSSP  hhlllhhhhhhhhhhhhhhhhhhllhhhhhhhhhhhhhllllllllllllllllllll


DSSP  ------------------------------------------------------------
Query ------------------------------------------------------------  194
ident 
Sbjct nyfsednitwtlhststspaeefytrhvlqihrskglqdlggiswqlalcimliftviyfsi  197
DSSP  llllllllllllllllhhhhhhhhllllhhhllllllllllllhhhhhhhhhhhhhhhhh


DSSP  ----------------------------------------llllllLLLL--LHHHHHHH
Query ----------------------------------------vleepkANDR--AHDLVDLQ  212
ident                                                           |
Sbjct wkgvktsgkvvwvtatfpyialsvllvrgatlpgawrgvlfylkpnWQKLleTGVWIDAA  257
DSSP  lllhhhhhhhhhhllhhhhhhhhhhhhhhlllllhhhhhhhhlllHHHHhlHHHHHHHH
```

```
Query LLEGLLL----------------------------DADLXPTRSACIAIFEARA------  238
ident       |                                         ||
Sbjct AQIFFSLgpgfgvllafasynkfnnncyqdalvtsVVNCMTSFVSGFVIFTVLGymaemr  317
DSSP  HHHHHHHlllllhhhhhhhllllllllhhhhhhhhHHHHHHHHHHHHHHHHHHHhhhhhl


DSSP  ------------------------------------------------------------
Query ------------------------------------------------------------  238
ident
Sbjct nedvsevakdagpsllfityaeaianmpastffaiifflmlitlgldssfaglegvitav  377
DSSP  llllhhhllllhhhhhlhhhhhhhhllllhhhhhhhhhhhhhhhhhhhhhhhhhhhhhhh


DSSP  ------------------------------------------------------------
Query ------------------------------------------------------------  238
ident
Sbjct ldefphvwakrrerfvlavvitcffgslvtltfggayvvklleeyatgpavltvalieav  437
DSSP  hhhllllllllhhhhhhhhhhhhhhhhllllllhhhhhhhhhhhhllhhhhhhhhhhhhh


DSSP  ----------llllllllllllhhhhhhhhhhlLLLL-LLLL--LLHHH-----------
Query ----------qhpwpprvatlphwpliyagaleGLDH-LELA--RTVDA-----------  274
ident                                       |
Sbjct avswfygitqfcrdvkemlgfspgwfwricwvaISPLfLLFIiaSFLMSppqlrlfqyny  497
DSSP  hhhllllhhhhhhhhhhhlllllhhhhhhhhhlHHHHhHHHHhhHHHHLlllllllllll


DSSP  -----------------------------hhhhhhhhhhhhhhllll
Query -----------------------------aaqavqrfvaridratkr  292
ident
Sbjct pywsiilgyaigtssficiptyiayrliitpgtfkeriiksitpetp  544
DSSP  lllhhhhhhhhhhllllhhhhhhhhhhhhllllhhhhhhhhhlllll
```

No 745: Query=mol1A Sbjct=2wzkA Z-score=2.2

back to top

```
DSSP  llllllllllhhhhhhhhhhhhhhllllhhhhhhhhhhhhhhhhhhlllllllllleeee
Query aapysspptnlrslrdrltqvaerqgvvfgrlqrhvaxivvaqfaatltddtgaplllvk   60
ident
Sbjct ------------------------------------------------------------    0
DSSP  ------------------------------------------------------------


DSSP  hhhhhhhhllllllllllleeeeellllhhhhhhhhhhhhhhlllleeeeeellleelllll
Query ggsslelrrgipdsrtskdfatvarrdieliheqladagetgwegftaiftapeeiaapg  120
ident
Sbjct ------------------------------------------------------------    0
DSSP  ------------------------------------------------------------


DSSP  llllleeeeeeeeeelleeeeeeeeeeellllllllleeeeelhhhhlllllleeeeeeLL
Query xpvkprrftaklsyrgrafatvpievssveagnadqfdtltsdalglvgvpaavavpcXT  180
ident
Sbjct ------------------------------------------------------slqfED    6
DSSP  ------------------------------------------------------lllhHH


DSSP  HHHHHHHHHHHHHLLllllllLLLLH-HHHHHHHHHHHL---LHHHHHHHHHHHHHHHHH
Query IPWQIAQKLHAVTAVleepkaNDRAH-DLVDLQLLEGLL---LDADLXPTRSACIAIFEA  236
ident                               ||           |        |
Sbjct KWDFMHPIVLKLLRQ------ESVTKqQWFDLFSDVHAVclwDDKGSSKIHQALKEDILE   60
DSSP  HHHHHHHHHHHHHHL------LLLLHhHHHHHHHHHHHHhhhLLLHHHHHHHHHHHHHHH


DSSP  HLL------------------------llllllLLLLlllhhhhhhhhllllllllllllh
Query RAQ------------------------hpwppRVATlphwpliyagalegldhlelartv  272
ident
Sbjct FIKqaqarvlshqddtallkayivewrkfftqCDIL------------------------   96
DSSP  HHHhhhhhhhhllllhhhhhhhhhhhhhhhhhLLLL------------------------


DSSP  hhhhhhhHHHHHHH----------------------------------------------
Query daaaqavQRFVARI----------------------------------------------  286
```

```
DSSP  ------------------------------------------------------------
Query ------------------------------------------------------------  286
ident
Sbjct geafdsqlvigvresyvnlcsnpedklqiyrdnfekayldsterfyrtqapsylqqngvq  209
DSSP  lllllhhhhhhhhhhhlllllllllhhhhhlhhhhhhhhhhhhhhhhhhhhhhhhhhhhh


DSSP  ------------------------------------------------------------
Query ------------------------------------------------------------  286
ident
Sbjct nymkyadaklkeeekralryletrrecnsvealmeccvnalvtsfketilaecqgmikrn  269
DSSP  hhhhhhhhhhhhhhhhhhhhlllllllhhhhhhhhhhhhhlhhhhhhhhllhhhhhhll


DSSP  ------------------------------------------------------------
Query ------------------------------------------------------------  286
ident
Sbjct eteklhlmfslmdkvpngiepmlkdleehiisagladmvaaaetittdsekyreqldtlf  329
DSSP  lhhhhhhhhhhlllllllhhhhhhhhhhhhhhhhhhhhhhhhhhllllhhhhhhhhhhhh


DSSP  ----------------------------------hhllll
Query ----------------------------------dratkr  292
ident
Sbjct nrfsklvkeafqddprfltardkaykavvndatifkaenl  369
DSSP  hhhhhhhhhhlllhhhhhhhhhhhhhhhhhhhhhllllll
```

No 746: Query=mol1A Sbjct=4yayA Z-score=2.2

back to top

```
DSSP  lllllllllhhhhhhhhhhhhhhllllhhhhhhhhhhhhhhhhhlllllllllleeee
Query aapysspptnlrslrdrltqvaerqgvvfgrlqrhvaxivvaqfaatltddtgaplllvk   60
ident
Sbjct ------------------------------------------------------------    0
DSSP  ------------------------------------------------------------


DSSP  hhhhhhhhlllllllllleeeeelllhhhhhhhhhhhhhhlllleeeeellleelllll
Query ggsslelrrgipdsrtskdfatvarrdielihehqladagetgwegftaiftapeeiaapg  120
ident
Sbjct ------------------------------------------------------------    0
DSSP  ------------------------------------------------------------


DSSP  lllllleeeeeeeeelleeeeeeeeeeeellllllllleeeeelhhhhllllleeeeeelL
Query xpvkprrftaklsyrgrafatvpievssveagnadqfdtltsdalglvgvpaavavpcxT  180
ident
Sbjct --------------------------------------------------------dleD    4
DSSP  --------------------------------------------------------lhhH


DSSP  HHHHHHHHHHHHHLllllllLLLL-LHHHHHHHHHHHHHLL---------hhhhhhhhhh
Query IPWQIAQKLHAVTAvleepkANDR-AHDLVDLQLLEGLLL---------dadlxptrsac  230
ident         |                   |
Sbjct NWETLNDNLKVIEK----adNAAQvKDALTKMRAAALDAQkatppkledkspdspemkdf   60
DSSP  HHHHHHHHHHHHHH----llLHHHhHHHHHHHHHHHHHHHlllllllllllllhhhhhh


DSSP  hhhhhhlllllllllLLLLHhhhhhhhlllllllllllhhhhHHHHHHHHHHHHH-L
Query iaifearaqhpwpprvaTLPHWpliyagalegldhlelartvdaaAQAVQRFVARIDR-A  289
ident                                                |
Sbjct rhgfdilvgqiddalklANEGK-------------------vkeaQAAAEQLKTTRNAyI  101
DSSP  hhhhhhhhhhhhhhhhhHHHLL-------------------lhhhHHHHLLHHHHHHHhH


DSSP  LLL---------------------------------------------------------
Query TKR---------------------------------------------------------  292
ident  |
Sbjct QKYlilnssdcpkagrhnyifvmiptlysiifvvgifgnslvviviyfymklktvasvfl  161
DSSP  HHHllllllllllllllhhhhhhhhhhhhhhhhhhhhhhhhhhhhhhhllllllhhhhhh
```

```
ident
Sbjct lnlaladlcflltlplwavytameyrwpfgnylckiasasvsfnlyasvflltclsidry  221
DSSP  hhhhhhhhhhhhhhhhhhhhhhllllllllhhhhhhhhhhhhhhhhhhhhhhhhhhhhhh


DSSP  ------------------------------------------------------------
Query ------------------------------------------------------------  292
ident
Sbjct laivhpmksrlrrtmlvakvtciiiwllaglaslpaiihrnvffiitvcafhyetlpigl  281
DSSP  hhhlllllllhhhhhhhhhhhhhhhhhhhhlhhhhhheeeelllleeeellllllllhhh


DSSP  ------------------------------------------------------------
Query ------------------------------------------------------------  292
ident
Sbjct gltknilgflfpfliiltsytliwkalkknddifkiimaivlffffswiphqiftfldvl  341
DSSP  hhhhhhhhlhhhhhhhhhhhhhhhlllllhhhhhhhhhhhhhhhhhhhhhhhhhhhhhhh


DSSP  ----------------------------------------------------
Query ----------------------------------------------------  292
ident
Sbjct iqlgiirdcriadivdtampiticiayfnnclnplfygflgkkfkryflqll  393
DSSP  hhhlllllhhhhhhhhhhhhhhhhhhhlllhhhhhhhhhlhhhhhhhhhhhl
```

No 747: Query=mol1A Sbjct=2wm9A Z-score=2.2

back to top

```
DSSP  ---------------------------------------------------------lll
Query ---------------------------------------------------------aap    3
ident
Sbjct stpelrktwldsmarihvkngdlseaamcyvhvtalvaeyltrkgvfrqgctafrvitpn   60
DSSP  lhhhhhhhhhhhhhhhhhhhhllhhhhhhhhhhhhhhhhhhhhhllllllhhhhllllhh


DSSP  llllllhhhhhhhhhhhhhhhhhllllhhhhhhhhhhhhhhhhhhllllllllleeeehhh
Query ysspptnlrslrdrltqvaerqgvvfgrlqrhvaxivvaqfaatltddtgaplllvkggs   63
ident
Sbjct ideeadvhfnedvlmelleqcadglwkaeryeliadiykliipiyekrrdferlahlydt  120
DSSP  hhhhlllllhhhhhhhhhhhhhhhhhhhlllhhhhhhhhlllhhhhhhlllhhhhhhhhh


DSSP  hhhhhhllllllllleeeeelllhhhhhhhhhhhhhhhlllLEEEeellleellllllll
Query slelrrgipdsrtskdfatvarrdielihegladagetgweGFTAiftapeeiaapgxpv  123
ident
Sbjct lhrayskvtevmhsgrrllgtyfrvaffgqgffededgkeyIYKE---------------  165
DSSP  hhhhhhhhhhhhhhlllllleeeeeeeeelhhhhhhhllleeEEEE---------------


DSSP  lleeeeeeeeelleeEEEEeeeeeelllllllllleeeeelhHHHLL--------------
Query kprrftaklsyrgraFATVpievssveagnadqfdtltsdaLGLVG--------------  169
ident                  |
Sbjct --------------pKLTP------------------lseiSQRLLklysdkfgsenvkm  193
DSSP  --------------lLLLL------------------hhhhHHHHHhhhhhhhlhhheee


DSSP  ---------------------------------------------llllEEEE-------
Query ---------------------------------------------vpaaVAVP-------  177
ident
Sbjct iqdsgkvnpkdldskyayiqvthvipffdekelqerktefershnirrfMFEMpftqtgk  253
DSSP  elllllllhhhllllleeeeeeeeeeelllhhhhhhllllhhhllleeeeEEEEeelllll


DSSP  -------------------------------------------eLLHHHHHHHHHHHHHL
Query -------------------------------------------cXTIPWQIAQKLHAVTA  194
ident                                                       |
Sbjct rqggveeqckrrtiltaihcfpyvkkripvmyqhhtdlnpievaIDEMSKKVAELRQLCS  313
DSSP  lllllllleeeeeeeeeeeeellllleeeeeeeeeeeeelhhhhhHHHHHHHHHHHHHHHH


DSSP  LLllllLLLLLHHHHHHHHHH----hhLLHHH-------------hhhhHHHHHhhhhhh
Query VLeepkANDRAHDLVDLQLLE----glLLDAD-------------lxptRSACIaifear  237
ident         |       ||            |
```

```
DSSP  lllllLLLL--LLLLlhhhhhhhhhllllllllllLHHHHHHHHHHHHHHHHH-LLLL--
Query aqhpwPPRV--ATLPhwpliyagalegldhlelarTVDAAAQAVQRFVARIDR-ATKR--  292
ident
Sbjct --eacGQALavNERL-----------------ikeDQLEYQEEMKANYREMAKeLSEImh  404
DSSP  --hhhHHHHhhHHHH-----------------lllLLLHHHHHHHHHHHHHHHhHHHHhl


DSSP  ---
Query ---  292
ident
Sbjct eql  407
DSSP  lll
```

No 748: Query=mol1A Sbjct=4owwA Z-score=2.2

back to top

```
DSSP  llllllllllhhhhhhhhhhhhhhllllhhhhhhhhhhhhhhhhhhlllllllllleeee
Query aapysspptnlrslrdrltqvaerqgvvfgrlqrhvaxivvaqfaatltddtgaplllvk   60
ident
Sbjct ------------------------------------------------------------    0
DSSP  ------------------------------------------------------------


DSSP  hhhhhhhhhlllllllllleeeeelllhhhhhhhhhhhhhhlllleeeEELLLEEL----
Query ggsslelrrgipdsrtskdfatvarrdieliheqladagetgwegftaIFTAPEEI----  116
ident                                                      |
Sbjct -----------------------------------------rlllstsLDAKDELEerle   19
DSSP  -----------------------------------------lllllllLLLLLHHHhhhh


DSSP  -----llllllllleeeeeeeeeelleeeeeeeeeeelllllllllleeeeelhhhhllll
Query -----aapgxpvkprrftaklsyrgrafatvpievssveagnadqfdtltsdalglvgvp  171
ident
Sbjct rcmsivtsmtagvsereandalnayvckglpqheeiclglftliltepaqaqkcyrdlal   79
DSSP  hhhhhhhhllllllhhhhhhhhhhhhlllhhhhhhhhhhhhhhhlhhhhhhhhhhhhhhh


DSSP  lleEEEE--------------------------------------LLHHHHHHHHhHHHH
Query aavAVPC--------------------------------------XTIPWQIAQKlHAVT  193
ident
Sbjct vsrDGMNivlnkinqilmekylklqdtcrtqlvwlvrelvksgvlGADGVCMTFM-KQIA  138
DSSP  hllLLLHhhhhhhhhhhhhhhhhllhhhhhhhhhhhhhhhhlllLHHHHHHHHH-HLLL


DSSP  LLlllllllLLLHHHHHHHHHH-HHLL--HHHHhHHHHhhhhhhhhhlllllLLLLLLll
Query AVleepkanDRAHDLVDLQLLE-GLLL--DADLxPTRSaciaifearaqhpwPPRVATlp  250
ident
Sbjct GG---dvtaKNIWLAESVLDILtEQREwvLKSS-ILIA--------mavytyLRLIVD--  184
DSSP  LL---lllhHHHHHHHHHHHHHhHLHHhhLLLH-HHHH--------hhhhhhHHHHLL--


DSSP  lhhhhhhhhllllllllllLLHHHHHHHHHHHHHHHH-HLLL------------------
Query hwpliyagalegldhlelaRTVDAAAQAVQRFVARID-RATK------------------  291
ident                        |  |    |
Sbjct ---------------hhgtAQLQALRQKEVDFCISLLrERFMeclmigrdlvrllqnvar  229
DSSP  ---------------llllHHHHHHHHHHHHHHHHHHhHLHHhhhhhllhhhhhhhllll


DSSP  ------------------------------------------------------------
Query ------------------------------------------------------------  291
ident
Sbjct ipefellwkdiihnpqalspqftgilqllqsrtsrkflacrltpdmetkllfmtsrvrfg  289
DSSP  lhhhhhhhhhhhhlhhhllllllllhhhhhllllllhhhhhllllhhhhhhhhhhlllllll


DSSP  ------------------------------------------------------------
Query ------------------------------------------------------------  291
ident
Sbjct qqkryqdwfqrqylstpdsqslrcdliryicgvvhpsnevlssdilprwaiigwllttct  349
DSSP  llhhhhhhhhhhhhlllhhhhhhhhhhhhhhhhhhhlllllhhhhlllllhhhhhhhlll
```

```
Sbjct snvaasnaklalfydwlffspdkdsimniepailvmhhsmkphpaitatlldfmcriipn  409
DSSP  lhhhhhhhhhhhllllllllllllhhhhhhhhhhhhhhllllhhhhhhhhhhhhhhhhhh


DSSP  -----------------------------------------------------l
Query -----------------------------------------------------r  292
ident
Sbjct fyppleghvrqgvfsslnhivekrvlahlaplfdnpkldkelramlrekfpefc  463
DSSP  llhhhhhhhhhhhhhhhhhhhhlllllllhhhhlllllllhhhhhhhhhhlhhhl
```

No 749: Query=mol1A Sbjct=5i7nA Z-score=2.1

back to top

```
DSSP  ---llllllllllhhhHHHH-HHHHHhHLLL----------------------lHHHHHH
Query ---aapysspptnlrsLRDR-LTQVAeRQGV----------------------vFGRLQR   34
ident                 |  |          |
Sbjct alearvghyyqmdnpyLVGReKVREY-ARAVqdyhpshwdaaaaadlgysgvvaPLTFTS   59
DSSP  lllllllleeellllleELLHhHHHHH-HHHLllllhhhllhhhhhhllllllllLHHHHH


DSSP  HHHHHHHHHHHHLLllllllllleeeehhhhhhhhhllllllllleEEEElllhhhhhhh
Query HVAXIVVAQFAATLtddtgaplllvkggsslelrrgipdsrtskdfATVArrdieliheq   94
ident   |
Sbjct TPAMACNRRMFESV------------------------------vvGYDTylqteevfeq   89
DSSP  HHHHHHLHHHHHLL------------------------------llLLLEeeeeeeeeee


DSSP  hhhhhhlllLEEEEELLLEELLllllllLLEEEEEEEEEL-----LEEEEEEEEEEEEL
Query ladagetgweGFTAIFTAPEEIAapgxpvKPRRFTAKLSYR-----GRAFATVPIEVSSV  149
ident                 |     |           |
Sbjct hrpivagdelHIDVELTSIRRVA------GRDLITVTNTFTdmageRVHTLHTTVVGITA  143
DSSP  llllllllleeEEEEEEEEEEEEL------LEEEEEEEEEEEllllleEEEEEEEEEEEEELH


DSSP  --LLLL-LLLL---EEEE------------------------------------------
Query --EAGN-ADQF---DTLT------------------------------------------  161
ident
Sbjct deISPGtMAAVqkaMMHDvdsyiktvrpagevrvaqdtarnpgtpsfddvkvgdelpvhh  203
DSSP  hhLLLLhHHHHhhhLLLLllllllllllllleellllllllllllhhhlllllllllee


DSSP  -----elhhhhllllleeeeellhhhhHHHHhhhhhllllllllllllhHHHHhhhhh
Query -----sdalglvgvpaavavpcxtipwqIAQKlhavtavleepkandrahDLVDlqlleg  216
ident                                                     |
Sbjct trlsrgdlvnyagvagdanpihwdeeiaKLAG-----------------lPDVI------  240
DSSP  eeelhhhhhhhhhhllllllhhhllhhhhHHLL-----------------lLLLL------


DSSP  hllhhhhhhhhhhhhhhhhhhllllllllllllllhhhhhhhhhllLLLL----------
Query llldadlxptrsaciaifearaqhpwpprvatlphwpliyagalegLDHL----------  266
ident                                                |
Sbjct ----ahgmltmglgagffsawsgdpgavtryavrlsapaivsaaegADIEfggkiksldp  296
DSSP  ----llhhhhhhhhhhhhhhhhhllhhheeeeeeeeellllllllllEEEEeeeeeeeeel


DSSP  ---llllhhhhhhhhhhhhhhhhhhllll
Query ---elartvdaaaqavqrfvaridratkr  292
ident
Sbjct qtrtgivvvtakasgkkifglatmsvrfs  325
DSSP  llleeeeeeeeeeelleeleeeeeeeeell
```

No 750: Query=mol1A Sbjct=1wa5B Z-score=2.1

back to top

```
DSSP  ------------------------------------------------------------
Query ------------------------------------------------------------    0
ident
Sbjct fvpeyrrtelrrrrdtqqvelrkakrdealakrrnfqelpqmtqqlnsddmqeqlsatvk   60
DSSP  lllhhhllllllllllllllllllllllllllllllllhhhhhhhhllllhhhhhhhhhh
```

```
Query ------------------------------------------------------------    0
ident
Sbjct frqilsrehrppidvviqagvvprlvefmrenqpemlqleaawaltniasgtsaqtkvvv  120
DSSP  hhhhllllllllhhhhhhlllhhhhhhlllllllhhhhhhhhhhhhhhllllhhhhhhhh


DSSP  ---LLLLllllllhhHHHHHHHHHHHHHLL-------------------------llhHHH
Query ---AAPYssppptnlrSLRDRLTQVAERQG-------------------------vvfGRL   32
ident    | |         |
Sbjct dadAVPLfiqllytgSVEVKEQAIWALGNvagdstdyrdyvlqcnamepilglfnsnKPS  180
DSSP  hllLHHHhhhhhhhlLHHHHHHHHHHHHHHhhlllhhhhhhhhhlllhhhhhhhhhllLHH


DSSP  HHHHhhhhhhhhhhlllllllllleeeehhhhhhhhhLLLL-------------------
Query QRHVaxivvaqfaatltddtgaplllvkggsslelrrGIPD-------------------   73
ident
Sbjct LIRT----------------------------atwtlSNLCrgkkpqpdwsvvsqalptl  212
DSSP  HHHH----------------------------hhhhhHHHHllllllllhhhhhhhhhhh


DSSP  --lllLLLEeeeelllhhhhhhhhhhhhhhllllleeeeellleelllllllllleeeeee
Query --srtSKDFatvarrdielihеqladagetgwegftaiftapeeiaapgxpvkprrftak  131
ident      | |
Sbjct akliySMDT---------------------------------------------------  221
DSSP  hhhllLLLH---------------------------------------------------


DSSP  eeelleeeeeeeeeeelllllllLLEEeeelhhhhllllllleeeeELLHhHHHHHHHHH
Query lsyrgrafatvpievssveagnadQFDTltsdalglvgvpaavavpCXTIpWQIAQKLHA  191
ident                                                       |
Sbjct --etlvdacwaisylsdgpqeaiqAVID------------------VRIP-KRLVELLSH  260
DSSP  --hhhhhhhhhhhhhhlllhhhhhHHHH------------------LLLH-HHHHHHHHL


DSSP  hhlllllllllLLLH-HHHHHHHHHHH-LLHH-HHHHHHHHHhhhhhhhllllLLLL--L
Query vtavleepkanDRAH-DLVDLQLLEGL-LLDA-DLXPTRSACiaifearaqhpWPPR--V  246
ident                    |                   |                |
Sbjct -----------ESTLvQTPALRAVGNIvTGNDlQTQVVINAG---------vlPALRllL  300
DSSP  -----------LLHHhHHHHHHHHHHHlLLLHhHHHHHHHLL---------hhHHHHhhL


DSSP  LLLllhhhhhhhhhllllllllllHHHHHHHHHHHHHHHHHLLLL--------------
Query ATLphwpliyagaleg1dhlelartVDAAAQAVQRFVARIDRATKR-------------  292
ident                                            |
Sbjct SSP----------------------KENIKKEACWTISNITAGNTEqiqavidanlippl  338
DSSP  LLL----------------------LHHHHHHHHHHHHHHHLLLLHHhhhhhhhlllhhhh


DSSP  ------------------------------------------------------------
Query ------------------------------------------------------------  292
ident
Sbjct vkllevaeyktkkeacwaisnassgglqrpdiirylvsqgcikplcdlleiadnriievt  398
DSSP  hhhhhhllhhhhhhhhhhhhhhhhhlllllhhhhhhhhlllhhhhhhhlllllhhhhhhh


DSSP  ------------------------------------------------------------
Query ------------------------------------------------------------  292
ident
Sbjct ldalenilkmgeadkeararglninenadfiekaggmekifncqqnendkiyekaykiiety  458
DSSP  hhhhhhhhhhhhhhhhhhhlllllhhhhhhhhllhhhhhhhhhhlllhhhhhhhhhhhhh


DSSP  -----
Query -----  292
ident
Sbjct fgeee  463
DSSP  lllll
```

No 751: Query=mol1A Sbjct=4hbdA Z-score=2.1

back to top

```
DSSP  llllllllllhhhhhhhhhhhhhhllllhhhhhhhhhHHHHHHHHHLL----------ll
Query aapysspptnlrslrdrltqvaerqgvvfgrlqrhvaXIVVAQFAATL----------td   50
```

```
DSSP  LLLL---LLEE---EEHH---------hhHHHHHLllllllllleeeeelllhhhhhhhh
Query DTGA---PLLL---VKGG---------ssLELRRGipdsrtskdfatvarrdieliheql   95
ident
Sbjct ERELkvaYTTVlqeWLRLacrsdahpelvRRHLVT-------------------------   60
DSSP  HHHHhhhHHHHhhhHHHHhhlllllhhhhHHHHHH-------------------------
```

```
DSSP  hhhhhhlllleeeeelllee llllllllll eeeeeeeeellee eeeeeeeeeeLLLLlll
```

```
DSSP  hhhhhhlllleeeeeelllleelllllllllleeeeeeeeelleeeeeeeeeeeeLLLLlll
Query adagetgwegftaiftapeeiaapgxpvkprrftaklsyrgrafatvpievssVEAGnad  155
ident                                                         |
Sbjct -------------------------------------framsarlldyvvniaDSNG---   80
DSSP  -------------------------------------hhhhlhhhhhhhhlllLLLL---
```

```
DSSP  lleeeeelhhhhlllllleeeeellhhHHHHHHHHHHhllllllllllllhHHHHHHHHH
Query qfdtltsdalglvgvpaavavpcxtipWQIAQKLHAVtavleepkandrahDLVDLQLLE  215
ident                                                         | |
Sbjct --------------------------nTALHYSVSHA--------------NFPVVQQLL  100
DSSP  --------------------------lLHHHHHHHLL--------------LHHHHHHHH
```

```
DSSP  HHLLhhhhhHHHHHhhhhhhhhllllLLLL-LLLLllhhhhhhhhhlllllllllllHHH
Query GLLLdadlxPTRSAciaifearaqhpWPPR-VATLphwpliyagalegldhlelartVDA  274
ident
Sbjct DSGV----cKVDKQ----nragyspiMLTAlATLK----------------------TQD  130
DSSP  HLLL----lLLLLL----llllllhhHHHHhLLLL----------------------LHH
```

```
DSSP  HHHHHHHHHHHHHHLLL-------------------------------------------
Query AAQAVQRFVARIDRATK-------------------------------------------  291
ident     |           |
Sbjct DIETVLQLFRLGNINAKasqagqtalmlavshgrvdvvkallaceadvnvqdddgstalm  190
DSSP  HHHHHHHHHHHLLLLLLlllllllhhhhhhhlllhhhhhhhhhllllllllllllllhhh
```

```
DSSP  ------------------------------------------------------l
Query ------------------------------------------------------r  292
ident
Sbjct cacehghkeiaglllavpscdisltdrdgstalmvaldagqseiasmlysrmnik  245
DSSP  hhhhllhhhhhhhhllllllllllllllllhhhhhhhhhllhhhhhhhhhhllll
```

No 752: Query=mol1A Sbjct=4i2wA Z-score=2.1

back to top

```
DSSP  ------------------------------------------------------------
Query ------------------------------------------------------------    0
ident
Sbjct vqtaeeirdegnaavkdqdyikadelytealqlttdedkalrpvlyrnraxarlkrddfe   60
DSSP  lllhhhhhhhhhhhhhhllhhhhhhhhhhhhhhlllllhhhlhhhhhhhhhhhhhlllhh
```

```
DSSP  ---------------------------------------------llllllllLLHHHHH
Query ---------------------------------------------aapyssppTNLRSLR   15
ident
Sbjct gaqsdctkalefdgadvkalfrrslareqlgnvgpafqdakealrlspndkgiVEVLQRL  120
DSSP  hhhhhhhhhhllllllhhhhhhhhhhhhhhlllhhhhhhhhhhhhlllllhhhHHHHHHH
```

```
DSSP  HHhhhhhhhllllhhhhhhhhhhhhhhHHHHlllllllllLEEE----------------
Query DRltqvaerqgvvfgrlqrhvaxivvaQFAAtltddtgapLLLV----------------   59
ident                            |
Sbjct VK------------------anndkikQTTS-------laNKVTdxeklafrgeakdteq  155
DSSP  HH------------------hhhllhhHHHH-------hhHHHHhhhhhllllllllhhh
```

```
DSSP  ---ehHHHHHHHHLllllllllleeeeelllhhhhhhhhhhhhhhlllleeeeellleel
Query ---kgGSSLELRRGipdsrtskdfatvarrdieliheqladagetgwegftaiftapeei  116
ident         | | |
Sbjct kxtalNNLLVLCRE----------------------------------------------  169
DSSP  hhhhhHHHHHHHHL----------------------------------------------
```

```
ident
Sbjct -----------------------------------------------------sesgatg  176
DSSP  -----------------------------------------------------lhhhhhh


DSSP  eeLLHHhHHHHHHHHHHLLllllllLLLL-HHHHHHHHHHHHL-LHHHHHHHHHHhhhhh
Query pcXTIPwQIAQKLHAVTAVleepkaNDRA-HDLVDLQLLEGLL-LDADLXPTRSAciaif  234
ident             |                         |          |
Sbjct vwNQGA-LVPFVLNLINDA------SENEeVTVTAIRILDETIkNSVRCXKFLAX-hdpd  228
DSSP  hhHHHL-HHHHHHHHHHLL------LLLHhHHHHHHHHHHHHLlLHHHHHHHHHL-llll


DSSP  hhhlllLLLL--LLLLlllhhhhhhhhhhllllllllllhHHHHHHHHHHHHHHHH-LLL
Query earaqhPWPP--RVATlphwpliyagalegldhlelartvDAAAQAVQRFVARIDR-ATK  291
ident                                                          |
Sbjct gpksvrFVCRlxCKKS------------------------TKDFVDATGILVQRVFnAXA  264
DSSP  hhhhhhHHHLhhHHLL------------------------LHHHHHHHHHHHHHHHhHHL


DSSP  L-----------------------------------------------------------
Query R-----------------------------------------------------------  292
ident
Sbjct Kxdrqkexkpdpevaeankiwiirvllelqexlqdpkvgavqretcidlflknlxhxdgg  324
DSSP  Lllllllllllhhhhhhlhhhhhhhhhhhhhhlllllllhhhhhhhhhhhhhhhllllll


DSSP  ------------------------------------------------------------
Query ------------------------------------------------------------  292
ident
Sbjct iprgwswkfveergllalldvasqipelceypvsaetrqhvaiclqrleedxvfdtkrti  384
DSSP  llllhhhhhhllhhhhhhhhhhllllllllllllllhhhhhhhhhhhhhhllllhhhhhh


DSSP  ------------------------------------------------------------
Query ------------------------------------------------------------  292
ident
Sbjct fkekvdxffnalisrctnddeghkyriklscflitxlqgpvdiginlitndqltpixlex  444
DSSP  hhhhhhhhhhhhhlllllllllhhhhhhhhhhhhhhhhllhhhhhhhhllllhhhhhhhh


DSSP  ------------------------------------------------------------
Query ------------------------------------------------------------  292
ident
Sbjct aasqdhlxqgiaaelivatvskherainxlkvgipvlralydsedptvkvralvglckig  504
DSSP  hhlllhhhhhlllllllllllllllhhhhhhhhhhhhhhhhhlllhhhhhhhhlllllll


DSSP  ------------------------------------------------------------
Query ------------------------------------------------------------  292
ident
Sbjct aeeavislaktckkflletekysvdirryaceglsylsldadvkewivddslllkalvll  564
DSSP  lllllhhhhhhhhhhhhllllllhhhhhhhhhhhhhllllhhhhhhhhhlhhhhhhhhhh


DSSP  ------------------------------------------------------------
Query ------------------------------------------------------------  292
ident
Sbjct akkagalcvytlatiyanlsnafekpkfakhhvpethpkdteeyvekrvralveegavpa  624
DSSP  hhhhhhlhhhhhhhhhhhllllllllllllllllllllllllllhhhhhhhhhhllllhh


DSSP  ------------------------------------------------------------
Query ------------------------------------------------------------  292
ident
Sbjct cvavsktesknaleliarsllafaeyedlrgriiaeggtvlclrltkeasgegkikagha  684
DSSP  hhhllllllhhhhhhhhhhhhllllllllllllllllhhhhhhhhhhhlllhhhhhhhhh


DSSP  ------------------------------------------------------------
Query ------------------------------------------------------------  292
ident
Sbjct iaklgakadpxisfpgqrayevvkplcdllhpdvegkanydslltltnlasvsdsirgri  744
DSSP  hhlllllllhhhlllllhhhhlllhhhlllllllllllllllhhhhhhhllllhhhhhhh


DSSP  ------------------------------------------------------------
Query ------------------------------------------------------------  292
```

```
DSSP  ------------------------------------------------------------
Query ------------------------------------------------------------  292
ident
Sbjct eeerlsrasaagfailtedenacarixdeikswpevfkdiaxhedaetqrrglxgianix  864
DSSP  llllhhhhhhhhhhhhllhhhhhhlhhhlllhhhhhhhhhhlllllhhhhhhhhhlhhhh


DSSP  ----------------------------------------
Query ----------------------------------------  292
ident
Sbjct hssnklcseivssevfrvlvavtklgtinqeragsteqak  904
DSSP  llllhhhhllllhhhhhhllllllllhhhhhhhhhhhlll
```

No 753: Query=mol1A Sbjct=3q0qA Z-score=2.1

back to top

```
DSSP  lllllllllLHHHhHHHHhhhhhhllllHHHHHHhhHHHHHHHH--------------hh
Query aapyssspptNLRSlRDRLtqvaerqgvvFGRLQRhvAXIVVAQF--------------aa   46
ident                  |            |           |
Sbjct grsrlledfRNNR-FPNL----------QLRDLI-gHIVEFSQDqhgsrfiqqkleratp   48
DSSP  lllhhhhhhHLLL-LLLL----------LHHHHL-lLHHHHHHLLhhhhhhhhhhhllllh


DSSP  llllllllleEEEH---------HHHHHHHHHLlllllllleeeeellllhhhhhhhhhh
Query tltddtgapllLVKG---------GSSLELRRGipdsrtskdfatvarrdieliheqlad   97
ident
Sbjct aerqmvfneilQAAYqlmtdvfgnYVIQKFFEF---------------------------   81
DSSP  hhhhhhhhhhhHLHHhhhlllllhHHHHHHHHH---------------------------


DSSP  hhhhlllleeeeeellleellllllllleeeeeeeeeelleeeeeeeeeeeellllllll
Query agetgwegftaiftapeeiaapgxpvkprrftaklsyrgrafatvpievssveagnadqf  157
ident
Sbjct ------------------------------------------------------------   81
DSSP  ------------------------------------------------------------


DSSP  eeeeelhhhhlllllleeeeeelLHHHHHHHHHHHHhllllllllllLLHHHHHHHHHHHH
Query dtltsdalglvgvpaavavpcxTIPWQIAQKLHAVtavleepkandRAHDLVDLQLLEGL  217
ident                            |                          |
Sbjct ---------------gsldqklALATRIRGHVLPL---------alQMYGCRVIQKALES  117
DSSP  ---------------llhhhhhHHHHHHLLLHHHH---------hhLLLHHHHHHHHHHH


DSSP  LLHHHhhHHHHHHhhhhhhhllllLLLLllllllhhhhhhhhhlllllllllllhHHHHH
Query LLDADlxPTRSACiaifearaqhpWPPRvatlphwpliyagalegldhlelartvDAAAQ  277
ident           |                                            |
Sbjct ISSDQ--QVISEM-------vkelDGHV----------------------lkcvkDQNGN  146
DSSP  LLLLL--HHHHHH-------hhllLLLH----------------------hhhhhLLLHH


DSSP  HHHHHHHHhhHLLL----------------------------------------------
Query AVQRFVARidRATK----------------------------------------------  291
ident  |
Sbjct HVVQKCIEcvQPQSlqfiidafkgqvfvlsthpygcrviqrilehctaeqtlpileelhq  206
DSSP  HHHHHHHHhlLHHHhhhhhhhllllhhhhhllllhhhhhhhhhhhllhhhhhhhhhhhhh


DSSP  ------------------------------------------------------------
Query ------------------------------------------------------------  291
ident
Sbjct hteqlvqdqygnyviqhvlehgrpedkskivseirgkvlalsqhkfasnvvekcvthasr  266
DSSP  lhhhhlllllhhhhhhhhhhhllhhhhhhhhhhhlllhhhhhllllhhhhhhhhhhhllh


DSSP  ------------------------------------------------------------
Query ------------------------------------------------------------  291
ident
Sbjct aerallidevccqndgphsalytmmkdqyanyvvqkmidmaepaqrkiimhkirphittl  326
DSSP  hhhhhhhhhhhllllllllhhhhhhllllhhhhhhhhhhhhllhhhhhhhhhhhhhhhhh
```

```
ident
Sbjct rkytygkhilaklekyy  343
DSSP  hhlhhhllhhhhhhlll
```

No 754: Query=mol1A Sbjct=1f7uA Z-score=2.1

back to top

```
DSSP  llllllllLLHHHHHHHHHH---------hhhhlLLLHHHHHHHHHHHHHHHHHHlllll
Query aapyssppTNLRSLRDRLTQ---------vaerqGVVFGRLQRHVAXIVVAQFAAtltdd   51
ident                  |                 |    | |
Sbjct --------ASTANMISQLKKlsiaepavakdshpDVNIVDLMRNYISQELSKISG-----   47
DSSP  --------LHHHHHHHHHHHlllllhhhhlllllLLLHHHHHHHHHHHHHHHHHL-----

DSSP  llllleeeehhhhhhhhhlllllllLLLE----------------EEEEL-LLHHHHHHH
Query tgaplllvkggsslelrrgipdsrtSKDF----------------ATVAR-RDIELIHEQ   94
ident                                                            |
Sbjct -------------------vdssliFPALewtntmergdllipipRLRIKgANPKDLAVQ   88
DSSP  -------------------llhhhhHHHLeellllhhhlleeeehhHHLLLlLLHHHHHHH

DSSP  HHHHHhhLLLL-eeEEELLLeellllllllleEEEEEEE--------------------
Query LADAGetGWEG-ftAIFTAPeeiaapgxpvkprRFTAKLS--------------------  133
ident  |                                |
Sbjct WAEKF--PCGDfleKVEANG-------------PFIQFFFnpqflaklvipdiltrkedy  133
DSSP  HHHLL--LLLLleeEEEEEL-------------LEEEEEElhhhhhhhhhhhhhhhhhhl

DSSP  ------------------------------------------------------------
Query ------------------------------------------------------------  133
ident
Sbjct gscklvenkkviiefsspniakpfhaghlrstiiggflanlyeklgwevirmnylgdwgk  193
DSSP  lllllllleeeeellllllllllhhhhhhhhhhhhhhhhhhllleeeeeeeeellllh

DSSP  ---------------------------------------------------------ell
Query ---------------------------------------------------------yrg  136
ident
Sbjct qfgllavgferygneealvkdpihhlfdvyvrinkdieeegdsipleqstngkareyfkr  253
DSSP  hhhhhhhhhhhhllhhhhhhlhhhhhhhhhhhhhhhhhhlllllllhhhhllhhhhhhhhh

DSSP  eeeeeeeeeeeellllllllleeeeelhhhhllllllleeeeellhhhhhhhhhhhhhlll
Query rafatvpievssveagnadqfdtltsdalglvgvpaavavpcxtipwqiaqklhavtavl  196
ident
Sbjct medgdeealkiwkrfrefsiekyidtyarlnikydvysgesqvskesmlkaidlfkekgl  313
DSSP  hhhllhhhhhhhhhhhhhhhhhhhhhhhhhlllllleeeehhhllhhhhhhhhhhhhhlll

DSSP  lllllllllhhhhhhhhhhhhllhhhhhhhhhhhhhhhhhhhlllllllLLLL---------
Query eepkandrahdlvdlqllegllldadlxptrsaciaifearaqhpwpPRVA---------  247
ident
Sbjct thedkgavlidltkfnkklgkaivqksdgttlyltrdvgaamdryekYHFDkmiyviasq  373
DSSP  eeeelleeeeehhhllllllleeeeellllllllhhhhhhhhhhhhhhHLLLeeeeellhh

DSSP  ---------------------LLLL-----------------------------------
Query ---------------------TLPH-----------------------------------  251
ident                       | |
Sbjct qdlhaaqffeilkqmgfewakDLQHvnfgmvqgmstrkgtvvfldnileetkekmhevmk  433
DSSP  hhhhhhhhhhhhhhlllhhhhHEEEellllleellllhhhllllllhhhhhhhhhhhhhhhh

DSSP  ------------------------------------------------------------
Query ------------------------------------------------------------  251
ident
Sbjct knenkyaqiehpeevadlvgisavmiqdmqgkrinnyefkwermlsfegdtgpylqyahs  493
DSSP  llhhhhlllllhhhhhhhhhhhhhhhhhhhllllllleellhhhhhhlllllllhhhhhhhhh

DSSP  ----------------------------------hhhhhhhhHLLL-LLLLLLL------
Query ----------------------------------wpliyagaLEGL-DHLELAR------  270
ident
```

```
DSSP  --------------------------------lhhhhhhhhhhhhhhhhhllll
Query --------------------------------tvdaaaqavqrfvaridratkr  292
ident
Sbjct fklthqvsscydvlwvagqteelatarlalygaarqvlyngmrllgltpvermr  607
DSSP  hhhhhhhhhhhhhlllllllhhhhhhhhhhhhhhhhhhhhhhhhhlllllllll
```

No 755: Query=mol1A Sbjct=5emwA Z-score=2.1

back to top

```
DSSP  lllllllllhhhhhhhhhhhhhhllllhhhhHHHHHhhhhhHHHHlLLLL--------L
Query aapyssptnlrslrdrltqvaerqgvvfgrlQRHVAxivvaQFAAtLTDD--------T   52
ident
Sbjct ------------------------------tiASSRL---rlLEYS-AFMEvqrdpdtyS   26
DSSP  ------------------------------llLLLLL---eeEEEE-EEEEeeeelleeE


DSSP  LLLLLEEeehhhhhhhhhllLLLLLL----------------------------LLEEEEE
Query GAPLLLvkggsslelrrgiPDSRTS----------------------------KDFATVA   84
ident                    |                                       |
Sbjct KHLFVH-----------igPPLEAVdvrqiydkfpekkgglkelyekgppnafFLVKFWA   75
DSSP  EEEEEE-----------elLLLEEEehhhlhhhlllllllhhhhhhhllhhheEEEEEEE


DSSP  LL------lhhhhhhhhhhhhhllLLEEEEEELLLEE-----------------------
Query RR------dieliheqladagetgwEGFTAIFTAPEE-----------------------  115
ident
Sbjct DLnstiqegpgafygvssqyssadsMTISVSTKVCSFgkqvvekveteyarlengrfvyr  135
DSSP  LLllllllllleeeeeeeeeellLEEEEEEEEEELleeeeeeeeeeeleeelleeeee


DSSP  ------------------lllLLLLLL----lEEEEEEEEEL--LEEEEEEEEEEEELLL
Query ------------------iaaPGXPVK----pRRFTAKLSYR--GRAFATVPIEVSSVEA  151
ident                                          |
Sbjct ihrspmxeyminfihklkhlpEKYMMNsvlenFTILQVVTSRdsQETLLVIAFVFEVSTS  195
DSSP  eeeeellhhhhhhhhhhlllLHHHHHhhhllEEEEEEEEEELllLLEEEEEEEEEEEELLL


DSSP  LLLllleeeeelhhhhllllllleeeeellhhhhhhhhhhhhhllllllllllllhhhhhh
Query GNAdqfdtltsdalglvgvpaavavpcxtipwqiaqklhavtavleepkandrahdlvdl  211
ident
Sbjct EHG---------------------------------------------------------  198
DSSP  LLL---------------------------------------------------------


DSSP  hhhhhhllhhhhhhhhhhhhhhhhhhhhllllllllllllllhhhhhhhhhlllllllll
Query qlleglllldadlxptrsaciaifearaqhpwpprvatlphwpliyagalegldhlelart  271
ident
Sbjct ------------------------------------------------------------  198
DSSP  ------------------------------------------------------------


DSSP  hhhhhhhhhhhhhhhhhllll
Query vdaaaqavqrfvaridratkr  292
ident
Sbjct -----------aqhhvyklvk  208
DSSP  -----------leeeeeeeel
```

No 756: Query=mol1A Sbjct=3hbzA Z-score=2.1

back to top

```
DSSP  lLLLLLLLllhhhhhhhhhhhhhhllllhhhhhhhhhhhHHHHhhHLLLllllLLLE-EE
Query aAPYSSPPtnlrslrdrltqvaerqgvvfgrlqrhvaxiVVAQfaATLTddtgAPLL-LV   59
ident       |                                        |
Sbjct -GREEAPN----------------------------aeaDILS--CRLP----GVVXtTS   25
DSSP  -LLLLLLL----------------------------lllLEEE--EELL----LLLLlLL


DSSP  EHhhhhhhhhlllllllllLEEEEEL--LLHHHhHHHHhhhhhhLLLLEEEEEllleell
```

```
DSSP  LE-------------elllEEEEELLllLLLLL-LLLE----eeELLLLEEEL------l


DSSP  lllllllLEEEEEEEEEL-LEEEEEEEEEEEELLLlllllleeeeelhhhhlllllleee
Query apgxpvkPRRFTAKLSYR-GRAFATVPIEVSSVEAgnadqfdtltsdalglvgvpaavav  176
ident                    |         |   |
Sbjct sgtardfHSPQQYTVTAAdGFWKKKYTVSVIDTEL-------------------------   96
DSSP  lllllllLLLEEEEEEELLlLLLEEEEEEEEEELLLL-------------------------


DSSP  eellhhhhhhhhhhhhHLLLlllllLLLL-------------------------------
Query pcxtipwqiaqklhavTAVLeepkaNDRA-------------------------------  205
ident                           |
Sbjct --------------atIYNF-----EDTLggqkyyifveregekvvxewasgnagyaxtg  137
DSSP  --------------llEELL-----LLLLlllllllleeeelleeeeellllhhhhhlll


DSSP  ------------------------------------------hhHHHH------------
Query ------------------------------------------hdLVDL------------  211
ident                                             |
Sbjct vpktaddyptfqfangktgkclslvtrstgffgsixgxpiaagnLFIGsfdvgnaxsnpl  197
DSSP  llllhhhlleeeellllllleeeeeleelhhhhhhllllеelleEELEeelhhhllllhh


DSSP  ----------------------------------------------------------hh
Query ----------------------------------------------------------ql  213
ident
Sbjct katkfglpfrhiptylagyykykagdqfteggkpvsgkrdicdiyaixyetsesvptldg  257
DSSP  hheeeleellllleeeeeeeeeeellllleeeelleelllllllleeeeeeeellllllll


DSSP  hhhhlLHHHHH--------hhhhhhhhhhhhhlllllllllllllhhhhhhhhhhllLLL
Query legllLDADLX--------ptrsaciaifearaqhpwpprvatlphwpliyagalegLDH  265
ident
Sbjct tnaftSPNLVSiariddaketdewtyfklpfhxlsgkyidkekltagkynvaivftsSLE  317
DSSP  lllllLLLEEEeeellllllеeeeeeeeeeleelllllllhhhhhhlleeeeeeeelLLL


DSSP  LL------llLLHHhhhhhhhhhhhhhhhllll
Query LE------laRTVDaaaqavqrfvaridratkr  292
ident
Sbjct GDhfngaigsTLLI---------develiyrse  341
DSSP  HHhleeelllEEEE---------eeeeeeelll
```

No 757: Query=mol1A Sbjct=2hljA Z-score=2.1

back to top

```
DSSP  llllllllllhhhHHHHhhHHHHH-llllHHHHHHHHHHHHHHHHHHLLlllllLLLEEE
Query aapysspptnlrsLRDRltQVAER-qgvvFGRLQRHVAXIVVAQFAATLtddtgAPLLLV   59
ident
Sbjct ----gxpalityrTTVQ--EDWVDynghlRDAFYLLIFSYATDALXDRIgldadSRGQSG   54
DSSP  ----llllleeeeEELL--HHHLLlllllLHHHHHHHHHHHHHHHHHHHlllllLLLLLL


DSSP  EhhhhhhhhhllllllllleeeeelllhhhhhhhhhhhhhhhlllLEEEEELLLEEllll
Query KggsslelrrgipdsrtskdfatvarrdieliheqladagetgweGFTAIFTAPEEiaap  119
ident
Sbjct N-----------------------slftleahinylhevklgtevWVQTQILGFDR----   87
DSSP  L-----------------------leeeeeeeeeellllllllееEEEEEEEEELL----


DSSP  llllllEEEEEEEEEL------LEEEEEEEEEEEELlllllllleeeeelhhhhllllll
Query gxpvkpRRFTAKLSYR------GRAFATVPIEVSSVeagnadqfdtltsdalglvgvpaa  173
ident        |     |          |
Sbjct ------KRLHVYHSLHragfdeVLAASEQXLLHVDL------------------------  117
DSSP  ------LEEEEEEEEEelleeeEEEEEEEEEEELLL------------------------


DSSP  eeeeellhhhhhhhhhhhhhhlllllllllllhhhhhhhhhhhhllhhhhhhhhhhhhhh
Query vavpcxtipwqiaqklhavtavleepkandrahdlvdlqllegllldadlxptrsaciai  233
ident
Sbjct -----------------------------------------------qsapfghttvcrl  130
DSSP  -----------------------------------------------llllllhhhhhhh
```

```
Query fearaqhpwpprvatlphwpLIYAgalegldhlelartvdaaaqavqrfvaridratkr  292
ident                        |
Sbjct nhlveqqegaqapqyxgrtiKLPA-----------------------------------  154
DSSP  hhhhhlllllllllllllllLLLL-----------------------------------
```

No 758: Query=mol1A Sbjct=3b18A Z-score=2.1

back to top

```
DSSP  ---------------------------------llllllllllhhhhhhhHHHHHhlll
Query ---------------------------------aapysspptnlrslrdrlTQVAErqgv   27
ident
Sbjct sgtvpiaeellarvlepysckgcrylidaqysatedsvlaygnftigesayIRSTG----   56
DSSP  llleelllllhhhhlhhhhllllllleeeeeeeeeelleeeeeeeellllllLLLLL----


DSSP  lhhhhHHHHHHHHHHHHH-HHLLLL-------------llllllleEEEHhhhhhhhllll
Query vfgrlQRHVAXIVVAQF-AATLTD-------------dtgapllLVKGgsslelrrgipd   73
ident                |
Sbjct ---hfNAVELILCFNQLaYSAFAPavlneeirvlrgwsiddycqHQLS------------  101
DSSP  ---llLLHHHHHHHHHHhHHHHHHhhhlllllllllllhhhhhhLHHH------------


DSSP  llllleeeeelllhhhhhhhhhhhhhhllLLEEEEEELLLEELLLLlllLLLEEEEEEEEE
Query srtskdfatvarrdieliheqladagetgwEGFTAIFTAPEEIAAPgxpVKPRRFTAKLS  133
ident                                           |
Sbjct -----------smlirkassrfrkplnpqkFSARLLCRDLQVIERT---WRYLKVPCVIE  147
DSSP  -----------heeeeelleeellllllllEEEEEEEELLEEELLL---LLEEEEEEEEE


DSSP  EL---lEEEEEEEEEEEELllllllllleeeeelhhhhllllllleeeeellhhhhhhhhh
Query YR---gRAFATVPIEVSSVeagnadqfdtltsdalglvgvpaavavpcxtipwqiaqklh  190
ident        |
Sbjct FWdnggAASGEIELAALNI-----------------------------------------  166
DSSP  EEllllEEEEEEEEEEELLL-----------------------------------------


DSSP  hhhlllllllllllllhhhhhhhhhhhllhhhhhhhhhhhhhhhhhhhllllllllllll
Query avtavleepkandrahdlvdlqlleglllddadlxptrsaciaifearaqhpwpprvatlp  250
ident
Sbjct ------------------------------------------------------------  166
DSSP  ------------------------------------------------------------


DSSP  lhhhhhhhhlllllllllllhhhhhhhhhhhhhhhhhhllll
Query hwpliyagalegldhlelartvdaaaqavqrfvaridratkr  292
ident
Sbjct -----------------------------------------p  167
DSSP  -----------------------------------------l
```

No 759: Query=mol1A Sbjct=1kgzB Z-score=2.1

back to top

```
DSSP  llllllllllhhhhhhhhhhhhhhllllhhhhhhhhhhhhhhhhhhhhlllllllllleeEE
Query aapysspptnlrslrdrltqvaerqgvvfgrlqrhvaxivvaqfaatltddtgapllLVK   60
ident
Sbjct --------------------------------------------------------thQP    4
DSSP  --------------------------------------------------------llHH


DSSP  HHHHHHhhhllllllllllleeeeellllhhhhhhhhhhhhhhllllleeeeellleelllll
Query GGSSLElrrgipdsrtskdfatvarrdieliheqladagetgwegftaiftapeeiaapg  120
ident     |
Sbjct ILEKLF------------------------------------------------------   10
DSSP  HHHHHL------------------------------------------------------


DSSP  lllllleeeeeeeeelleeeeeeeeeeeellllllllllleeeeelhhhhlllllllleeeeell
Query xpvkprrftaklsyrgrafatvpievssveagnadqfdtltsdalglvgvpaavavpcxt  180
ident
```

```
DSSP  hHHHHHHHHHHHHllllllllllLLHH-HHHHHHHHHHLlhhhHHHHHHHHHHHHHHHL-
Query iPWQIAQKLHAVTavleepkandRAHD-LVDLQLLEGLLldadLXPTRSACIAIFEARA-  238
ident    |                           |                        | |
Sbjct eSHQLFAAIVRGE---------lEDSQlAAALISMKMRG---eRPEEIAGAASALLADAq   66
DSSP  hHHHHHHHHLLLL---------lLHHHhHHHHHHHHHHL---lLHHHHHHHHHHHHHHLl


DSSP  ------------------------------------------------------------
Query ------------------------------------------------------------  238
ident 
Sbjct pfprpdydfadivgtggdgtnsinistasafvaascgakvakhgnrsvlagscdllqafg  126
DSSP  lllllllleeeeeellllllllllhhhhhhhhhhhlllleeeeellllllhhhhhhhhhl


DSSP  ---------------------------LLLL-lLLLLllllhhhhhhhhhllllllllll
Query ---------------------------QHPW-pPRVAtlphwpliyagalegldhlelar  270
ident 
Sbjct irldmsaedsrqalddlnvcflfapqyHTGFrhAMPV-----------------------  163
DSSP  lllllhhhhhhhhhhhhleeeeehhhhLHHHhhHHHH-----------------------


DSSP  lhHHHHHHhhHHHHHHHH---------------------LLLL-----------------
Query tvDAAAQAvqRFVARIDR---------------------ATKR-----------------  292
ident 
Sbjct -rQQLKTR--TIFNVLGPlinparppkaligvyspelvlPIAQalkvlgyknaavvhggg  220
DSSP  -hHHHLLL--LLHHHHHHhllllllleeeeelllhhhhhHHHHhhhhlllleeeeeelll


DSSP  ------------------------------------------------------------
Query ------------------------------------------------------------  292
ident 
Sbjct mdevaihtptqvaelnngeiesyqlspqdfglqsyslnalqggtpeenrdilarllqgkg  280
DSSP  lllllllleeeeeeelleeeeeeeelhhhhlllllllhhhllllhhhhhhhhhhhhllll


DSSP  --------------------------------------------------
Query --------------------------------------------------  292
ident 
Sbjct daaharqvaanvalllklfgqdnlrhnaqlaletirsgtafervtalaar  330
DSSP  lhhhhhhhhhhhhhhhhhhllllhhhhhhhhhhhhhllhhhhhhhhhhll
```

No 760: Query=mol1A Sbjct=3l7tA Z-score=2.1

back to top

```
DSSP  llllllllllhhhhhhhhhhhhhhllLLHHhhhhhhhhhhhhhhhhllllllllllleeee
Query aapyssptnlrslrdrltqvaerqgVVFGrlqrhvaxivvaqfaatltddtgaplllvk   60
ident 
Sbjct --------------------------KLKA------------------------------    4
DSSP  --------------------------LLLE------------------------------


DSSP  hhhhhhhhlllllllllLLEEEEElllHHHHHHHH-HHHHhhlllleeEEELLLEELLLl
Query ggsslelrrgipdsrtsKDFATVArrdIELIHEQL-ADAGetgwegftAIFTAPEEIAAp  119
ident                                 |      |
Sbjct --------------vhhVALIVSD---YDKSYEFYvNQLG--------FEVIRENHRPK-   38
DSSP  --------------eeeEEEELLL---HHHHHHHHhHLLL--------LEEEEEEEELL-


DSSP  llllLLEEEEEEEEelleeEEEEEEEEEEL--lllllllLEEE--------eelhhhhLL
Query gxpvKPRRFTAKLSyrgraFATVPIEVSSV--eagnadqFDTL--------tsdalglVG  169
ident            |            |
Sbjct ---rHDYKLDLKCG-----DIELEIFGNKLtdsnycappERISwpreacglrhlafyvED   90
DSSP  ---lLEEEEEEEEL-----LEEEEEEELLLlllllllllLLLLlllllleeeeeeeelLL


DSSP  LL-------------------------LLEEEeellhhhhhhhhhhhhhlllllllllll
Query VP-------------------------AAVAVpcxtipwqiaqklhavtavleepkandr  204
ident |                          |
Sbjct VEasrqelialgirveevryddytgkkMAFFF----------------------------  122
DSSP  HHhhhhhhhhhllllllleellllllleEEEEE---------------------------
```

```
Sbjct ------------------------------------------------------------  122
DSSP  ------------------------------------------------------------


DSSP  lllllllhhhhhhhhhhhhhhhhhllll
Query hlelartvdaaaqavqrfvaridratkr  292
ident
Sbjct -----------------dpdglplelhe  133
DSSP  -----------------llllleeeeel
```

No 761: Query=mol1A Sbjct=4tnmA Z-score=2.1

back to top

```
DSSP  llllllllllhhhhhhhhhhhhhHLLLLhhhhHHHhhhhhhhhhhhlllllllllleeee
Query aapyssppptnlrslrdrltqvaeRQGVVfgrlQRHVaxivvaqfaatltddtgaplllvk   60
ident
Sbjct ----------------------pAMVAG--iwSQLE------------------------   12
DSSP  ----------------------lLLLLL--llLLHH------------------------


DSSP  hhhhhhhhhLLLL--------------------------llLLLLeeeeelllhhhhhhh
Query ggsslelrrGIPD--------------------------srTSKDfatvarrdieliheq   94
ident                                            |
Sbjct ----atnllRKLLsieqnppinevvqsgvvprvvkflsrddFPKL---------------   53
DSSP  ----hhhhhHHHHllllllllhhhhhhllhhhhhhhhllllLHHH---------------


DSSP  hhhhhhhlllleeeeellleellllllllllleeeeeeeeelleeeeeeeeeeeeelllll
Query ladagetgwegftaiftapeeiaapgxpvkprrftaklsyrgrafatvpievssveagna  154
ident
Sbjct ----------------------------------------qfeaawaltniasgtsentn   73
DSSP  ----------------------------------------hhhhhhhhhhhhlllhhhhh


DSSP  LLLEeeeelhhhhlllllleeeeELLHhHHHHHHHhhhhlllllllllLLLHHHHHHHHH
Query DQFDtltsdalglvgvpaavavpCXTIpWQIAQKLhavtavleepkanDRAHDLVDLQLL  214
ident                                 | |
Sbjct VIIE-------------------SGAV-PIFIQLL-----------ssASEDVREQAVWA  102
DSSP  HHHH-------------------LLLH-HHHHHHH-----------hlLLHHHHHHHHHH


DSSP  HhhllhHHHHHHHHHHHHHhhhhllllllLLLLLLLlhhhhhhhhllllllllllllHHH
Query EgllldADLXPTRSACIAIfearaqhpwpPRVATLPhwpliyagalegldhlelartVDA  274
ident
Sbjct LgnvagDSPKCRDLVLSYG-----amtplLSQFNEN--------------------tKLS  137
DSSP  HhhhhhHLHHHHHHHHHLL-----lhhhhHLLLLLL--------------------lLHH


DSSP  HHHHHHHHHHHHHH---LLLL---------------------------------------
Query AAQAVQRFVARIDR---ATKR---------------------------------------  292
ident              |
Sbjct MLRNATWTLSNFCRgkpPPAFeqtqpalpvlerlvqsmdeevltdacwalsylsdnsndk  197
DSSP  HHHHHHHHHHHHHLlllLLLHhhhllhhhhhhhhlllllhhhhhhhhhhhhhlllllhhh


DSSP  ------------------------------------------------------------
Query ------------------------------------------------------------  292
ident
Sbjct iqavieagvvprliqllghsspsvlipalrtignivtgddlqtqmvldqqalpcllnllk  257
DSSP  hhhhhhhllhhhhhhhlllllhhhhhhhhhhhhhhhlllhhhhhhhhllllhhhhhhhhh


DSSP  ------------------------------------------------------------
Query ------------------------------------------------------------  292
ident
Sbjct nnykksikkeacwtisnitaqiqavidagiiqslvwvlqsaefevkkeaawgisnatsgh  317
DSSP  llllhhhhhhhhhhhhhhlllhhhhhhllhhhhhhhhhhlllhhhhhhhhhhhhhhhhll


DSSP  ------------------------------------------------------------
Query ------------------------------------------------------------  292
ident
Sbjct dqikfmvsqgcikplcdlltcpdlkvvtvclealenilvvgeaeknlgednlyaqmidea  377
```

```
DSSP  ------------------------------
Query ------------------------------  292
ident
Sbjct eglekienlqshdnndiydkavkiletfwt  407
DSSP  lhhhhhhhhhhlllhhhhhhhhhhhhhhll
```

No 762: Query=mol1A Sbjct=3s4kA Z-score=2.1

back to top

```
DSSP  -----------lllllllllhhhHHHHHHhhhhhlLLLHhhHHHHHHHHHHHHHhhlll
Query -----------aapysspptnlrsLRDRLTqvaerqGVVFgrLQRHVAXIVVAQFaatlt   49
ident                          |  |                 |
Sbjct vpfdselglqftelgpdgaraqldVRPKLL-----qLTGV--VHGGVYCAMIESI-----   48
DSSP  lhhhhhhlleeeeeelleeeeeeeELHHHL-----lLLLL--LLHHHHHHHHHHH-----


DSSP  lllllleeeehhhhhhhhhllllllllleeeeeLLLHhhhhhhhhhhhhhlllLEEEE
Query ddtgaplllvkggsslelrrgipdsrtskdfatvaRRDIeliheqladagetgweGFTAI  109
ident
Sbjct ----------------------asmaafawlnsegGSVV-gvnnntdfvrsissgMVYGT   85
DSSP  ----------------------hhhhhhhhllllLEEE-eeeeeeeellllleEEEEE


DSSP  ELLLEELllllllLLLEEEEEEEEEL-LEEEEEEEEEEEELLLLlllllleeeeelhhhhl
Query FTAPEEIaapgxpVKPRRFTAKLSYR-GRAFATVPIEVSSVEAGnadqfdtltsdalglv  168
ident                             |  |         ||
Sbjct AEPLHRG------RRQQLWLVTITDDtDRVVARGQVRLQNLEAR----------------  123
DSSP  EEEEEEL------LLEEEEEEEEELLlLLEEEEEEEEEEEEELLL----------------


DSSP  lllllleeeeellhhhhhhhhhhhhhlllllllllllhhhhhhhhhhhhllhhhhhhhhh
Query gvpaavavpcxtipwqiaqklhavtavleepkandrahdlvdlqlleglllldadlxptrs  228
ident
Sbjct ------------------------------------------------------------  123
DSSP  ------------------------------------------------------------


DSSP  hhhhhhhhhllllllllllllllhhhhhhhhhllllllllllhhhhhhhhhhhhhhhh
Query aciaifearaqhpwpprvatlphwpliyagalegldhlelartvdaaaqavqrfvaridr  288
ident
Sbjct ------------------------------------------------------------  123
DSSP  ------------------------------------------------------------


DSSP  llll
Query atkr  292
ident
Sbjct ---p  124
DSSP  ---l
```

No 763: Query=mol1A Sbjct=5dqqA Z-score=2.1

back to top

```
DSSP  lllllllllhhhhhhhhhhhhhhllllhhhhhhhhhhhhhhhhhhhlllllllllleeee
Query aapysspptnlrslrdrltqvaerqgvvfgrlqrhvaxivvaqfaatltddtgaplllvk   60
ident
Sbjct ------------------------------------------------------------    0
DSSP  ------------------------------------------------------------


DSSP  hhhhhhhhhllllllllleeeeelllhhhhhhhhhhhhhhlllleeeeellleellllll
Query ggsslelrrgipdsrtskdfatvarrdieliheqladagetgwegftaiftapeeiaapg  120
ident
Sbjct ------------------------------------------------------------    0
DSSP  ------------------------------------------------------------


DSSP  llllleeeeeeeeelleeeeeeeeeeeellllllllleeeeelhhhhllllllleeeeELL
Query xpvkprrftaklsyrgrafatvpievssveagnadqfdtltsdalglvgvpaavavpCXT  180
```

```
DSSP  HHHHHHHHHHHHHHLlllllllLLLH----------------------------------
Query IPWQIAQKLHAVTAvleepkanDRAH----------------------------------  206
ident
Sbjct KAAALVDLAEDGIG------lpVEILdqssfsaryyfiftrldliwslnyfallflnffe   57
DSSP  HHHHHHHHHHHHHH------llLLLLlllllllhhhhhhhhhhlhhhhhhhhhhhhhhhh


DSSP  ------------------------------------------------------------
Query ------------------------------------------------------------  206
ident
Sbjct qplwceknpkpsckdrdyyylgelpyltnaesiiyevitlaillvhtffpisyegsrifw  117
DSSP  hhhllllllllllllllllllllllllhhhhhhhhhhhhhhhhhhhhhhhhhhllhhhhh


DSSP  ------------------------------------------------------------
Query ------------------------------------------------------------  206
ident
Sbjct tsrlnlvkvacvvilfvdvlvdflyfriapyvrviifilsirelrdtlvllsgmlgtyln  177
DSSP  hlhhhhhhhhhhhhhhhhhhhhhhlllllhhhhhhhhhlllhhhhhhhhhhhhhhhhhhh


DSSP  ------------------------------------------------------------
Query ------------------------------------------------------------  206
ident
Sbjct ilalwmlfllfaswiafvmfedtqqgltvftsygatlyqmfilfttsnnpdvwipaykss  237
DSSP  hhhhhhhhhhhhhhhhhhhllllhhhllllllhhhhhhhhhhhhhlllhhhhlhhhhhhl


DSSP  --------------------------------------------HHHHHHHHHHHL----
Query --------------------------------------------DLVDLQLLEGLL----  218
ident                                                 |    ||
Sbjct rwssvffvlyvligvyfvtnlilavvydsfkeqlakqvsgmdqmKRRMLEKAFGLIdsdk  297
DSSP  llhhhhhhhhhhhhhhlhhhhhhhhhhhhhhhhhhhhhhhhhhhHHHHHHHHHHHHllll


DSSP  ----lHHHHHHHHHHHHHHHHHhllllllllllllllhhhhhhhhhllllllllllllhhh
Query ----lDADLXPTRSACIAIFEAraqhpwpprvatlphwpliyagalegldhlelartvda  274
ident
Sbjct ngeidKNQCIKLFEQLTNYRTL--------------pkiskeefglifdelddtrdfkin  343
DSSP  lleeeHHHHHHHHHHHHHHLLL--------------llllllhhhhhhhhhhllllleee


DSSP  hhHHHHHHHH--------------------------------------------------
Query aaQAVQRFVA--------------------------------------------------  284
ident          |
Sbjct kdEFADLCQAialrfqkeevpslqiyhsalsqqlrafvrspnfgyaisfiliinfiavvv  403
DSSP  hhHHHHHHHHhhhlllllllllllllllhhhhhhhhhhhllhhhhhhhhhhhhhhhhhhh


DSSP  ------------------------------------------------------------
Query ------------------------------------------------------------  284
ident
Sbjct ettldieessaqkpwqvaefvfgwiyvlemalkiytygfenywreganrfdflvtwvivi  463
DSSP  hhhhhhhllllhhhhhhhhhhhhhhhhhhhhhhhhhhlhhhhhhlhhhhhhhhhhhhhhh


DSSP  ------------------------------------------------------------
Query ------------------------------------------------------------  284
ident
Sbjct getatfitpfsngewiryllllarmlrlirllmnvqryrafiatfitlipslmpylgtifc  523
DSSP  hhhhhhhlllhhhhhhhhhhhhhhhhhhhhhhhllllhhhhhhhhhhhhhhhhhhhhhhh


DSSP  ------------------------------------------------------------
Query ------------------------------------------------------------  284
ident
Sbjct vlciycsigvqvfgglgnkklfetelaeddyllfnfndypngmvtlfnllvmgnwqvwme  583
DSSP  hhhhhhhhhhhhhllllhhhhhhhhhllhhhhhlllllhhhhhhhhhhhhhllllhhhhh


DSSP  ----------------------------------------------------------hh
Query ----------------------------------------------------------ri  286
ident
Sbjct sykdltgtwwsityfvsfyvitillllnlvvafvleafftelrdleeeekcqgqdsqekrn  643
DSSP  hhhhhhllllhhhhhhhhhhhhhlllhhhhhhhhhhhhhhhhhhhhhhhllllllllllll
```

```
Query dratkr  292
ident
Sbjct rrrsag  649
DSSP  llllll
```

No 764: Query=mol1A Sbjct=4n6hA Z-score=2.1

back to top

```
DSSP  lllllllllhhhhhhhhhhhhhhllllhhhhhhhhhhhhhhhhhlllllllllleeee
Query aapyssppptnlrslrdrltqvaerqgvvfgrlqrhvaxivvaqfaatltddtgaplllvk   60
ident
Sbjct ------------------------------------------------------------    0
DSSP  ------------------------------------------------------------


DSSP  hhhhhhhhhlllllllllleeeeeelllhhhhhhhhhhhhhhlllleeeeellleellll
Query ggsslelrrgipdsrtskdfatvarrdieliheqladagetgwegftaiftapeeiaapg  120
ident
Sbjct ------------------------------------------------------------    0
DSSP  ------------------------------------------------------------


DSSP  lllllleeeeeeeeelleeeeeeeeeeeellllllllleeeeelhhhhllllllleeeeeLL
Query xpvkprrftaklsyrgrafatvpievssveagnadqfdtltsdalglvgvpaavavpcXT  180
ident
Sbjct --------------------------------------------------------dlED    4
DSSP  --------------------------------------------------------lhHH


DSSP  HHHHHHHHHHHHHHLLLllllLLLL-LHHHHHHHHHHHHHLL---------hhhhhhhhhh
Query IPWQIAQKLHAVTAVLeepkANDR-AHDLVDLQLLEGLLL---------dadlxptrsac  230
ident          |                   |
Sbjct NWETLNDNLKVIEKAD----NAAQvKDALTKMRAAALDAQkatppkledkspdspemkdf   60
DSSP  HHHHHHHHHHHHHHHLL----LHHHhHHHHHHHHHHHHHHHllllhhhlllllllhhhhhh


DSSP  hhhhhhhlllllllllLLLLHhhhhhhhhllllllllllhhhhhhhHHHHHHHHHHL-
Query iaifearaqhpwpprvaTLPHWpliyagalegldhlelartvdaaaqaVQRFVARIDRA-  289
ident
Sbjct rhgfdilvgqiddalklANEGK-------------------vkeaqaaAEQLKTTRNAYi  101
DSSP  hhhhhhhhhhhhhhhhhHHLLL-------------------hhhhhhhHHHHHHHHHHHl


DSSP  ------------------------------------------------------------
Query ------------------------------------------------------------  289
ident
Sbjct qkylgspgarsasslalaiaitalysavcavgllgnvlvmfgivrytkmktatniyifnl  161
DSSP  hhhhllllhhhhhhhhhhhhhhhhhhhhhhhhhhhhhhhhhhhhhlhhhllhhhhhhhhh


DSSP  ------------------------------------------------------------
Query ------------------------------------------------------------  289
ident
Sbjct aladalatstlpfqsakylmetwpfgellckavlsidyynmftsiftltmmsvdryiavc  221
DSSP  hhhhhhhhhlhhhhhhhhhhllllllhhhhhhhhhhhhhhhhhhhhhhhhhhhhhhhhhh


DSSP  ------------------------------------------------------------
Query ------------------------------------------------------------  289
ident
Sbjct hpvkaldfrtpakaklinicIwvlasgvgvpimvmavtrprdgavvcmlqfpspswywdt  281
DSSP  llllhhhhllhhhhhhhhhhhhhhhhhhhhhhhhhhhheeeeeelleeeeeellllllhhhhhh


DSSP  ------------------------------------------------------------
Query ------------------------------------------------------------  289
ident
Sbjct vtkicvflfafvvpiliitvcyglmllrlrsvrllsgskekdrslrritrmvlvvvgafv  341
DSSP  hhhhhhhhhllhhhhhhhhhhhhhhhhhhhhhhlllllllhhhhhhhhhhhhhhhhhhhhhh


DSSP  ------------------------------------------------------------
Query ------------------------------------------------------------  289
```

```
DSSP  ----lll
Query ----tkr  292
ident
Sbjct lcrkpcg  408
DSSP  hhhllll
```

No 765: Query=mol1A Sbjct=4fmlA Z-score=2.1

back to top

```
DSSP  lllllllllhhhhhhhhhhhhhhllllhhhhhhhhhhhhhhhhhhlllllllllleeee
Query aapysspptnlrslrdrltqvaerqgvvfgrlqrhvaxivvaqfaatltddtgaplllvk   60
ident
Sbjct ------------------------------------------------------------    0
DSSP  ------------------------------------------------------------


DSSP  hhhhhhhhhllllllllleeeeeellllhhhhhhhhhhhhhhllllleeeeeellleelllll
Query ggsslelrrgipdsrtskdfatvarrdielihеqladagetgwegftaiftapeeiaapg  120
ident
Sbjct ------------------------------------------------------------    0
DSSP  ------------------------------------------------------------


DSSP  lllllеeeeeeeeelleeeeeeeeeeeelllllllleeeeelhhhhlllllleeeeelL
Query xpvkprrftaklsyrgrafatvpievssveagnadqfdtltsdalglvgvpaavavpcxT  180
ident
Sbjct ------------------------------------------gshmiskadealryysaQ   18
DSSP  ------------------------------------------llllllhhhhhhhhhhhL


DSSP  HHHHHHHHHHHhhllllllllllLLHHhHHHHHHHHHLlhhhhhhhhhhhhhhhhhhlll
Query IPWQIAQKLHAvtavleepkandRAHDlVDLQLLEGLLldadlxptrsaciaifearaqh  240
ident           |
Sbjct GYTLLNNYLRD-----------rPYKQ-REAIDTLLSR----------------------   44
DSSP  LHHHHHHHHLL-----------lLLLH-HHHHHHHHHL----------------------


DSSP  llllllllllhhhhhhhhhlllllLLLLLLHHHHHHHHHHHHHHHHH-LLLL-------
Query pwpprvatlphwpliyagaleglhLELARTVDAAAQAVQRFVARIDR-ATKR-------  292
ident                                      |     ||        |
Sbjct ----------------------sylNDEPTSAGEFDKAMKAYVADVEAgLAKLpaspels   82
DSSP  ----------------------lllLLLLLLHHHHHHHHHHHHHHHHHHhHHHLlllllll


DSSP  ------------------------------------------------------------
Query ------------------------------------------------------------  292
ident
Sbjct fvyrglaldkpelaalkeqftgvgnivvepgfmststspdkawvndtllkirlpaghggrll  142
DSSP  eeeeeeellllhhhhhhhhhhlllllеeeelllеeellllllllleeeeeellllllleel


DSSP  ------------------------------------------------------------
Query ------------------------------------------------------------  292
ident
Sbjct gdaahfkgeaemlfptqtrlrvdrvvsstsgdfdtllntiptsdnasdnrnrikrlievs  202
DSSP  llllllllllleeellleeeeeeeeeelllllhhhhhlllllllllllllllleeeeee


DSSP  --
Query --  292
ident
Sbjct vl  204
DSSP  el
```

No 766: Query=mol1A Sbjct=1g87B Z-score=2.1

back to top

```
Sbjct ---------------------------------------------gtynygealqksimf   15
DSSP  ---------------------------------------------llllhhhhhhhhhhh


DSSP  HHHHHhhHHLLllllLLLLeeeeelllhhhhhhhhhhhhhhlllleeeeeellleellll
Query GGSSLelRRGIpdsrTSKDfatvarrdieliheqladagetgwegftaiftapeeiaapg  120
ident                   |
Sbjct YEFQR--SGDL--paDKRD-----------------------------------------   30
DSSP  HHHHL--LLLL--llLLLL-----------------------------------------


DSSP  llllleeeeeeeeelleeeeeeeeeeeellllllllLEEEEelhhhhlllllleeeeelL
Query xpvkprrftaklsyrgrafatvpievssveagnadqFDTLTsdalglvgvpaavavpcxT  180
ident
Sbjct -------------nwrddsgmkdgsdvgvdltggwyDAGDH-------------vkfnlP   64
DSSP  -------------lllllllllllllllllllllllLLLLL-------------leehhH


DSSP  HHHHHHHHHHHHH-LLLLllLLLLLLHHHHHHHHHHHH-LLHH-----------------
Query IPWQIAQKLHAVT-AVLEepKANDRAHDLVDLQLLEGL-LLDA-----------------  221
ident      |              |
Sbjct MSYTSAMLAWSLYeDKDAydKSGQTKYIMDGIKWANDYfIKCNptpgvyyyqvgdggkdh  124
DSSP  HHHHHHHHHHHHHhLHHHhhHHLLHHHHHHHHHHHHHHhHHLLlllleeeeeellhhhhh


DSSP  -------------------------hhHHHHHHHhhhhhhhlllllLLLLlllllhhhhh
Query -------------------------dlXPTRSACiaifearaqhpwPPRVatlphwpliy  256
ident                                 |
Sbjct swwgpaevmqmerpsfkvdaskpgsavCASTAAS--------lasaAVVF----------  166
DSSP  lllllhhhlllllleeeeelllllhhhHHHHHHH--------hhhhHHHH----------


DSSP  hhhhllllllllllllhhhhHHHHHHHHHHHHH-LLLL-----------------------
Query agalegldhlelartvdaaAQAVQRFVARIDR-ATKR-----------------------  292
ident
Sbjct ----------------kssDPTYAEKCISHAKnLFDMadkaksdagytaasgyyssssfy  210
DSSP  ----------------lllLHHHHHHHHHHHHhHHHHhhhhllllllllllllllllllh


DSSP  ------------------------------------------------------------
Query ------------------------------------------------------------  292
ident
Sbjct ddlswaavwlylatndstyldkaesyvpnwgkeqqtdiiaykwgqcwddvhygaelllak  270
DSSP  hhhhhhhhhhhhhhlllhhhhhhhhlhhhlllllllllllllllllllllhhhhhhhhhh


DSSP  ------------------------------------------------------------
Query ------------------------------------------------------------  292
ident
Sbjct ltnkqlykdsiemnldfwttgvngtrvsytpkglawlfqwgslrhattqaflagvyaewe  330
DSSP  hhllhhhhhhhhhhhhhlllllllllleellllleellllllhhhhhhhhhhhhhhhhlll


DSSP  ------------------------------------------------------------
Query ------------------------------------------------------------  292
ident
Sbjct gctpskvsvykdflksqidyalgstgrsfvvgygvnppqhphhrtahgswtdqmtsptyh  390
DSSP  lllhhhhhhhhhhhhhhhhhhhlllllllllllllllllllllhhhhlllllllllllll


DSSP  ------------------------------------------------------------
Query ------------------------------------------------------------  292
ident
Sbjct rhtiygalvggpdnadgytdeinnyvnneiacdynagftgalakmykhsggdpipnfkai  450
DSSP  llllllllllllllllllllllllhhhhlllhhhhhhhhhhhhhhhhhhlllllllllll


DSSP  ------------------------------------------------------------
Query ------------------------------------------------------------  292
ident
Sbjct ekitndeviikaglnstgpnyteikavvynqtgwparvtdkisfkyfmdlseivaagidp  510
DSSP  lllllleeeeeeeeeeeeelleeeeeeeeeelllllllllleeeeeeeeelhhhhllllh


DSSP  ------------------------------------------------------------
Query ------------------------------------------------------------  292
ident
```

```
DSSP  -------------------------------------------
Query -------------------------------------------  292
ident
Sbjct qgttywnpkndfsydglpttstvntvtnipvydngvkvfgnep  613
DSSP  llllllhhhlhhhllllllllleellllleeelleeeellll
```

No 767: Query=mol1A Sbjct=3chlA Z-score=2.0

back to top

```
DSSP  ------------------------------------------------------------
Query ------------------------------------------------------------    0
ident
Sbjct tgvtavvqkvveacqdeskrldlieiarsyppnqlrnmqrtfqaitgtfldaflkkhlsk   60
DSSP  llhhhhhhhhhhhhhlhhhllhhhhhhhhllhhhhhhhhhhhhhhhlllhhhhhhhhllh


DSSP  --------------------------------llllllllllhhhhhhhhhHHHHHLLLL
Query --------------------------------aapysspptnlrslrdrltQVAERQGVV   28
ident                                                    |
Sbjct dfeslvlmlykpraqllcelirgatkgagtdekclvdvlltiethevreirQLYYQLYND  120
DSSP  hhhhhhhhhlllhhhhhhhhhhhlllllllhhhhhhhhllllhhhhhhhhHHHHHHHLL


DSSP  HHHHHHHHHHH-----HHHH---------hhhlllLLLLLlleeeehhhhhhhhhlllll
Query FGRLQRHVAXI-----VVAQ---------faatltDDTGApllvkggsslelrrgipds   74
ident
Sbjct SLGDVVRKDCGdkymwAKLInavatgdriprdtheLEEDL--------------------  160
DSSP  LHHHHHHHHHLlllhhHHHHhhhhhllllllhhhHHHHH--------------------


DSSP  llllleeeeellllhHHHHHHHHH------hhhhllleeeeellleellllllLLLLEee
Query rtskdfatvarrdiELIHEQLAD------agetgwegftaiftapeeiaapgxPVKPRrf  128
ident
Sbjct ----vlvrkaietkGVKKDEVSTwirifatytradfrqlhkmysakyngdslrAGVED--  214
DSSP  ----hhhhhhhhllLLLHHHHHHhhhhhhhllhhhhhhhhhhhhhhlllllhhHHHHH--


DSSP  eeeeeelleeeeeeeEEEEellllllllleeeeelhhhhlllllleeeeELLHHHHHHHH
Query taklsyrgrafatvpIEVSsveagnadqfdtltsdalglvgvpaavavpCXTIPWQIAQK  188
ident                 |                                        |
Sbjct ----------efqglDEYA-----------------------fklahdfLYDPCCAAAFS  241
DSSP  ----------hllhhHHHH-----------------------hhhhhhhHHLHHHHHHHH


DSSP  HHHHHLLlllllLLLLLH-HHHHHHHHHHHLlhhhhhHHHHHHHHHHHhhlllLLLL-LL
Query LHAVTAVleepkANDRAH-DLVDLQLLEGLLldadlxPTRSACIAIFEaraqhPWPP-RV  246
ident      |                                                    |
Sbjct MNVAFAG-----SGSDSNrLNRITAMHFREC-----kGCKYYYKKVYG-----QAFDeRC  286
DSSP  HHHHLLL-----LLLLHHhHHHHHHHHHLLLL-----lLHHHHHHHHHL-----LLHHhHH


DSSP  LLlllhhhhhhhhhlllllllllllHHHHHHHHHHHHHH----hhhllll
Query ATlphwpliyagalegldhlelartVDAAAQAVQRFVAR----idratkr  292
ident ||                             |
Sbjct AT---------------------elKGVYGDAIKLLWEPvtvpllsmddy  315
DSSP  HH---------------------hlLHHHHHHHHHHLLLlllllllllll
```

No 768: Query=mol1A Sbjct=3nowA Z-score=2.0

back to top

```
DSSP  ------------------------------------------------------------
Query ------------------------------------------------------------    0
ident
Sbjct naktstkvkqmmdltfdlatpidkrraaannlvvlakeqtgaellykdhciakvasltkv   60
DSSP  lhhhhhhhhhhhhhhhllllllhhhhhhhhhhhhhlllhhhhhhhhhllhhhhhhhhhhh


DSSP  ------------------------------------------------------------
```

```
DSSP  lllhhhhhhhhhhhhhhhlllhhhhhhhhhhhllhhhhhhhhhlllllhhhhhhhhhhhh


DSSP  ------------------------------------------------------------
Query ------------------------------------------------------------    0
ident
Sbjct nalsglknkpdskpdkelctrnnreidtlltclvysitdrtisgaardgvielitrnvhy  180
DSSP  hhhhlllllllllllhhhhhhlhhhhhhhhhhhhhhhlllllhhhhhhhhhhhhhhhlll


DSSP  ------------------------------------------------------------
Query ------------------------------------------------------------    0
ident
Sbjct talewaerlveirglcrlldvcseledykyesamditgssstiasvclariyenmyydea  240
DSSP  llllhhhhhhhllhhhhhhhhhllllllllllllllllhhhhhhhhhhhhhhhllllhhh


DSSP  ------------------------------------------------------------
Query ------------------------------------------------------------    0
ident
Sbjct karftdqideyikdkllapdmeskvrvtvaitallngpldvgnqvvaregilqmilamat  300
DSSP  hhhhhhhhhhhhhhhhllllhhhhhhhhhhhhhhhlllhhhhhhhhhlllhhhhhhhhhh


DSSP  ------------------------------------------------------------
Query ------------------------------------------------------------    0
ident
Sbjct tddelqqrvacecliaasskkdkakalceqgvdilkrlyhskndgirvralvglcklgsy  360
DSSP  lllhhhhhhhhhhhhhhhhhlllhhhhhllhhhhhhhhhllllhhhhhhhhhhhhhhhll


DSSP  --------------------------lllllllllLHHHHHHHHHHHH------------
Query --------------------------aapysspptNLRSLRDRLTQVA------------   22
ident
Sbjct ggqdaairpfgdgaalklaeacrrflikpgkdkdiRRWAADGLAYLTLdaeckekliedk  420
DSSP  lllllllllllllhhhhhhhhhhhhhhllllllhHHHHHHHHHHHLLlhhhhhhhhhlh


DSSP  --------------------hhlllLHHH-------------------------------
Query --------------------erqgvVFGR-------------------------------   31
ident                          |
Sbjct asihalmdlarggnqsclygvvttfVNLCnayekqemlpemielakfakqhipeeheldd  480
DSSP  hhhhhhhhhhhlllhhhhhhhhhhhHHHLllllllllllhhhllllllllllllllhhh


DSSP  --------------hhhhHHHHHHhHHHHlllllllllleeeehhhhhhhhhLLLLL---
Query --------------lqrhVAXIVVaQFAAtltddtgaplllvkggsslelrrGIPDS---   74
ident
Sbjct vdfinkritvlanegittALCALA-KTES------------hnsqeliarvlNAVCGlke  527
DSSP  hhhhhhhhhhhhhllhhhHHHHHH-LLLL------------hhhhhhhhhhhHHHHLlhh


DSSP  ----------lllllEEEEelllhhhhhhhhhhhhhhlllleeeeellleelllllllll
Query ----------rtskdFATVarrdieliheqladagetgwegftaiftapeeiaapgxpvk  124
ident
Sbjct lrgkvvqeggvkallRMAL-----------------------------------------  546
DSSP  hhhhhhhllhhhhhhHHHH-----------------------------------------


DSSP  leeeeeeeeeelleeeeeeeEEEEllllllllleeeeeelhhhhllllleeeeeelLHHHH
Query prrftaklsyrgrafatvpiEVSSveagnadqfdtltsdalglvgvpaavavpcxTIPWQ  184
ident                     |
Sbjct --------------------EGTE--kgkrhatqalarigitinpevsfsgqrslDVIRP  584
DSSP  --------------------LLLH--hhhhhhhhhhhhhhllhhhhlllhhhhhhHLHHH


DSSP  HHHHhhhhhllllllllLLLLH-HHHHHHHHHHhllhhHHHHHHHHHHHHHhhhllllll
Query IAQKlhavtavleepkaNDRAH-DLVDLQLLEGlllldaDLXPTRSACIAIFearaqhpwp  243
ident                     |      |  |            |   |
Sbjct LLNL----------lqqDCTALeNFESLMALTN--lasMNESVRQRIIKEQ----gvski  628
DSSP  HHHL----------lllLLLHHhHHHHHHHHHH--hllLLHHHHHHHHHLL----hhhhh


DSSP  LLLLLlllhhhhhhhhhhllllllllllHHHHHHHHHHHHHHHHHLLL------------
Query PRVATlphwpliyagalegldhlelartVDAAAQAVQRFVARIDRATK------------  291
ident                                   |
Sbjct EYYLM---------------------edHLYLTRAAAQCLCNLVMSEDvikmfegnndrv  667
```

```
DSSP  ------------------------------------------------------------
Query ------------------------------------------------------------  291
ident
Sbjct kflallcededeetatacagalaiitsvsvkccekilaiaswldilhtlianpspavqhr  727
DSSP  hhhhhhhhlllhhhhhhhhhhhhhhhhhlhhhhhhhhllllhhhhhhhhhllllhhhhhh
```

```
DSSP  -----------------------------------------------------------l
Query -----------------------------------------------------------r  292
ident
Sbjct giviilnminageeiakklfetdimellsglgqlpddtrakarevatqclaaaeryrii  786
DSSP  hhhhhhhhhlllhhhhhhhhlllhhhhhlllllllllllhhhhhhhhhhhhhhhhhll
```

No 769: Query=mol1A Sbjct=3tb5A Z-score=2.0

back to top

```
DSSP  lllllllllhhhhHHHHhhhhhhllllhhhhhhHHHHHHHHHHHHHLLllllllLLEEee
Query aapysspptnlrslRDRLtqvaerqgvvfgrlqrHVAXIVVAQFAATLtddtgaPLLLvk   60
ident                                          |     |
Sbjct ----------mitlKSPR-----------eiemmDESGELLADVHRHL-----rTFIK--   32
DSSP  ----------llllLLHH-----------hhhhhHHHHHHHHHHHHHHH-----hHHLL--
```

```
DSSP  hhhhhhhhhlllllllllleeeeelLLHHHHHHHHHHHHHHHLllLEEE---------eEL
Query ggsslelrrgipdsrtskdfatvarRDIELIHEQLADAGETGweGFTA---------iFT  111
ident                               |     |  |    |  |           |
Sbjct -----------------------pgITSWDIEVFVRDFIESH--GGVAaqigyegykyAT   67
DSSP  -----------------------llLLHHHHHHHHHHHHHHHL--LLEElllllhhhlllLE
```

```
DSSP  LLEELL---------lllLLLLLEEEEEEEEELlEEEEEEEEEEEELLlllllllleeeee
Query APEEIA---------apgXPVKPRRFTAKLSYRgRAFATVPIEVSSVEagnadqfdtlts  162
ident                                    |           |
Sbjct CCSINDeichgfprkkvlKDGDLIKVDMCVDLK-GAISDSCWSYVVGE------------  114
DSSP  EEEELLeeelllllllllLLLLEEEEEEEEEEEL-LEEEEEEEEEELLL------------
```

```
DSSP  lhhhhlllllleeeeellhhhhhhHHHHHHhllllllllllllhhhhhhhhhhhllhhhh
Query dalglvgvpaavavpcxtipwqiaQKLHAVtavleepkandrahdlvdlqllegllldad  222
ident
Sbjct -----------------------sTPEIDR------------------------------  121
DSSP  -----------------------lLHHHHH------------------------------
```

```
DSSP  hhhhhhhhhhhhhhhlllllllllllllhhhhhHHHH----------------------
Query lxptrsaciaifearaqhpwpprvatlphwpliyAGAL----------------------  260
ident                                   |
Sbjct -------lmevtkkalylgieqaqvgnrigdighAIQTyvegegygvvrdfvghgigpti  174
DSSP  -------hhhhhhhhhhhhhhlllllllhhhhhhHHHHhhhhllleellllleeelllll
```

```
DSSP  --------LLLLLLL----------------------------------------llllh
Query --------EGLDHLE----------------------------------------lartv  272
ident          |
Sbjct hespmiphYGEAGKGlrlkegmvitiepmvntgtwrmkmdpngwtaytedgglscqyehs  234
DSSP  leeeeellLLLLLLLllllllleeeelleeellllleeellllllleeellllleeellee
```

```
DSSP  hhhhhhhhhhhhhhhhllll
Query daaaqavqrfvaridratkr  292
ident
Sbjct laitkegpriltsqgeelty  254
DSSP  eelllllleellllllllll
```

No 770: Query=mol1A Sbjct=2opdA Z-score=2.0

back to top

```
DSSP  lllllllllhhhhhHHHHhhhhhllllhhhHHHHHHHHHHHHHHHHLLlllllLLLEeee
Query aapysspptnlrslrDRLTqvaerqgvvfgrLQRHVAXIVVAQFAATLtddtgAPLLlvk   60
```

```
DSSP  hhhhhhhhllllllllllleeeeeLLLHHHHHHHHHHHHHHLLL-------lEEEEELLL
Query ggsslelrrgipdsrtskdfatvaRRDIELIHEQLADAGETGWE-------gFTAIFTAP  113
ident                           |   |   |
Sbjct ------------------------LDDNQTIKSKLERFVSGYKMnpkiaekyNVSVHFVN   65
DSSP  ------------------------LLLHHHHHHHHHHHHHHLLLlhhhhlleEEEEEELL


DSSP  EEllllllllllleeEEEEEEEL------leEEEEEEeeeeelllllllllleeeeelhhhh
Query EEiaapgxpvkprrFTAKLSYR------grAFATVPievssveagnadqfdtltsdalgl  167
ident  |
Sbjct KE---------kprAYSLVGVPktgtgytlSVWMNS------------------------   92
DSSP  LL---------lllLEEEEEEEllllllllEEEEEL------------------------


DSSP  llllleeeeellhhhhhhhhhhhhhhllllllllllllhhhhhhhhhhhhllhhhhhhhh
Query vgvpaavavpcxtipwqiaqklhavtavleepkandrahdlvdlqllegllldadlxptr  227
ident
Sbjct ------------------------------------------------------------   92
DSSP  ------------------------------------------------------------


DSSP  hhhhhhhhhhhlllllllllllllhhhhhhhhhhllllllllllhhhhhhhhhhhhhhhh
Query saciaifearaqhpwpprvatlphwpliyagalegldhlelartvdaaaqavqrfvarid  287
ident
Sbjct ------------------------------------vgdgykcrdaasarahletlssdv  116
DSSP  ------------------------------------llleeeellhhhhhhlllleelle


DSSP  hllll
Query ratkr  292
ident
Sbjct gceaf  121
DSSP  eeeel
```

No 771: Query=mol1A Sbjct=2i7rA Z-score=2.0

back to top

```
DSSP  llllllllllhhhhhhhhhhhhhhhhllllhhhhhhhhhhhhhhhhhhhlllllllleeee
Query aapysspptnlrslrdrltqvaerqgvvfgrlqrhvaxivvaqfaatltddtgaplllvk   60
ident
Sbjct ------------------------------------------------------------    0
DSSP  ------------------------------------------------------------


DSSP  hhhhhhhhhlllllLLLLLEEEEELlLHHHHHHHHHHHHHhllllleeeEELLLEElllll
Query ggsslelrrgipdsRTSKDFATVARrDIELIHEQLADAGEtgwegftaIFTAPEEiaapg  120
ident                                   |
Sbjct --------------XNLNQLDIIVS-NVPQVCADLEHILD--------KKADYAN-----   32
DSSP  --------------LEEEEEEEELL-LHHHHHHHHHHHHL--------LLLLEEE-----


DSSP  lllLLEEEEEEeeelleeeeEEEEEEEELL-LLLL-llLEEE------------------
Query xpvKPRRFTAKlsyrgrafaTVPIEVSSVE-AGNA-dqFDTL------------------  160
ident        ||                 |
Sbjct --dGFAQFTIG---------SHCLXLSQNHlVPLEnfqSGIIihievedvdqnykrlnel   81
DSSP  --lLEEEEEEL---------LEEEEEELLLlLLLLlllLLEEeeeelllhhhhhhhhhhh


DSSP  ---eelhHHHL--LLLLLEEeeellhhhhhhhhhhhhhllllllllllllhhhhhhhhhh
Query ---tsdaLGLV--GVPAAVAvpcxtipwqiaqklhavtavleepkandrahdlvdlqlle  215
ident              |
Sbjct gikvlhgPTVTdwGTESLLV----------------------------------------  101
DSSP  llleeeeEEELllLLEEEEE----------------------------------------


DSSP  hhllhhhhhhhhhhhhhhhhhhhlllllllllllllhhhhhhhhhhllllllllllhhhh
Query gllldadlxptrsaciaifearaqhpwpprvatlphwpliyagalegldhlelartvdaa  275
ident
Sbjct ------------------------------------------------------------  101
DSSP  ------------------------------------------------------------
```

```
ident
Sbjct ---qgpaglvldfyrxk  115
DSSP  ---elhhhleeeeeell
```

No 772: Query=mol1A Sbjct=3jcmI Z-score=2.0

back to top

```
DSSP  lllllllllhhhhhhhhhhhhhhllllhhhhhhhhhhhhhhhhhlllllllllleeee
Query aapysspptnlrslrdrltqvaerqgvvfgrlqrhvaxivvaqfaatltddtgaplllvk   60
ident
Sbjct ------------------------------------------------------------    0
DSSP  ------------------------------------------------------------


DSSP  hhhhhhhhhlllllllllleeeeelllhhhhhhhhhhhhhhllllleeeeelllleellll
Query ggsslelrrgipdsrtskdfatvarrdielihеqladagetgwegftaiftapeeiaapg  120
ident
Sbjct ------------------------------------------------------------    0
DSSP  ------------------------------------------------------------


DSSP  lllllleeeeeeeeelleeeeeeeeeeeellllllllleeeeelhhhhllllllleeeeeLL
Query xpvkprrftaklsyrgrafatvpievssveagnadqfdtltsdalglvgvpaavavpcXT  180
ident
Sbjct ----------------------------------------------------------EK    2
DSSP  ----------------------------------------------------------LL


DSSP  HHHHHHHHHHHHHL--LLLLLL--llLLLHHHHHHHHHHHHLlhhhhHHHHHhhhhhhh
Query IPWQIAQKLHAVTA--VLEEPK--anDRAHDLVDLQLLEGLLldadlXPTRSaciaifea  236
ident                                 |||
Sbjct FNPFEILPESIELFrtLALISLsetaQILPKIVDLKRILQQQ----eIKLLP--------   50
DSSP  HHHHHHHHHHHHHHhhLLLLLHhhhhLHHHHHHHHHHHHHHH----lLLHHH--------


DSSP  hlllllLLLLL-------------------------------------------------
Query raqhpwPPRVA-------------------------------------------------  247
ident        |
Sbjct -ffneiIPLIKsniklmhnflislysrrfpelsslipsplqyskvisilenenysknesd  109
DSSP  -hhhhhHHHHHhhhhhhhhhhhhhhhhhhlllhhhhlllhhhhhhhhhhhhlllhhhllll


DSSP  -------------------------------------------llLLHHhhhhhhhllll
Query -------------------------------------------tlPHWPliyagalegld  264
ident
Sbjct elffhlenkakltreqilvltmsmktsfknkepldiktrtqileaNSIL-----------  158
DSSP  hhhhhhhhhllllhhhhhhhhhhhhhllllllllhhhhhhhhhhHHHH-----------


DSSP  lllllllHHHHHHHH---------------------------------------------
Query hlelartVDAAAQAV---------------------------------------------  279
ident
Sbjct -----enLWKLQEDIgqyiaskisiiapnvcflvgpeiaaqliahaggvlefsripscni  213
DSSP  -----hhHHHHHHHHhhhhhhlhhhhlhhhhhhhlhhhhhhhhhhhllhhhhhhhllhhhh


DSSP  ------------------------------------------------------------
Query ------------------------------------------------------------  279
ident
Sbjct asigknkhlshelhtlesgvrqegylfasdmiqkfpvsvhkqmlrmlcakvslaarvdag  273
DSSP  llllllllhhhlllllllllllllhhhlhhhhlllhhhhhhhhhhhhhhhhhhhhhhhhh


DSSP  ------------------------------------------------------------
Query ------------------------------------------------------------  279
ident
Sbjct qkngdrntvlahkwkaelskkarklseapsisetkalpipedqpkkkragrkfrkykekf  333
DSSP  llllllllhhhhhhhhhhhhhhhhhhllllllllllllllllllllllhhhhhhhhh


DSSP  ------------------------------------------------------------
Query ------------------------------------------------------------  279
ident
```

```
DSSP  ----------hhhhhhhhhllll
Query ----------qrfvaridratkr  292
ident
Sbjct vmkhriseanqqadeflislghn  416
DSSP  hhhhhhhhhhhhhhhhhhhhhhl
```

No 773: Query=mol1A Sbjct=3nmzB Z-score=2.0

back to top

```
DSSP  --------------------LLLLLL----------------------llllhhHHHHHH
Query --------------------AAPYSS----------------------pptnlrSLRDRL   18
ident                         |                              |
Sbjct dkddmsrtllamsssqdsciSMRQSGclplliqllhgndknsrgskeararasaALHNII   60
DSSP  llhhhhhhhhhhhhlllllhHHHHHLhhhhhhhhhllllllllllhhhhhhhhhHHHHHH


DSSP  HH---hhHHLLlLHHHHHHHHHHHHHHHHHHLL--LLLL---------------------
Query TQ---vaERQGvVFGRLQRHVAXIVVAQFAATL--TDDT---------------------   52
ident                 |
Sbjct HSqpddkRGRR-EIRVLHLLEQIRAYCETCWEWqeAHEPgmdqdknpapvehqicpavcv  119
DSSP  LLllllhHHHH-HHHHHHHHHHHHHHHHHHHHHhhHLLLlllllllllllhhhlhhhhhh


DSSP  -------------------lllleEEEH-------------------HHHHHHHHLllll
Query -------------------gapllLVKG-------------------GSSLELRRGipds   74
ident                                                     |
Sbjct lmklsfdeehrhamnelgglqaiaELLQvdcemygltndhysitlrrYAGMALTNL----  175
DSSP  hhhhlllhhhhhhhhhllhhhhhhHHHHhhhhhlllllhhhhhhhhHHHHHHHHH----


DSSP  llllleeeeelllhhhhhhhhhhhhhhhlllleeeeelllleellllllllleeeeeeeee
Query rtskdfatvarrdieliheqladagetgwegftaiftapeeiaapgxpvkprrftaklsy  134
ident
Sbjct ------------------------------------------------------------  175
DSSP  ------------------------------------------------------------


DSSP  lleeeeeeeeeeeellllllllleeeeelhhhhlllllleeeeeLLHHHHHhHHHHHhhl
Query rgrafatvpievssveagnadqfdtltsdalglvgvpaavavpcXTIPWQIaQKLHAvta  194
ident                                                       |
Sbjct -----------------------------tfgdvankatlcsmkGCMRALV-AQLKS---  202
DSSP  -----------------------------hlllhhhhhhhhhlhHHHHHHH-HHHHL---


DSSP  llllllllLLLHHHHHHHHHHHHL----lHHHHHHHHHHHhhhhhhhllllllLLLLLll
Query vleepkanDRAHDLVDLQLLEGLL----lDADLXPTRSACiaifearaqhpwpPRVATlp  250
ident                        |            |
Sbjct --------ESEDLQQVIASVLRNLswradVNSKKTLREVG-------svkalmECALE--  245
DSSP  --------LLHHHHHHHHHHHHHHlllllHHHHHHHHHLL-------hhhhhhHHHLL--


DSSP  lhhhhhhhhlllllllllllLHHHHHHHHHHHHHHHHHLLLL------------------
Query hwpliyagalegldhlelarTVDAAAQAVQRFVARIDRATKR------------------  292
ident                             |
Sbjct -------------------vKKESTLKSVLSALWNLSAHCTEnkadicavdgalaflvgt  286
DSSP  -------------------lLLHHHHHHHHHHHHHHLLLLHHhhhhhhhlllhhhhhhhh


DSSP  ------------------------------------------------------------
Query ------------------------------------------------------------  292
ident
Sbjct ltyrsqtntlaiiesgggilrnvssliatnedhrqilrennclqtllqhlkshsltivsn  346
DSSP  llllllllllhhhhhhhhhhhhhhhhhlllhhhhhhhhhllhhhhhhhhhllllhhhhhh


DSSP  ------------------------------------------------------
Query ------------------------------------------------------  292
ident
Sbjct acgtlwnlsarnpkdqealwdmgavsmlknlihskhkmiamgsaaalrnlmanr  400
DSSP  hhhhhhhhllllhhhhhhhhhllllhhhhllllllllhhhhhhhhhhhhhhlll
```
