# Supplementary material for: Characterization of a toxin-antitoxin system in Mycobacterium tuberculosis suggests neutralization by phosphorylation as the antitoxicity mechanism
Source: Commun Biol. 2020 May 7;3:216. doi: 10.1038/s42003-020-0941-1 (PMC7205606; doi:10.1038/s42003-020-0941-1)
Supplement: Supplementary file 6 — Peer Review File [file 42003_2020_941_MOESM6_ESM.pdf]

Editorial Note: *This manuscript has been previously reviewed at another Nature Research journal. The manuscript was considered suitable for publication without further review at Communications Biology.*
